# *In silico* identification and characterization of a diverse subset of conserved microRNAs in bioenergy crop *Arundo donax* L.

**DOI:** 10.1038/s41598-018-34982-8

**Published:** 2018-11-12

**Authors:** Wuhe Jike, Gaurav Sablok, Giorgio Bertorelle, Mingai Li, Claudio Varotto

**Affiliations:** 1 Department of Biodiversity and Molecular Ecology, Research and Innovation Centre, Fondazione Edmund Mach, San Michele all’Adige, Trento, Italy; 2 Università degli Studi di Ferrara, Dipartimento di Scienze della Vita e Biotecnologie, Ferrara, Italy; 3 Department of Biosciences, University of Helsinki, Helsinki, Finland

## Abstract

MicroRNAs (miRNAs) are small non-coding RNA molecules involved in the post-transcriptional regulation of gene expression in plants. *Arundo donax* L. is a perennial C_3_ grass considered one of the most promising bioenergy crops. Despite its relevance, many fundamental aspects of its biology still remain to be elucidated. In the present study we carried out the first *in silico* mining and tissue-specific characterization of microRNAs and their putative targets in *A*. *donax*. We identified a total of 141 miRNAs belonging to 14 families along with the corresponding primary miRNAs, precursor miRNAs and a total of 462 high-confidence predicted targets and novel target sites were validated by 5′-race. Gene Ontology functional annotation showed that miRNA targets are constituted mainly by transcription factors, but three of the newly validated targets are enzymes involved in novel functions like RNA editing, acyl lipid metabolism and post-Golgi trafficking. Folding variability of pre-miRNA loops and phylogenetic analyses indicate variable selective pressure acting on the different miRNA families. The set of miRNAs identified in this study will pave the road to further miRNA research in *Arundo donax* and contribute towards a better understanding of miRNA-mediated gene regulatory processes in other bioenergy crops.

## Introduction

MicroRNAs (miRNAs) are endogenous small non-coding RNA molecules, containing approximately 22 nucleotides (nt), playing important roles in the regulation of gene expression at the post-transcriptional level^1^. In plants, miRNA genes are transcribed by RNA polymerase II into primary miRNAs (pri-miRNA)^2^, long precursors displaying polyadenylation like protein-coding mRNAs. In the nucleus, pri-miRNAs are processed by the Dicer-like 1 (DCL1) enzyme into shorther stem-loop hairpins, called precursor miRNAs (pre-miRNAs). After transportation into the cytoplasm by exportin 5, the pre-miRNAs are further cleaved by DCL1 to produce a duplex formed by the mature miRNA and its star miRNA (miRNA*), the nearly perfect reverse complementary RNA derived from the pre-miRNA stem^3^. Subsequently, the single strand of mature duplex corresponding to the mature miRNA is assembled with an Argonaute (AGO) RNA binding protein to form the RNA-induced silencing complex (RISC), which faciliate the interaction of mature miRNAs with their target mRNAs^4,5^. RISC-associated plant miRNAs recognize their target mRNA sequences by their nearly perfect or perfect complementarity, allowing them to identify with extremely high specificity only a small fraction of all transcribed mRNAs. This very high specificity is the key to enable microRNAs to regulate the expression of their targets. For the majority of plant miRNAs, target gene expression regulation is achieved by transcript cleavage, usually occurring between the 10^th^ and 11^th^ nucleotide at the 5′ end of the miRNA^6^. However, translational inhibition can be an additional/alternative mechanism used by some microRNAs to downregulate target expression^7,8^. In addition, the ability of a single miRNA to be potentially involved in the regulation of multiple target genes or of multiple miRNAs^9^ makes microRNAs very flexible regulators in a wide variety of metabolic and biological processes during all major growth and developmental stages of plants^10^.

Modern high-throughput sequencing technologies hold great promise to produce large sets of genomic or transcriptomic data in different tissues and at different developmental stages, which provides useful sequence resources to predict and analyze miRNAs in non-model plant species with non-sequenced genomes. In plants, computational approaches have been successfully applied and demonstrated effective to attain a comprehensive prediction of potential miRNAs, such as in *Cassava*, strawberry, *Arabidopsis thaliana* and many others^11–15^. Besides, the existence of curated online databases like miRBase^16^, collecting and organizing in a reference repository all miRNAs predicted by computational approaches, enormuously simplifies microRNA identification in novel transcriptomes. Both homology searches based on miRNA conservation among different plant species and the secondary hairpin loop structures of the pre-miRNA sequences along with the high negative minimal folding energy (MFE) are reliable criteria for the computational identification of miRNAs. This is why, even without experimental validation, miRNAs can reliably be distinguished from other types of small RNAs, thus reducing the number of false positive among predicted miRNAs^17,18^. Especially in plant species, the feature of nearly perfect or perfect complementarity of miRNAs to their target mRNA sequences also allows the reliable computational prediction of miRNA target genes. This, in turn, is very important for *in silico* prediction of miRNA functions, which allowed the genome-wide identification of microRNA genes^19–21^.

*Arundo donax L*., also called “giant reed”, is a perennial C_3_ fast growing grass^22^. Genetic studies indicate that *A*. *donax* originated in Eastern Asia, from where it spread, possibly by human intervention, to the Middle East and the Mediterranean^23^. More recently it was introduced in Africa and even Australia. In the large majority of its distribution area *A*. *donax* is reported to be a sterile species reproducing vegetatively, and it has been suggested that it may be a hybrid with uneven ploidy or possibly a (pseudo-) triploid species^24,25^. Despite its sterility, the vigorous growth and lack of natural antagonists allowed *A*. *donax* to become one of the most invasive riparian species in Southern USA (especially California), where it was introduced approximately one century ago to consolidate the sides of rivers^26^. This robustness makes it a very productive biomass species, as in optimal conditions *A*. *donax* fields become productive already after the second year and can provide dry biomass yields up to 40 tons per hectare for the next ten years. These yields are higher than those of other perennial rhizomatous grasses, thus constituting one of the most promising species for the production of biomass in the Mediterranean area^27^. In addition, *A*. *donax* requires little management input, as it is resistant to most pests and pathogens. It can grow without significant P or N fertilization, and is highly tolerant to heavy metals and saline soil^28–30^. *A*. *donax* has also been utilized as a raw material for bioethanol production with diluted oxalic acid pre-treatment, which is important to overcome recalcitrance of lignocellulose for ethanol production^31^. Recently, several transcriptomic studies have started to elucidate the content of expressed genes in *A*. *donax* (e.g.^32–34^), providing the opportunity for the in-depth mining of its gene space.

In the present study, we carried out the first computational identification and characterization of miRNAs for the biomass and bioenergy crop *A*. *donax* using tissue-specific transcriptomic data, as this could provide novel insights into the mechanism of *A*. *donax* development, metabolism and biology. We also predicted their putative target transcripts, providing through network analysis an in-depth dissection of gene ontologies and functional annotations for both putative miRNAs and target genes. These findings advance our understanding of miRNAs in *A*. *donax*, and have the potential to be further utilized for controlling secondary metabolism for improving the production of biomass and fermentation efficiency.

## Methods

### Prediction of potential *A*. *donax* miRNAs

All previously known 1616 miRNA precursor sequences from 12 monocotyledon species were downloaded from the miRBase database^35^ (Release 21.0; http://www.mirbase.org/ (2017)). These precursor miRNAs were used as query sequences for BLASTN searches against the four reference bud, culm, leaf and root transcriptomes of *A*. *donax*^32^ using default parameters and an E-value cut-off of 10. Only the best hit for each query sequence was retained and after elimination of redundant hits, these candidate primary miRNA sequences were scanned for hairpin-like secondary structures using the miRNA identification pipeline of the C-mii software^36^ (Supplementary Fig. 1). For prediction, only the miRNAs of *Oryza sativa* were used as reference, as the annotation of microRNA genes in this species is by far the most complete and reliable. To reduce type I (false positive) errors at the possible expense of a somehow inflated number of false negatives, we applied a stringent filtering of the primary microRNAs (pri-miRNAs) identified by C-mii (Supplementary Fig. 1). Only candidate sequences fitting the following criteria were considered as putative miRNAs in *A*. *donax*: (1) The length of predicted mature miRNAs should be in the range of 19–25 nucleotides; (2) A maximum of two mismatches compared with known rice mature miRNAs should be allowed; (3) The mature miRNA should be localized in only one arm within the predicted stem–loop structure; (4) No more than five mismatches should be allowed between miRNA sequence and guide miRNA sequence in the stem–loop structure; (5) miRNAs should have high A + U content (30–70%); and (6) minimal folding free energy (MFE) and minimal free energy index (MFEI) value of the secondary structure should be highly negative, with a cut-off value of −0.85 kcal/mol^10,37,38^. *A*. *donax* putative microRNAs were renamed according to the closest homologous locus in rice, identified as the best hit in BLASTN searches against all rice pre-miRNAs. Clustering of *A*. *donax* pri-miRNAs was finally carried out to identify tentative genetic loci, as no reference genome sequence is available for this species. Sequences with less than two mismatches in BLASTN searches over the whole alignment length were considered alleles of the same locus and renamed accordingly.

### Position-specific base composition of mature microRNAs

Nucleotide composition and their dominance at particular positions in mature *A*. *donax* miRNAs and reference *O*.*sativa* were analyzed using BioEdit^39^. Base composition frequency were calculated for each position of *A*. *donax* and *O*. *sativa* mature miRNAs, the average percentage of A, C, G and U bases was then calculated across all families. The position-specific nucleotide frequency of predicted mature miRNAs was summarized in graphical form.

### Structural and phylogenetic reconstruction of different microRNA families

The precursor sequences of the identified *A*. *donax* miRNAs were further analyzed to investigate stem-loop structure variabilities of pre-miRNAs. MUSCLE was used to align sequences which were subsequently used for phylogenetic analysis in MEGA 7.0 by employing the neighbor joining method with 1000 bootstrap replicates^40^.

### Prediction and functional annotation of putative *A*. *donax* miRNA targets

Putative microRNA targets were identified with two different programs: psRNATarget^41^ and TargetFinder^42^. The parameter sets for prediction by the psRNATarget server (http://plantgrn.noble.org/psRNATarget/home) were: maximum expectation of the score between small RNAs and their target transcripts: 3.0; complementarity scoring length (hspsize): 20; maximum allowed unpaired energy (UPE): 25; flanking length for analysis of target accessibility: 17 nt upstream and 13 nt downstream of the target site; central mismatch range leading to translation inhibition: 10-11 nt. Prediction of candidate targets with a stand-alone version of the TargetFinder program was carried out with default parameters. Sequences with a score lower than 4 were regarded as predicted miRNA target genes. The transcripts identified by both programs after removing non-coding transcripts by BLAST similarity search against the non-redundant protein databases from NCBI protein database were considered as putative microRNA targets and used for subsequent analyses. To better understand the function of *A*. *donax* miRNAs and their regulating targets, Gene Ontology (GO) annotation of the predicted *A*. *donax* miRNA targets were performed by using the annotation web tool FunctionAnnotator (http://fa.cgu.edu.tw/index.php)^43^. Further functional annotation of the predicted targets was carried out performing BLASTX searches against the *Arabidopsis thaliana* (https://www.arabidopsis.org/ (2018)) and *Setaria italica* (http://www.uniprot.org/ (2018)) protein databases using default parameters and an E-value cut-off of 1e-5. The biological networks formed by the putative miRNAs and their targets were visualized by Cytoscape version 3.5^44^.

### Total RNA extraction and cleavage site identification of miRNA targets

To confirm and identify miRNA-directed cleavage sites on target genes, total RNA isolations from root and bud in *A*. *donax* were performed as formerly described^34^. 5′ RACE System for Rapid Amplification of cDNA Ends, Version 2.0 kit (Invitrogen) was used for 5′ race experiments according to manufacturer’s instructions. The PCR products amplified using second nested primers were gel purified, cloned into pGEM-T (Promega) and sequenced. All primers used are listed in Supplementary Table 1.

### Comparative genomic analyses of conserved miRNA targets in *Arundo donax* and other plants

All predicted targets of the 11 conserved miRNA families from *Oryza sativa*, *Zea mays*, *Arabidopsis thaliana* and *Vitis vinifera* were downloaded from the PNRD database (http://structuralbiology.cau.edu.cn/PNRD) (Yi *et al*., 2015) and used for TBLASTN and BLASTX searches against the putative *Arundo donax* targets with an E-value cut-off of 1e^−5^. Hits with a Score value greater than 50 and with sequence coverage to the query greater than 50% (R. Pearson, 2013) were retained as conserved homologs, while the others were considered novel targets.

## Results

### Identification of putative miRNAs in *A*. *donax* and their characteristics

Through blast searches of the reference transcriptome of *A*. *donax* (1,195,562 transcript sequences) and microRNA prediction with the C-mii program we identified a total of 310 miRNA candidates. For reducing the false positives and improving the accuracy of the prediction, we retained for subsequent analyses only the predicted pre-miRNA with highly negative values of *MFEI* (< = −0.85 kcal/mol). In this way, we identified a total of 141 high-confidence putative miRNAs belonging to 14 different families (Supplementary Table 2, Supplementary sequences), corresponding to the most common miRNA families in *O*. *sativa*. *A*. *donax* putative miRNAs varied from 20 to 22 nucleotides in length, with the majority of them being 21 nt in length (85.82%), followed by 20 nt (7.80%), and 22 nt (6.38%), respectively. The lengths of precursor miRNAs varied from 60 to 193 nt with an average value of 99 nt, in line with what has been found in other plant species (Supplementary sequences). The *Ado-MIR444d-1c_b*, *Ado-MIR444d-2c_b*, *Ado-MIR444d-2c_c*, *Ado-MIR444d-2c_l*, *Ado-MIR444d-3c_c*, *Ado-MIR444d-4c_l and Ado-MIR444d-5c_r* exhibited the shortest precursor length of 60 nt, whereas *Ado-MIR169n-2_r* showed the longest precursor length of 193 nt (Supplementary Fig. 2 and Supplementary Table 2).

**Figure 1:**
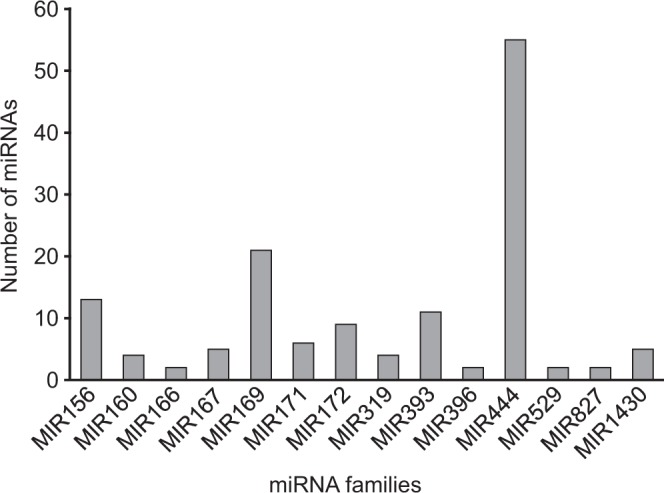
Number of miRNA member (s) predicted for each of 14 families identified in *A*. *donax*.

Among the 14 miRNA families, 10 (MIR166, MIR396, MIR529, MIR827, MIR160, MIR319, MIR1430, MIR167, MIR171 and MIR172) contained one to nine members, while the remaining four families (MIR156, MIR169, MIR393 and MIR444) were found to have more than ten members. The MIR444 family was the largest family with 55 members (Supplementary Table 2 and Fig. 1). We used the 94 miRNA loci of *O*. *sativa* corresponding to the 14 families from miRBase^35^ as reference to reliably identify tentative miRNA loci in *A*. *donax*. Based on the number of paralogs present in rice, a total of 69 loci were identified in *A*. *donax*. The MIR169 family had the highest number of loci both in *O*. *sativa* and *A*. *donax*. 18.75% of *A*. *donax* MIR169 loci corresponded to single loci in *O*. *sativa*. The highest number of *A*. *donax* loci per rice gene were seven, in line with the polyploidy of the giant reed (Table 1). 26.24% of the miRNAs generated from unique loci and primary transcripts, e.g., locus_17 and locus_18 (Supplementary Table 2).The miRNA identified from these loci did not show marked tissue-specific preferences, with 29 loci expressed in roots, followed by 20, 17 and 15 in culms, buds and leaves, respectively. However, 13.04% of loci generated multiple primary transcripts and miRNAs. For instance, there were three transcripts corresponding to locus_63. The miRNAs identified from this locus belong to the MIR444 family, and these miRNAs were expressed in buds, culms and leaves; another example was the two transcripts from locus_69, which belonged to MIR827 family and they were expressed in buds and roots (Supplementary Table 2).

**Table 1:** Rice homologs of *A*. *donax* microRNA loci.

miRNA family	loci in *Oryza sativa*	loci in *Arundo donax*	loci in common^#^
MIR156	12	8	a(1), b(1), c(1), g(3), j(1), k(1)
MIR160	6	4	b(3), c(1)
MIR166	13	2	a(2)
MIR167	10	5	d(2), g(3)
MIR169	18	16	a(1), c(3), i(1), n(3), p(1), q(7)
MIR171	9	5	a(1), c(2), f(1), i(1)
MIR172	4	3	b(1), d(2)
MIR319	2	2	a(2)
MIR393	2	4	a(1), b(3)
MIR396	8	2	a(1), b(1)
MIR444	6	12	a(2), c(4), d(5), e(1)
MIR529	2	1	a(1)
MIR827	1	1	*(1)
MIR1430	1	4	*(4)

^#^Letters correspond to the names of single loci in *O*. *sativa*, numbers in brackets correspond to the number of inferred loci in *A*. *donax*.

^*^Single locus in *O*. *sativa*.

**Figure 2:**
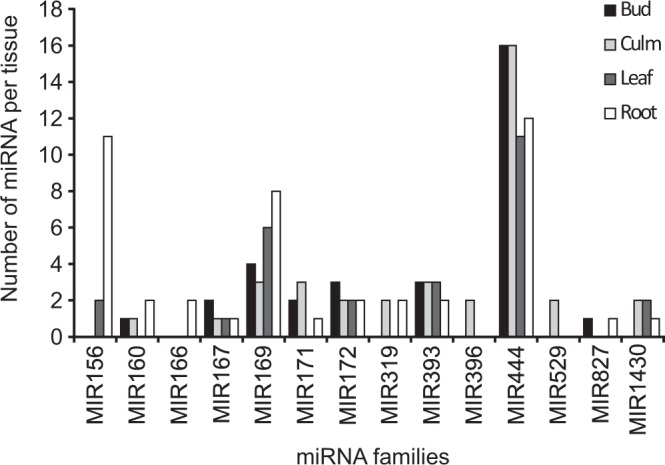
Number of mature miRNAs present in different tissues (bud, culm, leaf and root) of *A*. *donax*.

By analyzing more in-depth the tissue-specific co-expression pattern, we found that most of the identified miRNA families were preferentially expressed in root (31.9% of the mature miRNAs; Fig. 2), followed by culm (26.2%), bud (22.7%) and leaf (19.1%). Among the conserved miRNAs, about one third of the families were expressed in all four tissues studied, namely MIR444 (the largest family distributed on a per-tissue basis), MIR169, MIR167, MIR393 and MIR172. Only a minority of the sampled families were expressed only in one tissue, namely MIR166, which showed specific expression in the root, and MIR396 and MIR529, which were specifically expressed only in the culm (Supplementary Table 3). Overall, a relatively moderate differential expression was observed for all the predicted miRNA in the four tissues.MFE is an important parameter for determining the reliability of secondary structures of pre-miRNA, as the stability of the stem-loop structures of the precursor miRNAs is more stable when MFE has highly negative values. In the present study, the range of MFE (-kcal/mol) calculated was −26.4 to −81.8 (kcal/mol) with an average value of −48.67 (kcal/mol). MFEI is the minimal folding energy index, which can be used to distinguish pre-miRNA from other coding or non-coding RNA and RNA fragments. MFEI values ranged from −0.85 (kcal/mol; the maximal cut-off used for prediction) to −1.402 (kcal/mol) with an average of −1.03 (kcal/mol). These values were significantly lower than other reported small RNAs such as tRNAs (−0.64 kcal/mol), rRNAs (−0.59 kcal/mol) and mRNAs (0.62–0.66 kcal/mol), indicating that the identified *A*. *donax* miRNAs were putative miRNAs with high confidence^18,45,46^ (Supplementary Table 2).

### Analysis of position-specific nucleotide preference in *A*. *donax* mature miRNAs

**Figure 3:**
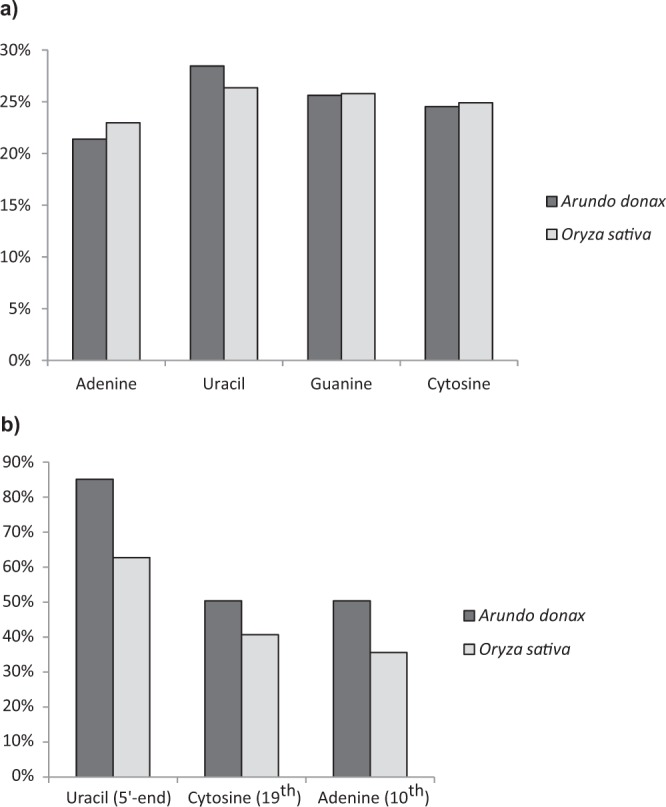
Composition of nucleotides in mature miRNAs from *A*. *donax* and *O*. *sativa*. (**a**) Overall nucleotide compositions (%) compared among mature miRNAs. (**b**) Selected position-specific nucleotide compositions of mature microRNAs in *A*. *donax* and *O*. *sativa*.

The overall percentage of each base of mature *A*. *donax* miRNAs was found to be 28.46% for uracil, 24.54% for cytosine, 25.62% for guanine and 21.39% for adenine (Supplementary Table 4). These values were in line with base compositions in *Oryza sativa* mature miRNAs. In general, a slightly lower GC content was apparent in *A*. *donax* (50.15%) than in *O*. *sativa* (50.69%) (Fig. 3a; Supplementary Table 4).

In the 5′-end of *A*. *donax* miRNA, uracil was found in 85.11% of the sequences, while in *O*. *sativa* it was present in 62.71% of the cases. Also other positions showed different base preferences as compared to rice. Cytosine was found to be a dominating base at position 19 (50.35%) in *A*. *donax*, while in rice it was present in only 40.68% of the cases. Adenine (50.35%) was abundant at the 10^th^ nt position of *A*. *donax* mature miRNAs, but less abundant in the same position in rice (35.59%; Fig. 3b; Supplementary Table 4). We notice, however, that the differences observed here only refer to the 14 microRNA families identified in *A*. *donax* with high conservation to rice and highly stringent selection parameters, which most likely do not provide a comprehensive picture of all microRNAs present in *A*. *donax*.

### Variability of stem-loop structures in *A*. *donax* pre-miRNAs

**Figure 4:**
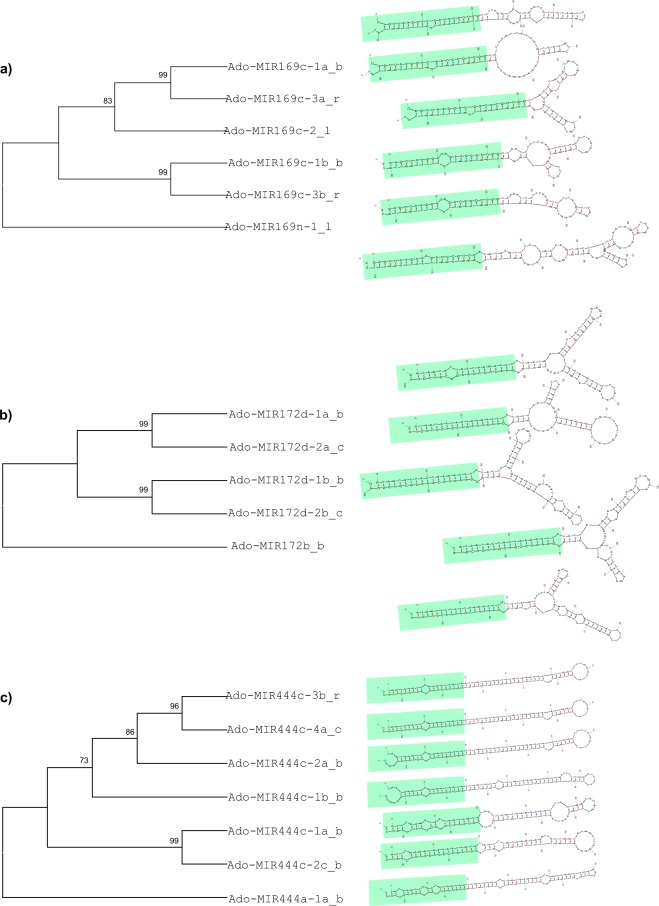
Stem-loop structures and phylogenetic relationships of *A*. *donax* pre-miRNA MIR169c (**a**), MIR172d (**a**) and MIR444c subfamilies (**c**). The single outgroups are at the base of the each cladogram. Uppercase letters identify mature microRNAs. Regions highlighted in light green are the stem structure of each pre-miRNA. Pre-miRNA structures were drawn with the C-mii software.

To understand folding variability of stem-loop structures and phylogenetic relationship among *A*. *donax* pre-miRNAs, the stem-loop structures of each family member and phylogenetic trees of corresponding family members were constructed using MIR169c, MIR172d and MIR444c multicopy loci as examples. As shown in Fig. 4, the stem structures were conserved among MIR169c (Fig. 4a) and MIR172d (Fig. 4b) paralogs, while divergent in MIR444c (Fig. 4c). On the contrary, the loop strctures were conserved among MIR444c paralogs, while divergent in MIR169c and MIR172d subfamilies. These results were consistent with the phylogenetic relationships of MIR169c (Fig. 4a), MIR172d (Fig. 4b) and MIR444c (Fig. 4c) loci. In general, the more similar the miRNA structures were, the more likely they formed supported clades in the respective phylogenetic trees.

### Target prediction of *A*. *donax* miRNAs and cleavage site validation

**Figure 5:**
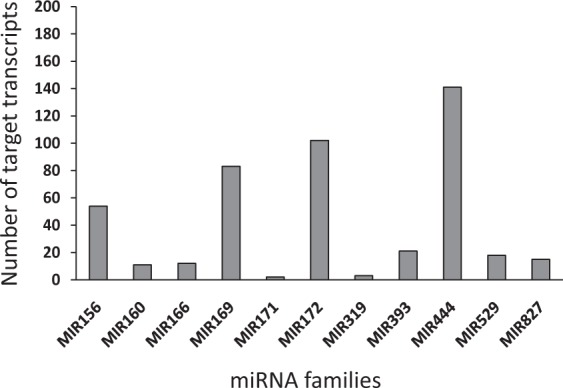
Number of predicted targets for potential *A*. *donax* miRNA families.

**Figure 6:**
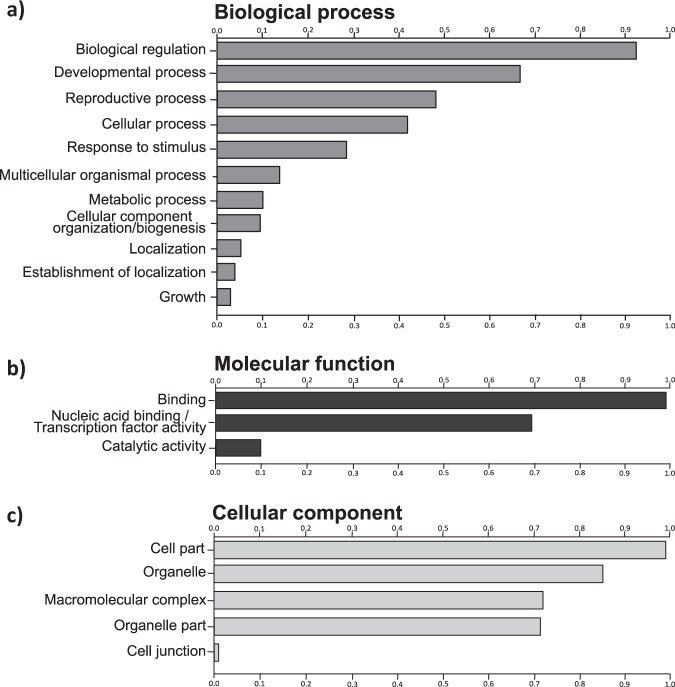
Functional annotation of predicted targets. (**a**) biological process, (**b**) molecular function and (**c**) cellular component.

Despite the high sequence complementarity of plant microRNAs and their targets, different algorithms display marked differences in the total number of predicted targets from the same set of microRNAs/mRNA transcripts^47^. For this reason we combined the predictions carried out with two popular programs, Targetfinder and psRNATarget, to attain a more reliable set of true positive targets^48^. By considering only the common hits obtained with these two programs, and removed non-coding transcripts by sequences similarity search against known biological protein database, we predicted a total of 107 mature miRNAs out of 141 mature miRNAs to regulate with high reliability 462 non-redundant target transcripts in *A*. *donax* (Supplementary Fig. 3; Supplementary Table 5; Supplementary sequences). MIR156, MIR160, MIR166, MIR169, MIR171, MIR172, MIR319, MIR393, MIR444, MIR529 and MIR827 were observed to regulate more than one gene (Fig. 5). 102 out of 107 *A*. *donax* miRNAs were found to target more than one type of transcript, whereas Ado-MIR160b-2_r, Ado-MIR160b-3_r, Ado-MIR319a-1a_c, Ado-MIR393b-1a_b and Ado-MIR393b-2c_c only targeted single genes (Supplementary Fig. 4). miRNAs, therefore, tend to regulate multiple distinct genes, and their targets belong to several gene families which are involved in different biological process, cellular component and molecular function (Fig. 6). These results indicate that miRNAs in *A*. *donax* combinatorially control multiple processes by regulating different target genes during plant growth and development, as suggested for other species^9,49^.

The type of post-transcriptional regulation was automatically analyzed for the predicted targets by the psRNATarget program. Out of 462 targets, 449 were predicted to undergo a cleavage type of inhibition. In particular, miRNA in the families MIR156, MIR160, MIR166, MIR169, MIR171, MIR172, MIR319, MIR393, MIR444, MIR529 and MIR827showed cleavage type of inhibition for all predicted target transcripts. Besides the 449 target transcripts regulated by transcriptional cleavage, MIR444 was the only one predicted to regulate also a total of 13 transcripts by translational suppression. These results indicate that miRNA-mediated post-transcriptional gene silencing (PTGS) in *A*. *donax* took place mainly through cleavage of the target transcripts, while, in line with what previously observed in plants^50^, translational regulation contributes marginally to overall microRNA regulatory activity.

**Figure 7:**
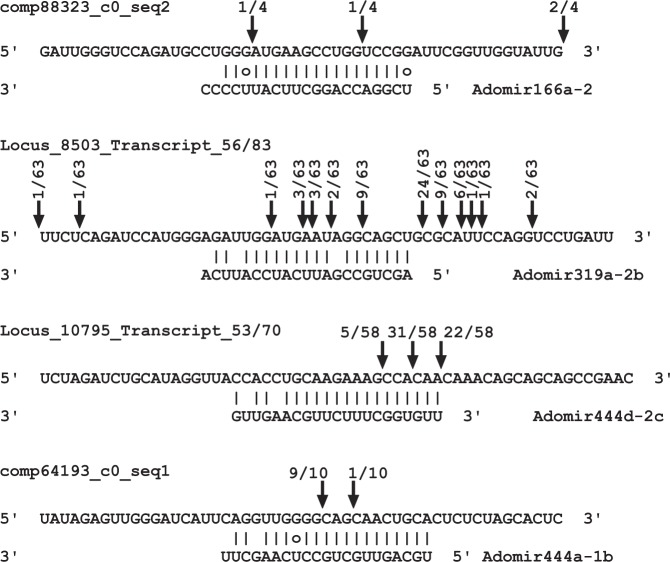
The cleavage site identification of four miRNA target genes with 5′-race in *A*. *donax*. The target gene is shown on top and corresponding miRNA on the bottom. the arrows indicate cleavage sites, and the numbers indicate the fraction of cloned PCR products which were used to determine the cleavage sites.

To evaluate the method applied here for miRNA target prediction, four targets (one conserved and three not homologous to previously reported targets in other species) were used to determine the nucleotide position at which a cleavage event occurs using 5′-RACE by sequencing the amplified PCR products. Based on these analyses, the target cleavage sites reported in Fig. 7 were observed. The major cutting site of *Ado-MIR444a-1b*_*b:comp64193_c0_seq*. *1* in buds was at the 11^th^ position, while the target *comp88323_c0_seq*. *2* of *Ado-MIR166a-2_r* in roots was cut with low efficiency and less precisely; The target *Locus_8503_Transcript_56/83_Confidence_0*.*566_Length_5268* of *Ado-MIR319a-2b_r* in roots was sliced in several positions including canonical sites, while the other *Ado-MIR444d-2c_b* new target in *Locus_10795_Transcript_53/70_Confidence_0*.*480_Length_2677* was cleaved preferentially in two positions in buds, but not in canonical sites.

### Functional annotation of predicted targets

For functional annotation, gene ontology (GO) analysis was carried out for the predicted *A*. *donax* targets, which indicated their involvement in regulating diverse physiological processes (Supplementary Table 6). In line with the major role of microRNAs in the regulation of transcriptional cascades by targeting transcription factors^51^, the main molecular functions associated to the predicted *A*. *donax* target genes were binding and more specifically nucleic acid binding transcription factor activity, while only a minority of the functions referred to catalytic activity. For the cellular component category, the majority of the target genes were associated with cell part, organelle and macromolecular complex. Under the biological process category, the majority of targets were associated with biological regulation, developmental process and reproductive process. The functional annotation of the targets also was performed by sequence similarity searches against the *Arabidopsis thaliana* and *Setaria italica* proteins using the BLASTx algorithm. A total of 425 (91.99%) target genes functional hits were found in *Arabidopsis thaliana*, and a total of 455 (98.48%) in *Setaria italica*, both species with fully sequenced genomes.

### Conservation of miRNA targets among *Arundo donax* and other plants

In order to investigate the conservation of the putative miRNA targets in *Arundo donax* compared to other known species namely *Oryza sativa*, *Zea mays*, *Arabidopsis thaliana* and *Vitis vinifera*, BLAST sequence similarity search approach was utilized to identify conserved miRNA targets. A total of 390 homologous targets for 10 conserved miRNA families were identified in *A*. *donax*, while 72 targets for eight *A*. *donax* conserved miRNA families were identified as novel targets (Table 2). Thus 84.42% of *A*. *donax* mRNA targets for the conserved miRNA families considered were conserved in *A*. *donax* and the other species. The remaining 15.58% of the conserved miRNAs targeted novel target transcripts.

**Table 2:** Numbers of *A*. *donax* conserved miRNA targets and novel targets in conserved miRNA families across representative monocots and dicots plant genomes.

miRNA families	*A. donax*	*O. sativa*	*Z. mays*	*A. thaliana*	*V. vinifera*	Novel targets
MIR156	50	48	45	40	47	4
MIR160	11	10	9	9	10	0
MIR166	11	11	11	11	11	1
MIR169	77	75	77	68	58	6
MIR171	2	2	2	2	2	0
MIR172	74	74	74	51	60	28
MIR319	0	0	0	0	0	3
MIR393	19	19	19	19	19	2
MIR444	120	118	110	—	—	21
MIR529	11	10	1	—	—	7
MIR827	15	15	15	0	—	0
Total	390	382	363	200	207	72

Note: “—” represent the miRNA targets absence; “0” indicates lack of the corresponding miRNA in the species.

## Discussion

Identification of expressed microRNA loci from *A*. *donax*, an important biomass species, may have relevant applications to improve the quality/amount of its biomass, thanks to the establishment of promising protocols for regeneration and genetic transformation^52,53^. In addition, it can significantly contribute to elucidate the evolutionary trajectories followed by such important regulators of gene expression in polyploid species^23^. Transcriptome sequencing is one of the most efficient and cost-effective approaches available for gene discovery, and can provide massive and valuable information for the identification of low abundance or tissue-specific miRNA and their targets^54–57^, phylogenetic inferences and characterization of polyploid speciation^58,59^. In addition, the choice to leverage on the reference transcriptome of *A*. *donax* for microRNA identification has been dictated by the lack of a fully sequenced genome for this species. As such, we did not expect to be able to obtain a full representation of all microRNA loci existing in *A*. *donax*. We, thus, decided to carry out a stringent identification of the putative microRNA pri-miRNA by relying on a series of filtering steps. Especially the use of a threshold of −0.85 kal/mol for MFEI provided a set of well resolved miRNA candidates with respect to tRNAs (average MFEI = −0.64), rRNAs (MFEI = −0.59) and mRNAs (MFEI = −0.65)^12,46^. The length distributions of predicted pre- and mature-microRNAs (from 60 to193 nt and from 20 to 22 nt, respectively) are in line with those observed in other species^12,14,60,61^, thus supporting the reliability of the identification. Also the analysis of position-specific nucleotide preferences confirms similarity of *A*. *donax* mature miRNAs to those from other species. In particular, dominance of uracil at the first position of the 5′ terminus may play an important role in miRNA biogenesis or RISC formation, while preference for cytosine at position 19 seems to be relevant for targeting RISC or Dicer-mediated cleavage to specific sites in pre-miRNAs^60,62,63^. We note, however, that the set of identified microRNAs is not comprehensive and, therefore, there is a concrete possibility that bias in the nucleotide preference estimation may exist. Comparison of the number of microRNA loci present in the rice genome with the number of tentative loci identified in *A*. *donax* indicates that the major and evolutionarily most conserved families have been identified in our screening^63^. The relatively higher numbers of loci identified for families MIR444 and MIR1430 further suggest that lineage-specific expansion of these microRNAs possibly took place in *A*. *donax* after its divergence from the rice lineage, occurred approximately 80 million years ago^64^. While the involvement of MIR444 in mediating perception of viral infections is well established (see below), the function of MIR1430 remains to be fully elucidated. Previous studies indicate that in rice MIR1430 post-transcriptionally regulates a gene encoding a member of the nuclear factor Y-A (NF-YA) gene family^65,66^ (LOC_Os12g42400). The transcript of LOC_Os12g42400 is expressed specifically in callus, flower and panicles^67^ and it is co-regulated by MIR169 family members^65^. In Arabidopsis MIR169 takes part in stress-induced early flowering^68^. MIR1430 family expansion may predate *A*. *donax* loss of fertility associated to polyploidization^69^, when transition to the reproductive phase likely still constituted a relevant stress-escape strategy for this species. Another possibility is that the likely lineage-specific expansion of MIR1430 gene family may be related to suppression of fungal resistance, as differential repression of NF-YA genes by MIR169 has been found to negatively regulate rice immunity against the blast fungus *M*. *oryzae*^70^.

Validation of four target genes confirmed in all cases slicing within the target region, albeit with different efficiency and specificity. Previous studies demonstrated that the cleavage sites of miRNA targets do not only occur at the canonical position between 10^th^ and 11^th^ nucleotides (nt) from the 5′ end of the miRNA and miRNA:target base pairing regions, but also happen outside of base pairing regions^71,72^. The conserved targets comp88323_c0_seq. 2 of MIR166a, for instance, had diverse cleavage sites compared to the canonical position in its *Oryza sativa* orthologs^72,73^. This is likely due to the highly dynamic nature of microRNA slicing sites, as even the same microRNA can cleave its target in different places in different physiological conditions^74^. In addition, cleavage site position has been documented to vary among species for the same miRNA/target combination^74^. Worth of note, the other three targets we validated are specific for *A*. *donax*. Other microRNA/target pairs have been demonstrated to be species-specific^72^, indicating that they contribute to interspecific differences in gene regulation. Our results, thus suggest that in addition to the functions already demonstrated in rice (tillering control^75^; antiviral defence^76^), based on the function of Arabidopsis target orthologs AT3G49170 and AT1G13640, *A*. *donax* MIR444a and MIR444d could play a role also in additional cellular mechanisms, like, respectively, chloroplast RNA editing and acyl lipid metabolism^77,78^. Analogously, MIR319 is possibly involved in regulation of post-Golgi trafficking by targeting the orthologue of Arabidopsis AT3G60860, an ARF guanine-nucleotide exchange factor^79^. While as expected the large majority of miRNA/target mRNA pairs are conserved in other angiosperm species (Table 2), our results indicate that up to 15% of the targets of the highly conserved microRNA families may be novel in *A*. *donax* and/or other species.

In line with the known tendence of microRNAs to preferentially target transcription factors^80^, we found that the major molecular function classes targeted in *A*. *donax* were “binding” and “nucleic acid binding transcripton factor activity”. Also the most common biological process GO classes indicate that the sets of microRNAs identified in *A*. *donax* play an important role in the regulation of development, reproduction, metabolism as well as stress response. In particular, network analysis of *A*. *donax* miRNAs and their target genes highlighted the large number of targets regulated by MIR444, MIR172, MIR169 and MIR156. In *Oryza sativa*, RNA-dependent RNA ploymerase1 (RDR1) is a central component in the antiviral RNA-silencing pathway, and MIR444 plays an important role in transducing the antiviral signal from virus infection to RDR1 expression^76^. The capacity of *A*. *donax* to asyntomatically stand viral infections may, thus, in part depend on the possible expansion of the MIR444 familiy in this species^81^. Previous studies showed that MIR156 regulates developmental timing by repressing the expression of functionally distinct SPL transcription factors, while MIR172 regulates flowering time and flower formation by regulating the expression of AP2-like transcription factors^82,83^. MIR172 could, therefore, be a useful tool to modulate flowering time in *A*. *donax*, analogously to what observed in *Sorghum*^84^. The MIR169/NF-YA (Nuclear factor Y, subunit A) is a well established regulatory module functioning in developmental processes and responding to environmental stresses^85,86^. Also MIR166 may possibly respond to environmental stress and it controls root architecture^87^, a trait that may be relevant for improving *A*. *donax* tolerance to drought^34^.

The growing interest towards improvement of biofuel/bioenergy crops has stimulated in recent years the search for novel approaches to improve their productivity. As specific miRNAs regulate several bioenergy traits, genetic transformation of bioenergy crops like switchgrass and poplar with selected microRNAs has been already demonstrated a viable option to improve plant biomass, decreasing the lignin content, modulating stress responses and flowering time^88,89^. This is, however, still just a small fraction of the 30 microRNA families of potential interest for bioenergy crop improvement^90^. Dissecting the genetic architecture of miRNA loci in the crop of interest is the first fundamental step for any subsequent attempt to improve biofuel feedstock species^90^. The putative microRNA loci identified in the present study from the transcriptome of *A*. *donax* provide novel opportunities for the genetic improvement of biomass yield and quality in this emerging biomass species. They also shed new light into the complex dynamics of microRNA evolution in this highly polyploid species, providing evidence for lineage-specific amplification of microRNA families and targeting of novel cellular mRNAs involved in important aspects of plant fitness and productivity, like RNA editing, acyl lipid metabolism and post-Golgi trafficking. In plants several non-conserved, species-specific microRNAs families have been found^91^. As mining of species-specific miRNAs has still not been carried out in *A*. *donax*, it seems likely that the number of microRNA targets yet to be identified could be significantly larger than that estimated in our study. In-depth identification of such novel targets will thus be required to attain a more complete understanding of microRNA regulation in this important bioenergy crop.

## Electronic supplementary material

List of supplementary files and legends

Supplementary Figures 1-4

Supplementary Table 1

Supplementary Table 2

Supplementary Table 3

Supplementary Table 4

Supplementary Table 5

Supplementary Table 6

Sequences

## Data Availability

All data analysed during this study are included in this article (and its Supplementary Information files).
