# Supplementary material for: In silico identification and characterization of a diverse subset of conserved microRNAs in bioenergy crop Arundo donax L
Source: Sci Rep. 2018 Nov 12;8:16667. doi: 10.1038/s41598-018-34982-8 (PMC6232160; doi:10.1038/s41598-018-34982-8)
Supplement: Supplementary file 1 — List of supplementary files and legends [file 41598_2018_34982_MOESM1_ESM.pdf]

In silico identification and characterization of a diverse subset of conserved microRNAs in bioenergy crop *Arundo donax* L.

Wuhe Jike, Gaurav Sablok, Giorgio Bertorelle, Mingai Li, Claudio Varotto

Legends and list of supplementary data

Supplementary Figures

Supplementary Fig. 1 Schematic workflow of the analyses for the identification of *A. donax* microRNAs and their targets.

Supplementary Fig. 2 Length distributions of mature miRNA and pre-miRNA.

Supplementary Fig. 3 Venn diagrams of putative targets predicted by both psRNAtarget and TargetFinder.

Supplementary Fig. 4 Functional networks of *A. donax* microRNAs and their putative targets (Cytoscape). Blue dots represent miRNA genes, while red dots are their targets for each of four tissues (bud, culm, leaf and root). Lines represent miRNA-target relationships. Clusters of lines originating from single miRNAs indicate the size of the post-transcriptional gene silencing (PTGS) co-regulation for each miRNA gene.

Supplementary Tables

Supplementary Table 1 Primers used in this study.

Supplementary Table 2 Features of putative miRNA identified from *A. donax*. The highest number of *A. donax* loci per rice gene highlighted in green.

Supplementary Table 3 Distribution in different tissues of primary miRNA transcripts by family.

Supplementary Table 4 Base composition of *A. donax* miRNAs and *O. sativa* miRNAs. In bold red the interspecific differences at specific positions highlighted in the text.

Supplementary Table 5 Putative targets of *A. donax* miRNAs.

Supplementary Table 6 Functional annotation of putative targets of *A. donax* miRNAs.

SEQUENCES

Predicted *A. donax* primary miRNAs.

Predicted *A. donax* precursor miRNAs.

Predicted *A. donax* miRNA targets.
