# Supplementary material for: In silico identification and characterization of a diverse subset of conserved microRNAs in bioenergy crop Arundo donax L
Source: Sci Rep. 2018 Nov 12;8:16667. doi: 10.1038/s41598-018-34982-8 (PMC6232160; doi:10.1038/s41598-018-34982-8)
Supplement: Supplementary file 2 — Supplementary Figures 1-4 [file 41598_2018_34982_MOESM2_ESM.pdf]

In silico identification and characterization of a diverse subset of conserved microRNAs in bioenergy crop *Arundo donax* L.

Wuhe Jike, Gaurav Sablok, Giorgio Bertorelle, Mingai Li, Claudio Varotto

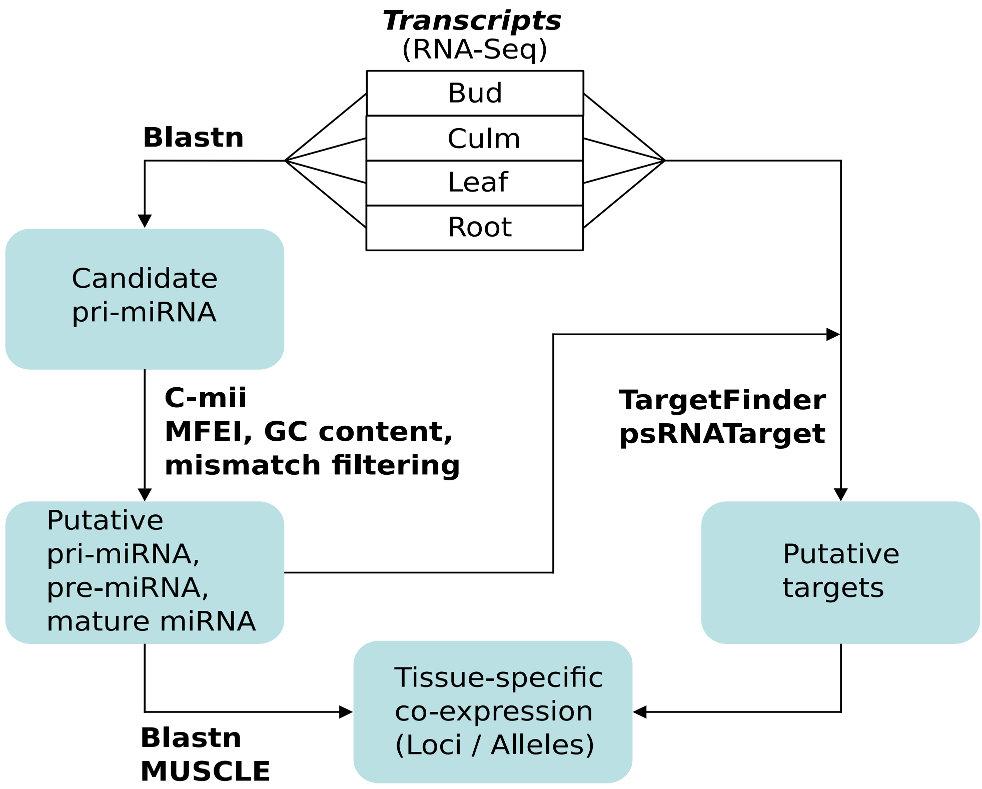

Suppl. Fig. 1: Schematic workflow of the analyses for the identification of *A. donax* microRNAs and their targets

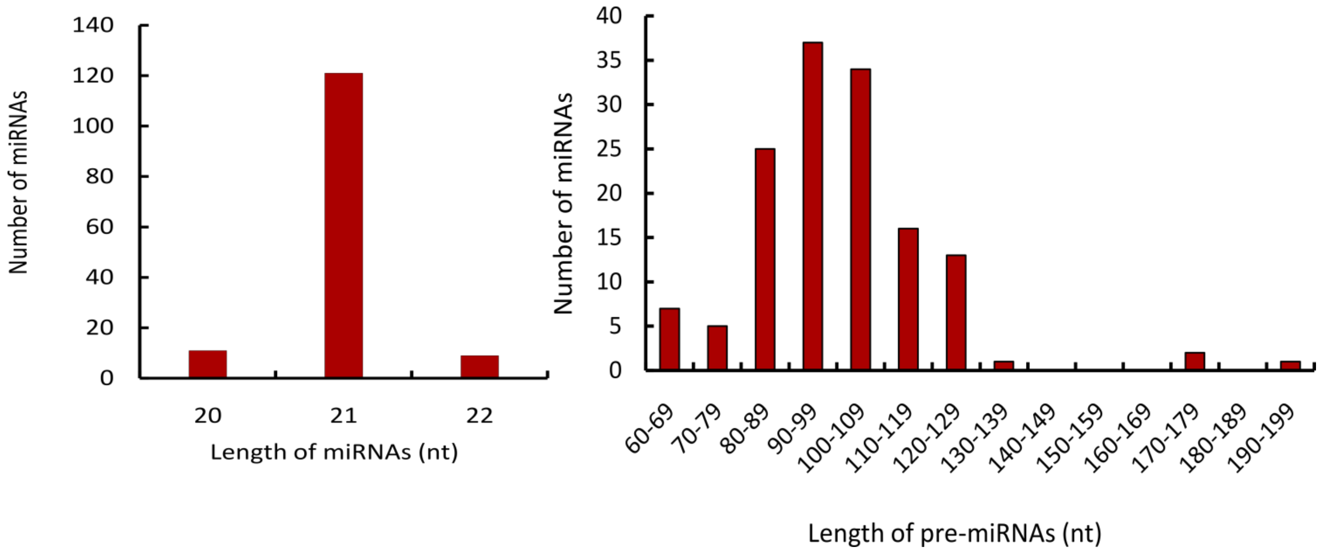

Suppl. Fig. 2: Length distributions of mature miRNA and pre-miRNA

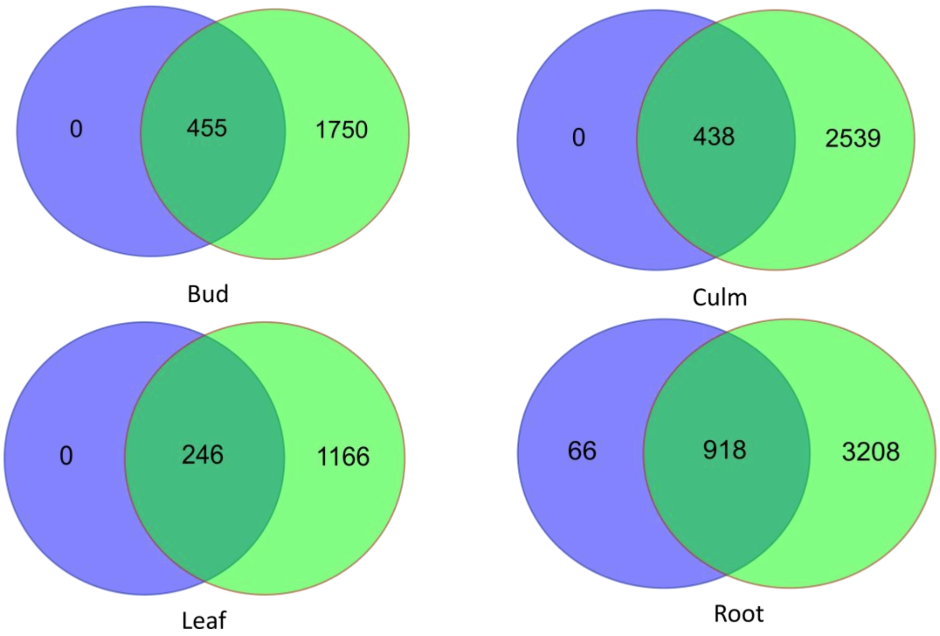

Suppl. Fig. 3: Venn diagrams of putative targets predicted by both psRNAtarget and TargetFinder.

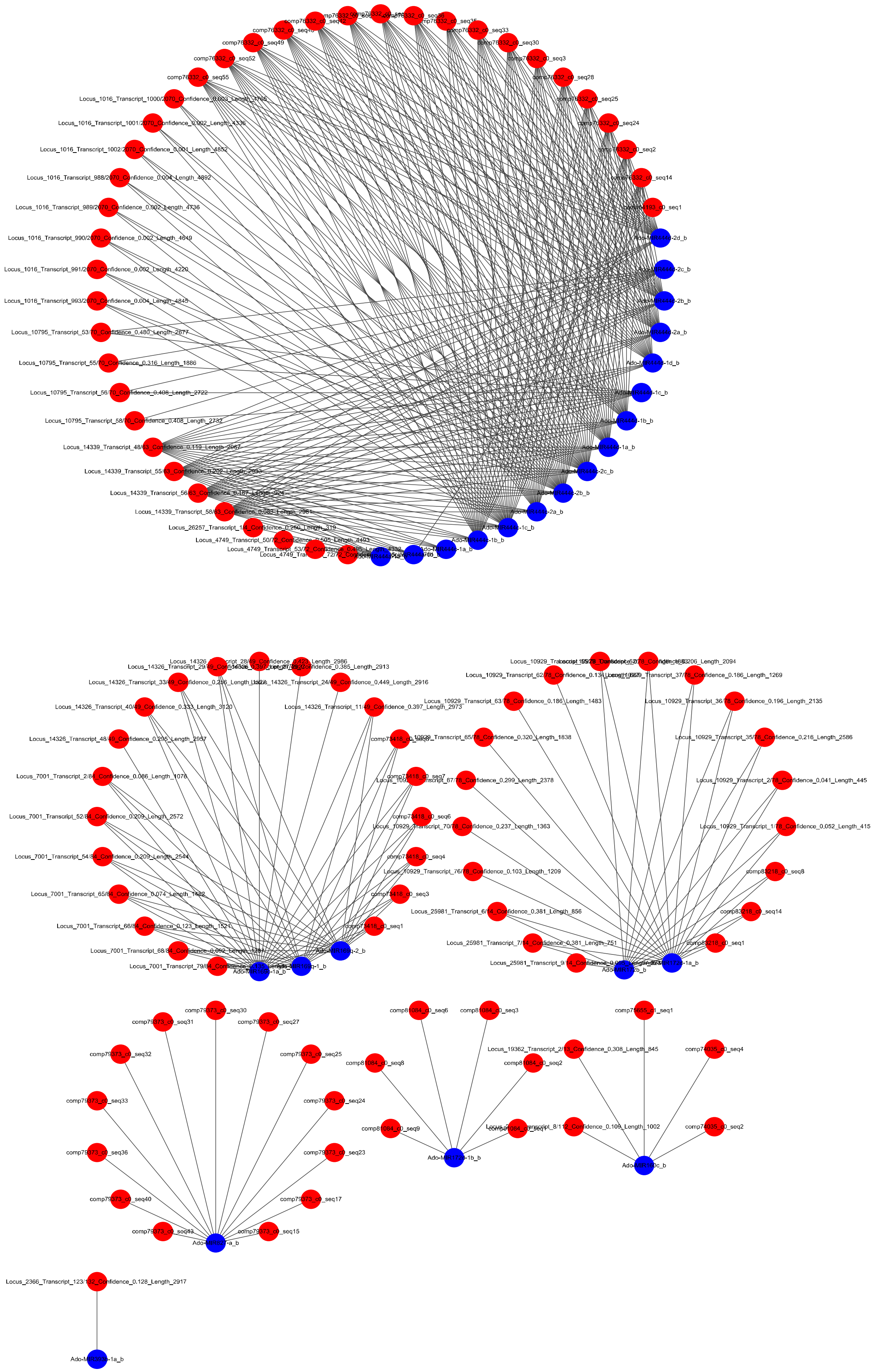

BUD

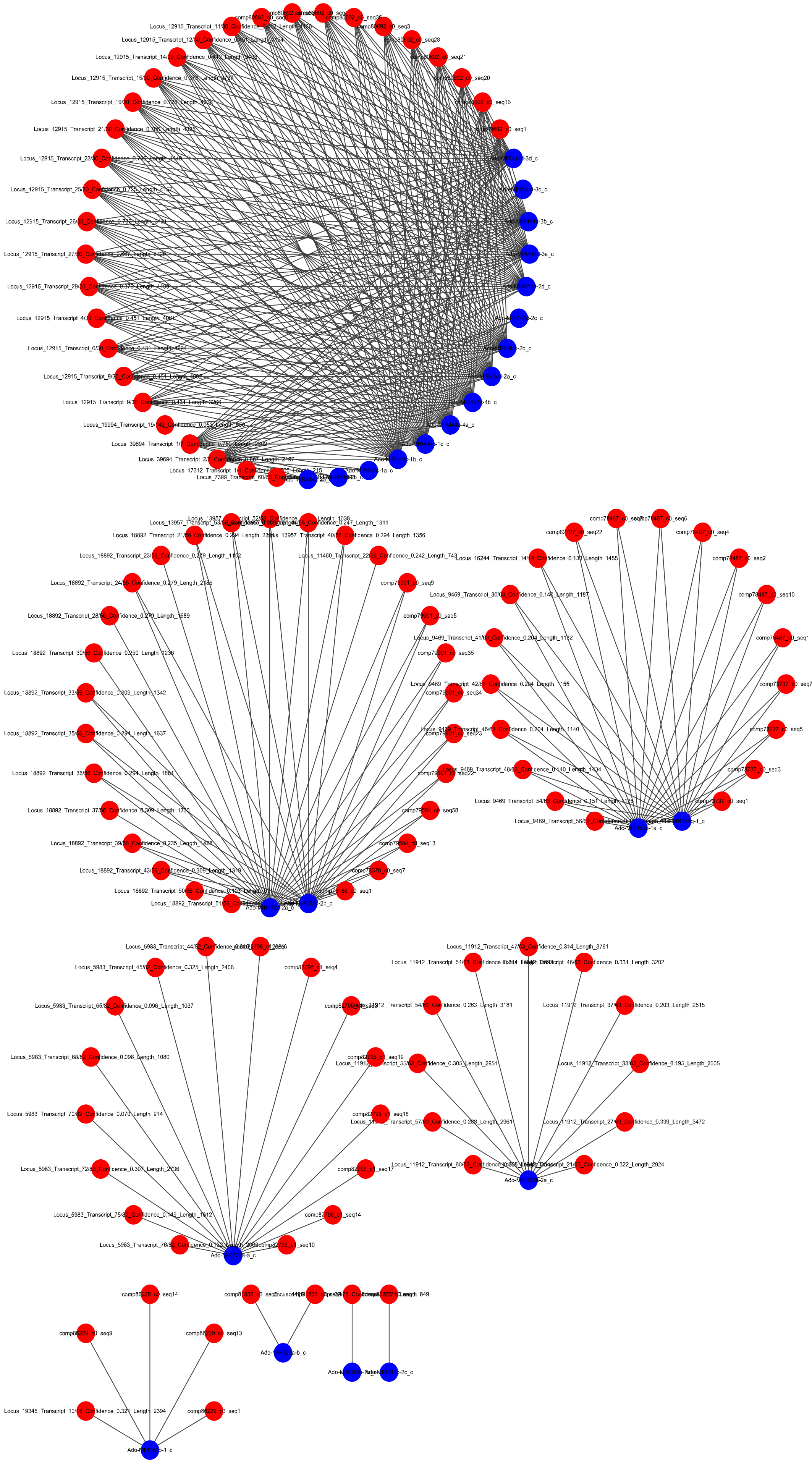

CULM

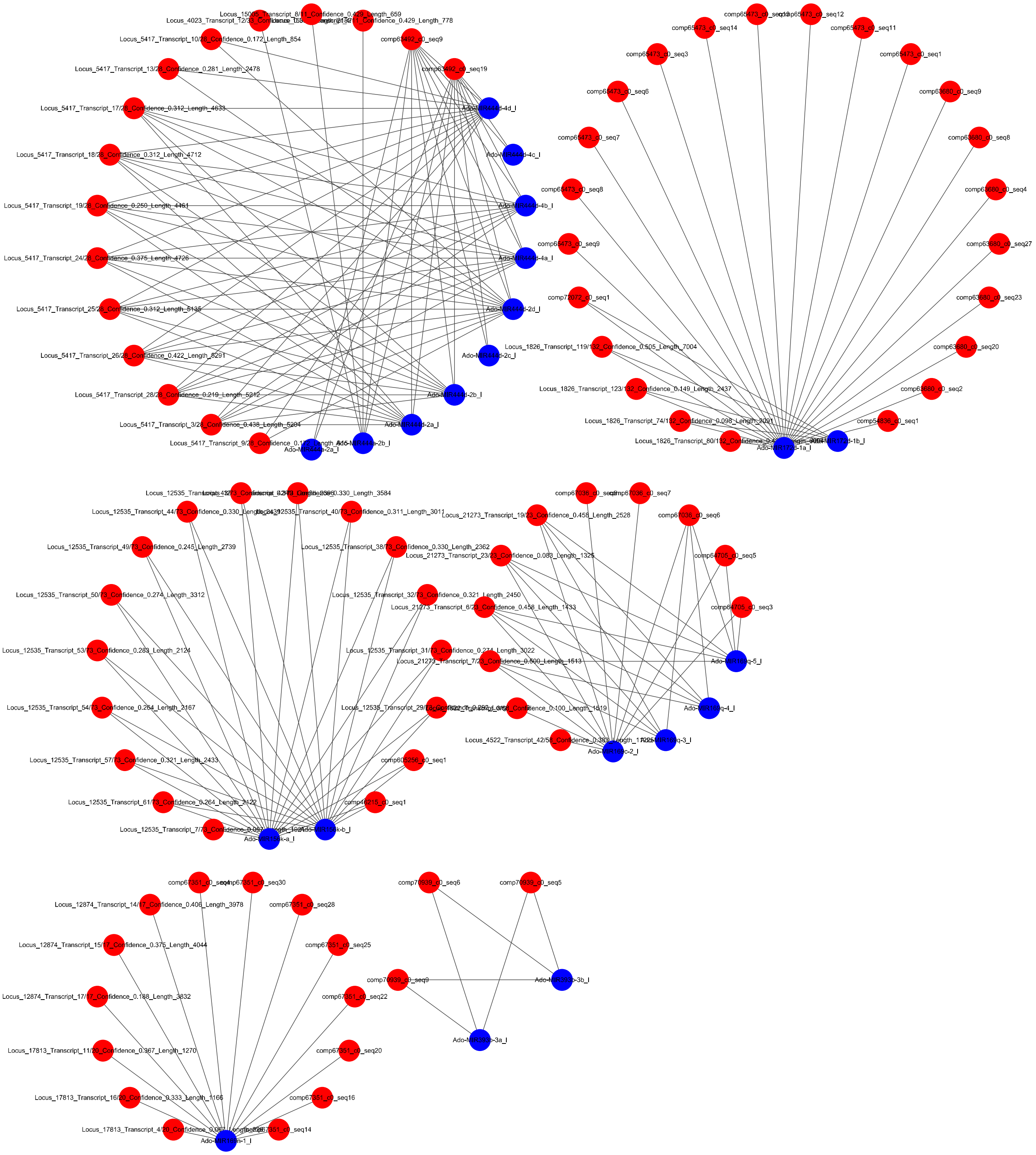
Locus_5417_Transcript_10/28_Confidence_0.172_Length_854
Locus_5417_Transcript_13/28_Confidence_0.281_Length_2478
Locus_5417_Transcript_17/28_Confidence_0.312_Length_4633
Locus_5417_Transcript_18/28_Confidence_0.312_Length_4712
Locus_5417_Transcript_19/28_Confidence_0.250_Length_4461
Locus_5417_Transcript_24/28_Confidence_0.375_Length_4726
Locus_5417_Transcript_25/28_Confidence_0.312_Length_5135
Locus_5417_Transcript_26/28_Confidence_0.422_Length_5291
Locus_5417_Transcript_28/28_Confidence_0.219_Length_5212
Locus_5417_Transcript_3/28_Confidence_0.438_Length_5204
comp63492_c0_seq9
comp63492_c0_seq19
Ado-MIR444d-2b_l
Ado-MIR444d-2a_l
comp65473_c0_seq14
comp65473_c0_seq11
comp65473_c0_seq3
comp65473_c0_seq1
comp65473_c0_seq6
comp63680_c0_seq9
comp65473_c0_seq7
comp63680_c0_seq8
comp65473_c0_seq8
comp63680_c0_seq4
comp65473_c0_seq9
comp63680_c0_seq27
comp72672_c0_seq1
comp63680_c0_seq23
Locus_1826_Transcript_119/132_Confidence_0.505_Length_7004
comp63680_c0_seq20
Locus_1826_Transcript_123/132_Confidence_0.149_Length_2437
comp63680_c0_seq2
Locus_1826_Transcript_74/132_Confidence_0.098_Length_2091
comp54838_c0_seq1
Locus_12535_Transcript_49/73_Confidence_0.245_Length_2739
Locus_12535_Transcript_38/73_Confidence_0.330_Length_2362
Locus_12535_Transcript_50/73_Confidence_0.274_Length_3312
Locus_12535_Transcript_32/73_Confidence_0.321_Length_2450
Locus_12535_Transcript_53/73_Confidence_0.283_Length_2124
Locus_12535_Transcript_31/73_Confidence_0.274_Length_3022
Locus_12535_Transcript_54/73_Confidence_0.264_Length_2167
Locus_12535_Transcript_57/73_Confidence_0.321_Length_2433
comp60256_c0_seq1
Locus_12535_Transcript_61/73_Confidence_0.264_Length_2122
comp46215_c0_seq1
Locus_21273_Transcript_19/23_Confidence_0.458_Length_2528
comp67036_c0_seq6
Locus_21273_Transcript_23/23_Confidence_0.083_Length_1325
comp64705_c0_seq5
Locus_21273_Transcript_6/23_Confidence_0.458_Length_1433
comp64705_c0_seq3
Locus_21273_Transcript_7/23_Confidence_0.500_Length_1513
Locus_4522_Transcript_42/58_Confidence_0.383
Locus_12874_Transcript_14/17_Confidence_0.406_Length_3978
comp67351_c0_seq28
Locus_12874_Transcript_15/17_Confidence_0.375_Length_4044
comp67351_c0_seq25
Locus_12874_Transcript_17/17_Confidence_0.188_Length_3832
comp67351_c0_seq22
Locus_17813_Transcript_11/20_Confidence_0.367_Length_1270
comp67351_c0_seq20
Locus_17813_Transcript_16/20_Confidence_0.333_Length_1166
comp67351_c0_seq16
comp70939_c0_seq6
comp70939_c0_seq5
comp70939_c0_seq9
Ado-MIR1428b-3b_l
Ado-MIR1428b-3a_l

LEAF

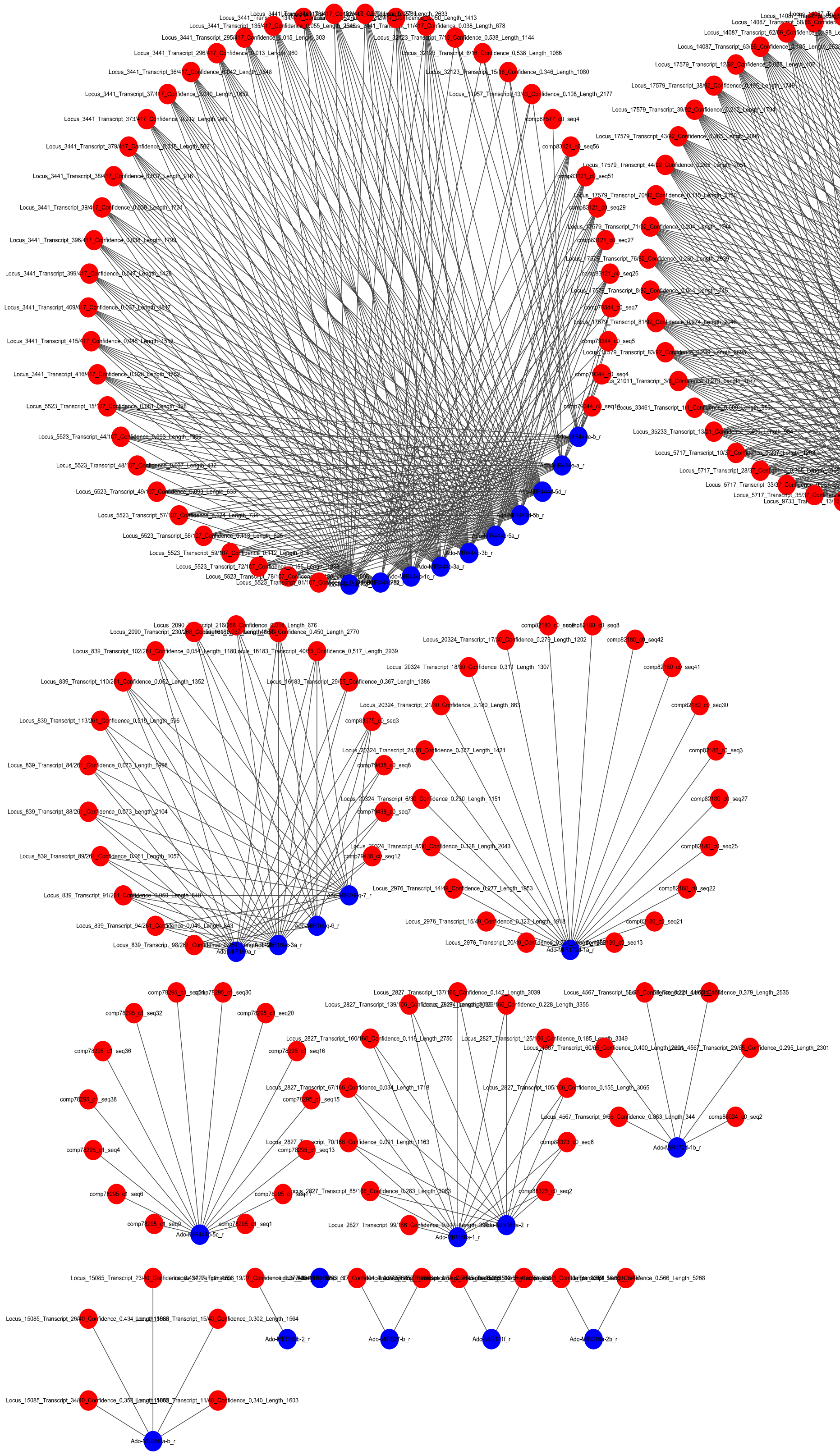

ROOT

Suppl. Fig. 4: Functional networks of *A. donax* microRNAs and their putative targets (Cytoscape). Blue dots represent miRNA genes, while red dots are their targets for each of four tissues (bud, culm, leaf and root). Lines represent miRNA-target relationships. Clusters of lines originating from single miRNAs indicate the size of the post-transcriptional gene silencing (PTGS) co-regulation for each miRNA gene.
