# Supplementary material for: In silico identification and characterization of a diverse subset of conserved microRNAs in bioenergy crop Arundo donax L
Source: Sci Rep. 2018 Nov 12;8:16667. doi: 10.1038/s41598-018-34982-8 (PMC6232160; doi:10.1038/s41598-018-34982-8)
Supplement: Supplementary file 9 — Sequences [file 41598_2018_34982_MOESM9_ESM.pdf]

In silico identification and characterization of a diverse subset of conserved microRNAs in bioenergy crop *Arundo donax* L.

Wuhe Jike, Gaurav Sablok, Giorgio Bertorelle, Mingai Li, Claudio Varotto

SEQUENCES

Predicted *A. donax* primary miRNAs

```
>culm_36
AGCAAGUGAAGAGGAGGGAAGCCUUACAUGGAGACGAGAAACCCAUCUCUAUUAGCCAAGAAUGGCUUGCCUAUCUCCACUAUUUGGUUCAUAAGGGAUU
CGGAGUCCUGAUGAUGGAUGAAAUGUGGAUGAUGGCGAGCCUUCUUCGGCUAAGAGAGUGAUUUUCAUCCCCCAUGCUAGGCUGCCCCUCUUUCUAAUUU
AUUUGCGGAAUAUCGUCUUUCUAAUUCUCAUUUCUCAAUUUCUCUUUGAAUGUCAAAGUUCAUUAUGACCUUUGAUAACGGGUUCACUCGUUUGUUCUAG
AGUUCAGAUCAUUUAUCACCGACUAAGAACAUGGUUUCUUGAAGGUAACAAGGUAAUUAAAAGAAGGGCAGGAAGUGGCAACUGAUAUAGGUCGUUUCUU
AUUCUGCUUUUUGUAUCCUCCUCUUUGAGAUGUGGCGGUUGGGCAGUUAUUUCUCAUUUGAAGAUGAAGAAGCACCUAGGU
>leaf_17
UUAGCCAAGAAUGGCUUGCCUAUCUCCACUAUUUGGUUCAUAAGGGAUUCGGAGUCCUGAUGAUGGAUGAAAUGUGGAUGAUGGCGAGCCUUCUUCGGCU
AAGAGAGUGAUUUUCAUCCCCCAUGCUAGGCUGCCCCUCUUUCUAAUUUAUUUGCGGAAUAUCGUCUUUCUAAUUCUCAUUUCUCAAUUUCUCUUUGAAU
GUCAAAGUUCAUUAUGACCUUUGAUAACGGGUUCACUCGUUUGUUCUAGAGUUCAGAUCAUUUAUCACCGACUAAGAACAUGGUUUCUUGAAGGUAACAA
GGUAAUUAAAAGAAGGGCAGGAAGUGGCAACUGAUAUAGGUCGUUUCUUAUUCUGCUUUUUGUAUCCUCCUCUUUGAGAUGUGGCGGUUGGGCAGUUAUU
UCUCAUUUGAAGAUGAAGAAGCACCUAGGUGGUUGAGGGACUUGAACAUGGCUGUUGUAGGAGGUUAAAAAGCCUCCCAGCCAGCAAGCCCAUUUGUUUG
UUUCCAUUAAACAAUUAUUUAUAAUGUUUAUAGUGACAGUAUGAUAAAAAAUAUCAUUUGUAGUGAUAUGGUUGCGGUACUUCACUGCCAUUUAGGGACG
UAAUUGUAUAAUUUAGCAGCUGGAAUGUUGAGAAUUAUACUAAAAUAAUGGUUUGCUAGGGUUCUACUCCAAAUGUUGGUGGUCUGUAUUUUAUCUUCUA
UGACAUUUUGACCAGGAAUAUUAAUCAUUCUACCCUUUGUAGCAUGCAAUACAGAUCAAGUUUACAAAAAUGUAUUGAAGUUGUUAAGAGUAACAUUGAU
GUUAAUUUUCAUGACUUAUUAGCUGUUUGGUUCCAUCCAGGAUUUUAGGAAGACAUAUAUACAUCAUUUUGGAAGUGAAUGUACCAGGCACGAACCCCGU
GUAUGCGCACGCCACUGUGUUGAACGGAUCUUGAAGAAAACAACAGGGGUGCGCCAUAUAUGUCACCAUAGAGGUGUGGGUGCAAAUUAAAAGUGCUUCC
AAGGAACAUUUAUAUGAGUUGAUUUUUUGAAUUUAUAUAUAUGAGUUGUAAUAGGGGGAAACUGAAAUAGAACCGGUGUCCUAUGCAACCCCAGGCGCAA
AUUUGAUGUGACCAUGGUGCUAAGAAUGAGAUACAGAUAUACUGAAUUAUAUUAGUUGUAACUUGUAAGGAGGCAACUUCCUGCUUUUCUUUUCCUAAAA
AA
>culm_52
UAUUACGAAAAGAAGACUGGGAAGUUGCAUUGCCUCCUUACAACUAAUACAAUUUAGUAUAUCUAUCUCAUUCUUAGCACAAUGGUCACCGGUUCUAUUU
CAGCUUUCCCCUAUUACAACCCACAUAUAUAAAUUAAGCAAAACAACUCAUAUAUAAAUGUGUCCUUGGAAGCACUUUAAAUUUGCACCCUCACCCCUAU
GGUAACAUAUAUGGUGCACCCCUGUUGUUUUCUUCAAGAUCCGAGCAUCGUUCAACACAGCGGUGAGCAUACGUAUAUAGGGUUUGUGCCUGGUACAUUU
GCUUCCAAAAUGAUGCAUGGAUACGUCUUCCUAAACUCCUGUAUGAAACCAAACAGCUAAUUAGUCAUGAAAGAAAACAACAAUAGUGUCACUCUAAACU
GCAAUACAUUUUUGAAAAUAAACUUGGUCCGAUUGCAUGCUACAAAGGGUAGAAUGAUUAAUUUACCUGGUCAAAAUUUCACAGAAGCAAAAGUAGAGAC
CACGAACAUCUAGUAGUACCCUAGCAAGUCAUUGUUUCAGUAUAAUUCUUAACAUCGCAGCUACUAAUUAAAUUAUACAAUAAGGUGCCCUAAAUAGCAG
UGAAGAAUUACCACAAUCAUAUCACUAAAAAUGAUUUUUUAUCAUAUACUGUCACUGUAAUAAAUAAUUGUUUAAUGGGAAUAAGCAAGCGUGCUUGGUG
GCUGGCAGACUGUUUUAACCUCCUGGUACAGCCAUGUUCAUGUCCCACGACCACCUAAGUCCCUCCUUCAUCUUUACAAGAGAAAUAAUCACCGAACCGC
CACAUCUCAAAGAGGAGGAUACAGAAAGUAGUUCAAGAAAUGGCCUAUAUCAGUUGCCACUUCCAGCCCUUCUUUUACUUACCUUGUUACAUACAAUUCU
UGAAAUUGUUACAUUCAAUUCUUGAAACCAUGCUUCUUGAUAAAUGAACAAAAGAGUGAAACCACUAUCAGAGGUCAUAACCAACUUUGAUAUUCAAGGA
CAAAUUAUAGAAAUGAGAAUUAGAAAGAGGAUCUGCCCACAAAAAGUUUAGAAAGAGGGACAGCCUAGCAUGGGGGAUGAGAAUCACUCUCUUAGCCAAA
AAAGGCUCACCAUCAUCCACAUUUCAUCCAUCAUCAGGACUCCGAAUCCCUUAUGAACCAAAUAGUGGAGAUAGGCAAGCCAUUCUUGGCUAAUAGAGAU
GGGUUUCUCGUCUCCAUGUAAGGCUUCCCUCCUCUUCACUUGCUCACUCAU
>leaf_24
AGCACAAUGGUCACCGGUUCUAUUUCAGCUUUCCCCUAUUACAACCCACAUAUAUAAAUUAAGCAAAACAACUCAUAUAUAAAUGUGUCGUUGGAAGCAC
UUUAAAUUUGCACCCUCACCCCUAUGGUAACAUAUAUGGUGCACCCCUGUUGUUUUCUUCAAGAUCCGAGCAUCGUUCAACACAGCGGUGAGCAUACGUA
UAUAGGGUUUGUGCCUGGUACAUUUGCUUCCAAAAUGAUGCAUGGAUACGUCUUCCUAAACUCCUGUAUGAAACCAAACAGCUAAUUAGUCAUGAAAGAA
AACAACAAUAGUGUCACUCUAAACUGCAAUACAUUUUUGAAAAUAAACUUGGUCCGAUUGCAUGCUACAAAGGGUAGAAUGAUUAAUUUACCUGGUCAAA
AUUUCAUAGAAGCAAAAGUAGAGACCACGAACAUCUAGUAGUACCCUAGCAAGUCAUUGUUUCAGUAUAAUUCUUAACAUCGCAGCUACUAAUUAAAUUA
UACAAUAAGGUGCCCUAAAUAGCAGUGAAGAAUUACCACAAUCAUAUCACUAAAAAUGAUUUUUUAUCAUAUACUGUCACUGUAAUAAAUAAUUGUUUAA
UGGGAAUAAGCAAGCGUGCUUGGUGGCUGGCAGACUGUUUUAACCUCCUGGUACAGCCAUGUUCAUGUCCCACGACCACCUAAGUCCCUCCUUCAUCUUG
UUACAUACAAUUCUUGAAAUUGUUACAUUCAAUUCUUGAAACCAUGCUUCUUGAUAAAUGAACAAAAGAGUGAAACCACUAUCAGAGGUCAUAACCAACU
UUGAUAUUCAAGGACAAAUUAUAGAAAUGAGAAUUAGAAAGAGGAUCUGCCCACAAAAAGUUUAGAAAGAGGGACAGCCUAGCAUGGGGGAUGAGAAUCA
CUCUCUUAGCCAAAAAAGGCUCACCAUCAUCCACAUUUCAUCCAUCAUACAUAUGGACUCCCUGAUGAACCAAAAAGUGGAGAUAGGCAAGCCAUUCUUG
GCUAAUAGAGAUAAGCUCCUCAUCUCCAUGUAAGGCUUCCCACCUCUUCACUUGCGA
>root_35
CAAGUGAAGAGGUGGGAAGCCUUACAUGGAGAUGAGGAGCUUAUCUCUAUUAGCCAAGAAUGGCUUGCCUAUCUCCACUUUUUGGUUCAUCAGGGAGUCC
AUAUGUAUGAUGGAUGAAAUGUGGAUGAUGGUGAGCCUUUUUUUGGCUAAGAGAGUGAUUCUCAUCCCCCAUGCUAGGCUGUCCCUCUUUCUAAACUUUU
UGUGGGCAGAUCCUCUUUCUAAUUCUCAUUUCUAUAAUUUGUCCUUGAAUAUCAAAGUUGGUUAUGACCUCUGAUAGUGGUUUCACUCUUUUGUUCAUUU
AUCAAGAAGCAUGGUUUCAAGAAUUGAAUGUAACAAUUUCAAGAAUUGUAUGUAACAAGGUAAGUAAAAGAAGGGCUGGAAGUGGCAACUGAUAUAGGCC
AUUUCUUGAACUACUUUCUGUAUCCUCCUCUUUGAGAUGUGGCGGUUCGGUGAUUAUUUCUCUUGUAAAGAUGAAGGAGGGACUUAGGUGGUCGUGGGAC
AUGAACAUGGC
>root_86
AGGUGACAGUAAAAGUUUUUUAAGAUGCACGUGACAAUAAAAGUUACUCGGAAGGAUCAAGAUUUCCUUGUUGCUAAUGGGAAAUCUCGACCUUUUACCC
UCUAUGGGAUUUUCUGAUCCAUUUUGAAUGGAAAAUGUUAACCAGCUAAUUUACUUGCUUUUAGAUAAUGGAGUAAGAAACAUACCAGAGUCUCUACAUC
AGUAUAUUGUGUGAUGUAUGUAGCUGCUCCAACCAACAUGCCGUAAUUUCUGAAAGUUUAGACCUAAAUUUGUUUGUAAGCUGAUAUCUAUGUUUAUCGG
CACUCAGCAUGUACCGUUACUGGCGAAAUAUGAAACCCUUGUGGUUUAAACAAGACACAUAUGUAGGACAUGAGAGAGAGAGAGAGGUGACAAGAAGAGA
AGUGAGCACACGUAGCUCCAAGCAUGAGGGCGCCAUGCAUGCAGGGAAACGACCACGUGUGCUCACUCUCUUCUGUCACCCUUCAUACCAAACCUUUAAU
```

UUCGAGCAGCUAUAUCUCUCCCAUGCUUCAAUGCUUGAGACCUUCAUGCAUCUGGUUUGCGCAAUGCAUUAGUUCUGCUGUGUGAGUUGUACAUGUAGCU
UGCUGCUUCCUUACUUGUCUUGCUCAUGUGUAACACACUCAUAUAUAGAUCUGUGAUUAAUUGGAGAGUACUGGAGUGA
>root_55
UGCAUAGCUAUUAGCUAACAUACUGCACUUGAAUUACACACUGAAGUACUAUUAUCUGAUAAUGUUCAGUUGUAAUUUAUAUUUACAGGUCUUGUUAGUG
UUGUUGUAGGCCUCACUAACAUUACUACUAGUAUAUAGCAUAAUCCUCGGGUAUCCUCUAUUCAGCACAACAAAACUAGGAGAAAGUCUUGAGUCUCUAA
CUCGAGCUAAUUGAUGAAUCUUCACGCAAAACAUACAAAAUUAGCAACUGUUGUAUGUAGCGAAUCUGUAUACUAGUUAUAGGUAGACAAGUUCUGAUCA
CGAGGUCACAUAUACAUAUGUUCAGAACUCCAUCCGUCCACAAAUGUAAGCAUUUCUGGUGUUCAAAAUUUGUCCCAAAAUAUAAGCAUUCCUAGAAAUA
GGGCAAGCCCAACCCUACACAACCCCACAUAGAUAUGUGUGUCAUAGUGCAAUAAUCAGGGAUAUCCAAAUCUUUUCUUUUUUUUAUAAUUUAUUCUUAA
AAUCUUAGGAAUACUUACAUUUAUGAACAGAGGUAGUACCUUCUUUGCGAGACUGGGAACCUUUAACCAUUACACAUGAUCCAGAGUUUACAGUGGUAAA
UAAUCAUUGAGAGAUAAAAAAAAAAGACAAUAACUAAACACACUCCUGUAUUUGAUCAUAAAUAGCUUCUGAAAACAUGAAGAACCUUGCUUAAUUACUU
AAUAUAGUCGAUCGUUACCCUGCAGAAAAUUAAGAUCGAAGAGAAACCAUGCAUGAAUCAGAUCACAGGUUAGGAAGAAUUAACAAAGUACAAUACAAAC
AUACAGAUCCACAUGUAAAUUUGUCUGUUACAAGCAAAGAGGUCUGCCAACGAAUUGAGUCCAAUCUAAAAGGCUCAAUUAACACAAAGAAGAGAUGGGC
CUGAUGAGUGGCCGACAGAUAGAGAGUGAGCACGCACAGCUCCCAGCAUGGCGCUUGCAUGCUAAGAAACCACCGUGUGUGCUCACUCUCUUCUGUCAAA
CCUUCAAAAACCCAGCUCCCCUUGUCUUCCACUUCAUGCAAACUGCAAGAACCCUAAGUACCCACGAACUGCAUGUGUGAUUCAUUAAGUGACGUAUUUA
UAUAUGGAUGUGUGUGUGCGUGCGCUAGCAUGAAAUGUCCCUAUGCAAAGAACCACCAUGGGUUUCUGGGUGCUCAAUAUGAAAAGAGAAUAACAAGCAG
CUAGAAGAAACCACCAUGUUUGCUCAAGAAAUAAAAGAUGGAGAGAGAGAGAGAGAGA
>root_29
GGGUGGGAAGGGAGGGGAGGUGGAGGCUAACAGAAGAGAGUGAGCACACAUGGUGCCUUUCUUGCAUGCUGAAUGAGAGAGUUCAUGCUUGGAGCUAUGU
GUGCUCACUUCUCUCUCUGUCAGCCAUUAGAGCUCUCUCUCUCUCUCUCUCUCCAUCUUUUAUUUCUUGAGCAAACAUGGUGGUUUCUUCUAGCUGCUUG
UUAUUCUCUUUUCAUAUUGAGCACCCAGAAACCCAUGGUGGUUCUUUGCAUAGGGACAUUUCAUGCUAGCGCACGCACACACACAUCCAUAUAUAAAUAC
GUCACUUAAUGAAUCACACAUGCAGUUCGUGGGUACUUAGGGUUCUUGCAGUUUGCAUGAAGUGGAAGACAAGGGGAGCUGGGUUUUUGAAGGUUUGACA
GAAGAGAGUGAGCACACACGGUGGUUUCUUAGCAUGCAAGCGCCAUGCUGGGAGCUGUGCGUGCUCACUCUCUAUCUGUCGGCCACUCAUCAGGCCCAUC
UCUUCUUUGUGUUAAUUGAGCCUUUUAGAUUGGACUCAAUUCGUUGGCAGACCUCUUUGCUUGUAACAGACAAAUUUACAUGUGGAUCUGUAUGUUUGUA
UUGUACUUUGUUAAUUCUUCCUAACCUGUGAUCUGAUUCAUGCAUGGUUUCUCUUCGAUCUUAAUUUUCUGCAGGGUAACGAUCGACUAUAUUAAGUAAU
UAAGCAAGGUUCUUCAUGUUUUCAGAAGCUAUUUAUGAUCAAAUACAGGAGUGUGUUUAGUUAUUGUCUUUUUUUUUUAUCUCUCAAUGAUUAUUUACCA
CUGUAAACUCUGGAUCAUGUGUAAUGGUUAAAGGUUCCCAGUCUCGCAAAGAAGGUACUACCUCUGUUCAUAAAUGUAAGUAUUCCUAAGAUUUUAAGAA
UAAAUUAUAAAAAAAAGAAAAGAUUUGGAUAUCCCUGAUUAUUGCACUAUGACACACAUAUCUAUGUGGGGUUGUGUAGGGUUGGGCUUGCCCUAUUUCU
AGGAAUGCUUAUAUUUUGGGACAAAUUUUGAACCACAUGAAUGCUUAUAUUUGUGGACAGAUGGAGUAUAUUUCAGGGUGUGUCUCCCGUUUUUCUUAAU
UUGUCCACAGAUUCAAUAAAGGAAUCUGCUUAUGCUCAUAUUUACUUGCCGUCAUUUCAUGUGCACAAUUGUGAGGCCUAUAUAUGCAGACAUUUUCUCU
UGUCGUGACUGCGGUUCCCAGCCUCUUGUAAGUAAAAUUAGAGACCGCUCGAUCAAUACAUCAGCAUAUCUAUCUUGUAAGAGCUAAACAUACGCGCGCG
CACCGAAGAAAUUAAGCUAUAGUUUAUU
>root_38
GGCGGUGGUUUCCAUGGCUAACUGACAGAAGAGAGUGAGCACACAACGGGCAGACUGCAUCGAUCUAAACCUUUGGAACCGAGGCGGCACAAGUACGAAU
GAUGCAGCUGCUGUUGCGUGCUCACUUCUCUCUCUGUCAGUCCUCUCCGGCCUCUCCCUGCCACCACUACUCGCCAUGAUCUACUGUGUGUGUGUGUGUG
UGUGUGACUGCAGCUUGCGCAGCCUGUGAUGAGUGUGUGGUUUCAGGCUUUCAGCGAUGUACGGGAUAAGGCCGGUUUGGGCGGGGUCUCGUCCUUAUCU
UGUACUGGCCGGCUACCUGCCUGAUUGCCCUACGUCUCGCCGCCCGAGGAGAGGCGGUGGUGCAGUUGGACCCGUACUUUGGGGCAAGGUACAUGUCCAC
UAGCCGUGCUCCUGCUGCGCGCAUGUCCCUGCUGCUCGCUUUCUCUCUUUCCUCUU
>root_67
GCCCAACUAAUGGGUUACACAGGCGCUCUAUUGUGCAUACAAUAAAACGACCAGGUGUUGCACUGUGCUAUUCCCAAUUUCACACAUGGGAGAUCAUAAA
AAGGACUGAAAACCCCCGGCGAUGAGCGAGCUAGGAAAACAAGUAGAUAAGCUUGGACCAAAGGGUAACUAGCUAAGCCCUGAUUUUAUCUCGUAGAUUU
CCGAGCUAGGAACACAAGAAACGCGCAUAUGCAAGGCUAAAACGAACACCAAAGACAACAACUGCAUGAAUGAAACCGCCAGUUAACUAAAAGAAAACUC
AUAGCUAGCUCAGAUGGCUGGUACAAGUGCCAUGAGAUGCAAAAUCCCCAAUCGCCUUUAGAGACGAAAACAUCCAUGGUCAAAUUAAAUUCCAAAACAA
GGCAAGUAACAAUAAAAGGACAACACUACAAGAAGGCCAAGUACGAGGAGCUAGGUAUGCACGCUUUAGCAGUAGCUAAGGAAGAGGGGAAGCAUGGACU
CACCGGAGAAGAAAACAUUUCCAGAUCUGGAAGCCGCCGCCGUCCCCGGUAGGUGCUGCACCCCUUUCCACUUGCAACCAACCAGAUCUGCCUUCCACCA
CCGCCAUGGCUGCUGCCCCCACCUAUCUCACAACACUCACAGCACCACGUGAGACGAGCAAAAUAAAAAGGGGAAAAAGAUCCAAUUUUUCGCUGUGAAA
UCUUUACGAAAAAGGAGAGGAGAGAAUAGAUAAGCGAGAGGGAGGAGGAAGGGGCCGAGGUAGCAAAGGACUAGUCAGACAGUGGUAUCCGUUAAGCACA
CCUGGGGAGGCGGCUUAACCUGGGGAGGCGAGCAAAGCGCGGUGUUAAUCCAUCCUCGGAAGGCACAUAGAUAGCAACGAGAAGCUCGCCGAGAAAAGAA
ACGUGAAUUGCCCGGCGCGACGAACCAUGUCGUGCAUGCGCGCAGAGCCUGCCAUGAAAUUAAGAGUAGUACCUUAUUAUAAGAGCAGGCAGGCCUUCGG
AAUCCGGGCCGGGGACAAGAUAAGGUUUCAGGUAGGGACACCACCAGGCCGGCCGGCAGACGAACAGUGCGGCCACGGCGAUCGAUACGAUGCAGUGGUG
CGCAACGAUGGAUGGACGUGGUAGGGGAGAGAGGAGAUGGAUGUGGCGGAACAAGUCCCGAGAGAGAGCUGACAGAAAGAGAAGUGAGCACGCAACAGCA
GCUGCGUCUAGGCAAACCAUCAUCGAUCCGUCAUCUUUAUCGCCGUAUUUAGAUGCAAUCUGGCCGCUGCGUGCUCACUCUCUUCUGUCAGCCGCACCAC
CACCAUCACCCCGUCCCAUCCCAUCGUCGGCCACCACUGCAAAAGUGCCGAGCAGAGGCAGCAAGAACUCGACACGAA
>root_99_part1
AAGCCAGUAGGAAGGAUAGCGCCCUCCCAGAUCUGGCCGGCUGCCGCCGCGUUCUUCCCCAAUUCGGGCAAGACGACGACGACGAAACGUAUGUAUGUGU
AACAACAGGGUUUGCUUCCUCUCUCCCUCCCUCCCUCUCCCUCUCUCUUCGUGUCGAGUUCUUGCUGCCUCUGCUCGGCACUUUUGCAGUGGUGGCCGAC
GAUGGGAUGGGACGGGGUGAUGGUGGUGGUGCGGCUGACAGAAGAGAGUGAGCACGCAGCGGCCAGAUUGCAUCUAAAUACGGCGAUAAAGAUGACGGAU
CGAUGAUGGUUUGCCUAGACGCAGCUGCUGUUGCGUGCUCACUUCUCUUUCUGUCAGCUCUCUCUCUCUCUCUCUCUCUUCCUUCGAGACUUGUUCCG
CCACAUCCACCUCCCCUGCUACCUCCAUCGCUACGCACCACUGCGUCGUGCAGCCGUUCUCGCUCGCAUAGCAUCGAUCGCCGUGGUGGCACUGUUCGCC
UGCCGGCCUAGCCGUGUCCCUACCUGGAACCUUAUCUUGUCCCCCGCUCGGAUUCCGAUGGCCUGCCUGCUCUUGGAAUAAGGUGUGCUUAACCGAAACC
ACUGUUUGACUAGUCCUUGCUAUCCCCUUCUCGCUCCCGGCCCCUUCCUCCUCACUUAUCCAGUACUCUCUUACUCUCGCUUUUUUCGUAAAGAUUUCAC
AGCAAAAAAAUGGGUCUCUUCCCCUUUCGUUGUGCUCAUCUCAUGUGGUGCUGUGAGCGUUGUGAGGUAGGUAGGGAGCAGCAAUGGCGGUUGUGGAAGG
CAGAUCUGCUUAGCUGCUAGUGGAAAGGGGCACCUACCGGCGGCGGCGGCUUCCAGAUCUGGAAAUUAAUGUUUCUUCUCCGGCCGGUUUCAGUGGAUGG
GUGCAUAUAUGCAACCCAGCUGUCUCGCCAUACAACAUGCGAGCUAUGAUUCUGAAAUCCAGUUGCUGUUUGCUCGAGCUCUGAAUUUGUUCUCUGAAAU
UUCUUCUAGUCCAGCAUGCCUAUUUGCAUUAUCUUUUGAAAAUAAAUAACAAGAUGGAGAUCAAGACUAGGUAAAUAUCAAUUGGGGAUGGCAAGGGGAA
AAUAUGGUUGAGCUAGUCGAAGCUUCUCAUAGAACUAAGUUCCCAACAACAUAGGACAGUUGCUAUGCUUUAUUAGUACCCAAGUUUUUGAAUCACGUUC
UGAACUUCCUGGUGAUCUUGGUUCAGAACCAUUUCAAUUACGAUUUGGAGACAGAUCCCUAGUAUCCACUGGGAUUCUGGCAACCGGUUGUUUUCCGAUU
UCCAUGUUGAUAACUGUUACGCUGGUUUCGGAACCCUGGGGCAAUCACCAUAAUUACCGUCGGUGCACAUCCUGUCAAUAUUCAAGUCCUAAGUAAGGUU
CAUUCCGGGUCUCUACAAGGGCCAUGUAUAUGACGCAUGUGAAAUGAGCAGGCUAUACAGUGUGAUCAUCAUUAAACUAAAUGACAUUGUACAAAGGAUC
UAUCCCUUUUUGUCCAUGCUAGAUCGAGAUGAUUUCUAGUCGGAGAAAUGCAGCACGUAGCUACUCAAUUCCAUUGUGUGAUCUAGUUAUGCUGGGGUUA
UCCCUUGAUGCAUAUUCUUGAGCAAGUUCCUUUCUGUUUUUCGUUGUUUCUUGAGGGCUGGAAGCAUCUUUUGUUUAUUUGAUCCUAUCUAGAGAUUUAU
AUUUUUGCACCUGUUAUAUCCUAAAACUGCAGAGUGCAAUUGGAUUUCUCCAGAAAUUUUGCUGAUAUAGUUCUGGGUUCAACCAUGCAGUUUCUCUUGC
AGAUUGCAAUACGGGCAUUGAGCCUCCCUGCCUGGAAUAACGUUGAUAUGGAAACUCUAGGUUUGGAUAUGCUGUUUACGUACCAUCAGAUUCUUCAUCU
CUGUAAAGGCUCCAAGCAAAAGUUGUCACGCACGUAAAUACGGGGGCUACAUCUGCAUACAGUUAUACCACUCUCGGUGACCUGAUUUUUUUCUUUUUAU
UUAUUGACUGAACUGUAGGGUUUCCCCUACAGGUUAUAUAUUAAUAAUAAUAAUAGUAAUAAUAAAAUGUAAUAAUAAUAAUAAUAAAAUAUUACAAAAG
GAACAGAAAAAACAAAAGAGCAAGAAGCUAGAAAAAACAUCUAGCUAGAGGAAAGUAUUCCUUUGUUCUCUAUGAUAACCAUGGCAAAUUCUCAACAAAA
CUUGGUUCCGCUAACUUCAAUAGUCGCCAGCUCUUGAUUAAAAUUUUGGUCAUUGCUUGAGGUCAUGUGCUCCAGCUCAAGGGUAUGAUGAUUUCCAUGU
AAAAUGUCACAUCGAGUUGCUGUUUGAAUUCUUCCAAAACUAAGAAAGGCUGAACUUGAUCAAUGUGCAAUCCAAGGAGUGUCCAGCAGCUUUGGCAAA
ACUACAUUAGGAAGAGAUGUUCCACCGUCUGAGUUUCAGAAGAUGAGCAGAACACAGUGAUACGAGGGAAGCUCCAUAAUUUUACGCUAUAAUAAAAUCA

```
CCUUGUGUUUAGCCUAUCUUUGAUCUUGUGGCGCCAUGAUUCAUGGUUAGCUAUGCUCUUUGAGCAAGAGAGAAUGGAUGGACGAGGAGUGUGCUCACCU
UGGACCUCAUUUGGCCGAAUGUUAUAAUGGGGUCUUUGGUGGCCACCCUCCCUAAUAGGGGAAAAGUGUCCUAAUUUUCUAUUGCCAUGUAAUUAUAUUU
CUUCUAUUUUGUAUGGCUUUAUUUUUAAACUAAGUGACACAUCACUUUUGAUGUGACUUUUUGCAUGGUUUUGUGGCUUGAUGAUUUGACACCUAACCGG
UCUAAGUGUCAUUUAAGGGCAUUGUGGUCACUACAAAAAUAAUAAUGGCAUUUUGAUUAUUUGGUGAAACUUGGAUGCUCAUUUUUGUAGGCUAAAGUUG
UGCACAGUUUGCACAUAGGUCCUUGGCUAUUUUGAUAUUUGCACUUGGGUUUUUGUUCUAGUUGGUUUUGUAAUUUUGGCGAUCUCUGAGAGCUUUUGUG
>root_68
CAUCGCCUCCCCUCCUACAUCGAACGAACCAGACACCAAGAAGACCAGAUUCUUCUCUCCGUUCUUCCUCCUCCUUCUCCCUUCCAGUUCCAGUACAGCC
GCCGCUCACCGGAUUCAAGAAGAGCCGUCUCAUCUCCCUCCCUGCUAAUCGCUCCCUAUUUCGUUUGCGUUUGUUUGGUUUUAGCAGCUAGCGCUUGAGG
CGUGGGAAAGGCCCUCAAGAGAUUGACAGAAGAGAGUGAGCACACGGCGCGGUGGCCGGCACCGAACGGGUGCCGUCCCCGCCGCGUGCUCGCUCCUCUU
UCUGUCAGCCUCUCCCUCGGUCCUGGGCCGCGAGAGCCGGAGCCGGUCUACGAUUCAGUCGUCCUGAUCUGGGUUGAGGUGACAGACGCUAAGGCCAGCA
GUGGAGGAGAAGAGAGAGAAGCUUUUGCAGCUGUGCUGUGCCGCAGUGGAAACCGUGCCGGGCAGAUAGAGAUGGACACGCAUGCCGGGAGGGGAGGACG
ACGCUGGCUUUUGCAGGCUUGCAAGGGCAUGUCACUUCGUUAUCUUCUAGAUAUACGCAUAUAGAUUUGCGGACGAUUUGCAUAUUUUUUGCUCUCGAUU
UCGAUCCCUUUCGGGCAGGCUGUCGGGGAGCUACUACCGAGGAGGAGGAAGCAGCUGAUAUUAAUGGCACGGCAUGGACUGAGGAGUUACUACUCCCAGG
AACAAGCGGUGGGUUGUGGCUUUGCAGUACACUGAAAAAUGAUUGGUUUCCCAUUUAUUUUGGCUUGCUCGUCCGCAAGAUUGGAUUUCUCCUCCCAGCU
UCUCGCUGCCUGCCUUGGGAGCUGAAUCAGGA
>leaf_58
GCGCGCCUGCACGCGCAAGUAGCGCACGAAAACAACCAGAAAAUGCACGCGUUGCGGUGAAUUUACGAGAAGGGGACGCUUGCAUGGGCACAGAGAGAUG
AUGGAUGGGUGGCCGGACGGCAAUGACAGUGAGAGCAGAGAGCACACGGCCCUCGCAGAAGCGAGCCGUCCUCGCUGGUGCUGGGUUGUGCUCUCUCUCU
UCUGUCAUCACUCUCGGUCCACUCCACCGUUUCUCCAGGGCCGGCGGCAGGCAUCCCCCGUCUCCCUUCGCCGCGAUCCCUGGCUCCCUGCACCAACAAG
AAGGAAUUCAUCCAUGAUCCGCAUGCAGCGAAACUCGCAUCAGAAAACUAAACUGUGAAAAACAUUGCUCAACUUCGCGAAGAGGAGAAUCGUGAUCGAG
AGCGAG
>culm_61
UUUAAAAAUUAAUAAAUUUUUUUAGAAAAAGACAUUCAGUCAAAAUUACUAUAGUAAAUUAUUGCUGAUAAAUAAAUAAAAGUAGAAGAAAAUACAACAC
AUAUUAUAUGCACACAUGGUCAUGUCACGUAUUCGAAGGAUCAUGCUUACGCUCCCUGCAGCGCAACUUUGUGCUACUGCCAGCACCGGCAAACAUCAAU
GGCUGCAUACAGGUGGCCAGGUGCUGCAAACCAUCUGAGUAGACUGGAGUAGUUAAGUUCCAAAUUCUAGCCAAAUAGAAAUUUGCUCUACUUCCAUCAA
AAUCACUAUUAAAUAUUGGUUUCAAGCGAGAGUUUUCCCUGAGCACUUAAUGCAAGAGAGAUGGAACCGAUUUUUGCCUGAUUCUUCUUCCCCACAACAA
GCUGCAAGACGUGUCGAGUACAACAUGACGUCUCGCAGAAUCUAUCGGUCUGGGGAUAUCUCUUUCAGUAUUCACUUCUUGAACAUCAGCUUGUACGGUC
AGAUUAGACCGUGCGUGUACGACUGUACGUACGUACACAUGCAUCUCUCGCGCGGUCGCCUGCAGCUUGCUCAUUCCAGAUUCCAAGUUCCAAAAGGUUC
UGCUCUGAAAAGUGAAAAGCCUUUGUUCCAUAGGCGGUUAAACACUUAAACAGGUGCGCUAGUAUAGCAUCAUAUUCGAUGAGAAAUGAUAGUAUCUAAU
CCAUAGACUAAACGACUACUUCUGAGUUAUAGUAUGUAUGUAUUGUCUUACUAAAGAGUACUCUGAUGUAUAAAUCGAUUUUUUUCAAUUCAGUCGAAA
ACUAAGGUAAAAAUAUGUUCUACUAGUAUUCAGCAAGUAGAGGUCGUUGCCGACAGAUGACAGCUAGUUAGCACUGACAUGUACAAAGUAUGAGAGAGAG
UUCAGCGGGUACAUUCAGGGCGCAUAGAAACUGCUUGUAUGAUUUAUGAAACUAUCUAAUGAACAAUGAAAUACAUUGCACGUUCUUAAAAAAAAAAGAU
GUAUGGAAGUAUCAAACAUCAUGGUAUAACAUAGAGAUCCUAUACAUUUGUGAUUUGCCUCAACAUGACUAUUUUCAAAGAAACCCUCACUUACAGAUAG
GAUGGACUGCCACUUUAGGUAUAAAAUUCAGGGGUAUAAUCUACGAGUACAUUAUGAUUUAUAUUGUUUGUGUUUGUAUUACCAUUUAGGAAUUAGAUAA
GCACACAUUUGUAAAUUCAAUCCAAAGAGAAAACUCCGAAGACAUAGCAUAUUCUUUUAUGAGGAAACAUAUCUGAGAAGCAUACGAUAAGAACAAUAAU
CAAUUAUACCUAAGAACAGUGGAAAUAUAUUUGGGAAGGGAGUGCUAUACAUUUUUUAAACUAAAGGAAAGAACAGAUGGACUGAUGAAGGCCUAGCUUA
UAAGUCUUCAAAUUUCUCGGUCAAACAAACUACUGGUAAAUAGUAACUGAAGCUUGUAUCUAUUACACCUGGCCAUGUGCAGCAGAAAUUUGAUGUACGU
CAGUUUUCCGAAAUGAAAUACAGUUAUAUCCGUUUCAAUUGCGCACUAGUCCAGAAGAGAACUCAAGAACUAAACUACAAAAAGACCGUCACAUAGAGGU
GUGUCGUGUGCACAUCUACAGGUUUGUUUCCAUCUCCAUAAGAAGUUGGGCAAAACUAAACAAAAGGUAAAGAAUUGUCAGAUCAUCGACCAAUUGAUCU
AAUAAAUAUAGCAAAGAACGGAUGCUCUAUUCCAGAAAAAUUUGUAAGCAUCUCUUGGCCAUUUCUAUCCACUCAAGGGAAACCAGAAAAUUAACAUUCA
AAAUUAGCAACAGGCCAUCUACCAAACAUAUCUACUACACGAAAGAAAGGACAUGUUUUAGAGGACAAUAAAGAAGGACGAGUUGUAAAUUAAACCCUCU
CUCAUCAAUGAUCUGGAUCAAGAGGUUGAAGAGGAACACCCACCUAUUCCGAAUGCUAAAUUCUGCCCCCCCACCCCCCUGCAUAACAAAUUCAAAACCA
AGCAGGCGCAGAUAGCGCGUCCUCUAUGUAUGCCAACAUGAAAUAUGUGCAUCAGCAUAUCAAAAAACAACCAAAAAUCAACAGAUCAAAAGACAGAGAC
GAGAUGAUCAUGGUUACGCAUGCUUGGCUCCUUGCACGCCACCCACAGCAUCCAAUCGCGCCACGGAUUGGGCUACAUGUGUGGCAUACAGGGAGCCAGG
CACACACCAGCUGAUCCAUCGACCCAACCUUAUUGGCUUGCCUCCAUGUCGAUGAACAAACCGCAGCCGGAUGGAGAUCAGUCCCCUCCAACACCCCGAA
GCCAGCUAGCAGAAACGCAGAUGCACAAGCAGGAAGGUGGAGAGAGCAAAUCUUGGCCAGCAAUAAGAGACGAGGAGACUGGAGAAACAGGUAGAAAGCA
UGGGCGGCUAAGGGAGGAG
>root_15
CCUUCCUCCCAGUGUUCUCCCCUAGCCGCUCAUACUUUCUACCUGUUUCUCCAGUCUCCUUGUCUCUUGGUGGCCACCAUGAUUAUUUGCUCUCUACUCC
UUCCUGCUCAUGCAUCUGCGUUUCCGCUGGAUCGGGAUGCUUGAGGCGAUCAAUGAUCCCCAUCGGGCUGCGGAUUGUUCAUCGACAUGGAGAGAAGCUG
UCAGGUUGGGUCGAUCGAUCAGCUGGUGUGUGCCUGGCUCCCUGUAUGCCACACAUGUAGCCCAAUCCGUGGCGCGAUUGGAUGCUGUGGGUGGCGUGCA
AGGAGCCAAGCAUGCAUAACCUGGAUCAUCUCCUCUCUUUUGAUCUGUUGAUUUUUGGUUCUUUUUGGGAGAUGCUGAUGUAUAUAUUUCAUGCUAUUGC
AUAUAUGCCUUUCUGUGUUUGCUUGGGUUUGAAUUGUUUGUGCAGGGGGCAAAAUUUAGGAUUCGGAAUAGGCGGGUGUUCCUUUUCGACCUCUUGAUCU
AGAUCAUUGAGAGAGGGUUUACAACUCGUCAGCUCGUACUUUUUCAUCCUCUACAAACAUAUUCUUUCUUUUUCAUAUAGUUGAUAUGUUUGGUACAUGG
CCUCUUUCUAAUUUUGAGUAUUAAUUUUUUGGUUCCCCUUGAGUAGAUAGAAAUGGCCAAGAGAUGCUUACAAAUUUUGCUGGAAUAAAGUAUCCAUUCU
UUGCUAGAUUUGGAUCAAUUGGUCGAUGAUCUGACACAUUGACAGAUAUUUACCUUUUGUUUAGUUUUGCCCAACUUCUUAUGGAGAUGGAAACAAACCU
GUAGAUGUGCACACGACACACCUCUAUGUGACGGUCUUUUUGUAGUUUAGUUCUUGAGUUCUCUUCUGGACUAGUGCGCAAUUGAAACGGAUAUAACUGU
AUUUCAUUUCGGAAAACUGACGUACAUCAAAUUUCUGCUGCACAUGGCCAGGUGUAAUAGAUACAAGCUUCAGUUACUAUUUACCAGUAGUUUGUUUGAC
CGAGAAAUUUGAAGACUUAUAAGCUAGGCCUUCAUCAGUCCAUCUGUUCUUUCCUUUAGUUUAAAAAAUGUAUAGCACUCCCUUCCCAAAUAUAUUUCCA
CUGUUCUUAGGUAUAAUUGAUUAUUGUUCUUAUCGUAUGCUUCUCAGAUAUGUUUCCUCAUAAAAGAAUAUGCUAUGUCUUCGGAGUUUUCUCUUUGGAU
UGAAUUUACAAAUGUGUGCUUAUCUAAUUCCUAAAUGGUAAUACAAACACAAACAAUAUAAAUCAUAAUGUACUCGUAGAUUAUACCCCUGAAUUUUAUA
CCUAAAGUGGCAGUCCAUCCUAUCUGUAAGUGAGGGUUUCUUUGAAAAUAGUCAUGUUGAGGCAAAUCACAAAUGUAUAGGAUCUCUAUGUUAUACCAUG
AUGUUUGAUACUUCCAUACAUCUUUUUUUUUAAGAACGUGCAAUGUAUUUCAUUGUUCAUUAGAUAGUUUCAUAAAUCAUACAAGCAGUUUCUAUGCGCC
CUGAAUGUACCCGCUGAACUCUCUCUCAUACUUUGUACAUGUCAGUGCUAACUAGCUGUCAUCUGUCGGCAACGACCUCUACUUGCUGAAUACUAGUAGA
ACAUAUUUUUACCUUAGUUUUCGACUGAAUUGAAAAAAAAAUCGAUUUAUACAUCAGAGUACUCUUUAGUAAGACAAUACAUACAUACUAUAACUCAGAA
GUAGUCGUUUAGUCUAUGGAUUAGAUACUAUCAUUUCUCAUCGAAUAUGAUGCUAUACUAGCGCACCUGUUUAAGUGUUUAACCGCCUAUGGAACAAAGG
CUUUUCACUUUUCAGAGCAGAACCUUUUGGAACUUGGAAUCUGGAAUGAGCAAGCUGCAGGCGACCGCGCGAGAGAUGCAUGUGUACGUACGUACAGUCG
UACACGCACGGUCUAAUCUGACCGUACAAGCUGAUGUUCAAGAAGUGAAUACUGAAAGAGAUAUCCCCAGACCGAUAGAUUCUGCGAGACGUCAUGUUGU
ACUCGACACGUCUUGCAGCUUGUUGUGGGGAAGAAGAAUCAGGCAAAAAUCGGUUCCAUCUCUCUUGCAUUAAGUGCUCAGGGAAAACUCUCGCUUGAAA
CCAAUAUUUAAUAGUGAUUUUGAUGGAAGUAGAGCAAAUUUCUAUUUGGCUAGAAUUUGGAACUUAACUACUCCAGUCUACUCAGAUGGUUUGCAGCACC
UGGCCACCUGUAUGCAGCCAUUGAUGUUUGCCGGUGCUGGCAGUAGCACAAAGUUGCGCUGCAGGGAGCGUAAGCAUGAUCCUUCGAAUACGUGACAUGA
CCAUGUGUGCAUAUAAUAUGUGUUGUAUUUUCUUCUACUUU
>root_72
GGUACUCGUAGGAGUAAGUAGUAGUGACAACAAAAAGAUAGAGGGCAGGCAGGCAUAUCGUCCCUCUAUAAAUCCAGCCUCUCCCCCUUGCUUCUACCUC
ACCCUUCCUCCCACUGUUCUCUCCUCCCUUAGCCGCCCAUGCUUUCUACCUGUUUCUCCAGUCUCCUCGUCUCUUAUUGCUGGCCAAGAUUUGCUCUCUC
CACCUUCCUGCUUGUGCAUCUGCGUUUCUGCUAGCUGGCUUCGGGGUGUUGGAGGGGACUGAUCUCCAUCCGGCUGCGGUUUGUUCAUCGACAUGGAGGC
AAGCCAAUAAGGUUGGGUCGAUGGAUCAGCUGGUGUGUGCCUGGCUCCCUGUAUGCCACACAUGUAGCCCAAUCCGUGGCGCGAUUGGAUGCUGUGGGUG
GCGUGCAAGGAGCCAAGCAUGCGUAACCAUGAUCAUCUCGUCUCUGUCUUUUGAUCUGUUGAUUUUUGGUUGUUUUUUGAUAUGCUGAUGCACAUAUUUC
```

AUGUUGGCAUACAUAGAGGACGCGCUAUCUGCGCCUGCUUGGUUUUGAAUUUGUUAUGCAGGGGGGUGGGGGGGCAGAAUUUAGCAUUCGGAAUAGGUGG
GUGUUCCUCUUCAACCUCUUGAUCCAGAUCAUUGAUGAGAGAGGGUUUAAUUUACAACUCGUCCUUCUUUAUUGUCCUCUAAAACAUGUCCUUUCUUUCG
UGUAGUAGAUAUGUUUGGUAGAUGGCCUGUUGCUAAUUUUGAAUGUUAAUUUUCUGGUUUCCCUUGAGUGGAUAGAAAUGGCCAAGAGAUGCUUACAAAU
UUUUCUGGAAUAGAGCAUCCGUUCUUUGCUAUAUUUAUUAGAUCAAUUGGUCGAUGAUCUGACAAUUCUUUACCUUUUGUGACGGUCUUUUUGUAGUUUA
GUUCUUGAUUUCUCAUCUGGACUAGUAGUGCGCAACUGAAACGGAUAUAGCUGUAUUUCAUUUGGGAAAACUACUUCAAAUUUCUGCUGCCAAUGGCCAG
GUGUAGUGGAUUCAAACUUCAGUUACUACUUACCAGUAGUUUGUUUGACUGAGAAAUUUGGACUUAUAAGCUAGGCCUUCAUCAGCCCAUCUGUUCUUUC
CUUAGUUUCAAAAAUGUAUAGCACCCACUUCCCAAGUAUAUUUCCACUGUUCUUAGGUACAAUUAAUUCUUGUUCUUAUCGUAUGCUUCUCAAAUAUGUU
UCUCCAUGAAGGUAUGUGCUGUUUCUUCGGAGUUCUCUUUUUGGAUUGAAUUUACAAAUGUGUGCUCAUCUAAUUCAGAAAUGGUAAUGCAAACACAAAC
AAGAUAAAUCAUAGUGUACUAGUAGAUUAUACUCGUGAAAUUUAUACCUGCAGUGGCAGUCGAGCCUAUCUGUAAGUGAGGGAGGGUUUCUUUGAAAAUA
GUUAUGUUGAGGCAUGUAUCUACACAUUGCAAAUCACAAAUAUAUGAUCUCUAUGUUAUACCAUCAUACGAGCAGUUUCUAUACGCUCUGAAUGUUUCCC
ACUGUAAACCCACAAAUCACUAUACCAUAUGUUUUUUCCUGCAAAAAGUCUACAGAUACUUUGAAAGUUCUUAUUAAUAGAGAUGUCAUGAAACAUGCUU
CAUUAAUAUUGUACAUGCUAUACGUAUAUAGUAAUUUGUGAGUUACAAGUAAAUGGAAAGGGAGGGAGCACAUGCCGGUGCUGACUAGCUAUUAUCUGUA
GGCAACGACCUCUACUUCCUAAAUAGAACAUGUUUUUACCUUGAGAGUUACACUGAAAUGAAAAUGAUUGAUUUAUACAUCAGAGUACUCUUUAGUAAGA
CAAUACAUACAUACUAUAACUCAGAAGUAGUCGUUUAGUCUAUGGAUUAGAUACUAUCAUUUCUCAUCGAAUAUGAUGCUAUACUAGCGCACCUGUUUAA
GUGUUUAACCGCCUAUGGAACAAAGGCUUUUCACUUUUCAGAGCAGAACCUUUUGGAACUUGGAAUCUGGAAUGAGCAAGCUGCAGGCGACCGCGCGAGA
GAUGCAUGUGUACGUACGUACAGUCGUACACGCACGGUCUAAUCUGACCGUACAAGCUGAUGUUCAAGAAGUGAAUACUGAAAGAGAUAUCCCCAGACCG
AUAGAUUCUGCGAGACGUCAUGUUGUACUCGACACGUCUUGCAGCUUGUUGUGGGGAAGAAGAAUCAGGCAAAAAUCGGUUCCAUCUCUCUUGCAUUAAG
UGCUCAGGGAAAACUCUCGCUUGAAACCAAUAUUUAAUAGUGAUUUUGAUGGAAGUAGAGCAAAUUUCUAUUUGGCUAGAAUUUGGAACUUAACUACUCC
AGUCUACUCAGAUGGUUUGCAGCACCUGGCCACCUGUAUGCAGCCAUUGAUGUUUGCCGGUGCUGGCAGUAGCACAAAGUUGCGCUGCAGGGAGCGUAAG
CAUGAUCCUUCGAAUACGUGACAUGACCAUGUGUGCAUAUAAUAUGUGUUGUAUUUUCUUCUACUUU
>Bud_67
AAGGGUUUUGCUAGAGAUCUUCAGUAAAUCUAGAGCAAAGGUGACGAUGACGCUUAAUUUGGGAUGUGCCUGGCUCCCUGUAUGCCACUCAUCUAGAGCA
ACAUUGUUAUGAGGUUGCCUAACGAUGGAUGGCGUGCACGGAGCCAAGCAUAUCUCCAUACUCCCGAGCUUGUUCUUGACCCCCCUUAGUCCCUCCUCCC
ACAUCUUUCCCAACUUAUUAGAUUCAUCAUUUCUGUUGCUCAUUGUUAUACAUGUGUACUUUUAGUUCUCUGGCAUUUCAACUCUAGCUCAGUGAUGUAC
AUUGGUUAUGUGCAUGAUAUUGUCUAGAAAAGGCUAGUUUUCUGCUCUAUUCAGAUAAGUUAUUUGUUAACCUCCGUAGUACCCAUGUUUUUUAUGCCCA
UGCUUUAACAAGAAAUUUCGUCGCAAGAUUUGUAUGGCUACAAUCAGAUAUUAAUACAAUUUAUUUCUCACAAUUAAUUAGCCACAACAAACAUUUAUUU
GGGGUCAUUCUUGACUGUUUAUUGCUGAACCAAAUAUUGUGCUAAUGCAUUUUCACAUGUUUUUCUUUUUGUAUUGGCAGUAUGCAACGCAAGCAUAAGU
GAACAAUAUAUUUUUUUUAAAAAAAGGGACUUAUUUAGUUGCUUAUCAUGACCCAUGCUCAUUCUUGCUUAUAACUCAAAAUCUGGCAACGUCAUCAUGU
CAUUACUAACGUUUUGUAUCCCUUUAUAAUGCUA
>root_12
UGCUUGUCUAUACAUACCCAUGCUCCUCACACAUAGCUUCCUGCAAAGGAAGAGAGUUCAGAAGACGAGAGGAGAGAGAACUGAGAGAUUGGGAAGGAUA
UGAAGCUAUUUGCUUCUGAGUGGAAUGUUGUCUGGUUCAAGGUUUUGCCUGUGAUUUGAGGAUGAUGUAUGUGUAGUUUUUAAUUCCUCGAAUCUUCGGG
AUCUCGGACCAGGCUUCAUUCCCCUCAGGAAUAGCUUCAAACAUGAUAUUCAGUGGUGGAUAUAGAAAAAUUGUUCUUGUCCUUUUGAAAUAUUUGUGAG
GAUUGUUCAUGUUGUUCUUCAUGUGGUGUGGAGUGUGCUUUCACUUCUGGAUCUUAUCUCGCACUCCAAUACAAAUUUUUGUUUGUUUGUUGUGAACCGA
GGAGUAGCAAGAUUGUGUAAUGAUGGCCUGCUUUGACUCCUCUUUCCUCUGUAUAUCUCGCAGGUGCUGAAAGUUUUCAUCAUUACCUUUUGAUUGGGAG
GAUCUUGGCUUUCUCUCUCCUCUCUCUCCAUUUUAAUUUCUCUCUCUGUGCCUUUUUGUUUCCAUUUCCUUCUUCUCCGCUCGAUCUAUAUGCAUGCAUG
AUGCAUGGAGUACUCACAACGUUUUCGAGAACAUCGUAUUUAAAACUGGUGCAAACUUAUAUAUUUAUCUGCAUCUGGUUAUUCUUGCUUUCAGAUAUCC
AUCAUUUCUAGGUUUGAAGAUAUAUAGUCUUGGACUCCUGGUGCUGACCUUCAAAGGUUUGAGGCGUGUGAUUCUCAGGACAAAAGAUGCAGUAUGCGUG
UGUAUUUUUAUUCAGCUAUUAUAUGUUGGGCCCUGGAGCGAAUUACAUCGUUGUAUAUCUAAGUACUCAGUUGUAUGUUUGCUCUUCUCAGCAGGCCUCU
CAUCUGUGGUUUCUUGAUGUGCAUGUUCAGAAGCUGUAGGAGCUCGUUUGAUGUUGCGUCUAGAUUUGAAUAGUGCGACUUGUCUUUUGCCUGCAGCUGG
AUUAUGGAUCUAAGAAGUAAUCUGAGCUUCUUUUUUCUCCCUUGUUUUACUGUAUAAUCUUUUAGCUAGCACACAAGACUAUUCGAUGUGCCUUGUACUU
UGAUUGAGCAAAAGGUAAAUGUAUAACCUUAUGUUGCGUGAACACUUGAUGUUGUCUGUGUUAACAGUUUCCCUCUUUUAAUUUUUUGUGUAACUUGAUC
UCAUUUCUAAAUUGGGUUUAGAUAUGCUGAGACCCUAAGGAGCU
>root_13
UGCUUGUCUAUACAUACCCAUGCUCCUCACACAUAGCUUCCUGCAAAGGAAGAGAGUUCAGAAGACGAGAGGAGAGAGAACUGAGAGAUUGGGAAGGAUA
UGAAGCUAUUUGCUUCUGAGUGGAAUGUUGUCUGGUUCAAGGUUUUGCCUGUGAUUUGAGGAUGAUGUAUGUGUAGUUUUUAAUUCCUCGAAUCUUCGGG
AUCUCGGACCAGGCUUCAUUCCCCUCAGGAAUAGCUUCAAAUAUGAUAUUCAGUGGUAGAUACUGAAAUUUUGUUCUAGUUCUUUUGAAAUAUUUGUGAG
GAUUGUUCACGCAAUUGUUCAUGUUGUUCUUGGUAAGGCGCGGAGUGUACUUGCACUUCUGGAUCUUAUAUCGCACUCCAAUGUGUCUUUUUGUUUGUUU
GUUGUGACAACCGAGGAGUAGCAAGAUUGGUGUAAUGAUGACCCUGCUUUGAUUCCUCUUUUAUUCUGCGUAUCUGGCAGGUGCUGAAAGUUUUCAUCAU
UACCUUUUGAUUGAGAGAAUCUUCACUUUCUCGCUCCUCUCUCCAUUAUCUCUCUCUCUCUGCUUUGUUUCUAUUUCCUUCUUCUCCCCUCUCUCUAUGC
AUGCAUCGUGCAUUUGGAGUAAAUAUUCACGAAGUUCUCGAGAACAUAGCAUUCGAAGUUGGUGCCAACUUAUAUAUUUACCUGCAUCUGAUUAUUCUUG
CUUUCAGCUGAUAUUUAUCUUUUCUAGAUUUGAAGAUAGUCUUGGACUCUUGGUGCUAGCCUUCAAAGUUUUGAAGCGUUUAAUUCUCAGGGCAAAAGAU
GCAGUAUGUGUGUGUAUUUUCUCCAGUUAUGUUGGGACCUGGACCUCUACCUAGACCAGGACGAGUUCCUAAUUCCUCAAGCGAAUUACAUCGUUGUAUU
GUGUAUAUAAGUAGUUGUAUGUUUGUUGUUUCUCAGCAGGCCCUCUUAUCUGUGGUUUCUUGAUGCGCAUGUUCAGUAGCUGUUGGAGCUCAUUUGAUGC
UGCGUCUAGAUUUGAAUAGUUCCACUGCUUGUCUUUUGCCAUCAUCUGGAUAUGGAUCUAAGAAGUAAUCUGAUUUUUUUGCCUUGUUUUACUAUAUAAU
CUUUUAGCUAGAACGCAAGAGACUAUCCGAUGCAGCUUGUACUUGGUUUGAGCAAAGGCAAAAUGUAUAACCUUCUGUUGCGUGAACACUUGAUAUUGUG
UGUGUAACAGUUUUCCCUCUU
>Bud_57
GAGUCGAUGAGCGCCUGCGCCGCCUCCGACGCGCCGCCGCCCGCCGGGUCCGUCUUCAUCGCCAGGUACGACGCGAACCCCUUCCCCAUCGCCGACGCAC
CCAGCGAGGCUCCCGCAAAUCUCUCUCUCUCUCUCUGUCUGGAGUACUUAUCUUCUUGCUCGUCUUCCUCCAUUAGAGGCCGGGUAGAGGUGCACCACAA
GUUGGUGAAGCUGCCAGCAUGAUCUGAUGAGUGGCUAUGCAGUUCACAGUAAUAUUAUAUUAGUGAUCAAAGCUAGCUUCCAGAUCAUGCUGUGCAGUUU
CAUCUGCUCGUGGAUGCACGGCAGGUCGAAACUGCAUCAGCGCGCCAAGGUAAUUCACAGAUUUCCCGCAACACGUUGGCUCUUUCAUGUUUUUCUGUAA
UAUUUCUUGGAACUAGGUGAUAUCGGCGGAGCAUCAGUCGUAGCACAAAAUGAACAAGUCAAGAAUAAUCGAAGGUGAAAAUUUACAGCUAACGGUAAGC
AAACAUAGUUCUUUUCACUUUCUUAUUCCCUCUACUUGCAUUAUCAACACGACUAGCUUCCCCAGCUACUUUUCUUGUUCCUCCAAUAUGAUCUGCGCAG
GUGUUGUAGUGAUCUGAUUGUGAGAAGCUAUAUAUAAUAUACGGGACCAGUAAUUAGAGGGGAACCUGUUCUUGGAGUUAAUUGGGUUCUCAUGCAAGGA
AUGAAUGUUACUCAAUUGAGAAGUCAAAUGAUCAAUCAUGACGAAUUAACUAUUUCUUUUGAGUAAUCAUCAUGAAGAAGUAUCUCUAAUUUUAUCAAUC
UAACUAAAGAUUCGCAUAAGUAUAUUAGUUUGUGUUUCUGUACACACAUAAGUAUGUGUUUGUGUAAUUUGUGUGUGCCACCCACCUGAAUCUGUAUUCU
UUCCCCUUUCUUUAUCUAUUACAUCAGAUAAUUUAUUGUCGAUAUGAGUCUGCUAAUUUUCAGAAUUAAAUAGAGGUGCUCUAUUGUUAAAUAAUGGGAU
AUUAAUAGUCCAGCCAACUUGCGGCUGUAGUAGAAAUGGACCGAGAAUCUAAUGUGGUACACACCACUCAACCGACUAAUCAAUCAGCCAUUAUUUAACA
>leaf_49
AAAAAUAGGAAAGUCUAUCUCUUCACUCAUGAAGUUAAUACAAGUGAGGAUUGAUUUCUGUUAUUCAUAUAAUUCAGAAGAGGAAGACUGUCUUCUCCGG
CACCACAAUGGUGGCAAGGAAAGAACGGGCAGGUAAAGGCGAAGAGCCCCACCAAACAAGGAAGAAAGCUAGCCCCGAUCGCCCUACGCUAUUUAGCUUU
UCACAAUUCGAUCACUACAACACUGUUAGAUGUUAAUUUUCACCUUCAAUUGUUCUUGAGUUGUUCAUUUUGUGCUACGACUGAUGCUCCGCCGAUAUCA
CCUAGUUCCAAGAAAUAUUACAGAAAAACAUGAAAGAGCCAACGUGUUGCGGGAAAUCUGUGAAUUACCUUGGCGCGCUGAUGCAGUUUCGACCUGCCGU
GCAUCCACGAGCAGAUGAAACUGCACAGCAUGAUCUGGAAGCUAGCUUUGAUCACUAAUAUAAUAUUACUGUGAACUGCAUAGCCACUCAUCAGAUCAUG
CUGGCAGCUUCAGCUCCUAGAGAAUGCACCACAACGAGUCG
>Bud_58

CCCCCCACCCUCGCCUCGUUGUGGUGCAUUCUCUAGGAGCUGAAGCUGCCAGCAUGAUCUGAACUCAGGGAAACAUUAGUGAUCAAAGCUAACUUUCAGA
UCAUGCUGUGCGGUUUCAUCUGCUCGUGGAUGCAUAGCAGAAACUGCAUCGGCGUGCGAGGGUAUUCACAAACUUCUCGCAACAGAUUUGACUCAUUCGU
GUUGCCCCAUAAAUUUCUUCAAGUUUCGUUAUAUCGGCAGAGCAUCAGUCAUAGCAUAAACUGCACGAGUCAAGAAUAAAUGGAGGUGAAAAAUAACCGC
UAAUGGUAAGCAAAAGCUUAGUUCUUUUCAGUUUCUUGUUUCCUUUACUUGCAUUAUCAACACAUGACUAGCUUCCCCAGCUACUUUUCUUGUUCCUCCA
AUAUGAUUUGCAGGUGUUCUAAUGAUAAUUAUGAGAAGCUAUAUAUAAUAUAUGGGAUCAGUAACUAGAGCCUGUACUCGGAAUUGGAGAGGGUCUAUUC
UCAUGCAAGGAAGGAUAUUCAAUUGAGAAGUGAAAUGAUCGAUCAUGAUGAAUUAAAUAUUUCUUUCAAGAAAUCAUCUUGAAGAAAUACAUCUAAUUUU
UAUAUGUAGUUUGUGUGUAUCUGUAGCACAUAUACACAUAUGUGUUUGUAUAAUUUCUGUGUGCUACCUACCUAAAUUAUUCUUUUCCCUUUCUUUCUAA
UGCAUCAGAUAAUUUAUUGUCCAUAUGAAUCAGCUAAUUCUCUGAAUUAAAUAGAGGUGCAUGUUAA
>culm_89
AAUUAUCAUUAGAACACCAUUAGCGGUUAUUUUUCACCUCCAUUUAUUCUUGACUCGUGCAGUUUAUGCUAUGACUGAUGCUCUGCCGAUAUAACGAAAC
UUGAAGAAAUUUAUGGGGCAACACGAAUGAGUCAAAUCUGUUGCGAGAAGUUUGUGAAUACCCUCGCACGCCGAUGCAGUUUCUGCUAUGCAUCCACGAG
CAGAUGAAACCGCACAGCAUGAUCUGAAAGUUAGCUUUGAUCACUAAUGUUUCCCUGAGUUCAGAUCAUGCUGGCAGCUUCAGCUCCUAGAGAAUGCACC
ACAACGAGUCGAGGGUG
>root_37
AAUUCAGAGAAUUAGCUGAUUCAUAUGGACAAUAAAUUAUCUGAUGCAUUAGAAAGAAAGGGAAAAGAAUAAUUUAGGUAGGUAGCACACAGAAAUUAUA
CAAACACAUAUGUGUAUAUGUGCUACAGAUACACACAAACUACAUAUAAAAAUUAGAUGUAUUUCUUCAAGAUGAUUUCUUGAAAGAAAUAUUUAAUUCA
UCAUGAUCGAUCAUUUCACUUCUCAAUUGAAUAUCCUUCCUUGCAUGAGAAUAGACCCUCUCCAAUUCCGAGUACAGGCUCUAAUUACUGAUCCCAUAUA
UUAUAUAUAGCUUCUCAUAAUUAUCAUUAGAACACCUGCAAAUCAUAUUGGAGGAACAAGAAAAGUAGCUGGGGAAGCUAGUCAUGUGUUGAUAAUGCAA
GUAAAGGAAACAAGAAACUGAAAAGAACUAAGCUUUUGCUUACCAUUAGCGGUUAUUUUUCACCUCCAUUUAUUCUUGACUCGUGCAGUUUAUGCUAUGA
CUGAUGCUCUGCCGAUAUAACGAAACUUGAAGAAAUUUAUGGGGCAACACGAAUGAGUCAAAUCUGUUGCGAGAAGUUUGUGAAUACCCUCGCACGCCGA
UGCAGUUUCUGCUAUGCAUCCACGAGCAGAUGAAACCGCACAGCAUGAUCUGAAAGUUAGCUUUGAUCACUAAUGUUUCCCUGAGUUCAGAUCAUGCUGG
CAGCUUCAGCUCCUAGAGAAUGCACCACAACGAGGCGAGGGUGGGGGGAGAGAUUUGAGG
>root_47
UAGUAGCGUCCCUGAGCCAUUAAAAACCAUGGCGAUGUAUCAAACGUUUUCAAGAACACGCAAAAGAUUUACAAAUAUUUGUUCAAAAAAAGGAUAAAC
UAAUCAUGGAUGGUUGCUCAGGUAAGGAACCGAAGUGAAGAUAUGCAUCAGGGAAGAAGUCAAGUCAAAAGACAAAUUUAUCAGCACAUUUCACAGAACU
AGCUAAUGGAAGCACACUGAAAUUGGGCAGCUUAUUAAAUACCAUGCUUGGAGUAGGUUAUGUCCCCUUGACCUUGCCCAGAUCGGAAGUGUCACCACAG
ACAAAGCCUUUCAUGAGGAGCAGGGGCCAAGAUGUAGCCAAGGACAACUUGCCGAUGACCAAACAAACACGCAUCAAUCUGACGAUCACAGGCAUCAUCG
AUCGGCAAGUCAUCCUUGGCUGCACCAUAGUCCCCGAGCCUAAUGUUAGGCCGUGCGGCGAGUCCGGCGACGGCGAGUUUGCACCCCUGGCAGCAGCCAU
GGUUGGCACGGGAGGAGAGGAAUGGAGAUGAG
>Bud_32
CAUGAGAGCAAAGCAGCCAUGGAAGGGGAAGCAAACCCACACCACAUGAGAGCAAAGCAGCCAUGGAAGGGGAAGCAAGUCAGAUGAGGAAGGAGGUUGG
UACCUGUAGUAUUAGAACCUAUACCGGGCAGGCUUCUGCUGGAAAGUUGCCAUGCGCUAAGAGGCAGAGAGCGGGAUGCAGCCAAGGAUGACUUGCCGGC
UCCUGGCGUUGGGCUGCCUCAGCUUUGUUGAGGCUUAAAGAGUUAGCCGGCAAGUCUGUCCUUGGCUACACCUAGCUCUCUUCUUCUCGUGUGUGGUCUU
CAAGCAUCUUCAAGACCUCUAAUAAUUAGCAUAUGCAUUCACGUCAGCUAUAUGUGCGUGUAAUAGUUAGAAACAUCUUACUGUUUCUUCUGCAGUUGAA
AUGCACAAAUAUGCAAGCAUAUAGGGCUUGCGUACAUGCUCAACUUCCACUUGACAUCACAUGCCAAAUUCUAUACGUGCAUAUGUCAGACAAUAUUCUC
ACUGUUUUGUACCUUUAUCGUAUGCU
>culm_80
GCACGUAUAGAAUUUGGCAUGUGAUGUCAAGUGGAAGUUGAGCAUGUACGCAAGCCCUAUAUGCUUGCAUAUUUGUGCAUUUCAACUGCAGAAGAAACAG
UAAGAUGUUUCUAACUAUUACACGCACAUAUAGCUGACGUGAAUGCAUAUGCUAAUUAUUAGAGGUCUUGAAGAUGCUUGAAGACCACACACGAGAAGAA
GAGAGCUAGGUGUAGCCAAGGACAGACUUGCCGGCUAACUCUUUAAGCCUCAACAAAGCUGAGGCAGCCCAACGCCAGGAGCCGGCAAGUCAUCCUUGGC
UGCAUCCCGCUCUCUGCCUCUUAGCGCAUGGCAACUUUCCAGCAGAAGCCUGCCCGGUAUAGGUUCUAAUACUACAGGUACCAACCUCCUUCCUCAUCUG
ACUUGCUUCCCCUUCCAUGGCUGCUU
>leaf_41
AAAUGACAACAUAGAGGCCGGCCAGAGUAGCUGCUAGAGAAGAAGCGACAUAGGUUCCAAUCCACUGCCUGGGGGCCAUGCACGAAAGUGGCAGAGAGCA
GGAGAUGCAGCCAAGGAUGACUUGCCGGCGUCCGCGCGUCGACGCCGACGGCAUUGUUGCCAAUGCGGCCGGCAAGUCUGUCCUUGGCUACACCUUACUC
UCCGCCCAUCAUGCGUUGCCCUUGCUUGGCUCGAGAGUGAUGAUUUAGUUGCCAAAUUAUGAUACUAGUAUCUUGUUAUGCAUGAAGUACCACAAGUAUG
UAUACGUAUUACUCCCUCAGUCUCUUUUGUAUACGAUGCUGGGAGAACUAAAACCAAUAAUUCCCCUAACGUCAUAUAUACAUGAUUGGAGGGAGUAGGU
AUUAGACAUGUAUGCUCGAGUCGCCCUCCAUCAAGUUGCGAUAGCGGCAAUGCACAGGUAAUAAGGUUAUGAGUAAAUUUCGUAAAACCCAAGCUCUUAU
GGUCCUUGUGCCAUAAAACCCUACGGUUUAUAGUACGUGUCAUGUAACUCCAGACUUUAUGGUACUAAUAUUUCAAAAAAACCACUAUAACCAAUAUUUA
UCAAUCUAUUUUAAAUCAGUAUUUUUUCAGCAUGUGGUAGUGUACCCAAGUUGGCAUCCCGAUGAAAGAAGCACAAACACCAUCAUUUUGUAUCUAGGAA
CAGGAAGUUCGAUUUCAAGGAAAAUAGUUAGAGAUGGGAUUUUCUGAAACAUUAGUGCUACAAAAUUUAUGAUUAUUUGACACAAACUAUAAAUCAUGUG
GUUCUGUGACAUGAGGACCAUGAUACCUGGGGUCUUGCAAAAUUUAUUUUAAGGUCAUAAGAGUAGGAAACCAUGCACUAGAUUGGGUUGAGAUUUGUCC
CAUUCAAACAGAGGCCUUAGCAAGUUGUUCUCCACGGCAUCCACCAUUGAUUCAUGUUUGUACCCGAGAUCGAGAUGUUUAAUUUGCAUGUAAUCGAGAG
AGCGCGAACACACGCACCUUAUUUUAGUACCAACUAGUAAGGUGACCCAUGCAUUUUCACACGAGCUAGAGUUUAUUAUAACUUUGAUGUAACUAAAAAU
AAUUAAUGUCAACAAUCUUCAUAUAACACACACCAAUCCGUGUCUUCAUUGGUAUCUCUUGUGUCUAUGGUAGGUUGUGACUUGUGACUUCACCAUCUAC
GGCCUGCUGGCUAUGCCUUGCCAUGCACCACAAUCUUGGGCUACGCAACAACAAUUCUCUAGUUUGUUUUUCCAAUUUCCCAAAUGAAUUCAGUCAAGCA
UAGCCUGUCGCCAACUUGCUGCCAUGGUGUGAGGAUGAAGCACAAUUCUGUACCCUGUACCGCAGAUCCACAGCUCCUUCGGUGUCCAUUGUCAUUGCCA
GAUGCUGCGCCUCUAACGUCAACAAUAUGUGAGUUGUUCCUCAGCUGCUAUUAGAGAUGAAAGUGAAAAUGAGAAUUAUUAAAAUUUUAAAGUUUAUUUU
CGAAAAAAAUCGAAAAAACAGCUUGUAUUUAUUUCUGAAAUUUUUCAACAAUUUUCCAUCUUAUCAUUUUCGUUUCCAAUUCCAAGAAAAAAAUAUGAAA
GUGAAAAUAAUUUAGUCAAUUUCUCGAUCGUUU
>root_90
GGCUUACAUCUAUGCUUGUUCAUGCAUUUCCGUAUUGAAGAAGUCGACAUGAAUGCAAAUGUCAGAAGUCUUGUGACCAUGCUUGAGGGCCACACACGAG
AAGAAGAGAGCUAGGUGUAGCCAAGGACAGACUUGCCGGCUAAAUCUUUUAAGCCUCAGCAAAGCAAAUGGCAGGAGCCGGCAAGUCAUCCUUGGCUGCA
UCCCGUUCUCUGCCUCUUA
>root_18
GGCCACAGGCAAGUCAUCCUUGGCUAUCAGUAUCAACUAUUAUCACCAUGAGAGCUGCGUAUUAUGAUAUUAAGAAGGAAAUAAAAGGUUCUGUACAACC
GGUUUACAGCUACAGCCCCAUGGGCAACUCGACUUGCAGUAAGUUCAAGACGUUGAAAGACUCUUCUAUUACACUCUUUCCAAAUUCAGCCAUACCAUUA
AAAGAUCUUUUCAUUUUUUCCCCGAGAUAUGUGCAAAUUUCAAUCACAGGAAGGACCAAAGAGAAGGGUUCGACUAAUGCAUUCACGAACUUUGUGUCGA
ACAACCAUUGAUGCAAGCAUGGAUGGAUCACCUGACUUAUUAUGGAUGGUGAUCAUCGCAAUCAAAUGAAAUCUCUCUCCAGCUUGCUUAAUUGGUCAAC
UGGCAAGAGAAAUGCCUGGUUAUAUUAAGAAGGAAUAAAGUCAGGAGUACAAAGCUGCAAGGCAGUGAGAAAAAUUAAGAUUACAGCAAGAAGAUGAAAC
AAAGCUAGAAAAAGACCACAAUCAUGGGGGAAUCAACAUCAUAAUCUGCUUGAGUGGACUGUAGGAAGAACUGAAUUUCGUCCUUGAUCAUGGAAGAAAC
CUGUAGAAAGGACUUUUCUUGCUGUUGAAAGAUCCUCUUUCUCACCUUCCAAAUUCCCCACCGGAAGUAGAUAAGUAGGCCAGUCUUCAGUCUCUUGGUA
UGUUUAUCACUUCUGGCGCCAACUGUUGUUAUAUAUCCAGUCCUGGAGCGACGACAACCUCUGUGUGUAAUCUACCUCGAUGCAAAGAAGUACUCCAUCC
AUUUUGUAUCUUCUCGUCUUCUUCAUGUGUUGAAGUUGAACUACUUGCAUGCAUGCAUAUGCACACCACCUUUCAAUGUUCUUUAGCCUUCAACCAAGAU
CACACACAUAAAAAAAAACAUAGACACAGAUAACUGAAUAAACAUGAGCUGUCAAUACAUGCAUGUCAAUCAGAAUCUACGAUGAAUAGCUUCUAUAUGUA
CUUAUAUAAUAAAUAUAUACACCCCAAGAGCUUGCUCAAGCAGUAUUAUUCAGAAGUAACUAGAUACAAGAAGAUCCAAGUCUAGCAUGAGCAAUAAGAG
CCACUCAGGCUAGCCAAGGAGACUGCCCAUGAGACCACUCAAAGGCUCAUUGUUUUGCUGAUCCUUUAGGGAGGACACAGGCAAGUCAUCCUUGGCUACC
AGAGCUACACCCUUAUCGCCAUGUAAGCCUUGCUUUCUUCUUAGUCUCUCUAAUUUUAUGUGCGCAAUGGCUGCU

>leaf_36
AUCCCAAGGACGACUACCAGCCAUGAAUAAAGGAGGCGCGAGACAGAAAUUGAAGAAGAAGAAGAGGGGGACCUUACAUGGUGAUGCAGAGACUCGCAUU
UGGUUGAGCCAAGAAUGACUUGCCUAUGCAUGCCUUCUGUUAGCAUCUCCAACAGCCAUGGCAACGGGAAGAGAAAUUCUUAUGAUGCGAUGCAAUGUGG
CUGCACGGGCAGGUCUUCUUGGCUCUUCAGAGUGGCUCUCAUCCACCAUGCCAGGCCACCCUCUUCCUUAUUUGACAAUUUCACAGAUUUCAGCCCUCUG
CACGGGAAAUUUGGUGAUGUAUCCUGAACCCAGUCUUUGAAUUCAAAUAUAUAGAGUUUUAUAUUUGUUCAAAGUCAAAUAUUUUAGGAUAGAGGGAGUA
AGGAAGGUCUCUGUCUUCUCUGCAAACAGAAGAAUUUUACAUGGAUGCCUUCAUCUCUACACGAUUUCAACUGAUAAGUAAAGAAAUAAAUCAAGAAGUC
UGCAUCUCGUUCUAGCUUAUCAUGUAGUAGUACAUCCGUGAUUCCCUGUUGUUCUGUGAUCAAGGAGCCGCAAAUCCAGCACAGGUCCUAAACGCACUUC
AGUCAGUGAUUGUUGUCUACCUUUCGUACAUAAAAAAAAUAGGGCUGAAAGUUUUUUUAUCCAUGAUCACAGAGAAUAUAAUGAAAAGAGCAACUUUUGC
CAUCAAUAUUUUUUUAUUUCAGGCGACAAAGCAACAAAACAGAUUUUAGGUUAGGGCAUUUUAGGAUGGGGUUUGUAAGCUUAUCUCGUUGCUGGGACU
UAGAGAAAGGGCUCCGGGGAGGCAGAAUGAACCAGCGGAGGAAGCGGGCGGUGGGCAGGGCGACGAAGCGGUGGGAGCGUCGUCCGCCACGAGUGGGCGG
AGGACCGCCGGUGAGCAGGUUUGGGGCAGGAUUAGCGCGGUGGAGGGUGGUGGCAGCGAAUCCGGCGGAGGGGAGUCGCUGUAGAUGGGGGAGGCUGGUG
GUGCAUUGGAGCGGAAAAUGACAAAGAAAACUAAAGAGAAGGAAGAAACUACAGGCAGCAAAUUGAAACCACGCAUCUCAAGAAAUACGUAGGGAAUUGA
GGAACGAGCAACAGGCAGCAAAUUGAAACCAUGCACGCAUCUCAAGAAGACAAGAUCUGAUCAUACAGUACAUAUGCUCUGCUCCAACACGUUGGCACGA
GAUAAAUUAAGUUAAAUUGACCACAUCAAAGAAGAAAAUUAAUUAAACUGCUAAUUAUUCCUACUGCCCGCGAGGAGGCAGGGUGUGAUCGUGUUGUGCC
UCUCGAUCUUCUUCAUGUCUCUCGUAACGUCAAAAAACUCCACCUUCUUCCUGAAGUUCACACGGCACUCAUCACCGUACGUGCGAACUUGCCACACCCA
CAACGCAAGCACACGUGCGAUCAGGACGACGAUAAGGGCGACCACGGCGACGAGCAUCCACGUGUGGCCAAAGGCCGCGACGAACACGCCGACCACGCCG
AGAGGAGAGCCGACGCAGCCCAAGCCGACAUUGACGAGAGCUUCCCUGACGGCGAAGGGCGCCCGCGGCAAGU
>root_39
AAAAAAUAAUAAUGCCCAGCUUGGCUGUUAGCUUACUACUCUUUGCUCAUAAUAUGGAAUCAAUGGAUAAAAUUCUUCCAAAAGGUUCAAUUGGAAAUCU
GACACUGCAUGCCCCCCUUUUCCAGUUUGAGGAACUGAAAAGACCAUGCUCUUCAAUCUGCACCACAGAAUGGUCUGAAUCCAAAACUUCCCUGAAAAGA
AGCAACAAUUUUUCCUCAUCUGCAAAGAUUAGAAGCUUUUUCUAGAAAGGAUGCACAAACAAGAAUUCAAAGACUGGGUUCAGGAUACAUCACCAAAAAA
UUCAGGUGCAGAUAGCUAAAAUAUGGGAAAAUUCAGGAAUCAGGAAGAGGGUGGCCUGGCAUGGUGGACGAGAGCCACUCUGGCUAGCCAAGAUGACCGG
CCCAUGCAGCCACAUUGCAUCAUCCAUCACACAAGAAUUUCUUCAGAAAUCCUUCCCAUUGCCAUGGCUGAUGGAGCUGCUAACAGAAGGCAUGCCUUCU
UCAGAAAUCCUUCCCAUUGCCAUGGCUGAUGGAGCUGCUAAUAGAAGGCAUGCAUAGGCAAGUCAUUCUUGGCUACCAAAACAGAGG
>root_51
GACGAAAUCCUCGACGCAUGCAACGUGCAAACGAGGAAAGCACACGUUGGAACUAGCUGUGAGUCCAGUGUGCUACACGUGUGGCGACCAAAGUCUCACA
CACUGCCACACACGCAGACGCAGUCACAUACUCCUCGUGGCCGUGCAUGUUGGCAGGAGCACUUCUUGGAUCUGGUGACUGCCACUACAUAUACCUGAUG
AUCAUCUACAGAAGUAGCAUCAGUAGUCAUGCAAAUAGAAAAAUAAUAAUGCCCAGCUUGGCUGUUAGCUUACUACUCUUUGCUCAUAAUAUGGAAUCAA
UGGAUAAAAUUCUUCCAAAAGGUUCAAUUGGAAAUCUGACACUGCAUGCCCCCCUUUUCCAGUUUGAGGAACUGAAAAGACCAUGCUCUUCAAUCUGCAC
CACAGAAUGGUCUGAAUCCAAAACUUCCCUGAAAAGAAGCAACAAUUUUUCCUCAUCUGCAAAGAUUAGAAGCUUUUUCUAGAAAGGAUGCACAAACAAG
AAUUCAAAGACUGGGUUCAGGAUACAUCACCAAAAAAUUCAGGUGCAGAUAGCUAAAAUAUGGGAAAAUUCAGGAAUCAGGAAGAGGGUGGCCUGGCAUG
GUGGAUGAGAGCCACUCUGAAGAGCCAAGAAGACCUGCCCGUGCAGCCACAUUGCAUCGCAUCAUAAGAAUUUCUCUACCCGUUGCCAUGGCUGUUGGAG
AUGCUAACAGAAGGCAUGCAUAGGCAAGUCAUUCUUGGCUCAACCAAAUGCGAGUCUCUGCAUCACCAUGUAAGGUCCCCCUCUUCUUCUUCUUCAAUUU
CUGUCUCGCGCCUCCUUUAUUCAU
>Bud_48
UCAUGCAUAUGUUAUGGAUAUAUUCUCCUACAUGCCUGAUACUUUUUCACAGAAAGGAAUACCAAGUGGAAGAUCAGAACCAAACCUGUCAAACAUUAUU
AGCAUCCAGCAUGAUAUCAUCAGAUUAUUCAUAUCUUAAUUUGUCAACCAUGUACGCAUGAUAACUACCUGUAUAGCCAAUGAUCAAAGGUCAAUUGAAC
UAUUUUUCAUCGUUUUGCAAAGAGAUGUCGAUCAUAGGAAUCAAAUCCCUUUCGGUGGGUCAAUUGGUAGCUUUAGUCAUAUUUCAGCAUCAGAAAGGCC
CUUUCGGAGUGUCAAAAUGUUCUAUGUUCACAGGAACCAGGGAAACCUUCAAUUCCUAUAUAUGUAAACUAAAGGUGUAUGAAUGGCGAUAAGGUCAGGU
UGUGAUCACUAUCUUUGAUAUCUACUCGUGUCUCCAGCAACCAUAAAAGUUUCGCAUAUAUUGGUAAAGUUAGAAUAUAUUCUGCUACCCAAACAGGAGG
AUAAAAUAUUCUGAUUGAGCAAAGAUUUGAUUCUCUCCACAAGGUAUGUAUAAAUAGAUGUUGAAUCAAAGAAAUACAGCAGCCGCGGCCAUAGCCAGAG
GAGAAGUCUUACAUGGUGAUAAGGGCCUGCCUCUGAUAGCCAAGGAUGACUUGCCUAUGUCCUUUGUACCGAAGGAUAAGCAAUGUGAGCCUUUGAGAUG
GUUCAUGGGCAAGUUUCCUUGGCUAGCCUGAGUGGCCCGUAUUGCUCAUGCCAGGCUCAAUCGAUCUCUUUGACUUGCGCGUGCAAUUACUCUUGAUAGU
AUGUGAUUACUCUUCAUACUCCACACGGAAGAAAAGAUCGUGCAUACCUGCAGAUCAUCAUCUCAUGCUGCUGUGUUUCAAUGUGUACACAACAUUGACA
AAGCGGCAUGGCAUUUGCUUUUUCAGAACAAUCCGCAAAAAAUCCUCUGACCUUAUUCCUGACUAUAUGCCUGCGGUAAGGAUCAAUGCAUUCUCAUUUA
GUAGCAGUGCAGCUAGGUUAUCCAGUGGCAAAUGCUCAUAUAAUUUGUCACACGCAUUUGCCCCCAGAUCACCUAACCUUGCGACAAUUUGCAUCCAUGU
UUAUGAACUGCCCCCUUUGUUCUCCGUGCAAGUUUUGGUUUGCAUGCAUCUCAAGAUGAAGGGUCGGGAUUCAGGAGAACAUGUUUUUCUUUAAAAAAAU
GAGUUAAGGCCUCCGGCGUCCAAAAGAAAACAAAGCUUAGAGCCAGAGCUUACAUU
>culm_60
AAAAAAAAAGAUCCGAGCAUAAUACAACACAUACGACAAGAACACGAUAAAUACAACAGGGGAACACUAAGAAACACAACUCCCAUACAAAUCAAUGAGA
CAACGCCAAGAUUUAGGCACAAGCCAUCUCCAAAUACACGAACAUGCACGCAUGGAUUGUGUUAGUUGAAUAUUUCUGUGCUAUUUGGUGCAUCUCAGGA
GUCCAGUGAUCACUGUACAAGUGUACAUAAUUGUAGUUUGUCAAGAAUGUUUAAUUUAAUCUCACGAUCAUGGUCUCAGCUUGAUCUUGCAUGCGGGAUU
CACUGGCCUUUUCGCGGGCAUGGAGGCGUUGGGCUUGGGCCAUGCAAUAGAAGCGACGCUGGGAUACACAAGCAAAGCGUCAAGCCGCUCCAGCGGUUCA
GAUACUGCACCUGUUACACAGACUGGCGGAUGCGAAGCGAGCUGCUCUCUGACGUAGCUAACCGAACCUCAGAGGAUCUGGUUCCGCCCGCCGCCGGAGG
ACGUCCUCUCCUGCUCCGGUCUCCGCCUCUCCGCCCCUUGCUCACGGAUUUCGUUCUCGCCGCCGGCGACCGCGCGGAGUUGCUCCUUCCUCAGUCGUUC
CCUGGGUGGCCCGGGGCGCAUAGGCAUUUGGAGGCUGCGGCCGUGGCGGCCAGUUGAGGUGGACGGGAAUGGCGACGGUUCGACCCGCACAACAGCGGAG
AUGGCGACUGGCAACAUCUCCUCCGGCAAAAGCUAAGUUCUCUGUGUUGCAGGAAUUUAGGGUUUGGAAGUAAGCAGAAGAGGAACGCAACAAUCACUGA
CUACAUAUAAUUCUCCACUUUGAGCCUCGUUCUGAAAGUUGGUGGAAACACACACAUACUGGUUUUCUCUACUUGUGGCGCUUUUUCUUUUUCUUUUCCC
UCUGUUGCACAGGGAAAAGAUAACUGACACUGAAUUUAUUUGUUGAACAUGUCAGGUUGUACAUUGAGCUAAUAAACAUCUACAGGAUUAACCGAGACAC
GGCACAGGUAGUCCAACUACACCUACUACAAUAUGGCGAUCUCCGCUUUUGCAAUACUUAAGUGCUAGCGCUGUUACAUUCUGAGACAAGUUGAAGUGAC
CAAGCACUAGCAAUUCCUAAAGUAUAAUGUAAGCUCUGGCUCUAAGCUUUGUUUUCUUUUGGACGCCGGAGGCCUUAACUCAUUUUUUUAAAGAAAAACA
UGUUCUCCUGAAUCCCGACCCUUCAUCUUGAGAUGCAUGCAAACCAAAACUUGCACGGAGAACAAAGGGGGCAGUUCAUAAACAUGGAUGCAAAUUGUCG
CAAGGUUAGGUGAUCUGGGGGCAAAUGCGUGUGACAAAUUAUAUGAGCAUUUGCCACUGGAUAACCUAGCUGCACUGCUACUAAAUGAGAAUGCAUUGAU
CCUUACCGCAGGCAUAUAGUCAGGAAUAAGGUCAGAGGAUUUUUUGCGGAUUGUUCUGAAAAAGCAAAUGCCAUGCCGCUUUGUCAAUGUUGUGUACACA
UUGAAACACAGCAGCAUGAGAUGAUGAUCUGCAGGUAUGCACGAUCUUUUCUUCCGUGUGGAGUAUGAAGAGUAAUCACAUACUAUCAAGAGUAAUUGCA
CGCGCAAGUCAAAGAGAUCGAUUGAGCCUGGCAUGAGCAAUACGGGCCACUCAGGCUAGCCAAGGAAACUUGCCCAUGAACCAUCUCAAAGGCUCACAUU
GCUUAUCCUUCGGUACAAAGGACAUAGGCAAGUCAUCCUUGGCUAUCAGAGGCAGGCCCUUAUCGCCAUGUAAGACUUCUCCUCUGGCUAUGG
>Bud_49
CUUGUAUACAGAAACAGAUGUGUCAUCAGUUGGCUAAUAGCUUACAUGCAAACCGCUAGCUGAUUUCUGAAGCUCAUGCAAGCCAGAUUCUCCCUCAAGC
UCUCAUGUGGUGCUUCUGCUCACCAAGUUGAUGUAGAGUGGUCAAAAGCUCCUCGGAUCUCAGGCGAUGAAUUUAACACACAUGAAGUUGUUCAGCCAUC
AGCUAAUCUCUUAUUUUCUGCUUCCAAAACAUGGUGAUAAGGGCCUGCCUCUGAUAGCCAAGGAUGACUUGCCUAUGUCCUUUGUACCGAAGGAUAAGCA
AUGUGAGCCUUUGAGAUGGUUCAUGGGCAAGUUUCCUUGGCUAGCCUGAGUGGCCCGUAUUGCUCAUGCCAGGCUCAAUCGAUCUCUUUGACUUGCGCGU
GCAAUUACUCUUGAUAGUAUGUGAUUACUCUUCAUACUCCACACGGAAGAAAAGAUCGUGCAUACCUGCAGAUCAUCAUCUCAUGCUGCUGUGUUUCAAU
GUGUACACAACAUUGACAAAGCGGCAUGGCAUUUGCUUUUUCAGAACAAUCCGCAAAAAAUCCUCUGACCUUAUUCCUGACUAUAUGCCUGCGGUAAGGA
UCAAUGCAUUCUCAUUUAGUAGCAGUGCAGCUAGGUUAUCCAGUGGCAAAUGCUCAUAUAAUUUGUCACACGCAUUUGCCCCCAGAUCACCUAACCUUGC
GACAAUUUGCAUCCAUGUUUAUGAACUGCCCCCUUUGUUCUCCGUGCAAGUUUUGGUUUGCAUGCAUCUCAAGAUGAAGGGUCGGGAUUCAGGAGAACAU
GUUUUUCUUUAAAAAAAUGAGUUAAGGCCUCCGGCGUCCAAAAGAAAACAAAGCUUAGAGCCAGAGCUUACAUU
>leaf_07

AGCAAGACUUACAUGGCGACAAGAGCUUGGCUCUGGUAGCCAAGGAUGACUUGCCUGUGUCCUCCCUAAAGGAUCAGCAAAACAAUGAGCCUUUGAGUGG
UCUCAUGGGCAGUCUCCUUGGCUAGCCUGAGUGGCUCUUAUUGCUCAUGCUAGACUUGGAUCUUCUUGUAUCUAGUUACUUCUGAAUAAUACUGCUUGAG
CAAGCUCUUGGGGUGUAUAUAUUUAUUAUAUAAGUACAUAUAGAAGCUAUUCAUCGUAGAUUCUGAUUGACAUGCAUGUAUUGACAGCUCAUGUUUAUUC
AGUUAUCUGUGUCUAUGUUUUUUUUAUGUGUGUGAUCUUGGUUGAAGGCUAAAGAACAUUGAAAGGUGGUGUGCAUAUGCAUGCAUGCAAGUAGUUCAAC
UUCAACACAUGAAGAAGACGAGAAGAUACAAAAUGGAUGGAGUACUUCUUUGCAUCGAGGUAGAUUACACACAGAGGUUGUCGUCGCUCCAGGACUGGAU
AUAUAACAACAGUUGGCGCCAGAAGUGAUAAACAUACCAAGAGACUGAAGACUGGCCUACUUAUCUACUUCCGGUGGGGAAUUUGGAAGGUGAGAAAGAG
GAUCUUUCAACAGCAAGAAAAGUCCUUUCUACAGGUUUCUUCCAUGAUCAAGGACGAAAUUCAGUUCUUCCUACAGUCCACUCAAGCAGAUUAUGAUGUU
GAUUCCCCCAUGAUUGUGGUCUUUUUCUAGCUUUGUUUCAUCUUCUUGCUGUAAUCUUAAUUUUUCUCACUGCCUUGCAGCUUUGUACUCCUGACUUUAU
UCCUUCUUAAUAUAACCAGGCAUUUCUCUUGCCUGAACUUUCAAAAAAAAGAUUACACAGAGAGGUUAGGUUAUAUAUGGAUGUACUGUGCUGCGAGUUU
ACAUGGAGCACUGAAUAAAU
>leaf_18
GAGGAAGAAGAGAAGAGGCCUUGCAUGGUGAUAAGAGCUGAUCCUGAUAGCCAAGGAUGACUUGCCUGUGGCCUCGCAAGCUCGCUUGCAGUUUGUCAGG
CUGGCAGGUGAUUCGUCAUAGGCAGUCUCCUUGGCUAGCCCGGGUGGCUCUUCUCCUUCAUGCUAGGCGUUUCCCUCUACCAUGGAUCAUUGGAACUUCU
UGUAUCGCACAGUUUCGAGCUCGACUUCAUCUCCAUGUUCAGAGCCACAUACUCACAUUUCCGAAAGGCAACUUUAGUCUGCUGCAGACCACGUAAAUCU
UCUUUAAUCAACUCAUCUUGCUUCUGGAGGCAAAAUAUGAAAAUGGAAGAAAUUAUGAGUAUUUUAAAUCAUGUUAGCCGAUUGAACUUCUGCAUAUUUG
UGCUGCAUACAUACCAUUUGUCCAAUGAUAAUGUCAGCAAUAAUUUUCUUUUUUGGGUCAUUUUCUUUAGGGGUAUACCCGUUACCCAUCCUGCUUUGCU
UGUAGGAGAAAGGAUUCACUGUCCAUCAUCUGAUGUGUGCUUGCAGCUGAUUCUGGUCAGUUACUCACAGGAGAUGCAUGUCUGAAUUCCACAACAAAUC
CAUCAGCUUUGGGUGAUGCAUAAUACCUAAUUAAGGAGGCUUGAUGAUCAGUAGUUAUUCGCUUAUCUGGAUGCCAAAUUAAUUUGUAUCAUAUAUGGCG
ACAGCACUAGCUUUGUAGCUCCGUGCUUA
>leaf_47
GUAUGUAUAAAUAGAUGUUGAAUCAAAGAAAUACAGCAGCCGCGGCCAUAGCCAGAGGAGAAGUCUUACAUGGCGAUAAGGGCCUGCCUCUGAUAGCCAA
GGAUGACUUGCCUAUGUCCUUUGUACCGAAGGAUAAGCAAUGUGAGCCUUUGAGAUGGUUCAUGGGCAAGUUUCCUUGGCUAGCCUGAGUGGCCCGUAUU
GCUCAUGCCAGGCUCAAUCGAUCUCUUUGACUUGCGCGUGCAAUUACUCUUGAUAGUAUGUGAUUACUCUUCAUACUCCACACGGAAGAAAAGAUCGUGC
AUACCUGCAGAUCAUCAUCUCAUGCUGCUGUGUUUCAAUGUGUACACAACAUUGACAAAGCGGCAUGGCAUUUGCUUUUUCAGAACAAUCCGCAAAAAAU
CCUCUGACCUUAUUCCUGACUAUAUGCCUGCGGAAGGAGGUUGCCAUUUUCUCCUCAGGCCACACAAACACAGAAGCGCUACCUUAGCUUUUGCCGGAGG
AGAUGUUGCCAGUCGCCAUCUCCGCUGUUGUGCGGGUAGAACCGUCGCCAUUCCCGUCCACCUCAACUGGCCGCCACGGCCGCAGCCUCCAAAUGCCUAU
GCGCCCCGGGCCACCCAGGGAACGACUGAGGAAGGAGCAACUCCGCGCGGUCGCCGGCGGCGAGAACGAAAUCCGUGAGCAAGGGGCGGAGAGGCGGAGA
CCGGAGCAGGAGAGGACGUCCUCCGGCGGCGGGCGGAACCAGGUCCUCUGAGGUUCGGUUAGCUACGUCAGAGAGCAGCUCGCUUCGCAUCCGCCAGUCU
GUGUAACAGGUGCAGUAUCUGAACCGCUGGAGCGGCUUGACGCUUUGCUUGUGUAUCCCAGCGUGGCUUCUAUUGCAUGGCCCAAGCCCAACGCCUCCAU
GCCCGCGAAAAGGCCAGUGAAUCCCGCAUGCAAGAUCAAGCUGAGACCAUGAUCGUGAGAUUAAAUUAAACAUUCUUGACAAACUACAAUUAUGUACACU
UGUACAGUGAUCACUGGACUCCUGAGAUGCACCAAAUAGCACAGAAAUAUUCAACUAACACAAUCCAUGCGUGCAUGUUCGUGUAUUUGGAGAUGGCUUG
UGCCUAAAUCUUGGCGUUGUCUCAUUGAUUUGUAUGGGAGUUGUGUUUCUUAGUGUCCCCCUGUUGUAUUUCUUGUGUUCUUAUCGUAUGUGUUGUAUUA
UGCUCGGAUCUUUUUUUU
>root_57
UGGCGACGGUUCGACCCGCACAACAGCGGAGAUGGCGACUGGCAACAUCUCCUCCGGCAAAAGCUAAGUUCUCUGUGUUGCAGGAAUUUAGGGUUUGGAA
GUAAGCAGAAGAGGAACGCAACAAUCACUGACUACAUAUAAUUCUCCACUUUGAGCCUCGUUCUGAAAGUUGGUGGAAACACACACAUACUGGUUUUCUC
UACUUGUGGCGCUUUUUCUUUUUCUUUUCCCUCUGUUGCACAGGGAAAAGAUAACUGACACUGAAUUUAUUUGUUGAACAUGUCAGGUUGUACAUUGAGC
UAAUAAACAUCUACAGGAUUAACCAAGACACGGCACAGGCAUAUAGUCAGGAAUAAGGUCAGAGGAUUUUUUGCGGAUUGUUCUGAAAAGCAAAUGCCA
UGCCGCUUUGUCAAUGUUGUGUACACAUUGAAACACAGCAGCAUGAGAUGAUGAUCUGCAGGUAUGCACGAUCUUUUCUUCCGUGUGGAGUAUGAAGAGU
AAUCACAUACUAUCAAGAGUAAUUGCACGCGCAAGUCAAAGAGAUCGAUUGAGCCUGGCAUGAGCAAUACGGGCCACUCAGGCUAGCCAAGGAAACUUGC
CCAUGAACCAUCUCAAAGGCUCACAUUGCUUAUCCUUCGGUACAAAGGACAUAGGCAAGUCAUCCUUGGCUAUCAGAGGCAGGCCCUUAUCGCCAUGGCC
CUAAUCAUAUAAUGUUUUGAUCGAUGAUCAGUAAUGGGAGGGCUCAAAUUUGACUGGGUUUUGGAAGCAGAAAAUAAGAGAUUAGCUGAUGGCUGAACAA
CUUCAUGUGUGUUAAAUUCAUCGCCUGAGAUCCAAGGAGCUUUUGAUCACUCUACAUCAACUUGGUGAGCAGAAGCACCACAUGAGAGCUUGAGGGAGAA
UCUGGCUUGCAUGAGCUUCAGAAAUCAGCUAGCGGUUUGCAUGUAAGCUAUUAGCCAACUGAUGACACAUCUGUUUCUGUAUACAAGUUGUACCGGGGAU
UCGGUUAAGUACAUGAUUUAUAUCAUGUAAAAAACUUGUACUACUGCAAUAUUGUACAAAUUAAGGUGAUC
>culm_45
AAUAAUACUGAAAAAAUGAUAUAUACUGAGGUAUGCUAGUGACCGAUCAAACCAACUGCGCGUUAGCUAAGGCAAUAAAAAUUCAGAACACUAAAAUUAA
GUUGAUUGUUUCCAAGAGAACAAGGGCAAAAGAAUGGUGAAUAUAAUUGAAGAACACAAUCAGCUGGACAACAAGAAAUUAGUGACUGAUCGAUCUAACC
UUGCAAGUAGAACUCUUUCUGUGUAUUUAUGUGGUGGAAGGAAGUGAUAUUGGCGCGGCUCAAUCAGAUACCGGCUUUACUGUCACGUAAAACCCCAUCA
UCGGAUUGAACCUCACCAAUAUCGCUCUCUGCAUCCACCAUGCAAUUCGUCACGUAGAGUACCGGCCGGCCAGCAAACCGUUGAAGGCAGCUAGUAUUGG
U
>Bud_59
GACCAGCUUUUCAAGUCUUCAGAAACUCCCAAUGCUAUAGCUAGCAACAGACGAUAGCAAUGAUGCAUGCACCAUUUUGUCUCUACUAUCUUCUGCUCCU
UUAAUUGAUCUUAGGUCUCCAAGAGAACUGAUUCUGUGCUGUUCCUCAAAAUCCAUUUGCUUCAGUGGGCAAAGCUUGACCUCCUGUUACACUAUCAUUU
UUGCUGCAUAUUGCUAGCUAUGUACAUGGAAGAAACAAAGGGAAAAGUGGCGGGAAAUUAUGCUAUACUAAAUCGCAUGGAAGUAGUGACAACUGUCCUA
AAAAUAAUGCUACUGCUAACUGUCAUAUCUUCAGCUUAAGAAUCUAGUCAGGAGUACUAGCUCUUUUAGUCCCACCGUUGUAAUGGCCACAAAAAUUAUG
UACUGUAAAGCUGAAUUAUAAGGAGAGGCAACGUGCAAAGGAAUCCUGCUACUGAUAAGACAGCUUUGCAUAGAAUCAUUACCUAUUUUUAAAAAUGGAA
AGAAUUUCAUAGCGAUGAAAUUUUAAGGACAGAAAACUUGCAUAUGAGGUUUGCAAUGUGGAUGGCAGCAUGAAAUCAAGAAUAGCAUUAACAUAUAAUC
UCAAAAUAUGCGAACCAGAUCAUAUAACCAACUUUGAAACCUUCCCUGUUAGAAAUCAGUGUAUGCAAGCAAAAAGACUUUCAGUAUCGGAAACAUGCAA
GAAAAAUCAGUGCCUGUGAUUCCGCAGUAUAUUAACACCAAAAGCCACAAACUUGCAGUUGAUCAGGUAACUGACAUGGAAAAAGAAUAUCAACAGUAAG
AACAAUGAGAGACAUUAGACAUCCUUACAUAGGCAAAGUAACGACGUGAUAUUGGCACGGCUCAAUCAAAGAGUGGAGCCCCACGCCUUUGGAGCACAAG
CUUUCUGAUUGAACCGCGCCAAUAUCCCGUUCCUGAAGAGAAUAAGCCCAACAUUAUUCUUACCACAUAGAAACACCACCAGACAACGGAGAGAAUCAAA
UAGAAGGCAACAGCAAUACACAACCUUCCUGACUAACAAGGAACGAGAACCGGAGAACUAAAACUCGCUUGAGGACACAUGAGAAACAGCAGCCAAGGCA
CU
>culm_79
GCUAUGAAAUUAUAUCAUUAUAGGAUUCAACCAUAGGCGUAUACUAAACGUUUCAGUUCAGGAAACAGUCAUAUGCACUUCAAGUCAAGAUAUCAGGAAC
AAUAUAUCCUAAGACGAACAAAUAGGUGGCAUAGCGCAAAGAGAAUCUCAUGCAUCCCAUCGUUAUGUAAAAGAAAAAAAACCAUGAAAUGUAACAAAUC
AGAUUCCCUGCACACCAUAUUCAGUUAACUCAUAAUUGGCAUUUGAAACAACUACAUCUAGUCAUCAACAAUUUCGGCUACCAAUGCAAUUCAUACAAAA
GCAAAAUGGAACAACAAGCUAGAAGAUUUAAUUUCAUGCACUUGCAAAUGCGACGUGAUAUUGGCACGGCUCAAUCAAAGAGCUGAGCCCCUCGCCUCCG
GAGCGCAAGCUCUCUAAUUGAACUGCGCCAAUACCCCGUUGCUACAAGGAUUAACUCCCUAGCUCCAUGGAAAACGGCAUUGAGACACAAGAGAAAAUGG
UUACUGCAAGGAAGCUCCAUGAACAGGAAUGGAGCAGU
>root_58
GUUUUUUCGGUGGCAUAUAAGGAAUGCUCUCUAAAAUUUUAUUUCGGUAACAUGCAAAAGUUACCGUUAACAUGCUUUUUUUUGCUAUGAAAAGGACUUU
AAGAUGUUUAUUAUAUGUACAAAGAUUACCUUUGAGCAUUAGUACCAGACCGGGCAAUAUAAACUUCUGCUGCUCCACUAACUUAGCUAACCGGCCAAAA
UAAUCUACAUUGCUACAUGCCAAGAUAGGUUUGGCAAAGUUUAUAGGAAACUGGUUUGUUGUUGACUUAAAAGUUUGUACCUUGCACAUACUCUCUAUUG
UCCUAGGAUAUGUUGAGCACUACUUAACUUAGAAGGAUAAACAGAAGUUCAACAUGCAUGCCUGAGUCCAAUUUACCAUGAGAAGAUUACGGCUUCUGUA
UGUUUUCAUCUCAAAUACAACAAGGGAGAUACAUAUUUAUGCAAUUGAAAAGUUGGAUAUAGCCCCAAUAUGUCAUCAACUCGAUCGUCAUGAUGCACUC
GUUUAAAUAGCAUGAGAUAGCCAUCAGCCAAAAACCAUAGGUCAACUACUGCCACCUAUACCUGUAACUGUGACCUGAUAAUACCACGAACAAUAUCUUU

CUGCACGACAUCAUAGGGAUUGAUUUGAUGCAAUACCAAAACUUGAUUCUAAAGGCGUGUAAAAUUUUAAAUGUAAAAUCCCAUACCUUGAGAAAGCAUU
AGUACGUCGGACUAUAAGGCUAGGGAAGUUUGUGAUAUUGGCACGGCUCAAUCAGACCCGGGAAAUAGAACAUCCCUGAACCUCGCAAGUGCAAAUUUUA
CCCGGUUUGAUUGAACCAUGCCAACAUCACACGCUCCCAGCCAAUCCUUGUCGAAGCAAACGCCAUCAUCUCACAGCCAGGAGCCAGCCUUUUCGAUCCC
CUGCACCCAAGCUCCCAAUGCAAACUCAAGAGAUAAACUUGAUUGACAAUGGAACAAGCAAGCA
>Bud_81
ACACAGCACCUCCUUUUUUCCCUCUCUAGACCAGUAACUGAAUUCCAGAGAGGGGCUUUCCUAUCAGCCAUCAUGUACUUCGUUUCAUCCAAUUAACAAG
GAAACUCCUGGAAACCCUAGAUUGCUUGGAAACCAACUCGAUCUGUCCAUGUGUUUUCGUGAAGACAUCUUGGCAUCAGACAACUUGCCUUAGAUCGAAU
CAGCUCUCCAUGAAGAACAACAGGGGGCCAGCAAUCUAGGUAGGAGUCAAGCAAGGGGAGAUAUUGACGCGGCUCAAUCCAAGAACAUGGCUGCUGUUCC
AAUCAGCCACGCCUUCGGAUUGAGGCGCACCAAUACCUCCUUAUCCUAGCAACAAGCAAGCUGAAGCUUCAUGCCUUCCCUUCAUUAUUUUUCUUGCCGU
GCGAACACAAACCAUCUCUGCAAGAUCACUCUCUUCGGCAAAUAUAAUCAGCUUAAAAAAAAACGAGAAAGUGGACAAAAGGCAGAGAUAGCUAGGCCUG
UGUGUGUGUGUGAGAGAGAGA
>culm_44
UAGAUCGAAUCAGCUCUCCAUGAAGAACAACAGGGGGCCAGCAAGCUAGGUAGGAGUCAAGCAAGGGGAGAUAUUGACGCGGCUCAAUCCAAGAACAUGG
CUGCUGUUCCAAUCAGCCACGCCUUCAGAUUGAGGCGCACCAAUACCUCCUUAUCCUAGCAACAAGCAAGCUGAAGCUUCAUGCCUUCCCUUCAUUAUUU
UUCUUGCCGUGCGAACACAAACCAUCUCUGCAAGAUCACUCUCUUCAGCAAAUAUAAUCAGCUUAAAAAAAA
>Bud_82
AUCUGUGUGUGUAUAGUAAGAAGAAGUGGAUGUCUAUGGUAGCAAGUGGAUGAUCAUGUAUGAGGCCCAAUGUGUGGCGAUAUAAUGCAAGAUCAGACGA
CGUAAUAGCACUAUAUAUAACAGUCGGUGAUUUCUGGUGUGGCAUCAUCAAGAUUCACACAGCUAUAUACAGCGUUGGCUGUCGAUGCAAGCAUCAUAUU
CUGUGAUGUAUGCCUUUGCAUGUGGGAAUCUUGAUGAUGCUGCAUUGGGAACCAAGGGCUACCAGCAGAUCGAUCGAGAUGGAAUGAAACCGCCGACGCC
GGAUCACGAUCGGAGCUAGGAAUGGAUCAAGAUGAUAUGCAUGCUUACCUGUCAUCUCACGUCUGUUGUCUAUUCCAGUGGAGAAGUGAAAGUCCGAAAG
AAAGAAAAAAAGAUGUAUCGCUA
>Bud_45
UCCGGCUGCGGUGGCGAAUUGCGGUGCGUGGCGCCUGGAAAGAUGAAUAGAUCGUGCAAAUCGACCGGUUCUUGAUUCUUCGAUUCUCUACCUCCUCCCU
GCAGUCUUGCUACCCAUCUGCAUCUCAAAUUUGUUUUGCAUCCCCCCUGUUGUUUAAUUAGAUGCAUGGUUCUUAAUUCGUUACUUACUUAUAUAUGCAU
UAUUCCUCUUCUUGAUCCAAAGUUAUGCAUAGAUGUGGAACAAACCUCUUGAUGUGUAUUGGCCGGGUUUCUGUUUUGAUUAGAUGCAUGCAUACAUAGA
UUUCCAUCUCUGAUAUAUAUAAUAGACCUUCUUGUUGGAUCACCUCGAGUCACAGGCACAUGAAGCAAACAGUCGGUGCUUGCAGGUGCAGCACCAUCAA
GAUUCACAUCGAAACUCAUCUCGAGAGAGGUGGUUUGCAGUUUCCAUAUAAACAUGUAUAUAUGAUGAGAAUCUUGAUGAUGCUGCAUCAGCAAACAUCC
ACUCAUCGACCAUUUAGCUUGAAGAGAAUGAGGAAAUCUGCUGCAAUUGAGUACUUCUAGUCCUUCUUGCCUUUAAUUACCUUGGUUUUGGAUUUUCACA
UGAAUUCCUCUCUCUUUGCACAUUGUUUUUAUCUUGCAACUAUGGAGAAAUCCUACUCUAAUUCAUGGAGUAGAUUGGCUGAGCUUGGACCUCAAUUAAA
AAGAAGCGCCGCCGAGAUGUUGUAAUUUCCCUUUUCUUCACCAAAGCUCUAUUUGAAUUGGCAUGAAUGAAGUCUUCAUUCUCAAAGGGUGGAAGUGCAA
GGAAGAGCAAGGAAGCUCGUCAUCAGUUAAUUCCCAUGGCAGCAUGUGCAUACAUGUUCAGUAAUUUUGAGAUCAGAAGUAAUAAUAGAUGCAUGGUACU
CCCUCUGGUCAGAUGAGCUUGAUGUUCAGUUCGAUGUGUUGUCUGAACGUCAAGCUGAGCGGAGGAAGUAUACCGUAGUGGCUACUCCAAUAAUUUUCGU
GUAUGUCCGUCACAGGAAGGAGAAGGAAUAUCUAUCAAGCAAGGAUACAUCAUACAUAUAUAUAUAUGAUGCAUCAUAUCAAGCCUGAGCAGUGCUAGUU
CAUCAUCAAGACGAGACGACCACACCACUGGCUGCUAGAUGGAUCAAUGUAAGUGGACCAUGCAUGCAGGGGACCGGGGUACAUACGUAGUACAUUGCUA
UUAUUGUGCCAAAGCAAGGGAAGGGGACCAAUAUAAUGUGGUGCACCGGCCGGGACAAGCCUUGCAGAGUGGUGGUACAACUACUGCUGCGUCAAAGCUA
GCUACUAGCUACUAGCUGCUGAAAAUUUUCACCUCGAAGGAUCACUUUACUUGCUUUUGCCUUCCGUAGGCAGGCUGCAAUAACCUAGGUUGCUCGGAUA
CUCCAAUACGGAUAUAGAUAUGUGAUGUAGUGAUACUUCGAUACAAUAUUUUUUUAGAAACAUUGAUAUAGCAAUACGCGUAUAUAUAUAAAAGAAAAGU
CUAGUUUACACCUUUCAAUUUUUGUAGGAGUCCAAUUUCCAUCUGUUGAGUAUAUAUAUUGUCUAAUUUCGGAGUUGUUGGUUGAAAAUCAGACUCUUGC
AAGAGUUGUAGUGUGUAAACUG
>leaf_51
CAGUUUACACACUACAACCCUUGCAAGAGUCUGAUUUUCAACCAACAACUCCGAAAUUAGACAAUAUAUAUACUCAACAAAUGGAAAUUGGACUCCUACA
AAAAUUAAAAGGUGUAAACUAGACUUUUCUUUUAUAUAUAUACGCGUAUUGCUAUAUCAAUGUUUCUAAAAAAAUAUUGUAUCGAAGUAUCACUACAUCA
CAUAUCUAUAUCCGUAUUGGAGUAUCCAAGCAACCUAGGUUAUUGCAGCCUGCCUACGGAAGGCAAAAGCAAGUAAAGUGAUCCUUCGAGGUGAAAAUUU
UCAGCAGCUAGUAGCUAGUAGCUAGCUUUGACGCAGCAGUAGUUGUACCACCACUCUGCAAGGCUUGUCCCGGCCGGUGCACCACAUUAUAUUGGUCCCC
UUCCCUUGCUUUGGCACAAUAAUAGCAAUGUACUACGUAUGUACCCCGGUCCCCUGCAUGCAUGGUCCACUUACAUUGAUCCAUCUAGCAGCCAGUGGUG
UGGUCGUCUCGUCUUGAUGAUGAACUAGCACUGCUCAGGCUUGAUAUGAUGCAUCAUAUAUAUAUGUAUGAUGUAUCCUUGCUUGAUAGAUAUUCCUUCU
UCUUCCUAUGACGGACAUACACGAAAAUUAUUGGAGUAGCCACUACGGUAUACUUCCUCCGCUCAGCUUGACGUUCAGACAACACAUCGAACUGAACAUC
AAGCUCAUCUGACCAGAGGGAGUACCAUGCAUCUAUUAUUACUUCUGAUCUCAAAAUUACUGAACAUGUAUGCACAUGCUGCCAUGGGAAUUAACUGAUG
ACGAGCUUCCUUGCUCUUCCUUGCACUUCCACCCUUUGAGAAUGAAGACUUCAUUCAUGCCAAUUCAAAUAGAGCUUUGGUGAAGAAAAGGGAAAUUACA
ACAUCUCGGCGGCGCUUCUUUUUAAUUGAGGUCCAAGCUCAGCCAAUCUACUCCAUGAAUUAGAGUAGGAUUUCUCCAUAGUUGCAAGAUAAAAACAAUG
UGCAAAGAGAGAGGAAUUCAUGUGAAAAUCCAAAACCAAGGUAAUUAAAGGCAAGAAGGACUAGAAGUACUCAAUUGCAGCAGAUUUCCUCAUUCUCUUC
AAGCUAAAUGGUCGAUGAGUGGAUGUUUGCUGAUGCAGCAUCAUCAAGAUUCUCAUCAUAUAUACAUGUUUAUAUGGAAACUGCAAACCACCUCUCUCGA
GAUGAGUUUCGAUGUGAAUCUUGAUGGUGCUGCACCUGCAAGCACCGACUGUUUGCUUCAUGUGCCUGUGACUCGAGGUGAUCCAACAAGAAGGUCUAUU
AUAUAUAUCAGAGAUGGAAAUCUAUGUAUGCAUGCAUCUAAUCAAAACAGAAACCCGGCCAAUACACAUCAAGAGGUUUGUUCCACAUCUAUGCAUAACU
UUGGAUCAAGAAGAGGAAUAAUGCAUAUAUAAGUAAGUAACGAAUUAAGAACCAUGCAUCUAAUUAAACAACAGGGGGGAUGCAAAACAAAUUUGAGAUG
CAGAUGGGUAGCAAGACUGCAGGGAGGAGGUAGAGAAUCGAAGAAUCAAGAACCGGUCGAAUUGCACGAUCUAUUCAUCUUUCCAGGCGCCACGCACCGC
AAUUCGCCACCGCAGCCGGACAAGAAGGGGCCGAGCCGGGAUGAUAGAUCGAUCUACACGUGUGUGUGUGUAUAGACUAGAGAAAGUAGUAAGAGUAGAU
GGAGGAAGAAGAAGAAGACGAAGAAGAAGAAGAAGAAGGGGAAGAUUGGUUGGCACUUGGCAAUUGGCAUGGCCACCUCACAUAGAAAGAAGCAGCUUAG
GAGAGGGCCGGGAGAUGAGGAAGGGGGUCGGCUGGCUACAUCUGGGCAAGAUAGAUAGCUUGGUAUAUAUAAGGCCCUGGCUAGAACAAGAGGGGGAUCG
AAGGGCAGCAAAAGCAAAAUCCUAGACAGAUAUAUAGGAGUGGUACUGGAUCUUGUUGUUUGUAGCCUCGUAGCUAGCUAGGCCCUCAACCUCCCCUCCA
UGGAGGAUCGAGGUGAAGGACUAAUGGAAUGGAGAAGGGAGAGAGGGGGAGAGUAAUGAUAAGGUUUAGAGGCAGUGAAAGGCCGGUGCU
>root_88
UCCUAUGACGGACAUACACGAAAAUUAUUGGAGUAGCCACUACGGUAUACUUCCUCCGCUCAGCUUGACGUUCAGACAACACAUCGAACUGAACAUCAAG
CUCAUCUGACCAGAGGGAGUACCAUGCAUCUAUUAUUACUUCUGAUCUCAAAAUUACUGAACAUGUAUGCACAUGCUGCCAUGGGAAUUAACUGAUGACG
AGCUUCCUUGCUCUUCCUUGCACUUCCACCCUUUGAGAAUGAAGACUUCAUUCAUGCCAAUUCAAAUAGAGCUUUGGUGAAGAAAAGGGAAAUUACAACA
UCUCGGCGGCGCUUCUUUUUAAUUGAGGUCCAAGCUCAGCCAAUCUACUCCAUGAAUUAGAGUAGGAUUUCUCCAUAGUUGCAAGAUAAAAACAAUGUGC
AAAGAGAGAGGAAUUCAUGUGAAAAUCCAAAACCAAGGUAAUUAAAGGCAAGAAGGACUAGAAGUACUCAAUUGCAGCAGAUUUCCUCAUUCUCUUCAAG
CUAAAUGGUCGAUGAGUGGAUGUUUGCUGAUGCAGCAUCAUCAAGAUUCUCAUCAUAUAUACAUGUUUAUAUGGAAACUGCAAACCACCUCUCUCGAGAU
GAGUUUCGAUGUGAAUCUUGAUGGUGCUGCACCUGCAAGCACCGACUGUUUGCUUCAUGUGCCUGUGACUCGAGGUGAUCCAACAAGAAGGUCUAUUAUA
UAUAUCAGAGAUGGAAAUUUAUGUAUACAUGCAUCUAAUCAAAACAGAAACCCGGCCAAUACACAUCAAGAGGUUUGUUCCACAUCUAUGCAUAACUUUG
GAUCAAGAAGAGGAAUAAUGCAUAUAUAAGUAAGUAACG
>culm_69
UUGGCACAAUAAUAGCAAUGUACUACGUACGUGCCCAGGCCGGCCCUCUGCGGUCCACUUGAAAUUGAACGAUCUAGCAGCAAGUAGUGAUGUCUCGAUG
AUGAACUGGCACUGUUCGUGAUGCAGCAUCCUUGAUAUUCCUAUGGCUACAGGCCCUUGUUUCAUUAUUUACCUCAAAGUUAAGCUUCAAUUGAACAAAG
UGCGCACAUUAUGCGGAGGGUGUAACUGAUGAUAAGCUUCCUCCUGUCGCUCAGCCGGUCACAAAAGGGAACUAGCUUGCAAUUAAACUCAUUACACACC
AUAAAAUCAUGGUUUCCCCUUAAUCAUCAAGUAUUCUCCCCAACCAGCAAUGAACCACACAAAUCCAAGGUACUCGCGUUGCACUCCUUGAGAAUAUAAA
GGCUCAAUGCCAAUUGAAAUAGAGCUUGGCGAAGGAAAGGAAAAUUACAACAUCGGCGGCACUUCUUUUCGGGUGUUUUUUUUUUUGCGACCAACUUCUC
UUCGGGUGUUCAGCCUCAGUCAAUCUACUUCGGAAGGAGCUGAGUAGGAUUUUCUUCAAGUGAUCGACGAGUGAGUGUUUGCUGAUGCAGCAUCAUCAAG

AUUCUCAUCAUGUAUACAAGUGUAGAAACUGUAAACAACACCUGCCUCUCAAGAUGAGUUGGAUGUGAAUCUUGAUGAUGCUGCACCUGCAAGCACCGAC
UGUUUGCUUCAUGUGCCUGUGACUCGAGGUGAUCCAACAAGAAGGUCUAUUAUAUAUAUCAGAGAUGGAAAUCUAUGUAUGCAUGCAUCUAAUCAAAACA
GAAACCCGGCCAAUACACAUCAAGAGGUUUGUUCCACAUCUAUGCAUAACUUUGGAUCAAGAAGAGGAAUAAUGCAUAUAUAAGUAAGUAACGAAUUAAG
AACCAUGCAUCUAAUUAAACAACAGGGGGGAUGCAAAACAAAUUUGAGAUGCAGAUGGGUAGCAAGACUGCAGGGAGGAGGUAGAGAAUCGAAGAAUCAA
GAACCGGUCGAAUUGCACGAUCUAUUCAUCUUUCCAGGCGCCACGCACCGCAAUUCGCCACCGCAGCCGGACAAGAAGGGGCCGAGCCGGGAUGAUAGAU
CGAUCUACACGUGUGUGUGUGUGUGUAUAGACUAGAGAAAGUAGUAAGAGUAGAUGGAGGAAGAAGAAGAAGACGAAGAAGAAGAAGGGGAAGAUUGGUU
GGCACUUGGCAAUUGGCAUGGCCACCUCACAUAGAAAGAAGCAGCUUAGGAGAGGGCCGGGAGAUGAGGAGGGGGGUUGGCUGGCUACAUCUGGGCAAGA
UAGAUAGCUUGGUAUAUAUAAGGCCCUGGCUAGAACAAGAGGGGGAUCGAAGGGCAGCAAAAGCAAAAUCCUAGACAGAUAUAUAGGAGUGGUACUGGAU
CUUGUUGGUUCUAGCCCCUGUAGCAGCUGGCUUGGCUUGGCUAGCUCCAUGGAGGUGGGGUGAAGGCAGGCAGGACUAAUGGAGAAGAGAGAGAAUAAUA
AUGAUAAGGUU
>culm_55
UCAUUUCUCCUCCCAACUGCUAGCAAUAUAGCUAGCCCCGGAAUACAAGAAACCUCUUCCUCUUCCCCUUCUCCCAGUUGUAGCUUCUCCUCUCAAGGUU
CCCCCCUCCAUUGCUUGUUUUACUGGUAUACUGUUUUGCAAGCUCUAGAUUGAGUUUCUGUCCUGUUAAUGAUCUGGAUGAGACAAACCGUGAAUUCUUA
UAUCGACAUCAAGUGAAGCCCCUGCAUUCUGUCCACAAUUUUCUCUUGUGUUUUUGUGUUCUCAUUUGGGGCUGGGAGUUCAUGGCUUAUAUAUGAAAGA
GAGCUCUCUUCAGUCCACUCUGAAAUGGCUGUAGGGUUUCAUUAGCUGCCGAUUCAUCCAUUCACCUACCAAGAACCAUGAAGAGGGAUAUGUUCUUGGU
AGCCGAGUGGAUGGCGCGGGAGCUAAAAUCAAGCUCUGCGCUGUUUGUGGUUGGACUGAAGGGUGCUCCCUUGCGCCAACACUACUGCAUGAAAUCAUCU
GCCGUAACACCAAUCUUACGUCUUCUAUUUGGAUAUAAUCAUAUUCGCUGGAUUUCUCUUGAUGUUUAAUUUGACUUUCUUUUGGGAGGAAAACUGUGUU
CAAAUUUCUUUCUUCAGAUUUUUUUAAAGAGAGAAAUCAAACUGCAGUUCACAAAAGAAUAUGAUGCAUACUUUUGGAUUUCUCUUGAUGCGUGCUUUUG
GGUUUAUUCAGAAUCUUUUAUGUUGCUCUUUUCCAGCCUGCCUGGGGUGGUUUCUCACCCACAAUUCUUUUUCAUUAUUUUCAAACUGUAUAAUUCUAUU
CUUUUUUAUUUUGCUGUACAUUGGUGAGGCCGGGCGUCUCCAACUCUCUGCCGCUGCUCUCUUCAAGAUACAAGUUCGUCAACAUUUCGAAUUUGUUACA
UGGAAACCUUUCUUCUGGUCCACAUUUGUGUUGAGCGAUUAAUGAAAGUAUCUCAAUGGUAAGGAGAUUGUUUAUCAUCGAUUAUUAAUAUUCGUCAGGG
CAGACCGAUUCAAUGUAUGAAGAAUCCAAUGAACGAAAUGCCGCAGCUAAAAGUUUCUAUUUUGCAACUCCUGUAUGUCGGUUUGCUGCUACAAAUUGUU
CUUACUUAGAAGAAUUAAGCUUCAUCUUGUGUUUUUUUCAAGGGGUUCUUGUGAAAUUAGAAAUAUGAACUAUGCUUGCUGUAUUUAAUUCCUAUCACCA
UGUACAUAGUUAAUUAGUGCAGAUAUUUCGGACUAAUUUUUGUUGAUUUCUGUGUAAAAAGGCAGUGGUGCUACCAGGCACAGUGUACCUUCUUGUUUGU
CCGGAAUAGUACUGAGAUCUUCGUCUUGUCCAUUUCUCAAAUGAGUGCAUCUGCUUCGAUUACCAUUUCUUGUACAACUAUACUAAAGAAUUUGUCGCGG
AAAACAGGCUCACCUUAAUGGAGUACAGAGAGACAUCUGACAUGGAAAUUGGUUAGAUGUAAUAAAUGAAUAGUUAAGGUAGUUCAAUGAUUUCUUUUGU
ACCAUAUAUAUUGGUUGCGAUGCUCCAGUUACACUCAACUAUAAAAUUCACGAAAAUCCAUGUGGAAUUCCUCUGUAAGGAUCGAACUACAUGUGGGUGC
AAUUGUUACAUUUUUUUAUAUUGAGUUUCAGUUGUUGGAAUAUCUGAUCUUCUCUUUAUUCUUCCUUGCUUUGUUGACAGCAUGUAAAGCAUUUGAAGCU
UGUAUUUUACUCUUCACCCCUAUAUUUACAGGA
>root_32
CUCCUCCCAACUGCUAGCAAUAUAGCUAGCCCCGGAAUACAAGAAACCUCUUCCUCUUCCCCUUCUCCCAGUUGUAGCUUCUCCUCUCAAGGUUCCCCCC
UCCAUUGCUUGUUUUACUGGUAUACUGUUUUGCAAGCUCUAGAUUGAGUUUCUGUCCUGUUAAUGAUCUGGAUGAGACAAACCGUGAAUUCUUAUAUCGA
CAUCAAGUGAAGCCCCUGCAUUCUGUCCACAAUUUUCUCUUGUGUUUUUGUGUUCUCAUUUGGGGCUGGGAGUUCAUGGCUUAUAUAUGAAAGAGAGCUC
UCUUCAGUCCACUCUGAAAUGGCUGUAGGGUUUCAUUAGCUGCCGAUUCAUCCAUUCACCUACCAAGAACCAUGAAGAGGGAUAUGUUCUUGGUAGCCGA
GUGGAUGGCGCGGGAGCUAAAAUCAAGCUCUGCGCUGUUUGUGGUUGGACUGAAGGGUGCUCCCUUGCCAGGAAAUCUGCAUGAAAUCAUCUCCCCGCAA
CACUAAUCUUGUUUCUGAGAGACCCUUUACGAUUUACUUACUGCACGACCUUUGUUUGGAUAUAAUCAUCUUAGCAGGAUUUCUCUUGAUGUUUUUACUU
UUUUGGGGGAGGAAAAUCGUGUUCAACUUUCGUGGAUUAUAUUUUCUUAAUGAGAAAUUAAGCUGCAGUUCAAGAACUAUCCAUGCUUUAAUUUGGAAUU
AUUCAGAAUUUCUUAAGUUGCUCUUUUCCAGCCAGACUGGAGCUUGUGGUUUGUCACGAACAGUUCUUUUUUAUUAUUUCCAACCUGUAUAAUUCUAUUC
UGCUUGAUUUUGCUGUUUAUUGGUGAGGUCGGAUGUAUCCAACUGUCUGCUCCUAUCCUCCUACUCUCUUCAAGAUUCAAGUCUGUCAGCAUCAACAUUA
AUGACAUUUCAAAUUUGUUACACGGAAACCUUUCUUCUGGUUCACAUUGUGUUGAGUUAUAGAUUAUCAGUACUCUUCAGGGCAGAUUCAGUGUAUGAAC
AAUCCAACGAAGUAAACGCCGCAGCUAGAAGUUUUCUAUUUUGCAACAUCUAUAUGCCGAUUCGCUGCUACAAAUUGAUCUUACUUCGAAGAAUAAAGGU
UCAUUCUUGUGUCUUUCUUCAAGGGGUUUUUGUGAAAUUAGAAGUAGGGACUAUGCUUGCUGUAUUUAAUUCCUAGCACCUUGUACAUAGUAGUAUGUCA
UUAAGCACAGAUUUCGGAUUCAGUUUUGUUGAUUUCUGUGUAAAAGGGCAGUGGUGCUGCCAGGCACAAUGUACCUUCUUGUCCGGAACAGUACUGAGAU
CUUCGUCUUGUCUAUUUCUCAAAUGAGUGCAUCGGCUUUGAUUACCAUUUCUUGUAGUACUACCUUUCACUAUGGAAACUGGUUAGAUGAAAUAAAUCAA
UAGUGAAAGGUAGUUCUAUGAUCCCCCCCCCAUGCCAUAAAUAUUGUCUGCGAUGCUCCAGCUAUUCUCAACUUUAAUAUUCAUGAAAGUUCAUGUGGAA
UUCUACAUAUGGGUGCAAUUGUUAAAUUUUAGGAUUGAGUUUUAGCUGUUGGAACAUAUAGUCCAUCUACAUGUGGAAU
>root_16
AGAGAGAGAGAGAGAGAGAGAAGCAACAACAAUGUCUGCGGGGAAGCAUCCAAAGGGAUCGCAUUGAUCCUUCAUUCAUCCAUGGUGGUCGUCGUCUACAAG
CGCGGCUCGAUGGAUCAUGCGAUCCUUUUGGAGGCUUCCUUCAGCCAUUACUCGAUCUUGGUAUUCACUCUCUGGCGCUUGAUUGGCUUCGUCGUCCUUC
ACAUCUUUCACAUCUCCUAUUCACCUGGGGACGUUGGCUAUUUUAGCACUGCUUCCCAUCUCCUAUGACUGUUUCACAAGAAGGUAUGCAGUUAAUUGCUU
UUGCAGGCUGUGAUCAGUGCGAUGUUGGCUAGCUUGACCCUUUCUUGCUUAAAAUUUGCUUGUUUUAUAAUAAGCAUGACCAGACAGCUUCCGGUGCAGU
UGCAUGCUGACAAUUGACGAAAGUAGACAAGCUUUGCGAGGCUGUACCAGCUGGUGUGUAUUCUUGUCUUCUGUAGGUGAAUGAAAGAUUUGCAAAUGAA
ACCAUCUGUUGUUAGAUACUUGAUACAUGGUAAAUUAGUCCCCGGAGUAAAAAAGAAAAGGUGACGUUUAGGAUGACUGCAGUUACACCUUCUAAGCGUU
GGCUAUCGGUACCUAUACGGAUAUUAAUUUAUACUGAUUGCAUAAAAAUGAGAUAUACAUGGAGAGAUUCAUAUAUCUUUAAGUACUGUCAUGCUUUUCU
AGUUUAGUAAGAACGUGUCACAGUUUAUAUUGUCGAUGGAAUGACUGAUCAAUUUGCAUUUUAAGGGCGUCGAAAAAGUCGCUUACUUGCGACUAAACUA
AAACUCAGUAAUUGGCAGCUUCCCUUGUGUUCUGGUAGAUUCAACGAACGUCCUGUCCCCCAAUUAAUCUGCCAUAUUUAUUUGUAGCAUCGAGCAUAAU
UACUUCAGAUGCACUGGAAUAAACUAGUGACUGAAAGGACUGUCUCCCAAGCUGAUCAUCAGAUGCAUGCAGGCCCUAGCAGUACUAUUUAAGAUUAAUA
AGUUAGGUAAGCAGCUAGGCGGGCCUCCAGAUAGAUCUUUCGCUCAUCACAUGACCUAUACUAUAAGGAUAAUAAACAGCAUAGACAUAUACCGUUAGUC
GGUUAGCUAACAAAAAUACCGGAUCGAUGCUGCCUGCUUGUACAGUCGCGGUGCCAGAUGCCCUUUGACCGUCCGAGAUCCAGUCUCCUCGCAGGCAGCA
UGUGCAUGCACAUCAAAAGUGAAGC
>Bud_52
UUCAGGAAGCUAGUGGAGGACUCCAAAGGGAUCGCAUUGAUCUAAUCCGCCGCCGCCGCCGGCGUGCAAGCACAAGUAAUCAUGCAUGUCACAGAUGCGU
GCAUGGCUGCGGCGCGCGAGGACAAGAUCAGUGCAAUCCCUUUGGAAUUUUCCACUUGCGCCUCCGUACCUACGCUUCCAUCGGUCUCCACGCGAGCCAA
CGUCGUCUAUCGAUUCGCCGGCGCCCGCGCAACGUCGUCGAUGGGCUGCAUGGAUCGUGAAUUUGGAGAGCUACACAUCGCUAACCGAUCGACCGGUAGG
GUUCGAGGCCUCAAUUAAUGGUUUCUUGA
>culm_53
CUCAUAAAUGGAACUUGUCUGGGCCUUGCAUCUUUUACCCUAACCGGCAAAAGAGCCUCUAUCACUGCAGAUAUUACAUAUGUGUGUGCACUUUAUUUAU
ACAGAAAUUAAACAUGUUAGUAAUGCAUGCAGCAUGCAUAGCUAAUAUAUAAUAUAAUACAUAUUAAGUUCGCCCAGAAUGCGCUUCUUAUGUGCAAUCA
UUCCUCAGCUGCAAUUAUAUUUUUUAUUUGAUUGUACGCAUGAACUCGAAACCUCUCUGUCCUGAUAUAUAUACCUGUUCUAAAAUUGUUCUUAUUGUUG
CGUAGAGAGGGGUCAGAUGUUAGAAUAGCCCCCCGCGUCAGGGACGUUAAGGAUCAGAUUCUCUCUACGAUAGAUCUGGCUGGUACUACGUUGUUGCGUA
GAGAAAAUUAAAGAUAGCAGGACUGGCUCUGUUGAGAGAGAAAGAUGACAAAAUUUGGAGCAUAUAUGCUAUCAGUUGCGAGACGCAAGAACUGAGAACA
AGAUAAUGAAGUAAUAUAACCCAUCAUCUCUGACCAAGAAAGGAAGAAAAGAAGAUGUAAAUUGUCAACAUGCUUAAGAUUUUUCGCGUAAAGUUUAUGC
AAAACAGACACUCCACGUUUCUUGACCACUAUCCUUUCCUUUCCGUUUACCUCCACCUGAACCACUUCGAUCGGCAACCACUGCAAAUUAAGGUUGAACA
GUGACGAUCCUUGCAAGUUGCCAGGGAUAGUUCCUCCCAAAUUUUACCAAACAAGCAAUUCUCUCAAGGUUAUUCGUUGCGAAAAGAAAAAAACAUAUAU
UAUGGAAGCUAUAAAACUCUAGAGAUAUGCAUGAUUCAUGGGCACAUCUAUAUGCGCUAUGCAUGUAUAUAUGUUUGAAGAAAAAAUAUAUCAAAUUUCG
UAAACAAAAAUGUCGAGCCAGUUGUAGAGCAAGUAAAAACAAAUAACCAUGAAACUUUUUGCGAUCGAUUAAAUCCGAAUCAUGGAGACUCCAUGUCUAA
UUACAUCUACGAUCGAACUAGCUACUAGUGGUACUACCUCAAAGUUUGCCUCACGAAACCAUUAAUUGAGAUAAUCGAACCCUACCGGCUGGUUCUCCAA
AUUAAUUUCAGUCCAGGAUGCAUGUUAUGUUAGUGCGUACCUUAGCCGGCCGAUCGACGACGUAGGCGUGAAGAGCGAGGUGUACGUGCGGAGGCGCAAG

UGGAAAAUUCCAAAGGGAUUGCACUGAUCUUGUCCUCGCGCGCCGCAGCCAUGCACGCAUCUGUGACAUGCAUGAUUACUUGUGCUUGCACGCCGGCGGC
GGCGGCGGAUUAGAUCAAUGCGAUCCCUUUGGAGUCCUCCACUAGCUUCCUGAAAGAAGAAGAAGAAGAAGAAG
>leaf_37
UUGUUCCUUUUUGUUGCGUAGCUAGGGAGAAAAGAAAGCAGGACCGCGUAUAUUUGUUCUGAAAGUGAAAGAUGACAAAUUCUGGAGCAUAUAUGCAGCA
GUCAGUUGCGAGACGCAAGUUAACGGAGCAAAAGAGAAUUAAGUAACUCAUCAUCUGACCAAGAAAAGGACCAAAAGAAGAUGCAAAUUGCCAACAUGUU
UAAGAUUUUUUUGGUGUAAAGUUUGUGAAAAAACCGAUGCUCCACGUUUCUUGAUGAGCACUAUCCUUUUGUUUCCAUUUAUCUCCACUUGAACCACUUC
GGCAACCACUACGUACAAAUUAAGGUUGAACAGUGACGAUCCUUGCAAGUUGCCAGGGAUAGUUCCUCCCAAAUUUUACCAAACAAGCAAUUCUCUCAAG
GUUAUUCGUUGCGAAAAGAAAAAACAUAUAUUAUGGAAGCUAUAAAACUCUAGAGAUAUGCAUGAUUCAUGGGCACAUCUAUAUGCGCUAUGCAUGUAUA
UAUGUUUGAAGAAAAAAUAUAUCAAAUUUCGUAAACAAAAAUGUCGAGCCAGUUGUAGAGCAAGUAAAAACAAAUAACCAUGAAACUUUUUGCGAUCGAU
UAAAUCCGAAUCAUGGAGACUCCAUGUCUAAUUACAUCUACGAUCGAACUAGCUACUAGUGGUACUACCUCAAAGUUUGCCUCACGAAACCAUUAAUUGA
GAUAAUCGAACCCUACCGGCUGGUUCUCCAAAUUAAUUUCAGUCCAGGAUGCAUGUUAUGUUAGUGCGUACCUUAGCCGGCCGAUCGACGACGUAGGCGU
GAAGAGCGAGGUGUACGUGCGGAGGCGCAAGUGGAAAAUUCCAAAGGGAUUGCACUGAUCUUGUCCUCGCGCGCCGCAGCCAUGCACGCAUCUGUGACAU
GCAUGAUUACUUGUGCUUGCACGCCGGCGGCGGCGGCGGAUUAGAUCAAUGCGAUCCCUUUGGAGUCCUCCACUAGCUUCCUGAAUGAAAUUGAAGAAGG
AGAAGAAGAAGAAAGCGCCUAGCUCAUCACUGACAAAGAAUAAUUGCUGCUGCUUACUUUCUGAUAUAUGCAUGCCUGCCAUGGUAGUAGCUAGCAUAGC
UAGGGGAGAGGGGUUUAUAAUUAUAACGCCAUGGAUUGGAUAGGAAGAGAAGAGCUCGCAUAGAGACACACACAAGAGGUAGAGAGAGACGAGGGAAGGG
GAGGGGAGGCAGGUGAAAGCUACAUGGCAGGCAGGUGUUGGGGAUGGCUGUUGCUUCGCUCGCUAUAGCUGCCUCCCU
>culm_40
GAUGACGCCCCAAGCCUAAGCUCCUCUGGCCAGUUGCCGCUAAGACUCGCGCCACAGAGGGUGCCGCCGCCAUGGACGAGCAGCUCAGCGAAGGAGAUGG
AAGAGUGGCGCAAGUCCGGGACUUGUGGAGAUGUAGUGGGUGGUGGACAUGUAGAAGGGAUAAAAGAUGGGCUUCAGCCUGCGCUGACUGCUCUGUCGUC
UCCCACAUCAGUAUCAGGGAUCUGUGCUGCCCUGCCGCGCCCGACGACAGCCGCAAGAUGAAUCGGUCUUGGCUUCGGGCAGCUGAUCCGGGACGGUGCU
GUUGGAUUGCUGCACGAGGCCGAACGAGGGACACACCAAAAGCCAUCACGUUCACCGUGGUUUUUUUUGGGAUUUCUCAAUCGGACAUGUAUGUGGGAUU
UUCUCUGAUUCGAUGCUCCAUUAUUUCCUCUGUGCCUGAGGCUAACUACAAGAGAAGACCGUUGGCUGCUUUCACAUAUCUCCCUCUCGUGUGCUACUCA
UGAUGUAGAAUUUUCCCUGGUCAGCAUGCAUCUACAAGCUGGAGCUGACGAAUACUCCACCAUGCUGCUCACUUGGUCUCUCUCUGCAAUUUCCCACAGC
UUUAUUGAACUGCUCUGUAACUCAAGCCAAAAUGCAAUCUAUUCUAAAAGAGCUGCCACUUCGAGCUCAUGCAGUUCAAGAAAGCUGUGGAAGAUCACAA
AGAGGGCCAUCAUGCUUCUUAAUAUGGAUGCCCAAUCAGUAGCAUGAAAUUAACCAACCAUUCAUGAAGCUGAAAAUUGGAAGGGAUAGAAGCUAUGGCU
GUUCAGUGUUCACUUCUUUGUGCUGCAUAUGUAGGAGCCUGAAGUUUGAAUGUCAGGUGCAAGAAGCAACUGCUGCUUGUG
>culm_41
GAUGACGCCCCAAGCCUAAGCUCCUCUGGCCAGUUGCCGCUAAGACUCGCGCCACAGAGGGUGCCGCCGCCAUGGACGAGCAGCUCAGCGAAGGAGAUGG
AAGAGUGGCGCAAGUCCGGGACUUGUGGAGAUGUAGUGGGUGGUGGACAUGUAGAAGGGAUAAAAGAUGGGCUUCAGCCUGCGCUGACUGCUCUGUCGUC
UCCCACAUCAGUAUCAGGGAUCUGUGCUGCCCUGCCGCGCCCGACGACAGCCGCAAGAUGAAUCGGUCUUGGCUUCGGGCAGCUGAUCCGGGACGGUGCU
GUUGGAUUGCUGCACGAGGCCGAACGAGGGACACACCAAAAGCCAUCACGUUCACCGUGGUUUUUUUUGGGAUUUCUCAAUCGGACAUGUAUGUGGGAUU
UUCUCUGAUUCGAUGCUCCAUUAUUUCCUCUGUGCCUGAGGCUAACUACAAGAGAAGACCGUUGGCUGCUUUCACAUAUCUCCCUCUCGUGUGCUACUCA
UGAUGUAGAAUUUUCCCUGGUCAGCAUGCAUCUACAAGCUGGAGCUGACGAAUACUCCACCAUGCUGCUCACUUGGUCUCUCUCUGCAAUUUCCCACAGC
UUUAUUGAACUGCAGCUACGCAAGUAGAAGCUAGCUGCAUCUCCAGCAGAUGCAGUUCAAGAAAGCUGUGGAAGAUCACAAAGAGGGCCAUCAUGCUUCU
UAAUAUGGAUGCCCAAUCAGUAGCAUGAAAUUAACCAACCAUUCAUGAAGCUGAAAAUUGGAAGGGAUAGAAGCUAUGGCUGUUCAGUGUUCACUUCUUU
GUGCUGCAUAUGUAGGAGCCUGAAGUUUGAAUGUCAGGUGCAAGAAGCAACUGCUGCUUGUG
>Bud_18
UCGAUAUGGCAUUGAUCCUCCAAUUCAGAAUAAAAAGACAUUAGGAGCACCUUGUAUGAGUUGAUAUUUCAUUGAACUAACCUUGGAUUAUUUUGAUUAG
AUAUGCACUUGGGGGCAGCAAGCUAGAUGCAGCAACUGCAUAAAUUGCAAGAGCUUGUUGGUUGAGGAUCAUACCGAUGGUGCUCUUGCAAGUUAUGCAG
UUGCUGCCUCAAGCUUCCUGCCUCCCUUUGCCAAAGCUAGUAAGAUACAGAGCUUUCAAUUAACAUAGAUGUAUAACUUUGCAGCCAUAACUGCAUAUGG
GAUUCAAGAAAUAGAAAUACAGCAUGCAAUCAGAUGCUACAAGGCUAUGUAAACAUAAUAUACAAUAUCAUUUUUGUGAGAUAGCAACUUACACACGAGG
AUAUCUUUGGUUUGAAAAAUGCUUAAUAGUUUAAGACUGAUUAGUCAAAGGAUAUUGUUUGACUGUUCAUGCAGCAACACAAUCAAAUCUUGAAGUUGGU
AGUUAUUACUUUGUUUGUCAUCCUUGUUUACUUAACUGAAAUUUUUACCUCUCAAGAUAACUUGAUGUGGAUAUUCAUUGUCCCUUUAAACAUGUUGCGA
UUACUCUUUUCUUUAUGUAACUUAAGUUCACUUCGAUAUUCUUCUCCUGUUAUACAUAAACAUCAGAAAAUGUUAUCAUUUAGCAGAACUUGAAUGGUGG
AUUGAUGUAUCCUGAACAUGUUACUUAUAAUAUAGAACGAACUUGAACAAAUUAGCCAUAAUAUUUGUGCUUGAGUAAAUGCACUUAACAUAUUGGAGGU
AGUCAGUGGCCAGUAGGUUUAAAUACUGCAUGAUACAUAUUGCACUAAGUUACAAAUAUUUCACACCUUUUCAAACUAUUUUUCUUGGUUGUUUUGUAAA
CUUACACCAUUUUAUACUAUUUUGAAAACAAAUGUAAAAAAGUUGUUGUAGCUUGGUGCUUGCGGGUUAAUGUUAUCACUUGAAUCUGGUGGAUUUAUAA
UCACCUUCCUGAUGUCUUGUCAAGAUUCUGCAGUUAGUAAUAUGGGUCUCCAAGACUCCAACACAAGUUAUGGAGGAUGAAUGGUCGUUGGGACAUAAAA
AGUGUUUGUAACAGUUUUGGGUUAACAGAAUUUAUGAAUAUAGCUGGUGAUAAUUUACAGCGGCUGCUUCAUUAUGUAGCAUUAAAGUCACCUAUUGAUG
AUCUGGGCAUGGUUACUUUUCAAUAGUUUGUGUGGAAAUGUAGAAUGCAAAGAAGAGUGAUGACUGAUGAUAUAUUAUCACUCAUCAUGGAUCCAUGCUG
UUGGAACGAUGGUUAUGAUCCGAUAUGUUAUCACUUAUCUU
>culm_71
AUGAAUAUCCACAUCAAGUUAUCUUGAGAGGUAAAUGUUUCAGUUAGGAAAACAAGGAUGACAAACGAAGUAAUAACUACCAAUUUCAAGAUUUGAUUGU
GUUGCCACAUAAACAGUCAAACAAUAUCCAUUGACUAAUUGAUCUAAAACCAUUAAGCAUUUUCAGACCAAAGAUAUCCCCGUGUAUAAGUUGCUGUCUC
ACAAAAAUGAUACUGUAUAUUAUGUUUACAUAGCCUUGUAGCAUCUGAUCGCAUGCUGUAUUUCUAUUUCUUGAAUCCCGUAUGCUAAAGUAAAGAACUU
AUGGCUGCAAAGUUAUUCAUCUAUGUUAAUUGAAAACCCUGUAUCUUACUAGUUUUGGCAAAGGGAGGCAGGAAGCUUGAGGCAGCAACUGCAUAACUUG
CAAGAGCACCAUCGGUAUGAUCUUCAACCAACAAGCUCUUGCAAGUUAUGCAGUUGCCGCCUCUAGCUUGCUGCCCCCAAGUGCAUAUCUGGACGGAUCC
CUCCUCGCUCCGUCGCGAGGUCGAUCCCCCCCGCGCGCUCGCUCCACGAAACCUAAACCCUAGAUCAAAUAUCAGGUUGGGAGUUGGGGGAGAGUUGUGG
AUGGUGAGGGGGGAGGGGCCGCGUGGUGCGGGAGUGGAGUCUAUAGGCAGCCGCGGAGGCAGUCGUGAGAGCGGAGUGCUUGCAGGAUUAAA
>leaf_35
UCAGUUAGGAAAACAAGGAUGACAAACGAAGUAAUAACUACCAAUUUCAAGAUUUGAUUGUGUUGCCACAUAAACAGUCAAACAAUAUCCAUUGACUAAU
UGGUCUAAAACCAUUAAGCAUUUUAAGACAAAAGAUAUCCCCGUGUAUAAGUUGCUGUCUCACAAAAAUGAUACUGUAUAUUAUGUUUACAUAGCCUUGU
AGCAUCUGAUCGCAUGCUGUAUUUCUAUUUCUUGAAUCCCGUAUGCUAAAGUAAAGAACUUAUGGCUGCAAAGUUAUUCAUCUAUGUUAAUUGAAAACCC
UGUAUCUUACUAGUUUUGGCAAAGGGAGGCAGGAAGCUUGAGGCAGCAACUGCAUAACUUGCAAGAGCACCAUCGGUAUGAUCUUCAACCAACAAGCUCU
UGCAAGUUAUGCAGUUGCCGCCUCUAGCUUGCUGCCCCCAAGUGCAUAUCUGGACGGAUCCCUCCUCGCUCCGUCGCGAGGUCGAUCCCCCCCGCGCGCU
CGCUCCACGAAACCUAAACCCUAGAUCAAAUAUCAGGUUGGGAGUUGGGGGAGAGUUGUGGAUGGUGAGGGGGGAGGGGCCGCGUGGUGCGGGAGUGGAG
UCUAUAG
>Bud_03
GCGUCGCGUCGCCUCCUCGACUUCUCUCUCUACCUAGUUUAUAAUCCACCAUCCCGGCUCGGUGUUGCCUUCUCCUACCCUUUGCAUAGCACAAGCGCCC
CUCAGAAUCACAAUUCAGAAUGGCCUUGCUUCCCUCCCCCGUCUCUCGAGCCGCCGCGGCGCGCCGUAGUCUCAGAUUGAUGCAAGGGGUGGUGACAAGC
UAGAGGCAGCAACUGCAUAUCUUGCAAGAAAAUUGGUUUUCGGACCAUACCGAUGAUUUUCUUGCAAGUUGUGCAGUUGUUGUCUCAAGCUUGCUGCCUC
CCUUUGCCAAAACUAUCAAAAGUAAAAGCACGGCUUAGGGUAUUGUUGAACCCCUAAGAAAAAAUAAAAUUAUAAACAUGUGCCAAUAACAAAUAUGAUG
AAUUAUUAAUUGAGAUCCUGUGGCAGGAAGUAAUAUUUUGCAGCAUACUUAACACAAUUGCUAUCCAGCACAAAACUAAGAUGGAAAUGACAUUUAUUGU
UAAGAAAACACAAGCUCGCUUGUCCAUUUUUGCAAUUUGGAGGCUAUCCUUAUGGUCUUGUUUAGUUUUGCAUUAACAAGCAGUAUUUUAAGGUGGUGAU
UGGUGACCUAGCAAACAAUAUAUUUCAGCAGACACACUGCCAUUACGCAGUGGCAAGCAAACUCCAUGAAGUCGUAACAAGGUGAGUGAAAAUUCUGAUA
AAAAGAACAUGUCAUCAGUAUAAGUAAAUGAUCUCUUGUGAUUUAUUAUUUGAAGGAUGCAGUGAUUGCCUUUGUCAGUCAAUGCUGAUUCUUCUACAGU
UGUGUUCAGGCUUCGGAUUGCUCAAUAUCUACACUGCGGUAAGCUUUGGAUGGCAGUAGAGCUUGAACUGUACUGUAACUUCUUUUUGCUGUUAUGUGUG
GUCCACAAAUCACAGUAAUGAGGACGAGCUAAUCAUCUUAUAGCUUCAUCCUACUUUCUUUGUUUUGCAAGGUCACAAGAUGCAUGGGAACAAAUGGGAG

UUCAGUUGCAUUUGACCUGCCCUGUUAUAUCUUUAUUAAGGGGGCAGCUUCUGAUGUAUGCAGGACUGCCCAGCCGAAGGCAUUUUCAGUACUGAAAUUU
CUAAAUUUCAGUGUCAUUUGUAACCAAACUCACUACAGAGGAAGCGACAUCUUGUCACUUCUGGAUUUCGUGAUGGAAGAUAAGAAUUCCUCUAGUUAAC
AUGCAGUGGAAGAUACGAACUCUGUUGGAACUCCUAAUUAACCCCUUCGUCAGCGAAAGCUAGUUGUAAUUGGCAUUUCAAGCAGGCGGCGGUUCUAGUU
CAGUAAAUUGUAAUUUUCGCUUUUGAAACUAUUUUAAAGAGUAGCAUGCGUCUUUUGAUAUGUAUUCCUUUCUAUGCUUCAUAUCAGCUGUACACCUGGU
AAAUUCACCUUUGUUUGAGAAGACAAGACCAUGUCUUUUGAUAUGUAUUCCUUUCUAUGCUUCAUAUCAGCUGUACACCUGG
>culm_34
CGGCUCGGUGUUGCCUUCUCCUACCCUUUGCAUAGCACAAGCGCCCCUCAGAAUCACAAUUCAGAAUGGCCUUGCUUCCCUCCCCCGUCUCUCGAGCCGC
CGCGGCGCGCCGUAGUCUCAGAUUGAUGCAAGGGGUGGUGACAAGCUAGAGGCAGCAACUGCAUAUCUUGCAAGAAAAUUGGUUUUCGGACCAUACCGAU
GAUUUUCUUGCAAGUUGUGCAGUUGUUGUCUCAAGCUUGCUGCCUCCCUUUGCCAAAACUAUCAAAAGUAAAAGCACGGCUUAGGGUAUUGUUGAACCCC
UAAGAAAAAAUAAAAUUAUAAACAUGUGCCAAUAACAAAUAUGAUGAAUUAUUAAUUGAGAUCCUGUGGCAGGAAGUAAUAUUUUGCAGCAUACUUAACA
CAAUUGCUAUCCAGCACAAAACUAAGAUGGAAAUGACAUUUAUUGUUAAGAAAACACAAGCUCGCUUGUCCAUUUUUGCAAUUUGGAGGCUAUCCUUAUG
GUCUUGUUUAGUUUUGCAUUAACAAGCAGUAUUUUAAGGUGGUGAUUGGUGACCUAGCAAACAAUAUAUUUCAGCAGACACACUGCCAUUACGCAGUGGC
AAGCAAACUCCAUGAAGUCGUAACAAGGUGAGUGAAAAUUCUGAUAAAAAGAACAUGUCAUCAGUAUAAGUAAAUGAUCUCUUGUGAUUUAUUAUUUGAA
GGAUGCAGUGAUUGCCUUUGUCAGUCAAUGCUGAUUCUUCUACAGUUGUGUUCAGGCUUCGGAUUGCUCAAUAUCUACACUGCGGUCACAAGAUGCAUGG
GAACAAAUGGGAGUUCAGUUGCAUUUGACCUGCCCUGUUAUAUCUUUAUUAAGGGGGCAGCUUCUGAUGUAUGCAGGACUGCCCAGCCGAAGGCAUUUUC
AGUACUGAAAUUUCUAAAUUUCAGUGUCAUUUGUAACCAAACUCACUACAGAGGAAGCGACAUCUUGUCACUUCUGGAUUUCGUGAUGGAAGAUAAGAAU
UCCUCUAGUUAACAUGCAGUGGAAGAUACGAACUCUGUUGGAACUCCUAAUUAACCCCUUCGUCAGCGAAAGCUAGUUGUAAUUGGCAUUUCAAGCAGGC
GGCGGUUCUAGUUCAGUAAAUUGUAAUUUUCGCUUUUGAAACUAUUUUAAAGAGUAGCAUGCGUCUUUUGAUAUGUAUUCCUUUCUAUGCUUCAUAUCAG
CUGUACACCUGGUAAAUUCACCUUUGUUUGAGAAGACAAGACCAUGUCUUUUGAUUGUAAACGCUUGUAGAUUGUAAGAACCUGAAGUUGUUGUCGUUGU
CAGAAUGCUAUACUAGUUCUGCAAACAGAGCUAUGUCUAACUCUGCAAUGCAUCAUCAUAGUAAUA
>root_66
CCUACAAGAGCACCCACCCCGCGGCAGCACCUGCUCGCAUCGAGCCGGGAGGUUUCGAAAUCCAGCUCAGAUUUGGCCUCCUUUCUCGGUCCUUCUCCCG
CCUUCUCUCUCCGCGCUGGUGAGCCCCCGCGGGCGACGUGUGUCCCGUCCGUUGGGGUUGGCUUGCCUUCGACGGCCAUCUGUUGCCUCGCAUGCCUACC
UCCUGCCAAGCGUCCUCGCUCGGAGCCGGCCGAGCGCCGCGGCGUCCAGCCAGAUCCGCGUCGCGUCGCCUCCUCGACUUCUCUCUCUACCUAGUUUAUA
AUCCACCAUCCCGGCUCGGUGUUGCCUUCUCCUACCCUUUGCAUAGCACAAGCGCCCCUCAGAAUCACAAUUCAGAAUGGCCUUGCUUCCCUCCCCCGUC
UCUCGAGCCGCCGCGGCGCGCCGUAGUCUCAGAUUGAUGCAAGGGGUGGUGACAAGCUAGAGGCAGCAACUGCAUAUCUUGCAAGAAAAUUGGUUUUCGG
ACCAUACCGAUGAUUUUCUUGCAAGUUGUGCAGUUGUUGUCUCAAGCUUGCUGCCUCCCUUUGCCAAAACUAUCAAAAGUAAAAGCACGGCUUAGGGUAU
UGUUGAACCCCUAAGAAAAAAUAAAAUUAUAAACAUGUGCCAAUAACAAAUAUGAUGAAUUAUUAAUUGAGAUCCUGUGGCAGGAAGUAAUAUUUUGCAG
CAUACUUAACACAAUUGCUAUCCAGCACAAAACUAAGAUGGAAAUGACAUUUAUUGUUAAGAAAACACAAGCUCGCUUGUCCAUUUUUGCAAUUUGGAGG
CUAUCCUUAUGGUCUUGUUUAGUUUUGCAUUAACAAGCAGUAUUUUAAGGUGGUGAUUGGUGACCUAGCAAACAAUAUAUUUCAGCAGACACACUGCCAU
UACGCAGUGGCAAGCAAACUCCAUGAAGUCGUAACAAGGUGAGUGAAAAUUCUGAUAAAAAGAACAUGUCAUCAGUAUAAGUAAAUGAUCUCUUGUGAUU
UAUUAUUUGAAGGAUGC
>Bud_70
ACUGCUCUGAAUUAAGAGGCGUUGAAAUAUUGUCCGGUCUACAUAUAGGCACUUGAGGUGUAUUGACAGACAGUUCCCCUUAGUUUGCUACAACACAUCA
GGCGAAUUCCACCAAAUUAGGAUGAUGUUUGUUUCUUAGACACAGGAUAGCAAUGCCAAAAGUCUUUUAUAAUUUUCCGCUUCACCGCAGAUUUGGCAUC
UCAAAGGGAGUUGUGGCAAGAUUCUCUUGACCAAGCUUUCUUGCAGAUUUGGCAAGAAAGAUAAAAAAAAUGUUGGUAUACUUAAGCAUCCUUAAUAGAA
ACUAAACAGGUCUUAGAUUGAAAUUAUCAUGAAUAAACCCUUUUUCACUUUAGCUGUAGUCCGGAUGAAAUCUUAGGGGCCUUGCUUAGCCUGCCCUGAA
GCUUGCCAGUAAGUCGAAGCUGAUGCUGACACAGCACUGACAUAGAUCCCAAUGAACACAAGUUAUUUCGAGAAGAUAAAAUACCAAGCAAAGCCAGGAU
UGCAUUAAAAGAUAGUGUAUUUCUACUAUACGAUCUGAAAGCUUAAUUUAUUAUAUCAAUAAUCAAAUUGGCAGCAGUGUGGAACAGAUAUAAAGAAGAU
GCUUCAAAUCACAAAUAACUACAGAUAAAUUGAAGGGUGAAGGUUCAUUCAACCACCAAACACCCAAAAUGUCGAGCUGCAUAUGCACAGAACCUAAGUA
CGGUACAGUUCAUAGUUUGGGAAAAUAGACUGCAAAUGUGCACAGAUAAUACAGAUUGUUACUCGAUGAACAGUAAGCACGGUAUUUGAAAUCCUUCUAA
CCAUCCAUCCAGGCAGUGCCUCGUAUACUCAAACCUCUUUUUGGAGGAAGCCAAACCAUGUGAUCCUUUUCAACCCAAAUACAUAUAUUCAAGAUGAAAC
UAACAAAAAGACAGUUGAUAAAGAAACACGAAAGAGAGUAGAAAAAGGGUACCUGUUUCAAGUUCUCAUCUUCUGAAAAUGAUCAGCAGUCAAUAGAUGU
UUUAGUCCAUUCUCCAGAAAAAUGAGUGCCGCAUGAAUCCUUUCCGGUUUAUAGUGAAACAUUUUAGACGAUGACCUUUAACAUUCUAGAAAACGAAGAG
AAAACCUACAGUCAUCUAAUUUCAGCUUAAACAUGUUCCAGCAAGAUCAUCACCAUCCUGACAUGUUCCGUUUUGUCCCUAGACCCGAGCACAAGGUAUU
GCGAGGCUAAUUUAGCUCAAUUCAGUUGUCAGAGCAAUUGUGUGUGAAAAGUGUGAACAACAGCAUAGGAGACAAUACAGCAAGUGUAGCGUGCCAAUAU
GUGUCCAGCUUGAUAGUUCACUAUAAUAAAGUUCUUUAAUUCAAGUAUUUGAAUAAUAUAGAAUCUCAACCUUCAAGUUUAAAAGGUUUUGUAUUUGCUA
UUAUAAUCGUUGCCACCCCCUCACCCCUCUCAAAAAGAGAGUUACUUUGUAGCGCACUGUAAAUCUCUUGAUUGAAAUAGUGGCAUGUCAUCUUAUUUAA
UUCAGUUAUUUCAUAAGGUACAAUUUGUUUAGCUGACAGCUCAGACAUGCUUCUAUGCAACAUGUUUAUACCAAUGCUGUAUUUUGGCAAUCCUGGAUGU
GGCAUUACUUAACUGUGCUUUCAUAUUCAAUAGUUCUGGCAAAGGGAGGCUGCAAGCUUGAGACAACAACUGCACAACCUGCAAGAAAGUCAUCGGUAUG
GCUAGAAAACCGAUUUUCUUGCAAGUUAUGCAGUUGUUGUCUCUAGCUUGCCGCCUCCACUUGCAGUAACUGAGACGCCGGCGCCGAGCUUGAGCUUGAA
ACCUAGACUACGGGGACAGGGUAGACAGGAGACGCGAAACCCCAAACCAGCGCAAAACGACGCGCAGGGAGGGAGUGUCACUGAAGGAGCGACAGGGAAA
GCAGCGGCACGCCGGGGGAAAUUCCGAUCUGAGAGAACGGAUCAGCCAUGCAUGACAGGAGCUGCGGAGGCAGCAGGGGGGGGGGCUCCCGCUGCCACG
GGACAGUAGGGCACGCCAGAUCGGUGAAAUCCAGGCGAAGGACGAGGCGUGGGCGAGAUUUACACGACGGCGCGAUAGGAUCUAGCCCGGAUUUGGAUUC
GACCGACGAGGGCGGAAGGAACGGCAGGGUGGUGCUCUUGUAGAGAGAGAAGGAGUCGGAGGGGGCGGAGAGAGAGAAAGGGGGCAGGUGGGGUUUCAGG
UAGUAAAAUAUGGAGCGG
>root_04
CGCAUAUGGCUGCGCUAGAACUACUGCUCUGAAUUAAGAGGCGUUGAAAUAUUGUCCGGUCUACAUAUAGGCACUUGAGGUGUAUUGACAGACAGUUCCC
CUUAGUUUGCUACAACACAUCAGGCGAAUUCCACCAAAUUAGGAUGAUGUUUGUUUCUUAGACACAGGAUAGCAAUGCCAAAAGUUUUUUAUAAUUUUCC
GCUUCACCGCAGAUUUGGCAUCUCAAAGGGAGUUGUGGCAAGAUUCUCUUGGGCAAGCUUUCUUGCAGCUGUAACUUUCUUCUUCGCCACAGAUUUGGCA
AGAAUGAUAAAAUUUUGGUAUACUUAAGCAUCCUUAAUAGAAACUAAACAUAUCUUUGAUUGAAAUUCUCAUGAAAUAAACUUAUUUAAGACUCGAGGCU
GAGAUGAACCCUGCUUAGCCUGCCCUGAAGCUUGUUAGAAAGUCGAAGCUGAUGUUGACACAGCACCUGGCCUAGAUCCAAAUGAACUUAAGUUAUUUAG
AUAAGAUAAAAAUACCAAGCAAAGCCAGGAUUGCAGUAAUUGGUAACAUAGUGCAUUUCCAAGAUCCGAAACCUCAAUUUCUAUCAAUAAUUCAAGUGUC
GCACAGCUACAAAGAUGCUCCUAAUUAUGAAGAGAAAAAAAAACAGAUGAAUUAAAGGCUGAAGGUUCAUUCAACCACCAAGCACCCAAAAUGUCGAUCU
GCAUAUGCACAGAACCUCAGUACAGUUCACUGGUUGAGAAAAUAGACUGCAAAUGUGCAAAUAAUACAAAUUGUUACUGGAAGAACAGUAAACACAAUAC
CAGAAAUCCUUCUAACGGUAACCAUUGGAGGAAGCCUUGUGAUCCUGGUCCAAGUUCCCAUCCUCUGAAAAAGAUCAGCAGUCAAUAGAUGCUUCACUCA
UCCUCCAGAAAAAUGAGUGCCGCACGAAUCGUUUCCGGAUUAUAGUGAACCAUUUAAGAUGACGUCCUUUAACAAAUAUAAAAAGGAACCUACAAUGCAC
UAGUUUCAGAUUAAACAUGUUACAGCAAGAUCGUCGAUAUCCAGAAACGUUCCCUUUUUUCCUAGACCCUAGCACAAGAUAUUGCGAGGCUAAUUUAGCU
CAAUUCAGACGCCGGAGCAAUUGUGUGUGAGAAAAGUGUGGAUAACAACAUAGGACACAAUACAGCAAGUGUAGCGUGCCAUGUGUCCAGCUUGAUAGUU
CACUAUAAUAAAGCUCUUUAAUUCAAGUAUAUGAAUGAAAUCGAAUCUCAACCUUCAAUUCAAGAAAGGUUUUGUACGUAACCUUAUUUUGCAAAGAAGA
AUUACUUUGUAGUGUACUGUGUAAAUCUCUUGAUUGAAAUAGUGGCAUGUCAUCUCACUCAAUUCAGUUACUUCAUAAGAAUGUACCUAAAAUUUGUUUA
GCUGAUAAGCCAUGCUUCCAUGCAACAUCUUUAUACGAAUGCUGCCUUUUGGAAAUCCUGGAUGUGCCAUUACUUAACUGUGCUUUCACAUUCUAUAGUU
CUGGCAAAGGGAGGCAGCAAGCUUGAGACAACAACUGCACAACUUGCAAGAAAGUCAUCGGUAUGGCUCGAAACCGAUUUUCUUGCAAGUUAUGCAGUUG
CUGUCUCUAGCUUGCCGCCUCCACUUGCAGUAACAGUGGUCAUCAGCAAUAUAGGGAAAAGCCAAACUGAUGCGUACGCAGACCCUUUCGUCAAAGUUAG
UAAGGUGGAUUUUACUCUUGGGUGAGCAGAAUCAUAGGUCGGUCUUUUCCCGUGGCAUGCUCUGAGACGCCGGCGCCGAGCUUGAGCUUGAAACCCUAGA
CUAUGGGGACAGGGUAGACAAGAGUUGCCAAACCCCAAACCAACGCAAAACGACGCGCAGGGAGUGCCACUGAAAGGAGCGAAAGGAAAAGCAGCGGCAC
GCCGGGCAAAAUUCCGAUCUGAGAGAACGGAUCAGCCAUGCAUGACAUGAGCUGCGGAGGCAGCAAGGGGGGCGGCUCCCGCUGCCACGGGACAGUAGGG

```
CAUGCCAGAUCGGUGAAAUCCAGGCGAAGGGCGAGGCGUGGGCGAGAUUUACAUGACGGCGCACGGCGGCGCGAGAGGAUCUAGCCCGGAUUUGGAUUCG
ACGAGGGCGGAAGGGACGGCGGGGGUGGUUCUCUUGUAGAGAGAGAAGGAGUCGGAGGGG
>culm_17
AAUUAAAGGCUGAAGGUUCAUUCAACCACCAAGCACCCAAAAUGUCGAUCUGCAUAUGCACAGAACCUCAGUACAGUUCACUGGUUGAGAAAAUAGACUG
CAAAUGUGCAAAUAAUACAAAUUGUUACUGGAAGAACAGUAAACACAAUACCAGAAAUCCUUCUAACGGUAACCAUUGGAGGAAGCCUUGUGAUCCUGGU
CCAAGUUCCCAUCCUCUGAAAAAGAUCAGCAGUCAAUAGAUGCUUCACUCGUCCUCCAGAAAAAUGAGUGCCGCAUGAAUCCUUUCCGGAUUAUAGUGAA
CCAUUUAAGAUGACGUCCUUUAACAAAUAUAAAAAGGAACCUACAAUGCACUAAUUUCAGAUUAAACAUGUUACAGCAAGAUCGUCGAUAUCCAGAAACG
UUCCCUUUUUUCCUAGACCCUAGCACAAGAUAUUGCGAGGCUAAUUUAGCUCAAUUCAGACACCGGAGCAAUUGUGUGUGAGAAAAGUGUGGAUAACAAC
AUAGGACACAAUACAGCAAGUGUAGCGUGCCAUGUGUCCAGCUUGAUAGUUCACUAUAAUAAAGCUCUUUAAUUCAAGUAUAUGAAUGAAAUCGAAUCUC
AACCUUCAAUUCAAGAAAGGUUUUGUACGUAACCUUAUUUUGCAAAGAAGAAUUACUUUGUAGUGUACUGUGUAAUCUCUUGAUUGAAAUAGUGGCAUGU
CAUCUCACUCAAUUCAGUUACUUCAUAAGAAUGUACCUAAAAUUUGUUUAGCUGAUAAGCCAUGCUUCCAUGCAACAUCUUUAUACGAAUGCUGCCUUUU
GACAAUCCUGGAUGUGCCAUUACUUAACUGUGCUUUCACAUUCUAUAGUUCUGGCAAAGGGAGGCAGCAAGCUUGAGACAACAACUGCACAACUUGCAAG
AAAGUCAUCGGUAUGGCUCGAAACCGAUUUUCUUGCAAGUUAUGCAGUUGCUGUCUCUAGCUUGCCGCCUCCACUUGCAGUAACUGAGACGCCGGCGCCG
AGCUUGAGCUUGAAACCCUAGACUAUGGGGACAGGGUAGACAAGAGUUGCCAAACCCCAAACCAGCGCAAAACGACGCGCAGGGAGUGCCACUGAAAGGA
GCGAAAGGAAAAGCAGCGGCACGCCGGGCAAAAUUCCGAUCUGAGAGAACGGAUCAGCCAUGCAUGACUUGAGCUGCGGAGGCAGCAAGGGGGGCGGCUC
CCGCUGCCACGGGACAGUAGGGCAUGCCAGAUCGGUGAAAUCCAGGCGAAGGGCGAGGCGUGGGCGAGAUUUACAUGACGGCGCACGGCGGCGCGAGAGG
AUCUAGCCCGGAUUUGGAUUCGACGAGGGCGGAAGGGACGGCGGGGGUGGUUCUCUUGUAGAGAGAGAAG
>Bud_13
AGCUCUGGCAAAGGGAGGCAGCAAGCUUGAGGCAACAACUGCACAACUUGCAAGAAAGCCACAAGUAUGAUCUUCCAAUUGAUUUUGUGACUUCCUUGCA
AGUAAUGCAGUUCCUGCCUCAUGCUUGGUGCCUCCGCAUGCGAUAAUCCAAUAGCUAGUCAAGCAAAAGGACGACAAAUGUCAGGACACUCAGCAGUAAA
UGUUACCUGUUUAAGAAAUGUAUACUGACAAUAAAACAAAAAUAUAGCACACCAUCAGUGCUACUAGUAAUAACCAAACGUCGCUACGCUCGUAUAAAUU
GCCAAAAUCACCUCACCACAAAUCAAGGUUUUCACAGGUUUAAAUCGGCAGUACUAAAUUAGCUAAUGUAUAAUCGGCGUUUGAGGCAGUUAGCUACUUA
UAUCGUCAGAUGACCGAAGUUAAGUUUAAAUCGGACAGUGGACUGCAGGAGCAACUAGCUUUCCAAGAGGAAAUUGAUAACUAAAGGAACACUGAGGCAU
GUCAGAAACCACAUUUAAUUUGAAUUCCAGACAUAUAUAAAAAAAAUUAGCAUCCCGGUUGAUAACUAAAGGAAUCUGGUCAAUCUGCUGCAAAUGUCCU
UACAAGAACCACAGUGACGAUCAUCCCUCUAUCACCCGUGCACCACGCGGCGAUCACCGCCCAACAAGCACAUCAGAAACGAAAUCAGAGCGAAAAAUGG
CAUCGAAAUCUAUCGAGAAGACUGUACCAAAAAAAAAGAGGAGCAGCAGCCUCUAGAAACU
>Bud_62
AAGCCGCUCAUUGGCUGGAGAGGGUGGAAGUCACGGAGGAUUCGAGGUAUGGGGGAGUUUCUAGAGGCUACUCUACAGCUUCAGCUUCUUCUUCUUCUUC
UUCGUUUUUUAGGAUCAUCUUGAUAGAUCUCGAUGCCAUUUUUCGGUCCGAUUCCGUUUGUGAUGUGCUUGUUGGGCGGUGAUCGCGGCGUGGUGCACGG
AUGAUAGAUCCGCCGGGGUUUGGUGUGUUGCCAUUGUUUUCAGCUGGGGACUUUGAGAGGGAUGAUCUUAGCCAUGAGUUGUUCGAAGGACAUUUGCAGA
AGAUUUACCAAGUUCCUUUAGUUGAUUAAUUGGGUGCAAACGGGGACGCCAAGUUGUCUAUCUGGAAUCCUGACUAAGUGACGAGUCUAAUUUCCGAUAU
GCUUGCUUGCAGUUUUUACUAUAAGGAAGCUAAGGUAACUGCUCCCCUAGUCCAUUUGUCCGAUUUCAACUUUAUUACUUUGGUCAUCUGAGGACGAUGA
UACACUAGCUUAUUUGGUACUUAGCCGAUUUGAAUCUGUGGAAACCAUGAUUUGUGGUAAGCCUGGUUUGGCAUUUUCGACCAGUUUAGCGUUUUAACUU
CUGUUUAUUACAUUUAGCAUUGAUGCUGUGUUGUUUGAUUGUUUCAUCGUCAGUAUGUACUUCUUAUAGCCCCUUUUCUAAGGGGGUGUUUGGAUCAUUU
GGCUAAUUUUUAGCCCCAUGUUUGGACCCCAAUUAGGGGGACUAAAUAUGGACUAAUUGUGAACUAAUUAUAGUUAGCUUUAGCUAAUUGAUGAUUAGAG
CUAAUUAACCCUGAUUAGCUUAUGAUUAGCUCAUGUGCUAAUGGGCUAAACAUGGGCUAAUCAUAGGCUAAUCAUAGGCUAAUUAGAGCUUAUGGGCUCU
AAUCACUAAUUGCAAUUAGUCUAUAAUUAGCCCAUGUUCAGUCCUCCUAUUUAACAUCCAAAUAUAGAGCUAAAAAAUUAGCUAAAGGAUCGAAACACC
CCCUAAAAAAAUAUACUUCUCAUAGCCGUAUACAAUUCUCAAACAGUUAACAUGGCUUGCUCAGUUUUCUGACAUCUGUUAUCCUCUUGCUUCACUAGUU
AUCGGUUUAUUGCAUGCGGCGGCACCAAGCAUGAGGCAGGAACCGCAUUACUUGCAAGGAAGUCACAAAAUCAAUUGGAAGAUCAUACUUGUGGCUUUCU
UGCAAGUUGUGCAGUUGUUGCCUCAAGCUUGCUGCCUCCCUUUGCCACAGCUAUCAGUAAAACAAGAAGUACUGUCAGUUUCUUAUCUACAGAAAAUAAU
CUAGAAAUGUGUGGCGUGAUGUACUAAUACAUUGCUAAAAAUCAACCAAUACUUAAUAUCUGCACAUUCUACCGGUUUGGUGCUAACAUACCUACUUAAU
UUACAUGCAGCUCUUUGAACCUAUUGUGCUCUGAUUUAGUUGUUGACCAGCCAUUUGACUAUGCAUGCUGUGAUUUAUGAUCCAUGUUAGCACAACUAAA
UUUAAUAACUGAAACUACAU
>culm_64
CUAAUUUUUAGCCCCAUGUUUGGACCCCAAUUAGGGGGACUAAAUAUGGACUAAUUGUGAACUAAUUAUAGUUAGCUUUAGCUAAUUGAUGAUUAGAGCU
AAUUAACCCUGAUUAGCUUAUGAUUAGCUCAUGUGCUAAUGGGCUAAACAUGGGCUAAUCAUAGGCUAAUCAUAGGCUAAUUAGAGCUUAUGGGCUCUAA
UCACUAAUUGCAAUUAGUCUAUAAUUAGCCCAUGUUCAGUCCUCCUAUUUAACAUCCAAAUAUAGAGCUAAAAAAUUAGCUAAAGGAUCGAAACACCCC
CUAAAAAAAUAUACUUCUCAUAGCCGUAUACAAUUCUCAAACAGUUAACAUGGCUUGCUCAGUUUUCUGACAUCUGUUAUCCUCUUGCUUCACUAGUUAU
CGGUUUAUUGCAUGCGGCGGCACCAAGCAUGAGGCAGGAACCGCAUUACUUGCAAGGAAGUCACAAAAUCAAUUGGAAGAUCAUACUUGUGGCUUUCUUG
CAAGUUGUGCAGUUGUUGCCUCAAGCUUGCUGCCUCCCUUUGCCAGAGCUAUCAGUAAAACAAGAAGUACUGUCAGUUUCUUAUCUACAGAAAAUAAUCU
AGAAAUGUGUGGCGUGAUGUACUAAUACAUUGCUAAAAAUCAACCAAUACUUAAUAUCUGCACAUUCUACCGGUUUGGUGCUAACAUACCUACUUAAUUU
ACAUGCAGCUCUUUGAACCUAUUGUGCUCUGAUUUAGUUGUUGACCAGCCAUUUGACUAUGCAUGCUGUGAUUUAUGAUCCAUGUUAGCACAACUAAAUU
UAAUAACUGAAACUACAUUUCCAUUUCCAUACAAUCGUAGCUUACAGGUAGCAUGAUAUUGUUACACUUACAGUCCCCAAUGUGAUUUAAGGCAAACUCA
UUUUGGUUUUUUUCUGUUUUCCAAACAAUACCUUUUCAAUUACAUUCUCCAUGUGCAUAUGUAACAGUGAUUUCUGUGGCUGUUGCUAUAUUUUCUUUUG
CAUUUGGUAUCCUGUUAGUGUAUGGUGUCUUCAUCUUCGGAGCAUUAUAUUGCCAAACAGAAAUAUUGAUCUAUGAAGCAUGACUAAGUCCAAGAGUAGU
GCCUUCAUGACCAUGUAGCUUGUAGACGAUGAUUUGGUGUUCCUUGAUAUUUACAAAAGAAAAGGAAAGAAUCCAUCCAUACACCACUUUAAGGUGUUCU
UUUUGCCUAUACCACCGACUUGUGUCAAUGACAUGUAGGACUAGACCCCACUCGUCAUUGACACAGCCUGAUGGUAUAAAUGAAAAUUGUACCUUGAUUG
GCGGUAUGGAUAAAAAUUGCUCAAAGAAAAACCUUUGGUAAACGGUACCCUCAUGUAAGGAUGUUUGGGAAGCUAGUCUAUCCAUUGCAUACCUAUUACA
GCUCUUCCAGUUAGACAUGGUUAGACAUGACAUCAGUAUCAUACUUACUAAUAUCAGUUACAUUUUUUAUGGUUCUAGAUAUUCCAGCUUUUAGUUUUAC
UGCUCUUUUUCUUGUGAUGGUGAAUAAUGUCAAUUUAUUUCUUUAGAAAUCUUUCCCUUGUGAUUUGAGGGAACAUUGCCAUCUCCUCUUAACUUGCCAG
UUGCCAGAGCAUUACUAUGCGAGCUCUAAAGUUCUGAAAUUAAGGUAGUAAACAGAUGCAGUAAUUAUUCAGUGCGUGUGAUCGUUGGUCUGUGCUAUGG
UUUCUAUUGCAGAGUAUAAGGGUGCAGUGAAACUGCUGGGUAAGUUUCUAAAACCAGUUCAUAGGCUUGUUCUUUCAAAAGUAAAACAACUGCUUGUACU
UGGCUUUUGAUUCAGUGCACUCUUUUUUUCAAUCUAACUUUAUCUUAUAAUAUGGCUUCUCAGGAUGAACUUUGGAUGACUUGGCAUGGUUUUGGCGUCG
UGAUGGUCGUUAUCAAGUGCUGUGUGUGGCUUCAUGAGAAAGUGUGCUGCACUGCUGCCUUGGAGAAUCUAAGAUGGUUUCCUAACUGCUGUACAUGCUG
ACUGAUUGGACGGUGUUCUCCUUUUACAUGGUACAUGGACACAUGGUCAAGCAGGAAGGCAUAAGCGUUCCAGAAUUAUUUCAAUUAUUAAGAUAAGCCG
GCAUUUUCCAUAGUCAAGCUUGUUGUGUUGAAGACAUUUUUCUUGUAUUGAAUUGCCACACACAGUAUGGACAGAAUUUACCUUGUCGCGGACGAAUUGU
UUCCACUUGUUCGAACAUAGUCUUUGUUGUAGUCUCAGAUCAAUGCUACUACUUCAGAACACCGUCUGAUAACUGUCAAGUCUACGGGGCUUACAGAUGA
AAAUGCCCAACUGCAAGUAAUCAGUCCAACAAGUCCAUUCCCACUUCACUUAGGUAGGGAUUUGGUCGCAUCCUCCCAGAUUAAUAGGGAAAUUAGUUCA
UCUUUUCUCCUUAUUCUAAAAGAGGAGUCGGCUCUGUUCCCUGCCUAUCCAAACAAGUGUUCUUAUUUGCCCAACAGUAUGUUAGUAAGGCUGCAAACUG
GUUGAUGAGGCGGAUAUCAGACAUAGAUGGAGAUACUACAUAUUCACGUACAUUGCGGGAAUAAUCAAUUUUUCAGUCUUAAGAAGGUUAGUAAAACAAA
UAAGCUGAAUCCGGAACAAGAAUUUUGGCAUACAAAUUAUGGAACCACCGUGGACUGGUUGGCUGGUUGGUGAAAACAUGGGAGAAUCUAAGGUUAUAUU
CUCGUGGCUAUGCAACGGAGUGUUAUGUGUCCUUUGGUCUGGGUGUGUGUUAGAAGGCGGUGGGCGGUGGCUAUGCAAGUGUGUGUUAUGGGGUGUGCUU
CGAAAUAUUAUGAAAACAUGAUUAUAUAAGGGAAAUUGUAACAAAAAGCACCAACAAAGCCAUAAGCACUUAUUGAAGUACUCUCUCCGUUUCUUCAUAA
UACUUGUGAUUUUAGC
>leaf_09
CGGCACCAAGCAUGAGGCAGGAACCGCAUUACUUGCAAGGAAGUCACAAAAUCAAUUGGAAGAUCAUACUUGUGGCUUUCUUGCAAGUUGUGCAGUUGUU
GCCUCAAGCUUGCUGCCUCCCUUUGCCAGAGCUAUCAGUAAAACAAGAAGUACUGUCAGUUUCUUAUCUACAGAAAAUAAUCUAGAAAUGUGUGGCGUGA
UGUACUAAUACAUUGCUAAAAAUCAACCAAUACUUAAUAUCUGCACAUUCUACCGGUUUGGUGCUAACAUACCUACUUAAUUUACAUGCAGCUCUUUGAA
```

```
CCUAUUGUGCUCUGAUUUAGUUGUUGACCAGCCAUUUGACUGUGCAUGCUGUGAUUUAUGAUCCAUGUUAGCACAACUAAAUUUAAUAACUGAAACUACA
UUUCCAUUUCCAUACAAUCGUAGCUUACAGGUAGCAUGAUAUUGUUACACUUACAGUCCCCAAUGUGAUUUAAGGCAAACUCAUUUUGGUUUUUUUCUGU
UUUCCAAACAAUACCUUUUCAAUUACAUUCUCCAUGUGCAUAUGUAACAGUGAUUUCUGUGGCUGUUGCUAUAUUUUCUUUUGCAUUUGGUAUCCUGUUA
GUGUAUGGUGUCUUCAUCUUCGGAGCAUUAUAUUGCCAAACAGAAAUAUUGAUCUAUGAAGCAUGACUAAGUCCAAGAGUAGUGCCUUCAUGACCAUGUA
GCUUGUAGACGAUGAUUUGGUGUUCCUUGAUAUUUACAAAAGAAAAGGAAAGAAUCCAUCCAUACACCACUUUAAGGUGUUCUUUUUGCCUAUACCACCG
ACUUGUGUCAAUGACAUGUAGGACUAGACCCCACUCGUCAUUGACACAGCCUGAUGGUAUAAAUGAAAAUUGUACCUUGAUUGGCGGUAUGGAUAAAAAU
UGCUCAAAGAAAAACCUUUGGUAAACGGUACCCUCAUGUAAGGAUGUUUGGGAAGCUAGUCUAUCCAUUGCAUACCUAUUACAGCUCUUCCAGUUAGACA
UGGUUAGACAUGACAUCAGUAUCAUACUUACUAAUAUCAGUUACAUUUUUUAUGGUUCUAGAUAUUCCAGCUUUUAGUUUUACUGCUCUUUUUCUUGUGA
UGGUGAAUAAUGUCAAUUUAUUUCUUUAGAAAUCUUUCCCUUGUGAUUUGAGGGAACAUUGCCAUCUCCUCUUAACUUGCCAGUUGCCAGAGCAUUACUA
UGCGAGCUCUAAAGUUCUGAAAUUAAGGUAGUAAACAGAUGCAGUAAUUAUUCAGUGCGUGUGAUCGUUGGUCUGUGCUAUGGUUUCUAUUGCAGAGUAU
AAGGGUGCAGUGAAACUGCUGGGUAAGUUUCUAAAACCAGUUCAUAGGCUUGUUCUUUCAAAAGUAAAACAACUGCUUGUACUUGGCUUUUGAUUCAGUG
CACUCUUUUUUUCAAUCUAACUUUAUCUUAUAAUAUGGCUUCUCAGGAUGAACUUUGGAUGACUUGGCAUGGUUUUGGCGUCGUGAUGGUCGUUAUCAAG
UGCUGUGUGUGGCUUCAUGAGAAAGUGUGCUGCACUGCUGCCUUGGAGAAUCUAAGAUGGUUUCCUAACUGCUGUACAUGCUGACUGAUUGGACGGUGUU
CUCCUUUUACAUGGUACAUGGACACAUGGUCAAGCAGGAAGGCAUAAGCGUUCCAGAAUUAUUUCAAUUAUUAAGAUAAGCCGGCAUUUUCCAUAGUCAA
GCUUGUUGUGUUGAAGACAUUUUUCUUGUAUUGAAUUGCCACACACAGUAUGGACAGAAUUUACCUUGUCGCGGACGAAUUGUUUCCACUUGUUCGAACA
UAGUCUUUGUUGUAGUCUCAGAUCAAUGCUACUACUUCAGAACACCGUCUGAUAACUGUCAAGUCUACGGGGCUUACAGAUGAAAAUGCCCAACUGCAAG
UAAUCAGUCCAACAAGUCCAUUCCCACUUCACUUAGGUAGGG
>culm_63
GCUCAACCUUAAUCACCCGCUAAGCCGUUCCCUCCUCUCUCUCCUUCGCCCAAAAGCCAAUCGCGUUGCAUCACCUCUCGCUUUUCCUAUCUCCACUUGG
AGCGGGGCUUCUAGACCCGUCGAUUCGAUCCUUCUAGACUCUUCGCCGCCCCUAAUAUAAAGCCGGCGCCACCCGCACACCAGCGCCCGCACGCAGCAAG
CCGCUCAUUGGCUGGAGAGGGUGGAAGUCACGGAGGAUUCGAGCUAUUGGAUUAUCGCAUGCGGAGGCACCAAGCAUGAGGCAGGAACUGCAUUACUUGC
AAGGAAGUCACAAAAUCAAUUGGAAGAUCAUACUUGUGGCUUUCUUGCAAGUUGUGCAGUUGUUGCCUCAAGCUUGCUGCCUCCCUUUGCCAGAGCUAUC
AGUAAAACAAGAAGUACUGUCAGUUUCUUAUCUACAGAAAGUAAUCUAGAAAAAUAAUCUAGAAAUGUGUGGCGUGAUGUACUAAUACAUUGCUAAAAAU
CAACCAAUACUUAAUAUCUGCACAUUCUACCGGUUUGGUGCUAACAUACCUACUUAAUUUACAUGCAGCUCUUUGAACCUAUUGUGCUCUGAUUUAGUUG
UUGACCAGCCAUUUGACUAUGCAUGCUGUGAUUUAUGAUCCAUGUUAGCACAACUAAAUUUAAUAACUGAAACUACAUUUCCAUUUCCAUACAAUCGUAG
CUUACAGGUAGCAUGAUAUUGUUACACUUACAGUCCCCAAUGUGAUUUAAGGCAAACUCAUUUUGGUUUUUUUCUGUUUUCCAAACAAUACCUUUUCAAU
UACAUUCUCCAUGUGCAUAUGUAACAGUGAUUUCUGUGGCUGUUGCUAUAUUUUCUUUUGCAUUUGGUAUCCUGUUAGUGUAUGGUGUCUUCAUCUUCGG
AGCAUUAUAUUGCCAAACAGAAAUAUUGAUCUAUGAAGCAUGACUAAGUCCAAGAGUAGUGCCUUCAUGACCAUGUAGCUUGUAGACGAUGAUUUGGUGU
UCCUUGAUAUUUACAAAAGAAAAGGAAAGAAUCCAUCCAUACACCACUUUAAGGUGUUCUUUUUGCCUAUACCACCGACUUGUGUCAAUGACAUGUAGGA
CUAGACCCCACUCGUCAUUGACACAGCCUGAUGGUAUAAAUGAAAAUUGUACCUUGAUUGGCGGUAUGGAUAAAAAUUGCUCAAAGAAAAACCUUUGGUA
AACGGUACCCUCAUGUAAGGAUGUUUGGGAAGCUAGUCUAUCCAUUGCAUACCUAUUACAGCUCUUCCAGUUAGACAUGGUUAGACAUGACAUCAGUAUC
AUACUUACUAAUAUCAGUUACAUUUUUUAUGGUUCUAGAUAUUCCAGCUUUUAGUUUUACUGCUCUUUUUCUUGUGAUGGUGAAUAAUGUCAAUUUAUUU
CUUUAGAAAUCUUUCCCUUGUGAUUUGAGGGAACAUUGCCAUCUCCUCUUAACUUGCCAGUUGCCAGAGCAUUACUAUGCGAGCUCUAAAGUUCUGAAAU
UAAGGUAGUAAACAGAUGCAGUAAUUAUUCAGUGCGUGUGAUCGUUGGUCUGUGCUAUGGUUUCUAUUGCAGAGUAUAAGGGUGCAGUGAAACUGCUGGG
AUGAACUUUGGAUGACUUGGCAUGGUUUUGGCGUCGUGAUGGUCGUUAUCAAGUGCUGUGUGUGGCUUCAUGAGAAAGUGUGCUGCACUGCUGCCUUGGA
GAAUCUAAGAUGGUUUCCUAACUGCUGUACAUGCUGACUGAUUGGACGGUGUUCUCCUUUUACAUGGUACAUGGACACAUGGUCAAGCAGGAAGGCAUAA
GCGUUCCAGAAUUAUUUCAAUUAUUAAGAUAAGCCGGCAUUUUCCAUAGUCAAGCUUGUUGUGUUGAAGACAUUUUUCUUGUAUUGAAUUGCCACACACA
GUAUGGACAGAAUUUACCUUGUCGCGGACGAAUUGUUUCCACUUGUUCGAACAUAGUCUUUGUUGUAGUCUCAGAUCAAUGCUACUACUUCAGAACACCG
UCUGAUAACUGUCAAGUCUACGGGGCUUACAGAUGAAAAUGCCCAACUGCAAGUAAUCAGUCCAACAAGUCCAUUCCCACUUCACUUAGGUAGGGAUUUG
GUCGCAUCCUCCCAGAUUAAUAGGGAAAUUAGUUCAUCUUUUCUCCUUAUUCUAAAAGAGGAGUCGGCUCUGUUCCCUGCCUAUCCAAACAAGUGUUCUU
AUUUGCCCAACAGUAUGUUAGUAAGGCUGCAAACUGGUUGAUGAGGCGGAUAUCAGACAUAGAUGGAGAUACUACAUAUUCACGUACAUUGCGGGAAUAA
UCAAUUUUUCAGUCUUAAGAAGGUUAGUAAAACAAAUAAGCUGAAUCCGGAACAAGAAUUUUGGCAUACAAAUUAUGGAACCACCGUGGACUGGUUGGCU
GGUUGGUGAAAACAUGGGAGAAUCUAAGGUUAUAUUCUCGUGGCUAUGCAACGGAGUGUUAUGUGUCCUUUGGUCUGGGUGUGUGUUAGAAGGCGGUGGG
CGGUGGCUAUGCAAGUGUGUGUUAUGGGGUGUGCUUCGAAAUAUUAUGAAAACAUGAUUAUAUAAGGGAAAUUGUAACAAAAAGCACCAACAAAGCCAUA
AGCACUUAUUGAAGUACUCUCUCCGUUUCUUCAUAAUACUUGUGAUUUUAGC
>leaf_45
CUGCUAUGGCAACUGGCAAGUUUAAAGGAGAUGGCAAAGUGGCCCUUCAAAUCAUACAGUGAUGGAUUUGUAACGAAUUCAACUGACAAGCUCUGGCAAA
GGGAGGCAGCAAGCUUGAGGCAACAACUGCACAACUUGCAAGAAAGCCACAAGUAUGAUCUUCCAAUUGAUUUUGUGACUUCCUUGCAAGUAAUGCAGUU
CCUGCCUCAUGCUUGGUGCCUCCGCAUGCGAUAAUCCAAUAGCUCGAAUCCUCCGUGACUUCCACCCUCUCCAGCCAAUGAGCGGCUUGCUGCGUGCGGG
CGCUGGUGUGCGGGUGGCGCCGGCUUUAUAUUAGGGGCGGCGAAGAGUCUAGAAGGAUCGAAUCGACGGGUCUAGAAGCCCAGCUCCAAGUGGAGAUAGG
GAAAGCGAGGAAGCGAUGCAACGCGAUUGGCUUUUGGGCGAAGGAGAGAGAGGAGGGAACGGCUUAGCGGGUGAUUAAGGUUGAGCGGAUUUAAUUAGUG
GAUUGUUAAUUGCGGCUUGCGG
>root_23
UGAUUUGUGGUAAGCCUGGUUUGGCAUUUUCGACCAGUUUAGCGUUUUAACUUCUGUUUAUUACAUUUAGCAUUGAUGCUGUGUUGUUUGAUUGUUUCAU
CGUCAGUAUGUACUUCUUAUAGCCCCUUUUCUAAGGGGGUGUUUGGAUCAUUUGGCUAAUUUUUAGCCCCAUGUUUGGACCCCAAUUAGGGGGACUAAAU
AUGGACUAAUUGUGAACUAAUUAUAGUUAGCUUUAGCUAAUUGAUGAUUAGAGCUAAUUAACCCUGAUUAGCUUAUGAUUAGCUCAUGUGCUAAUGGGCU
AAACAUGGGCUAAUCAUAGGCUAAUUAGAGCUUAUGGGCUCUAAUCACUAAUUGCAAUUAGUCUAUAAUUAGCCCAUGUUCAGUCCUCCUAUUUAACAUC
CAAAUAUAGAGCUAAAAAAUUAGCUAAAGGGAUCGAAACACCCCCUAAAAAAAAUAUACUUCUCAUAGCCGUAUACAAUUCUCAAACAGUUAACAUGGCUU
GCUCAGUUUUCUGACAUCUGUUAUCCUCUUGCUUCACUAGUUAUCGGUUUAUUGCAUGCGGCGGCACCAAGCAUGAGGCAGGAACCGCAUUACUUGCAAG
GAAGUCACAAAAUCAAUUGGAAGAUCAUACUUGUGGCUUUCUUGCAAGUUGUGCAGUUGUUGCCUCAAGCUUGCUGCCUCCCUUUGCCAGAGCUGUCAGU
AAAACAAGAAACAUUGUCAGUUUUUGAGCUACAAAAAGUAAUUUUAGAAAAAGAAUGUAGAAAUGUAAGGCGUAGUAUACUAAUACAUUGCUUAAAGUUA
AUCUAUGCCCAAUAACUGCAACAUCUUCAGCUUUGGGGAUAACGUUAACUAUUUUAAAAACAGAAACUACAUAUACAUGUCCAUACAAUCUUAGCUAACU
UCAGGGGGUUGUGGUCAAUCAAUAAGUUGGCACAAUAUCUGUAGUGUCAUAUUCUAACAUUUACAGUCCCCAAUGUGAUUUCAAGGAAAAUUCAGUUUGG
UUUUCCUGUUUUCAGAUAAUGCCUGUUCAGUUGCAUUAUCAAUGUGCAUGUGUGAUAUUACAUUUUGAUCCUGUUGCGAUAUAGUCCUUUACAUUGGGAU
CCUGUUUCAUCAUCUUCAUUUCUUCAAAGCAUAAUAUUGCCCAAUAAAAUGAUGUUCCUCCGGCAUGACGCCUUAUAGGUAAUGAUUUGGCCUUGCGUCA
UAUUUACAAAAAGAAAAAUCUUCGGUAAACAGUAUCCACGUGGAAGGACAUUUUAAGGAGCUAGUCUAUCCAUAGCAUACAUACUCCAGUUAGACAUAAC
AUCAGUAUCAUACUUACUAAUAUCAGUUACAUUUUUAUGGUUCUGGCUCUUUCUAGCUUGCUAAUAUCAGUUACAUUUUUUUGGUUCUGGCUCUUUCCAG
CUUUUAAUAUUUUACUGCCCUUUUUUCUUGCGCAAGUGAACAGUGUCAGUUGAAUUCGUUACAAAUCCAUCACUGUAUGAUUUGAAGGGCCACUUUGCCA
UCUCCUUUAAACUUGCCAGUUGCCAUAGCAGAGUAAAAGGGUGCAGUCUGAUCUUGCAGAAAAACUGCCGUGAUGACC
>root_33
AACAACAAAGCAUGAGGAGUGCAUAAGAUAAUAUACUAACAAGGAAUAUUUAUUCAAUGUGACCACAAAAUCAAUUGUUUCUUUUAAUUUAAUCUUGUAC
AUAAAUAAGUGACAUUUUGGACACGGGGACAAGUUUGUGCAAUACAACUUCGACCAUCAAUCUCUACCAUGUAGUCAAGAUAGUUUGACUAUCCCGUUAC
UGCUCUAUAUACAGAGUCAACAGUUGGGCCGCCGAUAUUACAAUCUUAUUUUCAGGGCCUUCUGCUCAUGUAUGACACACAAAUCAGAAGAACAUUUUUU
UACUAGUGCAAAACACAGCAACAACGAUACUUCAUUUUUACGGCCAUGGACAACAACUUAACGGUACGUGAGCAGCUAAGAAUGGAUUAGCUUGCAAGCA
AAUUAACAACUCGAUAAGAUCGUACAUCUAGCAUUGUUGCUUUUCUCUUUGAUCUCUCAUGCUAAACCUAAAAUACAUCGGCUAUGGGAGCUCUCUAGUC
ACUUUUUUCAUCGUAUUUCCUGGUUUGGCAAUGGGAGGCAGUAAGCUUGAGGCAGCAACUGCAUAACUUGCGGCAAAAUCAUCGGCAUGACCAUCAUCCA
ACAAGUUUCUCGCAAGUUAUGCAGUUGCCGCCUCUAGCUUACUGCCCCCAAAUGCAAAACCUGGAUAUACAGAUGAGAUCUUUAAUCCCAAAAGUCACAG
CGACUAAGCUUGCUCUCCACAGCACACCUUGACCCCCAUCACUUCUAACCCUCGAAGGGGGAACAAGAACACAGAGCUGUGGAGAGCAAGC
```

>culm_73
UCAUCAAGGCUAAAGGGAGAAGAGAGAGAGUACAGCCUUGUUACCAUAUAUACACCUCCGAUGUCAUCAGAUAUAUGUGAGAUGUGUUGAAGGCUGUACC
CUCUCUCUUCUUCUCUUAGCUUACUGAUGCAAAGUUGCAAACACACUUGGAGAGCUCUUGCAGAAGAAAGUUGCGGACCUCGAUCACUGAUCAGGAUCCG
GUCGAACACUGACAUUUUCGUUAGUUUCUUUUUCUUUUAUUUCAAAGCACGCUCAUAACACGC
>Bud_22
AAUUAUUGGGAACUCUUGGAGAUGCUCUUAGACCAAUUCUCUUUCAGUUCUUCACCAGAAUUACAAGCUGAACCGCACAUCAUCGCAGCAGGUUAACUAA
CUGCAUCUGCAUCACUGUAUGAGCAUAGCAAACCCAAGGCCGAAUGCAUCGCAAGAUGAAAAAACUGGAAACGUUUUUUCAUUAUUUUAAAAUAUAGCA
UAGCAAACCCGGUCUGUAAGACCGUACUUAACAAAUCCAGUUAAAAUCGAACACGCGGUCUUUGAUAUAAGCAUGAAGCGCAUUUCUGAAAUGCUAAUAA
GCUCGUCCCCAAAUCCGUAGGAGAAUCAAAUUACUCCGCAAUAAUUUGGGCAAAUGAGAGUCACGCGAUAUUUGUCCCUACCCUGAAAAGAGUCGAGAGA
UAUUUCCAGUUUCAGCUUCAGCUGCAUGGCGACAGGAAGAACAAUUUCAUUGAUUGGAGGGGGUUGCAAGUGAUAUAAGCGCUACUCGCAUCUCCAGGAA
ACAAACUGCAUCUGCACACAAGAAACACAAAACUGAGAAGCAAAACUGACGACAGGAGCACGCAAAGAGUACGAUCACCUUUGACUUCUCCAAGGAAUGU
AACGUUGAGGAGAAAGAGACACGCAUGUGUUACGCUUCAACGGAGUCAGAACUCGGAAGCAUAAACAACCAUGAAUCCAUGAUCAGGGAAGCCAAAAACC
AUCCUUCUGCCUGCAUGCCUCACGAACAUGUUUGCUGAUGGUCAUCUAACGAUUCAUGCAUGCGUGAUCAUGAUAAUCCUGCGAGCAUAGUUAAAUGACC
ACCAACAAAACAAAUUCAGAGCUGCAUUAACCGGAAGGGUUGAGUUGGAAGGAACCUGCUUGAUUAGCUAAAGGGGGCCUAGAACAGCAGGGAC
>root_59
GGACGAGAUAGGCGCAGAGUGCGACGCUUUCUCGCUGCUCAUCUCGUCGCUGCUGUUCUAGGCCCCCUUUAGCUAAUCAAGCAGGUUCCUUCCAACUCAA
CCCUUCCGGUUAAUGCAGCUCUGAAUUUGUUUUGUUGGUGGUCAUUUAACUAUGCUCGCAGGAUUAUCAUGAUCACGCAUGCAUGAAUCGUUAGAUGACC
AUCAGCAAACAUGUUCGUGAGGCAUGCAGGCAGAAGGAUGGUUUUUGGCUUCCCUGAUCAUGGAUUCAUGGUUGUUUAUGCUUCCGAGUUCUGACUCCGU
UGAAGCGUAACACAUGCGUGUCUCUUUCUCCUCAACGUUACAUUCCUUGGAGAAGUCAAAGGUGAUCGUACUCUUUGCGUGCUCCUGUCGUCAGUUUUGC
UUCUCAGUUUUGUGUUUCUUGUGUGCAGAUGCAGUUUGUUUCCUGGAGAUGCGAGUAGCGCUUAUAUCACUUGCAACCCCCUCCAAUCAAUGAAAUUGUU
CUUCCUGUCGCCAUGCAGCUGAAGCUGAAACUGGAAAUAUCUCUCGACUCUUUUCAGGGUAGGGACAAAUAUCGCGUGACUCUCAUUUGCCCAAAUUAUU
GCGGAGUAAUUUGAUUCUCCUACGGAUUUGGGGACGAGCUUAUUAGCAUUUCAGAAAUGCGCUUCAUGCUUAUAUCAAAGACCGCGUGUUCGAUUUUAAC
UGGAUUUGUUAAGUACGGUCUUACAGACCGGGUUUGCUAUGCUAUAUUUUAAAAAUAAUGAAAAAACGUUUCCAGUUUUUCAUCUUGCGAUGCAUUCGG
CCUUGGGUUUGCUAUGCUCAUACAGUGAUGCAGAUGCAGUUAGUUAACCUGCUGCGAUGAUGUGCGGUUCAGCUUGUAAUUCUGGUGAAGAACUGAAAGA
GAAUUGGUCUAAGAGCAUCUCCAAGAGUUCCCAAUAAUUUCAGUCCCAAAAACCUAAGAUUGGGACCUUCCCAAAAUAUUUGGGCAGAAAAAACACCCU
AUUCUCCAACAGUAGCCUUAUUUUUUGUUCCCACAAAAUUGAAUUGUGCCACAUCAUCCCAGGUGGGACCAUACGGACCGGAUCAAACUGAUCUUUGUUA
CCAAUCAAGUCUGACUUCCGAUCAAGUCUUUUUUGUUCGGUCAAAGCUGCUCCUGGAGAUAGUAUAUAGAAAAACAACUAUCAUCCCAUCAUAACCUCAG
UUAAAGCGAAAUCUUUCUCAUCUCUUCCAAGCAAAUUAUACGAAAUAUAACUCAUGUAGGAAUCCCAAGAUAAUCUAUUGAUGUGACUUCAUAUACAUGG
AGAGUGGGGCAAGUAGGCAUGGGCAUGGGCAUACAACAAAAUGAAUCGAUGAACAAUCAUUACCAGACCAGACGACGAGCAGUGAGACCAGCGGCAGCUU
GGACUCCAGUCGCGUGUGCGGCUUGGACUCUAGGCGCGUAUGCAGCAGCUUGGACUCCGAGCGCGGCGGCAACGGCUUAGGCUCCAGGGGCUACUACGGC
UUCAACUCGACGGCAGCGGCAUCCACACGU

Predicted *A. donax* precursor miRNAs

>Ado-MIR1430-1_c
UAGCCAAGAAUGGCUUGCCUAUCUCCACUAUUUGGUUCAUAAGGGAUUCGGAGUCCUGAUGAUGGAUGAAAUGUGGAUGAUGGCGAGCCUUCUUCGGCUA
AG
>Ado-MIR1430-1_l
UAGCCAAGAAUGGCUUGCCUAUCUCCACUAUUUGGUUCAUAAGGGAUUCGGAGUCCUGAUGAUGGAUGAAAUGUGGAUGAUGGCGAGCCUUCUUCGGCUA
AG
>Ado-MIR1430-2_c
UAGCCAAGAAUGGCUUGCCUAUCUCCACUAUUUGGUUCAUAAGGGAUUCGGAGUCCUGAUGAUGGAUGAAAUGUGGAUGAUGGUGAGCCUUUUUUGGCUA
AG
>Ado-MIR1430-3_l
UAGCCAAGAAUGGCUUGCCUAUCUCCACUUUUUGGUUCAUCAGGGAGUCCAUAUGUAUGAUGGAUGAAAUGUGGAUGAUGGUGAGCCUUUUUUGGCUAAG
>Ado-MIR1430-4_r
UAGCCAAGAAUGGCUUGCCUAUCUCCACUUUUUGGUUCAUCAGGGAGUCCAUAUGUAUGAUGGAUGAAAUGUGGAUGAUGGUGAGCCUUUUUUUGGCUAA
G
>Ado-MIR156a_r
UGACAGAAGAGAGUGAGCACACGUGGUCGUUUCCCUGCAUGCAUGGCGCCCUCAUGCUUGGAGCUACGUGUGCUCACUUCUCUUCUUGUCACC
>Ado-MIR156b_r
UGACAGAAGAGAGUGAGCACACACGGUGGUUUCUUAGCAUGCAAGCGCCAUGCUGGGAGCUGUGCGUGCUCACUCUCUAUCUGUCGG
>Ado-MIR156c_r
UAACAGAAGAGAGUGAGCACACAUGGUGCCUUUCUUGCAUGCUGAAUGAGAGAGUUCAUGCUUGGAGCUAUGUGUGCUCACUUCUCUCUCUGUCAGC
>Ado-MIR156g-1_r
UGACAGAAGAGAGUGAGCACACAACGGGCAGACUGCAUCGAUCUAAACCUUUGGAACCGAGGCGGCACAAGUACGAAUGAUGCAGCUGCUGUUGCGUGCU
CACUUCUCUCUCUGUCAGU
>Ado-MIR156g-2_r
UGACAGAAGAGAGUGAGCACGCAGCGGCCAGAUUGCAUCUAAAUACGGCGAUAAAGAUGACGGAUCGAUGAUGGUUUGCCUAGACGCAGCUGCUGUUGCG
UGCUCACUUCUCUUUCUGUCAGC
>Ado-MIR156g-3_r
UGACAGAAGAGAGUGAGCACGCAGCGGCCAGAUUGCAUCUAAAUACGGCGAUAAAGAUGACGGAUCGAUGAUGGUUUGCCUAGACGCAGCUGCUGUUGCG
UGCUCACUUCUCUUUCUGUCAGC
>Ado-MIR156j_r
UGACAGAAGAGAGUGAGCACACGGCGCGGUGGCCGGCACCGAACGGGUGCCGUCCCCGCCGCGUGCUCGCUCCUCUUUCUGUCAGC
>Ado-MIR156k_l
UGACAGAAGAGAGAGAGCACAACCCAGCACCAGCGAGGACGGCUCGCUUCUGCGAGGGCCGUGUGCUCUCUGCUCUCACUGUCAUU
>Ado-MIR160b-1_c
UGCCUGGCUCCCUGUAUGCCACACAUGUAGCCCAAUCCGUGGCGCGAUUGGAUGCUGUGGGUGGCGUGCAAGGAGCCAAGCAUG
>Ado-MIR160b-2_r
UGCCUGGCUCCCUGUAUGCCACACAUGUAGCCCAAUCCGUGGCGCGAUUGGAUGCUGUGGGUGGCGUGCAAGGAGCCAAGCAUG
>Ado-MIR160b-3_r
UGCCUGGCUCCCUGUAUGCCACACAUGUAGCCCAAUCCGUGGCGCGAUUGGAUGCUGUGGGUGGCGUGCAAGGAGCCAAGCAUG
>Ado-MIR160c_b
UGCCUGGCUCCCUGUAUGCCACUCAUCUAGAGCAACAUUGUUAUGAGGUUGCCUAACGAUGGAUGGCGUGCACGGAGCCAAGCAUA
>Ado-MIR166a-1_r
GGAAUGUUGUCUGGUUCAAGGUUUUGCCUGUGAUUUGAGGAUGAUGUAUGUGUAGUUUUUAAUUCCUCGAAUCUUCGGGAUCUCGGACCAGGCUUCAUUC
CCC
>Ado-MIR166a-2_r
GGAAUGUUGUCUGGUUCAAGGUUUUGCCUGUGAUUUGAGGAUGAUGUAUGUGUAGUUUUUAAUUCCUCGAAUCUUCGGGAUCUCGGACCAGGCUUCAUUC
CCC
>Ado-MIR167d-1_b
UGAAGCUGCCAGCAUGAUCUGAUGAGUGGCUAUGCAGUUCACAGUAAUAUUAUAUUAGUGAUCAAAGCUAGCUUCCAGAUCAUGCUGUGCAGUUUCAUC
>Ado-MIR167d-2_l
UGAAGCUGCCAGCAUGAUCUGAUGAGUGGCUAUGCAGUUCACAGUAAUAUUAUAUUAGUGAUCAAAGCUAGCUUCCAGAUCAUGCUGUGCAGUUUCAUC
>Ado-MIR167g-1_b
UGAAGCUGCCAGCAUGAUCUGAACUCAGGGAAACAUUAGUGAUCAAAGCUAACUUUCAGAUCAUGCUGUGCGGUUUCAUC
>Ado-MIR167g-2_c
UGAAGCUGCCAGCAUGAUCUGAACUCAGGGAAACAUUAGUGAUCAAAGCUAACUUUCAGAUCAUGCUGUGCGGUUUCAUC
>Ado-MIR167g-3_r
UGAAGCUGCCAGCAUGAUCUGAACUCAGGGAAACAUUAGUGAUCAAAGCUAACUUUCAGAUCAUGCUGUGCGGUUUCAUC
>Ado-MIR169a_r
CAGCCAAGGAUGACUUGCCGAUCGAUGAUGCCUGUGAUCGUCAGAUUGAUGCGUGUUUGUUUGGUCAUCGGCAAGUUGUCCUUGGCUAC
>Ado-MIR169c-1-1_b
CAGCCAAGGAUGACUUGCCGGCUCCUGGCGUUGGGCUGCCUCAGCUUUGUUGAGGCUUAAAGAGUUAGCCGGCAAGUCUGUCCUUGGCUAC
>Ado-MIR169c-1-2_b
UAGCCAAGGACAGACUUGCCGGCUAACUCUUUAAGCCUCAACAAAGCUGAGGCAGCCCAACGCCAGGAGCCGGCAAGUCAUCCUUGGCUGCA
>Ado-MIR169c-1-1_c
CAGCCAAGGAUGACUUGCCGGCUCCUGGCGUUGGGCUGCCUCAGCUUUGUUGAGGCUUAAAGAGUUAGCCGGCAAGUCUGUCCUUGGCUAC
>Ado-MIR169c-1-2_c
UAGCCAAGGACAGACUUGCCGGCUAACUCUUUAAGCCUCAACAAAGCUGAGGCAGCCCAACGCCAGGAGCCGGCAAGUCAUCCUUGGCUGCA
>Ado-MIR169c-2_l
CAGCCAAGGAUGACUUGCCGGCGUCCGCGCGUCGACGCCGACGGCAUUGUUGCCAAUGCGGCCGGCAAGUCUGUCCUUGGCUAC
>Ado-MIR169c-3-1_r
CAGCCAAGGAUGACUUGCCGGCUCCUGCCAUUUGCUUUGCUGAGGCUUAAAAGAUUUAGCCGGCAAGUCUGUCCUUGGCUAC
>Ado-MIR169c-3-2_r
UAGCCAAGGACAGACUUGCCGGCUAAAUCUUUUAAGCCUCAGCAAAGCAAAUGGCAGGAGCCGGCAAGUCAUCCUUGGCUGCA
>Ado-MIR169i_r
UGGCGAUAAGGGUGUAGCUCUGGUAGCCAAGGAUGACUUGCCUGUGUCCUCCCUAAAGGAUCAGCAAAACAAUGAGCCUUUGAGUGGUCUCAUGGGCAGU
CUCCUUGGCUAGCCUGAGUGGCUCUUAUUGCUCAUG

>Ado-MIR169n-1_l
GAGCCAAGAAUGACUUGCCUAUGCAUGCCUUCUGUUAGCAUCUCCAACAGCCAUGGCAACGGGAAGAGAAAUUCUUAUGAUGCGAUGCAAUGUGGCUGCA
CGGGCAGGUCUUCUUGGCUCUU
>Ado-MIR169n-2_r
UAGCCAAGAAUGACUUGCCUAUGCAUGCCUUCUAUUAGCAGCUCCAUCAGCCAUGGCAAUGGGAAGGAUUUCUGAAGAAGGCAUGCCUUCUGUUAGCAGC
UCCAUCAGCCAUGGCAAUGGGAAGGAUUUCUGAAGAAAUUCUUGUGUGAUGGAUGAUGCAAUGUGGCUGCAUGGGCCGGUCAUCUUGGCUAGC
>Ado-MIR169n-3_r
GAGCCAAGAAUGACUUGCCUAUGCAUGCCUUCUGUUAGCAUCUCCAACAGCCAUGGCAACGGGUAGAGAAAUUCUUAUGAUGCGAUGCAAUGUGGCUGCA
CGGGCAGGUCUUCUUGGCUCUU
>Ado-MIR169p_l
UAGCCAAGGACAGACUUGCCGGCCGCAUUGGCAACAAUGCCGUCGGCGUCGACGCGCGGACGCCGGCAAGUCAUCCUUGGCUGCA
>Ado-MIR169q-1_b
UAGCCAAGGAUGACUUGCCUAUGUCCUUUGUACCGAAGGAUAAGCAAUGUGAGCCUUUGAGAUGGUUCAUGGGCAAGUUUCCUUGGCUAGC
>Ado-MIR169q-1_c
UAGCCAAGGAUGACUUGCCUAUGUCCUUUGUACCGAAGGAUAAGCAAUGUGAGCCUUUGAGAUGGUUCAUGGGCAAGUUUCCUUGGCUAGC
>Ado-MIR169q-2_b
UAGCCAAGGAUGACUUGCCUAUGUCCUUUGUACCGAAGGAUAAGCAAUGUGAGCCUUUGAGAUGGUUCAUGGGCAAGUUUCCUUGGCUAGC
>Ado-MIR169q-3_l
UAGCCAAGGAUGACUUGCCUGUGUCCUCCCUAAAGGAUCAGCAAAACAAUGAGCCUUUGAGUGGUCUCAUGGGCAGUCUCCUUGGCUAGC
>Ado-MIR169q-4_l
UAGCCAAGGAUGACUUGCCUGUGGCCUCGCAAGCUCGCUUGCAGUUUGUCAGGCUGGCAGGUGAUUCGUCAUAGGCAGUCUCCUUGGCUAGC
>Ado-MIR169q-5_l
UAGCCAAGGAUGACUUGCCUAUGUCCUUUGUACCGAAGGAUAAGCAAUGUGAGCCUUUGAGAUGGUUCAUGGGCAAGUUUCCUUGGCUAGC
>Ado-MIR169q-6_r
UAGCCAAGGAUGACUUGCCUGUGUCCUCCCUAAAGGAUCAGCAAAACAAUGAGCCUUUGAGUGGUCUCAUGGGCAGUCUCCUUGGCUAGC
>Ado-MIR169q-7_r
UAGCCAAGGAUGACUUGCCUAUGUCCUUUGUACCGAAGGAUAAGCAAUGUGAGCCUUUGAGAUGGUUCAUGGGCAAGUUUCCUUGGCUAGC
>Ado-MIR171a_c
UAUUGGUGAGGUUCAAUCCGAUGAUGGGGUUUUACGUGACAGUAAAGCCGGUAUCUGAUUGAGCCGCGCCAAUAUC
>Ado-MIR171c-1_b
UAUUGGCGCGGUUCAAUCAGAAAGCUUGUGCUCCAAAGGCGUGGGGCUCCACUCUUUGAUUGAGCCGUGCCAAUAUC
>Ado-MIR171c-2_c
UAUUGGCGCAGUUCAAUUAGAGAGCUUGCGCUCCGGAGGCGAGGGGCUCAGCUCUUUGAUUGAGCCGUGCCAAUAUC
>Ado-MIR171f_r
UGUUGGCAUGGUUCAAUCAAACCGGGUAAAAUUUGCACUUGCGAGGUUCAGGGAUGUUCUAUUUCCCGGGUCUGAUUGAGCCGUGCCAAUAUC
>Ado-MIR171i-1_b
UAUUGGUGCGCCUCAAUCCGAAGGCGUGGCUGAUUGGAACAGCAGCCAUGUUCUUGGAUUGAGCCGCGUCAAUAUC
>Ado-MIR171i-2_c
UAUUGGUGCGCCUCAAUCUGAAGGCGUGGCUGAUUGGAACAGCAGCCAUGUUCUUGGAUUGAGCCGCGUCAAUAUC
>Ado-MIR172b_b
GUGGCAUCAUCAAGAUUCACACAGCUAUAUACAGCGUUGGCUGUCGAUGCAAGCAUCAUAUUCUGUGAUGUAUGCCUUUGCAUGUGGGAAUCUUGAUGAU
GCUGCAU
>Ado-MIR172d-1-1_b
GCAGCACCAUCAAGAUUCACAUCGAAACUCAUCUCGAGAGAGGUGGUUUGCAGUUUCCAUAUAAACAUGUAUAUAUGAUGAGAAUCUUGAUGAUGCUGCA
U
>Ado-MIR172d-1-2_b
GCAGCAUCAUCAAGAUUCUCAUCAUAUAUACAUGUUUAUAUGGAAACUGCAAACCACCUCUCUCGAGAUGAGUUUCGAUGUGAAUCUUGAUGGUGCUGCA
C
>Ado-MIR172d-1-1_l
GCAGCACCAUCAAGAUUCACAUCGAAACUCAUCUCGAGAGAGGUGGUUUGCAGUUUCCAUAUAAACAUGUAUAUAUGAUGAGAAUCUUGAUGAUGCUGCA
U
>Ado-MIR172d-1-2_l
GCAGCAUCAUCAAGAUUCUCAUCAUAUAUACAUGUUUAUAUGGAAACUGCAAACCACCUCUCUCGAGAUGAGUUUCGAUGUGAAUCUUGAUGGUGCUGCA
C
>Ado-MIR172d-1-1_r
GCAGCACCAUCAAGAUUCACAUCGAAACUCAUCUCGAGAGAGGUGGUUUGCAGUUUCCAUAUAAACAUGUAUAUAUGAUGAGAAUCUUGAUGAUGCUGCA
U
>Ado-MIR172d-1-2_r
GCAGCAUCAUCAAGAUUCUCAUCAUAUAUACAUGUUUAUAUGGAAACUGCAAACCACCUCUCUCGAGAUGAGUUUCGAUGUGAAUCUUGAUGGUGCUGCA
C
>Ado-MIR172d-2-1_c
GCAGCAUCAUCAAGAUUCACAUCCAACUCAUCUUGAGAGGCAGGUGUUGUUUACAGUUUCUACACUUGUAUACAUGAUGAGAAUCUUGAUGAUGCUGCAU
>Ado-MIR172d-2-2_c
GCAGCAUCAUCAAGAUUCUCAUCAUGUAUACAAGUGUAGAAACUGUAAACAACACCUGCCUCUCAAGAUGAGUUGGAUGUGAAUCUUGAUGAUGCUGCAC
>Ado-MIR319a-1-1_c
GAGCUCUCUUCAGUCCACUCUGAAAUGGCUGUAGGGUUUCAUUAGCUGCCGAUUCAUCCAUUCACCUACCAAGAACCAUGAAGAGGGAUAUGUUCUUGGU
AGCCGAGUGGAUGGCGCGGGAGCUAAAAUCAAGCUCUGCGCUGUUUGUGGUUGGACUGAAGGGUGCUCCC
>Ado-MIR319a-1-2_c
AGCUGCCGAUUCAUCCAUUCACCUACCAAGAACCAUGAAGAGGGAUAUGUUCUUGGUAGCCGAGUGGAUGGCGCGGGAGCUAA
>Ado-MIR319a-2-1_r
GAGCUCUCUUCAGUCCACUCUGAAAUGGCUGUAGGGUUUCAUUAGCUGCCGAUUCAUCCAUUCACCUACCAAGAACCAUGAAGAGGGAUAUGUUCUUGGU
AGCCGAGUGGAUGGCGCGGGAGCUAAAAUCAAGCUCUGCGCUGUUUGUGGUUGGACUGAAGGGUGCUCCC
>Ado-MIR319a-2-2_r
AGCUGCCGAUUCAUCCAUUCACCUACCAAGAACCAUGAAGAGGGAUAUGUUCUUGGUAGCCGAGUGGAUGGCGCGGGAGCUAA
>Ado-MIR393a_r
UCCAAAGGGAUCGCAUUGAUCCUUCAUUCAUCCAUGGUGGUCGUCGUCUACAAGCGCGGCUCGAUGGAUCAUGCGAUCCUUUUGGAGG
>Ado-MIR393b-1_b

UCCAAAGGGAUCGCAUUGAUCUAAUCCGCCGCCGCCGCCGGCGUGCAAGCACAAGUAAUCAUGCAUGUCACAGAUGCGUGCAUGGCUGCGGCGCGCGAGG
ACAAGAUCAGUGCAAUCCCUUUGGAAU
>Ado-MIR393b-2_c
UCCAAAGGGAUCGCAUUGAUCUAAUCCGCCGCCGCCGCCGGCGUGCAAGCACAAGUAAUCAUGCAUGUCACAGAUGCGUGCAUGGCUGCGGCGCGCGAGG
ACAAGAUCAGUGCAAUCCCUUUGGAAU
>Ado-MIR393b-3_l
UCCAAAGGGAUCGCAUUGAUCUAAUCCGCCGCCGCCGCCGGCGUGCAAGCACAAGUAAUCAUGCAUGUCACAGAUGCGUGCAUGGCUGCGGCGCGCGAGG
ACAAGAUCAGUGCAAUCCCUUUGGAAU
>Ado-MIR396a_c
UUCCACAGCUUUCUUGAACUGCAUGAGCUCGAAGUGGCAGCUCUUUUAGAAUAGAUUGCAUUUUGGCUUGAGUUACAGAGCAGUUCAAUAAAGCUGUGGG
AAA
>Ado-MIR396b_c
UUCCACAGCUUUCUUGAACUGCAUCUGCUGGAGAUGCAGCUAGCUUCUACUUGCGUAGCUGCAGUUCAAUAAAGCUGUGGGAAA
>Ado-MIR444a-1-1_b
AGCAAGCUAGAUGCAGCAACUGCAUAAAUUGCAAGAGCUUGUUGGUUGAGGAUCAUACCGAUGGUGCUCUUGCAAGUUAUGCAGUUGCUGCCUCAAGCUU
CCUGC
>Ado-MIR444a-1-2_b
GCUAGAUGCAGCAACUGCAUAAAUUGCAAGAGCUUGUUGGUUGAGGAUCAUACCGAUGGUGCUCUUGCAAGUUAUGCAGUUGCUGCCUCAAGCUU
>Ado-MIR444a-2-1_c
AGCAAGCUAGAGGCGGCAACUGCAUAACUUGCAAGAGCUUGUUGGUUGAAGAUCAUACCGAUGGUGCUCUUGCAAGUUAUGCAGUUGCUGCCUCAAGCUU
CCUGC
>Ado-MIR444a-2-2_c
GCUAGAGGCGGCAACUGCAUAACUUGCAAGAGCUUGUUGGUUGAAGAUCAUACCGAUGGUGCUCUUGCAAGUUAUGCAGUUGCUGCCUCAAGCUU
>Ado-MIR444a-2-1_l
AGCAAGCUAGAGGCGGCAACUGCAUAACUUGCAAGAGCUUGUUGGUUGAAGAUCAUACCGAUGGUGCUCUUGCAAGUUAUGCAGUUGCUGCCUCAAGCUU
CCUGC
>Ado-MIR444a-2-2_l
GCUAGAGGCGGCAACUGCAUAACUUGCAAGAGCUUGUUGGUUGAAGAUCAUACCGAUGGUGCUCUUGCAAGUUAUGCAGUUGCUGCCUCAAGCUU
>Ado-MIR444c-1-1_b
GCUUGAGACAACAACUGCACAACUUGCAAGAAAAUCAUCGGUAUGGUCCGAAAACCAAUUUUCUUGCAAGAUAUGCAGUUGCUGCCUCUAGCUU
>Ado-MIR444c-1-2_b
UGACAAGCUAGAGGCAGCAACUGCAUAUCUUGCAAGAAAAUUGGUUUUCGGACCAUACCGAUGAUUUUCUUGCAAGUUGUGCAGUUGUUGUCUCAAGCUU
GCUGCC
>Ado-MIR444c-1-3_b
GCUAGAGGCAGCAACUGCAUAUCUUGCAAGAAAAUUGGUUUUCGGACCAUACCGAUGAUUUUCUUGCAAGUUGUGCAGUUGUUGUCUCAAGCUU
>Ado-MIR444c-1-1_c
GCUUGAGACAACAACUGCACAACUUGCAAGAAAAUCAUCGGUAUGGUCCGAAAACCAAUUUUCUUGCAAGAUAUGCAGUUGCUGCCUCUAGCUU
>Ado-MIR444c-1-2_c
UGACAAGCUAGAGGCAGCAACUGCAUAUCUUGCAAGAAAAUUGGUUUUCGGACCAUACCGAUGAUUUUCUUGCAAGUUGUGCAGUUGUUGUCUCAAGCUU
GCUGCC
>Ado-MIR444c-1-3_c
GCUAGAGGCAGCAACUGCAUAUCUUGCAAGAAAAUUGGUUUUCGGACCAUACCGAUGAUUUUCUUGCAAGUUGUGCAGUUGUUGUCUCAAGCUU
>Ado-MIR444c-1-1_r
UGACAAGCUAGAGGCAGCAACUGCAUAUCUUGCAAGAAAAUUGGUUUUCGGACCAUACCGAUGAUUUUCUUGCAAGUUGUGCAGUUGUUGUCUCAAGCUU
GCUGCC
>Ado-MIR444c-1-2_r
GCUUGAGACAACAACUGCACAACUUGCAAGAAAAUCAUCGGUAUGGUCCGAAAACCAAUUUUCUUGCAAGAUAUGCAGUUGCUGCCUCUAGCUU
>Ado-MIR444c-1-3_r
GCUAGAGGCAGCAACUGCAUAUCUUGCAAGAAAAUUGGUUUUCGGACCAUACCGAUGAUUUUCUUGCAAGUUGUGCAGUUGUUGUCUCAAGCUU
>Ado-MIR444c-2-1_b
CGGCAAGCUAGAGACAACAACUGCAUAACUUGCAAGAAAAUCGGUUUUCUAGCCAUACCGAUGACUUUCUUGCAGGUUGUGCAGUUGUUGUCUCAAGCUU
GCAGCC
>Ado-MIR444c-2-2_b
GCUAGAGACAACAACUGCAUAACUUGCAAGAAAAUCGGUUUUCUAGCCAUACCGAUGACUUUCUUGCAGGUUGUGCAGUUGUUGUCUCAAGCUU
>Ado-MIR444c-2-3_b
GCUUGAGACAACAACUGCACAACCUGCAAGAAAGUCAUCGGUAUGGCUAGAAAACCGAUUUUCUUGCAAGUUAUGCAGUUGUUGUCUCUAGCUU
>Ado-MIR444c-3-1_r
GCUAGAGACAGCAACUGCAUAACUUGCAAGAAAAUCGGUUUCGAGCCAUACCGAUGACUUUCUUGCAAGUUGUGCAGUUGUUGUCUCAAGCUU
>Ado-MIR444c-3-2_r
CGGCAAGCUAGAGACAGCAACUGCAUAACUUGCAAGAAAAUCGGUUUCGAGCCAUACCGAUGACUUUCUUGCAAGUUGUGCAGUUGUUGUCUCAAGCUUG
CUGCC
>Ado-MIR444c-4-1_c
CGGCAAGCUAGAGACAGCAACUGCAUAACUUGCAAGAAAAUCGGUUUCGAGCCAUACCGAUGACUUUCUUGCAAGUUGUGCAGUUGUUGUCUCAAGCUUG
CUGCC
>Ado-MIR444c-4-2_c
GCUAGAGACAGCAACUGCAUAACUUGCAAGAAAAUCGGUUUCGAGCCAUACCGAUGACUUUCUUGCAAGUUGUGCAGUUGUUGUCUCAAGCUU
>Ado-MIR444d-1-1_b
ACCAAGCAUGAGGCAGGAACUGCAUUACUUGCAAGGAAGUCACAAAAUCAAUUGGAAGAUCAUACUUGUGGCUUUCUUGCAAGUUGUGCAGUUGUUGCCU
CAAGCUUGCUGC
>Ado-MIR444d-1-2_b
CACCAAGCAUGAGGCAGGAACUGCAUUACUUGCAAGGAAGUCACAAAAUCAAUUGGAAGAUCAUACUUGUGGCUUUCUUGCAAGUUGUGCAGUUGUUGCC
UCAAGCUUGCUGCC
>Ado-MIR444d-1-3_b
ACUUGCAAGGAAGUCACAAAAUCAAUUGGAAGAUCAUACUUGUGGCUUUCUUGCAAGUUG
>Ado-MIR444d-1-4_b
GCAUGAGGCAGGAACUGCAUUACUUGCAAGGAAGUCACAAAAUCAAUUGGAAGAUCAUACUUGUGGCUUUCUUGCAAGUUGUGCAGUUGUUGCCUCAAGC
UU
>Ado-MIR444d-2-1_b

ACCAAGCAUGAGGCAGGAACCGCAUUACUUGCAAGGAAGUCACAAAAUCAAUUGGAAGAUCAUACUUGUGGCUUUCUUGCAAGUUGUGCAGUUGUUGCCU
CAAGCUUGCUGC
>Ado-MIR444d-2-2_b
CACCAAGCAUGAGGCAGGAACCGCAUUACUUGCAAGGAAGUCACAAAAUCAAUUGGAAGAUCAUACUUGUGGCUUUCUUGCAAGUUGUGCAGUUGUUGCC
UCAAGCUUGCUGCC
>Ado-MIR444d-2-3_b
ACUUGCAAGGAAGUCACAAAAUCAAUUGGAAGAUCAUACUUGUGGCUUUCUUGCAAGUUG
>Ado-MIR444d-2-4_b
GCAUGAGGCAGGAACCGCAUUACUUGCAAGGAAGUCACAAAAUCAAUUGGAAGAUCAUACUUGUGGCUUUCUUGCAAGUUGUGCAGUUGUUGCCUCAAGC
UU
>Ado-MIR444d-2-1_c
ACCAAGCAUGAGGCAGGAACCGCAUUACUUGCAAGGAAGUCACAAAAUCAAUUGGAAGAUCAUACUUGUGGCUUUCUUGCAAGUUGUGCAGUUGUUGCCU
CAAGCUUGCUGC
>Ado-MIR444d-2-2_c
CACCAAGCAUGAGGCAGGAACCGCAUUACUUGCAAGGAAGUCACAAAAUCAAUUGGAAGAUCAUACUUGUGGCUUUCUUGCAAGUUGUGCAGUUGUUGCC
UCAAGCUUGCUGCC
>Ado-MIR444d-2-3_c
ACUUGCAAGGAAGUCACAAAAUCAAUUGGAAGAUCAUACUUGUGGCUUUCUUGCAAGUUG
>Ado-MIR444d-2-4_c
GCAUGAGGCAGGAACCGCAUUACUUGCAAGGAAGUCACAAAAUCAAUUGGAAGAUCAUACUUGUGGCUUUCUUGCAAGUUGUGCAGUUGUUGCCUCAAGC
UU
>Ado-MIR444d-2-1_l
ACCAAGCAUGAGGCAGGAACCGCAUUACUUGCAAGGAAGUCACAAAAUCAAUUGGAAGAUCAUACUUGUGGCUUUCUUGCAAGUUGUGCAGUUGUUGCCU
CAAGCUUGCUGC
>Ado-MIR444d-2-2_l
CACCAAGCAUGAGGCAGGAACCGCAUUACUUGCAAGGAAGUCACAAAAUCAAUUGGAAGAUCAUACUUGUGGCUUUCUUGCAAGUUGUGCAGUUGUUGCC
UCAAGCUUGCUGCC
>Ado-MIR444d-2-3_l
ACUUGCAAGGAAGUCACAAAAUCAAUUGGAAGAUCAUACUUGUGGCUUUCUUGCAAGUUG
>Ado-MIR444d-2-4_l
GCAUGAGGCAGGAACCGCAUUACUUGCAAGGAAGUCACAAAAUCAAUUGGAAGAUCAUACUUGUGGCUUUCUUGCAAGUUGUGCAGUUGUUGCCUCAAGC
UU
>Ado-MIR444d-3-1_c
ACCAAGCAUGAGGCAGGAACUGCAUUACUUGCAAGGAAGUCACAAAAUCAAUUGGAAGAUCAUACUUGUGGCUUUCUUGCAAGUUGUGCAGUUGUUGCCU
CAAGCUUGCUGC
>Ado-MIR444d-3-2_c
CACCAAGCAUGAGGCAGGAACUGCAUUACUUGCAAGGAAGUCACAAAAUCAAUUGGAAGAUCAUACUUGUGGCUUUCUUGCAAGUUGUGCAGUUGUUGCC
UCAAGCUUGCUGCC
>Ado-MIR444d-3-3_c
ACUUGCAAGGAAGUCACAAAAUCAAUUGGAAGAUCAUACUUGUGGCUUUCUUGCAAGUUG
>Ado-MIR444d-3-4_c
GCAUGAGGCAGGAACUGCAUUACUUGCAAGGAAGUCACAAAAUCAAUUGGAAGAUCAUACUUGUGGCUUUCUUGCAAGUUGUGCAGUUGUUGCCUCAAGC
UU
>Ado-MIR444d-4-1_l
ACCAAGCAUGAGGCAGGAACUGCAUUACUUGCAAGGAAGUCACAAAAUCAAUUGGAAGAUCAUACUUGUGGCUUUCUUGCAAGUUGUGCAGUUGUUGCCU
CAAGCUUGCUGC
>Ado-MIR444d-4-2_l
CACCAAGCAUGAGGCAGGAACUGCAUUACUUGCAAGGAAGUCACAAAAUCAAUUGGAAGAUCAUACUUGUGGCUUUCUUGCAAGUUGUGCAGUUGUUGCC
UCAAGCUUGCUGCC
>Ado-MIR444d-4-3_l
ACUUGCAAGGAAGUCACAAAAUCAAUUGGAAGAUCAUACUUGUGGCUUUCUUGCAAGUUG
>Ado-MIR444d-4-4_l
GCAUGAGGCAGGAACUGCAUUACUUGCAAGGAAGUCACAAAAUCAAUUGGAAGAUCAUACUUGUGGCUUUCUUGCAAGUUGUGCAGUUGUUGCCUCAAGC
UU
>Ado-MIR444d-5-1_r
ACCAAGCAUGAGGCAGGAACCGCAUUACUUGCAAGGAAGUCACAAAAUCAAUUGGAAGAUCAUACUUGUGGCUUUCUUGCAAGUUGUGCAGUUGUUGCCU
CAAGCUUGCUGC
>Ado-MIR444d-5-2_r
CACCAAGCAUGAGGCAGGAACCGCAUUACUUGCAAGGAAGUCACAAAAUCAAUUGGAAGAUCAUACUUGUGGCUUUCUUGCAAGUUGUGCAGUUGUUGCC
UCAAGCUUGCUGCC
>Ado-MIR444d-5-3_r
ACUUGCAAGGAAGUCACAAAAUCAAUUGGAAGAUCAUACUUGUGGCUUUCUUGCAAGUUG
>Ado-MIR444d-5-4_r
GCAUGAGGCAGGAACCGCAUUACUUGCAAGGAAGUCACAAAAUCAAUUGGAAGAUCAUACUUGUGGCUUUCUUGCAAGUUGUGCAGUUGUUGCCUCAAGC
UU
>Ado-MIR444e-1_r
AGUAAGCUAGAGGCGGCAACUGCAUAACUUGCGAGAAACUUGUUGGAUGAUGGUCAUGCCGAUGAUUUUGCCGCAAGUUAUGCAGUUGCUGCCUCAAGCU
UACUGC
>Ado-MIR444e-2_r
GCUAGAGGCGGCAACUGCAUAACUUGCGAGAAACUUGUUGGAUGAUGGUCAUGCCGAUGAUUUUGCCGCAAGUUAUGCAGUUGCUGCCUCAAGCUU
>Ado-MIR529a_c
AGAAGAGAGAGAGUACAGCCUUGUUACCAUAUAUACACCUCCGAUGUCAUCAGAUAUAUGUGAGAUGUGUUGAAGGCUGUACCCUCUCUCUUCUUC
>Ado-MIR827-1_b
UUUUGUUGGUGGUCAUUUAACUAUGCUCGCAGGAUUAUCAUGAUCACGCAUGCAUGAAUCGUUAGAUGACCAUCAGCAAACA
>Ado-MIR827-2_r
UUUUGUUGGUGGUCAUUUAACUAUGCUCGCAGGAUUAUCAUGAUCACGCAUGCAUGAAUCGUUAGAUGACCAUCAGCAAACA

Predicted *A. donax* miRNA targets

```
>Locus_15085_Transcript_23/40_Confidence_0.434_Length_1898
GCTGCTCGAGGAGCTCAGCTTCAAGCGCATGGTTGTCACCGACGAGTGCCTCGAGATGATCGCGGTTTCCTTCAGGAACTTCCAGGTCCTGCGCCTTGTC
TCCTGCGAGGGCTTCAGCACCGCCGGGCTCGCCGCCATTGCCGCCGGCTGCAGAAATCTAAGGGAACTTGACCTGCAGGAGAATGAGATTGAGGATTGCT
CTAGTCATTGGCTCAGTCTCTTTCCAGAATCCCTCACTTCGTTGGTAACACTAAATTTTTCATGCGTAGAGGGGGATGTCAATTTCACTGTACTTGAACG
ACTAGTAACCAGATGTCGCAACCTCAAGACTCTCAAGCTCAACAATGCTATCCCTCTTGACAAGCTTGCTAGCCTCCTTCGTAAGGCTCCTCAAATTGTG
GAGCTTGGAACAGGAAGGTTCTCTGCTGACTACCACCCAGATCTCTTTGCAAAGCTTGAAGCAGCGTTTGCTGGTTGTAAAAGCCTACAAAGGCTTTCTG
GGGCTTGGGATTCTGTTCCAGAGTATCTGCCAGCATTTTATTGTGTATGTGAGGGCCTCACATCACTTAATCTGAGTTATGCTACTGTGCGAGGCCCTGA
GCTGATAAAATTTATTAGCAGATGCAAAAACTTGCAACTATTATGGGTGATGGACTTAATTGAGGACCACGGTCTAGCTGTTGTGGCATCATCTTGCAAT
AAACTACAGGAGTTGCGGGTCTTCCCTTCTGCCCCTTTTGATCCAGCTGAGCAAGTTTCATTGACTGAAAGGGGACTTGTTGATGTTTCTGCCAGTTGCC
CGAATGTTGGAGTCAGTCCTCTACTTCTGCAGACGGATGACTAATGAGGCCCTTATTACCATAGCAAAGAACCGGCCCAACTTCACCTGCTTTCGCTTAT
GCATCATCGAGCCTCACACTCCAGATTACATCACGCATCAGCCTCTTGATGCAGGTTTCAGTGCCATTGTGGAATCATGCAAAGGCCTCAGGCGCCTATC
TGTCTCAGGCCTTCTCACAGATCGTGTATTTAAATCCATTGGGGCACATGCTGATCGTCTTGAGATGCTCTCAATCGCCTTTGCTGGGAATAGCGGATTG
GGCCTTCATTACATCCTTTCGGGCTGCAAGAGCTTGAAGAAGCTGGAGATCAGGGACTGCCCATTTGGTGATAAGCCCTTGCTGGCAAATGCTGCCAAGC
TGGAGACAATGCGATCCCTTTGGATGTCGTCGTGCTCACTGACCCTGGGCGCATGCCGACAGCTTGCACGCAAGATGCCCCGCCTTAGTGTGGAGGTCAT
GAATGATCCTCGACGGGCATGCCCCTTGGATTCACTTACAGATGAAAGCCATGTCGAGACATTGTATGTCTACCGGACAATCGCAGGTCCAAGGTCCGAC
ACACCAGCCTGTGTCCAGATTATTTAGGGGGACCACCTGTGGTATGGGATAGGTACTATGGAAGTGTTCCTTGTCAATCCCGATTTGTACCTTCAACACC
TTCCTAGCATAACATGGATTGTCTCCTTGAAGTATGAAGGCTGGTGGATTGGCTGATCCTGTTATAACCGCTGCTCAGCTATGAAACTGTTTGTGAGTTT
AGGACGAGACTCGGGGCCCTTTCAGGTGTTAATAACTGTAGGTGGAGATTCGATGAGTTACAGAAACTACTAGATAGCGTCTTGAGAAAACTTGGGTTTG
TTTTCTTAGCGTGTTTAGTTCTTTTCATGTATAAAAATATGGGATCATTTCAATCTCGCAATGCACTGTACTATCGGAACAAATTGTACCAGGAACCTCT
ACTTTTTTTACCTCCCCTCCCTAAGTATGGGTTGTTGCTTCAACTTGTTGACTTGTTGTGAATGCGGGAATAATTCAAGCTTGTGGTGCTGGGATGAT
>Locus_11912_Transcript_47/63_Confidence_0.314_Length_3761
CACTGTCCAGATTCGCCTCCCTCCCCTCCCCCTAGCTCCTACTTCCCGCTCCTCCAGCTACGGCCGCGCGCTCCCCCTCCCCGCCGGCCACCTTGGGGGG
CTCCTCCCCGCCGCGCGTCCGGCCGCTCTTTTCCTGGCGCCTCCCGCCGGGCGCTCGCCCCGGATTTCTCGGCTCCCCATCCGGCGGACGCCTTGACCGG
GGTGGGTTCTTGGTTCCGGCGGCTCGGTGATCTGTGCGTGCGAAAGGCGGGGCTTTTAGCTCGTTCTGTGGGGGGAGGAGGAAGACGGCGGCGAGGCGTT
CATGGGTTAGATCTGGCGGTCCGGCGCCGCGAATCTGAGCGAGGTTTCGGTGGAGGGATCGGGGCGGAGAAGTCGCAGTCTCTGGCTCGTTCTGAGCAGC
TTGGGCCTCTCATGTCCAAATCGGGTATGGATGATGGGTGCGGTAACTGTCGGAAGGAGCGTCGCTTGCAGATCTGAAAGCCATCCAGCTCCCTTGTTCG
GTTGACCTGGAATGGCCGTGCTTTCTGCTGTTTGACTGTCAGTAGTTGCTGCTTAGTGTGTGTGCAAGAAATTTAGCTGGAGGTGATGTGCTTAGGAAAC
TAGTCTAGTTGCTGATATCTTGGTCTCTGTTGAAGAACGCTGAAGCTGGAAGGAAGTAGGTCGCATTCCTGTGTTGTTGATATTTTGTTGTTTCTCGAAT
AACCTGGAGGCTGGAAGGGATCAGATCCAGTTCCTGTGGCACCGGTAACAAGGTGCTGTTGGGGGAAGGCACCATGACCTACTTCCCTGAGGAGGTGGTG
GAGCATATATTCAGCTTCTTGCCGTCGCACAGTGACCGGAACACGGTCTCGTTAGTGTGCAAGGTGTGGTATGAGGTTGAGAGGCTGAGCCGGTGTGCTG
TCTTTGTGGGGAACTGCTACGCGGTGCGCCCAGAACGGGTGGTGCTGCGGTTCCCCAATGTGAAGGCCCTGACGCTGAAGGGGAAGCCGCACTTCGCGGA
CTTCAACCTCGTGCCGCCTGATTGGGGTGGCTACGCGGGGCCATGGATTGAGGCGGCAGCGAGGGGCTGCGTGGGTCTGGAGGAGCTGCGGATGAAGCGG
ATGGTGGTGTCGGATGAGAGCCTTGAGCTGCTAGCTCGGTCCTTCCCGAGATTCAAGGTCCTCGTCCTCATCAGTTGCGAGGGGTTTAGCACCGATGGGC
TAGCAGCTATTGCGAGTCACTGCAAGCTCCTGAGGGAGTTAGATTTGCAGGAAAATGATGTGGAGGACCGTGGGCCTAGGTGGCTCTCCTGCTTCCCTGA
TTCCTGCACCTCTCTTGTCTCCTTGAATTTTGCCTGCATCAAAGGGGAGGTGAATTCTGGTTCATTGGAGAGGCTTGTTGCTAGGTCTCCAAACTTACGC
AGCTTGAGGTTAAATCGATCTGTATCAGTAGATACACTCTCAAAGATATTAGCATGCACCCCTAATTTGGAGGACTTGGGGACAGGGAATTTGACAGATG
AGTTTCAAACTGAATCCTACTTCAGGCTGGCTAATGCTTTGGAGAAATGCAAAATGTTGAAGAGTTTATCAGGATTTTGGGATGCTTCTCCTGTTTGCGT
TCCATTTATCTATCCCCTCTGTCATCAACTAACTGGACTAAACTTGAGCTATACTCCCACTCTGGATGCTTCTGATCTCACTAAAATGATCAGCCGCTGT
GTGAAACTCCAGCGTCTTTGGGTACTGGATTGCATCTCAGATAAGGGTTTGCAAGTGGTGGCCTCCAGCTGCAAAGATCTACAAGAACTGAGGGTGTTCC
CGTCAGATTTTTATGTTGCTGGTTATTCTGCCGTGACAGAAGAGGGGCTTGTTGCAATATCGTTAGGCTGTCCAAAACTTAGCTCCCTGCTGTATTTCTG
TCACCAGATGACCAATGATGCACTAGTTACTATAGCTAAGAACTGCCCAAATTTCATAAGATTTAGACTCTGTATTCTTGAGCCAAAGAAGCCAGACGCC
ATGACAAACCAGCCGTTAGATGAAGGCTTTGGTGCAATTGTTCGTGAATGCAAGGGGCTAAGGCGACTGTCAATGTCGGGTCTTCTCACTGACAAGGTTT
TCATGTATATTGGAAAGTATGCCAAGCAACTTGAGATGCTTTCTATAGCATTTGCTGGAGATAGTGATAAGGGGATGATGCATGTTATGAATGGATGCAA
GAATCTAAGGAAGCTGGAGATTAGAGACAGTCCATTTGGTGATGTTGCGCTCTTGGGGAATGTTGCCAAGTATGAAACAATGCGATCCCTTTGGATGTCA
TCATGCAATGTCACATTAAAGGGGTGCCAAGTCCTTGCGTCTAAGATGCCGATGCTCAATGTGGAGATCATGAATGAGCTAGATGGAAGCAGTGAAATGG
AGGAAAACCATGGAGATCAATCTAAGGTGGAGAAGCTATATGTTTACCGCACAACTGCTGGAGCGAGGGATGATGCACCAAATTTTGTTAAAATCCTATA
GTTAGCTGAGAGTAGCCTGGTATCTCCTCACTCTAAAAGGTGATCTTTCTTCCAGATCTATCTTCTTTCATTTGGAAATGTCTTGTCTGTTGGACAATTT
GTCCGCTACTCTGCTAGCCCAGTGCTAGTTTGTGTCTACCGTTCAAATTTAGATATTTCATGCTTAGTAGCTCTATTGAATACTGCATCTTATACCGAAT
GCTCCGCAACATGCAAACTAGCGTCTTTAGGTTCATATAGCTCATTATGTTGGCTTAAATGTTTCATTCAGTAGCGTCTGAATTAACTCGTTTCCTATTT
GTACGTCATTATATCAGAACTGTATTATTAAGGTTTGAAATGCTGCAGGAAGAAGCTTGTGCTTTCATCATTAACTTAAACCAAGACTGATTTCTGAAAA
GATCCGAATGGGTAGTAATTGAACTGATGTTTGAACCGCCTTTATTCTGCAAAATCTTAGCTGCTCTTTTTGTTTCTTCCTTAGTACATCGATCGCTTAG
CTACTCATAGAGCTAATTTCCGTCAACCTGTGTATCATTCATATTCCATTGCGAGTATGGAGCAGCAGAGCATCTGATATGTCTAACTCTGTCTTCCCAC
TCCCTACTCGATTGCTTTTACAGATGGTCGGTGGAATCCGGCAGGCTGGGGGCCGCGTCAACAGTGGTAAGTTCTTGAGGTGTCTGTCTCTCCCTGTGCA
GGGGTCAGGAAAAGCCACACCGATGTGCACCCTTATCTTGGAGCAACTGGCTGGGATCCATCATCATCCCCTGTACCACCGCCCCCTACCCTTTTGTGTT
TGGAATTCCTTTGTGTTTGCTCACTCATATCCACTGAGGCCCTTTATCATCCAGAGCATTCTCACTTGAGAGGACCGCCTTTTTTCCGTTTATCCCACTG
TAATCTGTAGTGTACCAGTATCCATTTGCATTTGACATTGGCAGTGGACTGTTTTGCCGCTGTTGTTGTAGTGTTTGTTTCAGCCTGACGATTTGTTTCT
TGCTCCTGTGAATGCAGTGCGTGGAATAATTTGCGGTTGTTATCTGCGACATTCTATTCTCTGGTTTTGAATTCTTGGTGTTAGCCGTACCAGTTTTACG
TGCTTGTTACTGAGCAAGAAGGCACCAGTACTCGTGCATTTTAAGGGCGTGCTCAGTTTCTGATGCCATTTTTCTCGCTTACATCATCTTTCTGTATTGC
TGCCCTCAAATATGAATTCAAATTAACGGTGAAATCCAAAGCAACTTCTGAATCAATCCCG
>comp81836_c0_seq4
AGAGAGAGAGAGAGAGAGTTGAAAATTCATCAAGGCTAAAGGGAGAAGAGAGAGAGTACAGCCTTGCCATTTATACACCTCACAGCTCACCAGTTCTTTT
GCTATTATGAGTGTGATGAAGGCTGTACCCTCTCTCTTCTCCTCTTAGCTTCTTGATGCAAAGCTGCAAACACACTTTGGGAGCTCTTGCAGAAGTACCG
AATCTTGATCATCCATAAGGATCCTGTCAAACACTGACATTTTGGTTAGTTTCTTTTTCTTTAATTTCAAAGCGCTCCTAACACATGTGTTGGACTAAGT
CGTGTATATATGAAAGGCTGAACTGCATATGCTACTCTAGAATGTTGATATGTATAGTTGTTTTTGTTACTTAATTAGTATGTGCCGTTAACACTTGGCA
CTTCATGCGGTGTATCCACAAGTCTGACTTTGTCATTCCTGTCATTGCAACTGTTTGTCAACCTATAGTACTCCATAATACTTGAATTAGTTCTGTATGT
GTCACAACAAGGGACATTTTTCTTACCTGCTTAACCACTTTCCGTGCCCCCACACTATAATAAGACTACATGCCTATTAGAGTGCAATATCTAGCTAATA
TCCCTGTAGTTTGTATCTTCCTTCACAAAAGGAAACGATTTTTTGAAAATTGAATGTAAGTTAAAAGGGCCTTTTGGGCTATTTTCTTGAAACTGTAAAA
AAATAAGGTTGACGAAAATCTCAGTGCACCCGTTATTTGCTCACCACAATATCAACCCAAAAATCTCTCAGCGTGTTTCTGACGAGATAATAGTGGAGTG
TAGAGTATCTCTATTATTTGAGACACACTCATCAAACTACTACAAGTGAATGGACCTATAACATGCTAGAACGTAACAGAAGGAACTCGACCGCAAGTTA
AATTTCATGGCACCTAAGGCTAAGGGATATTTTTCCTTTGCTTGATGTAATAGAACTTCCCTTTATCTAAAAAATAGGCTTTCAATATAATGCATATATG
TGCGCTCGTGTTACAATTTCATGTTCTAATGTAGCAGAACTCTATATTGACAACTAAATGATGGATGGGATGATGAAACTGTGATTCTCGCCAATACTAA
AATATTCACAAGAACCTAAAGGCTATGTAGTCTCTGATATTATGTTTTTATATTTTTTCCAATGTAATTTTCTACTCTTTAAAAATATTTATCATATAGG
ACTCTGGCGTGCATAACTAATTTTTAGTTAAAAGAGTAAATATAATATCATCTATCAACTCTGCAAAAAAAAAAGCAAATACCATATATAAAGGTGAGAT
GAAGTCATATTATTTTTTACTTTTTATCTCAACGATCACCTAGCCGTCCATTGACTTTTTGAATGTACGTGGTAGACCTCATTTGTTGGTATATCATTCA
GACTTGAATTATGTATATTGTCTGGATTTGTCATTTTACTAAGAACCAGCACAAAAAAGAATTATGTTAAGTGAACTTACAGGACATTGAAACAACAGGG
ACAGATGCAGAATAAATATTCATTGATGTCACGCTGAGCAATTTTTTTAGATAAAGGAAAAAGTTCCGGTCTTATGCATCAAACAATGCATACGGCCTGT
```

GTTCATTATTACAAAGTCACAACATTTCATTGGAAACTTAAGATTAGAATAATAAACTAACAAGACTGTAAAATTAATTGTATAACCTATGATTAGATCA
CTACCCATTCTTGACGAACACCTCTATTGCCACCACTTCAATGGGCTGGCATGCTTGCATGACATCCTCTCTTTCTGCTTCCTTCTGCAACAATCTCCAG
AACCTTAGATAGTTTGTGCCCCTGAAAATTGCCTATAAAACTGATAGTGAAGATTTCTTATTAAATATGATATCAATTACGGCATAACCATATAGCTCAA
AATAAAGCTGCTACCCCTACCAAGACCTTTTTCTTTTGTGATACTCCCATATTGCTAAGCCAACCACTCATAATATGAGTGACATTTCTAGGTATAACAA
TGCCTAGGGCAATCCTAATTATTCGCCAAATGGTTTTTGCAAAGAAACATTAAAAAAAGATGTTGTATCGTTTCATTATGGTTACAAAAACAACATTTTA
GGCTTCTGGTCCAATTCCTTTTAGCCAAATTATCTTTGGTTAGAATGACATCTATACCTAAATACCACAGGAAGATCCTGATTTTGAGAGGGAGTTTAAA
TTTCCATAACTAATTTGTTGGGAAAGAGGAGGGTTGGTTTATCATTCGCTTGTACATAGAATGGACAGAGAATTGTCCATTGGCATACAATCCCCACCTA
AAGAAATCACGTCCATCATTCAAATTAATATGGACTAAATGTGCAATAAGATCATTCCATGCATTCAGTTTGTCCCCAATAATTGGTCTACGGAAGGACA
GGTTAAGAGATACTGTTGCCATTACATCCACCACAGTTGCATTCTTCCTTCTCACCACATCATATAGAGTAGAATACTAGGTGGTTAATGGCCTATCATT
CAGCCAAACACTGTGCCAAAATGTGGTTTGGCTGCCATCTTTAACTTGGAAGGAACCATATTGTAAGAAGGTAGTTTTAACTTTTATTAGACCAGCCCAG
AATTGAGAATCACCTGATTTGACTTCTACTTGAGCGATGGATTTTCCTTGGAGGTATTTGTTTCTGAGCAACCTTTGCCAAACCCCATCCTCATTAAGCA
ATTTAAATAATCATTTAAATCACGCTAAGCATGTGTTCATTCTTTGCACTAAGATTAGTTGGAACAAGCAAGAACTTCACTTTGACATGCAAATATCCAG
TGGTGTCACTCGACACCATATCTTACAATGTAGATCCATCCCTAGGAACAACAATAGTCAATTCAGATATTAGTTTCCTTGTATGGACCGTTACTGGTGA
GTACGATTTATCCCACTAGAATGTGCAAGTAGATCAGAAATATGTAAATAATTCTTAGTTCAAATTGCAAAAGAGTGGGATGCAAATAGATATTGAGCTC
ATAACATTAGAGAACATCATGAATTCACTGAATGACAAACATATTGCTATTTGGCATAAGAAGACTGAACTTATATAATTGCAATTTTTCGCCACCATTT
CAAAATACTTAAATTTTTTTCCTGTGAGGAATCTAAAACACTTGCTGATGAGACATACATGCTAATATCTTCTTTTCTGTTTTCTCTTAACATGTTGTTG
TACATGGCTGGACCTTTCGTGCAACGCCACTTTTTGTTTGACTCCTTTTTTAACCATGAATTTGTACCGCATTCAGTTTATGACACAACTGACTGCACTG
TTAATTGAATGACTCAAGAATCACTATGCCGCAACAAATGCCTTCACTGGCCTGTTATATCAGTCTCCATGATTAGTATTTCTACTAACTAATTCCCTTT
TTGAGAAATCGATCGGAACCAATGTAACGTCGCGTAGACTGATCATTTGATGGAAACAACATATTGGAAAGTGTACCATAAACATCTTTCTTACTACACT
ACACCAAAACAGGTCATTGGATGCGCCTTATTAGTGTGGTTGCACGAAAAGTGGTACTGATAACATGGTGGCTTATAACTTTCTCGCGCTCAATCAATGA
TTGAGCAGTAAGAACCGTTACTGATAATCAGTATCAATAGCGGCTAGTAAAATCCAACCAGCACTGATAATCTTTATCAATAGCGGTTCCTTTTGATGAC
TAAGAACCGCTATTAATATTTTTTATCAGTGCTGGTCCTTGTTACGAGCCGACTGTGATAATGGATGATATCATATCTTCAAATAATTATAATTTTTTCA
TACGGTGTCAGATGGATACAAATTTTATATAAAAATTATAGCTCTAAAAGAGATCTACAATTTTAAAGTTGATAACTTTTTATTTGAAGTCATATAGATA
TCCAAACTTGAGTTATAAACAATTTAACACATGTAACTTATAAGACTCGAGTTATTTTGTTATGGTTCACTCATGAACTTCATAAAAATGGATAAAACTA
GTAATAAACATTGAAATAATAATATATGCAATGGTGAATTGATAAGACATACAATGCTAAAATTTGTTCATGTTGAAGCGGGAAAAAGATGTGAGTCTGC
ATATATTTTTAGTTGGATTTTCAAAAAACCATAAAATATTCATATATTTATAGATAAAAAACTATTTATATAAAAATTATAGCTCTCGACGAGATCTATA
ATTTTGTAATTGATAACCATTTTATTTAAAGTCATTTACATAGTCAAATGTTTATACAAAGTATTCAGCAAGAAGGATAAAAAGATATAAGTATCGCATT
GTGATCACAAAGAGTTGATAGCTGATATGGGAAAGAAGCTACGCGTGAGGGGGGAGGTCACAAGTTCAAATCCCATGAACGATACTTGTGCAAAAAATTA
TGTAACTTGCAAGGTGGGACAATGCACGCGTGTGGTGGCTCGGGTGGCTTGTTTGGTGAATTGTTTTCATTTTTCAGCCCCACAAAGTTCAATTTTGAAC
CAACTATCAGTAGCAGTTCTTAATAAGGACTGCCACTATTAAGTGTATCAATAGCGGTTGTATAACCGCCATTGATAATCATCGATTATAAGCATCGAGT
AATTAGTGGGGGTTCAAAAATAGGCACTGATAGAATTTTTGAGCCGCCACTGATGACCCTTTTTCTAGTAGTTCATGTAGCATTAATGTGCAGCAAATTG
CACTTACATTTGCACCGCATGTCAATACCACAGATCCCTACAGTAGCAATGACGTCATTAGTGATGGGCTTTTCCGACCCATTGCTATTGGGTCACCAGC
GACGGTCGTTGTGAAGGCTGTCGCCAACGTTGTGGCCAGCACGAATCTGTAGCAATGACGTCCTAATAGTGGCTGTCGCCATTAAATGGACAGCGCAATG
GCGTCGGGCGTTAGGTAACGTACATCGCCATTGATCCGTCATAAAGGCGATGTACTTCCCGTTGACGAGCGTCGCCAAAGATGGGACAACAACCAGCGAC
GATTTTTTCACAGCCTCCATCGCCAAAGAACGGCCTTCACACCTCTTCGCATTCCCATGTGCCTCCTCACCTTCCCATAGTGCAATAACTACCCACACTT
TGCGAGGTGCTGCGATGTGGCATATCAACTGCTCCTCCAAATGCTCCATATAATTAACTGGTGTGGGACGAAACTTCAACCACACACTACATAGAAAACC
CCAAACGCCTCCTTAGTCCTCATCACTCCTTCATCATCCTCACGCTTCGATCTCTACTAGGTCCCTCAGTTTTCAGGAACGACGACGACATGGTTGAGGC
AACGACAAGAGGAAGAGGTCACTGGAGGACGAGATTCGGGTGAGCAAGTGTCCCAAGAAGCGTCGGTAGGAGTTGGAGGAGGAGATCTAGGCCCTGCGGG
AGTCCCTTCGTGCGACCGAGGAGGAGCATGCCATGGCCCTCATCGCGGTTTAGGAGTTGCATGCCACGGTCCTCAAGATGACCATGGAGGAGCAAGTGTG
GCCCTCCACGCCACTGAGGAGGAGTGTGCCATAGCGTGCTGAGCTTCGATGAGACATCTGCGACGTGTTAGGGGCCATGGGCTATAACCTTGATAGGGTT
TTCGACTCTATCAAGGTTTGAAACCCAGGCATCCAGGGAAAGGCCAGGAGAAATGGCCTGGAGGTCTTTAGCAAGGACTTCCTCACCAAGGACCTCATGG
TGAGGGTCTGTTGTCAGGTCATGGCTCCACGCGTCCTGCTCGGCGCTGACGTCGACTTGCCTCTATCGTCCTCTCTCGGGTAAAGGTCCGCTATCCCACC
GTCGGCCTCGTCCTGTCCACAGAGCCAAACCCCAACGAGGATGACGCCATCTGCGAGGCCTGCTGCATCGTCGACAAGGTCGTGCATCATCTGGGCTTCG
GTGACAAGGGTGTTGCGGGAAGCTCCGACTCTGACAATGCAGCCTCTGACAGTTTCGGCTCTGGGGGCATGGCCTCTAGCAGTTTTGGCTCTAGTGGCTG
CTCAGCCTAATCCATCGGTGGTGAGGACGGCAACAATGAGCAGAAAGACAGTAGCCACGACGAGGAGGCCCTTTAATTTATTTTCTTTATGATTACTATG
CCCTTTTTTGTTTTGTAAATATTAAAAACTACTCTATTAGTGGTAATAACCGCCTAATATTATTATCTGGTCGCCTAAATTTATTATCTCCTCTATTTCT
TATTTTTTTTGCCGACTAATGATCGCTAGTGCGCTATTAAGATGAGCCACTAGCACGAATCTCAAAATTTGAACAGTTATCACTCCCAATTCTAGCGCTT
CACTTGCCCAGTGTCGCTTACATACAGGCCCCAGTATTCCGGCTCCACTGCTCCAGCCCTACATCCAGTGAGACGAGGTTAAAGAGAAGGATGTGAGGAG
AGCTTAGGGTTTAGAGTGCGCAAAAATGATCCCGCTCGAGTGTTTAGAGTGAGCACAAGTCCCTGCAGGGAGCTTAGGGTTTCATGCTCAAGTCGCCACC
ACTCGAGTCCCTCACTTGGGGTTTGTAGCTCCTGCGCCACCGCTGGATCCCATCACCATCCTTTGTTGAGTGCCATGCATCGCTGTTGTGCTTCCCCATC
GACGCCACCGAGCTTCCCCATCAACACCGCCGTGCTTCCCCATCGACGCCGACACCACCGACTCCACAGAATAGGGAGAAAGAGGGATGTTGCCTTCCCG
ACCGTGATGTGGAACTTGATGCCTCCTCCGCGAACGACACACCGTCGTCCTCGGCTTAATTCCCTATCGATGTCCTCCTCTTCATCCTTGTCGTCTCCAG
GCATCCGTGCTAGCCTCGCATCCTGGACTGGGTAGTTTCCTTTCTTGCCGAGTTTCTTGATGCTAGAATTATTGTGCCGCAATCTAGTGTTACCACTTCT
TACCCTT
>Locus_4567_Transcript_44/65_Confidence_0.379_Length_2535
CCAAGTGAATTATCTCACCTATAAATGAACAGTTGTTCCGAACTCTAACCGTGTTAAAACTGGAAATCCAAAAACTGGATGCATGTCTTAAAATCTAAAA
GCGGGATGCATGTCTGTAATAACATTCCATGAAGCAATTTCAAAGCTTAAACCCTCTTAATCATAGAAGCCAAAATCTATTTTCCAGGGATTTCACCAGT
TAACAGACCAGAGCAGAACTGTGCTCTAATTTATTGGTCACCTGTACTTGCGTTTTTTAATGCACCAAGGTTCCTTTTTAGAGACTTGACAGCAGACAAA
ATCTGCAGCTTCTCTTTGGATGCCCCATCAGGTTTCTGATCATCGTTCTTACTGGCTGCCTCATCAGGTGGCAACCTACCTCCCGCAGACGATCCCTCCA
CAGGAAGTTCCCTGTCTGCATTGCTGGCATGTGCCCCCTGCTTCCCAGCTTGCTTTCGCATGAAGGGCGCACTCCTCTTGTACTGGGGATCAGTAGGATC
CAATGCGAACAAATGAGATGTGAACATTTCTGAGAACCTCTCATCTTTGCTAAGATCAATATCAGGAAGTTTGTCCTCGACAGATTCCTCTTTACCCTTC
TTACCCTTGTTACCCTTTTTGTGTTTCAAGTTATAGCCCTTTGCACCATTGGCAGAATCCTGGTCAGCAGCAACCAAGAGCTCCAACTCTTCTTTCGTTG
CTTCTGGCTCCAAATGTTCCTCTGGTGATTTGTCCTTTCCTTTCCCCTTTGCTTTATCTTTAGTCTTCGCCTTTTGTTTCTTGATTTTAATTGGCTTAGT
TTCTTCATCAGACAGTTCCTCAGCGAAGAAATCATCATCGTCATCGGGCTCATCTTCGCTGCTATAGTCATCGTCATCATCCTTCAACCCCCTCTTCGTA
GCCTTCCTTTTCTCTTTCATTTTCTCTTGGTGCCTCTCCCAGACAGTTTTCTCCTCATTGGTCTTTCTCTCTAGGATACGTTTACTGAGATCCTCAAGCT
CTGTGTTGAATGTAATCTCCATATCCTGGCCACCAGCTTGTTCCTCTTCAGATTTGTCGCCTTCCAGTAAAAGAGCCAACCTCTCCTCTTTTGTTAGCTT
TCGTTTGGATCCCCCATTTGGTAGAGACTCGTCATCAGAATTGTCGACACCGTCATCATCTGACGCACTGTCATCACTTGCTAAATACATGTCAAGCTCA
TCTAACTGATCGTCAGTGAACTTTCGCCTCAAGACCTTCTTACGTTCTGGTTCATCATCATCCCAAGTTAGCTTAACTCTGCTATGTTGCAGAGCACGAG
TCTCAAAATCAGGCTCCTTGTAACTCGGAGGTGCCTCTGTAGCAATGTCTCGAGCAGGATGTTTGAACTCCATCGAATCAGGTATAAACTGCAAATCAAA
TACATTTGCTGTTTTCAAGAACTCAGTACCATCAAGATTCATGTACAGGTGATTTGCAGTTGCACTAGAATCACACACCACAACTGCATAGTAATACCTC
AGTCTGTTTAGCTCGTAAGTACGCAGCTTGTTATTCTCTTTGTCAGAGTCAACTTCTTCTTCGTCGTCATCAGTGTTGAATTCATCATCATCAGTGTCGT
CTTCACCATCGTCGTTATCATCATCATCGTTGTCCTCATCATTATCATCAGTATTGGCATTAACAAGAGCTGCTGGGCCCTGAGTTGATTCAATTTCCAT
GCACTTCAGTCCAAATTCTGATGGATATATTGAGACTGATAAAACCCGCCCACCTTTTGGGACGCAAGATGTCATCACCATATACAGGTCAACTGCCTTG
ATATGATCCCAGTCCATATTAACAGCAGCAAGCCTGTGGGTTTCCTTGTCAATCATAGGTGTATCATCGTGCCGGCCCATTAAGTAATGAGCAATGTCAC
TGCCGACAGAGTACTCGTCATCATCAACGTCTTCATCCTCGTCGTCGTCGCTACTAGAACTCTCTTCTTCCTCCTGCTCATCCTCTTCATCCACGTCCTC
GTCCTCCTCTTCTTCAACTAACTTCTCTTTCTCCTTCTTTGCCTTCTCTTTAGTCTCATCCTCCTCCTCGTCTTGGTTGAGATAGTAACGGAGCATGGGG
TTCTCCCTGCCGCTCTTCTTCCGTCGCCTGCCGCGCTTGTCCACCGGCGCCGCCGAGGAGGAGAACCTCGGGTCCGTCATCATGCTGCTGAACCTCGCGT
CCAGCGCCACCTTGGCCTCCTTCCGCCGCATCGGCCGGAACCTCGGGTCCGTCCGCGCCGCCGCGAACCGCTCGTCCTCCATTGCCCGCCTCATCTTCTC

GTCCCTCCCCTCCACCTCCTCCGCTCTCACCGTCTTGCCCTCAGGCTTCCCCTCCTTCTTGGACTTCTTGCCGTCCCGCTTCTCCTCGCGCCCCTCCTTG
TGCTTCCGCTTCTTGCGCTCGGCCTCGGGGCGAGGGGCTTCCTCGGTGGCCGCCTGACGCTCTTGATTCTTCTTCTTGTGCTTCCTCTCTTCCTTATCGG
ACCTCGATTTCTTGGCCTTCTTGAGGCGCGCGGGC
>Locus_35233_Transcript_13/21_Confidence_0.400_Length_984
TATCTGACAATTTATTTAATATTTTGTAAAGAAAAACTTTGACACTTGATTTTCAAAGAGTTTACAAAAGTTTTCAAAACTTAGCTGGTTTCAGTCCTAA
CATGATCTGTACCCTTCAGTTACATCCTTATCAGCTAGTATTACCTAATCAACATTGCTTTGAAATTCAGGAACACGATTCTCACCATTTACAACTCCGA
GACCGGATGCGAATTGGCCAGGGGTAATCAAAACTGAGGAGAGTCCATACTACACTCATCAGCTTCCTTTGGGCGCCAGCAACAGGCAGCATTTCGTCGG
CTCCACGTCTGCCTACACTAAAGACGGCCGACGCTTCCCTTTCCTACAGGAGGGCGAGATAAGTTTCGCCACCGGCGTCGTGCTGGAGGCCTCAGTGTGC
CAGCCACTCCTTAAGACGGTGGCAGCTCCTCCCGAGAGCGGCGGCGGCGGCAAGATGTTCTCTGATGGGTTGACTCGAGTGCTCGACTCGGATTGTGCTC
TCTCTCTTCTGTCAGCTCCGGCGAACTCCTCGGGCATCGACGTCAGCCGGATGGTCCGGCCCACTGAGCAGGTCCCCATGGCTCAACCTCTGGTCTCCGG
CCTGCAGTTCGGCAGCTCGTCGTGGTTCTCGCGTCCTCAGGCTTCCACCGGCATCGCCATCTCGACGACGGCCGGCTTCTCCTCCTTCCCGGTGGTGGGG
AGCGAGCAGCTGAACGCCGTGCTGAGCTCGAACGACAACGAGATGAACTACAACGGGATGTTCCATGTCGGCGGCGAGGGCTCCTCGTCGCTGCCCTTCT
CGTGGCAGTAGTTCGCAGTAGCTGTTCTTGCGTGCTTTCGACTGGCAGAGCTGTTCGATTTAGTGGATCAACATTGTTTCTGTTCTTTCTACATTCCTTT
TTCTTGTATTGATTTCTACTCGCCAGCATTCGTGTGGACAACTTCAGCATAAATTATTGCCAAATTTCCATGAAATTTTCTTTT
>comp82180_c0_seq27
AATATAGGGGGCCCCGCTCCCGCAGCTCGCAGTACCGCGGCGTCACCTTCTACCGCCGCACCGGCCGCTGGGAGTCCCATATTTGGGATTGCGGCAAGCA
AGTGTACTTAGGTGGATTTGACACTGCTCATACTGCTGCAAGGGCGTACGATCGAGCTGCCATCAAGTTCCGTGGCTTCGACGCCGACATAAACTTCAAT
CTCAGTGACTATGAGGATGACATGAAGCAGATGAAGAGCCTGTCCAAGGAGGAGTTCGTGCACGTCCTGCGACGGCAGAGCACCGGGTTCTCGCGTGGCA
GCTCCAAGTACAGAGGCGTCACCCTGCACAAGTGCGGCCGGTGGGAGGCTCGCATGGGACAGTTCCTCGGCAAGAAGTACATATATCTTGGGCTATTCGA
CAGCGAAGTAGAGGCTGCAAGGGCTTATGATAAGGCCGCGATCAAATGCAATGGTCGAGAAGCTGTGACGAACTTCGAGCCAAGCACGTATGATGGGGAG
CTGCTGACTGAAGTTGCTGATGAAGGTGCTGATGTTGATCTGAACTTGAGCATATCTCAACCAGCTTCTCAGAGCCCGAAAAGGGACAAGAACTCCCTTG
GTCTGCAACTCCACCATGGATCGTTTGAAGGCTACGAATTGAAAAGAACAAAGATTGATGCTCCCTCTGAACTGGCGGGCCGCCCTCATCGATTCCCTCT
TCTGACCGAGCATCCACCAATCTGGACTGCCCAGTCTCACCCCTTCTATTCAAATGATGAGAATGCATCAAGAGATCTTAACAGGAGGCCAGATCAGGGG
GGTGTTCCCAGCTGGGCGTGGAAGGTGACCGCCCCTCCTCCCACGCAGCCATTGCCGCTGTTCTCGTCGGCGTCATCATCCGCTGCAGCATCATCAGGAT
TCTCCAATACCGCCACGACAGCTGCCCACACCGCCCCATCGGCCTCCCTCCGGTTCGACCCGCCGCCGCCGCCGTCCAGCCGCCACCGCTGAACGTTAAG
AAGCCACGCTGTAAATTTGCCAGGAAGCCGACATTTTTTCCTCTCTCGGCGTTGCAACTTTTTAGGTTTTGCGCCGGGGTGGTTTCTTGTAGTGGAGTGG
ATTCATAATTAACTGTATTTGCATGCTGCCCATGTGAAATGCTTCTCTCTCTTTTTACGCTCTCTGCACCGGATGTTCTGGGAGTTCTTGTTGCCGGGAA
TTAACTAAATTCACCGTCTGAGATTGATCTATACATTGTTGTAGAGAATCGAATCATTGTTGAAATTAGTTACAGAATTATAGATATCATAAGGCACCCT
CTACTTGTGGACAAAGTTCAGGAAGATTATTCCGGTTCTTTTCTTGGCTGTCAATTCGGCTCTTGTCCTTGGTGTGGAGCCACTTGCCTACTGTCTTGTT
CTGTTGCTGTTTGTTCTTGGCTTCTTGCACAGTTACAAATTTGTTGGCTCGAATGGATGGTGAGACATTTGGTGCAA
>Locus_14087_Transcript_49/66_Confidence_0.358_Length_2484
GATTCAAAATTGTAGCAGATTTGTCCTCTACCCACCCCTCTGAATCCTTTTTATACATACACTGACCATGGCACCAATGATCAAACCAAACACCAGCTGC
AAAGATCACCCTGGATTCCTGGAACAAAGAGGGCCTAGAGCAACGCAGCAACCTTCGCCACTTGAAGAGGATAGGTACAATGTGGCTTAGCTAGCACCTT
GCAGTCAGGTAGAATTATTCAACCGGATAAAGATTATTTCAAGTAAAGGAGGGCTACCAGTTTATACCTGTTCAGGTGGGTGCAGCAGTTGAGAACTTTT
TTTCGATACAGAGCAGTTGAGATCTTTAATTCTGGCATGGGTTCCTTTGGGGTGAACTGGAATCAGAAGAACTCCGCGGTGTGCGATTGGGAAAATTTAG
CACCTTCTGTTCCAAATGCAATCGGAAACCCCAGGAACGGAGCTACGAGGCATGGATCTGTGAATTCATCTGGTGGCACTCTCACTTCTAGCTCAGAGCT
AGGGCATGGTTCATCCAAGAGCTCCATGTCGGCATCCATTGATTCACCGTCCAAAGTAGGAAACAGCTTGGAGTTCAACTTTGCTGCTGTCAAAGGCCAT
GTTAAGAACATGGATAAGGATTTTAGATTTGACGACTTGGGGACTTCTCCATCGTCGATGATAGCATTCAGCCATGGAGAGCCATTAATCAGCCTTAAGC
TTGGAAAAAGGACTTACTTTGAAAATGTCTGCGGAGGGCAGGATTCCAAGAGCACTGCACCTTCTACCAGGACTTCTCCATCAACCGTTCTCAAGAAGAC
CAAGGTGTCTCAGCAGAATACACAAATGTCATACTGTCAGGTTGAAGGATGCGGAATTGATCTGTCCTCCGCTAAAGATTACCACCGCAAGCACAAAGTC
TGTGAAGCTCATTCTAAAGCCCCGAAGGTTGTTGTTGCTGGTGTAGAGCGCCGTTTTTGCCAGCAGTGTAGCCGGTGATATTTCCTTGACTGAGTGCATT
TTCATTATATAGTATGCCTCTTTGTATTCACCGAATCCTTTTTTAATGTTGGCATCTCTAATTGAAAAGAGAGAACATAAAAAATGTGATCAGTTGAAAG
CAATACAACTATTTAGTCGTAGAACGACCAAGGACCAACTGCTAGATGATGCCCAATTATTTTGCATGACTGAGACTTCCAAAGTATCTGTGATGTTTAT
TACTCCTTTGCTGATCAAGGTACACAAGAACACTGGAGTGACATGAACACTGAAGAAAATTGGCATTTCTTTTTGTTTCTGGCACTAGTTTGTTATTGAT
TTGTAAGCGGAATGCTGATAAACATCTTGAAAAGTGAAATTCAGATAAACTAATTACAATACAGTTTGATCTTTTAGCGACATAATGGACATTTATTTCC
TTTTCTTTAATTCTTCATTGGTTTGTAGGTTTCATGGTTTAGCTGAGTTTGACCAGAACAAACGAAGCTGCCGTAGGCGTCTTACTCATCATAATGCACG
GAGGAGAAAACCACAGGCAGATACAATTTCATTCAGTTCATCGCGGCTCTCGACAATGTTTTATGATACAAACCAGCAGAAAAATCTTTTCTGTAGTCAA
CCTCATTTTGGCCAAGCGAGAAGCAATGCAGTTTTTTCTTGGGAAAACTCGGGAGATTCCAAATTTATGGAAACCAAACATCTGTTGATGCAGCCAACGA
AAATGTTAGGTCTTGATGGGCTGCATTTCTCAACCCCCCAGATATCAAATAGTGTTGTGGCTCACTCTGTACATCATCGTAATTTCGATGGGCTCATGCC
ATTCAAGGGAACCAACACAAAGGTCCTCAACCAAGGCGTGGAAGCTTCTGCGATCGCTTCCAACTCGAGTGGAGACCCGGATCTTGGGTGTGCTCTCTCT
CTTCTGTCAAACGGTTCGTGGGGTTCAACTTCAGCCGTCATCCAACAGCCTAGTTCTCATGCGCAAGCTGGTGCGGTGCCACCCTTCGCCACCGTCGCCG
TCTCCAACCCTGCAATGCATCCTCTGGACTCATCCCCGGGAGGATTCTGGCAAGACGTCCCTCCCCCGCTCGATGAAACTCCACATATTCAGGCATTCAC
GCACCTCTGATCAAATGCTGCATCCCCTGGGAACAGCAGGTGGTTATACCTTCCGTAATCGTCCTAGGTTTGCTTTGCCAGTGACTGGCTCTGCACAATG
TCGATCCATCACCGGAGGCATTTGGTAAGGTGAAGACTAAAGACTCAAGAATAACCGGAGACTAACTCGCTTCTCTTCCAGGCTCAAGACTGTTTGGGTC
CAGCGGTGTTGCTACCTGATCCTGTTTTCGGTTCTGAGTACATCCATTACTAATCATGGGATTACTTGTTTAGGCCAACCAGACATATTCTGGGGTGAGA
TTACGACATGTTAATTTACATTGACGGTGTTTCTACATGTGTTAGTACTAATGATGTTATCGCTCTTGGCCCCGTTACGTTTCA
>Locus_17579_Transcript_12/92_Confidence_0.088_Length_452
CGCCTCAATGATCACAATGCCCGCAGACGGAAGCCACAGCCTGAAGCAATTCCTTTCGGTTCATCAAGGCTCTCTGCAATGTTTTATGATGCAAGGCAAC
AGACAAGTCTTCTGCTTGGTCAAGCTCCTTATGGTCACATGAGAAGCTGTGCAAGTTCTTCATGGGATAACCCAGGAGGCTTCAAATTTACAGAAGCAAA
AGCTCCTTGGTTAAAACCAACGAGAGCTGCAGGTGTTGATGGGATGCATATTTCAAGCCAGCAGGTGTGGAACAACATTACGCCACATGGTGCACATCAT
GATTTTAATGATATCATGGCTTTCAAGGGAACCAGTGGAAAGGCCCTTAATGAAGGCGTCGAAGCTTCTGCGGTCGTCTCCAACACGAACGGAGCCCCGG
ATCTTCAGCGTGCTCTCTCTCTTCTGTCAAACAATTCAGCTGGTGCTGCCAA
>Locus_5523_Transcript_81/107_Confidence_0.174_Length_1782
AAGGCAACAAAGAGAATGCAGGGAGATTGTTACATACTGCAGTGTACAACTTGGTTCAAATCGTTCCAAGTTTTACATGCACTTACTGCTAAGAAATGTA
TAGCATGATTGATGAATCAACAACACAGAACTACATTACAAAGCTTAAACAAAAGGAAAAGTTCACCTTGGATCATACTGTAACATCAATCTGCTCCCAC
GTTGCCATAAGGGTCAGCGAGGAGCATGAATGAGATTAGGATGAAACCTGGGGCAAGTCAGTTTCTGCGGAACCGCAGCACAGTCGCTGCGCGTCTATTT
CAAGCATCAGATACAGCAGCCACAAGAACTAGGAGTTTTGGTCGTTGGCAATCTCCTTTTCTTTCTCTTTCTCTTCACCGGATCCATCTCCGGTCATTCC
ATCCAGGAAGCGGACGATGACAACTGTGATGTTATCAGAACTCCCCCTTTGGTAAGCTTCTTGGAGAAGTTTCTCTGCTGCTTGCTGGGGCTGGTCCTGT
ATGGGCTTCACCATGGCAACAGCTTCCTCATTAGTGACAACATCCCAGAGTCCATCACTAGCAAGGATGAGGAATTCAAGGGAGCTATCCACCACCTCCT
CCTTGATTTCTGGGTCAGCGACAACATACTGCTTCAAGAGTTTATCACCAAATGCTCGAGAAACAGCAAGAACACCACCCACACGCCATGTCCCAGCCCA
CATCACAAAGCCCCCAGCATCCTCAATTCGTTGTCTCTCATCAGACTGATCAGGTTTGTGATCTCTTGAAACCGCAATAGCCTCCCCTCCTTTACAAACA
ACAGCTCTAGAGTCTCCAACATTTGCAACAAGCAAACGATCACCCACAATGATAGCTGTTGAGGCAGTTGAGCCAGCATCTCGAGTTTGACTACTTTCGG
CTTTAAGAAATTCTGAATCTGTGTGGTTGTATGTTTCCGCGATAGCAGACTTGGTATCACTGATGAACTTTGGATGTTTTATTAGGTTGCTGAATAGGTG
CTGCTTGACATACTCTGCTGCTCGTGCTCCACCATGACCATCATATACGCCGAACAATCCAACTTTCTCTCCATCAATTTCATCTATCCTTGCCTCATAA
AAATCCTCCATTGATGCTCTTTTCCCAGGAGAACTTGCAAATCCATAACTGAACCGGCCATTTACACTGCGCTGGCCTCCGCTGTCCGCCAACCCGGTCC
CGACGAGCCCCATGCGCGCCCCTTCCGCGTCCGCCAGGCACCACCAACAGGACGCCCGCCCCGCGAGCAGGAGGAAGAGCAGCCAGAAGACGAGCAGCGA
GGACGGGAGGCGCACGGCAGGTGGTGGCCCCGACGACCTCTCGCGCATGAAAAGAGGGAGGGAGGGAGGAAGAAAGGGGAGGAATGGATGGACGAGCTGC
GATTGCAAGGGGAGAGAGAGAGTAGAGAGAGATCTGTGCTACCTGTGGGGGAGAAGATGGGAAGAGGGAAGATAGTGATCAAGAGGATTGACAACACGAC

AAGCCGGCAGGTGACGTTCTCCAAGCGCCGGAGCGGGTTGTTCAAGAAGGCCAAGGAGCTCGCCATCCTTTGCGACGCCGAGATCGGACTCATCGTCTTC
TCCAGCACCGGTCGCCTCTATGACTTCTCCAACACCAGCATGAAGTCTGTGATCGAAAGATACAATGAAGCAAAAGAGGATCATCACCTGACCATGAGCG
CAAGTGCGGAGGCTAAGTTTTGGCAAAGGGAGGCAGGAAGCTTGAGGCAGCAACTGCATAACTTGCAAAGAGCACCATCGGT
>comp73418_c0_seq1
AGGAAGGGACGGCGGGGAGGGGGCAGGCACAGAGAGCAGCTTTGCTTGGCGGACGCACCGAGGGAGGCGCCTGGGAGCCCTGCTTCTCCCCTCTTCGTCT
TCCTCCTCCGCTTCCAAAGGTTACCGTCTCCCGCCGCCATAAGACTAGTAGCCTTCTTGCTGTGTCAATCCGTCATGCACGCGTCCCTGGATTGCGCTGG
TGACTCCTTCGGGAAAATGGTTAATGATCATACGAGGTCAAATTTGGTTTTTGATAATAAGCAATCTCTATTTGCAAGTCATAACATTGACTACGGCCAG
CCAATAGTAAGACTCATGGTGATATAAAACATCAGTTTTTTCAACTCTGTTAAAGTCACCATTCTAGTTTTAAAAAACAATAAAAAGCTCATCCTATTCT
TTACATGGGGATGTGCATCTGATTTTATTTTTCTACCATATTACCCAGGCTTGTATATCATACCCATACAATGATTCGGGCTCAGGAGGTGTTTGGGCAG
CCTTTGGGTCACGCACTAGCGCTGCAGCTGTGTTCCATCCCCAAATTGCTTGTGGGGGCACATCTGCGAGAGTTCCCTTACCTCTGGAATTAGCAGATGA
TGAGCCCATATATGTCAATCCCAAACAATATCATGGTATACTTCGCAGAAGACAGCTACGTGCTAAGTTAGAGGCTCAGAACAAGCTAGTCAAAAACCGA
AAGCCTTACCTTCATGAGTCTCGGCATCTTCATGCAATGAAGAGGGCAAGAGGTTCTGGCGGACGTTTCCTTAATACTAAACAGCTCCAGGAGCAGCAGC
AGCTGAAGTCTCGCAATGCCTCCACCAGGTCCACCACAAATGGCGCAAATTCCTCAGGTTCAACACATCTACGGCTTGGTGGTGGTGCAGATGGAGATCA
AACCATGTCGGGGACGAAAACAATGGCCTCACAAAACAATAGCAAAAAGGCTGTTTCTTCTTCTGCAGCTCTTGCTTTCACCGTGACTCCTATGGTGCGC
AAAGATGACACCTTCTTCCAGCACCTCAGCCACAATGTCAGCTTCTCCAGCCATTTTGGCCAGGCAAGCGCCCAAACCGGCGTTGGAGGCATCCATAATG
AGACCCAGCATAGGGTTTCCGTGATACAATGACGGTTTGCGAAGCTTATCTGGTGATCCAGGCTTCTAGGTGTCCTCGTGTCCGGTGTAGTCTTGGTCGC
TCAGGCAATTCATCCTTGGCTTAGTTTTTGGTGTGTTAGAACCTACAAATGTTTCATACTCTGTTGTGGTTTGCAGAGCCCATAAATCAGGGCTCTCCTA
AAAAAAATCAGGGCTTGATGGCGACACTACTATCCAAGTATTGTTGTAATGGTGTGTGTTAGAAACTCGCAAAAAACCCTTCTTTGTGCTCGTATTAGTA
CATTATGCTGCACAATTAGCTGCTATTCTGTTCGTACTACAGTCTGGT
>Locus_5523_Transcript_44/107_Confidence_0.093_Length_1396
TCTCCTGTCTTTCAATTCTGATCTAAGGAGCTTTAACAAATAAAAAGGATCATAACCATCATTCTAACAAAATGGGTCCAAGATAAGTGATAACATATCA
GATCATAACCATCGTTCCAACAACATGGGTCCATGATGAGTGATAATATATCATCAGTCATCACTCTTCTTTGCATTCTACATTTCCACACAAACTATTG
AAAAGTAACCATGCCCAGATCATCAATAGGTGACTTTAATGCTACATAATGAAGCAGCCGCTGTAAATTATCACCAGCTATATTCATAAATTCTGTTAAC
CCAAAACTGTTACAAACACTTTTTATGTCCCAACGACCATTCATCCTCCATAACTTGTGTTGGAGTCTTGGAGACCCATATTACTAACTGCAGAATCTTG
ACAAGACATCAGGAAGGTGATTATAAATCCACCAGATTCAAGTGATAACATTAACCCGCAAGCACCAAGCTACAACAACTTTTTTACATTTGTTTTCAAA
ATAGTATAAAATGGTGTAAGTTTACAAAACAACCAAGAAAAATAGTTTGAAAAGGTGTGAAATATTTGTAACTTAGTGCAATATGTATCATGCAGTATTT
AAACCTACTGGCCACTGACTACCTCCAATATGTTAAGTGCATTTACTCAAGCACAAATATTATGGCTAATTTGTTCAAGTTCGTTCTATATTATAAGTAA
CATGTTCAGGATACATCAATCCACCATTCAAGTTCTGCTAAATGATAACATTTTCTGATGTTTATGTATAACAGGAGAAGAATATCGAAGTGAACTTAAG
TTACATAAAGAAAAGAGTAATCGCAACATGTTTAAAGGGACAATGAATATCCACATCAAGTTATCTTGAGAGGTAAAAATTTCAGTTAAGTAAACAAGGA
TGACAAACAAAGTAATAACTACCAACTTCAAGATTTGATTGTGTTGCTGCATGAACAGTCAAACAATATCCTTTGACTAATCAGTCTTAAACTATTAAGC
ATTTTTCAAACCAAAGATATCCTCGTGTGTAAGTTGCTATCTCACAAAAATGATATTGTATATTATGTTTACATAGCCTTGTAGCATCTGATTGCATGCT
GTATTTCTATTTCTTGAATCCCATATGCAGTTATGGCTGCAAAGTTATACATCTATGTTAATTGAAAGCTCTGTATCTTACTAGCTTTGGCAAAGGGAGG
CAGGAAGCTTGAGGCAGCAACTGCATAACTTGCAAAGAGCACCATCGGTCAGTTGTTGGGTCAGCAGCTTTCTGGCCTGGATGTAAAAGGTTTGCAAGAT
TTAGAGAATAAGCTGGAGATGAGCCTAAGAAATATTCGTCTGAAGAAGGACCAACTTATGATTGATCAAATTCAAGAATTAAACAGGAAGGGAAGC
>Locus_12535_Transcript_40/73_Confidence_0.311_Length_3011
TTCCAATAAGACACAGATTCTTCAGAAAGAGGTTTTCCTCTCCCTGCCCTTTTGATCTCCAACTATGGCAGTGGTGACAAGGAGCAAATTTGCAATCTTA
GGGTGGTTCTCGTCGTGAAACCTGAAAGTTTGGGGAAATAAATTTGGGGGTGTGGATGTCATGATATGTAGGGTCCGGCACTGAAGTGGTTGATGCTGTG
GTTTCCAGGTTAAGGGGAGAAGAGGAATAGGATATAGATCTCAGGAAGCCAGAGATGAGGTCGAGGGGAAGCCAGTGGCCAAGCTTGTGCTCCAGTGAAG
CTGATTCTCTTGTTCCCAGTTGATCCATGCGCCGTGGCCTCAGTTCGCAGGATCAGCAGCTCCATGGTTCTGTGTGGTGCTGCAGTTTGAGAAGAAGGTA
CTTGGTCTTTTGCACCTTGATGTACTTGTGTTTTTTTGCCTTTCTTGATGCTATAATGATGGTGGTGCTGTTCTTGCCATGATTGACGATTCCCCTAGAA
AAGGTTGAACTACTCTGCACTCTCTCTGGCACATGGTAGCACCTCGTGTACTTCTCTGATGATGTATAGTTGAGTGCTCAGATATGTTGTTGCAAATCTG
TCCAATTTCTGCTACCCATATGTCAAATCTTTTCTGTTTTAGTCTGTTTCACTCTTCCATTTTGTTGCTTCAAAGGTAGCTGCTTTTGCATACAATTAG
GAGCAGCAGTTGAGATATTCAATTCGGGCATGGGTTCTTTTGGGATGGACTGGAACCAGAAAGGCTCGGTGTTGTGGGATTGGGAGAATTTGCCGCCGAT
AGGCAGAAATGCAAACGAGAACCTCAAGATTGCGCCACAGGCTGAACCGAAGTTTGCAGGTGTTGAGGCAACAAGGCATGAATCGGGGCATTCTTCTTGT
GGTACTTTCTCTTCCAGCTCGGAGATGGGGTATGGTTCATCCAAGAGTTCCATATCAGCGTCGATTGATTCTTCACCCAAGGTGGGAAACAACGTGGAGC
TCAATCTTGCAGCTGTCAAAGTGCCTGACAAAAACACCGTCAAGAACACTGATTTGGGTAAAGTTGATGACGCTGGAACTTCTCCCATCATCGATGATAGC
CGTCAGCAGTGGAGAACCGGTGATTGGCCTGAAACTTGGCCAAAGAACCTATTTTGAAGATGCATGCGGAGGGCAAAGTGTCAAGAGTTCACCATCCAAT
GTGAGTGCAGTGACCCCTGCTTCTGCGAAGAAAGCAAAGATGATTCAAAACGCACAGAACTCGTACTGTCAGGTTGAAGGTTGCAAGTTTGATCTCTCTT
CTGCTAAAGATTATCATCGGAAGCACCGAGTCTGTGAAACTCATTCTAAGGCTCCCAAGGTTATTGTTGCTGGTCTGGAGCGACGCTTTTGTCAGCAGTG
TAGCCGGTTCCATGCTTTAACTGAGTTCGACCAGAAAAAACGAAGCTGCCGGAGACGCCTCAATGATCACAATGCCCGCAGACGGAAGCCACAGCCTGAA
GCAATTCCTTTCGGTTCATCAAGGCTCTCTGCAATGTTTTATGGTATCGCTAAGCCTTTGGTTGCCTAATGGTTTTGGGAAACTGGTGCATTAATTATTG
TTCTTTTAGCAACTGTTCTGTTTAAAAATCTTGCATTTTTCATGTATGCCCAGAACTAAACGGTGGTAAAATTTATGAGTACAAAGTGCAATGTTGTGTG
TCTGAAGTTGTCCCTTATTTGTTTATTCATAACTCAGTGCCATGATTTTTGAGATGTCATGAATTTACATACTTCTGATATTAGATGACATGATAACTTT
GGCAATACCGGTAATTAATGAAACTCCATCTGAGTACTGCCATTTGTGATGATTACTATAGATGGGACAAAAGAGATATCAATTAAGTTGTATTTATTTT
ATGAATAGGTGCATAATGCTATATTCTTTTCGATTTAGATATCCATGACATGATTCTACTTTATCTGCCATGATGCCATCTCTCCATTATAGGAAGTCGG
GAGACAATAGGAAGCTGTTAAGTATCTGAATAAACATCAGTTCAAATATCTACAGATGCATGTTGAATCCACTATATTATCAATAATTTGTACGAAAGTA
GCTTTAGCTTTTTGTATTAAGCAAATCGTTTGTCCTCTTTCTGAATCTGTTTGGCTTTAGTGATGAAAAATTGTCGTGGCGGTCTTAAACAGATGCAAGG
CAACAGACAAGTCTTCTGTTTGGTCAAGCTCCTTATGGTCAAATGAGAAGCTGTGCAAACTCTTCATGGGATAGCCCAGTAGGAGGCTTCAAATTTGGAG
AAACAAAAGCTCCTTGGTTAAAGCCAACGAGAGCTGCAGGTGTTGATGGGATGCATGTATCAAGCCAGCAGGTGTGGAACAATATTACGCCACACGGTGC
ACATCATGATTTTAATGGTATCATGGCTTTCAAGGGAACCAGTGCAAATGTCCTTAATCAAGGTGCCGAAGCTTCTCCGGTCGTCTCCAACTCGAATGGA
GCCCCAGATCTTCAGCGTGCTCTCTCTCTTCTGTCAAACAATTCAGCTGGTGCTGCCAACGACCAGCCAACTCCTCAGCTGCACCCTGGGCTGACCACCT
TCGTCGGCTCCTCCAACCCTGTCACTGTGATGGAAGCCTCACCACCAGGACTCTGGCAAGACAGCACGCCCCTTGATCATCAGGCGCAGTTCCAGGCTTT
CGATCCCATTGCCGCACCGCACAGCTCCAGCTCCCAAAACCCCCTTCCTTCAACAGCTCCACTTCCAGCTATGGCCAGATGCAGTGATGGATGCTTCCT
TCAGTTCTGCTGTCTGAAACTCTGAAATTTGAAACTGCTGTGTTCTAAAAAATAAAGAAAAGCCTGTGTGCGAAAACTCAGGCTTGAAAAATTGATCCAG
TAGTGTTCCCGTTCAATGGCTTGAATAATCCATCCTTGTGATGGCGTGTAACTCAAATGCTTGGATTGCGCTGGCAAGTGATCATGGACTCTTGTGTTCT
TGATGGCATCT
>comp64705_c0_seq3
AGGAGAGATAAGGGCCAGCAGTCTTGCCTGGTTTATTCAGTGTAATTAGACTAAGGATTAGGGGAGCAGACAGAGGAGAGATAAGGGCCAGCAGCCAAAC
AGTCCATCAGAAAAACCAGGCAAGACTGCAAGAGAGCAAGAGACCGTGTGTGTGAGAGAGAGGGGGAGAGAGAGGCTTTGCAGAAGAGAGAAGCAAGGTG
GGAAAGAGGCAGCAAGGGAGGAGGGCTACAGGAAGGGGAACATGCTCCCTTCTCATCTCACAGAAAATGGCACCATAATGATTCAATTTGGTCATCAAAT
GCCTGATTACGACTCATCAGCTACCCAATCGACCAGCGAGAGTCATCAAGAAGCGTCTGGAATGAGTGAAGGAAGCTTCAATGAGCATAATGATCGATCA
GGTAATCTTGATGGTTACACGAAGAGAGATGAAAATAAGATGATATCAGCTTTATCTCTGGGCAATCCGGAAACTGCTTATGCACATCCAAAGCCTGACC
GTAGCCAGTCCTTTGCCATATCATACCCGTATGCTGATTCATTCTATGGTAGTGCAGTGGCGGCTTATGGCTCGCATGCTATTATGAATCCCCAGATTGT
GGGCATGATGTCATCTTCTCGAGTGCCATTACCAATTGAACCAGCTGCTGAAGAGCCCATTTATGTAAATGCAAAGCAATACCATGCGATTCTCCGAAGG
AGACAACTCCGTGCAAAGTTAGAGGCCGAAAACAAGCTGGTGAAGAGTCGCAAGCCATACCTTCACGAGTCTCGGCATCAGCATGCCATGAAGAGAGCTC
GGGGAACAGGCGGGCGGTTTCTCAACACGAAGCAGCAGTCAGAGGCTCCTGGCGGCGGCACCTCGGACGCGCAACTCATGCCACCAAATGGTGGCCTGTT
CGCGAAGCACGAGCACAGCTTACCACCCAGTGATCTCCATTATCGCGCAAGAGGGGGCGCTTAAGGAGTCCCATGGCAACTCATCCTTGGCTTAAAAAAA
ATGTGTGGCGCTCAGCAATTCGACCTCTGCTAGTTCAATGTAAAGGTGTGGGACTGTAACTGTGGGTGCTTGAATTGGCCGCAGCCATTCCTGCGACGCG

TCGCAGGTGTTGCCGGTTCTGTGGTCGTGCTAGACTTGTGACCGGCACGACGAAACTGGCACCTGTTTCCTGTAACTGTTGTCTAAGATGATGATGACTA
CTCCAATAACCATTCTGTATGATGCTTTGACGTCGGTGTTGCACCG
>Locus_9469_Transcript_42/63_Confidence_0.204_Length_1155
GAGAGAGAGAGAGAGAGAGAGAGAGAGAGAGAGAGAGAGACCCTGCAGAGGAGAGGAGCAAGGTGGGAACGAGGCAGCAAGGGAGGAGAGCTGCAGGAAGGG
GAACATGCTCCCTTCTCATCTCACAGTATGAACTGAAAAAACAAGAATCGAGAATTTCCATGAAATGAGACAAAATGGCACAATAATGATTCAGTTTGGT
CATCAAATGCCTGGTTACGACTCATCAGCTACCCAATCAACCAGCGAGAGTCATCAAGAAGTGTCTGGAATGAGTGAAGGAAGCCTCAACGAGCATAACG
ATCGATCAGGTAATCTCGATGGTTACACAAAGAGTGATGAAAATAAGATGATGTCAGCTTTATCTCTGGGCAATCCAGAAACTACTTATGCGCATCCAAA
ACCTGACCGTAGTCAGCCCTTTGCCATTTCGTACCCGTATTGTGATTCATTCTATGGTGGTGCAGTGGCAACGTATGGCTCACATGCTATTATGAATCCC
CAGATTGTGGGCATGATGTCGTCCTCCCGAGTGCCATTACCAATTGAACCAGCTGCAGAAGAGCCCATTTATGTGAATGCAAAACAATACCATGCGATTA
TCCGAAGGAGACAGCTCCGTGCAAAGCTAGAGGCTGAAAACAAGCTGGTGAAAAGTCGCAAGCCATACCTTCACGAGTCTCGGCATCTGCATGCCATGAA
GAGAGCTCGGGGAACAGGCGGGCGGTTTCTCAACACGAAGCAGCAGTCAGAGGCTCCTGGCGGCGGCACCTCGGACGCGCAACTCATGCCACCAAATGGT
GGCCTGTTCGCGAAGCACGAGCACAGCTTACCACCCAGTGATCTCCATTATCGCGCAAGAGGGGGCGCTTAAGGAGTCCCATGGCAACTCATCCTTGGCT
TAAAAAAAATGTGTGGCGCTCAGCAATTCGACCTCTGCTAGTTCAATGTAAAGGTGTGGGACTGTAACTGTGGGTGCTTGAATTGGCCGCAGCCATTCCT
GCGACGCGTCGCAGGTGTTGCCGGTTCTGTGGTCGTGCTAGACTTGTGACCGGCACGACGAAACTGGCACCTGTTTCCTGTAACTGTTGTCTAAGATGAT
GATGACTACTCCAATAACCATTCTGTATGATGCTTTGACGTCGGTGTTGCTCCTT
>Locus_20324_Transcript_6/30_Confidence_0.230_Length_1151
AGTTGCTGTACTCCCGTTAGTGTTAACTTTTGGTGTACTTAGCTTGATTATGCTTTGCTGATGCTATTTCCCCCCCTTCAGGTGGATTTGATACAGCGCA
CGCTGCGGCAAGGGCCTATGATCGCGCTGCGATCAAGTTCAGGGGCCTTGATGCGGACATCAACTTCCAGTTGAAGGACTATGAGGACGACTGGAAGCAG
ATGAGGAAATGGTCCAAGGAGGAGTTTGTTCACATACTCCGACGTCAAAGCACTGGGTTTACAAGGGGGAGCTCCATGTACCGCGGTGTGACACTGCACA
AGTGCGGCCGGTGGGAAGCTCGGATGGGTCAGCTTCTTGGAAAGAAGTACATCTACCTTGGGTTGTTTGACAGTGAAGTTGAAGCTGCAAGAGCATATGA
CAGGGCAACCATTCGCTTCAATGGAAGGGATGCTGTTACTAATTTTGATCCTAGTTCCTATGATGGAGATGTTCCACCTGAAATTGAGAAAGAAGGGGTT
GATGGGGACATCCTTGATTTAAATTTGAGGATCTCGCAACCTAACATGCATGATTCGAAAAGTGATGGCATCCTGACTGGGTTCGGATTAAGTTGTGATT
TTCCTGAAGCTTCAAGTTCTGTTGTTTCTCAGGAAGTGCCTATGGAGAAACGACCCGAGTTGTGTCCCCAGTCGTTCCCTCCGTGTGCATGGCAAAAGCA
GGGTTCCCCTGCGCCATCACTCCCTACTGCAGCATCATCAGGATTCTCTACTGCCACCGGCGGCGCGGACCCGGCCAGCGTGCCGCCGTCCCGCCCCCGG
CCCTTCGCCACCGGCCACCAGCTCCACTTCCCCCCAACCGTCTGACAGCCTCCCCCTGTTCAACTGGCGACGATAACGCGGTCGTCTGGGCGCCCCATGC
TTCGCTGCTGGTCGGCAACAGTCGCAATTGGTTCGGGTAGAGAGAGATGGAGGCTCCAGCACACGACCTCTCTGTCTCTCTCCCCACCTGGTAATTATCA
TCAGAACCACCATGCTTATGATTCTTTTATCTTCATTTTGAGGGCGGATCCACGGATCATGAGCGTGTACCTCCAAAATTCTAAAATAAACCATTTACGA
TAACTCTGGCTGAGGCTCTGTTTGTTTGGGCTTCTGGCCAACTTTGAACGG
>Locus_16244_Transcript_14/14_Confidence_0.133_Length_1455
CAGAGGAAGGGACGGCGGGGAGGGGGCAGGCACAGAGAGCAGCTTTGCTTGGCGGACGCACCGAGGGAGGCGCCTGGGAGCCCTGCTTCTCCCCTCTTCG
TCTTCCTCCTCCGCTTCCAAAGGTTACCGTCTCCCGCCGCCATAAGACTAGTAGCCTTCTTGCTGTGTCAATCCGTCATGCACGCGTCCCTGGATTGCGC
TGGTGACTCCTTCGGGAAAATGGTTAATGATCATACGAGGTCAAATTTGGTTTTTGATAATAAGCAATCTCTATTTGCAAGTCATAACATTGACTACGGC
CAGCCAATAGTAAGACTCATGGTGATATAAAACATCAGTTTTTTCAACTCTGTTAAAGTCACCATTCTAGTTTTAAAAAACAATAAAAAGCTCATCCTAT
TCTTTACATGGGGATGTGCATCTGATTTTATTTTTCTACCATATTACCCAGGCTTGTATATCATACCCATACAATGATTCGGGCTCAGGAGGTGTTTGGG
CAGCCTTTGGGTCACGCACTAGCGCTGCAGCTGTGTTCCATCCCCAAATTGCTTGTGGGGGCACATCTGCGAGAGTTCCCTTACCTCTGGAATTAGCAGA
TGATGAGCCCATATATGTCAATCCCAAACAATATCATGGTATACTTCGCAGAAGACAGCTACGTGCTAAGTTAGAGGCTCAGAACAAGCTAGTCAAAAAC
CGAAAGCCTTACCTTCATGAGTCTCGGCATCTTCATGCAATGAAGAGGGCAAGAGGTTCTGGCGGACGTTTCCTTAATACTAAACAGCTCCAGGAGCAGC
AGCAGCTGAAGTCTCGCAATGCCTCCACCAGGTCCACCACAAATGGCGCAAATTCCTCAGGTTCAACACATCTACGGCTTGGTGGTGGTGCAGATGGAGA
TCAAACCATGTCGGGGACGAAAACAATGGCCTCACAAAACAATAGCAAAAAGGCTGTTTCTTCTTCTGCAGCTCTTGCTTTCACCGTGACTCCTATGGTG
CGCAAAGATGACACCTTCTTCCAGCACCTCAGCCACAATGTCAGCTTCTCCAGCCATTTTGGCCAGGCAAGCGCCCAAACCGGCGTTGGAGGCATCCATA
ATGAGACCCAGCATAGGGTTTCCGTGATACAATGACGGTTTGCGAAGCTTATCTGGTGATCCAGGCTTCTAGGTGTCCTCGTGTCCGGTGTAGTCTTGGT
CGCTCAGGCAATTCATCCTTGGCTTAGTTTTTGGTGTGTTAGAACCTACAAATGTTTCATACTCTGTTGTGGTTTGCAGAGCCCATAAATCAGGGCTCTC
CTAAAAAAAATCAGGGCTTGATGGCGACACTACTATCCAAGTATTGTTGTAATGGTGTGTGTTAGAAACTCGCAAAAAACCCTTCTTTGTGCTCGTATTA
GTACATTATGCTGCACAATTAGCTGCTATTCTGTTCGTACTACAGTCTGGTATGC
>Locus_3441_Transcript_373/417_Confidence_0.012_Length_249
GAGGTCGGCCTCATGATCTTCTCCAGCACCGGCCGCCTCTATGAGTACTCCAGCACAAGCATGAAGTCAGTAATAGATCGATATGGCAAGGCAAAGGAAG
AGCAGCAGGTCGTCGCAAATCCCAACTCGGAGCTTAAGTTTTGGCAAAGGGAGGCAGCAAGCTTGAGACAACAACTGCACAACTTGCAAGAAAATCATCG
GCAGTTGATGGGTGAAGATCTATCTGGGCTGAATGCTAAGGAACTGCAG
>Locus_12535_Transcript_44/73_Confidence_0.330_Length_2439
TTCCAATAAGACACAGATTCTTCAGAAAGAGGTTTTCCTCTCCCTGCCCTTTTGATCTCCAACTATGGCAGTGGTGACAAGGAGCAAATTTGCAATCTTA
GGGTGGTTCTCGTCGTGAAACCTGAAAGTTTGGGGAAATAAATTTGGGGGTGTGGATGTCATGATATGTAGGGTCCGGCACTGAAGTGGTTGATGCTGTG
GTTTCCAGGTTAAGGGGAGAAGAGGAATAGGATATAGATCTCAGGAAGCCAGAGATGAGGTCGAGGGGAAGCCAGTGGCCAAGCTTGTGCTCCAGTGAAG
CTGATTCTCTTGTTCCCAGTTGATCCATGCGCCGTGGCCTCAGTTCGCAGGATCAGCAGCTCCATGGTTCTGTGTGGTGCTGCAGTTTGAGAAGAAGGTA
CTTGGTCTTTTGCACCTTGATGTACTTGTGTTTTTTTGCCTTTCTTGATGCTATAATGATGGTGGTGCTGTTCTTGCCATGATTGACGATTCCCCTAGAA
AAGGTTGAACTACTCTGCACTCTCTCTGGCACATGGTAGCACCTCGTGTACTTCTCTGATGATGTATAGTTGAGTGCTCAGATATGTTGTTGCAAATCTG
TCCAATTTCTGCTACCCATATGTCAAATCTTTTCTGTTTTTAGTCTGTTTCACTCTTCCATTTTGTTGCTTCAAAGGTAGCTGCTTTTGCATACAATTAG
GAGCAGCAGTTGAGATATTCAATTCGGGCATGGGTTCTTTTGGGATGGACTGGAACCAGAAAGGCTCGGTGTTGTGGGATTGGGAGAATTTGCCGCCGAT
AGGCAGAAATGCAAACGAGAACCTCAAGATTGCGCCACAGGCTGAACCGAAGTTTGCAGGTGTTGAGGCAACAAGGCATGAATCGGGGCATTCTTCTTGT
GGTACTTTCTCTTCCAGCTCGGAGATGGGGTATGGTTCATCCAAGAGTTCCATATCAGCGTCGATTGATTCTTCACCCAAGGTGGGAAACAACGTGGAGC
TCAATCTTGCAGCTGTCAAAGTGCCTGACAAAAACACCGTCAAGAACACTGATTTGGGTAAAGTTGATGACGCTGGAACTTCTCCATCATCGATGATAGC
CGTCAGCAGTGGAGAACCGGTGATTGGCCTGAAACTTGGCCAAAGAACCTATTTTGAAGATGCATGCGGAGGGCAAAGTGTCAAGAGTTCACCATCCAAT
GTGAGTGCAGTGACCCCTGCTTCTGCGAAGAAAGCAAAGATGATTCAAAACGCACAGAACTCGTACTGTCAGGTTGAAGGTTGCAAGTTTGATCTCTCTT
CTGCTAAAGATTATCATCGGAAGCACCGAGTCTGTGAAACTCATTCTAAGGCTCCCAAGGTTATTGTTGCTGGTCTGGAGCGACGCTTTTGTCAGCAGTG
TAGCCGGTTCCATGCTTTAACTGAGTTCGACCAGAAAAAACGAAGCTGCCGGAGACGCCTCAATGATCACAATGCCCGCAGACGGAAGCCACAGCCTGAA
GCAATTCCTTTCGGTTCATCAAGGCTCTCTGCAATGTTTTATGATGCAAGGCAACAGACAAGTCTTCTGTTTGGTCAAGCTCCTTATGGTCAAATGAGAA
GCTGTGCAAACTCTTCATGGGATAGCCCAGTAGGAGGCTTCAAATTTGGAGAAACAAAAGCTCCTTGGTTAAAGCCAACGAGAGCTGCAGGTGTTGATGG
GATGCATGTATCAAGCCAGCAGGTGTGGAACAATATTACGCCACACGGTGCACATCATGATTTTAATGGTATCATGGCTTTCAAGGGAACCAGTGCAAAT
GTCCTTAATCAAGGTGCCGAAGCTTCTCCGGTCGTCTCCAACTCGAATGGAGCCCCAGATCTTCAGCGTGCTCTCTCTCTTCTGTCAAACAATTCAGCTG
GTGCTGCCAATGACCAGCCAACTCGTCAGCTGCACTCTGGGCTGACCACCCTTGCCAGCACCTCCAGCCCTGTCACTGTGATGCAAGCCTCACCACCAGG
ACTCTGGCAAGACGGCACGCCCCTCGATCATCAGGCGCGGTTTCAGTTTTTTGATCCCCTGGGCGACGGCAGTGCCATTGCCACACCGCATCAGCTCCAG
CTCCTAAAACCCTTCGCCTACGACAGCTCCTCGTCCCACTATGACCAGATGCACTGATGCTTCCTTCAGTTAGCGCAAACCGCGGTTCAGTTGCGCGTAA
TCAGACATGGTTTCCACTGTCTGAAACTCTGAATTTTGAAACTGCGATGTTCTGAAAAATAAAAGCCTGCGTGCCGCAAACTCAGGTTCGAGTTGATCTA
GTAGTGTTCCCGTTCAGGGGCTTGAATAATCCATCCCTGTGATGGCGTGTAACACAAATGCTTCGATTGCGCCGGCAATTGATCATTGACTCTTTTCGTG
TTCTTGATAACATCTCAATTACACCTTGAATGGAACACT
>comp76332_c0_seq36
CAATTTCTATCAATAATTCAAGTGTCGCACAGCTACAACGATGCTCCTAATTATGAAGAGAAAAAAACAGATGAATTAAAGGCTGAAGGTTCATTCAACC
ACCAAGCACCCAAAATGTCGATCTGCATATGCACAGAACCTCAGTACAGTTCACTGGTTGAGAAAATAGACTGCAAATGTGCAAATAATACAAATTGTTA
CTGGAAGAACAGTAAACACAATACCAGAAATCCTTCTAACGGTAACCATTGGAGGAAGCCTTGTGATCCTTTTCAACCCAAGTACATATATTGAAGATGA

AATTAACAAAAGATAGAATTGATAAAGCATAAGAAACAGAGGAACAAAAAAAAAGTACCTGGTCCAAGTTCCCATCCTCTGAAAAAGATCAGCAGTCAAT
AGATGCTTCACTCGTCCTCCAGAAAAATGAGTGCCGCATGAATCCTTTCCGGTTTATAGTGAAACATTTTAGACGATGACCTTTAACATTCTAGAAAACG
AAGAGAAAACCTACAGTCATCTAATTTCAGCTTAAACATGTTCCAGCAAGATCATCACCATCCTGACATGTTCCGTTTTGTCCCTAGACCCGAGCACAAG
GTATTGCGAGGCTAATTTAGCGGGGCAATTGATTTTTTGCCACTATGATACAACTGCAATTACCCGCTGTCACTGACACGTGGGGTCATCTGAGTCACTG
AAATGTGGGCCCAATAGCATTTTGTTTGCCACTCACCCTTCCAGAGTGGCAAAAAGTTAATTGTCCCTAATTTAGCTCAATTCAGTTGTCAGAGCAATTG
TGTGTGAAAAGTGTGAACAACAGCATAGGAGACAATACAGCAAGTGTAGCGTGCCAATATGTGTCCAGCTTGATAGTTCACTATAATAAAGTTCTTTAAT
TCAAGTATTTGAATAATATAGAATCTCAACCTTCAAGTTTAAAAGGTTTTGTATTTGCTATTATAATCGTTGCCACCCCCTCACCCCTCTCAAAAAGAGA
GTTACTTTGTAGCGCACTGTAAATCTCTTGATTGAAATAGTGGCATGTCATCTTATTTAATTCAGTTATTTCATAAGGTACAATTTGTTTAGCTGACAGC
TCAGACATGCTTCTATGCAACATGTTTATACCAATGCTGTATTTTGGCAATCCTGGATGTGGCATTACTTAACTGTGCTTTCATATTCAATAGTTCTGGC
AAAGGGAGGCTGCAAGCTTGAGACAACAACTGCACAACCTGCAAGAAAGTCATCGGTATGGCTAGAAAACCCTGCAAATTTTACAAATGTACAAGTCAGA
AAATTTGCCTAATGTAAAAGGATCAAACTACAGGATTTGTCAGCATGAGCACAAGTGCCTAACTTCCGAGTTAGATAACTCTGGAGGAAGGAAATCCTTC
CTACTGTTTGTTTCAAGAATATACAAAAATTCAAAACAACATTAGAATAGAACCAAAAGAAGAAACAATATTCACCATGTAGCAATACTCAATTCCTGGT
TGTCTTATTTATAAAGGTATAAAACTAGGTTCTGACGAGACAATTATGAATCAACTACACCATGTTACAGATCATCCATAACTCTTGGTGATGCAAATTA
AGGGCTGTAAAGTGACAAGTATACCTCCACCAACTAATTTGTGCAGAGTGTATAAAATTGTAAAACAATGGATAAGAATGAATTTAGTATACAAGATAGC
ATACAATGCCCAAGTTTAAGGAGACATACTGATTTACTGACTCCATAGGACAATCTTTTGCTTATGAGGACATCAGTTTTCATAAACAATATCAATGTAA
AAGGAGGTAACAGTTTATGAATTTCCTATGCCAAGCCATTTATAAGTCAAAAAATGTACCTATTTAGCTTTTATGCATTCCAATTAACTTCCAAAGAAGA
AGAGTACATACTAATGGAAAACAAATATGATATTATCATCTGTTTTGCCCGGTAGTTTCTTGAGTCTCTGATTTGTCCGCACTTCACTCCCAACCAACAC
AACATTAATACACATGGGCAGAGGAATATCAGGTTATCAGGTAAACTGAATACAGTTTGATTGACTGCAGTCAGTTGATGGGACAAGATCTTTCTGGATT
GAGCGTCAAGGAACTCCAAAATCTAGAAAATCAGCTAGAAATGAGCCTACGTTGCATCCGGACAAAAAAGGTAAGGATGCTAGCCATATTTCTGTTTTTC
GTTACCAGATTGCCTCATCCAAGAATACAACTGATGATGACGTTTTTTACTTTCAGGACCAACTCTTGATTGATGAAATTCACGAACTGAATCGAAAGGT
TCTGGAATATTCTGTTATTACTCTGCAATGAGAGGTCTCTGAGTGGATTTCAAGAAGCTGACTCTACTGGCTCATTTTCAGGGAAGTCTCATGCAACAAG
ACAAGATGGAACTATACAAAAAGGTCAACCTAATCCGTCAGGAAAACATTGATTTATACAAGAAGGTATTTGATAAATCGTGAATGAAAATTCACAGGAT
ATACACCCCATCCATTATTGACTGAGGTTTATTTCTCTACTCAGCTCTACGAGAAAGAGGTGACAAGTGAAGTCAACCGAGATTCATCAACTCTAAACTT
TGCAGTTGTCGAGAACGTCAACATTCCTGTTCATCTTGAACTTAACACTCCACCGCAAGAAAATGATGTTGAGCAAACTGCACCTCCTAAACTGGGGTAA
CCCTGCTTTTTTCTGCAAAGGAAGATTTTCTTGCAAGTTATGCAGTTGTTGTCTCTAGCTTGCCGCCTCCACTTGCAGTAACTGAGACGCCGGCGCCGAG
CTTGAGCTTGAAACCTAGACTACGGGGACAGGGTAGACAGGAGACGCGAAACCCCAAACCAGCGCAAAACGACGCGCAGGGAG
>comp82045_c1_seq2
AAGGGGCGGCTGAGCAAGGAGGAGGGGAGGTGAGGTGCCAGGTCGAGGGGTGCGGGATAGACCTCGGCGCCGCCAAGGAGTACCACCGGAAGCACCGCGT
CTGCGAGGCCCACACCAAGTGTCCCCGCGTCATCATCGCCGGCCAGGAGCGCCGCTTCTGCCAGCAGTGCAGCCGGTTCCATGCACTCTCGGAGTTTGAT
CAGAAGAAGAGGAGTTGCAGGAGGCGTCTGTCCGATCACAATGCCCGTCGGCGGAAGCCTCAGCCAGATGCCTTCGCCTTTGCCTCGGCAAGGCTGCCAC
CGTCATTATTTGATGATAGGCGACAAATAAGTTTTGTCTGGAATAAAGCTCCTCTTAGCCATGTAAGACCTTTCACTTCTTCTACATGGGACAGCTCATC
TGACTTCAAGCTTCCACATGCGAAGGAAATAAGAGAGCTATCAACAGGAGTTGGGACTATTACTGGACAATTTCATCTGGATAAATCTCATCTGTCCAAT
GCCATACCAACACTTAGCCATGGCAAAGATGAGCCGTTACCAATGAAAGGTCCGGACACATCTTTAACTGCTTCAAAATTCGATGGAGCACCGGATCTTC
AGCGTGCTCTCTCTCTTCTGTCAGCTGGCTCTTGTGGATTGCCTGATCCTGTACAGCAAGCATCTTGTCTTATCCAGTTCTCTGGTGCCAGCGAGAACAG
CAGTGACCTTCATTCATCACATGGTGGGAACTCTGCTCCAGTCTCATGCGCTGATGAACAGCACATAGCACCTCAGTCTCAGCTAGTCCGTTTTACCATG
GATGCCAACAGCAGTGGCTACGAGTCCACTTTCTTCGGTGTAAACCAGATAAATTAAGGTGTGAAAGGCACAAGCCTCTTGATCATTTGGATAAACAGCC
ACGATGATGGCCTGAACTTATGCAAGAACAGCTTCTCTATCAACTGCCAGAGTGCAAAACCTTGTTAATGTTATCTAAGTTGAACTGAACGTAGGCCAGC
TTGTGTTGAGTATGCAGTTCCAGCCTGTGATTGACCCATTTTTTTCTGTTCTTTCCCCCCGTTGACGTGCCTACTTTGAAACTCTATGTGATATTGCTAT
TTTACACTTGTACAACCTACTGCCAACCGTTGGATCTCTTTGAAAAGGTCCTTGATAAATCATGTGTATTTTGTGCGCATAGTTATTACTACAATGCTGT
TCTTTTTCAATAAAATGCTTCGTGTT
>Locus_7001_Transcript_79/84_Confidence_0.135_Length_2928
TGCTTCTGCGAGAAATGGATGGTTATCCAGTCCATTCCATGCCCAACTATCTCTGATGGAGACTCCTCATCAACAAAGTCTGAGCAGTGTCGCCAGGACT
TATCTGCAGTGAGTGACAGCAGCCTCAATGGACAACACAACCAACACAATCTGGTAAAGAATTTTCGCAAACACCATTATATCGCTACAAACCTATTCTT
TTGATTCTTTTTACTAATTTAAGATTCTTACGAAGAACATAGTTAGATGTGTTCCTAGGTTTGCAAATTATATAGATATGTTGTAATATTTCAAACAATA
ATACTGATAGCATAAAGTTGTCTTCAAATCAGATTAGAATTAAATTCTGATTAATTTTGGTAGTGTTTGATTTGTAAGCTTTAGAACTGGTAATAATAAT
AATTGGTGCTGCAGTCTTGGCCAACCTTTTCATACTGTGTACAATATTTTTTCACATCGTGACAATGCCATAAATACTCCACATTGAAATTATGTTTGCT
ATAGTCAAATATGTGCCACCATTGTCTATAAATTGTTACTGGATTTTTTCTTTTGTCTCTAATGTATTAACTATTAAGCTTCTCATCAGAATGTTCTACT
TGCTTTTAGATCATTCTAGGTATGCTGCATTCGAATGCCATTTTCTTTATTTATTTGTGGGCTTCCTAGATCTAATAAAGTTCCAGGGATTTTGGTTCTG
GACTCCACCTTGGTGGGAAAGTTTGCCATTCTAAAGATATTGATAAAATGCAATGTCGTACTTGCAATAATTGGTTAGATTGTGAATTAGTATAGAGAAG
TGATAAGATTGTGATCTTTGGAAAGGATACTAAATGTGCTTGTAGTTACAAAGATAATATGTGGATTTGCAGGTGTACTAGTACACAACATTTTTTATTC
TGTACCTAGATTGACCATGGCACAGTACCAACAAGCCACCAGGATGAAACTGAAAATGGCCCAGTACCATTCCTTTTTTTGGAACACTGGGCCCCTTTCT
CTGACAACTTCTTTCATAGAAACCTTGATAAGCCCAGAACTGGCTGCTCCCCACAAAATGGCTTCTGAAGCGAAATTTCCAAGTATTTGTGTGGGTGGAG
TTTGAAGCAAAATCCCAAACGAAGAGAATAACCATGGTAGCTTTTCAAACAACAGTTCCAAGTTTAAATTTTTACCATTTTTCTGCCACCGGTATGTTTT
AAACTTGTTTGTTTAAGAACAGATTGAGTACTAATTAATGAAGTGGAACTCATATTCCTGATGGTCAAGCTTCGTTTTTTAGTAATGGAAGTACAAGTCC
TGGCCTAATGGTCGAGCTTCATATCTATCATAGTTTAAATATTTTAAATTTAAAATAGTCTTTTATTGGGTAAAATATAATATGTGCTGATCAGTCTTTT
ATTGATTGTTGCATGTGTTTCACTTGTTCTGTATTGTGAGTTCTCTTCTGGTTCTTATTCTGAAGCAGACATCGAAAATTCTATGTAATCATGTCTTTTG
GAAATAACCTATCATGTCAGTCATTGCTCAATTATTCTATTGTTCCAAAAAAAGAAACTTTAGGACATGAAGGCATCCGATGGATACATCTGAATAGGCC
TTGATGTGTCTGATAAATTCTTATAGGCTACACGAGAGAACACCTTTATAGCAGACCTAAACGTCTAAGTACGATTCAGCCTATCAGGATGCAAGGGTCC
TAGCTTGAAACATTAGGGCCTTCAACTGTAGATGGTGCTAAATTAATTGACCAATCAATTATTCACTCCTTATTTCAAATATTTGACTTATGCCAAATAA
TTTATTTATTTGTAGCAAGTACAAAACAATAGAAAATTGAATTGTGTTAGGACCTCTGTAGTGTTTCTTATGGTATAACAGCTTTCTTCTCCCACTCAGA
CAACAATGATAGTAGTGGAAAGCGAGACCAGGGCATGGTAAAGTCTGTATTATCCTTGGGAAACCCAGAAACTGCTTTGTCATCTCCAAAGTTTGATTAC
AGCCAGCCTTTCGCTTGCGTTTCTTATCCATATGCTGCCGATCCATGTTATGGTGGGATCTTAACAGGATACACATCAAATGTCGTTGTTCATCCCCAAA
TTAATGGTACAGCAAACTCTAGGATACCCTTGCCTATTGAACCTGCAGCCGAAGAGCCCATATTTGTCAACGCAAAGCAATACCATGCAATCCTCAGGAG
AAGGCAAATGCGTGCAAAATTTGAGGCCCAAAATAAACTGGTGAAAGGCCGGAAGCCGTACCTTCACGAATCTCGACACCGTCATGCAATGAAGCGAGCC
CGTGGGTCGGGAGGCCGGTTCCTTACCAAAAAGGAGCTGCAGGAGCAGCAGCAGCAGAAGACACTGCCTTCACTTCAGACTCCAACATGTGTGGCAAGTA
AAATGTCACTCGGCAGGAATCAATGCCCTGAAAACAGCACATCTCGCTCGCCTTCTACACCGACAGACTCTGGGATCTCAAGTGTTTCCAACGGAGGTGG
CATGCTGGCTCACCAAAAGCACATCACCTTCACATCTACTAATTTCCTTCCCAGCATGAACTTCAGCACCCAGAATGGAGGTGAAAAGATGGCTGTCAAT
GGTGCGCACCACCGTGCCTCCATCGTGAGGTGAACAGCCTCGATTGTGGGGCGGTGGTATGTACCATCACTGGCGCGCTCCGGTGAGCCCGTGGCAATTC
ATCCTTGGCTTATGAAGTATCTGTATCTGGTAATAATTTGCTTTCAGTTTAATATGTAGAGCTTGTCCGAGATGAACTCATTTATATTTGTTGCAACTAA
AACGAGGAGCTACTTGTTGATATATGTGGATATTGTGGATACAGAAATATCCTGATGTTTCTTGATGTACCAAGTCCACTATTAAAGTATGACTGGAAAA
AACGTTTAATAACGTAATCATGGTTTGG
>comp82796_c1_seq17
AGAAGCAGTGGCAGAAGTAGTTTGTTGTGGGGAGGGCATGTGCCTGGTTGCTGTGTGGGGGGAGGCAGCCACACACTGGGCCTCACTCGTCCTGCCTCAG
GTGGGGTTTGTGGAGGGAGGTAGGGGAAAGGTTGCCATCTCCATCTTGTGCGTCTCTGTTGGATCTTTGTGAGTCATGGCCTCTTTGCTCTCTTGCTTTT
CTTCGTTTCTTGTTGAACTCATGGTTCTTGTTTTTCTTGTGCTTGCCAGGTAGAATTATTCAACCGGATAAAGATTATTTCAAGTAAAGGAGGGCTACCA
GTTTATACCTGTTCAGGTGGGTGCAGCAGTTGAGAACTTTTTTTCGATACAGAGCAGTTGAGATCTTTAATTCTGGCATGGGTTCCTTTGGGGTGAACTG
GAATCAGAAGAACTCCGCGGTGTGCGATTGGGAAAATTTAGCACCTTCTGTTCCAAATGCAATCGGAAACCCCAGGAACGGAGCTACGGGGCATGGATCT
GTGAATTCATCTGGTGGCACTCTCACTTCTAGCTCAGAGCTAGGGCATGGTTCATCCAAGAGCTCCATGTCGGCATCCATTGATTCACCGTCCAAAGTAG

GGAACAGCTTGGAGTTCAATTTTGCTGCTGTCAATACGTATGGTAAGAACATGGATAAGGATTGTAGAGTTGACGACTCAGGGACTTGTCCATCGTCCAT
GATAGCATTCAGCCATGGAGAGCCATTAATCAGTCTTAAGCTTGGAAAAAGGACTTACTTTGAAAATGTCTGCGGAGGACAGGATTCCAAGAGCACTGCA
CCTTCTACCAGGACTTCTCCATCAACCGTTCTCAAGAAGACCAAGGTGTCTCAGCAGAATACACAAATGTCATACTGTCAGGTTGAAGGTTGCGGAGTTG
ATCTATCTTCTGCTAAAGATTACCATCGCAAGCACAAAGTCTGCGAAGCTCATTCTAAAGCTCCGAAGGTGGTTGTTGCTGGTGTACAGCGCCGTTTTTG
CCAGCAGTGTAGCCGGTTTCATGGCTTAGCCGAGTTTGACCAGAACAAACGAAGCTGCCGTAGACGTCTTACTCATCATAACGCACGAAGGAGGAAACCA
CAGGCGGATACAATTTCATTCAGTTCATCACGTCTCTCAACAATGTTTTATGATACAAGCCAGCGGACAAATCTTTTCTGTAGTCAGCCTCTTTTTGGCC
AAGTGAGAAGCAATGCAGTTTCTTCATGGGATAACTTGGGAAGCTTCAATTTTATGGAAACAAAACATCTGTTGATGCAGCCAACGAAAATGTTAGGTCT
TGATGGGCTGCATTTCTCAACCCCCCAGATATCAAATAGTGTTGTGGCTCACTCTGTACATCATCATAATTTTGATGGGTTCACGCCATTCAAGGGAACC
AACACAAAGGTCCTCAACCAAGGTGTCGAAGCTTCTGCGATCGCTTCCAACTCGAGTGGAGACCCGGATCTTGGGTGTGCTCTCTCTCTTCTGTCAAACG
GTTCGTGGGGTTCAACTTCAGCCGTCATCCAACAGCCTAGTTCTCATGCGCAAGCTGGTGCGGTGCCACCCTTCGCCACCGTCGCCGTCTCCAACCCTGC
AATGCATCCTCTGGACTCATCCCCAGGAGGATTCTGGCAAGACGACCCTTCCCCGCTCGATGGAACTCCGGAGATTCAGGCATTCGGACACCTCTAACTA
TGGTCAAACAAATGTACTCATGCTGCATGCCCTCGGAACAGCTGATTTAGTCACCTCATCATAATCTGCAACTCTGCTTTGCCAGTGGCAGATCTCTGCA
TAATGTCAGTCCTATCATCGGAGGCATTTGGTGAGGTGAAGACTGAAGAATAATATAGGAGGCAACCCCCTGATCTTCCTGATTTAGGATGCATCTAGGA
TCCAGTTGTGTGTGTGCTTGTGGTTTTGCCAGAACCGGTTTTTTGTTTGCCGTCCCGTTAGAGAGAAGGGATCAAGTACATTCATGACTGCTCATGAGAT
TACTTGTTTCACTAATATTACATGATCTGGAGCGAGATTTCGCTCGGTTCACGTTGATGGT
>comp78487_c0_seq1
ACCCGATTTGTTTAGATTGCAATTAGACTAAGTATAGTTGGTGAGAAGACAGGAGTAGAGATAAGGGCCAACAGCCCAACAGAAAAACCAAGCAAGACTG
CAACTGCAAGAGAGATGGGGAGAGAGAGGAAACCTTGCAGAGGAGAGAAGCAAGGTGGGAAAGAGGCAGCAAGGGAGGAGGGCTACAGGAAGGGGAACAT
GCTCCCTTCTCATCTCACAGTATGAACTGAAAAACAGGAATAGAGAATTTCCGTGAGATGAGACGTAAGGAAAATCTCCATGATACTGTTCAATTTGAAT
ATTGCGAGTACTCTGTCCTTTTTCATGTCTTCGGTAGTTTAGTCTCATGCAGCTAGAATTTTAACTCCTACGGTGCTCCTCTAGCAGAAAATGGCACCAT
AATGATTCAATTTGGTCATCAAATGCCTGATTACGACTCATCAGCTACCCAATCAACCAGCGAGAGTCATCAAGAAGTGTCTGGAATGAGTGAAGGAAGC
CTCAACGAGCATAACGATCGATCAGGTAATCTCGATGGTTACACAAAGAGTGATGAAAATAAGATGATGTCAGCTTTATCTCTGGGCAATCCAGAAACTA
CTTATGCGCATCCAAAACCTGACCGTAGTCAGCCCTTTGCCATTTCGTACCCGTATTGTGATTCATTCTATGGTGGTGCAGTGGCAACGTATGGCTCACA
TGCTATTATGAATCCCCAGATTGTGGGCATGATGTCGTCCTCCCGAGTGCCATTACCAATTGAACCAGCTGCAGAAGAGCCCATTTATGTGAATGCAAAA
CAATACCATGCGATTATCCGAAGGAGACAGCTCCGTGCAAAGCTAGAGGCTGAAAACAAGCTGGTGAAAAGTCGCAAGCCATACCTTCACGAGTCTCGGC
ATCAGCATGCCATGAAGAGAGCTCGGGGAACAGGCGGGCGGTTTCTGAACACGAAGCAGCAGTCAGAGGCTCCAGGTGGTGGCACCTCAGATGCGCAACA
CATGGCCGGCAATGGTGGTCTGTTCAGGCTGCACGAGCACAACTTACCACCCAGTGATCTCCATTATGGCGCGAGAGGTGGCTCTTAAGGAGTCCCCTGG
CAACTCATCCTTGGCTTAGTAACGTGTGGCGCTCAGCGATTCGACCTCGGCTTCGATGTAATGGTGTACGATTGTATCTATGGTGCTTGAATTGGCAGCA
GCCATTCCTGCAGCAATGCGTCGCAGGTGTTGCCGGTTCTGTGATAGTGCCAGACTTGTGACCGGCACAGAACTGGCATCTGTTTCCTGTAACTTTAGTT
TTTCTAAGACGATGACGATGACTACCCAATAACCATTCTGTCTGTATGGTGCCTTGCGTTTTCCGTTCGCCCGTGACGGTGTTGCTCCTTAAAAGTCAGT
GGCGGGAATTCTTCTTTGTCTCCGCGATGAACTGGTGAAGTTATTTCTTTTTTCTCT
>Locus_10929_Transcript_67/78_Confidence_0.299_Length_2378
GAGCAGGGATTGCGGGAAGCAAGTCTACTTAGGTAAGTGATTGAGTCCTATACGTTCCAGCTCGAGCTTCTATTAACTGCTGATCAGTTTCCGCCTGTTT
GATCTGGTTTACGATGTGCTGATGCTGCTTCTATTCAGGTGGTTTTGACACTGCGCACGCGGCTGCGAGGGCCTACGATCGTGCTGCGATCAAGTTCCGA
GGGCTCGACGCGGACATCAACTTCACTCTGAACGATTACGAGGATGATTTGAAGCAGATGAGGAACTGGACCAAGGAGGAGTTCGTGCACATCCTCCGCC
GCCAGAGCACGGGGTTCGCGAGGGGGAGCTCCAAGTACCGCGGCGTGACGCTGCACAAGTGCGGCCGCTGGGAGGCGAGGATGGGTCAACTTCTTGGCAA
GAAGTAAGCCAACCTCCTGGCTTTGACCTTTCTACTCTGTTAGCCTAGTTAAGTGTTTTTTACCCTGGTTATATGAACAAAATATGTCGAAATGATGTTC
AATTAACTTGTCATTTCACGTTGGGTTCGATCAGGGCTATATCCCTTATGTTCATGTTAAGTAGTTTCAGGTGATTGTAGAGAATACAACAAGGTGATCT
TGAAGTATCACTGATTATAATCCATGCTACTAGTAGTTTGAGCGTCTCTGTTGTGCCGGTGAAATCTCCACCCCTCTTGTGACTTTACCCCTCTACCGTT
GAATCCGGGAATCCGGAATGGACCTACAGATCGAGATATGTGCCTTGATGAACAGTGTTTCGGTGAACAGCAAAATCGGCACCCGTGCACAGTGTCCTGA
TGGACGCAACCTCTAGAGTTCACATATATGCATTGGCCAACAATGCAATTCCTGCTTTTTAGTCAGGAACCAGTTTTGTCAAAGTCTACGACACTAGGCA
TCCCTCTGTTGAGACATATCTCAGAACTGGAATTTACATGCAGGTACATCTATCTTGGACTCTTTGACAGCGAAGTTGAAGCTGCAAGAGCATATGACAG
GGCAGCCCTTCGCTTCAATGGGGGGGAAGCTGTTACTAATTTTGGGCCTAGCTCCTACAATGGAGGAGATGCTCTACCCGACACCGAAAATGAGGCTGTT
GTTGATGGCGATGCGGTTGATTTGGATTTGCTTTCACAACCTAATGTACACGACGCTAAAAGGGATAATACCTTAGCTGGCTTCCAGCTAACATGCGACT
CCCCTGAATCTTCAAATATGGTGGCCTCTCATGTAAACTTTCTTCACGATTACACCCCATTTAAATGCTCGGTTCCATTTTTTATGACTTACTACTGTTA
GTAGTAGCTAAAAAGGTACCCGGAAAAATAAAATCTTAAGATGGAAGCAATCTAGGTTGAATTGTCTTGTATCTCTGTGCGGAATCCTTGACCTTTGTTA
GTTCCTAAGCTATAAAGATTCATAGGAGCATTTGATTGGCGTGTCGTTGGTTGAGATATTAACTATTAGAGTGTCCCCAATGAGAGTTAGCTTATACTAG
CTCTAAACATATTCATGTTATTTCATATATTGCACTAAAGTTTAGATTTTAGTAACTATCTTTTAGCTTTTTTTTAAATTTAATACTTACGTGAGTATAT
ACATCTCGTTTAGTGCTATAGTTTAACTCTTCACTAAGAGTTGCTAAGTTTTTTATATTTAATCTCCTTTACATAAAGTAAATGATGACATAGACATGTT
TAAAGCTAGCTAAGGAGGCTTCTTCGGGGCTCTTAGTTCAGGTCTTATAACCTTTATTGAACTTACGACATTTGCACCTATCTGTTGCTGCTGTAGCCAA
TGAGTTCATCGTCCCCGTGGCCTGTGTATCAACAGAGCACGGCAGTACCACCTCACCATCAGCGTTTGTACTCATCTGCTTGTCCTGGCTTCTTTCCGAA
CCTCCAGGAAAGGCCAGTGGAGCGAAGGCCTGATTTGGGCCCCCAGTCGTTCCCCACCTGGGGACGGCAAACGCAGGGCTCCCCTCACATGCCGTTGCAC
CACTCCGCAGCATCATCAGGATTCTCTACCGCCGCGAACGCCGGCGTCCCATTGCCGTCTCACCCGCCGGCGCCGTTCTCGGACCACCAGTTCTACCTCC
CACCGACGGCCTGAGCTTGTTGGTCCGGTTCAACTCTTGGGACAGCAGCTGACGCTCTGTGTGTGAACGTCTCGCAGCTTGCCGGTAACAGCTGTTAATT
AATGGGAGAGAGAGAGAGAGAGAGAGAGAGAGAGAGAGAGAAGAGAGAGAGCCAGAAGACCCAGTATGGCTGACTCTGTGGCCTGTGCCTTGTTATGATCTGAG
TTATTGCTACTACTACTACACCGTGGATTCATCATGGTTTGAGATTCTTTACCCAAGTTTCAACTTGAAAGTGCAAAT
>Locus_12915_Transcript_25/30_Confidence_0.725_Length_4157
GCTCCTTGAGAAACACCATCCAATTACAAGACTATTTGATGCAACAATCACGCATATGGCTGCGCTAGAACTACTGCTCTGAATTAAGAGGCGTTGAAAT
ATTGTCCGGTCTACATATAGGCACTTGAGGTGTATTGACAGACAGTTCCCCTTAGTTTGCTACAACACATCAGGCGAATTCCACCAAATTAGGATGATGT
TTGTTTCTTAGACACAGGATAGCAATGCCAAAAGTTTTTTATAATTTTCCGCTTCACCGCAGATTTGGCATCTCAAAGGGAGTTGTGGCAAGATTCTCTT
GACCAAGCTTTCTTGCAGATTTGGCAAGAAAGATAAAAAAAATGTTGGTATACTTAAGCATCCTTAATAGAAACTAAACAGGTCTTAGATTGAAATTATC
ATAAATAAACCCTTTTTCACTTTAGCTGTAGTCCGGATGAAATCTTAGGGGCCTTGCTTAGCCTGCCCTGAAGCTTGCCAGTAAGTCGAAGCTGATGCTG
ACACAGCACTGACATAGATCCCAATGAACACAAGTTATTTCGAGAAGATAAAATACCAAGCAAAGCCAGGATTGCATTAAAAGATAGTGTATTTCTACTA
TACGATCTGAAAGCTTAATTTATTATATCAATAATCAAATTGGCAGCAGTGTGGAACAGATATAAAGAAGATGCTTCAAATCACAAATAACTACAGATAA
ATTGAAGGGTGAAGGTTCATTCAACCACCAAACACCCAAAATGTCGAGCTGCATATGCACAGAACCTAAGTACGGTACAGTTCATAGTTTGGGAAAATAG
ACTGCAAATGTGCACAGATAATACAGATTGTTACTCGATGAACAGTAAGCACGGTATTTGAAATCCTTCTAACCATCCATCCAGGCAGTGCCTCGTATAC
TCAAACCTCTTTTTGGAGGAAGCCAAACCATGTGATCCTTTTCAACCCAAATACATATATTCAAGATGAAACTAACAAAAAGACAGTTGATAAAGAAACA
CGAAAGAGAGTAGAAAAAGGGTACCTGTTTCAAGTTCTCATCTTCTGAAAATGATCAGCAGTCAATAGATGTTTTAGTCCATTCTCCAGAAAAATGAGTG
CCGCATGAATCCTTTCCGGTTTATAGTGAAACATTTTAGACGATGACCTTTAACATTCTAGAAAACGAAGAGAAAACCTACAGTCATCTAATTTCAGCTT
AAACATGTTCCAGCAAGATCATCACCATCCTGACATGTTCCGTTTTGTCCCTAGACCTGAGCACAAGGTATTGCGAGGCTAATTTAGCGGGGCAATTGAT
TTTTTGCCACTATGATACAACTGCAATTACCCGCTGTCACTGACACGTGGGGTCATCTGAGTCACTGAAATGTGGGCCCAGTGGCATTTTGTTTGCCACT
CACCCTTCCAGAGTGGCAAAAAGTTAATTGTCCCTAATTTAGCTCAATTCAGTTGTCGGAGCAATTGTGTGTGAAAAGTGTGAACAACAGCATAGGAGAC
AATACAGCAAGTGTAGCGTGCCAATATGTGTCCAGCTTGATAGTTCACTATAATAAAGTTCTTTAATTCAAGTATTTGAATAATATAGAATCTCAACCTT
CAAGTTTAAAAGGTTTTGTATTTGCTATTATAATCGTTGCCACCCCCTCACCCCTCTCAAAAAGAGAGTTACTTTGTAGCGCACTGTAAATCTCTTGATT
GAAATAGTGGCATGTCATCTTATTTAATTCAGTTATTTCATAAGGTACAATTTGTTTAGCTGACAGCTCAGACATGCTTCTATGCAACATGTTTATACCA
ATGCTGTATTTTGGCAATCCTGGATGTGGCATTACTTAACTGTGCTTTCATATTCAATAGTTCTGGCAAAGGGAGGCTGCAAGCTTGAGACAACAACTGC
ACAACCTGCAAGAAAGTCATCGGTATGGCTAGAAAACCCTGCAAATTTTACAAATGTACAAGTCAGAAAATTTGCCTAATGTAAAAGGATCAAACTACAG
GATTTGTCAGCATGAGCACAAGTGCCTAACTTCCGAGTTAGATAACTCTGGAGGAAGGAAATCCTTCCTACTGTTTGTTTCAAGAATATACAAAAATTCA

AAACAACATTAGAATAGAACCAAAAGAAGAAACAATATTCACCATGTAGCAATACTCAATTCCTGGTTGTCTTATTTATAAAGGTATAAAACTAGGTTCT
GACGAGACAATTATGAATCAACTACACCATGTTACAGATCATCCATAACTCTTGGTGATGCAAATTAAGGGCTGTAAAGTGACAAGTATACCTCCACCAA
CTAATTTGTGCAGAGTGTATAAAATTGTAAAACAATGGATAAGAATGAATTTAGTATACAAGATAGCATACAATGCCCAAGTTTAAGGAGACATACTGAT
TTACTGACTCCATAGGACAATCTTTTGCTTATGAGGACATCAGTTTTCATAAACAATATCAATGTAAAAGGAGGTAACAGTTTATGAATTTCCTATGCCA
AGCCATTTATAAGTCAAAAAATGTACCTATTTAGCTTTTATGCATTCCAATTAACTTCCAAAGAAGAAGAGTACATACTAATGGAAAACAAATATGATAT
TATCATCTGTTTTGCCCGGTAGTTTCTTGAGTCTCTGATTTGTCCGCACTTCACTCCCAACCAACACAACATTAATACACATGGGCAGAGGAATATCAGG
TTATCAGGTAAACTGAATACAGTTTGATTGACTGCAGTCAGTTGATGGGACAAGATCTTTCTGGATTGAGCGTCAAGGAACTCCAAAATCTAGAAAATCA
GCTAGAAATGAGCCTACGTTGCATCCGGACAAAAAAGGTAAGGATGCTAGCCATATTTCTGTTTTTCGTTACCAGATTGCCTCATCCAAGAATACAACTG
ATGATGACGTTTTTTACTTTCAGGACCAACTCTTGATTGATGAAATTCACGAACTGAATCGAAAGGGAAGTCTCATGCAACAAGACAAGATGGAACTATA
CAAAAAGGTCAACCTAATCCGTCAGGAAAACATTGATTTATACAAGAAGCTCTACGAGAAAGAGGTGACAAGTGAAGTCAACCGAGATTCATCAACTCTA
AACTTTGCAGTTGTCGAGAACGTCAACATTCCTGTTCATCTTGAACTTAACACTCCACCGCAAGAAAATGATGTTGAGCAAACTGCACCTCCTAAACTGG
GATTACAACTAAATCCATGAAGGCATGCAGCATGGCACCGCTTATGTTCATTATTCTCAACGGTACGATTCAAAGCAACCAGAAACACTATTGGTTGTAA
AGAGCTTAAACACATAGCAAGACTGACGCGGTATGCATGCCATGAGTGATTGATAATGGTAGTGTAAATCTATTTCAAATAAAATGTATTTTATTGAAGA
ATACAGCAAATGATTTGTATTATAACTATTTGACACATTCTGCAAGTTTGTTGACCAACGTGAGTACATGATAGCTACAAATCTTATATTGACAAAAAGA
TATGGTGTGTGGAAAAAATTCAGGTGCTATTTTTTTCTATTTTGAGAATTGTATGCATAGCAGAAGAGCACAAATTGCATTCTGGTTTATCCGTGCAGGC
ATTCCAGAGTATCAGAATGGGGATCAACCTTTCTTCAGCTACATATGTAAATATACGGATGCTAGTTGCTCACTTAAAATTTTGTAGTGTTCAAAGGAAT
AACAACAGTAAGACGTTGACGCACCGAAAATAACCAGAAATAACGGATTGTACTATGACAAGACACACGACAGATACACTAGCTTAAGTTTGTACAGGAA
AATATTTGGCCTATTCTTATGTCACAGTAAATTAAGATTATCACCTTAATCACAGAAATTCAAGGGGTAAACGATTATAGCACAAAACGATCCTGGACCC
CAAGACCTGGGTCTCTGAACGGCTGAACCTCCAATTATACCTCTATACATTTGGAATGTGTGTAGTCAAACTTTCGCTACTGGTATCTTTACAAATATTT
ACATTGAACACATCAAAATGATATACATGGATTCCTCTCGATATGTCTTTGAGGTTTGGGTTCTAAAAATGATATACAAATTATTAGGTTCTAAACTTAA
ATGATATATACACATCAAAATGATATACAAATTATTGGGTTCTAAACTTAAATGATG
>Locus_2090_Transcript_216/268_Confidence_0.014_Length_676
ATCTGCACCACCACCAAGCCGTAGATGTGTTGAACCTGAGGAATTTGCGCCATTTGTGGTGGACCTGGTGGAGGCATTGCGAGACTTCAGCTGCGCAAAT
TCCTCAGGTTCAACACATCTACGGCTTGGTGGTGGTGCAGATGGAGATCAAACCATGTCGGGGACGAAAACAATGGCCTCACAAAACAATAGCAAAAAGG
CTGTTTCTTCTTCTGCAGCTCTTGCTTTCACCGTGACTCCTATGGTGCGCAAAGATGACACCTTCTTCCAGCACCTCAGCCACAATGTCAGCTTCTCCAG
CCATTTTGGCCAGGCAAGCGCCCAAACCGGCGTTGGAGGCATCCATAATGAGACCCAGCATAGGGTTTCCGTGATACAATGACGGTTTGCGAAGCTTATC
TGGTGATCCAGGCTTCTAGGTGTCCTCGTGTCCGGTGTAGTCTTGGTCGCTCAGGCAATTCATCCTTGGCTTAGTTTTTGGTGTGTTAGAACCTACAAAT
GTTTCATACTCTGTTGTGGTTTGCAGAGCCCATAAATCAGGGCTTGATGGTGACAGTGACACTACTATCCAAGTATTGTTGTAATGGTGTGTGTTAGAGT
AAACCCTTCTTTGTGTTCATGTTCGTACATTATGCTGCACAATTTGCTCTTTTTTCTGTCACTACTACAGTCTGGT
>comp82796_c1_seq18
AAGATCACCCTGGATTCCTGGAACAAAGAGGGCCTAGAGCAACGCAGCAACCTTCGCCACTTGAAGAGGATAGGTACAAAAGCAGCCCCATGGCCATGGC
TTAGCTAGCACCTTGCAGTCAGGTAGAATTATTCAACCGGATAAAGATTATTTCAAGTAAAGGAGGGCTACCAGTTTATACCTGTTCAGGTGGGTGCAGC
AGTTGAGAACTTTTTTTCGATACAGAGCAGTTGAGATCTTTAATTCTGGCATGGGTTCCTTTGGGGTGAACTGGAATCAGAAGAACTCCGCGGTGTGCGA
TTGGGAAAATTTAGCACCTTCTGTTCCAAATGCAATCGGAAACCCCAGGAACGGAGCTACGGGGCATGGATCTGTGAATTCATCTGGTGGCACTCTCACT
TCTAGCTCAGAGCTAGGGCATGGTTCATCCAAGAGCTCCATGTCGGCATCCATTGATTCACCGTCCAAAGTAGGGAACAGCTTGGAGTTCAATTTTGCTG
CTGTCAATACGTATGGTAAGAACATGGATAAGGATTGTAGAGTTGACGACTCAGGGACTTGTCCATCGTCCATGATAGCATTCAGCCATGGAGAGCCATT
AATCAGTCTTAAGCTTGGAAAAAGGACTTACTTTGAAAATGTCTGCGGAGGACAGGATTCCAAGAGCACTGCACCTTCTACCAGGACTTCTCCATCAACC
GTTCTCAAGAAGACCAAGGTGTCTCAGCAGAATACACAAATGTCATACTGTCAGGTTGAAGGTTGCGGAGTTGATCTATCTTCTGCTAAAGATTACCATC
GCAAGCACAAAGTCTGCGAAGCTCATTCTAAAGCTCCGAAGGTGGTTGTTGCTGGTGTACAGCGCCGTTTTTGCCAGCAGTGTAGCCGGTTTCATGGCTT
AGCCGAGTTTGACCAGAACAAACGAAGCTGCCGTAGACGTCTTACTCATCATAACGCACGAAGGAGGAAACCACAGGCGGATACAATTTCATTCAGTTCA
TCACGTCTCTCAACAATGTTTTATGATACAAGCCAGCGGACAAATCTTTTCTGTAGTCAGCCTCTTTTTGGCCAAGTGAGAAGCAATGCAGTTTCTTCAT
GGGATAACTTGGGAAGCTTCAATTTTATGGAAACAAAACATCTGTTGATGCAGCCAACGAAAATGTTAGGTCTTGATGGGCTGCATTTCTCAACCCCCCA
GATATCAAATAGTGTTGTGGCTCACTCTGTACATCATCATAATTTTGATGGGTTCACGCCATTCAAGGGAACCAACACAAAGGTCCTCAACCAAGGTGTC
GAAGCTTCTGCGATCGCTTCCAACTCGAGTGGAGACCCGGATCTTGGGTGTGCTCTCTCTCTTCTGTCAAACGGTTCGTGGGGTTCAACTTCAGCCGTCA
TCCAACAGCCTAGTTCTCATGCGCAAGCTGGTGCGGTGCCACCCTTCGCCACCGTCGCCGTCTCCAACCCTGCAATGCATCCTCTGGACTCATCCCCGGG
AGGATTCTGGCAAGACGTCCCTCCCCCGCTCGATGAAACTCCACATATTCAGGCATTCACGCACCTCTGATCAAATGCTGCATCCCCTGGGAACAGCAGG
TGGTTATACCTTCCGTAATCGTCCTAGGTTTGCTTTGCCAGTGACTGGCTCTGCACAATGTCGATCCATCACCGGAGGCATTTGGTAAGGTGAAGACTAA
AGACTCAAGAATAACCGGAGACTAACTCGCTTCTCTTCCAGGCTCAAGACTGTTTGGGTCCAGCGGTGTTGCTACCTGATCCTGTTTCGGTTCTGAGTA
CATCCATTACTAATCATGGGATTACTTGTTTAGGCCAACCAGACATATTCTGGGGTGAGATTACGACATGTTAATTTACATTGACGGTGTTTCTACATGT
GTTAGTACTAATGATGTTATCGCTCTTGGCCCCGTTACGTTTCAAGATCTGTTACCATTTGAGT
>Locus_10795_Transcript_53/70_Confidence_0.480_Length_2677
ACATTAATCTGAGGTCTGAACAATGTTCAATTACCACATCAGAAGCTTCTGTTGAATGTTTATTTGAATGAAACTTTTGAATGCATATCATGGGTGGAGG
AATCGGGCGGCCAAGAACTGGGCAGACATCACATCCCAACTTCCTTCAAGCAACTTATGTCTAGCAGTCTAGCTAGAAAAGGAAAATAAAAGAGAAAAAA
AAACTATGTACAGAGCAAGGCCATCATGTCGGCATGGACCTCCGGTAGGTGCTCAATTACTCAAATCCTATTGCAATCCTGTAAAGGATGGCTTTATTCT
AGATCTGCATAGGTTACCACCTGCAAGAAAGCCACAACAAACAGCAGCAGCCGAACAGATGCTCACCTCCCTACTGCGCTAACTCAAAACTGGCAAGAAG
TGCCCAGCCTCTGCTTCGCCCTTTGCTCTGTAGCGCAGCACTTGCGCGAGCGAAATGCCTCAGGCAGCAGCTCCTGGAACTTCTCAAGGAACAACCCCCA
CGTCTCTGGTCCCATGTCAGCCAGCTTCACGAAACACACACTTGTTGGGAGCATATCGTTTGCACTACCCCAGTTACCAGTTTGGTTGCTGTTGCTCTCT
GAAGTTTTCACAGAATTCACACCCTTTGGAACAGCAGACAGCCGATGCTGGCTTTTGTCAGTTAGAGTGACACCCTTCAGTGAGGTTGACAGCTTCGAAA
TGTTAGCAAGCCACTTATTGACAGGTTCAGGGCAAGCTAACTTTCCTGCATTGCTTTCTACCTCCTCCTCAATGTCGTTGTCGTCGTCGTCATCATCATC
ATCATCATCATCTTCTTCTTCTTTCTTCTTTGGCCTCATCCAGTTTAGACAATGAGGTTCTGGAGGTTTGCCCCTCCCCTTACATTCAAAATGGTAGGCT
GATGGTTCTTTTGGCATTTCATGATCTTCACTATCGAGTTCAAATTGAGTTAAATTCTCATTGCCAGTGTCATGTTCTATGGAACAGTCTTCTTGTTCTG
TTGCTAGCCTCCTTGCCTCCATGCAGACAAACTCTAGCTCACTTGGCTGATCTTCCATCCCAGTAAACTCTCTGCTCATCATCTCCCCAATCTCTGCAAG
GCAGAGACCAAATGCTGTGGCTTCTTTCAGAAATATGGTTGAGAGTATTAGTACTCTGAGGCATGCTTCATGGATCATAGGCAACTCCATACGAAGCATG
TCAGAATCTTTGATTGGATCAAGATTTGTGATGTACTCAAGCTCATCCTCGGAGAATGGGATTGATGCCTGTGGCCAGTGGATCCATTCAAAATAGGGAT
CCTCCAAACACTCTGGGAGGCAGAGACCATGATCAATAGGAATAAGCTCAGTCTGATTCCCAAATTTTCCTGCTCCAGTCTGCTTCCTTACCAGAAGATT
TCCAGCATGTCTATCAGTATTGAAGATCCTGATGTCAAGAATACCAATCCTATGAACAGCAGAGACAGGAAAACTTGAAGTGCCGTAGTCACTGGCATCA
AAATCATGAGGAATAAACTGCTGAAATGATGCAATCTTGCTAACAGCTTGTGTACCACCATCAGCAGCATTCTTGTTAGCACAATTGACGCTTTCATTCA
TATGAAATACAGGATGAGAAATCTTAACAAGAACTGTCAGAGGGACATTAGCAGAGTTATGATAATCCAGAAGGTAGGCAGCAACCTCTCTGAAACCAGT
CTCCCCGACTCGAACTGATCTCTTGAGACCTGGCTGCCCAAGGGCTTTCCCTGTAAAACCTTTCGGATTGTTGGGTGCAAATGGCTCCTCATCATTTGGC
TTCACAATGGCAGCATTCTCACCTTTGCTATTCCTGAAATAGTAGGCACCACCAAGCCCACTACTGACAGGTATGGGATCCACACCGTTTCTTATAGCCC
TAGTAACATCCTTCACAAGCTGCCTTGTTCTAGAGCAGCGATTCGGGCATACTAACAGTTCAATTGGACTACCCCTATCTCTTTGCTGCTGGAGATCCTT
GCCTGTAGGAGATGACATGCATGGTGTGGAGGAACTCCGATGCATGAAACTCTTGGTGAGAAGTAACGGGGAGTCATTTCTGATGGTGCTGAGGTCATTT
TTTAGGACACGATCACCTAATGTCAGAGAACTCTCCTCGGTGGGAAAATTGAAGGCCACCTGCAACTTTCTTTTGACTGTGTGTGCATTGTCGCCACGAT
CTAGTTGAACAGCCAGGACACAACCAGTGTCAGTTTGGACAAATAGGCGTTTCCATCCGCCAGCCCTGGTCTCGCTTCTACTTTTGGTAGGGTACTCAGC
ACTAAGAGCTCGGCCCAAAGCTGAAACTGCCATCTGGGTCTGCACTGGGCTCTCCAAGTTGCGAGACATGGCGGACAACGACAAAGGAATTGAGGCCAAT
GCACTAGAGGCAGCTGCCACAATGAGGCCTTTCTTGCTCCTCTCTTGTTTTCTTCTGCGCTCGTCAAATTCAGCAGACGAGTGCGAGAGGGATGTCACGG
CAGTGATCATCAGGCAATGAAGGACATCTTGCACACTAAACAGCACCAGAAAGACAATAAATAAGGAAGGTGAAGACAGCAGCAACCCTCCTACAACTGG
GAGCCCTTATATATGAAACCCGACAAACTAGATCCTGATCCAATGTGGGGCCTCGGGAATGGTGGCCTGCCGCCGGC

>Locus_839_Transcript_84/261_Confidence_0.073_Length_1998
CAGGAAGGGGAACATGCTCCCTTCTCATCTCACAGGTCTTCCTCGCTGACTCATCTCTCTCCAGCCTCCCTGATCTGCGCTCTATTTCTTCCCTGTGGTG
TTAGTGTGAGCGGAGCTCCGCTCCCAGCCGAGCGGGTTTTCGAACCGTTTCGCGACTCTGGCTGGTTTATGGTGGATCTGGCTGATTTCTTGGTTGCTGG
GTTTGTTTTTGAGTTGATGCGGTGCTGAGATTTGTGGGTGCATTTCTGGGTGGAAGTCAGGTGATGGCTGCAGTGTGTGCCCTGTGCTAAGGCCCTGTTC
GATTTTGTTAAAGTACACCGCATGTTGGATATGCGCTTCTTAATTGTTTGTTTTGTTGGTTTCCTGTGTTCCTGCCTGTGTAATCCGTGTGAATTCACGG
GTGTCAGTTGTGCTTGGCTAGTTATGGTTAAGGGGAAAGGAGGTGATTTGGACAGGGAGCTTGCCACGCTGCAGCTTTGTTGGATTTAAGTAAAATCTAA
GTAGCAGAGATTTTGACATGCAAATTAGTAATGGTTCGCGAATTATTTCAAGAAAACAAAGTCTTACACATAATTGTTGCATGGGATTGTATGAATTGGA
GGTGGAAAGAACCAGTGGCCTTAAGATGCAGCGCCAATACGCATTTGGGCCTAGTGGCAAAACTAACTAGTCATGAAGGCTTGTTTCAGGCTTTCAGCAC
ATAATTCGATTGCACATATAGTTGTTGCGGTTGTGAAGAGACTTGGTTGCATGAACGATCCGAACTTTTAGGGTGTTACCGAGTTGATGCAGGGTACTTA
CTGATGCAAAATAATAGCTATCTTAGATCATATGCATGTCAGTTTGAGTGAATTTTTGTATGGTTTCATGTACCAGCGGCTGTGGATATTAGGCAGACTG
TATTTGTTTCAAATATTTTATCATTATATGTTGTTAGGACTGATCTGTGCTTATATTTGGACAGTATGAACTGAAAAACAGGAATAGAGAATTTCCGTGA
GATGAGACAAAATGGCACCATAATGATTCAATTTGGTCATCAAATGCCTGATTACGACTCATCAGCTACCCAATCGACCAGCGAGAGTCATCAAGAAGCG
TCTGGAATGAGTGAAGGAAGCTTCAATGAGCATAATGATCGATCAGGTAATCTTGATGGTTACACGAAGAGAGATGAAAATAAGATGATATCAGCTTTAT
CTCTGGGCAATCCGGAAACTGCTTATGCACATCCAAAGCCTGACCGTAGCCAGTCCTTTGCCATATCATACCCGTATGCTGATTCATTCTATGGTAGTGC
AGTGGCGGCTTATGGCTCGCATGCTATTATGAATCCCCAGATTGTGGGCATGATGTCATCTTCTCGAGTGCCATTACCAATTGAACCAGCTGCTGAAGAG
CCCATTTATGTAAATGCAAAGCAATACCATGCGATTCTCCGAAGGAGACAACTCCGTGCAAAGTTAGAGGCCGAAAACAAGCTGGTGAAGAGTCGCAAGC
CATACCTTCACGAGTCTCGGCATCAGCATGCCATGAAGAGAGCTCGGGGAACAGGTGGGCGGTTTCTGAACACGAAGCAGCAGTCAGAGGCTCCTGGCGG
CGGCACCTCGGACGCGCAACTCATGCCACCAAATGGTGGCCTGTTCGCGAAGCACGAGCACAGCTTACCACCCAGTGATCTCCATTATCGCGCAAGAGGG
GGCGCTTAAGGAGTCCCATGGCAACTCATCCTTGGCTTAAAAAAAATGTGGCGCTCAGCAATTCGACCTCTGCTAGTTCAATGTAAAGGTGTGGGACT
GTAACTGTGGGTGCTTGAATTGGCCGCAGCCATTCCTGCGACGCGTCGCAGGTGTTGCCGGTTCTGTGGTCGTGCTAGACTTGTGACCGGCACGACGAAA
CTGGCACCTGTTTCCTGTAACTGTTGTCTAAGATGATGATGACTACTCCAATAACCATTCTGTATGATGCTTTGACGTCGGTGTTGCTCCTTCAAGTC
>Locus_7001_Transcript_66/84_Confidence_0.123_Length_1521
TTTTGATAAATAATAATAAATAATAAAATTACAATACTATTTATCAAAACTCAATTTACTCTTTCTTCGTGATTTTAGGGGGTAAAGGCATAAGAATCGG
ATCCGTTGAGTGCGGATTTGGCTGCAGGGGAGGGGCCGCTTCGTCATACTTCAGCTGTAGCGTCATTAAACCTTTTCCGGACACGAGAGCGAGCGAGCGA
GAGAGAGAGGTGGACTGGATTTGGGGGAGGACGGAGAGGAAGAAGCAAAATGTTTATGGAGCTTGAGTGATGCATCTGGTAACGAAAATGCTTCTGCGTG
AAATGGATGGTTATCCAGTCCATTCCATGCCCAACTATGATTTCCTGTCTGGGAATGGTTATTCAATGAAGCAGTTTATCCTTGGCAATCCTGATGGAGA
CTCCTCATCAACAAAGTCTGAGCAGTGTTGCCAAGATTTATCTGCAGTGAGTGACAGCAGCCTCAATGGACAACACACACCAACACAATCTGACAACAAT
GATAGTTGTGGGAAGGTAGACCAGGGCATGGTAAAGTCTGTACTGTCCTTGGGAAACCCAGAAGCTACATTTCCCCCTCCAAAGTTTGATTACAGCCAGC
CTTTGGCTTGTGTTTCTTATCCGTATGCTGCTGATCCATATTATGGTGGGGTCTTAACAGGATATACATCAAATGCTGTTGTTCATCCCCAAATTAATGG
TACAGCAAATTGTAGGGTCCCATTGCCTATTGAACCTGCAGCCGAAGAGCCCATATTTGTCAACGCAAAGCAATACCATGCAATCCTCAGGAGAAGGCAA
ATGCGTGCAAAATTTGAGGCCCAAAATAAACTGGTGAAAGGCCGGAAGCCGTACCTTCACGAATCTCGACACCGTCATGCAATGAAGCGAGCCCGTGGGT
CGGGAGGCCGGTTCCTTACCAAAAAGGAGCTGCAGGAGCAGCAGCAGCAGAAGACACTGCCTTCACTTCAGACTCCAACATGTGTGGCAAGTAAAATGTC
ACTCGGCAGGAATCAATGCCCTGAAAACAGCACATCTCGCTCGCCTTCTACACCGACAGACTCTGGGATCTCAAGTGTTTCCAACGGAGGTGGCATGCTG
GCTCACCAAAAGCACATCACCTTCACATCTACTAATTTCCTTCCCAGCATGAACTTCAGCACCCAGAATGGAGGTGAAAAGATGGCTGTCAATGGTGCGC
ACCACCGTGCCTCCATCGTGAGGTGAACAGCCTCGATTGTGGGGCGGTGGTATGTACCATCACTGGCGCGCTCCGGTGAGCCCGTGGCAATTCATCCTTG
GCTTATGAAGTATCTGTATCTGGTAATAATTTGCTTTCAGTTTAATATGTAGAGCTTGTCCGAGATGAACTCATTTATATTTGTTGCAACTAAAACGAGG
AGCTACTTGTTGATATATGTGGATATTGTGGATACAGAAATATCCTGATGTTTCTTGATGTACCAAGTCCACTATTAAAGTATGACTGGAAAAAACGTTT
AATAACGTAATCATGGTTTGG
>Locus_10929_Transcript_70/78_Confidence_0.237_Length_1363
CTCCTACAATCGAGGAGATGCTCTACCCGACGAAAATGAGGCAATTGTTGTTGATGGCGATGCGGTTGATTTGGATTTGCTTTCACAACCTAATGTACAC
GACGCTAAAAGGGATAATACCTTAGCTGGCTTCCAGCTAACATGCGACTCCCCTGAATCTTCAAATATGGTGGCCTCTCATGTAAACTTTCTTCACGATT
ACACCCCATTTAAATGCTCGGTTCCATTTTTTATGACTTACTACTGTTAGTAGTAGCTAAAAAGGTACCCGGAAAAATAAAATCTTAAGATGGAAGCAAT
CTAGGTTGAATTGTCTTGTATCTCTGTGCGGAATCCTTGACCTTTGTTAGTTCCTAAGCTATAAAGATTCATAGGAGCATTTGATTGGCGTGTCGTTGGT
TGAGATATTAACTATTAGAGTGTCCCCAATGAGAGTTAGCTTATACTAGCTCTAAACATATTCATGTTATTTCATATATTGCACTAAAGTTTAGATTTTA
GTAACTATCTTTTAGCTTTTTTTAAATTTAATACTTACGTGAGTATATACATCTCGTTTAGTGCTATAGTTTAACTCTTCACTAAGAGTTGCTAAGTTT
TTTATATTTAATCTCCTTTACATAAAGTAAATGATGACATAGACATGTTTAAAGCTAGCTAAGGAGGCTTCTTCGGGGCTCTTAGTTCAGGTCTTATAAC
CTTTATTGAACTTACGACATTTGCACCTATCTGTTGCTGCTGTAGCCAATGAGTTCATCGTCCCCGTGGCCTGTGTATCAACAGAGCACGGCAGTACCAC
CTCACCATCAGCGTTTGTACTCATCTGCTTGTCCTGGCTTCTTTCCGAACCTCCAGGAAAGGCCAGTGGAGCGAAGGCCTGATTTGGGCCCCCAGTCGTT
CCCCACCTGGGGACGGCAAACGCAGGGCTCCCCTCACATGCCGTTGCACCACTCCGCAGCATCATCAGGATTCTCTACCGCCGCGAACGCCGGCGTCCCA
TTGCCGTCTCACCCGCCGGCGCCGTTCTCGGACCACCAGTTCTACCTCCCACCGACGGCCTGAGCTTGTTGGTCCGGTTCAACTCTTGGGACAGCAGCTG
ACGCTCTGTGTGTGAACGTCTCGCAGCTTGCCGGTAACAGCTGTTAATTAATGAGAGAGAGAGAGAGAGAGAGAGAGAGAGAGAGAGAGAGATAGCGGAGAG
AGAGAGAGAGAGAGAGAGAGAGAGAGAGAGAGAGCCAGAAGACCCAGTATGGCTGACTCTGTGGCCTGTGCCTTGTTATGATCTGAGTTATTGCTACTACTA
CTACACCGTGGATTCATCATGGTTTGAGATTCTTTACCCAAGTTTCAACTTGAAAGTGCAAAT
>Locus_14339_Transcript_58/63_Confidence_0.083_Length_2981
AGCTCTGGCAAAGGGAGGCAGCAAGCTTGAGGCAACAACTGCACAACTTGCAAGAAAGCCACAAGTATGATCTTCCAATTGATTCTGCAATGAAAATACA
TCTCATCAATACAAAGTTTAGATGCCACGTGGAAAATTTTGTAATAACTGTAAAACCAAAATGAAACGTATCATCCAGTGAAGATGAGGGAGAAAAAAAT
CTAGTTACTGAAAGACATTTATAGTGTTCACTATTCAGAAAGGAAATTGAAAGTGGCTTTGTCACAGCAACATATATGTGGTTTTGGTTCGAACCGTGTA
CCCACGTAAATTGATATTGGTTTAGTTTCAATCTACACAAGATAATCTGACGAACTCTTTTGCATTGTTGTAGGCAACTAATGGGAGAGGAGCTTTCAGG
CCTAAGTGTGAGAGACCTCCAAGGTTTAGAGAATCGTCTTGAAATGAGCCTACTTAGTGTCAGAATGAGGAAGGTCATATTCATTTTATTTCCACATTCT
GTTTTTCTGTTCACTTCAATCGAATATGCAGTAAACATCTATCAAACAGGGCCACCTTTTGACAAGTGAAATTGAAGAGTTACATAGGAAGGTTAAGCCC
TACAATGAATGCCCTTTTATTATTTCAAACAAATGATCATGCATGGAAGTTGCTGACATCTATACTGATGATGAAACAGGGCAGCCTAATTCATCAGGAA
AACACGGAACTCTGTAGAAGAGTGAATATCATGTCACAACAAAAAATGGAACTTCATAGAAAGGTTTGATGAACTATCTTTTGCAGAAACTTCAACCAAA
CGAAGATGCATTGTCAGCAGAAAGCTAAAATGACTTAATTTCAGCTCCTGGCCAGTGAACCAGGAGGTGTTGCTGATGCAAATAAAAGCTCTAGCACTCC
CTACGGCTTTGGTACAGCACAAGGCGCAGATGTCCCAGCTAATCTTGAACTGAGCCAGTCATCACAAAAAGAGGGAGAGCAATGCAAAACAGGGGCCCCG
GAACTGGGGTAAATATTCTTCTTTAGGATCCAAAATTCCCATTACTCCAAGAAGTTCCCATTACTCCAAGAAGTATGAGGAAGCTTAATGAGAACTCTAT
GCTTTAATATGTAGCAGACTTCAGCTGCACTAAGGAGAAGCAGTACAAGCTGGACAATGCCATAATGGTCCACAAATTTCACCATGCGATGCAAGAGCAT
TTTGGTTGCAATGACTATTTTGTGCAATATACTAGCTGGGTAAACAAATGGGTTGTGCAAAGACTACAGATAGGACCAGTTATGTTATCTGAGGCTCTGC
TGTGTCTAAAATGCAACATATTCGTTATTGTAATGACACATGATAAACTGACTGTCCTACCGTTGACCTTGAAGATAGCTCTGCAGTGAAAAGGTGTATT
ATGGGCTTGCCAATATTAAAGAAGTGTTAGGGATCAACTAGTAGGATCTCACAAAGAAGTAATTAGTCCACATTAGTGCCAGCTGCTGGGGATGATATCT
GACTCAGCCTGCCAACCCTCTCTTAGCCATGGTGTTTCAACTAGTATTAGAAAAGAATGTTCATCCAATTGAGTTCCCAAACAACGAAATTTATATTATG
TGTTGCAAAGTGTCGACACCAGTGGAACAGAGTCCATATATGTCAAGCAAATGCCAAATGTCAATTTTGCTCCAGTGCACGTGGATTCTGTTCTATTCTC
AAGAATTTCAACCCTTGATAAGGCCCTTTTTTTCCTGGTGAAAGGATTAACAACGATGAAGCTTCGAGATGGTTTAGTGATTGTTAATAGAGGTACTTAGT
GCATCCATGCTTGAAAGTGTTCTTCCTTTCAGCGAAAGGTTTTCTATTCTACTTGCTTGTTATCTTTTGGCCAGTATGTCAAGCAAGAATTTAATCTGCT
AATCAAGAATGTAATGCGTTCTTTTAACATGAATATCAACAGTTTTCAAGTAACTGAAAACAGTAATGTAGTATTCTCTCGCCGAAAATAAAGCTGTGGT
TCCTTGATAGCAACCGGCAGTTATGCCAATACCACATTAAAGTGGTTGTGTCCCTTATAATACTCTCTCCATTCTACTTAATTCAATGCTATTTGTTTTT
TAACGCATGTCAAGATCTGTCTTTGACCATTCCTTTAATCCTTTTGAATATATATTTTTTTAAAAAGTGGTGATATAGCTTTCGTAGGGCCAATATAAGA
TTTTTTAAGAACATATTAGAGTTGGTCGTGTTTAGGTAGAGCTGTGTGAGGGTATGTGGTGATGCTAATCTGTAACAAGACGGTCTAAAGATACCGCCTT
GCGCCTTCATACTTGTGCCGAATGTATGTAAGCTGGAAACCCCAGCAAACTACTCTTTTGCTTTCTATAAAGCAGAGCAAGGTGCTTATGGTCTAAAAAC

ATATTAGCAGCCAAAATTCAGAAAGTTATAGTACAAAAAAAAAGTTTGGCTACACAAGTTCTGGAATATCAATGTTCTTAAGTCTAGAAGTTTGACTACA
CTGATTCTAAAGCATCCAATAAAATGGAACGGAGGGAGTAGTAGACAATCATCATGCGGCGTCTAGTTCTTTGTAAGAACCAAGGTATGTGGATTTGGCT
TCAAATTTGCGCTTCCTAAATTCAATTTAAAAATGTTTGTGTTCCAGTTGAAGCACACCACGCGAACGCAGAAATTATGATGTATTTCGTTACCTATAAT
TTGGAAAACATGACATGATATAGATTAGGTAATAATGAACAAATCCTCAGCTGCTCAGAAAGTTACAAGGGGATAAAGGCCCATAAAAGAGAATTCCAGG
ACCAGAGCTGCCACAAAGATAATCAACCCAACAATTTTTCGACAGCACTCATGGTAATACAAAGGTAAAAAGGGACGTGTCTTCAACAATAGCTTGACGA
AACCACTGAAGCAATAATAATACGACTTCGGACGAGCTATGTGCGTTGGTGTCTTACTTGTGACTTCCTTGCAAGTAATGC
>Locus_2976_Transcript_20/49_Confidence_0.231_Length_1926
AGTCCTAATATGTATCGCTTCAAAAAAATAAATTTCAGTATATCAAAGTTTCATGATTGGAGCAGCATAGCCAAGCTACACTGATTGGCATATGAAGGGC
ATTGTACTCACTATGGTGCTTAGGGCAGTACAGGTAAATTGCACTTGACAAAATAGTATCGCTCAATTGCTGTGACATGTTTATTAACATGAGATCACAA
CGTTTGAAGGGAAACTCAAGCTAAATAACCTCACTTCAAAAAAGTTGGAATTCATGACTTTGGCCTGGATCCGAAATCTGTAAAAGAGCACTGCTCATCT
TGTACCAGTGAAAAATAAACTTCAGATCGGTATGCTACAAAGCTATTTGACAGATGCTTTTACAGCTTTCTCTGCCGCATCATCAAGATCCTCAGCAGTG
ATCAGTGTCATTCCACTTTCCTTAAGAATCCTCTTTCCTTGGTCCACATTGGTGCCTTCTAACCGAACAACAACAGGAACCTTCAGGTCAACCTGTTTAG
CAGCATTTACAATTCCACTTGCTATCACATCACATTTCATGATGCCCCCAAATATGTTCACTAGAATTGCCTTCACTCTATCATCTGAAGTCAGTATCTT
AAATGCTTCCACAACCTGTCCCTCTGATGCACTTCCACCAACATCAAGGAAATTGGCAGGTGTTCCGCCATGCAACTTGATAATGTCCATGGTAGCCATA
GCCAATCCTGCTCCGTTCACCATGCAACCAATCTCTCCATCAAGACCAATATAGTTCAAATCTGCTTTGGCAGCAGCAACCTCCGTCCCTTGCCCGCAGC
AGAGAGAGAGAGAGAGGAGTCGTCGTCGGCACCGCGAGCTCCAGCGAACGCCGCCGCCGCGATGGATCCGAAGCTGACGGAGGTGACCCAGATCTTCGCC
CGCTTCAAGGCGGCGTACGCTCGGAACGACCTCGACACCTGCGTCACCCTCCTCTCGCAGCTCAAGGTCCTACTCACGAAGTTCCCTAGCCTTCCGCCGT
CGTTTCAGCAGACTACGAACGCGGTAGAGGAGCTAAAACTTGCAAGGGACATTTATGAGCATGCAGTTGTTTTGAGTGTGAAACTTGAAGACCAGGATGC
TTTTGAACGTGATTTCTGCCAGCTTAAGCCTTATTACATGGACACATGTGACATAATTCCCTCATCACCGGAGGAGTACCCAATTTTGGGACTTAATCTT
CTGAGGCTGTTGGTCCAGAATAGAATTGCAGAGTTCCACACTGAGCTGGAGCTTCTGCCTGTCAAAGCGCTCGAGCATCCTTGCATCAAGCACGCGGTGG
AGCTTGAGCAATCGTTTATGGAGGGTGCCTACAACCGAGTGTTGAGCGCTCGACAGTCTGTGCCCCACGAGACGTATGTTTACTTCATGGACCTTCTAGC
TAAAACAGTCAGAGATGAAATCGCTGGTTGCAGCGAGAAGGGGTATGATTCCCTATCCATAAGTGATGCGAAACAAATGCTGATGTTCAGTTCGGACCAA
GAACTGCACCAGTACATCACAGAGGAGCATCCCGAATGGGAGATTAAGAACGGCGCCGTCTTCTTCCAGAAGGCGAATGAGTCCCAACCCTGCAAGGTGA
TTCCTTCGCTGCAGCTGATCAACCAGACGCTCAGCTATGCTAGGGAGCTGGAGCGCATTGTATGATTTAGTTGCACAATGATCTCGATGAATCTTTTTTC
TGTGGTCAGTTGGGATGACCTGTACTATCATTACGTTTGAGATCAGCCCGAGTCACAGCTTTGAACTGACTGAAACATCCTATGACTTTGTTAAGGGCGG
GGCCTGTCAGTCATTGGATTGTTAACACCGTTTGTATTTCACTTTGATCAGTCACTTTATTGTTTTCAAATGCTCGGCGTTCATTGCAACTGCAAACTTT
CAGGTTTTAGATGCCGAAGTTACATT
>comp83375_c0_seq3
TCATAGAAATCTGTATAATCCCAGAACTGGCTGCTCTGGCCACAAAATGGTTTATGAAGCGAAATTCCGAAGTATCTTTGTGGGTGGAGTATGAAGCAAA
ATCCCAAACAAAGAGAATAACCATGGTTGCTTTTCAAGCAACAGTTCCAACCTTCAATTTTTATCGATTTTCTGCCACCGATATGTTTCAAACTTGTTAG
TTTTAGAACAGATTGAGTACTAACTACTGAAGTGAAACTCATATACCTACTGGTCAATTCTATGTAATCGTGTCTTTTGAAAACAGCTTATCATGTCAGC
CGTCGATCAACTATTCTGCTGTTCCAAAAAAGAAATTTTAGGCCATGAAGGAATACGTTGGATACGATTAGATCTGAATGTGTCTGATAGATTTTTGTAG
GCCACACGAGAGAACACCTTTATAGCAGACATAGATGTCTAAGTACAATTCAGCCTATCAGGATAAATAAATATATATGCTTGAAACATTAGGGTGTTTC
AACCTAAGTAGATGATGCTAAATTAATTGGATAATAGATTATTCACTCCTTATTTCAATCATTTGACTTATGACGAACAATTTATTCACTTTTATGAGGG
GCAACTCGATAGCAAGTTGAATTGTGTTAGGAGCTCTGTTGCGTTGCTTATTGACAGCTTTCTTCTCCCTCTCAGACAACAATGATAGTTGTGGGAAGGT
AGACCAGGGCATGGTAAAGTCTGTACTGTCCTTGGGAAACCCAGAAGCTACATTTCCCCCTCCAAAGTTTGATTACAGCCAGCCTTTGGCTTGTGTTTCT
TATCCGTATGCTGCTGATCCATATTATGGTGGGGTCTTAACAGGATATACATCAAATGCTGTTGTTCATCCCCAAATTAATGGTACAGCAAATTGTAGGG
TCCCATTGCCTGTTGAACCTGCAGCCAAAGAGACCATTTTTGTCAACGCAAAGCAATACCATGCAATCCTCAGGAGAAGGCAAATGCGTGCAAAATTTGA
GGCCCAAAATAAACTGGTGAAAGGCCGGAAGCCGTACCTTCACGAATCTCGACACCGTCATGCAATGAAGCGAGCCCGTGGGTCGGGAGGCCGGTTCCTT
ACCAAAAAGGAGCTGCAGGAGCAGCAGCAGCAGAAGACACTGCCTTCACTTCAGACTCCAACATGTGTGGCAAGTAAAATGTCACTCGGCAGGAATCAAT
GCCCTGAAAACAGCACATCTCGCTCGCCTTCTACACCGACAGACTCTGGGATCTCAAGTGTTTCCAACGGAGGTGGCATGCTGGCTCACCAAAAGCACAT
CACCTTCACATCTACTAATTTCCTTCCCAGCATGAACTTCAGCACCCAGAATGGAGGTGAAAAGATGGCTGTCAATGGTGCGCACCACCGTGCCTCCATC
GTGAGGTGAACAGCCTCGATTGTGGGGCGGTGGTATGTACCATCACTGGCGCGCTCCGGTGAGCCCGTGGCAATTCATCCTTGGCTTATGAAGTATCTGT
ATCTGGTAATAATTTGCTTTCAGTTTAATATGTAGAGCTTGTCCGAGATGAACTCATTTATATTTGTTGCAACTAAAACGAGGAGCTACTTGTTGATATA
TGTGGATATTGTGGATACAGAAATATCCTGATGTTTCTTGATGTACCAAGTCCACTATTAAAGTATGACTGGAAAAAACGTTTAATAACGTAATCATG
>Locus_10929_Transcript_35/78_Confidence_0.216_Length_2586
TCGCAGTACAGGGGCGTCACCTTCTACAGGAGGACGGGCCGGTGGGAGTCTCACATCTGGGATTGCGGGAAGCAAGTCTACTTAGGTGGTTTTGACACTG
CGCACGCGGCTGCGAGGGCCTACGATCGTGCTGCGATCAAGTTCCGAGGGCTCGACGCGGACATCAACTTCACTCTGAACGATTACGAGGATGATTTGAA
GCAGATGAGGAACTGGACCAAGGAGGAGTTCGTGCACATCCTCCGCCGCCAGAGCACGGGGTTCGCGAGGGGGAGCTCCAAGTACCGCGGCGTGACGCTG
CACAAGTGCGGCCGCTGGGAGGCGAGGATGGGTCAACTTCTTGGCAAGAAGTAAGCCAACCTCCTGGCTTTGACCTTTCTACTCTGTTAGCCTAGTTAAG
TGTTTTTTACCCTGGTTATATGAACAAAATATGTCGAAATGATGTTCAATTAACTTGTCATTTCACGTTGGGTTCGATCAGGGCTATATCCCTTATGTTC
ATGTTAAGTAGTTTCAGGTGATTGTAGAGAATACAACAAGGTGATCTTGAAGTATCACTGATTATAATCCATGCTACTAGTAGTTTGAGCGTCTCTGTTG
TGCCGGTGAAATCTCCACCCCTCTTGTGACTTTACCCCTCTACCGTTGAATCCGGGAATCCGGAATGGACCTACAGATCGAGATATGTGCCTTGATGAAC
AGTGTTTCGGTGAACAGCAAAATCGGCACCCGTGCACAGTGTCCTGATGGACGCAACCTCTAGAGTTCACATATATGCATTGGCCAACAATGCAATTCCT
GCTTTTTAGTCAGGAACCAGTTTTGTCAAAGTCTACGACACTAGGCATCCCTCTGTTGAGACATATCTCAGAACTGGAATTTACATGCAGGTACATCTAT
CTTGGACTCTTTGACAGCGAAGTTGAAGCTGCAAGAGCATATGACAGGGCAGCCCTTCGCTTCAATGGGGGGGAAGCTGTTACTAATTTTGGGCCTAGCT
CCTACAATGGAGGAGATGCTCTACCCGACACCGAAAATGAGGCTGTTGTTGATGGCGATGCGGTTGATTTGGATTTGCTTTCACAACCTAATGTGCACGA
CACTAAAAGGGACAATACCTTAGCTGGAGGCCAGCTAACATGCGACTCCCTTGAATCTTCAAACACGATGGCCTCTCAGCCAATGAGCTCATCGTACCCG
TGGCCTGTGTATCACCAAAGCACGACAGCAGTACCACCTCACCATCAACGCTTGTACTCATCTGCTTGTCCTGGCTTCTTTTCGAACCTCCAGGTATTAC
CAAGCTAAAGCCTATTTTTTATGTGCAAATTGCTACTCCTACGTGATCAATTAGTTCAAACTGTTATTCGAATCCAGGATTGTTTACTGAATATGTCCTG
TCTTCAGAAAAAATAACACAAAAAGGAACATAATATGTCCTGTCTAATTGTGGACTGATACTAGCCAATTAAACAGTTTCCCATATCCATTTCCTGTGTT
TATTGTTTTGATTTGCTGGCATGGTTAAACTATAGTAGCTTGATTCGATAAAAACACTAAGCAGGCTTTGATATTTTGAATGAAATGTCGTTCTTGGAAA
TACAGCCTTTTCCTTGGTATATCAAGGACAGTTGTCCATCTGCACTCTGCACCCTCAGCTTGTGTAACTGTTTGCCTTGTGAACTGATTGATGTGTCAAT
CAGGCAGTTCTGCCAGTTTGCATCTTTTGAAATAGTGCAGTCTTCCAGTCTAACAGCTCCATGACATAACTTGGGATGCATGCTGCAGCTTTCTTACTGA
GGATGCTCTAGATTTACAGAAATTCTCGCAGTTAGCAGAGGGCTATCAAATCTGATTTGCTTAGCTTCCTGCAGACAATAAGATAAGAACTCTAGAATCC
TGAAGAAATCTTACTTGTACATGTTTACCAAAGTATCCTTACTCCTTAGTTGAGCCAATTTTGGTGAAGAACCGATAGCTCATTACTCTGTTTCCTGGGA
ATGATGTGGGCCTAAACCATTCTGTGGTCAAAGTTTAACAAAGCCCATGCTTGTGCTGTTGCAACTGCAGGAAAGGCCAATGGAGCGAAGGCCTGAGTTG
GGTACCCAGTCATTCCCCACCTGGGGATGGCAAATGCAGGGCTCCCCTCACATGCCGTTGCATCACTCTGCAGCATCATCAGGATTCTCTACCGCCGTCG
GCGCAAACGCCGGCGTCTCGTTGCCTTCTCACCCGCCGGTGCCGTTCCCGGACCACCAGTTCTACTTCCCACCGACGGCATGAGCTTGTTGGTCTGGTTC
AACTCTTGGGACGGCAGCTGATCTTATGTGTGAACATTTCGCAGCTTGCCGGTGACCGTTGTTAATTAATCGGGGAGAGAGAGCCAGAAGACCCAGTATG
GCTATCTCTCTGTGGCCCGTGCCTTGTTATGATCTTAGTTATTGCTACTACACCGTGGATTCATCATGGTTTATGAGATTCTTTACTCAAGTTTCAGCTT
AAATGTCCAAATACATGAATGCATCATACACAGGATCCGTCATTTTCTAAAAAAATACAGGATCTGTGTGAAGTGTAAACATAAGG
>comp78295_c1_seq15
ACAACTGCACAACTTGCAAGAAAGCCACAAGCAACTAATGGGTGAGAAGCTTTCTGGCCTAGGTGTGAGAGACCTCCAGAGTTTAGAGAATCGTCTTGAA
ATGAGCCTACGTAGTATCAGAACGAGGAAGGTCATATTCATTTTGTTTCCACGTTTTGTTTTTCTATTCACTCATCTTAATATGCATAAACATCCATCAA
ACAGGACAATCTTTTGAAAAGTGAAATTGAAGAGTTGCACAGGAAGGTAAAGCCTTACAATCCATGTCTTTTTATTATCTCAAACAAATGACCATGCATG
GAGGTTGCTGACATCTTAACTTAATGCTGATTGAAACAGGGTAGCCTAATTCACCAGGAAAACTTGGAACTCTGTAGAAGAGTAAATACCATGTCACAAC
AAAATATGGATCTACAAAGAAAGGCGAGTGAACCAAGAGGTGTTGCCGATGCAAATAAAAGCTCTAGCACTCCCTACAGATCTTGTAGTGCACAAGATGC

AGATATCACGGTTAATCTTGAATTGAGCCAATCACAGCAAAAAGAGGGGGAGCAGTGCCAAAGAGGGGCTCCAGAACTGGGGCTTCAGCTGCACAAAGAA
GATGCACTACAAGCTGGAGAGTATCATGATGTTCCACCAATTCTAACATGCAATGCGGAGCACTTAAACTACAATTAGTATTTTGTGCAACATGTTAGAT
GAGTGAACAAAATGGTTTATGTACAGACTACAGATATGACCATCTATCTTATCTGTGGCTCTACTGTACTGAAATTGCAAGAAATGGTTATTGTAATGAC
TAATATGATACATTGACTGTCATACCGTTGACCTTGAAGATAGTTCTATAACGGAAAGGTCCAATTTGAAACAAGTGTTACGGATGAGCTAGTAGGATTT
TTTTCACAAACAATTAGATAGTCCACGTTAGTGGTAGCTGCTGGGTATGACATTTGACCCAGCCTGCCGACCCTCCCTTTCCCATGGGAGTTCAATTAAC
ATTAGGAATGAATATCGATATTATGGTTGATTATTTGAATCGTATGTTTTCCACCCTTAGATGTAAGGCCCTATTTTACTGTTTTAATGGATTAACAAGG
TGAGCTTTCGAGATGGTAGCTCATTGATTCGCATGCATTTTAGTGGCTGTTAAGAGGTACTTAGTGCATCCATGCTTGAAGGTGTTGTTAGTTCGACAAA
CCATTTTCTATCCTACTGGATTACTATCTTTTGGTCAGTATGACAAGCAAGAATTTAATCTGCCAATTAAGAATTTAATGTGTTCTTTTGACATGAATGT
CAACAGCATTTAAGTAACTCAACTTTTAATACAAAGTACTGGAATGTTTTCTCACCGAAAGTAAAGATGTGGTTCCTTGATAGCAAACAATGTTTATGCC
AATATCGCATTAAGGTGGTTGTATCCTTTAGAATGATCTCCTCATTCGTGCTTAATTTGATGCTATTGTCTTTTTACGGATGCCTAGATGTGGTTTTGAC
CATTACTAAGTACTTTAATATATATTTAACAAAAATAATGACATAGCTGTTGTATGGCCAATCTAGATTTTGTTTTCGAAACATATTTGCAGTCAAACTA
TAGGAAGTAAGAAACTTCAATGTGGTTCTCGAAAACCATGCTTAAAGTTTAGAAAGTTTGACTACATAGAGTATATACTACATAGTCTTAAAGCATCAAA
TGAAGAGGAATGGAGGGAGTAGCAGACAATCATCATTCAATATCTATTTCTTGGTTGCCTTCAAATTTGGGCTTACTAAAATCGATTTAAAATTCTCGTG
TTCCAGTTGAAGCTCACTTCAAGTAAATCAGGAATTACCATATTTTTCATTGCTCATAATTTGGAAACATGGCATGATATTTGATAGAAAATGTACAAAT
CCTCAGCTGCTCAGAAAGTTACAAAGGGAGGAACGCCCATGAAAGAGAATTCATGGCTGCAACAACGAACATAAACACAGCCTATTCTGTCCAGCAGTCA
TGGTAATACAAGCTAAAAAGGACCGCTCTGGAACAATAGCTTGAAAAGCAATATTAATACAATTTCAGATGAACTATGTGCGTCGATGTCTTACTTGTGA
CTTCCTTGCAAGTAATGCGGTTCCTGCCTCATGCTTGGTGCCGCCGCATGCAATAAACCGATAACTAGTGAAGCAAGAGGATAACAGATGTCAGAAAACT
GAGCAAGCCATGTTAACTGTTTGAGAATTGTATACGGCTATGAGAAGTATATTTTTTTAGGGGGTGTTTCGATCCCTTTAGCTAATTTTTTAGCTCTATA
TTTGGATGTTAAATAGGAGGACTGAACATGGGCTAATTATAGACTAATTGCAATTAGTGATTAGAGCCCATAAGCTCTAATTAGCCTATGATTAGCCCAT
GTTTAGCCCATTAGCACATGAGCTAATCATAAGCTAATTAGGGTTAATTAGCTCTAATCATCAATTAGCTAAAGCTAACTATAATTAGTTCACAATTAGT
CCATATTTAGTCCCCCTAATTGGGGTCCAAACATGGGGCTAAAAATTAG
>Locus_14087_Transcript_3/66_Confidence_0.062_Length_409
GATACAAGCCAGCGGACAAATCTTTTCTGTAATCAGCCTCTTTTTGGCCAAGTGAGAAGCAGTGCAGTTTCTTCATGGAATAACTTAGGAGGCTTCAGAT
CTATGGAAACGAAACATCTGTTGATGCAGCCATCGAAAACTGTAGGCCTTGATGAGCTGCATTTCTCAACCCCCCAGATATCAAATAGTGCTGTGGCTCA
TTCTGTACATCATCGTAATTTCGATGGGCTCATGCCATTCAAGGGAACCAACACAAAGGTCCTCAACCAAGGCGTGGAAGCTTCTGCGATCGCTTCCAAC
TCGAGTGGAGACCCGGATCTTGGGTGTGCTCTCTCTCTTCTGTCAAACGGTTCGTGGGTTTCAAGTTCAACCGTCATCCAGCAGCCTAGTTCTCATGTGC
ACACTGGTG
>comp79901_c0_seq5
GACGCGGCGCGGGCCGAGGTCCCGGAGCTCGCAGTACAGGGGCGTCACCTTCTACAGGAGGACGGGCCGGTGGGAGTCGCACATCTGGGATTGTGGGAAG
CAAGTCTACTTAGGTGGTTTCGACACTGCTCACGCGGCTGCGAGGGCTTATGATCGTGCAGCGATCAAGTTCCGAGGGCTCGACGCGGACATCAACTTCA
CTTTGAGCGACTATGAGGATGACTTGAAGCAGATGAGCAATTGGACAAAGGAAGAGTTCGTGCACATACTCCGCCGCCAGAGCACGGGGTTCGCGAGGGG
GAGCTCCAAGTACCGCGGCGTGACGCTGCACAAGTGCGGCCGCTGGGAGGCGAGGATGGGTCAACTTCTTGGCAAGAAGTAAGCCAACCTCCTGGCTTTG
ACCTTTCTACTCTGTTAGCCTAGTTAAGTGTTTTTTACCCTGGTTATATGAACAAAATATGTCGAAATGATGTTCAATTAACTTGTCATTTCACGTTGGG
TTCGATCAGGGCTATATCCCTTATGTTCATGTTAAGTAGTTTCAGGTGATTGTAGAGAATACAACAAGGTGATCTTGAAGTATCACTGATTATAATCCAT
GCTACTAGTAGTTTGAGCGTCTCTGTTGCCGGTGAAATCTCCACCCCTCTTGTGACTTTACCCCTCTACCGTTGAATCCGGGAATCCGGAATGGACCTAC
AAGATCGAGATATGTGCCTTGATGAACAGTGTTTCGGTGAACAGCAAAATCGGCACCCGTGCACAGTGTCCTGATGGACGCAACCTCTAGAGTTCACATA
TATGCATTGGCCAACAATGCAATTCCTGCTTTTTAGTCAGGAACCAGTTTTGTCAAAGTCTACGACACTAGGCATCCCTCTGTTGAGACATATCTCAGAA
CTGGAATTTACATGCAGGTACATCTATCTTGGACTCTTTGACAGCGAAGTTGAAGCTGCAAGAGCATATGACAGGGCAGCCCTTCGCTTCAATGGGGGG
AAGCTGTTACTAATTTTGGGCCTAGCTCCTACAATGGAGGAGATGCTCTACCCGACACCGAAAATGAGGGTATCACAACCAGCCTCGCCATTTATTTCTT
TTCATATAAGCAAATGTATCTCAGGTGCACCTTATAATCATCGCTGTGTTCTTAGCAACTGTTGATGGTGACGCGGTTGATTTGGATTTGCGGATTTCAC
AACCTAATGTGCACGACACTAAAAGGGACAATACCTTAGCTGGAGGCCAGCTAACATGCGACTCCCTTGAATCTTCAAACACGATGGCCTCTCAGCCAAT
GAGCTCATCGTACCCGTGGCCTGTGTATCACCAAAGCACGACAGCAGTACCACCTCACCATCAACGCTTGTACTCATCTGCTTGTCCTGGCTTCTTTTCG
AACCTCCAGGTATTACCAAGCTAAAGCCTATTTTTTATGTGCAAATTGCTACTCCTACGTGATCAATTAGTTCAAACTGTTATTCGAATCCAGGATTGTT
TACTGAATATGTCCTGTCTTCAGAAAAAATAACACAAAAAGGAACATAATATGTCCTGTCTAATTGTGGACTGATACTAGCCAATTAAACAGTTTCCCAT
ATCCATTTCCTGTGTTTATTGTTTTGATTTGCTGGCATGGTTAAACTATAGTAGCTTGATTCGATAAAAACACTAAGCAGGCTTTGATATTTTGAATGAA
ATGTCGTTCTTGGAAATACAGCCTTTTCCTTGGTATATCAAGGACAGTTGTCCATCTGCACTCTGCACCCTCAGCTTGTGTAACTGTTTGCCTTGTGAAC
TGATTGATGTGTCAATCAGGCAGTTCTGCCAGTTTGCATCTTTTGAAATAGTGCAGTCTTCCAGTCTAACAGCTCCATGACATAACTTGGGATGCATGCT
GCAGCTTTCTTACTGAGGATGCTCTAGATTTACAGAAATTCTCGCAGTTAGCAGAGGGCTATCAAATCTGATTTGCTTAGCTTCCTGCAGACAATAAGAT
AAGAACTCTAGAATCCTGAAGAAATCTTACTTGTACATGTTTACCAAAGTATCCTTACTCCTTAGTTGAGCCAATTTTGGTGAAGAACCGATAGCTCATT
ACTCTGTTTCCTGGGAATGATGTGGGCCTAAACCATTCTGTGGTCAAAGTTTAACAAAGCCCATGCTTGTGCTGTTGCAACTGCAGGAAAGGCCAATGGA
GCGAAGGCCTGAGTTGGGTACCCAGTCATTCCCCACCTGGGGATGGCAAATGCAGGGCTCCCCTCACATGCCGTTGCATCACTCTGCAGCATCATCAGGA
TTCTCTACCGCCGTCGGCGCAAACGCCGGCGTCTCGTTGCCTTCTCACCCGCCGGTGCCGTTCCCGGACCACCAGTTCTACTTCCCACCGACGGCATGAG
CTTGTTGGTCTGGTTCAACTCTTGGGACGGCAGCTGATCTTATGTGTGAACATTTCGCAGCTTGCCGGTGACCGTTGTTAATTAATCGGGGAGAGAGAGC
CAGAAGACCCAGTATGGCTATCTCTCTGTGGCCCGTGCCTTGTTATGATCTTAGTTATTGCTACTACACCGTGGATTCATCATGGTTTATGAGATTCTTT
ACTCAAGTTTCAGCTTAAATGTCCAAATACATGAATGCATCATACACAGGATCCGTCATTTTCTAAAAAAATACAGGATCTGTGTGAAGTGT
>Locus_14339_Transcript_55/63_Confidence_0.202_Length_2933
ATTTTTAGCAATGTATTAGTACATCACGCCACACATTTCTAGATTATTTTCTGTAGATAAGAAACTGACAGTACTTCTTGTTTTACTGATAGCTCTGGCA
AAGGGAGGCAGCAAGCTTGAGGCAACAACTGCACAACTTGCAAGAAAGCCACAAGTATGATCTTCCAATTGATTCTGCAATAGAGAGAGATCACTCATCA
ATACACAGTTTATATGACACATGGAAAGATTTACTAGCATGAGAATTGTCAAAAACCAAAATGAAATGTTTCAATCAGCCAGGATGAGGGGAAAATCTAT
TTACTGAAAGACATTTAAACTATTCAGTATTCAGGAAAGGAAATTGATAGTGGCTCTCTCCCAACAACATATAAGTGGTTTTGCTTTGACCTGTGTACCC
ACGAGATTTATATTCGGTTAGTTTCAATGTTGTACACAAGATCTAACGTGATAAACTCTTTTGCATTGTTGTAGGCAACTAATGGGAGAGGAGCTTTCAG
GCCTAAGTGTGAGAGACCTCCAAGGTTTAGAGAATCGTCTTGAAATGAGCCTACTTAGTGTCAGAATGAGGAAGGTCATATTCATTTTATTTCCACATTC
TGTTTTTCTGTTCACTTCAATCGAATATGCAGTAAACATCTATCAAACAGGGCCACCTTTTGACAAGTGAAATTGAAGAGTTACATAGGAAGGTTAAGCC
CTACAATGAATGCCCTTTTATTATTTCAAACAAATGATCATGCATGGAAGTTGCTGACATCTATACTGATGATGAAACAGGGCAGCCTAATTCATCAGGA
AAACACGGAACTCTGTAGAAGAGTGAATATCATGTCACAACAAAAAATGGAACTTCATAGAAAGGTTTGATGAACTATCTTTTGCAGAAACTTCAACCAA
ACGAAGATGCATTGTCAGCAGAAAGCTAAAATGACTTAATTTCAGCTCCTGGCCAGTGAACCAGGAGGTGTTGCTGATGCAAATAAAAGCTCTAGCACTC
CCTACGGCTTTGGTACAGCACAAGGCGCAGATGTCCCAGCTAATCTTGAACTGAGCCAGTCATCACAAAAAGAGGGAGAGCAATGCAAAACAGGGGCCCC
GGAACTGGGGTAAATATTCTTCTTTAGGATCCAAAATTCCCATTACTCCAAGAAGTATGAGGAAGCTTAATGAGAACTCTATGCTTTAATATGTAGCAGA
CTTCAGCTGCACTAAGGAGAAGCAGTACAAGCTGGACAATGCCATAATGGTCCACAAATTTCACCATGCGATGCAAGAGCATTTTGGTTGCAATGACTAT
TTTGTGCAATATACTAGCTGGGTAAACAAATGGGTTGTGCAAAGACTACAGATAGGACCAGTTATGTTATCTGAGGCTCTGCTGTGTCTAAAATGCAACA
TATTCGTTATTGTAATGACACATGATAAACTGACTGTCCTACCGTTGACCTTGAAGATAGCTCTGCAGTGAAAAGGTGTATTATGGGCTTGCCAATATTA
AAGAAGTGTTAGGGATCAACTAGTAGGATCTCACAAAGAAGTAATTAGTCCACATTAGTGCCAGCTGCTGGGGATGATATCTGACTCAGCCTGCCAACCC
TCTCTTAGCCATGGTGTTTCAACTAGTATTAGAAAAGAATGTTCATCCAATTGAGTTCCCAAACAACGAAATTTATATTATGTGTTGCAAAGTGTCGACA
CCAGTGGAACAGAGTCCATATATGTCAAGCAAATGCCAAATGTCAATTTTGCTCCAGTGCACGTGGATTCTGTTCTATTCTCAAGAATTTCAACCCTTGA
TAAGGCCCTTTTTTCCTGGTGAAAGGATTAACAACGATGAAGCTTCGAGATGGTTTAGTGATTGTTAATAGAGGTACTTAGTGCATCCATGCTTGAAGGT
GTTGTTAGTTCGACAAACCATTTTCTATCCTACTGGATTACTATCTTTTGGTCAGTATGACAAGCAAGAATTTAATCTGCCAATTAAGAATTTAATGTGT
TCTTTTGACATGAATGTCAACAGCATTTAAGTAACTCAACTTTTAATACAAAGTACTGGAATGTTTTCTCACCGAAAGTAAAGATGTGGTTCCTTGATAG
CAAACAATGTTTATGCCAATATCGCATTAAGGTGGTTGTATCCTTTAGAATGATCTCCTCATTCGTGCTTAATTTGATGCTATTGTCTTTTTACGGATGC

CTAGATGTGGTTTTGACCATTACTAAGTACTTTAATATATATATTTAACAAAAATAATGACATAGCTGTTGTATGGCCAATCTAGATTTTGTTTTCGAAA
CATATTTGCAGTCAAACTATAGGAAGTAAGAAACTTCAATGTGGTTCTCGAAAACCATGCTTAAAGTTTAGAAAGTTTGACTACATAGAGTATATACTAC
ATAGTCTTAAAGCATCAAATGAAGAGGAATGGAGGGAGTAGCAGACAATCATCATTCAATATCTATTTCTTGGTTGCCTTCAAATTTGGGCTTACTAAAA
TCGATTTAAAATTCTCGTGTTCCAGTTGAAGCTCACTTCAAGTAAATCAGGAATTACCATATTTTTCATTGCTCATAATTTGGAAACATGGCATGATATT
TGATAGAAAATGTACAAATCCTCAGCTGCTCAGAAAGTTACAAAGGGAGGAACGCCCATGAAAGAGAATTCATGGCTGCAACAACGAACATAAACACAGC
CTATTCTGTCCAGCAGTCATGGTAATACAAGCTAAAAAGGACCGCTCTGGAACAATAGCTTGAAAAGCAATATTAATACAATTTCAGATGAACTATGTGC
GTCGATGTCTTACTTGTGACTTCCTTGCAAGTAATGCGGTTCCTGCCTCATGCTTGGTGCCGCCGCATGCAATAAACCGATAACTCGAATCCTCCGTGAC
TTCCACCCTCTCCAGCCAATGAGCGGCTTGCTG
>comp70939_c0_seq9
CCGGGCGCTCGCCCCGGATTTCTCGGCTCCCCATCCGGCGGACGCCTTGACCGGGGTGGGTTCTTGGTTCCGGCGGCTCGGTGATCTGTGCGTGCGAAAG
GCGGGGCTTTTAGCTCGTTCTGTGGGGGGAGGAGGAATACGGCGGCGAGGCGTTCATGGGTTAGATCTGGCGGTCCGGCGCCGCGAATCTGAGCGAGGTT
TCGGTGGAGGGATCGGGGCGGAGAAGTCGCAGTCTCTGGCTCGTTCTGAGCAGCTTGGGCCTCTCATGTCCAAATCGGGTATGGATGATGGGTGCGGTAA
CTGTCGGAAGGAGCGTCGCTTGCAGATCTGAAAGCCATCCAGCTCCCTTGTTCGGTTGACCTGGAATGGCCGTGCTTTCTGCTGTTTGACTGTCAGTAGT
TGCTGCTTAGTGTGTGTGCAAGAAATTTAGCTGGAGGTGATGTGCTTAGGAAACTAGTCTAGTTGCTGATATCTTGGTCTCTGTTGAAGAACGCTGAAGC
TGGAAGGAAGTAGGTCGCATTCCTGTGTTGTTGATATTTTGTTGTTTCTCGAATAACCTGGAGGCTGGAAGGGATCAGATCCAGTTCCTGTGGCACCGGT
AACAAGGTGCTGTTGGGGGAAGGCACCATGACCTACTTCCCTGAGGAGGTGGTGGAGCATATATTCAGCTTCTTGCCGTCGCACAGTGACCGGAACACGG
TCTCGTTAGTGTGCAAGGTGTGGTATGAGGTTGAGAGGCTGAGCCGGTGTGCTGTCTTTGTGGGGAACTGCTACGCGGTGCGCCCAGAACGGGTGGTGCT
GCGGTTCCCCAATGTGAAGGCCCTGACGCTGAAGGGGAAGCCGCACTTCGCGGACTTCAACCTCGTGCCGCCGGACTGGGGTGGCTATGCGGGGCCATGG
ATTGAGGCTGCGGCGAGGCGATGCGTGGGTCTTGAGGAGCTCCGGATGAAGCGGATGGTGGTGTCAGATGAGAGCCTTGAATTGCTAGCTCGGTCCTTCC
CGAGATTCAAGGTCCTCGTCCTTATCAGCTGCGAGGGGTTCAGCACTGATGGGCTAGCAGCTATTGCGAATCACTGCAAGCTCCTGAGGGAGTTAGATTT
GCAGGAAAATGATGTGGAGGACCGTGGGCCTAGATGGCTCTCCTGCTTCCCTGATTCTTGCACATCACTTGTTTCCTTGAATTTTGCCTGCATCAAAGGG
GAGGTGAATTCTGGTTCATTGGAGAGGCTTGTTGCTAGGTCTCCAAACTTACGCAGCTTGAGGTTAAATCGATCTGTATCAGTAGATACACTCTCAAAGA
TATTAGCATGCACCCCTAATTTGGAAGACTTGGGGACAGGGAATTTGACGGATGAGTTCCAAACTGAATCCTACTTCAGGCTGGCTAATGCTTTGGAGAA
ATGCAAAATGTTGAAGAGTTTATCAGGATTTTGGGATGCTTCTCCTGTTTGTGTTCCGTTTATCTATCCCCTATGTCATCAACTAACAGGACTGAACTTG
AGCTATACTCCCACACTGGATGCCTCTGATCTCACTAAAATGATCAGCCGCTGTGTGAAACTCCAGCGTCTTTGGGTACTGGATTGCATCTCGGATAAGG
GTTTGCAAGTGGTGGCTTCCAGCTGCAAAGATCTACAAGAACTGAGGGTGTTCCCGTCAGATTTTTATGTTGCTGGTTATTCTGCAGTGACAGAAGAGGG
ACTTGTTGCAATATCGTTAGGCTGTCCAAAACTTAGCTCCCTGCTGTATTTCTGTCACCAGATGACCAATGATGCACTAGTTACTATAGCTAAGAACTGC
CCAAATTTCATACGATTTAGGCTCTGTATTCTTGAGCCAAAGAAGCCAGACGCCATGACAAACCAGCCGTTAGATGAAGGCTTTGGTGCAATTGTTCGTG
AATGCAAGGGGCTAAGGCGACTGTCAATGTCGGGTCTTCTCACTGACAAGGTTTTCATGTATATTGGAAAGTATGCCAAGCAACTTGAGATGCTTTCTAT
AGCATTTGCTGGAGATAGTGATAAGGGGATGATGCATGTTATGAATGGATGCAAGAATCTAAGGAAGCTGGAGATTAGAGACAGTCCATTTGGTGATGTT
GCACTGTTGGGGAATGTTGCCAAATATGAAACAATGCGATCCCTTTGGATGTCCTCATGCAATGTCACATTAAAGGGGTGCCAAGTCCTTGCGTCTAAGA
TGCCGATGCTCAATGTGGAGATCATGAATGAGCTAGATGGAAGCAGTGAAATGGAGGAAAACCATGGAGATCAATCTAAGGTGGAGAAGCTATATGTTTA
CCGCACAACTGCTGGAGCGAGGGATGATGCACCAAATTTTGTTAAAATCCTATAGAGCTAGCTGAGAGTAGTACGGTATCTTCTCACTCTAAAAGGTGAT
CTTTCTTCCAGATCTATCTTCTTTCATTTGGAAATGTCTTGTCTGTTGGACAATTTGTCCGCTACTCTGCTAGCCCAGTGCTAGTTTGTGTCTACCGTTC
AAATTTAGATATTTCATGCTTAGTAGCTCTATTGAATACTGCATCTTATACCGAATGCTCCGCAACATGCAAACTAGCGTCTTTAGGTTCATATAGCTCA
TTATGTTGGCTTAAATGTTTCATTCAGTAGCGTCTGAATTAACTCGTTTCCTATTTGTACGTCATTATATCAGAACTGTATTATTAAGGTTTGAAATGCT
GCAGGAAGAAGCTTGTGCTTTCATCATTAACTTAAACCAAGACTGATTTCTGAAAAGATCCGAATGGGTAGTAATTGAACTGATGTTTGAACCGCCTTTA
TTCTGCAAAATCTTAGCTGCTCTTTTTGTTTCTTCCTTAGTACATCGATCGCTTAGCTACTCATAGAGCTAATTTCCGTCAACCTGTGTATCATTCATAT
TCCATTGCGAGTATGGAGCAGCAGAGCATCTGATATGTGATATATCTTACTGTCTTCCCACTCCCTACTCGATTGCTTTTACAGATGGTCGGTGGAATCC
GGCAGGCTGGGGGCCGCGTCAACAGTGGTAAGTTCTTGAGGTGTCTGTCTCTCCCTGTGCAGGGGTCAGGAAAAGCCACACCGATGTGCACCCTTATCTT
GGAGCAACTGGCTGGGATCCATCATCATCCCCTGTACCACCGCCCCCTACCCTTTTGTGTTTGGAATTCCTTTGTGTTTGCTCACTCATATCCACTGAGG
CCCTTTATCATCCAGAGCATTCTCACTTGAGAGGACCGCCTTTTTTCCGTTTATCCCACTGTAATCTGTAGTGTACCAGTATCCATTTGCATTTGACATT
GGCAGTGGACTGTTTTGCCGCTGTTGTTGTAGTGTTTGTTTCAGCCTGACGATTTGTTTCTTGCTCCTGTGAATGCAGTGCGTGGAATAATTTGCGGTTG
TTATCTGCGACATTCTATTCTCTGGTTTTGAATTCTTGGTGTTAGCCGTACCAGTTTTACGTGCTTGTTACTGAGCAAGAAGGCACCAGTACTCGTGCAT
TTTAAGGGCGTGCTCAGTTTCTGATGCCATTTTTCTCGCTTACATCATCTTTCTGTATTGCTGCCCTCAAATATGAATTCAAATTAACGGT
>Locus_32123_Transcript_7/16_Confidence_0.538_Length_1144
ATCTCTCTCTCTCTCTCTCTCTCTCTCTCTCTCCAGCTAGCCGCCGTTTGACCCTGCAGATTGCTTCCTTCTGCACGGCGCGATGGTACGGGGAAAGACCAT
ATTGAAGAAGATCGAGAACGAATCAAGCCGGCTTGTGACCTTCTCCAAGAGGAGGGGCGGGTTGTTCAAGAAGGCAAAGGAACTGGCAATCCTATGCGAC
GCTCAGGTCGCTGTTCTCATCTTCTCCAGCACCGGCCAACTCTACGACTTCTCCAGCTGCAGCATGAAATCAATAATCGAAAGATACCGCCAAGTAAAAG
AAGGTCAGCAAAATGTAACGGCAAGTAGGGAGGCCAAGTACTGGCAAGGGGAGGCGGAAAGCTTGAGGCAGCAACTACACAACTTGGAAGAGAATCATAG
GCACTTGCTGGGACAACATCTTTCTGGCTTGTCAATGAGAGACCTGCAGACTTTAGAGAACCAGCTGGAAACGAGCCTAACTAATATTCGGCTAGCAAAG
GAACGAATTATGATTGATCTGATTCAAGAATTGAACAAGAAGTCAAGCCTCATAGACATCGAAAACAAGGAGCTACACCACAAAATCTGTGCCATTAGTC
AAGAGAATGCCAATTTGAAGAAAGAGGTAACACAAAGAGTATCCACAATAATGTCTTAATTGTGAGCTATCTTCCGTCAGTGTTTATGTCTTTCCATTCA
TCAGGTACATGGACAGCCGGATGCCATTGAAGAAAATACTGGTTCTGTTACTCAGTATAGTACTAACACTCCTGAAGACCTAAATGCGACGTGTCTCGAG
CTACGCCAACCACATCATGTAGAAATAGAGCAACCAGATGTGCCTACATTGGAACTGCGTCTCCACTGATTGCAAGAATCAGCAGCAGAATCATCGGAAA
TGAGGAGGCCAGCCATTCAACGCAAATTCATAAGACTTTGTCTCCACTGATTAAATACAAAGAGGAGTTCCTGTGCAAATTACTATTATATGAATGTAAC
AGATGTAATTTATTTTTTATGTATCTGCAGTACACATATGCCCACCATAAACTTATGTTCAAAAGGCATATATACCTATTGCCACCAAGCTACGACGATT
ATCATTGTTATGCTACTTGGATCAGAGATGAAGGTTAAAACTTG
>comp83218_c0_seq8
CGCAGCTCGCAGTACCGCGGCGTCACCTTCTACCGCCGCACCGGCCGCTGGGAGTCCCATATTTGGGATTGCGGCAAGCAAGTGTACTTAGGTGGATTTG
ACACTGCTCATACTGCTGCAAGGGCGTACGATCGAGCTGCCATCAAGTTCCGTGGCTTCGACGCCGACATAAACTTCAATCTCAGTGACTATGAGGACGA
CATGAAGCAGATGAAGAGCCTGTCCAAGGAGGAGTTCGTGCACGTCCTGCGACGGCAGAGCACCGGGTTCTCACGGGGCAGCTCCAAGTACAGAGGCGTG
ACCCTGCACAAGTGCGGCCGATGGGAGGCTCGCATGGGGCAGTTCCTCGGCAAGAAGTACATATATCTTGGGCTATTCGACAGCGAAGTAGAGGCTGCAA
GGTTGCTGATCTTGGATTCTTCTATTGATGCAAAAAAAATTTTGCTTTACCTTCTCTGTTGATTTCCAAAGGGTTGAGTACCGACTCGATTCTCTTCTTT
GTTGTTTTTCTTCTTTCTGTTTCAAAATTCAGGGCTTATGATAAGGCCGCGATCAAATGCAATGGTAGAGAAGCCATGACGAACTTCGAGCCTAGCACGT
ATGATGGGGAGCTATTGACTGAAGTTGGTACTGAAGGTGCTGAAGTCGATCTGAACTTGAGCATATCTCAACCAGCTTCTCAAAGCCCGAAAAGGGACAA
GAATTCCCTTGGTCTGCAACTCCACTATGGATCGTTTGAAGGCTCGGAATTGAAAAGAACAAAGATTGATGCTCCCTCTGAACTCACGGGCCGACCTCAT
CGGTTCCCTCTTCTGACCAAGCATCCACCAATCTGGCATGCCCAATCTCATCCCTTCTATTCAAATAATGAGAATGCATCAAGAGATCTTAACAGGAGGC
CTGATCAGGGAGGCACTGTTCCTAGATGGGCGTGGAAGGTGACCGCCCCTCCTCCCACCCAACCATTGCCGCTGTTCTCGTCATCGTCGTCGTCGTCCGC
TGCAGCATCATCAGGATTCTCCAATACCGCCACGACAGCTGCCCCCACCGCCCCGTCGTCCTCCTCCCTCCGGTTCGACCCGCCGCCGCCGTCGAGCCGC
CACCGATGAACATTGAGAAGCCACGCTGTAAATTTGCCGGGAAGCCGGCATTTTTTCCCTCTCCAGGCGTTGCAACTTTTTCGGTTTTGCGCCGGGGTGG
TTTCTTAATTGTAGTGGATTGGATTCATAACTATATTTGCATACTACCGCGAATGGGTTTCTAGCTGAATTAGTTAGATGGCTTATTACCACTCCTCAGA
TTCTAGGTTCGATTTCTCATGAGAGTGAATTTCAGATTAGGGTTAAAAAAATCTCTTTGTCTGTCCTACGCCAAAGCACAGGTTTAAAGCTCGGCCCGGT
CCTCATACGGGCTATTGTGCCACTGTGTGGTTCGGAGGTTTTCTTGATCTACGTGAGAAGATCTTCTTCTTAATGAAATACCCGGGGACTGTCCCCCGCA
GGTCAAGTTTATTTGCATACTGCCCCATGTGAAACCTTCTTTCTCACTCTCTTTACACACTCTGCAACGGATAGATGTTCTTGTTGATGGAAATTAGCTA
ACTTCACCGTCTGAGATGATCTATACATTGTTGTAGAGAA
>Locus_14087_Transcript_50/66_Confidence_0.370_Length_2506

GATTCAAAATTGTAGCAGATTTGTCCTCTACCCACCCCTCTGAATCCTTTTTATACATACACTGACCATGGCACCAATGATCAAACCAAACACCAGCTGC
AAAGATCACCCTGGATTCCTGGAACAAAGAGGGCCTAGAGCAACGCAGCAACCTTCGCCACTTGAAGAGGATAGGTACAATGTGGCTTAGCTAGCACCTT
GCAGTCAGGTAGAATTATTCAACCGGATAAAGATTATTTCAAGTAAAGGAGGGCTACCAGTTTATACCTGTTCAGGTGGGTGCAGCAGTTGAGAACTTTT
TTTCGATACAGAGCAGTTGAGATCTTTAATTCTGGCATGGGTTCCTTTGGGGTGAACTGGAATCAGAAGAACTCCGCGGTGTGCGATTGGGAAAATTTAG
CACCTTCTGTTCCAAATGCAATCGGAAACCCCAGGAACGGAGCTACGAGGCATGGATCTGTGAATTCATCTGGTGGCACTCTCACTTCTAGCTCAGAGCT
AGGGCATGGTTCATCCAAGAGCTCCATGTCGGCATCCATTGATTCACCGTCCAAAGTAGGAAACAGCTTGGAGTTCAACTTTGCTGCTGTCAAAGGCCAT
GTTAAGAACATGGATAAGGATTTTAGATTTGACGACTTGGGGACTTCTCCATCGTCGATGATAGCATTCAGCCATGGAGAGCCATTAATCAGCCTTAAGC
TTGGAAAAAGGACTTACTTTGAAAATGTCTGCGGAGGGCAGGATTCCAAGAGCACTGCACCTTCTACCAGGACTTCTCCATCAACCGTTCTCAAGAAGAC
CAAGGTGTCTCAGCAGAATACACAAATGTCATACTGTCAGGTTGAAGGATGCGGAATTGATCTGTCCTCCGCTAAAGATTACCACCGCAAGCACAAAGTC
TGTGAAGCTCATTCTAAAGCCCCGAAGGTTGTTGTTGCTGGTGTAGAGCGCCGTTTTTGCCAGCAGTGTAGCCGGTTTCATGGCTTAGCCGAGTTTGACC
AGAACAAACGAAGTTGCCGTAGCGTCTTACTCATCATAATGCACGGAGGAGAAAACCACAGGCAGATACAATTTCATTCAGTTCATCGCGGCTCTCGACA
ATGTTTTATGGTAGTATGGAGCCCTTTGTTCATGAGTATATATTATTCTCAAAAACACTATCTAAACTGATCACTCATACAGTACCTCTTTTGAGAGGCA
TTATTTCTGAACCCTACATGTTGAAAGAGCACTTTCTGTAGGTGTCAAATGGTTAATCTTCTATTATAGCTTGTTCTCTCCATATTGTCAAATTAATGAC
TGAAGGCTAATCCTTTATCCAAATTTATACTTCTGAATATGATGCTGCTGCTGCTGCTTTAAGCATTGTAAATATGCATTGGCTTAGATTGTCGCAGATG
CATATTCACAGAGTTAGTCGATTATTTGGAAGGGGACTTAACCCAACTTCATGACACTTTTAGTATGCACATACGCTTCTCTTTTGGCGGGACTACTCTT
AGAATATTCTTCTTAAATTATTATAAATGCAATTTTGCTTCTGGGAGAACACTTAAGTTTTAATGCTGGTATTGTTCGCATTTGCAGATACAAACCAGCA
GAAAAATCTTTTCTGTAGTCAACCTCATTTTGGCCAAGCGAGAAGCAATGCAGTTTTTTCTTGGGAAAACTCGGGAGATTCCAAATTTATGGAAACCAAA
CATCTGTTGATGCAGCCAACGAAAATGTTAGGTCTTGATGGGCTGCATTTCTCAACCCCCCAGATATCAAATAGTGTTGTGGCTCACTCTGTACATCATC
GTAATTTCGATGGGCTCATGCCATTCAAGGGAACCAACACAAAGGTCCTCAACCAAGGCGTGGAAGCTTCTGCGATCGCTTCCAACTCGAGTGGAGACCC
GGATCTTGGGTGTGCTCTCTCTCTTCTGTCAAACGGTTCGTGGGGTTCAACTTCAGCCGTCATCCAACAGCCTAGTTCTCATGCGCAAGCTGGTGCGGTG
CCACCCTTCGCCACCGTCGCCGTCTCCAACCCTGCAATGCATCCTCTGGACTCATCCCCGGGAGGATTCTGGCAAGACGTCCCTCCCCCGCTCGATGAAA
CTCCACATATTCAGGCATTCACGCACCTCTGATCAAATGCTGCATCCCCTGGGAACAGCAGGTGGTTATACCTTCCGTAATCGTCCTAGGTTTGCTTTGC
CAGTGACTGGCTCTGCACAATGTCGATCCATCACCGGAGGCATTTGGTAAGGTGAAGACTAAAGACTCAAGAATAACCGGAGACTAACTCGCTTCTCTTC
CAGGCTCAAGACTGTTTGGGTCCAGCGGTGTTGCTACCTGATCCTGTTTTCGGTTCTGAGTACATCCATTACTAATCATGGGATTACTTGTTTAGGCCAA
CCAGACATATTCTGGGGTGAGATTACGACATGTTAATTTACATTGACGGTGTTTCTACATGTGTTAGTACTAATGATGTTATCGCTCTTGGCCCCGTTAC
GTTTCA
>comp83121_c0_seq27
GGAGGCAGCAAGCTTGAGACAACAGCTGCACAGCTTGCAAGAAAGTCACCGACAGTTGATGGGACAAGATCTTTCAGGATTGGGTGTCAAGGAGCTCCAA
AATCTGGAAAATCAGCTAGAAATGAGCATACGTTGCATCCGGACAAAAAAGGACCAAGTCTTGATTGATGAAATTCACGAACTGAATCGAAAGGGAAGTC
TCATGCAACAAGACAAGATGGAACTATACAAAAAGGTCAACCTAATCCGTCAGGAAAACATTGATTTATACAAGAAGGTATTTGATAAATCGTGAATGAA
AATTCACAGGATATACACCCCATCCATTATTGACTGAGGTTTATTTCTCTACTCAGCTCTACGAGAAAGAGGTGACAAGTGAAGTCAACCGAGATTCATC
AACTCTAAACTTTGCAGTTGTCGAGAACGTCAACATTCCTGTTCATCTTGAACTTAACAGTCCACCGCAAGAAAATGACGTTGAGCAAACTGCACCCCCT
AAACTAGGGTTACAACTAAATCCATGAAGGCATGCAGCATGGCACTGCTAATGTTCATTATTTTCAACGGCTAGGATTCAAAGCAGCTAGAATACCATTG
GTTGCCTAAACACATAGCAAGACAGACGTGGCATGCTGGGCGACGGTAATGGTAGTGTAAATCTATTTTCAAATAAAATGTATTTTATTGAAGACTACAG
AAAATTATTTGTCTTATAACTATTTCACGCATTCTGCAAGTTTGTTCACCAACTTGAGTACATGACAGCTACAAATC
>Locus_14087_Transcript_25/66_Confidence_0.296_Length_1702
TTTTTAGTTTTTAAATCTGAAGAAGATTTCAGACCGAGATTTGAATCATTTGATCTATCCATTTCTGTTCCAATTTGTCAAGATGTATATCGCATCTGTT
CTTCTCGTTCTTGCAGTGCTAGCGGTTTTTACCTGTTCGGGTGGGAGCACAAGTTGAGATCTTTAATTCTAGCATGGGTTCCTTTGGGGTGAACTGGAAT
CAGAAGAACTCCGCGGTGTGGGATTGGGAAAAATTAGCTCCATCTGTTCCAAATGCAATCGGAAACCCCAGGAACGGAGCTACGGGGCATGGATCTGTGA
ATTCATCTGGTGGCACTCTCACTTCTAGCTCAGAGCTAGGGCATGGTTCATCCAAGAGCTCCATGTCGGCATCCATTGATTCACCGTCCAAAGTAGGGAA
CAGCTTGGAGTTCAATTTTGCTGCTGTCAATACGTATGGTAAGAACATGGATAAGGATTGTAGAGTTGACGACTCAGGGACTTGTCCATCGTCCATGATA
GCATTCAGCCATGGAGAGCCATTAATCAGTCTTAAGCTTGGAAAAAGGACTTACTTTGAAAATGTCTGCGGAGGACAGGATTCCAAGAGCACTGCACCTT
CTACCAGGACTTCTCCATCAACCGTTCTCAAGAAGACCAAGGTGTCTCAGCAGAATACACAAATGTCATACTGTCAGGTTGAAGGATGCGGAATTGATCT
GTCCTCCGCTAAAGATTACCACCGCAAGCACAAAGTCTGTGAAGCTCATTCTAAAGCCCCGAAGGTTGTTGTTGCTGGTGTAGAGCGCCGTTTTTGCCAG
CAGTGTAGCCGGTTTCATGGCTTAGCCGAGTTTGACCAGAACAAACGAAGTTGCCGTAAGCGTCTTACTCATCATAACGCACGAAGGAGGAAACCACAGG
CAGATACAATTTTATTCAGTTCATCACGTCTCTCAACAATGTTTTATGATACAAGCCAGCGGACAAATCTTTTCTGTAGTCAGCCTCTTTTTGGCCAAGT
GAGAAGCAATGCAGTTTCTTCATGGGATAACTTGGGAAGCTTCAATTTTATGGAAACAAAACATCTGTTGATGCAGCCAACGAAAACGGCAGCCCCTGAT
GAGCTGCATTTCTCAACCCCCCAGATGTCAAATGGTGTTATGGTTCACGCTGTACATCATCATAATGTCGGTGAGTTCATGCCATTCAAGGATACCACCA
CAAAGATCCTCAACCAAGGTGTGGAAGCTTCTGCGATTGGATCCAACTCAAATGGAGACCCGGATCTTGGGCGTGCTCTCTCTCTTCTGTCAAACGGTTC
TTGGGGTTCAAGTTCAACCGTCATCCAGCAGCCTAGTTCTCATGTGCACACTGGTGCGGTGCCGCCCCTCGCCACCGTTGCTGTCTCCAACCCTGCAATG
CATCCTCTGGACTCATCCCCGGGAGGATTCTGGCAAGACGACCCTTCCCCGCTCGATGGAACTCCGGAGATTCAGGCATTCGGACACCTCTGACTATGAT
CAAACAAATGTACTGATGCTGCAACCTGGGAACAGTTGATGTAGTCACTTCACCATAATTTGCAGTCAGTCTCAGTACAATGTCATTTCTATCAGTGGAG
GCATTTGTTGAGGTGAAGACTGAATAATATAGGAGGCAACCCCCTAATCTTCCTGATTTAGGATACATTTGTGTGCTTGTATTGCTGCCTGAAGTGGTTT
TT
>comp78295_c1_seq36
ACAACTGCACAACTTGCAAGAAAGCCACAAGCAACTAATGGGTGAGAAGCTTTCTGGCCTAGGTGTGAGAGACCTCCAGAGTTTAGAGAATCGTCTTGAA
ATGAGCCTACGTAGTATCAGAACGAGGAAGGTCATATTCATTTTGTTTCCACGTTTTGTTTTTCTATTCACTCATCTTAATATGCATAAACATCCATCAA
ACAGGACAATCTTTTGAAAAGTGAAATTGAAGAGTTGCACAGGAAGGTAAAGCCTTACAATCCATGTCTTTTTATTATCTCAAACAAATGACCATGCATG
GAGGTTGCTGACATCTTAACTTAATGCTGATTGAAACAGGGTAGCCTAATTCACCAGGAAAACTTGGAACTCTGTAGAAGAGTAAATACCATGTCACAAC
AAAATATGGATCTACAAAGAAAGGTTTGATGAACGATCTCATGCAGAATCCTTAACCAAATGAAGGTGCATTGTCAGCATAAAGTTAAATTACTTAATTT
CATCTCCAGGCGAGTGAACCAAGAGGTGTTGCCGATGCAAATAAAAGCTCTAGCACTCCCTACAGATCTTGTAGTGCACAAGATGCAGATATCACGGTTA
ATCTTGAATTGAGCCAATCACAGCAAAAAGAGGGGGAGCAGTGCCAAAGAGGGGCTCCAGAACTGGGGTAAATATTCTTTTTCCAGAATCCAAAAACTGC
ATTACTCCAAGAAGTATCAAGAAGCCTAATGAAGAACCCATGCTTTGACATGTAACAGGCTTCAGCTGCACAAAGAAGATGCACTACAAGCTGGAGAGTA
TCATGATGTTCCACCAATTCTAACATGCAATGCGGAGCACTTAAACTACAATTAGTATTTTGTGCAACATGTTAGATGAGTGAACAAAATGGTTTATGTA
CAGACTACAGATATGACCATCTATCTTATCTGTGGCTCTACTGTACTGAAATTGCAAGAAATGGTTATTGTAATGACTAATATGATACATTGACTGTCAT
ACCGTTGACCTTGAAGATAGTTCTATAACGGAAAGGTCCAATTTGAAACAAGTGTTACGGATGAGCTAGTAGGATTTTTTTCACAAACAATTAGATAGTC
CACGTTAGTGGTAGCTGCTGGGTATGACATTTGACCCAGCCTGCCGACCCTCCCTTTCCCATGGGAGTTCAATTAACATTAGGAATGAATATCGATATTA
TGGTTGATTATTTGAATCGTATGTTTTCCACCCTTAGATGTAAGGCCCTATTTTACTGTTTTAATGGATTAACAAGGTGAGCTTTCGAGATGGTAGCTCA
TTGATTCGCATGCATTTTAGTGGCTGTTA
>Locus_12915_Transcript_9/30_Confidence_0.431_Length_3266
GCTCCTTGAGAAACACCATCCAATTACAAGACTATTTGATGCAACAATCACGCATATGGCTGCGCTAGAACTACTGCTCTGAATTAAGAGGCGTTGAAAT
ATTGTCCGGTCTACATATAGGCACTTGAGGTGTATTGACAGACAGTTCCCCTTAGTTTGCTACAACACATCAGGCGAATTCCACCAAATTAGGATGATGT
TTGTTTCTTAGACACAGGATAGCAATGCCAAAAGTTTTTTATAATTTTCCGCTTCACCGCAGATTTGGCATCTCAAAGGGAGTTGTGGCAAGATTCTCTT
GACCAAGCTTTCTTGCAGATTTGGCAAGAAAGATAAAAAAAATGTTGGTATACTTAAGCATCCTTAATAGAAACTAAACAGGTCTTAGATTGAAATTATC
ATAAATAAACCCTTTTTCACTTTAGCTGTAGTCCGGATGAAATCTTAGGGGCCTTGCTTAGCCTGCCCTGAAGCTTGCCAGTAAGTCGAAGCTGATGCTG
ACACAGCACTGACATAGATCCCAATGAACACAAGTTATTTCGAGAAGATAAAATACCAAGCAAAGCCAGGATTGCATTAAAAGATAGTGTATTTCTACTA
TACGATCTGAAAGCTTAATTTATTATATCAATAATCAAATTGGCAGCAGTGTGGAACAGATATAAAGAAGATGCTTCAAATCACAAATAACTACAGATAA
ATTGAAGGGTGAAGGTTCATTCAACCACCAAACACCCAAAATGTCGAGCTGCATATGCACAGAACCTAAGTACGGTACAGTTCATAGTTTGGGAAAATAG

ACTGCAAATGTGCACAGATAATACAGATTGTTACTCGATGAACAGTAAGCACGGTATTTGAAATCCTTCTAACCATCCATCCAGGCAGTGCCTCGTATAC
TCAAACCTCTTTTTGGAGGAAGCCAAACCATGTGATCCTTTTCAACCCAAATACATATATTCAAGATGAAACTAACAAAAAGACAGTTGATAAAGAAACA
CGAAAGAGAGTAGAAAAAGGGTACCTGTTTCAAGTTCTCATCTTCTGAAAATGATCAGCAGTCAATAGATGTTTTAGTCCATTCTCCAGAAAAATGAGTG
CCGCATGAATCCTTTCCGGTTTATAGTGAAACATTTTAGACGATGACCTTTAACATTCTAGAAAACGAAGAGAAAACCTACAGTCATCTAATTTCAGCTT
AAACATGTTCCAGCAAGATCATCACCATCCTGACATGTTCCGTTTTGTCCCTAGACCCGAGCACAAGGTATTGCGAGGCTAATTTAGCTCAATTCAGTTG
TCAGAGCAATTGTGTGTGAAAAGTGTGAACAACAGCATAGGAGACAATACAGCAAGTGTAGCGTGCCAATATGTGTCCAGCTTGATAGTTCACTATAATA
AAGTTCTTTAATTCAAGTATTTGAATAATATAGAATCTCAACCTTCAAGTTTAAAAGGTTTTGTATTTGCTATTATAATCGTTGCCACCCCCTCACCCCT
CTCAAAAAGAGAGTTACTTTGTAGCGCACTGTAAATCTCTTGATTGAAATAGTGGCATGTCATCTTATTTAATTCAGTTATTTCATAAGGTACAATTTGT
TTAGCTGACAGCTCAGACATGCTTCTATGCAACATGTTTATACCAATGCTGTATTTTGGCAATCCTGGATGTGGCATTACTTAACTGTGCTTTCATATTC
AATAGTTCTGGCAAAGGGAGGCTGCAAGCTTGAGACAACAACTGCACAACCTGCAAGAAAGTCATCGTCAGTTGATGGGACAAGATCTTTCTGGATTGAG
CGTCAAGGAACTCCAAAATCTAGAAAATCAGCTAGAAATGAGCCTACGTTGCATCCGGACAAAAAAGGTAAGGATGCTAGCCATATTTCTGTTTTTCGTT
ACCAGATTGCCTCATCCAAGAATACAACTGATGATGACGTTTTTTACTTTCAGGACCAACTCTTGATTGATGAAATTCACGAACTGAATCGAAAGGGAAG
TCTCATGCAACAAGACAAGATGGAACTATACAAAAAGGTCAACCTAATCCGTCAGGAAAACATTGATTTATACAAGAAGGTATTTGATAAATCGTGAATG
AAAATTCACAGGATATACACCCCATCCATTATTGACTGAGGTTTATTTCTCTACTCAGCTCTACGAGAAAGAGGTGACAAGTGAAGTCAACCGAGATTCA
TCAACTCTAAACTTTGCAGTTGTCGAGAACGTCAACATTCCTGTTCATCTTGAACTTAACACTCCACCGCAAGAAAATGATGTTGAGCAAACTGCACCTC
CTAAACTGGGATTACAACTAAATCCATGAAGGCATGCAGCATGGCACCGCTTATGTTCATTATTCTCAACGGTACGATTCAAAGCAACCAGAAACACTAT
TGGTTGTAAAGAGCTTAAACACATAGCAAGACTGACGCGGTATGCATGCCATGAGTGATTGATAATGGTAGTGTAAATCTATTTCAAATAAAATGTATTT
TATTGAAGAATACAGCAAATGATTTGTATTATAACTATTTGACACATTCTGCAAGTTTGTTGACCAACGTGAGTACATGATAGCTACAAATCTTATATTG
ACAAAAAGATATGGTGTGGAAAAAATTCAGGTGCTATTTTTTTCTATTTGAGAATTGTATGCATAGCAGAAGAGCACAAATTGCATTCTGGTTTATC
CGTGCAGGCATTCCAGAGTATCAGAATGGGGATCAACCTTTCTTCAGCTACATATGTAAATATACGGATGCTAGTTGCTCACTTAAAATTTTGTAGTGTT
CAAAGGAATAACAACAGTAAGACGTTGACGCACCGAAAATAACCAGAAATAACGGATTGTACTATGACAAGACACACGACAGATACACTAGCTTAAGTTT
GTACAGGAAAATATTTGGCCTATTCTTATGTCACAGTAAATTAAGATTATCACCTTAATCACAGAAATTCAAGGGGTAAACGATTATAGCACAAAACGAT
CCTGGACCCCAAGACCTGGGTCTCTGAACGGCTGAACCTCCAATTATACCTCTATACATTTGGAATGTGTGTAGTCAAACTTTCGCTACTGGTATCTTTA
CAAATATTTACATTGAACACATCAAAATGATATACATGGATTCCTCTCGATATGTCTTTGAGGTTTGGGTTCTAAAAATGATATACAAATTATTAGGTTC
TAAACTTAAATGATATATACACATCAAAATGATATACAAATTATTGGGTTCTAAACTTAAATGATG
>Locus_11912_Transcript_33/63_Confidence_0.195_Length_2505
CCCTCCCCGCCGGCCACCTCGGGGAGCTCATCCCCGCCGCTTTGCCGGCCGCGCTTTCCCTGGCGCCTCCCGCGCGGGCGCTCGCCCGGGATTTTTCTCG
GCGCCCCCTTCCGGCGGACGCCTTGACCGGGGTGGGTTCTTGGTCGCGGCGGCGCGTGATCTCTGCGTCTTGAAGAAGGTGTTGCTAGCGGGTTCTGCGG
GGGGTGGGGAGGACGGAGGAAGGAAGCCGGGGGGGACTGCGAGGCGTTCATGGGTTAGATCTGGCGGTCCGGCGCCCGCGGATCTGAGCGAGGTTTTAGG
TGGAGGGATTGGGGCCGAGAGGTCGCCGTCGTCGGCTCATTTTGAGTAGCTTGGGCCTCTCATGTCCAAATCGGGGGCGGGTGATGGATGCGGTAACTGT
CGGAAGGAGCGTCGCTCGCAGATCTGAAGGCCGTCCAGCTCCCTTGTTCAGTTGACCTGCTCGCTGGTCAGCTGGATGGAGTGAATGGCAATGCTTTCTG
ATGTTTGACTGCCGTTAGTTGCTGCTTGGTGTTTATTCAAGAAACTTATCAGGAGGTGGTGTGTGCCCCGGTAACTATTCAGTTGCTGATATCTTGGCCT
GTGTTGGATAACGAGGTAGCAGATCACATTCCTGTGTTTTTGTTGATATCTTGGTTGTTCTTGAATAACCTGGAGGCTGGAAGGGGTCAGATCCAGTTCC
TGTGGCCCTGGTAATAAGGTGCTGTTGGGGGAAGGCACCATGACCTACTTCCCTGAGGAGGTGGTGGAGCACATCTTCAGCTTCTTGCCATCGCACAGCG
ACCGGAACACGGTGTCCCTGGTCTGCAAGGTGTGGTATGAGGTCGAGAGGCTGAGCCGGCGAGCTGTCTTCGTGGGGAACTGCTACGCTGTGCGCCCGGA
GCGGGTGGTGCTGCGGTTCCCCAATGTGAAGGCCCTGACGGTGAAGGGGAAGCCGCACTTTGCGGACTTCAACCTGGTGCCGCCCGATTGGGGTGGCTAC
GCGGGGCCGTGGATTGAGGCGGCAGCGAGGGGCTGCGTGGGTCTGGAGGAGCTGCGGATGAAGCGGATGGTGGTGTCGGATGAGAGCCTTGAGCTGCTAG
CTCGGTCCTTCCCGAGATTCAAGGTCCTCGTCCTCATCAGTTGCGAGGGGTTTAGCACCGATGGGCTAGCAGCTATTGCGAGTCACTGCAAGCTCCTGAG
GGAGTTAGATTTGCAGGAAAATGACGTGGAGGACCGTGGGCCTAGGTGGCTCTCCTGCTTCCCTGATTCCTGCACTTCTCTTGTCTCCTTGAATTTTGCC
TGCATCAAAGGGGAGGTAAATCCTGGTTCATTGGAGAGACTTGTTTCTAGGTCCCCGAACCTACGCAGTTTGAGGTTGAATCGATCTGTATCAGTAGATA
CACTCTCAAAGATATTAGTACGCACCCCTAATTTGGAAGACCTGGGGACAGGGAATTTGACGGATGAGTTCCAAACTGAATCCTACTTCAGGCTGACTAA
TGCTTTGGAGAAATGCAAAATGTTGAAGAGTTTATCAGGATTTTGGGATGCTTCTCCTGTTTGCGTTCCATTTATCTATCCCCTCTGTCATCAACTAACT
GGACTAAACTTGAGCTATACTCCCACTCTGGATGCTTCTGATCTCACTAAAATGATCAGCCGCTGTGTGAAACTCCAACGTCTTTGGGTACTGGACTGCA
TCTCAGATAAGGGTTTGCAAGTGGTGGCCTCCAGTTGCAAAGATCTACAAGAACTCAGGGTGTTCCCATCAGATTTTTATGTTGCTGGTTATTCTGCCGT
GACAGAAGAGGGGCTTGTTGCAATATCGTCAGGCTGTCCAAAATTGAGCTCTTTGCTGTATTTCTGTCACCAGATGACCAACGATGCACTTATTACTGTA
GCTAAGAACTGCCCAAATTTCGTAAGATTTAGACTCTGTATTCTTGAGCCAAAGAAGCCAGACGCCATGACAAACCAGCCGTTAGATGAAGGCTTTGGTG
CAATTGTTCGTGAATGCAAGGGGCTAAGGCGACTGTCAATGTCGGGTCTTCTCACTGACAAGGTTTTCATGTATATTGGAAAGTATGCCAAGCAACTTGA
GATGCTTTCTATAGCATTTGCTGGAGATAGTGATAAGGGGATGATGCATGTTATGAATGGATGCAAGAATCTAAGGAAGCTGGAGATTAGAGACAGTCCA
TTTGGTGATGTTGCGCTCTTGGGGAATGTTGCCAAGTATGAAACAATGCGATCCCTTTGGATGTCATCATGCAATGTCACATTAAAGGGGTGCCAAGTCC
TTGCGTCTAAGATGCCGATGCTCAATGTGGAGATCATGAATGAGCTAGATGGAAGCAGTGAAATGGAGGAAAACCATGGAGATCAATCTAAGGTGGAGAA
GCTATATGTTTACCGCACAACTGCTGGAGCGAGGGATGATGCACCAAATTTTGTTAAAATCCTATAGTTAGCTGAGAGTAGCCTGGTATCTCCTCACTCT
AAAAG
>comp81084_c0_seq3
GTTGCTTCTGGCTCCAAATGTTCCTCTGTTGGTTTGTCCTTTCCTTTCCCCTTTGCTTTATCTTTATTTTTCGCCTTTTGTTTCTTGATTTTGCTTGGTT
TAGTTTCTTCATCAGACAGCTCCTCGGCGAAGAAATCATCGTCGTCGTCAGGCTCATCTTTGCTGCTATAGTCATCATCATCATCATCCTTCAACCCCCT
CTTCGTAGCCTTCCTTTTCTCTTGGTGCCTCTCCCAGACAGTCTTCTCGTTGGTCTTTCTCTCTAGGATACGTTTACTGAGATCCTCAAGCTCTGTGTTG
AATGTAATCTCCATATCCTGGCCACCAGCTTGTTCCTCTTCAGATTTGTCGCCTTCCAGTAAAAGAGCCAACCTCTCCTCTTTTGTTAGCTTTCGTTTGG
ATCCCCCATTTGGTAGAGACTCATTATCAGGATTGTCTACACCGTCATCATCTGACGCACTGTCATCACTTGCTAAATACATGTCAAGCTCATCTAACTG
ATCATCAGTGAACTTTCGCCTCAAGACCTTCTTACGTTCTGGTTCATCATCATCCCAAGTTAGCTTAACTCTGCTATGTTGCAGAGCACGAGTCTCAAAA
TCAGGCTCCTTGTAACTCGGAGGTGCCTCTGTAGCAATGTCTCGAGCAGGATGTTTGAACTCCATAGAATCAGGTATAAACTGCAAATCAAATACATTTG
CTGTTTTCAAGAACTCAGTACCATCAAGATTCATGTACAGGTGATTTGCTGTTGCACTAGAATCACAGACCACAACAGCATAATAATACCTCAGTCTGTT
TAGCTCATAAGTACGCAACTTGTTATTCTCTTTGTCGGAGTCAACTTCTTCGTCGTCGTCATCATCAGTGTTGTCTTCATCATCATCGTTGCTATCATCG
TCGTTGTCATCATCATTATCATCAATATTGGCATTAACAAGAGCAGATGGGCCCTGAGTTGACTCAATTTCCATGCACTTCAGTCCAAATTCTGATGGAT
ATATTGAGACTGATAAAACTCGCCCACCTTTTGGGACGCAAGATGTCATCACCATATACAGGTCAACTGCCTTGATATGATCCCAGTCCATATTAACAAC
AGCAAGCCTGTGAGTTTCCTTGTCAATCATAGGCGTATCATCGTGCCGGCCCATTAAGTAATGAGCAATGTCACTGCCGACAGAGTACTCGTCATCATCA
ACGTCTTCATCCTCGTCGTCGTCGCTACTAGAACTCTCTTCTTCCTCCTGCTCATCCTCTTCATCCACGTCCTCGTCCTCCTCTTCTTCAACTAACTTCT
CTTTCTCCTTCTTTGCCTTCTCTTTAGTCTCATCCTCCTCCTCGTCTTGGTTGAGATAGTAACGGAGCATGGGGTTCTCCCTGCCGCTCTTCTTCCGTCG
CCTGCCGCGCTTGTCCACCGGCGCCGCCGAGGAGGAGAACCTCGGGTCCGTCATCATGCTGCTGAACCTCGAGTCCAGCGCCACCTTCGCCTCCTTCCGC
CGCATCGACCGGAACCGCGGGTCCATCCGCGCGGCCGCGAACCGCTCGTCCTGCATCGCCCGCTTCATCCTCTCGTCCCGCCCCTCCACCGCCTCCGCTT
CAGCCGCCTTGCCCTCGTGCTTCCCCTCCTTCTTGGGCTTCTTGCCGTGCTGCCGCTTCTCGTCCTCCTCGCGCCCCTCCTTGTGCTTCCGCTTCTTGCG
CTCGGCCTCGGGGCGAGGGGCTTCCTCGGTGGCCGCCTGACGCTCTTGATTCTTCTTCTTGTGCTTCCTCTCTTCCTTATCGGACCTCGATTTCTTGGCC
TTCTTGAGGCGCGCGGGCTCGTCGGAGCCGGTCGGCGG
>comp83121_c0_seq56
GGAGGCAGCAAGCTTGAGACAACAGCTGCACAGCTTGCAAGAAAGTCACCGACAGTTGATGGGACAAGATCTTTCAGGATTGGGTGTCAAGGAGCTCCAA
AATCTGGAAAATCAGCTAGAAATGAGCATACGTTGCATCCGGACAAAAAAGGACCAAGTCTTGATTGATGAAATTCACGAACTGAATCGAAAGGTTCTGG
AATATTCTGTTATTACTCTGCAATGAGAGGTCTCTGAGTGGATTTCAAGAAGCTGACTCTACTGGCTCATTTTCAGGGAAGTCTCATGCAACAAGACAAG
ATGGAACTATACAAAAAGGTCAACCTAATCCGTCAGGAAAACATTGATTTATACAAGAAGGTATTTGATAAATCGTGAATGAAAATTCACAGGATATACA
CCCCATCCATTATTGACTGAGGTTTATTTCTCTACTCAGCTCTACGAGAAAGAGGTGACAAGTGAAGTCAACCGAGATTCATCAACTCTAAACTTTGCAG

TTGTCGAGAACGTCAACATTCCTGTTCATCTTGAACTTAACAGTCCACCGCAAGAAAATGACGTTGAGCAAACTGCACCCCCTAAACTAGGGTTACAACT
AAATCCATGAAGGCATGCAGCATGGCACTGCTAATGTTCATTATTTTCAACGGCTAGGATTCAAAGCAGCTAGAATACCATTGGTTGCCTAAACACATAG
CAAGACAGACGTGGCATGCTGGGCGACGGTAATGGTAGTGTAAATCTATTTTCAAATAAAATGTATTTTATTGAAGACTACAGAAAATTATTTGTCTTAT
AACTATTTCACGCATTCTGCAAGTTTGTTCACCAACTTGAGTACATGACAGCTACAAATC
>comp79344_c0_seq7
CTCTCTCTCTCTCTCTCTCTCCAGCTAGCCGCCGTTTGACCCTGCAGATTGCTTCCTTCTGCACGGCGCGATGGTACGGGGAAAGACCATATTGAAGAAG
ATCGAGAACGAATCAAGCCGGCTTGTGACCTTCTCCAAGAGGAGGGGCGGGTTGTTCAAGAAGGCAAAGGAACTGGCAATCCTATGCGACGCTCAGGTCG
CTGTTCTCATCTTCTCCAGCACCGGCCAACTCTACGACTTCTCCAGCTGCAGCATGAAATCAATAATCGAAAGATACCGCCAAGTAAAAGAAGGTCAGCA
AAATGTAACGGCAAGTAGGGAGGCCAAGTACTGGCAAGGGGAGGCGGAAAGCTTGAGGCAGCAACTACACAACTTGGAAGAGAATCATAGGTAAGAAAAT
CCACCAACAGTATTGACAGAGGTGAATACTTTCTTACTGAGCAACCTTGGATGAAACTTAAAATGCAGTGTAATGGAGTCCATCTAATAGCAACGAATGT
GAAAACTTTAATAACAAGTCATATATTTTTATGTCCTTAGGGAGCCATTGGGGTCCATGGATTCTGTAACATCCTAGCTGGCATGTTTATAGTGGTCTTG
GCCTATGTGGTCTAGATGTTGAAGTGTGATGGGATTAGTTCTATCTTGGGTCTTTAGGTAGGTCAGAGCCTACATATAAACCTTGTCTTTTAGAACATAG
CACATCTGGATTAACCCTTTTACCAGAAGTGAACATGAAAGCATAAGAAACATGCAATGCCCATGCGTGAGCTTGCTCGATGTGGGACTTGATAATAAAC
AGGTTTTAGATATAGGGTGTTACAAATTCTTATTTGTGGTTGACAGAACAGGGAGCATGCATTGTTGTAGGGGAAATCATGCAAACGTTTTCAGGTTGTC
AATTTGGCACCACAGTACAAGCCTGAAACACAAGATAACAAGTGCAAACTGCCACAAGATGTACGAAAATTGTGAGAAGATCAATATTACTGCAAGCAAT
GTATCACAGCCAGGTGGTTATATAATATAGAGAAGTAGAACGCATGACATCTACCTTCAACAACTCCTGCAGGCACTTGCTGGGACAACATCTTTCTGGC
TTGTCAATGAGAGACCTGCAGACTTTAGAGAACCAGCTGGAAACGAGCCTAACTAATATTCGGCTAGCAAAGGAACGAATTATGATTGATCTGATTCAAG
AATTGAACAAGAAGTCAAGCCTCATAGACATCGAAAACAAGGAGCTACACCACAAAATCTGTGCCATTAGTCAAGAGAATGCCAATTTGAAGAAAGAGGT
AACACAAAGAGTATCCACAATAATGTCTTAATTGTGAGCTATCTTCCGTCAGTGTTTATGTCTTTCCATTCATCAGGTACATGGACAGCCGGATGCCATT
GAAGAAAATACTGGTTCTGTTACTCAGTATAGTACTAACACTCCTGAAGACCTAAATGCGACGTGTCTCGAGCTACGCCAACCACATCATGTAGAAATAG
AGCAACCAGATGTGCCTACATTGGAACTGCGTCTCCACTGATTGCAAGAATCAGCAGCAGAATCATCGGAAATGAGGAGGCCAGCCATTCAACGCAAATT
CATAAGACTTTGTCTCCACTGATTAAATACAAAGAGGAGTTCCTGTGCAAATTACTATTATATGAATGTAACAGATGTAATTTATTTTTTATGTATCCGC
AGTACACATATGGCCACCATAAACTTATGTTCAAAAGGCATATATACCTATTGCTATCAAGCTACG
>Locus_7399_Transcript_60/60_Confidence_0.131_Length_3993
TTGAAGATGTTGTTCCACATAAAGAACCGATATTCAGACATGCTGACACCTGTCCAGCGAGACGAGCTGGACACCCGTGTGAGGCAGTTAGTTTTTGAGG
AAAAAGACGATATCAGTGACCCAAGTGTCAATTGTGGCACAAAAGCAGTGAATGTTTTGGCTCCATTTGGGCAGGTTTCATTAGGGAGGCTACCATTTGA
ATCAGGAGCAGCAAATCCATTTAGTGGCTCTAGCTTGCCGAGGTTGGAAATACCTGCAAAAAGGATTAGTCCCTTGTTGGATCTTCATGCAGATTATGAT
GAGAACAGCTTGCCTTCGCCCACTCGGGATAGTGCGCCATCTTTTCCTGTGCCGAGGCCCATTGGGTTTGGAGCATTTCCATTGGCACATGAGAAACTAT
CTTTCCCAGAAAGAGCTGAGCCTCCAAAGAATTCGTTGTATCCATCCTTAAATGATCCTCTGAAGGCTGTTTCCTCTTACCAGCAGAAGTACGGGCAGAA
GTCTGTCTTTCCAAGTGATGATCTACCAAGTCCAACTCCTGGTGATGAGGGTAAATCCGCAGACAGAGGTGGAGACATATTTGGTGAGGTTTCCAGCTTC
TCAATTCCAAAGAAGACTGCACTTCCAAGTACAAGTCAGATGCCTGCTTCTTGGCCAATCACTGTTGGCAGCAGCAATGATAGTTATTCAGGTGCCCCCC
CTGGTTATGCTAAACAAATTGAACAGTCGGCTGCAGGACCAAATCATGGACCAAAGGCTACATCTAAAAGTAGGGATCCAAGGCTCAAGTTTTTGAACCA
TGATTCTGCTGGTGCTACCGATGTGAATCGGCGTTCAAATTTGTCAGAACCAAAGGAAGGGAACTTGGGTGGTGGAGTATCAATTAATAGTCGTAAACAC
AAGGCAGTTGATGAAAGTAAGGTGGATGAAAACATGTTAAAAAGGCTTAGGACTGGTACCGGAAACCCAAGAGACATGCTGGTGTCAACAGGGAATAATC
CTAATCTTAGAACAGGTAATCAGCTTATTAACAACATAGGTGGTACCCACAACTAGCAGTGGAACAAATACTAAAATTTTGCAACCCCCTCAAACTACTGC
TCTACAAACTAGTGCAGCTCCTGCTGTTTCTTTGCCTGATGTGTTGAAGGACATTGCTGGGAACCCAGCAATGATCATGCATTGGATTCAAATGGAACAG
CAAAAGATGTCGGCATCAGAACCTCAGCTGAATGTGGTTGCTCCGGGTGGCATGTCTAGTGGCATGACCAGTACTGTAACTGCAGCAACGGTTTTACCAC
CTGGCTATACTCCAAAGGCCACAGAAGTTCCACAAATTCCATCTGTTAGGCCACATGTTCCAGTGCAGACACCTCCTTTGAACCAACAAAATGATGCTGC
TGGAATTATACGTATGAAGCCCCGTGATCCCCGGCGGATCCTCCACAGTAACATAGCACAGAAGAATGATACAGTGGGCTTGGAGCAAGTGAAAACTAAT
GGAGCTACCCAACCAGACACTCAGGGCAGCAAAGACCATTTAACCAACCATGAACAACAGCCAGATCAACAGCAGACTAGCTCATTGCCTTCTCAACCAG
CCTTGCTGTCAAACATTACGCGACCATCCACCATTAACTCAAAACCTGTTGATGCTGTCTCAAACTCACAGTTAGCTGCTACAGCACTTATAGCTCCAAC
ACAGCAAGCTTTAGGCAGCAATAGGGTAGATCCAAGACTAGTGGTTGGACAGAATGGTCCAAATGCTGAAGCAGCAACAAACGCTCCTCCTGCGCCAGCA
CTTGAAGCTGTGCAGCCAGTTGATCCGTGGGGTGATGTTGACCATCTCCTTGACGGATATGATGACCAGCAGAAGGCACTCATACAAAAGGAAAGGGCAA
GACGGATCTCAGAACAGCAAAATCTGTTTTCGGCAAGGAAACTATGCTTAGTCCTTGATTTGGATCATACACTACTCAATTCCGCAAAGTTTGGAGAAGT
GGACCCTGTTCATGAAGAGATTTTGCGGAAGAAAGAGGAACAAGACAGGTCTTTGCTAGAACGGCATCTGTATCGTTTCCAGCATATGGGAATGTGGACC
AAGCTGAGACCAGGAATATGGAACTTCCTTAAGAAGGCGAGTAAGCTTTTCAAGTTGCATCTGTACACGATGGGGAACAAGCTGTATGCTACTGAGATGG
CCAAGGTTCTCGATCCAAGCGGGAAATTGTTTTCAGGGCGGGTCATCTCAAGAGGTGATGATGGCGATCCTTTAGACAGTGATGATCGAGTCAAAAGTAA
AGATCTTGATGGGGTATTGGGGATGGAATCTGCAGTAGTGATAATAGACGACTCTATAAGAGTCTGGCCTCACAACAAGCACAATTTGATAGTTGTAGAG
AGATACACCTATTTCCCCTGTAGCAGACGTCAATTTGGCCTTCCTGGACCATCGCTTCTTGAAATTGATCGAGATGAGAGGCCAGAGGATGGCACACTTG
CTTCTTCACTGGCGGTTATTGAGCGAATTCACCAAAACTTCTTTTCTCATCCTAACCTCAATGAGGCTGATGTGCGGAGCATACTAGCATCTGAGCAGCG
GAGGATTCTCGCGGGTTGTTGTATTGTCTTTAGCCGGATTTTCCCGGTTGGAGAGGCCAACCCCCACTTGCATCCGCTATGGCAGACCGCAGAGCAGTTT
GGTGCAGTTTGCACAAACCAGATTGACGACCGGGTAACTCATGTTGTTGCCAACTCCCTTGGGACCGACAAGGTGAACTGGGCACTAGCCAATGGCAGAT
TCGTGGTTCATCCAGGATGGGTAGAGGCTTCAGCTCTATTGTACCGGCGTGCCAATGAGCTTGACTTCGCAGTCAAATAACACATCCCTGAGCCCCAATT
CTCCATCTAGGGTCCCAAGATTCTTCCGGGGATGAAATCCGGTCATGAAAAACTAACATGATAAGAAACCAAACAACATTCTAAAACCGGACCCCATTAA
GAAGCAGACCATACCACCTCTAACAGAATCAGGCCTAAACAGGAAAGAACGAAAATGCTAAGTGATAGCCTGACAGAGGGGATGGCGGTCGATGTCATTA
CCAAGGTGGTGAGTATTTTGGTTCAAAAGTGCTTTAGAAAGAACCATCGGAGAAGGATGGCTAAAAGTGAAGTAGATGATCAAACGCTCATTACCTCCCC
TTTTGTAACATGTAGTAGTGGGGCATTTTTGGTATAATCCTAGTCAAGATGTTGCCTCCGGCTGCAAGCCTGCTGCTGGCCACGGAGTTGGGGTTGGTAG
CAGGTGATGGTCGCCTATCAAAGTGGCAACGCCCAGCAGCGTTCTTGGTAATGAAGTCATGAACGAGGCGGGCATCTAGAGTCTGATGGGTATTTTGTGG
CTTGGCTGCAAGTGTGTAGTTGGTTCTCGCTGTGACCTGAAACTGAAAGCCACATGGAAAAGGACACCTGGGGCTGCGCCTCTGCTGCTCAACGAAAAGA
GTGTACAGAAAAGGGTCCAGAAAAGGGGAGTGGAGAACTGTTGTAGAATACCAGAACCAGTAGACACCTGGGCGCTTTCTTGTATGAAACCAAGTAACCG
GAGTAAATGGTGGCACAGAAGAATGCGTTAAGTGTGATCAGTTGGATACTGGTTATGGAGAACGAAGCGTATGACCGGGTTTTTACTGAAACTGTAAGCG
ATTATTGGGGTTGCTCGTGGCATGGCTATATTCTGTAAGGTCCGTTCGAAGGGTTTTTTCTTTTTCTTTTTGGTGTGGTTGGGGTGGGGGTTGTTTCATG
ACAGCGTTTTTACCGTTTCTTTTTGTGATTCTTTTTAAAATTTATTTGTAAAATATGTGTTCCAAACTGGTCCAAATGTGCGGGGCTTCATTCTAATTTC
AGTAAGTTAAACGGGACTTCCGTGGTGAAGCTTTAAACAACTGCTTAGTCTTTTTTTTTAAAAAAGGAATTGGACTTTCGGAACTCCAGCACC
>comp67351_c0_seq28
GGCAAGACGGATTACGACGTGAGAGGCGAGAGTAGCGAAGAGTCACGGAGATCAGGGGAATCAAAAGATGGAAGTGTTGGTCAAGAAAACAACCATGCAA
CATCACATATGCCTGCTTTGGTGTCGGAGTATTTAGCACCATATACACAGCTGGAACTGAACCAATCAGTTGTGTGCAATCTTTTCTCCATGGACTCTGT
TTATTGCCTGTGTTTCCATTTTCTGTTGACTTGTCTCTAATGAGCTTATCGTCTCATGTTCACATCTTTCCTTGTGTAGGCTCCTGCAACATATCAGTAC
CCAGATCCTTACTATGCAGGCATGGTTGGTCCCTATGGAACTCAACCTGTGACCCATTTCCAGCTACCTGGGTTAACTCACCCTCGCATGCCATTACCTC
TTGAAATATCTGAGGAGCCTGTCTATGTAAATGCAAAGCAGTACCATGGAATTTTAAGACGAAGGCAGTCACGTGCCAAGGCTGAGCTTGAGAAAAAGGT
GGTTAAAGTCAGAAAGCCTTATCTTCACGAATCTCGCCATCAACATGCAATGAGGAGGGCAAGAGGAAATGGGGGTCGCTTCCTAAACACAAAGAAAAGC
GACAATGGTACTCCCAACGGTAAAGCTGAACCTAAGAAAGGAGAGCAAAACTCCAAGCATATCCATGTCCCTCCTGATTTGCAACTTCGACAGAAGGAGG
CATGAAGTAGCGGTCTGGCAACCTAAAACAGTGGCTTCTGTCCACAGGCATACACACTTTTGAGTCCGGATTTGGAGCCCCAGGGTTGCGCTGCCATCTT
TGCAGTTCGTCTTCTTTGTGCAAAGAGGTCCAAGGTTCGTCCACCCGAATGAGCTTGGTACCAGTTGTTAGTCCTGGCAGAATGCAATCTGGGTGCAGGA
GACCGTTGAAGTGTCCAATGCCATGGGTAGGTCTGCTGTTTTCAGGCAATTCATTCTTGGCTTTCACATTCCGCTCTTGTTTGTACCTGGTGTGTTGTAA
GTGGAACTAGCTTGTGTTGTGTCTTCGGCGTCCTGTGAGACATGTATAATTAAGATCAACTTGGGATCATCGTTGTAATCCTATGTCTGGCTTTCTTTTA
GTTTTGAACTGTAACAAGGCCAAAGCTGAACTCTTTATACAGAGATAGCAACTCTTGCTGGCCTGTTCGTTGTTCAGCAACGTCAGTGCTGTACTTAATC
CTGTGCAAATGAATAAGCTT

>Locus_10929_Transcript_55/78_Confidence_0.216_Length_1883
GAGCAGGGATTGCGGGAAGCAAGTCTACTTAGGTAAGTGATTGAGTCCTATACGTTCCAGCTCGAGCTTCTATTAACTGCTGATCAGTTTCCGCCTGTTT
GATCTGGTTTACGATGTGCTGATGCTGCTTCTATTCAGGTGGTTTTGACACTGCGCACGCGGCTGCGAGGGCCTACGATCGTGCTGCGATCAAGTTCCGA
GGGCTCGACGCGGACATCAACTTCACTCTGAACGATTACGAGGATGATTTGAAGCAGATGAGGAACTGGACCAAGGAGGAGTTCGTGCACATCCTCCGCC
GCCAGAGCACGGGGTTCGCGAGGGGGAGCTCCAAGTACCGCGGCGTGACGCTGCACAAGTGCGGCCGCTGGGAGGCGAGGATGGGTCAACTTCTTGGCAA
GAAGTACATCTATCTTGGACTCTTTGACAGCGAAGTTGAAGCTGCAAGAGCATATGACAGGGCAGCCCTTCGCTTCAATGGGGGGGAAGCTGTTACTAAT
TTTGGGCCTAGCTCCTACAATGGAGGAGATGCTCTACCCGACACCGAAAATGAGGCTGTTGTTGATGGCGATGCGGTTGATTTGGATTTGCTTTCACAAC
CTAATGTACACGACGCTAAAAGGGATAATACCTTAGCTGGCTTCCAGCTAACATGCGACTCCCCTGAATCTTCAAATATGGTGGCCTCTCATGTAAACTT
TCTTCACGATTACACCCCATTTAAATGCTCGGTTCCATTTTTTATGACTTACTACTGTTAGTAGTAGCTAAAAAGGTACCCGGAAAAATAAAATCTTAAG
ATGGAAGCAATCTAGGTTGAATTGTCTTGTATCTCTGTGCGGAATCCTTGACCTTTGTTAGTTCCTAAGCTATAAAGATTCATAGGAGCATTTGATTGGC
GTGTCGTTGGTTGAGATATTAACTATTAGAGTGTCCCCAATGAGAGTTAGCTTATACTAGCTCTAAACATATTCATGTTATTTCATATATTGCACTAAAG
TTTAGATTTTAGTAACTATCTTTTAGCTTTTTTTTAAATTTAATACTTACGTGAGTATATACATCTCGTTTAGTGCTATAGTTTAACTCTTCACTAAGAG
TTGCTAAGTTTTTTATATTTAATCTCCTTTACATAAAGTAAATGATGACATAGACATGTTTAAAGCTAGCTAAGGAGGCTTCTTCGGGGCTCTTAGTTCA
GGTCTTATAACCTTTATTGAACTTACGACATTTGCACCTATCTGTTGCTGCTGTAGCCAATGAGTTCATCGTCCCCGTGGCCTGTGTATCAACAGAGCAC
GGCAGTACCACCTCACCATCAGCGTTTGTACTCATCTGCTTGTCCTGGCTTCTTTCCGAACCTCCAGGAAAGGTCAATGGAGCGGAGGCCTGAATTGGGT
CCCCAGCCCTACCCCACCTGGGGATGGCAAATGCAGGGCTCCCTTCACATGCCGTTGCACCACTCTGCAGCATCATCAGGATTCTCTACCGCCGTCGGCG
CAAACGCCGGCGTCTCGTTGCCTTCTCACCCGCCGGTGCCGTTCCCGGACCACCAGTTCTACTTCCCACCGACGGCATGAGCTTGTTGGTCTGGTTCAAC
TCTTGGGACGGCAGCTGATCTTATGTGTGAACATTTCGCAGCTTGCCGGTGACCGTTGTTAATTAATCGGGGAGAGAGAGCCAGAAGACCCAGTATGGCT
ATCTCTCTGTGGCCCGTGCCTTGTTATGATCTTAGTTATTGCTACTACACCGTGGATTCATCATGGTTTATGAGATTCTTTACTCAAGTTTCAGCTTAAA
TGTCCAAATACATGAATGCATCATACACAGGATCCGTCATTTTCTAAAAAAATACAGGATCTGTGTGAAGTGTAAACATAAGG
>Locus_21273_Transcript_7/23_Confidence_0.500_Length_1513
TCCAATTATATCATTCGTTCTCGAGCTTAGCAGCATCTAAAATCTCGATACAAATTAGTCCTCTCTATCCCAAACAACCCTTCTAAATAAACCACAAACA
ACTCATGTAGCCTTGCTACGGCAAGCCAGTAACTTTGCTTCGGGGGTCCGTACCATTTACACTTTCTTCTTCCTAATTTTTGCTCTGCCCCCACCAGAGG
AAGGGACGGCGGGGAGGGGGCAGGCACAGAGAGCAGCTTTGCTTGGCGGACGCACCGAGGGAGGCGCCTGGGAGCCCTGCTTCTCCCCTCTTCGTCTTCC
TCCTCCGCTTCCAAAGGTTACCGTCTCCCGCCGCCATAAGACTAGTAGCCTTCTTGCTGTGTCAATCCGTCATGCACGCGTCCCTGGATTGCGCTGGTGA
CTCCTTCGGGAAAATGGTTAATGATCATACGAGGTCAAATTTGGTTTTTGATAATAAGCAATCTCTATTTGCAAGTCATAACATTGACTACGGCCAGCCA
ATAGCTTGTATATCATACCCATACAATGATTCGGGCTCAGGAGGTGTTTGGGCAGCCTTTGGGTCACGCACTAGCGCTGCAGCTGTGTTCCATCCCCAAA
TTGCTTGTGGGGGCACATCTGCGAGAGTTCCCTTACCTCTGGAATTAGCAGATGATGAGCCCATATATGTCAATCCCAAACAATATCATGGTATACTTCG
CAGAAGACAGCTACGTGCTAAGTTAGAGGCTCAGAACAAGCTAGTCAAAAACCGAAAGCCTTACCTTCATGAGTCTCGGCATCTTCATGCAATGAAGAGA
GCAAGAGGTTCTGGCGGACGTTTCCTTAATACTAAACAGCTCCAGGAGCAGCAGCAGCTGAAGTCTCGCAATGCCTCCACCAGGTCCACCACAAATGGCG
CAAATTCCTCAGGTTCAACACATCTACGGCTTGGTGGTGGTGCAGATGGAGATCAAACCATGTCGGGGACGAAAACAATGGCCTCACAAAACAATAGCAA
AAAGGCTGTTTCTTCTTCTGCAGCTCTTGCTTTCACCGTGACTCCTATGGTGCGCAAAGATGACACCTTCTTCCAGCACCTCAGCCACAATGTCAGCTTC
TCCAGCCATTTTGGCCAGGCAAGCGCCCAAACCGGCGTTGGAGGCATCCATAATGAGACCCAGCATAGGGTTTCCGTGATACAATGACGGTTTGCGAAGC
TTATCTGGTGATCCAGGCTTCTAGGTGTCCTCGTGTCCGGTGTAGTCTTGGTCGCTCAGGCAATTCATCCTTGGCTTAGTTTTTGGTGTGTTAGAACCTA
CAAATGTTTCATACTCTGTTGTGGTTTGCAGAGCCCATAAATCAGGGCTCTCCTAAAAAAAAATCAGGGCTTGATGGCGACACTACTATCCAAGTATTGTT
GTAATGGTGTGTGTTAGAAACTCGCAAAAAACCCTTCTTTGTGCTCGTATTAGTACATTATGCTGCACAATTAGCTGCTATTCTGTTCGTACTACAGTCT
GGTATGCAGTGGT
>comp67036_c0_seq8
GATTGCATGGGGTGGCGTACTGTGGGCCGTGGAGTTGATGGAATCATGGAAATCCGCAGTCCCTTTTGTTGGATAAGGGTGGTTGAAGCTAATTTTTTGC
GAATTGTGGCGGTTTCTGGATATCCTGGTTGAATTAGTGGTGTGATAGATTGTTCTAGAGAATAGGAAGATATATGTTTTATTGTTAGAACATCAAGAGA
GTGTTGAATCAGGTATGTCAATGTGTGAACTTATTGATGGGAGCTAAATGCTTGCTGACGTATCTTCGCTATAATGACCTAATCAAACCTAACAAGCACT
AAATACTGATCAATTCTCAGTTATACAAAGATTTTCCAGAGAGTTCTTCAGAGCTCGAACTTCTGGGATGAAAATAGTTTGGCTCAAGATAGTCCACTGG
AAGGAGTTTAGGATATCTGTTTGAGCTCTGCCTGCAATATATGGAAATCTGAAGGTGATATGGTAGTAGAGTTGGGGACCCTGATCCCCGAGCCAAAAGA
AGTTCGGTAGAATTTCTCAGGAATATTGTGGTAGTAAGAAGCAAGTTAAAAGGAACCTAATTAGGAAAAAAAGACATGCCTTGCTTGGTCATGAGAAAAC
ATTATTGCTGTATAAAATAGGTATGCCACCTCCAGTCAGTACTTGATTAATTTCATTGAAAATGGAGGGCTGTAATAGATCCTTATCTATTAATGTGTTG
TACCTAAACTTGGAAATCATCAGCTGAGGGCATTGTGCATGGTGCGTGGAGTACTAGCCCTCGGGAGATGCTTCTTAATTAGGCAGCGAGAAGGATAGAG
ATCGGTTTGACATATGTTCCATTTGTAGCTGTACTGGATATTTCTGCATGTTCCCCTGGAGCAGTTAGTTTCAGAATTATGATATTTCTACTTGTGGCAG
GATTGTTCTGTTTCTATTATTTGTTCATATTTATGCTAGTATGTTTGTCTTGTCTGTTGCAATATCTATTGTCTATTGCACTTCAAGTGACTATGCATAA
CCATATTCCAGTGATTTAGTGAATGGTTATGAATTAAAGTAAGATGACGTTTTCATCAATTATCCTTGTTCTTTATCACGTCATTATCAATTCAGCTGAT
TAAATTCTGGCATCTTGTCATGATGGTTACTTTAGCTGATTTAATGTTTCTCAGAGCTAAGGATACCATCTCATAGTGCTCACTCTTTTGTGGGAAATGG
AGGATCCCATGCCGAAGTCCAACAATGGCTTCTTGTCAGGAAATGGCTATCAGATGAAGCAATTTGGTCATAAAATCGACGATAGGGACTCGTCATCAGA
GTCCGGTCGGTCTCACCAAGAAGCTTCCACAGCGAGTGAGAACAGTATAAATGAACAGCACACCTCAACACAATCTGACAATGATGATGGTCATGGGAAG
CATAATGAGGACATGATGAAGTCAGCAGTGTCCTTGGGGGAACCAGGATCAGCCTTTTTGGCCCCAAAACTAGATTACAGCCCATCTTTTGCTTGTGTTC
CTTATACTGCTGATGCTTTTTATGGTGGGGTCTTGACAGGATATGCTCCACATGCCATTGTTCATCCCCAGCAAAATGATTCAGCAAACTCTCGGGTTCC
GTTGCCTGTTGAACCTGTAGCAGAAGAGCCAATATATGTTAACGCAAAGCAATACCATGCAATCCTCAGGAGGAGGCAGATACGTGCTAAACAGGAGGCT
GAGAATAAGCTGGTGAAAGGTCGGAAGCCATACCTCCATGAATCTCGACACCGCCACGCAATGAAGCGAGCCCGTGGATCAGGGGGGCGGTTCCTCAACT
CAAAGCAGCTCCAGGAGCAGAACCAGCAGCAGCAGGCATCAGGTGGTTCAAGCTGCACAAAGGCCATCGGCAATAACACAAGCTCCCAAAGTGGTCCCAG
TCACACGCCTCTGACACTTGCTTCCTCTGACACTGCAAGCGCTTCGAGGGCCAACCAGGAGCGCACCTGCTTCCCCTCGGCTGGCTTCCGTCCCACAATG
AACTTCCGCGCGCGAGGTGGAGACGCAAAGCTAGCTGTGAATGGCATACAGCAGCATGTTTCCTTCATCAGGTGAAGCAAGTGAAATGGGGCGCTGCCTG
CGGTAACTCATCCTTGGCTTATGAAACTGTGAATGTGTAATGGACATGTAACTTCAGTCTCAGAATAACCAAACATATGGGAGTCCTCCTGAAGTTTCCT
GTAGTTCAAAGAACTGATAGGTTATGGCCTTATGGGTTTGCTTCTGGAGACTGAAATCCGTGCTTGATATACTATCTTGTGCGAGTTCTGAGGTGAAAGA
CCTTGAAACTTGCTCTTGTGGTTCAGTACCTGTTGCTTTTGTGTCTGCAAGGTTAGCTCTGCAATTTGCAATGTC
>comp80692_c0_seq36
GCAAAGGGAGGCAGCAAGCTTGAGACAACAGCTGCACAGCTTGCAAGAAAGTCACCGACAGTTGATGGGACAAGATCTTTCAGGATTGGGTGTCAAGGAG
CTCCAAAATCTGGAAAATCAGCTAGAAATGAGCATACGTTGCATCCGGACAAAAAAGGACCAAGTCTTGATTGATGAAATTCACGAACTGAATCGAAAGG
TTCTGGAATATTCTGTTATTACTCTGCAATGAGAGGTCTCTGAGTGGATTTCAAGAAGCTGACTCTACTGGCTCATTTTCAGGGAAGTCTCATGCAACAA
GACAAGATGGAACTATACAAAAAGGTCAACCTAATCCGTCAGGAAAACATTGATTTATACAAGAAGGTATTTGATAAATCGTGAATGAAAATTCACAGGA
TATACACCCCATCCATTATTGACTGAGGTTTATTTCTCTACTCAGCTCTACGAGAAAGAGGTGACAAGTGAAGTCAACCGAGATTCATCAACTCTAAACT
TTGCAGTTGTCGAGAACGTCAACATTCCTGTTCA
>comp76332_c0_seq40
ACTGCTCTGAATTAAGAGGCGTTGAAATATTGTCCGGTCTACATATAGGCACTTGAGGTGTATTGACAGACAGTTCCCCTTAGTTTGCTACAACACATCA
GGCGAATTCCACCAAATTAGGATGATGTTTGTTTCTTAGACACAGGATAGCAATGCCAAAAGTCTTTTATAATTTTCCGCTTCACCGCAGATTTGGCATC
TCAAAGGGAGTTGTGGCAAGATTCTCTTGACCAAGCTTTCTTGCAGATTTGGCAAGAAAGATAAAAAAAATGTTGGTATACTTAAGCATCCTTAATAGAA
ACTAAACAGGTCTTAGATTGAAATTATCATGAATAAACCCTTTTTCACTTTAGCTGTAGTCCGGATGAAATCTTAGGGGCCTTGCTTAGCCTGCCCTGAA
GCTTGCCAGTAAGTCGAAGCTGATGCTGACACAGCACTGACATAGATCCCAATGAACACAAGTTATTTCGAGAAGATAAAATACCAAGCAAAGCCAGGAT
TGCATTAAAAGATAGTGTATTTCTACTATACGATCTGAAAGCTTAATTTATTATATCAATAATCAAATTGGCAGCAGTGTGGAACAGATATAAAGAAGAT
GCTTCAAATCACAAATAACTACAGATAAATTGAAGGGTGAAGGTTCATTCAACCACCAAACACCCAAAATGTCGAGCTGCATATGCACAGAACCTAAGTA
CGGTACAGTTCATAGTTTGGGAAAATAGACTGCAAATGTGCACAGATAATACAGATTGTTACTCGATGAACAGTAAGCACGGTATTTGAAATCCTTCTAA

CCATCCATCCAGGCAGTGCCTCGTATACTCAAACCTCTTTTTGGAGGAAGCCAAACCATGTGATCCTTTTCAACCCAAATACATATATTCAAGATGAAAC
TAACAAAAAGACAGTTGATAAAGAAACACGAAAGAGAGTAGAAAAAGGGTACCTGTTTCAAGTTCTCATCTTCTGAAAATGATCAGCAGTCAATAGATGT
TTTAGTCCATTCTCCAGAAAAATGAGTGCCGCATGAATCCTTTCCGGTTTATAGTGAAACATTTTAGACGATGACCTTTAACATTCTAGAAAACGAAGAG
AAAACCTACAGTCATCTAATTTCAGCTTAAACATGTTCCAGCAAGATCATCACCATCCTGACATGTTCCGTTTTGTCCCTAGACCCGAGCACAAGGTATT
GCGAGGCTAATTTAGCTCAATTCAGTTGTCAGAGCAATTGTGTGTGAAAAGTGTGAACAACAGCATAGGAGACAATACAGCAAGTGTAGCGTGCCAATAT
GTGTCCAGCTTGATAGTTCACTATAATAAAGTTCTTTAATTCAAGTATTTGAATAATATAGAATCTCAACCTTCAAGTTTAAAAGGTTTTGTATTTGCTA
TTATAATCGTTGCCACCCCCTCACCCCTCTCAAAAAGAGAGTTACTTTGTAGCGCACTGTAAATCTCTTGATTGAAATAGTGGCATGTCATCTTATTTAA
TTCAGTTATTTCATAAGGTACAATTTGTTTAGCTGACAGCTCAGACATGCTTCTATGCAACATGTTTATACCAATGCTGTATTTTGGCAATCCTGGATGT
GGCATTACTTAACTGTGCTTTCATATTCAATAGTTCTGGCAAAGGGAGGCTGCAAGCTTGAGACAACAACTGCACAACCTGCAAGAAAGTCATCGGTATG
GCTAGAAAACCCTGCAAATTTTACAAATGTACAAGTCAGAAAATTTGCCTAATGTAAAAGGATCAAACTACAGGATTTGTCAGCATGAGCACAAGTGCCT
AACTTCCGAGTTAGATAACTCTGGAGGAAGGAAATCCTTCCTACTGTTTGTTTCAAGAATATACAAAAATTCAAAACAACATTAGAATAGAACCAAAAGA
AGAAACAATATTCACCATGTAGCAATACTCAATTCCTGGTTGTCTTATTTATAAAGGTATAAAACTAGGTTCTGACGAGACAATTATGAATCAACTACAC
CATGTTACAGATCATCCATAACTCTTGGTGATGCAAATTAAGGGCTGTAAAGTGACAAGTATACCTCCACCAACTAATTTGTGCAGAGTGTATAAAATTG
TAAAACAATGGATAAGAATGAATTTAGTATACAAGATAGCATACAATGCCCAAGTTTAAGGAGACATACTGATTTACTGACTCCATAGGACAATCTTTTG
CTTATGAGGACATCAGTTTTCATAAACAATATCAATGTAAAAGGAGGTAACAGTTTATGAATTTCCTATGCCAAGCCATTTATAAGTCAAAAAATGTACC
TATTTAGCTTTTATGCATTCCAATTAACTTCCAAAGAAGAAGAGTACATACTAATGGAAAACAAATATGATATTATCATCTGTTTTGCCCGGTAGTTTCT
TGAGTCTCTGATTTGTCCGCACTTCACTCCCAACCAACACAACATTAATACACATGGGCAGAGGAATATCAGGTTATCAGGTAAACTGAATACAGTTTGA
TTGACTGCAGTCAGTTGATGGGACAAGATCTTTCTGGATTGAGCGTCAAGGAACTCCAAAATCTAGAAAATCAGCTAGAAATGAGCCTACGTTGCATCCG
GACAAAAAAGGTAAGGATGCTAGCCATATTTCTGTTTTTCGTTACCAGATTGCCTCATCCAAGAATACAACTGATGATGACGTTTTTTACTTTCAGGACC
AACTCTTGATTGATGAAATTCACGAACTGAATCGAAAGGTTCTGGAATATTCTGTTATTACTCTGCAATGAGAGGTCTCTGAGTGGATTTCAAGAAGCTG
ACTCTACTGGCTCATTTTCAGGGAAGTCTCATGCAACAAGACAAGATGGAACTATACAAAAAGGTCAACCTAATCCGTCAGGAAAACATTGATTTATACA
AGAAGCTCTACGAGAAAGAGGTGACAAGTGAAGTCAACCGAGATTCATCAACTCTAAACTTTGCAGTTGTCGAGAACGTCAACATTCCTGTTCATCTTGA
ACTTAACACTCCACCGCAAGAAAATGATGTTGAGCAAACTGCACCTCCTAAACTGGGGTAACCCTGCTTTTTTCTGCAAAGGAAGATTTTCTTGCAAGTT
ATGCAGTTGTTGTCTCTAGCTTGCCGCCTCCACTTGCAGTAACTGAGACGCCGGCGCCGAGCTTGAGCTTGAAACCTAGACTACGGGGACAGGGTAGACA
GGAGACGCGAAACCCCAAACCAGCGCAAAACGACGCGCAGGGAGGGAGTGTCACTGAAGGAGCGACAGGGAAAGCAGCGGCACGCCGGGGGAAATTCCGA
TCTGAGAGAACGGATCAGCCATGCATGACAGGAGCTGCGGAGGCAGCAGGGGGGGGGGGCTCCCGCTGCCACGGGACAGTAGGGCACGCCAGATCGGTGA
AATCCAGGCGAAGGACGAGGCGTGGGCGAGATTTACACGACGGCGCGATAGGATCTAGCCCGGATTTGGATTCGACCGACGAGGGCGGAAGGAACGGCAG
GGTGGTGCTCTTGTAGAGAGAAGGAGTCGGAGGGGGCGGAGAGAGAGAAAGGGGGCAGGTGGGGTTTCAGGTAGTAAAATATGGAGCGG
>Locus_7001_Transcript_2/84_Confidence_0.086_Length_1076
GAGACTCCTCATCAACAAAGTCTGAGCAGTGTCGCCAGGACTTATCTGCAACAACAATGATAGTAGTGGAAAGCGAGACCAGGGCATGGTAAAGTCTGTA
TTATCCTTGGGAAACCCAGAAACTGCTTTGTCATCTCCAAAGTTTGATTACAGCCAGCCTTTCGCTTGCGTTTCTTATCCATATGCTGCCGATCCATGTT
ATGGTGGGATCTTAACAGGATACACATCAAATGTCGTTGTTCATCCCCAAATTAATGGTACAGCAAACTCTAGGATACCCTTGCCTATTGAACCTGCAGC
CGAAGAGCCCATATTTGTCAACGCAAAGCAATACCATGCAATCCTTAGGAGAAGGCAAATGCGTGCAAAACTGGAGGCCCAAAATAAAATGGTGAAAGGT
CGGAAGCCGTACCTCCACGAATCTCGGCATCATCATGCAATGAAGCGAGCCCGTGGATCGGGAGGCCGGTTCCTCACCAAAAAGGAGCTCCAGGAGCAGC
AGCAGAAGGCACTGCCTTCACTTCAGACTCCAACAGGTGGAGTCAGCAAAATGGCTCTCGGCAGGAATCAATGCCCTGAAAACGGCACATCTCGCTCGCC
TTCAACACCGACAAGCTCTGGGATCTCAAGTGTTTCAAACGGCGGTAGCATGCTGGCTCATCAAGAGCACACTAGATTCTCATCTACTAATTTCCTCCCC
AGCATGAACTTCAGCACCCAGAATGGAGGTGAAAAGATGGCTGTCAATGGTGCGCACCACCGTGCCTCCATCGTGAGGTGAACAGCCTCGATTGTGGGGC
GGTGGTATGTACCATCACTGGCGCGCTCCGGTGAGCCCGTGGCAATTCATCCTTGGCTTATGAAGTATCTGTATCTGGTAATAATTTGCTTTCAGTTTAA
TATGTAGAGCTTGTCCGAGATGAACTCATTTATATTTGTTGCAACTAAAACGAGGAGCTACTTGTTGATATATGTGGATATTGTGGATACAGAAATATCC
TGATGTTTCTTGATGTACCAAGTCCACTATTAAAGTATGACTGGAAAAAACGTTTAATAACGTAATCATGGTTTGG
>Locus_839_Transcript_94/261_Confidence_0.040_Length_843
AGGTAATCTCGATGGTTACACAAAGAGTGATGAAAATAAGATGATGTCAGCTTTATCTCTGGGCAATCCAGAAACTACTTATGCGCATCCAAAACCTGAC
CGTAGTCAGCCCTTTGCCATTTCGTACCCGTATTGTGATTCATTCTATGGTGGTGCAGTGGCAACGTATGGCTCACATGCTATTATGAATCCCCAGATTG
TGGGCATGATGTCATCTTCTCGAGTGCCATTACCAATTGAACCAGCTGCTGAAGAGCCCATTTATGTAAATGCAAAGCAATACCATGCGATTCTCCGAAG
GAGACAACTCCGTGCAAAGTTAGAGGCCGAAAACAAGCTGGTGAAGAGTCGCAAGCCATACCTTCACGAGTCTCGGCATCAGCATGCCATGAAGAGAGCT
CGGGGAACAGGTGGGCGGTTTCTGAACACGAAGCAGCAGTCAGAGGCTCCTGGCGGCGGCACCTCGGACGCGCAACTCATGCCACCAAATGGTGGCCTGT
TCGCGAAGCACGAGCACAGCTTACCACCCAGTGATCTCCATTATCGCGCAAGAGGGGGCGCTTAAGGAGTCCCATGGCAACTCATCCTTGGCTTAAAAAA
TGTGTGGCGCTCAGCAACTCGACCTCTGCTAGTTCCATGTAAAGGTGTGTGACTGTAAATGCTGGTGCTTGAATTGGCAGCAGCCATTCCTGCAGCAATG
CGTCGCAGGTGTTGCCGGTTCTGTGATAGTGCCAGACTTGTGACCGGCACAGAACTGGCATCTGTTTCCTGTAACTTTAGTTTTTCTAAGACGATGACGA
TGACTACCCAATAACCATTCTGTCTGTATGGTGCCTTGCGTTT
>comp82180_c0_seq41
AATATAGGGGGCCCCGCTCCCGCAGCTCGCAGTACCGCGGCGTCACCTTCTACCGCCGCACCGGCCGCTGGGAGTCCCATATTTGGGATTGCGGCAAGCA
AGTGTACTTAGGTGGATTTGACACTGCTCATACTGCTGCAAGGGCGTACGATCGAGCTGCCATCAAGTTCCGTGGCTTCGACGCCGACATAAACTTCAAT
CTCAGTGACTATGAGGATGACATGAAGCAGATGAAGAGCCTGTCCAAGGAGGAGTTCGTGCACGTCCTGCGACGGCAGAGCACCGGGTTCTCGCGTGGCA
GCTCCAAGTACAGAGGCGTCACCCTGCACAAGTGTGGCCGGTGGGAGGCTCGCATGGGTCAGTTCCTTGGCAAGAAGTAAGAACACTTCACAACCTGAAT
TACCTAATACTAGACGTGTTATTTCTATACTGCAGATCCTTGTGATTTTTCCTATCTCAGTCTCATTCTAGAGTCATGAACCTAGTACATGAACTCCTAT
GATTTAGTATTACTCCATATTGTCTAGAAATAGTAGGTTAGATTCATGTAGCATAACGGTCGTTCCTACAAAAGAAATTAAACAGAAAAAAGGGCAGTGG
ATAGGGAATTCCTCACCGGTGGGAACGCCATGCTTGGGTGCAGGTACATATATCTTGGGCTATTCGACAGCGAAGTAGAGGCTGCAAGGGCTTATGATAA
GGCCGCGATCAAATGCAATGGTCGAGAAGCTGTGACGAACTTCGAGCCAAGCACGTATGATGGGGAGCTGCTGACTGAAGTTGCTGATGAAGGTGCTGAT
GTTGATCTGAACTTGAGCATATCTCAACCAGCTTCTCAGAGCCCGAAAAGGGACAAGAACTCCCTTGGTCTGCAACTCCACCATGGATCGTTTGAAGGCT
ACGAATTGAAAAGAACAAAGATTGATGCTCCCTCTGAACTCACGGGCCGACCTCATCGGTTCCCTACTCTTCTGACCAAGCATCCACCAATCTGGCATGCCCA
ATCTCATCCCTTCTATTCAAATAATGAGGTTTGATGATACTAAATTACTAAAATACAGGCAATTACTTTCCCAAACATATTGCTTTGGCTCCTAAACCTC
ACAATTGCAAGCATGTTCTGAGCATCACATGGTTCGGATCGAGCTGTCCGGCACCAATTTTGAACGTAATCTTGACCATCTGAGGAACTGATTGTGCAGC
CTGAAGGCGTGCGTTCAAATGTTAGATTTGTGTTACTTTCGTTCATTTTCACTTCTTGTGCCGCTGTCAATTTTGGCTCTAGTCTTACATCACCGCTTCA
TTTCCTTGGATTCTATAGCCCGCCTGTTTCCTGTCGATTTCGGAGATATTAATGCGTAGCACAGGTTTCATATGTAGGATCTTGAGTTTTCTGAGATGGC
CCATTCTGGAAACAGTAATAGTCGCCTTAGGAATAGAGATGTGTTTGATTTTTTTCCTTCAAAAGGAACATCCTAGAGAGGTGACTATTCGACATTAGGT
ATGCTAATCCATCGGTCAACTATACCTGCCTGCGGCTAAGCACCTTTCAATGTTTCTTTTTTCATTCATCGTATCATATAAAAGTAGTAAGTATGACTAT
TATTCAAACCTTTCTTGAAAAAAATAGTTAGGATGTTGCCATCTTGTAATGCAATCATAGAATTGGAAGTGCTGAATTTGCCATTTGTTTCAAACAAGCA
TTGTATATTGGTTTATCATTCAACTTGAGAGTTTGGTCAAATTGAACTCTCGAACTTTTCAACTCCTAAACTTTAAGGTCAGGCTCACATATGTCATTTT
CAAATTTTATTGGACGGAAAGTTGAGCTGGACGTTTCGAGCTCGGCTTTCTATCCAACTGAACTAATGGAAACACTGATTTCATCGCAACAAATTTTCAG
CAATAGATATTCAATCTCTTCTTGAATTCATCCTTCATCAGTATGGTGAATAAATTGTTCTTGGATTATGCAGAATGCATCAAGAGATCTTAACAGGAGG
CCAGATCAGGGATGCACAGTAGGGGGTGTTCCCAGCTGGGCGTGGAAGGTGACCGCCCCTCCTCCCCACGCAGCCATTGCCGCTGTTCTCGTCGGCGTCAT
CATCCGCTGCAGCATCATCAGGATTCTCCAATACCGCCACGACAGCTGCCCCACACCGCCCCATCGGCCTCCCTCCGGTTCGACCCGCCGCCGCCGCCGTC
CAGCCGCCACCGCTGAACGTTAAGAAGCCACGCTGTAAATTTGCCAGGAAGCCGACATTTTTTCCTCTCTCGGCGTTGCAACTTTTTAGGTTTTGCGCCG
GGGTGGTTTCTTGTAGTGGAGTGGATTCATAATTAACTGTATTTGCATGCTGCCCATGTGAAATGCTTCTCTCTCTTTTTACGCTCTCTGCACCGGATGT
TCTGGGAGTTCTTGTTGCCGGGAATTAACTAAATTCACCGTCTGAGATTGATCTATACATTGTTGTAGAGAATCGAATCATTGTTGAAATTAGTTACAGA
ATTATAGATATCATAAGGCACCCTCTACTTGTGGACAAAGTTCAGGAAGATTATTCCGGTTCTTTTCTTGGCTGTCAATTCGGCTCTTGTCCTTGGTGTG

GAGCCACTTGCCTACTGTCTTGTTCTGTTGCTGTTTGTTCTTGGCTTCTTGCACAGTTACAAATTTGTTGGCTCGAATGGATGGTGAGACATTTGGTGCA
A
>comp79438_c0_seq7
AGTAGGCTCATATTTGGCATCAGCAGAGGCCTCGCGGTGCCCCGTATTCTCATTTCCTCGCCGTATTTTCTCTCTCCAGCTCCAGACTCCAGAGTCCGGA
GGAAGCAGAGAGAGAAGCTCGACGGGAAGGTAGAGAGAGGTATAGTGCTCCTGATCCCTCTTTGAAATCCTCGATTGGTCAGTTCTGTTCCTGGATATAC
TGTTGCTGCAGATTGGATGCGTCTGCTTTCCTTTCGGGGAGTGGTGTCTAGCTCTGTTGGTTCTTGCCTTCATAGTTAGTACTATGCCTTAATCTTGCTC
CGCGATTGGTTTGATTCATGTTTTTCCTTTAATCTTGCTACCCCAGGATTTTCCCCTTTGGTTTTCTTGGTTTGCTTCTCGGAAATCCGTGATTTTAACA
GGATTTTTGGCTGGTGTTGCTCGATTTTTGTTCTTGACTGTGCTTCCTTGGGATGAGCTTTTGCCTAATTCGACATTACAAGATGTGTTCTTTCTAGAAG
TTAAATTGATCTTCTATGAGCCGGGCGCTGTATCGTGCATTACATGGGGGTGGCGTACTGGTACTTTGGGCCGTAGAGTTGATGGAAATCTGCAGTCCCT
TTTGTTGGATAAGGGGCTTTTTCCTTGGATAAGGAGGTTTGAAGCTAATTTTGTGCGAATTGTGGCGGTTTCTGGATATCCTGGTTGAATTAGTGGTGTG
ATAGATTGTTCTAGAGAATAGGAAGATATATGTTTTATTGTTAGAACATCAAGAGAGTGTTGAATCAGGTATGTCAATGTGTGAACTTATTGATGGGAGC
TAAATGCTTGCTGACGTATCTTCGCTATAATGACCTAATCAAACCTAACAAGCACTAAATACTGATCAATTCTCAGTTATACAAAGATTTTCCAGAGAGT
TCTTCAGAGCTCGAACTTCTGGGATGAAAATAGTTTGGCTCAAGATAGTCCACTGGAAGGAGTTTAGGATATCTGTTTGAGCTCTGCCTGCAATATATGG
AAATCTGAAGGTGATATGGTAGTAGAGTTGGGGACCCTGATCCCCGAGCCAAAAGAAGTTCGGTAGAATTTCTCAGGAATATTGTGGTAGTAAGAAGCAA
GTTAAAAGGAACCTAATTAGGAAAAAAGACATGCCTTGCTCGCTCATGAGAATATAAAACAGGTATGCCACCTCCAGTCAGTACTTGATTAATTTCATTG
AAAATGGAGGGCTGTAATAGATCCTTATCTATTAATGTGTTGTACCTAAACTTGGAAATCATCAGCTGAGGGCATTGTGCATGGTGCGTGGAGTACTAGC
CCTCGGGAGATGCTTCTTAATTAGGCAGCGAGAAGGATAGAGATCGGTTTGACATATGTTCCATTTGTAGCTGTACTGGATATTTCTGCATGTTCCCCTG
GAGCAGTTAGTTTCAGAATTATGATCTTTCTACTTGTGGCAGGATTGTTCTGTTTCTATTATCTGTTCATATTTATGCTAGTCTGTCTTTTATGTTGTAA
TATCTATTGTCTATTGCACTTCAAGTGACTATGTATAACCATATTCCAGTGATTCAGTGAATGATTATGAATTAAAGTAAGAGGACATTTACATCAATTA
TCCTTGTTCTTTCTCACGTCATTATCAATTCGGTTGATTAAATTCTGGCAACTTGCCATGATGGTTACTTTAGCTGATTAAATGCTACTCGGAGCTAAGG
ATACCTTCTCGTAGTGCTCCCGCTTTTGTGAGAAATGGAGGATCATCCAGTCCATCCCATGTCGAAGTCCAACAATGGCTTCTTGTCAGGAAATGGCTAT
CAGATGAAGCAATTTGGTCATAAAATCCACAATAGGGACTCGTCATCAGAGTCCGGTCGGTCTCACCAAGAAGCTTCCACAGCGAGTGAGAACAGTATAA
ATGAACAGCACACCTCAACACAATCTGACAATGATGATGGTCATGGGAAGCATAATGAGGACATGATGAAGTCAGCAGTGTCCTTGGGGGAACCAGGATC
AGCCTTTTTGGCCCCAAAACTAGATTACAGCCCATCTTTTGCTTGTGTTCCTTATACTGCTGATGCTTTTTATGGTGGGGTCTTGACAGGATATGCTCCA
CATGCCATTGTTCATCCCCAGCAAAATGATTCAGCAAACTCTCGGGTTCCGTTGCCTGTTGAACCTGTAGCAGAAGAGCCAATATATGTTAACGCAAAGC
AATACCGTGCTATCCTTAGGAGGAGGCAGATACGTGCTAAACAGGAGGCTGAGAATAAGCTGGTGAAAGGTCGGAAGCCATACCTCCATGAATCTCGACA
CCGCCACGCAATGAAGCGAGCCCGTGGATCAGGGGGGCGGTTCCTCAACTCAAAGCAGCTCCAGGAGCAGAACCAGCAGCAGCAGGCATCAGGTGGTTCA
AGCTGCACAAAGGCCATCGGCAATAACACAAGCTCCCAAAGTGGTCCCAGTCACACGCCTCTGACACTTGCTTCCTCTGACACTGCAAGCGCTTCGAGGG
CCAACCAGGAGCGCACCTGCTTCCCCTCGGCTGGCTTCCGTCCCACAATGAACTTCGGCGCGCGAGGTGGAGACGCAAAGCTAGCTGTGAATGGCATACA
GCAGCATGTTTCCTTCATCAGGTGAAGCAAGTGAAATGGGGCGCTGCCTGCGGTAACTCATCCTTGGCTTATGAAACTGTGAATGTGTAATGGACATGTA
ACTTCAGTCTCAGAATAACCAAACATATGGGAGTCCTCCTGAAGTTTCCTGTAGTTCAAAGAACTGATAGGTTATGGCCTTATAGGTTTGCTTCTGGAGA
CTGAAATCCGTGCTTGATATACTATCTTGTGCGAGTTCTGAGGTGAAAGACCTTGAAACTTGCTCTTGTGGTTCAGTACCTGTTGCTTTTGTGTCTGCAA
GGTTAGCACTGCAATTTGCAATGTCGGATTTCTGTTATGCCACTAATTTTT
>comp65473_c0_seq8
GCCAAGAAGAGCCGCCGCGGCCCGCGCTCCCGCAGCTCGCAGTACCGCGGCGTCACCTTCTACCGCCGCACCGGCCGCTGGGAGTCCCATATTTGGGATT
GCGGCAAGCAAGTGTACTTAGGTGGATTTGACACTGCTCATACTGCTGCAAGGGCGTACGATCGAGCTGCCATCAAGTTCCGTGGCTTCGACGCCGACAT
AAACTTCAATCTCAGTGACTATGAGGATGACATGAAGCAGATGAAGAGCCTGTCCAAGGAGGAGTTCGTGCACGTCCTGCGACGGCAGAGCACCGGGTTC
TCGCGTGGCAGCTCCAAGTACAGAGGCGTCACCCTGCACAAGTGTGGCCGGTGGGAGGCTCGCATGGGTCAGTTCCTTGGCAAGAAGTAAGAACACTTCA
CAACCTGAATTACCTAATACTAGACGTGTTATTTCTATACTGCAGATCCTTGTGATTTTTCCTATCTCAGTCTCATTCTAGAGTTATGAACCTAATACAT
GAACTCCCATGATTTAATAGTCTAGAAATAGTAGGTTAGATTGATGTGGCATAATGGTTGTTTGAACAAAAGAAATTAAAGAGAAAAAAGGCAGTGGATA
GGGAATTCCTCACCGGTGGGAACGCCATGCTTGGGTGCAGGTACATATATCTTGGGCTATTCGACAGCGAAGTAGAGGCTGCAAGGTTGCTGATCTTGGA
TTCTTCTATTGATGCAAAAAAAATTTTGCTTTACCTTCTCTGTTGATTTCCAAAGGGTTGAGTACCGACTCGATTCTCTTCTTTGTTGTTTTTCTTCTTT
CTGTTTCAAAATTCAGGGCTTATGATAAGGCCGCGATCAAATGCAATGGTAGAGAAGCCATGACGAACTTCGAGCCTAGCACGTATGATGGGGAGCTGCT
GACTGAAGTTGGTACTGAAGGTGCTGAAGTCGATCTGAACTTGAGCATATCTCAACCAGCTTCTCAAAGCCCGAAAAGGGACAAGAATTCCCTTGGTCTG
CAACTCCACTATGGATCGTTTGAAGGCTCGGAATTGAAAAGAACAAAGATTGATGCTCCTTCTGAACTGGCGGGCCGCCCTCATCGGTTCCCTCTTCTGA
CCGAGCATCCACCAATCTGGACTGCCCAGTCTCACCCCTTCTATTCAAATAATGAGGTTAAGTGATACTAAATTACTAAAATACATGCAATAACTTTCTC
AAATATATTGCTTTAGCTCCCTAAACCTCACAATTGCAAGCATGTTCTGAGCATCACATGGTTCGGATCGAGCTGTCCGGCACCAATTTTGAACGTAATC
TTGACCATCTGAGGAACTGATTGTGCAGCCTGAAGGCGTGCGTTCAAATGTTAGATTTGTGTTACTTTCGTTCATTTTCACTTCTTGTGCCGCTGTCAAT
TTTGGCTCTAGTCTTACATCACCGCTTCATTTCCTTGGATTCTATAGCCCGCCTGTTTCCTGTCGATTTCGGAGATATTAATGCGTAGCACAGGTTTCAT
ATGTAGGATCTTGAGTTTTCTGAGATGGCCCATTCTGGAAACAGTAATAGTCGCCTTAGGAATAGAGATGTGTTTGATTTTTTTCCTTCAAAAGGAACAT
CCTAGAGAGGTGACTATTCGACATTAGGTATGCTAATCCATCGGTCAACTATACCTGCCTGCGGCTAAGCACCTTTCAATGTTTCTTTTTTCATTCATCG
TATCATATAAAAGTAGTAAGTATGACTATTATTCAAACCTTTCTTGAAAAAAATAGTTAGGATGTTGCCATCTTGTAATGCAATCATAGAATTGGAAGTG
CTGAATTTGCCATTTGTTTCAAACAAGCATTGTATATTGGTTTATCATTCAACTTGAGAGTTTGGTCAAATTGAACTCTCGAACTTTTCAACTCCTAAAC
TTTAAGGTCAGGCTCACATATGTCATTTTCAAATTTTATTGGACGGAAAGTTGAGCTGGACGTTTCGAGCTCGGCTTTCTATCCAACTGAACTAATGGAA
ACACTGATTTCATCGCAACAAATTTTCAGCAATAGATATTCAATCTCTTCTTGAATTCATCCTTCATCAGTATGGTGAATAAATTGTTCTTGGATTATGC
AGAATGCATCAAGAGATCTTAACAGGAGGCCAGATCAGGGGGGTGTTCCCAGCTGGGCGTGGAAGGTGACCGCCCCTCCTCCCACGCAGCCATTGCCGCT
GTTCTCGTCGGCGTCATCATCCGCTGCAGCATCATCAGGATTCTCCAATACCGCCACGACAGCTGCCCACACCGCCCCATCGGCCTCCCTCCGGTTCGAC
CCGCCGCCGCCGCCGTCCAGCCGCCACCGCTGAACGTTAAGAAGCCACGCTGTAAATTTGCCAGGAAGCCGACATTTTTTCCTCTCTCGGCGTTGCAACT
TTTTAGGTTTTGCGCCGGGGTGGTTTCTTGTAGTGGAGTGGATTCATAATTAACTGTATTTGCATGCTGCCCATGTGAAATGCTTCTCTCTCTTTTTACG
CTCTCTGCACCGGATGTTCTGGGAGTTCTTGTTGCCGGGAATTAACTAAATTCACCGTCTGAGATTGATCTATACATTGTTGTAGAGAATCGAATCATTG
TTGAAATTAGTTACAGAATTATAGATATCATAAGGCACCCTCTACTTGTGGACAAAGTTCAGGAAGATTATTCCGGTTCTTTTCTTGGCTGTCAATTCGG
CTCTTGTCCTTGGTGTGGAGCCACTTGCCTACTGTCTTGTTCTGTTGCTGTTTGTTTTTGGCTTCTTGCACAGTTACAAATTTGTTGGCTCGAATGGATG
GTGAGACATT
>comp82045_c1_seq1
AAGGGGCGGCTGAGCAAGGAGGAGGGGAGGTGAGGTGCCAGGTCGAGGGGTGCGGGATAGACCTCGGCGCCGCCAAGGAGTACCACCGGAAGCACCGCGT
CTGCGAGGCCCACACCAAGTGTCCCCGCGTCATCATCGCCGGCCAGGAGCGCCGCTTCTGCCAGCAGTGCAGCCGGTTCCATGCACTCTCGGAGTTTGAT
CAGAAGAAGAGGAGTTGCAGGAGGCGTCTGTCCGATCACAATGCCCGTCGGCGGAAGCCTCAGCCAGATGCCTTCGCCTTTGCCTCGGCAAGGCTGCCAC
CGTCATTATTTGATGATAGGCGACAAATAAGTTTTGTCTGGAATAAAGCTCCTCTTAGCCATGTAAGACCTTTCACTTCTTCTACATGGGACAGCTCATC
TGACTTCAAGCTTCCACATGCGAAGGAAATAAGAGAGCTATCAACAGGAGTTGGGACTATTACTGGACAATTTCATCTGGATAAATCTCATCTGTCCAAT
GCCATACCAACACTTAGCCATGGCAAAGATGAGCCGTTACCAATGAAAGGTCCGGACACATCTTTAACTGCTTCAAAATTCGATGGAGCACCGGATCTTC
AGCGTGCTCTCTCTCTTCTGTCAGCTGGCTCTTGTGGATTGCCTGATCCTGTACAGCAAGCATCTTGTCTTATCCAGTTCTCTGGTGCCAGCGAGAACAG
CAGTGACCTTCATTCATCACATGGTGGGAACTCTGCTCCAGTCTCATGCGCTGATGAACAGCACATAGCACCTCAGTCTCAGCTAGTCCGTTTTACCATG
GATGCCAACAGCAGTGGCTACGAGTCCACTTTCTTCGGTGTAAACCAGATAAATTAAGGTGTGAAAGGCACACGCCTCTTGATCATTTGGATAAAGAGCC
ATGACGATGGCCTGGACTTGTGCAAGAATAGCTTCTGTCAACTACCAGAGTTCAAAACCTCTCTGTTAATGTTATCTACATATTTATTCGCCATCCCTGA
ACTAAGTTGAACTGAGCGTAGGCTTGTTTGTGTTGAGTTCCAGCCTGTGATTGACCCATTTTTTCTGTTCATTTCCAGCCTTACTGTGCCTACTTTGAAA
CCTTGAAGTGTGATATTGCTATTTTACAATTGTACAACCTACTGCTGAATCTCTTTGAAACTGTCCTTGATGAATCTTGACATGTATTATTTGTGTGTAT
AGTTGGATCTCAACTGCCATGTTGTGTAGCGAGA
>comp63680_c0_seq2

AGTATCTCGGCGTGGGACAGAGGCCGGTGGTGGTCGCGAAGAAGACGCGGCGCGGGCCGAGGTCCCGGAGCTCGCAGTACAGGGGCGTCACCTTCTACAG
GAGGACGGGCCGGTGGGAGTCGCACATCTGGGATTGTGGGAAGCAAGTCTACTTAGGTGGTTTCGACACTGCTCACGCGGCTGCGAGGGCTTATGATCGT
GCAGCGATCAAGTTCCGAGGGCTCGACGCGGACATCAACTTCACTTTGAGCGACTATGAGGATGACTTGAAGCAGATGAGCAATTGGACAAAGGAAGAGT
TCGTGCACATACTCCGCCGCCAGAGCACGGGGTTCGCGAGGGGGAGCTCCAAGTACCGCGGCGTGACGCTGCACAAGTGCGGCCGCTGGGAGGCGAGGAT
GGGTCAACTTCTTGGCAAGAAGTAAGCCAACCTCCTGGCTTTGACCTTTCTACTCTGTTAGCCTAGTTAAGTGTTTTTACCCTGGTTATATGAACAAAA
TATGTCGAAATGATGTTCAATTAACTTGTCATTTCACGTTGGGTTCGATCAGGGCTATATCCCTTATGTTCATGTTAAGTAGTTTCAGGTGATTGTAGAG
AATACAACAAGGTGATCTTGAAGTATCACTGATTATAATCCATGCTACTAGTAGTTTGAGCGTCTCTGTTGCCGGTGAAATCTCCACCCCTCTTGTGACT
TTACCCCTCTACCGTTTAATCCGGGAATCCGGAATGGACCTACAGATCGAGATATGTGCCTTGATGAACAGTGTTTCGGTGAACAGCAAAATCGGCACCC
GTGCACAGTGTCCTGATGGACGGTACATCTATCTTGGACTCTTTGACAGCGAAGTTGAAGCTGCAAGAGCATATGACAGGGCAGCCCTTCGCTTCAATGG
GGGGGAAGCTGTTACTAATTTGGGCCTAGCTCCTACAATGGAGGAGATGCTCTACCCGACACCGAAAATGAGGGTATCACAACCAGCCTCGCCATTTAT
TTCTTTTCATATAAGCAAATGTATCTCAGGTGCACCTTATAATCATCGCTGTGTTCTTAGCAACTGTTGATGGTGACGCGGTTGATTTGGATTTGCGGAT
TTCACAACCTAATGTGCACGACACTAAAAGGGACAATACCTTAGCTGGAGGCCAGCTAACATGCGACTCCCTTGAATCTTCAAACACGATGGCCTCTCAG
CCAATGAGCTCATCGTACCCGTGGCCTGTGTATCACCAAAGCACGACAGCAGTACCACCTCACCATCAACGCTTGTACTCATCTGCTTGTCCTGGCTTCT
TTTCGAACCTCCAGGAAAGGCCAATGGAGCGAAGGCCTGAGTTGGGTACCCAGTCATTCCCCACCTGGGGATGGCAAATGCAGGGCTCCCCTCACATGCC
GTTGCATCACTCTGCAGCATCATCAGGATTCTCTACCGCCGTCGGCGCAAACGCCGGCGTCTCGTTGCCTTCTCACCCGCCGGTGCCGTTCCCGGACCAC
CAGTTCTACTTCCCACCGACGGCATGAGCTTGTTGGTCTGGTTCAACTCTTGGGACGGCAGCTGATCTTATGTGTGAACATTTCGCAGCTTGCCGGTGAC
CGTTGTTAATTAATCGGGGAGAGAGAGCCAGAAGACCCAGTATGGCTATCTCTCTGTCGCCCGTGCCTTGTTATGATCTTAGTTATTGCTACTACACCGT
GGATTCATCATGGTTTATGAGATTCTTTACTCAAGTTTCAGCTTAAATGTCCAAATACATGAATGCATCATACACAGGATCCGTCATTTTCTAAAAAAAT
ACAGGATCTGTGTGAAGTGTAAACATAAGG
>comp73737_c0_seq1
AAGCTTTACTTGATTAGTACAAGAAACACCTGTTACTGACTTTGCTTTTGCTACATCTGGTAAGGACATGATGATCTACTGCACCGCGATTTTCTTTGGT
AATTTAACGTTGTACAATAATGCCTTTATTTTTTTTGTATCAGGTGACTCCTTCGGGAAAATGGTTAATGATCATACGAGGTCAAATTTGGTTTTTGATA
ATAAGCAATCTCTATTTGCAAGTCATAACATTGACTACGGCCAGCCAATAGCTTGTATATCATACCCATACAATGATTCGGGCTCAGGAGGTGTTTGGGC
AGCCTTTGGGTCACGCACTAGCGCTGCAGCTGTGTTCCATCCCCAAATTGCTTGTGGGGGCACATCTGCGAGAGTTCCCTTACCTCTGGAATTAGCAGAT
GATGAGCCCATATATGTCAATCCCAAACAATATCATGGTATACTTCGCAGAAGACAGCTACGTGCTAAGTTAGAGGCTCAGAACAAGCTAGTCAAAAACC
GAAAGCCTTACCTTCATGAGTCTCGGCATCTTCATGCAATGAAGAGGGCAAGAGGTTCTGGCGGACGTTTCCTTAATACTAAACAGCTCCAGGAGCAGCA
GCAGCTGAAGTCTCGCAATGCCTCCACCAGGTCCACCACAAATGGCGCAAATTCCTCAGGTTCAACACATCTACGGCTTGGTGGTGGTGCAGATGGAGAT
CAAACCATGTCGGGGACGAAAACAATGGCCTCACAAAACAATAGCAAAAAGGCTGTTTCTTCTTCTGCAGCTCTTGCTTTCACCGTGACTCCTCATGGTGC
GCAAAGATGACACCTTCTTCCAGCACCTCAGCCACAATGTCAGCTTCTCCAGCCATTTTGGCCAGGCAAGCGCCCAAACCGGCGTTGGAGGCATCCATAA
TGAGACCCAGCATAGGGTTTCCGTGATACAATGACGGTTTGCGAAGCTTATCTGGTGATCCAGGCTTCTAGGTGTCCTCGTGTCCGGTGTAGTCTTGGTC
GCTCAGGCAATTCATCCTTGGCTTAGTTTTTGGTGTGTTAGAACCTACAAATGTTTCATACTCTGTTGTGGTTTGCAGAGCCCATAAATCAGGGCTCTCC
TAAAAAAAATCAGGGCTTGATGGCGACACTACTATCCAAGTATTGTTGTAATGGTGTGTGTTAGAAACTCGCAAAAAACCCTTCTTTGTGCTCGTATTAG
TACATTATGCTGCACAATTAGCTGCTATTCTGTTCGTACTACAGTCTGGTATGCAGTGGTTAGTGGTCCAGTTTA
>comp79373_c0_seq23
GAAACCTGGTAAACCCTGGCCACGGACGCGCATGCCATCCAAGATGACGTAGTCCACACCGAATCTTCCACGTCCGCACACGCGAGCACAACGCCACCGC
AGGCCAAGTGCCGTCTCCACCACAACTCGCTGATCCCTTCCTCCTCGCCGGCTCTCTCTCCATCCCTCGTCCTTCCCCATCTGTCCCTCCCTTGGCTTCT
TCCTCCAAGCTCCAACCAATCCCCACGCGCGCGCGCGCTATAGCCTACAAATAAATACTCGCCACTCTTCCCTTCCTCCCGCCTATCGTCTTGCCAAGCG
CTAGAGCAGCTGCGCCATTGCACGCTTCCGGCCCGTCGTCCTGCACATCTGTCCTCCCCGTACTGCCTTGTACAAACTCCGTATGTAAACTACTGGGTTG
TCGCCAGTGTTCGTGTTTGCTGATGCTCATCTAATTATCGTCGAGGAGATCCAAGGAATTGTCCGCGGAGCGCCGGGGAACAAAGGCTGCGCGCGGTACT
ATATCAATTACAAACTGATGAAGAAAATGTTAAAGCAATATGTTCAACAGACCCAACTTGGTGGGAAAGATCATGAACAAGTTCTTAAAGAGTTCTCAAG
GATTCTTGATGACCAGATTGAAAGGATTGTGCTTTTTCTGCTACAACAACAAGGCCACCTTGCCAGCAGGATTGAGGAATTGGGAGAACAACGCACTGCT
CTTATGGAACATTATGATATATCACAAGTTTCCCAGCTACGTGATGCGTATAGAGAAGTTGGGAGAGATCTTATTAAGCTTCTGCGCTTTGTTGACATGA
ATGCTACTGGTATACGCAAGATACTAAAGAAATTTGATAAGCGTTTTGGCTATAAGTTTACATATTATTATGTCACCACTCGTGCAAATCATCCCTATTC
TCAGCTTCAACAAGTATTTAAGCAAGTGGGAATTGTAGCTGTTGTAGGTGCATTATCGCGCAATCTTGAATATCTGCAACATCATCAAGGAAGCTTTGTA
TCCATCTATGATCATCCATCAGTTACCTTGAAGGACCCTATAATAGACGAAGTAAACCATGCGGTACAGAAATTTACGCATGCCACCAATTTTATGAAAT
TCTTGGGACAACACGCGCTTATTGTTCCAGAGGATGCACGAAGCGGGTCTGAGGATCTTGTTGATGATCAGAGCTACCATTTCATGTCTCTGCTGCTTAA
CCTAGCGAACACTTTTCTTTACATGGTGAACACATATATCATTGTGCCAACTGCAGATGACTATTCAGTAAGCCTTGGGGCTGCTGCGACTGTCTGTGGT
GTAATTATCGGATCAATGGCAGTCACTCAAGTGTTCTCCTCGGTTTATTTCAGTGCATGGTCAAATAAGTCATACTTCAGACCACTTGTATTCAGTAGCA
TTATGCTATTTTTTGGGAACCTGCTATACGCATTGGCATATGACCTGAATTCATTAATAGTTCTCCTGATTGGACGACTACTATGCGGGTTGGGCTCTGC
AAGAGCAGTGAACCGTCGCTATATTAGTGATTGTGTGCCTCTCAAAATCAGGCTACAAGCTTCTGCAGGATTTGTCAGTGCTAGTGCTCTTGGCATGGCA
TGTGGCCCTGCTCTTGCTGGTTTCCTCCAGACAAAATTTAAGATATACTCGCTCACTTTTAATCAGAGCACATTGCCCGGATGGGTCATGTCCATTGCTT
GGCTTCTTTACTTGTTGTGGCTGTGGTTTACATTCAAAGAGCCAGAACACTTCGCTAAAACTGTCATCAATGCACAGCCGTCTGAATCAGGTCACCAAGG
AAGTGCTAATTTGGAGGAAGGTCTAGCTCAACCATTGCTTCTGGGTACAGAACAAAGACAGGGCGAGAATGCGGATGACAATGATGATAATGAAGTAGAC
TCTGAAAACTCTCATGAACCAGCAACATCAATTACTTCAGCATACAGATTGCTGACACCATCTGTGAAGGTTTCCTATGCTTCCCTTATCGAGTACTCTT
TTGTCTTGTGTTCCTCTCAAGCATGCCTAAAACAAAGTTCGCAGTCTAAATAGGGTTCTTTTGTAAAGAAAGTAAAGTAAATGAACCGTGTAACATTAGG
AAATGTATCTTAGTGTTAGTGATAATGATACTAGAAGGTACATCTGCAAGACACAAGCATGCACTGTACTTACACGCTATATCAAGTGAATTGAAACCAG
ATGAAAAACCTTCTTTTCATCTTTGGACCTTTGCATTTTCAATTGCCCTTGACCTCTTTCAGTGTATTTATAGGAGCTACCATCTTCTGTATCTTCCATT
TCATATGTTAATAAGAGTGGCTATTTCTCAGGTCCAGCTATTGATATACTTTATGCTCAAGTATGCTATGGAAATTTTACTATCTGAATCAAGCGTTATC
ACAACATACTATTTAGCTGGTCTACGAGTGCTGTGGCTATCTTTTTGGCAATTCTTGGATTAACTGTCCTTCCAGTAAATGCCATTGTTGGAAGCTACA
TTACAAATTTGTTTGAGGACAGGCAAATTCTGTTGGCATCTGAAGTCATAGTTCTCATTGGTATCATCATGAGCTTTCGTTTCACCCCTCACTACTCCAT
TCCACAATACGTCACTTCAGCTCTCATCACATTTGTGTTTGCTGAGGTACTCGAAGGAGTGAATCTGTCCTTGCTCTCACGAGTAATGTCGTCTAGGCTT
TCGCGAGGGACCTACAACGGTGGACTCCTCTCGACAGAGGCCGGGACATTGGCCCGTGTAATTGCAGACGCGACTATTACTGCAGCAGGTTATCTAGGCC
CGGACCTCCTCCTCAACGTCACCCTGCTGCCACCTCTAGTGATCTGCATAGTGTCCATCGTCGCAACGTTCTGCACTTACAACAACCTTTGCTGATAAAG
CAGCCCCTCTGTGTCCAGCCAGTTAAAATGACTCCTGCCATATTGGGCCGTACCGTACTACTTGAAGTAGCATCAGCGTTGTAAATCTGGTAAATCTACA
AATACCATGGACGGTGCCGGTGTGGCGGTGTGTAGTGTATTGTTATGCATTCAACAACTTTCTTGTGTCTAGTAGAGTTAAATATTCGTCAAATTGAAAA
TTCAAATGCATTAACCAAGGAACATCACTGTGTTGGAAAGACAAGATGATTTATTGCAA
>comp83121_c0_seq51
GGAGGCAGCAAGCTTGAGACAACAGCTGCACAGCTTGCAAGAAAGTCACCGACAGTTGATGGGACAAGATCTTTCAGGATTGGGTGTCAAGGAGCTCCAA
AATCTGGAAAATCAGCTAGAAATGAGCATACGTTGCATCCGGACAAAAAAGGACCAAGTCTTGATTGATGAAATTCACGAACTGAATCGAAAGGGAAGTC
TCATGCAACAAGACAAGATGGAACTATACAAAAAGGTCAACCTAATCCGTCAGGAAAACATTGATTTATACAAGAAGGTATTTGATAAATCGTGAATGAA
AATTCACAGGATATACACCCCATCCATTATTGACTGAGGTTTATTTCTCTACTCAGCTCTACGAGAAAGAGGTGACAAGTGAAGTCAACCGAGATTCATC
AACTCTAAACTTTGCAGTTGTCGAGAACGTCAACATTCCTGTTCATCTTGAACTTAACAGTCCACCGCAAGAAAATGACGTTGAGCAAACTGCACCCCCT
AAACTAGGGTTACAACTAAATCCATGAAGGCATGCAGCATGGCACTTGCTTATGTTCTTTATTCTCAACAGCTACGATTCAAAGCAGCCTGAAAAACTAT
TGGTTGTAAAGAGCCTAAATACAGCAAGGCAGACGCAGTATGCATGCCATGGGCGACCGATAATGGTCGTGTAAATCTATTTCCAATAAATTGTATTTTA
TTGAAGGGATGCACCAAACGATTGTCTTACAACTATTTGACACATTCTGCAAGTTTGTTGACCAACGTGAGTACATGAT
>Locus_18892_Transcript_24/56_Confidence_0.279_Length_2185
ATCTGGGATTGTGGGAAGCAAGTCTACTTAGGTGAGTAACTGAGTTGAGTGAGTTCTATACGTTCCAGCTCGAGCTTCCATTAACTGCTGATCAGTTTCC
GTCCGTTTGATCTGGTTTACGATGTGCTGATGCTGTTCTATTCAGGTGGTTTCGACACTGCTCACGCGGCTGCGAGGGCTTATGATCGTGCAGCGATCAA

GTTCCGAGGGCTCGACGCGGACATCAACTTCACTTTGAGCGACTATGAGGATGACTTGAAGCAGATGAGCAATTGGACAAAGGAAGAGTTCGTGCACATA
CTCCGCCGCCAGAGCACGGGGTTCGCGAGGGGGAGCTCCAAGTACCGCGGCGTGACGCTGCACAAGTGCGGCCGCTGGGAGGCGAGGATGGGTCAACTTC
TTGGCAAGAAGTACATCTATCTTGGACTCTTTGACAGCGAAGTTGAAGCTGCAAGAGCATATGACAGGGCAGCCCCTTCGCTTCAATGGGGGGGAAGCTGT
TACTAATTTTGGGCCTAGCTCCTACAATGGAGGAGATGCTCTACCCGACACCGAAAATGAGGGTATCACAACCAGCCTCGCCATTTATTTCTTTTCATAT
AAGCAAATGTATCTCAGGTGCACCTTATAATCATCGCTGTGTTCTTAGCAACTGTTGATGGTGACGCGGTTGATTTGGATTTGCGGATTTCACAACCTAA
TGTGCACGACACTAAAAGGGACAATACCTTAGCTGGAGGCCAGCTAACATGCGACTCCCTTGAATCTTCAAACACGATGGCCTCTCAGCCAATGAGCTCA
TCGTACCCGTGGCCTGTGTATCACCAAAGCACGACAGCAGTACCACCTCACCATCAACGCTTGTACTCATCTGCTTGTCCTGGCTTCTTTTCGAACCTCC
AGGTATTACCAAGCTAAAGCCTATTTTTTATGTGCAAATTGCTACTCCTACGTGATCAATTAGTTCAAACTGTTATTCGAATCCAGGATTGTTTACTGAA
TATGTCCTGTCTTCAGAAAAAAATAACACAAAAAGGAACATAATATGTCCTGTCTAATTGTGGACTGATACTAGCCAATTAAACAGTTTCCCATATCCATT
TCCTGTGTTTATTGTTTTGATTTGCTGGCATGGTTAAACTATAGTAGCTTGATTCGATAAAAACACTAAGCAGGCTTTGATATTTTGAATGAAATGTCGT
TCTTGGAAATACAGCCTTTTCCTTGGTATATCAAGGACAGTTGTCCATCTGCACTCTGCACCCTCAGCTTGTGTAACTGTTTGCCTTGTGAACTGATTGA
TGTGTCAATCAGGCAGTTCTGCCAGTTTGCATCTTTTGAAATAGTGCAGTCTTCCAGTCTAACAGCTCCATGACATAACTTGGGATGCATGCTGCAGCTT
TCTTACTGAGGATGCTCTAGATTTACAGAAATTCTCGCAGTTAGCAGAGGGCTATCAAATCTGATTTGCTTAGCTTCCTGCAGACAATAAGATAAGAACT
CTAGAATCCTGAAGAAATCTTACTTGTACATGTTTACCAAAGTATCCTTACTCCTTAGTTGAGCCAATTTTGGTGAAGAACCGATAGCTCATTACTCTGT
TTCCTGGGAATGATGTGGGCCTAAACCATTCTGTGGTCAAAGTTTAACAAAGCCCATGCTTGTGCTGTTGCAACTGCAGGAAAGGCCAATGGAGCGAAGG
CCTGAGTTGGGTACCCAGTCATTCCCCACCTGGGGATGGCAAATGCAGGGCTCCCCTCACATGCCGTTGCATCACTCTGCAGCATCATCAGGATTCTCTA
CCGCCGTCGGCGCAAACGCCGGCGTCTCGTTGCCTTCTCACCCGCCGGTGCCGTTCCCGGACCACCAGTTCTACTTCCCACCGACGGCATGAGCTTGTTG
GTCTGGTTCAACTCTTGGGACGGCAGCTGATCTTATGTGTGAACATTTCGCAGCTTGCCGGTGACCGTTGTTAATTAATCGGGGAGAGAGAGCCAGAAGA
CCCAGTATGGCTATCTCTCTGTGGCCCGTGCCTTGTTATGATCTTTAGTTATTGCTACTACACCGTGGATTCATCATGGTTTATGAGATTCTTTACTCAAG
TTTCAGCTTAAATGTCCAAATACATGAATGCATCATACACAGGATCCGTCATTTTCTAAAAAAATACAGGATCTGTGTGAAGTGT
>Locus_1016_Transcript_1002/2070_Confidence_0.001_Length_4852
GTTTCAAGCTCAAGCTCGGCGCCGGCGTCTCAGTGAACCACTCTGCTGGGGGTGTACTTCAATTTGTTGTTGCCAGTGTTATTAGCAGTCACTGCAGCTC
AAGTAGCAGGCTGGAAGCTTTTAATCTGACATTTGTTCTTTCCTTTTCTGATAATTACTGCAAGTGGAGGCGGCAAGCTAGAGATAACAACTGCATAACT
TGCAAGAAAATCGTAAGTTTCTCAATTTTTCTGTTCTATACATGAGAAAACGGTGTTGATTTGTGTGCATGCGTGCGTTTGTCAATGATCTGAATGATTT
GCACATGATTTGTGTTAACATGCTGTGAATTCAGGGTATGGCAAATTTGCCAAATATCTTTTCAGGATGCTTAAATTCACTTTGTTTAGAACTGTTTTGA
GAACCGTATGGATTCAATAGACACCCAATTACTTGGTTATAATTGAGTTATTTACTTTACGAAAGGTCGATGTGGTCATAGCATATTAAAGTTCCTTTTA
ATTGACCAATGACCGTATGGTTACTCTCATTCCTTCAATTCATTGCTCTCCCAGTCACAAATAAGTGATGTTTGGGACATTGACAAGGCCTCCAAAACTC
AAATGACCACCTAATTCTGCCATAGTATATTTTTAGAACCTAATAATTTGTATATCATTTCGATGTGTATACATCATTTAAGTTTAGAACCCAATAATTT
GTATATCATTTTGATGTGTATATATCATTTAAGTTTAGAACCTAATAATTTGTATATCATTTTTAGAACCCAAACCTCAAAGACATATCGAGAGGAATCC
ATGTATATCATTTTGATGTGTTCAATGTAAATATTTGTAAAGATACCAGTAGCGAAAGTTTGACTACACACATTCCAAATGTATAGAGGTATAATTGGAG
GTTCAGCCGTTCAGAGACCCAGGTCTTGGGGTCCAGGATCGTTTTGTGCTATAATCGTTTACCCCTTGAATTTCTGTGATTAAGGTGATAATCTTAATTT
ACTGTGACATAAGAATAGGCCAAATATTTTCCTGTACAAACTTAAGCTAGTGTATCTGTCGTGTGTCTTGTCATAGTACAATCCGTTATTTCTGGTTATT
TTCGGTGCGTCAACGTCTTACTGTTGTTATTCCTTTGAACACTACAAAATTTTAAGTGAGCAACTAGCATCCGTATATTTACATATGTAGCTGAAGAAAG
GTTGATCCCCATTCTGATACTCTGGAATGCCTGCACGGATAAACCAGAATGCAATTTGTGCTCTTCTGCTATGCATACAATTCTCAAAATAGAAAAAAAT
AGCACCTGAATTTTTTCCACACACCATATCTTTTTGTCAATATAAGATTTGTAGCTATCATGTACTCACGTTGGTCAACAAACTTGCAGAATGTGTCAAA
TAGTTATAATACAAATCATTTGCTGTATTCTTCAATAAAATACATTTTATTTGAAATAGATTTACACTACCATTATCAATCACTCATGGCATGCATACCG
CGTCAGTCTTGCTATGTGTTTAAGCTCTTTACAACCAATAGTGTTTCTGGTTGCTTTGAATCGTACCGTTGAGAATAATGAACATAAGCGGTGCCATGCT
GCATGCCTTCATGGATTTAGTTGTAATCTGCAAGTTTTCAGTTAGTAGTAAACAATATGCCACTAAAGAATTAAGAACAGAATCAATAGTTCCTTTGCAG
AAAAAAGCAGGGTTACCCCAGTTTAGGAGGTGCAGTTTGCTCAACATCATTTTCTTGCGGTGGAGTGTTAAGTTCAAGATGAACAGGAATGTTGACGTTC
TCGACAACTGCAAAGTTTAGAGTTGATGAATCTCGGTTGACTTCACTTGTCACCTCTTTCTCGTAGAGCTGAGTAGAGAAATAAACCTCAGTCAATAATG
GATGGGGTGTATATCCTGTGAATTTTCATTCACGATTTATCAAATACCTTCTTGTATAAATCAATGTTTTCCTGACGGATTAGGTTGACCTTTTTGTATA
GTTCCATCTTGTCTTGTTGCATGAGACTTCCCTGAAAATGAGCCAGTAGAGTCAGCTTCTTGAAATCCACTCAGAGACCTCTCATTGCAGAGTAATAACA
GAATATTCCAGAACCTTTCGATTCAGTTCGTGAATTTCATCAATCAAGAGTTGGTCCTGAAAGTAAAAAACGTCATCATCAGTTGTATTCTTGGATGAGG
CAATCTGGTAACGAAAAACAGAAATATGGCTAGCATCCTTACCTTTTTTGTCCGGATGCAACGTAGGCTCATTTCTAGCTGATTTTCTAGATTTTGGAGT
TCCTTGACGCTCAATCCAGAAAGATCTTGTCCCATCAACTGACTGCAGTCAATCAAACTGTATTCAGTTTACCTGATAACCTGATATTCCTCTGCCCATG
TGTATTAATGTTGTGTTGGTTGGGAGTGAAGTGCGGACAAATCAGAGACTCAAGAAACTACCGGGCAAAACAGATGATAATATCATATTTGTTTTCCATT
AGTATGTACTCTTCTTCTTTGGAAGTTAATTGGAATGCATAAAAGCTAAATAGGTACATTTTTTGACTTATAAATGGCTTGGCATAGGAAATTCATAAAC
TGTTACCTCCTTTTACATTGATATTGTTTATGAAAACTGATGTCCTCATAAGCAAAAGATTGTCCTATGGAGTCAGTAAATCAGTATGTCTCCTTAAACT
TGGGCATTGTATGCTATCTTGTATACTAAATTCATTCTTATCCATTGTTTTACAATTTTATACACTCTGCACAAATTAGTTGGTGGAGGTATACTTGTCA
CTTTACAGCCCTTAATTTGCATCACCAAGAGTTATGGATGATCTGTAACATGGTGTAGTTGATTCATAATTGTCTCGTCAGAACCTAGTTTTATACCTTT
ATAAATAAGACAACCAGGAATTGAGTATTGCTACATGGTGAATATTGTTTCTTCTTTTGGTTCTATTCTAATGTTGTTTTGAATTTTTGTATATTCTTGA
AACAAACAGTAGGAAGGATTTCCTTCCTCCAGAGTTATCTAACTCGGAAGTTAGGCACTTGTGCTCATGCTGACAAATCCTATAGTTTGATCCTTTTACA
TTAGGCAAATTTTCTGACTTGTACATTTGTAAAATTTGCAGGGTTTTCTAGCCATACCGATGACTTTCTTGCAGGTTGTGCAGTTGTTGTCTCAAGCTTG
CAGCCTCCCTTTGCCAGAACTATTGAATATGAAAGCACAGTTAAGTAATGCCACATCCAGGATTGCCAAAATACAGCATTGGTATAAACATGTTGCATAG
AAGCATGTCTGAGCTGTCAGCTAAACAAATTGTACCTTATGAAATAACTGAATTAAATAAGATGACATGCCACTATTTCAATCAAGAGATTTACAGTGCG
CTACAAAGTAACTCTCTTTTTGAGAGGGGTGAGGGGGTGGCAACGATTATAATAGCAAATACAAAACCTTTTAAACTTGAAGGTTGAGATTCTATATTAT
TCAAATACTTGAATTAAAGAACTTTATTATAGTGAACTATCAAGCTGGACACATATTGGCACGCTACACTTGCTGTATTGTCTCCTATGCTGTTGTTCAC
ACTTTTCACACACAATTGCTCTGACAACTGAATTGAGCTAAATTAGCCTCGCAATACCTTGTGCTCGGGTCTAGGGACAAAACGGAACATGTCAGGATGG
TGATGATCTTGCTGGAACATGTTTAAGCTGAAATTAGATGACTGTAGGTTTTCTCTTCGTTTTCTAGAATGTTAAAGGTCATCGTCTAAAATGTTTCACT
ATAAACCGGAAAGGATTCATGCGGCACTCATTTTTCTGGAGAATGGACTAAAACATCTATTGACTGCTGATCATTTTCAGAAGATGAGAACTTGAAACAG
GTACCCTTTTTCTACTCTCTTTCGTGTTTCTTTATCAACTGTCTTTTTGTTAGTTTCATCTTGAATATATGTATTTGGGTTGAAAAGGATCACATGGTTT
GGCTTCCTCCAAAAAGAGGTTTGAGTATACGAGGCACTGCCTGGATGGATGGTTAGAAGGATTTCAAATACCGTGCTTACTGTTCATCGAGTAACAATCT
GTATTATCTGTGCACATTTGCAGTCTATTTTCCCAAACTATGAACTGTACCGTACTTAGGTTCTGTGCATATGCAGCTCGACATTTTGGGTGTTTGGTGG
TTGAATGAACCTTCACCCTTCAATTTATCTGTAGTTATTTGTGATTTGAAGCATCTTCTTTATATCTGTTCCACACTGCTGCCAATTTGATTATTGATAT
AATAAATTAAGCTTTCAGATCGTATAGTAGAAATACACTATCTTTTAATGCAATCCTGGCTTTGCTTGGTATTTTATCTTCTCGAAATAACTTGTGTTCA
TTGGGATCTATGTCAGTGCTGTGTCAGCATCAGCTTCGACTTACTGGCAAGCTTCAGGGCAGGCTAAGCAAGGCCCCTAAGATTTCATCCGGACTACAGC
TAAAGTGAAAAAGGGTTTATTCATGATAATTTCAATCTAAGACCTGTTTAGTTTCTATTAAGGATGCTTAAGTATACCAACATTTTTTTTATCTTTCTTG
CCAAATCTGCAAGAAAGCTTGGTCAAGAGAATCTTGCCACAACTCCCTTTGAGATGCCAAATCTGCGGTGAAGCGGAAAATTATAAAAAACTTTTGGCAT
TGCTATCCTGTGTCTAAGAAACAAACATCATCCTAATTTGGTGGAATTCGCCTGATGTGTTGTAGCAAACTAAGGGGAACTGTCTGTCAATACACCTCAA
GTGCCTATATGTAGACCGGACAATATTTCAACGCCTCTTAATTCAGAGCAGT
>Locus_18892_Transcript_35/56_Confidence_0.294_Length_1637
TGGTGGCTGCGCCGTCTCCTGCGGCTCTATCTCCCGCCGCCGCCGGGAAGAAGAGCCGGCGGAGGCCGAGGTCCCGGAGCTCGCAGTACAGGGGCGTCAC
CTTCTACAGGAGGACGGGCCGGTGGGAGTCGCACATCTGGGATTGTGGGAAGCAAGTCTACTTAGGTGGTTTCGACACTGCTCACGCGGCTGCGAGGGCT
TATGATCGTGCAGCGATCAAGTTCCGAGGGCTCGACGCGGACATCAACTTCACTTTGAGCGACTATGAGGATGACTTGAAGCAGATGAGCAATTGGACAA
AGGAAGAGTTCGTGCACATACTCCGCCGCCAGAGCACGGGGTTCGCGAGGGGGAGCTCCAAGTACCGCGGCGTGACGCTGCACAAGTGCGGCCGCTGGGA
GGCGAGGATGGGTCAACTTCTTGGCAAGAAGTACATCTATCTTGGACTCTTTGACAGCGAAGTTGAAGCTGCAAGAGCATATGACAGGGCAGCCCCTTCGC
TTCAATGGGGGGGAAGCTGTTACTAATTTTGGGCCTAGCTCCTACAATGGAGGAGATGCTCTACCCGACACCGAAAATGAGGCAATTGTTGATGGCGATG
CGGTTGATTTGGATTTGCGGATTTCACAACCTAATGTGCACGACACTAAAAGGGACAATACCTTAGCTGGAGGCCAGCTAACATGCGACTCCCTTGAATC

TTCAAACACGATGGCCTCTCAGGTAAACTGACTTCAAGACTACACAGCAGGGAAAAATGCTCGGTTTCTCTATGACTTTCTACTACTTGCAGTAACTAAA
AAAGTTTCCCAGAAAAATAAAATCTTTAAGATGGAAGCAACTAGGTTGAATTGTCTTGTATCTTTGCGTGGAATCCAACCTTTGATAGTCCCTACGCTAT
AAAGATTCATAGGAGTATTTGATTGGTGTGTCGTCGGTTGAGATATTAACTAATTGGTCCAGGTCCTATAACCTTTATTGAACTTACGATATTTGCACCT
ATCTGTTGCTCCTGTAGCCAATGAGCTCATCGTACCCGTGGCCTGTGTATCACCAAAGCACGACAGCAGTACCACCTCACCATCAACGCTTGTACTCATC
TGCTTGTCCTGGCTTCTTTTCGAACCTCCAGGAAAGGCCAATGGAGCGAAGGCCTGAGTTGGGTACCCAGTCATTCCCCACCTGGGGATGGCAAATGCAG
GGCTCCCCTCACATGCCGTTGCATCACTCTGCAGCATCATCAGGATTCTCTACCGCCGTCGGCGCAAACGCCGGCGTCTCGTTGCCTTCTCACCCGCCGG
TGCCGTTCCCGGACCACCAGTTCTACTTCCCACCGACGGCATGAGCTTGTTGGTCTGGTTCAACTCTTGGGACGGCAGCTGATCTTATGTGTGAACATTT
CGCAGCTTGCCGGTGACCGTTGTTAATTAATCGGGGAGAGAGAGCCAGAAGACCCAGTATGGCTATCTCTCTGTGGCCCGTGCCTTGTTATGATCTTAGT
TATTGCTACTACACCGTGGATTCATCATGGTTTATGAGATTCTTTACTCAAGTTTCAGCTTAAATGTCCAAATACATGAATGCATCATACACAGGATCCG
TCATTTTCTAAAAAAATACAGGATCTGTGTGAAGTGT
>Locus_25981_Transcript_7/14_Confidence_0.381_Length_751
TCTACAGGAGGACCGGCCGCTGGGAGTCGCACATCTGGGACTGCGGGAAGCAGGTCTACCTGGGTGGATTTGATACAGCGCACGCTGCGGCAAGGGCCTA
TGATCGCGCTGCGATCAAGTTCAGGGGCCTTGATGCGGACATCAACTTCCAGTTGAAGGACTATGAGGACGACTGGAAGCAGATGAGGAAATGGTCCAAG
GAGGAGTTTGTTCACATACTCCGACGTCAAAGCACTGGGTTTACAAGGGGGAGCTCAAAGTACCGCGGTGTGACACTGCACAAGTGCGGCCGGTGGGAAG
CTCGGATGGGTCAGCTTCTTGGAAAGAAGTACATCTACCTTGGGTTGTTTGACAGTGAAGTTGAAGCTGCAAGAGCATATGACAGGGCAACCATTCGCTT
CAATGGAAGGGATGCTGTTACTAATTTTGATCCTAGTTCCTATGATGGAGATGTTCCACCTGAAATTGAGAAAGAAGGGGTTGATGGGGACATCCTTGAT
TTAAATTTGAGGATCTCGCAACCTAACATGCATGATTCGAAAAGTGATGGCATCCTGACTGGGTTCGGATTAAGTTGTGATTTTCCTGAAGCTTCAAGTT
CTGTTGTTTCTCAGGAAGTGCCTATGGAGAAACGACCCGAGTTGTGTCCCCAGTCGTTCCCTCCGTGTGCATGGCAAAAGCAGGGTTCCCCTGCGCCATC
ACTCCCTACTGCAGCATCATCAGGATTCTCTACTGCCACCGGCGGCGCGGA
>comp80692_c0_seq46
GCTCCTTGAGAAACACCATCCAATTACAAGACTATTTGATGCAACAATCACGCATATGGCTGCGCTAGAACTACTGCTCTGAATTAAGAGGCGTTGAAAT
ATTGTCCGGTCTACATATAGGCACTTGAGGTGTATTGACAGACAGTTCCCCTTAGTTTGCTACAACACATCAGGCGAATTCCACCAAATTAGGATGATGT
TTGTTTCTTAGACACAGGATAGCAATGCCAAAAGTTTTTTATAATTTTCCGCTTCACCGCAGATTTGGCATCTCAAAGGGAGTTGTGGCAAGATTCTCTT
GACCAAGCTTTCTTGCAGATTTGGCAAGAAAGATAAAAAAAATGTTGGTATACTTAAGCATCCTTAATAGAAACTAAACAGGTCTTAGATTGAAATTATC
ATAAATAAACCCTTTTTCACTTTAGCTGTAGTCCGGATGAAATCTTAGGGGCCTTGCTTAGCCTGCCCTGAAGCTTGCCAGTAAGTCGAAGCTGATGCTG
ACACAGCACTGAAATAGATCCCAATGAACACAAGTTATTTCGAGAAGATAAAATACCAAGCAAAGCCAGGATTGCATTAAAAGATAGTGTATTTCTACTA
TACGATCTGAAAGCTTAATTTATTATATCAATAATCAAATTGGCAGCAGTGTGGAACAGATATAAAGAAGATGCTTCAAATCACAAATAACTACAGATAA
ATTGAAGGGTGAAGGTTCATTCAACCACCAAACACCCAAAATGTCGAGCTGCATATGCACAGAACCTAAGTACGGTACAGTTCATAGTTTGGGAAAATAG
ACTGCAAATGTGCACAGATAATACAGATTGTTACTCGATGAACAGTAAGCACGGTATTTGAAATCCTTCTAACCATCCATCCAGGCAGTGCCTCGTATAC
TCAAACCTCTTTTTGGAGGAAGCCAAACCATGTGATCCTGTTTCAAGTTCTCATCTTCTGAAAATGATCAGCAGTCAATAGATGTTTTAGTCCATTCTCC
AGAAAAATGAGTGCCGCATGAATCCTTTCCGGTTTATAGTGAAACATTTTAGACGATGACCTTTAACATTCTAGAAAACGAAGAGAAAACCTACAGTCAT
CTAATTTCAGCTTAAACATGTTCCAGCAAGATCATCACCATCCTGACATGTTCCGTTTTGTCCCTAGACCCGAGCACAAGGTATTGCGAGGCTAATTTAG
CTCAATTCAGTTGTCAGAGCAATTGTGTGTGAAAAGTGTGAACAACAGCATAGGAGACAATACAGCAAGTGTAGCGTGCCAATATGTGTCCAGCTTGATA
GTTCACTATAATAAAGTTCTTTAATTCAAGTATTTGAATAATATAGAATCTCAACCTTCAAGTTTAAAAGGTTTTGTATTTGCTATTATAATCGTTGCCA
CCCCCTCACCCCTCTCAAAAAGAGAGTTACTTTGTAGCGCACTGTAAATCTCTTGATTGAAATAGTGGCATGTCATCTTATTTAATTCAGTTATTTCATA
AGGTACAATTTGTTTAGCTGACAGCTCAGACATGCTTCTATGCAACATGTTTATACCAATGCTGTATTTTGGCAATCCTGGATGTGGCATTACTTAACTG
TGCTTTCATATTCAATAGTTCTGGCAAAGGGAGGCTGCAAGCTTGAGACAACAACTGCACAACCTGCAAGAAAGTCATCGGTATGGCTAGAAAACCCTGC
AAATTTTACAAATGTACAAGTCAGAAAATTTGCCTAATGTAAAAGGATCAAACTACAGGATTTGTCAGCATGAGCACAAGTGCCTAACTTCCGAGTTAGA
TAACTCTGGAGGAAGGAAATCCTTCCTACTGTTTGTTTCAAGAATATACAAAAATTCAAAACAACATTAGAATAGAACCAAAAGAAGAAACAATATTCAC
CATGTAGCAATACTCAATTCCTGGTTGTCTTATTTATAAAGGTATAAAACTAGGTTCTGACGAGACAATTATGAATCAACTACACCATGTTACAGATCAT
CCATAACTCTTGGTGATGCAAATTAAGGGCTGTAAAGTGACAAGTATACCTCCACCAACTAATTTGTGCAGAGTGTATAAAATTGTAAAACAATGGATAA
GAATGAATTTAGTATACAAGATAGCATACAATGCCCAAGTTTAAGGAGACATACTGATTTACTGACTCCATAGGACAATCTTTTGCTTATGAGGACATCA
GTTTTCATAAACAATATCAATGTAAAAGGAGGTAACAGTTTATGAATTTCCTATGCCAAGCCATTTATAAGTCAAAAAATGTACCTATTTAGCTTTTATG
CATTCCAATTAACTTCCAAAGAAGAAGAGTACATACTAATGGAAAACAAATATGATATTATCATCTGTTTTGCCCGGTAGTTTCTTGAGTCTCTGATTTG
TCCGCACTTCACTCCCAACCAACACAACATTAATACACATGGGCAGAGGAATATCAGGTTATCAGGTAAACTGAATACAGTTTGATTGACTGCAGTCAGT
TGATGGGACAAGATCTTTCTGGATTGAGCGTCAAGGAACTCCAAAATCTAGAAAATCAGCTAGAAATGAGCCTACGTTGCATCCGGACAAAAAAGGTAAG
GATGCTAGCCATATTTCTGTTTTTCGTTACCAGATTGCCTCATCCAAGAATACAACTGATGATGACGTTTTTTACTTTCAGGACCAACTCTTGATTGATG
AAATTCACGAACTGAATCGAAAGGTTCTGGAATATTCTGTTATTACTCTGCAATGAGAGGTCTCTGAGTGGATTTCAAGAAGCTGACTCTACTGGCTCAT
TTTCAGGGAAGTCTCATGCAACAAGACAAGATGGAACTATACAAAAAGGTCAACCTAATCCGTCAGGAAAACATTGATTTATACAAGAAGCTCTACGAGA
AAGAGGTGACAAGTGAAGTCAACCGAGATTCATCAACTCTAAACTTTGCAGTTGTCGAGAACGTCAACATTCCTGTTCATCTTGAACTTAACACTCCACC
GCAAGAAAATGATGTTGAGCAAACTGCACCTCCTAAACTAGGGTAACCCTGCTTTTTTCTGCAAAGGAACTATTGATTCTGTTCTTAATTCTTTAGTGGC
ATATTGTTTACTACTAACTGAAAACTTGCAGATTACAACTAAATCCATGAAGGCATGCAGCATGGCACCGCTTATGTTCATTATTCTCAACGGTACGATT
CAAAGCAACCAGAAACACTATTGGTTGTAAAGAGCTTAAACACATAGCAAGACTGACGCGGTATGCATGCCATGAGTGATTGATAATGGTAGTGTAAATC
TATTTCAAATAAAATGTATTTTATTGAAGAATACAGCAAATGATTTGTATTATAACTATTTGACACATTCTGCAAGTTTGTTGACCAACGTGAGTACATG
ATAGCTACAAATCTTATATTGACAAAAAGATATGGTGTGTGGAAAAAAATTCAGGTGCTATTTTTTTCTATTTTGAGAATTGTATGCATAGCAGAAGAGCA
CAAATTGCATTCTGGTTTATCCGTGCAGGCATTCCAGAGTATCAGAATGGGGATCAACCTTTCTTCAGCTACATATGTAAATATACGGATGCTAGTTGCT
CACTTAAAATTTTGTAGTGTTCAAAGGAATAACAACAGTAAGACGTTGACGCACCGAAAATAACCAGAAATAACGGATTGTACTATGACAAGACACACGA
CAGATACACTAGCTTAAGTTTGTACAGGAAAATATTTGGCCTATTCTTATGTCACAGTAAATTAAGATTATCACCTTAATCACAGAAATTCAAGGGGTAA
ACGATTATAGCACAAAACGATCCTGGACCCCAAGACCTGGGTCTCTGAACGGCTGAACCTCCAATTATACCTCTATACATTTGGAATGTGTGTAGTCAAA
CTTTCGCTACTGGTATCTTTACAAATATTTACATTGAACACATCAAAATGATATACATGGATTCCTCTCGATATGTCTTTGAGGTTTGGGTTCTAAAAAT
GATATACAAATTATTAGGTTCTAAACTTAAATGATATATACACATCAAAATGATATACAAATTATTGGGTTCTAAACTTAAATGATGTATACACATCG
>Locus_839_Transcript_113/261_Confidence_0.019_Length_596
CTTTATACCGTGGATAAGTTTCAGATTCATCTGGTGCTTTCGTTTCCTGCATAACCTTTAGCTCACACTCCAAACATAACATCCCTCTTCGTGGCAGCCA
TACCTTCACGAGTCTCGGCATCAGCATGCCATGAAGAGAGCTCGGGGAACAGGTGGGCGGTTTCTGAACACGAAGCAGCAGTCAGAGGCTCCTGGCGGCG
GCACCTCGGACGCGCAACTCATGCCACCAAATGGTGGCCTGTTCGCGAAGCACGAGCACAGCTTACCACCCAGTGATCTCCATTATCGCGCAAGAGGGGG
CGCTTAAGGAGTCCCATGGCAACTCATCCTTGGCTTAAAAAAAATGTGTGGCGCTCAGCAATTCGACCTCTGCTAGTTCAATGTAAAGGTGTGGGACTGT
AACTGTGGGTGCTTGAATTGGCCGCAGCCATTCCTGCGACGCGTCGCAGGTGTTGCCGGTTCTGTGGTCGTGCTAGACTTGTGACCGGCACGACGAAACT
GGCACCTGTTTCCTGTAACTGTTGTCTAAGATGATGATGACTACTCCAATAACCATTCTGTATGATGCTTTGACGTCGGTGTTGCTCCTTCAAGTC
>Locus_20324_Transcript_21/30_Confidence_0.180_Length_863
AGTTGCTGTACTCCCGTTAGTGTTAACTTTTGGTGTACTTAGCTTGATTATGCTTTGCTGATGCTATTTCCCCCCCTTCAGGTGGATTTGATACAGCGCA
CGCTGCGGCAAGGGCCTATGATCGCGCTGCGATCAAGTTCAGGGGCCTTGATGCGGACATCAACTTCCAGTTGAAGGACTATGAGGACGACTGGAAGCAG
ATGAGGAAATGGTCCAAGGAGGAGTTTGTTCACATACTCCGACGTCAAAGCACTGGGTTTACAAGGGGGAGCTCCATGTACCGCGGTGTGACACTGCACA
AGTGCGGCCGGTGGGAAGCTCGGATGGGTCAGCTTCTTGGAAAGAAGTACATCTACCTTGGGTTGTTTGACAGTGAAGTTGAAGCTGCAAGAGCATATGA
CAGGGCAACCATTCGCTTCAATGGAAGGGATGCTGTTACTAATTTTGATCCTAGTTCCTATGATGGAGATGTTCCACCTGAAATTGAGAAAGATGTGGTT
GATGTTGATGGGGACATCCTCGATTTAAATTTGAGGATCTCACAACCTAACATGCATGATTCCAAAAGTGATGGCGTCCTCACTGGGGGATTAAATTGTG
ATTCCCCTGAAGCTTCAAGTTCGGTTGTTTCTCAGCCAATAAGCCCTCAGTGGCCTGTACATCACCAGAGCACATCGACTGCACCCCAGCATCCACATTT
GTATGCATCTCCTTGTCCGGGCTTCTCTGTGAACCTCAGGGAAGTGCCTATGGAGAAACGACCCGAGTTGCGTCCCCAGTCGTTCCCCTCATGGGCATGG
CAAATGCAGGGCTCCCCTGCGCGATCACTCCCTACTGCAGCATCATCAGGATTCTCTACTGCC

>comp81836_c0_seq5
AGAGAGAGAGAGAGAGAGTTGAAAATTCATCAAGGCTAAAGGGAGAAGAGAGAGAGTACAGCCTTGCCATTTATACACCTCACAGCTCACCAGTTCTTTT
GCTATTATGAGTGTGATGAAGGCTGTACCCTCTCTCTTCTCCTCTTAGCTTCTTGATGCAAAGCTGCAAACACACTTTGGGAGCTCTTGCAGAAGTACCG
AATCTTGATCATCCATAAGGATCCTGTCAAACACTGACATTTTGGTTAGTTTCTTTTTCTTTAATTTCAAAGCGCTCCTAACACATGTGTTGGACTAAGT
CGTGTATATATGAAAGGCTGAACTGCATATGCTACTCTAGAATGTTGATATGTATAGTTGTTTTTGTTACTTAATTAGTATGTGCCGTTAACACTTGGCA
CTTCATGCGGTGTATCCACAAGTCTGACTTTGTCATTCCTGTCATTGCAACTGTTTGTCAACCTATAGTACTCCATAATACTTGAATTAGTTCTGTATGT
GTCACAACAAGGGACATTTTTCTTACCTGCTTAACCACTTTCCGTGCCCCCACACTATAATAAGACTACATGCCTATTAGAGTGCAATATCTAGCTAATA
TCCCTGTAGTTTGTATCTTCCTTCACAAAAGGAAACGATTTTTTGAAAATTGAATGTAAGTTAAAAGGGCCTTTTGGGCTATTTTCTTGAAACTGTAAAA
AAATAAGGTTGACGAAAATCTCAGTGCACCCGTTATTTGCTCACCACAATATCAACCCAAAAATCTCTCAGCGTGTTTCTGACGAGATAATAGTGGAGTG
TAGAGTATCTCTATTATTTGAGACACACTCATCAAACTACTACAAGTGAATGGACCTATAACATGCTAGAACGTAACAGAAGGAACTCGACCGCAAGTTA
AATTTCATGGCACCTAAGGCTAAGGGATATTTTTCCTTTGCTTGATGTAATAGAACTTCCCTTTATCTAAAAAATAGGCTTTCAATATAATGCATATATG
TGCGCTCGTGTTACAATTTCATGTTCTAATGTAGCAGAACTCTATATTGACAACTAAATGATGGATGGGATGATGAAACTGTGATTCTCGCCAATACTAA
AATATTCACAAGAACCTAAAGGCTATGTAGTCTCTGATATTATGTTTTTATATTTTTTCCAATGTAATTTTCTACTCTTTAAAAATATTTATCATATAGG
ACTCTGGCGTGCATAACTAATTTTTAGTTAAAAGAGTAAATATAATATCATCTATCAACTCTGCAAAAAAAAAAGCAAATACCATATATAAAGGTGAGAT
GAAGTCATATTATTTTTTACTTTTTATCTCAACGATCACCTAGCCGTCCATTGACTTTTTGAATGTACGTGGTAGACCTCATTTGTTGGTATATCATTCA
GACTTGAATTATGTATATTGTCTGGATTTGTCATTTTACTAAGAACCAGCACAAAAAAGAATTATGTTAAGTGAACTTACAGGACATTGAAACAACAGGG
ACAGATGCAGAATAAATATTCATTGATGTCACGCTGAGCAATTTTTTTAGATAAAGGAAAAAGTTCCGGTCTTATGCATCAAACAATGCATACGGCCTGT
GTTCATTATTACAAAGTCACAACATTTCATTGGAAACTTAAGATTAGAATAATAAACTAACAAGACTGTAAAATTAATTGTATAACCTATGATTAGATCA
CTACCCATTCTTGACGAACACCTCTATTGCCACCACTTCAATGGGCTGGCATGCTTGCATGACATCCTCTCTTTCTGCTTCCTTCTGCAACAATCTCCAG
AACCTTAGATAGTTTGTGCCCCTGAAAATTGCCTATAAAACTGATAGTGAAGATTTCTTATTAAATATGATATCAATTACGGCATAACCATATAGCTCAA
AATAAAGCTGCTACCCCTACCAAGACCTTTTTCTTTTGTGATACTCCCATATTGCTAAGCCAACCACTCATAATATGAGTGACATTTCTAGGTATAACAA
TGCCTAGGGCAATCCTAATTATTCGCCAAATGGTTTTTGCAAAGAAACATTAAAAAAAGATGTTGTATCGTTTCATTATGGTTACAAAAACAACATTTTA
GGCTTCTGGTCCAATTCCTTTTAGCCAAATTATCTTTGGTTAGAATGACATCTATACCTAAATACCACAGGAAGATCCTGATTTTGAGAGGGAGTTTAAA
TTTCCATAACTAATTTGTTGGGAAAGAGGAGGGTTGGTTTATCATTCGCTTGTACATAGAATGGACAGAGAATTGTCCATTGGCATACAATCCCCACCTA
AAGAAATCACGTCCATCATTCAAATTAATATGGACTAAATGTGCAATAAGATCATTCCATGCATTCAGTTTGTCCCCAATAATTGGTCTACGGAAGGACA
GGTTAAGAGATACTGTTGCCATTACATCCACCACAGTTGCATTCTTCCTTCTCACCACATCATATAGAGTAGAATACTAGGTGGTTAATGGCCTATCATT
CAGCCAAACACTGTGCCAAAATGTGGTTTGGCTGCCATCTTTAACTTGGAAGGAACCATATTGTAAGAAGGTAGTTTTAACTTTTATTAGACCAGCCCAG
AATTGAGAATCACCTGATTTGACTTCTACTTGAGCGATGGATTTTCCTTGGAGGTATTTGTTTCTGAGCAACCTTTGCCAAACCCCATCCTCATTAAGCA
ATTTAAATAATCATTTAAATCACGCTAAGCATGTGTTCATTCTTTGCACTAAGATTAGTTGGAACAAGCAAGAACTTCACTTTGACATGCAAATATCCAG
TGGTGTCACTCGACACCATATCTTACAATGTAGATCCATCCCTAGGAACAACAATAGTCAATTCAGATATTAGTTTCCTTGTATGGACCGTTACTGGTGA
GTACGATTTATCCCACTAGAATGTGCAAGTAGATCAGAAATATGTAAATAATTCTTAGTTCAAATTGCAAAAGAGTGGGATGCAAATAGATATTGAGCTC
ATAACATTAGAGAACATCATGAATTCACTGAATGACAAACATATTGCTATTTGGCATAAGAAGACTGAACTTATATAATTGCAATTTTTCGCCACCATTT
CAAAATACTTAAATTTTTTTCCTGTGAGGAATCTAAAACACTTGCTGATGAGACATACATGCTAATATCTTCTTTTCTGTTTTCTCTTAACATGTTGTTG
TACATGGCTGGACCTTTCGTGCAACGCCACTTTTTGTTTGACTCCTTTTTTAACCATGAATTTGTACCGCATTCAGTTTATGACACAACTGACTGCACTG
TTAATTGAATGACTCAAGAATCACTATGCCGCAACAAATGCCTTCACTGGCCTGTTATATCAGTCTCCATGATTAGTATTTCTACTAACTAATTCCCTTT
TTGAGAAATCGATCGGAACCAATGTAACGTCGCGTAGACTGATCATTTGATGGAAACAACATATTGGAAAGTGTACCATAAACATCTTTCTTACTACACT
ACACCAAAACAGGTCATTGGATGCGCCTTATTAGTGTGGTTGCACGAAAAGTGGTACTGATAACATGGTGGCTTATAACTTTCTCGCGCTCAATCAATGA
TTGAGCAGTAAGAACCGTTACTGATAATCAGTATCAATAGCGGCTAGTAAAATCCAACCAGCACTGATAATCTTTATCAATAGCGGTTCCTTTTGATGAC
TAAGAACCGCTATTAATATTTTTTATCAGTGCTGGTCCTTGTTACGAGCCGACTGTGATAATGGATGATATCATATCTTCAAATAATTATAATTTTTTCA
TACGGTGTCAGATGGATACAAATTTTATATAAAAATTATAGCTCTAAAAGAGATCTACAATTTTAAAGTTGATAACTTTTTATTTGAAGTCATATAGATA
TCCAAACTTGAGTTATAAACAATTTAACACATGTAACTTATAAGACTCGAGTTATTTTGTTATGGTTCACTCATGAACTTCATAAAAATGGATAAAACTA
GTAATAAACATTGAAATAATAATATATGCAATGGTGAATTGATAAGACATACAATGCTAAAATTTGTTCATGTTGAAGCGGGAAAAAGATGTGAGTCTGC
ATATATTTTTAGTTGGATTTTCAAAAAACCATAAAATATTCATATATTTATAGATAAAAAACTATTTATATAAAAATTATAGCTCTCGACGAGATCTATA
ATTTTGTAATTGATAACCATTTTATTTAAAGTCATTTACATAGTCAAATGTTTATACAAAGTATTCAGCAAGAAGGATAAAAAGATATAAGTATCGCATT
GTGATCACAAAGAGTTGATAGCTGATATGGGAAAGAAGCTACGCGTGAGGGGGGAGGTCACAAGTTCAAATCCCATGAACGATACTTGTGCAAAAAATTA
TGTAACTTGCAAGGTGGGACAATGCACGCGTGTGGTGGCTCGGGTGGCTTGTTTGGTGAATTGTTTTCATTTTTCAGCCCCACAAAGTTCAATTTTGAAC
CAACTATCAGTAGCAGTTCTTAATAAGGACTGCCACTATTAAGTGTATCAATAGCGGTTGTATAACCGCCATTGATAATCATCGATTATAAGCATCGAGT
AATTAGTGGGGGTTCAAAAATAGGCACTGATAGAATTTTTGAGCCGCCACTGATGACCCTTTTTCTAGTAGTTCATGTAGCATTAATGTGCAGCAAATTG
CACTTACATTTGCACCGCATGTCAATACCACAGATCCCTACAGTAGCAATGACGTCATTAGTGATGGGCTTTTCCGACCCATTGCTATTGGGTCACCAGC
GACGGTCGTTGTGAAGGCTGTCGCCAACGTTGTGGCCAGCACGAATCTGTAGCAATGACGTCCTAATAGTGGCTGTCGCCATTAAATGGACAGCGCAATG
GCGTCGGGCGTTAGGTAACGTACATCGCCATTGATCCGTCATAAAGGCGATGTACTTCCCGTTGACGAGCGTCGCCAAAGATGGGACAACAACCAGCGAC
GATTTTTTCACAGCCTCCATCGCCAAAGAACGGCCTTCACACCTCTTCGCATTCCCATGTGCCTCCTCACCTTCCCATAGTGCAATAACTACCCACACTT
TGCGAGGTGCTGCGATGTGGCATATCAACTGCTCCTCCAAATGCTCCATATAATTAACTGGTGTGGGACGAAACTTCAACCACACTTAACTTCAACCACA
CACTACATAGAAAACCCCAAACGCCTCCTTAGTCCTCATCACTCCTTCATCATCCTCACGCTTCGATCTCTACTAGGTCCCTCAGTTTTCAGGAACGACG
ACGACATGGTTGAGGCAACGACAAGAGGAAGAGGTCACTGGAGGACGAGATTCGGGTGAGCAAGTGTCCCAAGAAGCGTCGGTAGGAGTTGGAGGAGGAG
ATCTAGGCCCTGCGGGAGTCCCTTCGTGCGACCGAGGAGGAGCATGCCATGGCCCTCATCGCGGTTTAGGAGTTGCATGCCACGGTCCTCAAGATGACCA
TGGAGGAGCAAGTGTGGCCCTCCACGCCACTGAGGAGGAGTGTGCCATAGCGTGCTGAGCTTCGATGAGACATCTGCGACGTGTTAGGGGCCATGGGCTA
TAACCTTGATAGGGTTTTCGACTCTATCAAGGTTTGAAACCCAGGCATCCAGGGAAAGGCCAGGAGAAATGGCCTGGAGGTCTTTAGCAAGGACTTCCTC
ACCAAGGACCTCATGGTGAGGGTCTGTTGTCAGGTCATGGCTCCACGCGTCCTGCTCGGCGCTGACGTCGACTTGCCTCTATCGTCCTCTCTCGGGTAAA
GGTCCGCTATCCCACCGTCGGCCTCGTCCTGTCCACAGAGCCAAACCCCAACGAGGATGACGCCATCTGCGAGGCCTGCTGCATCGTCGACAAGGTCGTG
CATCATCTGGGCTTCGGTGACAAGGGTGTTGCGGGAAGCTCCGACTCTGACAATGCAGCCTCTGACAGTTTCGGCTCTGGGGGCATGGCCTCTAGCAGTT
TTGGCTCTAGTGGCTGCTCAGCCTAATCCATCGGTGGTGAGGACGGCAACAATGAGCAGAAAGACAGTAGCCACGACGAGGAGGCCCTTTAATTTATTTT
CTTTATGATTACTATGCCCTTTTTTGTTTTGTAAATATTAAAAACTACTCTATTAGTGGTAATAACCGCCTAATATTATTATCTGGTCGCCTAAATTTAT
TATCTCCTCTATTTCTTATTTTTTTTGCCGACTAATGATCGCTAGTGCGCTATTAAGATGAGCCACTAGCACGAATCTCAAAATTTGAACAGTTATCACT
CCCAATTCTAGCGCTTCACTTGCCCAGTGTCGCTTACATACAGGCCCCAGTATTCCGGCTCCACTGCTCCAGCCCTACATCCAGTGAGACGAGGTTAAAG
AGAAGGATGTGAGGAGAGCTTAGGGTTTAGAGTGCGCAAAAATGATCCCGCTCGAGTGTTTAGAGTGAGCACAAGTCCCTGCAGGGAGCTTAGGGTTTCA
TGCTCAAGTCGCCACCACTCGAGTCCCTCACTTGGGGTTTGTAGCTCCTGCGCCACCGCTGGATCCCATCACCATCCTTTGTTGAGTGCCATGCATCGCT
GTTGTGCTTCCCCATCGACGCCACCGAGCTTCCCCATCAACACCGCCGTGCTTCCCCATCGACGCCGACACCACCGACTCCACAGAATAGGGAGAAAGAG
GGATGTTGCCTTCCCGACCGTGATGTGGAACTTGATGCCTCCTCCGCGAACGACACACCGTCGTCCTCGGCTTAATTCCCTATCGATGTCCTCCTCTTCA
TCCTTGTCGTCTCCAGGCATCCGTGCTAGCCTCGCATCCTGGACTGGGTAGTTTCCTTTCTTGCCGAGTTTCTTGATGCTAGAATTATTGTGCCGCAATC
TAGTGTTACCACTTCTTACCCTT
>comp67351_c0_seq16
GGCAAGACGGATTACGACGTGAGAGGCGAGAGTAGCGAAGAGTCACGGAGATCAGGGGAATCAAAAGATGGAAGTGTTGGTCAAGAAAACAACCATGCAA
CATCACATATGCCTGCTTTGGTGTCGGAGTATTTAGCACCATATACACAGCTGGAACTGAACCAATCAGTTGTGTGCAATCTTTTCTCCATGGACTCTGT
TTATTGCCTGTGTTTCCATTTTCTGTTGACTTGTCTCTAATGAGCTTATCGTCTCATGTTCACATCTTTCCTTGTGTAGGCTCCTGCAACATATCAGTAC
CCAGATCCTTACTATGCAGGCATGACCCATTTCCAGCTACCTGGGTTAACTCACCCTCGCATGCCATTACCTCTTGAAATATCTGAGGAGCCTGTCTATG
TAAATGCAAAGCAGTACCATGGAATTTTAAGACGAAGGCAGTCACGTGCCAAGGCTGAGCTTGAGAAAAAGGTGGTTAAAGTCAGAAAGCCTTATCTTCA
CGAATCTCGCCATCAACATGCAATGAGGAGGGCAAGAGGAAATGGGGGTCGCTTCCTAAACACAAAGAAAAGCGACAATGGTACTCCCAACGGTAAAGCT

GAACCTAAGAAAGGAGAGCAAAACTCCAAGCATATCCATGTCCCTCCTGATTTGCAACTTCGACAGAAGGAGGCATGAAGTAGCGGTCTGGCAACCTAAA
ACAGTGGCTTCTGTCCACAGGCATACACACTTTTGAGTCCGGATTTGGAGCCCCAGGGTTGCGCTGCCATCTTTGCAGTTCGTCTTCTTTGTGCAAAGAG
GTCCAAGGTTCGTCCACCCGAATGAGCTTGGTACCAGTTGTTAGTCCTGGCAGAATGCAATCTGGGTGCAGGAGACCGTTGAAGTGTCCAATGCCATGGG
TAGGTCTGCTGTTTTCAGGCAATTCATTCTTGGCTTTCACATTCCGCCCCTTGTTTGTACCTGGTTTGTTGTAACTTGTAAGTGGAACTAGCTTGTGCTAT
CTTCGGCGCCCTGTGAAACATATGTATAATTAAGATAAACTTCGGATCATATTTGTATCCCGGATCATATTTGTATCCTATGTCTGGCTTTCGTTCAGTT
TGAACTGTAACAAGGCCAAAGCTGAACTCGTTAAACAGAGGTAGCAACTCATGTTGGCCTGTTGGGTGTTCATCAATGTTGTTGTAATGAATCCTGTACA
AAGGCAGAAGCTTATTTCGA
>Locus_17813_Transcript_16/20_Confidence_0.333_Length_1166
TGCTTATACCACTGAATATGCCCGCGCAGCCGCAGAGAGTGCTCCCTGATGTCCTGAAGTTCTCCATGGCTCAAGGTAAAGTGGGGAAAGGCTCTGAGCA
TTCTGCCACTGTTGCTTTGCAATCACCATTCACAGTATACAATGGTCGTTTTGAGCTGGGCCTTGGTCAGTCAATGCCTGCCGGGCGTATGCTTATACCA
CTGAATATGCCAACTGATGCCCCAATTTATGTGAATGCCAAACAATACGAAGGCATCCTTCGCCGCCGCCGTGCTCGTGCCAAGGCAGAGAGGGAAAATA
GGCTGGTCAAAGTCAGAAAGCCATATCTCCATGAGTCACGTCATCTTCATGCACTGCGTCGTGCAAGAGGCTCTGGTGGACGCTTCCTGAACACAAAGAA
AGAGGTCAATGGGAAGGACACTGGTGTCGGAAGCAAGGCAATAGTCAGCAATCCACTAATGCGCTTGACTGCATCCCCAAGTTCAGAGATTCAGCAGTCA
GACCTGGGCAACCCAAGCAGCGTCTCCAGCCTATCCGGCTCGGAGGTGACAAGCATGTATGATCATGAAGATGTGGATCACTACCACAGCTTTGATCACC
TCCGGACACCCTTTTTCACCCCACTTCCGAGCATCATGGACGGTGAGCATGGGGATGGTAACCCCTTCAAGTGGGCAGCAGCCTGTGAAGGCTGCTGCGA
CCTCCTCAAAGACTGGAGGGGATACCGGGCAGCTGAGGGTCAGGGATTGTCCCTGCCATCGTTTGGCTGTCGGTAGGCAAATCATTCTTGGCTCAAGGCA
TTGTGGTGCTTTCTGCTCATCGCCTAATTCTCCTAGAAACCATAGTTGCTGGGATCTTGTGGTGAGTGGAACACCCCATGCCTGAAAAAAAATCTGATCT
AAATTAGTCGTACTAGCTACTGCTATCTGAATCGTTTCGCGGTAGCAGGTTTCAGCTTAGTTTTCTGAATAAATCCTGAAACTTAAGTTTAGTTTGGAGT
TTCAGAGGTATTGTCATTGTAGTATGTTTGGACTTTGGTGTCTGATTAGTATCAAGACTATGGCTTTGTTGGTGATGAATTGTGGTGTGCTTGAAGCTTG
TGCGGACTTTGGTTTGTACTTTATGGTGTGTTCAAAACTTGAATTTGGCAATGTAATAATGTACTG
>Locus_14087_Transcript_42/66_Confidence_0.296_Length_2209
CTTGAAGAGGATAGGTACAATGTGGCTTAGCTAGCACCTTGCAGTCAGGTTTGGAATTATTGCTTTCTTTGTAAACCAGTATATTTTTTGTGGCATGGTA
TTGTTTGTGGACGAGAGAAGTGAGATTCTTCCCCCATGCTCACTCAGACACCTCCTCCAATGGGGCAAGACGAATTGGCCGTCTATGGAGGTGGGCTGTA
GATGATATCCACCACAAATGGGGTGCTCTTCCCTTTCTTACAGGATTGAAAGCTGCTTTTGAGTGGAGTAGTACTGAACTACTGATTATCTAGTAGAGAG
GCAGTGGCAGGAGTAGTAGTTGTTGGGGTTTCAGGGGAGGACATGTGCATGGTTGCTGTGTGGGGAGGTAGCCATCCACTGGGCCTCACACTCTTCCCTG
CCTCAGGTGGGGTTTGTGGTGAGAAGGCAGCGGAAAGCTTGCCTACCATCTCTGTGTGTGTTGGATCTTTGCGAGCCGTGGCCTCTTCGTCCTCTTGTTT
TTCTCTCTTTTCTTGTTGAACTCATGGTTCTTGTTTCTCTTATGCCTGCCAGGTAGAATTATTCAACCGGATAAAGATTATTTCAAGTAAAGGAGGGCTA
CCAGTTTATACCTGTTCAGGTGGGTGCAGCAGTTGAGAACTTTTTTTCGATACAGAGCAGTTGAGATCTTTAATTCTGGCATGGGTTCCTTTGGGGTGAA
CTGGAATCAGAAGAACTCCGCGGTGTGCGATTGGGAAAATTTAGCACCTTCTGTTCCAAATGCAATCGGAAACCCCAGGAACGGAGCTACGAGGCATGGA
TCTGTGAATTCATCTGGTGGCACTCTCACTTCTAGCTCAGAGCTAGGGCATGGTTCATCCAAGAGCTCCATGTCGGCATCCATTGATTCACCGTCCAAAG
TAGGAAACAGCTTGGAGTTCAACTTTGCTGCTGTCAAAGGCCATGTTAAGAACATGGATAAGGATTTTAGATTTGACGACTTGGGGACTTCTCCATCGTC
GATGATAGCATTCAGCCATGGAGAGCCATTAATCAGCCTTAAGCTTGGAAAAAGGACTTACTTTGAAAATGTCTGCGGAGGGCAGGATTCCAAGAGCACT
GCACCTTCTACCAGGACTTCTCCATCAACCGTTCTCAAGAAGACCAAGGTGTCTCAGCAGAATACACAAATGTCATACTGTCAGGTTGAAGGATGCGGAA
TTGATCTGTCCTCCGCTAAAGATTACCACCGCAAGCACAAAGTCTGTGAAGCTCATTCTAAAGCCCCGAAGGTTGTTGTTGCTGGTGTAGAGCGCCGTTT
TTGCCAGCAGTGTAGCCGGTTTCATGGCTTAGCCGAGTTTGACCAGAACAAACGAAGTTGCCGTAAGCGTCTTACTCATCATAACGCACGAAGGAGGAAA
CCACAGGCAGATACAATTTTTATTCAGTTCATCACGTCTCTCAACAATGTTTTATGATACAAGCCAGCGGACAAATCTTTTCTGTAGTCAGCCTCTTTTTG
GCCAAGTGAGAAGCAATGCAGTTTCTTCATGGGATAACTTGGGAAGCTTCAATTTTATGGAAACAAAACATCTGTTGATGCAGCCAACGAAAACGGCAGC
CCCTGATGAGCTGCATTTCTCAACCCCCCAGATGTCAAATGGTGTTATGGTTCACGCTGTACATCATCATAATGTCGGTGAGTTCATGCCATTCAAGGAT
ACCACCACAAAGATCCTCAACCAAGGTGTGGAAGCTTCTGCGATTGGATCCAACTCAAATGGAGACCCGGATCTTGGGCGTGCTCTCTCTCTTCTGTCAA
ACGGTTCGTGGGGTTCAACTTCAGCCGTCATCCAACAGCCTAGTTCTCATGCGCAAGCTGGTGCGGTGCCACCCTTCGCCACCGTCGCCGTCTCCAACCC
TGCAATGCATCCTCTGGACTCATCCCCGGGAGGATTCTGGCAAGACGACCCTTCCCCGCTCGATGGAACTCCGGAGATTCAGGCATTCGGACACCTCTGA
CTATGATCAAACAAATGTACTGATGCTGCAACCTGGGAACAGTTGATGTAGTCACTTCACCATAATTTGCAGTCAGTCTCAGTACAATGTCATTTCTATC
AGTGGAGGCATTTGTTGAGGTGAAGACTGAATAATATAGGAGGCAACCCCCTAATCTTCCTGATTTAGGATACATTTGTGTGCTTGTATTGCTGCCTGAA
GTGGTTTTT
>comp76332_c0_seq33
ACTGCTCTGAATTAAGAGGCGTTGAAATATTGTCCGGTCTACATATAGGCACTTGAGGTGTATTGACAGACAGTTCCCCTTAGTTTGCTACAACACATCA
GGCGAATTCCACCAAATTAGGATGATGTTTGTTTCTTAGACACAGGATAGCAATGCCAAAAGTCTTTTATAATTTTCCGCTTCACCGCAGATTTGGCATC
TCAAAGGGAGTTGTGGCAAGATTCTCTTGACCAAGCTTTCTTGCAGATTTGGCAAGAAAGATAAAAAAAATGTTGGTATACTTAAGCATCCTTAATAGAA
ACTAAACAGGTCTTAGATTGAAATTATCATGAATAAACCCTTTTTCACTTTAGCTGTAGTCCGGATGAAATCTTAGGGGCCTTGCTTAGCCTGCCCTGAA
GCTTGCCAGTAAGTCGAAGCTGATGCTGACACAGCACTGTTTCAAGTTCTCATCTTCTGAAAATGATCAGCAGTCAATAGATGTTTTAGTCCATTCTCCA
GAAAAATGAGTGCCGCATGAATCCTTTCCGGTTTATAGTGAAACATTTTAGACGATGACCTTTAACATTCTAGAAAACGAAGAGAAAACCTACAGTCATC
TAATTTCAGCTTAAACATGTTCCAGCAAGATCATCACCATCCTGACATGTTCCGTTTTGTCCCTAGACCCGAGCACAAGGTATTGCGAGGCTAATTTAGC
GGGGCAATTGATTTTTTGCCACTATGATACAACTGCAATTACCCGCTGTCACTGACACGTGGGGTCATCTGAGTCACTGAAATGTGGGCCCAATAGCATT
TTGTTTGCCACTCACCCTTCCAGAGTGGCAAAAAGTTAATTGTCCCTAATTTAGCTCAATTCAGTTGTCAGAGCAATTGTGTGTGAAAAGTGTGAACAAC
AGCATAGGAGACAATACAGCAAGTGTAGCGTGCCAATATGTGTCCAGCTTGATAGTTCACTATAATAAAGTTCTTTAATTCAAGTATTTGAATAATATAG
AATCTCAACCTTCAAGTTTAAAAGGTTTTGTATTTGCTATTATAATCGTTGCCACCCCCTCACCCCTCTCAAAAAGAGAGTTACTTTGTAGCGCACTGTA
AATCTCTTGATTGAAATAGTGGCATGTCATCTTATTTAATTCAGTTATTTCATAAGGTACAATTTGTTTAGCTGACAGCTCAGACATGCTTCTATGCAAC
ATGTTTATACCAATGCTGTATTTTGGCAATCCTGGATGTGGCATTACTTAACTGTGCTTTCATATTCAATAGTTCTGGCAAAGGGAGGCTGCAAGCTTGA
GACAACAACTGCACAACCTGCAAGAAAGTCATCGGTATGGCTAGAAAACCCTGCAAATTTTACAAATGTACAAGTCAGAAAATTTGCCTAATGTAAAAGG
ATCAAACTACAGGATTTGTCAGCATGAGCACAAGTGCCTAACTTCCGAGTTAGATAACTCTGGAGGAAGGAAATCCTTCCTACTGTTTGTTTCAAGAATA
TACAAAAATTCAAAACAACATTAGAATAGAACCAAAAGAAGAAACAATATTCACCATGTAGCAATACTCAATTCCTGGTTGTCTTATTTATAAAGGTATA
AAACTAGGTTCTGACGAGACAATTATGAATCAACTACACCATGTTACAGATCATCCATAACTCTTGGTGATGCAAATTAAGGGCTGTAAAGTGACAAGTA
TACCTCCACCAACTAATTTGTGCAGAGTGTATAAAATTGTAAAACAATGGATAAGAATGAATTTAGTATACAAGATAGCATACAATGCCCAAGTTTAAGG
AGACATACTGATTTACTGACTCCATAGGACAATCTTTTGCTTATGAGGACATCAGTTTTCATAAACAATATCAATGTAAAAGGAGGTAACAGTTTATGAA
TTTCCTATGCCAAGCCATTTATAAGTCAAAAAATGTACCTATTTAGCTTTTATGCATTCCAATTAACTTCCAAAGAAGAAGAGTACATACTAATGGAAAA
CAAATATGATATTATCATCTGTTTTGCCCGGTAGTTTCTTGAGTCTCTGATTTGTCCGCACTTCACTCCCAACCAACACAACATTAATACACATGGGCAG
AGGAATATCAGGTTATCAGGTAAACTGAATACAGTTTGATTGACTGCAGTCAGTTGATGGGACAAGATCTTTCTGGATTGAGCGTCAAGGAACTCCAAAA
TCTAGAAAATCAGCTAGAAATGAGCCTACGTTGCATCCGGACAAAAAAGGTAAGGATGCTAGCCATATTTCTGTTTTTCGTTACCAGATTGCCTCATCCA
AGAATACAACTGATGATGACGTTTTTTACTTTCAGGACCAACTCTTGATTGATGAAATTCACGAACTGAATCGAAAGGTTCTGGAATATTCTGTTATTAC
TCTGCAATGAGAGGTCTCTGAGTGGATTTCAAGAAGCTGACTCTACTGGCTCATTTTCAGGGAAGTCTCATGCAACAAGACAAGATGGAACTATACAAAA
AGGTCAACCTAATCCGTCAGGAAAACATTGATTTATACAAGAAGCTCTACGAGAAAGAGGTGACAAGTGAAGTCAACCGAGATTCATCAACTCTAAACTT
TGCAGTTGTCGAGAACGTCAACATTCCTGTTCATCTTGAACTTAACACTCCACCGCAAGAAAATGATGTTGAGCAAACTGCACCTCCTAAACTGGGGTAA
CCCTGCTTTTTTCTGCAAAGGAAGATTTTCTTGCAAGTTATGCAGTTGTTGTCTCTAGCTTGCCGCCTCCACTTGCAGTAACTGAGACGCCGGCGCCGAG
CTTGAGCTTGAAACCTAGACTACGGGGACAGGGTAGACAGGAGACGCGAAACCCCAAACCAGCGCAAAACGACGCGCAGGGAGGGAGTGTCACTGAAGGA
GCGACAGGGAAAGCAGCGGCACGCCGGGGGAAATTCCGATCTGAGAGAACGGATCAGCCATGCATGACAGGAGCTGCGGAGGCAGCAGGGGGGGGGGGCT
CCCGCTGCCACGGGACAGTAGGGCACGCCAGATCGGTGAAATCCAGGCGAAGGACGAGGCGTGGGCGAGATTTACACGACGGCGCGATAGGATCTAGCCC
GGATTTGGATTCGACCGACGAGGGCGGAAGGAACGGCAGGGTGGTGCTCTTGTAGAGAGAGAAGGAGTCGGAGGGGGCGGAGAGAGAGAAAGGGGGCAGG
TGGGGTTTCAGGTAGTAAAATATGGAGCGG

>Locus_19362_Transcript_2/13_Confidence_0.308_Length_845
TTTGGCAAGTCCAATTTGTTGGATACCCTCTCCTTGTGAAGAGACTCAGGTTGTCTGACACAATTGGAGATGACCCCATGGCTGCTCCACTGATCGGACT
GGAAAGCCAGGAGATGGCGATACTACTGGGCGCACCAATTCCTGCTGGCATGCAGGGAGCCAGGCACAACGTCCCTACTGATGTACCCTCTTCTTCCTCT
GCAATGATCACAACTCAGCAGCTGTTCCCCATGGATCTTCCGATGCCACCGAGTCTAAGTGGTGGCTCCTCTGAGATTTTGGATCCTGAAATTGGCTCTC
CTCCCAACAACTCAGTAAACATGCATCCATCCAACATTCCTGTGGAAGCGAGGAGCATACAGCTTTTTGGCACCACAATCACACCCCATGTAGAGCAAAG
TGCTACCGATGGTGCTTCTGAAGAAGTGAATGAAGTTCCTGATGGTGTGGTTGATGAAAATGCTGAAGGAGTTGTTTAAACCTTGCAGCTGTACTTGAAG
ATGGTTCGATCAGACTGGGACTAATACAAGGGCGTGCGGCTGCCTTTTGTACTGCAGGATCCTACCAAGTGTCTCGTTTATTCCTCGTCCATCTGACGGG
ATTGGGTGTTTCTGATGCATGCAGTTTATCTGTAGTTTTAGTAGGGTAGGCTGGGTCGTTCACTTGTTATCTGGAGATCGAAGAGGAGAACTTGGGTGCA
GGTGAACCCTTGCTACTTGTTACAGAGGAGCTAGCACTAGTCCGTTCCATTTAGTTTTTCATTGTTTGTTAGACTAATCTAATGTTGATTCCGGGAGACT
CGTATTGGTCTGGTCTGGTCTGGTCTGGTCTGGTCAAAAATGGTC
>comp63680_c0_seq20
AGTATCTCGGCGTGGGACAGAGGCCGGTGGTGGTCGCGAAGAAGACGCGGCGCGGGCCGAGGTCCCGGAGCTCGCAGTACAGGGGCGTCACCTTCTACAG
GAGGACGGGCCGGTGGGAGTCGCACATCTGGGATTGTGGGAAGCAAGTCTACTTAGGTGAGTAACTGAGTTGAGTGAGTTCTATACGTTCCAGCTCGAGC
TTCCATTAACTGCTGATCAGTTTCCGTCCGTTTGATCTGGTTTACGATGTGCTGATGCTGTTCTATTCAGGTGGTTTCGACACTGCTCACGCGGCTGCGA
GGGCTTATGATCGTGCAGCGATCAAGTTCCGAGGGCTCGACGCGGACATCAACTTCACTTTGAGCGACTATGAGGATGACTTGAAGCAGATGAGCAATTG
GACAAAGGAAGAGTTCGTGCACATACTCCGCCGCCAGAGCACGGGGTTCGCGAGGGGGAGCTCCAAGTACCGCGGCGTGACGCTGCACAAGTGCGGCCGC
TGGGAGGCGAGGATGGGTCAACTTCTTGGCAAGAAGTAAGCCAACCTCCTGGCTTTGACCTTTCTACTCTGTTAGCCTAGTTAAGTGTTTTTTACCCTGG
TTATATGAACAAAATATGTCGAAATGATGTTCAATTAACTTGTCATTTCACGTTGGGTTCGATCAGGGCTATATCCCTTATGTTCATGTTAAGTAGTTTC
AGGTGATTGTAGAGAATACAACAAGGTGATCTTGAAGTATCACTGATTATAATCCATGCTACTAGTAGTTTGAGCGTCTCTGTTGCCGGTGAAATCTCCA
CCCCTCTTGTGACTTTACCCCTCTACCGTTTAATCCGGGAATCCGGAATGGACCTACAGATCGAGATATGTGCCTTGATGAACAGTGTTTCGGTGAACAG
CAAAATCGGCACCCGTGCACAGTGTCCTGATGGACGGTACATCTATCTTGGACTCTTTGACAGCGAAGTTGAAGCTGCAAGAGCATATGACAGGGCAGCC
CTTCGCTTCAATGGGGGGGAAGCTGTTACTAATTTTGGGCCTAGCTCCTACAATGGAGGAGATGCTCTACCCGACACCGAAAATGAGGCAACTGTTGATG
GTGACGCGGTTGATTTGGATTTGCGGATTTCACAACCTAATGTGCACGACACTAAAAGGGACAATACCTTAGCTGGAGGCCAGCTAACATGCGACTCCCT
TGAATCTTCAAACACGATGGCCTCTCAGCCAATGAGCTCATCGTACCCGTGGCCTGTGTATCACCAAAGCACGACAGCAGTACCACCTCACCATCAACGC
TTGTACTCATCTGCTTGTCCTGGCTTCTTTTCGAACCTCCAGGAAAGGCCAATGGAGCGAAGGCCTGAGTTGGGTACCCAGTCATTCCCCACCTGGGGAT
GGCAAATGCAGGGCTCCCCTCACATGCCGTTGCATCACTCTGCAGCATCATCAGGATTCTCTACCGCCGTCGGCGCAAACGCCGGCGTCTCGTTGCCTTC
TCACCCGCCGGTGCCGTTCCCGGACCACCAGTTCTACTTCCCACCGACGGCATGAGCTTGTTGGTCTGGTTCAACTCTTGGGACGGCAGCTGATCTTATG
TGTGAACATTTCGCAGCTTGCCGGTGACCGTTGTTAATTAATCGGGGAGAGAGAGCCAGAAGACCCAGTATGGCTATCTCTCTGTCGCCCGTGCCTTGTT
ATGATCTTAGTTATTGCTACTACACCGTGGATTCATCATGGTTTATGAGATTCTTTACTCAAGTTTCAGCTTAAATGTCCAAATACATGAATGCATCATA
CACAGGATCCGTCATTTTCTAAAAAAATACAGGATCTGTGTGAAGTGTAAACATAAGG
>Locus_13957_Transcript_40/58_Confidence_0.294_Length_1356
GCCGCGGGCCCCGCTCCCGCAGCTCGCAGTACCGCGGCGTCACCTTCTATCGCCGCACCGGCCGCTGGGAGTCCCATATTTGGGATTGCGGCAAGCAAGT
GTACTTAGGTGGATTTGACACTGCTCATGCTGCTGCAAGGGCGTATGATCGAGCGGCTATCAAGTTCCGTGGTGTCGACGCCGACATCAACTTCAATCTC
AGTGACTATGAGGACGACATGAAGCAGATGAAGAGCCTGTCCAAGGAGGAGTTCGTGCACGTCCTGCGACGGCAGAGCACCGGGTTCTCACGGGGCAGCT
CCAAGTACAGAGGCGTGACCCTGCACAAGTGCGGCCGATGGGAGGCTCGCATGGGGCAGTTCCTCGGCAAGAAGTACATATATCTTGGGCTATTCGACAG
CGAAGTAGAGGCTGCAAGGGCTTATGATAAGGCCGCGATCAAATGCAATGGTCGAGAAGCTGTGACGAACTTCGAGCCAAGCACGTATGATGGGGAGCTG
CTGACTGAAGTTGCTGATGAAGGTGCTGATGTTGATCTGAACTTGAGCATATCTCAACCAGCTTCTCAAAGCCCGAAAATGGACAAGAACTCCCTTGGTC
TGCAACTCCACCATGGATCGTTTGAAGGCTACGAATTGAAAAGAACAAAGATTGATGCTCCCTCTGAACTCACGGGCCGACCTCATCGGTTCCCTCTTCT
GACCAAGCATCCACCAATCTGGCATGCCCAATCTCATCCCTTCTATTCAAATAATGAGAATGCATCAAGAGATCTTAACAGGAGGCCTGATCAGGGAGGC
ACTGTTCCTAGATGGGCGTGGAAGGTGACCGCCCCTCCTCCCACCCAACCATTGCCGCTGTTCTCGTCATCGTCGTCGTCGTCCGCTGCAGCATCATCAG
GATTCTCCAATACCGCCACGACAGCTGCCCCCACCGCCCCGTCGTCCTCCTCCCTCCGGTTCGACCCGCCGCCGCCGTCGAGCCGCCACCGATGAACATT
GAGAAGCCACGCTGTAAATTTGCCGGGAAGCCGGCATTTTTTCCCTCTCCAGGCGTTGCAACTTTTTCGGTTTTGCGCCGGGGTGGTTTCTTAATTGTAG
TGGATTGGATTCATAACTGTATTTGCATACTGCCCCATGTGAAATGCTTCGTTCTCACTCTCTCTTTACACACTCTGCAACGGATAGATGTTCTTGTTGA
TGGAAATTAGCTAACTTCACCGTCTGAGATGATCTATACATTGTTGTAGAGAATCGAATCATTGTTGAAATTAGTTACAGAATTATAGATATCATAAGGC
ACCCTCTACTTGTGGACAAAGTTCAGGAAGATTATTCCGGTTCTTTTCTTGGCTGT
>Locus_14339_Transcript_48/63_Confidence_0.119_Length_2967
ATTTTTAGCAATGTATTAGTACATCACGCCACACATTTCTAGATTATTTTCTGTAGATAAGAAACTGACAGTACTTCTTGTTTTACTGATAGCTCTGGCA
AAGGGAGGCAGCAAGCTTGAGGCAACAACTGCACAACTTGCAAGAAAGCCACAAGTATGATCTTCCAATTGATTCTGCAATAGAGAGAGATCACTCATCA
ATACACAGTTTATATGACACATGGAAAGATTTACTAGCATGAGAATTGTCAAAAACCAAAATGAAATGTTTCAATCAGCCAGGATGAGGGGAAAATCTAT
TTACTGAAAGACATTTAAACTATTCAGTATTCAGGAAAGGAAATTGATAGTGGCTCTCTCCCAACAACATATAAGTGGTTTTGCTTTGACCTGTGTACCC
ACGAGATTTATATTCGGTTAGTTTCAATGTTGTACACAAGATCTAACGTGATAAACTCTTTTGCATTGTTGTAGGCAACTAATGGGTGAGAAGCTTTCTG
GCCTAGGTGTGAGAGACCTCCAGAGTTTAGAGAATCGTCTTGAAATGAGCCTACGTAGTATCAGAACGAGGAAGGTCATATTCATTTTGTTTCCACGTTT
TGTTTTTCTATTCACTCATCTTAATATGCATAAACATCCATCAAACAGGACAATCTTTTGAAAAGTGAAATTGAAGAGTTGCACAGGAAGGTAAAGCCTT
ACAATCCATGTCTTTTTATTATCTCAAACAAATGACCATGCATGGAGGTTGCTGACATCTTAACTTAATGCTGATTGAAACAGGGTAGCCTAATTCACCA
GGAAAACTTGGAACTCTGTAGAAGAGTAAATACCATGTCACAACAAAATATGGATCTACAAAGAAAGGTTTGATGAACGATCTCATGCAGAATCCTTAAC
CAAATGAAGGTGCATTGTCAGCATAAAGTTAAATTACTTAATTTCATCTCCAGGCGAGTGAACCAAGAGGTGTTGCCGATGCAAATAAAAGCTCTAGCAC
TCCCTACAGATCTTGTAGTGCACAAGATGCAGATATCACGGTTAATCTTGAATTGAGCCAATCACAGCAAAAAGAGGGGGAGCAGTGCCAAAGAGGGGCT
CCAGAACTGGGGTAAATATTCTTTTTCCAGAATCCAAAAACTGCATTACTCCAAGAAGTATCAAGAAGCCTAATGAAGAACCCATGCTTTGACATGTAAC
AGGCTTCAGCTGCACAAAGAAGATGCACTACAAGCTGGAGAGTATCATGATGTTCCACCAATTCTAACATGCAATGCGGAGCACTTAAACTACAATTAGT
ATTTTGTGCAACATGTTAGATGAGTGAACAAAATGGTTTATGTACAGACTACAGATATGACCATCTATCTTATCTGTGGCTCTACTGTACTGAAATTGCA
AGAAATGGTTATTGTAATGACTAATATGATACATTGACTGTCATACCGTTGACCTTGAAGATAGTTCTATAACGGAAAGGTCCAATTTGAAACAAGTGTT
ACGGATGAGCTAGTAGGATTTTTTTCACAAACAATTAGATAGTCCACGTTAGTGGTAGCTGCTGGGTATGACATTTGACCCAGCCTGCCGACCCTCCCTT
TCCCATGGGAGTTCAATTAACATTAGGAATGAATATCGATATTATGGTTGATTATTTGAATCGTATGTTTTCCACCCTTAGATGTAAGGCCCTATTTTAC
TGTTTTAATGGATTAACAAGGTGAGCTTTCGAGATGGTAGCTCATTGATTCGCATGCATTTTAGTGGCTGTTAAGAGGTACTTAGTGCATCCATGCTTGA
AAGTGTTCTTCCTTTCAGCGAAAGGTTTTCTATTCTACTTGCTTGTTATCTTTTGGCCAGTATGTCAAGCAAGAATTTAATCTGCTAATCAAGAATGTAA
TGCGTTCTTTTAACATGAATATCAACAGTTTTCAAGTAACTGAAAACAGTAATGTAGTATTCTCTCGCCGAAAATAAAGCTGTGGTTCCTTGATAGCAAC
CGGCAGTTATGCCAATACCACATTAAAGTGGTTGTGTCCCTTATAATACTCTCTCCATTCTACTTAATTCAATGCTATTTGTTTTTTAACGCATGTCAAG
ATCTGTCTTTGACCATTCCTTTAATCCTTTTGAATATATATTTTTTTAAAAAGTGGTGATATAGCTTTCGTAGGGCCAATATAAGATTTTTTAAGAACAT
ATTAGAGTTGGTCGTGTTTAGGTAGAGCTGTGTGAGGGTATGTGGTGATGCTAATCTGTAACAAGACGGTCTAAAGATACCGCCTTGCGCCTTCATACTT
GTGCCGAATGTATGTAAGCTGGAAACCCCAGCAAACTACTCTTTTGCTTTCTATAAAGCAGAGCAAGGTGCTTATGGTCTAAAAACATATTAGCAGCCAA
AATTCAGAAAGTTATAGTACAAAAAAAAAGTTTGGCTACACAAGTTCTGGAATATCAATGTTCTTAAGTCTAGAAGTTTGACTACACTGATTCTAAAGCA
TCCAATAAAATGGAACGGAGGGAGTAGTAGACAATCATCATGCGGCGTCTAGTTCTTTGTAAGAACCAAGGTATGTGGATTTGGCTTCAAATTTGCGCTT
CCTAAATTCAATTTAAAAATGTTTGTGTTCCAGTTGAAGCACACCACGCGAACGCAGAAATTATGATGTATTTCGTTACCTATAATTTGGAAAACATGAC
ATGATATAGATTAGGTAATAATGAACAAATCCTCAGCTGCTCAGAAAGTTACAAGGGGATAAAGGCCCATAAAAGAGAATTCCAGGACCAGAGCTGCCAC
AAAGATAATCAACCCAACAATTTTTCGACAGCACTCATGGTAATACAAAGGTAAAAAGGGACGTGTCTTCAACAATAGCTTGACGAAACCACTGAAGCAA
TAATAATACGACTTCGGACGAGCTATGTGCGTTGGTGTCTTACTTGTGACTTCCTTGCAAGTAATGC
>comp76332_c0_seq48

ACTGCTCTGAATTAAGAGGCGTTGAAATATTGTCCGGTCTACATATAGGCACTTGAGGTGTATTGACAGACAGTTCCCCTTAGTTTGCTACAACACATCA
GGCGAATTCCACCAAATTAGGATGATGTTTGTTTCTTAGACACAGGATAGCAATGCCAAAAGTCTTTTATAATTTTCCGCTTCACCGCAGATTTGGCATC
TCAAAGGGAGTTGTGGCAAGATTCTCTTGACCAAGCTTTCTTGCAGATTTGGCAAGAAAGATAAAAAAAATGTTGGTATACTTAAGCATCCTTAATAGAA
ACTAAACAGGTCTTAGATTGAAATTATCATGAATAAACCCTTTTTCACTTTAGCTGTAGTCCGGATGAAATCTTAGGGGCCTTGCTTAGCCTGCCCTGAA
GCTTGCCAGTAAGTCGAAGCTGATGCTGACACAGCACTGTTTCAAGTTCTCATCTTCTGAAAATGATCAGCAGTCAATAGATGTTTTAGTCCATTCTCCA
GAAAAATGAGTGCCGCATGAATCCTTTCCGGTTTATAGTGAAACATTTTAGACGATGACCTTTAACATTCTAGAAAACGAAGAGAAAACCTACAGTCATC
TAATTTCAGCTTAAACATGTTCCAGCAAGATCATCACCATCCTGACATGTTCCGTTTTGTCCCTAGACCCGAGCACAAGGTATTGCGAGGCTAATTTAGC
GGGGCAATTGATTTTTTGCCACTATGATACAACTGCAATTACCCGCTGTCACTGACACGTGGGGTCATCTGAGTCACTGAAATGTGGGCCCAATAGCATT
TTGTTTGCCACTCACCCTTCCAGAGTGGCAAAAAGTTAATTGTCCCTAATTTAGCTCAATTCAGTTGTCAGAGCAATTGTGTGTGAAAAGTGTGAACAAC
AGCATAGGAGACAATACAGCAAGTGTAGCGTGCCAATATGTGTCCAGCTTGATAGTTCACTATAATAAAGTTCTTTAATTCAAGTATTTGAATAATATAG
AATCTCAACCTTCAAGTTTAAAAGGTTTTGTATTTGCTATTATAATCGTTGCCACCCCCTCACCCCTCTCAAAAAGAGAGTTACTTTGTAGCGCACTGTA
AATCTCTTGATTGAAATAGTGGCATGTCATCTTATTTAATTCAGTTATTTCATAAGGTACAATTTGTTTAGCTGACAGCTCAGACATGCTTCTATGCAAC
ATGTTTATACCAATGCTGTATTTTGGCAATCCTGGATGTGGCATTACTTAACTGTGCTTTCATATTCAATAGTTCTGGCAAAGGGAGGCTGCAAGCTTGA
GACAACAACTGCACAACCTGCAAGAAAGTCATCGGTATGGCTAGAAAACCCTGCAAATTTTACAAATGTACAAGTCAGAAAATTTGCCTAATGTAAAAGG
ATCAAACTACAGGATTTGTCAGCATGAGCACAAGTGCCTAACTTCCGAGTTAGATAACTCTGGAGGAAGGAAATCCTTCCTACTGTTTGTTTCAAGAATA
TACAAAAATTCAAAACAACATTAGAATAGAACCAAAAGAAGAAACAATATTCACCATGTAGCAATACTCAATTCCTGGTTGTCTTATTTATAAAGGTATA
AAACTAGGTTCTGACGAGACAATTATGAATCAACTACACCATGTTACAGATCATCCATAACTCTTGGTGATGCAAATTAAGGGCTGTAAAGTGACAAGTA
TACCTCCACCAACTAATTTGTGCAGAGTGTATAAAATTGTAAAACAATGGATAAGAATGAATTTAGTATACAAGATAGCATACAATGCCCAAGTTTAAGG
AGACATACTGATTTACTGACTCCATAGGACAATCTTTTGCTTATGAGGACATCAGTTTTCATAAACAATATCAATGTAAAAGGAGGTAACAGTTTATGAA
TTTCCTATGCCAAGCCATTTATAAGTCAAAAAATGTACCTATTTAGCTTTTATGCATTCCAATTAACTTCCAAAGAAGAAGAGTACATACTAATGGAAAA
CAAATATGATATTATCATCTGTTTTGCCCGGTAGTTTCTTGAGTCTCTGATTTGTCCGCACTTCACTCCCAACCAACACAACATTAATACACATGGGCAG
AGGAATATCAGGTTATCAGGTAAACTGAATACAGTTTGATTGACTGCAGTCAGTTGATGGGACAAGATCTTTCTGGATTGAGCGTCAAGGAACTCCAAAA
TCTAGAAAATCAGCTAGAAATGAGCCTACGTTGCATCCGGACAAAAAAGGTAAGGATGCTAGCCATATTTCTGTTTTTCGTTACCAGATTGCCTCATCCA
AGAATACAACTGATGATGACGTTTTTTACTTTCAGGACCAACTCTTGATTGATGAAATTCACGAACTGAATCGAAAGGTTCTGGAATATTCTGTTATTAC
TCTGCAATGAGAGGTCTCTGAGTGGATTTCAAGAAGCTGACTCTACTGGCTCATTTTCAGGGAAGTCTCATGCAACAAGACAAGATGGAACTATACAAAA
AGGTCAACCTAATCCGTCAGGAAAACATTGATTTATACAAGAAGGTATTTGATAAATCGTGAATGAAAATTCACAGGATATACACCCCATCCATTATTGA
CTGAGGTTTATTTCTCTACTCAGCTCTACGAGAAAGAGGTGACAAGTGAAGTCAACCGAGATTCATCAACTCTAAACTTTGCAGTTGTCGAGAACGTCAA
CATTCCTGTTCATCTTGAACTTAACACTCCACCGCAAGAAAATGATGTTGAGCAAACTGCACCTCCTAAACTGGGGTAACCCTGCTTTTTTCTGCAAAGG
AACTATTGATTCTGTTCTTAATTCTTTAGTGGCATATTGTTTACTACTAACTGAAAACTTGCAGATTACAACTAAATCCATGAAGGCATGCAGCATGGCA
CCGCTTATGTTCATTATTCTCAACGGTACGATTCAAAGCAACCAGAAACACTATTGGTTGTAAAGAGCTTAAACACATAGCAAGACTGACGCGGTATGCA
TGCCATGAGTGATTGATAATGGTAGTGTAAATCTATTTCAAATAAAATGTATTTTATTGAAGAATACAGCAAATGATTTGTATTATAACTATTTGACACA
TTCTGCAAGTTTGTTGACCAACGTGAGTACATGATAGCTACAAATCTTATATTGACAAAAAGATATGGTGTGTGGAAAAAATTCAGGTGCTATTTTTTTC
TATTTTGAGAATTGTATGCATAGCAGAAGAGCACAAATTGCATTCTGGTTTATCCGTGCAGGCATTCCAGAGTATCAGAATGGGGATCAACCTTTCTTCA
GCTACATATGTAAATATACGGATGCTAGTTGCTCACTTAAAATTTTGTAGTGTTCAAAGGAATAACAACAGTAAGACGTTGACGCACCGAAAATAACCAG
AAATAACGGATTGTACTATGACAAGACACACGACAGATACACTAGCTTAAGTTTGTACAGGAAAATATTTGGCCTATTCTTATGTCACAGTAAATTAAGA
TTATCACCTTAATCACAGAAATTCAAGGGGTAAACGATTATAGCACAAAACGATCCTGGACCCCAAGACCTGGGTCTCTGAACGGCTGAACCTCCAATTA
TACCTCTATACATTTGGAATGTGTGTAGTCAAACTTTCGCTACTGGTATCTTTACAAATATTTACATTGAACACATCAAAATGATATACATGGATTCCTC
TCGATATGTCTTTGAGGTTTGGGTTCTAAAAATGATATACAAATTATTAGGTTCTAAACTTAAATGATATATACACATCAAAATGATATACAAATTATTG
GGTTCTAAACTTAAATGATGTATACACATCG
>Locus_20324_Transcript_18/30_Confidence_0.311_Length_1307
CCTCCATTTGTTGATGGGGTTTTTTTACTGCGGGTACCGGAGCTCGCAGTACAGGGGCGTCACCTTCTACAGGAGGACCGGCCGCTGGGAGTCGCACAT
CTGGGACTGCGGGAAGCAGGTCTACCTGGGTGGATTTGATACAGCGCACGCTGCGGCAAGGGCCTATGATCGCGCTGCGATCAAGTTCAGGGGCCTTGAT
GCGGACATCAACTTCCAGTTGAAGGACTATGAGGACGACTGGAAGCAGATGAGGAAATGGTCCAAGGAGGAGTTTGTTCACATACTCCGACGTCAAAGCA
CTGGGTTTACAAGGGGGAGCTCCATGTACCGCGGTGTGACACTGCACAAGTGCGGCCGGTGGGAAGCTCGGATGGGTCAGCTTCTTGGAAAGAAGTACAT
CTACCTTGGGTTGTTTGACAGTGAAGTTGAAGCTGCAAGAGCATATGACAGGGCAACCATTCGCTTCAATGGAAGGGATGCTGTTACTAATTTTGATCCT
AGTTCCTATGATGGAGATGTTCCACCTGAAATTGAGAAAGATGTGGTTGATGTTGATGGGGACATCCTCGATTTAAATTTGAGGATCTCACAACCTAACA
TGCATGATTCCAAAAGTGATGGCGTCCTCACTGGGGGATTAAATTGTGATTCCCCTGAAGCTTCAAGTTCGGTTGTTTCTCAGCCAATAAGCCCTCAGTG
GCCTGTACATCACCAGAGCACATCGACTGCACCCCAGCATCCACATTTGTATGCATCTCCTTGTCCGGGCTTCTCTGTGAACCTCAGGGAAGTGCCTATG
GAGAAACGACCCGAGTTGTGTCCCCAGTCGTTCCCTCCGTGTGCATGGCAAAAGCAGGGTTCCCCTGCGCCATCACTCCCTACTGCAGCATCATCAGGAT
TCTCTACTGCCACCGGCGGCGCGGACCCGGCCAGCGTGCCGCCGTCCCGCCCCCGGCCCTTCGCCACCGGCCACCAGCTCCACTTCCCCCCAACCGTCTG
ACAGCCTCCCCCTGTTCAACTGGCGACGATAACGCGGTCGTCTGGGCGCCCCATGCTTCGCTGCTGGTCGGCAACAGTCGCAATTGGTTCGGGTAGAGAG
AGATGGAGGCTCCAGCACACGACCTCTCTGTCTCTCTCCCCACCTGGTAATTATCATCAGAACCACCATGCTTATGATTCTTTTATCTTCATTTTGAGGG
CGGATCCACGGATCATGAGCGTGTACCTCCAAAATTCTAAAATAAACCATTTACGATAACTCTGGCTGAGGCTCTGTTTGTTTGGGCTTCTGGCCAACTT
TGAACGG
>comp81084_c0_seq9
AAATGAACAGTTGTTCCGAACTCTAACCGTGTTAAAACTGGAAATCCAAAAACTGGATGCATGTCTTAAAATCTAAAAGCGGGATGCATGTCTGTAATAA
CATTCCATGAAGCAATTTCAAAGCTTAAACCCTTTTAATCATGGAAGCCAAAATCTATTTTCCAGGGATTTCACCAGTTAACAGACCAGAGCAGAACTGT
GCTCTAATTTATTGGTCACCTGTACTTGCGTTTTTTAATGCACCAAGGTTCCTTTTTAGAGACTTGACAGCAGACAAAATCTGCAGCTTCTCTGCGGACG
GCCCGTCAGGTTTCTGATCATCGTTCCCAGTCGCAGCATCATCAGGTGGCAACGAGTCTCCCAAAGATGATCCCTCCACATGAGGTTCCCTGTCCAGCTG
GCCTGCATGTGCCCCTTGCTTCCCAGCTTGCTTTCGCATGAAGGCCGCACTCCTCTTGTACTGGGGATCAGTAGGATCCAATGCGAACAAATGTGACGTG
AACATTGCTGAGAACCTCTCGTCCTTGCTAAGATCGATATCAGGAAGTTTGTCCTCAACCGAGTCCTCTTTATCCTTCTTGCCCTTTTTACCCTTTTTGC
GCTTCAAATTATAACCCTTTGCAACATTGGCAGTATCCTGGTCAGCAGCAACCAAGAGCTCCAACTCTTCTTTGGTTGCTTCTTGCTCCAAATGTTCCTC
TGGGAATTTGTCCTTTCTTTTCCCCTTTGCTTTATCTTTGGTCTTTGCCTTTTGCTTCTTGCTTTTGCTTGGCCTAGTTTCTTCATCAGAAAATTCCTCA
GCGAAGAAGTCATCATCCTCATCAGGCTCATCTTCACTGCTATAGTCATCATCATGCTCCTTCAACCCCCTCTTCCTAGCTTTCCTTTTTTCTTTCATTT
TCTCTTGGTGCTTCTCCCAGACAGTTTTCTCCTCATTGGTCTTTCTCTCTAGGATACGTTTACTGAGATCCTCAAGCTCTGTGTTGAATGTAATCTCCAT
ATCGTGGCCATCAGTTTCTTCATCATCAGATTTGTCACCTTGCAGTAAAAGAGCCAACCTCTCCTCTTTTGTTAGCTTTCGTTTGGATCCCCCATTTGGT
AGAGACTCATTATCAGGATTGTCTACACCGTCATCATCTGACGCACTGTCATCACTTGCTAAATACATGTCAAGCTCATCTAACTGATCATCAGTGAACT
TTCGCCTCAAGACCTTCTTACGTTCTGGTTCATCATCATCCCAAGTTAGCTTAACTCTGCTATGTTGCAGAGCACGAGTCTCAAAATCAGGCTCCTTGTA
ACTCGGAGGTGCCTCTGTAGCAATGTCTCGAGCAGGATGTTTGAACTCCATAGAATCAGGTATAAACTGCAAATCAAATACATTTGCTGTTTTCAAGAAC
TCAGTACCATCAAGATTCATGTACAGGTGATTTGCTGTTGCACTAGAATCACAGACCACAACAGCATAGTAATACCTCAGTCTGTTTAGCTCGTAAGTAC
GCAGCTTGTTATTCTCTTTGTCAGAGTCAACTTCTTCTTCGTCGTCATCAGTGTTGAATTCATCATCATCAGTGTCGTCTTCACCATCGTCGTTATCATC
ATCATCGTTGTCCTCATCATTATCATCAGTATTGGCATTAACAAGAGCTGCTGGGCCCTGAGTTGATTCAATTTCCATGCACTTCAGTCCAAATTCTGAT
GGATATATTGAGACTGATAAAACTCGCCCACCTTTTGGGACGCAAGATGTCATCACCATATACAGGTCAACTGCCTTGATATGATCCCAGTCCATATTAA
CAACAGCAAGCCTGTGAGTTTCCTTGTCAATCATAGGCGTATCATCGTGCCGGCCCATTAAGTAATGAGCAATGTCACTGCCGACAGAGTACTCGTCATC
ATCAACGTCTTCATCCTCGTCGTCGTCGCTACTAGAACTCTCTTCTTCCTCCTGCTCATCCTCTTCATCCACGTCCTCGTCCTCCTCTTCTTCAACTAAC
TTCTCTTTCTCCTTCTTTGCCTTCTCTTTAGTCTCATCCTCCTCCTCGTCTTGGTTGAGATAGTAACGGAGCATGGGGTTCTCCCTGCCGCTCTTCTTCC
GTCGCCTGCCGCGCTTGTCCACCGGCGCCGCCGAGGAGGAGAACCTCGGGTCCGTCATCATGCTGCTGAACCTCGAGTCCAGCGCCACCTTCGCCTCCTT
CCGCCGCATCGACCGGAACCGCGGGTCCATCCGCGCGGCCGCGAACCGCTCGTCCTGCATCGCCCGCTTCATCCTCTCGTCCCGCCCCTCCACCGCCTCC

GCTTCAGCCGCCTTGCCCTCGTGCTTCCCCTCCTTCTTGGGCTTCTTGCCGTGCTGCCGCTTCTCGTCCTCCTCGCGCCCCTCCTTGTGCTTCCGCTTCT
TGCGCTCGGCCTCGGGGCGAGGGGCTTCCTCGGTGGCCGCCTGACGCTCTTGATTCTTCTTCTTGTGCTTCCTCTCTTCCTTATCGGACCTCGATTTCTT
GGCCTTCTTGAGGCGCGCGGGCTCGTCGGAGCCGGTCGGCGG
>comp63492_c0_seq19
GTGGTCAAATCCCTGCCTGTCTGTTAAACGGGAAAGGATTTGGTGGATTGATTATTTACACCTGGGCAATGTACTAATATGTAAGCCTTGCAAGCTTAAC
AATTAGTAGACGGTGTTCTGAAGTAACATTGACCTTAGACTACAACAAAGATTATGTTCGCACATATATACATGAGCGCTCTATATACATGACACAAGAC
GAAGCAATTTTCCTCAACAGTATTAGCAAATTTTCTGTCCACACCGTGCCTAGTAACTCCATACAAGTTAAAGCGTCCTCAAAACAACAAGCTTGATTAT
GGAAAGCTTCTGAATTACCAGGTTAGCGAAATTCAAAACTCCAGAACCTTTCTGCCTGACCATGTGCCATGTAAAGGGGAACACCGTCCAAGCAATCAGG
AAGGAAATACAGTAGTTGGAGCACCCATCTTAAAAGTTGGAGCACCCATCTTAAATTCTCCATGGCCAAAGCCATAACACAGCACTTGAAAACCATTATC
GTTGTAATGAAGCCAGGCCAAAGCCATGCCAAGTCATCCAAAGGTCATCACGGCAGTTTTTCTGCAAGATCAGACTGCACCCTTTTACTCTGAAATAGAT
ACCATATACCACAAATCAAAGGTTGTCATGATGAATGTTACTCAAATCTATTGACAACCTTGGCTTCCGTACTTTAGAGCTCGACATAGTAATTCACACC
TGCTATGGCAACTGGCAAGTTTAAAGGAGATGGCAAAGTGGCCCTTCAAATCATACAGTGATGGATTTGTAACGAATTCAACTGACACTGTTCACTTGCG
CAAGAAAAAAGGGCAGTAAAATATTAAAAGCTGGAAAGAGCCAGAACCAAAAAAATGTAACTGATATTAGCAAGCTAGAAAGAGCCAGAACCATAAAAAT
GTAACTGATATTAGTAAGTATGATACTGATGTTATGTCTAACTGGAGTATGTATGCTATGGATAGACTAGCTCCTTAAAATGTCCTTCCACGTGGATACT
GTTTACCGAAGATTTTTCTTTTTGTAAATATGACGCAAGGCCAAATCATTACCTATAAGGCGTCATGCCGGAGGAACATCATTTTATTGGGCAATATTAT
GCTTTGAAGAAATGAAGATGATGAAACAGGATCCCAATGTAAAGGACTATATCGCAACAGGATCAAAATGTAATATCACACATGCACATTGATAATGCAA
CTGAACAGGCATTATCTGAAAACAGGAAAACCAAACTGAATTTTCCTTGAAATCACATTGGGGACTGTAAATGTTAGAATATGACACTACAGATATTGTG
CCAACTTATTGATTGACCACAACCCCCTGAAGTTAGCTAAGATTGTATGGACATGTATATGTAGTTTCTGTTTTAAAATAGTTAACGTTATCCCCAAAG
CTGAAGATGTTGCAGTTATTGGGCATAGATTAACTTTAAGCAATGTATTAGTATACTACGCCTTACATTCTACATTCTTTTTCTAAAATTACTTTTTGT
AGCTCAAAAACTGACAATGTTTCTTGTTTTACTGACAGCTCTGGCAAAGGGAGGCAGCAAGCTTGAGGCAACAACTGCACAACTTGCAAGAAAGCCACAA
GTATGATCTTCCAATTGATTCTGCAATAGAGAGAGATCACTCATCAATACACAGTTTATATGACACATGGAAAGATTTACTAGCATGAGAATTGTCAAAA
ACCAAAATGAAATGTTTCAATCAGCCAGGATGAGGGGAAAATCTATTTACTGAAAGACATTTAAACTATTCAGTATTCAGGAAAGGAAATTGATAGTGGC
TCTCTCCCAACAACATATAAGTGGTTTTGCTTTGACCTGTGTACCCACGAGATTTATATTCGGTTAGTTTCAATGTTGTACACAAGATCTAACGTGATAA
ACTCTTTTGCATTGTTGTAGGCAACTAATGGGTGAGAAGCTTTCTGGCCTAGGTGTGAGAGACCTCCAGAGTTTAGAGAATCGTCTTGAAATGAGCCTAC
GTAGTATCAGAACGAGGAAGGTCATATTCATTTTGTTTCCACGTTTTGTTTTTCTATTCACTCATCTTAATATGCATAAACATCCATCAAACAGGACAAT
CTTTTGAAAAGTGAAATTGAAGAGTTGCACAGGAAGGTAAAGCCTTACAATCCATGTCTTTTTATTATCTCAAACAAATGACCATGCATGGAGGTTGCTG
ACATCTTAACTTAATGCTGATTGAAACAGGGTAGCCTAATTCACCAGGAAAACTTGGAACTTTGTGACTTCCTTGCAAGTAATGCGGTTCCTGCCTCATG
CTTGGTGCCGCCGCATGCAATAAACCGATAACTCGAATCCTCCGTGACTTCCACCCTCTCCAGCCAATGAGCGGCTTGCTGCGTGCGGGCGCTGGTGTGC
GGGTGGCGCCGGCTTTATATTAGGGGCGGCGAAGAGTCTAGAAGGATCGAATCGACGGGTCTAGAAGCCCAGCTCCAAGTGGAGATAGGGAAAGCGAGGA
AGCGATGCAACGCGATTGGCTTTTGGGCGAAGGAGAGAGAGGAGGGAACGGCTTAGCGGGTGATTAAGGTTGAGCGGATTTAATTAGTGGATTGTTAATT
GCGGCTTGCGG
>comp74035_c0_seq4
AGGCATACAGGGAGCCAGGCATGCGCAATTCGGTCCATCCTTCTCGGATCTCCACATCGGCAACCTGCAGCAAAGCCTGCTGTTCTCCGGCATCCTCTTC
CCTGACCACCACGCCCCTCACCCTCCGAGGATCAGCACCGACCTGACAATTGGCAGCCCACACGCTGGCCAAGACGCGGCGTGCTCCCTATCGTCTGGCG
CCAAGAAGCCTGACGACGTCAAGGCGACGAGACTAGTGCTCTTTGGACGGGCGATACTGACGGAGGAGCAGATCAAGGCCAACGCTTCCGTTGGCCCGTC
CTCGCTGGGAGCCACCGGGAACGGCTCGTCCAAATCCGACTGCGACGCCGAGAAAGCACCTACCAACACGTCCGAAGGCTCCAGCTCAGGTGTCATCCAG
GGCAGTCCTACCAAAAACAGCTCGTCCTCCTGGAGACTGCAGTGGTTTGGCGACAATGGCAGTCAGTCACCATCTGAGTTCGGGCTGGAACCTGGCCAGT
GCAAGGTGTTCGTGGAGTCGGACGCCATAGGCCGGAACCTTGATCTCTCGGCGCTGGGATCGTGCGAGGAGCTGTACGCCCCGCTTGTCTGACATGTTTTG
CGTTGACAACGCTGAGCTGAGGAGCCATATGCTATACCGCACCGCCGCTGGCGAGGTCAAGCACGTCGGCGATGAGCCTTTCAGTGTGTTTGTGAAATCG
GCGCGGAGGATCATAATACTGACAGATGCGGGCAGCGACAACATAGGGAGTTAGGAACGAAGCGATCAGCCAAATAACTTAGGCACTACTCCTAGGCAGT
AATAAGCCGCCTACATGCATGTACAGCCGAACGAGATGCGGTTTCTTCTTCAATCAGGCCTAGAGAGTTTAGTCGGGAAATTAAGCTGACGATGTGTTCA
TTGCTTCAGTTGAGTTCTTTTAGTCATGCGAACAAAATGTAATCTTGATAATCAAACGCACTTGTTAATGTTGTTTCCGTTTTGGATTTCAAACCTTGAG
CCGGGCCACAGAGACAAGAGCAGCAGGTGAAAAGGGTTAAATGCCATTGCTGG
>Locus_1826_Transcript_119/132_Confidence_0.505_Length_7004
CCGCTTCGCCCCCCCACCGCATCCACCGCTGCAGGAGTTAGGGTTCCGGCGCCGCGCGCCGGGGTATGAGCAGCACCCGCCGCCCCGCAGGATCTAGGG
TTCCGACGACTCTGCTCACGGATGGAGGGTGGTAGAAGGGCAAGCGAATCCCCCTCGGCATACACCAATGGATCATGATGATACTGATTTTCAGAGCCAA
AACTTTCAACTAGCTGGTGAAGACAATAGCAAATTCCCATCGGGTTTGCGGCCATTTGCACTGCCAAAACTTGATATTGATGACCAACTACAAGGCCACC
TTCAATTTGATAGATTGATAGATTCAGAAGTATTTTTCAGCGTACAAGGGCACGAGAGTAGTTGGGTTGAGGTTTTGTCCACTGGAAGTAGTGTTGTTGA
TTTTAGTTCCAGTGCTGCTGAATCTTGCTCCATATCTAAGACTAATAATGTCTGGTCAGAGGCAACATCCACCGAATCCGTGGAAATGTTATTGAAATCA
GTGGGAGCAAATGAGATGACTGGTAACATGGAAGGTGATGCACATCTTCAGTTAAGTGGTATGGACAGTCAAATGGATGCATCCAACGTGCAGCCTAAAT
CCAGTAACTCTCCAACAGATAGCACTGTAGTGCCAACTGAGAATGATCAGTCACAGAGGACTCATTCTAGAATGATGGATGATCCTGTTCGTTCTACGAA
CACCAATTCCAGAATGTCTGACAATCCTTCAAGCACTCAACCACAGCTTGAACATTTTGCATCTTTCTTGATTGTCGAGAAAACTGAAAAGGCAGCAGGG
TCAATTTTATCAGATAGGAACTCCAATTTTGTGTTGGAGTCTGTTGCTGAGAGGTGTATTGTAAGTGAGAAGTTGTCTTCTTCCACATCAGAAAGCTGCC
CAGATGTTGGTGACCATTTCAAGGCTGTCCATGATGACCATTCTTTGGACAAACTGAGAGTACCCTCAGCTGAAGTAGATTCTAGGAAGATGAATAATGA
ACCTTTCCCAGAATTAGCTCCACTTCAGAACATTTATGACTCGTATCACTTTGAGCAAGATAATCAAGGATCTGAGGTTGGTATTACTCCTCAGGATTCA
AAGATTAGTCATATCAGTGAAAATAAAGTCGAAGGAGGACTACATGAATTGCAAAATTTGTCATGTGCTGGTCAGCCCTTTGGGTGCTGTAAATTTATCCA
GTCAAGTAAGCAATGAAACTCTATTGTCAGAAAGTTCTGACGGATTGCTAGAGGCCATTACGAATCCGGTTAAGATGATGCACATGAATGATGATACTTG
TAAGAGAGCCAGTGGCACTCTGCAACCATCTTTTCCACCAATACAACATGCAGCAGAAGGCTTGAAAAGTTCAGTTGGCATGAACAATGAACTTGTAAAG
GAGTTTGTTATTGGTTCCAACTCTGCTCTATCTAACCAATCTGAGGCAGACACGAGAAATTCTAGTCCTCATCTTGTTACTTCTGTGTCAACCGAAAGAA
GCAAGATGATTCAATCTCCCCAAAGAAAGCTTGCTCACGCCACTGGTGTTCCTGAAGAAACAAAAAATGCTTGGGCCGATAATACAAACATCTTCAGTGG
TGATGAATCAAAACTTGGAGCGTTGGAGCACCATCAAGATTCTCTCGACAACCTAAAAAGTGATGCCATGGAAGAAAAGACAACCAGGGAGAAAATATCA
GCAGTTTCAGGAAATATTGATCAAATGGTGGAAAGTGGTCATGACGAAAATGCTACATGTGCTACTGGTACATCAAGAGATAAGTTTGACTCTTCGGACA
ATGCTGCACCTGACAAACTTTCAGATGACACTTTGGATAATTCAGAGGATCAGAACATTCCCTTAGTAAAGCATGAGGGGTCATTCAAGGAAGGTGACAT
GCCTGCTTCAGTAGAAGAGCCTGAAAACACGGTCCTACCTGCTTCTGGGCCTCAAGAGAAAATGTCAGCACCATTGGTCAATTCAAGAAGTGATATCGTT
TCGACCACTGTTACTGGCACTTCCGGTAATTCAGAGGATAAAAATGATTGTTCAGGTGTTTTTCCTGATGATTCTTCTGCTGCTTTGCCTGATGAGAAGG
ATTTGAAAACGTCCACAATGGATTATGAGAGGTCATTCAAGGAAGTTGCTAAACCTGCTTTAGAAGATGAAGACCACAATGTTATTTCTCCTGGCTCCGA
ACCAGTTGGAGAACTATCAGCAGTTTCAGGGAACTCAGACATAGATGCTATTTGTAGTGGTACTGTTTCTGCTGCGCAAAAGGCGGAATACAAGGAGCAG
GCTAATTCTTTGGGTGGTTTGACCACAGGAGAAACTGAGGATAAATCAGGCAACCATCCCGATGCTTATTCACAGAAATGCCAGACTGACGAATCTTTGG
TACAATCTGAACATCGTGTAGATCCAGCCACACCATCTGCATTAGGCATCTCAACTGACAAGGCTGTTGAAAAGATCGTAGAAACTCCACTGAATGCAAA
GGATGATTTGAATGCACATGTGCAAGATACAGTTTCAAGTCATTGTACAGATCATAGTCCTGGTACTGTTCCATCCCAAGGCAAGCTAGGCTCAAGCTTA
TTAGAACCTGGGAACAGTGACGGAATCTGCTCTGATGCTACACATGATTCCTCTCCAGTGATTAGTTGCACTGAGCCCTCTCCCCAAGGTGGACATGGCA
GCAACACTATGGTTCATCAGACACTAGATGAACAATCTGAGGATCCAAAAGATCGTGAAGCCTCAGCTGACGCTACGCAGAGCTCAAAACAGTGTTCTAC
TGGACATGTAAATCCTACTCCTGGTTCTGAGCAGACTAATTCAGCGGGAGACGATAAAAGTTTCTCATTTGAAGTGGGAGCTCAACCACATGTATCTGAG
AAAGCTCATAGCCCTGCTTGGAGCCCTTTCCCCAGATACAGAGCTTCTCAGAGTACTGAGGTAACCACAGAAAAACCTCAGGCTGGAAGTTCATTGGAGA
ATAGTGGTGATGATAGTAAAAGAACATCTATTGTGAAGGCTGGTAAAGAACAACTGTCAGAAAGGAAAGTGAATGGAAGTGCCGGGGGTCCCTCAGACAA
CTCTAATATTAGCGATAACACTAAATTTTCACCGCCGGAGCAGTCACAACAGCATCCAACCCCTGAGCTGTCTGATGTGGCGAATCTACCTTTCACAGAT
GTGCAGCATTTGCAGCTACGTGCAGATATTTGTTTACGGAGCTCTTATCCAAGGAACACCACCAGGAGAGGCTTACATGGTGGCAGCTTTTGCAGAACCT
GGTGGTGGTAAGCCTACATGGGAGGTAGCTTGGCGAACTGCTCTTGAAAGATTTCAGCATCAGAAACCAATTTTTCCCGGCTTAGAGACCCCCACAAGCT

CACGTATAGCTGAGTCTGAAAAAGCTAGTAAGGGCACAACAGTTAGAACCGCGACAGCTAGCAAAAAAGGTGGGAAAACAGTGTTGCCAGCACATTCTGC
TGTAGCCTTGCACTCGCCTACTTTCAATGCGCCTCTTGGTAGTTCCACATTCAACCTGCAACGAGGTACTCATCTGGATTTTAGCCAGGCTGTCTCACCA
GTATTTACATATAATTCACACATGAGGCAGCCTTCTACTGGTGTCGCATCGTGGTATTCTCAGAGCCCTGGTCCACGTTCTGCACCCTGGCTGGTTCCAC
CACAAAACTTAATATTTGATTCATCTATGCAACCGGCTGTGCCTACAAATGAAACTGCAAAAGGATCTTCTTCCAAAAATATATCAATTTCACATGCTGT
TTCACCTGGGGTGTCCCTTCCAAGTCCAGCACCTTCTATTGTTTCTCCTCCGTTGGCAGTTGTACATGAGGAAAAAAAGAAGGCACCGGTTTCTACTTCT
AAGCATGGAACAACATCGCAAAAGCCAAGAAAAAGAAAGAAAGCTTCAGCAAGTCCTGAACAGCAAGCTGTCATCCCTTCTCCTCAGCTCAAAACAGACA
CGGCGTTGTTTACTCCTGCTGCTAAGCATACAGGAGGCTTCACCTTATCTACTCATTCCCCAAGCAATGCCTTGGGCAGCAGGCTTATTTCTAACACAGG
TCAGATCACCTCTGTGCCTAACTACCAGATTACTGGTACTATGGATGCTGAGCAAAGAATCATCTTTTCAGAACAGATCCGTGGTGCAATAGAACAATCA
ACAGGACAAGCCAAAGGTGCAAGTATGCACTCTATGGCAGCAGTTAGACACAAAGAAAGCATATGGAGCCACCTATCAACAATCTCAAGAAACAAGTTAC
CTCCAGAAGTTGAAGGGAAGCTTACCTCAGCTGCAGCTGCTGCTGAAGCAGCAGTTTCTGTTGCAAAGGCAGCTGCAGAAGCTGCTAAGATGGCGTCAGA
GGCTGCCTTGCAGGCGAAAATGATGGCAGAGGAAGCTCTTAGCTCCTCTAAATCTGTAATTTCCTTGCAGAGTCATGAAGCTGGTGAGGTTAATGTCAAT
AGCAATCCACCTAGCTTGTCAAGTTCAACACCCACATCATCTTGGAAAATTAAGGACAACAGTCATGCCCAAGGTTCCATCGTTTCTGTGGCACGAGAGG
TTGCTAGAAAGAGGGTTGAAGAGGCATCTGCAGCTACAAAACGTGCAGAAAACTTGGATGCCATACTGAAAGCTGCTGAGCTTGCTGCCGAGGCCGTGTT
CAATGCAGGGACCATTATTGGAATGGGTGAACCTCTACCATTTACTCTAAGCGATCTTTTGGCAGCTGGCCCGGATGGCTACTGGAAGTCTGAGCGAGTT
AGGAATAAGAAAGCTGGAAGTGTCAATGATAATCCGGTAACAGAAACATTGGAGGTAAATGTGCCTGCTGATCTAAAAAAATCTGGCCGAAAGCGTGGCC
GGAAACCTAAAGATCAAGCACTACTGAATGCAGAGCCATCTTCAAGTGGCAAAGAATTGCAGCCAGATGGAATGCACACAGATGGGGTCAAAGATATTCC
CACCACAACATCGCTTGGTGCTAAAAGTAATGATACAGAACCAATAAACATTATCTGGAATGGTATTGGAAAGGGATCTGCTGTTGAGGTTTTATCTGAC
AAGGGTGGGTCTGGAGTAGCTTGGTTTTCTGCTAAGGTTGTTGATATAAATGAACATAGTGCGTTCGTCAGCTATTACTACCACAATGAAGGAACTGGTC
CTCGCGAAGAATGGGTGCCATTGAAACAAGAGGGCGAAAAGGCGCCTCAAATACGCCTTGCTCATCCTGCTACCCTCTCAAAAGTCAAAACTAGGAAGCG
TCGTAGGGAAACTGCTGGAAATGGTTCCTGGGTCATTGGAGATCATGTAGATGCTTGGGTTAAAGATAGTTGGCAAGAGGGAATTATTGCTCAGAATTGT
GAAGCTGATGGAACAAAATTTGTTGTGCAATTTTCTGGAGGTGCTGCTGATTCCTTGGTTGTTGATGCTTGGAGTCTTCGTCCATCACTTGTTTGGAAAG
ATGGCCAGTGGGCAGAGTGGTTCCAAGCGAGAGAAAGGAAATCTAAATATAATAAGGGTGATTCACCTCTTGAGAAACGCCGGAGGACAGGCCTACAACA
AGCAGGTGGTGATCTACCTATCGGTGGTGAAGCAGGAGGTCCTTCTAATGACAAGAGCACCAACAATGCAAAAAAGCCAGAGGAGCCACAGCCACTGGCT
TTGTCTCAGGCAGACATGATTTTCAACATTGGGAAGAGTGTAGTTGAGAATAAAACTGATGCTCTTGCATTCAAGCGGCCTGGATTACAGAAAGAAGGAT
CAAAGGTTTTCTACGGTGTTCCAAAACATGGAAAGAAGAAGAAGTTTATGGAAGTAAGCAAACATTATGTTGCAGGCCAAGCTGACAAGATTTCTGAGGG
TAATGCACCTAACAGATTTGCGAAACATGTGGTGCCACAATTATCAAGACCACGGGACAATACTTCCAAAGTTGATCATAGAGGAAGGAGGGTAGGCGAG
ACGAGGTCCAGAATACCTAAACCCTCAAAGTCTCAGAATGTGGCAGCTAACAGTGTTCCTGACAAGGATCCTTTACCCTTGTCTCTCCCAAATTCTGGTG
TTTCTGAAAGGAGTTTTGCTTTTGTGGGAAGTACGGCAAGTGCTTCCAACACTGAGAAGCCCACTGTAGAAAAAAATAAAGCCGCGCTTGCTGCTGCCCT
TAGAACTGAAGATCCTTCTGTTTCTGAAATGCAAGTTGCATCAACTGTTCCTAGTTCCAAGCAGAATGTACCCACTACTAATCGAGCTAAAAGGAAATAT
GTTCCTACTGTGGATAACTTGAACAGAAGTACCATAAAAACTTCTGAAAAGACTACTAATTCTGATTCTGCTGAACCCCGAAGGTCCAATCGTCGAATTC
AACCAACTTCACGGTTACTGGAGGGTCTGCAAAGTTCTCTTATAATTTCTAAAGTTCCTGGAGAGAAGGGTCCTAGGAGCCTTTACAGAGGTCCTTCGTC
GAGAGGAAGAGCTCATGGCTGAGAAAAGGCACCATAGTGCGTTTTTTGCGAGACAGCTCTTCGCTTTGTACATAGCTGTAGCTTGGCAAGTATGGAAGCA
GAAGGATAGGATTGGACTAAAAGGCTCTATTGGAGCACAAGGATTCAGATGTATTATAAACTAGCTTTTAAGTGTAGCTTTATTATGTTCCCATTTTCTT
TTGCTTGCACTGGACAGAAACTCTTCGAGTTGTAGAGTAGCCATTAATATTTTAGAACCCTCCTTTTAGTCTGTATGGATACACTGGGAAAGAAATTGGG
TGTGGTTTAAATTCGTCTGACGGTCTATCGTGTGTGTTGCTATGTGGTGAAGTGAGTGCTTGGACTCGTGGAGTGACATTTGCGCTACTTGCCTGATCAG
CCTTTTCTTTTGGGTACTGCTTGTTGCACTCGTGGAAGCGACATGTGCGCTACCTGTGCAATTTGCATTGTTCAAAATTTTTGGTAGTCCTTGTTGCGCT
AGTG
>comp605256_c0_seq1
ATTGTGCTCTCTCTCTTCTGTCAGCTCCGGCGAACTCCTTAGGCACAGACGTCGGCCGGATGGTCCGGCCCACAGAGCATGTCCCCATGGCTCAACCTCT
GGTCCCCGGCCTGCAGTTCGGCAGCTCGTCGTGGTTCTCGCGCCCTCAGGCTTCCAACGGCGCGGTCTCGACGACGACGGCCGGGTTCTCCTCCTGCCCG
GTGGTGGAGAGCGA
>Locus_4567_Transcript_29/65_Confidence_0.295_Length_2301
GCTCTTATCTACCGGTCACCCTTGCTTGCGGCTTTGAATGCACCAAGGTTCCTTTTGAGAGACTTCACAGCAGACAAAATCTGCAGCTTCTCTTTGGATG
CCGCATCAGGTTTCTGATCATCGTTCTTACTAGCTGCCTCATCACGTGGCAATCTACCTCCCGCAGACGATGCCTCCACAGGAGGTTCCCTGTCTGCATT
GGCTGCATGTGCCCGCTGCTTCCCAGCTTGCTTTCGCATGAAGGTCGCACTCCTCTTGTACTGGGAATCAGTAGGATCCAACGCGAACAAATGAGACGTG
AACATTTCTGAGAACCTCTCATCTTTGCTAAGATCAATATCAGGAAGTTTGTCCTCAACAGATTCCTCTTTACCCTTCTTGCCCTTTTTACCTTTTTTGC
ACTTCAAGTTATAGCCCTTTGCACCATTGGCAGTATCCTGGTAAGCAGCAACCAAGAGCTCCAACTCTTCTTTTGTTGCTTCTGGCTCCAAATGTTCCTC
TGGTGATTTGTCCTTTCCTTTCCCCTTTGCTTTATCTTTATTTTTCGCCTTTTGTTTCTTGATTTTGCTTGGTTTAGTTTCTTCATCAGACAGCTCCTCG
GCGAAGAAATCATCGTCGTCGTCAGGCTCATCTTTGCTGCTATAGTCATCATCATCATCATCCTTCAACCCCCTCTTCCTAGCCTTCCTTTTCTCTTGGT
GCCTCTCCCAGACAGTCTTCTCGTTGGTCTTTCTCTCTAGGATACGTTTACTGAGATCCTCAAGTTCTGTGTTGAATGTAATCTCCATATCCTGGCCATC
AGTTTGTTCCTCATCAGATTTGTCGCCTTGCAGTAAAAGAGCCAACCTCTCCTCTTTTGTTAGCTTTTGTTTGGATCCCCCATTTGGTAGAGACTCGTCA
TCAGAATTGTCGACACCGTCATCATCTGACGCACTGTCATCACTTGCTAAATACATGTCAAGCTCATCTAACTGATCGTCAGTGAACTTTCGCCTCAAGA
CCTTCTTACGTTCTGGTTCATCATCATCCCAAGTTAGCTTAACTCTGCTATGTTGCAGAGCACGAGTCTCAAAATCAGGCTCCTTGTAACTCGGAGGTGC
CTCTGTAGCAATGTCTCGAGCAGGATGTTTGAACTCCATCGAATCAGGTATAAACTGCAAATCAAATACATTTGCTGTTTTCAAGAACTCAGTACCATCA
AGATTCATGTACAGGTGATTTGCAGTTGCACTAGAATCACACACCACAACTGCATAGTAATACCTCAGTCTGTTTAGCTCGTAAGTACGCAGCTTGTTAT
TCTCTTTGTCAGAGTCAACTTCTTCTTCGTCGTCATCAGTGTTGAATTCATCATCATCAGTGTCGTCTTCACCATCGTCGTTATCATCATCATCGTTGTC
CTCATCATTATCATCAGTATTGGCATTAACAAGAGCTGCTGGGCCCTGAGTTGATTCAATTTCCATGCACTTCAGTCCAAATTCTGATGGATATATTGAG
ACTGATAAAACCCGCCCACCTTTTGGGACGCAAGATGTCATCACCATATACAGGTCAACTGCCTTGATATGATCCCAGTCCATATTAACAGCAGCAAGCC
TGTGGGTTTCCTTGTCAATCATAGGTGTATCATCGTGCCGGCCCATTAAGTAATGAGCAATGTCACTGCCGACAGAGTACTCGTCATCATCAACGTCTTC
ATCCTCGTCGTCGTCGCTACTAGAACTCTCTTCTTCCTCCTGCTCATCCTCTTCATCCACGTCCTCGTCCTCCTCTTCTTCAACTAACTTCTCTTTCTCC
TTCTTTGCCTTCTCTTTTCCCTCATCTTCCTCCTCGTCTTGGTTGAGATAGTAATGGAGCATGGGGTTCTCCTTGCCGCTCTTCTTTCGTCGCCTGCCGC
GCTTGTCCACCGGCGCCGCCGTCGAGGCGAAACTCGGGTCCGTCATCATGCTGCTGAACCTCGCGTCCAGCGCCACCTTGGCCTCCTTCCGCCGCATCGG
CCGGAACCTCGGGTCCGTCCGCGCCGCCGCGAACCGCTCGTCCTCCATTGCCCGCCTCATCTTCTCGTCCCTCCCCTCCACCTCCTCCGCTCTCACCGTC
TTGCCCTCAGGCTTCCCCTCCTTCTTGGACTTCTTGCCGTCCCGCTTCTCCTCGCGCCCCTCCTTGTGCTTCCGCTTCTTGCGCTCGGCCTCGGGGCGAG
GGGCTTCCTCGGTGGCCGCCTGACGCTCTTGATTCTTCTTCTTGTGCTTCCTCTCTTCCTTATCGGACCTCGATTTCTTGGCCTTCTTGAGGCGCGCGGG
C
>Locus_1826_Transcript_123/132_Confidence_0.149_Length_2437
TTGTCTGAAATGCTGTCCCCTCCTGCCCAAAACACTCCTCCCCCCTATCTCCGTCAAACACACACATATGAATTGTTGTATATGTAAATGTGATGCTCCA
GCAAATATTTAGCTTGCTCGTAAGCAATTGATGTTTGATTTCTTGTTGGGACTTCTTTATGTTTCAAGCGCCTCCATTTTGCAGGCATACACCAATGGAT
CATGATGATACTGATTTTCAGAGCCAAAACTTTCAACTAGCTGGTGAAGACAATAGCAAATTCCCATCGGGTTTGCGGCCATTTGCTCTGCCGAAGCTTG
ATATTGATGACCAATTACAAGGCCACCTTCGATATGATAATTTGATAGATTTGGAAGTATTTTTCAGCGTACAAGGGCACGAGAGTAGTTGGGTTGAGGT
TTTGTCCACTGGAAGTAGTGTTGTTGATTTTAGTTCCAGTGCTGCTGAATCTTGCTCCATATCTAAGACTAATAATGTCTGGTCAGAGGCAACATCCACC
GAATCCGTGGAAATGTTATTGAAATCAGTGGGAGCAAATGAGATGACTGGTAACATGGAAGGTGATGCACATCTTCAGTTAAGTGGTATGGACAGTCAAA
TGGATGCATCCAACGTGCAGCCTAAATCCAGTAACTCTCCAACAGATAGCACTGTAGTGCCAACTGAGAATGATCAGTCACAGAGGACTCATTCTAGAAT
GATGGATGATCCTGTTCGTTCTACGAACACCAATTCCAGAATGTCTGACAATCCTTCAAGCACTCAACCACAGCTTGAACATTTTGCATCTTTCTTGATT
GTCGAGAAAACTGAAAAGGCAGCAGGGTCAATTTTATCAGATAGGAACTCCAATTTTGTGTTGGAGTCTGTTGCTGAGAGGTGTATTGTAAGTGAGAAGT
TGTCTTCTTCCACATCAGAAAGCTGCCCAGATGTTGGTGACCATTTCAAGGCTGTCCATGATGACCATTCTTTGGACAAACTGAGAGTACCCTCAGCTGA
AGTAGATTCTAGGAAGATGAATAATGAACCTTTCCCAGAATTAGCTCCACTTCAGAACATTTATGACTCGTATCACTTTGAGCAAGATAATCAAGGATCT

GAGGTTGGTATTACTCCTCAGGATTCAAAGATTAGTCATATCAGTGAAAATAAAGTCGAAGGAGGACTACATGAATTGCAAAATTTGTCATGTGCTGGTC
AGCCTTTGGGTGCTGTAAATTTATCCAGTCAAGTAAGCAATGAAACTCTATTGTCAGAAAGTTCTGACGGATTGCTAGAGGCCATTACGAATCCGGTTAA
GATGATGCACATGAATGATGATACTTGTAAGAGAGCCAGTGGCACTCTGCAACCATCTTTTCCACCAATACAACATGCAGCAGAAGGCTTGAAAAGTTCA
GTTGGCATGAACAATGAACTTGTAAAGGAGTTTGTTATTGGTTCCAACTCTGCTCTATCTAACCAATCTGAGGCAGACACGAGAAATTCTAGTCCTCATC
TTGTTACTTCTATGTCAACCGAAAGCAGCAAGATGATTCAATCTCCCAAAAGAAAGGTTGCTCATGCCACTGGTGTTCCTGAAGAAACAAAGAATGCTTG
GGCCGATAGTACAAACATCTTCAGTGGTGATGAATCAAAACTTGGAGCGTTGGAGCACCATCAAGATTCTCTCGACAACCTAAAAAGTGATGCCATGGAA
GAAAAGACAACCAGGGAGAAAATATCAGCAGTTTCAGGAAATATTGATCAAATGGTGGAAAGTGGTCATGACGAAAATGCTACATGTGCTACTGGTACAT
CAAGAGATAAGTTTGACTCTTCGGACAATGCTGCACCTGACAAACTTTCAGATGACACTTTGGATAATTCAGAGGATCAGAACATTCCCTTAGTAAAGCA
TGAGGGGTCATTCAAGGAAGGTGACATGCCTGCTTCAGTAGAAGAGCCTGAAAACACGGTCCTACCTGCTTCTGGGCCTCAAGAGAAAATGTCAGCACCA
TTGGTCAATTCAAGAAGTGATATCGTTTCGACCACTGTTACTGGCACTTCCGGTAATTCAGAGGATAAAAATGATTGTTCAGGTGTTTTTCCTGATGATT
CTTCTGCTGCTTTGCCTGATGAGAAGGATTTGAAAACGTCCACAATGGATTATGAGAGGTCATTCAAGGAAGTTGCTAAACCTGCTTTAGAAGATGAAGA
CCACAATGTTATTTCTCCTGGCTCCGAACCAGTTGGAGAACTATCAGCAGTTTCAGGGAACTCAGACATAGATGCTATTTGTAGTGGTACTGTTTCTGCT
GCGCAAAAGGCGGAATACAAGGAGCAGGCTAATTCTTTGGGTGGTTTGACCACAGGAGAAACTGAGGATAAATCAGGCAACCATCCCGATGCTTATTCAC
AGAAATGCCAGACTGACGAATCTTTGGTACAATCTGA
>comp82180_c0_seq30
AATATAGGGGGCCCCGCTCCCGCAGCTCGCAGTACCGCGGCGTCACCTTCTACCGCCGCACCGGCCGCTGGGAGTCCCATATTTGGGATTGCGGCAAGCA
AGTGTACTTAGGTGGATTTGACACTGCTCATACTGCTGCAAGGGCGTACGATCGAGCTGCCATCAAGTTCCGTGGCTTCGACGCCGACATAAACTTCAAT
CTCAGTGACTATGAGGATGACATGAAGCAGATGAAGAGCCTGTCCAAGGAGGAGTTCGTGCACGTCCTGCGACGGCAGAGCACCGGGTTCTCGCGTGGCA
GCTCCAAGTACAGAGGCGTCACCCTGCACAAGTGTGGCCGGTGGGAGGCTCGCATGGGTCAGTTCCTTGGCAAGAAGTAAGAACACTTCACAACCTGAAT
TACCTAATACTAGACGTGTTATTTCTATACTGCAGATCCTTGTGATTTTTCCTATCTCAGTCTCATTCTAGAGTCATGAACCTAGTACATGAACTCCTAT
GATTTAGTATTACTCCATATTGTCTAGAAATAGTAGGTTAGATTCATGTAGCATAACGGTCGTTCCTACAAAAGAAATTAAACAGAAAAAAGGGCAGTGG
ATAGGGAATTCCTCACCGGTGGGAACGCCATGCTTGGGTGCAGGTACATATATCTTGGGCTATTCGACAGCGAAGTAGAGGCTGCAAGGGCTTATGATAA
GGCCGCGATCAAATGCAATGGTCGAGAAGCTGTGACGAACTTCGAGCCAAGCACGTATGATGGGGAGCTGCTGACTGAAGTTGCTGATGAAGGTGCTGAT
GTTGATCTGAACTTGAGCATATCTCAACCAGCTTCTCAGAGCCCGAAAAGGGACAAGAACTCCCTTGGTCTGCAACTCCACCATGGATCGTTTGAAGGCT
ACGAATTGAAAAGAACAAAGATTGATGCTCCCTCTGAACTCACGGGCCGACCTCATCGGTTCCCTCTTCTGACCAAGCATCCACCAATCTGGCATGCCCA
ATCTCATCCCTTCTATTCAAATAATGAGGTTTGATGATACTAAATTACTAAAATACAGGCAATTACTTTCCCAAACATATTGCTTTGGCTCCTAAACCTC
ACAATTGCAAGCATGTTCTGAGCATCACATGGTTCGGATCGAGCTGTCCGGCACCAATTTTGAACGTAATCTTGACCATCTGAGGAACTGATTGTGCAGC
CTGAAGGCGTGCGTTCAAATGTTAGATTTGTGTTACTTTCGTTCATTTTCACTTCTTGTGCCTGTCAATTTTGGCTCTAGTCTTACATCACCGCTTCA
TTTCCTTGGATTCTATAGCCCGCCTGTTTCCTGTCGATTTCGGAGATATTAATGCGTAGCACAGGTTTCATATGTAGGATCTTGAGTTTTCTGAGATGGC
CCATTCTGGAAACAGTAATAGTCGCCTTAGGAATAGAGATGTGTTTGATTTTTTTCCTTCAAAAGGAACATCCTAGAGAGGTGACTATTCGACATTAGGT
ATGCTAATCCATCGGTCAACTATACCTGCCTGCGGCTAAGCACCTTTCAATGTTTCTTTTTTCATTCATCGTATCATATAAAAGTAGTAAGTATGACTAT
TATTCAAACCTTTCTTGAAAAAAAATAGTTAGGATGTTGCCATCTTGTAATGCAATCATAGAATTGGAAGTGCTGAATTTGCCATTTGTTTCAAACAAGCA
TTGTATATTGGTTTATCATTCAACTTGAGAGTTTGGTCAAATTGAACTCTCGAACTTTTCAACTCCTAAACTTTAAGGTCAGGCTCACATATGTCATTTT
CAAATTTTATTGGACGGAAAGTTGAGCTGGACGTTTCGAGCTCGGCTTTCTATCCAACTGAACTAATGGAAACACTGATTTCATCGCAACAAATTTTCAG
CAATAGATATTCAATCTCTTCTTGAATTCATCCTTCATCAGTATGGTGAATAAATTGTTCTTGGATTATGCAGAATGCATCAAGAGATCTTAACAGGAGG
CCAGATCAGGGGGGTGTTCCCAGCTGGGCGTGGAAGGTGACCGCCCCTCCTCCCACGCAGCCATTGCCGCTGTTCTCGTCGGCGTCATCATCCGCTGCAG
CATCATCAGGATTCTCCAATACCGCCACGACAGCTGCCCACACCGCCCCATCGGCCTCCCTCCGGTTCGACCCGCCGCCGCCGCCGCCGTCCAGCCGCCACCG
CTGAACGTTAAGAAGCCACGCTGTAAATTTGCCAGGAAGCCGACATTTTTTCCTCTCTCGGCGTTGCAACTTTTTAGGTTTTGCGCCGGGGTGGTTTCTT
GTAGTGGAGTGGATTCATAATTAACTGTATTTGCATGCTGCCCATGTGAAATGCTTCTCTCTCTTTTTACGCTCTCTGCACCGGATGTTCTGGGAGTTCT
TGTTGCCGGGAATTAACTAAATTCACCGTCTGAGATTGATCTATACATTGTTGTAGAGAATCGAATCATTGTTGAAATTAGTTACAGAATTATAGATATC
ATAAGGCACCCTCTACTTGTGGACAAAGTTCAGGAAGATTATTCCGGTTCTTTTCTTGGCTGTCAATTCGGCTCTTGTCCTTGGTGTGGAGCCACTTGCC
TACTGTCTTGTTCTGTTGCTGTTTGTTCTTGGCTTCTTGCACAGTTACAAATTTGTTGGCTCGAATGGATGGTGAGACATTTGGTGCAA
>Locus_5717_Transcript_33/37_Confidence_0.237_Length_1451
TTGCTTTCGGTCGCTCGCCGGAGCTCCGCTCTTGCTCTCCATTGCTCTGATTGGCCTCTGCGAAGCTGCAATCTTTCGCCTTCCTGCGGTTGCTTTCTGC
AAAGCTGCCTCTTTGCTCCTTCTTTGATTATTATGCTATAATTGCGTGATTAGTCGATTAATTTGCATAAGCTTGTGTTTTTACTTGTTCACTGTTCGTC
CTTCGATTGAAGCTTGGTACTTTACTTTTGTGGCTCTGCTGTGCGTGTACGGCATGTAAAACTGCTTGTGCGGTTGTGGTTTTGGTACGGAGCTGCGTTT
TTCCTGATCAGGGAAGGAAAGATGCTGCCTTTCCACTATTAGTACTGCTGTACGTGTGGAGAATCGTACGCATGGACAAAATGGCTGTGCAAGTATAATG
CTTGTTTCCACTTTCCAGTTTTGGTTCCATGCACTCTCGGAGTTTGATCAGAAGAAGAGGAGTTGCAGGAGGCGTCTGTCCGATCACAATGCCCGTCGGC
GGAAGCCTCAGCCAGATGCATTTGCCTTTGCCTCTGCAAGGCTACCGTCATCCTTATTTGATGATAGGCGACAAATAAGTTTTGTCTGGATAAAGCTCC
TCTTAGCCATGTAAGACCTTTCACTTCTTCTACATGGGACAGCTCATCTGACTTCAAGCTTCCACATGCGAAGGAAATAAGAGAGCTATCAACAGGAGTT
GGGACTATTACTGGACAATTTCATCTGGATAAATCTCATCTGTCCAATGCCATACCAACACTTAGCCATGGCAAAGATGAGCCGTTACCAATGAAAGGTC
CGGACACATCTTTAACTGCTTCAAAATTCGATGGAGCACCGGATCTTCAGCGTGCTCTCTCTCTTCTGTCAGCTGGCTCTTGTGGATTGCCTGATCCTGT
ACGGCAAGCATCTCGTCTTATCCAGTTCTCTGGTGCCAGCGAGAATAGTGGTGGCCTTCATTCATCACATGGAGGGAACTCTGCTCCTGTCTCATGCGCT
GAGGAACAGCACATAGCACCTCAGTCTCAGCTAGTTCGTTTTACCATGGATGCCAACAGCAATGGCTACGAGTCCACTTACTTTGGTGTAAACCAGATAA
ATTAAGCGTGAAAGGCACACGCTTCTTGATCATTTGGAGACAGCCACGAAGATGGCCTGGACTTATGCAAGAACAGCTTCTATCAACTACCAGAGTGCAA
AACCCCTCTGTTAATGTTATCTACATATTTATTCGCCACCCCTGAACTAAGTCGAACTGAGCGTAGGCCAGCTTGTATTGTGTACGCAGTTCCAGCCTGT
GATTGACCCATTTTTTCTGTTCTTTTCCCCTTGATGTGCCTACTTTGAAACTTCGAAGTGTGATATTGCTACACTTGTACAACCCACTGCCAACTTTTGG
ATCTCTTCGAAACCTCGATGAATCTGAACATGTATTTTTTGTGTGTGTATG
>comp65473_c0_seq3
GCCAAGAAGAGCCGCCGCGGCCCGCGCTCCCGCAGCTCGCAGTACCGCGGCGTCACCTTCTACCGCCGCACCGGCCGCTGGGAGTCCCATATTTGGGATT
GCGGCAAGCAAGTGTACTTAGGTGGATTTGACACTGCTCATACTGCTGCAAGGGCGTACGATCGAGCTGCCATCAAGTTCCGTGGCTTCGACGCCGACAT
AAACTTCAATCTCAGTGACTATGAGGATGACATGAAGCAGATGAAGAGCCTGTCCAAGGAGGAGTTCGTGCACGTCCTGCGACGGCAGAGCACCGGGTTC
TCGCGTGGCAGCTCCAAGTACAGAGGCGTCACCCTGCACAAGTGTGGCCGGTGGGAGGCTCGCATGGGTCAGTTCCTTGGCAAGAAGTAAGAACACTTCA
CAACCTGAATTACCTAATACTAGACGTGTTATTTCTATACTGCAGATCCTTGTGATTTTTCCTATCTCAGTCTCATTCTAGAGTTATGAACCTAATACAT
GAACTCCCATGATTTAATAGTCTAGAAATAGTAGGTTAGATTGATGTGGCATAATGGTTGTTTGAACAAAAGAAATTAAAGAGAAAAAAGGCAGTGGATA
GGGAATTCCTCACCGGTGGGAACGCCATGCTTGGGTGCAGGTACATATATCTTGGGCTATTCGACAGCGAAGTAGAGGCTGCAAGGTTGCTGATCTTGGA
TTCTTCTATTGATGCAAAAAAAATTTTGCTTTACCTTCTCTGTTGATTTCCAAAGGGTTGAGTACCGACTCGATTCTCTTCTTTGTTGTTTTTCTTCTTT
CTGTTTCAAAATTCAGGGCTTATGATAAGGCCGCGATCAAATGCAATGGTAGAGAAGCCATGACGAACTTCGAGCCTAGCACGTATGATGGGGAGCTGCT
GACTGAAGTTGGTACTGAAGGTGCTGAAGTCGATCTGAACTTGAGCATATCTCAACCAGCTTCTCAAAGCCCGAAAAGGGACAAGAATTCCCTTGGTCTG
CAACTCCACTATGGATCGTTTGAAGGCTCGGAATTGAAAAGAACAAAGATTGATGCTCCTTCTGAACTGGCGGGCCGCCCTCATCGGTTCCCTCTTCTGA
CCGAGCATCCACCAATCTGGACTGCCCAGTCTCACCCCTTCTATTCAAATAATGAGAATGCATCAAGAGATCTTAACAGGAGGCCAGATCAGGGATGCAC
AGTAGGGGGTGTTCCCAGCTGGGCGTGGAAGGTGACCGCCCCTCCTCCCACACAGCCATTGGCGCTGTTCTCGTCGTCATCGTCGTCCGCTGCAGCATCA
TCAGGATTCTCCAATACCGCCACGACAGCTGCCCACACCGCCCCATCGGCCTCCCTCCGGTTCGACCCGCCGCCGCCGCCGTCCAGCCGCCACCGCTGAA
CGTTAAGAAGCCACGCTGTAAATTTGCCAGGAAGCCGACATTTTTTCCTCTCTCGGCGTTGCAACTTTTTAGGTTTTGCGCCGGGGTGGTTTCTTGTAGT
GGAGTGGATTCATAATTAACTGTATTTGCATGCTGCCCATGTGAAATGCTTCTCTCTCTTTTTACGCTCTCTGCACCGGATGTTCTGGGAGTTCTTGTTG
CCGGGAATTAACTAAATTCACCGTCTGAGATTGATCTATACATTGTTGTAGAGAATCGAATCATTGTTGAAATTAGTTACAGAATTATAGATATCATAAG
GCACCCTCTACTTGTGGACAAAGTTCAGGAAGATTATTCCGGTTCTTTTCTTGGCTGTCAATTCGGCTCTTGTCCTTGGTGTGGAGCCACTTGCCTACTG
TCTTGTTCTGTTGCTGTTTGTTTTTGGCTTCTTGCACAGTTACAAATTTGTTGGCTCGAATGGATGGTGAGACATT

>Locus_10929_Transcript_76/78_Confidence_0.103_Length_1209
CCGTGCAGGCGTTGTCGACTGAGGTCGAGCAGAGAAAGAAGTTGGGGGCAGGGAGATGAGAGTGAGCCCGGGCACAAGTTTAGGATACTGTGCGAGACTA
TCAAGTTCTGAGACGATAGTATTTGTGTTTTTACGCCCTCTCTCCTCCGCATCGAAGCGAATCTAACGTTAAGGATGCGGAGAAAGGAAATAAAATAAGG
TGACTTGTGGGTCTAATATAACATGTTGAAATACATGAACAGCCTGATGGTTGTAGGGTGGTCTACAGACTTGTCTCACGGGTGATGTGGCAGAATATGA
GCCGGATATGAGCCAACACCATTGGCCTTGCTCCTGTTTGCCCTGTGAACTGATTGATGTCTCAATCGGGCAGTTCAGCCAGTTCGCATCTTTTGAAATA
CTGCAGCCGTCCAGTCTAACAGTTCCATGACATAACTTGGGATGCATCTTACGCTGGTCACATGCTGCAGCTTTAGTCCTGAGGATGCTCTAGATTTACA
GAAAGTCTCGCAGTGAGCAGTGCTAGTACCAAGTATTGATTTGCTTAAATTTCCTGCAGATAATAAGAAATCTAGTATCCTGAAGAAATCTTGCATATTC
ACCAAAGCATCCTTTAGCTGAGTCAATTTTGGTGAAGAACCGATAGTTCATTACTCTGTTTCTTGTAAATGATGTGCAGCTAAACCATTATGGCGGTCAG
GTTTAACAAAGCTCATGCTTGTGCTGTTGCAACTGCAGGAAAGGCCAGTGGAGCGAAGGCCTGATTTGGGCCCCCAGTCGTTCCCCACCTGGGGACGGCA
AACGCAGGGCTCCCCTCACATGCCGTTGCACCACTCCGCAGCATCATCAGGATTCTCTACCGCCGCGAACGCCGGCGTCCCATTGCCGTCTCACCCGCCG
GCGCCGTTCTCGGACCACCAGTTCTACCTCCCACCGACGGCCTGAGCTTGTTGGTCCGGTTCAACTCTTGGGACAGCAGCTGACGCTCTGTGTGTGAACG
TCTCGCAGCTTGCCGGTAACAGCTGTTAATTAATGGGAGAGAGAGAGAGAGAGAGAGAGAGAGAGAGAGAAGAGAGAGAGCCAGAAGACCCAGTATGGCTGA
CTCTGTGGCCTGTGCCTTGTTATGATCTGAGTTATTGCTACTACTACTACACCGTGGATTCATCATGGTTTGAGATTCTTTACCCAAGTTTCAACTTGAA
AGTGCAAAT
>Locus_11912_Transcript_60/63_Confidence_0.305_Length_3544
TTTTCCTGGCGCCTCCCGCCGGGCGCTCGCCCCGGATTTCTCGGCTCCCCTTTCGGTGGATGCCTTGACCGGGGTGGGTTCTTGGCTGCGGCGGCGCGTG
ATCTCTGCGTACTTAAGGAGGGGTTGCTAGCGGGGTTTGTGGGAGAAGGCGAAGGAAGCCGGGGGCTGCGAGGCGTTCATGGGTTAGATCTGGCCGTCCG
GCGCCGCAGATCTGAGCGAGGTTTCGGTGGAGGGATCGGGGCCGAGAGGTCGCCGTCTTCGGCTCATTTGGAGTAGCTTGGGCCTCTCATGTCCAAATCG
GGTATGGATGGTGGATGCGGTAACTGTCGGAAGGAGGGCTTGTTGCAGATCTGAAAGCCATCCAGCTCCCTTGTTCAGTTTACCTGCTTGCTGGTCAGCT
GGATGGAGTGAATGGCAATGCTTTCTGATGTTTGACTGCCGTTAGTTGCTGCTTGGTGTTTATTCAAGAAACTTATCAGGAGGTGGTGTGTGCCCCGGTA
ACTATTCAGTTGCTGATATCTTGGCCTGTGTTGGATAACGAGGTAGCAGATCACATTCCTGTGTTTTTGTTGATATCTTGGTTGTTCTTGAATAACCTGG
AGGCTGGAAGGGGTCAGATCCAGTTCCTGTGGCCCTGGTAATAAGGTGCTGTTGGGGGAAGGCACCATGACCTACTTCCCTGAGGAGGTGGTGGAGCACA
TCTTCAGCTTCTTGCCATCGCACAGCGACCGGAACACGGTGTCCCTGGTCTGCAAGGTGTGGTATGAGGTCGAGAGGCTGAGCCGGCGAGCTGTCTTCGT
GGGGAACTGCTACGCTGTGCGCCCGGAGCGGGTGGTGCTGCGGTTCCCCAATGTGAAGGCCCTGACGGTGAAGGGGAAGCCGCACTTTGCGGACTTCAAC
CTGGTGCCGCCCGATTGGGGTGGCTACGCGGGGCCGTGGATTGAGGCGGCAGCGAGGGGCTGCGTGGGTCTGGAGGAGCTGCGGATGAAGCGGATGGTGG
TGTCGGATGAGAGCCTTGAGCTGCTAGCTCGGTCCTTCCCGAGATTCAAGGTCCTCGTCCTCATCAGTTGCGAGGGGTTTAGCACCGATGGGCTAGCAGC
TATTGCGAGTCACTGCAAGCTCCTGAGGGAGTTAGATTTGCAGGAAAATGATGTGGAGGACCGTGGGCCTAGGTGGCTCTCCTGCTTCCCTGATTCCTGC
ACCTCTCTTGTCTCCTTGAATTTTGCCTGCATCAAAGGGGAGGTGAATCCTGGTTCATTGGAGAGACTTGTTTCTAGGTCCCCGAACCTACGCAGTTTGA
GGTTGAATCGATCTGTATCAGTAGATACACTCTCAAAGATATTAGTACGCACCCCTAATTTGGAAGACCTGGGGACAGGGAATTTGACGGATGAGTTCCA
AACTGAATCCTACTTCAGGCTGACTAATGCTTTGGAGAAATGCAAAATGTTGAAGAGTTTATCAGGATTTTGGGATGCTTCTCCTGTTTGCGTTCCATTT
ATCTATCCCCTCTGTCATCAACTAACTGGACTAAACTTGAGCTATACTCCCACTCTGGATGCTTCTGATCTCACTAAAATGATCAGCCGCTGTGTGAAAC
TCCAACGTCTTTGGGTACTGGACTGCATCTCAGATAAGGGTTTGCAAGTGGTGGCCTCCAGTTGCAAAGATCTACAAGAACTCAGGGTGTTCCCATCAGA
TTTTTATGTTGCTGGTTATTCTGCCGTGACAGAAGAGGGGCTTGTTGCAATATCGTCAGGCTGTCCAAAATTGAGCTCTTTGCTGTATTTCTGTCACCAG
ATGACCAACGATGCACTTATTACTGTAGCTAAGAACTGCCCAAATTTCGTAAGATTTAGACTCTGTATTCTTGAGCCAAAGAAGCCAGACGCCATGACAA
ACCAGCCGTTAGATGAAGGCTTTGGTGCAATTGTTCGTGAATGCAAGGGGCTAAGGCGACTGTCAATGTCGGGTCTTCTCACTGACAAGGTTTTCATGTA
TATTGGAAAGTATGCCAAGCAACTTGAGATGCTTTCTATAGCATTTGCTGGAGATAGTGATAAGGGGATGATGCATGTTATGAATGGATGCAAGAATCTA
AGGAAGCTGGAGATTAGAGACAGTCCATTTGGTGATGTTGCGCTCTTGGGGAATGTTGCCAAGTATGAAACAATGCGATCCCTTTGGATGTCATCATGCA
ATGTCACATTAAAGGGGTGCCAAGTCCTTGCGTCTAAGATGCCGATGCTCAATGTGGAGATCATGAATGAGCTAGATGGAAGCAGTGAAATGGAGGAAAA
CCATGGAGATCAATCTAAGGTGGAGAAGCTATATGTTTACCGCACAACTGCTGGAGCGAGGGATGATGCACCAAATTTTGTTAAAATCCTATAGTTAGCT
GAGAGTAGCCTGGTATCTCCTCACTCTAAAAGGTGATCCTTTTTCCAGATATATTTTCTTTCATTTGGGTTTTTTGTGTCTGTTGGGCAATTTGTCTGCT
ACTCTGTGGTAGCCCAAAAGTAGTTTGCATATACTGTTCAAATTTTGATTTTTTTTCATGCTTAATAGCTCTGTTGGATACTGCATCTTACTTTATGGTC
CACAACACGCAAATGTTGGCTTAAATGTTACATTTAGAAGCGTCTGAGTTAACTTGTTTCTTAGTTTGTAAGTGCATTATATCAGAAGGGTCTATAATTA
TTGCTTGAAATGCTGCATGAAGAAGCTTGTGCCATCATCATTAACTAAAACCAAGACTGATTTCTAAAAGATCCGAATGGATAATACTTGAGAACTGATG
TTTGAACCACTTCTATTCTGCCAAATGTTTAGTTGCTCTTTTTGTTTCGTCCTCAGTACATGATCGCGTAGCCACTCAATAGAGCTAATTTCCGTCAACA
TGTATATCATTCGTATTCCATTGCGAGTATGGAGCAGCAGAGTATCTGATATGTCTAACTCTGTCAGAGCATCTGATATGTCTTACTCTGTCTCCGCACT
CCCTACTCGATTGCTTTTACAGATGGTCGGTGGAATCCGGCAGGCCGAGGGGCTGCGGCAACAGTGGAAAGTTCTTGAGGTGTCTGCCTCTCCCTTTGCA
GGGGTCAGGAAAAGCAAGCCACACCGGTGTGCGCCCTTATCTTGGAGCAACTGGCCGGGATCCGTCATCATCCTCTGTACCACCGCCCCTACCCTTTTAG
TGTTTGGTCACTCATATGGCCACTGAGGCCCTTTATCATCCTGTGCATTCTCACTCGAGAGGACAACGTTCTTTTTCTCCCTTTATCCCAATTGTAATC
GTAGTGTACCAGTACCCATTTGCTTTTGACATTTGCACAGTAGTGGATTGTGTTACCGCCGCCGACGCTGCTGCTGTAATAGTGTTTGTTTCGGCCTGAA
TGATCGGGACATGACATTCCGGGGACGACCATTGGTTTCTTGCTCCTGTGAATGCATTGCATGGAATAATTTGAGGTTCACCTGTGACATTCTTCTATTA
TTTGGTTTTGAATGATTGGTGCCCTGGCGAACGTTTTTGACTGT
>Locus_18892_Transcript_50/56_Confidence_0.103_Length_671
AAAATTTAATACTTACATGAGTATATACATCTCGTTTAGTGCTATAGTTTAACTCTTCACTAAGAGTTTGCTAAGTTTTTTTTATTTAATCTCCTTTACA
TAAGGTAAATGATGACATAGACATGTTTAAAGCTAGCTAAGGAGGCTTCTTCGGGGCTCTTAGTTCAGGTCTTATAACCTTTATTGAACTTACGACATTT
GCACCTATCTGTTGCTGCTGTAGCCAATGAGTTCATCGTCCCCGTGGCCTGTGTATCACCTAAGCACAGCAGTACCACCTCACCATCAGCGTTTGTACTC
ATCTGCTTGTCCTGGCTTCTTTCCGAACCTCCAGGAAAGGCCAGTGGAGCGAAGGCCTGATTTGGGCCCCCAGTCGTTCCCCACCTGGGGACGGCAAACG
CAGGGCTCCCCTCACATGCCGTTGCACCACTCCGCAGCATCATCAGGATTCTCTACCGCCGCGAACGCCGGCGTCCCATTGCCGTCTCACCCGCCGGCGC
CGTTCTCGGACCACCAGTTCTACCTCCCACCGACGGCCTGAGCTTGTTGGTCCGGTTCAACTCTTGGGACAGCAGCTGACGCTCTGTGTGTGAACGTCTC
GCAGCTTGCCGGTAACAGCTGTTAATTAATGGGAGAGAGAGAGAGAGAGAGAGAGAGAGAGAGAAAAGAGCCG
>Locus_33461_Transcript_1/1_Confidence_0.000_Length_553
CGGCCTTCGGCTGCCAGCCGTTCGCCATCACCCCTTCCTTAGAGAGCAGCAGTAAGCAGAGCAATGGCAACTGTGCTCTCTCTCTTCTGTCAGACAACCA
GACGCCGGCACAGATGTTGATCCCCACCGCGCAGCCCCTCGGCGCGGCGCTGCAGTACGGCAATGTGGCACGGCTCACCGACGACGGCGACGTCTCGCTC
ACCGGGATGTCTTACGTCAGGGCGGGAGACAAGCAGACATCCATTCTGGCCACATCTGTTGGTCACACTGCAGCAGCAGCAGCAGCTGCTTCTCCTGCTC
CTGCAAGTGCAGCAGCAGCACAGCTGCAGCAATACCATGGTTACTACCATGTGAGTGGCGTCGATCATCAGGGCAACCCAGACGGTGCCGCCATTCAGGC
CCTTCCCTTCTCATCGTGGTAGTGTTCTTCGCAATCAGGTGTTCGGCAGTCGTCGCTCTTATTGTTCATCGAATATGTCAGTCATAATAGTGGTCTTGAT
GGTACTGAACTTCGACAAGAGATGTCAAAATTTTCAATTTATCCTGATAGTTC
>Locus_10929_Transcript_36/78_Confidence_0.196_Length_2135
TCGCAGTACAGGGGCGTCACCTTCTACAGGAGGACGGGCCGGTGGGAGTCTCACATCTGGGATTGCGGGAAGCAAGTCTACTTAGGTGGTTTTGACACTG
CGCACGCGGCTGCGAGGGCCTACGATCGTGCTGCGATCAAGTTCCGAGGGCTCGACGCGGACATCAACTTCACTCTGAACGATTACGAGGATGATTTGAA
GCAGATGAGGAACTGGACCAAGGAGGAGTTCGTGCACATCCTCCGCCGCCAGAGCACGGGGTTCGCGAGGGGGAGCTCCAAGTACCGCGGCGTGACGCTG
CACAAGTGCGGCCGCTGGGAGGCGAGGATGGGTCAACTTCTTGGCAAGAAGTACATCTATCTTGGACTCTTTGACAGCGAAGTTGAAGCTGCAAGAGCAT
ATGACAGGGCAGCCCTTCGCTTCAATGGGGGGGAAGCTGTTACTAATTTTGGGCCTAGCTCCTACAATGGAGGAGATGCTCTACCCGACACCGAAAATGA
GGGTATCACAACCAGCCTCGCCATTTATTTCTTTTCATATAAGCAAATGTATCTCAGGTGCACCTTATAATCATCGCTGTGTTCTTAGCAACTGTTGATG
GTGACGCGGTTGATTTGGATTTGCGGATTTCACAACCTAATGTGCACGACACTAAAAGGGACAATACCTTAGCTGGAGGCCAGCTAACATGCGACTCCCT
TGAATCTTCAAACACGATGGCCTCTCAGCCAATGAGCTCATCGTACCCGTGGCCTGTGTATCACCAAAGCACGACAGCAGTACCACCTCACCATCAACGC
TTGTACTCATCTGCTTGTCCTGGCTTCTTTTCGAACCTCCAGGTATTACCAAGCTAAAGCCTATTTTTTATGTGCAAATTGCTACTCCTACGTGATCAAT
TAGTTCAAACTGTTATTCGAATCCAGGATTGTTTACTGAATATGTCCTGTCTTCAGAAAAAATAACACAAAAAGGAACATAATATGTCCTGTCTAATTGT
GGACTGATACTAGCCAATTAAACAGTTTCCCATATCCATTTCCTGTGTTTATTGTTTTGATTTGCTGGCATGGTTAAACTATAGTAGCTTGATTCGATAA

AAACACTAAGCAGGCTTTGATATTTTGAATGAAATGTCGTTCTTGGAAATACAGCCTTTTCCTTGGTATATCAAGGACAGTTGTCCATCTGCACTCTGCA
CCCTCAGCTTGTGTAACTGTTTGCCTTGTGAACTGATTGATGTGTCAATCAGGCAGTTCTGCCAGTTTGCATCTTTTGAAATAGTGCAGTCTTCCAGTCT
AACAGCTCCATGACATAACTTGGGATGCATGCTGCAGCTTTCTTACTGAGGATGCTCTAGATTTACAGAAATTCTCGCAGTTAGCAGAGGGCTATCAAAT
CTGATTTGCTTAGCTTCCTGCAGACAATAAGATAAGAACTCTAGAATCCTGAAGAAATCTTACTTGTACATGTTTACCAAAGTATCCTTACTCCTTAGTT
GAGCCAATTTTGGTGAAGAACCGATAGCTCATTACTCTGTTTCCTGGGAATGATGTGGGCCTAAACCATTCTGTGGTCAAAGTTTAACAAAGCCCATGCT
TGTGCTGTTGCAACTGCAGGAAAGGCCAATGGAGCGAAGGCCTGAGTTGGGTACCCAGTCATTCCCCACCTGGGGATGGCAAATGCAGGGCTCCCCTCAC
ATGCCGTTGCATCACTCTGCAGCATCATCAGGATTCTCTACCGCCGTCGGCGCAAACGCCGGCGTCTCGTTGCCTTCTCACCCGCCGGTGCCGTTCCCGG
ACCACCAGTTCTACTTCCCACCGACGGCATGAGCTTGTTGGTCTGGTTCAACTCTTGGGACGGCAGCTGATCTTATGTGTGAACATTTCGCAGCTTGCCG
GTGACCGTTGTTAATTAATCGGGGAGAGAGAGCCAGAAGACCCAGTATGGCTATCTCTCTGTGGCCCGTGCCTTGTTATGATCTTAGTTATTGCTACTAC
ACCGTGGATTCATCATGGTTTATGAGATTCTTTACTCAAGTTTCAGCTTAAATGTCCAAATACATGAATGCATCATACACAGGATCCGTCATTTTCTAAA
AAAATACAGGATCTGTGTGAAGTGTAAACATAAGG
>comp82796_c1_seq4
AGAAGCAGTGGCAGAAGTAGTTTGTTGTGGGGAGGGCATGTGCCTGGTTGCTGTGTGGGGGGAGGCAGCCACACACTGGGCCTCACTCGTCCTGCCTCAG
GTGGGGTTTGTGGAGGGAGGTAGGGGAAAGGTTGCCATCTCCATCTTGTGCGTCTCTGTTGGATCTTTGTGAGTCATGGCCTCTTTGCTCTCTTGCTTTT
CTTCGTTTCTTGTTGAACTCATGGTTCTTGTTTTTCTTGTGCTTGCCAGGTAGAATTATTCAACCGGATATAGATTCCTTCAAGTAAAGGAGTGCTAGCG
GTTTTTACCTGTTCGGGTGGGAGCACAAGTTGAGATCTTTAATTCTAGCATGGGTTCCTTTGGGGTGAACTGGAATCAGAAGAACTCCGCGGTGTGGGAT
TGGGAAAAATTAGCTCCATCTGTTCCAAATGCAATCGGAAACCCCAGGAACGGAGCTACGGGGCATGGATCTGTGAATTCATCTGGTGGCACTCTCACTT
CTAGCTCAGAGCTAGGGCATGGTTCATCCAAGAGCTCCATGTCGGCATCCATTGATTCACCGTCCAAAGTAGGGAACAGCTTGGAGTTCAATTTTGCTGC
TGTCAATACGTATGGTAAGAACATGGATAAGGATTGTAGAGTTGACGACTCAGGGACTTGTCCATCGTCCATGATAGCATTCAGCCATGGAGAGCCATTA
ATCAGTCTTAAGCTTGGAAAAAGGACTTACTTTGAAAATGTCTGCGGAGGACAGGATTCCAAGAGCACTGCACCTTCTACCAGGACTTCTCCATCAACCG
TTCTCAAGAAGACCAAGGTGTCTCAGCAGAATACACAAATGTCATACTGTCAGGTTGAAGGTTGCGGAGTTGATCTATCTTCTGCTAAAGATTACCATCG
CAAGCACAAAGTCTGCGAAGCTCATTCTAAAGCTCCGAAGGTGGTTGTTGCTGGTGTACAGCGCCGTTTTTGCCAGCAGTGTAGCCGGTTTCATGGCTTA
GCCGAGTTTGACCAGAACAAACGAAGCTGCCGTAGGCGTCTTACTCATCATAATGCACGGAGGAGAAAACCACAGGCAGATACAATTTCATTCAGTTCAT
CGCGGCTCTCGACAATGTTTTATGATACAAACCAGCAGAAAAATCTTTTCTGTAGTCAACCTCATTTTGGCCAAGCGAGAAGCAATGCAGTTTTTTCTTG
GGAAAACTCGGGAGATTCCAAATTTATGGAAACCAAACATCTGTTGATGCAGCCAACGAAAATGTTAGGTCTTGATGGGCTGCATTTCTCAACCCCCCAG
ATATCAAATAGTGTTGTGGCTCACTCTGTACATCATCATAATTTTGATGGGTTCACGCCATTCAAGGGAACCAACACAAAGGTCCTCAACCAAGGTGTCG
AAGCTTCTGCGATCGCTTCCAACTCGAGTGGAGACCCGGATCTTGGGTGTGCTCTCTCTCTTCTGTCAAACGGTTCGTGGGGTTCAACTTCAGCCGTCAT
CCAACAGCCTAGTTCTCATGCGCAAGCTGGTGCGGTGCCACCCTTCGCCACCGTCGCCGTCTCCAACCCTGCAATGCATCCTCTGGACTCATCCCCGGGA
GGATTCTGGCAAGACGTCCCTCCCCCGCTCGATGAAACTCCACATATTCAGGCATTCACGCACCTCTGATCAAATGCTGCATCCCCTGGGAACAGCAGGT
GGTTATACCTTCCGTAATCGTCCTAGGTTTGCTTTGCCAGTGACTGGCTCTGCACAATGTCGATCCATCACCGGAGGCATTTGGTAAGGTGAAGACTAAA
GACTCAAGAATAACCGGAGACTAACTCGCTTCTCTTCCAGGCTCAAGACTGTTTGGGTCCAGCGGTGTTGCTACCTGATCCTGTTTTCGGTTCTGAGTAC
ATCCATTACTAATCATGGGATTACTTGTTTAGGCCAACCAGACATATTCTGGGGTGAGATTACGACATGTTAATTTACATTGACGGTGTTTCTACATGTG
TTAGTACTAATGATGTTATCGCTCTTGGCCCCGTTACGTTTCAAGATCTGTTACCATTTGAGT
>comp73418_c0_seq7
AGGAAGGGACGGCGGGGAGGGGGCAGGCACAGAGAGCAGCTTTGCTTGGCGGACGCACCGAGGGAGGCGCCTGGGAGCCCTGCTTCTCCCCTCTTCGTCT
TCCTCCTCCGCTTCCAAAGGTTACCGTCTCCCGCCGCCATAAGACTAGTAGCCTTCTTGCTGTGTCAATCCGTCATGCACGCGTCCCTGGATTGCGCTGG
TGACTCCTTCGGGAAAATGGTTAATGATCATACGAGGTCAAATTTGGTTTTTGATAATAAGCAATCTCTATTTGCAAGTCATAACATTGACTACGGCCAG
CCAATAGCTTGTATATCATACCCATACAATGATTCGGGCTCAGGAGGTGTTTGGGCAGCCTTTGGGTCACGCACTAGCGCTGCAGCTGTGTTCCATCCCC
AAATTGCTTGTGGGGGCACATCTGCGAGAGTTCCCTTACCTCTGGAATTAGCAGATGATGAGCCCATATATGTCAATCCCAAACAATATCATGGTATACT
TCGCAGAAGACAGCTACGTGCTAAGTTAGAGGCTCAGAACAAGCTAGTCAAAAACCGAAAGCCTTACCTTCATGAGTCTCGGCATCTTCATGCAATGAAG
AGGGCAAGAGGTTCTGGCGGACGTTTCCTTAATACTAAACAGCTCCAGGAGCAGCAGCAGCTGAAGTCTCGCAATGCCTCCACCAGGTCCACCACAAATG
GCGCAAATTCCTCAGGTTCAACACATCTACGGCTTGGTGGTGGTGCAGATGGAGATCAAACCATGTCGGGGACGAAAACAATGGCCTCACAAAACAATAG
CAAAAAGGCTGTTTCTTCTTCTGCAGCTCTTGCTTTCACCGTGACTCCTATGGTGCGCAAAGATGACACCTTCTTCCAGCACCTCAGCCACAATGTCAGC
TTCTCCAGCCATTTTGGCCAGGCAAGCGCCCAAACCGGCGTTGGAGGCATCCATAATGAGACCCAGCATAGGGTTTCCGTGATACAATGACGGTTTGCGA
AGCTTATCTGGTGATCCAGGCTTCTAGGTGTCCTCGTGTCCGGTGTAGTCTTGGTCGCTCAGGCAATTCATCCTTGGCTTAGTTTTTGGTGTGTTAGAAC
CTACAAATGTTTCATACTCTGTTGTGGTTTGCAGAGCCCATAAATCAGGGCTCTCCTAAAAAAAAATCAGGGCTTGATGGCGACACTACTATCCAAGTATT
GTTGTAATGGTGTGTGTTAGAAACTCGCAAAAAACCCTTCTTTGTGCTCGTATTAGTACATTATGCTGCACAATTAGCTGCTATTCTGTTCGTACTACAG
TCTGGT
>Locus_9733_Transcript_13/16_Confidence_0.190_Length_991
GCATGCTCCGTCGACGGGTGCCGGTCCGACCTGAGCCGGTGCCGCGAGTACCACCGGCGGCACAAGGTCTGCGAGGCGCACTCCAAGACGCCGGTTGTGG
TCGTCGGTGGTCAGGAGCAGCGGTTCTGCCAGCAATGCAGCCGGTTTCACATGCTGTCTGAGTTTGACGAGGGGAAAAGAAGTTGCCGGAAGCGTTTGGA
TGGCCATAATAGGCGTCGAAGAAAGCCACAGCATGATATGACGAATCTGGGGGGTTTCTTTCCGTACCACCAAGTGAATCAGTTTGAGATCTATCCACGA
GCAATTCCAACAGCCATCCAGAACTCTGACGCCATACACACAGTCGATCGTCAGCCGCCGTTTTCCATCTCATTCTCAGGAGCCTTCAAGGGTCCAAAGC
AGTCCCCGTTCTCTCAGAGCGGTGGTGGCACGCTCAGCGCACCCCACCCCGGCCATCTTCTGGCTGAAGAGAGCAGCCACGCTAGAAGCAACGCATGCAA
CGGTTTCTCCGGCACGCTGGGTCCCGAGTGTGCTCTCTCTCTTCTGTCATCGTCGCTGCATCATCCCTCCCCTGCCATCATCCCCACCGCAGCCCAAGCG
CAGGCCACCTCTTCGCTCGCCCGGTTTGCTCCCGCGTCGCAGGCCGCAACCACTGCATTCGCATCCGGTGGCGGCCACCATGTGTTTGTTCCTGATGCTG
TCTTTGAGGATCCTTCACAGGCACTGCCGTTCCCTTGGCAGTAGCAGAGGGGTGGAAAATGTCTTGTGTTTCTGTGTTGCTGATGAGTGCTCAGTTGCTG
GTAATGGCAGGATCAGGCTGTTGGTCAGAGATGTAGATCAAGTGGACAGGTCAAGTCCTGTTCTCTGGATGATGATGGAGGTGGTGGTGGTAGCTGTTGT
GCAGATTGTTTAGTTGATTAATTCTGTTCTATTCATAATTGTGTACAATTTTGTGCATGTTTTGTAGCAAAAAAATTATGCATTTTTGCAA
>comp78295_c1_seq4
ACAACTGCACAACTTGCAAGAAAGCCACAAGCAACTAATGGGTGAGAAGCTTTCTGGCCTAGGTGTGAGAGACCTCCAGAGTTTAGAGAATCGTCTTGAA
ATGAGCCTACGTAGTATCAGAACGAGGAAGGTCATATTCATTTTGTTTCCACGTTTTGTTTTTCTATTCACTCATCTTAATATGCATAAACATCCATCAA
ACAGGACAATCTTTTGAAAAGTGAAATTGAAGAGTTGCACAGGAAGGTAAAGCCTTACAATCCATGTCTTTTTATTATCTCAAACAAATGACCATGCATG
GAGGTTGCTGACATCTTAACTTAATGCTGATTGAAACAGGGTAGCCTAATTCACCAGGAAAACTTGGAACTCTGTAGAAGAGTAAATACCATGTCACAAC
AAAATATGGATCTACAAAGAAAGGTTTGATGAACGATCTCATGCAGAATCCTTAACCAAATGAAGGTGCATTGTCAGCATAAAGTTAAATTACTTAATTT
CATCTCCAGGCGAGTGAACCAAGAGGTGTTGCCGATGCAAATAAAAGCTCTAGCACTCCCTACAGATCTTGTAGTGCACAAGATGCAGATATCACGGTTA
ATCTTGAATTGAGCCAATCACAGCAAAAAGAGGGGGAGCAGTGCCAAAGAGGGGCTCCAGAACTGGGGCTTCAGCTGCACAAAGAAGATGCACTACAAGC
TGGAGAGTATCATGATGTTCCACCAATTCTAACATGCAATGCGGAGCACTTAAACTACAATTAGTATTTTGTGCAACATGTTAGATGAGTGAACAAAATG
GTTTATGTACAGACTACAGATATGACCATCTATCTTATCTGTGGCTCTACTGTACTGAAATTGCAAGAAATGGTTATTGTAATGACTAATATGATACATT
GACTGTCATACCGTTGACCTTGAAGATAGTTCTATAACGGAAAGGTCCAATTTGAAACAAGTGTTACGGATGAGCTAGTAGGATTTTTTTCACAAACAAT
TAGATAGTCCACGTTAGTGGTAGCTGCTGGGTATGACATTTGACCCAGCCTGCCGACCCTCCCTTTCCCATGGGAGTTCAATTAACATTAGGAATGAATA
TCGATATTATGGTTGATTATTTGAATCGTATGTTTTCCACCCTTAGATGTAAGGCCCTATTTTACTGTTTTAATGGATTAACAAGGTGAGCTTTCGAGAT
GGTAGCTCATTGATTCGCATGCATTTTAGTGGCTGTTAAGAGGTACTTAGTGCATCCATGCTTGAAGGTGTTGTTAGTTCGACAAACCATTTTCTATCCT
ACTGGATTACTATCTTTTGGTCAGTATGACAAGCAAGAATTTAATCTGCCAATTAAGAATTTAATGTGTTCTTTTGACATGAATGTCAACAGCATTTAAG
TAACTCAACTTTTAATACAAAGTACTGGAATGTTTTCTCACCGAAAGTAAAGATGTGGTTCCTTGATAGCAAACAATGTTTATGCCAATATCGCATTAAG
GTGGTTGTATCCTTTAGAATGATCTCCTCATTCGTGCTTAATTTGATGCTATTGTCTTTTTACGGATGCCTAGATGTGGTTTTGACCATTACTAAGTACT
TTAATATATATTTAACAAAAATAATGACATAGCTGTTGTATGGCCAATCTAGATTTTGTTTTCGAAACATATTTGCAGTCAAACTATAGGAAGTAAGAAA
CTTCAATGTGGTTCTCGAAAACCATGCTTAAAGTTTAGAAAGTTTGACTACATAGAGTATATACTACATAGTCTTAAAGCATCAAATGAAGAGGAATGGA

GGGAGTAGCAGACAATCATCATTCAATATCTATTTCTTGGTTGCCTTCAAATTTGGGCTTACTAAAATCGATTTAAAATTCTCGTGTTCCAGTTGAAGCT
CACTTCAAGTAAATCAGGAATTACCATATTTTTCATTGCTCATAATTTGGAAACATGGCATGATATTTGATAGAAAATGTACAAATCCTCAGCTGCTCAG
AAAGTTACAAAGGGAGGAACGCCCATGAAAGAGAATTCATGGCTGCAACAACGAACATAAACACAGCCTATTCTGTCCAGCAGTCATGGTAATACAAGCT
AAAAAGGACCGCTCTGGAACAATAGCTTGAAAAGCAATATTAATACAATTTCAGATGAACTATGTGCGTCGATGTCTTACTTGTGACTTCCTTGCAAGTA
ATGCGGTTCCTGCCTCATGCTTGGTGCCGCCGCATGCAATAAACCGATAACTCGAATCCTCCGTGACTTCCACCCTCTCCAGCCAATCAGCGGCTTGCTG
CGTGCGGGCGCTGGTGTGCGGGGGGCGCCGGCTTTATATTAGGGGCGGGGGAGAGTCTTGAAG
>Locus_11912_Transcript_46/63_Confidence_0.331_Length_3202
CACTGTCCAGATTCGCCTCCCTCCCCTCCCCCTAGCTCCTACTTCCCGCTCCTCCAGCTACGGCCGCGCGCTCCCCCTCCCCGCCGGCCACCTTGGGGGG
CTCCTCCCCGCCGCGCGTCCGGCCGCTCTTTTCCTGGCGCCTCCCGCCGGGCGCTCGCCCCGGATTTCTCGGCTCCCCATCCGGCGGACGCCTTGACCGG
GGTGGGTTCTTGGTTCCGGCGGCTCGGTGATCTGTGCGTGCGAAAGGCGGGGCTTTTAGCTCGTTCTGTGGGGGGAGGAGGAAGACGGCGGCGAGGCGTT
CATGGGTTAGATCTGGCGGTCCGGCGCCGCGAATCTGAGCGAGGTTTCGGTGGAGGGATCGGGGCGGAGAAGTCGCAGTCTCTGGCTCGTTCTGAGCAGC
TTGGGCCTCTCATGTCCAAATCGGGTATGGATGATGGGTGCGGTAACTGTCGGAAGGAGCGTCGCTTGCAGATCTGAAAGCCATCCAGCTCCCTTGTTCA
GTTTACCTGCTTGCTGGTCAGCTGGATGGAGTGAATGGCAATGCTTTCTGATGTTTGACTGCCGTTAGTTGCTGCTTAGTGTCTGTACAAGAAATTTAGC
AGGAGGTGGTGTGCTTATGAGACTCTTCTAGGTGCTGATATCTTGGTCTCTGTTGAATAACGCTGGAGTTGGAAGGAAGAAGATCACATTCCTGTGTTTT
TGTTGGTATCTGTGTCTTTCTTGAATAACCTTGAGGCTGGAGGTTATCAGATCCAGTTCATGTGGCCCTGCTAATAAGGTGCTGGTGGGGGAAGGTACCA
TGACCTACTTCCCTGAGGAGGTGGTGGAGCATATATTCAGCTTCTTGCCGTCGCACAGTGACCGGAACACGGTCTCGTTAGTGTGCAAGGTGTGGTATGA
GGTTGAGAGGCTGAGCCGGTGTGCTGTCTTTGTGGGGAACTGCTACGCGGTGCGCCCAGAACGGGTGGTGCTGCGGTTCCCCAATGTGAAGGCCCTGACG
CTGAAGGGGAAGCCGCACTTCGCGGACTTCAACCTCGTGCCGCCTGATTGGGGTGGCTACGCGGGGCCATGGATTGAGGCGGCAGCGAGGGGCTGCGTGG
GTCTGGAGGAGCTGCGGATGAAGCGGATGGTGGTGTCGGATGAGAGCCTTGAGCTGCTAGCTCGGTCCTTCCCGAGATTCAAGGTCCTCGTCCTCATCAG
TTGCGAGGGGTTTAGCACCGATGGGCTAGCAGCTATTGCGAGTCACTGCAAGCTCCTGAGGGAGTTAGATTTGCAGGAAAATGATGTGGAGGACCGTGGG
CCTAGGTGGCTCTCCTGCTTCCCTGATTCCTGCACCTCTCTTGTCTCCTTGAATTTTGCCTGCATCAAAGGGGAGGTGAATTCTGGTTCATTGGAGAGGC
TTGTTGCTAGGTCTCCAAACTTACGCAGCTTGAGGTTAAATCGATCTGTATCAGTAGATACACTCTCAAAGATATTAGCATGCACCCCTAATTTGGAGGA
CTTGGGGACAGGGAATTTGACAGATGAGTTTCAAACTGAATCCTACTTCAGGCTGGCTAATGCTTTGGAGAAATGCAAAATGTTGAAGAGTTTATCAGGA
TTTTGGGATGCTTCTCCTGTTTGCGTTCCATTTATCTATCCCCTCTGTCATCAACTAACTGGACTAAACTTGAGCTATACTCCCACTCTGGATGCTTCTG
ATCTCACTAAAATGATCAGCCGCTGTGTGAAACTCCAGCGTCTTTGGGTACTGGATTGCATCTCAGATAAGGGTTTGCAAGTGGTGGCCTCCAGCTGCAA
AGATCTACAAGAACTGAGGGTGTTCCCGTCAGATTTTTATGTTGCTGGTTATTCTGCCGTGACAGAAGAGGGGCTTGTTGCAATATCGTTAGGCTGTCCA
AAACTTAGCTCCCTGCTGTATTTCTGTCACCAGATGACCAATGATGCACTAGTTACTATAGCTAAGAACTGCCCAAATTTCATAAGATTTAGACTCTGTA
TTCTTGAGCCAAAGAAGCCAGACGCCATGACAAACCAGCCGTTAGATGAAGGCTTTGGTGCAATTGTTCGTGAATGCAAGGGGCTAAGGCGACTGTCAAT
GTCGGGTCTTCTCACTGACAAGGTTTTCATGTATATTGGAAAGTATGCCAAGCAACTTGAGATGCTTTCTATAGCATTTGCTGGAGATAGTGATAAGGGG
ATGATGCATGTTATGAATGGATGCAAGAATCTAAGGAAGCTGGAGATTAGAGACAGTCCATTTGGTGATGTTGCGCTCTTGGGGAATGTTGCCAAGTATG
AAACAATGCGATCCCTTTGGATGTCATCATGCAATGTCACATTAAAGGGGTGCCAAGTCCTTGCGTCTAAGATGCCGATGCTCAATGTGGAGATCATGAA
TGAGCTAGATGGAAGCAGTGAAATGGAGGAAAACCATGGAGATCAATCTAAGGTGGAGAAGCTATATGTTTACCGCACAACTGCTGGAGCGAGGGATGAT
GCACCAAATTTTGTTAAAATCCTATAGTTAGCTGAGAGTAGCCTGGTATCTCCTCACTCTAAAAGATGGTCGGTGGAATCCGGCAGGCTGGGGCCGCGTC
AACAGTGGTAAGTTCTTGAGGTGTCTGTCTCTCCCTGTGCAGGGGTCAGGAAAAGCCACACCGATGTGCACCCTTATCTTGGAGCAACTGGCTGGGATCC
ATCATCATCCCCTGTACCACCGCCCCCTACCCTTTTGTGTTTGGAATTCCTTTGTGTTTGCTCACTCATATCCACTGAGGCCCTTTATCATCCAGAGCAT
TCTCACTTGAGAGGACCGCCTTTTTTCCGTTTATCCCACTGTAATCTGTAGTGTACCAGTATCCATTTGCATTTGACATTGGCAGTGGACTGTTTTGCCG
CTGTTGTTGTAGTGTTTGTTTCAGCCTGACGATTTGTTTCTTGCTCCTGTGAATGCAGTGCGTGGAATAATTTGCGGTTGTTATCTGCGACATTCTATTC
TCTGGTTTTGAATTCTTGGTGTTAGCCGTACCAGTTTTACGTGCTTGTTACTGAGCAAGAAGGCACCAGTACTCGTGCATTTTAAGGGCGTGCTCAGTTT
CTGATGCCATTTTTCTCGCTTACATCATCTTTCTGTATTGCTGCCCTCAAATATGAATTCAAATTAACGGTGAAATCCAAAGCAACTTCTGAATCAATCC
CG
>Locus_7685_Transcript_9/54_Confidence_0.085_Length_2115
AGCGGCGAGAGCTGGGATGCTGTGCTCGGCAACGCCGCCGCGGCGGGACAGGACCAGTCTTTCTTGAACTGGATCATTGGGGCCGCCGGCGACCTGGATC
AGCAAGGGCCGCCTCTTCCGGTGCACCAGCAGCCGATTCTTGACAATGCGGGGTTCGGTTTCCCGGCCACGGATCCCCTGGGCTTCTCGCTGGACCCCCA
CCTCGGCGGCGTCGCTTCCGACCTGTCGTCCCCTGGTGCGGTGTCGCACACTACCAACAGCGGCGGTGGCACCAAAGGGTCCTTAGCGTTCGGCCTCTTC
TCGTCAGAGTCTGCCTCCCTCCAGCCGCCGCCGCCTCCGATGTTGTACCACGAAGGTATCGACACAAAGCCTTCTCTTCTTGCTGCGCAGCCGCCCGGCC
TCCATCACCAGTACCAGCACCAGCCGCCTCCTCCCGCCTCAACTTTCTTCATGCCCCTCCCGTCCTTCCCTGATCACAACCAGCAGTCCCCACAGCTCCA
GCCACGGCCCAAACACCATCAATCCATGGCAGACGACCTCTACCTCGCCCGAAACCGGCTCCTTCCGGCAGCGGCGGCGCAAGGACTCGCCTTTCCACCA
TTACATGGCCCTGCTTCATTCCAGCTCCAGCCTTCGCCGCCACCACATCGTGGGGCGATGAAGACGACGGCTGTGGAGGCGGCGCAGCAGCAATTGCTGG
ACGAGCTGGCGGCAGCGGCAAAGGCCGCCGAAGCCGGCAATTCCACTGGCGCGCGAGAGATATTGGCGCGGCTCAATCACCAGCTTCCCCCGCTCGGGAA
GCCCCTCCTCCGCTCCGCCTCCTACCTCAAGGAGGCCCTCCTCCTCGCGCTCGCCGAAGGTCACCACAGTGCCTCCCGCCTCACCTCGCCGTTCGACGTC
GTCCTCAAGCTCGCGGCTTACAAGTCTTTTTCTGACCTGTCGCCCGTGCTCCAGTTCACCAACTTCACCGCAACGCAGGCACTTCTTGACGAAATTTCCG
GCAGCGCAGCTTCCTGCATCCATGCCATGGACTTTGATCTTGGCGTCGGCGGCCAGTGGGCTTCCTTCTTGCAGGAGCTCGCTCACCGACGCTGCACTGG
CGGTGCAGCTCTGCCTTTCGTCAAGCTCACTGCCTTTGTATCAGCTGCTTCCCACCATCCACTGGAGCTGCGTCTTACCAGGGATAACATCTCGCAGTTT
GCTGCTGACCTGGGTATTCCTTTTGAGTTCAATGTCGTCAGTGTTGATGCATTCAGTCCTACGGAGCTCATTTCTCCCACTGGCGATGAAGTTGTAGCAG
TTTGCCTCCCAGTCGGTTGCTCTGCTCGTTCGCCGTCCCTGTTGGCGATCCTTCGGTTGATGAAACAGCTTGGTCCTAAGATTGTGGTGGCTATAGACCA
TGGAGGTGATCGTGCTGACCTTCCGTTTTCGCAGCACTTCTTGAATTGCTTTCAGTCGTGTATGTTCCTCCTTGATTCGCTTGATGCTGCCGGCATTGAT
GCTGATTCTGCCTGCAAGATTGACAAGTTTCTGATTCAGCCAAGAATTGAAGATGCGGTGCTTGGGCGGTGCAAGGTGGACAAGCCAATGGCATGGAGGA
GTGTGTTTGCAGCTGCAGGGTTCGTGCCTGTGCCGCCCAGCAACCTGGCGGAGGCACAAGCCGACTGCCTCCTGAAGCGGGTGCAGGTCCGCGGCTTCCA
TGTGGAGAAAGGTGGGGTTGGGCTTACCCTCTATTGGCAGCGTGGCGAGCTTGTCACCGTATCCGCATGGCGGTGCTGATCAGCAACCTCGGCCTGTGCC
CGCCAACGCTTCACCACCATTTGCTGGCATCTTCATCTCCAGTGATGTGTATTTTGTCTTGCAATTAAATGTTGCAAAGGAAACTAATGTCTTGGTGTTG
AACACTTAATGTTGCTTCTCTTTGCAGCCTATTAGCTAGGACTAGTGAATTTAGCAATGTTGTTGCCAGTGTCTCTAGTGTAATGATGTTGCTCTAGGTT
CTTACCACCGCTCCCTCTCTGCTCACTCCACCATTCTCATGTCATCTGAAATACTGCTTGTGAGTAGAGTGGATAGTTAACCTATCAAATGTTTCTGCGG
TACAGTGTTAATATC
>Locus_5417_Transcript_13/28_Confidence_0.281_Length_2478
AGGATGGGGCGAGGCAAGATAGTGATCCGGCGGATTGACAACTCCACCAGCCGGCAGGTGACGTTCTCGAAGCGGCGGAACGGGATCTTCAAGAAGGCCA
AGGAGCTGGCCATCCTCTGCGACGCCGAGGTCGGACTGGTCATCTTCTCAAGCACCGGCCGCCTCTACGAGTACGCCAGCACCAGCATGAAGTCAGTGAT
TGATCGATATGGCAGAGCGAAGGAAGAGCAGCAGCTTGTGGCAAATCCCAATTCAGAACTTAAGTTCTGGCAAAGGGAGGCAGCAAGCTTGAGACAACAA
CTGCACAACTTGCAAGAAAGTCATCGGCAGTTGATGGGACAAGATCTTTCAGGATTGGGCGTCAAGGAGCTCCAAAATCTAGAAAATCAGCTAGAAATGA
GCCTACGTTGCATCAGGACAAAAAAGGACCAACTCTTGATTGATGAAATTCATGAACTGAATCGAAAGGTTCTGGAATATTCTGTTATTACTCTGCAATG
AGAGGTCTCTGAGTGGATTTCAAGAAGCTGACTCTACTGGCTCATTTTCAGGGAAGTCTCATGCAACAAGACAAGATGGAACTATACAAAAAGGTCAACC
TAATCCGTCAGGAAAACATTGATTTATACAAGAAGGTATTTGATAAATCGTGAATGAAAATTCACAGGATATACACCCCATCCATTATTGACTGAGGTTT
ATTTCTCTACTCAGCTCTACGAGAAAGAGGTGACAAGTGAAGTCAACCGAGATTCATCAACTCTAAACTTTGCAGTTGTCGAGAACGTCAACATTCCTGT
TCATCTTGAACTTAACACTCCACCGCAAGAAAATGATGTTGAGCAAACTGCACCTCCTAAACTGGGGTAACCCTGCTTTTTTCTGCAAAGGAACTATTGA
TTCTGTTCTTAATTCTTTAGTGGCATATTGTTTACTACTAACTGAAAACTTGCAGATTACAACTAAATCCATGAAGGCATGCAGAATGGCACCGCTTATG
TTCATTATTCTCAACGGTACGATTCAAAGCAACCAGAAACACTATTGGTTGTAAAGAGCTTAAACACATAGCAAGACTGACGCGGTATGCATGCCATGAG
TGATTGATAATGGTAGTGTAAATCTATTTCAAATAAAATGTATTTTATTGAAGAATACAGCAAATGATTTGTATTATAACTATTTGACACATTCTGCAAG
TTTGTTGACCAACGTGAGTACATGATAGCTACAAATCTTATATTGACAAAAAGATATGGTGTGTGGAAAAAATTCAGGTGCTATTTTTTTCTATTTTGAG
AATTGTATGCATAGCAGAAGAGCACAAATTGCATTCTGGTTTATCCGTGCAGGCATTCCAGAGTATCAGAATGGGGATCAACCTTTCTTCAGCTACATAT

GTAAATATACGGATGCTAGTTGCTCACTTAAAATTTTGTAGTGTTCAAAGGAATAACAACAGTAAGACGTTGACGCACCGAAAATAACCAGAAATAACGG
ATTGTACTATGACAAGACACACGACAGATACACTAGCTTAAGTTTGTACAGGAAAATATTTGGCCTATTCTTATGTCACAGTAAATTAAGATTATCACCT
TAATCACAGAAATTCAAGGGGTAAACGATTATAGCACAAAACGATCCTGGACCCCAAGACCTGGGTCTCTGAACGGCTGAACCTCCAATTATACCTCTAT
ACATTTGGAATGTGTGTAGTCAAACTTTCGCTACTGGTATCTTTACAAATATTTACATTGAACACATCAAAATGATATACATGGATTCCTCTCGATATGT
CTTTGAGGTTTGGGTTCTAAAAATGATATACAAATTATTAGGTTCTAAACTTAAATGATATATACACATCAAAATGATATACAAATTATTGGGTTCTAAA
CTTAAATGATGTATACACATCGAAATGATATACAAATTATTAGGTTCTAAAAATATACTATGGCAGAATTAGGTGGTCATTTGAGTTTTGGAGGCCTTGT
CAATGTCCCAAACATCACTTATTTGTGACTGGGAGAGCAATGAATTGAAGGAATGAGAGTAACCATACGGTCATTGGTCAATTAAAAGGAACTTTAATAT
GCTATGACCACATCGACCTTTCGTAAAGTAAATAACTCAATTATAACCAAGTAATTGGGTGTCTATTGAATCCATACGGTTCTCAAAACAGTTCTAAACA
AAGTGAATTTAAGCATCCTGAAAAGATATTTGGCAAATTTGCCATACCCTGAATTCACAGCATGTTAACACAAATCATGTGCAAATCATTCAGATCATTG
ACAAACGCACGCATGCACACAAATCAACACCGTTTTCTCATGTATAGAACAGAAAAATTGAGAAACTTACGATTTTCTTGCAAGTTATGCAGTTGTTATC
TCTAGCTTGCCGCCTCCACTTGCAGTAACTGAGACGCCGGCGCCGAGCTTGAGCTTGAAACCTAGACTACGGGGACAG
>Locus_13957_Transcript_44/58_Confidence_0.247_Length_1311
GCCGCGGGCCCCGCTCCCGCAGCTCGCAGTACCGCGGCGTCACCTTCTATCGCCGCACCGGCCGCTGGGAGTCCCATATTTGGGATTGCGGCAAGCAAGT
GTACTTAGGTGGATTTGACACTGCTCATGCTGCTGCAAGGGCGTATGATCGAGCGGCTATCAAGTTCCGTGGTGTCGACGCCGACATCAACTTCAATCTC
AGTGACTATGAGGACGACATGAAGCAGATGAAGAGCCTGTCCAAGGAGGAGTTCGTGCACGTCCTGCGACGGCAGAGCACCGGGTTCTCACGGGGCAGCT
CCAAGTACAGAGGCGTGACCCTGCACAAGTGCGGCCGATGGGAGGCTCGCATGGGGCAGTTCCTCGGCAAGAAGGCTTATGATAAGGCCGCGATCAAATG
CAATGGTCGAGAAGCTGTGACGAACTTCGAGCCAAGCACGTATGATGGGGAGCTGCTGACTGAAGTTGCTGATGAAGGTGCTGATGTTGATCTGAACTTG
AGCATATCTCAACCAGCTTCTCAAAGCCCGAAAATGGACAAGAACTCCCTTGGTCTGCAACTCCACCATGGATCGTTTGAAGGCTACGAATTGAAAAGAA
CAAAGATTGATGCTCCCTCTGAACTCACGGGCCGACCTCATCGGTTCCCTCTTCTGACCAAGCATCCACCAATCTGGCATGCCCAATCTCATCCCTTCTA
TTCAAATAATGAGAATGCATCAAGAGATCTTAACAGGAGGCCTGATCAGGGAGGCACTGTTCCTAGATGGGCGTGGAAGGTGACCGCCCCTCCTCCCACC
CAACCATTGCCGCTGTTCTCGTCATCGTCGTCGTCGTCCGCTGCAGCATCATCAGGATTCTCCAATACCGCCACGACAGCTGCCCCCACCGCCCCGTCGT
CCTCCTCCCTCCGGTTCGACCCGCCGCCGCCGTCGAGCCGCCACCGATGAACATTGAGAAGCCACGCTGTAAATTTGCCGGGAAGCCGGCATTTTTTCCC
TCTCCAGGCGTTGCAACTTTTTCGGTTTTGCGCCGGGGTGGTTTCTTAATTGTAGTGGATTGGATTCATAACTGTATTTGCATACTGCCCCATGTGAAAT
GCTTCGTTCTCACTCTCTCTTTACACACTCTGCAACGGATAGATGTTCTTGTTGATGGAAATTAGCTAACTTCACCGTCTGAGATGATCTATACATTGTT
GTAGAGAATCGAATCATTGTTGAAATTAGTTACAGAATTATAGATATCATAAGGCACCCTCTACTTGTGGACAAAGTTCAGGAAGATTATTCCGGTTCTT
TTCTTGGCTGT
>comp81084_c0_seq2
AAATGAACAGTTGTTCCGAACTCTAACCGTGTTAAAACTGGAAATCCAAAAACTGGATGCATGTCTTAAAATCTAAAAGCGGGATGCATGTCTGTAATAA
CATTCCATGAAGCAATTTCAAAGCTTAAACCCTTTTAATCATGGAAGCCAAAATCTATTTTCCAGGGATTTCACCAGTTAACAGACCAGAGCAGAACTGT
GCTCTAATTTATTGGTCACCTGTACTTGCGTTTTTTAATGCACCAAGGTTCCTTTTTAGAGACTTGACAGCAGACAAAATCTGCAGCTTCTCTGCGGACG
GCCCGTCAGGTTTCTGATCATCGTTCCCAGTCGCAGCATCATCAGGTGGCAACGAGTCTCCCAAAGATGATCCCTCCACATGAGGTTCCCTGTCCAGCTG
GCCTGCATGTGCCCCTTGCTTCCCAGCTTGCTTTCGCATGAAGGCCGCACTCCTCTTGTACTGGGGATCAGTAGGATCCAATGCGAACAAATGTGACGTG
AACATTGCTGAGAACCTCTCGTCCTTGCTAAGATCGATATCAGGAAGTTTGTCCTCAACCGAGTCCTCTTTATCCTTCTTGCCCTTTTTACCCTTTTTGC
GCTTCAAATTATAACCCTTTGCAACATTGGCAGTATCCTGGTCAGCAGCAACCAAGAGCTCCAACTCTTCTTTGGTTGCTTCTTGCTCCAAATGTTCCTC
TGGGAATTTGTCCTTTCTTTTCCCCTTTGCTTTATCTTTGGTCTTTGCCTTTTGCTTCTTGCTTTTGCTTGGCCTAGTTTCTTCATCAGAAAATTCCTCA
GCGAAGAAGTCATCATCCTCATCAGGCTCATCTTCACTGCTATAGTCATCATCATGCTCCTTCAACCCCCTCTTCCTAGCTTTCCTTTTTTCTTTCATTT
TCTCTTGGTGCTTCTCCCAGACAGTTTTCTCCTCATTGGTCTTTCTCTCTAGGATACGTTTACTGAGATCCTCAAGCTCTGTGTTGAATGTAATCTCCAT
ATCGTGGCCATCAGTTTCTTCATCATCAGATTTGTCACCTTGCAGTAAAAGAGCCAACCTCTCCTCTTTTGTTAGCTTTCGTTTGGATCCCCCATTTGGT
AGAGACTCATTATCAGGATTGTCTACACCGTCATCATCTGACGCACTGTCATCACTTGCTAAATACATGTCAAGCTCATCTAACTGATCATCAGTGAACT
TTCGCCTCAAGACCTTCTTACGTTCTGGTTCATCATCATCCCAAGTTAGCTTAACTCTGCTATGTTGCAGAGCACGAGTCTCAAAATCAGGCTCCTTGTA
ACTCGGAGGTGCCTCTGTAGCAATGTCTCGAGCAGGATGTTTGAACTCCATAGAATCAGGTATAAACTGCAAATCAAATACATTTGCTGTTTTCAAGAAC
TCAGTACCATCAAGATTCATGTACAGGTGATTTGCTGTTGCACTAGAATCACAGACCACAACAGCATAATAATACCTCAGTCTGTTTAGCTCATAAGTAC
GCAACTTGTTATTCTCTTTGTCGGAGTCAACTTCTTCGTCGTCGTCATCATCAGTGTTGTCTTCATCATCATCGTTGCTATCATCGTCGTTGTCATCATC
ATTATCATCAATATTGGCATTAACAAGAGCAGATGGGCCCTGAGTTGACTCAATTTCCATGCACTTCAGTCCAAATTCTGATGGATATATTGAGACTGAT
AAAACTCGCCCACCTTTTGGGACGCAAGATGTCATCACCATATACAGGTCAACTGCCTTGATATGATCCCAGTCCATATTAACAACAGCAAGCCTGTGAG
TTTCCTTGTCAATCATAGGCGTATCATCGTGCCGGCCCATTAAGTAATGAGCAATGTCACTGCCGACAGAGTACTCGTCATCATCAACGTCTTCATCCTC
GTCGTCGTCGCTACTAGAACTCTCTTCTTCCTCCTGCTCATCCTCTTCATCCACGTCCTCGTCCTCCTCTTCTTCAACTAACTTCTCTTTCTCCTTCTTT
GCCTTCTCTTTAGTCTCATCCTCCTCCTCGTCTTGGTTGAGATAGTAACGGAGCATGGGGTTCTCCCTGCCGCTCTTCTTCCGTCGCCTGCCGCGCTTGT
CCACCGGCGCCGCCGAGGAGGAGAACCTCGGGTCCGTCATCATGCTGCTGAACCTCGAGTCCAGCGCCACCTTCGCCTCCTTCCGCCGCATCGACCGGAA
CCGCGGGTCCATCCGCGCGGCCGCGAACCGCTCGTCCTGCATCGCCCGCTTCATCCTCTCGTCCCGCCCCTCCACCGCCTCCGCTTCAGCCGCCTTGCCC
TCGTGCTTCCCCTCCTTCTTGGGCTTCTTGCCGTGCTGCCGCTTCTCGTCCTCCTCGCGCCCCTCCTTGTGCTTCCGCTTCTTGCGCTCGGCCTCGGGGC
GAGGGGCTTCCTCGGTGGCCGCCTGACGCTCTTGATTCTTCTTCTTGTGCTTCCTCTCTTCCTTATCGGACCTCGATTTCTTGGCCTTCTTGAGGCGCGC
GGGCTCGTCGGAGCCGGTCGGCGG
>Locus_14087_Transcript_55/66_Confidence_0.235_Length_1931
ACGATTCATTGGTCTTTTATATAGGTTGAGGAACCGGGAGGCGTAGGATAGAGAGATCTCAAGGTCAGGTGAGAGCTCAGTGTAGGTCAATGGCGTCCGT
GCCGTCGCTGGTGCTTCAGTTGCCAGTCAATTGATGCACCGTGGTTACTGGTTCAGCTCGCCGAGCAGGTGGATTTCAAGTGCTAGCAGTTTTTACCTGT
TCGGGCAGGAGCAATAGTGGAGATCTTTAGTTCTGGCATGGGTTCCTTTGGGATGAACTGGAATCAAAAGAACTCCATGGTGTGGGATTGGGAAAATTTA
GCACCGTCTGTTCCAAATGCAATCGGAAACCCCAGGAATGGAGCTACGAGGCATGGATCTGTGAATTCATCTGGTGGCACTCTCACTTCTAGCTCAGAGC
TAGGGCATGGTTCATCCAAGAGCTCCATGTCGGCATCCATTGATTCACCGTCCAAAGTAGGAAACAGCTTGGAGTTCAACTTTGCTGCTGTCAAAGGCCA
TGTTAAGAACATGGATAAGGATTTTAGATTTGACGACTTGGGGACTTCTCCATCGTCGATGATAGCATTCAGCCATGGAGAGCCATTAATCAGCCTTAAG
CTTGGAAAAAGGACTTACTTTGAAAATGTCTGCGGAGGGCAGGATTCCAAGAGCACTGCACCTTCTACCAGGACTTCTCCATCAACCGTTCTCAAGAAGA
CCAAGGTGTCTCAGCAGAATACACAAATGTCATACTGTCAGGTTGAAGGATGCGGAATTGATCTGTCCTCCGCTAAAGATTACCACCGCAAGCACAAAGT
CTGTGAAGCTCATTCTAAAGCCCCGAAGGTTGTTGTTGCTGGTGTAGAGCGCCGTTTTTGCCAGCAGTGTAGCCGGTTTCATGGCTTAGCCGAGTTTGAC
CAGAACAAACGAAGTTGCCGTAAGCGTCTTACTCATCATAACGCACGAAGGAGGAAACCACAGGCAGATACAATTTTATTCAGTTCATCACGTCTCTCAA
CAATGTTTTATGATACAAGCCAGCGGACAAATCTTTTCTGTAGTCAGCCTCTTTTTGGCCAAGTGAGAAGCAATGCAGTTTCTTCATGGGATAACTTGGG
AAGCTTCAATTTTATGGAAACAAAACATCTGTTGATGCAGCCAACGAAAACGGCAGCCCCTGATGAGCTGCATTTCTCAACCCCCCAGATGTCAAATGGT
GTTATGGTTCACGCTGTACATCATCATAATGTCGGTGAGTTCATGCCATTCAAGGATACCACCACAAAGATCCTCAACCAAGGTGTGGAAGCTTCTGCGA
TTGGATCCAACTCAAATGGAGACCCGGATCTTGGGCGTGCTCTCTCTCTTCTGTCAAACGGTTCGTGGGGTTCAACTTCAGCCGTCATCCAACAGCCTAG
TTCTCATGCGCAAGCTGGTGCGGTGCCACCCTTCGCCACCGTCGCCGTCTCCAACCCTGCAATGCATCCTCTGGACTCATCCCCGGGAGGATTCTGGCAA
GACGTCCCTCCCCCGCTCGATGAAACTCCACATATTCAGGCATTCACGCACCTCTGATCAAATGCTGCATCCCCTGGGAACAGCAGGTGGTTATACCTTC
CGTAATCGTCCTAGGTTTGCTTTGCCAGTGACTGGCTCTGCACAATGTCGATCCATCACCGGAGGCATTTGGTAAGGTGAAGACTAAAGACTCAAGAATA
ACCGGAGACTAACTCGCTTCTCTTCCAGGCTCAAGACTGTTTGGGTCCAGCGGTGTTGCTACCTGATCCTGTTTTCGGTTCTGAGTACATCCATTACTAA
TCATGGGATTACTTGTTTAGGCCAACCAGACATATTCTGGGGTGAGATTACGACATGTTAATTTACATTGACGGTGTTTCTACATGTGTTAGTACTAATG
ATGTTATCGCTCTTGGCCCCGTTACGTTTCA
>Locus_18892_Transcript_30/56_Confidence_0.250_Length_1236
ATCTGGGATTGTGGGAAGCAAGTCTACTTAGGTGAGTAACTGAGTTGAGTGAGTTCTATACGTTCCAGCTCGAGCTTCCATTAACTGCTGATCAGTTTCC
GTCCGTTTGATCTGGTTTACGATGTGCTGATGCTGTTCTATTCAGGTGGTTTCGACACTGCTCACGCGGCTGCGAGGGCTTATGATCGTGCAGCGATCAA
GTTCCGAGGGCTCGACGCGGACATCAACTTCACTTTGAGCGACTATGAGGATGACTTGAAGCAGATGAGCAATTGGACAAAGGAAGAGTTCGTGCACATA

CTCCGCCGCCAGAGCACGGGGTTCGCGAGGGGGAGCTCCAAGTACCGCGGCGTGACGCTGCACAAGTGCGGCCGCTGGGAGGCGAGGATGGGTCAACTTC
TTGGCAAGAAGTACATCTATCTTGGACTCTTTGACAGCGAAGTTGAAGCTGCAAGAGCATATGACAGGGCAGCCCTTCGCTTCAATGGGGGGGAAGCTGT
TACTAATTTTGGGCCTAGCTCCTACAATGGAGGAGATGCTCTACCCGACACCGAAAATGAGGGTATCACAACCAGCCTCGCCATTTATTTCTTTTCATAT
AAGCAAATGTATCTCAGGTGCACCTTATAATCATCGCTGTGTTCTTAGCAACTGTTGATGGTGACGCGGTTGATTTGGATTTGCGGATTTCACAACCTAA
TGTACACGACGCTAAAAGGGACAATACCGTAGCTGGCTTCCAGCTAACATGCGACTCCCCTGAATCTTCAAATATGATGGCCTCTCAGCCAATGAGCTTA
TCGTCCCCGTCGCCTGTCTATCACCTAAGCACAGCAGTACCACCTCACCATCAGCGTTTGTACTCATCTGCTTGTCCTGGCTTCTTTCCGAACCTCCAGG
AAAGGCCAGTGGAGCGAAGGCCTGATTTGGGCCCCCAGTCGTTCCCCACCTGGGGACGGCAAACGCAGGGCTCCCCTCACATGCCGTTGCACCACTCCGC
AGCATCATCAGGATTCTCTACCGCCGCGAACGCCGGCGTCCCATTGCCGTCTCACCCGCCGGCGCCGTTCTCGGACCACCAGTTCTACCTCCCACCGACG
GCCTGAGCTTGTTGGTCCGGTTCAACTCTTGGGACAGCAGCTGACGCTCTGTGTGTGAACGTCTCGCAGCTTGCCGGTAACAGCTGTTAATTAATGGGAG
AGAGAGAGAGAGAGAGAGAGAGAGAGAGAAAAGAGCCG
>Locus_3441_Transcript_38/417_Confidence_0.037_Length_916
TGTAAATCTCTTGATTGAAATAGTGGCATGTCATCTTATTTAATTCAGTTATTTCATAAGGTACAATTTGTTTAGCTGACAGCTCAGACATGCTTCTATG
CAACATGTTTATACCAATGCTGTATTTTGGCAATCCTGGATGTGGCATTACTTAACTGTGCTTTCATATTCAATAGTTCTGGCAAAGGGAGGCTGCAAGC
TTGAGACAACAACTGCACAACTTGCAAGAAAGTCATCGGCAGTTGATGGGACAAGATCTTTCAGGATTGGGCGTCAAGGAGCTCCAAAATCTAGAAAATC
AGCTAGAAATGAGCCTACGTTGCATCCGGACAAAAAAGGACCAACTCTTGATTGATGAAATTCACGAACTGAATCGAAAGGTTCTGGAATATTCTGTTAT
TACTCTGCAATGAGAGGTCTCTGAGTGGATTTCAAGAAGCTGACTCTACTGGCTCATTTTCAGGGAAGTCTCATGCAACAAGACAAGATGGAACTATACA
AAAAGGTCAACCTAATCCGTCAGGAAAACATTGATTTATACAAGAAGGTATTTGATAAATCGTGAATGAAAATTCACAGGATATACACCCCATCCATTAT
TGACTGAGGTTTATTTCTCTACTCAGCTCTACGAGAAAGAGGTGACAAGTGAAGTCAACCGAGATTCATCAACTCTAAACTTTGCAGTTGTCGAGAACGT
CAACATTCCTGTTCATCTTGAACTTAACACTCCACCGCAAGAAAATGATGTTGAGCAAACTGCACCTCCTAAACTGGGGTAACCCTGCTTTTTTCTGCAA
AGGAACTATTGATTCTGTTCTTAATTCTTTAGTGGCATATTGTTTACTACTAACTGAAAACTTGCAGATTACAACTAAATCCATGAAGGCATGCAGCATG
GCACCGCTTATGTTCA
>comp67351_c0_seq30
GGCAAGACGGATTACGACGTGAGAGGCGAGAGTAGCGAAGAGTCACGGAGATCAGGGGAATCAAAAGATGGAAGTGTTGGTCAAGAAAACAACCATGCAA
CATCACATATGCCTGCTTTGGTGTCGGAGTATTTAGCACCATATACACAGCTGGAACTGAACCAATCAGTTGTGTGCAATCTTTTCTCCATGGACTCTGT
TTATTGCCTGTGTTTCCATTTTCTGTTGACTTGTCTCTAATGAGCTTATCGTCTCATGTTCACATCTTTCCTTGTGTAGGCTCCTGCAACATATCAGTAC
CCAGATCCTTACTATGCAGGCATGACCCATTTCCAGCTACCTGGGTTAACTCACCCTCGCATGCCATTACCTCTTGAAATATCTGAGGAGCCTGTCTATG
TAAATGCAAAGCAGTACCATGGAATTTTAAGACGAAGGCAGTCACGTGCCAAGGCTGAGCTTGAGAAAAAGGTGGTTAAAGTCAGAAAGCCTTATCTTCA
CGAATCTCGCCATCAACATGCAATGAGGAGGGCAAGAGGAAATGGGGGTCGCTTCCTAAACACAAAGAAAAGCGACAATGGTACTCCCAACGGTAAAGCT
GAACCTAAGAAAGGAGAGCAAAACTCCAAGCATATCCATGTCCCTCCTGATTTGCAACTTCGACAGAAGGAGGCATGAAGTAGCGGTCTGGCAACCTAAA
ACAGTGGCTTCTGTCCACAGGCATACACACTTTTGAGTCCGGATTTGGAGCCCCAGGGTTGCGCTGCCATCTTTGCAGTTCGTCTTCTTTGTGCAAAGAG
GTCCAAGGTTCGTCCACCCGAATGAGCTTGGTACCAGTTGTTAGTCCTGGCAGAATGCAATCTGGGTGCAGGAGACCGTTGAAGTGTCCAATGCCATGGG
TAGGTCTGCTGTTTTCAGGCAATTCATTCTTGGCTTTCACATTCCGCTCTTGTTTGTACCTGGTGTGTTGTAAGTGGAACTAGCTTGTGTTGTGTCTTCG
GCGTCCTGTGAGACATGTATAATTAAGATCAACTTGGGATCATCGTTGTAATCCTATGTCTGGCTTTCTTTTAGTTTTGAACTGTAACAAGGCCAAAGCT
GAACTCTTTATACAGAGATAGCAACTCTTGCTGGCCTGTTCGTTGTTCAGCAACGTCAGTGCTGTACTTAATCCTGTGCAAATGAATAAGCTT
>Locus_11912_Transcript_55/63_Confidence_0.305_Length_2951
TTTTCCTGGCGCCTCCCGCCGGGCGCTCGCCCCGGATTTCTCGGCTCCCCTTTCGGTGGATGCCTTGACCGGGGTGGGTTCTTGGCTGCGGCGGCGCGTG
ATCTCTGCGTACTTAAGGAGGGGTTGCTAGCGGGGTTTGTGGGAGAAGGCGAAGGAAGCCGGGGGCTGCGAGGCGTTCATGGGTTAGATCTGGCCGTCCG
GCGCCGCAGATCTGAGCGAGGTTTCGGTGGAGGGATCGGGGCCGAGAGGTCGCCGTCTTCGGCTCATTTGGAGTAGCTTGGGCCTCTCATGTCCAAATCG
GGTATGGATGGTGGATGCGGTAACTGTCGGAAGGAGGGCTTGTTGCAGATCTGAAAGCCATCCAGCTCCCTTGTTCAGTTTACCTGCTTGCTGGTCAGCT
GGATGGAGTGAATGGCAATGCTTTCTGATGTTTGACTGCCGTTAGTTGCTGCTTGGTGTTTATTCAAGAAACTTATCAGGAGGTGGTGTGTGCCCCGGTA
ACTATTCAGTTGCTGATATCTTGGCCTGTGTTGGATAACGAGGTAGCAGATCACATTCCTGTGTTTTTGTTGATATCTTGGTTGTTCTTGAATAACCTGG
AGGCTGGAAGGGGTCAGATCCAGTTCCTGTGGCCCTGGTAATAAGGTGCTGTTGGGGGAAGGCACCATGACCTACTTCCCTGAGGAGGTGGTGGAGCACA
TCTTCAGCTTCTTGCCATCGCACAGCGACCGGAACACGGTGTCCCTGGTCTGCAAGGTGTGGTATGAGGTCGAGAGGCTGAGCCGGCGAGCTGTCTTCGT
GGGGAACTGCTACGCTGTGCGCCCGGAGCGGGTGGTGCTGCGGTTCCCCAATGTGAAGGCCCTGACGGTGAAGGGGAAGCCGCACTTTGCGGACTTCAAC
CTGGTGCCGCCCGATTGGGGTGGCTACGCGGGGCCGTGGATTGAGGCGGCAGCGAGGGGCTGCGTGGGTCTGGAGGAGCTGCGGATGAAGCGGATGGTGG
TGTCGGATGAGAGCCTTGAGCTGCTAGCTCGGTCCTTCCCGAGATTCAAGGTCCTCGTCCTCATCAGTTGCGAGGGGTTTAGCACCGATGGGCTAGCAGC
TATTGCGAGTCACTGCAAGCTCCTGAGGGAGTTAGATTTGCAGGAAAATGATGTGGAGGACCGTGGGCCTAGGTGGCTCTCCTGCTTCCCTGATTCCTGC
ACCTCTCTTGTCTCCTTGAATTTTGCCTGCATCAAAGGGGAGGTGAATTCTGGTTCATTGGAGAGGCTTGTTGCTAGGTCTCCAAACTTACGCAGCTTGA
GGTTAAATCGATCTGTATCAGTAGATACACTCTCAAAGATATTAGCATGCACCCCTAATTTGGAAGACTTGGGGACAGGGAATTTGACGGATGAGTTCCA
AACTGAATCATACTTCAGGCTGACTAGTGCTATGGAGAAATGCAAAATGTTGAGGAGTTTGTCAGGATTTTGGGATGCTTCTCCTGTTTGTGTTCCGTTT
ATCTATCCCCTATGTCATCAACTAACAGGACTGAACTTGAGCTATACTCCCACACTGGATGCCTCTGATCTCACTAAAATGATCAGCCGCTGTGTGAAAC
TCCAACGTCTTTGGGTACTGGACTGCATCTCAGATAAGGGTTTGCAAGTGGTGGCCTCCAGTTGCAAAGATCTACAAGAACTCAGGGTGTTCCCATCAGA
TTTTTATGTTGCTGGTTATTCTGCCGTGACAGAAGAGGGGCTTGTTGCAATATCGTCAGGCTGTCCAAAATTGAGCTCTTTGCTGTATTTCTGTCACCAG
ATGACCAACGATGCACTTATTACTGTAGCTAAGAACTGCCCAAATTTCGTAAGATTTAGACTCTGTATTCTTGAGCCAAAGAAGCCAGACGCCATGACAA
ACCAGCCGTTAGATGAAGGCTTTGGTGCAATTGTTCGTGAATGCAAGGGGCTAAGGCGACTGTCAATGTCGGGTCTTCTCACTGACAAGGTTTTCATGTA
TATTGGAAAGTATGCCAAGCAACTTGAGATGCTTTCTATAGCATTTGCTGGAGATAGTGATAAGGGGATGATGCATGTTATGAATGGATGCAAGAATCTA
AGGAAGCTGGAGATTAGAGACAGTCCATTTGGTGATGTTGCGCTCTTGGGGAATGTTGCCAAGTATGAAACAATGCGATCCCTTTGGATGTCATCATGCA
ATGTCACATTAAAGGGGTGCCAAGTCCTTGCGTCTAAGATGCCGATGCTCAATGTGGAGATCATGAATGAGCTAGATGGAAGCAGTGAAATGGAGGAAAA
CCATGGAGATCAATCTAAGGTGGAGAAGCTATATGTTTACCGCACAACTGCTGGAGCGAGGGATGATGCACCAAATTTTGTTAAAATCCTATAGTTAGCT
GAGAGTAGCCTGGTATCTCCTCACTCTAAAAGATGGTCGGTGGAATCCGGCAGGCTGGGGGGGCCGCGGCAACAGTGGTAAGTTCTTGAGGTGCCTCTCT
CTCCCTTTGCAGGAGTCAGGAAAAGCCACACCGGTGTGCGCCCTTATCATCTTGGAGCAACTGGCCGGGATCCGTCATCATCCTCTGTACCACCGCCCCTACC
CTTTTAGTGTTTGGTCACTCATATGGCCACTGAGGCCCTTTATCATCCTGTGCATTCTCACTCGAGAGGACAACGTTCTTTTTCTCCCTTTTATCCCAAT
TGTAATCGTAGTGTACCAGTACCCATTTGCTTTTGACATTTGCACAGTAGTGGATTGTGTTACCGCCGCCGACGCTGCTGCTGTAATAGTGTTTGTTTCG
GCCTGAATGATCGGGACATGACATTCCGGGGACGACCATTGGTTTCTTGCTCCTGTGAATGCATTGCATGGAATAATTTGAGGTTCACCTGTGACATTCT
TCTATTATTTGGTTTTGAATGATTGGTGCCCTGGCGAACGTTTTTGACTGT
>Locus_5983_Transcript_68/82_Confidence_0.096_Length_1080
CCATGGAGAGCCATTAATCAGCCTTAAGCTTGGAAAAAGGACTTACTTTGCAAATGTCTGCGGAGGACAGGATGTCAAGAGTTCTGCACCTTCAGTCGTG
ACTTCTCCATCAACAGTTGTGAAGAAGACCAAAGTGTCGGCGCAGAACACACAAAGCTCATACTGTCAGGTTGAAGGTTGCGGAGTTGATCTGTCTTCTG
CTAAAGATTACCATCGCAAGCACAAAGTCTGTGAAGCTCATTCTAAAGCTCCCAAAGTTGTTGTTGCCGGTCTAGAGCGCCGTTTTTGCCAGCAGTGTAG
CCGATACAAGCCAGCGGACAAATCTTTTCTGTAATCAGCCTCTTTTTGGCCAAGTGAGAAGCAGTGCAGTTTCTTCATGGAATAACTTAGGAGGCTTCAG
ATCTATGGAAACGAAACATCTGTTGATGCAGCCATCGAAAACTGTAGGCCTTGATGAGCTGCATTTCTCAACCCCCCAGATATCAAATAGTGCTGTGGCT
CATTCTGTACATCATCGTAATTTCGATGGGCTCATGCCATTCAAGGGAACCAACACAAAGGTCCTCAACCAAGGCGTGGAAGCTTCTGCGATCGCTTCCA
ACTCGAGTGGAGACCCGGATCTTGGGTGTGCTCTCTCTCTTCTGTCAATCGGTTCGTGGGTTTCAAGTTCAACCGTCATCCAGCAGCCTAGTTCTCATGT
GCACGCTGGTGTGGTGCCGCCCCTCGCCACCGTTGCCGTCTCCAACCCTGCAATGCATCCTCTGGACCCATCCCTGGGAGAATTCTGGCAAGACGACTCT
CCCTCGCTCGATGAAACTCCACAGATTCAGGCATTCACGCACCTCTGATCAAATGCTGCGTACCCTGGGAACAGTTGGTTAGACCTCTCGTAATCGTCCT
AGGTTTGCTTTGCCAGTGACTGGTTCTGCACAATGTTGACCCATCGCCGGAGGGATTTGGTAAGGTGAAGACTAAATATTGAAGAATAATAGGCCCCGTT
CTCTTCCAGGTTCAGGCTGTTTGGGTGCAGCGGTGTTGCTACCTGATCCTGTTTTCGGTTCTGCCATTAGAGAGCTGATT
>comp78487_c0_seq2

ACCCGATTTGTTTAGATTGCAATTAGACTAAGTATAGTTGGTGAGAAGACAGGAGTAGAGATAAGGGCCAACAGCCCAACAGAAAAACCAAGCAAGACTG
CAACTGCAAGAGAGATGGGGAGAGAGAGGAAACCTTGCAGAGGAGAGAAGCAAGGTGGGAAAGAGGCAGCAAGCGAGGCGGGCTGCAGGAACGGGAACAT
GCTCCCTCCTCATCTCACAGAAAATGGCACAATAATGATTCAATTTGGTCATCAAATGCCTGATTGCGACTCATCAACTACCCAATCAACCAGCGAGAGT
CATCAAGAAGTGTCTGGAATGAGTGAAGGAAGCCTCAACGAGCATAACGATCGATCAGGTAATCTCGATGGTTACACAAAGAGTGATGAAAATAAGATGA
TGTCAGCTTTATCTCTGGGCAATCCAGAAACTACTTATGCGCATCCAAAACCTGACCGTAGTCAGCCCTTTGCCATTTCGTACCCGTATTGTGATTCATT
CTATGGTGGTGCAGTGGCAACGTATGGCTCACATGCTATTATGAATCCCCAGATTGTGGGCATGATGTCGTCCTCCCGAGTGCCATTACCAATTGAACCA
GCTGCAGAAGAGCCCATTTATGTGAATGCAAAACAATACCATGCGATTATCCGAAGGAGACAGCTCCGTGCAAAGCTAGAGGCTGAAAACAAGCTGGTGA
AAAGTCGCAAGCCATACCTTCACGAGTCTCGGCATCAGCATGCCATGAAGAGAGCTCGGGGAACAGGCGGGCGGTTTCTGAACACGAAGCAGCAGTCAGA
GGCTCCAGGTGGTGGCACCTCAGATGCGCAACACATGGCCGGCAATGGTGGTCTGTTCAGGCTGCACGAGCACAACTTACCACCCAGTGATCTCCATTAT
GGCGCGAGAGGTGGCTCTTAAGGAGTCCCCTGGCAACTCATCCTTGGCTTAGTAACGTGTGGCGCTCAGCGATTCGACCTCGGCTTCGATGTAATGGTGT
ACGATTGTATCTATGGTGCTTGAATTGGCAGCAGCCATTCCTGCAGCAATGCGTCGCAGGTGTTGCCGGTTCTGTGATAGTGCCAGACTTGTGACCGGCA
CAGAACTGGCATCTGTTTCCTGTAACTTTAGTTTTTCTAAGACGATGACGATGACTACCCAATAACCATTCTGTCTGTATGGTGCCTTGCGTTTTCCGTT
CGCCCGTGACGGTGTTGCTCCTTAAAAGTCAGTGGCGGGAATTCTTCTTTGTCTCCGCGATGAACTGGTGAAGTTATTTCTTTTTTCTCT
>comp63680_c0_seq27
AGTATCTCGGCGTGGGACAGAGGCCGGTGGTGGTCGCGAAGAAGACGCGGCGCGGGCCGAGGTCCCGGAGCTCGCAGTACAGGGGCGTCACCTTCTACAG
GAGGACGGGCCGGTGGGAGTCGCACATCTGGGATTGTGGGAAGCAAGTCTACTTAGGTGAGTAACTGAGTTGAGTGAGTTCTATACGTTCCAGCTCGAGC
TTCCATTAACTGCTGATCAGTTTCCGTCCGTTTGATCTGGTTTACGATGTGCTGATGCTGTTCTATTCAGGTGGTTTCGACACTGCTCACGCGGCTGCGA
GGGCTTATGATCGTGCAGCGATCAAGTTCCGAGGGCTCGACGCGGACATCAACTTCACTTTGAGCGACTATGAGGATGACTTGAAGCAGATGAGCAATTG
GACAAAGGAAGAGTTCGTGCACATACTCCGCCGCCAGAGCACGGGGTTCGCGAGGGGGAGCTCCAAGTACCGCGGCGTGACGCTGCACAAGTGCGGCCGC
TGGGAGGCGAGGATGGGTCAACTTCTTGGCAAGAAGTACATCTATCTTGGACTCTTTGACAGCGAAGTTGAAGCTGCAAGAGCATATGACAGGGCAGCCC
TTCGCTTCAATGGGGGGGAAGCTGTTACTAATTTTGGGCCTAGCTCCTACAATGGAGGAGATGCTCTACCCGACACCGAAAATGAGGCAACTGTTGATGG
TGACGCGGTTGATTTGGATTTGCGGATTTCACAACCTAATGTGCACGACACTAAAAGGGACAATACCTTAGCTGGAGGCCAGCTAACATGCGACTCCCTT
GAATCTTCAAACACGATGGCCTCTCAGCCAATGAGCTCATCGTACCCGTGGCCTGTGTATCACCAAAGCACGACAGCAGTACCACCTCACCATCAACGCT
TGTACTCATCTGCTTGTCCTGGCTTCTTTTCGAACCTCCAGGTATTACCAAGCTAAAGCCTATTTTTTATGTGCAAATTGCTACTCCTACGTGATCAATT
AGTTCAAACTGTTATTCGAATCCAGGATTGTTTACTGAATATGTCCTGTCTTCAGAAAAAATAACACAAAAAGGAACATAATATGTCCTGTCTAATTGTG
GACTGATACTAGCCAATTAAACAGTTTCCCATATCCATTTCCTGTGTTTATTGTTTTGATTTGCTGGCATGGTTAAACTATAGTAGCTTGATTCGATAAA
AACACTAAGCAGGCTTTGATATTTTGAATGAAATGTCGTTCTTGGAAATACAGCCTTTTCCTTGGTATATCAAGGACAGTTGTCCATCTGCACTCTGCAC
CCTCAGCTTGTGTAACTGTTTGCCTTGTGAACTGATTGATGTGTCAATCAGGCAGTTCTGCCAGTTTGCATCTTTTGAAATAGTGCAGTCTTCCAGTCTA
ACAGCTCCATGACATAACTTGGGATGCATGCTGCAGCTTTCTTACTGAGGATGCTCTAGATTTACAGAAATTCTCGCAGTTAGCAGAGGGCTATCAAATC
TGATTTGCTTAGCTTCCTGCAGACAATAAGAACTCTAGAATCCTGAAGAAATCTTACTTGTACATGTTTACCAAAGTATCCTTACTCCTTAGTTGAGCCA
ATTTTGGTGAAGAACCGATAGCTCATTACTCTGTTTCCTGGGAATGATGTGGGCCTAAACCATTCTGTGGTCAAAGTTTAACAAAGCCCATGCTTGTGCT
GTTGCAACTGCAGGAAAGGCCAATGGAGCGAAGGCCTGAGTTGGGTACCCAGTCATTCCCCACCTGGGGATGGCAAATGCAGGGCTCCCCTCACATGCCG
TTGCATCACTCTGCAGCATCATCAGGATTCTCTACCGCCGTCGGCGCAAACGCCGGCGTCTCGTTGCCTTCTCACCCGCCGGTGCCGTTCCCGGACCACC
AGTTCTACTTCCCACCGACGGCATGAGCTTGTTGGTCTGGTTCAACTCTTGGGACGGCAGCTGATCTTATGTGTGAACATTTCGCAGCTTGCCGGTGACC
GTTGTTAATTAATCGGGGAGAGAGAGAGCCAGAAGACCCAGTATGGCTATCTCTCTGTCGCCCGTGCCTTGTTATGATCTTAGTTATTGCTACTACACCGTG
GATTCATCATGGTTTATGAGATTCTTTACTCAAGTTTCAGCTTAAATGTCCAAATACATGAATGCATCATACACAGGATCCGTCATTTTCTAAAAAAATA
CAGGATCTGTGTGAAGTGTAAACATAAGG
>Locus_14087_Transcript_45/66_Confidence_0.333_Length_2026
GATTCAAAATTGTAGCAGATTTGTCCTCTACCCACCCCTCTGAATCCTTTTTATACATACACTGACCATGGCACCAATGATCAAACCAAACACCAGCTGC
AAAGATCACCCTGGATTCCTGGAACAAAGAGGGCCTAGAGCAACGCAGCAACCTTCGCCACTTGAAGAGGATAGGTACAATGTGGCTTAGCTAGCACCTT
GCAGTCAGGTAGAATTATTCAACCGGATAAAGATTATTTCAAGTAAAGGAGGGCTACCAGTTTATACCTGTTCAGGTGGGTGCAGCAGTTGAGAACTTTT
TTTCGATACAGAGCAGTTGAGATCTTTAATTCTGGCATGGGTTCCTTTGGGGTGAACTGGAATCAGAAGAACTCCGCGGTGTGCGATTGGGAAAATTTAG
CACCTTCTGTTCCAAATGCAATCGGAAACCCCAGGAACGGAGCTACGAGGCATGGATCTGTGAATTCATCTGGTGGCACTCTCACTTCTAGCTCAGAGCT
AGGGCATGGTTCATCCAAGAGCTCCATATCGGCATCCATTGATTCGCCATCCAAAGTAGGGAACAACTTAGAGTTCGACTTTGCTGCTGTCGAGAGGCAT
GGTAAGAACATGGGTAGTGATGGTAGTAGAGTTGAGGACTCAGGAACTTCTCCATCGTCAGCGTTCAACCATGGAGAGCCATTAATCAGCCTGAAGCTTG
GAAAAAGGGCTTACTTTGAAAATGTTTGCGGAGGACAGGATATCAAGAGCTCTGCACCTTCAACAGTGACTTCTCCATCAATGGTTGTCAAGAAGACCAA
GATGTCTCAGCAGAACGCACAAAGCTCAAACTGTCAGGTTGAAGGTTGCAGAGTTGATCTGTCTTCTGCTAAAGAATACCATCGCAAGCACAAAGTCTGT
GAAGCTCATTCTAAAGCTCCCAAGGTTGTTGTTGCTGGTGTAGAGCGCCGTTTTTGCCAGCAGTGTAGCCGGTTTCATGGCTTAGCCGAGTTTGACCAGA
ACAAACGAAGTTGCCGTAGCGTCTTACTCATCATAATGCACGGAGGAGAAAACCACAGGCAGATACAATTTCATTCAGTTCATCGCGGCTCTCGACAATG
TTTTATGATACAAACCAGCAGAAAAATCTTTTCTGTAGTCAACCTCATTTTGGCCAAGCGAGAAGCAATGCAGTTTTTTCTTGGGAAAACTCGGGAGATT
CCAAATTTATGGAAACCAAACATCTGTTGATGCAGCCAACGAAAATGTTAGGTCTTGATGGGCTGCATTTCTCAACCCCCCAGATATCAAATAGTGTTGT
GGCTCACTCTGTACATCATCGTAATTTCGATGGGCTCATGCCATTCAAGGGAACCAACACAAAGGTCCTCAACCAAGGCGTGGAAGCTTCTGCGATCGCT
TCCAACTCGAGTGGAGACCCGGATCTTGGGTGTGCTCTCTCTCTTCTGTCAAACGGTTCGTGGGGTTCAACTTCAGCCGTCATCCAACAGCCTAGTTCTC
ATGCGCAAGCTGGTGCGGTGCCACCCTTCGCCACCGTCGCCGTCTCCAACCCTGCAATGCATCCTCTGGACTCATCCCCGGGAGGATTCTGGCAAGACGT
CCCTCCCCCGCTCGATGAAACTCCACATATTCAGGCATTCACGCACCTCTGATCAAATGCTGCATCCCCTGGGAACAGCAGGTGGTTATACCTTCCGTAA
TCGTCCTAGGTTTGCTTTGCCAGTGACTGGCTCTGCACAATGTCGATCCATCACCGGAGGCATTTGGTAAGGTGAAGACTAAAGACTCAAGAATAACCGG
AGACTAACTCGCTTCTCTTCCAGGCTCAAGACTGTTTGGGTCCAGCGGTGTTGCTACCTGATCCTGTTTTCGGTTCTGAGTACATCCATTACTAATCATG
GGATTACTTGTTTAGGCCAACCAGACATATTCTGGGGTGAGATTACGACATGTTAATTTACATTGACGGTGTTTCTACATGTGTTAGTACTAATGATGTT
ATCGCTCTTGGCCCCGTTACGTTTCA
>Locus_14087_Transcript_63/66_Confidence_0.185_Length_2628
TCTGTTCCAAATGCAATCGGAAACCCCAGGAATGGAGCTACGAGGCATGGATCTGTGAATTCATCTGGTGGCACTCTCACTTCTAGCTCAGAGCTAGGGC
ATGGTTCATCCAAGAGCTCCATATCGGCATCCATTGATTCGCCATCCAAAGTAGGGAACAACTTAGAGTTCGACTTTGCTGCTGTCGAGAGGCATGGTAA
GAACATGGGTAGTGATGGTAGTAGAGTTGAGGACTCAGGAACTTCTCCATCGTCAGCGTTCAACCATGGAGAGCCATTAATCAGCCTGAAGCTTGGAAAA
AGGGCTTACTTTGAAAATGTTTGCGGAGGACAGGATATCAAGAGCTCTGCACCTTCAACAGTGACTTCTCCATCAATGGTTGTCAAGAAGACCAAGATGT
CTCAGCAGAACGCACAAAGCTCAAACTGTCAGGTTGAAGGTTGCAGAGTTGATCTGTCTTCTGCTAAAGAATACCATCGCAAGCACAAAGTCTGTGAAGC
TCATTCTAAAGCTCCCAAGGTTGTTGTTGCTGGTGTAGAGCGCCGTTTTTGCCAGCAGTGTAGCCGGTGATATTTCCTTGACTGAGTGCATTTTCATTAT
ATAGTATGCCTCTTTGTATTCACCGAATCCTTTTTTAATGTTGGCATCTCTAATTGAAAAGAGAGAACATAAAAAATGTGATCAGTTGAAAGCAATACAA
CTATTTAGTCGTAGAACGACCAAGGACCAACTGCTAGATGATGCCCAATTATTTTGCATGACTGAGACTTCCAAAGTATCTGTGATGTTTATTACTCCTT
TGCTGATCAAGGTACACAAGAACACTGGAGTGACATGAACACTGAAGAAAATTGGCATTTCTTTTTGTTTCTGGCACTAGTTTGTTATTGATTTATGAAC
ACTGAAGAAAATTGGCATTTCTTTTTTGTTTCTGGCACTAGTTTGTTATTGATTTGTAAGCGGAATGCTGATAAACATCTTGAAAAGTGAAATTCAGATA
AACTAATTACAATACAGTTTGATCTTTTAGCGACATAATGGACATTTATTTCCTTTTCTTTAATTCTTCATTGGTTTGTAGGTTTCATGGTTTAGCTGAG
TTTGACCAGAACAAACGAAGCTGCCGTAGGCGTCTTACTCATCATAATGCACGGAGGAGAAAACCACAGGCAGATACAATTTCATTCAGTTCATCGCGGC
TCTCGACAATGTTTTATGGTAGTATGGAGCCCTTTGTTCATGAGTATATATTATTCTCAAAAACACTATCTAAACTGATCACTCATACAGTACCTCTTTT
GAGAGGCATTATTTCTGAACCCTACATGTTGAAAGAGCACTTTCTGTAGGTGTCAAATGGTTAATCTTCTATTATAGCTTGTTCTCTCCATATTGTCAAA
TTAATGACTGAAGGCTAATCCTTTATCCAAATTTATACTTCTGAATATGATGCTGCTGCTGCTGCTTTAAGCATTGTAAATATGCATTGGCTTAGATTGT
CGCAGATGCATATTCACAGAGTTAGTCGATTATTTGGAAGGGGACTTAACCCAACTTCATGACACTTTTAGTATGCACATACGCTTCTCTTTTGGCGGGA
CTACTCTTAGAATATTCTTCTTAAATTATTATAAATGCAATTTTGCTTCTGGGAGAACACTTAAGTTTTAATGCTGGTATTGTTCGCATTTGCAGATACA
AACCAGCAGAAAAATCTTTTCTGTAGTCAACCTCATTTTGGCCAAGCGAGAAGCAATGCAGTTTTTTCTTGGGAAAACTCGGGAGATTCCAAATTTATGG

AAACCAAACATCTGTTGATGCAGCCAACGAAAATGTTAGGTCTTGATGGGCTGCATTTCTCAACCCCCCAGATATCAAATAGTGTTGTGGCTCACTCTGT
ACATCATCGTAATTTCGATGGGCTCATGCCATTCAAGGGAACCAACACAAAGGTCCTCAACCAAGGCGTGGAAGCTTCTGCGATCGCTTCCAACTCGAGT
GGAGACCCGGATCTTGGGTGTGCTCTCTCTCTTCTGTCAAACGGTTCGTGGTGTTCAGGTTCAACTGTCATCCAGCAGCCTAGTTCTCGTGTACACGCTG
GTGCGGTGCCGCCCCTCGCTACTTTTGTGGTCTCTAATCCTGCAATGCATCCTCTGGACCCAGCCCCAGGAGGGTTCTGGCAAGACGACCCTGCCTCGCT
CGATAGAACTCCGGAGATTCAGGCATTCGGACACCTCTAACTATGGTCAAACAAATGTACTCATGCTGCATGCCCTCGGAACAGCTGATTTAGTCACCTC
ATCATAATCTGCAACTCTGCTTTGCCAGTGGCAGATCTCTGCATAATGTCAGTCCTATCATCGGAGGCATTTGGTGAGGTGAAGACTGAAGAATAATATA
GGAGGCAACCCCCTGATCTTCCTGATTTAGGATGCATCTAGGATCCAGTTGTGTGTGTGCTTGTGGTTTTGCCAGAACCGGTTTTTTGTTTGCCGTCCCG
TTAGAGAGAAGGGATCAAGTACATTCATGACTGCTCATGAGATTACTTGTTTCACTAATATTACATGATCTGGAGCGAGATTTCGCTCGGTTCACGTTGA
TGGTCCTGCAACTTTTGTGTTTCATAGT
>comp63680_c0_seq8
AGTATCTCGGCGTGGGACAGAGGCCGGTGGTGGTCGCGAAGAAGACGCGGCGCGGGCCGAGGTCCCGGAGCTCGCAGTACAGGGGCGTCACCTTCTACAG
GAGGACGGGCCGGTGGGAGTCGCACATCTGGGATTGTGGGAAGCAAGTCTACTTAGGTGGTTTCGACACTGCTCACGCGGCTGCGAGGGCTTATGATCGT
GCAGCGATCAAGTTCCGAGGGCTCGACGCGGACATCAACTTCACTTTGAGCGACTATGAGGATGACTTGAAGCAGATGAGCAATTGGACAAAGGAAGAGT
TCGTGCACATACTCCGCCGCCAGAGCACGGGGTTCGCGAGGGGGAGCTCCAAGTACCGCGGCGTGACGCTGCACAAGTGCGGCCGCTGGGAGGCGAGGAT
GGGTCAACTTCTTGGCAAGAAGTACATCTATCTTGGACTCTTTGACAGCGAAGTTGAAGCTGCAAGAGCATATGACAGGGCAGCCCTTCGCTTCAATGGG
GGGGAAGCTGTTACTAATTTTGGGCCTAGCTCCTACAATGGAGGAGATGCTCTACCCGACACCGAAAATGAGGCAACTGTTGATGGTGACGCGGTTGATT
TGGATTTGCGGATTTCACAACCTAATGTGCACGACACTAAAAGGGACAATACCTTAGCTGGAGGCCAGCTAACATGCGACTCCCTTGAATCTTCAAACAC
GATGGCCTCTCAGCCAATGAGCTCATCGTACCCGTGGCCTGTGTATCACCAAAGCACGACAGCAGTACCACCTCACCATCAACGCTTGTACTCATCTGCT
TGTCCTGGCTTCTTTTCGAACCTCCAGGAAAGGCCAATGGAGCGAAGGCCTGAGTTGGGTACCCAGTCATTCCCCACCTGGGGATGGCAAATGCAGGGCT
CCCCTCACATGCCGTTGCATCACTCTGCAGCATCATCAGGATTCTCTACCGCCGTCGGCGCAAACGCCGGCGTCTCGTTGCCTTCTCACCCGCCGGTGCC
GTTCCCGGACCACCAGTTCTACTTCCCACCGACGGCATGAGCTTGTTGGTCTGGTTCAACTCTTGGGACGGCAGCTGATCTTATGTGTGAACATTTCGCA
GCTTGCCGGTGACCGTTGTTAATTAATCGGGGAGAGAGAGCCAGAAGACCCAGTATGGCTATCTCTCTGTCGCCCGTGCCTTGTTATGATCTTAGTTATT
GCTACTACACCGTGGATTCATCATGGTTTATGAGATTCTTTACTCAAGTTTCAGCTTAAATGTCCAAATACATGAATGCATCATACACAGGATCCGTCAT
TTTCTAAAAAAATACAGGATCTGTGTGAAGTGTAAACATAAGG
>comp81587_c0_seq6
GGCCATCTTCTGGCTGAAGAGAGCAGCCACGCTAGAAGCAACGCATGCAACGGTTTCTCCGGCACGCTGGGTCCCGAGTGTGCTCTCTCTCTTCTGTCAT
CGTCGCTGCATCATCCCTCCCCTGCCATCATCCCCACCGCAGCCCAAGCGCAGGCCACCTCTTCGCTCGCCCGGTTTGCTCCCGCGTCGCAGGCCGCAAC
CACTGCATTCGCATCCGGTGGCGGCCACCATGTGTTTGTTCCTGATGCTGTCTTTGAGGATCCTTCACAGGCACTGCCGTTCCCTTGGCAGTAGCAGAGG
GGTGGAAAATGTCTTGTGTTTCTGTGTTGCTGATGAGTGCTCAGTTGCTGGTAATGGCAGGATCAGGCTGTTGGTCAGAGATGTAGATCAAGTGGACAGG
TCAAGTCCTGTTCTCTGGATGATGATGGAGGTGGTGGTGGTAGCTGTTGTGCAGATTGTTTAGTTGATTAATTCTGTTCTATTCATAATTGTGTACAATT
TTGTGCATGTTTTGTAGCAAAAAAATTATGCATTTTTGCAAAACTTGGAGTGCTTGAATCAGGCTCTAAAGGCCGCACGCAGTCTGGAAAGCGTTCAGGA
GTAAAAGGTCTGGTTT
>Locus_12535_Transcript_7/73_Confidence_0.057_Length_1921
GCACATCATGATTTTAATGGTATCATGGCTTTCAAGGGAACCAGTGCAAAGGTCCTTAATCAAGGCAGGTTCCTCTCTATTATAACTGACGTGACATTAA
TAAGTTGTGGTCCTTCACTTGCAAGAAATCTGTTTTTAATAAAATTCCTAAGTAGAAAAAAATTCATTGATAGATGCACCCATATGATATTATTACTCTA
GGAGCCATTCGATAGAATTTTATCCTTTGGATGATTCTTCTACATTCTAGCTGTTTGTGTATGACTGCTTGTTTGAAGCTGTTAGGTTGTTAGTACTACC
TGACATGAAGTTTTATGAAATTTGTGACACATACCTACATCATTTCATCATACAAGTGAGTCACCATACAAGTATATGGCATGTTTCTAATGTTGCAATA
TGAAAGCCTTTCTTTGTGAGGGAGGATATGAGAACCTAATTGCTAGGCCACATTCTTCTCCTTGTTTTTATCTTGGTCAAGGAATGACCATTACCTGCCA
TTTTCAGTAACATCCAAGTAATCTCTGTCTTTGCAAGATGTTTGCACATTTTTCCTCTAGGCTTAAATGCAGTAGTGTACCATATATGTTTTGCAAGTGT
TGAGGCAGCAAGGATTTGCAGTTCATGTAAAAATTATCCAGTTTACCAGAGCTGTCACCATCCTTTAGTGGCTGTCACCTAAACCTTGAGTCAACTCTTC
ATGATATGGCCACAGGACACAGCACGCTAAACCAACTTTTCAGACACCACTCCAGCATGATTGGATCCTAGGACAGTTTGTCTGTAAGGTGCGTGGTGTA
TGTCATGGTTGGCTTCAAGATTTGGTACGTTGGGAGAAAGATTGCAACCGCCAAATGCTTTTCGTTGTCTTTAATTGATCAATTGCCTAGAGCATCCATA
TTCCAGGAAAGGCTAACTGATTGATTGCCTTAATGTCACTGTCTAGTGTGAAGCAATGATTGCGCCTCTGCTGCTCAACATGTGTGGGTTGATGGTGTTA
CAAACGCAGGCAGGCATTGGAGTCATAATGTTGTTATTCTGTTCAATGATCTTATCATTATTAATTCCAGTAGGACCATGTTCTCCATATGCAAAATCTT
GGCTCATCTATTAACTAGAGTACTAACTGAATTTCCCATTGCACTCTTCTCATATAGTTGAATTATAGCCTTCATAAGCATGTTCAATAAATTTGAGAAT
TTTGATTATTGATGAACATAATAGTAACATTTTTCCTACCTCTTAACATAATAGAAACATTTTTCCTACCTCTTACCCTTTTTCAGGCGTCGAAGCTTCT
GCGGTCGTGCACATCATGATTTTGATGGTATCATGGCTTTCAAGGGAACCAGTGCAAAGGTCCTTAATCAAGGCGTCGAAGCTTCTGCGGTCGTCTCCAA
CACGAACGGAGCCCCGGATCTTCAGCGTGCTCTCTCTCTTCTGTCAAACAATTCAGCTGGTGCTGCCAACGACCAGCCAACTCCTCAGCTGCACCCTGGG
CTGACCACCTTCGTCGGCTCCTCCAACCCTGTCACTGTGATGGAAGCCTCACCACCAGGACTCTGGCAAGACAGCACGCCCCTTGATCATCAGGCGCAGT
TCCAGGCTTTCGATCCCATTGCCGCACCGCAACAGCTCCAGCTCCCAAAACCCCCTTCCTTCAACAGCTCCACTTCCAGCTATGGCCAGATGCAGTGATG
GATGCTTCCTTCAGTTCTGCTGTCTGAAACTCTGAAATTTGAAACTGCTGTGTTCTAAAAAATAAAGAAAAGCCTGTGTGCGAAAACTCAGGCTTGAAAA
ATTGATCCAGTAGTGTTCCCGTTCAATGGCTTGAATAATCCATCCTTGTGATGGCGTGTAACTCAAATGCTTGGATTGCGCTGGCAAGTGATCATGGACT
CTTGTGTTCTTGATGGCATCT
>Locus_5983_Transcript_72/82_Confidence_0.307_Length_2736
TGCCATCTCCATCTTGTGCGTCTCTGTTGGATCTTTGTGAGTCATGGCCTCTTTGCTCTCTTGCTTTTCTTCGTTTCTTGTTGAACTCATGGTTCTTGTT
TTTCTTGTGCTTGCCAGGTAGAATTATTCAACCGGATATAGATTCCTTCAAGTAAAGGAGTGCTAGCGGTTTTTACCTGTTCGGGTGGAGCACAAGTTG
AGATCTTTAATTCTAGCATGGGTTCCTTTGGGGTGAACTGGAATCAGAAGAACTCCGCGGTGTGGGATTGGGAAAAATTAGCTCCATCTGTTCCAAATGC
AATCGGAAACCCCAGGAACGGAGCTACGGGGCATGGATCTGTGAATTCATCTGGTGGCACTCTCACTTCTAGCTCAGAGCTAGGGCATGGTTCATCCAAA
AGCTCCATGTCGGCATCCATTGATTCACCGTCCAAAGTAGGGAACAGCTTGGAGTTCAATTTTGCTGCTGTCAATACGTATGGTAAGAACATGGATAAGG
ATTGTAGAGTTGACGACTCAGGGACTTGTCCATCGTCCATGATAGCATTCAGCCATGGAGAGCCATTAATCAGTCTTAAGCTTGGAAAAAGGACTTACTT
TGAAAATGTCTGCGGAGGACAGGATTCCAAGAGCACTGCACCTTCTACCAGGACTTCTCCATCAACCGTTCTCAAGAAGACCAAGGTGTCTCAGCAGAAT
ACACAAATGTCATACTGTCAGGTTGAAGGTTGCGGAGTTGATCTATCTTCTGCTAAAGATTACCATCGCAAGCACAAAGTCTGTGAAGCTCATTCTAAAG
CTCCCAAGGTTGTTGTTGCTGGTCTAGAGCGCCGTTTTTGCCAGCAGTGTAGCCGGTTTCATGGCTTAGCCGAGTTTGACCAGAACAAACGAAGCTGCCG
TAGACGTCTTACTCATCATAATGCACGGAGGAGAAAACCACAGGCAGATACAATTTCATTCAGTTCATCACGTCTCTCAACAATGTTTTATGATACAAGC
CAGCGGACAAATCTTTTCTGTAATCAGCCTCTTTTTGGCCAAGTGAGAAGCAGTGCAGTTTCTTCATGGAATAACTTAGGAGGCTTCAGATCTATGGAAA
CGAAACATCTGTTGATGCAGCCATCGAAAACTGTAGGCCTTGATGAGCTGCATTTCTCAACCCCCCAGATATCAAATAGTGCTGTGGCTCATTCTGTACA
TCATCGTAATTTCGATGGGCTCATGCCATTCAAGGGAACCAACACAAAGGTCCTCAACCAAGGCACTATGCTAATCTACTGATCAATTACTGAACTTAAT
TTTAAAAAAAACCTCTGGCAGCATTATCGTAACAGTTATCTACACTACTGTTGTAGTTCTTAGGAGTTTCACCTCTCACGCTATTGAGCTAGCATTTTGT
TCTTCATGGTTCCAATTTGCACAACAATTATGTTACGCATTTTATGAATTTGATGATTTGTTATTGGTACTCAAGTTCTTGTTCATGATATTTTTTTAA
CATGTCTTCTGGGCTATGAAAGCCCAAAAATTTGCTGGCCCCTTTGCATGTTCCTGTGCACTTCACGCTATTCTGAGTCTACCATCTGGAGAAAGAAAAT
GAACACCTATCATCTCCTGCAAGATTATATACTTGTTGAATCATGTGTAAAATTGCACTGTGGCATAGTAATATAGGAGTTATAATATTTCAATCTGAAC
CCTTCCAGATTCTGTCTGGAGCCTTTCACCATCAGAAAAGGCACGAAAGTGCAAATGCCAAACAGCTTGGGCCCCTGGACTGGGTTTATGGTCAGAGGCT
CAAAGCACTGCACTGCTAATGCGCTTGCCAGAAATTCTGGCTGGCAGCCCGGTACCACCTGATCCTAGTATTTAGGTTCCAGAAAAGAGATTGATTGAAT
ATGCCAGCTTAATGTTGCCTGAGTGTGACTACTGATAGATAGTCGCATGAATGCTCTTACCTTACAACCTTTGGAACTAAAAATATTGCTCTGTTGAGCA
GTCCGATCGTTCAGATTTCTAATCTAAGGATGAAGCTCAGCATAACAATTCTATGCTACTATTTCAGGTGTCGAAGCTTCTGCGATCGCTTCCAACTCGA
GTGGAGACCCGGATCTTGGGTGTGCTCTCTCTCTTCTGTCAATCGGTTCGTGGGTTTCAAGTTCAACCGTCATCCAGCAGCCTAGTTCTCATGTGCACGC
TGGTGTGGTGCCGCCCCTCGCCACCGTTGCCGTCTCCAACCCTGCAATGCATCCTCTGGACTCATCCCCAGGAGGATTCTGGCAAGACGTCCCTCCCCCG
CTCGATGAAACTCCACATATTCAGGCATTCACGCACCTCTGATCAAATGCTGCATCCCCTGGGAACAGCAGGTGGTTATACCTTCCGTAATCGTCCTAGG

TTTGCTTTGCCAGTGACTGGCTCTGCACAATGTCGATCCATCACCGGAGGCATTTGGTAAGGTGAAGACTAAAGACTCAAGAATAACCGGAGACTAACTC
GCTTCTCTTCCAGGCTCAAGACTGTTTGGGTCCAGCGGTGTTGCTACCTGATCCTGTTTTCGGTTCTGAGTACATCCATTACTAATCATGGGATTACTTG
TTTAGGCCAACCAGACATATTCTGGGGTGAGATTACGACATGTTAATTTACATTGACGGTGTTTCTACATGTGTTAGTACTAATGATGTTATCGCTCTTG
GCCCCGTTACGTTTCAAGATCTGTTACCATTTGAGT
>comp79373_c0_seq17
GAAACCTGGTAAACCCTGGCCACGGACGCGCATGCCATCCAAGATGACGTAGTCCACACCGAATCTTCCACGTCCGCACACGCGAGCACAACGCCACCGC
AGGCCAAGTGCCGTCTCCACCACAACTCGCTGATCCCTTCCTCCTCGCCGGCTCTCTCTCCATCCCTCGTCCTTCCCCATCTGTCCCTCCCTTGGCTTCT
TCCTCCAAGCTCCAACCAATCCCCACGCGCGCGCGCGCTATAGCCTACAAATAAATACTCGCCACTCTTCCCTTCCTCCCGCCTATCGTCTTGCCAAGCG
CTAGAGCAGCTGCGCCATTGCACGCTTCCGGCCCGTCGTCCTGCACATCTGTCCTCCCCGTACTGCCTTGTACAAACTCCGTATGTAAACTACTGGGTTG
TCGCCAGTGTTCGTGTTTGCTGATGCTCATCTAATTATCGTCGAGGAGATCCAAGGAATTGTCCGCGGAGCGCCGGGGAACAAAGGCTGCGCGCGTTTCA
AAATCAGTTATTGAAACGGCAAGATGGTTAATTTTGGAAAGAAGTTGGCTGCAGATCAAGTAGAAGAATGGAAAGGGTACTATATCAATTACAAACTGAT
GAAGAAAATGTTAAAGCAATATGTTCAACAGACCCAATTAGGCGGGAAAGATTGTGAACAAATCCTTAAAGAGTTCTCGAGGATTCTTGATGACCAGATT
GAAAGGATTGTGCTTTTTCTGCTACAACAACAAGGCCACCTTGCCAGCAGGATTGAGGAATTGGGAGAACAACGCACTGCTCTTATGGAACATTATGATA
TATCACAAGTTTCCCAGCTACGTGATGCGTATAGAGAAGTTGGGAGAGATCTTATTAAGCTTCTGCGCTTTGTTGACATGAATGCTACTGGTATACGCAA
GATACTAAAGAAATTTGATAAGCGTTTTGGCTATAAGTTTACATATTATTATGTCACCACTCGTGCAAATCATCCCTATTCTCAGCTTCAACAAGTATTT
AAGCAAGTGGGAATTGTAGCTGTTGTAGGTGCATTATCGCGCAATCTTGAATATCTGCAACATCATCAAGGAAGCTTTGTATCCATCTATGATCATCCAT
CAGTTACCTTGAAGGACCCTATAATAGACGAAGTAAACCATGCGGTACAGAAATTTACGCATGCCACCAATTTTATGAAATTCTTGGGACAACACGCGCT
TATTGTTCCAGAGGATGCACGAAGCGGGTCTGAGGATCTTGTTGATGATCAGAGCTACCATTTCATGTCTCTGCTGCTTAACCTAGCGAACACTTTTCTT
TACATGGTGAACACATATATCATTGTGCCAACTGCAGATGACTATTCAGTAAGCCTTGGGGCTGCTGCGACTGTCTGTGGTGTAATTATCGGATCAATGG
CAGTCACTCAAGTGTTCTCCTCGGTTTATTTCAGTGCATGGTCAAATAAGTCATACTTCAGACCACTTGTATTCAGTAGCATTATGCTATTTTTTGGGAA
CCTGCTATACGCATTGGCATATGACCTGAATTCATTAATAGTTCTCCTGATTGGACGACTACTATGCGGGTTGGGCTCTGCAAGAGCAGTGAACCGTCGC
TATATTAGTGATTGTGTGCCTCTCAAAATCAGGCTACAAGCTTCTGCAGGATTTGTCAGTGCTAGTGCTCTTGGCATGGCATGTGGCCCTGCTCTTGCTG
GTTTCCTCCAGACAAAATTTAAGATATACTCGCTCACTTTTAATCAGAGCACATTGCCCGGATGGGTCATGTCCATTGCTTGGCTTCTTTACTTGTTGTG
GCTGTGGTTTACATTCAAAGAGCCAGAACACTTCGCTAAAACTGTCATCAATGCACAGCCGTCTGAATCAGGTCACCAAGGAAGTGCTAATTTGGAGGAA
GGTCTAGCTCAACCATTGCTTCTGGGTACAGAACAAAGACAGGGCGAGAATGCGGATGACAATGATGATAATGAAGTAGACTCTGAAAACTCTCATGAAC
CAGCAACATCAATTACTTCAGCATACAGATTGCTGACACCATCTGTGAAGGTCCAGCTATTGATATACTTTATGCTCAAGTATGCTATGGAAATTTTACT
ATCTGAATCAAGCGTTATCACAACATACTATTTTAGCTGGTCTACGAGTGCTGTGGCTATCTTTTTGGCAATTCTTGGATTAACTGTCCTTCCAGTAAAT
GCCATTGTTGGAAGCTACATTACAAATTTGTTTGAGGACAGGCAAATTCTGTTGGCATCTGAAGTCATAGTTCTCATTGGTATCATCATGAGCTTTCGTT
TCACCCCTCACTACTCCATTCCACAATACGTCACTTCAGCTCTCATCACATTTGTGTTTGCTGAGGTACTCGAAGGAGTGAATCTGTCCTTGCTCTCACG
AGTAATGTCGTCTAGGCTTTCGCGAGGGACCTACAACGGTGGACTCCTCTCGACAGAGGCCGGGACATTGGCCCGTGTAATTGCAGACGCGACTATTACT
GCAGCAGGTTATCTAGGCCCGGACCTCCTCCTCAACGTCACCCTGCTGCCACCTCTAGTGATCTGCATAGTGTCCATCGTCGCAACGTTCTGCACTTACA
ACAACCTTTGCTGATAAAGCAGCCCCTCTGTGTCCAGCCAGTTAAAATGACTCCTGCCATATTGGGCCGTACCGTACTACTTGAAGTAGCATCAGCGTTG
TAAATCTGGTAAATCTACAAATACCATGGACGGTGCCGGTGTGGCGGTGTGTAGTGTATTGTTATGCATTCAACAACTTTCTTGTGTCTAGTAGAGTTAA
ATATTCGTCAAATTGAAAATTCAAATGCATTAACCAAGGAACATCACTGTGTTGGAAAGACAAGATGATTTATTGCAA
>comp80692_c0_seq3
GCTCCTTGAGAAACACCATCCAATTACAAGACTATTTGATGCAACAATCACGCATATGGCTGCGCTAGAACTACTGCTCTGAATTAAGAGGCGTTGAAAT
ATTGTCCGGTCTACATATAGGCACTTGAGGTGTATTGACAGACAGTTCCCCTTAGTTTGCTACAACACATCAGGCGAATTCCACCAAATTAGGATGATGT
TTGTTTCTTAGACACAGGATAGCAATGCCAAAAGTTTTTTATAATTTTCCGCTTCACCGCAGATTTGGCATCTCAAAGGGAGTTGTGGCAAGATTCTCTT
GACCAAGCTTTCTTGCAGATTTGGCAAGAAAGATAAAAAAAATGTTGGTATACTTAAGCATCCTTAATAGAAACTAAACAGGTCTTAGATTGAAATTATC
ATAAATAAACCCTTTTTCACTTTAGCTGTAGTCCGGATGAAATCTTAGGGGCCTTGCTTAGCCTGCCCTGAAGCTTGCCAGTAAGTCGAAGCTGATGCTG
ACACAGCACTGTTTCAAGTTCTCATCTTCTGAAAATGATCAGCAGTCAATAGATGTTTTAGTCCATTCTCCAGAAAAATGAGTGCCGCATGAATCCTTTC
CGGTTTATAGTGAAACATTTTAGACGATGACCTTTAACATTCTAGAAAACGAAGAGAAAACCTACAGTCATCTAATTTCAGCTTAAACATGTTCCAGCAA
GATCATCACCATCCTGACATGTTCCGTTTTGTCCCTAGACCCGAGCACAAGGTATTGCGAGGCTAATTTAGCTCAATTCAGTTGTCAGAGCAATTGTGTG
TGAAAAGTGTGAACAACAGCATAGGAGACAATACAGCAAGTGTAGCGTGCCAATATGTGTCCAGCTTGATAGTTCACTATAATAAAGTTCTTTAATTCAA
GTATTTGAATAATATAGAATCTCAACCTTCAAGTTTAAAAGGTTTTGTATTTGCTATTATAATCGTTGCCACCCCCTCACCCCTCTCAAAAAGAGAGTTA
CTTTGTAGCGCACTGTAAATCTCTTGATTGAAATAGTGGCATGTCATCTTATTTAATTCAGTTATTTCATAAGGTACAATTTGTTTAGCTGACAGCTCAG
ACATGCTTCTATGCAACATGTTTATACCAATGCTGTATTTTGGCAATCCTGGATGTGGCATTACTTAACTGTGCTTTCATATTCAATAGTTCTGGCAAAG
GGAGGCTGCAAGCTTGAGACAACAACTGCACAACCTGCAAGAAAGTCATCGGTATGGCTAGAAAACCCTGCAAATTTTACAAATGTACAAGTCAGAAAAT
TTGCCTAATGTAAAAGGATCAAACTACAGGATTTGTCAGCATGAGCACAAGTGCCTAACTTCCGAGTTAGATAACTCTGGAGGAAGGAAATCCTTCCTAC
TGTTTGTTTCAAGAATATACAAAAATTCAAAACAACATTAGAATAGAACCAAAAGAAGAAACAATATTCACCATGTAGCAATACTCAATTCCTGGTTGTC
TTATTTATAAAGGTATAAAACTAGGTTCTGACGAGACAATTATGAATCAACTACACCATGTTACAGATCATCCATAACTCTTGGTGATGCAAATTAAGGG
CTGTAAAGTGACAAGTATACCTCCACCAACTAATTTGTGCAGAGTGTATAAAATTGTAAAACAATGGATAAGAATGAATTTAGTATACAAGATAGCATAC
AATGCCCAAGTTTAAGGAGACATACTGATTTACTGACTCCATAGGACAATCTTTTGCTTATGAGGACATCAGTTTTCATAAACAATATCAATGTAAAAGG
AGGTAACAGTTTATGAATTTCCTATGCCAAGCCATTTATAAGTCAAAAAATGTACCTATTTAGCTTTTATGCATTCCAATTAACTTCCAAAGAAGAAGAG
TACATACTAATGGAAAACAAATATGATATTATCATCTGTTTTGCCCGGTAGTTTCTTGAGTCTCTGATTTGTCCGCACTTCACTCCCAACCAACACAACA
TTAATACACATGGGCAGAGGAATATCAGGTTATCAGGTAAACTGAATACAGTTTGATTGACTGCAGTCAGTTGATGGGACAAGATCTTTCTGGATTGAGC
GTCAAGGAACTCCAAAATCTAGAAAATCAGCTAGAAATGAGCCTACGTTGCATCCGGACAAAAAAGGTAAGGATGCTAGCCATATTTCTGTTTTTCGTTA
CCAGATTGCCTCATCCAAGAATACAACTGATGATGACGTTTTTTACTTTCAGGACCAACTCTTGATTGATGAAATTCACGAACTGAATCGAAAGGTTCTG
GAATATTCTGTTATTACTCTGCAATGAGAGGTCTCTGAGTGGATTTCAAGAAGCTGACTCTACTGGCTCATTTTCAGGGAAGTCTCATGCAACAAGACAA
GATGGAACTATACAAAAAGGTCAACCTAATCCGTCAGGAAAACATTGATTTATACAAGAAGCTCTACGAGAAAGAGGTGACAAGTGAAGTCAACCGAGAT
TCATCAACTCTAAACTTTGCAGTTGTCGAGAACGTCAACATTCCTGTTCATCTTGAACTTAACACTCCACCGCAAGAAAATGATGTTGAGCAAACTGCAC
CTCCTAAACTAGGATTACAACTAAATCCATGAAGGCATGCAGCATGGCACTGCTAATGTTCATTATTTTCAACGGCTAGGATTCAAAGCAGCTAGAATAC
CATTGGTTGCCTAAACACATAGCAAGACAGACGTGGCATGCTGGGCGACGGTAATGGTAGTGTAAATCTATTTTCAAATAAAATGTATTTTATTGAAGAC
TACAGAAAATTATTTGTCTTATAACTATTTCACGCATTCTGCAAGTTTGTTCACCAACTTGAGTACATGACAGCTACAAATCTTATATTGACAAAAGATA
TGGTGTGCGGAAATAATTCAGGTACTGTTTTTCCTAAAAAAA
>comp88323_c0_seq2
TTCTCTCTCTCTCTCTCTCTGTTGCTCGCGACTGCACGCTGCTGCTGCTGTTCCACATCTCTGTCGCCGCTTTAAGGCTGTCACCTTTCCCCCCTCGCTC
TCCTTCTCTCTCCTCAATTACGACGCCACAACCAGACCCAAACCCTAGTACTACCACGAGGCGGAGTAGGTGTGCAGCCGGCGGCGCTGCCCCTTGATCC
GATCCGTCCGGCGCTGCGGGGGATTACGACTTGTGAGTGCTCACCGGCGATCGATGGGAGGCGCCGCGGGAGGCGGCTCAGGCGCGGGGAGGCTGTGAGG
TCGGTGGGTGTGCTGTGCGTCTGCTCCGGCGGCGCTCCCCTCGGCGAGCGGGAGCTTGTCGGTGGGTCTCGGCGGTGTTCGCTTCGTTTGCAGATAACTT
CGCGAGCTGAAAGTTGAAGTGTAAGCGGGGAGCTAAGAGCAGTAGTTGAACGAAGCGGAAGAGGAGGACGTGATTTGAGTAGTTGGGCTCTTGCATTGGC
GAGGAAATAATGGCTGCGGCCGTGGCGATGCGAGGGGGAAGCAGTGACAGCGGAGGGTTTGATAAGGTTCCGGGGATGGACTCGGGGAAATACGTGCGGT
ACACCCCGGAGCAAGTTGAGGTACTCGAGCGGCTGTACATCGATTGCCCCAAGCCAAGCTCCTCGCGGCGGCAGCAATTGCTGCGCGAGTGCCCTATACT
TGCCAACATTGAGCCCAAGCAGATCAAGGTTTGGTTCCAGAACAGAAGGTGTCGCGATAAGCAGCGGAAGGAGTCTTCGCGGCTTCAGGCTGTGAACCGG
AAGTTAGCGGCAATGAACAAGCTTCTGATGGAAGAGAATGAGCGTCTTCAGAAGCAGGTCTCTCAGTTGGTTCATGAGAATGCACACATGCGGCAGCAGC
TGCAGAATACTTCATTGGCCAATGATACAAGCTGTGAATCAAATGTGACTACCCCTCCAAACCCTATAAGGGATGCAAGTAACCCTTCTGGACTCCTTTC
GATTGCAGAGGAGACCTTCACAGAGTTCCTCTCAAAGGCTACTGGGACAGCTATTGATTGGGTCCAGATGCCTGGGATGAAGCCTGGTCCGGATTCGGTT
GGTATTGTGGCCATTTCGCATGGTTGCCGTGGTGTTGCTGCCCGCGCCTGTGGTTTGGTGAATCTAGAACCAACAAAGATTGTGGAGATCTTGAAAGACC

GCCCATCTTGGTTCCGTGATTGTCGGAGTCTTGAAGTCTTTACAATGTTACCAGCTGGAAATGGTGGAACAGTTGAGCTTGTTTGCATGCAGATGTATGC
GCCTACTACTTTAGTTCCTGCACGAGATTTTTGGACGCTGAGATACACAACCACTATACAGGATGGCAGTCTTGTGGTCTGTGAGAGATCTTTAAGTGGT
TCAGGAGGTGGTCAAAGTACTGCATCGGCACAACAATTTGTTAGAGCTGAGATGCTTCCTAGTGGCTATTTAGTCCGCCCATGTGAGGGCGGGGGCTCAA
TTGTGCATATAGTGGACCATCTGGACCTTGAGGCTTGGAGTGTCCCTGAAGTTCTTCGTCCGCTTTACGAGTCGTCTAGGGTAGTTGCTCAGAAGATGAC
AACTGCAGCACTACGGCACATCAGACAAATTGCTCAAGAAACAAGTGGGGAAGTAGTGTACGCCTTGGGCAGGCAACCTGCAGTCCTACGGACATTTAGT
CAAAGGCTGAGCAGGGGCTTCAATGATGCCATTAGCGGTTTCAATGATGATGGCTGGTCTATAATGGGGGGAGATGGTATTGAAGACGTAGTTGTTGCTT
GCAACTCAACTAAGAAAATTAGGAACAACAGCAATGCTGGAGTTGCGTTTGGAGCCCCTGGAGGTATTATATGTGCGAAGGCATCCATGTTACTGCAGAG
TGTTCCACCAGCAGTATTGGTTCGATTTCTGAGGGAGCATAGATCTGAATGGGCCGATTACAATATTGATGCATATTTGGCTTCAGCGCTGAAGGCCAGT
GCGGGCTCACTTCCTGGGTTACGGGCCATGAGATTTTCTGGGGGCCAGATGATCCTGCCACTTGCCCACACAGTGGAGAATGAGGAGATTCTTGAAGTTG
TCCGCCTTGAAGGACAATCTCTTACTCACGATGAGGCTCTTATTTCAAGGGATATCCACCTGCTTCAGCTTTGCACTGGAATAGATGAGAAATCAATGGG
ATCCTGCTTCCAGCTTGTCTTTGCACCTATCGATGAGCTTTTCCCTGATGACGCTCCATTGATATCTTCAGGCTTCCGTGTTATACCGTTGGACATAAAA
ACATATGGTGTACCCTCTGGAAGAACATTAGATTTGGCCTCCAGCCTTGAAGTTGGTTCAACTATGCAACAAGCCTCGGGAAATGCATCTCAGGATGATT
GCAGCCTACGATCTGTGCTAACAATTGCCTTTCAGTTCCCCTTCGAAGTGGATCTCCAAGATAGTGTTGCAACTATGGCCCGCCAATATGTTCGGAGCAT
TGTCTCTGCTGTGCAGAGAGTATCCATGGCTATTTCTCCCTCGCGGTCTGGCTTGAATACTGGACAGAAGATAATTTCTGGCTTCCCTGAAGCTGCGACA
CTTGTTCACTGGATTTGCCAAAGCTATCACTAAGCCCATTTGTCATTTTTCCTTTCCCTTCATATCTCCTGTTTTAAAAACAAAAAAATGTCAAGGGAGT
GCTGCTTTGTTACTCTAATTCTTGACGGTTTTGTATGAAGACCAGTGTTGAAAACCGAATGCGTACCAAAGAATAACATAATTTATATGCAAGCTAATAA
AAATACTTCATATTTGCAAGTATCACTTGGGGCTGGAATTATTCCCTCAGATGAAGTTGGCGAATCATTATTGAGAATGTTCTGGGATCATCAGGACGCT
ATCTTGTGCTGCTCCTTCAAGGAAAAGCCTGTGTTTACTTTTGGCAACCAGATGGGAATTGACATGTTAGAAACGACTCTAATTGCTCTACAAGATCTCT
CCCTGGACAAGATCTTCGATGAACCCGGTAGAAAGGCATTATACGCAGAGATCCGGAAATTGATGGAACAGGGCTATGCATACCTGCCGGCTGGTGTCTG
CTTGTCCGGAATGGGCCGCCACATCTCCTTCGAGCAAGCCGTAGCTTGGAAAGTTCTTGGCGAGGACAGCAACGTGCACTGCCTCGCCTTCTGCTTCATG
AACTGGTCGTTCGTCTGATCCAATCTGGCCGCCCTGCGCGAACCTTACAAGCAGCCTCCGTTTTTGCGTTCTCTGTAGAAGAGAACGGTACGTCTCGCTG
TTTGGTGATCCTATGTTTCAGCTAGTTCTAGACCTGGAAGAAAGTCAGCTATGTAAAGATGCTGTGCCCTTTGTAAATCTGTTCTAGTTCCCGCTGCTGG
TCTATGGGATGGTGAAACACAAGCCGCAGTTTTCAGATGTATCATATCAGATCGCCGCATTGCATTAGGTCTATCTGTTGTGCCTCTGAACTTGTTGCCC
CTACTGATCGACCAAGTAACTGAAACTACTGTTAGCACAAGCAACAGTCAACATAATGTGCAGTGCTATGTTCCCTGTTCAAAAAAAAAAAAAAAAAAAA
>comp67036_c0_seq7
GAAGACAGAAAAGAGGTTATACAGTAGGCTCATATTTGGCATCAGCAGAGGCCTCGCGGTGCCCCGTATTCTCATTTCCTCGCCGTATTTTCTCTCTCCA
GCTCCAGAGTCCGGAGGAAGCAGAGAGAGAAACTCAACGGGAAGGTAGAGAGAGCTGATTAAATGGTACTTGGAGCTAAGGATACCTTCTCGTAGTGCTC
CCGCTTTTGTGAGAAATGGAGGATCATCCAGTCCATCCCATGTCGAAGTCCAACAACGGCTTCTTGTCAGGAAATGGCTATCAGATGAAGCAATTTGGTC
ATAAAATCCATGATAGGGACTCGTCATCAGAGTCTGGTCGGTCTCACCAAGAAGCATCCGCAGTGAGTGAGAACAGTATAAATGAACAGCACACCTCAAC
ACAATCTGACAATGATGATGGTCATGGGAAGCATAATGAGGACATGATGAAGTCAGCAGTGTCCTTGGGGGAACCAGGATCAGCCTTTTTGGCCCCAAAA
CTAGATTACAGCCCATCTTTTGCTTGTGTTCCTTATACTGCTGATGCTTTTTATGGTGGGGTCTTGACAGGATATGCTCCACATGCCATTGTTCATCCCC
AGCAAAATGATTCAGCAAACTCTCGGGTTCCGTTGCCTGTTGAACCTGTAGCAGAAGAGCCAATATATGTTAACGCAAAGCAATACCATGCAATCCTCAG
GAGGAGGCAGATACGTGCTAAACAGGAGGCTGAGAATAAGCTGGTGAAAGGTCGGAAGCCATACCTCCATGAATCTCGACACCGCCACGCAATGAAGCGA
GCCCGTGGATCAGGGGGGCGGTTCCTCAACTCAAAGCAGCTCCAGGAGCAGAACCAGCAGCAGCAGGCATCAGGTGGTTCAAGCTGCACAAAGGCCATCG
GCAATAACACAAGCTCCCAAAGTGGTCCCAGTCACACGCCTCTGACACTTGCTTCCTCTGACACTGCAAGCGCTTCGAGGGCCAACCAGGAGCGCACCTG
CTTCCCCTCGGCTGGCTTCCGTCCCACAATGAACTTCCGCGCGCGAGGTGGAGACGCAAAGCTAGCTGTGAATGGCATACAGCAGCATGTTTCCTTCATC
AGGTGAAGCAAGTGAAATGGGGCGCTGCCTGCGGTAACTCATCCTTGGCTTATGAAACTGTGAATGTGTAATGGACATGTAACTTCAGTCTCAGAATAAC
CAAACATATGGGAGTCCTCCTGAAGTTTCCTGTAGTTCAAAGAACTGATAGGTTATGGCCTTATGGGTTTGCTTCTGGAGACTGAAATCCGTGCTTGATA
TACTATCTTGTGCGAGTTCTGAGGTGAAAGACCTTGAAACTTGCTCTTGTGGTTCAGTACCTGTTGCTTTTGTGTCTGCAAGGTTAGCTCTGCAATTTGC
AATGTC
>comp78295_c1_seq1
ACAACTGCACAACTTGCAAGAAAGCCACAAGCAACTAATGGGTGAGAAGCTTTCTGGCCTAGGTGTGAGAGACCTCCAGAGTTTAGAGAATCGTCTTGAA
ATGAGCCTACGTAGTATCAGAACGAGGAAGGTCATATTCATTTTGTTTCCACGTTTTGTTTTTCTATTCACTCATCTTAATATGCATAAACATCCATCAA
ACAGGACAATCTTTTGAAAAGTGAAATTGAAGAGTTGCACAGGAAGGTAAAGCCTTACAATCCATGTCTTTTTATTATCTCAAACAAATGACCATGCATG
GAGGTTGCTGACATCTTAACTTAATGCTGATTGAAACAGGGTAGCCTAATTCACCAGGAAAACTTGGAACTCTGTAGAAGAGTAAATACCATGTCACAAC
AAAATATGGATCTACAAAGAAAGGCGAGTGAACCAAGAGGTGTTGCCGATGCAAATAAAAGCTCTAGCACTCCCTACAGATCTTGTAGTGCACAAGATGC
AGATATCACGGTTAATCTTGAATTGAGCCAATCACAGCAAAAAGAGGGGGAGCAGTGCCAAAGAGGGGCTCCAGAACTGGGGTAAATATTCTTTTTCCAG
AATCCAAAAACTGCATTACTCCAAGAAGTATCAAGAAGCCTAATGAAGAACCCATGCTTTGACATGTAACAGGCTTCAGCTGCACAAAGAAGATGCACTA
CAAGCTGGAGAGTATCATGATGTTCCACCAATTCTAACATGCAATGCGGAGCACTTAAACTACAATTAGTATTTTGTGCAACATGTTAGATGAGTGAACA
AAATGGTTTATGTACAGACTACAGATATGACCATCTATCTTATCTGTGGCTCTACTGTACTGAAATTGCAAGAAATGGTTATTGTAATGACTAATATGAT
ACATTGACTGTCATACCGTTGACCTTGAAGATAGTTCTATAACGGAAAGGTCCAATTTGAAACAAGTGTTACGGATGAGCTAGTAGGATTTTTTTCACAA
ACAATTAGATAGTCCACGTTAGTGGTAGCTGCTGGGTATGACATTTGACCCAGCCTGCCGACCCTCCCTTTCCCATGGGAGTTCAATTAACATTAGGAAT
GAATATCGATATTATGGTTGATTATTTGAATCGTATGTTTTCCACCCTTAGATGTAAGGCCCTATTTTACTGTTTTAATGGATTAACAAGGTGAGCTTTC
GAGATGGTAGCTCATTGATTCGCATGCATTTTAGTGGCTGTTAAGAGGTACTTAGTGCATCCATGCTTGAAGGTGTTGTTAGTTCGACAAACCATTTTCT
ATCCTACTGGATTACTATCTTTTGGTCAGTATGACAAGCAAGAATTTAATCTGCCAATTAAGAATTTAATGTGTTCTTTTGACATGAATGTCAACAGCAT
TTAAGTAACTCAACTTTTAATACAAAGTACTGGAATGTTTTCTCACCGAAAGTAAAGATGTGGTTCCTTGATAGCAAACAATGTTTATGCCAATATCGCA
TTAAGGTGGTTGTATCCTTTAGAATGATCTCCTCATTCGTGCTTAATTTGATGCTATTGTCTTTTTACGGATGCCTAGATGTGGTTTTGACCATTACTAA
GTACTTTAATATATATTTAACAAAAATAATGACATAGCTGTTGTATGGCCAATCTAGATTTTGTTTTCGAAACATATTTGCAGTCAAACTATAGGAAGTA
AGAAACTTCAATGTGGTTCTCGAAAACCATGCTTAAAGTTTAGAAAGTTTGACTACATAGAGTATATACTACATAGTCTTAAAGCATCAAATGAAGAGGA
ATGGAGGGAGTAGCAGACAATCATCATTCAATATCTATTTCTTGGTTGCCTTCAAATTTGGGCTTACTAAAATCGATTTAAAATTCTCGTGTTCCAGTTG
AAGCTCACTTCAAGTAAATCAGGAATTACCATATTTTTCATTGCTCATAATTTGGAAACATGGCATGATATTTGATAGAAAATGTACAAATCCTCAGCTG
CTCAGAAAGTTACAAAGGGAGGAACGCCCATGAAAGAGAATTCATGGCTGCAACAACGAACATAAACACAGCCTATTCTGTCCAGCAGTCATGGTAATAC
AAGCTAAAAAGGACCGCTCTGGAACAATAGCTTGAAAAGCAATATTAATACAATTTCAGATGAACTATGTGCGTCGATGTCTTACTTGTGACTTCCTTGC
AAGTAATGCGGTTCCTGCCTCATGCTTGGTGCCGCCGCATGCAATAAACCGATAACTAGTGAAGCAAGAGGATAACAGATGTCAGAAAACTGAGCAAGCC
ATGTTAACTGTTTGAGAATTGTATACGGCTATGAGAAGTATATTTTTTTAGGGGGTGTTTCGATCCCTTTAGCTAATTTTTTAGCTCTATATTTGGATGT
TAAATAGGAGGACTGAACATGGGCTAATTATAGACTAATTGCAATTAGTGATTAGAGCCCATAAGCTCTAATTAGCCTATGATTAGCCCATGTTTAGCCC
ATTAGCACATGAGCTAATCATAAGCTAATTAGGGTTAATTAGCTCTAATCATCAATTAGCTAAAGCTAACTATAATTAGTTCACAATTAGTCCATATTTA
GTCCCCCTAATTGGGGTCCAAACATGGGGCTAAAAATTAG
>comp83121_c0_seq25
GGAGGCAGCAAGCTTGAGACAACAGCTGCACAGCTTGCAAGAAAGTCACCGACAGTTGATGGGACAAGATCTTTCAGGATTGGGTGTCAAGGAGCTCCAA
AATCTGGAAAATCAGCTAGAAATGAGCATACGTTGCATCCGGACAAAAAAGGACCAAGTCTTGATTGATGAAATTCACGAACTGAATCGAAAGGTTCTGG
AATATTCTGTTATTACTCTGCAATGAGAGGTCTCTGAGTGGATTTCAAGAAGCTGACTCTACTGGCTCATTTTCAGGGAAGTCTCATGCAACAAGACAAG
ATGGAACTATACAAAAAGGTCAACCTAATCCGTCAGGAAAACATTGATTTATACAAGAAGGTATTTGATAAATCGTGAATGAAAATTCACAGGATATACA
CCCCATCCATTATTGACTGAGGTTTATTTCTCTACTCAGCTCTACGAGAAAGAGGTGACAAGTGAAGTCAACCGAGATTCATCAACTCTAAACTTTGCAG
TTGTCGAGAACGTCAACATTCCTGTTCATCTTGAACTTAACAGTCCACCGCAAGAAAATGACGTTGAGCAAACTGCACCCCCTAAACTAGGGTTACAACT
AAATCCATGAAGGCATGCAGCATGGCACTTGCTTATGTTCTTTATTCTCAACAGCTACGATTCAAAGCAGCCTGAAAAACTATTGGTTGTAAAGAGCCTA
AATACAGCAAGGCAGACGCAGTATGCATGCCATGGGCGACCGATAATGGTCGTGTAAATCTATTTCCAATAAATTGTATTTTATTGAAGGGATGCACCAA
ACGATTGTCTTACAACTATTTGACACATTCTGCAAGTTTGTTGACCAACGTGAGTACATGAT

>Locus_12874_Transcript_17/17_Confidence_0.188_Length_3832
TAATCAACTACATGCAAAAAAATCATATATGGCCAATTATACACTCAAGAAAGAAGCCGTATATTAGGTTTAGGATCCGTTGCATTACAACAAAATGAGA
CGACGAGAAGAAGCTGGTACCACTAACTTGACAATCCACAGGCGATGCTACTGATTGACAATACCATGAATAAAGCCCCAAACCAATAGCTCGCACATAC
TTGGCATTCAAGTGCTGCAGCTCACGTCATCATCCTTCTTGTGCTCCTCGCACTCATCTAATTTTAATATGTTACAAAACGCCCACCTTTTTTTCAGAAG
GACCGCGTGCCATTCCATTATAGAAGTAAAACTCAGTTTTTTCCTACAACTCACACAACAGATTGAAAATAAAGCAAGGAAAGGAAAACGAACACCACAC
AATTAGCAGAAGTCAGGCCCTAGCACCTGCATTGAACTCGGATTCCTCACGTCATCAGAATGGTGATCCAGGGATAGACCCTGTCATTGCGCTCTGGCCA
GCAGCTGGGCTGGGCCATTCCACGAGCACCGGCCATGCACCATAAGTCGGCTTCCTCCTTGACTGAACGAAGCACCGTAGCTGTGCTCGGTCTGGCCGCT
TGGAAGACACACTAGTTGAGATGCTTCCAAATGATCCATGCTACTAGCATGACCAGGGATTTGACGCCTTTCCTGATGTCACCTCCAACGCTATCCTCCA
CGTGACGTCACCAATCGACGAAGATCACATCATGCGGTTGCGGGGTGTGCTACTGAAGACCGAGCTCCGCCAGCACCATGTGTCAAACCTCCCTCGTGAA
GATACAGGTAGTCAGGAGCTGCTGCATCGTCTCATCTTTCTGATCACAGAGAGGGCATTTGTCCGGTTGTTGTAACGCGCTATGCTTGAGTAGTACTCCA
TTCCCTCACACAAGATGTTGGCTGTAGGACGACCTGGATGCATGTTAACGGCATCCTTGAACATGGACTCTACAGCCCTTCTGGTGGGTTCGTCGGTACA
CTCGACCTCAATGTGTGCATCTATTCCCTTGAGGGCCGGTAGGTGCTTGATGCCAGCAGGCGCAGCGCCGTACCTCTCCCATTCTTTCTGCACATTAGCA
TAAAGCACCAACCTTTCGAGCCTGGCCATTGCCCCTGCCTTGAAGGTCCAGTACGACATCCTGCTACAGCGAACTTGAAAATGCTTCAGAACAGGGAACC
AGCTTCCACGGATGATGATCTTGTCTTCAGGAGCTCTTAGGATGTTCATGCTGAGTTGGGTAAGGGAGGGCAACTGTGCTAGCTTTCCGACATCCTCCTC
CAACACCTCGTTAATAACAAGCTTCAGGTCACAAAGATCATGGAGTTGGCCGATCCACTCAGGAACCCTTGAGAACCAAGAATTATATACATGGAATCTC
TGCAGATGGCAGAATGAAGCGGGTACTGAACTAAATACATCCAAGCAGCTGCCAGCCATAGGCTTCAAGCATAGATATCTGAGGTTGCACAATTTCTCAA
GGCAAGTGCGCAGAACTTCCCTACCTCTTTCTGCCAACTTATCACTAGGTATAGAATTGCCATAGTGAGGGTGCAAAACAATTTCAAGATCGGTCAAATT
GGTCAGTTCTCCGAGACCCTTGAGATTGTCTGGTTCCATGGCCAAATTAAAGCCGCGTAAGGTATGCAATGATTTCATGTATCCTATCCCATCAGGAAAT
GTTCTCCAACTTGGAACAATCAGACACGATAACCGGCTCATATGAACAATGTCCATTGGAAGTTGCTGATACTGCTGTCCTTCAGCTTGAAGTGATGAAG
ATCTAGTTTCTACTTCATAAAATGTTTCCAACTGTTGCAGACCCCCAATTTTACTAGGCAACAACACTACATGGTAGTAACTCGCTGCAATCCTTAAGTA
TCTCAGTTGAAATAAATGGCATATTGCACTGAAGTCCAGAAACACCAACAACGACTGTGACCCTTCTGGAATTTCAATTGTTAAAACTCGGAGATGCTTG
AACAGTGCAAAAGGAGGTAAATATGAAGAAGTTCCAAATCTTGCAAGCATTCGTATTTGGGATAGCGGGACAGATCCCACTACTCTATCGTCATCTATTG
CACCATCCAATTGGAGGCTGAGTCGACGGATCTTGCCATTCTGTCCTGTCAAATCTTGTACATCATCTATTACAGTGATAAAATTCTCTTCTCTGGACTT
ATGTACGATAAGATCAAGCATCATATCATGTACCTTGCAAGACACCACCTCACCGTTGTTGCAAGACACCACCTCACCGTTGTAGTCTGTATCTGCGGGT
TGAATCATACTCCTATTGATGAGCTCATTGAAATAGCTCTTTGCAACATGCTCCGGGTCTGTCCCGTGAGCTTTACTTATAAAACCTTCAGCTGTCCATT
GCCTAGCCAAATCATTCTTGGCTATTGTGTAATCCTCTGGATAAATACCCAAATATAGCATGCAACTCTTTAAATAATGAGGAAGATGTATGTAGCTGAG
GTTCAATATTTGTCTCATGCCTTCCAAGGTTGGGCTGACTTCAAAGTTGGAGCCCAACAATTTGTGTACATGCTGCCATTGCGCCTTCAGTTGGTTTGGC
TGACTGGCAAGAAGGCTAGATACCGTGATAATTGCAAGTGGCAAACCACCACACATTTTCAAAATTTGATCTGAAACTTCTTTCAAATATCGAGGACAAG
CGTCCTCTGAACCAAATACTCTAATATCGAGGACAAGCGTCCTCTGAACCAAATACTCTTTTGAAGAATAACCTCCTTGAGTCTTCACCATCAAGTGGCT
TCATCTTATAAATATAGTCATATTGATTACTACAGCACTCTCCGGCCACGGTTTCTATCCGCGTAGTTGTTATTACTCTGCTACCATTCCTGTTTTCTAG
AAGAGCACATGTAATAATATGCCATGCAGAAGCATCCCATATGTCATCAATTACAATAAGGTACCTCTTGTCCAAAAGGAACTCTCGCAGTGCAGTAATC
ATTTCATACGCATCCCATGTTTCCATGTTTGTGTGCTCAGGGGCTTTAAAGCCAACTTGAGACAGTATCATTCTCAGGATCCTCCTTATGTTTGGTTTCT
GGGATACAAAAACTAGTGCTCGACACTTGAACTTCCCCTCTAGCTTGCGATATATCTCATTTGCAAGCGTAGTTTTTCCAAGGCCCCCGAATCCTACAAT
AGAGAGCACCTTTCTTTCATGTGCAGACACACCCTCTTCATCCATCAACTGAATTAATTCGTCCCTCGGGCCCTCAATACCCACAAGGCCTGATGTCTCT
GCATAAATTGCCAACAAGCGGAGATCAATGGTTGTGTCCTTTGGCTTTGAGACAGCGTCGTCAACCTTGTACCTTGTGCGCCGCTCAGTCACCTCCTTGA
CACGGCTCTTGAGGCCTTCAAACTCTTTGGCAATCTCATGCCGAGTATTCATGATTGTCAACAAGTTCATGCTCCGGTCGATGAACCCCTTGAATCCACG
TGGCTTGCTGGACTGGCGTTCCACGCGGAGCATGAACTCATTGACGCTGTCCTCGATGTCATAGGACAGCTCACGTACCTCCTTGGCCCAACACTTGTCT
TGCTCGTCGGGCTCCTCGGTATCTGACATCCTCTTGAGGAATGCGTGCATACTCTCGAGCTCAGCATTGAGGAACATGACCTGTCCCTTAGCCCCTTTGA
GCAGCTTGTACTTGTCTGTGAGCAGATCACTAAGCTTCCCGAGCAGGGAGGCCATGGCGCCGTGCGATGCGGTCACCACGGCAGCCTCCATCGCCTTTCC
TTTCTTGTGCTTGCTCTCAATTTGACAGAGTG
>comp78295_c1_seq13
ACAACTGCACAACTTGCAAGAAAGCCACAAGCAACTAATGGGTGAGAAGCTTTCTGGCCTAGGTGTGAGAGACCTCCAGAGTTTAGAGAATCGTCTTGAA
ATGAGCCTACGTAGTATCAGAACGAGGAAGGTCATATTCATTTTGTTTCCACGTTTTGTTTTTCTATTCACTCATCTTAATATGCATAAACATCCATCAA
ACAGGACAATCTTTTGAAAAGTGAAATTGAAGAGTTGCACAGGAAGGGTAGCCTAATTCACCAGGAAAACTTGGAACTCTGTAGAAGAGTAAATACCATG
TCACAACAAAATATGGATCTACAAAGAAAGGTTTGATGAACGATCTCATGCAGAATCCTTAACCAAATGAAGGTGCATTGTCAGCATAAAGTTAAATTAC
TTAATTTCATCTCCAGGCGAGTGAACCAAGAGGTGTTGCCGATGCAAATAAAAGCTCTAGCACTCCCTACAGATCTTGTAGTGCACAAGATGCAGATATC
ACGGTTAATCTTGAATTGAGCCAATCACAGCAAAAAGAGGGGGAGCAGTGCCAAAGAGGGGCTCCAGAACTGGGGCTTCAGCTGCACAAAGAAGATGCAC
TACAAGCTGGAGAGTATCATGATGTTCCACCAATTCTAACATGCAATGCGGAGCACTTAAACTACAATTAGTATTTTGTGCAACATGTTAGATGAGTGAA
CAAAATGGTTTATGTACAGACTACAGATATGACCATCTATCTTATCTGTGGCTCTACTGTACTGAAATTGCAAGAAATGGTTATTGTAATGACTAATATG
ATACATTGACTGTCATACCGTTGACCTTGAAGATAGTTCTATAACGGAAAGGTCCAATTTGAAACAAGTGTTACGGATGAGCTAGTAGGATTTTTTTCAC
AAACAATTAGATAGTCCACGTTAGTGGTAGCTGCTGGGTATGACATTTGACCCAGCCTGCCGACCCTCCCTTTCCCATGGGAGTTCAATTAACATTAGGA
ATGAATATCGATATTATGGTTGATTATTTGAATCGTATGTTTTCCACCCTTAGATGTAAGGCCCTATTTTACTGTTTTAATGGATTAACAAGGTGAGCTT
TCGAGATGGTAGCTCATTGATTCGCATGCATTTTAGTGGCTGTTAAGAGGTACTTAGTGCATCCATGCTTGAAGGTGTTGTTAGTTCGACAAACCATTTT
CTATCCTACTGGATTACTATCTTTTGGTCAGTATGACAAGCAAGAATTTAATCTGCCAATTAAGAATTTAATGTGTTCTTTTGACATGAATGTCAACAGC
ATTTAAGTAACTCAACTTTTAATACAAAGTACTGGAATGTTTTCTCACCGAAAGTAAAGATGTGGTTCCTTGATAGCAAACAATGTTTATGCCAATATCG
CATTAAGGTGGTTGTATCCTTTAGAATGATCTCCTCATTCGTGCTTAATTTGATGCTATTGTCTTTTTACGGATGCCTAGATGTGGTTTTGACCATTACT
AAGTACTTTAATATATATTTAACAAAAATAATGACATAGCTGTTGTATGGCCAATCTAGATTTTGTTTTCGAAACATATTTGCAGTCAAACTATAGGAAG
TAAGAAACTTCAATGTGGTTCTCGAAAACCATGCTTAAAGTTTAGAAAGTTTGACTACATAGAGTATATACTACATAGTCTTAAAGCATCAAATGAAGAG
GAATGGAGGGAGTAGCAGACAATCATCATTCAATATCTATTTCTTGGTTGCCTTCAAATTTGGGCTTACTAAAATCGATTTAAAATTCTCGTGTTCCAGT
TGAAGCTCACTTCAAGTAAATCAGGAATTACCATATTTTTCATTGCTCATAATTTGGAAACATGGCATGATATTTGATAGAAAATGTACAAATCCTCAGC
TGCTCAGAAAGTTACAAAGGGAGGAACGCCCATGAAAGAGAATTCATGGCTGCAACAACGAACATAAACACAGCCTATTCTGTCCAGCAGTCATGGTAAT
ACAAGCTAAAAAGGACCGCTCTGGAACAATAGCTTGAAAAGCAATATTAATACAATTTCAGATGAACTATGTGCGTCGATGTCTTACTTGTGACTTCCTT
GCAAGTAATGCGGTTCCTGCCTCATGCTTGGTGCCGCCGCATGCAATAAACCGATAACTCGAATCCTCCGTGACTTCCACCCTCTCCAGCCAATCAGCGG
CTTGCTGCGTGCGGGCGCTGGTGTGCGGGGGGCGCCGGCTTTATATTAGGGGCGGGGGAGAGTCTTGAAG
>Locus_3441_Transcript_12/417_Confidence_0.050_Length_1413
CTGTGCTTTCATATTCAATAGTTCTGGCAAAGGGAGGCTGCAAGCTTGAGACAACAACTGCACAACTTGCAAGAAAGTCATCGGTATGGCTCGAAACCCT
GCAAATTTTACAAATGTACAAGTCAGAAAATTTGCCTAATGTAAAAGGATCAAACTACAGGATTTGTCAGCATGAGCACAAGTGCCTAACTTCCGAGTTA
GATAACTCTGGAGGAAGGAAATCCTTCCTACTGTTTGTTTCAAGAATATACAAAAATTCAAAACAACATTAGAATAGAACCAAAAGAAGAAACAATATTC
ACCATGTAGCAATACTCAATTCCTGGTTGTCTTATTTATAAAGGTATAAAACTAGGTTCTGACGAGACAATTATGAATCAACTACACCATGTTACAGATC
ATCCATAACTCTTGGTGATGCAAATTAAGGGCTGTAAAGTGACAAGTATACCTCCACCAACTAATTTGTGCAGAGTGTATAAAATTGTAAAACAATGGAT
AAGAATGAATTTAGTATACAAGATAGCATACAATGCCCAAGTTTAAGGAGACATACTGATTTACTGACTCCATAGGACAATCTTTTGCTTATGAGGACAT
CAGTTTTCATAAACAATATCAATGTAAAAGGAGGTAACAGTTTATGAATTTCCTATGCCAAGCCATTTATAAGTCAAAAAATGTACCTATTTAGCTTTTA
TGCATTCCAATTAACTTCCAAAGAAGAAGAGTACATACTAATGGAAAACAAATATGATATTATCATCTGTTTTGCCCGGTAGTTTCTTGAGTCTCTGATT
TGTCCGCACTTCACTCCCAACCAACACAACATTAATACACATGGGCAGAGGAATATCAGGTTATCAGGTAAACTGAATACAGTTTGATTGACTGCAGGCA
GTTGATGGGACAAGATCTTTCTGGATTGGGCGTCAAGGAACTCCAAAATCTAGAAAATCAGCTAGAAATGAGCCTACGTTGCATCCGGACAAAAAAGGAC
CAACTCTTGATTGATGAAATTCACGAACTGAATCGAAAGGGAAGTCTCGTCCAACAAGACAACATGGAACTATACAAAAAGGTCAACCTAATCCGTCAGG
AAAACATTGATTTATACAAGAAGCTCTACGAGAAAGAGGTGACAAGTGAAGTCAACCGAGATTCATCAACTCTAAACTTTGCAGTTGTCGAGAACGTCAA
CATTCCTGTTCATCTTGAACTTAACACTCCACCGCAAGAAAATGATGTTGAGCAAACTGCACCTCCTAAACTGGGGTAACCCTGCTTTTTTCTGCAAAGG

AACTATTGATTCTGTTCTTAATTCTTTAGTGGCATATTGTTTACTACTAACTGAAAACTTGCAGATTACAACTAAATCCATGAAGGCATGCAGCATGGCA
CCGCTTATGTTCA
>comp76332_c0_seq14
ACTGCTCTGAATTAAGAGGCGTTGAAATATTGTCCGGTCTACATATAGGCACTTGAGGTGTATTGACAGACAGTTCCCCTTAGTTTGCTACAACACATCA
GGCGAATTCCACCAAATTAGGATGATGTTTGTTTCTTAGACACAGGATAGCAATGCCAAAAGTCTTTTATAATTTTCCGCTTCACCGCAGATTTGGCATC
TCAAAGGGAGTTGTGGCAAGATTCTCTTGACCAAGCTTTCTTGCAGATTTGGCAAGAAAGATAAAAAAAATGTTGGTATACTTAAGCATCCTTAATAGAA
ACTAAACAGGTCTTAGATTGAAATTATCATGAATAAACCCTTTTTCACTTTAGCTGTAGTCCGGATGAAATCTTAGGGGCCTTGCTTAGCCTGCCCTGAA
GCTTGCCAGTAAGTCGAAGCTGATGCTGACACAGCACTGTTTCAAGTTCTCATCTTCTGAAAATGATCAGCAGTCAATAGATGTTTTAGTCCATTCTCCA
GAAAAATGAGTGCCGCATGAATCCTTTCCGGTTTATAGTGAAACATTTTAGACGATGACCTTTAACATTCTAGAAAACGAAGAGAAAACCTACAGTCATC
TAATTTCAGCTTAAACATGTTCCAGCAAGATCATCACCATCCTGACATGTTCCGTTTTGTCCCTAGACCCGAGCACAAGGTATTGCGAGGCTAATTTAGC
GGGGCAATTGATTTTTTGCCACTATGATACAACTGCAATTACCCGCTGTCACTGACACGTGGGGTCATCTGAGTCACTGAAATGTGGGCCCAATAGCATT
TTGTTTGCCACTCACCCTTCCAGAGTGGCAAAAAGTTAATTGTCCCTAATTTAGCTCAATTCAGTTGTCAGAGCAATTGTGTGTGAAAAGTGTGAACAAC
AGCATAGGAGACAATACAGCAAGTGTAGCGTGCCAATATGTGTCCAGCTTGATAGTTCACTATAATAAAGTTCTTTAATTCAAGTATTTGAATAATATAG
AATCTCAACCTTCAAGTTTAAAAGGTTTTGTATTTGCTATTATAATCGTTGCCACCCCCTCACCCCTCTCAAAAAGAGAGTTACTTTGTAGCGCACTGTA
AATCTCTTGATTGAAATAGTGGCATGTCATCTTATTTAATTCAGTTATTTCATAAGGTACAATTTGTTTAGCTGACAGCTCAGACATGCTTCTATGCAAC
ATGTTTATACCAATGCTGTATTTTGGCAATCCTGGATGTGGCATTACTTAACTGTGCTTTCATATTCAATAGTTCTGGCAAAGGGAGGCTGCAAGCTTGA
GACAACAACTGCACAACCTGCAAGAAAGTCATCGGTATGGCTAGAAAACCCTGCAAATTTTACAAATGTACAAGTCAGAAAATTTGCCTAATGTAAAAGG
ATCAAACTACAGGATTTGTCAGCATGAGCACAAGTGCCTAACTTCCGAGTTAGATAACTCTGGAGGAAGGAAATCCTTCCTACTGTTTGTTTCAAGAATA
TACAAAAATTCAAAACAACATTAGAATAGAACCAAAAGAAGAAACAATATTCACCATGTAGCAATACTCAATTCCTGGTTGTCTTATTTATAAAGGTATA
AAACTAGGTTCTGACGAGACAATTATGAATCAACTACACCATGTTACAGATCATCCATAACTCTTGGTGATGCAAATTAAGGGCTGTAAAGTGACAAGTA
TACCTCCACCAACTAATTTGTGCAGAGTGTATAAAATTGTAAAACAATGGATAAGAATGAATTTAGTATACAAGATAGCATACAATGCCCAAGTTTAAGG
AGACATACTGATTTACTGACTCCATAGGACAATCTTTTGCTTATGAGGACATCAGTTTTCATAAACAATATCAATGTAAAAGGAGGTAACAGTTTATGAA
TTTCCTATGCCAAGCCATTTATAAGTCAAAAAATGTACCTATTTAGCTTTTATGCATTCCAATTAACTTCCAAAGAAGAAGAGTACATACTAATGGAAAA
CAAATATGATATTATCATCTGTTTTGCCCGGTAGTTTCTTGAGTCTCTGATTTGTCCGCACTTCACTCCCAACCAACACAACATTAATACACATGGGCAG
AGGAATATCAGGTTATCAGGTAAACTGAATACAGTTTGATTGACTGCAGTCAGTTGATGGGACAAGATCTTTCTGGATTGAGCGTCAAGGAACTCCAAAA
TCTAGAAAATCAGCTAGAAATGAGCCTACGTTGCATCCGGACAAAAAAGGTAAGGATGCTAGCCATATTTCTGTTTTTCGTTACCAGATTGCCTCATCCA
AGAATACAACTGATGATGACGTTTTTTACTTTCAGGACCAACTCTTGATTGATGAAATTCACGAACTGAATCGAAAGGTTCTGGAATATTCTGTTATTAC
TCTGCAATGAGAGGTCTCTGAGTGGATTTCAAGAAGCTGACTCTACTGGCTCATTTTCAGGGAAGTCTCATGCAACAAGACAAGATGGAACTATACAAAA
AGGTCAACCTAATCCGTCAGGAAAACATTGATTTATACAAGAAGCTCTACGAGAAAGAGGTGACAAGTGAAGTCAACCGAGATTCATCAACTCTAAACTT
TGCAGTTGTCGAGAACGTCAACATTCCTGTTCATCTTGAACTTAACACTCCACCGCAAGAAAATGATGTTGAGCAAACTGCACCTCCTAAACTGGGGTAA
CCCTGCTTTTTTCTGCAAAGGAACTATTGATTCTGTTCTTAATTCTTTAGTGGCATATTGTTTACTACTAACTGAAAACTTGCAGATTACAACTAAATCC
ATGAAGGCATGCAGCATGGCACCGCTTATGTTCATTATTCTCAACGGTACGATTCAAAGCAACCAGAAACACTATTGGTTGTAAAGAGCTTAAACACATA
GCAAGACTGACGCGGTATGCATGCCATGAGTGATTGATAATGGTAGTGTAAATCTATTTCAAATAAAATGTATTTTATTGAAGAATACAGCAAATGATTT
GTATTATAACTATTTGACACATTCTGCAAGTTTGTTGACCAACGTGAGTACATGATAGCTACAAATCTTATATTGACAAAAAGATATGGTGTGTGGAAAA
AATTCAGGTGCTATTTTTTTCTATTTTGAGAATTGTATGCATAGCAGAAGAGCACAAATTGCATTCTGGTTTATCCGTGCAGGCATTCCAGAGTATCAGA
ATGGGGATCAACCTTTCTTCAGCTACATATGTAAATATACGGATGCTAGTTGCTCACTTAAAATTTTGTAGTGTTCAAAGGAATAACAACAGTAAGACGT
TGACGCACCGAAAATAACCAGAAATAACGGATTGTACTATGACAAGACACACGACAGATACACTAGCTTAAGTTTGTACAGGAAAATATTTGGCCTATTC
TTATGTCACAGTAAATTAAGATTATCACCTTAATCACAGAAATTCAAGGGGTAAACGATTATAGCACAAAACGATCCTGGACCCCAAGACCTGGGTCTCT
GAACGGCTGAACCTCCAATTATACCTCTATACATTTGGAATGTGTGTAGTCAAACTTTCGCTACTGGTATCTTTACAAATATTTACATTGAACACATCAA
AATGATATACATGGATTCCTCTCGATATGTCTTTGAGGTTTGGGTTCTAAAAATGATATACAAATTATTAGGTTCTAAACTTAAATGATATATACACATC
AAAATGATATACAAATTATTGGGTTCTAAACTTAAATGATGTATACACATCG
>comp76332_c0_seq30
ACTGCTCTGAATTAAGAGGCGTTGAAATATTGTCCGGTCTACATATAGGCACTTGAGGTGTATTGACAGACAGTTCCCCTTAGTTTGCTACAACACATCA
GGCGAATTCCACCAAATTAGGATGATGTTTGTTTCTTAGACACAGGATAGCAATGCCAAAAGTCTTTTATAATTTTCCGCTTCACCGCAGATTTGGCATC
TCAAAGGGAGTTGTGGCAAGATTCTCTTGACCAAGCTTTCTTGCAGATTTGGCAAGAAAGATAAAAAAAATGTTGGTATACTTAAGCATCCTTAATAGAA
ACTAAACAGGTCTTAGATTGAAATTATCATGAATAAACCCTTTTTCACTTTAGCTGTAGTCCGGATGAAATCTTAGGGGCCTTGCTTAGCCTGCCCTGAA
GCTTGCCAGTAAGTCGAAGCTGATGCTGACACAGCACTGACATAGATCCCAATGAACACAAGTTATTTCGAGAAGATAAAATACCAAGCAAAGCCAGGAT
TGCATTAAAAGATAGTGTATTTCTACTATACGATCTGAAAGCTTAATTTATTATATCAATAATCAAATTGGCAGCAGTGTGGAACAGATATAAAGAAGAT
GCTTCAAATCACAAATAACTACAGATAAATTGAAGGGTGAAGGTTCATTCAACCACCAAACACCCAAAATGTCGAGCTGCATATGCACAGAACCTAAGTA
CGGTACAGTTCATAGTTTGGGAAAATAGACTGCAAATGTGCACAGATAATACAGATTGTTACTCGATGAACAGTAAGCACGGTATTTGAAATCCTTCTAA
CCATCCATCCAGGCAGTGCCTCGTATACTCAAACCTCTTTTTGGAGGAAGCCAAACCATGTGATCCTTTTCAACCCAAATACATATATTCAAGATGAAAC
TAACAAAAAGACAGTTGATAAAGAAACACGAAAGAGAGTAGAAAAAGGGTACCTGTTTCAAGTTCTCATCTTCTGAAAATGATCAGCAGTCAATAGATGT
TTTAGTCCATTCTCCAGAAAAATGAGTGCCGCATGAATCCTTTCCGGTTTATAGTGAAACATTTTAGACGATGACCTTTAACATTCTAGAAAACGAAGAG
AAAACCTACAGTCATCTAATTTCAGCTTAAACATGTTCCAGCAAGATCATCACCATCCTGACATGTTCCGTTTTGTCCCTAGACCCGAGCACAAGGTATT
GCGAGGCTAATTTAGCTCAATTCAGTTGTCAGAGCAATTGTGTGTGAAAAGTGTGAACAACAGCATAGGAGACAATACAGCAAGTGTAGCGTGCCAATAT
GTGTCCAGCTTGATAGTTCACTATAATAAAGTTCTTTAATTCAAGTATTTGAATAATATAGAATCTCAACCTTCAAGTTTAAAAGGTTTTGTATTTGCTA
TTATAATCGTTGCCACCCCCTCACCCCTCTCAAAAAGAGAGTTACTTTGTAGCGCACTGTAAATCTCTTGATTGAAATAGTGGCATGTCATCTTATTTAA
TTCAGTTATTTCATAAGGTACAATTTGTTTAGCTGACAGCTCAGACATGCTTCTATGCAACATGTTTATACCAATGCTGTATTTTGGCAATCCTGGATGT
GGCATTACTTAACTGTGCTTTCATATTCAATAGTTCTGGCAAAGGGAGGCTGCAAGCTTGAGACAACAACTGCACAACCTGCAAGAAAGTCATCGGTATG
GCTAGAAAACCCTGCAAATTTTACAAATGTACAAGTCAGAAAATTTGCCTAATGTAAAAGGATCAAACTACAGGATTTGTCAGCATGAGCACAAGTGCCT
AACTTCCGAGTTAGATAACTCTGGAGGAAGGAAATCCTTCCTACTGTTTGTTTCAAGAATATACAAAAATTCAAAACAACATTAGAATAGAACCAAAAGA
AGAAACAATATTCACCATGTAGCAATACTCAATTCCTGGTTGTCTTATTTATAAAGGTATAAAACTAGGTTCTGACGAGACAATTATGAATCAACTACAC
CATGTTACAGATCATCCATAACTCTTGGTGATGCAAATTAAGGGCTGTAAAGTGACAAGTATACCTCCACCAACTAATTTGTGCAGAGTGTATAAAATTG
TAAAACAATGGATAAGAATGAATTTAGTATACAAGATAGCATACAATGCCCAAGTTTAAGGAGACATACTGATTTACTGACTCCATAGGACAATCTTTTG
CTTATGAGGACATCAGTTTTCATAAACAATATCAATGTAAAAGGAGGTAACAGTTTATGAATTTCCTATGCCAAGCCATTTATAAGTCAAAAAATGTACC
TATTTAGCTTTTATGCATTCCAATTAACTTCCAAAGAAGAAGAGTACATACTAATGGAAAACAAATATGATATTATCATCTGTTTTGCCCGGTAGTTTCT
TGAGTCTCTGATTTGTCCGCACTTCACTCCCAACCAACACAACATTAATACACATGGGCAGAGGAATATCAGGTTATCAGGTAAACTGAATACAGTTTGA
TTGACTGCAGTCAGTTGATGGGACAAGATCTTTCTGGATTGAGCGTCAAGGAACTCCAAAATCTAGAAAATCAGCTAGAAATGAGCCTACGTTGCATCCG
GACAAAAAAGGTAAGGATGCTAGCCATATTTCTGTTTTTCGTTACCAGATTGCCTCATCCAAGAATACAACTGATGATGACGTTTTTTACTTTCAGGACC
AACTCTTGATTGATGAAATTCACGAACTGAATCGAAAGGTTCTGGAATATTCTGTTATTACTCTGCAATGAGAGGTCTCTGAGTGGATTTCAAGAAGCTG
ACTCTACTGGCTCATTTTCAGGGAAGTCTCATGCAACAAGACAAGATGGAACTATACAAAAAGGTCAACCTAATCCGTCAGGAAAACATTGATTTATACA
AGAAGGTATTTGATAAATCGTGAATGAAAATTCACAGGATATACACCCCATCCATTATTGACTGAGGTTTATTTCTCTACTCAGCTCTACGAGAAAGAGG
TGACAAGTGAAGTCAACCGAGATTCATCAACTCTAAACTTTGCAGTTGTCGAGAACGTCAACATTCCTGTTCATCTTGAACTTAACACTCCACCGCAAGA
AAATGATGTTGAGCAAACTGCACCTCCTAAACTGGGGTAACCCTGCTTTTTTCTGCAAAGGAAGATTTTCTTGCAAGTTATGCAGTTGTTGTCTCTAGCT
TGCCGCCTCCACTTGCAGTAACTGAGACGCCGGCGCCGAGCTTGAGCTTGAAACCTAGACTACGGGGACAGGGTAGACAGGAGACGCGAAACCCCAAACC
AGCGCAAAACGACGCGCAGGGAGGGAGTGTCACTGAAGGAGCGACAGGGAAAGCAGCGGCACGCCGGGGGAAATTCCGATCTGAGAGAACGGATCAGCCA
TGCATGACAGGAGCTGCGGAGGCAGCAGGGGGGGGGGGCTCCCGCTGCCACGGGACAGTAGGGCACGCCAGATCGGTGAAATCCAGGCGAAGGACGAGGC

GTGGGCGAGATTTACACGACGGCGCGATAGGATCTAGCCCGGATTTGGATTCGACCGACGAGGGCGGAAGGAACGGCAGGGTGGTGCTCTTGTAGAGAGA
GAAGGAGTCGGAGGGGGCGGAGAGAGAGAAAGGGGGCAGGTGGGGTTTCAGGTAGTAAAATATGGAGCGG
>comp82180_c0_seq25
AATATAGGGGGCCCCGCTCCCGCAGCTCGCAGTACCGCGGCGTCACCTTCTACCGCCGCACCGGCCGCTGGGAGTCCCATATTTGGGATTGCGGCAAGCA
AGTGTACTTAGGTGGATTTGACACTGCTCATACTGCTGCAAGGGCGTACGATCGAGCTGCCATCAAGTTCCGTGGCTTCGACGCCGACATAAACTTCAAT
CTCAGTGACTATGAGGATGACATGAAGCAGATGAAGAGCCTGTCCAAGGAGGAGTTCGTGCACGTCCTGCGACGGCAGAGCACCGGGTTCTCACGGGGCA
GCTCCAAGTACAGAGGCGTGACCCTGCACAAGTGCGGCCGATGGGAGGCTCGCATGGGGCAGTTCCTCGGCAAGAAGGCTTATGATAAGGCCGCGATCAA
ATGCAATGGTCGAGAAGCTGTGACGAACTTCGAGCCAAGCACGTATGATGGGGAGCTGCTGACTGAAGTTGCTGATGAAGGTGCTGATGTTGATCTGAAC
TTGAGCATATCTCAACCAGCTTCTCAGAGCCCGAAAAGGGACAAGAACTCCCTTGGTCTGCAACTCCACCATGGATCGTTTGAAGGCTACGAATTGAAAA
GAACAAAGATTGATGCTCCCTCTGAACTCACGGGCCGACCTCATCGGTTCCCTCTTCTGACCAAGCATCCACCAATCTGGCATGCCCAATCTCATCCCTT
CTATTCAAATAATGAGGTTTGATGATACTAAATTACTAAAATACAGGCAATTACTTTCCCAAACATATTGCTTTGGCTCCTAAACCTCACAATTGCAAGC
ATGTTCTGAGCATCACATGGTTCGGATCGAGCTGTCCGGCACCAATTTTGAACGTAATCTTGACCATCTGAGGAACTGATTGTGCAGCCTGAAGGCGTGC
GTTCAAATGTTAGATTTGTGTTACTTTCGTTCATTTTCACTTCTTGTGCCGCTGTCAATTTTGGCTCTAGTCTTACATCACCGCTTCATTTCCTTGGATT
CTATAGCCCGCCTGTTTCCTGTCGATTTCGGAGATATTAATGCGTAGCACAGGTTTCATATGTAGGATCTTGAGTTTTCTGAGATGGCCCATTCTGGAAA
CAGTAATAGTCGCCTTAGGAATAGAGATGTGTTTGATTTTTTTCCTTCAAAAGGAACATCCTAGAGAGGTGACTATTCGACATTAGGTATGCTAATCCAT
CGGTCAACTATACCTGCCTGCGGCTAAGCACCTTTCAATGTTTCTTTTTTCATTCATCGTATCATATAAAAGTAGTAAGTATGACTATTATTCAAACCTT
TCTTGAAAAAAATAGTTAGGATGTTGCCATCTTGTAATGCAATCATAGAATTGGAAGTGCTGAATTTGCCATTTGTTTCAAACAAGCATTGTATATTGGT
TTATCATTCAACTTGAGAGTTTGGTCAAATTGAACTCTCGAACTTTTCAACTCCTAAACTTTAAGGTCAGGCTCACATATGTCATTTTCAAATTTTATTG
GACGGAAAGTTGAGCTGGACGTTTCGAGCTCGGCTTTCTATCCAACTGAACTAATGGAAACACTGATTTCATCGCAACAAATTTTCAGCAATAGATATTC
AATCTCTTCTTGAATTCATCCTTCATCAGTATGGTGAATAAATTGTTCTTGGATTATGCAGAATGCATCAAGAGATCTTAACAGGAGGCCAGATCAGGGG
GGTGTTCCCAGCTGGGCGTGGAAGGTGACCGCCCCTCCTCCCACGCAGCCATTGCCGCTGTTCTCGTCGGCGTCATCATCCGCTGCAGCATCATCAGGAT
TCTCCAATACCGCCACGACAGCTGCCCACACCGCCCCATCGGCCTCCCTCCGGTTCGACCCGCCGCCGCCGCCGTCCAGCCGCCACCGCTGAACGTTAAG
AAGCCACGCTGTAAATTTGCCAGGAAGCCGACATTTTTTCCTCTCTCGGCGTTGCAACTTTTTAGGTTTTGCGCCGGGGTGGTTTCTTGTAGTGGAGTGG
ATTCATAATTAACTGTATTTGCATGCTGCCCATGTGAAATGCTTCTCTCTCTTTTTACGCTCTCTGCACCGGATGTTCTGGGAGTTCTTGTTGCCGGGAA
TTAACTAAATTCACCGTCTGAGATTGATCTATACATTGTTGTAGAGAATCGAATCATTGTTGAAATTAGTTACAGAATTATAGATATCATAAGGCACCCT
CTACTTGTGGACAAAGTTCAGGAAGATTATTCCGGTTCTTTTCTTGGCTGTCAATTCGGCTCTTGTCCTTGGTGTGGAGCCACTTGCCTACTGTCTTGTT
CTGTTGCTGTTTGTTCTTGGCTTCTTGCACAGTTACAAATTTGTTGGCTCGAATGGATGGTGAGACATTTGGTGCAA
>comp63680_c0_seq9
AGTATCTCGGCGTGGGACAGAGGCCGGTGGTGGTCGCGAAGAAGACGCGGCGCGGGCCGAGGTCCCGGAGCTCGCAGTACAGGGGCGTCACCTTCTACAG
GAGGACGGGCCGGTGGGAGTCGCACATCTGGGATTGTGGGAAGCAAGTCTACTTAGGTGAGTAACTGAGTTGAGTGAGTTCTATACGTTCCAGCTCGAGC
TTCCATTAACTGCTGATCAGTTTCCGTCCGTTTGATCTGGTTTACGATGTGCTGATGCTGTTCTATTCAGGTGGTTTCGACACTGCTCACGCGGCTGCGA
GGGCTTATGATCGTGCAGCGATCAAGTTCCGAGGGCTCGACGCGGACATCAACTTCACTTTGAGCGACTATGAGGATGACTTGAAGCAGATGAGCAATTG
GACAAAGGAAGAGTTCGTGCACATACTCCGCCGCCAGAGCACGGGGTTCGCGAGGGGGAGCTCCAAGTACCGCGGCGTGACGCTGCACAAGTGCGGCCGC
TGGGAGGCGAGGATGGGTCAACTTCTTGGCAAGAAGTAAGCCAACCTCCTGGCTTTGACCTTTCTACTCTGTTAGCCTAGTTAAGTGTTTTTTACCCTGG
TTATATGAACAAAATATGTCGAAATGATGTTCAATTAACTTGTCATTTCACGTTGGGTTCGATCAGGGCTATATCCCTTATGTTCATGTTAAGTAGTTTC
AGGTGATTGTAGAGAATACAACAAGGTGATCTTGAAGTATCACTGATTATAATCCATGCTACTAGTAGTTTGAGCGTCTCTGTTGCCGGTGAAATCTCCA
CCCCTCTTGTGACTTTACCCCTCTACCGTTTAATCCGGGAATCCGGAATGGACCTACAGATCGAGATATGTGCCTTGATGAACAGTGTTTCGGTGAACAG
CAAAATCGGCACCCGTGCACAGTGTCCTGATGGACGGTACATCTATCTTGGACTCTTTGACAGCGAAGTTGAAGCTGCAAGAGCATATGACAGGGCAGCC
CTTCGCTTCAATGGGGGGGAAGCTGTTACTAATTTTGGGCCTAGCTCCTACAATGGAGGAGATGCTCTACCCGACACCGAAAATGAGGGTATCACAACCA
GCCTCGCCATTTATTTCTTTTCATATAAGCAAATGTATCTCAGGTGCACCTTATAATCATCGCTGTGTTCTTAGCAACTGTTGATGGTGACGCGGTTGAT
TTGGATTTGCGGATTTCACAACCTAATGTGCACGACACTAAAAGGGACAATACCTTAGCTGGAGGCCAGCTAACATGCGACTCCCTTGAATCTTCAAACA
CGATGGCCTCTCAGCCAATGAGCTCATCGTACCCGTGGCCTGTGTATCACCAAAGCACGACAGCAGTACCACCTCACCATCAACGCTTGTACTCATCTGC
TTGTCCTGGCTTCTTTTCGAACCTCCAGGTATTACCAAGCTAAAGCCTATTTTTTATGTGCAAATTGCTACTCCTACGTGATCAATTAGTTCAAACTGTT
ATTCGAATCCAGGATTGTTTACTGAATATGTCCTGTCTTCAGAAAAAATAACACAAAAAGGAACATAATATGTCCTGTCTAATTGTGGACTGATACTAGC
CAATTAAACAGTTTCCCATATCCATTTCCTGTGTTTATTGTTTTGATTTGCTGGCATGGTTAAACTATAGTAGCTTGATTCGATAAAAACACTAAGCAGG
CTTTGATATTTTGAATGAAATGTCGTTCTTGGAAATACAGCCTTTTCCTTGGTATATCAAGGACAGTTGTCCATCTGCACTCTGCACCCTCAGCTTGTGT
AACTGTTTGCCTTGTGAACTGATTGATGTGTCAATCAGGCAGTTCTGCCAGTTTGCATCTTTTGAAATAGTGCAGTCTTCCAGTCTAACAGCTCCATGAC
ATAACTTGGGATGCATGCTGCAGCTTTCTTACTGAGGATGCTCTAGATTTACAGAAATTCTCGCAGTTAGCAGAGGGCTATCAAATCTGATTTGCTTAGC
TTCCTGCAGACAATAAGAACTCTAGAATCCTGAAGAAATCTTACTTGTACATGTTTACCAAAGTATCCTTACTCCTTAGTTGAGCCAATTTTGGTGAAGA
ACCGATAGCTCATTACTCTGTTTCCTGGGAATGATGTGGGCCTAAACCATTCTGTGGTCAAAGTTTAACAAAGCCCATGCTTGTGCTGTTGCAACTGCAG
GAAAGGCCAATGGAGCGAAGGCCTGAGTTGGGTACCCAGTCATTCCCCACCTGGGGATGGCAAATGCAGGGCTCCCCTCACATGCCGTTGCATCACTCTG
CAGCATCATCAGGATTCTCTACCGCCGTCGGCGCAAACGCCGGCGTCTCGTTGCCTTCTCACCCGCCGGTGCCGTTCCCGGACCACCAGTTCTACTTCCC
ACCGACGGCATGAGCTTGTTGGTCTGGTTCAACTCTTGGGACGGCAGCTGATCTTATGTGTGAACATTTCGCAGCTTGCCGGTGACCGTTGTTAATTAAT
CGGGGAGAGAGAGAGCCAGAAGACCCAGTATGGCTATCTCTCTGTCGCCCGTGCCTTGTTATGATCTTAGTTATTGCTACTACACCGTGGATTCATCATGGT
TTATGAGATTCTTTACTCAAGTTTCAGCTTAAATGTCCAAATACATGAATGCATCATACACAGGATCCGTCATTTTCTAAAAAAATACAGGATCTGTGTG
AAGTGTAAACATAAGG
>Locus_12535_Transcript_29/73_Confidence_0.292_Length_2373
AATTTTTTAAAAACTGAGTAATAGCTATTAAAAACTAGCATAAAACTAATTTGTTTAGATCCCACCCACTTTTCGAAAGTCAGTGCAAAAAAACTGAAAG
GATCCGAGCAGGCCTTAGAAGAATTTCAGCAAGACAAAGATGCTTCAGAAAAGGAGGTGAGATTCCTGATTTCACATAAGTCAGTTCAGAAAAACTGAAA
GGATCCGAGCAGGCCTTAGAAGAATTTCAGCGAGACAAAGATGCTTCAGAAAAGGAGGCTTGATGTGGTATATGTTTTCCCCTTCCCCGACCTTTTGATC
TCCAACCATGGCAGTGGAACAAGGAGCAAATTTGCAATCTTGGGGTAGTTCCGGTGGTGAAACGTGAAAGATTGGGGATTTAAGGGACGTGAAACTGAGG
GGGTGGTTGTCATGATGTGTAGGGTCCAGCACTGGAGTGGTTGATGCTGTGGTTTCCAGGTTAAGGGGAGAAGAGGAATAGGATATAGATCTCAGGAAGC
CAGAGATGAGGTCGAGGGGAAGCCAGTGGCCAAGCTTGTGCTCCAGTGAAGCTGATTCTCTTGTTCCCAGTTGATCCATGCGCCGTGGCCTCAGTTCGCA
GGATCAGCAGCTCCATGGTTCTGTGTGGTGCTGCAGTTTGAGAAAGTGGTTTTCCAGGGGGAGGAATCTCTTGTGGATTCCTTTTTCTGGGTGCAACGAA
GTGGGAGGTAGGAGCAGCAGTTGAGATTTTCAATTCTGGCATGGGTTCTTTTGGGATGGACTGGAACCAGAAAGGCTCGGTGTTGTGGGATTGGGAGAAT
TTGCCGCCGATAGGCAGAAATGCAAACGAGAACCTCAAGATTGCGCCACAGGCTGAACCGAAGTTTGCAGGTGTTGAGGCAACAAGGCATGAATCGGGGC
ATTCTTCTTGTGGTACTTTCTCTTCCAGCTCGGAGATGGGGTATGGTTCATCCAAGAGTTCCATATCAGCGTCGATTGATTCTTCACCCAAGGTGGGAAA
CAACGTGGAGCTCAATCTTGCAGCTGTCAAAGTGCCTGACAAAAACACCGTCAAGAACACTGATTTGGGTAAAGTTGATGACGCTGGAACTTCTCCATCA
TCGATGATAGCCGTCAGCAGTGGAGAACCGGTGATTGGCCTGAAACTTGGCCAAAGAACCTATTTTGAAGATGCATGCGGAGGGCAAAGTGTCAAGAGTT
CACCATCCAATGTGAGTGCAGTGACCCCTGCTTCTGCGAAGAAAGCAAAGATGATTCAAAACGCACAGAACTCGTACTGTCAGGTTGAAGGTTGCAAGTT
TGATCTCTCTTCTGCTAAAGATTATCATCGGAAGCACCGAGTCTGTGAAACTCATTCTAAGGCTCCCAAGGTTATTGTTGCTGGTCTGGAGCGACGCTTT
TGTCAGCAGTGTAGCCGGTTCCATGCTTTAACTGAGTTCGACCAGAAAAAACGAAGCTGCCGGAGACGCCTCAATGATCACAATGCCCGCAGACGGAAGC
CACAGCCTGAAGCAATTCCTTTCGGTTCATCAAGGCTCTCTGCAATGTTTTATGATGCAAGGCAACAGACAAGTCTTCTGTTTGGTCAAGCTCCTTATGG
TCAAATGAGAAGCTGTGCAAACTCTTCATGGGATAGCCCAGTAGGAGGCTTCAAATTTGGAGAAACAAAAGCTCCTTGGTTAAAGCCAACGAGAGCTGCA
GGTGTTGATGGGATGCATGTATCAAGCCAGCAGGTGTGGAACAATATTACGCCACACGGTGCACATCATGATTTTAATGGTATCATGGCTTTCAAGGGAA
CCAGTGCAAATGTCCTTAATCAAGGTGCCGAAGCTTCTCCGGTCGTCTCCAACTCGAATGGAGCCCCAGATCTTCAGCGTGCTCTCTCTCTTCTGTCAAA
CAATTCAGCTGGTGCTGCCAACGACCAGCCAACTCCTCAGCTGCACCCTGGGCTGACCACCTTCGTCGGCTCCTCCAACCCTGTCACTGTGATGGAAGCC
TCACCACCAGGACTCTGGCAAGACAGCACGCCCCTTGATCATCAGGCGCAGTTCCAGGCTTTCGATCCCATTGCCGCACCGCAACAGCTCCAGCTCCCAA

AACCCCCTTCCTTCAACAGCTCCACTTCCAGCTATGGCCAGATGCAGTGATGGATGCTTCCTTCAGTTCTGCTGTCTGAAACTCTGAAATTTGAAACTGC
TGTGTTCTAAAAAATAAAGAAAAGCCTGTGTGCGAAAACTCAGGCTTGAAAAATTGATCCAGTAGTGTTCCCGTTCAATGGCTTGAATAATCCATCCTTG
TGATGGCGTGTAACTCAAATGCTTGGATTGCGCTGGCAAGTGATCATGGACTCTTGTGTTCTTGATGGCATCT
>comp86229_c0_seq9
CGGGAGCGCGGCGACGAGCTCGGAGGCGACGCCGCGGACGCCCGGGACAAGCTGGCCTCCTTCGCCAAGACGCTCACGCAGTCCGACGCCAACAACGGCG
GCGGGTTCTCGGTGCCGCGCTACTGCGCGGAGACCATCTTCCCCAAGCTGGACTACCGGGCCGACCCGCCGGTGCAGACGGTGCTCGCCAAGGACGTGCA
CGGGGAGGTGTGGAAGTTCCGGCACATTTACCGGGGCACGCCGCGCCGGCATTTGCTCACCACGGGGTGGAGCACGTTCGTGAACCAGAAGAAGCTCGTC
GCGGGGGATTCCATCGTGTTCTTGCGCACCGAGCATGGCGAACTGTGCGTTGGGATACGGCGTGCGAAGCGGGTGTCTTGTGGTGGCATGGAGTGCATGT
CAGGGTGGAACGCCCCGGGGTATGGGGGATTCTCGGCGTTCTTGAAGGAGGAGAGTAAACTGATGAAGGGTCCAGATGGGTACATGAGGGGCAGGGGGAA
GGTGAAGATTGCGGATGTCGTGGATGCGGCAGGCCTAGCGGCGAGCGGGCAGCCTTTTGAGGTGGTGTACTACCCGAGAGCTAGCACGCCGGAGTTTGTT
GTGAAAGCCGCATCAGTGCAGAATGCGATGAGGATCCAGTGGTGCCCTGGGATGAGGTTCAAGATGGCATTTGAAACGGAGGATTCATCAAGGATTAGCT
GGTTTATGGGGACAATATCTTCTGTTCAGGTTGCTGATCCAATCCGATGGCCGAATTCACCTTGGCGGCTTCTTCAGGTTTTTACTTGATCGAAAGTTTT
TCATAGTTGAATACTTATATTCCATACTGCATTATAGACTTTTGTCTTGAGTGGGTTACTACTTGCTAGTATGATGTGTCTGGTATGTACGGGTGTGTCA
CTATTCTTCAGAAGTTCTTTTCCCTTCATATGTTGGCTCAGCAACTTGCTCAGATTACATCTAGCAAATGAATTGGTACTTGGAAGAGTAGAAAAAAGAA
TATAGAAAAGAAAAACTATTGGGGATTTGTGATGATTGGTAGCAAAATAGTCATGAAGGAATGGTTTTAGTTGTATGGACCGTTATTGGTTCTTTAAACT
TAGTGTTTATCTTTTCACTAAGTTGCTGTCCTCTGCCAAATAATTCGCTTTAAAACTTTTTACTGTTCCTTTTGCAATTACAAATAGCATCCAAAATTCC
TTCCTTGCAGCCTCAGACAGTCCGACTAGCTCGGACATCACATGTTTGTAGCCTTTAGCATAGCCCACATGATGCATCCAAAAGGCATTGGGTTACTTGA
CAAACTTGCAGTTAGAAATGGGATGGCTGAGGTATGCAGACATTCTAAGGTTGGCCGAAAAATTAAAGGTCTTAATTGTGTGGACTAGGATGAGAACAAA
TTGGACATAATTCTAAGAAAAAGGAAAAAGAGTACTACTTTACCTATAGGAAACCATGCTATCTGTTGGGTTGGTTTCTTGTTTGGATCTCGTTCTCTTT
GAATTGTTAGTATGTTTCATCAGTCTCTTGTGGTTACTGAGAACTGGGATGTATCATCATAAGCTTCCGAAAGCCCATTGGTCACCATTATCACCGTTGA
GTAGCCATTGACAGATTCAGGTTTGAGCATCCTTTGGCTGCTTAGCTTTCTGTCTCTACACACTTGAAAAGCTGGTAAGCGTCATTAATGTGACATCTTC
CCTAACTAATACTCACAAGAGGGAGGGAGGGAGATGAACTTGTATTGGTACAGAACTTTCAAAGCTTGCTATTGACTTCGCGAAAAGAGACAGCTGCTGA
ACAAGCAAAAAGGATGACCACCGATTTGGCACAACTACTCGAGCACTCCCATAGCACCAATAAAATTTTCCTACCATCAGACTACCTTTGCTTCTCACTA
TCCTGGTTTAATAATTTCATCACATAAGAGCCCAATGAATCAATTATTGATTTTATTTTGTTAGCCTCAGGAGGCATTATAAACACATTGAATTTAGGGC
TCAATACCAGTTTTGCTTTTTTAAATACATTAATTGATCTCTCTCTTAAAGCCCAAATTTCTCTATGTTTTGTAGCCAAATTAAAGCCTACTGATAGACT
GATATTACATAGTTATAGCTCAAATGTGAGGAGTGTACTCTTAAAACGTTCGTGGACAACAACCAACATAATTTTTAAACTGTGGTTCTGTTGGTCCTGA
ACTCCTGAAATAGTGGCAGTACCTTACATTTGTCTTTATGATCAGCCCCCTCATAAGATCGCTAGCGGATCCTTAAAAGCAGCCTGTAGCTAGCATTGTA
CTACATTTTGTACCGTGGGAAAGAGGTGTTGAACTAGTAGTAATAAGACTGATCTAGCATTAATTTAACCTAAACTTGCTAGCTCTATTTTCAGATGTAC
CTTAGGACCTTTTGGCTCTGTATCTCTCTCTTCGCCAATAGTATGTATTGATTGGTATTTTTGGAAGTGCTCGCTATATACTTTATCTGGCAATGTAGTA
CTACAAAGGGTGGAGTCTCACTAGTTACTACTAGTGTTAGGTGTGATATTGGAAACTCATCAGCTTATAGTAGCTATGATGTCATCAACTATAGCCGATC
TAGCAAGTCATCAATTACTTGTGAAATTGGTATAATAAATTGTCTGTAATTGCCTGCGTACTTGCTTTCATTGTTTGTACTGTATCTCTGCTCTTTCACA
TAGCTTCAAATTTCGTACAGGTGACATGGGATGAACCAGATTTGTTGCGGAATGTGAAATGTATCAGCCCATGGCTTGTGGAACTCGTATCAATCATTCC
CCCAATTCACCTGGGACCATTTTCTCCACCTAGAAAGAAGTTGCGGGTGCCCCAACATCCTGACTTTCCATTTGATGGTCAGCTGTTGAATCCAATTTTC
CACGGCAACCCACTTGATCCTAGCAACAGCCCCCTATGCTGTTTCCCGGACAACGCTCCTGCAGGCATACAGGGAGCCAGGCATGCGCAATTCGGTCCAT
CCTTCTCGGATCTCCACATCGGCAACCTGCAGCAAAGCCTGCTGTTCTCCGGCATCCTCTTCCCTGACCACCACGCCCCTCACCCTCCGAGGATCAGCAC
CGACCTGACAATTGGCAGCCCACACGCTGGCCAAGACGCGGCGTGCTCCCTATCGTCTGGCGCCAAGAAGCCTGACGACGTCAAGGCGACGAGACTAGTG
CTCTTTGGACGGGCGATACTGACGGAGGAGCAGATCAAGGCCAACGCTTCCGTTGGCCCGTCCTCGCTGGGAGCCACCGGGAACGGCTCGTCCAAATCCG
ACTGCGACGCCGAGAAAGCACCTACCAACACGTCCGAAGGCTCCAGCTCAGGTGTCATCCAGGGCAGTCCTACCAAAAACAGCTCGTCCTCCTGGAGACT
GCAGTGGTTTGGCGACAATGGCAGTCAGTCACCATCTGAGTTCGGGCTGGAACCTGGCCAGTGCAAGGTGTTCGTGGAGTCGGACGCCGTCGGCCGGAAC
CTTGATCTCTCGGCGCTGGGCTCGTTCGAGGAGCTGTACACCCGCCTGTCTGACATGTTTTGCGTTGACAACGCTGAGCTGAGGAGCCATATGCTATACC
GCACCGCCGCTGGCGAGGTCAAGCACGTCGGCGATGAGCCTTTCAGTGTGTTTGTGAAATCGGCGCGGAGGATCATAATACTGACAGATGCGGGCAGCGA
CAACATAGGGAGTTAGGAACGAAGCGATCAGCCAAATAACTTAGGCACTACTCCTAGGCAGTAATAAGCCGCCTACATGCATGTACAGCCGAACGAGATG
CGGTTTCTTCTTCAATCAGGCCTAGAGAGTTTAGTCGGGAAATTAAGCTGACGATGTGTTCATTGCTTCAGTTGAGTTCTTTTAGTCATGCGAACAAAAT
GTAATCTTGATAATCAAACGCACTTGTTAATGTTGTTTCCGTTTTGGATTTCAAACCTTGAGCCGGGCCACAGAGACAAGAGCAGCAGGTGAAAAGGGTT
AAATGCCATTGCTGGCTGTTGGAACATTTACTAGTCACCCACTGAAACTTCTGAATCTGGGGTTACTTGATCAGACTCCCAGCTCCAGGCATTTCATTTG
TTCCCTTGTTTGTCTGGGCAGGCAGATACTGTGCGCTGTGTTGCGTATCCCTGTTACCTCTGATGCATTGATGCCTCCAGGGCCCTGTCCTTTGCCTGCA
TAGGGGAAATAATTGTGCTGCTTGCCCATTTCGCCTCAGAATATTGTTGCATGAAATGGACATCTCAGCTAACACAACCAGAGGAAACAGTGTCCGCAGT
AGTGGCAGTGCT
>Locus_15005_Transcript_4/11_Confidence_0.429_Length_778
AGGAGCGCCAGGCCCTCTTCAGCCGCATTGCCCCCGTGTACGACCAGCTGAACGACATGCTCAGCCTGGGGCAGCACCGGACGTGGAAGAGCATCTGCGT
CTCTTGGTCAAGGGCGAAGAGAGGGGATCGTGTTCTTGATCTCTGCTGTGGGAGCGGGGATTTATCGTTCCTGCTGTCTCAGAAGGTCGGCCTAGATGGA
GAGGTGATGGCTGTCGATTTCTCGAGGCAGCAACTGCAGACTGCTGCTGACCGTCAGGAGCAACGCTGGAAGCTGTGCTACAAGAACATCAAATGGATCG
AGGGTGATGCACTCGATTTACCTTTTGCAGACTGCTACTTCGACGCTATCACAGTTGGCTATGGATTGCGCAATGTCGTTGACAAGTCCAAAGCAATGGA
AGAAATATTTAGAGTCCTGAAACCAGGATCAAGAGCGTCTATTCTTGATTTTAACAAGAGCTCATCCCTTTTCACGGCATCATTACAGGGTTGGATGATC
GACAATATTGTGGTTCCTTTGGCTAGTGGCTATGGACTCACTGAAGAATACAAGTACTTGAAAAGCTCCATATCACAGTATCTAACAGGGGAAGAGTTGG
AGAAGTTAGCCAAAGAAGCTGGGTTTTCTGCAGCCAGGCACTATGAGCTTGGTGGAGGGCTTATGGGGAACCTAGTAGCCACTCGCTGAGTACAATCATA
GCGAGAAAAGTTGACATGTATGGTTTTTCTGTATTATATCTGATCACATCTCTATACATGTCAATTCCGTATGAAGTG
>Locus_11912_Transcript_54/63_Confidence_0.263_Length_3181
CACTGTCCAGATTCGCCTCCCTCCCCTCCCCCTAGCTCCTACTTCCCGCTCCTCCAGCTACGGCCGCGCGCTCCCCCTCCCCGCCGGCCACCTTGGGGGG
CTCCTCCCCGCCGCGCGTCCGGCCGCTCTTTTCCTGGCGCCTCCCGCCGGGCGCTCGCCCCGGATTTCTCGGCTCCCCTTTCGGTGGATGCCTTGACCGG
GGTGGGTTCTTGGCTGCGGCGGCGCGTGATCTCTGCGTACTTAAGGAGGGGTTGCTAGCGGGGTTTGTGGGAGAAGGCGAAGGAAGCCGGGGGCTGCGAG
GCGTTCATGGGTTAGATCTGGCCGTCCGGCGCCGCAGATCTGAGCGAGGTTTCGGTGGAGGGATCGGGGCGGAGAAGTCGCAGTCTCTGGCTCGTTCTGA
GCAGCTTGGGCCTCTCATGTCCAAATCGGGTATGGATGATGGGTGCGGTAACTGTCGGAAGGAGCGTCGCTTGCAGATCTGAAAGCCATCCAGCTCCCTT
GTTCGGTTGACCTGGAATGGCCGTGCTTTCTGCTGTTTGACTGTCAGTAGTTGCTGCTTAGTGTGTGTGCAAGAAATTTAGCTGGAGGTGATGTGCTTAG
GAAACTAGTCTAGTTGCTGATATCTTGGTCTCTGTTGAAGAACGCTGAAGCTGGAAGGAAGTAGGTCGCATTCCTGTGTTGTTGATATTTTGTTGTTTCT
CGAATAACCTGGAGGCTGGAAGGGATCAGATCCAGTTCCTGTGGCACCGGTAACAAGGTGCTGTTGGGGGAAGGCACCATGACCTACTTCCCTGAGGAGG
TGGTGGAGCATATATTCAGCTTCTTGCCGTCGCACAGTGACCGGAACACGGTCTCGTTAGTGTGCAAGGTGTGGTATGAGGTTGAGAGGCTGAGCCGGTG
TGCTGTCTTTGTGGGGAACTGCTACGCGGTGCGCCCAGAACGGGTGGTGCTGCGGTTCCCCAATGTGAAGGCCCTGACGCTGAAGGGGAAGCCGCACTTC
GCGGACTTCAACCTCGTGCCGCCTGATTGGGGTGGCTACGCGGGGCCATGGATTGAGGCGGCAGCGAGGGGCTGCGTGGGTCTGGAGGAGCTGCGGATGA
AGCGGATGGTGGTGTCGGATGAGAGCCTTGAGCTGCTAGCTCGGTCCTTCCCGAGATTCAAGGTCCTCGTCCTCATCAGTTGCGAGGGGTTTAGCACCGA
TGGGCTAGCAGCTATTGCGAGTCACTGCAAGCTCCTGAGGGAGTTAGATTTGCAGGAAAATGATGTGGAGGACCGTGGGCCTAGGTGGCTCTCCTGCTTC
CCTGATTCCTGCACCTCTCTTGTCTCCTTGAATTTTGCCTGCATCAAAGGGGAGGTGAATTCTGGTTCATTGGAGAGGCTTGTTGCTAGGTCTCCAAACT
TACGCAGCTTGAGGTTAAATCGATCTGTATCAGTAGATACACTCTCAAAGATATTAGCATGCACCCCTAATTTGGAGGACTTGGGGACAGGGAATTTGAC
AGATGAGTTTCAAACTGAATCCTACTTCAGGCTGGCTAATGCTTTGGAGAAATGCAAAATGTTGAAGAGTTTATCAGGATTTTGGGATGCTTCTCCTGTT
TGCGTTCCATTTATCTATCCCCTCTGTCATCAACTAACTGGACTAAACTTGAGCTATACTCCCACTCTGGATGCTTCTGATCTCACTAAAATGATCAGCC
GCTGTGTGAAACTCCAGCGTCTTTGGGTACTGGATTGCATCTCAGATAAGGGTTTGCAAGTGGTGGCCTCCAGCTGCAAAGATCTACAAGAACTGAGGGT
GTTCCCGTCAGATTTTTATGTTGCTGGTTATTCTGCCGTGACAGAAGAGGGGCTTGTTGCAATATCGTTAGGCTGTCCAAAACTTAGCTCCCTGCTGTAT
TTCTGTCACCAGATGACCAATGATGCACTAGTTACTATAGCTAAGAACTGCCCAAATTTCATAAGATTTAGACTCTGTATTCTTGAGCCAAAGAAGCCAG

ACGCCATGACAAACCAGCCGTTAGATGAAGGCTTTGGTGCAATTGTTCGTGAATGCAAGGGGCTAAGGCGACTGTCAATGTCGGGTCTTCTCACTGACAA
GGTTTTCATGTATATTGGAAAGTATGCCAAGCAACTTGAGATGCTTTCTATAGCATTTGCTGGAGATAGTGATAAGGGGATGATGCATGTTATGAATGGA
TGCAAGAATCTAAGGAAGCTGGAGATTAGAGACAGTCCATTTGGTGATGTTGCGCTCTTGGGGAATGTTGCCAAGTATGAAACAATGCGATCCCTTTGGA
TGTCATCATGCAATGTCACATTAAAGGGGTGCCAAGTCCTTGCGTCTAAGATGCCGATGCTCAATGTGGAGATCATGAATGAGCTAGATGGAAGCAGTGA
AATGGAGGAAAACCATGGAGATCAATCTAAGGTGGAGAAGCTATATGTTTACCGCACAACTGCTGGAGCGAGGGATGATGCACCAAATTTTGTTAAAATC
CTATAGTTAGCTGAGAGTAGCCTGGTATCTCCTCACTCTAAAAGATGGTCGGTGGAATCCGGCAGGCTGGGGCCGCGTCAACAGTGGTAAGTTCTTGAGG
TGTCTGTCTCTCCCTGTGCAGGGGTCAGGAAAAGCCACACCGATGTGCACCCTTATCTTGGAGCAACTGGCTGGGATCCATCATCATCCCCTGTACCACC
GCCCCCTACCCTTTTGTGTTTGGAATTCCTTTGTGTTTGCTCACTCATATCCACTGAGGCCCTTTATCATCCAGAGCATTCTCACTTGAGAGGACCGCCT
TTTTTCCGTTTATCCCACTGTAATCTGTAGTGTACCAGTATCCATTTGCATTTGACATTGGCAGTGGACTGTTTTGCCGCTGTTGTTGTAGTGTTTGTTT
CAGCCTGACGATTTGTTTCTTGCTCCTGTGAATGCAGTGCGTGGAATAATTTGCGGTTGTTATCTGCGACATTCTATTCTCTGGTTTTGAATTCTTGGTG
TTAGCCGTACCAGTTTTACGTGCTTGTTACTGAGCAAGAAGGCACCAGTACTCGTGCATTTTAAGGGCGTGCTCAGTTTCTGATGCCATTTTTCTCGCTT
ACATCATCTTTCTGTATTGCTGCCCTCAAATATGAATTCAAATTAACGGTGAAATCCAAAGCAACTTCTGAATCAATCCCG
>Locus_6904_Transcript_3/7_Confidence_0.273_Length_2563
ACAAGCAAGCTCGCAGCAGCGCCATTGCACGCTTCCGGTCGGCCGCTGCTGCGCTGCACGTCTGTCCTCCCGCCCCGTACCGCCTCGTACACATGTAAAC
TGCTGTGCTGTAGGTTTGGAGCCCCCGTACAAGCGTGTTCGAGTTTGCTGATGTTCATCTAATTATCCCCGGGGAAGGAGTGATTGTCCGCGGAGAGAAG
GAAGAGATCGAATTGAAGGTCAAGATAAGTTATTGAAACGGCAAGATGGTTAATTTTGGAAAGAAGTTGATGACAGATCAAGTAGAAGAATGGAAAGGGT
ACTATATCAATTACAAACTGATGAAGAAAATGTTAAAGCAATATGTTCAACAGACCCAATTAGGCGGGAAAGATTGTGAACAAATCCTTAAAGAGTTCTC
GAGGATTCTTGATGACCAGATTGAAAGGATTGTGCTTTTTCTGCTACAACAACAAGGCCACCTTGCCAGCAGGATTGAGGAATTGGGAGAACAACACACT
GCTCTTATGGAACATTATGATATATCACAAGTTTCCCAGCTACGTGATGCGTATAGAGAAGTTGGGAGAGATCTTATTAAGCTTCTGCGCTTTGTTGACA
TGAATGCTACTGGTATACGCAAGATACTAAAGAAATTTGATAAGCGTTTTGGCTATAAGTTTACATATTATTATGTCACCACTCGTGCAAATCATCCCTA
TTCTCAGCTTCAACAAGTATTTAAGCAAGTGGGAATTGTAGCTGTTGTAGGTGCATTATCGCGCAATCTTGAATATCTGCAACATCATCAAGGAAGCTTT
GTATCCATCTATGATCATCCATCAGTTACCTTGAAGGACCCTATAATAGACGAAGTAAACCATGCGGTACAGAAATTTACGCATGCCACCAATTTTATGA
AATTCTTGGGACAACACGCGCTTATTGTTCCAGAGGATGCACGAAGCGGGTCTGAGGATCTTGTTGATGATCAGAGCTACCATTTCATGTCTCTGCTGCT
TAACCTAGCGAACACTTTTCTTTACATGGTGAACACATATATCATTGTGCCAACTGCAGATGACTATTCAGTAAGCCTTGGGGCTGCTGCGACTGTCTGT
GGTGTAATTATCGGATCAATGGCAGTCACTCAAGTGTTCTCCTCGGTTTATTTCAGTGCATGGTCAAATAAGTCATACTTCAGACCACTTGTATTCAGTA
GCATTATGCTATTTTTTGGGAACCTGCTATACGCATTGGCATATGACCTGAATTCATTAATAGTTCTCCTGATTGGACGACTACTATGCGGGTTGGGCTC
TGCAAGAGCAGTGAACCGTCGCTATATTAGTGATTGTGTGCCTCTCAAAATCAGGCTACAAGCTTCTGCAGGATTTGTCAGTGCTAGTGCTCTTGGCATG
GCATGTGGCCCTGCTCTTGCTGGTTTCCTCCAGACAAAATTTAAGATATACTCGCTCACTTTTAATCAGAGCACATTGCCCGGATGGGTCATGTCCATTG
CTTGGCTTCTTTACTTGTTGTGGCTGTGGTTTACATTCAAAGAGCCAGAACACTTCGCTAAAACTGTCATCAATGCACAGCCGTCTGAATCAGGTCACCA
AGGAAGTGCTAATTTGGAGGAAGGTCTAGCTCAACCATTGCTTCTGGGTACAGAACAAAGACAGGGCGAGAATGCGGATGACAATGATGATAATGAAGTA
GACTCTGAAAACTCTCATGAACCAGCAACATCAATTACTTCAGCATACAGATTGCTGACACCATCTGTGAAGGTCCAGCTATTGATATACTTTATGCTCA
AGTATGCTATGGAAATTTTACTATCTGAATCAAGCGTTATCACAACATACTATTTTAGCTGGTCTACGAGTGCTGTGGCTATCTTTTTGGCAATTCTTGG
ATTAACTGTCCTTCCAGTAAATGCCATTGTTGGAAGCTACATTACAAATTTGTTTGAGGACAGGCAAATTCTGTTGGCATCTGAAGTCATAGTTCTCATT
GGTATCATCATGAGCTTTCGTTTCACCCCTCACTACTCCATTCCACAATACGTCACTTCAGCTCTCATCACATTTGTGTTTGCTGAGGTACTCGAAGGAG
TGAATCTGTCCTTGCTCTCACGAGTAATGTCGTCTAGGCTTTCGCGAGGGACCTACAACGGTGGACTCCTCTCGACAGAGGCCGGGACATTGGCCCGTGT
AATTGCAGACGCGACTATTACTGCAGCAGGTTATCTAGGCCCGGACCTCCTCCTCAACGTCACCCTGCTGCCACCTCTTGTGATCTGCGTAGTCTCCATC
GTTGCAACAGTCTGCACCTACAACACACTCTACTAAAGAAGCAGGTCTCCCCTTGTAGCTGTACAGCACTATTCCCAGTTAAAGTGATTCCTGCCCACGT
TGGGTTGTGTATTTGTACTAGCATCTATGGTTTGTAAAAATTCAAGGAAGTGTAGTATTTGTAATGTTCAATGAACTGTGTATAATTCCATCCTGTATAT
GAGCCGGCAGCTGTACCAGTGCATTTATGTTCAGTTTATTTGTTTTCAAACATCTTATGTGTT
>Locus_4749_Transcript_72/72_Confidence_0.095_Length_2701
AGGACGAGGTGGAGCACGCCGTGGACGACGCCTCGGACGAGGAGAAGGAGGAGGGCGAGATTGAGGAAGGGGAGGCCGTGGAGACCTCCTCCTCGTCGCC
TCCGCACGTGAAGCCCGAGACTATCGATTTGGACTGTGACGCGCCGGAGAAGTCGGAGTCCATGGCTGCGGAGGGCGGTGGTAACGCTGCCCCTGCCGTC
AGTGCCGCCGAGGAGGAGGAGGAGGTGGATTTTGACCAGCGCGTGGGGAGTATACTCGAGGAGCTCGAGATGGTCTCCATTGAGGAAGCTGAGAAGTCGT
TTGAGGGTGCGTGTGCACGCCTGCGCACTTGCTTTGAGAGCCTGAAGCCGCTGTTCCCGGAGAATGGGAGCCCGATGCCCATGCTTGAACCTCTTGTACA
GCAGGCATTTATCGGAATTGACACCCTCACCACTGTAGCAAATTCGTATAATTTGCCGAGGAAGGAGCAGAACAAGACCATGCTCTTGAAGATGTTGTTC
CACATAAAGAACCGATATTCAGACATGCTGACACCTGACCAACGAGATGAGCTGGACACCCGTGTGAGGCAGTTAGTTTTTGAGGAAAAAGACGATATCA
GTGACCCAAGTGTCAATTGTGGCACAAAAGCAGTGAATGTTTTGGCTCCATTTGGGCAGGTTTCATTAGGGAGGCTACCATTTGAATCAGGAGCAGCAAA
TCCATTTAGTGGCTCTAGCTTGCCGAGGTTGGAAATACCTGCAAAAAGGATTAGTCCCTTGTTGGATCTTCATGCAGATTATGATGAGAACAGCTTGCCT
TCGCCCACTCGGGATAGTGCGCCATCTTTTCCTGTGCCGAGGCCCATTGGGTTTGGAGCATTTCCATTGGCACATGAGAAACTATCTTTCCCAGAAAGAG
CTGAGCCTCCAAAGAATTCGTTGTATCCATCCTTAAATGATCCTCTGAAGGCTGTTTCCTCTTACCAGCAGAAGTACGGGCAGAAGTCTGTCTTTCCAAG
TGATGATCTACCAAGTCCAACTCCTGGTGATGAGGGTAAATCCGCAGACAGAGGTGGAGACATATTTGGTGAGGTTTCCAGCTTCTCAATTCCAAAGAAG
ACTGCACTTCCAAGTACAAGTCAGATGCCTGCTTCTTGGCCAATCACTGTTGGCAGCAGCAATGATAGTTATTCAGGTGCCCCCCCTGGTTATGCTAAAC
AAATTGAACAGTCGGCTGCAGGACCAAATCATGGACCAAAGGCTACATCTAAAAGTAGGGATCCAAGGCTCAAGTTTTTGAACCATGATTCTGCTGGTGC
TACCGATGTGAATCGGCGTTCAAATTTGTCAGAACCAAAGGAAGGGAACTTGGGTGGTGGAGTATCAATTAATAGTCGTAAACACAAGGCAGTTGATGAA
AGTAAGGTGGATGAAAACATGTTAAAAAGGCTTAGGACTGGTACCGGAAACCCAAGAGACATGCTGGTGTCAACAGGGAATAATCCTAATCTTAGAACAG
GTAATCAGCTTATTAACAACATAGGTGGTACCACAACTAGCAGTGGAACAAATACTAAAATTTTGCAACCCCCTCAAACTACTGCTCTACAAACTAGTGC
AGCTCCTGCTGTTTCTTTGCCTGATGTGTTGAAGGACATTGCTGGGAACCCAGCAATGATCATGCATTGGATTCAAATGGAACAGCAAAAGATGTCGGCA
TCAGAACCTCAGCTGAATGTGGTTGCTCCGGGTGGCATGTCTAGTGGCATGACCAGTACTGTAACTGCAGCAACGGTTTTACCACCTGGCTATACTCCAA
AGGCCACAGAAGTTCCACAAATTCCATCTGTTAGGCCACATGTTCCAGTGCAGACACCTCCTTTGAACCAACAAAATGATGCTGCTGGAATTATACGTAT
GAAGCCCCGTGATCCCCGGCGGATCCTCCACAGTAACATAGCACAGAAGAATGATACAGTGGGCTTGGAGCAAGTGAAAACTAATGGAGCTACCCAACCA
GACACTCAGGGCAGCAAAGACCATTTAACCAACCATGAACAACAGCCAGATCAACAGCAGACTAGCTCATTGCCTTCTCAACCAGCCTTGCTGTCAAACA
TTACGCGACCATCCACCATTAACTCAAAACCTGTTGATGCTGTCTCAAACTCACAGTTAGCTGCTACAGCACTTATAGCTCCAACACAGCAAGCTTTAGG
CAGCAATAGGGTAGATCCAAGACTAGTGGTTGGACAGAATGGTCCAAATGCTGAAGCAGCAACAAACGCTCCTCCTGCGCCAGCACTTGAAGCTGTGCAG
CCAGTTGATCCGTGGGGTGATGTTGACCATCTCCTTGACGGATATGATGACCAGCAGAAGGCACTCATACAAAAGGAAAGGGCAAGACGGATCTCAGAAC
AGCAAAATCTGTTTTCGGCAAGGAAACTATGCTTAGTCCTTGATTTGGATCATACACTACTCAATTCCGCAAAGTTTGGAGAAGTGGACCCTGTTCATGA
AGAGATTTTGCGGAAGAAAGAGGAACAAGACAGGTCTTTGCTAGAACGGCATCTGTATCGTTTCCAGCATATGGGAATGTGGACCAAGCTGAGACCAGGA
ATATGGAACTTCCTTAAGAAGGCGAGTAAGCTTTTTCAGTTGCATCTGTACACAATGGGGAACAAGCTGTATGCTACTGAGATGGCCAAGGTTCTCGATC
C
>Locus_4567_Transcript_53/65_Confidence_0.221_Length_1541
TGCTCTACTTCTTTCTCAGCGCACAACTTTGTGTGTTTAAATGCTCGTAGTACTTACAACAGGTGCGTTGGTTGCTACTTTTAACCGGAACAAGTAGCAA
TCATGCACCTACAGTAAACAAACTTGTCCAAATTGATTCTTCGTTGTCCAAAATCCGTGAATTGGACGCACGTCTGTAAAGGTACAAACAATAAATAACA
GACCATATAAAGCTATTTAAAAGCCTAAATCCTCAAATTCATGAAAAACCGAAACTCAAAATTCTTCGATGAATTTCATCACGATGCTCTTATCTACCGG
TCACCCTTGCTTGCGGCTTTGAATGCACCAAGGTTCCTTTTGAGAGACTTCACAGCAGACAAAATCTGCAGCTTCTCTTTGGATGCCCCATCAGGTTTCT
GATCATCGTTCTTACTGGCTGCCTCATCAGGTGGCAACCTACCTCCCGCAGACGATCCCTCCACAGGAAGTTCCCTGTCTGCATTGCTGGCATGTGCCCC
CTGCTTCCCAGCTTGCTTTCGCATGAAGGGCGCACTCCTCTTGTACTGGGGATCAGTAGGATCCAATGCGAACAAATGAGATGTGAACATTTCTGAGAAC
CTCTCATCTTTGCTAAGATCAATATCAGGAAGTTTGTCCTCGACAGATTCCTCTTTACCCTTCTTACCCTTGTTACCCTTTTTGTGTTTCAAGTTATAGC
CCTTTGCACCATTGGCAGAATCCTGGTCAGCAGCAACCAAGAGCTCCAACTCTTCTTTCGTTGCTTCTGGCTCCAAATGTTCCTCTGGTGATTTGTCCTT
TCCTTTCCCCTTTGCTTTATCTTTATTTTTCGCCTTTTGTTTCTTGATTTTGCTTGGTTTAGTTTCTTCATCAGACAGCTCCTCGGCGAAGAAATCATCG

TCGTCGTCAGGCTCATCTTTGCTGCTATAGTCATCATCATCATCATCCTTCAACCCCCTCTTCCTAGCCTTCCTTTTCTCTTGGTGCCTCTCCCAGACAG
TCTTCTCGTTGGTCTTTCTCTCTAGGATACGTTTACTGAGATCCTCAAGTTCTGTGTTGAATGTAATCTCCATATCCTGGCCATCAGTTTGTTTCCTCATC
AGATTTGTCGCCTTGCAGTAAAAGAGCCAACCTCTCCTCTTTTGTTAGCTTTTGTTTGGATCCCCCATTTGGTAGAGACTCGTCATCAGAATTGTCGACA
CCGTCATCATCTGACGCACTGTCATCACTTGCTAAATACATGTCAAGCTCATCTAACTGATCGTCAGTGAACTTTCGCCTCAAGACCTTCTTACGTTCTG
GTTCATCATCATCCCAAGTTAGCTTAACTCTGCTATGTTGCAGAGCACGAGTCTCAAAATCAGGCTCCTTGTAACTCGGAGGTGCCTCTGTAGCAATGTC
TCGAGCAGGATGTTTGAACTCCATCGAATCAGGTATAAACTGCAAATCAAATACATTTGCTGTTTTCAAGAACTCAGTACCATCAAGATTCATGTACAGG
TGATTTGCAGTTGCACTAGAATCACACACCACAACTGCATA
>Locus_1016_Transcript_989/2070_Confidence_0.002_Length_4736
GTGGAGGCGGCAAGCTAGAGATAACAACTGCATAACTTGCAAGAAAATCGTAAGTTTCTCAATTTTTCTGTTCTATACATGAGAAAACGGTGTTGATTTG
TGTGCATGCGTGCGTTTGTCAATGATCTGAATGATTTGCACATGATTTGTGTTAACATGCTGTGAATTCAGGGTATGGCAAATTTGCCAAATATCTTTTC
AGGATGCTTAAATTCACTTTGTTTAGAACTGTTTTGAGAACCGTATGGATTCAATAGACACCCAATTACTTGGTTATAATTGAGTTATTTACTTTACGAA
AGGTCGATGTGGTCATAGCATATTAAAGTTCCTTTTAATTGACCAATGACCGTATGGTTACTCTCATTCCTTCAATTCATTGCTCTCCCAGTCACAAATA
AGTGATGTTTGGGACATTGACAAGGCCTCCAAAACTCAAATGACCACCTAATTCTGCCATAGTATATTTTTAGAACCTAATAATTTGTATATCATTTCGA
TGTGTATACATCATTTAAGTTTAGAACCCAATAATTTGTATATCATTTTGATGTGTATATATCATTTAAGTTTAGAACCTAATAATTTGTATATCATTTT
TAGAACCCAAACCTCAAAGACATATCGAGAGGAATCCATGTATATCATTTTGATGTGTTCAATGTAAATATTTGTAAAGATACCAGTAGCGAAAGTTTGA
CTACACACATTCCAAATGTATAGAGGTATAATTGGAGGTTCAGCCGTTCAGAGACCCAGGTCTTGGGGTCCAGGATCGTTTTGTGCTATAATCGTTTACC
CCTTGAATTTCTGTGATTAAGGTGATAATCTTAATTTACTGTGACATAAGAATAGGCCAAATATTTTCCTGTACAAACTTAAGCTAGTGTATCTGTCGTG
TGTCTTGTCATAGTACAATCCGTTATTTCTGGTTATTTTCGGTGCGTCAACGTCTTACTGTTGTTATTCCTTTGAACACTACAAAATTTTAAGTGAGCAA
CTAGCATCCGTATATTTACATATGTAGCTGAAGAAAGGTTGATCCCCATTCTGATACTCTGGAATGCCTGCACGGATAAACCAGAATGCAATTTGTGCTC
TTCTGCTATGCATACAATTCTCAAAATAGAAAAAAATAGCACCTGAATTTTTTCCACACACCATATCTTTTTGTCAATATAAGATTTGTAGCTATCATGT
ACTCACGTTGGTCAACAAACTTGCAGAATGTGTCAAATAGTTATAATACAAATCATTTGCTGTATTCTTCAATAAAATACATTTTATTTGAAATAGATTT
ACACTACCATTATCAATCACTCATGGCATGCATAAATAGATTTACACTACCATTATCAATCACTCATGGCATGCATACCGCGTCAGTCTTGCTATGTGTT
TAAGCTCTTTACAACCAATAGTGTTTCTGGTTGCTTTGAATCGTACCGTTGAGAATAATGAACATAAGCGGTGCCATGCTGCATGCCTTCATGGATTTAG
TTGTAATCTGCAAGTTTTCAGTTAGTAGTAAACAATATGCCACTAAAGAATTAAGAACAGAATCAATAGTTCCTTTGCAGAAAAAAGCAGGGTTACCCCA
GTTTAGGAGGTGCAGTTTGCTCAACATCATTTTCTTGCGGTGGAGTGTTAAGTTCAAGATGAACAGGAATGTTGACGTTCTCGACAACTGCAAAGTTTAG
AGTTGATGAATCTCGGTTGACTTCACTTGTCACCTCTTTCTCGTAGAGCTGAGTAGAGAAATAAACCTCAGTCAATAATGGATGGGGTGTATATCCTGTG
AATTTTCATTCACGATTTATCAAATACCTTCTTGTATAAATCAATGTTTTCCTGACGGATTAGGTTGACCTTTTTGTATAGTTCCATCTTGTCTTGTTGC
ATGAGACTTCCCTGAAAATGAGCCAGTAGAGTCAGCTTCTTGAAATCCACTCAGAGACCTCTCATTGCAGAGTAATAACAGAATATTCCAGAACCTTTCG
ATTCAGTTCGTGAATTTCATCAATCAAGAGTTGGTCCTGAAAGTAAAAAACGTCATCCATCATCAGTTGTATTCTTGGATGAGGCAATCTGGTAACGAAA
AACAGAAATATGGCTAGCATCCTTACCTTTTTTGTCCGGATGCAACGTAGGCTCATTTCTAGCTGATTTTCTAGATTTTGGAGTTCCTTGACGCTCAATC
CAGAAAGATCTTGTCCCATCAACTGACTGCAGTCAATCAAACTGTATTCAGTTTACCTGATAACCTGATATTCCTCTGCCCATGTGTATTAATGTTGTGT
TGGTTGGGAGTGAAGTGCGGACAAATCAGAGACTCAAGAAACTACCGGGCAAAACAGATGATAATATCATATTTGTTTTCCATTAGTATGTACTCTTCTT
CTTTGGAAGTTAATTGGAATGCATAAAAGCTAAATAGGTACATTTTTTGACTTATAAATGGCTTGGCATAGGAAATTCATAAACTGTTACCTCCTTTTAC
ATTGATATTGTTTATGAAAACTGATGTCCTCATAAGCAAAAGATTGTCCTATGGAGTCAGTAAATCAGTATGTCTCCTTAAACTTGGGCATTGTATGCTA
TCTTGTATACTAAATTCATTCTTATCCATTGTTTTACAATTTTATACACTCTGCACAAATTAGTTGGTGGAGGTATACTTGTCACTTTACAGCCCTTAAT
TTGCATCACCAAGAGTTATGGATGATCTGTAACATGGTGTAGTTGATTCATAATTGTCTCGTCAGAACCTAGTTTTATACCTTTATAAATAAGACAACCA
GGAATTGAGTATTGCTACATGGTGAATATTGTTTCTTCTTTTGGTTCTATTCTAATGTTGTTTTGAATTTTTGTATATTCTTGAAACAAACAGTAGGAAG
GATTTCCTTCCTCCAGAGTTATCTAACTCGGAAGTTAGGCACTTGTGCTCATGCTGACAAATCCTATAGTTTGATCCTTTTACATTAGGCAAATTTTCTG
ACTTGTACATTTGTAAAATTTGCAGGGTTTTCTAGCCATACCGATGACTTTCTTGCAGGTTGTGCAGTTGTTGTCTCAAGCTTGCAGCCTCCCTTTGCCA
GAACTATTGAATATGAAAGCACAGTTAAGTAATGCCACATCCAGGATTGCCAAAATACAGCATTGGTATAAACATGTTGCATAGAAGCATGTCTGAGCTG
TCAGCTAAACAAATTGTACCTTATGAAATAACTGAATTAAATAAGATGACATGCCACTATTTCAATCAAGAGATTTACAGTGCGCTACAAAGTAACTCTC
TTTTTGAGAGGGGTGAGGGGGTGGCAACGATTATAATAGCAAATACAAAACCTTTTAAACTTGAAGGTTGAGATTCTATATTATTCAAATACTTGAATTA
AAGAACTTTATTATAGTGAACTATCAAGCTGGACACATATTGGCACGCTACACTTGCTGTATTGTCTCCTATGCTGTTGTTCACACTTTTCACACACAAT
TGCTCTGACAACTGAATTGAGCTAAATTAGCCTCGCAATACCTTGTGCTCGGGTCTAGGGACAAAACGGAACATGTCAGGATGGTGATGATCTTGCTGGA
ACATGTTTAAGCTGAAATTAGATGACTGTAGGTTTTCTCTCGTTTTCTAGAATGTTAAAGGTCATCGTCTAAAATGTTTCACTATAAACCGGAAAGGAT
TCATGCGGCACTCATTTTTCTGGAGAATGGACTAAAACATCTATTGACTGCTGATCATTTTCAGAAGATGAGAACTTGAAACAGGTACCCTTTTTCTACT
CTCTTTCGTGTTTCTTTATCAACTGTCTTTTTGTTAGTTTCATCTTGAATATATGTATTTGGGTTGAAAAGGATCACATGGTTTGGCTTCCTCCAAAAAG
AGGTTTGAGTATACGAGGCACTGCCTGGATGGATGGTTAGAAGGATTTCAAATACCGTGCTTACTGTTCATCGAGTAACAATCTGTATTATCTGTGCACA
TTTGCAGTCTATTTTCCCAAACTATGAACTGTACCGTACTTAGGTTCTGTGCATATGCAGCTCGACATTTTGGGTGTTTGGTGGTTGAATGAACCTTCAC
CCTTCAATTTATCTGTAGTTATTTGTGATTTGAAGCATCTTCTTTATATCTGTTCCACACTGCTGCCAATTTGATTATTGATATAATAAATTAAGCTTTC
AGATCGTATAGTAGAAATACACTATCTTTTAATGCAATCCTGGCTTTGCTTGGTATTTTATCTTCTCGAAATAACTTGTGTTCATTGGGATCTATGTCAG
TGCTGTGTCAGCATCAGCTTCGACTTACTGGCAAGCTTCAGGGCAGGCTAAGCAAGGCCCCTAAGATTTCATCCGGACTACAGCTAAAGTGAAAAAGGGT
TTATTCATGATAATTTCAATCTAAGACCTGTTTAGTTTCTATTAAGGATGCTTAAGTATACCAACATTTTTTTATCTTTCTTGCCAAATCTGCAAGAAA
GCTTGGTCAAGAGAATCTTGCCACAACTCCCTTTGAGATGCCAAATCTGCGGTGAAGCGGAAAATTATAAAAAACTTTTGGCATTGCTATCCTGTGTCTA
AGAAACAAACATCATCCTAATTTGGTGGAATTCGCCTGATGTGTTGTAGCAAACTAAGGGGAACTGTCTGTCAATACACCTCAAGTGCCTATATGTAGAC
CGGACAATATTTCAACGCCTCTTAATTCAGAGCAGT
>comp79373_c0_seq25
CTGTCCTGCCCAGCAGCGCACAAGCAAGCTCGCAGCAGCGCCATTGCACGCTTCCGGTCGGCCGCTGCTGCGCTGCACGTCTGTCCTCCCGCCCCGTACC
GCCTCGTACACATGTAAACTGCTGTGCTGTAGGTTTGGAGCCCCCGTACAAGCGTGTTCGAGTTTGCTGATGTTCATCTAATTATCCCCGGGGAAGGAGT
GATTGTCCGCGGAGAGAAGGAAGAGATCGAATTGAAGGTCAAGATAAGTTATTGAAACGGCAAGATGGTTAATTTTGGAAAGAAGTTGATGACAGATCAA
GTAGAAGAATGGAAAGGGTACTATATCAATTACAAACTGATGAAGAAAATGTTAAAGCAATATGTTCAACAGACCCAATTAGGCGGGAAAGATTGTGAAC
AAATCCTTAAAGAGTTCTCGAGGATTCTTGATGACCAGATTGAAAGGATTGTGCTTTTTCTGCTACAACAACAAGGCCACCTTGCCAGCAGGATTGAGGA
ATTGGGAGAACAACGCACTGCTCTTATGGAACATTATGATATATCACAAGTTTCCCAGCTACGTGATGCGTATAGAGAAGTTGGGAGAGATCTTATTAAG
CTTCTGCGCTTTGTTGACATGAATGCTACTGGTATACGCAAGATACTAAAGAAATTTGATAAGCGTTTTGGCTATAAGTTTACATATTATTATGTCACCA
CTCGTGCAAATCATCCCTATTCTCAGCTTCAACAAGTATTTAAGCAAGTGGGAATTGTAGCTGTTGTAGGTGCATTATCGCGCAATCTTGAATATCTGCA
ACATCATCAAGGAAGCTTTGTATCCATCTATGATCATCCATCAGTTACCTTGAAGGACCCTATAATAGACGAAGTAAACCATGCGGTACAGAAATTTACG
CATGCCACCAATTTTATGAAATTCTTGGGACAACACGCGCTTATTGTTCCAGAGGATGCACGAAGCGGGTCTGAGGATCTTGTTGATGATCAGAGCTACC
ATTTCATGTCTCTGCTGCTTAACCTAGCGAACACTTTTCTTTACATGGTGAACACATATATCATTGTGCCAACTGCAGATGACTATTCAGTAAGCCTTGG
GGCTGCTGCGACTGTCTGTGGTGTAATTATCGGATCAATGGCAGTCACTCAAGTGTTCTCCTCGGTTTATTTCAGTGCATGGTCAAATAAGTCATACTTC
AGACCACTTGTATTCAGTAGCATTATGCTATTTTTTGGGAACCTGCTATACGCATTGGCATATGACCTGAATTCATTAATAGTTCTCCTGATTGGACGAC
TACTATGCGGGTTGGGCTCTGCAAGAGCAGTGAACCGTCGCTATATTAGTGATTGTGTGCCTCTCAAAATCAGGCTACAAGCTTCTGCAGGATTTGTCAG
TGCTAGTGCTCTTGGCATGGCATGTGGCCCTGCTCTTGCTGGTTTCCTCCAGACAAAATTTAAGATATACTCGCTCACTTTTAATCAGAGCACATTGCCC
GGATGGGTCATGTCCATTGCTTGGCTTCTTTACTTGTTGTGGCTGTGGTTTACATTCAAAGAGCCAGAACACTTCGCTAAAACTGTCATCAATGCACAGC
CGTCTGAATCAGGTCACCAAGGAAGTGCTAATTTGGAGGAAGGTCTAGCTCAACCATTGCTTCTGGGTACAGAACAAAGACAGGGCGAGAATGCGGATGA
CAATGATGATAATGAAGTAGACTCTGAAAACTCTCATGAACCAGCAACATCAATTACTTCAGCATACAGATTGCTGACACCATCTGTGAAGGTTTCCTAT
GCTTCCCTTATCGAGTACTCTTTTGTCTTGTGTTCCTCTCAAGCATGCCTAAAACAAAGTTCGCAGTCTAAATAGGGTTCTTTTGTAAAGAAAGTAAAGT
AAATGAACCGTGTAACATTAGGAAATGTATCTTAGTGTTAGTGATAATGATACTAGAAGGTACATCTGCAAGACACAAGCATGCACTGTACTTACACGCT
ATATCAAGTGAATTGAAACCAGATGAAAAACCTTCTTTTCATCTTTGGACCTTTGCATTTTCAATTGCCCTTGACCTCTTTCAGTGTATTTATAGGAGCT

ACCATCTTCTGTATCTTCCATTTCATATGTTAATAAGAGTGGCTATTTCTCAGGTCCAGCTATTGATATACTTTATGCTCAAGTATGCTATGGAAATTTT
ACTATCTGAATCAAGCGTTATCACAACATACTATTTTAGCTGGTCTACGAGTGCTGTGGCTATCTTTTTGGCAATTCTTGGATTAACTGTCCTTCCAGTA
AATGCCATTGTTGGAAGCTACATTACAAATTTGTTTGAGGACAGGCAAATTCTGTTGGCATCTGAAGTCATAGTTCTCATTGGTATCATCATGAGCTTTC
GTTTCACCCCTCACTACTCCATTCCACAATACGTCACTTCAGCTCTCATCACATTTGTGTTTGCTGAGGTACTCGAAGGTATGTATTTTGTACATGTGTG
CAACCATCTCAAAGCATCATGCACAGTAAGACATGAAAACAATCATACGCTATTGCTATAACCATCATACAATTTCTGATGACGAGCAATGCAGGAGTGA
ATCTGTCCTTGCTCTCACGAGTAATGTCGTCTAGGCTTTCGCGAGGGACCTACAACGGTGGACTCCTCTCGACAGAGGCCGGGACATTGGCCCGTGTAAT
TGCAGACGCGACTATTACTGCAGCAGGTTATCTAGGCCCGGACCTCCTCCTCAACGTCACCCTGCTGCCACCTCTTGTGATCTGCGTAGTCTCCATCGTT
GCAACACTCTACACTTACAACACTCTGTACTGAAGAAGCAGGCCTCCCTTTGTAGCTGTATAGCACCATTCCCAGTAAAAATGATTCCGGCCCACATTGG
GTTGTGTACTTGTACCAGCATTTATGGTTTGTAAAAAGTCAATGAAATGCATTTGTAGTGTTCAATGAATTGTGTAATTTTTCATCTTGTAATGATTTAT
GTGCCACGATTGCCAGTAGTTGTATTAGTGCATTGTGGTCAATTTATTTGTTT
>Locus_1826_Transcript_74/132_Confidence_0.098_Length_2091
AGGACTACATGAATTGCAAAATTTGTCATGTGCTGGTCAGCCTTTGGGTGCTGTAAATTTATCCAGTCAAGTAAGCAATGAAACTCTATTGTCAGAAAGT
TCTGACGGATTGCTAGAGGCCATTACGAATCCGGTTAAGATGATGCACATGAATGATGATACTTGTAAGAGAGTCAGCAGCACTCTGCAATCATCTTTTC
CACCAATAGAACATGCAGCAGAAGGTTTAAAAAGTTCAGTTGACAGGAACAATGAACTCGTAAAGGAGTTTGTCATTGGTTCCAACTCTGCTCTATCTAA
ACAATCTGAGGCAGACGAGGGAAATTCTAGTCCTCATCTTGTTACTTCTGTGTCAACCAAAAGCAGCAAGATTATTCAATCTCCCAAAAGAAAGCTTGCT
CATGCTTCTGGTGTTCCTGAAGAAACAAAGAATGCTTGGGCCGATAGTACAAACATCTTCAGTGGTGATGAATCAAAACTTGGAGCGTTGGAGCACCATC
AAGATTCTCTCGACAACCTAAAAAGTGATGCCATGGAAGAAAAGACAACCAGGGAGAAAATATCAGCAGTTTCAGGAAATATTGATCAAATGGTGGAAAG
TGGTCATGACGAAAATGCTACATGTGCTACTGGTACATCAAGAGATAAGTTTGACTCTTCAGACAATACTGCACCTGTCAAATTTTCAGCTGACACTTTG
GATACTTCAGAGGACCAAAGCATTCCCTCAGTAAATCATGAGGGGTCATTCAAGGGAGGTGGCATGCCTGCTTCAGAAGAGGAGCCTGAAAACACGCGTT
TGGTCCTACCAGCTTCTGGGCCTCAAGAGAAAATGTCAGCAGCATCAATCAATTCAAGAAGTGATATCGTTTCTACCGGTGTTACTGACACTTCAAATAA
GTCCGAGGATAAAAATGATTGTTCAGGTGTTTCTCCTGATGATTCTTCTGCTTCTTTGCCTGATGAGAAAGATTTGAAAATGTCCACAGTGGATTATGAG
GGATCATTCAAGGAAGGTGCTAAACCCGCTTCAGAAGATGAAGACCACAACGTTATTTCTTCTGGCTCTGAACCAGGTAGAGAATTATCAGCAGTTTCAG
GGAACTCAGGCATAGATGCTATTTGCAGTGGTACTGTTTCTGCTGCACAAGAGGCGGAACACAAGGAGCAGGCTAATTCTTTGGGTGGTTTGCCCACAGG
AGAAACTCAGGATAAATCGGGCAACCATCCAGATGCTTATTCACAGAAATGCCAGACTGACGGATCCTCGGTTCAATCTGAACATCGTGTAGATCCAGCC
ACACCATCTGCATTAGGCATCTCAACTGACAAGGCTGTTGAAAAGATCGTAGAAACTCCACTGAATGCAAAGGATGATTTGAATGCACATGTGCAAGATA
CAGTTTCAAGTCATTGTACAGATCATAGTCCTGGTACTGTTCCATCCCAAGGCAAGCTAGGCTCAAGCTTATTAGAACCTGGGAACAGTGACGGAATCTG
CTCTGATGCTACACATGATTCCTCTCCAGTGATTAGTTGCACTGAGCCCTCTCCCCAAGGTGGACATGGCAGCAACACTATGGTTCATCAGACACTAGAT
GAACAATCTGAGGATCCAAAAGATCGTGAAGCCTCAGCTGACGCTACGCAGAGCTCAAAACAGTGTTCTACTGGACATGTAAATCCTACTCCTGGTTCTG
AGCAGACTAATTCAGCGGGAGACGATAAAAGTTTCTCATTTGAAGTGGGAGCTCAACCACATGTATCTGAGAAAGCTCATAGCCCTGCTTGGAGCCCTTT
CCCCAGATACAGAGCTTCTCAGAGTACTGAGGTAACCACAGAAAAACCTCAGGCTGGAAGTTCATTGGAGAATAGTGGTGATGATAGTAAAAGAACATCT
ATTGTGAAGGCTGGTAAAGAACAGCTGTCAGAAAGGAAAATGAATGGAAGTGCCGGGGGGGCCTCAGACAACTCTAATATTTGCAATAGCACCAAAAGTT
CACCGCTGGAGCAGTCAGAACAGCATCCAACCCCCAAGATCTCTGATGTGGCGAATCTACCTTTCACAGATGCGCAGCATTTGCAGCTACG
>comp79901_c0_seq34
GACGCGGCGCGGGCCGAGGTCCCGGAGCTCGCAGTACAGGGGCGTCACCTTCTACAGGAGGACGGCCGGTGGGAGTCGCACATCTGGGATTGTGGGAAG
CAAGTCTACTTAGGTGAGTAACTGAGTTGAGTGAGTTCTATACGTTCCAGCTCGAGCTTCCATTAACTGCTGATCAGTTTCCGTCCGTTTGATCTGGTTT
ACGATGTGCTGATGCTGTTCTATTCAGGTGGTTTCGACACTGCTCACGCGGCTGCGAGGGCTTATGATCGTGCAGCGATCAAGTTCCGAGGGCTCGACGC
GGACATCAACTTCACTTTGAGCGACTATGAGGATGACTTGAAGCAGATGAGCAATTGGACAAAGGAAGAGTTCGTGCACATACTCCGCCGCCAGAGCACG
GGGTTCGCGAGGGGGAGCTCCAAGTACCGCGGCGTGACGCTGCACAAGTGCGGCCGCTGGGAGGCGAGGATGGGTCAACTTCTTGGCAAGAAGTACATCT
ATCTTGGGCTCTTTGACAGCGAAGTTGAAGCTGCAAGAGCATATGACAGGGCAGCCCTTCGCTTCAATGGGGGGGAAGCTGTTACTAATTTTGGGCCTAG
CTCCTACAATGGAGGAGATGCTCTACCCGACACCGAAAATGAGGCAACTGTTGATGGTGACGCGGTTGATTTGGATTTGCGGATTTCACAACCTAATGTG
CACGACACTAAAAGGGACAATACCTTAGCTGGAGGCCAGCTAACATGCGACTCCCTTGAATCTTCAAACACGATGGCCTCTCAGCCAATGAGCTCATCGT
ACCCGTGGCCTGTGTATCACCAAAGCACGACAGCAGTACCACCTCACCATCAACGCTTGTACTCATCTGCTTGTCCTGGCTTCTTTTCGAACCTCCAGGT
ATTACCAAGCTAAAGCCTATTTTTTATGTGCAAATTGCTACTCCTACGTGATCAATTAGTTCAAACTGTTATTCGAATCCAGGATTGTTTACTGAATATG
TCCTGTCTTCAGAAAAAATAACACAAAAAGGAACATAATATGTCCTGTCTAATTGTGGACTGATACTAGCCAATTAAACAGTTTCCCATATCCATTTCCT
GTGTTTATTGTTTTGATTTGCTGGCATGGTTAAACTATAGTAGCTTGATTCGATAAAAACACTAAGCAGGCTTTGATATTTTGAATGAAATGTCGTTCTT
GGAAATACAGCCTTTTCCTTGGTATATCAAGGACAGTTGTCCATCTGCACTCTGCACCCTCAGCTTGTGTAACTGTTTGCCTTGTGAACTGATTGATGTG
TCAATCAGGCAGTTCTGCCAGTTTGCATCTTTTGAAATAGTGCAGTCTTCCAGTCTAACAGCTCCATGACATAACTTGGGATGCATGCTGCAGCTTTCTT
ACTGAGGATGCTCTAGATTTACAGAAATTCTCGCAGTTAGCAGAGGGCTATCAAATCTGATTTGCTTAGCTTCCTGCAGACAATAAGATAAGAACTCTAG
AATCCTGAAGAAATCTTACTTGTACATGTTTACCAAAGTATCCTTACTCCTTAGTTGAGCCAATTTTGGTGAAGAACCGATAGCTCATTACTCTGTTTCC
TGGGAATGATGTGGGCCTAAACCATTCTGTGGTCAAAGTTTAACAAAGCCCATGCTTGTGCTGTTGCAACTGCAGGAAAGGCCAATGGAGCGAAGGCCTG
AGTTGGGTACCCAGTCATTCCCCACCTGGGGATGGCAAATGCAGGGCTCCCCTCACATGCCGTTGCATCACTCTGCAGCATCATCAGGATTCTCTACCGC
CGTCGGCGCAAACGCCGGCGTCTCGTTGCCTTCTCACCCGCCGGTGCCGTTCCCGGACCACCAGTTCTACTTCCCACCGACGGCATGAGCTTGTTGGTCT
GGTTCAACTCTTGGGACGGCAGCTGATCTTATGTGTGAACATTTCGCAGCTTGCCGGTGACCGTTGTTAATTAATCGGGGAGAGAGAGCCAGAAGACCCA
GTATGGCTATCTCTCTGTGGCCCGTGCCTTGTTATGATCTTAGTTATTGCTACTACACCGTGGATTCATCATGGTTTATGAGATTCTTTACTCAAGTTTC
AGCTTAAATGTCCAAATACATGAATGCATCATACACAGGATCCGTCATTTTCTAAAAAAATACAGGATCTGTGTGAAGTGT
>comp78295_c1_seq6
ACAACTGCACAACTTGCAAGAAAGCCACAAGCAACTAATGGGTGAGAAGCTTTCTGGCCTAGGTGTGAGAGACCTCCAGAGTTTAGAGAATCGTCTTGAA
ATGAGCCTACGTAGTATCAGAACGAGGAAGGTCATATTCATTTTGTTTCCACGTTTTGTTTTTCTATTCACTCATCTTAATATGCATAAACATCCATCAA
ACAGGACAATCTTTTGAAAAGTGAAATTGAAGAGTTGCACAGGAAGGGTAGCCTAATTCACCAGGAAAACTTGGAACTCTGTAGAAGAGTAAATACCATG
TCACAACAAAATATGGATCTACAAAGAAAGGCGAGTGAACCAAGAGGTGTTGCCGATGCAAATAAAAGCTCTAGCACTCCCTACAGATCTTGTAGTGCAC
AAGATGCAGATATCACGGTTAATCTTGAATTGAGCCAATCACAGCAAAAAGAGGGGGAGCAGTGCCAAAGAGGGGCTCCAGAACTGGGGCTTCAGCTGCA
CAAAGAAGATGCACTACAAGCTGGAGAGTATCATGATGTTCCACCAATTCTAACATGCAATGCGGAGCACTTAAACTACAATTAGTATTTTGTGCAACAT
GTTAGATGAGTGAACAAAATGGTTTATGTACAGACTACAGATATGACCATCTATCTTATCTGTGGCTCTACTGTACTGAAATTGCAAGAAATGGTTATTG
TAATGACTAATATGATACATTGACTGTCATACCGTTGACCTTGAAGATAGTTCTATAACGGAAAGGTCCAATTTGAAACAAGTGTTACGGATGAGCTAGT
AGGATTTTTTTCACAAACAATTAGATAGTCCACGTTAGTGGTAGCTGCTGGGTATGACATTTGACCCAGCCTGCCGACCCTCCCTTTCCCATGGGAGTTC
AATTAACATTAGGAATGAATATCGATATTATGGTTGATTATTTGAATCGTATGTTTTCCACCCTTAGATGTAAGGCCCTATTTTACTGTTTTAATGGATT
AACAAGGTGAGCTTTCGAGATGGTAGCTCATTGATTCGCATGCATTTTAGTGGCTGTTAAGAGGTACTTAGTGCATCCATGCTTGAAGGTGTTGTTAGTT
CGACAAACCATTTTCTATCCTACTGGATTACTATCTTTTGGTCAGTATGACAAGCAAGAATTTAATCTGCCAATTAAGAATTTAATGTGTTCTTTTGACA
TGAATGTCAACAGCATTTAAGTAACTCAACTTTTAATACAAAGTACTGGAATGTTTTCTCACCGAAAGTAAAGATGTGGTTCCTTGATAGCAAACAATGT
TTATGCCAATATCGCATTAAGGTGGTTGTATCCTTTAGAATGATCTCCTCATTCGTGCTTAATTTGATGCTATTGTCTTTTTACGGATGCCTAGATGTGG
TTTTGACCATTACTAAGTACTTTAATATATATTTAACAAAAATAATGACATAGCTGTTGTATGGCCAATCTAGATTTTGTTTTCGAAACATATTTGCAGT
CAAACTATAGGAAGTAAGAAACTTCAATGTGGTTCTCGAAAACCATGCTTAAAGTTTAGAAAGTTTGACTACATAGAGTATATACTACATAGTCTTAAAG
CATCAAATGAAGAGGAATGGAGGGAGTAGCAGACAATCATCATTCAATATCTATTTCTTGGTTGCCTTCAAATTTGGGCTTACTAAAATCGATTTAAAAT
TCTCGTGTTCCAGTTGAAGCTCACTTCAAGTAAATCAGGAATTACCATATTTTTCATTGCTCATAATTTGGAAACATGGCATGATATTTGATAGAAAATG
TACAAATCCTCAGCTGCTCAGAAAGTTACAAAGGGAGGAACGCCCATGAAAGAGAATTCATGGCTGCAACAACGAACATAAACACAGCCTATTCTGTCCA
GCAGTCATGGTAATACAAGCTAAAAAGGACCGCTCTGGAACAATAGCTTGAAAAGCAATATTAATACAATTTCAGATGAACTATGTGCGTCGATGTCTTA
CTTGTGACTTCCTTGCAAGTAATGCGGTTCCTGCCTCATGCTTGGTGCCGCCGCATGCAATAAACCGATAACTAGTGAAGCAAGAGGATAACAGATGTCA
GAAAACTGAGCAAGCCATGTTAACTGTTTGAGAATTGTATACGGCTATGAGAAGTATATTTTTTTAGGGGGTGTTTCGATCCCTTTAGCTAATTTTTTAG

CTCTATATTTGGATGTTAAATAGGAGGACTGAACATGGGCTAATTATAGACTAATTGCAATTAGTGATTAGAGCCCATAAGCTCTAATTAGCCTATGATT
AGCCCATGTTTAGCCCATTAGCACATGAGCTAATCATAAGCTAATTAGGGTTAATTAGCTCTAATCATCAATTAGCTAAAGCTAACTATAATTAGTTCAC
AATTAGTCCATATTTAGTCCCCCTAATTGGGGTCCAAACATGGGGCTAAAAATTAG
>comp65473_c0_seq1
GCCAAGAAGAGCCGCCGCGGCCCGCGCTCCCGCAGCTCGCAGTACCGCGGCGTCACCTTCTACCGCCGCACCGGCCGCTGGGAGTCCCATATTTGGGATT
GCGGCAAGCAAGTGTACTTAGGTGGATTTGACACTGCTCATACTGCTGCAAGGGCGTACGATCGAGCTGCCATCAAGTTCCGTGGCTTCGACGCCGACAT
AAACTTCAATCTCAGTGACTATGAGGATGACATGAAGCAGATGAAGAGCCTGTCCAAGGAGGAGTTCGTGCACGTCCTGCGACGGCAGAGCACCGGGTTC
TCGCGTGGCAGCTCCAAGTACAGAGGCGTCACCCTGCACAAGTGTGGCCGGTGGGAGGCTCGCATGGGTCAGTTCCTTGGCAAGAAGTAAGAACACTTCA
CAACCTGAATTACCTAATACTAGACGTGTTATTTCTATACTGCAGATCCTTGTGATTTTTCCTATCTCAGTCTCATTCTAGAGTTATGAACCTAATACAT
GAACTCCCATGATTTAATAGTCTAGAAATAGTAGGTTAGATTGATGTGGCATAATGGTTGTTTGAACAAAAGAAATTAAAGAGAAAAAAGGCAGTGGATA
GGGAATTCCTCACCGGTGGGAACGCCATGCTTGGGTGCAGGTACATATATCTTGGGCTATTCGACAGCGAAGTAGAGGCTGCAAGGTTGCTGATCTTGGA
TTCTTCTATTGATGCAAAAAAAATTTTGCTTTACCTTCTCTGTTGATTTCCAAAGGGTTGAGTACCGACTCGATTCTCTTCTTTGTTGTTTTTCTTCTTT
CTGTTTCAAAATTCAGGGCTTATGATAAGGCCGCGATCAAATGCAATGGTAGAGAAGCCATGACGAACTTCGAGCCTAGCACGTATGATGGGGAGCTGCT
GACTGAAGTTGGTACTGAAGGTGCTGAAGTCGATCTGAACTTGAGCATATCTCAACCAGCTTCTCAAAGCCCGAAAAGGGACAAGAATTCCCTTGGTCTG
CAACTCCACTATGGATCGTTTGAAGGCTCGGAATTGAAAAGAACAAAGATTGATGCTCCTTCTGAACTGGCGGGCCGCCCTCATCGGTTCCCTCTTCTGA
CCGAGCATCCACCAATCTGGACTGCCCAGTCTCACCCCTTCTATTCAAATAATGAGAATGCATCAAGAGATCTTAACAGGAGGCCAGATCAGGGGGGTGT
TCCCAGCTGGGCGTGGAAGGTGACCGCCCCTCCTCCCACGCAGCCATTGCCGCTGTTCTCGTCGGCGTCATCATCCGCTGCAGCATCATCAGGATTCTCC
AATACCGCCACGACAGCTGCCCACACCGCCCCATCGGCCTCCCTCCGGTTCGACCCGCCGCCGCCGCCGTCCAGCCGCCACCGCTGAACGTTAAGAAGCC
ACGCTGTAAATTTGCCAGGAAGCCGACATTTTTCCTCTCTCGGCGTTGCAACTTTTTAGGTTTTGCGCCGGGGTGGTTTCTTGTAGTGGAGTGGATTCA
TAATTAACTGTATTTGCATGCTGCCCATGTGAAATGCTTCTCTCTCTTTTTACGCTCTCTGCACCGGATGTTCTGGGAGTTCTTGTTGCCGGGAATTAAC
TAAATTCACCGTCTGAGATTGATCTATACATTGTTGTAGAGAATCGAATCATTGTTGAAATTAGTTACAGAATTATAGATATCATAAGGCACCCTCTACT
TGTGGACAAAGTTCAGGAAGATTATTCCGGTTCTTTTCTTGGCTGTCAATTCGGCTCTTGTCCTTGGTGTGGAGCCACTTGCCTACTGTCTTGTTCTGTT
GCTGTTTGTTTTTGGCTTCTTGCACAGTTACAAATTTGTTGGCTCGAATGGATGGTGAGACATT
>Locus_3441_Transcript_134/417_Confidence_0.052_Length_2117
CGCATATGGCTGCGCTAGAACTACTGCTCTGAATTAAGAGGCGTTGAAATATTGTCCGGTCTACATATAGGCACTTGAGGTGTATTGACAGACAGTTCCC
CTTAGTTTGCTACAACACATCAGGCGAATTCCACCAAATTAGGATGATGTTTGTTTCTTAGACACAGGATAGCAATGCCAAAAGTTTTTTATAATTTTCC
GCTTCACCGCAGATTTGGCATCTCAAAGGGAGTTGTGGCAAGATTCTCTTGACCAAGCTTTCTTGCAGATTTGGCAAGAAAGATAAAAAAAATGTTGGTA
TACTTAAGCATCCTTAATAGAAACTAAACAGGTCTTAGATTGAAATTATCATGAATAAACCCTTTTTCACTTTAGCTGTAGTCCGGATGAAATCTTAGGG
GCCTTGCTTAGCCTGCCCTGAAGCTTGCCAGTAAGTCGAAGCTGATGCTGACACAGCACTGTTTCAAGTTCTCATCTTCTGAAAATGATCAGCAGTCAAT
AGATGTTTTAGTCCATTCTCCAGAAAAATGAGTGCCGCATGAATCCTTTCCGGTTTATAGTGAAACATTTTAGACGATGACCTTTAACATTCTAGAAAAC
GAAGAGAAAACCTACAGTCATCTAATTTCAGCTTAAACATGTTCCAGCAAGATCATCACCATCCTGACATGTTCCGTTTTGTCCCTAGACCCGAGCACAA
GGTATTGCGAGGCTAATTTAGCTCAATTCAGTTGTCAGAGCAATTGTGTGTGAAAAGTGTGAACAACAGCATAGGAGACAATACAGCAAGTGTAGCGTGC
CAATATGTGTCCAGCTTGATAGTTCACTATAATAAAGTTCTTTAATTCAAGTATTTGAATAATATAGAATCTCAACCTTCAAGTTTAAAAGGTTTTGTAT
TTGCTATTATAATCGTTGCCACCCCCTCACCCCTCTCAAAAAGAGAGTTACTTTGTAGCGCACTGTAAATCTCTTGATTGAAATAGTGGCATGTCATCTT
ATTTAATTCAGTTATTTCATAAGGTACAATTTGTTTAGCTGACAGCTCAGACATGCTTCTATGCAACATGTTTATACCAATGCTGTATTTTGGCAATCCT
GGATGTGGCATTACTTAACTGTGCTTTCATATTCAATAGTTCTGGCAAAGGGAGGCTGCAAGCTTGAGACAACAACTGCACAACCTGCAAGAAAGTCATC
GGTATGGCTAGAAAACCCTGCAAATTTTACAAATGTACAAGTCAGAAAATTTGCCTAATGTAAAAGGATCAAACTACAGGATTTGTCAGCATGAGCACAA
GTGCCTAACTTCCGAGTTAGATAACTCTGGAGGAAGGAAATCCTTCCTACTGTTTGTTTCAAGAATATACAAAAATTCAAAACAACATTAGAATAGAACC
AAAAGAAGAAACAATATTCACCATGTAGCAATACTCAATTCCTGGTTGTCTTATTTATAAAGGTATAAAACTAGGTTCTGACGAGACAATTATGAATCAA
CTACACCATGTTACAGATCATCCATAACTCTTGGTGATGCAAATTAAGGGCTGTAAAGTGACAAGTATACCTCCACCAACTAATTTGTGCAGAGTGTATA
AAATTGTAAAACAATGGATAAGAATGAATTTAGTATACAAGATAGCATACAATGCCCAAGTTTAAGGAGACATACTGATTTACTGACTCCATAGGACAAT
CTTTTGCTTATGAGGACATCAGTTTTCATAAACAATATCAATGTAAAAGGAGGTAACAGTTTATGAATTTCCTATGCCAAGCCATTTATAAGTCAAAAAA
TGTACCTATTTAGCTTTTATGCATTCCAATTAACTTCCAAAGAAGAAGAGTACATACTAATGGAAAACAAATATGATATTATCATCTGTTTTGCCCGGTA
GTTTCTTGAGTCTCTGATTTGTCCGCACTTCACTCCCAACCAACACAACATTAATACACATGGGCAGAGGAATATCAGGTTATCAGGTAAACTGAATACA
GTTTGATTGACTGCAGGCAGTTGATGGGACAAGATCTTTCTGGATTGGGCGTCAAGGAACTCCAAAATCTAGAAAATCAGCTAGAAATGAGCCTACATTG
CATCCGGACAAAAAAGG
>Locus_32123_Transcript_6/16_Confidence_0.538_Length_1066
ATCTCTCTCTCTCTCTCTCTCTCTCTCTCTCCAGCTAGCCGCCGTTTGACCCTGCAGATTGCTTCCTTCTGCACGGCGCGATGGTACGGGGAAAGACCAT
ATTGAAGAAGATCGAGAACGAATCAAGCCGGCTTGTGACCTTCTCCAAGAGGAGGGGCGGGTTGTTCAAGAAGGCAAAGGAACTGGCAATCCTATGCGAC
GCTCAGGTCGCTGTTCTCATCTTCTCCAGCACCGGCCAACTCTACGACTTCTCCAGCTGCAGCATGAAATCAATAATCGAAAGATACCGCCAAGTAAAAG
AAGGTCAGCAAAATGTAACGGCAAGTAGGGAGGCCAAGTACTGGCAAGGGGAGGCGGAAAGCTTGAGGCAGCAACTACACAACTTGGAAGAGAATCATAG
GCACTTGCTGGGACAACATCTTTCTGGCTTGTCAATGAGAGACCTGCAGACTTTAGAGAACCAGCTGGAAACGAGCCTAACTAATATTCGGCTAGCAAAG
GAACGAATTATGATTGATCTGATTCAAGAATTGAACAAGAAGTCAAGCCTCATAGACATCGAAAACAAGGAGCTACACCACAAAATCTGTGCCATTAGTC
AAGAGAATGCCAATTTGAAGAAAGAGGTACATGGACAGCCGGATGCCATTGAAGAAAATACTGGTTCTGTTACTCAGTATAGTACTAACACTCCTGAAGA
CCTAAATGCGACGTGTCTCGAGCTACGCCAACCACATCATGTAGAAATAGAGCAACCAGATGTGCCTACATTGGAACTGCGTCTCCACTGATTGCAAGAA
TCAGCAGCAGAATCATCGGAAATGAGGAGGCCAGCCATTCAACGCAAATTCATAAGACTTTGTCTCCACTGATTAAATACAAAGAGGAGTTCCTGTGCAA
ATTACTATTATATGAATGTAACAGATGTAATTTATTTTTTATGTATCTGCAGTACACATATGCCCACCATAAACTTATGTTCAAAAGGCATATATACCTA
TTGCCACCAAGCTACGACGATTATCATTGTTATGCTACTTGGATCAGAGATGAAGGTTAAAACTTG
>comp72072_c0_seq1
CCGCTTCGCCCCCCCCACCGCATCCACCGCTGCAGGAGTTAGGGTTCCGGCGCCGCGCGCCGGGGTATGAGCAGCACCCGCCGCCCCGCAGGATCTAGGG
TTCCGACAGCTCCGCTCACTAATGGAGGGTGGTGGTAGAAGAGCAGGCGAATCCCCCTCGGCATACACCAATGGATCATGATGATACTGATTTTCAGAGC
CAAAACTTTCAACTAGCTGGTGAAGACAATAGCAAATTCCCATCGGGTTTGCGGCCATTTGCTCTGCCGAAGCTTGATATTGATGACCAATTACAAGGCC
ACCTTCGATATGATAATTTGATAGATTTGGAAGTATTTTTCAGCGTACAAGGGCACGAGAGTAGTTGGGTTGAGGTTTTGTCCACTGGAAGTAGTGTTGT
TGATTTTAGTTCCAGTGCTGCTGAATCTTGCTCCATATCTAAGACTAATAATGTCTGGTCAGAGGCAACATCCACCGAATCCGTGGAAATGTTATTGAAA
TCAGTGGGAGCAAATGAGATGACTGGTAACATGGAAGGTGATGCACATCTTCAGTTAAGTGGTATGGACAGTCAAATGGATGCATCCAACGTGCAGCCTA
AATCCAGTAACTCTCCAACAGATAGCACTGTAGTGCCAACTGAGAATGATCAGTCACAGAGGACTCATTCTAGAATGATGGATGATCCTGTTCGTTCTAC
GAACACTAATTCCAGAATGTCTGACAATCCTTCAAGCACTCAACCACAGCTTGAACATTTTGCATCTTTCTTGATTGTCGAGAAAACTGAAAAGGCAGCA
GGGTCAATTTTATCAGATAGGAACTCCAATTTTGTGTTGGAGTCTGTTGCTGAGAGGTGTATTGTAAGTGAGAAGTTGTCTTCTTCCACATCAGAAAGCT
GCCCAGATGTTGGTGACCATTTCAAGGCTGTCCATGATGACCATTCTTTGGACAAACTGAGAGTACCCTCAGCTGAAGTAGATTCTAGGAAGATGAATAA
TGAACCTTTCCCAGAATTAGCTCCACTTCAGAACATTTATGACTCGTATCACTTTGAGCAAGATAATCAAGGATCTGAGGTTGGTATTACTCCTCAGGAT
TCAAAGATTAGTCATATCAGTGAAAATAAAGTCGGAGGAGGACTACATGAGTTCCAAAATGTGTCATGTGCTGCTCAGCCTTTGGGTGCTGTAAATTTAT
CCAGTCAAGTAAGCAACGAAACTCTGTTGTCAGAAAGTTCTGACGGATTGCTAGAGGCCATTACGAATCCGGTTAAGATGATGCACATGAATGATGATAC
TTGTAAGAGAGCCAGTGGCACTCTGCAACCATCTTTTCCACCAATACAACATGCAGCAGAAGGCTTGAAAAGTTCAGTTGGCATGAACAATGAACTTGTA
AAGGAGTTTGTTATTGGTTCCAACTCTGCTCTATCTAACCAATCTGAGGCAGACACGAGAAATTCTAGTCCTCATCTTGTTACTTCTGTGTCAACCAAAA
GCAGCAAGATTATTCAATCTCCCAAAAGAAAGCTTGCTCATGCTTCTGGTGTTCCTGAAGAAACAAAGAATGCTTGGGCCGATAGTACAAACATCTTCAG
TGGTGATGAATCAAAACTTGGAGCGTTGGAGCACCATCAAGATTCTCTCGACAACCTAAAAAGTGATGCCATGGAAGAAAAGACAACCAGGGAGAAAATA
TCAGCAGTTTCAGGAAATATTGATCAAATGGTGGAAAGTGGTCATGACGAAAATGCTACATGTGCTACTGGTACATCAAGAGATAAGTTTGACTCTTCGG
ACAATGCTGCACCTGACAAACTTTCAGATGACACTTTGGATAATTCAGAGGATCAGAACATTCCCTTAGTAAAGCATGAGGGGTCATTCAAGGAAGGTGA

CATGCCTGCTTCAGTAGAAGAGCCTGAAAACACGGTCCTACCTGCTTCTGGGCCTCAAGAGAAAATGTCAGCACCATTGGTCAATTCAAGAAGTGATATC
GTTTCGACCACTGTTACTGGCACTTCCGGTAATTCAGAGGATAAAAATGATTGTTCAGGTGTTTTTCCTGATGATTCTTCTGCTGCTTTGCCTGATGAGA
AGGATTTGAAAATGTCCACAATGGATTATGAGAGGTCATTCAAGGAAGTTGCTAAACCTGCTTTAGAAGATGAAGACCACAATGTTATTTCTCCTGGCTC
CAAACCAGGTGGAGAACTATCAGCAGTTTCAGGGAACTCAGGCATAGATGCTATTTGCAGTGGTACTGTTTCTGCTGCACAAAAGGCGGAATACAAGGAG
CAGGCTAATTCTTTGGGTGGTTTGCCCACAGGAGAAACTCAGGATAAATCGGGCAACCATCCAGATGCTTATTCACAGAAATGCCAGACTGACGAATCTT
TGGTACAATCTGAACATCATATAGATCCAGCCACACCATCCGCATTAGGCATCTCAACTGACAAGGCTGTTGAAAAGATCGTAGAAACTCCACTGAATGC
AAAGGATGATTTGAATGCATATGCGCAAGATACAGTTCATGGTACAGATCATAGTCCTGGTACTGTTCCATCCCAAGGCAAGCTAAGCTCAAGCTTATTA
GAACCTGGGAACGGTGACGGAATCTGCTCTGATGCTACACGTGATTCCTCTCCAGTGATTAGTTGCACCGAGCCCTCTCCCCAAGGTGGACCTGACAGCA
ACACTATGGTTCATCAGACACTTGATGAACATGAGGATTCAAAAGATCATGAAGCCTCAGTTGACGCTACGCAGAGCTCAAAACAGTATTCTACCATACA
TGTAAATCCTACTCCTGGTTCTGAGGAGACTAATTCAGCGGGAGATGATAGAAGTTTCTCGTTTGAGGTGGGAGCTCAACCAAATGTATCTGAGAAAGCT
CATAGCCCTGCTTGGAGCCCTTTCCCCAGATACAGAGCTTCTCAGAGTACTGAGGTAACCACAGAAAAACCTCAGGCTGGAAGTTCATTGGAGAATAGTG
GTGATGATAGTAAAAGAACATCTATTGTGAAGGCTGGTAAAGAACAGCTGTCAGAAAGGAAAATGAATGGAAGTGCCGGGGGGGCCTCAGACAACTCTAA
TATTTGCAATAGCACCAAAAGTTCACCGCTGGAGCAGTCAGAACAGCATCCAACCCCCAAGATCTCTGATGTGGCGAATCTACCTTTCACAGATGCGCAG
CATTTGCAGCTACGTGCACAGATATTTGTTTACGGAGCTCTTATCCAAGGAACACCACCAGGAGAGGCTTACATGGTGGCAGCTTTTGCAGAACCTGGTG
GTGGTAAGCCTACATGGGAGGTAGCTTGGCGAACTGCTCTTGAAAGATTTCAGCATCAGAAACCAATTTTTCCCGGCTTAGAGACCCCCACAAGCTCACG
TATAGCTGAGTCTGAAAAAGCTAGTAAGGGTACAGCAGTTAGAACTGCGGCAGCTAGCAAAAAAGGTGGGAAAACAGTGTTGCCAGCACATTCTGCTGTA
GCCTTGCACTCGCCTACTTTCAATGCGCCTCTTGGTAGTTCCACATTCAACCTGCAACGAGGTACTCATCTGGATTTTAGCCAGGCTGTCTCACCAGTAT
TTACATATAATTCACACATGAGGCAGCCTTCTCCTGGTGTTGCATCTTGGTATCCTCAGAGCCCTGGTCCACGTCCTTCACCCTGGCTGGTTCCACCACA
AAACTTAATGTTTGATTCATCGATGCAACCGGCTGTGCCTACAAATGAAACTGCAAAGGGATCATCTTCCAAAAATACATCAATTTCACATGCTGTTTCA
CCTGGGGTGTCCCTTCCAAGTCCAGCACCTTCTATTGTTTCTCCTCCGTTGGCAGTTGTACATGAGGAAAAACAGAAGGCACCAGCTTCTACTTCTAAGC
ATGGAACAGCATCGCAGAAGCCAAGAAAAAGAAAGAAAGCTTCAGCAAGTCCTGAACAGCAAGCTGTCATCCCTTCTCCTCAGCTCAAAACAGACACGGC
GTTGTTTACTCCTGCTGCTAAGCATACAGGAGGCTTCACCTTATCTACTCATTCCCCAAGCAATGCCTTGGGCAGCAGGCTTGTTTCTAACACAGGTCAG
ATCACCTCTGTGCCTAACTACCAGATTACTGGTACTATGGATGCTGAGCAAAGAATCATCTTTTCAGAACAGATCTGTGGTGCAATAGAACAATCAACAG
GACAAGCCAAAGGTGCAAGTATGCACTCTATGGCAGCAGTTAGACACAAAGAAAGCATATGGAGCCACCTATCCACAATCTCAAGAAACAAGTTACCTCC
AGAAGTTGAAGGGAAGCTTACCTCAGCTGCAGCTGCTGCTGAAGCAGCAGCTTCTGTTGCAAAGGCAGCTGCAGAAGCTGCCAAGATGGCGTCAGAGGCT
GCATTGCAGGCGAAAATGATGACTGAGGAAGTTCTTAGCTCCTCTAAATCTGTAAATTCCTTTCAGGGTCATGAAGCTGGTGGATTTAATGTCAGTAGCA
ATCCTCCTAGCTTGTCAAGTTCAACACCGACATCACCTTGGAAAATTAAGGACAACAATCATGCCCAGGGTTCCATCATTTCAGTGGCACGAGAGGTTGC
TAGAAAGAGGGTTGAAGAGGCATCTGCAGCTACAAAACGTGCAGAAAACTTGGATGCCATACTGAAAGCTGCAGAGCTTGCTGCCGAGGCCGTGTATAAT
GCAGGGACCATTATTGGAATGGGTGAACCTCTACCATTTACTCTAAGCGATCTTTTGGCAGCTGGCCCGGATGGCTACTGGAAGTCTGAGCGAGTGAGGA
ATAAGAAAGCTGAAAGTGTCACTGATAATCCAGTAACAGAAACATTGGAGGTAGATGTGCCTGCTGATCTAAAAAAATCTGGCCGAAAGCGTGGCCGGAA
ACCTAAAGATCAAGCACAACTGAATTCAGAGCTGTCTTCAAGTGGCAAAGAATTGCAGCCAGATGGGATGCACTCAGATGGGATCAAAGATTTTCCCACC
ACAACGTTGCTTGGTGATAAAAGTAATGATACAGAACCAATAAATATTATCTGGAATGGTATTGAAAAGGGATCTGCTGTTGAGGTTTTATCTGACAAGG
GTGGGTCTGGAGTAGCTTGGTTTTCTGCTAAGGTTGTTGATATAAATGAACATAGTGCGTTCGTCAGCTATGACAACCACAATGAAGGAACTGATCCTTG
CGAAGAATGGGTGCCATTGAAACAAGAGGGTGAAAAGGCGCCTCAAATACGACTTGCTCATCCTGCTACCCTCTCTAAACTCACAACTAGGAAGCGTCGC
AGGGAAATGGCAGGAAATTGTTCCTGGGCTATTGGAGATCATGTAGATGCATGGGTCAAAGATAGTTGGCGAGAGGGAATTATTGCTCAGAATTATGAAG
CCGATGGAACAAAATTTCTTGTACAATTTTCAGGAGGTGCTGCTGATTCCTTGGTTGTTGATGCTTGGAGTCTTCGTCCATCACTTGTTCGGAAAGATGG
CCAGTGGATAGAGTGGTCCCAAGCGAGAGAAAGGAAATCTAAATATAATAAGGGTGATTCACCTCTTGAGAAACGCCGAAGGACAGGTCTGCAGCAAGCA
GGTGGCAATCTACCTATTGGTTGTGAAGCAGGAGATCCTTCTAATGACAAGAGCACCAACAATGCAAAAAAGCCAGAGGAGCCACTGGCTTTGTCTCAGA
CAGACATGATTTTCAACATTGGGAAGAGTGTAGTTGAGAATAAAACTGATGCTCTTGCATTCAAGCGGCCTGGATTACAGAAAGAAGGATCAAAGGTTTT
CTACGGTGTTCCAAAACATGGAAAGAAGAAGAAGTTTATGGAAGTAAGCAAACATTATGTTGCAGGCCAAGCTGACAAGATTTCTGAGGGTAATGCACCT
AACAGATTTGCGAAACATGTGGTGCCACAATTACCAAGACCGCGGGACAATACTTCCAAAGTTGATCATAGAGGAAGGAGAGTAGGCGAGACGAGGCCCA
GAATACCTAAACCCTCAAAGTCTCAGAATGTGGCAGCTAACAGTGTTCCTGACAAGGATCCTTTACCCTTGTCTCTCCCAAATTCTGGTGTTTCTGAAAG
GAGTTTTGCTTTTGTGGGAAGTGCGGCAAGTGCTTCCAACACTGAGAAGCCCACTGTAGAAAAAAATAAAGCCGCGCTTGCTGCTGGCCTTAGAACTGAA
GATCCTTCTGTTTCTGAAATGCAAGCTGCATCAACTTTTCCTAGTTCCAAGCAGAATGTACCCACTACTAATCGAGCTAAAAGGAAATATGTTCCTACTG
TGGATAACTTGAACAGAAGTACCATAAAAACTTCTGAAAAGACTACTAATTCTGATTCTGCTGAACCCCGAAGGTCCAATCGTCGAATTCAACCAACTTC
ACGGTTACTGGAGGGTCTGCAAAGTTCTCTTATAATTTCTAAAGTTCCTGGTGATAAGGGTCCTAGAAGCATTTACAGAGGTCCTTCGAGAGGAAGAGCT
CATGGCTGAGAAAAGGCACCATAGTGCGTTTTTTGCGAGACAGCTCTTCGCTTTGTACATAGCCGTAGCTTTAAGTATGGAAGCAGAAGGATGGCATTGG
AGTAAAAGGCTCTATTGGAGCACAAGGATTCAGATGTATTATAAACTAGCTTTATTATGTTCCCCTTTTCTTTTGCTTGCACCGGACAGAGCTCTTCGTG
TTGTAGAGTAGCCAT
>Locus_10795_Transcript_56/70_Confidence_0.408_Length_2722
ACATTAATCTGAGATCTGAACAATTTTCAAGTACCACATCAAAAGCTTCTGTTGAATGTTTATTTGAATGGAACACTTGAATGCATATCATGAATGTGGA
GGAATTGGGCGGCCAAGAACTAGGCAGACATCACATCCCATCTTCCTTCACGCAACTTATGTCTAGCAGTCTAGCTAGAAAAAGAAAAGAAAAGAAGGGG
GAAAACAAACTATGTACAGAGCAAGGCAAAAGAAAAGAAAAGAAGGAAAAAAAAACTATGTACAGAGCAAGGCCATCATCTCGGCATGGACCTCCGGTAG
GTGCTCAATTACTCAAATCCTATTGCAATCCTGTAAAGGATGGCTTTATTCTAGATCTGCATAGGTTACCACCTGCAAGAAAGCCACAACAAACAGCAGC
AGCCGAACAGATGCTCACCTCCCTACTGCGCTAACTCAAAACTGGCAAGAAGTGCCCAGCCTCTGCTTCGCCCTTTGCTCTGTAGCGCAGCACTTGCGCG
AGCGAAATGCCTCAGGCAGCAGCTCCTGGAACTTCTCAAGGAACAACCCCCATGTCTCTGGTCCCATGTCAGCCAGCTTGACGAAACTCACACTTGTTGG
GAGCATATCGTTTGCACTCCCCCAGTTACCAGTTTGGTTGCTGTTGCTCTCTGAAGTTTTCACAGAATTCACACCCTTTGGAACAGCAGACAGCCGATGC
TGGCTTTTGTCAGTTAGAGTGACACCCTTCAGTGAGGTTGACAGCTTCGAAATGTTAGCAAGCCACTTATTGACAGGTTCAGGGCAAGCTAACTTTCCTG
CATTGCTTTCTACCTCCTCCTCAATGTCGTTGTCGTCGTCGTCATCATCATCATCATCATCATCTTCTTCTTCTTTCTTCTTTGGCCTCATCCAGTTTAG
ACAATGAGGTTCTGGAGGTTTGCCCCTCCCCTTACATTCAAAATGGTAGGCTGATGGTTCTTTTGGCATTTCATGATCTTCACTATCGAGTTCAAATTGA
GTTAAATTCTCATTGCCAGTGTCATGTTCTATGGAACAGTCTTCTTGTTCTGTTGCTAGCCTCCTTGCCTCCATGCAGACAAACTCTAGCTCACTTGGCT
GATCTTCCATCCCAGTAAACTCTCTGCTCATCATCTCCCCAATCTCTGCAAGGCAGAGACCAAATGCTGTGGCTTCTTTCAGAAATATGGTTGAGAGTAT
TAGTACTCTGAGGCATGCTTCATGGATCATAGGCAACTCCATACGAAGCATGTCAGAATCTTTGATTGGATCAAGATTTGTGATGTACTCAAGCTCATCC
TCGGAGAATGGGATTGATGCCTGTGGCCAGTGGATCCATTCAAAATAGGGATCCTCCAAACACTCTGGGAGGCAGAGACCATGATCAATAGGAATAAGCT
CAGTCTGATTCCCAAATTTTCCTGCTCCAGTCTGCTTCCTTACCAGAAGATTTCCAGCATGTCTATCAGTATTGAAGATCCTGATGTCAAGAATACCAAT
CCTATGAACAGCAGAGACAGGAAAACTTGAAGTGCCGTAGTCACTGGCATCAAAATCATGAGGAATAAACTGCTGAAATGAAGCAATCTTCCTAACAGCT
TGTGTACCACCATCAGCAGCATTTTTGTTAGCACAGTTGACACTTTCATTCATATGAAATACGGGATGAGAAATCTTAACAAGAACTGTCGGAGGGACAT
TAGCAGAGTTATCATAATCCAGAAGGTAGGCAGCAACCTCTCTGAAACCAGTCTCGCCGACACGAACTGATCTCTTGAGGCCTGGCTGCCCGAGGGCTTT
CCCTATAAAACCTTTTGGATTGTTGGGTGCAAATGGCTCCTCATCATTTGGCTTCACAATGGCAGCATTCTCACCTTTGCTATTCCTGAAATAGTAGGCA
CCACCAAGCCCACTACTGACAGGTATGGGATCCACACCGTTTCTTATAGCCCTAGTAACATCCTTCACAAGCTGCCTTGTTCTAGAGCAGCGATTCGGGC
ATACTAACAGTTCAATTGGACTACCCCTATCTCTTTGCTGCTGGAGATCCTTGCCTGTAGGAGATGACATGCATGGTGTGGAGGAACTCCGATGCATGAA
ACTCTTGGTGAGAAGTAACGGGGAGTCATTTCTGATGGTGCTGAGGTCATTTTTTAGGACACGATCACCTAATGTCAGAGAACTCTCCTCGGTGGGAAAA
TTGAAGGCCACCTGCAACTTTCTTTTGACTGTGTGTGCATTGTCGCCACGATCTAGTTGAACAGCCAGGACACAACCAGTGTCAGTTTGGACAAATAGGC
GTTTCCATCCGCCAGCCCTGGTCTCGCTTCTACTTTTGGTAGGGTACTCAGCACTAAGAGCTCGGCCCAAAGCTGAAACTGCCATCTGGGTCTGCACTGG
GCTCTCCGAGTTGCGAGACATGGCGGACAATGACAAAGGAATTGAGGCCAATGCACTGGAGGCAGCTGCCACAATGAGGCCTTTCTTGCTCCTCTCTTGT
TTTCTTCTGCGCTCGTCAAATTCAGCAGACGAGTGCGAGAGGGATGTCACGGCAGTGATCATCAGGCAATGAAGGACATCTTGCACACTAGCTAGATGGC
ACCAGAAAGCGACAATAAATAAGGAAGGTGAAGACAGCAGCAACCCCCCTACAACTGGGAGCCGTTATGAAATCCGACAAGCTAGATCCTGATCCAATGC
TGAGCCTCGGGAATGGCGGCCT

>Locus_12535_Transcript_32/73_Confidence_0.321_Length_2450
AATTTTTTAAAAACTGAGTAATAGCTATTAAAAACTAGCATAAAACTAATTTGTTTAGATCCCACCCACTTTTCGAAAGTCAGTGCAAAAAAACTGAAAG
GATCCGAGCAGGCCTTAGAAGAATTTCAGCAAGACAAAGATGCTTCAGAAAAGGAGGTGAGATTCCTGATTTCACATAAGTCAGTTCAGAAAAACTGAAA
GGATCCGAGCAGGCCTTAGAAGAATTTCAGCGAGACAAAGATGCTTCAGAAAAGGAGGCTTGATGTGGTATATGTTTTCCCCTTCCCCGACCTTTTGATC
TCCAACCATGGCAGTGGAACAAGGAGCAAATTTGCAATCTTGGGGTAGTTCCGGTGGTGAAACGTGAAAGATTGGGGATTTAAGGGACGTGAAACTGAGG
GGGTGGTTGTCATGATGTGTAGGGTCCAGCACTGGAGTGGTTGATGCTGTGGTTTCCAGGTTAAGGGGAGAAGAGGAATAGGATATAGATCTCAGGAAGC
CAGAGATGAGGTCGAGGGGAAGCCAGTGGCCAAGCTTGTGCTCCAGTGAAGCTGATTCTCTTGTTCCCAGTTGATCCATGCGCCGTGGCCTCAGTTCGCA
GGATCAGCAGCTCCATGGTTCTGTGTGGTGCTGCAGTTTGAGAAAGTGGTTTTCCAGGGGGAGGAATCTCTTGTGGATTCCTTTTTCTGGGTGCAACGAA
GTGGGAGGTAGGAGCAGCAGTTGAGATTTTCAATTCTGGCATGGGTTCTTTTGGGATGGACTGGAACCAGAAAGGCTCGGTGTTGTGGGATTGGGAGAAT
TTGCCGCCGATAGGCAGAAATGCAAACGAGAACCTCAAGATTGCGCCACAGGCTGAACCGAAGTTTGCAGGTGTTGAGGCAACAAGGCATGAATCGGGGC
ATTCTTCTTGTGGTACTTTCTCTTCCAGCTCGGAGATGGGGTATGGTTCATCCAAGAGTTCCATATCAGCGTCGATTGATTCTTCACCCAAGGTGGGAAA
CAACGTGGAGCTCAATCTTGCAGCTGTCAAAGTGCCTGACAAAAACACCGTCAAGAACACTGATTTGGGTAAAGTTGATGACGCTGGAACTTCTCCATCA
TCGATGATAGCCGTCAGCAGTGGAGAACCGGTGATTGGCCTGAAACTTGGCCAAAGAACCTATTTTGAAGATGCATGCGGAGGGCAAAGTGTCAAGAGTT
CACCATCCAATGTGAGTGCAGTGACCCCTGCTTCTGCGAAGAAAGCAAAGATGATTCAAAACGCACAGAACTCGTACTGTCAGGTTGAAGGTTGCAAGTT
TGATCTCTCTTCTGCTAAAGATTATCATCGGAAGCACCGAGTCTGTGAAACTCATTCTAAGGCTCCCAAGGTTATTGTTGCTGGTCTGGAGCGACGCTTT
TGTCAGCAGTGTAGCCGGTTCCATGCTTTAACTGAGTTCGACCAGAAAAAACGAAGCTGCCGGAGACGCCTCAATGATCACAATGCCCGCAGACGGAAGC
CACAGCCTGAAGCAATTCCTTTCGGTTCATCAAGGCTCTCTGCAATGTTTTATGATGCAAGGCAACAGACAAGTCTTCTGTTTGGTCAAGCTCCTTATGG
TCAAATGAGAAGCTGTGCAAACTCTTCATGGGATAGCCCAGTAGGAGGCTTCAAATTTGGAGAAACAAAAGCTCCTTGGTTAAAGCCAACGAGAGCTGCA
GGTGTTGATGGGATGCATGTATCAAGCCAGCAGGTGTGGAACAATATTACGCCACACGGTGCACATCATGATTTTAATGGTATCATGGCTTTCAAGGGAA
CCAGTGCAAATGTCCTTAATCAAGGTGCCGAAGCTTCTCCGGTCGTCTCCAACTCGAATGGAGCCCCAGATCTTCAGCGTGCTCTCTCTCTTCTGTCAAA
CAATTCAGCTGGTGCTGCCAACGACCAGCCAACTCCTCAGCTGCACCCTGGGCTGACCACCCCTTGCCAGTACCTCCAACCCTGTCACTTTGATGCAAGCC
TCACCACCAGGACTCTGGCAAGACGGCACGCCCCTCGATCATCAGGCGCGGTTTCAGTTTTTTGATCCCCTGGGCGACGGCAGTGCCATTGCCACACCGC
ATCAGCTCCAGCTCCTAAAACCCTTCGCCTACGACAGCTCCTCGTCCCACTATGACCAGATGCACTGATGCTTCCTTCAGTTAGCGCAAACCGCGGTTCA
GTTGCGCGTAATCAGACATGGTTTCCACTGTCTGAAACTCTGAATTTTGAAACTGCGATGTTCTGAAAAATAAAAGCCTGCGTGCCGCAAACTCAGGTTC
GAGTTGATCTAGTAGTGTTCCCGTTCAGGGGCTTGAATAATCCATCCCTGTGATGGCGTGTAACACAAATGCTTCGATTGCGCCGGCAATTGATCATTGA
CTCTTTTCGTGTTCTTGATAACATCTCAATTACACCTTGAATGGAACACT
>comp78487_c0_seq10
ACCCGATTTGTTTAGATTGCAATTAGACTAAGTATAGTTGGTGAGAAGACAGGAGTAGAGATAAGGGCCAACAGCCCAACAGAAAAACCAAGCAAGACTG
CAACTGCAAGAGAGATGGGGAGAGAGAGGAAACCTTGCAGAGGAGAGAAGCAAGGTGGGAAAGAGGCAGCAAGGGAGGAGGGCTACAGGAAGGGGAACAT
GCTCCCTTCTCATCTCACAGTATGAACTGAAAAACAGGAATAGAGAATTTCCGTGAGATGAGACGTAAGGAAAATCTCCATGATACTGTTCAATTTGAAT
ATTGCGAGTACTCTGTCCTTTTTCATGTCTTCGGTAGTTTAGTCTCATGCAGCTAGAATTTTAACTCCTACGGTGCTCCTCTAGCAGAAAATGGCACCAT
AATGATTCAATTTGGTCATCAAATGCCTGATTACGACTCATCAGCTACCCAATCAACCAGCGAGAGTCATCAAGAAGTGTCTGGAATGAGTGAAGGAAGC
CTCAACGAGCATAACGATCGATCAGGTAATCTCGATGGTTACACAAAGAGTGATGAAAATAAGATGATGTCAGCTTTATCTCTGGGCAATCCAGAAACTA
CTTATGCGCATCCAAAACCTGACCGTAGTCAGCCCTTTGCCATTTCGTACCCGTATTGTGATTCATTCTATGGTGGTGCAGTGGCAACGTATGGCTCACA
TGCTATTATGAATCCCCAGATTGTGGGCATGATGTCGTCCTCCCGAGTGCCATTACCAATTGAACCAGCTGCAGAAGAGCCCATTTATGTGAATGCAAAA
CAATACCATGCGATTATCCGAAGGAGACAGCTCCGTGCAAAGCTAGAGGCTGAAAACAAGCTGGTGAAAAGTCGCAAGCCATACCTTCACGAGTCTCGGC
ATCAGCATGCCATGAAGAGAGCTCGGGGAACAGGCGGGCGGTTTCTGAACACGAAGCAGCAGTCAGAGGCTCCTGGCGGCGGCACCTCGGACGCGCAACG
CATACCAGCAAATGGTGGCCTGTTCAGCTTACCTCCCAGTGATCTCCACTATCGCGCGAGAGGGGGCGCTTAAGGAGTCCCATGGCAACTCATCCTTGGC
TTAAAAAAAAATGTGTGGCGCTCAGCAATTCGACCTCTGCTAGTTCAATGTAAAGGTGTGGGACTGTAACTGTGGGTGCTTGAATTGGCCGCAGCCATTC
CTGCGACGCGTCGCAGGTGTTGCCGGTTCTGTGGTCGTGCTAGACTTGTGACCGGCACGACGAAACTGGCACCTGTTTCCTGTAACTGTTGTCTAAGATG
ATGATGACTACTCCAATAACCATTCTGTATGATGCTTTGACGTCGGTGTTGCTCCTT
>Locus_17813_Transcript_11/20_Confidence_0.367_Length_1270
TGTGCCGGGAGCTCAGGCGTTGCTGGATCCTCTAGTGCCGCCGCCGCCGCCGAAGGGCGTGCAGCCGGAAAAGGTGCTCCCTGGTGTCCTGAAGTTCTCC
ATGGCTCAAGGTAAAAGGGGGAAAGGGTCTGAGCATTCTGCCACTGTTGCTTTGCAATCACCATTTGCACTGTACAATGGTCATTTTGAGCTAGGCCTCG
GTCAATCAATGGTTCCCTCCAATAATCCTTATGCTGACCAGCACTATGGCCTGCTTTCTCCTTATGCAATGGGAGCAACGCCTGGCGGGCGTATGCTTGT
ACCACTGAATATGCCAACTGATGCCCCAATTTATGTGAATGCCAAACAATACGAAGGCATCCTTCGTCGCCGCCGCCATGCCCGTGCCAAAGCAGAGAGGGAG
AATAGGCTGGTCAAAGTCAGAAAGCCATATCTGCATGAGTCACGGCATCTTCATGCACTGCGTCGCGCAAGAGGCTCTGGTGGACGCTTCCTGAACACAA
AGAAAGAGGTCAATGGGAAGGACACTGGTGTCGGAAGCAAGGCAATAGTCAGCAATCCACTAATGCGCTTGACTGCATCCCCAAGTTCAGAGATTCAGCA
GTCAGACCTGGGCAACCCAAGCAGCGTCTCCAGCCTATCCGGCTCGGAGGTGACAAGCATGTATGATCATGAAGATGTGGATCACTACCACAGCTTTGAT
CACCTCCGGACACCCTTTTTCACCCCACTTCCGAGCATCATGGACGGTGAGCATGGGGATGGTAACCCCTTCAAGTGGGCAGCAGCCTGTGAAGGCTGCT
GCGACCTCCTCAAAGACTGGAGGGGATACCGGGCAGCTGAGGGTCAGGGATTGTCCCTGCCATCGTTTGGCTGTCGGTAGGCAAATCATTCTTGGCTCAA
GGCATTGTGGTGCTTTCTGCTCATCGCCTAATTCTCCTAGAAACCATAGTTGCTGGGATCTTGTGGTGAGTGGAACACCCCATGCCTGAAAAAAAATCTG
ATCTAAATTAGTCGTACTAGCTACTGCTATCTGAATCGTTTCGCGGTAGCAGGTTTCAGCTTAGTTTTCTGAATAAATCCTGAAACTTAAGTTTAGTTTG
GAGTTTCAGAGGTATTGTCATTGTAGTATGTTTGGACTTTGGTGTCTGATTAGTATCAAGACTATGGCTTTGTTGGTGATGAATTGTGGTGTGCTTGAAG
CTTGTGCGGACTTTGGTTTGTACTTTATGGTGTGTTCAAAACTTGAATTTGGCAATGTAATAATGTACTG
>Locus_1016_Transcript_991/2070_Confidence_0.002_Length_4220
GTGGAGGCGGCAAGCTAGAGATAACAACTGCATAACTTGCAAGAAAATCGTAAGTTTCTCAATTTTTCTGTTCTATACATGAGAAAACGGTGTTGATTTG
TGTGCATGCGTGCGTTTGTCAATGATCTGAATGATTTGCACATGATTTGTGTTAACATGCTGTGAATTCAGGGTATGGCAAATTTGCCAAATATCTTTTC
AGGATGCTTAAATTCACTTTGTTTAGAACTGTTTTGAGAACCGTATGGATTCAATAGACACCCAATTACTTGGTTATAATTGAGTTATTTACTTTACGAA
AGGTCGATGTGGTCATAGCATATTAAAGTTCCTTTTAATTGACCAATGACCGTATGGTTACTCTCATTCCTTCAATTCATTGCTCTCCCAGTCACAAATA
AGTGATGTTTGGGACATTGACAAGGCCTCCAAAACTCAAATGACCACCTAATTCTGCCATAGTATATTTTTAGAACCTAATAATTTGTATATCATTTCGA
TGTGTATACATCATTTAAGTTTAGAACCCAATAATTTGTATATCATTTTGATGTGTATATATCATTTAAGTTTAGAACCTAATAATTTGTATATCATTTT
TAGAACCCAAACCTCAAAGACATATCGAGAGGAATCCATGTATATCATTTTGATGTGTTCAATGTAAATATTTGTAAAGATACCAGTAGCGAAAGTTTGA
CTACACACATTCCAAATGTATAGAGGTATAATTGGAGGTTCAGCCGTTCAGAGACCCAGGTCTTGGGGTCCAGGATCGTTTTGTGCTATAATCGTTTACC
CCTTGAATTTCTGTGATTAAGGTGATAATCTTAATTTACTGTGACATAAGAATAGGCCAAATATTTTCCTGTACAAACTTAAGCTAGTGTATCTGTCGTG
TGTCTTGTCATAGTACAATCCGTTATTTCTGGTTATTTTCGGTGCGTCAACGTCTTACTGTTGTTATTCCTTTGAACACTACAAAATTTTAAGTGAGCAA
CTAGCATCCGTATATTTACATATGTAGCTGAAGAAAGGTTGATCCCCATTCTGATACTCTGGAATGCCTGCACGGATAAACCAGAATGCAATTTGTGCTC
TTCTGCTATGCATACAATTCTCAAAATAGAAAAAAATAGCACCTGAATTTTTTCCACACACCATATCTTTTTGTCAATATAAGATTTGTAGCTATCATGT
ACTCACGTTGGTCAACAAACTTGCAGAATGTGTCAAATAGTTATAATACAAATCATTTGCTGTATTCTTCAATAAAATACATTTTATTTGAAATAGATTT
ACACTACCATTATCAATCACTCATGGCATGCATAAATAGATTTACACTACCATTATCAATCACTCATGGCATGCATACCGCGTCAGTCTTGCTATGTGTT
TAAGCTCTTTACAACCAATAGTGTTTCTGGTTGCTTTGAATCGTACCGTTGAGAATAATGAACATAAGCGGTGCCATGCTGCATGCCTTCATGGATTTAG
TTGTAATCTGCAAGTTTTCAGTTAGTAGTAAACAATATGCCACTAAAGAATTAAGAACAGAATCAATAGTTCCTTTGCAGAAAAAAGCAGGGTTACCCCA
GTTTAGGAGGTGCAGTTTGCTCAACATCATTTTCTTGCGGTGGAGTGTTAAGTTCAAGATGAACAGGAATGTTGACGTTCTCGACAACTGCAAAGTTTAG
AGTTGATGAATCTCGGTTGACTTCACTTGTCACCTCTTTCTCGTAGAGCTGAGTAGAGAAATAAACCTCAGTCAATAATGGATGGGGTGTATATCCTGTG
AATTTTCATTCACGATTTATCAAATACCTTCTTGTATAAATCAATGTTTTCCTGACGGATTAGGTTGACCTTTTTGTATAGTTCCATCTTGTCTTGTTGC
ATGAGACTTCCCTGAAAATGAGCCAGTAGAGTCAGCTTCTTGAAATCCACTCAGAGACCTCTCATTGCAGAGTAATAACAGAATATTCCAGAACCTTTCG
ATTCAGTTCGTGAATTTCATCAATCAAGAGTTGGTCCTGAAAGTAAAAAACGTCATCCATCATCAGTTGTATTCTTGGATGAGGCAATCTGGTAACGAAA
AACAGAAATATGGCTAGCATCCTTACCTTTTTTGTCCGGATGCAACGTAGGCTCATTTCTAGCTGATTTTCTAGATTTTGGAGTTCCTTGACGCTCAATC

CAGAAAGATCTTGTCCCATCAACTGACTGCAGTCAATCAAACTGTATTCAGTTTACCTGATAACCTGATATTCCTCTGCCCATGTGTATTAATGTTGTGT
TGGTTGGGAGTGAAGTGCGGACAAATCAGAGACTCAAGAAACTACCGGGCAAAACAGATGATAATATCATATTTGTTTTCCATTAGTATGTACTCTTCTT
CTTTGGAAGTTAATTGGAATGCATAAAAGCTAAATAGGTACATTTTTTGACTTATAAATGGCTTGGCATAGGAAATTCATAAACTGTTACCTCCTTTTAC
ATTGATATTGTTTATGAAAACTGATGTCCTCATAAGCAAAAGATTGTCCTATGGAGTCAGTAAATCAGTATGTCTCCTTAAACTTGGGCATTGTATGCTA
TCTTGTATACTAAATTCATTCTTATCCATTGTTTTACAATTTTATACACTCTGCACAAATTAGTTGGTGGAGGTATACTTGTCACTTTACAGCCCTTAAT
TTGCATCACCAAGAGTTATGGATGATCTGTAACATGGTGTAGTTGATTCATAATTGTCTCGTCAGAACCTAGTTTTATACCTTTATAAATAAGACAACCA
GGAATTGAGTATTGCTACATGGTGAATATTGTTTCTTCTTTTGGTTCTATTCTAATGTTGTTTTGAATTTTTGTATATTCTTGAAACAAACAGTAGGAAG
GATTTCCTTCCTCCAGAGTTATCTAACTCGGAAGTTAGGCACTTGTGCTCATGCTGACAAATCCTATAGTTTGATCCTTTTACATTAGGCAAATTTTCTG
ACTTGTACATTTGTAAAATTTGCAGGGTTTTCTAGCCATACCGATGACTTTCTTGCAGGTTGTGCAGTTGTTGTCTCAAGCTTGCAGCCTCCCTTTGCCA
GAACTATTGAATATGAAAGCACAGTTAAGTAATGCCACATCCAGGATTGCCAAAATACAGCATTGGTATAAACATGTTGCATAGAAGCATGTCTGAGCTG
TCAGCTAAACAAATTGTACCTTATGAAATAACTGAATTAAATAAGATGACATGCCACTATTTCAATCAAGAGATTTACAGTGCGCTACAAAGTAACTCTC
TTTTTGAGAGGGGTGAGGGGGTGGCAACGATTATAATAGCAAATACAAAACCTTTTAAACTTGAAGGTTGAGATTCTATATTATTCAAATACTTGAATTA
AAGAACTTTATTATAGTGAACTATCAAGCTGGACACATATTGGCACGCTACACTTGCTGTATTGTCTCCTATGCTGTTGTTCACACTTTTCACACACAAT
TGCTCTGACAACTGAATTGAGCTAAATTAGCCTCGCAATACCTTGTGCTCGGGTCTAGGGACAAAACGGAACATGTCAGGATGGTGATGATCTTGCTGGA
ACATGTTTAAGCTGAAATTAGATGACTGTAGGTTTTCTCTTCGTTTTCTAGAATGTTAAAGGTCATCGTCTAAAATGTTTCACTATAAACCGGAAAGGAT
TCATGCGGCACTCATTTTTCTGGAGAATGGACTAAAACATCTATTGACTGCTGATCATTTTCAGAAGATGAGAACTTGAAACAGTGCTGTGTCAGCATCA
GCTTCGACTTACTGGCAAGCTTCAGGGCAGGCTAAGCAAGGCCCCTAAGATTTCATCCGGACTACAGCTAAAGTGAAAAAGGGTTTATTCATGATAATTT
CAATCTAAGACCTGTTTAGTTTCTATTAAGGATGCTTAAGTATACCAACATTTTTTTTATCTTTCTTGCCAAATCTGCAAGAAAGCTTGGTCAAGAGAAT
CTTGCCACAACTCCCTTTGAGATGCCAAATCTGCGGTGAAGCGGAAAATTATAAAAAACTTTTGGCATTGCTATCCTGTGTCTAAGAAACAAACATCATC
CTAATTTGGTGGAATTCGCCTGATGTGTTGTAGCAAACTAAGGGGAACTGTCTGTCAATACACCTCAAGTGCCTATATGTAGACCGGACAATATTTCAAC
GCCTCTTAATTCAGAGCAGT
>comp76332_c0_seq25
ACTGCTCTGAATTAAGAGGCGTTGAAATATTGTCCGGTCTACATATAGGCACTTGAGGTGTATTGACAGACAGTTCCCCTTAGTTTGCTACAACACATCA
GGCGAATTCCACCAAATTAGGATGATGTTTGTTTCTTAGACACAGGATAGCAATGCCAAAAGTCTTTTATAATTTTCCGCTTCACCGCAGATTTGGCATC
TCAAAGGGAGTTGTGGCAAGATTCTCTTGACCAAGCTTTCTTGCAGATTTGGCAAGAAAGATAAAAAAAATGTTGGTATACTTAAGCATCCTTAATAGAA
ACTAAACAGGTCTTAGATTGAAATTATCATGAATAAACCCTTTTTCACTTTAGCTGTAGTCCGGATGAAATCTTAGGGGCCTTGCTTAGCCTGCCCTGAA
GCTTGCCAGTAAGTCGAAGCTGATGCTGACACAGCACTGTTTCAAGTTCTCATCTTCTGAAAATGATCAGCAGTCAATAGATGTTTTAGTCCATTCTCCA
GAAAAATGAGTGCCGCATGAATCCTTTCCGGTTTATAGTGAAACATTTTAGACGATGACCTTTAACATTCTAGAAAACGAAGAGAAAACCTACAGTCATC
TAATTTCAGCTTAAACATGTTCCAGCAAGATCATCACCATCCTGACATGTTCCGTTTTGTCCCTAGACCCGAGCACAAGGTATTGCGAGGCTAATTTAGC
TCAATTCAGTTGTCAGAGCAATTGTGTGTGAAAAGTGTGAACAACAGCATAGGAGACAATACAGCAAGTGTAGCGTGCCAATATGTGTCCAGCTTGATAG
TTCACTATAATAAAGTTCTTTAATTCAAGTATTTGAATAATATAGAATCTCAACCTTCAAGTTTAAAAGGTTTTGTATTTGCTATTATAATCGTTGCCAC
CCCCTCACCCCTCTCAAAAAGAGAGTTACTTTGTAGCGCACTGTAAATCTCTTGATTGAAATAGTGGCATGTCATCTTATTTAATTCAGTTATTTCATAA
GGTACAATTTGTTTAGCTGACAGCTCAGACATGCTTCTATGCAACATGTTTATACCAATGCTGTATTTTGGCAATCCTGGATGTGGCATTACTTAACTGT
GCTTTCATATTCAATAGTTCTGGCAAAGGGAGGCTGCAAGCTTGAGACAACAACTGCACAACCTGCAAGAAAGTCATCGGTATGGCTAGAAAACCCTGCA
AATTTTACAAATGTACAAGTCAGAAAATTTGCCTAATGTAAAAGGATCAAACTACAGGATTTGTCAGCATGAGCACAAGTGCCTAACTTCCGAGTTAGAT
AACTCTGGAGGAAGGAAATCCTTCCTACTGTTTGTTTCAAGAATATACAAAAATTCAAAACAACATTAGAATAGAACCAAAAGAAGAAACAATATTCACC
ATGTAGCAATACTCAATTCCTGGTTGTCTTATTTATAAAGGTATAAAACTAGGTTCTGACGAGACAATTATGAATCAACTACACCATGTTACAGATCATC
CATAACTCTTGGTGATGCAAATTAAGGGCTGTAAAGTGACAAGTATACCTCCACCAACTAATTTGTGCAGAGTGTATAAAATTGTAAAACAATGGATAAG
AATGAATTTAGTATACAAGATAGCATACAATGCCCAAGTTTAAGGAGACATACTGATTTACTGACTCCATAGGACAATCTTTTGCTTATGAGGACATCAG
TTTTCATAAACAATATCAATGTAAAAGGAGGTAACAGTTTATGAATTTCCTATGCCAAGCCATTTATAAGTCAAAAAATGTACCTATTTAGCTTTTATGC
ATTCCAATTAACTTCCAAAGAAGAAGAGTACATACTAATGGAAAACAAATATGATATTATCATCTGTTTTGCCCGGTAGTTTCTTGAGTCTCTGATTTGT
CCGCACTTCACTCCCAACCAACACAACATTAATACACATGGGCAGAGGAATATCAGGTTATCAGGTAAACTGAATACAGTTTGATTGACTGCAGTCAGTT
GATGGGACAAGATCTTTCTGGATTGAGCGTCAAGGAACTCCAAAATCTAGAAAATCAGCTAGAAATGAGCCTACGTTGCATCCGGACAAAAAAGGTAAGG
ATGCTAGCCATATTTCTGTTTTTCGTTACCAGATTGCCTCATCCAAGAATACAACTGATGATGACGTTTTTTACTTTCAGGACCAACTCTTGATTGATGA
AATTCACGAACTGAATCGAAAGGTTCTGGAATATTCTGTTATTACTCTGCAATGAGAGGTCTCTGAGTGGATTTCAAGAAGCTGACTCTACTGGCTCATT
TTCAGGGAAGTCTCATGCAACAAGACAAGATGGAACTATACAAAAAGGTCAACCTAATCCGTCAGGAAAACATTGATTTATACAAGAAGCTCTACGAGAA
AGAGGTGACAAGTGAAGTCAACCGAGATTCATCAACTCTAAACTTTGCAGTTGTCGAGAACGTCAACATTCCTGTTCATCTTGAACTTAACACTCCACCG
CAAGAAAATGATGTTGAGCAAACTGCACCTCCTAAACTGGGGTAACCCTGCTTTTTTCTGCAAAGGAACTATTGATTCTGTTCTTAATTCTTTAGTGGCA
TATTGTTTACTACTAACTGAAAACTTGCAGATTACAACTAAATCCATGAAGGCATGCAGCATGGCACCGCTTATGTTCATTATTCTCAACGGTACGATTC
AAAGCAACCAGAAACACTATTGGTTGTAAAGAGCTTAAACACATAGCAAGACTGACGCGGTATGCATGCCATGAGTGATTGATAATGGTAGTGTAAATCT
ATTTCAAATAAAATGTATTTTATTGAAGAATACAGCAAATGATTTGTATTATAACTATTTGACACATTCTGCAAGTTTGTTGACCAACGTGAGTACATGA
TAGCTACAAATCTTATATTGACAAAAAGATATGGTGTGTGGAAAAAATTCAGGTGCTATTTTTTTCTATTTTGAGAATTGTATGCATAGCAGAAGAGCAC
AAATTGCATTCTGGTTTATCCGTGCAGGCATTCCAGAGTATCAGAATGGGGATCAACCTTTCTTCAGCTACATATGTAAATATACGGATGCTAGTTGCTC
ACTTAAAATTTTGTAGTGTTCAAAGGAATAACAACAGTAAGACGTTGACGCACCGAAAATAACCAGAAATAACGGATTGTACTATGACAAGACACACGAC
AGATACACTAGCTTAAGTTTGTACAGGAAAATATTTGGCCTATTCTTATGTCACAGTAAATTAAGATTATCACCTTAATCACAGAAATTCAAGGGGTAAA
CGATTATAGCACAAAACGATCCTGGACCCCAAGACCTGGGTCTCTGAACGGCTGAACCTCCAATTATACCTCTATACATTTGGAATGTGTGTAGTCAAAC
TTTCGCTACTGGTATCTTTACAAATATTTACATTGAACACATCAAAATGATATACATGGATTCCTCTCGATATGTCTTTGAGGTTTGGGTTCTAAAAATG
ATATACAAATTATTAGGTTCTAAACTTAAATGATATATACACATCAAAATGATATACAAATTATTGGGTTCTAAACTTAAATGATGTATACACATCG
>Locus_11460_Transcript_22/26_Confidence_0.242_Length_743
ATATCAAAGTTTCATGATTGGAGCAGCATAGCCAGGATGCGCTGATTGGCATATGAAGAGCATGTACTCACCATGGTGCTTAGGGCAGTACAGGTAAATT
GCACTTGACAAAATAGTATCTGCTCAATTGCTGTGACATGTTTATTAACATGATATCACAACGTTTGAAGGGAAACTCAAGCTAAATAATCTCACTTCAA
AAAAGTTGCAATTCACGACTTCGGCCCGGACCCGAAATCTGTAAAAGAGCATTGCTCATCTTGTACCAGTGAAAAATAAACTTCAGATTGGTATGCTACA
AAGCTATTTGACAGATGCTTTTACAGCTTTCTCTGCCGCATCATCAAGATCCTCAGCAGTGATCAGTGTCATTCCACTTTCCTTAAGAATCCTCTTTCCT
TGGTCCACATTGGTGCCTTCTAACCGAACAACAACAGGAACCTTCAGGTCAACCTGTTTAGCAGCATTTACAATTCCACTTGCTATCACATCACATTTCA
TGATGCCCCCAAAAATGTTCACTAGAATTGCCTTCACTCTATCATCTGAAGTCAGTATCTTAAATGCTTCCACAACCTGTCCCTCTGATGCACTTCCACC
AACATCAAGGAAATTGGCAGGTGTTCCGCCATGCAACTTGATAATGTCCATGGTAGCCATAGCCAATCCTGCTCCGTTCACCATGCAACCAATCTCTCCA
TCAAGACCAATATAGTTCAAATCTGCTTTGGCAGCAGCAACCT
>Locus_14326_Transcript_11/49_Confidence_0.397_Length_2973
GGCCTCGCGGTGCCCCGTATTCTCATTTCCTCGCCGTATTTTCTCTCTCCAGCTCCAGAGTCCGGAGGAAGCAGAGAGAGAAACTCAACGGGAAGGTAGA
GAGAGGTACAATGCTCCTGATCCCTATTTGAAATCCTCGATTGGTCAGTTCTGTTCTTGGATACACTGTTGCTGCAGAATGGATGACTGTGCTTTCCTTT
CGGGGAGTGGCGTCGTGCTGTGTTGGTTCTTGCCTTCCTAATTAGTAATATGCCTTAGTCTTGCTCTACGGTTGGTTTGATTCACGTTTTTCCTGTAATC
TTTCTACCCCCAGGATTTTCCCCTTCGGTTTTCTTGGTTTGCTTCTCGGAAATTTGTGATTTTTAAGAGGATTTTTGGCTTGTGTTGCTCGATTTTGGTT
CTTGACCGTGCTTCCTTGGGATGAGCTTTTGCCTGGTTCGACATTACAAGATGTGTTATCCTAGAAGTTAAATTGATCTTCTATGAGCTGGGCGCTGCAT
TGTGGATTGCATGGGGTGGCATACTGTGGGCCGTGGAGTTGATGGAATCATGGAAATCCGCAGTCCCTTTTGTTGGATAAGGGTGGTTGAAGCTAATTTT
TTGCGAATTGTGGCGGTTTCTGGATATCCTGCTTGAATTAGTGGTGTGATAGATTGTTCTAGAGAATAGCTAGATATATGTTATATTGTTAGGACATCAA
GAGTGTTGAATCAGGTATGTTAATGTGTGAACTTGTTGATGGGAGCTAAATGGTTGCTGACGTTCCCTATAATGACCTAATCAAACCTAACAAGCACTAA
ATTAATGATAAACAGCACTGATCAGTAAGCCTACTGAAGTAAAAACCAATCACAATTCATACAGTATGCAACAGTATAAAGTAGTGAAAGAGTTCTTCAG
AGCTCGAACTTTTGGGATGAAAATAATTTGGCTCAGGATAGTCCACTGAAAGGAGTTTAGGATATCTGTTTGCGCTCTGCCTGCAATATATGAAAATCTG

AAGCTGATATGGTAGTAGAGTTGGGTACCCTGATTTCCGAGCCAAAAGAAGTTTAAGGTAGAATTTCTCAGGAATTATGTGGTAGTAAGAAGCAATTTAA
AAGGAACCTAATTAGGAAAAAAAGACATGCCTTGCTTGGTCATGAGAAAATATTATTGCTGTATAAAATAGGTATGCCACCTCCAGTCAGTACTTGATTA
ATTTCATTGAAAATGGAGGGCTGTAATAGATCCTTATCTATTAATGTGTTGTACCTAAACTTGGAAATCATCAGCTGAGGGCATTGTGCATGGTGCGTGG
AGTACTAGCCCTCGGGAGATGCTTCTTAATTAGGCAGCGAGAAGGATAGAGATCGGTTTGACATATGTTCCATTTGTAGCTGTACTGGATATTTCTGCAT
GTTCCCCTGGAGCAGTTAGTTTCAGAATTATGATATTTCTACTTGTGGCAGGATTGTTCTGTTTCTATTATCTGTTCATATTTATGCTAGTCTGTCTTTT
ATGTTGTAATATCTATTGTCTATTGCACTTCAAGTGACTATGTATAACCATATTCCAGTGATTCAGTGAATGATTATGAATTAAAGTAAGAGGACATTTA
CATCAATTATCCTTGTTCTTTCTCACGTCATTATCAATTCGGTTGATTAAATTCTGGCAACTTGCCATGATGGTTACTTTAGCTGATTAAATGCTACTCG
GAGCTAAGGATACCTTCTCGTAGTGCTCCCGCTTTTGTGAGAAATGGAGGATCATCCAGTCCATCCCATGTCGAAGTCCAACAATGGCTTCTTGTCAGGA
AATGGCTATCAGATGAAGCAATTTGGTCATAAAATCGACGATAGGGACTCGTCATCAGAGTCCGGTCGGTCTCACCAAGAAGCTTCCACAGCGAGTGAGA
ACAGTATAAATGAACAGCACACCTCAACACAATCTGACAATGATGATGGTCATGGGAAGCATAATGAGGACATGATGAAGTCAGCAGTGTCCTTGGGGGA
ACCAGGATCAGCCTTTTTGGCCCCAAAACTAGATTACAGCCCATCTTTTGCTTGTGTTCCTTATACTGCTGATGCTTTTTATGGTGGGGTCTTGACAGGA
TATGCTCCACATGCCATTGTTCATCCCCAGCAAAATGATTCAGCAAACTCTCGGGTTCCGTTGCCTGTTGAACCTGTAGCAGAAGAGCCAATATATGTTA
ACGCAAAGCAATACCGTGCAATCCTTAGGAGGAGGCAGATACGTGCTAAACAGGAGGCTGAGAATAAGCTGGTGAAAGGTCGGAAGCCATACCTCCATGA
ATCTCGACACCGCCACGCAATGAAGCGAGCCCGTGGATCAGGGGGGCGGTTCCTCAACTCAAAGCAGCTCCAGGAGCAAAACCAGCAGCATCAGGCATCA
GGTGGTTCAAGCTGCACAAATGCCATCGGCAATAACACAAGCTCCCAAAGCGGCCCCACTCACACGCCTTCGACACCTGCTTCCTCTGACACCGCAAGCG
CTTCGAGGGCCAACCAGGACCACATCTGCTTCCCCTCCGCTGGCTTCCGCCCCGCAATGAACATCAGCGATCATGGTGGAGGAGACACAAAGCTGTCTGC
GAACGGCATGCAGCAACGTGTTTCCTTCATCAGGTGAAGAAAAGTGGCACTGCCTGTGGCAACTCATCCTTGGCTTATGAAACTATGGATATGTAATGGG
CATGTAGGTTCGGTCTCAGAATAACCAAACATATGTGAGACCTTTTTAAGTTTCCTTTAGTTCAAAGAATGATAGGTTATGACCTTATGGCTTTGTTTCT
GGAGACTGAAATCCGTGCTTGATATACTATGTTGTACTGAGTTCTGAGGTGAAAGACATCGGGAGAGTATCAGGTGCAAATGGCACAAATGTTGAAAACG
GTGCAAATTCATCAAAATCTGTACCTAAAAGACTTCAATAAATTTTAAACTTTGCATAGCTCTAGGTGCATTT
>Locus_10929_Transcript_63/78_Confidence_0.186_Length_1483
TACCGCGGCGTGACGCTGCACAAGTGCGGCCGCTGGGAGGCGAGGATGGGCCAACTTCTTGGCAAGAAGTACATCTATCTTGGGCTCTTTGACAGCGAAG
TTGAAGCTGCAAGAGCATATGACAAGGCAGCCCTTCGCTTCAATGGGAGGGAAGCTGTTACTAACTTTGAGCCTTCCTCCTACAATCGAGGAGATGCTCT
ACCCGACACCGAAAAAGAGGGTATCATAATCAGCCTTACCGTCGATATTTTCATACAATTCTTCTTATCTTATATTTATCGCTGTGTTCTTAGCAATTGT
TGATGGCGATGCGGTTGATTTGGATTTGCGGATTTCACAACCTAATGTACACGACGCTAAAAGGGATAATACCTTAGCTGGCTTCCAGCTAACATGCGAC
TCCCCTGAATCTTCAAATATGGTGGCCTCTCATGTAAACTTTCTTCACGATTACACCCCATTTAAATGCTCGGTTCCATTTTTTATGACTTACTACTGTT
AGTAGTAGCTAAAAAGGTACCCGGAAAAATAAAATCTTAAGATGGAAGCAATCTAGGTTGAATTGTCTTGTATCTCTGTGCGGAATCCTTGACCTTTGTT
AGTTCCTAAGCTATAAAGATTCATAGGAGCATTTGATTGGCGTGTCGTTGGTTGAGATATTAACTATTAGAGTGTCCCCAATGAGAGTTAGCTTATACTA
GCTCTAAACATATTCATGTTATTTCATATATTGCACTAAAGTTTAGATTTTAGTAACTATCTTTTAGCTTTTTTTTAAATTTAATACTTACGTGAGTATA
TACATCTCGTTTAGTGCTATAGTTTAACTCTTCACTAAGAGTTGCTAAGTTTTTTATATTTAATCTCCTTTACATAAAGTAAATGATGACATAGACATGT
TTAAAGCTAGCTAAGGAGGCTTCTTCGGGGCTCTTAGTTCAGGTCTTATAACCTTTATTGAACTTACGACATTTGCACCTATCTGTTGCTGCTGTAGCCA
ATGAGTTCATCGTCCCCGTGGCCTGTGTATCAACAGAGCACGGCAGTACCACCTCACCATCAGCGTTTGTACTCATCTGCTTGTCCTGGCTTCTTTCCGA
ACCTCCAGGAAAGGCCAGTGGAGCGAAGGCCTGATTTGGGCCCCCAGTCGTTCCCCACCTGGGGACGGCAAACGCAGGGCTCCCCTCACATGCCGTTGCA
CCACTCCGCAGCATCATCAGGATTCTCTACCGCCGCGAACGCCGGCGTCCCATTGCCGTCTCACCCGCCGGCGCCGTTCTCGGACCACCAGTTCTACCTC
CCACCGACGGCCTGAGCTTGTTGGTCCGGTTCAACTCTTGGGACAGCAGCTGACGCTCTGTGTGTGAACGTCTCGCAGCTTGCCGGTAACAGCTGTTAAT
TAATGAGAGAGAGAGAGAGAGAGAGAGAGAGAGAGAGAGAGAGATAGCGCGGGAAGGAGAGAGAGAGAGAGAGAGAGAGAGTCGT
>Locus_7001_Transcript_65/84_Confidence_0.074_Length_1482
TTTTGATAAATAATAATAAATAATAAAATTACAATACTATTTATCAAAACTCAATTTACTCTTTCTTCGTGATTTTAGGGGGTAAAGGCATAAGAATCGG
ATCCGTTGAGTGCGGATTTGGCTGCAGGGGAGGGGCCGCTTCGTCATACTTCAGCTGTAGCGTCATTAAACCTTTTCCGGACACGAGAGCGAGCGAGCGA
GAGAGAGAGGTGGACTGGATTTGGGGGAGGACGGAGAGGAAGAAGCAAAATGTTTATGGAGCTTGAGTGATGCATCTGGTAACGAAAATGCTTCTGCGTG
AAATGGATGGTTATCCAGTCCATTCCATGCCCAACTATGATTTCCTGTCTGGGAATGGTTATTCAATGAAGCAGTTTATCCTTGGCAATCCTGATGGAGA
CTCCTCATCAACAAAGTCTGAGCAGTCTTGCCAAGACTTATCTGCAGTGAGTGACAGCAGCCTCAATGGACATCACACACCAACACAATCTGACGACAAT
GATAGTTGTGGGAAGCGAGACCAGGGCATGGTAAAGTCTGTACTGTCCTTGGGGAACCCAGAAGCTACATTTCCCCCTCCAAAGATTGATTACAGTCAGC
CTTTCGCTTGTGTTTCTTATCCCTATGCTGCTGATACATATTATGGTGGGGTCTTAACAGGATATACATCAAGTGCCATTGTTCATCCCCAAATTAATAG
TACAGCAAATTGTAGGGTCCCATTGCCTATTGAACCTGCAGCTGAAGAGCCCGTTTTTGTCAACGCAAAGCAATACCATGCAATCCTCAGGAGAAGGCAA
ATGCGTGCAAAATTGGAGGCCCAAAATAAACTGGTGAAAGGCCGGAAGCCATACCTTCACGAATCTCGACATCGTCATGCAATGAAGCGAGCCCGTGGGT
CGGGAGGCCGGTTCCTTACCAAAAAGGAGCTCCAGGAGCAGCAGCAGAAGAATACACTGCCTTCACTTCAGACTCCAACATGTGGAGTCAGCAAAATGTC
ACTCGGCAGGAATCAATGCCCTGAAAACAGCACATCTCGCTCGCCTTCTACACCGACAAACTCTGGGATCTCAAGTGTTTCAAACGGAGGTGGCATGCTG
GTTCATCAAGAGCACATCACCTTTACATCTAATTTCCTTCCCAGCATGAACTTCAGCTACTAGAATGGAGGTGAAAAGATGGCTGTCAATGGTGCGCACC
ACCGCGCCCCCATCGTGAGGTGAACAGCCTCGATTGTGGGGCTTTGGTATCTATCATCACTGGCGCGCTCTGGTGTGCCCGTGGCAATTCATCCTTGGCT
TATGAACTATCTGTATCTGGTAATAATTTGCTTTCAGTTTAATATGTAGAGCTTGTCCGAGATGAATTCATTATTTGTAGCAACTAAAAAGAGGAGCTAC
TTGTTGATATTTGTGGATACAGAAATATCCTGGTGTTTCTTGATGTACCAAGTACACTCGACTGGAAAAAACGTTTAATAAT
>Locus_12915_Transcript_14/30_Confidence_0.412_Length_3438
GCTCCTTGAGAAACACCATCCAATTACAAGACTATTTGATGCAACAATCACGCATATGGCTGCGCTAGAACTACTGCTCTGAATTAAGAGGCGTTGAAAT
ATTGTCCGGTCTACATATAGGCACTTGAGGTGTATTGACAGACAGTTCCCCTTAGTTTGCTACAACACATCAGGCGAATTCCACCAAATTAGGATGATGT
TTGTTTCTTAGACACAGGATAGCAATGCCAAAAGTTTTTTATAATTTTCCGCTTCACCGCAGATTTGGCATCTCAAAGGGAGTTGTGGCAAGATTCTCTT
GACCAAGCTTTCTTGCAGATTTGGCAAGAAAGATAAAAAAAATGTTGGTATACTTAAGCATCCTTAATAGAAACTAAACAGGTCTTAGATTGAAATTATC
ATAAATAAACCCTTTTTCACTTTAGCTGTAGTCCGGATGAAATCTTAGGGGCCTTGCTTAGCCTGCCCTGAAGCTTGCCAGTAAGTCGAAGCTGATGCTG
ACACAGCACTGACATAGATCCCAATGAACACAAGTTATTTCGAGAAGATAAAATACCAAGCAAAGCCAGGATTGCATTAAAAGATAGTGTATTTCTACTA
TACGATCTGAAAGCTTAATTTATTATATCAATAATCAAATTGGCAGCAGTGTGGAACAGATATAAAGAAGATGCTTCAAATCACAAATAACTACAGATAA
ATTGAAGGGTGAAGGTTCATTCAACCACCAAACACCCAAAATGTCGAGCTGCATATGCACAGAACCTAAGTACGGTACAGTTCATAGTTTGGGAAAATAG
ACTGCAAATGTGCACAGATAATACAGATTGTTACTCGATGAACAGTAAGCACGGTATTTGAAATCCTTCTAACCATCCATCCAGGCAGTGCCTCGTATAC
TCAAACCTCTTTTTGGAGGAAGCCAAACCATGTGATCCTTTTCAACCCAAATACATATATTCAAGATGAAACTAACAAAAAGACAGTTGATAAAGAAACA
CGAAAGAGAGTAGAAAAAGGGTACCTGTTTCAAGTTCTCATCTTCTGAAAATGATCAGCAGTCAATAGATGTTTTAGTCCATTCTCCAGAAAAATGAGTG
CCGCATGAATCCTTTCCGGTTTATAGTGAAACATTTTAGACGATGACCTTTAACATTCTAGAAAACGAAGAGAAAACCTACAGTCATCTAATTTCAGCTT
AAACATGTTCCAGCAAGATCATCACCATCCTGACATGTTCCGTTTTGTCCCTAGACCCGAGCACAAGGTATTGCGAGGCTAATTTAGCTCAATTCAGTTG
TCAGAGCAATTGTGTGTGAAAAGTGTGAACAACAGCATAGGAGACAATACAGCAAGTGTAGCGTGCCAATATGTGTCCAGCTTGATAGTTCACTATAATA
AAGTTCTTTAATTCAAGTATTTGAATAATATAGAATCTCAACCTTCAAGTTTAAAAGGTTTTGTATTTGCTATTATAATCGTTGCCACCCCCTCACCCCT
CTCAAAAAGAGAGTTACTTTGTAGCGCACTGTAAATCTCTTGATTGAAATAGTGGCATGTCATCTTATTTAATTCAGTTATTTCATAAGGTACAATTTGT
TTAGCTGACAGCTCAGACATGCTTCTATGCAACATGTTTATACCAATGCTGTATTTTGGCAATCCTGGATGTGGCATTACTTAACTGTGCTTTCATATTC
AATAGTTCTGGCAAAGGGAGGCTGCAAGCTTGAGACAACAACTGCACAACCTGCAAGAAAGTCATCGTCAGTTGATGGGACAAGATCTTTCTGGATTGAG
CGTCAAGGAACTCCAAAATCTAGAAAATCAGCTAGAAATGAGCCTACGTTGCATCCGGACAAAAAAGGTAAGGATGCTAGCCATATTTCTGTTTTTCGTT
ACCAGATTGCCTCATCCAAGAATACAACTGATGATGACGTTTTTTACTTTCAGGACCAACTCTTGATTGATGAAATTCACGAACTGAATCGAAAGGTTCT
GGAATATTCTGTTATTACTCTGCAATGAGAGGTCTCTGAGTGGATTTCAAGAAGCTGACTCTACTGGCTCATTTTCAGGGAAGTCTCATGCAACAAGACA
AGATGGAACTATACAAAAAGGTCAACCTAATCCGTCAGGAAAACATTGATTTATACAAGAAGGTATTTGATAAATCGTGAATGAAAATTCACAGGATATA
CACCCCATCCATTATTGACTGAGGTTTATTTCTCTACTCAGCTCTACGAGAAAGAGGTGACAAGTGAAGTCAACCGAGATTCATCAACTCTAAACTTTGC
AGTTGTCGAGAACGTCAACATTCCTGTTCATCTTGAACTTAACACTCCACCGCAAGAAAATGATGTTGAGCAAACTGCACCTCCTAAACTGGGGTAACCC
TGCTTTTTTCTGCAAAGGAACTATTGATTCTGTTCTTAATTCTTTAGTGGCATATTGTTTACTACTAACTGAAAACTTGCAGATTACAACTAAATCCATG

AAGGCATGCAGCATGGCACCGCTTATGTTCATTATTCTCAACGGTACGATTCAAAGCAACCAGAAACACTATTGGTTGTAAAGAGCTTAAACACATAGCA
AGACTGACGCGGTATGCATGCCATGAGTGATTGATAATGGTAGTGTAAATCTATTTCAAATAAAATGTATTTTATTGAAGAATACAGCAAATGATTTGTA
TTATAACTATTTGACACATTCTGCAAGTTTGTTGACCAACGTGAGTACATGATAGCTACAAATCTTATATTGACAAAAAGATATGGTGTGTGGAAAAAAT
TCAGGTGCTATTTTTTTCTATTTTGAGAATTGTATGCATAGCAGAAGAGCACAAATTGCATTCTGGTTTATCCGTGCAGGCATTCCAGAGTATCAGAATG
GGGATCAACCTTTCTTCAGCTACATATGTAAATATACGGATGCTAGTTGCTCACTTAAAATTTTGTAGTGTTCAAAGGAATAACAACAGTAAGACGTTGA
CGCACCGAAAATAACCAGAAATAACGGATTGTACTATGACAAGACACACGACAGATACACTAGCTTAAGTTTGTACAGGAAAATATTTGGCCTATTCTTA
TGTCACAGTAAATTAAGATTATCACCTTAATCACAGAAATTCAAGGGGTAAACGATTATAGCACAAAACGATCCTGGACCCCAAGACCTGGGTCTCTGAA
CGGCTGAACCTCCAATTATACCTCTATACATTTGGAATGTGTGTAGTCAAACTTTCGCTACTGGTATCTTTACAAATATTTACATTGAACACATCAAAAT
GATATACATGGATTCCTCTCGATATGTCTTTGAGGTTTGGGTTCTAAAAATGATATACAAATTATTAGGTTCTAAACTTAAATGATATATACACATCAAA
ATGATATACAAATTATTGGGTTCTAAACTTAAATGATG
>Locus_12535_Transcript_31/73_Confidence_0.274_Length_3022
AATTTTTTAAAAACTGAGTAATAGCTATTAAAAACTAGCATAAAACTAATTTGTTTAGATCCCACCCACTTTTCGAAAGTCAGTGCAAAAAAACTGAAAG
GATCCGAGCAGGCCTTAGAAGAATTTCAGCAAGACAAAGATGCTTCAGAAAAGGAGGTGAGATTCCTGATTTCACATAAGTCAGTTCAGAAAAACTGAAA
GGATCCGAGCAGGCCTTAGAAGAATTTCAGCGAGACAAAGATGCTTCAGAAAAGGAGGCTTGATGTGGTATATGTTTTCCCCTTCCCCGACCTTTTGATC
TCCAACCATGGCAGTGGAACAAGGAGCAAATTTGCAATCTTGGGGTAGTTCCGGTGGTGAAACGTGAAAGATTGGGGATTTAAGGGACGTGAAACTGAGG
GGGTGGTTGTCATGATGTGTAGGGTCCAGCACTGGAGTGGTTGATGCTGTGGTTTCCAGGTTAAGGGGAGAAGAGGAATAGGATATAGATCTCAGGAAGC
CAGAGATGAGGTCGAGGGGAAGCCAGTGGCCAAGCTTGTGCTCCAGTGAAGCTGATTCTCTTGTTCCCAGTTGATCCATGCGCCGTGGCCTCAGTTCGCA
GGATCAGCAGCTCCATGGTTCTGTGTGGTGCTGCAGTTTGAGAAAGTGGTTTTCCAGGGGGAGGAATCTCTTGTGGATTCCTTTTTCTGGGTGCAACGAA
GTGGGAGGTAGGAGCAGCAGTTGAGATTTTCAATTCTGGCATGGGTTCTTTTGGGATGGACTGGAACCAGAAAGGCTCGGTGTTGTGGGATTGGGAGAAT
TTGCCGCCGATAGGCAGAAATGCAAACGAGAACCTCAAGATTGCGCCACAGGCTGAACCGAAGTTTGCAGGTGTTGAGGCAACAAGGCATGAATCGGGGC
ATTCTTCTTGTGGTACTTTCTCTTCCAGCTCGGAGATGGGGTATGGTTCATCCAAGAGTTCCATATCAGCGTCGATTGATTCTTCACCCAAGGTGGGAAA
CAACGTGGAGCTCAATCTTGCAGCTGTCAAAGTGCCTGACAAAAACACCGTCAAGAACACTGATTTGGGTAAAGTTGATGACGCTGGAACTTCTCCATCA
TCGATGATAGCCGTCAGCAGTGGAGAACCGGTGATTGGCCTGAAACTTGGCCAAAGAACCTATTTTGAAGATGCATGCGGAGGGCAAAGTGTCAAGAGTT
CACCATCCAATGTGAGTGCAGTGACCCCTGCTTCTGCGAAGAAAGCAAAGATGATTCAAAACGCACAGAACTCGTACTGTCAGGTTGAAGGTTGCAAGTT
TGATCTCTCTTCTGCTAAAGATTATCATCGGAAGCACCGAGTCTGTGAAACTCATTCTAAGGCTCCCAAGGTTATTGTTGCTGGTCTGGAGCGACGCTTT
TGTCAGCAGTGTAGCCGGTTCCATGCTTTAACTGAGTTCGACCAGAAAAAACGAAGCTGCCGGAGACGCCTCAATGATCACAATGCCCGCAGACGGAAGC
CACAGCCTGAAGCAATTCCTTTCGGTTCATCAAGGCTCTCTGCAATGTTTTATGGTATCGCTAAGCCTTTGGTTGCCTAATGGTTTTGGGAAACTGGTGC
ATTAATTATTGTTCTTTTAGCAACTGTTCTGTTTAAAAATCTTGCATTTTTCATGTATGCCCAGAACTAAACGGTGGTAAAATTTATGAGTACAAAGTGC
AATGTTGTGTGTCTGAAGTTGTCCCTTATTTGTTTATTCATAACTCAGTGCCATGATTTTTGAGATGTCATGAATTTACATACTTCTGATATTAGATGAC
ATGATAACTTTGGCAATACCGGTAATTAATGAAACTCCATCTGAGTACTGCCATTTGTGATGATTACTATAGATGGGACAAAAGAGATATCAATTAAGTT
GTATTTATTTTATGAATAGGTGCATAATGCTATATTCTTTTCGATTTAGATATCCATGACATGATTCTACTTTATCTGCCATGATGCCATCTCTCCATTA
TAGGAAGTCGGGAGACAATAGGAAGCTGTTAAGTATCTGAATAAACATCAGTTCAAATATCTACAGATGCATGTTGAATCCACTATATTATCAATAATTT
GTACGAAAGTAGCTTTAGCTTTTTGTATTAAGCAAATCGTTTGTCCTCTTTCTGAATCTGTTTGGCTTTAGTGATGAAAAATTGTCGTGGCGGTCTTAAA
CAGATGCAAGGCAACAGACAAGTCTTCTGTTTGGTCAAGCTCCTTATGGTCAAATGAGAAGCTGTGCAAACTCTTCATGGGATAGCCCAGTAGGAGGCTT
CAAATTTGGAGAAACAAAAGCTCCTTGGTTAAAGCCAACGAGAGCTGCAGGTGTTGATGGGATGCATGTATCAAGCCAGCAGGTGTGGAACAATATTACG
CCACACGGTGCACATCATGATTTTAATGGTATCATGGCTTTCAAGGGAACCAGTGCAAATGTCCTTAATCAAGGTGCCGAAGCTTCTCCGGTCGTCTCCA
ACTCGAATGGAGCCCCAGATCTTCAGCGTGCTCTCTCTCTTCTGTCAAACAATTCAGCTGGTGCTGCCAACGACCAGCCAACTCCTCAGCTGCACCCTGG
GCTGACCACCTTCGTCGGCTCCTCCAACCCTGTCACTGTGATGGAAGCCTCACCACCAGGACTCTGGCAAGACAGCACGCCCCTTGATCATCAGGCGCAG
TTCCAGGCTTTCGATCCCATTGCCGCACCGCAACAGCTCCAGCTCCCAAAACCCCCTTCCTTCAACAGCTCCACTTCCAGCTATGGCCAGATGCAGTGAT
GGATGCTTCCTTCAGTTCTGCTGTCTGAAACTCTGAAATTTGAAACTGCTGTGTTCTAAAAAATAAAGAAAAGCCTGTGTGCGAAAACTCAGGCTTGAAA
AATTGATCCAGTAGTGTTCCCGTTCAATGGCTTGAATAATCCATCCTTGTGATGGCGTGTAACTCAAATGCTTGGATTGCGCTGGCAAGTGATCATGGAC
TCTTGTGTTCTTGATGGCATCT
>Locus_15085_Transcript_15/40_Confidence_0.302_Length_1564
CGACTAGTAACCAGATGTCGCAACCTCAAGACTCTCAAGCTCAACAATGCTATCCCTCTTGACAAGCTTGCTAGCCTCCTTCGTAAGGCTCCTCAAATTG
TGGAGCTTGGAACAGGAAGGTTCTCTGCTGACTACCACCCAGATCTCTTTGCAAAGCTTGAAGCAGCGTTTGCTGGTTGTAAAAGCCTACAAAGGCTTTC
TGGGGCTTGGGATTCTGTTCCAGAGTATCTGCCAGCATTTTATTGTGTATGTGAGGGCCTCACATCACTTAATCTGAGTTATGCTACTGTGCGAGGCCCT
GAGCTGATAAAATTTATTAGCAGATGCAAAAACTTGCAACTATTATGGGTGATGGACTTAATTGAGGACCACGGTCTAGCTGTTGTGGCATCATCTTGCA
ATAAACTACAGGAGTTGCGGGTCTTCCCTTCTGCCCCTTTTGATCCAGCTGAGCAAGTTTCATTGACTGAAAGGGGACTTGTTGATGTTTCTGCCAGTTG
CCCGAATGTTGGAGTCAGTCCTCTACTTCTGCAGACGGATGACTAATGAGGCCCTTATTACCATAGCAAAGAACCGGCCCAACTTCACCTGCTTTCGCTT
ATGCATCATCGAGCCTCACACTCCAGATTACATCACGCATCAGCCTCTTGATGCAGGTTTCAGTGCCATTGTGGAATCATGCAAAGGCCTCAGGCGCCTA
TCTGTCTCAGGCCTTCTCACAGATCGTGTATTTAAATCCATTGGGGCACATGCTGATCGTCTTGAGATGCTCTCAATCGCCTTTGCTGGGAATAGCGGAT
TGGGCCTTCATTACATCCTTTCGGGCTGCAAGAGCTTGAAGAAGCTGGAGATCAGGGACTGCCCATTTGGTGATAAGCCCTTGCTGGCAAATGCTGCCAA
GCTGGAGACAATGCGATCCCTTTGGATGTCGTCGTGCTCACTGACCCTGGGCGCATGCCGACAGCTTGCACGCAAGATGCCCCGCCTTAGTGTGGAGGTC
ATGAATGATCCTCGACGGGCATGCCCCTTGGATTCACTTACAGATGAAAGCCATGTCGAGACATTGTATGTCTACCGGACAATCGCAGGTCCAAGGTCCG
ACACACCAGCCTGTGTCCAGATTATTTAGGGGGACCACCTGTGGTATGGGATAGGTACTATGGAAGTGTTCCTTGTCAATCCCGATTTGTACCTTCAACG
CCTTCCTACCGTAACCTCTGCTCAGCTGTGAAACTGTTTGCGAGTTTAGGACGAGACTCAGGGACCCTTTTCAGGTGTCAATAACTGTAGGTGGAGACTC
GATGAGTTACAGAAACTACTAGATCTACTAGAGAGCGTCTTGAGAAAACTTGGGTTTGTTTTCTTAGCGTGATTAGTTCTTTTCATGTATAAAAAATATG
AGATCATTTCAATCTCGCAATGTACTGTACTATTGGAACAAATTGTACCAGGAGCCTCTACTTTTTTGACCTCCCCTCTCAAATTACGGGTTGTTGCCTC
AACTTGTTGTGAATGGGGAATAATTCAAGCTTATCTGTGTCTCTTTATTCATGAGGTATTTATC
>comp79901_c0_seq23
GACGCGGCGCGGGCCGAGGTCCCGGAGCTCGCAGTACAGGGGCGTCACCTTCTACAGGAGGACGGGCCGGTGGGAGTCGCACATCTGGGATTGTGGGAAG
CAAGTCTACTTAGGTGGTTTCGACACTGCTCACGCGGCTGCGAGGGCTTATGATCGTGCAGCGATCAAGTTCCGAGGGCTCGACGCGGACATCAACTTCA
CTTTGAGCGACTATGAGGATGACTTGAAGCAGATGAGCAATTGGACAAAGGAAGAGTTCGTGCACATACTCCGCCGCCAGAGCACGGGGTTCGCGAGGGG
GAGCTCCAAGTACCGCGGCGTGACGCTGCACAAGTGCGGCCGCTGGGAGGCGAGGATGGGTCAACTTCTTGGCAAGAAGTACATCTATCTTGGGCTCTTT
GACAGCGAAGTTGAAGCTGCAAGAGCATATGACAGGGCAGCCCTTCGCTTCAATGGGGGGGAAGCTGTTACTAATTTTGGGCCTAGCTCCTACAATGGAG
GAGATGCTCTACCCGACACCGAAAATGAGGGTATCACAACCAGCCTCGCCATTTATTTCTTTTCATATAAGCAAATGTATCTCAGGTGCACCTTATAATC
ATCGCTGTGTTCTTAGCAACTGTTGATGGTGACGCGGTTGATTTGGATTTGCGGATTTCACAACCTAATGTGCACGACACTAAAAGGACAATACCTTAG
CTGGAGGCCAGCTAACATGCGACTCCCTTGAATCTTCAAACACGATGGCCTCTCAGGTAAACTGACTTCAAGACTACACAGCAGGGAAAAATGCTCGGTT
TCTCTATGACTTTCTACTACTTGCAGTAACTAAAAAAGTTTCCCAGAAAAATAAAATCTTTAAGATGGAAGCAACTAGGTTGAATTGTCTTGTATCTTTG
CGTGGAATCCAACCTTTGATAGTCCCTACGCTATAAAGATTCATAGGAGTATTTGATTGGTGTGTCGTCGGTTGAGATATTAACTAATTGGTCCAGGTCC
TATAACCTTTATTGAACTTACGATATTTGCACCTATCTGTTGCTCCTGTAGCCAATGAGCTCATCGTACCCGTGGCCTGTGTATCACCAAAGCACGACAG
CAGTACCACCTCACCATCAACGCTTGTACTCATCTGCTTGTCCTGGCTTCTTTTCGAACCTCCAGGTATTACCAAGCTAAAGCCTATTTTTTATGTGCAA
ATTGCTACTCCTACGTGATCAATTAGTTCAAACTGTTATTCGAATCCAGGATTGTTTACTGAATATGTCCTGTCTTCAGAAAAAATAACACAAAAAGGAA
CATAATATGTCCTGTCTAATTGTGGACTGATACTAGCCAATTAAACAGTTTCCCATATCCATTTCCTGTGTTTATTGTTTTGATTTGCTGGCATGGTTAA
ACTATAGTAGCTTGATTCGATAAAAACACTAAGCAGGCTTTGATATTTTGAATGAAATGTCGTTCTTGGAAATACAGCCTTTTCCTTGGTATATCAAGGA
CAGTTGTCCATCTGCACTCTGCACCCTCAGCTTGTGTAACTGTTTGCCTTGTGAACTGATTGATGTGTCAATCAGGCAGTTCTGCCAGTTTGCATCTTTT
GAAATAGTGCAGTCTTCCAGTCTAACAGCTCCATGACATAACTTGGGATGCATGCTGCAGCTTTCTTACTGAGGATGCTCTAGATTTACAGAAATTCTCG
CAGTTAGCAGAGGGCTATCAAATCTGATTTGCTTAGCTTCCTGCAGACAATAAGATAAGAACTCTAGAATCCTGAAGAAATCTTACTTGTACATGTTTAC

CAAAGTATCCTTACTCCTTAGTTGAGCCAATTTTGGTGAAGAACCGATAGCTCATTACTCTGTTTCCTGGGAATGATGTGGGCCTAAACCATTCTGTGGT
CAAAGTTTAACAAAGCCCATGCTTGTGCTGTTGCAACTGCAGGAAAGGCCAATGGAGCGAAGGCCTGAGTTGGGTACCCAGTCATTCCCCACCTGGGGAT
GGCAAATGCAGGGCTCCCCTCACATGCCGTTGCATCACTCTGCAGCATCATCAGGATTCTCTACCGCCGTCGGCGCAAACGCCGGCGTCTCGTTGCCTTC
TCACCCGCCGGTGCCGTTCCCGGACCACCAGTTCTACTTCCCACCGACGGCATGAGCTTGTTGGTCTGGTTCAACTCTTGGGACGGCAGCTGATCTTATG
TGTGAACATTTCGCAGCTTGCCGGTGACCGTTGTTAATTAATCGGGGAGAGAGAGCCAGAAGACCCAGTATGGCTATCTCTCTGTGGCCCGTGCCTTGTT
ATGATCTTAGTTATTGCTACTACACCGTGGATTCATCATGGTTTATGAGATTCTTTACTCAAGTTTCAGCTTAAATGTCCAAATACATGAATGCATCATA
CACAGGATCCGTCATTTTCTAAAAAAATACAGGATCTGTGTGAAGTGT
>comp78295_c1_seq38
ACAACTGCACAACTTGCAAGAAAGCCACAAGCAACTAATGGGTGAGAAGCTTTCTGGCCTAGGTGTGAGAGACCTCCAGAGTTTAGAGAATCGTCTTGAA
ATGAGCCTACGTAGTATCAGAACGAGGAAGGTCATATTCATTTTGTTTCCACGTTTTGTTTTTCTATTCACTCATCTTAATATGCATAAACATCCATCAA
ACAGGACAATCTTTTGAAAAGTGAAATTGAAGAGTTGCACAGGAAGGGTAGCCTAATTCACCAGGAAAACTTGGAACTCTGTAGAAGAGTAAATACCATG
TCACAACAAAATATGGATCTACAAAGAAAGGCGAGTGAACCAAGAGGTGTTGCCGATGCAAATAAAAGCTCTAGCACTCCCTACAGATCTTGTAGTGCAC
AAGATGCAGATATCACGGTTAATCTTGAATTGAGCCAATCACAGCAAAAAGAGGGGGAGCAGTGCCAAAGAGGGGCTCCAGAACTGGGGTAAATATTCTT
TTTCCAGAATCCAAAAACTGCATTACTCCAAGAAGTATCAAGAAGCCTAATGAAGAACCCATGCTTTGACATGTAACAGGCTTCAGCTGCACAAAGAAGA
TGCACTACAAGCTGGAGAGTATCATGATGTTCCACCAATTCTAACATGCAATGCGGAGCACTTAAACTACAATTAGTATTTTGTGCAACATGTTAGATGA
GTGAACAAAATGGTTTATGTACAGACTACAGATATGACCATCTATCTTATCTGTGGCTCTACTGTACTGAAATTGCAAGAAATGGTTATTGTAATGACTA
ATATGATACATTGACTGTCATACCGTTGACCTTGAAGATAGTTCTATAACGGAAAGGTCCAATTTGAAACAAGTGTTACGGATGAGCTAGTAGGATTTTT
TTCACAAACAATTAGATAGTCCACGTTAGTGGTAGCTGCTGGGTATGACATTTGACCCAGCCTGCCGACCCTCCCTTTCCCATGGGAGTTCAATTAACAT
TAGGAATGAATATCGATATTATGGTTGATTATTTGAATCGTATGTTTTCCACCCTTAGATGTAAGGCCCTATTTTACTGTTTTAATGGATTAACAAGGTG
AGCTTTCGAGATGGTAGCTCATTGATTCGCATGCATTTTAGTGGCTGTTAAGAGGTACTTAGTGCATCCATGCTTGAAGGTGTTGTTAGTTCGACAAACC
ATTTTCTATCCTACTGGATTACTATCTTTTGGTCAGTATGACAAGCAAGAATTTAATCTGCCAATTAAGAATTTAATGTGTTCTTTTGACATGAATGTCA
ACAGCATTTAAGTAACTCAACTTTTAATACAAAGTACTGGAATGTTTTCTCACCGAAAGTAAAGATGTGGTTCCTTGATAGCAAACAATGTTTATGCCAA
TATCGCATTAAGGTGGTTGTATCCTTTAGAATGATCTCCTCATTCGTGCTTAATTTGATGCTATTGTCTTTTTACGGATGCCTAGATGTGGTTTTGACCA
TTACTAAGTACTTTAATATATATTTAACAAAAATAATGACATAGCTGTTGTATGGCCAATCTAGATTTTGTTTTCGAAACATATTTGCAGTCAAACTATA
GGAAGTAAGAAACTTCAATGTGGTTCTCGAAAACCATGCTTAAAGTTTAGAAAGTTTGACTACATAGAGTATATACTACATAGTCTTAAAGCATCAAATG
AAGAGGAATGGAGGGAGTAGCAGACAATCATCATTCAATATCTATTTCTTGGTTGCCTTCAAATTTGGGCTTACTAAAATCGATTTAAAATTCTCGTGTT
CCAGTTGAAGCTCACTTCAAGTAAATCAGGAATTACCATATTTTTCATTGCTCATAATTTGGAAACATGGCATGATATTTGATAGAAAATGTACAAATCC
TCAGCTGCTCAGAAAGTTACAAAGGGAGGAACGCCCATGAAAGAGAATTCATGGCTGCAACAACGAACATAAACACAGCCTATTCTGTCCAGCAGTCATG
GTAATACAAGCTAAAAAGGACCGCTCTGGAACAATAGCTTGAAAAGCAATATTAATACAATTTCAGATGAACTATGTGCGTCGATGTCTTACTTGTGACT
TCCTTGCAAGTAATGCGGTTCCTGCCTCATGCTTGGTGCCGCCGCATGCAATAAACCGATAACTAGTGAAGCAAGAGGATAACAGATGTCAGAAAACTGA
GCAAGCCATGTTAACTGTTTGAGAATTGTATACGGCTATGAGAAGTATATTTTTTTAGGGGGTGTTTCGATCCCTTTAGCTAATTTTTTAGCTCTATATT
TGGATGTTAAATAGGAGGACTGAACATGGGCTAATTATAGACTAATTGCAATTAGTGATTAGAGCCCATAAGCTCTAATTAGCCTATGATTAGCCCATGT
TTAGCCCATTAGCACATGAGCTAATCATAAGCTAATTAGGGTTAATTAGCTCTAATCATCAATTAGCTAAAGCTAACTATAATTAGTTCACAATTAGTCC
ATATTTAGTCCCCCTAATTGGGGTCCAAACATGGGGCTAAAAATTAG
>comp80692_c0_seq9
GCTCCTTGAGAAACACCATCCAATTACAAGACTATTTGATGCAACAATCACGCATATGGCTGCGCTAGAACTACTGCTCTGAATTAAGAGGCGTTGAAAT
ATTGTCCGGTCTACATATAGGCACTTGAGGTGTATTGACAGACAGTTCCCCTTAGTTTGCTACAACACATCAGGCGAATTCCACCAAATTAGGATGATGT
TTGTTTCTTAGACACAGGATAGCAATGCCAAAAGTTTTTTATAATTTTCCGCTTCACCGCAGATTTGGCATCTCAAAGGGAGTTGTGGCAAGATTCTCTT
GACCAAGCTTTCTTGCAGATTTGGCAAGAAAGATAAAAAAAATGTTGGTATACTTAAGCATCCTTAATAGAAACTAAACAGGTCTTAGATTGAAATTATC
ATAAATAAACCCTTTTTCACTTTAGCTGTAGTCCGGATGAAATCTTAGGGGCCTTGCTTAGCCTGCCCTGAAGCTTGCCAGTAAGTCGAAGCTGATGCTG
ACACAGCACTGTTTCAAGTTCTCATCTTCTGAAAATGATCAGCAGTCAATAGATGTTTTAGTCCATTCTCCAGAAAAATGAGTGCCGCATGAATCCTTTC
CGGTTTATAGTGAAACATTTTAGACGATGACCTTTAACATTCTAGAAAACGAAGAGAAAACCTACAGTCATCTAATTTCAGCTTAAACATGTTCCAGCAA
GATCATCACCATCCTGACATGTTCCGTTTTGTCCCTAGACCCGAGCACAAGGTATTGCGAGGCTAATTTAGCTCAATTCAGTTGTCAGAGCAATTGTGTG
TGAAAAGTGTGAACAACAGCATAGGAGACAATACAGCAAGTGTAGCGTGCCAATATGTGTCCAGCTTGATAGTTCACTATAATAAAGTTCTTTAATTCAA
GTATTTGAATAATATAGAATCTCAACCTTCAAGTTTAAAAGGTTTTGTATTTGCTATTATAATCGTTGCCACCCCCTCACCCCTCTCAAAAAGAGAGTTA
CTTTGTAGCGCACTGTAAATCTCTTGATTGAAATAGTGGCATGTCATCTTATTTAATTCAGTTATTTCATAAGGTACAATTTGTTTAGCTGACAGCTCAG
ACATGCTTCTATGCAACATGTTTATACCAATGCTGTATTTTGGCAATCCTGGATGTGGCATTACTTAACTGTGCTTTCATATTCAATAGTTCTGGCAAAG
GGAGGCTGCAAGCTTGAGACAACAACTGCACAACCTGCAAGAAAGTCATCGGTATGGCTAGAAAACCCTGCAAATTTTACAAATGTACAAGTCAGAAAAT
TTGCCTAATGTAAAAGGATCAAACTACAGGATTTGTCAGCATGAGCACAAGTGCCTAACTTCCGAGTTAGATAACTCTGGAGGAAGGAAATCCTTCCTAC
TGTTTGTTTCAAGAATATACAAAAATTCAAAACAACATTAGAATAGAACCAAAAGAAGAAACAATATTCACCATGTAGCAATACTCAATTCCTGGTTGTC
TTATTTATAAAGGTATAAAACTAGGTTCTGACGAGACAATTATGAATCAACTACACCATGTTACAGATCATCCATAACTCTTGGTGATGCAAATTAAGGG
CTGTAAAGTGACAAGTATACCTCCACCAACTAATTTGTGCAGAGTGTATAAAATTGTAAAACAATGGATAAGAATGAATTTAGTATACAAGATAGCATAC
AATGCCCAAGTTTAAGGAGACATACTGATTTACTGACTCCATAGGACAATCTTTTGCTTATGAGGACATCAGTTTTCATAAACAATATCAATGTAAAAGG
AGGTAACAGTTTATGAATTTCCTATGCCAAGCCATTTATAAGTCAAAAAATGTACCTATTTAGCTTTTATGCATTCCAATTAACTTCCAAAGAAGAAGAG
TACATACTAATGGAAAACAAATATGATATTATCATCTGTTTTGCCCGGTAGTTTCTTGAGTCTCTGATTTGTCCGCACTTCACTCCCAACCAACACAACA
TTAATACACATGGGCAGAGGAATATCAGGTTATCAGGTAAACTGAATACAGTTTGATTGACTGCAGTCAGTTGATGGGACAAGATCTTTCTGGATTGAGC
GTCAAGGAACTCCAAAATCTAGAAAATCAGCTAGAAATGAGCCTACGTTGCATCCGGACAAAAAAGGTAAGGATGCTAGCCATATTTCTGTTTTTCGTTA
CCAGATTGCCTCATCCAAGAATACAACTGATGATGACGTTTTTTACTTTCAGGACCAACTCTTGATTGATGAAATTCACGAACTGAATCGAAAGGTTCTG
GAATATTCTGTTATTACTCTGCAATGAGAGGTCTCTGAGTGGATTTCAAGAAGCTGACTCTACTGGCTCATTTTCAGGGAAGTCTCATGCAACAAGACAA
GATGGAACTATACAAAAAGGTCAACCTAATCCGTCAGGAAAACATTGATTTATACAAGAAGGTATTTGATAAATCGTGAATGAAAATTCACAGGATATAC
ACCCCATCCATTATTGACTGAGGTTTATTTCTCTACTCAGCTCTACGAGAAAGAGGTGACAAGTGAAGTCAACCGAGATTCATCAACTCTAAACTTTGCA
GTTGTCGAGAACGTCAACATTCCTGTTCATCTTGAACTTAACACTCCACCGCAAGAAAATGATGTTGAGCAAACTGCACCTCCTAAACTAGGATTACAAC
TAAATCCATGAAGGCATGCAGCATGGCACTGCTAATGTTCATTATTTTCAACGGCTAGGATTCAAAGCAGCTAGAATACCATTGGTTGCCTAAACACATA
GCAAGACAGACGTGGCATGCTGGGCGACGGTAATGGTAGTGTAAATCTATTTTCAAATAAAATGTATTTTATTGAAGACTACAGAAAATTATTTGTCTTA
TAACTATTTCACGCATTCTGCAAGTTTGTTCACCAACTTGAGTACATGACAGCTACAAATCTTATATTGACAAAAGATATGGTGTGCGGAAATAATTCAG
GTACTGTTTTTCCTAAAAAAA
>Locus_5417_Transcript_9/28_Confidence_0.172_Length_815
GCCAGCACCAGCATGAAGTCAGTGATTGATCGATATGGCAGAGCGAAGGAGGAGCAGCAGCTTGTGGCAAATCCCAATTCAGAACTTAAGTTCTGGCAAA
GGGAGGCAGCAAGCTTGAGACAACAACTGCACAACTTGCAAGAAAGTCATCGGCAGTTGATGGGACAAGATCTTTCAGGATTGGGCGTCAAGGAGCTCCA
AAATCTAGAAAATCAGCTAGAAATGAGCCTACGTTGCATCAGGACAAAAAAGGACCAACTCTTGATTGATGAAATTCATGAACTGAATCGGAAGGGAAGC
CTCATCCAACAAGACAACATGGAACTATACAAAAAGGCCAACCTAATTCGTCAGGAAAACGTTGATTTATACAAGCTCTACGAGAAAGAGGCAACAAGTG
AAATCAACCGTGATTCAACAACTACATACAACTTTGCAGTTGTCGAGAATGCCAACATTCCTGTTCATCTTGAACTTAACAGTCCACCGCAAGAAAATGA
CGTTGAGCAAACTGCACCCCCTAAACTAGGGTTACAACTAAATCCATGAAGGCATGCAGCATGGCACTTGCTTATGTTCTTTATTCTCAACAGCTACGAT
TCAAAGCAGCCTGAAAAACTATTGGTTGTAAAGAGCCTAAATACAGCAAGGCAGACGCAGTATGCATGCCATGGGCGACCGATAATGGTCGTGTAAATCT
ATTTCCAATAAATTGTATTTTATTGAAGGGATGCACCAAACGATTGTCTTACAACTATTTGACACATTCTGCAAGTTTGTTCACCAACGTGACTACGTGA
GTACATGACAGCTAA
>Locus_12874_Transcript_14/17_Confidence_0.406_Length_3978

TAATCAACTACATGCAAAAAAATCATATATGGCCAATTATACACTCAAGAAAGAAGCCGTATATTAGGTTTAGGATCCGTTGCATTACAACAAAATGAGA
CGACGAGAAGAAGCTGGTACCACTAACTTGACAATCCACAGGCGATGCTACTGATTGACAATACCATGAATAAAGCCCCAAACCAATAGCTCGCACATAC
TTGGCATTCAAGTGCTGCAGCTCACGTCATCATCCTTCTTGTGCTCCTCGCACTCATCTAATTTTAATATGTTACAAAACGCCCACCTTTTTTTCAGAAG
GACCGCGTGCCATTCCATTATAGAAGTAAAACTCAGTTTTTTCCTACAACTCACACAACAGATTGAAAATAAAGCAAGGAAAGGAAAACGAACACCACAC
AATTAGCAGAAGTCAGGCCCTAGCACCTGCATTGAACTCGGATTCCTCACGTCATCAGAATGGTGATCCAGGGATAGACCCTGTCATTGCGCTCTGGCCA
GCAGCTGGGCTGGGCCATTCCACGAGCACCGGCCATGCACCATAAGTCGGCTTCCTCCTTGACTGAACGAAGCACCGTAGCTGTGCTCGGTCTGGCCGCT
TGGAAGACACACTAGTTGAGATGCTTCCAAATGATCCATGCTACTAGCATGACCAGGGATTTGACGCCTTTCCTGATGTCACCTCCAACGCTATCCTCCA
CGTGACGTCACCAATCGACGAAGATCACATCATGCGGTTGCGGGGTGTGCTACTGAAGACCGAGCTCCGCCAGCACCATGTGTCAAACCTCCCTCGTGAA
GATACAGGTAGTCAGGAGCTGCTGCATCGTCTCATCTTTCTGATCACAGAGAGGGCATTTGTCCGGTTGTTGTAACGCGCTATGCTTGAGTAGTACTCCA
TTCCCTCACACAAGATGTTGGCTGTAGGACGACCTGGATGCATGTTAACGGCATCCTTGAACATGGACTCTACAGCCCTTCTGGTGGGTTCGTCGGTACA
CTCGACCTCAATGTGTGCATCTATTCCCTTGAGGGCCGGTAGGTGCTTGATGCCAGCAGGCGCAGCGCCGTACCTCTCCCATTCTTTCTGCACATTAGCA
TAAAGCACCAACCTTTCGAGCCTGGCCATTGCCCCTGCCTTGAAGGTCCAGTACGACATCCTGCTACAGCGAACTTGAAAATGCTTCAGAACAGGGAACC
AGCTTCCACGGATGATGATCTTGTCTTCAGGAGCTCTTAGGATGTTCATGCTGAGTTGGGTAAGGGAGGGCAACTGTGCTAGCTTTCCGACATCCTCCTC
CAACACCTCGTTAATAACAAGCTTCAGGTCACAAAGATCATGGAGTTGGCCGATCCACTCAGGAACCCTTGAGAACCAAGAATTATATACATGGAATCTC
TGCAGATGGCAGAATGAAGCGGGTACTGAACTAAATACATCCAAGCAGCTGCCAGCCATAGGCTTCAAGCATAGATATCTGAGGTTGCACAATTTCTCAA
GGCAAGTGCGCAGAACTTCCCTACCTCTTTCTGCCAACTTATCACTAGGTATAGAATTGCCATAGTGAGGGTGCAAAACAATTTCAAGATCGGTCAAATT
GGTCAGTTCTCCGAGACCCTTGAGATTGTCTGGTTCCATGGCCAAATTAAAGCCGCGTAAGGTATGCAATGATTTCATGTATCCTATCCCATCAGGAAAT
GTTCTCCAACTTGGAACAATCAGACACGATAACCGGCTCATATGAACAATGTCCATTGGAAGTTGCTGATACTGCTGTCCTTCAGCTTGAAGTGATGAAG
ATCTAGTTTCTACTTCATAAAATGTTTCCAACTGTTGCAGACCCCCAATTTTACTAGGCAACAACACTACATGGTAGTAACTCGCTGCAATCCTTAAGTA
TCTCAGTTGAAATAAATGGCATATTGCACTGAAGTCCAGAAACACCAACAACGACTGTGACCCTTCTGGAATTTCAATTGTTAAAACTCGGAGATGCTTG
AACAGTGCAAAAGGAGGTAAATATGAAGAAGTTCCAAATCTTGCAAGCATTCGTATTTGGGATAGCGGGACAGATCCCACTACTCTATCGTCATCTATTG
CACCATCCAATTGGAGGCTGAGTCGACGGATCTTGCCATTCTGTCCTGTCAAATCTTGTACATCATCTATTACAGTGATAAAATTCTCTTCTCTGGACTT
ATGTACGATAAGATCAAGCATCATATCATGTACCTTGCAAGACACCACCTCACCGTTGTAGTCTGTATCTGCGGGTTGAATCATACTCCTATTGATGAGC
TCATTGAAATAGCTCTTTGCAACATGCTCCGGGTCTGTCCCGTGAGCTTTACTTATAAAACCTTCAGCTGTCCATTGCCTAGCCAAATCATTCTTGGCTA
TTGTGTAATCCTCTGGATAAATACCCAAATATAGCATGCAACTCTTTAAATAATGAGGAAGATGTATGTAGCTGAGGTTCAATATTTGTCTCATGCCTTC
CAAGGTTGGGCTGACTTCAAAGTTGGAGCCCAACAATTTGTGTACATGCTGCCATTGCGCCTTCAGTTGGTTTGGCTGACTGGCAAGAAGGCTAGATACC
GTGATAATTGCAAGTGGCAAACCACCACACATTTTCAAAATTTGATCTGAAACTTCTTTCAAATATCGAGGACAAGCGTCCTCTGAACCAAATACTCTTT
TGAAGAATAACCTCCTTGAGTCTTCACCATCAAGTGGCTTCATCTTATAAATATAGTCATATTGATTACTACAGCACTCTCCGGCCACGGTTTCTATCCG
CGTAGTTGTTATTACTCTGCTACCATTCCTGTTTTCTAGAAGAGCACATGTAATAATATGCCATGCAGAAGCATCCCATATGTCATCAATTACAATAAGG
TACCTCTTGTCCAAAAGGAACTCTCGCAGTGCAGTAATCATTTCATACGCATCCCATGTTTCCATGTTTGTGTGCTCAGGGGCTTTAAAGCCAACTTGAG
ACAGTATCATTCTCAGGATCCTCCTTATGTTTGGTTTCTGGGATACAAAAACTAGTGCTCGACACTTGAACTTCCCCTCTAGCTTGCGATATATCTCATT
TGCAAGCGTAGTTTTTCCAAGGCCCCCGAATCCTACAATAGAGAGCACCTTTCTTTCATGTGCAGACACACCCTCTTCATCCATCAACTGAATTAATTCG
TCCCTCGGGCCCTCAATACCCACAAGGCCTGATGTCTCTGCATAAATTGCCAACAAGCGGAGATCAATGGTTGTGTCCTTTGGCTTTGAGACAGCGTCGT
CAACCTTGTACCTTGTGCGCCGCTCAGTCACCTCCTTGACACGGCTCTTGAGGCCTTCAAACTCTTTGGCAATCTCATGCCGAGTATTCATGGTTGTCAG
CAAGTTCATGCTCCTGTCGATGAACCCCTTGAATCCAGGAGACTTGCTGGGCTGGCATTCCACGCGGAGCATGAACTCATTGATACTGTCCTCAATGTCA
TAGGACAGCTCACGTACCTCCTTAGCCCAGCACTTGTCTTTCTCGTCGGGCTCCTCCGTATCTGACATCCTCTTGAGGAATGCATGCATGCTCTCGAGCT
CAGCTTTGAGAAACCTGATCTGTCCCTTAGCTTCTTTGATTAGCTTGTACTTGTCTGTGAGCAAATCACCAAGCTTCCCGAGCAGGGAGGCCATGGCGCC
ATGTGATGCGGTCACCACTGCAGCCTCCATCAGTTCTTCTTTCCTTTCTTGTGCTTGCTCTCAATATGACAGTGTACAGTTTTCTTTGGAGTGCATTCTA
AGGTCTGTCAAGCAAGTGCTTGGAATAAATGAGGCCCCGAGCAAAGATTCCTCTCTTCAGTTACTGATATATGAAGTCCTTTTTCTTTCTTTCTTTCCTT
TTCCTATTCTTTTCACATGTGTGCTCTCTTATCCTGAAGTCCTAATAATGGAGAATAGGATGCTCTTTGCTCGATACC
>Locus_12915_Transcript_27/30_Confidence_0.667_Length_3720
GCTCCTTGAGAAACACCATCCAATTACAAGACTATTTGATGCAACAATCACGCATATGGCTGCGCTAGAACTACTGCTCTGAATTAAGAGGCGTTGAAAT
ATTGTCCGGTCTACATATAGGCACTTGAGGTGTATTGACAGACAGTTCCCCTTAGTTTGCTACAACACATCAGGCGAATTCCACCAAATTAGGATGATGT
TTGTTTCTTAGACACAGGATAGCAATGCCAAAAGTTTTTTATAATTTTCCGCTTCACCGCAGATTTGGCATCTCAAAGGGAGTTGTGGCAAGATTCTCTT
GACCAAGCTTTCTTGCAGATTTGGCAAGAAAGATAAAAAAAATGTTGGTATACTTAAGCATCCTTAATAGAAACTAAACAGGTCTTAGATTGAAATTATC
ATAAATAAACCCTTTTTCACTTTAGCTGTAGTCCGGATGAAATCTTAGGGGCCTTGCTTAGCCTGCCCTGAAGCTTGCCAGTAAGTCGAAGCTGATGCTG
ACACAGCACTGTTTCAAGTTCTCATCTTCTGAAAATGATCAGCAGTCAATAGATGTTTTAGTCCATTCTCCAGAAAAATGAGTGCCGCATGAATCCTTTC
CGGTTTATAGTGAAACATTTTAGACGATGACCTTTAACATTCTAGAAAACGAAGAGAAAACCTACAGTCATCTAATTTCAGCTTAAACATGTTCCAGCAA
GATCATCACCATCCTGACATGTTCCGTTTTGTCCCTAGACCTGAGCACAAGGTATTGCGAGGCTAATTTAGCGGGGCAATTGATTTTTTGCCACTATGAT
ACAACTGCAATTACCCGCTGTCACTGACACGTGGGGTCATCTGAGTCACTGAAATGTGGGCCCAGTGGCATTTTGTTTGCCACTCACCCTTCCAGAGTGG
CAAAAAGTTAATTGTCCCTAATTTAGCTCAATTCAGTTGTCGGAGCAATTGTGTGTGAAAAGTGTGAACAACAGCATAGGAGACAATACAGCAAGTGTAG
CGTGCCAATATGTGTCCAGCTTGATAGTTCACTATAATAAAGTTCTTTAATTCAAGTATTTGAATAATATAGAATCTCAACCTTCAAGTTTAAAAGGTTT
TGTATTTGCTATTATAATCGTTGCCACCCCCTCACCCCTCTCAAAAAGAGAGTTACTTTGTAGCGCACTGTAAATCTCTTGATTGAAATAGTGGCATGTC
ATCTTATTTAATTCAGTTATTTCATAAGGTACAATTTGTTTAGCTGACAGCTCAGACATGCTTCTATGCAACATGTTTATACCAATGCTGTATTTTGGCA
ATCCTGGATGTGGCATTACTTAACTGTGCTTTCATATTCAATAGTTCTGGCAAAGGGAGGCTGCAAGCTTGAGACAACAACTGCACAACCTGCAAGAAAG
TCATCGGTATGGCTAGAAAACCCTGCAAATTTTACAAATGTACAAGTCAGAAAATTTGCCTAATGTAAAAGGATCAAACTACAGGATTTGTCAGCATGAG
CACAAGTGCCTAACTTCCGAGTTAGATAACTCTGGAGGAAGGAAATCCTTCCTACTGTTTGTTTCAAGAATATACAAAAATTCAAAACAACATTAGAATA
GAACCAAAAGAAGAAACAATATTCACCATGTAGCAATACTCAATTCCTGGTTGTCTTATTTATAAAGGTATAAAACTAGGTTCTGACGAGACAATTATGA
ATCAACTACACCATGTTACAGATCATCCATAACTCTTGGTGATGCAAATTAAGGGCTGTAAAGTGACAAGTATACCTCCACCAACTAATTTGTGCAGAGT
GTATAAAATTGTAAAACAATGGATAAGAATGAATTTAGTATACAAGATAGCATACAATGCCCAAGTTTAAGGAGACATACTGATTTACTGACTCCATAGG
ACAATCTTTTGCTTATGAGGACATCAGTTTTCATAAACAATATCAATGTAAAAGGAGGTAACAGTTTATGAATTTCCTATGCCAAGCCATTTATAAGTCA
AAAAATGTACCTATTTAGCTTTTATGCATTCCAATTAACTTCCAAAGAAGAAGAGTACATACTAATGGAAAACAAATATGATATTATCATCTGTTTTGCC
CGGTAGTTTCTTGAGTCTCTGATTTGTCCGCACTTCACTCCCAACCAACACAACATTAATACACATGGGCAGAGGAATATCAGGTTATCAGGTAAACTGA
ATACAGTTTGATTGACTGCAGTCAGTTGATGGGACAAGATCTTTCTGGATTGAGCGTCAAGGAACTCCAAAATCTAGAAAATCAGCTAGAAATGAGCCTA
CGTTGCATCCGGACAAAAAAGGTAAGGATGCTAGCCATATTTCTGTTTTTCGTTACCAGATTGCCTCATCCAAGAATACAACTGATGATGACGTTTTTTA
CTTTCAGGACCAACTCTTGATTGATGAAATTCACGAACTGAATCGAAAGGGAAGTCTCATGCAACAAGACAAGATGGAACTATACAAAAAGGTCAACCTA
ATCCGTCAGGAAAACATTGATTTATACAAGAAGGTATTTGATAAATCGTGAATGAAAATTCACAGGATATACACCCCATCCATTATTGACTGAGGTTTAT
TTCTCTACTCAGCTCTACGAGAAAGAGGTGACAAGTGAAGTCAACCGAGATTCATCAACTCTAAACTTTGCAGTTGTCGAGAACGTCAACATTCCTGTTC
ATCTTGAACTTAACACTCCACCGCAAGAAAATGATGTTGAGCAAACTGCACCTCCTAAACTGGGATTACAACTAAATCCATGAAGGCATGCAGCATGGCA
CCGCTTATGTTCATTATTCTCAACGGTACGATTCAAAGCAACCAGAAACACTATTGGTTGTAAAGAGCTTAAACACATAGCAAGACTGACGCGGTATGCA
TGCCATGAGTGATTGATAATGGTAGTGTAAATCTATTTCAAATAAAATGTATTTTATTGAAGAATACAGCAAATGATTTGTATTATAACTATTTGACACA
TTCTGCAAGTTTGTTGACCAACGTGAGTACATGATAGCTACAAATCTTATATTGACAAAAAGATATGGTGTGTGGAAAAAAATTCAGGTGCTATTTTTTTC
TATTTTGAGAATTGTATGCATAGCAGAAGAGCACAAATTGCATTCTGGTTTATCCGTGCAGGCATTCCAGAGTATCAGAATGGGGATCAACCTTTCTTCA
GCTACATATGTAAATATACGGATGCTAGTTGCTCACTTAAAATTTTGTAGTGTTCAAAGGAATAACAACAGTAAGACGTTGACGCACCGAAAATAACCAG
AAATAACGGATTGTACTATGACAAGACACACGACAGATACACTAGCTTAAGTTTGTACAGGAAAATATTTGGCCTATTCTTATGTCACAGTAAATTAAGA
TTATCACCTTAATCACAGAAATTCAAGGGGTAAACGATTATAGCACAAAACGATCCTGGACCCCAAGACCTGGGTCTCTGAACGGCTGAACCTCCAATTA
TACCTCTATACATTTGGAATGTGTGTAGTCAAACTTTCGCTACTGGTATCTTTACAAATATTTACATTGAACACATCAAAATGATATACATGGATTCCTC

TCGATATGTCTTTGAGGTTTGGGTTCTAAAAATGATATACAAATTATTAGGTTCTAAACTTAAATGATATATACACATCAAAATGATATACAAATTATTG
GGTTCTAAACTTAAATGATG
>Locus_12535_Transcript_49/73_Confidence_0.245_Length_2739
ATCTCCAACTATGGCAGTGGTGACAAGGAGCAAATTTGCAATCTTAGGGTGGTTCTCGTCGTGAAACCTGAAAGTTTGGGGAAATAAATTTGGGGGTGTG
GATGTCATGATATGTAGGGTCCGGCACTGAAGTGGTTGATGCTGTGGTTTCCAGGTTAAGGGGAGAAGAGGAATAGGATATAGATCTCAGGAAGCCAGAG
ATGAGGTCGAGGGGGAGCCAGTGGCCAAGCTTGTGCTCCAGTGAGCCTGATTCTCTTGTTCCCAGCTGATCGATGTGCCACTGCCTTGGTTCGCTGGATC
AGCGGATCTCCATGGTTCTGTGCGGTGCTGCAGATTGAGAAGAAGGTTTTCCAGGGGGAGGAATCTCTTGTGGGTTCCTTTTTCTGGGAGCAACGTAGTG
GGAGGTAGCTGCTTTTGCATACAATTAGGAGCAGCAGTTGAGATATTCAATTCGGGCATGGGTTCTTTTGGGATGGACTGGAACCAGAAAGGCTCGGTGT
TGTGGGATTGGGAGAATTTGCCGCCGATAGGCAGAAATGCAAACGAGAACCTCAAGATTGCGCCACAGGCTGAACCGAAGTTTGCAGGTGTTGAGGCAAC
AAGGCATGAATCGGGGCATTCTTCTTGTGGTACTTTCTCTTCCAGCTCGGAGATGGGGTATGGTTCATCCAAGAGTTCCATATCAGCGTCGATTGATTCT
TCACCCAAGGTGGGAAACAACGTGGAGCTCAATCTTGCAGCTGTCAAAGTGCCTGACAAAAACACCGTCAAGAACACTGATTTGGGTAAAGTTGATGACG
CTGGAACTTCTCCATCATCGATGATAGCCGTCAGCAGTGGAGAACCGGTGATTGGCCTGAAACTTGGCCAAAGAACCTATTTTGAAGATGCATGCGGAGG
GCAAAGTGTCAAGAGTTCACCATCCAATGTGAGTGCAGTGACCCCTGCTTCTGCGAAGAAAGCAAAGATGATTCAAAACGCACAGAACTCGTACTGTCAG
GTTGAAGGTTGCAAGTTTGATCTCTCTTCTGCTAAAGATTATCATCGGAAGCACCGAGTCTGTGAAACTCATTCTAAGGCTCCCAAGGTTATTGTTGCTG
GTCTGGAGCGACGCTTTTGTCAGCAGTGTAGCCGGTTCCATGCTTTAACTGAGTTCGACCAGAAAAAACGAAGCTGCCGGAGACGCCTCAATGATCACAA
TGCCCGCAGACGGAAGCCACAGCCTGAAGCAATTCCTTTCGGTTCATCAAGGCTCTCTGCAATGTTTTATGGTATCGCTAAGCCTTTGGTTGCCTAATGG
TTTTGGGAAACTGGTGCATTAATTATTGTTCTTTTAGCAACTGTTCTGTTTAAAAATCTTGCATTTTTCATGTATGCCCAGAACTAAACGGTGGTAAAAT
TTATGAGTACAAAGTGCAATGTTGTGTGTCTGAAGTTGTCCCTTATTTGTTTATTCATAACTCAGTGCCATGATTTTTGAGATGTCATGAATTTACATAC
TTCTGATATTAGATGACATGATAACTTTGGCAATACCGGTAATTAATGAAACTCCATCTGAGTACTGCCATTTGTGATGATTACTATAGATGGGACAAAA
GAGATATCAATTAAGTTGTATTTATTTTATGAATAGGTGCATAATGCTATATTCTTTTCGATTTAGATATCCATGACATGATTCTACTTTATCTGCCATG
ATGCCATCTCTCCATTATAGGAAGTCGGGAGACAATAGGAAGCTGTTAAGTATCTGAATAAACATCAGTTCAAATATCTACAGATGCATGTTGAATCCAC
TATATTATCAATAATTTGTACGAAAGTAGCTTTAGCTTTTTGTATTAAGCAAATCGTTTGTCCTCTTTCTGAATCTGTTTGGCTTTAGTGATGAAAAATT
GTCGTGGCGGTCTTAAACAGATGCAAGGCAACAGACAAGTCTTCTGTTTGGTCAAGCTCCTTATGGTCAAATGAGAAGCTGTGCAAACTCTTCATGGGAT
AGCCCAGTAGGAGGCTTCAAATTTGGAGAAACAAAAGCTCCTTGGTTAAAGCCAACGAGAGCTGCAGGTGTTGATGGGATGCATGTATCAAGCCAGCAGG
TGTGGAACAATATTACGCCACACGGTGCACATCATGATTTTAATGGTATCATGGCTTTCAAGGGAACCAGTGCAAATGTCCTTAATCAAGGTGCCGAAGC
TTCTCCGGTCGTCTCCAACTCGAATGGAGCCCCAGATCTTCAGCGTGCTCTCTCTCTTCTGTCAAACAATTCAGCTGGTGCTGCCAACGACCAGCCAACT
CCTCAGCTGCACCCTGGGCTGACCACCTTCGTCGGCTCCTCCAACCCTGTCACTGTGATGGAAGCCTCACCACCAGGACTCTGGCAAGACAGCACGCCCC
TTGATCATCAGGCGCAGTTCCAGGCTTTCGATCCCATTGCCGCACCGCAACAGCTCCAGCTCCCAAAACCCCCTTCCTTCAACAGCTCCACTTCCAGCTA
TGGCCAGATGCAGTGATGGATGCTTCCTTCAGTTCTGCTGTCTGAAACTCTGAAATTTGAAACTGCTGTGTTCTAAAAAATAAAGAAAAGCCTGTGTGCG
AAAACTCAGGCTTGAAAAATTGATCCAGTAGTGTTCCCGTTCAATGGCTTGAATAATCCATCCTTGTGATGGCGTGTAACTCAAATGCTTGGATTGCGCT
GGCAAGTGATCATGGACTCTTGTGTTCTTGATGGCATCT
>comp73418_c0_seq3
TAGGATGACTGTGTGGAGGATATCTAACGTGGTGCTTAAACTCGATGGCAGCCAATGGTGGTTCTTGCCTGCCTTACCATGAACTAGTAGTTTGGATCGG
AGTTGTTCTTGACTTACTCATGCGTATTCTTGGGAGTTGGGATCATATCAGTATGTATACATCTGTTATTGCTTGGTGGTGACTTTTCCTTAGAGGTGCC
TGATATGGTGGCGTTTTTTTCTTTGACACATGTTGTATGGATCATAAGTGATGTTATACTCTTTACTAATTTCATGGCCTTTATTATCTTTAGGTTTCGT
TTTCTCTTTTTCTTTTGTTATTATTGGTAAGAAGTATTCGTGTCCCTAGACTTTTGCCATGTATGTACATGTTCGTAGCGAGGTTAGTGATACTGTGAAC
TGTGGTAGTCTTGTTTCGCTGGGGGGAACTTTGATCTGTTGTTCTTTGCCTCAAAACACTGTAAGCCTATTTCTAATATCTAAAACTTGTAAGTGTGCAT
AATGCAGATCAAGCTTCCGAGGTGTTATTTTTCTCATTATGTTTTAGAAACTTAATGATAGATGCAAAGTTATTTTGTTAAATGGAGTCTCAGTCCCGTA
GCCATAAGGGAGGATCGAATCCAAACTTTAGGCTTGTGTGAGTCTTCTTGAGTGCAGCACATGATAAACATGGATCTTAGGGCATTGAGTTAACAGTTAG
TCATCTTCTTTTCTAAACATAAGGATTAGGGTGCTAGTCACACACCATTAACAATAATGTGAATCCAAATAGCATAAGTAGCACGCTTGAGGCCGGCCAA
CACACACTATTCATCTCTTGCTGATTCACTAATTGTCGGTTTGCATTTGGAAGTGTTCTCTAGTTTCTGTGCAGTAACTATTGAGCTAATGACACACTAT
GTATAAATGACAATCGTAACAGATCATTTTGAACCTTTACTTGATTATTATTAGAAACACCTAGGATTTGAAGGATATCGATGTTACTGACTTACTTCTC
TTTTGCTACATCTGGTTAGGACAGGATTCTACTGTGTTACAATAGGGTTTTCTTTGGAAATTTAACGTTGTACAATAATGCCTTTATTTTTTTTGTATCA
GGTGACTCCTTCGGGAAAATGGTTAATGATCATACGAGGTCAAATTTGGTTTTTGATAATAAGCAATCTCTATTTGCAAGTCATAACATTGACTACGGCC
AGCCAATAGCTTGTATATCATACCCATACAATGATTCGGGCTCAGGAGGTGTTTGGGCAGCCTTTGGGTCACGCACTAGCGCTGCAGCTGTGGTATATCC
TTCTAATAAACTGTTGTACTAAAATCTCTCAAGACCTTTGAACTTCAGTGCTGATGTCGCATTTCAGCATTTAAAATTTTACATGAGAATGTTAAACATA
TATGATCCTAGTTTGGGGAATCTCCTAGTTTTCAGTTCATTCTGATTGGGCTACAGTATGCTGTCTGAACGGTCCATCTTACCATGGTCTAATCAAATGT
AGAAACTTAAGTAGGCTAGTCGCCTAATACCTTAACTATGTTTATTTCAAGCAGACACTGCTTAATATGATCCTTGGTTCTTTGCATAAGCATGCATGGG
CCATGACAAAAGGTCTTTTTTATCACTGCTATAACCGATATACTAATGGCAACAAAATGCTCAAAAACTACCACATCTTTTTGCAGCATATTTAGCCATA
ACCATAATGGGAAACCGTGTGCTAAGCTAGTTGCAACTGCTTCTATTCTCCAGCGACAAATGATAATATCTAGAAAGCTAAGATCGCAGCACTTAATTGT
GGCCTATTTGCACAGGTTTGAACCAGATTTTGCATTTCTTTCTCATGCACATACACTGTTACGTAAATCACTCTATTATTTAGAAGCAACAAAAGGACCG
TACATTAATTAATTCTCTAGCTGGTTATAACAGAAGCTAAACTAGCTGGATTTTGGCAGAGTTGATCTTAGTATTAGGGTTAAAAGTGAAAACAATACTT
GACTGGATGCAGCAACAGTGCTTTGTTAATTCTCTGCTCCAGTAATGTTTTCAGAACTGCATCGTTGTTTGTGTGTCCAAATTGTGCTTTCTCAGTTCAT
GCACGACTCCATATATATCTATCTCAGTCTGCAGTTACTAGCGTGCTGGCAGCTCATAGACACATAGTGTCCGTTTAGATGATTTCGTTTAGATGATTGC
TGCTGTTTGTAGTTGTATAAATTGACTCATGATATCAACCAAGGTTACACATAATTGTTGCCAGGATTCCATTATATGCTAACGTTAGCTCACATATAAG
GCAGGGCCATCCATCCACCGTACAGCTTGTTTATCTGTAAGTTCATCACTTTTGAGGGTTTTGTCTAATCAATTGTACTTAAAGCAGTTCCATCCCCAAA
TTGCTTGTGGGGGCACATCTGCGAGAGTTCCCTTACCTCTGGAATTAGCAGATGATGAGCCCATATATGTCAATCCCAAACAATATCATGGTATACTTCG
CAGAAGACAGCTACGTGCTAAGTTAGAGGCTCAGAACAAGCTAGTCAAAAACCGAAAGCCTTACCTTCATGAGTCTCGGCATCTTCATGCAATGAAGAGG
GCAAGAGGTTCTGGCGGACGTTTCCTTAATACTAAACAGCTCCAGGAGCAGCAGCAGCTGAAGTCTCGCAATGCCTCCACCAGGTCCACCACAAATGGCG
CAAATTCCTCAGGTTCAACACATCTACGGCTTGGTGGTGGTGCAGATGGAGATCAAACCATGTCGGGGACGAAAACAATGGCCTCACAAAACAATAGCAA
AAAGGCTGTTTCTTCTTCTGCAGCTCTTGCTTTCACCGTGACTCCTATGGTGCGCAAAGATGACACCTTCTTCCAGCACCTCAGCCACAATGTCAGCTTC
TCCAGCCATTTTGGCCAGGCAAGCGCCCAAACCGGCGTTGGAGGCATCCATAATGAGACCCAGCATAGGGTTTCCGTGATACAATGACGGTTTGCGAAGC
TTATCTGGTGATCCAGGCTTCTAGGTGTCCTCGTGTCCGGTGTAGTCTTGGTCGCTCAGGCAATTCATCCTTGGCTTAGTTTTTGGTGTGTTAGAACCTA
CAAATGTTTCATACTCTGTTGTGGTTTGCAGAGCCCATAAATCAGGGCTCTCCTAAAAAAAAATCAGGGCTTGATGGCGACACTACTATCCAAGTATTGTT
GTAATGGTGTGTGTTAGAAACTCGCAAAAAACCCTTCTTTGTGCTCGTATTAGTACATTATGCTGCACAATTAGCTGCTATTCTGTTCGTACTACAGTCT
GGT
>Locus_2827_Transcript_139/166_Confidence_0.194_Length_3036
AGGCGGAGAGGCTGTGAGGTCGGTGGGTGTGCTGTGCGTCTGCTCCGGCGGCGCTCCCCTCGGCGAGCGGGAGCTTGTCGGTGGGTCTCGGCGGTGTTCG
CTTCGTTTGCAGATAACTTCGCGAGCTGAAAGTTGAAGTGTAAGCGGGGAGCTAAGAGCAGTAGTTGAACGAAGCGGAAGAGGAGGACGTGATTTGAGTA
GTTGGGCTCTTGCATTGGCGAGGAAATAATGGCTGCGGCCGTGGCGATGCGAGGGGGAAGCAGTGACAGCGGAGGGTTTGATAAGGTTCCGGGGATGGAC
TCGGGGAAATACGTGCGGTACACCCCGGAGCAAGTTGAGGTACTCGAGCGGCTGTACATCGATTGCCCCAAGCCAAGCTCCTCGCGGCGGCAGCAATTGC
TGCGCGAGTGCCCTATACTTGCCAACATTGAGCCCAAGCAGATCAAGGTTTGGTTCCAGAACAGAAGGTGTCGCGATAAGCAGCGGAAGGAGTCTTCGCG
GCTTCAGGCTGTGAACCGGAAGTTAGCGGCAATGAACAAGCTTCTGATGGAAGAGAATGAGCGTCTTCAGAAGCAGGTCTCTCAGTTGGTTCATGAGAAT
GCACACATGCGGCAGCAGCTGCAGAATACTTCTTTGGCCAATGATACAAGCTGTGAATCAAATGTGACTACCCCTCCAAACCCTATAAGGGATGCAAGTA
ACCCTTCTGGACTCCTTTCGATTGCAGAGGAGACCTTCACAGAGTTCCTCTCAAAGGCTACTGGGACAGCTATTGATTGGGTCCAGATGCCTGGGATGAA
GCCTGGTCCGGATTCGGTTGGTATTGTGTCTGTTTCACATGGTTGCCGAGGTGTTGCTGCCCGTGCCTGTGGTTTGGTGAATCTAGAACCAACAAAGATT
GTGGAGATCTTGAAAGACCGCCCATCTTGGTTGCGTGATTGTCGGAGCCTTGAAGTCTTTACAATGTTACCAGCTGGAAATGGTGGAACTGTTGAGCTTG
TTTGCATGCAGATGTATGCGCCTACTACTTTAGTTCCTGCACGAGATTTTTGGACGCTGAGATACACAACCACTATACAGGATGGCAGTCTTGTGGTCTG

TGAGAGATCTTTAAGTGGTTCAGGAGGTGGTCAAAGTACTGCATCGGCACAACAATTTGTTAGAGCTGAGATGCTTCCTAGTGGCTATTTAGTCCGTCCA
TGTGAGGGCGGGGGCTCAATTGTGCATATAGTGGACCATCTGGACCTTGAGGCTTGGAGTGTTCCTGAAGTTCTTCGGCCGCTCTACGAGTCGTCTAGGG
TAGTTGCTCGGAAGATGACAACTGCAGCACTACGGCACATCAGACAAATTGCTCAAGAAACAAGTGGAGATGTCATATATGCCTTGGGGAGACAACCAGC
TGTTCTACGAACATTTAGTCAGAGGCTCAGTAGAGGCTTTAACGATGCTATAAGTGGTTTCAATGATGATGGTTGGTCTGTCATGGGTGGAGATGGCGTC
GAAGATGTTATCATTGCTTGCAACTCAAAGAAGATAAGGAACAATACCAATCCTGCAAGTGCTTTTGGAGCCCCTGGTGGTATCATATGTGCTAAGGCAT
CCATGTTACTGCAGAGTGTTCCACCAGCAGTACTAGTTAGATTCTTGAGGGAACATCGGTCTGAATGGGCAGATTATAACTTCGACGCATATTCGGCCTC
GGCGTTGAAAACAAAACCTTGTTCCCTTCCTGGGTTGCGGCCTATGAGATTTTCTGGGAGCCAGATCATCATGCCACTTGCTCACACAGTGGAAAATGAG
GAGATCTTAGAAGTTGTTCGTCTTGAAGGACAGGCTCTTACACAAGATGAAGGTCTTTTATCAAGAGATATTCACTTGCTTCAGCTTTGCACTGGAATAG
ATGAGAAATCTGTGGGGTCCTCCTTCCAGCTCGTGTTTGCACCAATTGATGAACATTTTCCAGATGATGCTCCATTGATTTCTTCTGGCTTTCGTGTCAT
ACCACTTGATATGAAAACAGACGGCATATCTTCTGGTAGGACGTTAGATTTGGCATCTAGTCTTGATGTTGGTTCTACCACGCCCCACGCCTCAGGAGAT
GCATCACAGGATGATTGTAATTTGAGATCTGTGCTGACAATTGCCTTTCAATTCCCTTACGAGATGCATCTCCAAGACAGTGTTGCAACTATGGCCCGTC
AGTATGTTCGCAGTGTTGTTTCTGCTGTGCAAAGAGTGTCGATGGCTATCTCTCCCTCCCAATCTGGTATAAATGCTGGGCAGAGGATGCTTTCTGGCTT
CCCTGAGGCAGCCACACTTGCTCGCTGGGTTTGCCAGAGCTATCATTACCATCTAGGGGTAGAATTACTTAACCAGTCAGATGAAGCTGGCGAAGCATTG
TTGAAGATGCTCTGGCATCATCCAGATGCTATTTTGTGCTGCTCTTTTAAGGAAAAACCTATGTTTACATTCGCCAACAAGGCAGGGCTTGACATGTTAG
AAACGTCCCTTATTGCCTTACAAGACCTCAAATTAGACAAGATCTTTGATGAGTCTGGAAGAAAAGCGTTATTCTCAGATATCTCTAAATTGATGGAGCA
GGGCTACGTGTACCTGCCATCGGGCGTGTGCATGTCAGGAATGGGTCGCCATGTTTCGTTCGACCAAGCTGTGGCGTGGAAGGTCCTTGGTGAAGACAGC
AACGTCCACTGCCTGGCCTTCTGCTTCGTGAACTGGTCCTTCGTGTGAGCCTTTGCCATCCTCCGAATGCCGACTTGATCTGTGACGAGGAGATCCATTT
TTGTATTTATCTGGAGAAGGAAAATCTTCCACTGTGACAAGGCTTATGTTGCACTAGCTAGCTCTAGGTTTGGGAATCCAGTGTAAAATCTGATGTCTTT
CCATTTGGCTACCAAGCTGTGCTCTGGTTGTTTATCTTGTTTGGGACGATGCTAAGTTGCTAACATTGGGTTCCCAACTTACGCGTCGAAGTGTGTTTCA
TCGCATCAATCTTCAATCGCAGCTGAATTTTCGTTT
>Locus_14326_Transcript_29/49_Confidence_0.397_Length_2927
GTATTCTCATTTCCTCGCCGTATTTTCTCTCTCCAGCTCCAGACTCCAGAGTCCGGAGGAAGCAGAGAGAGAAGCTCGACGGGAAGGTAGAGAGAGATAT
AGTGCTCCTGATCCCTCTTTGAAATCCTCGATTGGTCAGTTCTGTTCCTGGATATACTGTTGCTGCAGATTGGATGCGTCTGCTTTCCTTTCGGGGAGTG
GTGTCTAGCTCTGTTGGTTCTTGCCTTCATAGTTAGTACTATGCCTTAATCTTGCTCCGCGATTGGTTTGATTCATGTTTTTCCTTTAATCTTGCTACCC
CAGGATTTTCCCCTTTGGTTTTCTTGGTTTGCTTCTCGGAAATCCGTGATTTTAACAGGATTTTTGGCTGGTGTTGCTCGATTTTTGTTCTTGACTGTGC
TTCCTTGGGATGAGCTTTTGCCTAATTCGACATTACAAGATGTGTTCTTTCTAGAAGTTAAATTGATCTTCTATGAGCCGGGCGCTGTATCGTGCATTAC
ATGGGGGTGGCGTACTGGTACTTTGGGCCGTAGAGTTGATGGAAATCTGCAGTCCCTTTTGTTGGATAAGGGGCTTTTTCCTTGGATAAGGAGGTTTGAA
GCTAATTTTGTGCGAATTGTGGCGGTTTCTGGATATCCTGGTTGAATTAGTGGTGTGATAGATTGTTCTAGAGAATAGGAAGATATATGTTTTATTGTTA
GAACATCAAGAGAGTGTTGAATCAGGTATGTCAATGTGTGAACTTATTGATGGGAGCTAAATGCTTGCTGACGTATCTTCGCTATAATGACCTAATCAAA
CCTAACAAGCACTAAATACTGATCAATTCTCAGTTATACAAAGATTTTCCAGAGAGTTCTTCAGAGCTCGAACTTCTGGGATGAAAATAGTTTGGCTCAA
GATAGTCCACTGGAAGGAGTTTAGGATATCTGTTTGAGCTCTGCCTGCAATATATGGAAATCTGAAGGTGATATGGTAGTAGAGTTGGGGACCCTGATCC
CCAAGCCAAAAGAAGTTCGGTAGAATTTCTCAGGAATATTGTGGTAGTAAGAAGCAAGTTAAAAGGAACCTAATTAGAAAAAAAGACATGCCTTGCTCGC
TCATGAGAATATAAAACAGGTATGCCACCTCCAGTCAGTACTTGATTAATTTCATTGAAAATGGAGGGCTGTAATAGATCCTTATCTATTAATGTGTTGT
ACCTAAACTTGGAAATCATCAGCTGAGGGCATTGTGCATGGTGCGTGGAGTACTAGCCCTCGGGAGATGCTTCTTAATTAGGCAGCGAGAAGGATAGAGA
TCGGTTTGACATATGTTCCATTTGTAGCTGTACTGGATATTTCTGCATGTTCCCCTGGAGCAGTTAGTTTCAGAATTATGATATTTCTACTTGTGGCAGG
ATTGTTCTGTTTCTATTATCTGTTCATATTTATGCTAGTCTGTCTTTTATGTTGTAATATCTATTGTCTATTGCACTTCAAGTGACTATGTATAACCATA
TTCCAGTGATTCAGTGAATGATTATGAATTAAAGTAAGAGGACATTTACATCAATTATCCTTGTTCTTTCTCACGTCATTATCAATTCGGTTGATTAAAT
TCTGGCAACTTGCCATGATGGTTACTTTAGCTGATTAAATGCTACTCGGAGCTAAGGATACCTTCTCGTAGTGCTCCCGCTTTTGTGAGAAATGGAGGAT
CATCCAGTCCATCCCATGTCGAAGTCCAACAATGGCTTCTTGTCAGGAAATGGCTATCAGATGAAGCAATTTGGTCATAAAATCGACGATAGGGACTCGT
CATCAGAGTCCGGTCGGTCTCACCAAGAAGCTTCCACAGCGAGTGAGAACAGTATAAATGAACAGCACACCTCAACACAATCTGACAATGATGATGGTCA
TGGGAAGCATAATGAGGACATGATGAAGTCAGCAGTGTCCTTGGGGGAACCAGGATCAGCCTTTTTGGCCCCAAAACTAGATTACAGCCCATCTTTTGCT
TGTGTTCCTTATACTGCTGATGCTTTTTATGGTGGGGTCTTGACAGGATATGCTCCACATGCCATTGTTCATCCCCAGCAAAATGATTCAGCAAACTCTC
GGGTTCCGTTGCCTGTTGAACCTGTAGCAGAAGAGCCAATATATGTTAACGCAAAGCAATACCGTGCAATCCTTAGGAGGAGGCAGATACGTGCTAAACA
GGAGGCTGAGAATAAGCTGGTGAAAGGTCGGAAGCCATACCTCCATGAATCTCGACACCGCCACGCAATGAAGCGAGCCCGTGGATCAGGGGGGCGGTTC
CTCAACTCAAAGCAGCTCCAGGAGCAGAACCAGCAGCAGGCATCAGGTGGTTCAAGCTGCACAAAGGCCATCGGCAATAACACAAGCTCCCAAAGTG
GTCCCAGTCACACGCCTCTGACACTTGCTTCCTCTGACACTGCAAGCGCTTCGAGGGCCAACCAGGAGCGCACCTGCTTCCCCTCGGCTGGCTTCCGTCC
CACAATGAACTTTGGCGCGCAAGGTGGAGGAGACGCAAAGCTAGCTGTGAACGGCATGCAACAGCATATTTCCTTCATCAGGTGAAGCAAGTGAAGTGGA
GCACTGCCTGTGGCAACTCATCCTTGGCTTATGAAACTATGGATATGTAATGGGCATGTAGGTTCGGTCTCAGAATAACCAAACATATGTGAGACCTTTT
TAAGTTTCCTTTAGTTCAAAGAATGATAGGTTATGACCTTATGGCTTTGTTTCTGGAGACTGAAATCCGTGCTTGATATACTATGTTGTACTGAGTTCTG
AGGTGAAAGACATCGGGAGAGTATCAGGTGCAAATGGCACAAATGTTGAAAACGGTGCAAATTCATCAAAATCTGTACCTAAAAGACTTCAATAAATTTT
AAACTTTGCATAGCTCTAGGTGCATTT
>comp86034_c0_seq2
AACCGGCACAGGGTACAACCATGCTACCGATACACCTCCGCTACAATAGCAACCAGATTCAACAAACTTCCAAGTGAATTATCTCACCTATAAATGAACA
GTTGTTCCGAACTCTAACCGTGTTAAAACTGGAAATCCAAAAACTGGATGCATGTCTTAAAATCTAAAAGCGGGATGCATGTCTGTAATAACATTCCATG
AAGCAATTTCAAAGCTTAAACCCTCTTAATCATAGAAGCCAAAATCTATTTTCCAGGGATTTCACCAGTTAACAGACCAGAGCAGTACTGTGCTCTAATT
TATCGGTCACCTGTACTTGCGTTTTTGAATGCACCAAGGTTCCTTTTGAGAGACTTGACAGCAGACAAAATCTGCAGCTTCTCTGCGGACGGCCCGTCAG
GTTTCTGATCATCGTTCCCAGTCGCAGCATCATCAGGTGGCAACGAGTCTCCCAAAGATGATCCCTCCACATGAGGTTCCCTGTCCAGCTGGCCTGCATG
TGCCCCTTGCTTCCCAGCTTGCTTTCGCATGAAGGCCGCACTCCTCTTGTACTGGGGATCAGTAGGATCCAATGCGAACAAATGTGACGTGAACATTGCT
GAGAACCTCTCGTCCTTGCTAAGATCGATATCAGGAAGTTTGTCCTCAACCGAGTCCTCTTTATCCTTCTTGCCCTTTTTACCCTTTTTGCGCTTCAAAT
TATAACCCTTTGCAACATTGGCAGTATCCTGGTCAGCAGCAACCAAGAGCTCCAACTCTTCTTTGGTTGCTTCTTGCTCCAAATGTTCCTCTGGGAATTT
GTCCTTTCTTTTCCCCTTTGCTTTATCTTTGGTCTTTGCCTTTTGCTTCTTGCTTTTGCTTGGCCTAGTTTCTTCATCAGAAAATTCCTCAGCGAAGAAG
TCATCATCCTCATCAGGCTCATCTTCACTGCTATAGTCATCATCATGCTCCTTCAACCCCCTCTTCCTAGCTTTCCTTTTTTCTTTCATTTTCTCTTGGT
GCTTCTCCCAGACAGTTTTCTCCTCATTGGTCTTTCTCTCTAGGATACGTTTACTGAGATCCTCAAGCTCTGTGTTGAATGTAATCTCCATATCCTGGCC
ACCAGCTTGTTCCTCTTCAGATTTGTCGCCTTCCAGTAAAAGAGCCAACCTCTCCTCTTTTGTTAGCTTTCTTTTGGATCCCCCATTCGGTACAGACTTG
TCACCAGAATTGTCCACACCGTCATCATCTGAGGCACTGTCATCACTTGCTAAATACATGTCAAGCTCATCTAACTGATCATCAGTGAACTTTCGCCTCA
AGACCTTCTTACGTTCTGGTTCATCATCATCCCAGGTTAGCTTAACTCTGCTATGTTGCAAAGCACGAGTCTCAAAATCAGGCTCCTTGTAACTCAGAGG
TGCCTCTGTAGCAACGTCTCGAGCAGGATGTTTGAACTCCATAGAGTCAGGAATAAACTGCAAATCAAATACATTTGCTGTTTTCAAGAACTCAGTACCA
TCAAGATTCATGTACAGGTGATTTGCAGTTGCACTAGAATCACACACCACAACTGCATAGTAATACCTCAGTCTGTTTAGCTCGTAAGTACGCAGCTTGT
TATTCTCTTTGTCAGAGTCAACTTCTTCTTCGTCGTCATCAGTGTTGAATTCATCATCATCAGTGTCGTCTTCACCATCGTCGTTATCATCATCATCGTT
GTCCTCATCATTATCATCAGTATTGGCATTAACAAGAGCTGCTGGGCCCTGAGTTGATTCAATTTCCATGCACTTCAGTCCAAATTCTGATGGATATATT
GAGACTGATAAAACTCGCCCACCTTTTGGGACGCAAGATGTCATCACCATATACAGGTCAACTGCCTTGATATGATCCCAGTCCATATTAACAACAGCAA
GCCTGTGAGTTTCCTTGTCAATCATAGGCGTATCATCGTGCCGGCCCATTAAGTAATGAGCAATGTCACTGCCGACAGAGTACTCGTCGTCATCAACGTC
TTCATCCTCGTCGTCGTCGCTACTAGAACTCTCTTCTTCCTCCTGCTCATCCTCTTCATCCACGTCCTCGTCCTCCTCTTCTTCAACTAACTTCTCTTTC
TCCTTCTTTGCCTTCTCTTTAGTCTCATCCTCCTCCTCGTCTTGGTTGAGATAGTAACGGAGCATGGGGTTCTCCCTGCCGCTCTTCTTCCGTCGCCTGC
CGCGCTTGTCCACCGGCGCCGCCGAGGAGGAGAACCTCGGGTCCGTCATCATGCTGCTGAACCTCGAGTCCAGCGCCACCTTCGCCTCCTTCCGCCGCAT
>Locus_14087_Transcript_23/66_Confidence_0.309_Length_1867
TTTTTAGTTTTTAAATCTGAAGAAGATTTCAGACCGAGATTTGAATCATTTGATCTATCCATTTCTGTTCCAATTTGTCAAGATGTATATCGCATCTGTT
CTTCTCGTTCTTGCAGTGCTAGCGGTTTTTACCTGTTCGGGTGGGAGCACAAGTTGAGATCTTTAATTCTAGCATGGGTTCCTTTGGGGTGAACTGGAAT

CAGAAGAACTCCGCGGTGTGGGATTGGGAAAAATTAGCTCCATCTGTTCCAAATGCAATCGGAAACCCCAGGAACGGAGCTACGGGGCATGGATCTGTGA
ATTCATCTGGTGGCACTCTCACTTCTAGCTCAGAGCTAGGGCATGGTTCATCCAAGAGCTCCATGTCGGCATCCATTGATTCACCGTCCAAAGTAGGGAA
CAGCTTGGAGTTCAATTTTGCTGCTGTCAATACGTATGGTAAGAACATGGATAAGGATTGTAGAGTTGACGACTCAGGGACTTGTCCATCGTCCATGATA
GCATTCAGCCATGGAGAGCCATTAATCAGTCTTAAGCTTGGAAAAAGGACTTACTTTGAAAATGTCTGCGGAGGACAGGATTCCAAGAGCACTGCACCTT
CTACCAGGACTTCTCCATCAACCGTTCTCAAGAAGACCAAGGTGTCTCAGCAGAATACACAAATGTCATACTGTCAGGTTGAAGGATGCGGAATTGATCT
GTCCTCCGCTAAAGATTACCACCGCAAGCACAAAGTCTGTGAAGCTCATTCTAAAGCCCCGAAGGTTGTTGTTGCTGGTGTAGAGCGCCGTTTTTGCCAG
CAGTGTAGCCGGTTTCATGGCTTAGCCGAGTTTGACCAGAACAAACGAAGTTGCCGTAAGCGTCTTACTCATCATAACGCACGAAGGAGGAAACCACAGG
CAGATACAATTTTATTCAGTTCATCACGTCTCTCAACAATGTTTTATGATACAAGCCAGCGGACAAATCTTTTCTGTAGTCAGCCTCTTTTTGGCCAAGT
GAGAAGCAATGCAGTTTCTTCATGGGATAACTTGGGAAGCTTCAATTTTATGGAAACAAAACATCTGTTGATGCAGCCAACGAAAACGGCAGCCCCTGAT
GAGCTGCATTTCTCAACCCCCCAGATGTCAAATGGTGTTATGGTTCACGCTGTACATCATCATAATGTCGGTGAGTTCATGCCATTCAAGGATACCACCA
CAAAGATCCTCAACCAAGGTGTGGAAGCTTCTGCGATTGGATCCAACTCAAATGGAGACCCGGATCTTGGGCGTGCTCTCTCTCTTCTGTCAAACGGTTC
TTGGGGTTCAAGTTCAACCGTCATCCAGCAGCCTAGTTCTCATGTGCACACTGGTGCGGTGCCGCCCCTCGCCACCGTTGCTGTCTCCAACCCTGCAATG
CATCCTCTGGACTCATCCCCGGGAGGATTCTGGCAAGACGTCCCTCCCCCGCTCGATGAAACTCCACATATTCAGGCATTCACGCACCTCTGATCAAATG
CTGCATCCCCTGGGAACAGCAGGTGGTTATACCTTCCGTAATCGTCCTAGGTTTGCTTTGCCAGTGACTGGCTCTGCACAATGTCGATCCATCACCGGAG
GCATTTGGTAAGGTGAAGACTAAAGACTCAAGAATAACCGGAGACTAACTCGCTTCTCTTCCAGGCTCAAGACTGTTTGGGTCCAGCGGTGTTGCTACCT
GATCCTGTTTTCGGTTCTGAGTACATCCATTACTAATCATGGGATTACTTGTTTAGGCCAACCAGACATATTCTGGGGTGAGATTACGACATGTTAATTT
ACATTGACGGTGTTTCTACATGTGTTAGTACTAATGATGTTATCGCTCTTGGCCCCGTTACGTTTCA
>comp79373_c0_seq30
GAAACCTGGTAAACCCTGGCCACGGACGCGCATGCCATCCAAGATGACGTAGTCCACACCGAATCTTCCACGTCCGCACACGCGAGCACAACGCCACCGC
AGGCCAAGTGCCGTCTCCACCACAACTCGCTGATCCCTTCCTCCTCGCCGGCTCTCTCTCCATCCCTCGTCCTTCCCCATCTGTCCCTCCCTTGGCTTCT
TCCTCCAAGCTCCAACCAATCCCCACGCGCGCGCGCGCTATAGCCTACAAATAAATACTCGCCACTCTTCCCTTCCTCCCGCCTATCGTCTTGCCAAGCG
CTAGAGCAGCTGCGCCATTGCACGCTTCCGGCCCGTCGTCCTGCACATCTGTCCTCCCCGTACTGCCTTGTACAAACTCCGTATGTAAACTACTGGGTTG
TCGCCAGTGTTCGTGTTTGCTGATGCTCATCTAATTATCGTCGAGGAGATCCAAGGAATTGTCCGCGGAGCGCCGGGGAACAAAGGCTGCGCGCGTTTCA
AAATCAGTTATTGAAACGGCAAGATGGTTAATTTTGGAAAGAAGTTGGCTGCAGATCAAGTAGAAGAATGGAAAGGGTACTATATCAATTACAAACTGAT
GAAGAAAATGTTAAAGCAATATGTTCAACAGACCCAATTAGGCGGGAAAGATTGTGAACAAATCCTTAAAGAGTTCTCGAGGATTCTTGATGACCAGATT
GAAAGGATTGTGCTTTTTCTGCTACAACAACAAGGCCACCTTGCCAGCAGGATTGAGGAATTGGGAGAACAACGCACTGCTCTTATGGAACATTATGATA
TATCACAAGTTTCCCAGCTACGTGATGCGTATAGAGAAGTTGGGAGAGATCTTATTAAGCTTCTGCGCTTTGTTGACATGAATGCTACTGGTATACGCAA
GATACTAAAGAAATTTGATAAGCGTTTTGGCTATAAGTTTACATATTATTATGTCACCACTCGTGCAAATCATCCCTATTCTCAGCTTCAACAAGTATTT
AAGCAAGTGGGAATTGTAGCTGTTGTAGGTGCATTATCGCGCAATCTTGAATATCTGCAACATCATCAAGGAAGCTTTGTATCCATCTATGATCATCCAT
CAGTTACCTTGAAGGACCCTATAATAGACGAAGTAAACCATGCGGTACAGAAATTTACGCATGCCACCAATTTTATGAAATTCTTGGGACAACACGCGCT
TATTGTTCCAGAGGATGCACGAAGCGGGTCTGAGGATCTTGTTGATGATCAGAGCTACCATTTCATGTCTCTGCTGCTTAACCTAGCGAACACTTTTCTT
TACATGGTGAACACATATATCATTGTGCCAACTGCAGATGACTATTCAGTAAGCCTTGGGGCTGCTGCGACTGTCTGTGGTGTAATTATCGGATCAATGG
CAGTCACTCAAGTGTTCTCCTCGGTTTATTTCAGTGCATGGTCAAATAAGTCATACTTCAGACCACTTGTATTCAGTAGCATTATGCTATTTTTTGGGAA
CCTGCTATACGCATTGGCATATGACCTGAATTCATTAATAGTTCTCCTGATTGGACGACTACTATGCGGGTTGGGCTCTGCAAGAGCAGTGAACCGTCGC
TATATTAGTGATTGTGTGCCTCTCAAAATCAGGCTACAAGCTTCTGCAGGATTTGTCAGTGCTAGTGCTCTTGGCATGGCATGTGGCCCTGCTCTTGCTG
GTTTCCTCCAGACAAAATTTAAGATATACTCGCTCACTTTTAATCAGAGCACATTGCCCGGATGGGTCATGTCCATTGCTTGGCTTCTTTACTTGTTGTG
GCTGTGGTTTACATTCAAAGAGCCAGAACACTTCGCTAAAACTGTCATCAATGCACAGCCGTCTGAATCAGGTCACCAAGGAAGTGCTAATTTGGAGGAA
GGTCTAGCTCAACCATTGCTTCTGGGTACAGAACAAAGACAGGGCGAGAATGCGGATGACAATGATGATAATGAAGTAGACTCTGAAAACTCTCATGAAC
CAGCAACATCAATTACTTCAGCATACAGATTGCTGACACCATCTGTGAAGGTCCAGCTATTGATATACTTTATGCTCAAGTATGCTATGGAAATTTTACT
ATCTGAATCAAGCGTTATCACAACATACTATTTTAGCTGGTCTACGAGTGCTGTGGCTATCTTTTTGGCAATTCTTGGATTAACTGTCCTTCCAGTAAAT
GCCATTGTTGGAAGCTACATTACAAATTTGTTTGAGGACAGGCAAATTCTGTTGGCATCTGAAGTCATAGTTCTCATTGGTATCATCATGAGCTTTCGTT
TCACCCCTCACTACTCCATTCCACAATACGTCACTTCAGCTCTCATCACATTTGTGTTTGCTGAGGTACTCGAAGGTATGTATTTTGTACATGTGTGCAA
CCATCTCAAAGCATCATGCACAGTAAGACATGAAAACAATCATACGCTATTGCTATAACCATCATACAATTTCTGATGACGAGCAATGCAGGAGTGAATC
TGTCCTTGCTCTCACGAGTAATGTCGTCTAGGCTTTCGCGAGGGACCTACAACGGTGGACTCCTCTCGACAGAGGCCGGGACATTGGCCCGTGTAATTGC
AGACGCGACTATTACTGCAGCAGGTATCTAGGCCCGGACCTCCTCCTCAACGTCACCCTGCTGCCACCTCTAGTGATCTGCATAGTGTCCATCGTCGCA
ACGTTCTGCACTTACAACAACCTTTGCTGATAAAGCAGCCCCTCTGTGTCCAGCCAGTTAAAATGACTCCTGCCATATTGGGCCGTACCGTACTACTTGA
AGTAGCATCAGCGTTGTAAATCTGGTAAATCTACAAATACCATGGACGGTGCCGGTGTGGCGGTGTGTAGTGTATTGTTATGCATTCAACAACTTTCTTG
TGTCTAGTAGAGTTAAATATTCGTCAAATTGAAAATTCAAATGCATTAACCAAGGAACATCACTGTGTTGGAAAGACAAGATGATTTATTGCAA
>comp65473_c0_seq6
GCCAAGAAGAGCCGCCGCGGCCCGCGCTCCCGCAGCTCGCAGTACCGCGGCGTCACCTTCTACCGCCGCACCGGCCGCTGGGAGTCCCATATTTGGGATT
GCGGCAAGCAAGTGTACTTAGGTGGATTTGACACTGCTCATACTGCTGCAAGGGCGTACGATCGAGCTGCCATCAAGTTCCGTGGCTTCGACGCCGACAT
AAACTTCAATCTCAGTGACTATGAGGATGACATGAAGCAGATGAAGAGCCTGTCCAAGGAGGAGTTCGTGCACGTCCTGCGACGGCAGAGCACCGGGTTC
TCGCGTGGCAGCTCCAAGTACAGAGGCGTCACCCTGCACAAGTGTGGCCGGTGGGAGGCTCGCATGGGTCAGTTCCTTGGCAAGAAGTAAGAACACTTCA
CAACCTGAATTACCTAATACTAGACGTGTTATTTCTATACTGCAGATCCTTGTGATTTTTCCTATCTCAGTCTCATTCTAGAGTTATGAACCTAATACAT
GAACTCCCATGATTTAATAGTCTAGAAATAGTAGGTTAGATTGATGTGGCATAATGGTTGTTTGAACAAAAGAAATTAAAGAGAAAAAAGGCAGTGGATA
GGGAATTCCTCACCGGTGGGAACGCCATGCTTGGGTGCAGGTACATATATCTTGGGCTATTCGACAGCGAAGTAGAGGCTGCAAGGGCTTATGATAAGGC
CGCGATCAAATGCAATGGTAGAGAAGCCATGACGAACTTCGAGCCTAGCACGTATGATGGGGAGCTGCTGACTGAAGTTGGTACTGAAGGTGCTGAAGTC
GATCTGAACTTGAGCATATCTCAACCAGCTTCTCAAAGCCCGAAAAGGGACAAGAATTCCCTTGGTCTGCAACTCCACTATGGATCGTTTGAAGGCTCGG
AATTGAAAAGAACAAAGATTGATGCTTCTTCTGAATTGGCGGGCCGCCCCTCATCGGTTCCCTCTTCTGACCGAGCATCCACCAATCTGGACTGCCCAGTC
TCACCCCTTCTATTCAAATAATGAGAATGCATCAAGAGATCTTAACAGGAGGCCAGATCAGGGATGCACAGTAGGGGGTGTTCCCAGCTGGGCGTGGAAG
GTGACCGCCCCTCCTCCCACACAGCCATTGGCGCTGTTCTCGTCGTCATCGTCGTCCGCTGCAGCATCATCAGGATTCTCCAATACCGCCACGACAGCTG
CCCACACCGCCCCATCGGCCTCCCTCCGGTTCGACCCGCCGCCGCCGCCGTCCAGCCGCCACCGCTGAACGTTAAGAAGCCACGCTGTAAATTTGCCAGG
AAGCCGACATTTTTTCCTCTCTCGGCGTTGCAACTTTTTAGGTTTTGCGCCGGGGTGGTTTCTTGTAGTGGAGTGGATTCATAATTAACTGTATTTGCAT
GCTGCCCATGTGAAATGCTTCTCTCTCTTTTTACGCTCTCTGCACCGGATGTTCTGGGAGTTCTTGTTGCCGGGAATTAACTAAATTCACCGTCTGAGAT
TGATCTATACATTGTTGTAGAGAATCGAATCATTGTTGAAATTAGTTACAGAATTATAGATATCATAAGGCACCCTCTACTTGTGGACAAAGTTCAGGAA
GATTATTCCGGTTCTTTTCTTGGCTGTCAATTCGGCTCTTGTCCTTGGTGTGGAGCCACTTGCCTACTGTCTTGTTCTGTTGCTGTTTGTTTTTGGCTTC
TTGCACAGTTACAAATTTGTTGGCTCGAATGGATGGTGAGACATT
>Locus_5717_Transcript_35/37_Confidence_0.186_Length_1638
TTGCTTTCGGTCGCTCGCCGGAGCTCCGCTCTTGCTCTCCATTGCTCTGATTGGCCTCTGCGAAGCTGCAATCTTTCGCCTTCCTGCGGTTGCTTTCTGC
AAAGCTGCCTCTTTGCTCCTTCTTTGATTATTATGCTATAATTGCGTGATTAGTCGATTAATTTGCATAAGCTTGTGTTTTTACTTGTTCACTGTTCGTC
CTTCGATTGAAGCTTGGTACTTTACTTTTGTGGCTCTGCTGTGCGTGTACGGCATGTAAAACTGCTTGTGCGGTTGTGGTTTTGGTACGGAGCTGCGTTT
TTCCTGATCAGGGAAGGAAAGATGCTGCCTTTCCACTATTAGTACTGCTGTACGTGTGGAGAATCGTACGCATGGACAAAATGGCTGTGCAAGTATAATG
CTTGTTTCCACTTTCCAGTTTTGGTATGGCTCTTGTCATGTATGTATGCTTTTGAGATGATACTTTGTTGTGTCTGTGTCTATTGTGTTTCAATTACTGT
GTGCGGATTGCAGATATGTTTTCTGTATGCACTTTTAGACGGTAATTAACCATAGTTAGATCTTATAATGTGTACACAGTTTTGCTGATTGCTTGGATTT
GGTAATCCAGGTTCCATGCACTCTCGGAGTTTGATCAGAAGAAGAGGAGCTGCAGGAGGCGTCTGTCCGATCACAATGCCCGTCGGCGGAAGCCTCAGCC
AGATGCATTTGCCTTTGCCTCTGCAAGGCTACCGTCATCCTTATTTGATGATAGGCGACAAATAAGTTTTGTCTGGAATAAAGCTCCTCTTAGCCATGTA
AGACCTTTCACTTCTTCTACATGGGACAGCTCATCTGACTTCAAGCTTCCACATGCGAAGGAAATAAGAGAGCTATCAACAGGAGTTGGGACTATTACTG
GACAATTTCATCTGGATAAATCTCATCTGTCCAATGCCATACCAACACTTAGCCATGGCAAAGATGAGCCGTTACCAATGAAAGGTCCGGACACATCTTT

AACTGCTTCAAAATTCGATGGAGCACCGGATCTTCAGCGTGCTCTCTCTCTTCTGTCAGCTGGCTCTTGTGGATTGCCTGATCCTGTACGGCAAGCATCT
CGTCTTATCCAGTTCTCTGGTGCCAGCGAGAATAGTGGTGGCCTTCATTCATCACATGGAGGGAACTCTGCTCCTGTCTCATGCGCTGAGGAACAGCACA
TAGCACCTCAGTCTCAGCTAGTTCGTTTTACCATGGATGCCAACAGCAATGGCTACGAGTCCACTTACTTTGGTGTAAACCAGATAAATTAAGCGTGAAA
GGCACACGCTTCTTGATCATTTGGAGACAGCCACGAAGATGGCCTGGACTTATGCAAGAACAGCTTCTATCAACTACCAGAGTGCAAAACCCCTCTGTTA
ATGTTATCTACATATTTATTCGCCACCCCTGAACTAAGTCGAACTGAGCGTAGGCCAGCTTGTATTGTGTACGCAGTTCCAGCCTGTGATTGACCCATTT
TTTCTGTTCTTTTCCCCTTGATGTGCCTACTTTGAAACTTCGAAGTGTGATATTGCTACACTTGTACAACCCACTGCCAACTTTTGGATCTCTTCGAAAC
CTCGATGAATCTGAACATGTATTTTTTGTGTGTGTATG
>comp64705_c0_seq5
AGGAGAGATAAGGGCCAGCAGTCTTGCCTGGTTTATTCAGTGTAATTAGACTAAGGATTAGGGGAGCAGACAGAGGAGAGATAAGGGCCAGCAGCCAAAC
AGTCCATCAGAAAAACCAGGCAAGACTGCAAGAGAGCAAGAGACCGTGTGTGTGAGAGAGAGGGGGAGAGAGAGGCTTTGCAGAAGAGAGAAGCAAGGTG
GGAAAGAGGCAGCAAGGGAGGAGGGCTACAGGAAGGGGAACATGCTCCCTTCTCATCTCACAGTATGAACTGAAAAACAGGAATAGAGAATTTCCGTGAG
ATGAGACAAAATGGCACCATAATGATTCAATTTGGTCATCAAATGCCTGATTACGACTCATCAGCTACCCAATCGACCAGCGAGAGTCATCAAGAAGCGT
CTGGAATGAGTGAAGGAAGCTTCAATGAGCATAATGATCGATCAGGTAATCTTGATGGTTACACGAAGAGAGATGAAAATAAGATGATATCAGCTTTATC
TCTGGGCAATCCGGAAACTGCTTATGCACATCCAAAGCCTGACCGTAGCCAGTCCTTTGCCATATCATACCCGTATGCTGATTCATTCTATGGTAGTGCA
GTGGCGGCTTATGGCTCGCATGCTATTATGAATCCCCAGATTGTGGGCATGATGTCATCTTCTCGAGTGCCATTACCAATTGAACCAGCTGCTGAAGAGC
CCATTTATGTAAATGCAAAGCAATACCATGCGATTCTCCGAAGGAGACAACTCCGTGCAAAGTTAGAGGCCGAAAACAAGCTGGTGAAGAGTCGCAAGCC
ATACCTTCACGAGTCTCGGCATCAGCATGCCATGAAGAGAGCTCGGGGAACAGGCGGGCGGTTTCTCAACACGAAGCAGCAGTCAGAGGCTCCTGGCGGC
GGCACCTCGGACGCGCAACTCATGCCACCAAATGGTGGCCTGTTCGCGAAGCACGAGCACAGCTTACCACCCAGTGATCTCCATTATCGCGCAAGAGGGG
GCGCTTAAGGAGTCCCATGGCAACTCATCCTTGGCTTAAAAAAAATGTGTGGCGCTCAGCAATTCGACCTCTGCTAGTTCAATGTAAAGGTGTGGGACTG
TAACTGTGGGTGCTTGAATTGGCCGCAGCCATTCCTGCGACGCGTCGCAGGTGTTGCCGGTTCTGTGGTCGTGCTAGACTTGTGACCGGCACGACGAAAC
TGGCACCTGTTTCCTGTAACTGTTGTCTAAGATGATGATGACTACTCCAATAACCATTCTGTATGATGCTTTGACGTCGGTGTTGCACCG
>Locus_12535_Transcript_54/73_Confidence_0.264_Length_2167
ATCTCCAACTATGGCAGTGGTGACAAGGAGCAAATTTGCAATCTTAGGGTGGTTCTCGTCGTGAAACCTGAAAGTTTGGGGAAATAAATTTGGGGGTGTG
GATGTCATGATATGTAGGGTCCGGCACTGAAGTGGTTGATGCTGTGGTTTCCAGGTTAAGGGGAGAAGAGGAATAGGATATAGATCTCAGGAAGCCAGAG
ATGAGGTCGAGGGGGAGCCAGTGGCCAAGCTTGTGCTCCAGTGAGCCTGATTCTCTTGTTCCCAGCTGATCGATGTGCCACTGCCTTGGTTCGCTGGATC
AGCGGATCTCCATGGTTCTGTGCGGTGCTGCAGATTGAGAAGAAGGTTTTCCAGGGGGAGGAATCTCTTGTGGGTTCCTTTTTCTGGGAGCAACGTAGTG
GGAGGTAGCTGCTTTTGCATACAATTAGGAGCAGCAGTTGAGATATTCAATTCGGGCATGGGTTCTTTTGGGATGGACTGGAACCAGAAAGGCTCGGTGT
TGTGGGATTGGGAGAATTTGCCGCCGATAGGCAGAAATGCAAACGAGAACCTCAAGATTGCGCCACAGGCTGAACCGAAGTTTGCAGGTGTTGAGGCAAC
AAGGCATGAATCGGGGCATTCTTCTTGTGGTACTTTCTCTTCCAGCTCGGAGATGGGGTATGGTTCATCCAAGAGTTCCATATCAGCGTCGATTGATTCT
TCACCCAAGGTGGGAAACAACGTGGAGCTCAATCTTGCAGCTGTCAAAGTGCCTGACAAAAACACCGTCAAGAACACTGATTTGGGTAAAGTTGATGACG
CTGGAACTTCTCCATCATCGATGATAGCCGTCAGCAGTGGAGAACCGGTGATTGGCCTGAAACTTGGCCAAAGAACCTATTTTGAAGATGCATGCGGAGG
GCAAAGTGTCAAGAGTTCACCATCCAATGTGAGTGCAGTGACCCCTGCTTCTGCGAAGAAAGCAAAGATGATTCAAAACGCACAGAACTCGTACTGTCAG
GTTGAAGGTTGCAAGTTTGATCTCTCTTCTGCTAAAGATTATCATCGGAAGCACCGAGTCTGTGAAACTCATTCTAAGGCTCCCAAGGTTATTGTTGCTG
GTCTGGAGCGACGCTTTTGTCAGCAGTGTAGCCGGTTCCATGCTTTAACTGAGTTCGACCAGAAAAAACGAAGCTGCCGGAGACGCCTCAATGATCACAA
TGCCCGCAGACGGAAGCCACAGCCTGAAGCAATTCCTTTCGGTTCATCAAGGCTCTCTGCAATGTTTTATGATGCAAGGCAACAGACAAGTCTTCTGTTT
GGTCAAGCTCCTTATGGTCAAATGAGAAGCTGTGCAAACTCTTCATGGGATAGCCCAGTAGGAGGCTTCAAATTTGGAGAAACAAAAGCTCCTTGGTTAA
AGCCAACGAGAGCTGCAGGTGTTGATGGGATGCATGTATCAAGCCAGCAGGTGTGGAACAATATTACGCCACACGGTGCACATCATGATTTTAATGGTAT
CATGGCTTTCAAGGGAACCAGTGCAAATGTCCTTAATCAAGGTGCCGAAGCTTCTCCGGTCGTCTCCAACTCGAATGGAGCCCCAGATCTTCAGCGTGCT
CTCTCTCTTCTGTCAAACAATTCAGCTGGTGCTGCCAATGACCAGCCAACTCGTCAGCTGCACTCTGGGCTGACCACCCTTGCCAGCACCTCCAGCCCTG
TCACTGTGATGCAAGCCTCACCACCAGGACTCTGGCAAGACGGCACGCCCCTCGATCATCAGGCGCGGTTTCAGTTTTTTGATCCCCTGGGCGACGGCAG
TGCCATTGCCACACCGCATCAGCTCCAGCTCCTAAAACCCTTCGCCTACGACAGCTCCTCGTCCCACTATGACCAGATGCACTGATGCTTCCTTCAGTTA
GCGCAAACCGCGGTTCAGTTGCGCGTAATCAGACATGGTTTCCACTGTCTGAAACTCTGAATTTTGAAACTGCGATGTTCTGAAAAATAAAAGCCTGCGT
GCCGCAAACTCAGGTTCGAGTTGATCTAGTAGTGTTCCCGTTCAGGGGCTTGAATAATCCATCCCTGTGATGGCGTGTAACACAAATGCTTCGATTGCGC
CGGCAATTGATCATTGACTCTTTTCGTGTTCTTGATAACATCTCAATTACACCTTGAATGGAACACT
>Locus_2827_Transcript_126/166_Confidence_0.228_Length_3355
CTCTCTCTTTCTCTCACTCTCTCTCTACCTGTGTTGCTCGCGACTGCACGCTGCTGCTGCGGCTGCCGTTCCACGTCTCTGTCTCCGCTTTAAGGCTGTC
ACCTTTCCCCCCTCGCTCTCCTTCTCTCTCCTCAATTACGACGCCACAACCAGACCCAAACCCTAGTACCACGCGCCGGAGTAGGTAGGTGGTGCTGCCC
CTTGATCCGATCCACCCGGCGCTGTAGAGCTTTCCGGCTTGTGCGTGCTCACCGGCGATCGATGGGAGGCGCCGCGGGAGGCGGCTCAGGCGCGGGGAGG
CTGTGAGGTCGGTGGGTGTGCTGCGCGTCTGTTCCGGCGGCGCTCCCTTCGTCTTGCGGGAGCCTGTCGGTGTGTCTCGGCGGCGTTCGCTTCGTTTGCA
GAAATCTTCACGAGCTGAAAGTTGAGGCGAAACCGAGGAGCTAAGAGCAGCAGTTGAGGTGTTGAAGTGCATAGGACAACTAAGAGGAAGAGGAGGACGA
GATTTGTGTGGTTGGGCTTTTGCATTGGCGAGGGAACAATGGCTGCGGCGGTGGCGATGCGAGGGGGAAGCAGTGACAGCGGAGGGTTTGATAAGGTTCC
GGGGATGGACTCGGGGAAATACGTGCGGTACACCCCGGAGCAAGTTGAGGTACTCGAGCGGCTGTACATCGATTGCCCCAAGCCAAGCTCCTCGCGGCGG
CAGCAATTGCTGCGCGAGTGCCCTATACTTGCCAACATTGAGCCCAAGCAGATCAAGGTTTGGTTCCAGAACAGAAGGTGTCGCGATAAGCAGCGGAAGG
AGTCTTCGCGGCTTCAGGCTGTGAACCGGAAGTTAGCGGCAATGAACAAGCTTCTGATGGAAGAGAATGAGCGTCTTCAGAAGCAGGTCTCTCAGTTGGT
TCATGAGAATGCACACATGCGGCAGCAGCTGCAGAATACTTCTTTGGCCAATGATACAAGCTGTGAATCAAATGTGACTACCCCTCCAAACCCTATAAGG
GATGCAAGTAACCCTTCTGGACTCCTTTCGATTGCAGAGGAGACCTTCACAGAGTTCCTCTCAAAGGCTACTGGGACAGCTATTGATTGGGTCCAGATGC
CTGGGATGAAGCCTGGTCCGGATTCGGTTGGTATTGTGTCTGTTTCACATGGTTGCCGAGGTGTTGCTGCCCGTGCCTGTGGTTTGGTGAATCTAGAACC
AACAAAGATTGTGGAGATCTTGAAAGACCGCCCATCTTGGTTGCGTGATTGTCGGAGCCTTGAAGTCTTTACAATGTTACCAGCTGGAAATGGTGGAACT
GTTGAGCTTGTTTGCATGCAGATGTATGCGCCTACTACTTTAGTTCCTGCACGAGATTTTTGGACGCTGAGATACACAACCACTATACAGGATGGCAGTC
TTGTGGTCTGTGAGAGATCTTTAAGTGGTTCAGGAGGTGGTCAAAGTACTGCATCGGCACAACAATTTGTTAGAGCTGAGATGCTTCCTAGTGGCTATTT
AGTCCGTCCATGTGAGGGCGGGGGCTCAATTGTGCATATAGTGGACCATCTGGACCTTGAGGCTTGGAGTGTTCCTGAAGTTCTTCGGCCGCTCTACGAG
TCGTCTAGGGTAGTTGCTCGGAAGATGACAACTGCAGCACTACGGCACATCAGACAAATTGCTCAAGAAACAAGTGGAGATGTCATATATGCCTTGGGGA
GACAACCAGCTGTTCTACGAACATTTAGTCAGAGGCTCAGTAGAGGCTTTAACGATGCTATAAGTGGTTTCAATGATGATGGTTGGTCTGTCATGGGTGG
AGATGGCGTCGAAGATGTTATCATTGCTTGCAACTCAAAGAAGATAAGGAACAATACCAATCCTGCAAGTGCTTTTGGAGCCCCTGGTGGTATCATATGT
GCTAAGGCATCCATGTTACTGCAGAGTGTTCCACCAGCAGTACTAGTTAGATTCTTGAGGGAACATCGGTCTGAATGGGCAGATTATAACTTCGACGCAT
ATTCGGCCTCGGCGTTGAAAACAAAACCTTGTTCCCTTCCTGGGTTGCGGCCTATGAGATTTTCTGGGAGCCAGATCATCATGCCACTTGCTCACACAGT
GGAAAATGAGGAGATCTTAGAAGTTGTTCGTCTTGAAGGACAGGCTCTTACACAAGATGAAGGTCTTTTATCAAGAGATATTCACTTGCTTCAGCTTTGC
ACTGGAATAGATGAGAAATCTGTGGGGTCCTCCTTCCAGCTCGTGTTTGCACCAATTGATGAACATTTTCCAGATGATGCTCCATTGATTTCTTCTGGCT
TTCGTGTCATACCACTTGATATGAAAACAGACGGCATATCTTCTGGTAGGACGTTAGATTTGGCATCTAGTCTTGATGTTGGTTCTACCACGCCCCACGC
CTCAGGAGATGCATCACAGGATGATTGTAATTTGAGATCTGTGCTGACAATTGCCTTTCAATTCCCTTACGAGATGCATCTCCAAGACAGTGTTGCAACT
ATGGCCCGTCAGTATGTTCGCAGTGTTGTTTCTGCTGTGCAAAGAGTGTCGATGGCTATCTCTCCCTCCCAATCTGGTATAAATGCTGGGCAGAGGATGC
TTTCTGGCTTCCCTGAGGCAGCCACACTTGCTCGCTGGGTTTGCCAGAGCTATCATTACCATCTAGGGGTAGAATTACTTAACCAGTCAGATGAAGCTGG
CGAAGCATTGTTGAAGATGCTCTGGCATCATCCAGATGCTATTTTGTGCTGCTCTTTTAAGGAAAAACCTATGTTTACATTCGCCAACAAGGCAGGGCTT
GACATGTTAGAAACGTCCCTTATTGCCTTACAAGACCTCAAATTAGACAAGATCTTTGATGAGTCTGGAAGAAAAGCGTTATTCTCAGATATCTCTAAAT
TGATGGAGCAGGGCTACGTGTACCTGCCATCGGGCGTGTGCATGTCAGGAATGGGTCGCCATGTTTCGTTCGACCAAGCTGTGGCGTGGAAGGTCCTTGG
TGAAGACAGCAACGTCCACTGCCTGGCCTTCTGCTTCGTGAACTGGTCCTTCGTGTGAGCCTTTGCCATCCTCCGAATGCCGACTTGATCTGTGACGAGG
AGATCCATTTTTGTATGATCCAGAGAAGGAAAATCATCAACTGTGGCAAGTCTTAGTTTGCACTAGCTCTAAGTATGGGAATCATCAACTGGAGAGTATG

CAAATCTGATCTCGTTTCATTTGGCTGCCAAGCCGCGCCCTGGCTGCTTGTTTGGGATATTGTGTTTCCCATTTTATGCGTCGAACTGTGGTACATTGCT
TCAGTCTTCAAATGGCAGCTGAATTTTCAACTGGTTCTCTGTTGAGATGAAATTT
>Locus_9469_Transcript_56/63_Confidence_0.151_Length_1190
CAGTGCAATTAGACTAAGTATAGTTGGTGAGAAGACAGGAGTAGAGATAAGGGCCAACAGCCCAACAGAAAAACCAAGCAAGACTGCAACTGCAAGAGAG
ATGGGGAGAGAGAGGAAACCTTGCAGAGGAGAGAAGCAAGGTGGGAAAGAGGCAGCAAGCGAGGCGGGCTGCAGGAACGGGAACATGCTCCCTCCTCATC
TCACAGAAAATGGCACAATAATGATTCAATTTGGTCATCAAATGCCTGATTGCGACTCATCAACTACCCAATCAACCAGCGAGAGTCATCAAGAAGTGTC
TGGAATGAGTGAAGGAAGCCTCAACGAGCATAACGATCGATCAGGTAATCTCGATGGTTACACAAAGAGTGATGAAAATAAGATGATGTCAGCTTTATCT
CTGGGCAACCCGGAAACTGCTTATGCACATCCAAAGCCTGACCGTAGTCAGTCATTTGCAATATCATACACGTATGCTGATTCATTCTATGGTGGTGCGG
TAGCAGCTTATGGCTCGCATTCTATTATGCACCCCCAGATCGTGGGCATGATGTCGTCCTCTCGAGTGCCATTACCAATTGAACCAGCTGCTGAAGAGCC
CATATATGTAAATGCAAAGCAGTACCATGCCATTCTTCGAAGGAGACAGCTCCGTGCAAAGTTAGAGGCTGAAAACAAGCTGGTGAAGAGTCGCAAGCCA
TACCTTCACGAGTCTCGGCATCAGCATGCCATGAAGAGAGCTCGGGGAACAGGCGGGCGGTTTCTCAACACGAAGCAGCAGTCAGAGGCTCCTGGCGGCG
GCACCTCGGACGCGCAACTCATGCCACCAAATGGTGGCCTGTTCGCGAAGCACGAGCACAGCTTACCACCCAGTGATCTCCATTATCGCGCAAGAGGGGG
CGCTTAAGGAGTCCCATGGCAACTCATCCTTGGCTTAAAAAAAATGTGTGGCGCTCAGCAATTCGACCTCTGCTAGTTCAATGTAAAGGTGTGGGACTGT
AACTGTGGGTGCTTGAATTGGCCGCAGCCATTCCTGCGACGCGTCGCAGGTGTTGCCGGTTCTGTGGTCGTGCTAGACTTGTGACCGGCACGACGAAACT
GGCACCTGTTTCCTGTAACTGTTGTCTAAGATGATGATGACTACTCCAATAACCATTCTGTATGATGCTTTGACGTCGGTGTTGCTCCTT
>Locus_1826_Transcript_80/132_Confidence_0.423_Length_6004
GATGAATAATGAACCTTTCCCAGAATTAGCTCCACTTCAGAACATTTATGACTCGTATCACTTTGAGCAAGATAATCAAGGATCTGAGGTTGGTATTACT
CCTCAGGATTCAAAGATTAGTCATATCAGTGAAAATAAAGTCGAAGGAGGACTACATGAATTGCAAAATTTGTCATGTGCTGGTCAGCCTTTGGGTGCTG
TAAATTTATCCAGTCAAGTAAGCAATGAAACTCTATTGTCAGAAAGTTCTGACGGATTGCTAGAGGCCATTACGAATCCGGTTAAGATGATGCACATGAA
TGATGATACTTGTAAGAGAGCCAGTGGCACTCTGCAACCATCTTTTCCACCAATACAACATGCAGCAGAAGGCTTGAAAAGTTCAGTTGGCATGAACAAT
GAACTTGTAAAGGAGTTTGTTATTGGTTCCAACTCTGCTCTATCTAACCAATCTGAGGCAGACACGAGAAATTCTAGTCCTCATCTTGTTACTTCTATGT
CAACCGAAAGCAGCAAGATGATTCAATCTCCCAAAAGAAAGGTTGCTCATGCCACTGGTGTTCCTGAAGAAACAAAGAATGCTTGGGCCGATAGTACAAA
CATCTTCAGTGGTGATGAATCAAAACTTGGAGCGTTGGAGCACCATCAAGATTCTCTCGACAACCTAAAAAGTGATGCCATGGAAGAAAAGACAACCAGG
GAGGAAATGTCTGCAGTTTCAGGAAATAATGAACAAATGATGGAAAGTGGTTATGACGAAAATGCTAAATGTGCTACTGATACATCAAGAGATAAGTTTG
ACTCTTCGGACAATGCTGCACCTGACAAACTTTCAGATGACACTTTGGATAATTCAGAGGATCAGAACATTCCCTTAGTAAAGCATGAGGGGTCATTCAA
GGAAGGTGACATGCCTGCTTCAGTAGAAGAGCCTGAAAACACGGTCCTACCTGCTTCTGGGCCTCAAGAGAAAATGTCAGCACCATTGGTCAATTCAAGA
AGTGATATCGTTTCGACCACTGTTACTGGCACTTCCGGTAATTCAGAGGATAAAAATGATTGTTCAGGTGTTTTTCCTGATGATTCTTCTGCTGCTTTGC
CTGATGAGAAGGATTTGAAAACGTCCACAATGGATTATGAGAGGTCATTCAAGGAAGTTGCTAAACCTGCTTTAGAAGATGAAGACCACAATGTTATTTC
TCCTGGCTCCGAACCAGTTGGAGAACTATCAGCAGTTTCAGGGAACTCAGACATAGATGCTATTTGTAGTGGTACTGTTTCTGCTGCGCAAAAGGCGGAA
TACAAGGAGCAGGCTAATTCTTTGGGTGGTTTGACCACAGGAGAAACTGAGGATAAATCAGGCAACCATCCCGATGCTTATTCACAGAAATGCCAGACTG
ACGAATCTTTGGTACAATCTGAACATCGTGTAGATCCAGCCACACCATCTGCATTAGGCATCTCAACTGACAAGGCTGTTGAAAAGATCGTAGAAACTCC
ACTGAATGCAAAGGATGATTTGAATGCACATGTGCAAGATACAGTTTCAAGTCATTGTACAGATCATAGTCCTGGTACTGTTCCATCCCAAGGCAAGCTA
AGCTCAAGCTTATTAGAACCTGGGAACGGTGACGGAATCTGCTCTGATGCTACACGTGATTCCTCTCCAGTGATTAGTTGCACCGAGCCCTCTCCCCAAG
GTGGACCTGACAGCAACACTATGGTTCATCAGACACTTGATGAACATGAGGATTCAAAAGATCATGAAGCCTCAGTTGACGCTACGCAGAGCTCAAAACA
GTATTCTACCATACATGTAAATCCTACTCCTGGTTCTGAGGAGACTAATTCAGCGGGAGATGATAGAAGTTTCTCGTTTGAGGTGGGAGCTCAACCAAAT
GTATCTGAGAAAGCTCATAGCCCTGCTTGGAGCCCTTTCCCCAGATACAGAGCTTCTCAGAGTACTGAGGTAACCACAGAAAAACCTCAGGCTGGAAGTT
CATTGGAGAATAGTGGTGATGATAGTAAAAGAACATCTATTGTGAAGGCTGGTAAAGAACAACTGTCAGAAAGGAAAGTGAATGGAAGTGCCGGGGGCCC
CTCAGATAACTCTAATATTAGCGATAACACTAAAATTTCACCGCCGGAGCAGTCACAACAGCATCCAACCCCTGAGCGGTCTGATGTGGCGAATCTACCT
TTCACAGATGCGCAGCATTTGCAGCTACGTGCAGATATTTGTTTACGGAGCTCTTATCCAAGGAACACCACCAGGAGAGGCTTACATGGTGGCAGCTTTT
GCAGAACCTGGTGGTGGTAAGCCTACATGGGAGGTAGCTTGGCGAACTGCTCTTGAAAGATTTCAGCATCAGAAACCAATTTTTCCCGGCTTAGAGACCC
CCACAAGCTCACGTATAGCTGAGTCTGAAAAAGCTAGTAAGGGCACAACAGTTAGAACCGCGACAGCTAGCAAAAAAGGTGGGAAAACAGTGTTGCCAGC
ACATTCTGCTGTAGCCTTGCACTCGCCTACTTTCAATGCGCCTCTTGGTAGTTCCACATTCAACCTGCAACGAGGTACTCATCTGGATTTTAGCCCGGCT
GTCTCACCAGTATTTACATATAATTCACACACGAGGCAGCCTTCTCCTGGTGTTGCATCCTGGTATTCTCAGAGCCCAGGTCCACGTCCTGCACCCTGGC
TGGTTCCACCACAAAACTTAATATTTGATTCATCTATGCAACCGGCTGTGCCTACAAATGAAACTGCAAAAGGATCTTCTTCCAAAAATATATCAATTTC
ACATGCTGTTTCACCTGGGGTGTCCCTTCCAAGTCCAGCACCTTCTATTGTTTCTCCTCCGTTGGCAGTTGTACATGAGGAAAAAAAGAAGGCACCGGTT
TCTACTTCTAAGCATGGAACAACATCGCAAAAGCCAAGAAAAAGAAAGAAAGCTTCAGCAAGTCCTGAACAGCAAGCTGTCATCCCTTCTCCTCAGCTCA
AAACAGACACGGCGTTGTTTACTCCTGCTGCTAAGCATACAGGAGGCTTCACCTTATCTACTCATTCCCCAAGCAATGCCTTGGGCAGCAGGCTTATTTC
TAACACAGGTCAGATCACCTCTGTGCCTAACTACCAGATTACTGGTACTATGGATGCTGAGCAAAGAATCATCTTTTCAGAACAGATCCGTGGTGCAATA
GAACAATCAACAGGACAAGCCAAAGGTGCAAGTATGCACTCTATGGCAGCAGTTAGACACAAAGAAAGCATATGGAGCCACCTATCAACAATCTCAAGAA
ACAAGTTACCTCCAGAAGTTGAAGGGAAGCTTACCTCAGCTGCAGCTGCTGCTGAAGCAGCAGTTTCTGTTGCAAAGGCAGCTGCAGAAGCTGCTAAGAT
GGCGTCAGAGGCTGCCTTGCAGGCGAAAATGATGGCAGAGGAAGCTCTTAGCTCCTCTAAATCTGTAATTTCCTTGCAGAGTCATGAAGCTGGTGAGGTT
AATGTCAATAGCAATCCACCTAGCTTGTCAAGTTCAACACCCACATCATCTTGGAAAATTAAGGACAACAGTCATGCCCAAGGTTCCATCGTTTCTGTGG
CACGAGAGGTTGCTAGAAAGAGGGTTGAAGAGGCATCTGCAGCTACAAAACGTGCAGAAAACTTGGATGCCATACTGAAAGCTGCTGAGCTTGCTGCCGA
GGCCGTGTTCAATGCAGGGACCATTATTGGAATGGGTGAACCTCTACCATTTACTCTAAGCGATCTTTTGGAAGCTGGTCCAGATGGCTACTGGAAGTCT
GAGCGAGTGAGGAATAAGAAAGCTGAAAGTGTCACTGATAATCCAGTAACAGAAACATTGGAGGTAGATGTGCCTGCTGATCTAAAAAAATCTGGCCGAA
AGCGTGGCCGGAAACCTAAAGATCAAGCACTACTGAATGCAGAGCCATCTTCAAGTGGCAAAGAATTGCAGCCAGATGGAATGCACACAGATGGGGTCAA
AGATATTCCCACCACAACATCGCTTGGTGCTAAAAGTAATGATACAGAACCAATAAACATTATCTGGAATGGTATTGGAAAGGGATCTGCTGTTGAGGTT
TTATCTGACAAGGGTGGGTCTGGAGTAGCTTGGTTTTCTGCTAAGGTTGTTGATATAAATGAACATAGTGCGTTCGTCAGCTATTACTACCACAATGAAG
GAACTGGTCCTCGCGAAGAATGGGTGCCATTGAAACAAGAGGGTGAAAAGGCGCCTCAAATACGACTTGCTCATCCTGCTACCCTCTCTAAACTCACAAC
TAGGAAGCGTCGTAGGGAAACGGCAGGAAACTGTTCCTGGGCTATTGGAGATCATGTAGATGCATGGGTCAAAGATAGTTGGCGAGAGGGAATTATTGCT
CAGAATTATGAAGCCAATGGAACAAAATTTCTTGTACAATTTTCAGGAGGTGCTGCTGATTCCTTGGTTGTTGATGCTTGGAATACTCGTCAATCACTTG
TTTGGAAAGATGGCCAGTGGGCAGAGTGGTTCCAAGCGAGAGAAAGGAAATCTAAATATAATAAGGGTGATTCACCTCTTGAGAAACGCCGGAGGACAGG
CCTACAACAAGCAGGTGGTGATCTACCTATCGGTGGTGAAGCAGGAGGCCCTTCTAATGACACGATTACCTACAATGCAAAAAAGCCAGAGGAGCCACTG
GCTTTGTCTCAGACAGACATGACTTTCAACATCGGGAAGAGTGTAGTTGAGAATAAAACTGATGCTCTTGCATTCAAGCGGCCTGGATTACAGAAAGAAG
GATCAAAGGTTTTCTACGGTGTTCCAAAACATGGAAAGAAGAAGAAGTTTATGGAAGTAAGCAAACATTATGTTGCAGGCCAAGCTGACAAGATTTCTGA
GGGTAATGCACCTAACAGATTTGCGAAACATGTGGTGCCACAATTACCAAGACCGCGGGACAATACTTCCAAAGTTGATCATAGAGGAAGGAGAGTAGGC
GAGACGAGGCCCAGAATACCTAAACCCTCAAAGTCTCAGAATGTGGCAGCTAACAGTGTTCCTGACAAGGATCCTTTACCCTTGTCTCTCCCAAATTCTG
GTGTTTCTGAAAGGAGTTTTGCTTTTGTGGGAAGTACGGCAAGTGCTTCCAACACTGAGAAGCCCACTGTAGAAAAAAATAAAGCCGCGCTTGCTGCTGG
CCTTAGAACTGAAGATCCTTCTGTTTCTGAAATGCAAGTTGCATCAACTGTTCCTAGTTCCAAGCAGAATGTACCCACTACTAATCGAGCTAAAAGGAAA
ATTGTTCCTACTGCGGATAACTTGAACAGAAGTATCATCAAAACTTCTGAAAGGACTACTAGTTCTGATTCTGCTGAACCCCGAAGGTCCAACCGGCGAA
TTCAACCAACTTCAAGGTTATTGGAGGGTCTGCAAAGTTCTCTTATAATTTCTAAAGTTCCTGGTGATAAGGGTCCTAGAAGCATTTACAGAGGTCCTTC
GAGAGGAAGAGCTCATGGCTGAAAAAAGGCACCATAGTGCATTTTTTGCGAGACGGCTCTTCGCTTTGTACATAACCATAGCTTGCCGAGTATGGAAGCA
GAAGGATAGGATTGGAGTAAAAGGCTCTATTGGAGCACAAGGATTCAGATGTATTATAAACTAGCTTTTAAGTGTAGCTTTATTATGTTCCCATTTTCTT
TTGCTTGCACTGGACAGAAACTCTTCGAGTTGTAGAGTAGCCATTAATATTTTAGAACCCTCCTTTTAGTCTGTATGGATACACTGGGAAAGAAATTGGG
TGTGGTTTAAATTCGTCTGACGGTCTATCGTGTGTGTTGCTATGTGGTGAAGTGAGTGCTTGGACTCGTGGAGTGACATTTGCGCTACTTGCCTGATCAG
CCTTTTCTTTTGGGTACTGCTTGTTGCACTCGTGGAAGCGACATGTGCGCTACCTGTGCAATTTGCATTGTTCAAAATTTTTGGTAGTCCTTGTTGCGCT
AGTG
>comp80692_c0_seq16

GCTCCTTGAGAAACACCATCCAATTACAAGACTATTTGATGCAACAATCACGCATATGGCTGCGCTAGAACTACTGCTCTGAATTAAGAGGCGTTGAAAT
ATTGTCCGGTCTACATATAGGCACTTGAGGTGTATTGACAGACAGTTCCCCTTAGTTTGCTACAACACATCAGGCGAATTCCACCAAATTAGGATGATGT
TTGTTTCTTAGACACAGGATAGCAATGCCAAAAGTTTTTATAATTTTCCGCTTCACCGCAGATTTGGCATCTCAAAGGGAGTTGTGGCAAGATTCTCTT
GACCAAGCTTTCTTGCAGATTTGGCAAGAAAGATAAAAAAAATGTTGGTATACTTAAGCATCCTTAATAGAAACTAAACAGGTCTTAGATTGAAATTATC
ATAAATAAACCCTTTTTCACTTTAGCTGTAGTCCGGATGAAATCTTAGGGGCCTTGCTTAGCCTGCCCTGAAGCTTGCCAGTAAGTCGAAGCTGATGCTG
ACACAGCACTGAAATAGATCCCAATGAACACAAGTTATTTCGAGAAGATAAAATACCAAGCAAAGCCAGGATTGCATTAAAAGATAGTGTATTTCTACTA
TACGATCTGAAAGCTTAATTTATTATATCAATAATCAAATTGGCAGCAGTGTGGAACAGATATAAAGAAGATGCTTCAAATCACAAATAACTACAGATAA
ATTGAAGGGTGAAGGTTCATTCAACCACCAAACACCCAAAATGTCGAGCTGCATATGCACAGAACCTAAGTACGGTACAGTTCATAGTTTGGGAAAATAG
ACTGCAAATGTGCACAGATAATACAGATTGTTACTCGATGAACAGTAAGCACGGTATTTGAAATCCTTCTAACCATCCATCCAGGCAGTGCCTCGTATAC
TCAAACCTCTTTTTGGAGGAAGCCAAACCATGTGATCCTTTTCAACCCAAATACATATATTCAAGATGAAACTAACAAAAAGACAGTTGATAAAGAAACA
CGAAAGAGAGTAGAAAAAGGGTACCTGTTTCAAGTTCTCATCTTCTGAAAATGATCAGCAGTCAATAGATGTTTTAGTCCATTCTCCAGAAAAATGAGTG
CCGCATGAATCCTTTCCGGTTTATAGTGAAACATTTTAGACGATGACCTTTAACATTCTAGAAAACGAAGAGAAAACCTACAGTCATCTAATTTCAGCTT
AAACATGTTCCAGCAAGATCATCACCATCCTGACATGTTCCGTTTTGTCCCTAGACCCGAGCACAAGGTATTGCGAGGCTAATTTAGCTCAATTCAGTTG
TCAGAGCAATTGTGTGTGAAAAGTGTGAACAACAGCATAGGAGACAATACAGCAAGTGTAGCGTGCCAATATGTGTCCAGCTTGATAGTTCACTATAATA
AAGTTCTTTAATTCAAGTATTTGAATAATATAGAATCTCAACCTTCAAGTTTAAAAGGTTTTGTATTTGCTATTATAATCGTTGCCACCCCCTCACCCCT
CTCAAAAAGAGAGTTACTTTGTAGCGCACTGTAAATCTCTTGATTGAAATAGTGGCATGTCATCTTATTTAATTCAGTTATTTCATAAGGTACAATTTGT
TTAGCTGACAGCTCAGACATGCTTCTATGCAACATGTTTATACCAATGCTGTATTTTGGCAATCCTGGATGTGGCATTACTTAACTGTGCTTTCATATTC
AATAGTTCTGGCAAAGGGAGGCTGCAAGCTTGAGACAACAACTGCACAACCTGCAAGAAAGTCATCGGTATGGCTAGAAAACCCTGCAAATTTTACAAAT
GTACAAGTCAGAAAATTTGCCTAATGTAAAAGGATCAAACTACAGGATTTGTCAGCATGAGCACAAGTGCCTAACTTCCGAGTTAGATAACTCTGGAGGA
AGGAAATCCTTCCTACTGTTTGTTTCAAGAATATACAAAAATTCAAAACAACATTAGAATAGAACCAAAAGAAGAAACAATATTCACCATGTAGCAATAC
TCAATTCCTGGTTGTCTTATTTATAAAGGTATAAAACTAGGTTCTGACGAGACAATTATGAATCAACTACACCATGTTACAGATCATCCATAACTCTTGG
TGATGCAAATTAAGGGCTGTAAAGTGACAAGTATACCTCCACCAACTAATTTGTGCAGAGTGTATAAAATTGTAAAACAATGGATAAGAATGAATTTAGT
ATACAAGATAGCATACAATGCCCAAGTTTAAGGAGACATACTGATTTACTGACTCCATAGGACAATCTTTTGCTTATGAGGACATCAGTTTTCATAAACA
ATATCAATGTAAAAGGAGGTAACAGTTTATGAATTTCCTATGCCAAGCCATTTATAAGTCAAAAAATGTACCTATTTAGCTTTTATGCATTCCAATTAAC
TTCCAAAGAAGAAGAGTACATACTAATGGAAAACAAATATGATATTATCATCTGTTTTGCCCGGTAGTTTCTTGAGTCTCTGATTTGTCCGCACTTCACT
CCCAACCAACACAACATTAATACACATGGGCAGAGGAATATCAGGTTATCAGGTAAACTGAATACAGTTTGATTGACTGCAGTCAGTTGATGGGACAAGA
TCTTTCTGGATTGAGCGTCAAGGAACTCCAAAATCTAGAAAATCAGCTAGAAATGAGCCTACGTTGCATCCGGACAAAAAAGGTAAGGATGCTAGCCATA
TTTCTGTTTTTCGTTACCAGATTGCCTCATCCAAGAATACAACTGATGATGACGTTTTTTACTTTCAGGACCAACTCTTGATTGATGAAATTCACGAACT
GAATCGAAAGGTTCTGGAATATTCTGTTATTACTCTGCAATGAGAGGTCTCTGAGTGGATTTCAAGAAGCTGACTCTACTGGCTCATTTTCAGGGAAGTC
TCATGCAACAAGACAAGATGGAACTATACAAAAAGGTCAACCTAATCCGTCAGGAAAACATTGATTTATACAAGAAGGTATTTGATAAATCGTGAATGAA
AATTCACAGGATATACACCCCATCCATTATTGACTGAGGTTTATTTCTCTACTCAGCTCTACGAGAAAGAGGTGACAAGTGAAGTCAACCGAGATTCATC
AACTCTAAACTTTGCAGTTGTCGAGAACGTCAACATTCCTGTTCATCTTGAACTTAACACTCCACCGCAAGAAAATGATGTTGAGCAAACTGCACCTCCT
AAACTAGGGTAACCCTGCTTTTTTCTGCAAAGGAACTATTGATTCTGTTCTTAATTCTTTAGTGGCATATTGTTTACTACTAACTGAAAACTTGCAGATT
ACAACTAAATCCATGAAGGCATGCAGCATGGCACCGCTTATGTTCATTATTCTCAACGGTACGATTCAAAGCAACCAGAAACACTATTGGTTGTAAAGAG
CTTAAACACATAGCAAGACTGACGCGGTATGCATGCCATGAGTGATTGATAATGGTAGTGTAAATCTATTTCAAATAAAATGTATTTTATTGAAGAATAC
AGCAAATGATTTGTATTATAACTATTTGACACATTCTGCAAGTTTGTTGACCAACGTGAGTACATGATAGCTACAAATCTTATATTGACAAAAAGATATG
GTGTGTGGAAAAAATTCAGGTGCTATTTTTTTCTATTTTGAGAATTGTATGCATAGCAGAAGAGCACAAATTGCATTCTGGTTTATCCGTGCAGGCATTC
CAGAGTATCAGAATGGGGATCAACCTTTCTTCAGCTACATATGTAAATATACGGATGCTAGTTGCTCACTTAAAATTTTGTAGTGTTCAAAGGAATAACA
ACAGTAAGACGTTGACGCACCGAAAATAACCAGAAATAACGGATTGTACTATGACAAGACACACGACAGATACACTAGCTTAAGTTTGTACAGGAAAATA
TTTGGCCTATTCTTATGTCACAGTAAATTAAGATTATCACCTTAATCACAGAAATTCAAGGGGTAAACGATTATAGCACAAAACGATCCTGGACCCCAAG
ACCTGGGTCTCTGAACGGCTGAACCTCCAATTATACCTCTATACATTTGGAATGTGTGTAGTCAAACTTTCGCTACTGGTATCTTTACAAATATTTACAT
TGAACACATCAAAATGATATACATGGATTCCTCTCGATATGTCTTTGAGGTTTGGGTTCTAAAAATGATATACAAATTATTAGGTTCTAAACTTAAATGA
TATATACACATCAAAATGATATACAAATTATTGGGTTCTAAACTTAAATGATGTATACACATCG
>comp80692_c0_seq21
GCTCCTTGAGAAACACCATCCAATTACAAGACTATTTGATGCAACAATCACGCATATGGCTGCGCTAGAACTACTGCTCTGAATTAAGAGGCGTTGAAAT
ATTGTCCGGTCTACATATAGGCACTTGAGGTGTATTGACAGACAGTTCCCCTTAGTTTGCTACAACACATCAGGCGAATTCCACCAAATTAGGATGATGT
TTGTTTCTTAGACACAGGATAGCAATGCCAAAAGTTTTTATAATTTTCCGCTTCACCGCAGATTTGGCATCTCAAAGGGAGTTGTGGCAAGATTCTCTT
GACCAAGCTTTCTTGCAGATTTGGCAAGAAAGATAAAAAAAATGTTGGTATACTTAAGCATCCTTAATAGAAACTAAACAGGTCTTAGATTGAAATTATC
ATAAATAAACCCTTTTTCACTTTAGCTGTAGTCCGGATGAAATCTTAGGGGCCTTGCTTAGCCTGCCCTGAAGCTTGCCAGTAAGTCGAAGCTGATGCTG
ACACAGCACTGAAATAGATCCCAATGAACACAAGTTATTTCGAGAAGATAAAATACCAAGCAAAGCCAGGATTGCATTAAAAGATAGTGTATTTCTACTA
TACGATCTGAAAGCTTAATTTATTATATCAATAATCAAATTGGCAGCAGTGTGGAACAGATATAAAGAAGATGCTTCAAATCACAAATAACTACAGATAA
ATTGAAGGGTGAAGGTTCATTCAACCACCAAACACCCAAAATGTCGAGCTGCATATGCACAGAACCTAAGTACGGTACAGTTCATAGTTTGGGAAAATAG
ACTGCAAATGTGCACAGATAATACAGATTGTTACTCGATGAACAGTAAGCACGGTATTTGAAATCCTTCTAACCATCCATCCAGGCAGTGCCTCGTATAC
TCAAACCTCTTTTTGGAGGAAGCCAAACCATGTGATCCTTTTCAACCCAAATACATATATTCAAGATGAAACTAACAAAAAGACAGTTGATAAAGAAACA
CGAAAGAGAGTAGAAAAAGGGTACCTGTTTCAAGTTCTCATCTTCTGAAAATGATCAGCAGTCAATAGATGTTTTAGTCCATTCTCCAGAAAAATGAGTG
CCGCATGAATCCTTTCCGGTTTATAGTGAAACATTTTAGACGATGACCTTTAACATTCTAGAAAACGAAGAGAAAACCTACAGTCATCTAATTTCAGCTT
AAACATGTTCCAGCAAGATCATCACCATCCTGACATGTTCCGTTTTGTCCCTAGACCCGAGCACAAGGTATTGCGAGGCTAATTTAGCTCAATTCAGTTG
TCAGAGCAATTGTGTGTGAAAAGTGTGAACAACAGCATAGGAGACAATACAGCAAGTGTAGCGTGCCAATATGTGTCCAGCTTGATAGTTCACTATAATA
AAGTTCTTTAATTCAAGTATTTGAATAATATAGAATCTCAACCTTCAAGTTTAAAAGGTTTTGTATTTGCTATTATAATCGTTGCCACCCCCTCACCCCT
CTCAAAAAGAGAGTTACTTTGTAGCGCACTGTAAATCTCTTGATTGAAATAGTGGCATGTCATCTTATTTAATTCAGTTATTTCATAAGGTACAATTTGT
TTAGCTGACAGCTCAGACATGCTTCTATGCAACATGTTTATACCAATGCTGTATTTTGGCAATCCTGGATGTGGCATTACTTAACTGTGCTTTCATATTC
AATAGTTCTGGCAAAGGGAGGCTGCAAGCTTGAGACAACAACTGCACAACCTGCAAGAAAGTCATCGGTATGGCTAGAAAACCCTGCAAATTTTACAAAT
GTACAAGTCAGAAAATTTGCCTAATGTAAAAGGATCAAACTACAGGATTTGTCAGCATGAGCACAAGTGCCTAACTTCCGAGTTAGATAACTCTGGAGGA
AGGAAATCCTTCCTACTGTTTGTTTCAAGAATATACAAAAATTCAAAACAACATTAGAATAGAACCAAAAGAAGAAACAATATTCACCATGTAGCAATAC
TCAATTCCTGGTTGTCTTATTTATAAAGGTATAAAACTAGGTTCTGACGAGACAATTATGAATCAACTACACCATGTTACAGATCATCCATAACTCTTGG
TGATGCAAATTAAGGGCTGTAAAGTGACAAGTATACCTCCACCAACTAATTTGTGCAGAGTGTATAAAATTGTAAAACAATGGATAAGAATGAATTTAGT
ATACAAGATAGCATACAATGCCCAAGTTTAAGGAGACATACTGATTTACTGACTCCATAGGACAATCTTTTGCTTATGAGGACATCAGTTTTCATAAACA
ATATCAATGTAAAAGGAGGTAACAGTTTATGAATTTCCTATGCCAAGCCATTTATAAGTCAAAAAATGTACCTATTTAGCTTTTATGCATTCCAATTAAC
TTCCAAAGAAGAAGAGTACATACTAATGGAAAACAAATATGATATTATCATCTGTTTTGCCCGGTAGTTTCTTGAGTCTCTGATTTGTCCGCACTTCACT
CCCAACCAACACAACATTAATACACATGGGCAGAGGAATATCAGGTTATCAGGTAAACTGAATACAGTTTGATTGACTGCAGTCAGTTGATGGGACAAGA
TCTTTCTGGATTGAGCGTCAAGGAACTCCAAAATCTAGAAAATCAGCTAGAAATGAGCCTACGTTGCATCCGGACAAAAAAGGTAAGGATGCTAGCCATA
TTTCTGTTTTTCGTTACCAGATTGCCTCATCCAAGAATACAACTGATGATGACGTTTTTTACTTTCAGGACCAACTCTTGATTGATGAAATTCACGAACT
GAATCGAAAGGGAAGTCTCATGCAACAAGACAAGATGGAACTATACAAAAAGGTCAACCTAATCCGTCAGGAAAACATTGATTTATACAAGAAGCTCTAC
GAGAAAGAGGTGACAAGTGAAGTCAACCGAGATTCATCAACTCTAAACTTTGCAGTTGTCGAGAACGTCAACATTCCTGTTCATCTTGAACTTAACACTC
CACCGCAAGAAAATGATGTTGAGCAAACTGCACCTCCTAAACTAGGGTAACCCTGCTTTTTTCTGCAAAGGAACTATTGATTCTGTTCTTAATTCTTTAG
TGGCATATTGTTTACTACTAACTGAAAACTTGCAGATTACAACTAAATCCATGAAGGCATGCAGCATGGCACCGCTTATGTTCATTATTCTCAACGGTAC
GATTCAAAGCAACCAGAAACACTATTGGTTGTAAAGAGCTTAAACACATAGCAAGACTGACGCGGTATGCATGCCATGAGTGATTGATAATGGTAGTGTA
AATCTATTTCAAATAAAATGTATTTTATTGAAGAATACAGCAAATGATTTGTATTATAACTATTTGACACATTCTGCAAGTTTGTTGACCAACGTGAGTA

CATGATAGCTACAAATCTTATATTGACAAAAAGATATGGTGTGTGGAAAAAATTCAGGTGCTATTTTTTTCTATTTTGAGAATTGTATGCATAGCAGAAG
AGCACAAATTGCATTCTGGTTTATCCGTGCAGGCATTCCAGAGTATCAGAATGGGGATCAACCTTTCTTCAGCTACATATGTAAATATACGGATGCTAGT
TGCTCACTTAAAATTTTGTAGTGTTCAAAGGAATAACAACAGTAAGACGTTGACGCACCGAAAATAACCAGAAATAACGGATTGTACTATGACAAGACAC
ACGACAGATACACTAGCTTAAGTTTGTACAGGAAAATATTTGGCCTATTCTTATGTCACAGTAAATTAAGATTATCACCTTAATCACAGAAATTCAAGGG
GTAAACGATTATAGCACAAAACGATCCTGGACCCCAAGACCTGGGTCTCTGAACGGCTGAACCTCCAATTATACCTCTATACATTTGGAATGTGTGTAGT
CAAACTTTCGCTACTGGTATCTTTACAAATATTTACATTGAACACATCAAAATGATATACATGGATTCCTCTCGATATGTCTTTGAGGTTTGGGTTCTAA
AAATGATATACAAATTATTAGGTTCTAAACTTAAATGATATATACACATCAAAATGATATACAAATTATTGGGTTCTAAACTTAAATGATGTATACACAT
CG
>Locus_9469_Transcript_30/63_Confidence_0.140_Length_1187
TGTTCTTGGGACTGATCTGTGCTTATATTTGGACAGTATGAACTGAAAAACAGGAATAGAGAATTTCCGTGAGATGAGACGTAAGGAAAATCTCCATGAT
ACTGTTCAATTTGAATATTGCGAGTACTCTGTCCTTTTTCATGTCTTCGGTAGTTTAGTCTCATGCAGCTAGAATTTTAACTCCTACGGTGCTCCTCTAG
CAGAAAATGGCACCATAATGATTCAATTTGGTCATCAAATGCCTGATTACGACTCATCAGCTACCCAATCGACCAGCGAGAGTCATCAAGAAGCGTCTGG
AATGAGTGAAGGAAGCTTCAATGAGCATAATGATCGATCAGGTAATCTTGATGGTTACACGAAGAGAGATGAAAATAAGATGATATCAGCTTTATCTCTG
GGCAATCCGGAAACTGCTTATGCACATCCAAAGCCTGACCGTAGCCAGTCCTTTGCCATATCATACCCGTATGCTGATTCATTCTATGGTAGTGCAGTGG
CGGCTTATGGCTCGCATGCTATTATGAATCCCCAGATTGTGGGCATGATGTCATCTTCTCGAGTGCCATTACCAATTGAACCAGCTGCTGAAGAGCCCAT
TTATGTAAATGCAAAGCAATACCATGCGATTCTCCGAAGGAGACAACTCCGTGCAAAGTTAGAGGCCGAAAACAAGCTGGTGAAGAGTCGCAAGCCATAC
CTTCACGAGTCTCGGCATCAGCATGCCATGAAGAGAGCTCGGGGAACAGGCGGGCGGTTTCTCAACACGAAGCAGCAGTCAGAGGCTCCTGGCGGCGGCA
CCTCGGACGCGCAACTCATGCCACCAAATGGTGGCCTGTTCGCGAAGCACGAGCACAGCTTACCACCCAGTGATCTCCATTATCGCGCAAGAGGGGGCGC
TTAAGGAGTCCCATGGCAACTCATCCTTGGCTTAAAAAAAATGTGTGGCGCTCAGCAATTCGACCTCTGCTAGTTCAATGTAAAGGTGTGGGACTGTAAC
TGTGGGTGCTTGAATTGGCCGCAGCCATTCCTGCGACGCGTCGCAGGTGTTGCCGGTTCTGTGGTCGTGCTAGACTTGTGACCGGCACGACGAAACTGGC
ACCTGTTTCCTGTAACTGTTGTCTAAGATGATGATGACTACTCCAATAACCATTCTGTATGATGCTTTGACGTCGGTGTTGCTCCTT
>comp76332_c0_seq3
ACTGCTCTGAATTAAGAGGCGTTGAAATATTGTCCGGTCTACATATAGGCACTTGAGGTGTATTGACAGACAGTTCCCCTTAGTTTGCTACAACACATCA
GGCGAATTCCACCAAATTAGGATGATGTTTGTTTCTTAGACACAGGATAGCAATGCCAAAAGTCTTTTATAATTTTCCGCTTCACCGCAGATTTGGCATC
TCAAAGGGAGTTGTGGCAAGATTCTCTTGACCAAGCTTTCTTGCAGATTTGGCAAGAAAGATAAAAAAAATGTTGGTATACTTAAGCATCCTTAATAGAA
ACTAAACAGGTCTTAGATTGAAATTATCATGAATAAACCCTTTTTCACTTTAGCTGTAGTCCGGATGAAATCTTAGGGGCCTTGCTTAGCCTGCCCTGAA
GCTTGCCAGTAAGTCGAAGCTGATGCTGACACAGCACTGACATAGATCCCAATGAACACAAGTTATTTCGAGAAGATAAAATACCAAGCAAAGCCAGGAT
TGCATTAAAAGATAGTGTATTTCTACTATACGATCTGAAAGCTTAATTTATTATATCAATAATCAAATTGGCAGCAGTGTGGAACAGATATAAAGAAGAT
GCTTCAAATCACAAATAACTACAGATAAATTGAAGGGTGAAGGTTCATTCAACCACCAAACACCCAAAATGTCGAGCTGCATATGCACAGAACCTAAGTA
CGGTACAGTTCATAGTTTGGGAAAATAGACTGCAAATGTGCACAGATAATACAGATTGTTACTCGATGAACAGTAAGCACGGTATTTGAAATCCTTCTAA
CCATCCATCCAGGCAGTGCCTCGTATACTCAAACCTCTTTTTGGAGGAAGCCAAACCATGTGATCCTTTTCAACCCAAATACATATATTCAAGATGAAAC
TAACAAAAAGACAGTTGATAAAGAAACACGAAAGAGAGTAGAAAAAGGGTACCTGTTTCAAGTTCTCATCTTCTGAAAATGATCAGCAGTCAATAGATGT
TTTAGTCCATTCTCCAGAAAAATGAGTGCCGCATGAATCCTTTCCGGTTTATAGTGAAACATTTTAGACGATGACCTTTAACATTCTAGAAAACGAAGAG
AAAACCTACAGTCATCTAATTTCAGCTTAAACATGTTCCAGCAAGATCATCACCATCCTGACATGTTCCGTTTTGTCCCTAGACCCGAGCACAAGGTATT
GCGAGGCTAATTTAGCGGGGCAATTGATTTTTTGCCACTATGATACAACTGCAATTACCCCGCTGTCACTGACACGTGGGGTCATCTGAGTCACTGAAATG
TGGGCCCAATAGCATTTTGTTTGCCACTCACCCTTCCAGAGTGGCAAAAAGTTAATTGTCCCTAATTTAGCTCAATTCAGTTGTCAGAGCAATTGTGTGT
GAAAAGTGTGAACAACAGCATAGGAGACAATACAGCAAGTGTAGCGTGCCAATATGTGTCCAGCTTGATAGTTCACTATATAATAAAGTTCTTTAATTCAAG
TATTTGAATAATATAGAATCTCAACCTTCAAGTTTAAAAGGTTTTGTATTTGCTATTATAATCGTTGCCACCCCCTCACCCCTCTCAAAAAGAGAGTTAC
TTTGTAGCGCACTGTAAATCTCTTGATTGAAATAGTGGCATGTCATCTTATTTAATTCAGTTATTTCATAAGGTACAATTTGTTTAGCTGACAGCTCAGA
CATGCTTCTATGCAACATGTTTATACCAATGCTGTATTTTGGCAATCCTGGATGTGGCATTACTTAACTGTGCTTTCATATTCAATAGTTCTGGCAAAGG
GAGGCTGCAAGCTTGAGACAACAACTGCACAACCTGCAAGAAAGTCATCGGTATGGCTAGAAAACCCTGCAAATTTTACAAATGTACAAGTCAGAAAATT
TGCCTAATGTAAAAGGATCAAACTACAGGATTTGTCAGCATGAGCACAAGTGCCTAACTTCCGAGTTAGATAACTCTGGAGGAAGGAAATCCTTCCTACT
GTTTGTTTCAAGAATATACAAAAATTCAAAACAACATTAGAATAGAACCAAAAGAAGAAACAATATTCACCATGTAGCAATACTCAATTCCTGGTTGTCT
TATTTATAAAGGTATAAAACTAGGTTCTGACGAGACAATTATGAGTGAATCAACTACACCATGTTACAGATCATCCATAACTCTTGGTGATGCAAATTAAGGGC
TGTAAAGTGACAAGTATACCTCCACCAACTAATTTGTGCAGAGTGTATAAAATTGTAAAACAATGGATAAGAATGAATTTAGTATACAAGATAGCATACA
ATGCCCAAGTTTAAGGAGACATACTGATTTACTGACTCCATAGGACAATCTTTTGCTTATGAGGACATCAGTTTTCATAAACAATATCAATGTAAAAGGA
GGTAACAGTTTATGAATTTCCTATGCCAAGCCATTTATAAGTCAAAAAATGTACCTATTTAGCTTTTATGCATTCCAATTAACTTCCAAAGAAGAAGAGT
ACATACTAATGGAAAACAAATATGATATTATCATCTGTTTTGCCCGGTAGTTTCTTGAGTCTCTGATTTGTCCGCACTTCACTCCCAACCAACACAACAT
TAATACACATGGGCAGAGGAATATCAGGTTATCAGGTAAACTGAATACAGTTTGATTGACTGCAGTCAGTTGATGGGACAAGATCTTTCTGGATTGAGCG
TCAAGGAACTCCAAAATCTAGAAAATCAGCTAGAAATGAGCCTACGTTGCATCCGGACAAAAAAGGTAAGGATGCTAGCCATATTTCTGTTTTTCGTTAC
CAGATTGCCTCATCCAAGAATACAACTGATGATGACGTTTTTTACTTTCAGGACCAACTCTTGATTGATGAAATTCACGAACTGAATCGAAAGGTTCTGG
AATATTCTGTTATTACTCTGCAATGAGAGGTCTCTGAGTGGATTTCAAGAAGCTGACTCTACTGGCTCATTTTCAGGGAAGTCTCATGCAACAAGACAAG
ATGGAACTATACAAAAAGGTCAACCTAATCCGTCAGGAAAACATTGATTTATACAAGAAGCTCTACGAGAAAGAGGTGACAAGTGAAGTCAACCGAGATT
CATCAACTCTAAACTTTGCAGTTGTCGAGAACGTCAACATTCCTGTTCATCTTGAACTTAACACTCCACCGCAAGAAAATGATGTTGAGCAAACTGCACC
TCCTAAACTGGGGTAACCCTGCTTTTTTCTGCAAAGGAAGATTTTCTTGCAAGTTATGCAGTTGTTGTCTCTAGCTTGCCGCCTCCACTTGCAGTAACTG
AGACGCCGGCGCCGAGCTTGAGCTTGAAACCTAGACTACGGGGACAGGGTAGACAGGAGACGCGAAACCCCAAACCAGCGCAAAACGACGCGCAGGGAGG
GAGTGTCACTGAAGGAGCGACAGGGAAAGCAGCGGCACGCCGGGGGAAATTCCGATCTGAGAGAACGGATCAGCCATGCATGACAGGAGCTGCGGAGGCA
GCAGGGGGGGGGGGCTCCCGCTGCCACGGGACAGTAGGGCACGCCAGATCGGTGAAATCCAGGCGAAGGACGAGGCGTGGGCGAGATTTACACGACGGCG
CGATAGGATCTAGCCCGGATTTGGATTCGACCGACGAGGGCGGAAGGAACGGCAGGGTGGTGCTCTTGTAGAGAGAGAAGGAGTCGGAGGGGGGCGGAGAG
AGAGAAAGGGGGCAGGTGGGGTTTCAGGTAGTAAAATATGGAGCGG
>Locus_14326_Transcript_24/49_Confidence_0.449_Length_2916
GGCCTCGCGGTGCCCCGTATTCTCATTTCCTCGCCGTATTTTCTCTCTCCAGCTCCAGAGTCCGGAGGAAGCAGAGAGAGAAACTCAACGGGAAGGTAGA
GAGAGGTACAATGCTCCTGATCCCTATTTGAAATCCTCGATTGGTCAGTTCTGTTCTTGGATACACTGTTGCTGCAGAATGGATGACTGTGCTTTCCTTT
CGGGGAGTGGCGTCGTGCTGTGTTGGTTCTTGCCTTCCTAATTAGTAATATGCCTTAGTCTTGCTCTACGGTTGGTTTGATTCACGTTTTTCCTGTAATC
TTTCTACCCCCAGGATTTTCCCCTTCGGTTTTCTTGGTTTGCTTCTCGGAAATTTGTGATTTTTAAGAGGATTTTTGGCTTGTGTTGCTCGATTTTGGTT
CTTGACCGTGCTTCCTTGGGATGAGCTTTTGCCTGGTTCGACATTACAAGATGTGTTATCCTAGAAGTTAAATTGATCTTCTATGAGCTGGGCGCTGCAT
TGTGGATTGCATGGGGTGGCATACTGTGGGCCGTGGAGTTGATGGAATCATGGAAATCCGCAGTCCCTTTTGTTGGATAAGGGTGGTTGAAGCTAATTTT
TTGCGAATTGTGGCGGTTTCTGGATATCCTGCTTGAATTAGTGGTGTGATAGATTGTTCTAGAGAATAGGAAGATATATGTTTTATTGTTAGAACATCAA
GAGAGTGTTGAATCAGGTATGTCAATGTGTGAACTTATTGATGGGAGCTAAATGCTTGCTGACGTATCTTCGCTATAATGACCTAATCAAACCTAACAAG
CACTAAATACTGATCAATTCTCAGTTATACAAAGATTTTCCAGAGAGTTCTTCAGAGCTCGAACTTCTGGGATGAAAATAGTTTGGCTCAAGATAGTCCA
CTGGAAGGAGTTTAGGATATCTGTTTGAGCTCTGCCTGCAATATATGGAAATCTGAAGGTGATATGGTAGTAGAGTTGGGGACCCTGATCCCCAAGCCAA
AAGAAGTTCGGTAGAATTTCTCAGGAATATTGTGGTAGTAAGAAGCAAGTTAAAAGGAACCTAATTAGAAAAAAAGACATGCCTTGCTCGCTCATGAGAA
TATAAAACAGGTATGCCACCTCCAGTCAGTACTTGATTAATTTCATTGAAAATGGAGGGCTGTAATAGATCCTTATCTATTAATGTGTTGTACCTAAACT
TGGAAATCATCAGCTGAGGGCATTGTGCATGGTGCGTGGAGTACTAGCCCTCGGGAGATGCTTCTTAATTAGGCAGCGAGAAGGATAGAGATCGGTTTGA
CATATGTTCCATTTGTAGCTGTACTGGATATTTCTGCATGTTCCCCTGGAGCAGTTAGTTTCAGAATTATGATATTTCTACTTGTGGCAGGATTGTTCTG
TTTCTATTATCTGTTCATATTTATGCTAGTCTGTCTTTTATGTTGTAATATCTATTGTCTATTGCACTTCAAGTGACTATGTATAACCATATTCCAGTGA
TTCAGTGAATGATTATGAATTAAAGTAAGAGGACATTTACATCAATTATCCTTGTTCTTTCTCACGTCATTATCAATTCGGTTGATTAAATTCTGGCAAC
TTGCCATGATGGTTACTTTAGCTGATTAAATGCTACTCGGAGCTAAGGATACCTTCTCGTAGTGCTCCCGCTTTTGTGAGAAATGGAGGATCATCCAGTC

CATCCCATGTCGAAGTCCAACAATGGCTTCTTGTCAGGAAATGGCTATCAGATGAAGCAATTTGGTCATAAAATCGACGATAGGGACTCGTCATCAGAGT
CCGGTCGGTCTCACCAAGAAGCTTCCACAGCGAGTGAGAACAGTATAAATGAACAGCACACCTCAACACAATCTGACAATGATGATGGTCATGGGAAGCA
TAATGAGGACATGATGAAGTCAGCAGTGTCCTTGGGGGAACCAGGATCAGCCTTTTTGGCCCCAAAACTAGATTACAGCCCATCTTTTGCTTGTGTTCCT
TATACTGCTGATGCTTTTTATGGTGGGGTCTTGACAGGATATGCTCCACATGCCATTGTTCATCCCCAGCAAAATGATTCAGCAAACTCTCGGGTTCCGT
TGCCTGTTGAACCTGTAGCAGAAGAGCCAATATATGTTAACGCAAAGCAATACCGTGCAATCCTTAGGAGGAGGCAGATACGTGCTAAACAGGAGGCTGA
GAATAAGCTGGTGAAAGGTCGGAAGCCATACCTCCATGAATCTCGACACCGCCACGCAATGAAGCGAGCCCGTGGATCAGGGGGGCGGTTCCTCAACTCA
AAGCAGCTCCAGGAGCAGAACCAGCAGCAGCAGGCATCAGGTGGTTCAAGCTGCACAAAGGCCATCGGCAATAACACAAGCTCCCAAAGTGGTCCCAGTC
ACACGCCTCTGACACTTGCTTCCTCTGACACTGCAAGCGCTTCGAGGGCCAACCAGGAGCGCACCTGCTTCCCCTCGGCTGGCTTCCGTCCCACAATGAA
CTTCGGCGCGCGAGGTGGAGACGCAAAGCTAGCTGTGAATGGCATACAGCAGCATGTTTCCTTCATCAGGTGAAGCAAGTGAAATGGGGCGCTGCCTGCG
GTAACTCATCCTTGGCTTATGAAACTGTGAATGTGTAATGGACATGTAACTTCAGTCTCAGAATAACCAAACATATGGGAGTCCTCCTGAAGTTTCCTGT
AGTTCAAAGAACTGATAGGTTATGGCCTTATAGGTTTGCTTCTGGAGACTGAAATCCGTGCTTGATATACTATGTTGTACTGAGTTCTGAGGTGAAAGAC
ATCGGGAGAGTATCAGGTGCAAATGGCACAAATGTTGAAAACGGTGCAAATTCATCAAAATCTGTACCTAAAAGACTTCAATAAATTTTAAACTTTGCAT
AGCTCTAGGTGCATTT
>Locus_14087_Transcript_47/66_Confidence_0.346_Length_2533
GATTCAAAATTGTAGCAGATTTGTCCTCTACCCACCCCTCTGAATCCTTTTTATACATACACTGACCATGGCACCAATGATCAAACCAAACACCAGCTGC
AAAGATCACCCTGGATTCCTGGAACAAAGAGGGCCTAGAGCAACGCAGCAACCTTCGCCACTTGAAGAGGATAGGTACAATGTGGCTTAGCTAGCACCTT
GCAGTCAGGTTTGGAATTATTGCTTTCTTTGTAAACCAGTATATTTTTTGTGGCATGGTATTGTTTGTGGACGAGAGAAGTGAGATTCTTCCCCCATGCT
CACTCAGACACCTCCTCCAATGGGGCAAGACGAATTGGCCGTCTATGGAGGTGGGCTGTAGATGATATCCACCACAAATGGGGTGCTCTTCCCTTTCTTA
CAGGATTGAAAGCTGCTTTTGAGTGGAGTAGTACTGAACTACTGATTATCTAGTAGAGAGGCAGTGGCAGGAGTAGTAGTTGTTGGGGTTTCAGGGGAGG
ACATGTGCATGGTTGCTGTGTGGGGAGGTAGCCATCCACTGGGCCTCACACTCTTCCCTGCCTCAGGTGGGGTTTGTGGTGAGAAGGCAGCGGAAAGCTT
GCCTACCATCTCTGTGTGTGTTGGATCTTTGCGAGCCGTGGCCTCTTCGTCCTCTTGTTTTTCTCTCTTTTCTTGTTGAACTCATGGTTCTTGTTTCTCT
TATGCCTGCCAGGTAGAATTATTCAACCGGATAAAGATTATTTCAAGTAAAGGAGGGCTACCAGTTTATACCTGTTCAGGTGGGTGCAGCAGTTGAGAAC
TTTTTTTCGATACAGAGCAGTTGAGATCTTTAATTCTGGCATGGGTTCCTTTGGGGTGAACTGGAATCAGAAGAACTCCGCGGTGTGCGATTGGGAAAAT
TTAGCACCTTCTGTTCCAAATGCAATCGGAAACCCCAGGAACGGAGCTACGAGGCATGGATCTGTGAATTCATCTGGTGGCACTCTCACTTCTAGCTCAG
AGCTAGGGCATGGTTCATCCAAGAGCTCCATGTCGGCATCCATTGATTCACCGTCCAAAGTAGGAAACAGCTTGGAGTTCAACTTTGCTGCTGTCAAAGG
CCATGTTAAGAACATGGATAAGGATTTAGATTTGACGACTTGGGGACTTCTCCATCGTCGATGATAGCATTCAGCCATGGAGAGCCATTAATCAGCCTT
AAGCTTGGAAAAAGGACTTACTTTGAAAATGTCTGCGGAGGGCAGGATTCCAAGAGCACTGCACCTTCTACCAGGACTTCTCCATCAACCGTTCTCAAGA
AGACCAAGGTGTCTCAGCAGAATACACAAATGTCATACTGTCAGGTTGAAGGATGCGGAATTGATCTGTCCTCCGCTAAAGATTACCACCGCAAGCACAA
AGTCTGTGAAGCTCATTCTAAAGCCCCGAAGGTTGTTGTTGCTGGTGTAGAGCGCCGTTTTTGCCAGCAGTGTAGCCGGTTTCATGGCTTAGCCGAGTTT
GACCAGAACAAACGAAGTTGCCGTAGCGTCTTACTCATCATAATGCACGGAGGAGAAAACCACAGGCAGATACAATTTCATTCAGTTCATCGCGGCTCTC
GACAATGTTTTATGATACAAACCAGCAGAAAAATCTTTTCTGTAGTCAACCTCATTTTGGCCAAGCGAGAAGCAATGCAGTTTTTTCTTGGGAAAACTCG
GGAGATTCCAAATTTATGGAAACCAAACATCTGTTGATGCAGCCAACGAAAATGTTAGGTCTTGATGGGCTGCATTTCTCAACCCCCCAGATATCAAATA
GTGTTGTGGCTCACTCTGTACATCATCGTAATTTCGATGGGCTCATGCCATTCAAGGGAACCAACACAAAGGTCCTCAACCAAGGCGTGGAAGCTTCTGC
GATCGCTTCCAACTCGAGTGGAGACCCGGATCTTGGGTGTGCTCTCTCTCTTCTGTCAAACGGTTCGTGGGGTTCAACTTCAGCCGTCATCCAACAGCCT
AGTTCTCATGCGCAAGCTGGTGCGGTGCCACCCTTCGCCACCGTCGCCGTCTCCAACCCTGCAATGCATCCTCTGGACTCATCCCCGGGAGGATTCTGGC
AAGACGTCCCTCCCCCGCTCGATGAAACTCCACATATTCAGGCATTCACGCACCTCTGATCAAATGCTGCATCCCCTGGGAACAGCAGGTGGTTATACCT
TCCGTAATCGTCCTAGGTTTGCTTTGCCAGTGACTGGCTCTGCACAATGTCGATCCATCACCGGAGGCATTTGGTAAGGTGAAGACTAAAGACTCAAGAA
TAACCGGAGACTAACTCGCTTCTCTTCCAGGCTCAAGACTGTTTGGGTCCAGCGGTGTTGCTACCTGATCCTGTTTTCGGTTCTGAGTACATCCATTACT
AATCATGGGATTACTTGTTTAGGCCAACCAGACATATTCTGGGGTGAGATTACGACATGTTAATTTACATTGACGGTGTTTCTACATGTGTTAGTACTAA
TGATGTTATCGCTCTTGGCCCCGTTACGTTTCA
>comp54836_c0_seq1
GGGCGTCACCTTCTACAGGAGGACCGGCCGCTGGGAGTCGCACATCTGGGACTGCGGGAAGCAGGTCTACCTGGGTGGATTTGATACAGCGCACGCTGCG
GCAAGGGCCTATGATCGCGCTGCGATCAAGTTCAGGGGCCTTGATGCGGACATCAACTTCCAGTTGAAGGACTATGAGGACGACTGGAAGCAGATGAGGA
AATGGTCCAAGGAGGAGTTTGTTCACATACTCCGACGTCAAAGCACTGGGTTTACAAGGGGGAGCTCAAAGTACCGCGGTGTGACACTGCACAAGTGCGG
CCGGTGGGAAGCTCGGATGGGTCAGCTTCTTGGAAAGAAGTACATCTACCTTGGGTTGTTTGACAGTGAAGTCGAAGCAGCAAGAGCATATGACAGGGCA
ACCATTCGCTTCAATGGAAGGGACGCTGTTACTAATTTTGATCCTAGTTCCTATGATGGAGATGTTCCACCTGAAATTGAGAAAGAAGGGGTTGATGGGG
ACATCCTTGATTTAAATTTGAGGATCTCGCAACCTAACATGCATGATTCGAAAAGTGATGGCATCCTGACTGGGTTCGGATTAAGTTGTGATTTTCCTGA
AGCTTCAAGTTCTGTTGTTTCTCAGGAAGTGCCTATGGAGAAACGACCCGAGCTGTGTCCCCAGTCGTTCCCCCCATGGGCATGGCAAATGCAGGGCTCC
CCTGCGCCATCACTCCCTACTGCAGCATCATCAGGATTCT
>comp78487_c0_seq7
ACCCGATTTGTTTAGATTGCAATTAGACTAAGTATAGTTGGTGAGAAGACAGGAGTAGAGATAAGGGCCAACAGCCCAACAGAAAAACCAAGCAAGACTG
CAACTGCAAGAGAGATGGGGAGAGAGAGGAAACCTTGCAGAGGAGAGAAGCAAGGTGGGAAAGAGGCAGCAAGGGAGGAGGGCTACAGGAAGGGGAACAT
GCTCCCTTCTCATCTCACAGTATGAACTGAAAAACAGGAATAGAGAATTTCCGTGAGATGAGACAAAATGGCACCATAATGATTCAATTTGGTCATCAAA
TGCCTGATTACGACTCATCAGCTACCCAATCGACCAGCGAGAGTCATCAAGAAGCGTCTGGAATGAGTGAAGGAAGCTTCAATGAGCATAATGATCGATC
AGGTAATCTTGATGGTTACACGAAGAGAGATGAAAATAAGATGATATCAGCTTTATCTCTGGGCAATCCGGAAACTGCTTATGCACATCCAAAGCCTGAC
CGTAGCCAGTCCTTTGCCATATCATACCCGTATGCTGATTCATTCTATGGTAGTGCAGTGGCGGCTTATGGCTCGCATGCTATTATGAATCCCCAGATTG
TGGGCATGATGTCATCTTCTCGAGTGCCATTACCAATTGAACCAGCTGCTGAAGAGCCCATTTATGTAAATGCAAAGCAATACCATGCGATTCTCCGAAG
GAGACAACTCCGTGCAAAGTTAGAGGCCGAAAACAAGCTGGTGAAGAGTCGCAAGCCATACCTTCACGAGTCTCGGCATCAGCATGCCATGAAGAGAGCT
CGGGGAACAGGCGGGCGGTTTCTGAACACGAAGCAGCAGTCAGAGGCTCCAGGTGGTGGCACCTCAGATGCGCAACACATGGCCGGCAATGGTGGTCTGT
TCAGGCTGCACGAGCACAACTTACCACCCAGTGATCTCCATTATGGCGCGAGAGGTGGCTCTTAAGGAGTCCCCTGGCAACTCATCCTTGGCTTAGTAAC
GTGTGGCGCTCAGCGATTCGACCTCGGCTTCGATGTAATGGTGTACGATTGTATCTATGGTGCTTGAATTGGCAGCAGCCATTCCTGCAGCAATGCGTCG
CAGGTGTTGCCGGTTCTGTGATAGTGCCAGACTTGTGACCGGCACAGAACTGGCATCTGTTTCCTGTAACTTTAGTTTTTCTAAGACGATGACGATGACT
ACCCAATAACCATTCTGTCTGTATGGTGCCTTGCGTTTTCCGTTCGCCCGTGACGGTGTTGCTCCTTAAAAGTCAGTGGCGGGAATTCTTCTTTGTCTCC
GCGATGAACTGGTGAAGTTATTTCTTTTTTCTCT
>Locus_39694_Transcript_2/7_Confidence_0.667_Length_2197
GCTGTGGCAAAGGGAGGCAGCAAGCTTGAGGCAACAACTGCACAACTTGCAAGAAAGCCACAAGTATGATCTTCCAATTGATTCTGCAATGAAAATACAT
CTCATCAATACAAAGTTTAGATGCCACGTGGAAAATTTTGTAATAACTGTAAAACCAAAATGAAACGTATCATCCAGTGAAGATGAGGGAGAAAAAAATC
TAGTTACTGAAAGACATTTATAGTGTTCACTATTCAGAAAGGAAATTGAAAGTGGCTTTGTCACAGCAACATATATGTGGTTTTGCTTCGAACCGTGTAC
CCACGTAAATTGATATTGGTTTAGTTTCAATCTACACAAGATAATCTGACGAACTCTTTTGCATTGTTGTAGGCAACTAATGGGAGAGGAGCTTTCAGGC
CTAAGTGTGAGAGACCTCCAAGGTTTAGAGAATCGTCTTGAAATGAGCCTACTTAGTGTCAGAATGAGGAAGGTCATATTCATTTTATTTCCACATTCTG
TTTTTCTGTTCACTTCAATCGAATATGCAGTAAACATCTATCAAACAGGGCCACCTTTTGACAAGTGAAATTGAAGAGTTACATAGGAAGGGCAGCCTAA
TTCATCAGGAAAACACGGAACTCTGTAGAAGAGTGAATATCATGTCACAACAAAAAATGGAACTTCATAGAAAGGTTTGATGAACTATCTTTTGCAGAAA
CTTCAACCAAACGAAGATGCATTGTCAGCAGAAAGCTAAAATGACTTAATTTCAGCTCCTGGCCAGTGAACCAGGAGGTGTTGCTGATGCAAATAAAAGC
TCTAGCACTCCCTACGGCTTTGGTACAGCACAAGGCGCAGATGTCCCAGCTAATCTTGAACTGAGCCAGTCATCACAAAAAGAGGGAGAGCAATGCAAAA
CAGGGGCCCCGGAACTGGGGTAAATATTCTTCTTTAGGATCCAAAATTCCCATTACTCCAAGAAGTATGAGGAAGCTTAATGAGAACTCTATGCTTTAAT
ATGTAGCAGACTTCAGCTGCACTAAGGAGAAGCAGTACAAGCTGGACAATGCCATAATGGTCCACAAATTTCACCATGCGATGCAAGAGCATTTTGGTTG
CAATGACTATTTTGTGCAATATACTAGCTGGGTAAACAAATGGGTTGTGCAAAGACTACAGATAGGACCAGTTATGTTATCTGAGGCTCTGCTGTGTCTA
AAATGCAACATATTCGTTATTGTAATGACACATGATAAACTGACTGTCCTACCGTTGACCTTGAAGATAGCTCTGCAGTGAAAAGGTGTATTATGGGCTT

GCCAATATTAAAGAAGTGTTAGGGATCAACTAGTAGGATCTCACAAAGAAGTAATTAGTCCACATTAGTGCCAGCTGCTGGGGATGATATCTGACTCAGC
CTGCCAACCCTCTCTTAGCCATGGTGTTTCAACTAGTATTAGAAAAGAATGTTCATCCAATTGAGTTCCCAAACAACGAAATTTATATTATGTGTTGCAA
AGTGTCGACACCAGTGGAACAGAGTCCATATATGTCAAGCAAATGCCAAATGTCAATTTTGCTCCAGTGCACGTGGATTCTGTTCTATTCTCAAGAATTT
CAACCCTTGATAAGGCCCTTTTTTCCTGGTGAAAGGATTAACAACGATGAAGCTTCGAGATGGTTTAGTGATTGTTAATAGAGGTACTTAGTGCATCCAT
GCTTGAAAGTGTTCTTCCTTTCAGCGAAAGGTTTTCTATTCTACTTGCTTGTTATCTTTTGGCCAGTATGTCAAGCAAGAATTTAATCTGCTAATCAAGA
ATGTAATGCGTTCTTTTAACATGAATATCAACAGTTTTCAAGTAACTGAAAACAGTAATGTAGTATTCTCTCGCCGAAAATAAAGCTGTGGTTCCTTGAT
AGCAACCGGCAGTTATGCCAATACCACATTAAAGTGGTTGTGTCCCTTATAATACTCTCTCCATTCTACTTAATTCAATGCTATTTGTTTTTTAACGCAT
GTCAAGATCTGTCTTTGACCATTCCTTTAATCCTTTTGAATATATATTTTTTTAAAAAGTGGTGATATAGCTTTCGTAGGGCCAATATAAGATTTTTTAA
GAACATATTAGAGTTGGTCGTGTTTAGGTAGAGCTGTGTGAGGGTATGTGGTGATGCTAATCTGTAACAAGACGGTCTAAAGATACCGCCTTGCGCC
>comp78295_c1_seq30
ACAACTGCACAACTTGCAAGAAAGCCACAAGCAACTAATGGGTGAGAAGCTTTCTGGCCTAGGTGTGAGAGACCTCCAGAGTTTAGAGAATCGTCTTGAA
ATGAGCCTACGTAGTATCAGAACGAGGAAGGTCATATTCATTTTGTTTCCACGTTTTGTTTTTCTATTCACTCATCTTAATATGCATAAACATCCATCAA
ACAGGACAATCTTTTGAAAAGTGAAATTGAAGAGTTGCACAGGAAGGGTAGCCTAATTCACCAGGAAAACTTGGAACTCTGTAGAAGAGTAAATACCATG
TCACAACAAAATATGGATCTACAAAGAAAGGTTTGATGAACGATCTCATGCAGAATCCTTAACCAAATGAAGGTGCATTGTCAGCATAAAGTTAAATTAC
TTAATTTCATCTCCAGGCGAGTGAACCAAGAGGTGTTGCCGATGCAAATAAAAGCTCTAGCACTCCCTACAGATCTTGTAGTGCACAAGATGCAGATATC
ACGGTTAATCTTGAATTGAGCCAATCACAGCAAAAAGAGGGGGAGCAGTGCCAAAGAGGGGCTCCAGAACTGGGGTAAATATTCTTTTTCCAGAATCCAA
AAACTGCATTACTCCAAGAAGTATCAAGAAGCCTAATGAAGAACCCATGCTTTGACATGTAACAGGCTTCAGCTGCACAAAGAAGATGCACTACAAGCTG
GAGAGTATCATGATGTTCCACCAATTCTAACATGCAATGCGGAGCACTTAAACTACAATTAGTATTTTGTGCAACATGTTAGATGAGTGAACAAAATGGT
TTATGTACAGACTACAGATATGACCATCTATCTTATCTGTGGCTCTACTGTACTGAAATTGCAAGAAATGGTTATTGTAATGACTAATATGATACATTGA
CTGTCATACCGTTGACCTTGAAGATAGTTCTATAACGGAAAGGTCCAATTTGAAACAAGTGTTACGGATGAGCTAGTAGGATTTTTTTCACAAACAATTA
GATAGTCCACGTTAGTGGTAGCTGCTGGGTATGACATTTGACCCAGCCTGCCGACCCTCCCTTTCCCATGGGAGTTCAATTAACATTAGGAATGAATATC
GATATTATGGTTGATTATTTGAATCGTATGTTTTCCACCCTTAGATGTAAGGCCCTATTTTACTGTTTTAATGGATTAACAAGGTGAGCTTTCGAGATGG
TAGCTCATTGATTCGCATGCATTTTAGTGGCTGTTA
>Locus_21273_Transcript_6/23_Confidence_0.458_Length_1433
TCCAATTATATCATTCGTTCTCGAGCTTAGCAGCATCTAAAATCTCGATACAAATTAGTCCTCTCTATCCCAAACAACCCTTCTAAATAAACCACAAACA
ACTCATGTAGCCTTGCTACGGCAAGCCAGTAACTTTGCTTCGGGGGTCCGTACCATTTACACTTTCTTCTTCCTAATTTTTGCTCTGCCCCCACCAGAGG
AAGGGACGGCGGGGAGGGGGCAGGCACAGAGAGCAGCTTTGCTTGGCGGACGCACCGAGGGAGGCGCCTGGGAGCCCTGCTTCTCCCCTCTTCGTCTTCC
TCCTCCGCTTCCAAAGGTGACTCCTTCGGGAAAATGGTTAATGATCATACGAGGTCAAATTTGGTTTTTGATAATAAGCAATCTCTATTTGCAAGTCATA
ACATTGACTACGGCCAGCCAATAGCTTGTATATCATACCCATACAATGATTCGGGCTCAGGAGGTGTTTGGGCAGCCTTTGGGTCACGCACTAGCGCTGC
AGCTGTGTTCCATCCCCAAATTGCTTGTGGGGGCACATCTGCGAGAGTTCCCTTACCTCTGGAATTAGCAGATGATGAGCCCATATATGTCAATCCCAAA
CAATATCATGGTATACTTCGCAGAAGACAGCTACGTGCTAAGTTAGAGGCTCAGAACAAGCTAGTCAAAAACCGAAAGCCTTACCTTCATGAGTCTCGGC
ATCTTCATGCAATGAAGAGAGCAAGAGGTTCTGGCGGACGTTTCCTTAATACTAAACAGCTCCAGGAGCAGCAGCAGCTGAAGTCTCGCAATGCCTCCAC
CAGGTCCACCACAAATGGCGCAAATTCCTCAGGTTCAACACATCTACGGCTTGGTGGTGGTGCAGATGGAGATCAAACCATGTCGGGGACGAAAACAATG
GCCTCACAAAACAATAGCAAAAAGGCTGTTTCTTCTTCTGCAGCTCTTGCTTTCACCGTGACTCCTATGGTGCGCAAAGATGACACCTTCTTCCAGCACC
TCAGCCACAATGTCAGCTTCTCCAGCCATTTTGGCCAGGCAAGCGCCCAAACCGGCGTTGGAGGCATCCATAATGAGACCCAGCATAGGGTTTCCGTGAT
ACAATGACGGTTTGCGAAGCTTATCTGGTGATCCAGGCTTCTAGGTGTCCTCGTGTCCGGTGTAGTCTTGGTCGCTCAGGCAATTCATCCTTGGCTTAGT
TTTTGGTGTGTTAGAACCTACAAATGTTTCATACTCTGTTGTGGTTTGCAGAGCCCATAAATCAGGGCTCTCCTAAAAAAAATCAGGGCTTGATGGCGAC
ACTACTATCCAAGTATTGTTGTAATGGTGTGTGTTAGAAACTCGCAAAAAACCCTTCTTTGTGCTCGTATTAGTACATTATGCTGCACAATTAGCTGCTA
TTCTGTTCGTACTACAGTCTGGTATGCAGTGGT
>Locus_12535_Transcript_61/73_Confidence_0.264_Length_2122
AAGGTGGGGGCAGGTATAGAGAGAGATGGGAAAAGTTGGCCATATTGTGTGTACTGGATTATTGGGGGTCTTGCCTTTCTTATCTGGTTGCTTTCCTTCC
CTGTCTTTTGGTTTTTGAGGTCCTCATATTTCCCTGTGCCTGCGAGTTAGAAGAATTCCAATAAGACACAGATTCTTCAGAAAGAGGTTAAGGGGAGAAG
AGGAATAGGATATAGATCTCAGGAAGCCAGAGATGAGGTCGAGGGGGAGCCAGTGGCCAAGCTTGTGCTCCAGTGAGCCTGATTCTCTTGTTCCCAGCTG
ATCGATGTGCCACTGCCTTGGTTCGCTGGATCAGCGGATCTCCATGGTTCTGTGCGGTGCTGCAGATTGAGAAGAAGGTTTTCCAGGGGGAGGAATCTCT
TGTGGGTTCCTTTTTCTGGGAGCAACGTAGTGGGAGGTAGCTGCTTTTGCATACAATTAGGAGCAGCAGTTGAGATATTCAATTCGGGCATGGGTTCTTT
TGGGATGGACTGGAACCAGAAAGGCTCGGTGTTGTGGGATTGGGAGAATTTGCCGCCGATAGGCAGAAATGCAAACGAGAACCTCAAGATTGCGCCACAG
GCTGAACCGAAGTTTGCAGGTGTTGAGGCAACAAGGCATGAATCGGGGCATTCTTCTTGTGGTACTTTCTCTTCCAGCTCGGAGATGGGGTATGGTTCAT
CCAAGAGTTCCATATCAGCGTCGATTGATTCTTCACCCAAGGTGGGAAACAACGTGGAGCTCAATCTTGCAGCTGTCAAAGTGCCTGACAAAAACACCGT
CAAGAACACTGATTTGGGTAAAGTTGATGACGCTGGAACTTCTCCATCATCGATGATAGCCGTCAGCAGTGGAGAACCGGTGATTGGCCTGAAACTTGGC
CAAAGAACCTATTTTGAAGATGCATGCGGAGGGCAAAGTGTCAAGAGTTCACCATCCAATGTGAGTGCAGTGACCCCTGCTTCTGCGAAGAAAGCAAAGA
TGATTCAAAACGCACAGAACTCGTACTGTCAGGTTGAAGGTTGCAAGTTTGATCTCTCTTCTGCTAAAGATTATCATCGGAAGCACCGAGTCTGTGAAAC
TCATTCTAAGGCTCCCAAGGTTATTGTTGCTGGTCTGGAGCGACGCTTTTGTCAGCAGTGTAGCCGGTTCCATGCTTTAACTGAGTTCGACCAGAAAAAA
CGAAGCTGCCGGAGACGCCTCAATGATCACAATGCCCGCAGACGGAAGCCACAGCCTGAAGCAATTCCTTTCGGTTCATCAAGGCTCTCTGCAATGTTTT
ATGATGCAAGGCAACAGACAAGTCTTCTGTTTGGTCAAGCTCCTTATGGTCAAATGAGAAGCTGTGCAAACTCTTCATGGGATAGCCCAGTAGGAGGCTT
CAAATTTGGAGAAACAAAAGCTCCTTGGTTAAAGCCAACGAGAGCTGCAGGTGTTGATGGGATGCATGTATCAAGCCAGCAGGTGTGGAACAATATTACG
CCACACGGTGCACATCATGATTTTAATGGTATCATGGCTTTCAAGGGAACCAGTGCAAATGTCCTTAATCAAGGTGCCGAAGCTTCTCCGGTCGTCTCCA
ACTCGAATGGAGCCCCAGATCTTCAGCGTGCTCTCTCTCTTCTGTCAAACAATTCAGCTGGTGCTGCCAACGACCAGCCAACTCCTCAGCTGCACCCTGG
GCTGACCACCTTCGTCGGCTCCTCCAACCCTGTCACTGTGATGGAAGCCTCACCACCAGGACTCTGGCAAGACAGCACGCCCCTTGATCATCAGGCGCAG
TTCCAGGCTTTCGATCCCATTGCCGCACCGCAACAGCTCCAGCTCCCAAAACCCCCTTCCTTCAACAGCTCCACTTCCAGCTATGGCCAGATGCAGTGAT
GGATGCTTCCTTCAGTTCTGCTGTCTGAAACTCTGAAATTTGAAACTGCTGTGTTCTAAAAAATAAAGAAAAGCCTGTGTGCGAAAACTCAGGCTTGAAA
AATTGATCCAGTAGTGTTCCCGTTCAATGGCTTGAATAATCCATCCTTGTGATGGCGTGTAACTCAAATGCTTGGATTGCGCTGGCAAGTGATCATGGAC
TCTTGTGTTCTTGATGGCATCT
>comp80692_c0_seq1
GCAAAGGGAGGCAGCAAGCTTGAGACAACAGCTGCACAGCTTGCAAGAAAGTCACCGACAGTTGATGGGACAAGATCTTTCAGGATTGGGTGTCAAGGAG
CTCCAAAATCTGGAAAATCAGCTAGAAATGAGCATACGTTGCATCCGGACAAAAAAGGACCAAGTCTTGATTGATGAAATTCACGAACTGAATCGAAAGG
TTCTGGAATATTCTGTTATTACTCTGCAATGAGAGGTCTCTGAGTGGATTTCAAGAAGCTGACTCTACTGGCTCATTTTCAGGGAAGTCTCATGCAACAA
GACAAGATGGAACTATACAAAAAGGTCAACCTAATTCGTCAAGAGAACGTTGATTTATACAATAAGCTCTATGAGAACGAGGCAGCAAGTGAAGTCAACC
AATATTCAACAACTCCATACAACTTTGCAGTAGCTGAGAATGTCAATATTCCTGTTCGTCTTGAACTTAACACTCCACCGCAAGAAAATGATGTTGAGCA
AACTGCACCTCCTAAACTAG
>comp67351_c0_seq4
GAGCGGCGCACGGATTAGCCTGTCCAGCAACCCGGTGGGTGGCGCGACCAAAGGCGCCGCGTTCCAGACGGGTGACGATGCGAGAGGCGAGAGCAGCGAG
GAGTCACGGAGATCAGGGGAATCAAAAGATGGAAGTGTTGGTCAAGACAGCAACCATGCCACATCACATATGCCTGCTTTGGTGTCGGAGTATTTAGCAC
CATATACACAGCTGGAACTGAACCAATCAGCTCCTGCAACATATCAGTACCCAGATCCTTACTATGCAGGCATGGTTGGTCCCTATGGAACTCAACCTGT
GACCCATTTCCAGCTACCTGGGTTAACTCACCCTCGCATGCCATTACCTCTTGAAATATCTGAGGAGCCTGTCTATGTAAATGCAAAGCAGTACCATGGA
ATTTTAAGACGAAGGCAGTCACGTGCCAAGGCTGAGCTTGAGAAAAAGGTGGTTAAAGTCAGAAAGCCTTATCTTCACGAATCTCGCCATCAACATGCAA
TGAGGAGGGCAAGAGGAAATGGGGGTCGCTTCCTAAACACAAAGAAAAGCGACAATGGTACTCCCAACGGTAAAGCTGAACCTAAGAAAGGAGAGCAAAA
CTCCAAGCATATCCATGTCCCTCCTGATTTGCAACTTCGACAGAAGGAGGCATGAAGTAGCGGTCTGGCAACCTAAAACAGTGGCTTCTGTCCACAGGCA
TACACACTTTTGAGTCCGGATTTGGAGCCCCAGGGTTGCGCTGCCATCTTTGCAGTTCGTCTTCTTTGTGCAAAGAGGTCCAAGGTTCGTCCACCCGAAT

GAGCTTGGTACCAGTTGTTAGTCCTGGCAGAATGCAATCTGGGTGCAGGAGACCGTTGAAGTGTCCAATGCCATGGGTAGGTCTGCTGTTTTCAGGCAAT
TCATTCTTGGCTTTCACATTCCGCCCTTGTTTGTACCTGGTTTGTTGTAACTTGTAAGTGGAACTAGCTTGTGCTATCTTCGGCGCCCTGTGAAACATAT
GTATAATTAAGATAAACTTCGGATCATATTTGTATCCCGGATCATATTTGTATCCTATGTCTGGCTTTCGTTCAGTTTGAACTGTAACAAGGCCAAAGCT
GAACTCGTTAAACAGAGGTAGCAACTCATGTTGGCCTGTTGGGTGTTCATCAATGTTGTTGTAATGAATCCTGTACAAAGGCAGAAGCTTATTTCGA
>Locus_6904_Transcript_2/7_Confidence_0.364_Length_2520
ACAAGCAAGCTCGCAGCAGCGCCATTGCACGCTTCCGGTCGGCCGCTGCTGCGCTGCACGTCTGTCCTCCCGCCCCGTACCGCCTCGTACACATGTAAAC
TGCTGTGCTGTAGGTTTGGAGCCCCCGTACAAGCGTGTTCGAGTTTGCTGATGTTCATCTAATTATCCCCGGGGAAGGAGTGATTGTCCGCGGAGAGAAG
GAAGAGATCGAATTGAAGGTCAAGATAAGTTATTGAAACGGCAAGATGGTTAATTTTGGAAAGAAGTTGATGACAGATCAAGTAGAAGAATGGAAAGGGT
ACTATATCAATTACAAACTGATGAAGAAAATGTTAAAGCAATATGTTCAACAGACCCAATTAGGCGGGAAAGATTGTGAACAAATCCTTAAAGAGTTCTC
GAGGATTCTTGATGACCAGATTGAAAGGATTGTGCTTTTTCTGCTACAACAACAAGGCCACCTTGCCAGCAGGATTGAGGAATTGGGAGAACAACACACT
GCTCTTATGGAACATTATGATATATCACAAGTTTCCCAGCTACGTGATGCGTATAGAGAAGTTGGGAGAGATCTTATTAAGCTTCTGCGCTTTGTTGACA
TGAATGCTACTGGTATACGCAAGATACTAAAGAAATTTGATAAGCGTTTTGGCTATAAGTTTACATATTATTATGTCACCACTCGTGCAAATCATCCCTA
TTCTCAGCTTCAACAAGTATTTAAGCAAGTGGGAATTGTAGCTGTTGTAGGTGCATTATCGCGCAATCTTGAATATCTGCAACATCATCAAGGAAGCTTT
GTATCCATCTATGATCATCCATCAGTTACCTTGAAGGACCCTATAATAGACGAAGTAAACCATGCGGTACAGAAATTTACGCATGCCACCAATTTTATGA
AATTCTTGGGACAACACGCGCTTATTGTTCCAGAGGATGCACGAAGCGGGTCTGAGGATCTTGTTGATGATCAGAGCTACCATTTCATGTCTCTGCTGCT
TAACCTAGCGAACACTTTTCTTTACATGGTGAACACATATATCATTGTGCCAACTGCAGATGACTATTCAGTAAGCCTTGGGGCTGCTGCGACTGTCTGT
GGTGTAATTATCGGATCAATGGCAGTCACTCAAGTGTTCTCCTCGGTTTATTTCAGTGCATGGTCAAATAAGTCATACTTCAGACCACTTGTATTCAGTA
GCATTATGCTATTTTTTGGGAACCTGCTATACGCATTGGCATATGACCTGAATTCATTAATAGTTCTCCTGATTGGACGACTACTATGCGGGTTGGGCTC
TGCAAGAGCAGTGAACCGTCGCTATATTAGTGATTGTGTGCCTCTCAAAATCAGGCTACAAGCTTCTGCAGGATTTGTCAGTGCTAGTGCTCTTGGCATG
GCATGTGGCCCTGCTCTTGCTGGTTTCCTCCAGACAAAATTTAAGATATACTCGCTCACTTTTAATCAGAGCACATTGCCCGGATGGGTCATGTCCATTG
CTTGGCTTCTTTACTTGTTGTGGCTGTGGTTTACATTCAAAGAGCCAGAACACTTCGCTAAAACTGTCATCAATGCACAGCCGTCTGAATCAGGTCACCA
AGGAAGTGCTAATTTGGAGGAAGGTCTAGCTCAACCATTGCTTCTGGGTACAGAACAAAGACAGGGCGAGAATGCGGATGACAATGATGATAATGAAGTA
GACTCTGAAAACTCTCATGAACCAGCAACATCAATTACTTCAGCATACAAATTGCTGACTCCATCTGTCAAGGTCCAGTTGTTGATATACTTCATGCTCA
AGTATGCTATGGAAATTTTACTCTCTGAATCAAGTGTGGTCACCACATACTATTTTAAGTGGTCTACAAGTTCTGTGGCAATCTTTCTAGCGATTCTTGG
ATTAACTGTTCTTCCAGTCAATGCCATTGTTGGGAGCTACATTACAAATCTGTTTGAGGACAGGCAAATTCTGGTTGCATCTGAAATCATGGTCCTCATC
GGTATAACCATGAGCTTTCGCTTCGCCCCTCACTACTACATTCCACAGTACGTGTCTTCAGCTCTCATCACATTTGTATTCGCTGAGGTGCTCGAAGGAG
TGAATCTATCGTTGCTTTCTCGGGTAATGTCGCCGAGGCTTTCGCGAGGGACCTACAATGGCGGACTCCTCTCCACAGAAGCCGGGACGTTGGCCCGTGT
AATTGCAGACGCGACGATTACTACAGCGGGTTACCTAGGCCCGGACCTCCTTCTCAACGTCACCCTGCTGCCACCTCTAGTGATCTGCATAGTGTCCATC
GTCGCAACGTTCTGCACTTACAACAACCTTTGCTGATAAAGCAGCCCCTCTGTGTCCAGCCAGTTAAAATGACTCCTGCCATATTGGGCCGTACCGTACT
ACTTGAAGTAGCATCAGCGTTGTAAATCTGGTAAATCTACAAATACCATGGACGGTGCCGGTGTGGCGGTGTGTAGTGTATTGTTATGCATTCAACAACT
TTCTTGTGTCTAGTAGAGTT
>Locus_5417_Transcript_28/28_Confidence_0.219_Length_5212
ATTACAAGACTATTTGATGCAACAATCACGCATATGGCTGCGCTAGAACTACTGCTCTGAATTAAGAGGCGTTGAAATATTGTCCGGTCTACATATAGGC
ACTTGAGGTGTATTGACAGACAGTTCCCCTTAGTTTGCTACAACACATCAGGCGAATTCCACCAAATTAGGATGATGTTTGTTTCTTAGACACAGGATAG
CAATGCCAAAAGTTTTTTATAATTTTCCGCTTCACCGCAGATTTGGCATCTCAAAGGGAGTTGTGGCAAGATTCTCTTGACCAAGCTTTCTTGCAGATTT
GGCAAGAAAGATAAAAAAAATGTTGGTATACTTAAGCATCCTTAATAGAAACTAAACAGGTCTTAGATTGAAATTATCATAAATAAACCCTTTTTCACTT
TAGCTGTAGTCCGGATGAAATCTTAGGGGCCTTGCTTAGCCTGCCCTGAAGCTTGCCAATAAGTCGAAGCTGATGCTGACACAGCACTGAAATAGATCCC
AATGAACACAAGTTATTTCGAGAAGATAAAATACCAAGCAAAGCCAGGATTGCATTAAAAGATAGTGTATTTCTACTATACGATCTGAAAGCTTAATTTA
TTATATCAATAATCAAATTGGCAGCAGTGTGGAACAGATATAAAGAAGATGCTTCAAATCACAAATAACTACAGATAAATTGAAGGGTGAAGGTTCATTC
AACCACCAAACACCCAAAATGTCGCGCTGCATATACACAGAACCTAAGTACGGTACAGTTCATAGTTTGGGAAAATAGACTGCAAATGTGCACAGATAAT
ACAGATTGTTACTCGATGAACAGTAAACACGGTATTCGAAATCCTTCTAACCATCCATCCAGGCAGTGCCTCGTATACTCAAACCTCTTTTTGGAGGAAG
CCAAACCATGTGATCCTTTTCAACCCAAATACATATATTCAAGATGAAACTAACAAAAAGACAGTTGATAAAGAAACACGGAAGAGAGTAGAAAAAGGGT
ACCTGTTTCAAGTTCCCATCTTCTGAAAATGATCAGCAGTCAATAGATGTTTTAGTCCATTCTCCAGAAAAATGAGTGCCGCATGAATCCTTTCCGGTTT
ATAGTGAAACATTTTAGACGATGACCTTTAACATTCTAGAAAACGAAGAGAAAACCTAAAGTCACCTAATTTCAGCTTAAACATGTTCCAGCAAGATCAT
CACCATCCTGACATGTTCCGTTTTGTCCCTAGACCTGAGCACAAGGTATTGCGAGGCTAATTTAGCGGGGCAATTGATTTTTTGCCACTATGATACAACT
GCAATTACCCGCTGCCACTGACACGTGAGGTCACCTGAGTCACTGAAATGTGGGCCCAGTGGCATTTTGTTTTGCCACTCCCCTTCCAGAGTGGCAAAAA
GTTAATTGTCCCTTAATTTAGCTCAATTCAGTTGTCGGAGCAATTGTGTGTGAAAAGTGTGAACAACAGCATAGGAGACAATACAGCAAGTGTAGCGTGC
CAATATGTGTCCAGCTTGATAGTTCACTATAATAAAGTTCTTTAATTCAAGTATTTGAATAATATAGAATCTCAACCTTCAAGTTTAAAAGGTTTTGTAT
TTGCTATTATAATCGTTGCCACCCCCTCACCCCTCTCAAAAAGAGAGTTACTTTGTAGCGCACTGTAAATCTCTTGATTGAAATAGTGGCATGTCATCTT
ATTTAATTCAGTTATTTCATAAGGTACAATTTGTTTAGCTGACAGCTCAGACATGCTTCTATGCAACATGTTTATACCAATGCTGTATTTTGGCAATCCT
GGATGTGGCATTACTTAACTGTGCTTTCATATTCAATAGTTCTGGCAAAGGGAGGCTGCAAGCTTGAGACAACAACTGCACAACCTGCAAGAAAGTCATC
GGTATGGCTAGAAAACCCTGCAAATTTTACAAATGTACAAGTCAGAAAATTTGCCTAATGTAAAAGGATCAAACTACAGGATTTGTCAGCATGAGCACAA
GTGCCTAACTTCCGAGTTAGATAACTCTGGAGGAAGGAAATCCTTCCTACTGTTTGTTTCAAGAATATACAAAAATTCAAAACAACATTAGAATAGAACC
AAAAGAAGAAACAATATTCACCATGTAGCAATACTCAATTCCTGGTTGTCTTATTTATAAAGGTATAAAACTAGGTTCTGACGAGACAATTATGAATCAA
CTACACCATGTTACAGATCATCCATAACTCTTGGTGATGCAAATTAAGGGCTGTAAAGTGACAAGTATACCTCCACCAACTAATTTGTGCAGAGTGTATA
AAATTGTAAAACAATGGATAAGAATGAATTTAGTATACAAGATAGCATACAATGCCCAAGTTTAAGGAGACATACTGATTTACTGACTCCATAGGACAAT
CTTTTGCTTATGAGGACATCAGTTTTCATAAACAATATCAATGTAAAAGGAGGTAACAGTTTATGAATTTCCTATGCCAAGCCATTTATAAGTCAAAAAA
TGTACCTATTTAGCTTTTATGCATTCCAATTAACTTCCAAAGAAGAAGAGTACATACTAATGGAAAACAAATATGATATTATCATCTGTTTTGCCCGGTA
GTTTCTTGAGTCTCTGATTTGTCCGCACTTCACTCCCAACCAACACAACATTAATACACATGGGCAGAGGAATATCAGGTTATCAGGTAAACTGAATACA
GTTTGATTGACTGCAGTCAGTTGATGGGACAAGATCTTTCTGGATTGAGCGTCAAGGAACTCCAAAATCTAGAAAATCAGCTAGAAATGAGCCTACGTTG
CATCAGGACAAAAAAGGTAAGGATGCTAGCCATATTTCTGTTTTTCGTTACCAGATTGCCTCATCCAAGAATACAACTGATGATGACGTTTTTTACTTTC
AGGACCAACTCTTGATTGATGAAATTCACGAACTGAATCGAAAGGTTCTGGAATATTCTGTTATTACTCTGCAATGAGAGGTCTCTGAGTGGATTTCAAG
AAGCTGACTCTACTGGCTCATTTTCAGGGAAGTCTCATGCAACAAGACAAGATGGAACTATACAAAAAGGTCAACCTAATCCGTCAGGAAAACATTGATT
TATACAAGAAGCTCTACGAGAAAGAGGTGACAAGTGAAGTCAACCGAGATTCATCAACTCTAAACTTTGCAGTTGTCGAGAACGTCAACATTCCTGTTCA
TCTTGAACTTAACACTCCACCGCAAGAAAATGATGTTGAGCAAACTGCACCTCCTAAACTGGGGTAACCCTGCTTTTTTCTGCAAAGGAACTATTGATTC
TGTTCTTAATTCTTTAGTGGCATATTGTTTACTACTAACTGAAAACTTGCAGATTACAACTAAATCCATGAAGGCATGCAGAATGGCACCGCTTATGTTC
ATTATTCTCAACGGTACGATTCAAAGCAACCAGAAACACTATTGGTTGTAAAGAGCTTAAACACATAGCAAGACTGACGCGGTATGCATGCCATGAGTGA
TTGATAATGGTAGTGTAAATCTATTTCAAATAAAATGTATTTTATTGAAGAATACAGCAAATGATTTGTATTATAACTATTTGACACATTCTGCAAGTTT
GTTGACCAACGTGAGTACATGATAGCTACAAATCTTATATTGACAAAAAGATATGGTGTGTGGAAAAAATTCAGGTGCTATTTTTTTCTATTTTGAGAAT
TGTATGCATAGCAGAAGAGCACAAATTGCATTCTGGTTTATCCGTGCAGGCATTCCAGAGTATCAGAATGGGGATCAACCTTTCTTCAGCTACATATGTA
AATATACGGATGCTAGTTGCTCACTTAAAATTTTGTAGTGTTCAAAGGAATAACAACAGTAAGACGTTGACGCACCGAAAATAACCAGAAATAACGGATT
GTACTATGACAAGACACACGACAGATACACTAGCTTAAGTTTGTACAGGAAAATATTTGGCCTATTCTTATGTCACAGTAAATTAAGATTATCACCTTAA
TCACAGAAATTCAAGGGGTAAACGATTATAGCACAAAACGATCCTGGACCCCAAGACCTGGGTCTCTGAACGGCTGAACCTCCAATTATACCTCTATACA
TTTGGAATGTGTGTAGTCAAACTTTCGCTACTGGTATCTTTACAAATATTTACATTGAACACATCAAAATGATATACATGGATTCCTCTCGATATGTCTT
TGAGGTTTGGGTTCTAAAAATGATATACAAATTATTAGGTTCTAAACTTAAATGATATATACACATCAAAATGATATACAAATTATTGGGTTCTAAACTT
AAATGATGTATACACATCGAAATGATATACAAATTATTAGGTTCTAAAAATATACTATGGCAGAATTAGGTGGTCATTTGAGTTTTGGAGGCCTTGTCAA
TGTCCCAAACATCACTTATTTGTGACTGGGAGAGCAATGAATTGAAGGAATGAGAGTAACCATACGGTCATTGGTCAATTAAAAGGAACTTTAATATGCT
ATGACCACATCGACCTTTCGTAAAGTAAATAACTCAATTATAACCAAGTAATTGGGTGTCTATTGAATCCATACGGTTCTCAAAACAGTTCTAAACAAAG

TGAATTTAAGCATCCTGAAAAGATATTTGGCAAATTTGCCATACCCTGAATTCACAGCATGTTAACACAAATCATGTGCAAATCATTCAGATCATTGACA
AACGCACGCATGCACACAAATCAACACCGTTTTCTCATGTATAGAACAGAAAAATTGAGAAACTTACGATTTTCTTGCAAGTTATGCAGTTGTTATCTCT
AGCTTGCCGCCTCCACTTGCAGTAACTGAGACGCCGGCGCCGAGCTTGAGCTTGAAACCTAGACTACGGGGACAGGGTAGACAGGAGACGCGAAACCCCA
AACCAGCGCAAAACGACGCGCAGGGAGGGAGTGTCACTGAAGGAGCGACAGGGAAAGCAGCGGCACGCCGGGGGAAATTCCGATCTGAGAGAACGGATCA
GCCATGCATGACAGGAGCTGCGGAGGCAGCAGGGGGGGGGGGCTCCCGCTGCCACGGGACAGTAGGGCACGCCAGATCGGTGAAATCCAGGCGAAGGACG
AGGCGTGGGCGAGATTTACACGACGGCGCGATAGGATCTAGCCCGGATTTGGATTCGACCGACGAGGGCGGAAGGAACGGCAGGGTGGTGCTCTTGTAGA
GAGAGAAGGAGT
>comp79584_c0_seq58
ATGATATAATAGTATGTATAATCTAGAAATAGTAGGTTAGATTCATGTAGCATAATGGTTGTTTGAACAAAAGAAATTAAAGAGAAAAAGGGCAGTGGAC
AGGGAATTCCTCACCGGTGGGAACGCCATGCTTGGGTGCAGGTACATATATCTTGGGCTATTCGACAGCGAAGTAGAGGCTGCAAGGTTGCTGATCTTGG
ATTCTTCTATTGATGCAAAAAAAAATTTTGCTTTACCTTCTCTGTTGATTTCCAAAGGGTTGAGTACCGACTCGATTCTCTTCTTTGTTGTTTTTCTTCT
TTCTGTTTCAAAATTCAGGGCTTATGATAAGGCCGCGATCAAATGCAATGGTAGAGAAGCCATGACGAACTTCGAGCCTAGCACGTATGATGGGGAGCTA
TTGACTGAAGTTGGTACTGAAGGTGCTGAAGTCGATCTGAACTTGAGCATATCTCAACCAGCTTCTCAAAGCCCGAAAAGGGACAAGAATTCCCTTGGTC
TGCAACTCCACTATGGATCGTTTGAAGGCTCGGAATTGAAAAGAACAAAGATTGATGCTTCTTCTGAATTGGCGGGCCGCCCTCATCGGTTCCCTCTTCT
GACCGAGCATCCACCAATCTGGACTGCCCAGTCTCACCCCTTCTATTCAAATAATGAGAATGCATCAAGAGATCTTAACAGGAGGCCAGATCAGGGGGGT
GTTCCCAGCTGGGCGTGGAAGGTGACCGCCCCTCCTCCCACGCAGCCATTGCCGCTGTTCTCGTCGGCGTCATCATCCGCTGCAGCATCATCAGGATTCT
CCAATACCGCCACGACAGCTGCCCACACCGCCCCATCGGCCTCCCTCCGGTTCGACCCGCCGCCGCCGCCGTCCAGCCGCCACCGCTGAACGTTAAGAAG
CCACGCTGTAAATTTGCCAGGAAGCCGACATTTTTTCCTCTCTCGGCGTTGCAACTTTTTAGGTTTTGCGCCGGGGTGGTTTCTTGTAGTGGAGTGGATT
CATAATTAACTGTATTTGCATGCTGCCCATGTGAAATGCTTCTCTCTCTTTTTACGCTCTCTGCACCGGATGTTCTGGGAGTTCTTGTTGCCGGGAATTA
ACTAAATTCACCGTCTGAGATTGATCTATACATTGTTGTAGAGAATCGAATCATTGTTGAAATTAGTTACAGAATTATAGATATCATAAGGCACCCTCTA
CTTGTGGACAAAGTTCAGGAAGATTATTCCGGTTCTTTTCTTGGCTGT
>Locus_2827_Transcript_67/166_Confidence_0.034_Length_1718
GCTGTTGATTGGGTCCAGATGCCTGGGATGAAGCCTGGTCCGGATTTGTTTGGTATTGTGTCTGTTTCACATGGTTGCCGAGGTGTTGCTGCCCGTGCCT
GTGGTTTGGTGAATCTAGAACCAACAAAGATTGTGGAGATCTTGAAAGACCGCCCATCTTGGTTGCGTGATTGTCGGAGCCTTGAAGTCTTTACAATGTT
ACCAGCTGGAAATGGTGGAACTGTTGAGCTTGTTTGCATGCAGATGTATGCGCCTACTACTTTAGTTCCTGCACGAGATTTTTGGACGCTGAGATACACA
ACCACTATACAGGATGGCAGTCTTGTGGTCTGTGAGAGATCTTTTGTGTGGCTCAGGAGGCGGTCAAAGTACTGTATCGGCACAACAATTTGTTAGAGCA
AATGATAGTGCAAACCTATGGGTGACAAGTTGTGTATATGGCGCGCTCGCTCCCCCTCCGCCTAGCCTCGCCGCCCGCAGCCCTCACTAATCTGAATGTT
ATATAATCCAGGTTGCTGAAATTGAATCCATTGGACAGGGAGAAGGGTGATGAAGCGTACGTCTCTCTCAGGCTTGAGCTGCCGCAAAGCTCTATGGAAC
CTGGCACTGTTGTAGACGTCTTTCAAGTTATTGATATATGACCAGATTTATGGAAAGCACAGTGAAAATCGAGTGAGCCACAATTTCCACACTGTAAGCG
CAAGCTCTGGGACTCATGCATTCCCCTGAGGACACTGAAGAAACAGTCCTCTGCATTCCTTGTTCGCAACAGCTGCGTCTTTGGTGTTGAGTTCATCAAA
GTTGTCACCGCAAAAGCAAACACAACATCTGAAACACTCTTTGTCCAGAAGACGAACACCATCAACGAGGCCAAAACTTACACTTGGGACGTCGAGGACT
TCTTTGCACTGAAGAACCCGGACTACTCTCCGGAGTTCGAAGGTCAATTGGAATGCTTCCAGATAGTGATGGGAATCACCTTTTCTTGTGCCTCAGAATG
GAAACACCAAAGAATCTCCTGAAAGACTCTGGGACCCTAGTAGAATGTACCATATCCATCAAAGACCAGGAAGGTGGCAAGCATCGGGAAATAACAGATT
CGCCTTTTTAGCTGAAATTTGCTTAATTGTCAGGGGTCCACATGCTGAATTATCACGCCAATTCTGAATTTGTTTCACTGGGAAATGTAGGCCGCTGCCA
GTTCTCAGAAAATAAGTCTGGACAGGGATGGGGCAAGTTCATTTCGCTAGAGGATTTCAAGGATCCATCGAACGGTTATCTTGTGAAATCCAAGTGCTGC
ATTTTAGCTGCGGTTGCGATCATTGGTTCCTCCAAGACGGACTAGACTATCTACATGATGTGCGTGTGATCATGTATGTGCACGCATGTTTCTTTGCTAG
TCTGGTGAAGATTGCTGAAATTGCAAGTGGAAAATAAGGTTCAGTTTCCCTGTCATGTCCTTTCTTTTGTGAAGGGATTAACTGCAATAGCGTAACTAGT
ACTAACACCTCTACTATCGTATCATGAATTCTGTATCCATTACATCTGTGTGGTAGCTTTGATCATTTCTGAGAACCGATGAGAAATGTAATACTAGGTT
GCATCAGCTCAGTTGGACGTATATATCTGCCGCATGCAGGAATATGAATATCCAACAAAGCATGCTTATGCATTGTATTTCTGATCTTCCTGCCCATGAT
TTTAATGCATTGTTCAGC
>Locus_5523_Transcript_48/107_Confidence_0.037_Length_432
TAGGAAAGCTTCATTCCTGCAACAAGGAGGATCAGAAAGAGAGAGATAGGGAGGCACCAAATCGGGCTCCTCCAATTAGTCAGAGGAGAGAGAGAGAGAT
CTGTGCTAGCTGTGGGGGAGGAGATGGGGAGAGGGAAGATAGTGATCCAGAGGATCGACAACACGACGAGCCGGCAGGTGACGTTCTCCAAGCGGCGGAG
CGGGCTGTTCAAGAAGGCCAAGGAGCTCGCCATCCTCTGCGACGCCAACATCGGGCTCATCGTCTTCTCCAGCACCGGTCGCCTCTATGACTTCTCCAAC
ACCAGCATGAAGTCTGTGATCGAAAGATACAATGAAGCAAAAGAGGATCATCACCTGACCATGAGCGCAAGTGCGGAGGCTAAGGAAGCAGGAAGCTTGA
GGCAGCAACTGCATAACTTGCAAGAGCACCAT
>Locus_17579_Transcript_43/92_Confidence_0.265_Length_2096
GAGGAATCTCTTGTGGATTCCTTTTTCTGGGTGCAACGAAGTGGGAGGTACTTGGTCTTCGCACCTTGATGTACTTGTGGGTGCGTGTGTGCGCGCGCGC
GCGCGCCTTTCTTGATGCTAGAATGATGGTGGTGCTGTTCTTGCCATGATTGACGATTCCCCTAGAAAAAGTTGAACTACTCTGCACTCTTTCTAGCACA
TGGATAGCACCTTGTGTACTTCTCTGATGATGTGTAGTTGGGTGCTCAGATATGTTGTTGCAAATCTGTCCAATTTCTGCTACTCTCATATGTCAAATCT
TTTCTGTTTTTACTCTGTTTCACTCTTCCACTTTGTTGCTTCAAAGGTAGGAGCAGCAGTTGAGATTTTCAATTCTGGCATGGGTTCTTTTGGGATGGAC
TGGAACCAGAAGGGCTCCGTGTTGTGGGATTGGGAGAATTTGCCTCCGATAGGCGGAAATGCATACGAGAACCTCAAGGTTGCACCGCAGGCTGAACCGA
AGGTTGCAGGTGTTGAGGGCACAAGGCATGAATCAGTGCATTCTTCCTGTGGTACTTTCTCTTCCAGCTCGGAGATGGGGTATGGTTCATCCAAGACTTC
CATGTCCGCGTCAACTGATTCTTCGCCCAAGGTGGGAAACAACATGGAGCTCAATTTTGCAGCTGTCAAAGTGCCTGACAAGAACACCGGCAAGAATACT
GATTTGGGTAAAGTTGATGACGCCGGAACTTCTCCGTCATCCGTGATAGCCGGCAGCAGTGGAGAACCGGTGATTGGCTTGAAACTTGGCAAGAGAACCT
ATTTTGAAGATGTATGTGGAGGGCAGAATGCCAAGAGTTCACCGTCAGATTCGACTACAGTGACTCCTGCTGCTTCTGCGAAGAAGGCAAAGATGGCTCA
AAACACACAGAACTCATACTGTCAGGTTGAAGGTTGCAAGGTTGATCTCTCTTCTGCTAAAGATTACCATCAGAAGCACAGAGTCTGTGAAGCTCATTCT
AAGGCTCCCAAGGTTATGGTTGCTGGTCTGGAGCGACGCTTTTGTCAACAGTGTAGCCGGTTTCATGCTTTAACCGAGTTCGATCAGAAAAAACGAAGCT
GCCGGAGGCGCCTCAATGATCACAATACCCGCAGACGGAAGCCACAGCCTGAAGCAATTCCTTTCGGTTCATCAAGGCTCTCTGCAATGTTTTATGATGC
AAGGCAACAGACAAGTCTTCTGTTTGGTCAAGCTCCTTATGGTCAAATGAGAAGCTGTGCAAACTCTTCATGGGATAGCCCAGTAGGAGGCTTCAAATTT
GGAGAAACAAAAGCTCCTTGGTTAAAGCCAACGAGAGCTGCAGGTGTTGATGGGATGCATGTATCAAGCCAGCAGGTGTGGAACAATATTACGCCACACG
GTGCACATCATGATTTTAATGGTATCATGGCTTTCAAGGGAACCAGTGCAAATGTCCTTAATCAAGGTGCCGAAGCTTCTCCGGTCGTCTCCAACTCGAA
TGGAGCCCCAGATCTTCAGCGTGCTCTCTCTCTTCTGTCAAACAATTCAGCTGGTGCTGCCAACGACCAGCCAACTCCTCAGCTGCACCCTGGGCTGACC
ACCTTCGTCGGCTCCTCCAACCCTGTCACTGTGATGCAAGCCTCACCACCAGGACTCTGGCAAGACGGCACGCCCCTCGATCATCAGGCGCGGTTTCAGT
TTTTTGATCCCCTGGGCGACGGCAGTGCCATTGCCACACCGCATCAGCTCCAGCTCCTAAAACCCTTCGCCTACGACAGCTCCTCGTCCCACTATGACCA
GATGCACTGATGCTTCCTTCAGTTAGCGCAAACCGCGGTTCAGTTGCGCGTAATCAGACATGGTTTCCACTGTCTGAAACTCTGAATTTTGAAACTGCGA
TGTTCTGAAAAATAAAAGCCTGCGTGCCGCAAACTCAGGTTCGAGTTGATCTAGTAGTGTTCCCGTTCAGGGGCTTGAATAATCCATCCCTGTGATGGCG
TGTAACACAAATGCTTCGATTGCGCCGGCAATTGATCATTGACTCTTTTCGTGTTCTTGATAACATCTCAATTACACCTTGAATGGAACACTGGTT
>Locus_17579_Transcript_76/92_Confidence_0.230_Length_2939
CTTTTTCTGGGAGCAACGTAGTGGGAGGTAGCTGCTTTTGCATACAATTAGGAGCAGCAGTTGAGATATTCAATTCGGGCATGGGTTCTTTTGGGATGGA
CTGGAACCAGAAAGGCTCGGTGTTGTGGGATTGGGAGAATTTGCCGCCGATAGGCAGAAATGCAAACGAGAACCTCAAGATTGCGCCACAGGCTGAACCG
AAGTTTGCAGGTGTTGAGGCAACAAGGCATGAATCGGGGCATTCTTCTTGTGGTACTTTCTCTTCCAGCTCGGAGATGGGGTATGGTTCATCCAAGAGTT
CCATATCAGCGTCGATTGATTCTTCACCCAAGGTGGGAAACAACGTGGAGCTCAATCTTGCAGCTGTCAAAGTGCCTGACAAAAACACCGTCAAGAACAC
TGATTTGGGTAAAGTTGATGACGCTGGAACTTCTCCATCATCGATGATAGCCGTCAGCAGTGGAGAACCGGTGATTGGCCTGAAACTTGGCCAAAGAACC
TATTTTGAAGATGCATGCGGAGGGCAAAGTGTCAAGAGTTCACCATCCAATGTGAGTGCAGTGACCCCTGCTTCTGCGAAGAAAGCAAAGATGATTCAAA
ACGCACAGAACTCGTACTGTCAGGTTGAAGGTTGCAAGTTTGATCTCTCTTCTGCTAAAGATTATCATCGGAAGCACCGAGTCTGTGAAACTCATTCTAA
GGCTCCCAAGGTTATTGTTGCTGGTCTGGAGCGACGCTTTTGTCAGCAGTGTAGCCGGTTCCATGCTTTAACTGAGTTCGACCAGAAAAAACGAAGCTGC
CGGAGACGCCTCAATGATCACAATGCCCGCAGACGGAAGCCACAGCCTGAAGCAATTCCTTTCGGTTCATCAAGGCTCTCTGCAATGTTTTATGATGCAA

GGCAACAGACAAGTCTTCTGTTTGGTCAAGCTCCTTATGGTCAAATGAGAAGCTGTGCAAACTCTTCATGGGATAGCCCAGTAGGAGGCTTCAAATTTGG
AGAAACAAAAGCTCCTTGGTTAAAGCCAACGAGAGCTGCAGGTGTTGATGGGATGCATGTATCAAGCCAGCAGGTGTGGAACAATATTACGCCACACGGT
GCACATCATGATTTTAATGGTATCATGGCTTTCAAGGGAACCAGTGCAAATGTCCTTAATCAAGGTAGGTTCCTCTCTATTATAACTGACGTGACATTAA
TAAGTTGTGGTCCTTCACTTGCAAGAAATCTGTTTTTAATAAAATTCCTAAGTAGAAAAAAATTCATTGATAGATGCACCCATATGATATTATTACTCTA
GGAGCCATTCGATAGAATTTTATCCTTTGGATGATTCTTCTACATTCTAGCTGTTTGTGTATGACTGCTTGTTTGAAGCTGTTAGGTTGTTAGTACTACC
TGACATGAAGTTTTATGAAATTTGTGACACATACCTACATCATTTCATCATATAAGTGAGTCACCATACAAGTATACGGCATGTTTCTAGTGTTGCAATA
TGAGAACCTTTCTTCGTGAGGGAGGATATGAGAACCTAATTGCTAGGACACATTCTTCTCCATGTTTTATCTTGGCCAGGGAATGACCATTACCTGCTAT
TTTCAGTAATATCCAAGTAATCTCCTTGTCTTTGCAAGATGTTTGCACATTTTTACTCTAGGTTTAAATGCAGTAGTGTACTATTTATGTTTTGCAAGTG
TTGAAGCAGCAAGGATTTGCAGTTAATGTAAAAATTATCCAGTTTACCAGAGCTGTCACCATCCTTTAGTGTCTGCCACCTATACCTTAAGTCAACTCTT
CATAATATGGCCACAGGATACAGCATGATAAGCCAACTTTCCAGACACCACTCCAGAATGATTGGAAACCTAGGACAGTTTGTCTGTAAAGTGCGTGGTG
TATGTCATGGTTGGCTCCAAGATTTGGTACGTTGCGAGAAAGATTGCAACCGCCAAATGCTTTTTGTTATCTCTAATTGATCGATTGCCCAGGGCATCCA
TATTCCAGGGAAGACTAACTGATTGATTGCCTTTATGTCACTGTCTAGTGTGAAGCATGATTGCGCCTCTGCTGCTCAACATGTGTGGGTTGATGGTGTT
ACAAACGCAGGCAGGCATTGGAGTCATAATGTTGTTATTCTGTTCAATGATCTTATCATTATTAATTCCAGTAGGACCATGTTCTCCATATGCAAAATCT
TGGCTCATCTATTAACTAGAGTACTAACTGAATTTCCCATTGCACTCTTATCTCTCATAGTTGAATTATAGCCATCATAAGCATGTTCAATACATTTGCG
AATTTTGATTAGTGATGAACATAATAGTAACATTTTCCCTATCTCTGATCCTTTTTCAGGCGTCGAAGCTTCTGCGGTCGTCTCCAACTCGAATGGAGCC
CCAGATCTTCAGCGTGCTCTCTCTCTTCTGTCAAACAATTCAGCTGGTGCTGCCAACGACCAGCCAACTCCTCAGCTGCACCCTGGGCTGACCACCTTCG
TCGGCTCCTCCAACCCTGTCACTGTGATGGAAGCCTCACCACCAGGACTCTGGCAAGACAGCACGCCCCTTGATCATCAGGCGCAGTTCCAGGCTTTCGA
TCCCATTGCCGCACCGCAACAGCTCCAGCTCCCAAAACCCCCTTCCTTCAACAGCTCCACTTCCAGCTATGGCCAGATGCAGTGATGGATGCTTCCTTCA
GTTCTGCTGTCTGAAACTCTGAAATTTGAAACTGCTGTGTTCTAAAAAATAAAGAAAAGCCTGTGTGCGAAAACTCAGGCTTGAAAAATTGATCCAGTAG
TGTTCCCGTTCAATGGCTTGAATAATCCATCCTTGTGATGGCGTGTAACTCAAATGCTTGGATTGCGCTGGCAAGTGATCATGGACTCTTGTGTTCTTGA
TGGCATCTCAATTACACCTTGAATGGAACTCTGGTTCTT
>Locus_15085_Transcript_11/40_Confidence_0.340_Length_1603
CGACTAGTAACCAGATGTCGCAACCTCAAGACTCTCAAGCTCAACAATGCTATCCCTCTTGACAAGCTTGCTAGCCTCCTTCGTAAGGCTCCTCAAATTG
TGGAGCTTGGAACAGGAAGGTTCTCTGCTGACTACCACCCAGATCTCTTTGCAAAGCTTGAAGCAGCGTTTGCTGGTTGTAAAAGCCTACAAAGGCTTTC
TGGGGCTTGGGATTCTGTTCCAGAGTATCTGCCAGCATTTTATTGTGTATGTGAGGGCCTCACATCACTTAATCTGAGTTATGCTACTGTGCGAGGCCCT
GAGCTGATAAAATTTATTAGCAGATGCAAAAACTTGCAACTATTATGGGTGATGGACTTAATTGAGGACCACGGTCTAGCTGTTGTGGCATCATCTTGCA
ATAAACTACAGGAGTTGCGGGTCTTCCCTTCTGCCCCTTTTGATCCAGCTGAGCAAGTTTCATTGACTGAAAGGGGACTTGTTGATGTTTCTGCCAGTTG
CCCGAATGTTGGAGTCAGTCCTCTACTTCTGCAGACGGATGACTAATGAGGCCCTTATTACCATAGCAAAGAACCGGCCCAACTTCACCTGCTTTCGCTT
ATGCATCATCGAGCCTCACACTCCAGATTACATCACGCATCAGCCTCTTGATGCAGGTTTCAGTGCCATTGTGGAATCATGCAAAGGCCTCAGGCGCCTA
TCTGTCTCAGGCCTTCTCACAGATCGTGTATTTAAATCCATTGGGGCACATGCTGATCGTCTTGAGATGCTCTCAATCGCCTTTGCTGGGAATAGCGGAT
TGGGCCTTCATTACATCCTTTCGGGCTGCAAGAGCTTGAAGAAGCTGGAGATCAGGGACTGCCCATTTGGTGATAAGCCCTTGCTGGCAAATGCTGCCAA
GCTGGAGACAATGCGATCCCTTTGGATGTCGTCGTGCTCACTGACCCTGGGCGCATGCCGACAGCTTGCACGCAAGATGCCCCGCCTTAGTGTGGAGGTC
ATGAATGATCCTCGACGGGCATGCCCCATGGATTCACTTACAGATGAAAGCCCTGTTGAGACATTGTATGTCTACCGAACGATTGCAGGTCCAAGGTCTG
ACACACCGGCGTGTGTCCAGATTGTTTAGGGGGACCGCCTGTGGTACGGGAAGGTAGTATGGAAGTATTCCTTCACAATCCTGATTTGCTTCAACACCTT
CCTAGCATAACATGGATTGTTTCCTTGAAGTATAAAGGCTGGTGGCTTGGCTGATCCTGTCATTACCTCGAAACTGTTTACGACTTTAGGACGAGCCTCG
GGGCCCTTTTCAGGTGTTAATAACTGTAGGTGGAGACTCGATGAGTTACAAAAACTACTAGATCTACTAGAGAGCGTCTTGAGAAAACTTGGGTTTGTTT
TCTTAGCGTGATTAGTTCTTTTCATGTATAAAAAATATGAGATCATTTCAATCTCGCAATGTACTGTACTATTGGAACAAATTGTACCAGGAGCCTCTAC
TTTTTTGACCTCCCCTCTCAAATTACGGGTTGTTGCCTCAACTTGTTGTGAATGGGGAATAATTCAAGCTTATCTGTGTCTCTTTATTCATGAGGTATTT
ATC
>comp80692_c0_seq28
GCTCCTTGAGAAACACCATCCAATTACAAGACTATTTGATGCAACAATCACGCATATGGCTGCGCTAGAACTACTGCTCTGAATTAAGAGGCGTTGAAAT
ATTGTCCGGTCTACATATAGGCACTTGAGGTGTATTGACAGACAGTTCCCCTTAGTTTGCTACAACACATCAGGCGAATTCCACCAAATTAGGATGATGT
TTGTTTCTTAGACACAGGATAGCAATGCCAAAAGTTTTTTATAATTTTCCGCTTCACCGCAGATTTGGCATCTCAAAGGGAGTTGTGGCAAGATTCTCTT
GACCAAGCTTTCTTGCAGATTTGGCAAGAAAGATAAAAAAAATGTTGGTATACTTAAGCATCCTTAATAGAAACTAAACAGGTCTTAGATTGAAATTATC
ATAAATAAACCCTTTTTCACTTTAGCTGTAGTCCGGATGAAATCTTAGGGGCCTTGCTTAGCCTGCCCTGAAGCTTGCCAGTAAGTCGAAGCTGATGCTG
ACACAGCACTGAAATAGATCCCAATGAACACAAGTTATTTCGAGAAGATAAAATACCAAGCAAAGCCAGGATTGCATTAAAAGATAGTGTATTTCTACTA
TACGATCTGAAAGCTTAATTTATTATATCAATAATCAAATTGGCAGCAGTGTGGAACAGATATAAAGAAGATGCTTCAAATCACAAATAACTACAGATAA
ATTGAAGGGTGAAGGTTCATTCAACCACCAAACACCCAAAATGTCGAGCTGCATATGCACAGAACCTAAGTACGGTACAGTTCATAGTTTGGGAAAATAG
ACTGCAAATGTGCACAGATAATACAGATTGTTACTCGATGAACAGTAAGCACGGTATTTGAAATCCTTCTAACCATCCATCCAGGCAGTGCCTCGTATAC
TCAAACCTCTTTTTGGAGGAAGCCAAACCATGTGATCCTGTTTCAAGTTCTCATCTTCTGAAAATGATCAGCAGTCAATAGATGTTTTAGTCCATTCTCC
AGAAAAATGAGTGCCGCATGAATCCTTTCCGGTTTATAGTGAAACATTTTAGACGATGACCTTTAACATTCTAGAAAACGAAGAGAAAACCTACAGTCAT
CTAATTTCAGCTTAAACATGTTCCAGCAAGATCATCACCATCCTGACATGTTCCGTTTTGTCCCTAGACCCGAGCACAAGGTATTGCGAGGCTAATTTAG
CTCAATTCAGTTGTCAGAGCAATTGTGTGTGAAAAGTGTGAACAACAGCATAGGAGACAATACAGCAAGTGTAGCGTGCCAATATGTGTCCAGCTTGATA
GTTCACTATAATAAAGTTCTTTAATTCAAGTATTTGAATAATATAGAATCTCAACCTTCAAGTTTAAAAGGTTTTGTATTTGCTATTATAATCGTTGCCA
CCCCCTCACCCCTCTCAAAAAGAGAGTTACTTTGTAGCGCACTGTAAATCTCTTGATTGAAATAGTGGCATGTCATCTTATTTAATTCAGTTATTTCATA
AGGTACAATTTGTTTAGCTGACAGCTCAGACATGCTTCTATGCAACATGTTTATACCAATGCTGTATTTTGGCAATCCTGGATGTGGCATTACTTAACTG
TGCTTTCATATTCAATAGTTCTGGCAAAGGGAGGCTGCAAGCTTGAGACAACAACTGCACAACCTGCAAGAAAGTCATCGGTATGGCTAGAAAACCCTGC
AAATTTTACAAATGTACAAGTCAGAAAATTTGCCTAATGTAAAAGGATCAAACTACAGGATTTGTCAGCATGAGCACAAGTGCCTAACTTCCGAGTTAGA
TAACTCTGGAGGAAGGAAATCCTTCCTACTGTTTGTTTCAAGAATATACAAAAATTCAAAACAACATTAGAATAGAACCAAAAGAAGAAACAATATTCAC
CATGTAGCAATACTCAATTCCTGGTTGTCTTATTTATAAAGGTATAAAACTAGGTTCTGACGAGACAATTATGAATCAACTACACCATGTTACAGATCAT
CCATAACTCTTGGTGATGCAAATTAAGGGCTGTAAAGTGACAAGTATACCTCCACCAACTAATTTGTGCAGAGTGTATAAAATTGTAAAACAATGGATAA
GAATGAATTTAGTATACAAGATAGCATACAATGCCCAAGTTTAAGGAGACATACTGATTTACTGACTCCATAGGACAATCTTTTGCTTATGAGGACATCA
GTTTTCATAAACAATATCAATGTAAAAGGAGGTAACAGTTTATGAATTTCCTATGCCAAGCCATTTATAAGTCAAAAAATGTACCTATTTAGCTTTTATG
CATTCCAATTAACTTCCAAAGAAGAAGAGTACATACTAATGGAAAACAAATATGATATTATCATCTGTTTTGCCCGGTAGTTTCTTGAGTCTCTGATTTG
TCCGCACTTCACTCCCAACCAACACAACATTAATACACATGGGCAGAGGAATATCAGGTTATCAGGTAAACTGAATACAGTTTGATTGACTGCAGTCAGT
TGATGGGACAAGATCTTTCTGGATTGAGCGTCAAGGAACTCCAAAATCTAGAAAATCAGCTAGAAATGAGCCTACGTTGCATCCGGACAAAAAAGGTAAG
GATGCTAGCCATATTTCTGTTTTTCGTTACCAGATTGCCTCATCCAAGAATACAACTGATGATGACGTTTTTTACTTTCAGGACCAACTCTTGATTGATG
AAATTCACGAACTGAATCGAAAGGGAAGTCTCATGCAACAAGACAAGATGGAACTATACAAAAAGGTCAACCTAATCCGTCAGGAAAACATTGATTTATA
CAAGAAGCTCTACGAGAAAGAGGTGACAAGTGAAGTCAACCGAGATTCATCAACTCTAAACTTTGCAGTTGTCGAGAACGTCAACATTCCTGTTCATCTT
GAACTTAACACTCCACCGCAAGAAAATGATGTTGAGCAAACTGCACCTCCTAAACTAGGATTACAACTAAATCCATGAAGGCATGCAGCATGGCACTGCT
AATGTTCATTATTTTCAACGGCTAGGATTCAAAGCAGCTAGAATACCATTGGTTGCCTAAACACATAGCAAGACAGACGTGGCATGCTGGGCGACGGTAA
TGGTAGTGTAAATCTATTTTCAAATAAAATGTATTTTATTGAAGACTACAGAAAATTATTTGTCTTATAACTATTTCACGCATTCTGCAAGTTTGTTCAC
CAACTTGAGTACATGACAGCTACAAATCTTATATTGACAAAAGATATGGTGTGCGGAAATAATTCAGGTACTGTTTTTCCTAAAAAAA
>Locus_18892_Transcript_28/56_Confidence_0.279_Length_1689
ATCTGGGATTGTGGGAAGCAAGTCTACTTAGGTGAGTAACTGAGTTGAGTGAGTTCTATACGTTCCAGCTCGAGCTTCCATTAACTGCTGATCAGTTTCC
GTCCGTTTGATCTGGTTTACGATGTGCTGATGCTGTTCTATTCAGGTGGTTTCGACACTGCTCACGCGGCTGCGAGGGCTTATGATCGTGCAGCGATCAA
GTTCCGAGGGCTCGACGCGGACATCAACTTCACTTTGAGCGACTATGAGGATGACTTGAAGCAGATGAGCAATTGGACAAAGGAAGAGTTCGTGCACATA
CTCCGCCGCCAGAGCACGGGGTTCGCGAGGGGGAGCTCCAAGTACCGCGGCGTGACGCTGCACAAGTGCGGCCGCTGGGAGGCGAGGATGGGTCAACTTC

TTGGCAAGAAGTAAGCCAACCTCCTGGCTTTGACCTTTCTACTCTGTTAGCCTAGTTAAGTGTTTTTTACCCTGGTTATATGAACAAAATATGTCGAAAT
GATGTTCAATTAACTTGTCATTTCACGTTGGGTTCGATCAGGGCTATATCCCTTATGTTCATGTTAAGTAGTTTCAGGTGATTGTAGAGAATACAACAAG
GTGATCTTGAAGTATCACTGATTATAATCCATGCTACTAGTAGTTTGAGCGTCTCTGTTGCCGGTGAAATCTCCACCCCTCTTGTGACTTTACCCCTCTA
CCGTTGAATCCGGGAATCCGGAATGGACCTACAAGATCGAGATATGTGCCTTGATGAACAGTGTTTCGGTGAACAGCAAAATCGGCACCCGTGCACAGTG
TCCTGATGGACGCAACCTCTAGAGTTCACATATATGCATTGGCCAACAATGCAATTCCTGCTTTTTAGTCAGGAACCAGTTTTGTCAAAGTCTACGACAC
TAGGCATCCCTCTGTTGAGACATATCTCAGAACTGGAATTTACATGCAGGTACATCTATCTTGGACTCTTTGACAGCGAAGTTGAAGCTGCAAGAGCATA
TGACAGGGCAGCCCTTCGCTTCAATGGGGGGGAAGCTGTTACTAATTTTGGGCCTAGCTCCTACAATGGAGGAGATGCTCTACCCGACACCGAAAATGAG
GCAACTGTTGATGGTGACGCGGTTGATTTGGATTTGCGGATTTCACAACCTAATGTACACGACGCTAAAAGGGACAATACCGTAGCTGGCTTCCAGCTAA
CATGCGACTCCCCTGAATCTTCAAATATGATGGCCTCTCAGCCAATGAGCTTATCGTCCCCGTCGCCTGTCTATCACCTAAGCACAGCAGTACCACCTCA
CCATCAGCGTTTGTACTCATCTGCTTGTCCTGGCTTCTTTCCGAACCTCCAGGAAAGGCCAGTGGAGCGAAGGCCTGATTTGGGCCCCCAGTCGTTCCCC
ACCTGGGGACGGCAAACGCAGGGCTCCCCTCACATGCCGTTGCACCACTCCGCAGCATCATCAGGATTCTCTACCGCCGCGAACGCCGGCGTCCCATTGC
CGTCTCACCCGCCGGCGCCGTTCTCGGACCACCAGTTCTACCTCCCACCGACGGCCTGAGCTTGTTGGTCCGGTTCAACTCTTGGGACAGCAGCTGACGC
TCTGTGTGTGAACGTCTCGCAGCTTGCCGGTAACAGCTGTTAATTAATGGGAGAGAGAGAGAGAGAGAGAGAGAGAGAGAGAAAAGAGCCG
>comp76332_c0_seq35
ACTGCTCTGAATTAAGAGGCGTTGAAATATTGTCCGGTCTACATATAGGCACTTGAGGTGTATTGACAGACAGTTCCCCTTAGTTTGCTACAACACATCA
GGCGAATTCCACCAAATTAGGATGATGTTTGTTTCTTAGACACAGGATAGCAATGCCAAAAGTCTTTTATAATTTTCCGCTTCACCGCAGATTTGGCATC
TCAAAGGGAGTTGTGGCAAGATTCTCTTGACCAAGCTTTCTTGCAGATTTGGCAAGAAAGATAAAAAAAATGTTGGTATACTTAAGCATCCTTAATAGAA
ACTAAACAGGTCTTAGATTGAAATTATCATGAATAAACCCTTTTTCACTTTAGCTGTAGTCCGGATGAAATCTTAGGGGCCTTGCTTAGCCTGCCCTGAA
GCTTGCCAGTAAGTCGAAGCTGATGCTGACACAGCACTGACATAGATCCCAATGAACACAAGTTATTTCGAGAAGATAAAATACCAAGCAAAGCCAGGAT
TGCATTAAAAGATAGTGTATTTCTACTATACGATCTGAAAGCTTAATTTATTATATCAATAATCAAATTGGCAGCAGTGTGGAACAGATATAAAGAAGAT
GCTTCAAATCACAAATAACTACAGATAAATTGAAGGGTGAAGGTTCATTCAACCACCAAACACCCAAAATGTCGAGCTGCATATGCACAGAACCTAAGTA
CGGTACAGTTCATAGTTTGGGAAAATAGACTGCAAATGTGCACAGATAATACAGATTGTTACTCGATGAACAGTAAGCACGGTATTTGAAATCCTTCTAA
CCATCCATCCAGGCAGTGCCTCGTATACTCAAACCTCTTTTTGGAGGAAGCCAAACCATGTGATCCTTTTCAACCCAAATACATATATTCAAGATGAAAC
TAACAAAAAGACAGTTGATAAAGAAACACGAAAGAGAGTAGAAAAAGGGTACCTGTTTCAAGTTCTCATCTTCTGAAAATGATCAGCAGTCAATAGATGT
TTTAGTCCATTCTCCAGAAAAATGAGTGCCGCATGAATCCTTTCCGGTTTATAGTGAAACATTTTAGACGATGACCTTTAACATTCTAGAAAACGAAGAG
AAAACCTACAGTCATCTAATTTCAGCTTAAACATGTTCCAGCAAGATCATCACCATCCTGACATGTTCCGTTTTGTCCCTAGACCCGAGCACAAGGTATT
GCGAGGCTAATTTAGCTCAATTCAGTTGTCAGAGCAATTGTGTGTGAAAAGTGTGAACAACAGCATAGGAGACAATACAGCAAGTGTAGCGTGCCAATAT
GTGTCCAGCTTGATAGTTCACTATAATAAAGTTCTTTAATTCAAGTATTTGAATAATATAGAATCTCAACCTTCAAGTTTAAAAGGTTTTGTATTTGCTA
TTATAATCGTTGCCACCCCCTCACCCCTCTCAAAAAGAGAGTTACTTTGTAGCGCACTGTAAATCTCTTGATTGAAATAGTGGCATGTCATCTTATTTAA
TTCAGTTATTTCATAAGGTACAATTTGTTTAGCTGACAGCTCAGACATGCTTCTATGCAACATGTTTATACCAATGCTGTATTTTGGCAATCCTGGATGT
GGCATTACTTAACTGTGCTTTCATATTCAATAGTTCTGGCAAAGGGAGGCTGCAAGCTTGAGACAACAACTGCACAACCTGCAAGAAAGTCATCGGTATG
GCTAGAAAACCCTGCAAATTTTACAAATGTACAAGTCAGAAAATTTGCCTAATGTAAAAGGATCAAACTACAGGATTTGTCAGCATGAGCACAAGTGCCT
AACTTCCGAGTTAGATAACTCTGGAGGAAGGAAATCCTTCCTACTGTTTGTTTCAAGAATATACAAAAATTCAAAACAACATTAGAATAGAACCAAAAGA
AGAAACAATATTCACCATGTAGCAATACTCAATTCCTGGTTGTCTTATTTATAAAGGTATAAAACTAGGTTCTGACGAGACAATTATGAATCAACTACAC
CATGTTACAGATCATCCATAACTCTTGGTGATGCAAATTAAGGGCTGTAAAGTGACAAGTATACCTCCACCAACTAATTTGTGCAGAGTGTATAAAATTG
TAAAACAATGGATAAGAATGAATTTAGTATACAAGATAGCATACAATGCCCAAGTTTAAGGAGACATACTGATTTACTGACTCCATAGGACAATCTTTTG
CTTATGAGGACATCAGTTTTCATAAACAATATCAATGTAAAAGGAGGTAACAGTTTATGAATTTCCTATGCCAAGCCATTTATAAGTCAAAAAATGTACC
TATTTAGCTTTTATGCATTCCAATTAACTTCCAAAGAAGAAGAGTACATACTAATGGAAAACAAATATGATATTATCATCTGTTTTGCCCGGTAGTTTCT
TGAGTCTCTGATTTGTCCGCACTTCACTCCCAACCAACACAACATTAATACACATGGGCAGAGGAATATCAGGTTATCAGGTAAACTGAATACAGTTTGA
TTGACTGCAGTCAGTTGATGGGACAAGATCTTTCTGGATTGAGCGTCAAGGAACTCCAAAATCTAGAAAATCAGCTAGAAATGAGCCTACGTTGCATCCG
GACAAAAAAGGTAAGGATGCTAGCCATATTTCTGTTTTTCGTTACCAGATTGCCTCATCCAAGAATACAACTGATGATGACGTTTTTTACTTTCAGGACC
AACTCTTGATTGATGAAATTCACGAACTGAATCGAAAGGTTCTGGAATATTCTGTTATTACTCTGCAATGAGAGGTCTCTGAGTGGATTTCAAGAAGCTG
ACTCTACTGGCTCATTTTCAGGGAAGTCTCATGCAACAAGACAAGATGGAACTATACAAAAAGGTCAACCTAATCCGTCAGGAAAACATTGATTTATACA
AGAAGCTCTACGAGAAAGAGGTGACAAGTGAAGTCAACCGAGATTCATCAACTCTAAACTTTGCAGTTGTCGAGAACGTCAACATTCCTGTTCATCTTGA
ACTTAACACTCCACCGCAAGAAAATGATGTTGAGCAAACTGCACCTCCTAAACTGGGGTAACCCTGCTTTTTTCTGCAAAGGAACTATTGATTCTGTTCT
TAATTCTTTAGTGGCATATTGTTTACTACTAACTGAAAACTTGCAGATTACAACTAAATCCATGAAGGCATGCAGCATGGCACCGCTTATGTTCATTATT
CTCAACGGTACGATTCAAAGCAACCAGAAACACTATTGGTTGTAAAGAGCTTAAACACATAGCAAGACTGACGCGGTATGCATGCCATGAGTGATTGATA
ATGGTAGTGTAAATCTATTTCAAATAAAATGTATTTTATTGAAGAATACAGCAAATGATTTGTATTATAACTATTTGACACATTCTGCAAGTTTGTTGAC
CAACGTGAGTACATGATAGCTACAAATCTTATATTGACAAAAAGATATGGTGTGTGGAAAAAATTCAGGTGCTATTTTTTTCTATTTTGAGAATTGTATG
CATAGCAGAAGAGCACAAATTGCATTCTGGTTTATCCGTGCAGGCATTCCAGAGTATCAGAATGGGGATCAACCTTTCTTCAGCTACATATGTAAATATA
CGGATGCTAGTTGCTCACTTAAAATTTTGTAGTGTTCAAAGGAATAACAACAGTAAGACGTTGACGCACCGAAAATAACCAGAAATAACGGATTGTACTA
TGACAAGACACACGACAGATACACTAGCTTAAGTTTGTACAGGAAAATATTTGGCCTATTCTTATGTCACAGTAAATTAAGATTATCACCTTAATCACAG
AAATTCAAGGGGTAAACGATTATAGCACAAAACGATCCTGGACCCCAAGACCTGGGTCTCTGAACGGCTGAACCTCCAATTATACCTCTATACATTTGGA
ATGTGTGTAGTCAAACTTTCGCTACTGGTATCTTTACAAATATTTACATTGAACACATCAAAATGATATACATGGATTCCTCTCGATATGTCTTTGAGGT
TTGGGTTCTAAAAATGATATACAAATTATTAGGTTCTAAACTTAAATGATATATACACATCAAAATGATATACAAATTATTGGGTTCTAAACTTAAATGA
TGTATACACATCG
>comp80692_c0_seq20
GCTCCTTGAGAAACACCATCCAATTACAAGACTATTTGATGCAACAATCACGCATATGGCTGCGCTAGAACTACTGCTCTGAATTAAGAGGCGTTGAAAT
ATTGTCCGGTCTACATATAGGCACTTGAGGTGTATTGACAGACAGTTCCCCTTAGTTTGCTACAACACATCAGGCGAATTCCACCAAATTAGGATGATGT
TTGTTTCTTAGACACAGGATAGCAATGCCAAAAGTTTTTTATAATTTTCCGCTTCACCGCAGATTTGGCATCTCAAAGGGAGTTGTGGCAAGATTCTCTT
GACCAAGCTTTCTTGCAGATTTGGCAAGAAAGATAAAAAAAATGTTGGTATACTTAAGCATCCTTAATAGAAACTAAACAGGTCTTAGATTGAAATTATC
ATAAATAAACCCTTTTTCACTTTAGCTGTAGTCCGGATGAAATCTTAGGGGCCTTGCTTAGCCTGCCCTGAAGCTTGCCAGTAAGTCGAAGCTGATGCTG
ACACAGCACTGAAATAGATCCCAATGAACACAAGTTATTTCGAGAAGATAAAATACCAAGCAAAGCCAGGATTGCATTAAAAGATAGTGTATTTCTACTA
TACGATCTGAAAGCTTAATTTATTATATCAATAATCAAATTGGCAGCAGTGTGGAACAGATATAAAGAAGATGCTTCAAATCACAAATAACTACAGATAA
ATTGAAGGGTGAAGGTTCATTCAACCACCAAACACCCAAAATGTCGAGCTGCATATGCACAGAACCTAAGTACGGTACAGTTCATAGTTTGGGAAAATAG
ACTGCAAATGTGCACAGATAATACAGATTGTTACTCGATGAACAGTAAGCACGGTATTTGAAATCCTTCTAACCATCCATCCAGGCAGTGCCTCGTATAC
TCAAACCTCTTTTTGGAGGAAGCCAAACCATGTGATCCTTTTCAACCCAAATACATATATTCAAGATGAAACTAACAAAAAGACAGTTGATAAAGAAACA
CGAAAGAGAGTAGAAAAAGGGTACCTGTTTCAAGTTCTCATCTTCTGAAAATGATCAGCAGTCAATAGATGTTTTAGTCCATTCTCCAGAAAAATGAGTG
CCGCATGAATCCTTTCCGGTTTATAGTGAAACATTTTAGACGATGACCTTTAACATTCTAGAAAACGAAGAGAAAACCTACAGTCATCTAATTTCAGCTT
AAACATGTTCCAGCAAGATCATCACCATCCTGACATGTTCCGTTTTGTCCCTAGACCCGAGCACAAGGTATTGCGAGGCTAATTTAGCTCAATTCAGTTG
TCAGAGCAATTGTGTGTGAAAAGTGTGAACAACAGCATAGGAGACAATACAGCAAGTGTAGCGTGCCAATATGTGTCCAGCTTGATAGTTCACTATAATA
AAGTTCTTTAATTCAAGTATTTGAATAATATAGAATCTCAACCTTCAAGTTTAAAAGGTTTTGTATTTGCTATTATAATCGTTGCCACCCCCTCACCCCT
CTCAAAAAGAGAGTTACTTTGTAGCGCACTGTAAATCTCTTGATTGAAATAGTGGCATGTCATCTTATTTAATTCAGTTATTTCATAAGGTACAATTTGT
TTAGCTGACAGCTCAGACATGCTTCTATGCAACATGTTTATACCAATGCTGTATTTTGGCAATCCTGGATGTGGCATTACTTAACTGTGCTTTCATATTC
AATAGTTCTGGCAAAGGGAGGCTGCAAGCTTGAGACAACAACTGCACAACCTGCAAGAAAGTCATCGGTATGGCTAGAAAACCCTGCAAATTTTACAAAT
GTACAAGTCAGAAAATTTGCCTAATGTAAAAGGATCAAACTACAGGATTTGTCAGCATGAGCACAAGTGCCTAACTTCCGAGTTAGATAACTCTGGAGGA
AGGAAATCCTTCCTACTGTTTGTTTCAAGAATATACAAAAATTCAAAACAACATTAGAATAGAACCAAAAGAAGAAACAATATTCACCATGTAGCAATAC
TCAATTCCTGGTTGTCTTATTTATAAAGGTATAAAACTAGGTTCTGACGAGACAATTATGAATCAACTACACCATGTTACAGATCATCCATAACTCTTGG

TGATGCAAATTAAGGGCTGTAAAGTGACAAGTATACCTCCACCAACTAATTTGTGCAGAGTGTATAAAATTGTAAAACAATGGATAAGAATGAATTTAGT
ATACAAGATAGCATACAATGCCCAAGTTTAAGGAGACATACTGATTTACTGACTCCATAGGACAATCTTTTGCTTATGAGGACATCAGTTTTCATAAACA
ATATCAATGTAAAAGGAGGTAACAGTTTATGAATTTCCTATGCCAAGCCATTTATAAGTCAAAAAATGTACCTATTTAGCTTTTATGCATTCCAATTAAC
TTCCAAAGAAGAAGAGTACATACTAATGGAAAACAAATATGATATTATCATCTGTTTTGCCCGGTAGTTTCTTGAGTCTCTGATTTGTCCGCACTTCACT
CCCAACCAACACAACATTAATACACATGGGCAGAGGAATATCAGGTTATCAGGTAAACTGAATACAGTTTGATTGACTGCAGTCAGTTGATGGGACAAGA
TCTTTCTGGATTGAGCGTCAAGGAACTCCAAAATCTAGAAAATCAGCTAGAAATGAGCCTACGTTGCATCCGGACAAAAAAGGTAAGGATGCTAGCCATA
TTTCTGTTTTTCGTTACCAGATTGCCTCATCCAAGAATACAACTGATGATGACGTTTTTTACTTTCAGGACCAACTCTTGATTGATGAAATTCACGAACT
GAATCGAAAGGTTCTGGAATATTCTGTTATTACTCTGCAATGAGAGGTCTCTGAGTGGATTTCAAGAAGCTGACTCTACTGGCTCATTTTCAGGGAAGTC
TCATGCAACAAGACAAGATGGAACTATACAAAAAGGTCAACCTAATCCGTCAGGAAAACATTGATTTATACAAGAAGCTCTACGAGAAAGAGGTGACAAG
TGAAGTCAACCGAGATTCATCAACTCTAAACTTTGCAGTTGTCGAGAACGTCAACATTCCTGTTCATCTTGAACTTAACACTCCACCGCAAGAAAATGAT
GTTGAGCAAACTGCACCTCCTAAACTAGGGTAACCCTGCTTTTTTCTGCAAAGGAACTATTGATTCTGTTCTTAATTCTTTAGTGGCATATTGTTTACTA
CTAACTGAAAACTTGCAGATTACAACTAAATCCATGAAGGCATGCAGCATGGCACCGCTTATGTTCATTATTCTCAACGGTACGATTCAAAGCAACCAGA
AACACTATTGGTTGTAAAGAGCTTAAACACATAGCAAGACTGACGCGGTATGCATGCCATGAGTGATTGATAATGGTAGTGTAAATCTATTTCAAATAAA
ATGTATTTTATTGAAGAATACAGCAAATGATTTGTATTATAACTATTTGACACATTCTGCAAGTTTGTTGACCAACGTGAGTACATGATAGCTACAAATC
TTATATTGACAAAAAGATATGGTGTGTGGAAAAAATTCAGGTGCTATTTTTTTCTATTTTGAGAATTGTATGCATAGCAGAAGAGCACAAATTGCATTCT
GGTTTATCCGTGCAGGCATTCCAGAGTATCAGAATGGGGATCAACCTTTCTTCAGCTACATATGTAAATATACGGATGCTAGTTGCTCACTTAAAATTTT
GTAGTGTTCAAAGGAATAACAACAGTAAGACGTTGACGCACCGAAAATAACCAGAAATAACGGATTGTACTATGACAAGACACACGACAGATACACTAGC
TTAAGTTTGTACAGGAAAATATTTGGCCTATTCTTATGTCACAGTAAATTAAGATTATCACCTTAATCACAGAAATTCAAGGGGTAAACGATTATAGCAC
AAAACGATCCTGGACCCCAAGACCTGGGTCTCTGAACGGCTGAACCTCCAATTATACCTCTATACATTTGGAATGTGTGTAGTCAAACTTTCGCTACTGG
TATCTTTACAAATATTTACATTGAACACATCAAAATGATATACATGGATTCCTCTCGATATGTCTTTGAGGTTTGGGTTCTAAAAATGATATACAAATTA
TTAGGTTCTAAACTTAAATGATATATACACATCAAAATGATATACAAATTATTGGGTTCTAAACTTAAATGATGTATACACATCG
>Locus_14087_Transcript_58/66_Confidence_0.284_Length_2834
GATTCAAAATTGTAGCAGATTTGTCCTCTACCCACCCCTCTGAATCCTTTTTATACATACACTGACCATGGCACCAATGATCAAACCAAACACCAGCTGC
AAAGATCACCCTGGATTCCTGGAACAAAGAGGGCCTAGAGCAACGCAGCAACCTTCGCCACTTGAAGAGGATAGGTACAATGTGGCTTAGCTAGCACCTT
GCAGTCAGGTAGAATTATTCAACCGGATAAAGATTATTTCAAGTAAAGGAGGGCTACCAGTTTATACCTGTTCAGGTGGGTGCAGCAGTTGAGAACTTTT
TTTCGATACAGAGCAGTTGAGATCTTTAATTCTGGCATGGGTTCCTTTGGGGTGAACTGGAATCAGAAGAACTCCGCGGTGTGCGATTGGGAAAATTTAG
CACCTTCTGTTCCAAATGCAATCGGAAACCCCAGGAACGGAGCTACGAGGCATGGATCTGTGAATTCATCTGGTGGCACTCTCACTTCTAGCTCAGAGCT
AGGGCATGGTTCATCCAAGAGCTCCATGTCGGCATCCATTGATTCACCGTCCAAAGTAGGAAACAGCTTGGAGTTCAACTTTGCTGCTGTCAAAGGCCAT
GTTAAGAACATGGATAAGGATTTTAGATTTGACGACTTGGGGACTTCTCCATCGTCGATGATAGCATTCAGCCATGGAGAGCCATTAATCAGCCTTAAGC
TTGGAAAAAGGACTTACTTTGAAAATGTCTGCGGAGGGCAGGATTCCAAGAGCACTGCACCTTCTACCAGGACTTCTCCATCAACCGTTCTCAAGAAGAC
CAAGGTGTCTCAGCAGAATACACAAATGTCATACTGTCAGGTTGAAGGATGCGGAATTGATCTGTCCTCCGCTAAAGATTACCACCGCAAGCACAAAGTC
TGTGAAGCTCATTCTAAAGCCCCGAAGGTTGTTGTTGCTGGTGTAGAGCGCCGTTTTTGCCAGCAGTGTAGCCGGTTTCATGGCTTAGCCGAGTTTGACC
AGAACAAACGAAGTTGCCGTAGCGTCTTACTCATCATAATGCACGGAGGAGAAAACCACAGGCAGATACAATTTCATTCAGTTCATCGCGGCTCTCGACA
ATGTTTTATGATACAAACCAGCAGAAAAATCTTTTCTGTAGTCAACCTCATTTTGGCCAAGCGAGAAGCAATGCAGTTTTTTCTTGGGAAAACTCGGGAG
ATTCCAAATTTATGGAAACCAAACATCTGTTGATGCAGCCAACGAAAATGTTAGGTCTTGATGGGCTGCATTTCTCAACCCCCCAGATATCAAATAGTGT
TGTGGCTCACTCTGTACATCATCGTAATTTCGATGGGCTCATGCCATTCAAGGGAACCAACACAAAGGTCCTCAACCAAGGCACTATGCTAATCTACTGA
TCAATTACTGAACTTAATTTTAAAAAAAACCTCTGGCAGCATTATCGTAACAGTTATCTACACTACTGTTGTAGTTCTTAGGAGTTTCACCTCTCACGCT
ATTGAGCTAGCATTTTGTTCTTCATGGTTCCAATTTGCACAACAATTATGTTACGCATTTTATGAATTTGATGATTTGTTATTGGTACTCAAGTTCTTGT
TCATGATATTTTTTTTAACATGTCTTCTGGGCTATGAAAGCCCAAAAATTTGCTGGCCCCTTTGCATGTTCCTGTGCACTTCACGCTATTCTGAGTCTAC
CATCTGGAGAAAGAAAATGAACACCTATCATCTCCTGCAAGATTATATACTTGTTGAATCATGTGTAAAATTGCACTGTGGCATAGTAATATAGGAGTTA
TAATATTTCAATCTGAACCCTTCCAGATTCTGTCTGGAGCCTTTCACCATCAGAAAAGGCACGAAAGTGCAAATGCCAAACAGCTTGGGCCCCTGGACTG
GGTTTATGGTCAGAGGCTCAAAGCACTGCACTGCTAATGCGCTTGCCAGAAATTCTGGCTGGCAGCCCGGTACCACCTGATCCTAGTATTTAGGTTCCAG
AAAAGAGATTGATTGAATATGCCAGCTTAATGTTGCCTGAGTGTGACTACTGATAGATAGTCGCATGAATGCTCTTACCTTACAACCTTTGGAACTAAAA
ATATTGCTCTGTTGAGCAGTCCGATCGTTCAGATTTCTAATCTAAGGATGAAGCTCAGCATAACAATTCTATGCTACTATTTCAGGTGTCGAAGCTTCTG
CGATCGCTTCCAACTCGAGTGGAGACCCGGATCTTGGGTGTGCTCTCTCTCTTCTGTCAAACGGTTCGTGGGGTTCAACTTCAGCCGTCATCCAACAGCC
TAGTTCTCATGCGCAAGCTGGTGCGGTGCCACCCTTCGCCACCGTCGCCGTCTCCAACCCTGCAATGCATCCTCTGGACTCATCCCCGGGAGGATTCTGG
CAAGACGTCCCTCCCCCGCTCGATGAAACTCCACATATTCAGGCATTCACGCACCTCTGATCAAATGCTGCATCCCCTGGGAACAGCAGGTGGTTATACC
TTCCGTAATCGTCCTAGGTTTGCTTTGCCAGTGACTGGCTCTGCACAATGTCGATCCATCACCGGAGGCATTTGGTAAGGTGAAGACTAAAGACTCAAGA
ATAACCGGAGACTAACTCGCTTCTCTTCCAGGCTCAAGACTGTTTGGGTCCAGCGGTGTTGCTACCTGATCCTGTTTTCGGTTCTGAGTACATCCATTAC
TAATCATGGGATTACTTGTTTAGGCCAACCAGACATATTCTGGGGTGAGATTACGACATGTTAATTTACATTGACGGTGTTTCTACATGTGTTAGTACTA
ATGATGTTATCGCTCTTGGCCCCGTTACGTTTCA
>Locus_4522_Transcript_42/58_Confidence_0.383_Length_11225
GGGGGGGGGGGGGGGGACAGGTAAGTCAGGGTCAGCCAAAACAAACTTGCACGTCTTCTTCATGCTCTTGAGCCAGGTCATCCAGGAGTAGTGCGTACCG
GGGGTGTTCAGTGTATATCATGCTAGATGCTACAGCCGGACTACTACAACACTGCACACTGCTTCAGTATTCCACATTTGTTGATGTCTGGAATACATGG
GGAATGGCTGTAAAGTGATTGTAAATGAGCAGGCCACAGGGCTGCAGATGATGCTCATTTACTCTGCTAGTCTTAGCCATTCATGCCACAGCATGGAGCC
TCTTCTTTGGAGTTTGGACCATAAAAGGTAGCACATTGACTGGCACTTGCGAGGCTGGATCGAATTCAAACCCTCCTACAGCTAATGCTTAAACAGTTAA
CCTCGGCCTTGCTTTGAGACAAGGAAAAATAATAGGCATAATCAACCATCTTAAACCTGGTTCACTATCATCCATAGCTACTAACAGACGTGGATTTCCT
TGACATGCTATTGTAATCCTGCTATACCACGGACCGTCTTCTGTACTGTCAAGCTCTGACCATTCACATTGAAGAAAGTCACATCTTTGAATCATATCCA
AGGTGTCCACCCCTCATACATATTACATAAGTTGTCAATGCTTGTTGCTTGTTTAAGAAGATGGTCAACTTGCTCGGAAATGTTCAAGGACCTGGTGCCA
TCTATATCCCATCCATGTAACTTGTGCTCCACTTGCTTCAGGATGGACAGAGCAAAAGGATTTTTACCTCTAGTCACTCGAGTCACAGAATCTGCATAGG
ATTTAGCATCACTGTTGTCACATTCTTGTCCCCTAACCTGTTTAAAGGCAGCACTGTTATCCTCACTTTCAGTTCTTCCCTCCACCAAATGGGTTTCAAT
CTCATGAATACTGCTAGCATTTGTCAGAGCAACATTGTTTAATATTGACTCGTTATTGCTATTGTCTGTGGTTTCCTGACCGTCAGCATGAGGACTGTCA
TCCATAAGGGCATCTATTTTATCTAAGTTGTCAGAAGAACTAATCTGGGTTAACCCTGTGGTGCATCCAGAACTGCTAGTATATGAGTGCGCTGGAGGAG
AAATCCAACATCCATCTTGCAGAGAAACATCATTGTTCATTACTAAATCCTGAGAGCCACCTTCCCGGCTTCCAGAAGAGCCTATATCCTTCTCAATTGA
TGAATCACTTTGGAAGAGATCTGGATGTGTTGAGGGTATATCCTGGGATCTCACAGTTTCCCTTTCTCCGAGAACTTCTAGAGCCTTATGAAGATTCCCT
GCATGAAGACTTGAAAGGCGTCCAAGCTTCATGACCATGGAATGCAGTTCCTTCAAATGCTCAGTAAGTGGACCCACCAGAGGTTCTAAATTTTTGCAAG
CTTCCCTTACTTTAATGTTATAAGATTGATATAATGCCTTTCCGTGAACAAGAGGCATACTGACATTGTTCTTCTCTCTTTCTTTTGAAATCGCCTCAGA
CATTACAGTTACATCAGCAGATAGGCAGGCTTCAAGTGGAAGTAAAATTGTATTTGCCTTGGAAAGATTGATCATCAGGGAACTGTGCACTGTAGCAGCT
TCCTTTCCCAGAGTGTTGACATCATGCAATCCATCTGCTATAGCCAAAACCTCCTCGAGTAAGGAACCACATTCAGAAGTCAAAGCAGTGCGATCTTGTG
ATACATGTTTGAATCCAGATAACAATGCACTTACTTCACAAGCACGCTCATACATGGCTACAAGAGTATCATGTAGATTAACATAAGTAGATTTGGCTTT
CACAGAGACTAGAGAAACCTGGTTGGTTGCTGAAAACAAACCCTTTCCAGCTGTTTCCAGGTTGAGCTGGCCACGTTTCCACTCTTGTTCTGCACATGTA
AAAGAATGATATGCTGTGTCTAGACGTGTCAGGAGATTCAAAAAAGCTTGCTGCCAGGCCCTGCCTTTGTCAATAGATTGTTCGGAAGTCTTATTCTCAG
GAATGAAGTAAAGTCCATCCCTAGATGCTTCAAACTCCACAACAGATGTGCAAAACTTAACAAGATCAGATGCTTCTTCTTGAACCACCCGAAGCAGCTT
CCTCCTTCTCAACAGGTGATCATGAAATTCATGAGGAATTCCTGAACCCTTTGCAGTTGAACCGACTGCTCCAGAAGTATTTGGACCAGCACAAGCCCAT
CCCATTGCTCTCTCAAGTTGTCCTTCAGCTGAAACAGAAATGCTTTCACAAGCTTGCAGGCATTCAATTGACCTTGTTATCAATGACACTGCAGACTGCA
TCTTATCTAACAAGTTGCTTCTGTTCAGATTTAAAATCACAGAGATGAACTTATCATCACCAAGAATATTTTGTGACAAAACTTTCACCTTCTGCACATG
AGCCGATGAAAGATCATTTAGCCATTCAACCTGAACTATTTCCAAAGTGATTTTGCATAGAGCCTCTGTAAGCTCACTTAATTGCCTTTTCATTAGAGAA
ACTGCAGTTCGGGCAGCTCTAGCTGTCTCATGAGCATTACAGTACTCCTCAAGCATTAATGCAACCCTTCCAACAGCAGCAGAATCTCTTTTAGGTTGAT

CTGATTCTCTTCTTCCTGGACTCAACTGCAGCAAATCACTAATTTCTTCGTCTAAACTTAGGATGAGTGCAGGAGCAATCCTTCCTGTAATATACCGAAA
AACTTGCTTTTCCACATGGATTGCGAGCTTCTTTTTGAGAGCGATATAAAGCTGATTGGTAGTGACACTGTGCTCAACAGAAGATGAAATCTCATGCATG
CATAAATCAAGAATTGAATCGAGAAAACTGAGTTCCCAAACAAAAAATGCATGATCCTTCAATGTGCCAGCTAAAGGCCACACCACATCGGATAGTGAAG
AACCTAAAGGCAAAACATCTTTCAAATTTGAAATGGGTCTATCTGTGTGAGAAGGAAACACTCCACACGATGATAGCATACGATCAAGCGATTCCAGGTC
AGCGAAAGGTTTTGCCAAGATTGAAAGTAGAGCCTTGCTTCGTCTCAAATGAAATTCGCTTTCCTGATCAGGATCCAACAAAGATAAGAAGTACTGCTTA
GAAGAAAGTAATGAATTCATGACACTGGCTTCTACTTTCATAAGTGATGAAATCCGGGTATCTTTCTGGCCCCATGTTTGATGGAGTTGTTCCAATTGTG
CCCTGCAGACTTCCTCCTGCGAAGCTAGCTCCTCTGCTTCTTCCCAAGATAGGTGTTCTCTACCTCTCGCAGCAGCTTGTTCAAGAGCTATTTGCTGCTC
AGTAATTCGACCAACTTTCACATCGTAGCTCTCTTCCAGTTCAGTAAGAGATAATCTCTCAAGTTCTACCTCGACCAGCTTCTCTAAAGCTGTATCAACA
GAACCACGTATCTGAGAAATCGAACCAAAGGCTTCCATTACCTCTGAATTGTATGAAACAAATGATCTAATAAATTCTGGAAGAAAAGCTTCAGTCATCT
GTTCAATCATATTGATACTTGAGCGCAAAACAGCCTGAAAAGTAGAAGCCCAGTGCCTAGGTCGGGGCTTTGCATAAGCAGCACTCGTTTCAGCTAATTT
TTTCTCAATCATTTGATGCACTTCATCAACAACTCCCGAAACTAGTGCACATTTCTCTATCTGCTGTTCTAACAATTGCAAGCCCATTCCAGCGTCAACT
TGAAGACCAGAATCATCTGTCCTAAATATAGCTTTTCCAATAGCTTTACTTTGAGAGGTCAGACACTTTGGCTCTAACATCACTATATAGCTGCCCCACA
GCAATCCCAAGAATAAGACCTTCCGTGTTGTCTCCTGTATGTCATCCTTGGCTGGAGTTTTGATCTCACCCTGTATAGGACTTCCTGAATGCGATAAATG
AACATTATCCCCATCCTTTTGTGATGACAACTGAACAGAATTCATGAATGCAGAAAGTATCCGCTCTTTGGATTGCAGTTCATTATCCAACCCAGTAGAA
TTCATCAGTTCAGAACATTCCCTTGCAAGTCTTTCCACACATGCAACATAACTTTCCATTTGACTTAAGAGATCCCGGTATCTTTGCTGCACTAGATTAA
TGCCTTCACCTTGAACCTTAGCAATGACTTCTGCAGCCTGTCTTTTGGCAAGGGGAAGCATGTCTTGTGACGCACTGCTTACAGATAGCTGCAAAACTTG
CGCCCAACTAATAATTGGACTCGTTGTGATGTAGTTAACTGGAAGAACTTGCTGCAAGACTAAAGCATATTCACCAAGTGATACTAAAGCAGAGGAAAGC
CCACCATGCAACTCAGATATTAATTGAGAAATCTCCCTATCCAACTCATGGCACTGTGCCCTCGTTGGCTCAGGTACAACTGTAAGTGGGACTCCCGACA
CAAGGACAGATGAAATAAGGCTTAAGGCCTCGTCCTTACAGTTCAATTGCATGCATGACTCAACGCCCATAATAGAATTGTCCCGTATGGCCTCAAGAAC
CCGCCCATGTTTTTCCATCCATATTGCAAGCTTGTTAGCTTCATCAACTGCTGCAGATTTTGCTTCTGCTAACTCGTGAGCATGAATTTCAAATGAAGTC
CGAGATTTCTCAGCAACTGAAGTAGCATCAGCTAGGATGGACTTAGCTGATGTTTCATTCTGCAAAGCACTTGATCTCTCTTTCTCAGCATGATAAAACA
TTGCAGATGCAACCTCATAGTGATTTAGAATATCCAAAAATTTCTTTAGAGCAGATAGTGTAGCTGGCAAGTTAGTTACAAAAAGATCCTGATGCTCCTG
CAAAGGAACACGAATTTCTTGGATTCTAGAAGAGAACAAGCTCAAACTAACTGCTAATTCCATTCCTTTCTTCTCTTCACCAGCTATTCCTGCTTCATCT
TGAATATTTCCACGTGTCCACTCGATCAATGGGTCCCAAACAAATACTTCCAACAACATCAAGATGACATCTTTGTTCTTCAAGAGGACACCCATAACTT
CCTCACAAGTAACTCTGAAAATGCCTTCCACTCCAGTCAATCCTAAAGCTGATTCCATAATTTGAGTGAGACGGAATGGAACAATTTCTGGAATTTTCAG
TCTTTTTCCTTTATCAAAGCATATATTGTAATCTATGTGAACTACATCACCATTGTTGAAATCCATAAGAATGTTGTCTAAATGTCTATCTCCAAGGCCC
AACATATGGCCAACCATGCTCATGGCTGCTACACTGCTAGAGAATCTCTTAACTTTGGAGTTAAAGTTTTTGAACCCTTCACTTGCACACCACATTTCTT
GCCACAGAATCTCTTTCGGGGTCTCCTTCATAAGCTCCAATAGAACTTTTCTTTTAACTTCTAGTGGCCAATCTCTCCGTGAAACTACCCTTTTGATCCC
TTTTTCTTTCAGTGCTGGTATAATCTTTCCATAGAACATATCACTTGGACGTGGAACAGGTGGAACAGGGTTGTGATAGTTACCGGAGCTGACTGAAGAC
AGCTGTGTCTCAGCCTGTGCCTGAGCCAACTGTGAACGCTTTTGCCATGACTTGTACACATTGTATATGCTGCTTACATTTTCCACCCACTGGATCAGAC
CTGCTCGTCCACTAACCGGTGTAACAGAATAAAACCGTAGCGCAATATTCCGACTTCGAGTGTCAGAAGATGAGTATAACAAGCTATTTATCGCCTCCAA
CAGCTGCATGATCCGAGAATCAAGACGTAAATCCTCCCGGCCCTTGAGAAGATAAGTATACCTCTGACCATCTGATCCTTGCAAGACGAGCTTCTTAGGT
CTTGTCTTTGTTGAAAGTATTGTTACCTCTTTGCAGAATGAAGATACTGTTACAGTGCCATGGTTTCCAGCAAAAGATTCTGAGGAATCAAGTATCTGCT
TCTCAAAACCAGGCATTGGAATGTCAGAGGTCGACAAGACTGCTAGCTGCGGAGCAATTTCACTTAACAATACACATGACTTTCTTTGATGAGAGGCTAA
AGAAGCAGCAATTGAATCAAAAGGCTGCCAAATCTCTCCTAATGCTGCTGGGGATCCAGGGGGGGTCTTAAGGGTTGCGATTGCAGATTTCAGCTGAGCA
TTATATTCCTTATGGAACCACATTTCATGAGATGTTTTTGGTTCGCGAGATGTAGATGCTAGACGACGTTCCAAGGCTACAACAATCGGAGTCATCATAG
CAGAATATTTTGCAGCATTGATCTTGTTCTTTTCAGCTAAACTCAGGGTAGAATTTGCAGCAACCCTTGCAGCTTCCTCTTTAAGTATGTTGATTCTCCT
TAAAACATCTGAACGGAGATCTTGTAAAGTGCTCAGCCATTGTTCCTCCCAAAGAACAGTAATCATTCCTAGCTCCTCAATGGCAAGCTTAACATCCTTA
ATTAAATTGGGGTATAGCTTAACCAGAAAGTCCGATACGCGCTGAAGCTCCTCAGAGGGTTCTCCTTCACATGCATTGATATCAACTAATGTAGGATATA
CAATAGAACAAGGAGAGAGTTTTCCTAACTTCAGCAATATGCTCTCCAACAGTCCCCTTACTATCTTCTCCGGGTGGGAACTCAAGCGAGCAAAAAGTTG
TGGTATAATCTCCTGCCACGGCAATAAAGGAACCGTTGAAAGCCCACGTTCAAGTGTTTCTTTCAGTTCCACACCATAGTTTAACATTATGTGGAGGAGA
TACAGCAATGCCCGCAGAGTGTAGCTCCTTGTTTTCCCTTTCAAAGCATCACGGTGATACGAAGGTTGAAGCTCAGTTGATGAATGAGATAGGTACTGAA
AATAAGCATGTGCGGCATGCCCGAAGAGTGATACCCTCCTTTGCCTTAAAGACCACAAAATTTCAATGAGCTTGTCAATCAAAGGAGTAATGTCCATTCC
AGAATCACATTTACAAAGAAGGACTGTCAGCTTTGATGTTAACACTGCACAGGGGTCTTCACCTTCACAGGACTCAGAGCCTGGTGCTCCAGCTGCAGTT
TCTATCAGATGAACTGCCTGTTCAATTAGTGAAGTTATGGGGTACTCCAGTGCAGAATCATAACATCCTGCTGTCACTGGATAATCATGGCCTAACTGAT
TTGCATGTTCATCAGCACAGATACTTCTAATTATTTCCTCCACCTCAAACTTCTCGTTGTCTGTCAAGTGATATCTATCTGGGGGAAGTTCAGACTGAAG
AACAGGGGACAGTGAATTCTGCAAGTTTGAATCAATTCCAGACAAAGAGTAATTGGCATGAGCGAAGCACCAGGATGCGTAAGCAAGCCAAGCCTTACCT
ATACTGGGACAAAGTTGACAAGATATCTTCCGAGCAGTGCCTATGATTTCTTGGGCAAGCGAATGACAATTCGAAGTTGAAACTGACAAACTATCACCAG
ATAAAGGTTTTTCTTCCCCACTTTGAAAGCCATCCGAATCATTGAAATCTTTTATTACCTTTGGAATGATAATATTTAATATAGGAGTTGAGTTCTCTTG
CTCCATCCAGGTTGAGAGTTTCAAACAAGCTTTAGCAATCAGTGGAAGTGAAGTGTCAATGTCAGATGAACTACTAGCTGTAGACAGAAAGCTAGCACGA
ACTAGTGACCATAGATCAGACAAAGCCTCTTCATTATTTCCTTCTTCATACTTCAGCAAGGCGCCTTCATACTTAATATTCAATTCAAGTATTTCCTTGT
CCGTCTCATCAGATGAATTCAAAGGATTATTTGTGAGGTACTGGTTTAAGCGGGTAGCCAACATAAAATTGCTCTGCTTTCTAGCAAGACTAGCAAGCTC
CTGACATAGAAGAAGAGTGGATAAAGAAGATGGCTGTGTATTACGGTACACTTTGAAGACCTTAAGCCACATGCTGCAATCTTGATGTATCCTATCGATG
GGATCATGCAATACTTTTAATAAAGAATCCATCATCGATTGTGATTGATTAGGTCCATTTCGGCAGATCAGCCCACAAGCTTCCTCAAATGCAAAGATGC
AATGAAGCTGGCCAGCACAAGCGGCAGCTTCAGTCAATCCACTGAGTGGGACAACAGAGAGTGCTTCATCCAACATCAATTTAGCCTTATCTAGTTCCTC
CCTCACTCCGTCCGACTTGCTATTAGATTGAAGCATTGCACGCAAGAGCATAAGTTCACTTCGCTCAACTGCGACCTTGGGATCAAGGGTGAGCTCACTG
CTGCTTTTTGGAGTCAAGTCAAGATAACCCCAAGCTGAATGAAAGTCCCCCTCATCAAAGCATGCCATCGCATGAATAGCATTAAGTTCATTGCCAGCAC
TCGTTAGAGCACCAGAATAAGGTTTGCCGGCATGTACAGAACGGAGCACTTGTAATTCTGCAAGCCAGTTCTCTAAGCATTTCCAATCAGATAGGGATGT
GTAGCACTCAATAACACGCTCTATGATGTACTGAATGCCATCAGACTCCATGAAGGTAAGGGCTTCCTCCGACTGAAGTAGATGTGAATAATGTGCAGCA
GCTTTTTCATACTGCCCCTGGGATTGATAGATCAGGCCTGACATCCAGGAAAAGTGCTTGTCACTATCAGATATACCACGCATTTGTTGATTGTCTTGCT
CAAAGCAAGTGTAGAATGTTGACACAGCCCACTTCTGAAGTCCAACTAAGGTGTCTGTTTCATTACACCTGCACAGTGCCAATGAAGCATGCCGGAGGGC
CTTCAGAACATCTGCTTTTAACCTCTCTCTAAAAGCATGATGACTTTCAGTAGCCCCTCCCATGTGACTGTTGCCCCTGAGGGATGATGCAGCCAGGTTT
CTGAGATCTACCAGGCGCAACGAACAGTAGTGAACAACTGCATCGCTACAGTGCATAGCTAATCCTGCATTCAACATTGGATCACAAATTCGAGAAAACC
ACTCTTCACAAACTCGTTTGTTTGCGCGGAAAAAATACGGAACTTGCTTTGGGAGGGGGTGATAGAACAAAGGAACCTTCATATGCATTATAAGCATATTT
CTTCAATGCCTCAACAAAATCCAATAGCAGGCGCATAGGCAATAAGCATATGTCAGATGGCCCTATATACCTGCCTTCACCTTCCTGAGCTTCCAGTGTC
AATACATTTGTTACATCCAAAAGCATGCGTTCTAGAGCTGCAAATGTCTGTGTGGGACCACCAAGATGAGTTCGAAGCCGAAGGTTTATGCAGTGCCAGG
CAGCTTCATGGATTCCCCACCAAACCGCAGCAAGATTGTTCACTGAGTATATCCTATCAATAGTAGCTCTGTCCATGCCAGAGCCTTTGGATAATTCATG
CCCATCAGCATCTCCAGAGATATCACTTTGACCGGAAAGCATGTCTTTCTTGCCACGGTAACTAAAGACCAACCGTTGCACCCAAGATGAAAGAGGTAAC
TTTAATCTGAGAGAAAGGTAACTCAAAATAGAAACTAGCTGCTGCCAATGGAGTTTCCTAGGCTGTTGCTTCACAGCAAGCACATGCCTCCAGCTCATGC
AATGGTTGCTCATGTTAGCATTAGCAACATATGAGGAATTCTGATAACTATGTTTGTCCGCAAGCAAGCCAAATGCATATGTGGTGATGGGCAAAGCAAT
TGAAAATAACCTTAGGAATGTGTTCTTTACAGTACTATCTGGATCGCTAAGCTTGTCAAGAAGAACCTGAGCAACAGCTGAGAAATTCAAGGGACTGATG
AGCCTACCCAGAAATAGCAACTCCAAGGATGATGCAACATGGCATCTCACTTTAGGCTCCCTGTCATATGCGCAATCCAAGATAGAGAAAAGGATAACAT
TTGAAAGAGCAGCATCCCCAAATACCTCATACGAGAATCTGACCTTAGCCATGTCCCTCTCTGTGCCACGAACCAAATTTTCAAACGAATCTATCCAATC
CAGAGTCTCTAATTTCAGTGTCATGGATGATGAAGTGTTAAGGCCCTGAACTATGTATTTTCCATATTTATGCACAAACTTCTTTTTGCATTCACAAGAA
GACTTCTTTTCTGCAACAATCAGCTGAGTTTTACTACCATCAGAGCTTCTTTCAAATGCTTCAGATCTTGGGATATTACTTGAGAGCTCCACGTTGCTCA
GTTTGTGTAGTGTAGAGAAAATCTGAAACTGCATCTCAAGGAAATTATGGAAAGGAGTGCCAAATGGGTTAAGCTTCTCTGAGATGAATGATGCTAACCT
ACTTGCTCGCTCATTTCTCCTTTTTGCATCGAGTGAAATCGTGGCCAGAAGAATCTTCAAATCAAATTTCATTAAGGAAAATAACTGATCCTCTGAATAC
CGCACCCAAGAGTGTGCACCTGAATTTGAATTGCCTTGTGTTTCATTGTTACACTCTAAGGAGAGATTCTGGATGTCAGGGTAACATACTTGCCCTTCCT

CAAGCAGTGATTTTATCAACACCATCTCTTTCATCAATGAGGCAGCTGCTTGGTCAACAACATCTTCCGATCCATGCTGCAGGCAAAAAAGGTAAGTGGC
TGCTGAGTTTGCAACCACGGTGTGGTTTGGATGAAGACGCAACCTAGATAAAACTGAACTGAAATCAAGCAAATCTCCCACGGCTGGTGCATGGATCCCC
AAATTTTGCAATGACAGAACTTGCAGGTTAGTCTTAAGTGCTGCCACCAATTGCGCTGATGAGGCTACTTTCAAGCTCTGGGCCAACACGTCCACAAATG
TAACATAGAATTCAGCAAACCTTTCCTGCAGAATCTCTGCGAGCAAGACCAAGCATCTGTATGCTTCTTCCATCCTCTCAGACCAGCCAAGCTTGGAGGC
TATCACTGATGCACACCTTAAAAGCTGTGGAGCTAACCCCTCCAGTGGCCCTGCTACAAGTTCCCTTAGGTTAGTTCTCTCAGCCACACCAGATGCCATG
ATCTGCAGTACCGTAGAGAAGCAAGCAAATAGGGGACGAAGCCGCCCAGAATTTTGGCTGGCTGCAAGGTTGGGGTCATGCACAAGCACCTCCATATCAG
CCAAGAACTTGGGGAGCAATCCAAGCGAGAACTGCAGATTCCCAAGCCAATGCCACTGGAACTGCGTGAAGCTATCCATGATCATGCTGCGATCAGCGTC
AGCGAGGTCCGGCACAAACGCCCACCCAAGGAGCAGGTCCATGATATCAACAAACTGCGGCCGGAAGCAGTGAGGGAACTTGGCGGCAACCAGCATGAGC
AGGCCAAGCAGACACCTCAGCACGG
>comp73186_c0_seq1
GTGAAGACGCCGCACCCGCCGCCACCGACCAACGCCGCAACCGCTACGGCAGCTGCAGCATCATCACGATTCCCACCCTACGTTACGACGCAAGCCCCGA
GCTGGGTTCAGAAGAATGGATTCCATTCGCTGACCAGACCCACCTAGATCAAGAACAGAGGGCACATTCATCGATCGTCTTCCATGTCAAAGATCGATCG
TTTGACCCAGGGAAGCGGAGGAAAGGATTCCTCAACCCGCGACGTTTCTTACCAGAGAAAAACTGGATGCGATTCTTCCCCTTTATCCTTTGTCATGGCC
GTTTTGTTCTCTGTTTCTTGATTGGGTGAGAGAGCTGGATTCGAGGATGGATGGACCTGTAAATGAGGAGTTCCCTAGATGACGAGAGTGATTGGTTAGC
GTTGCTTCAAGCAGTGTATCTTTTGGCCGCCTTTTCCTTTTCACCATTGGCATTGGCCAATGGCCAGCAACGATGGCATTGGGTGTCAACATCACAG
>Locus_5417_Transcript_26/28_Confidence_0.422_Length_5291
ATTACAAGACTATTTGATGCAACAATCACGCATATGGCTGCGCTAGAACTACTGCTCTGAATTAAGAGGCGTTGAAATATTGTCCGGTCTACATATAGGC
ACTTGAGGTGTATTGACAGACAGTTCCCCTTAGTTTGCTACAACACATCAGGCGAATTCCACCAAATTAGGATGATGTTTGTTTCTTAGACACAGGATAG
CAATGCCAAAAGTTTTTTATAATTTTCCGCTTCACCGCAGATTTGGCATCTCAAAGGGAGTTGTGGCAAGATTCTCTTGACCAAGCTTTCTTGCAGATTT
GGCAAGAAAGATAAAAAAAATGTTGGTATACTTAAGCATCCTTAATAGAAACTAAACAGGTCTTAGATTGAAATTATCATAAATAAACCCTTTTTCACTT
TAGCTGTAGTCCGGATGAAATCTTAGGGGCCTTGCTTAGCCTGCCCTGAAGCTTGCCAATAAGTCGAAGCTGATGCTGACACAGCACTGAAATAGATCCC
AATGAACACAAGTTATTTCGAGAAGATAAAATACCAAGCAAAGCCAGGATTGCATTAAAAGATAGTGTATTTCTACTATACGATCTGAAAGCTTAATTTA
TTATATCAATAATCAAATTGGCAGCAGTGTGGAACAGATATAAAGAAGATGCTTCAAATCACAAATAACTACAGATAAATTGAAGGGTGAAGGTTCATTC
AACCACCAAACACCCAAAATGTCGCGCTGCATATACACAGAACCTAAGTACGGTACAGTTCATAGTTTGGGAAAATAGACTGCAAATGTGCACAGATAAT
ACAGATTGTTACTCGATGAACAGTAAACACGGTATTCGAAATCCTTCTAACCATCCATCCAGGCAGTGCCTCGTATACTCAAACCTCTTTTTGGAGGAAG
CCAAACCATGTGATCCTTTTCAACCCAAATACATATATTCAAGATGAAACTAACAAAAAGACAGTTGATAAAGAAACACGGAAGAGAGTAGAAAAAGGGT
ACCTGTTTCAAGTTCCCATCTTCTGAAAATGATCAGCAGTCAATAGATGTTTTAGTCCATTCTCCAGAAAAATGAGTGCCGCATGAATCCTTTCCGGTTT
ATAGTGAAACATTTTAGACGATGACCTTTAACATTCTAGAAAACGAAGAGAAAACCTAAAGTCACCTAATTTCAGCTTAAACATGTTCCAGCAAGATCAT
CACCATCCTGACATGTTCCGTTTTGTCCCTAGACCTGAGCACAAGGTATTGCGAGGCTAATTTAGCGGGGCAATTGATTTTTTGCCACTATGATACAACT
GCAATTACCCGCTGCCACTGACACGTGAGGTCACCTGAGTCACTGAAATGTGGGCCCAGTGGCATTTTGTTTTGCCACTCCCCTTCCAGAGTGGCAAAAA
GTTAATTGTCCCTTAATTTAGCTCAATTCAGTTGTCGGAGCAATTGTGTGTGAAAAGTGTGAACAACAGCATAGGAGACAATACAGCAAGTGTAGCGTGC
CAATATGTGTCCAGCTTGATAGTTCACTATAATAAAGTTCTTTAATTCAAGTATTTGAATAATATAGAATCTCAACCTTCAAGTTTAAAAGGTTTTGTAT
TTGCTATTATAATCGTTGCCACCCCCTCACCCCTCTCAAAAAGAGAGTTACTTTGTAGCGCACTGTAAATCTCTTGATTGAAATAGTGGCATGTCATCTT
ATTTAATTCAGTTATTTCATAAGGTACAATTTGTTTAGCTGACAGCTCAGACATGCTTCTATGCAACATGTTTATACCAATGCTGTATTTTGGCAATCCT
GGATGTGGCATTACTTAACTGTGCTTTCATATTCAATAGTTCTGGCAAAGGGAGGCTGCAAGCTTGAGACAACAACTGCACAACCTGCAAGAAAGTCATC
GGTATGGCTAGAAAACCCTGCAAATTTTACAAATGTACAAGTCAGAAAATTTGCCTAATGTAAAAGGATCAAACTACAGGATTTGTCAGCATGAGCACAA
GTGCCTAACTTCCGAGTTAGATAACTCTGGAGGAAGGAAATCCTTCCTACTGTTTGTTTCAAGAATATACAAAAATTCAAAACAACATTAGAATAGAACC
AAAAGAAGAAACAATATTCACCATGTAGCAATACTCAATTCCTGGTTGTCTTATTTATAAAGGTATAAAACTAGGTTCTGACGAGACAATTATGAATCAA
CTACACCATGTTACAGATCATCCATAACTCTTGGTGATGCAAATTAAGGGCTGTAAAGTGACAAGTATACCTCCACCAACTAATTTGTGCAGAGTGTATA
AAATTGTAAAACAATGGATAAGAATGAATTTAGTATACAAGATAGCATACAATGCCCAAGTTTAAGGAGACATACTGATTTACTGACTCCATAGGACAAT
CTTTTGCTTATGAGGACATCAGTTTTCATAAACAATATCAATGTAAAAGGAGGTAACAGTTTATGAATTTCCTATGCCAAGCCATTTATAAGTCAAAAAA
TGTACCTATTTAGCTTTTATGCATTCCAATTAACTTCCAAAGAAGAAGAGTACATACTAATGGAAAACAAATATGATATTATCATCTGTTTTGCCCGGTA
GTTTCTTGAGTCTCTGATTTGTCCGCACTTCACTCCCAACCAACACAACATTAATACACATGGGCAGAGGAATATCAGGTTATCAGGTAAACTGAATACA
GTTTGATTGACTGCAGTCAGTTGATGGGACAAGATCTTTCTGGATTGAGCGTCAAGGAACTCCAAAATCTAGAAAATCAGCTAGAAATGAGCCTACGTTG
CATCAGGACAAAAAAGGTAAGGATGCTAGCCATATTTCTGTTTTTCGTTACCAGATTGCCTCATCCAAGAATACAACTGATGATGACGTTTTTTACTTTC
AGGACCAACTCTTGATTGATGAAATTCACGAACTGAATCGAAAGGTTCTGGAATATTCTGTTATTACTCTGCAATGAGAGGTCTCTGAGTGGATTTCAAG
AAGCTGACTCTACTGGCTCATTTTCAGGGAAGTCTCATGCAACAAGACAAGATGGAACTATACAAAAAGGTCAACCTAATCCGTCAGGAAAACATTGATT
TATACAAGAAGGTATTTGATAAATCGTGAATGAAAATTCACAGGATATACACCCCATCCATTATTGACTGAGGTTTATTTCTCTACTCAGCTCTACGAGA
AAGAGGTGACAAGTGAAGTCAACCGAGATTCATCAACTCTAAACTTTGCAGTTGTCGAGAACGTCAACATTCCTGTTCATCTTGAACTTAACACTCCACC
GCAAGAAAATGATGTTGAGCAAACTGCACCTCCTAAACTGGGGTAACCCTGCTTTTTTCTGCAAAGGAACTATTGATTCTGTTCTTAATTCTTTAGTGGC
ATATTGTTTACTACTAACTGAAAACTTGCAGATTACAACTAAATCCATGAAGGCATGCAGAATGGCACCGCTTATGTTCATTATTCTCAACGGTACGATT
CAAAGCAACCAGAAACACTATTGGTTGTAAAGAGCTTAAACACATAGCAAGACTGACGCGGTATGCATGCCATGAGTGATTGATAATGGTAGTGTAAATC
TATTTCAAATAAAATGTATTTTATTGAAGAATACAGCAAATGATTTGTATTATAACTATTTGACACATTCTGCAAGTTTGTTGACCAACGTGAGTACATG
ATAGCTACAAATCTTATATTGACAAAAAGATATGGTGTGTGGAAAAAAATTCAGGTGCTATTTTTTTCTATTTTGAGAATTGTATGCATAGCAGAAGAGCA
CAAATTGCATTCTGGTTTATCCGTGCAGGCATTCCAGAGTATCAGAATGGGGATCAACCTTTCTTCAGCTACATATGTAAATATACGGATGCTAGTTGCT
CACTTAAAATTTTGTAGTGTTCAAAGGAATAACAACAGTAAGACGTTGACGCACCGAAAATAACCAGAAATAACGGATTGTACTATGACAAGACACACGA
CAGATACACTAGCTTAAGTTTGTACAGGAAAATATTTGGCCTATTCTTATGTCACAGTAAATTAAGATTATCACCTTAATCACAGAAATTCAAGGGGTAA
ACGATTATAGCACAAAACGATCCTGGACCCCAAGACCTGGGTCTCTGAACGGCTGAACCTCCAATTATACCTCTATACATTTGGAATGTGTGTAGTCAAA
CTTTCGCTACTGGTATCTTTACAAATATTTACATTGAACACATCAAAATGATATACATGGATTCCTCTCGATATGTCTTTGAGGTTTGGGTTCTAAAAAT
GATATACAAATTATTAGGTTCTAAACTTAAATGATATATACACATCAAAATGATATACAAATTATTGGGTTCTAAACTTAAATGATGTATACACATCGAA
ATGATATACAAATTATTAGGTTCTAAAAATATACTATGGCAGAATTAGGTGGTCATTTGAGTTTTGGAGGCCTTGTCAATGTCCCAAACATCACTTATTT
GTGACTGGGAGAGCAATGAATTGAAGGAATGAGAGTAACCATACGGTCATTGGTCAATTAAAAGGAACTTTAATATGCTATGACCACATCGACCTTTCGT
AAAGTAAATAACTCAATTATAACCAAGTAATTGGGTGTCTATTGAATCCATACGGTTCTCAAAACAGTTCTAAACAAAGTGAATTTAAGCATCCTGAAAA
GATATTTGGCAAATTTGCCATACCCTGAATTCACAGCATGTTAACACAAATCATGTGCAAATCATTCAGATCATTGACAAACGCACGCATGCACACAAAT
CAACACCGTTTTCTCATGTATAGAACAGAAAAATTGAGAAACTTACGATTTTCTTGCAAGTTATGCAGTTGTTATCTCTAGCTTGCCGCCTCCACTTGCA
GTAACTGAGACGCCGGCGCCGAGCTTGAGCTTGAAACCTAGACTACGGGGACAGGGTAGACAGGAGACGCGAAACCCCAAACCAGCGCAAAACGACGCGC
AGGGAGGGAGTGTCACTGAAGGAGCGACAGGGAAAGCAGCGGCACGCCGGGGGAAATTCCGATCTGAGAGAACGGATCAGCCATGCATGACAGGAGCTGC
GGAGGCAGCAGGGGGGGGGGGCTCCCGCTGCCACGGGACAGTAGGGCACGCCAGATCGGTGAAATCCAGGCGAAGGACGAGGCGTGGGCGAGATTTACAC
GACGGCGCGATAGGATCTAGCCCGGATTTGGATTCGACCGACGAGGGCGGAAGGAACGGCAGGGTGGTGCTCTTGTAGAGAGAGAAGGAGT
>Locus_3441_Transcript_132/417_Confidence_0.057_Length_2633
CGCATATGGCTGCGCTAGAACTACTGCTCTGAATTAAGAGGCGTTGAAATATTGTCCGGTCTACATATAGGCACTTGAGGTGTATTGACAGACAGTTCCC
CTTAGTTTGCTACAACACATCAGGCGAATTCCACCAAATTAGGATGATGTTTGTTTCTTAGACACAGGATAGCAATGCCAAAAGTTTTTTATAATTTTCC
GCTTCACCGCAGATTTGGCATCTCAAAGGGAGTTGTGGCAAGATTCTCTTGACCAAGCTTTCTTGCAGATTTGGCAAGAAAGATAAAAAAAATGTTGGTA
TACTTAAGCATCCTTAATAGAAACTAAACAGGTCTTAGATTGAAATTATCATGAATAAACCCTTTTTCACTTTAGCTGTAGTCCGGATGAAATCTTAGGG
GCCTTGCTTAGCCTGCCCTGAAGCTTGCCAGTAAGTCGAAGCTGATGCTGACACAGCACTGACATAGATCCCAATGAACACAAGTTATTTCGAGAAGATA
AAATACCAAGCAAAGCCAGGATTGCATTAAAAGATAGTGTATTTCTACTATACGATCTGAAAGCTTAATTTATTATATCAATAATCAAATTGGCAGCAGT
GTGGAACAGATATAAAGAAGATGCTTCAAATCACAAATAACTACAGATAAATTGAAGGGTGAAGGTTCATTCAACCACCAAACACCCAAAATGTCGAGCT
GCATATGCACAGAACCTAAGTACGGTACAGTTCATAGTTTGGGAAAATAGACTGCAAATGTGCACAGATAATACAGATTGTTACTCGATGAACAGTAAGC

ACGGTATTTGAAATCCTTCTAACCATCCATCCAGGCAGTGCCTCGTATACTCAAACCTCTTTTTGGAGGAAGCCAAACCATGTGATCCTTTTCAACCCAA
ATACATATATTCAAGATGAAACTAACAAAAAGACAGTTGATAAAGAAACACGAAAGAGAGTAGAAAAAGGGTACCTGTTTCAAGTTCTCATCTTCTGAAA
ATGATCAGCAGTCAATAGATGTTTTAGTCCATTCTCCAGAAAAATGAGTGCCGCATGAATCCTTTCCGGTTTATAGTGAAACATTTTAGACGATGACCTT
TAACATTCTAGAAAACGAAGAGAAAACCTACAGTCATCTAATTTCAGCTTAAACATGTTCCAGCAAGATCATCACCATCCTGACATGTTCCGTTTTGTCC
CTAGACCCGAGCACAAGGTATTGCGAGGCTAATTTAGCTCAATTCAGTTGTCAGAGCAATTGTGTGTGAAAAGTGTGAACAACAGCATAGGAGACAATAC
AGCAAGTGTAGCGTGCCAATATGTGTCCAGCTTGATAGTTCACTATAATAAAGTTCTTTAATTCAAGTATTTGAATAATATAGAATCTCAACCTTCAAGT
TTAAAAGGTTTTGTATTTGCTATTATAATCGTTGCCACCCCCTCACCCCTCTCAAAAAGAGAGTTACTTTGTAGCGCACTGTAAATCTCTTGATTGAAAT
AGTGGCATGTCATCTTATTTAATTCAGTTATTTCATAAGGTACAATTTGTTTAGCTGACAGCTCAGACATGCTTCTATGCAACATGTTTATACCAATGCT
GTATTTTGGCAATCCTGGATGTGGCATTACTTAACTGTGCTTTCATATTCAATAGTTCTGGCAAAGGGAGGCTGCAAGCTTGAGACAACAACTGCACAAC
CTGCAAGAAAGTCATCGGTATGGCTAGAAAACCCTGCAAATTTTACAAATGTACAAGTCAGAAAATTTGCCTAATGTAAAAGGATCAAACTACAGGATTT
GTCAGCATGAGCACAAGTGCCTAACTTCCGAGTTAGATAACTCTGGAGGAAGGAAATCCTTCCTACTGTTTGTTTCAAGAATATACAAAAATTCAAAACA
ACATTAGAATAGAACCAAAAGAAGAAACAATATTCACCATGTAGCAATACTCAATTCCTGGTTGTCTTATTTATAAAGGTATAAAACTAGGTTCTGACGA
GACAATTATGAATCAACTACACCATGTTACAGATCATCCATAACTCTTGGTGATGCAAATTAAGGGCTGTAAAGTGACAAGTATACCTCCACCAACTAAT
TTGTGCAGAGTGTATAAAATTGTAAAACAATGGATAAGAATGAATTTAGTATACAAGATAGCATACAATGCCCAAGTTTAAGGAGACATACTGATTTACT
GACTCCATAGGACAATCTTTTGCTTATGAGGACATCAGTTTTCATAAACAATATCAATGTAAAAGGAGGTAACAGTTTATGAATTTCCTATGCCAAGCCA
TTTATAAGTCAAAAAATGTACCTATTTAGCTTTTATGCATTCCAATTAACTTCCAAAGAAGAAGAGTACATACTAATGGAAAACAAATATGATATTATCA
TCTGTTTTGCCCGGTAGTTTCTTGAGTCTCTGATTTGTCCGCACTTCACTCCCAACCAACACAACATTAATACACATGGGCAGAGGAATATCAGGTTATC
AGGTAAACTGAATACAGTTTGATTGACTGCAGGCAGTTGATGGGACAAGATCTTTCTGGATTGGGCGTCAAGGAACTCCAAAATCTAGAAAATCAGCTAG
AAATGAGCCTACATTGCATCCGGACAAAAAAGG
>Locus_5417_Transcript_17/28_Confidence_0.312_Length_4633
ATTACAAGACTATTTGATGCAACAATCACGCATATGGCTGCGCTAGAACTACTGCTCTGAATTAAGAGGCGTTGAAATATTGTCCGGTCTACATATAGGC
ACTTGAGGTGTATTGACAGACAGTTCCCCTTAGTTTGCTACAACACATCAGGCGAATTCCACCAAATTAGGATGATGTTTGTTTCTTAGACACAGGATAG
CAATGCCAAAAGTTTTTTATAATTTTCCGCTTCACCGCAGATTTGGCATCTCAAAGGGAGTTGTGGCAAGATTCTCTTGACCAAGCTTTCTTGCAGATTT
GGCAAGAAAGATAAAAAAAATGTTGGTATACTTAAGCATCCTTAATAGAAACTAAACAGGTCTTAGATTGAAATTATCATAAATAAACCCTTTTTCACTT
TAGCTGTAGTCCGGATGAAATCTTAGGGGCCTTGCTTAGCCTGCCCTGAAGCTTGCCAATAAGTCGAAGCTGATGCTGACACAGCACTGAAATAGATCCC
AATGAACACAAGTTATTTCGAGAAGATAAAATACCAAGCAAAGCCAGGATTGCATTAAAAGATAGTGTATTTCTACTATACGATCTGAAAGCTTAATTTA
TTATATCAATAATCAAATTGGCAGCAGTGTGGAACAGATATAAAGAAGATGCTTCAAATCACAAATAACTACAGATAAATTGAAGGGTGAAGGTTCATTC
AACCACCAAACACCCAAAATGTCGCGCTGCATATACACAGAACCTAAGTACGGTACAGTTCATAGTTTGGGAAAATAGACTGCAAATGTGCACAGATAAT
ACAGATTGTTACTCGATGAACAGTAAACACGGTATTCGAAATCCTTCTAACCATCCATCCAGGCAGTGCCTCGTATACTCAAACCTCTTTTTGGAGGAAG
CCAAACCATGTGATCCTTTTCAACCCAAATACATATATTCAAGATGAAACTAACAAAAAGACAGTTGATAAAGAAACACGGAAGAGAGTAGAAAAAGGGT
ACCTGTTTCAAGTTCCCATCTTCTGAAAATGATCAGCAGTCAATAGATGTTTTAGTCCATTCTCCAGAAAAATGAGTGCCGCATGAATCCTTTCCGGTTT
ATAGTGAAACATTTTAGACGATGACCTTTAACATTCTAGAAAACGAAGAGAAAACCTAAAGTCACCTAATTTCAGCTTAAACATGTTCCAGCAAGATCAT
CACCATCCTGACATGTTCCGTTTTGTCCCTAGACCCGAGCACAAGGTATTGCGAGGCTAATTTAGCTCAATTCAGTTGTCAGAGCAATTGTGTGTGAAAA
GTGTGAACAACAGCATAGGAGACAATACAGCAAGTGTAGCGTGCCAATATGTGTCCAGCTTGATAGTTCACTATAATAAAGTTCTTTAATTCAAGTATTT
GAATAATATAGAATCTCAACCTTCAAGTTTAAAAGGTTTTGTATTTGCTATTATAATCGTTGCCACCCCCTCACCCCTCTCAAAAAGAGAGTTACTTTGT
AGCGCACTGTAAATCTCTTGATTGAAATAGTGGCATGTCATCTTATTTAATTCAGTTATTTCATAAGGTACAATTTGTTTAGCTGACAGCTCAGACATGC
TTCTATGCAACATGTTTATACCAATGCTGTATTTTGGCAATCCTGGATGTGGCATTACTTAACTGTGCTTTCATATTCAATAGTTCTGGCAAAGGGAGGC
TGCAAGCTTGAGACAACAACTGCACAACCTGCAAGAAAGTCATCGGTATGGCTAGAAAACCCTGCAAATTTTACAAATGTACAAGTCAGAAAATTTGCCT
AATGTAAAAGGATCAAACTACAGGATTTGTCAGCATGAGCACAAGTGCCTAACTTCCGAGTTAGATAACTCTGGAGGAAGGAAATCCTTCCTACTGTTTG
TTTCAAGAATATACAAAAATTCAAAACAACATTAGAATAGAACCAAAAGAAGAAACAATATTCACCATGTAGCAATACTCAATTCCTGGTTGTCTTATTT
ATAAAGGTATAAAACTAGGTTCTGACGAGACAATTATGAATCAACTACACCATGTTACAGATCATCCATAACTCTTGGTGATGCAAATTAAGGGCTGTAA
AGTGACAAGTATACCTCCACCAACTAATTTGTGCAGAGTGTATAAAATTGTAAAACAATGGATAAGAATGAATTTAGTATACAAGATAGCATACAATGCC
CAAGTTTAAGGAGACATACTGATTTACTGACTCCATAGGACAATCTTTTGCTTATGAGGACATCAGTTTTCATAAACAATATCAATGTAAAAGGAGGTAA
CAGTTTATGAATTTCCTATGCCAAGCCATTTATAAGTCAAAAAATGTACCTATTTAGCTTTTATGCATTCCAATTAACTTCCAAAGAAGAAGAGTACATA
CTAATGGAAAACAAATATGATATTATCATCTGTTTTGCCCGGTAGTTTCTTGAGTCTCTGATTTGTCCGCACTTCACTCCCAACCAACACAACATTAATA
CACATGGGCAGAGGAATATCAGGTTATCAGGTAAACTGAATACAGTTTGATTGACTGCAGTCAGTTGATGGGACAAGATCTTTCTGGATTGAGCGTCAAG
GAACTCCAAAATCTAGAAAATCAGCTAGAAATGAGCCTACGTTGCATCAGGACAAAAAAGGACCAACTCTTGATTGATGAAATTCATGAACTGAATCGAA
AGGTTCTGGAATATTCTGTTATTACTCTGCAATGAGAGGTCTCTGAGTGGATTTCAAGAAGCTGACTCTACTGGCTCATTTTCAGGGAAGTCTCATGCAA
CAAGACAAGATGGAACTATACAAAAAGGTCAACCTAATCCGTCAGGAAAACATTGATTTATACAAGAAGCTCTACGAGAAAGAGGTGACAAGTGAAGTCA
ACCGAGATTCATCAACTCTAAACTTTGCAGTTGTCGAGAACGTCAACATTCCTGTTCATCTTGAACTTAACACTCCACCGCAAGAAAATGATGTTGAGCA
AACTGCACCTCCTAAACTGGGGTAACCCTGCTTTTTTCTGCAAAGGAACTATTGATTCTGTTCTTAATTCTTTAGTGGCATATTGTTTACTACTAACTGA
AAACTTGCAGATTACAACTAAATCCATGAAGGCATGCAGAATGGCACCGCTTATGTTCATTATTCTCAACGGTACGATTCAAAGCAACCAGAAACACTAT
TGGTTGTAAAGAGCTTAAACACATAGCAAGACTGACGCGGTATGCATGCCATGAGTGATTGATAATGGTAGTGTAAATCTATTTCAAATAAAATGTATTT
TATTGAAGAATACAGCAAATGATTTGTATTATAACTATTTGACACATTCTGCAAGTTTGTTGACCAACGTGAGTACATGATAGCTACAAATCTTATATTG
ACAAAAAGATATGGTGTGTGGAAAAAATTCAGGTGCTATTTTTTTCTATTTTGAGAATTGTATGCATAGCAGAAGAGCACAAATTGCATTCTGGTTTATC
CGTGCAGGCATTCCAGAGTATCAGAATGGGGATCAACCTTTCTTCAGCTACATATGTAAATATACGGATGCTAGTTGCTCACTTAAAATTTTGTAGTGTT
CAAAGGAATAACAACAGTAAGACGTTGACGCACCGAAAATAACCAGAAATAACGGATTGTACTATGACAAGACACACGACAGATACACTAGCTTAAGTTT
GTACAGGAAAATATTTGGCCTATTCTTATGTCACAGTAAATTAAGATTATCACCTTAATCACAGAAATTCAAGGGGTAAACGATTATAGCACAAAACGAT
CCTGGACCCCAAGACCTGGGTCTCTGAACGGCTGAACCTCCAATTATACCTCTATACATTTGGAATGTGTGTAGTCAAACTTTCGCTACTGGTATCTTTA
CAAATATTTACATTGAACACATCAAAATGATATACATGGATTCCTCTCGATATGTCTTTGAGGTTTGGGTTCTAAAAATGATATACAAATTATTAGGTTC
TAAACTTAAATGATATATACACATCAAAATGATATACAAATTATTGGGTTCTAAACTTAAATGATGTATACACATCGAAATGATATACAAATTATTAGGT
TCTAAAAATATACTATGGCAGAATTAGGTGGTCATTTGAGTTTTGGAGGCCTTGTCAATGTCCCAAACATCACTTATTTGTGACTGGGAGAGCAATGAAT
TGAAGGAATGAGAGTAACCATACGGTCATTGGTCAATTAAAAGGAACTTTAATATGCTATGACCACATCGACCTTTCGTAAAGTAAATAACTCAATTATA
ACCAAGTAATTGGGTGTCTATTGAATCCATACGGTTCTCAAAACAGTTCTAAACAAAGTGAATTTAAGCATCCTGAAAAGATATTTGGCAAATTTGCCAT
ACCCTGAATTCACAGCATGTTAACACAAATCATGTGCAAATCATTCAGATCATTGACAAACGCACGCATGCACACAAATCAACACCGTTTTCTCATGTAT
AGAACAGAAAAATTGAGAAACTTACGATTTTCTTGCAAGTTATGCAGTTGTTATCTCTAGCTTGCCGCCTCCACTTGCAGTAACTGAGACGCCGGCGCCG
AGCTTGAGCTTGAAACCTAGACTACGGGGACAG
>Locus_14339_Transcript_56/63_Confidence_0.167_Length_924
ATTTTTAGCAATGTATTAGTACATCACGCCACACATTTCTAGATTATTTTCTGTAGATAAGAAACTGACAGTACTTCTTGTTTTACTGATAGCTCTGGCA
AAGGGAGGCAGCAAGCTTGAGGCAACAACTGCACAACTTGCAAGAAAGCCACAAGTATGATCTTCCAATTGATTCTGCAATGAAAATACATCTCATCAAT
ACAAAGTTTAGATGCCACGTGGAAAATTTTGTAATAACTGTAAAACCAAAATGAAACGTATCATCCAGTGAAGATGAGGGAGAAAAAAATCTAGTTACTG
AAAGACATTTATAGTGTTCACTATTCAGAAAGGAAATTGAAAGTGGCTTTGTCACAGCAACATATATGTGGTTTTGGTTCGAACCGTGTACCCACGTAAA
TTGATATTGGTTTAGTTTCAATCTACACAAGATAATCTGACGAACTCTTTTGCATTGTTGTAGGCAACTAATGGGTGAGAAGCTTTCTGGCCTAGGTGTG
AGAGACCTCCAGAGTTTAGAGAATCGTCTTGAAATGAGCCTACGTAGTATCAGAACGAGGAAGGTCATATTCATTTTGTTTCCACGTTTTGTTTTTCTAT
TCACTCATCTTAATATGCATAAACATCCATCAAACAGGACAATCTTTTGAAAAGTGAAATTGAAGAGTTGCACAGGAAGGTAAAGCCTTACAATCCATGT
CTTTTTATTATCTCAAACAAATGACCATGCATGGAGGTTGCTGACATCTTAACTTAATGCTGATTGAAACAGGGTAGCCTAATTCACCAGGAAAACTTGG
AACTTTGTGACTTCCTTGCAAGTAATGCGGTTCCTGCCTCATGCTTGGTGCCGCCGCATGCAATAAACCGATAACTCGAATCCTCCGTGACTTCCACCCT
CTCCAGCCAATGAGCGGCTTGCTG

>comp83218_c0_seq14
CGCAGCTCGCAGTACCGCGGCGTCACCTTCTACCGCCGCACCGGCCGCTGGGAGTCCCATATTTGGGATTGCGGCAAGCAAGTGTACTTAGGTGGATTTG
ACACTGCTCATACTGCTGCAAGGGCGTACGATCGAGCTGCCATCAAGTTCCGTGGCTTCGACGCCGACATAAACTTCAATCTCAGTGACTATGAGGACGA
CATGAAGCAGATGAAGAGCCTGTCCAAGGAGGAGTTCGTGCACGTCCTGCGACGGCAGAGCACCGGGTTCTCACGGGGCAGCTCCAAGTACAGAGGCGTG
ACCCTGCACAAGTGCGGCCGATGGGAGGCTCGCATGGGGCAGTTCCTCGGCAAGAAGGCTTATGATAAGGCCGCGATCAAATGCAATGGTCGAGAAGCTG
TGACGAACTTCGAGCCAAGCACGTATGATGGGGAGCTGCTGACTGAAGTTGCTGATGAAGGTGCTGATGTTGATCTGAACTTGAGCATATCTCAACCAGC
TTCTCAGAGCCCGAAAAGGGACAAGAACTCCCTTGGTCTGCAACTCCACCATGGATCGTTTGAAGGCTACGAATTGAAAAGAACAAAGATTGATGCTCCC
TCTGAACTCACGGGCCGACCTCATCGGTTCCCTCTTCTGACCAAGCATCCACCAATCTGGCATGCCCAATCTCATCCCTTCTATTCAAATAATGAGAATG
CATCAAGAGATCTTAACAGGAGGCCTGATCAGGGAGGCACTGTTCCTAGATGGGCGTGGAAGGTGACCGCCCCTCCTCCCACCCAACCATTGCCGCTGTT
CTCGTCATCGTCGTCGTCGTCCGCTGCAGCATCATCAGGATTCTCCAATACCGCCACGACAGCTGCCCCCACCGCCCCGTCGTCCTCCTCCCTCCGGTTC
GACCCGCCGCCGCCGTCGAGCCGCCACCGATGAACATTGAGAAGCCACGCTGTAAATTTGCCGGGAAGCCGGCATTTTTTCCCTCTCCAGGCGTTGCAAC
TTTTTCGGTTTTGCGCCGGGGTGGTTTCTTAATTGTAGTGGATTGGATTCATAACTATATTTGCATACTACCGCGAATGGGTTTCTAGCTGAATTAGTTA
GATGGCTTATTACCACTCCTCAGATTCTAGGTTCGATTTCTCATGAGAGTGAATTTCAGATTAGGGTTAAAAAAATCTCTTTGTCTGTCCTACGCCAAAG
CACAGGTTTAAAGCTCGGCCCGGTCCTCATACGGGCTATTGTGCCACTGTGTGGTTCGGAGGTTTTCTTGATCTACGTGAGAAGATCTTCTTCTTAATGA
AATACCCGGGGACTGTCCCCCGCAGGTCAAGTTTATTTGCATACTGCCCCATGTGAAACCTTCTTTCTCACTCTCTTTACACACTCTGCAACGGATAGAT
GTTCTTGTTGATGGAAATTAGCTAACTTCACCGTCTGAGATGATCTATACATTGTTGTAGAGAA
>Locus_5417_Transcript_19/28_Confidence_0.250_Length_4461
ATTACAAGACTATTTGATGCAACAATCACGCATATGGCTGCGCTAGAACTACTGCTCTGAATTAAGAGGCGTTGAAATATTGTCCGGTCTACATATAGGC
ACTTGAGGTGTATTGACAGACAGTTCCCCTTAGTTTGCTACAACACATCAGGCGAATTCCACCAAATTAGGATGATGTTTGTTTCTTAGACACAGGATAG
CAATGCCAAAAGTTTTTTATAATTTTCCGCTTCACCGCAGATTTGGCATCTCAAAGGGAGTTGTGGCAAGATTCTCTTGACCAAGCTTTCTTGCAGATTT
GGCAAGAAAGATAAAAAAAATGTTGGTATACTTAAGCATCCTTAATAGAAACTAAACAGGTCTTAGATTGAAATTATCATAAATAAACCCTTTTTCACTT
TAGCTGTAGTCCGGATGAAATCTTAGGGGCCTTGCTTAGCCTGCCCTGAAGCTTGCCAATAAGTCGAAGCTGATGCTGACACAGCACTGAAATAGATCCC
AATGAACACAAGTTATTTCGAGAAGATAAAATACCAAGCAAAGCCAGGATTGCATTAAAAGATAGTGTATTTCTACTATACGATCTGAAAGCTTAATTTA
TTATATCAATAATCAAATTGGCAGCAGTGTGGAACAGATATAAAGAAGATGCTTCAAATCACAAATAACTACAGATAAATTGAAGGGTGAAGGTTCATTC
AACCACCAAACACCCAAAATGTCGCGCTGCATATACACAGAACCTAAGTACGGTACAGTTCATAGTTTGGGAAAATAGACTGCAAATGTGCACAGATAAT
ACAGATTGTTACTCGATGAACAGTAAACACGGTATTCGAAATCCTTCTAACCATCCATCCAGGCAGTGCCTCGTATACTCAAACCTCTTTTTGGAGGAAG
CCAAACCATGTGATCCTTTTCAACCCAAATACATATATTCAAGATGAAACTAACAAAAAGACAGTTGATAAAGAAACACGGAAGAGAGTAGAAAAAGGGT
ACCTGTTTCAAGTTCCCATCTTCTGAAAATGATCAGCAGTCAATAGATGTTTTAGTCCATTCTCCAGAAAAATGAGTGCCGCATGAATCCTTTCCGGTTT
ATAGTGAAACATTTTAGACGATGACCTTTAACATTCTAGAAAACGAAGAGAAAACCTAAAGTCACCTAATTTCAGCTTAAACATGTTCCAGCAAGATCAT
CACCATCCTGACATGTTCCGTTTTGTCCCTAGACCCGAGCACAAGGTATTGCGAGGCTAATTTAGCTCAATTCAGTTGTCAGAGCAATTGTGTGTGAAAA
GTGTGAACAACAGCATAGGAGACAATACAGCAAGTGTAGCGTGCCAATATGTGTCCAGCTTGATAGTTCACTATAATAAAGTTCTTTAATTCAAGTATTT
GAATAATATAGAATCTCAACCTTCAAGTTTAAAAGGTTTTGTATTTGCTATTATAATCGTTGCCACCCCCTCACCCCTCTCAAAAAGAGAGTTACTTTGT
AGCGCACTGTAAATCTCTTGATTGAAATAGTGGCATGTCATCTTATTTAATTCAGTTATTTCATAAGGTACAATTTGTTTAGCTGACAGCTCAGACATGC
TTCTATGCAACATGTTTATACCAATGCTGTATTTTGGCAATCCTGGATGTGGCATTACTTAACTGTGCTTTCATATTCAATAGTTCTGGCAAAGGGAGGC
TGCAAGCTTGAGACAACAACTGCACAACCTGCAAGAAAGTCATCGGTATGGCTAGAAAACCCTGCAAATTTTACAAATGTACAAGTCAGAAAATTTGCCT
AATGTAAAAGGATCAAACTACAGGATTTGTCAGCATGAGCACAAGTGCCTAACTTCCGAGTTAGATAACTCTGGAGGAAGGAAATCCTTCCTACTGTTTG
TTTCAAGAATATACAAAAATTCAAAACAACATTAGAATAGAACCAAAAGAAGAAACAATATTCACCATGTAGCAATACTCAATTCCTGGTTGTCTTATTT
ATAAAGGTATAAAACTAGGTTCTGACGAGACAATTATGAATCAACTACACCATGTTACAGATCATCCATAACTCTTGGTGATGCAAATTAAGGGCTGTAA
AGTGACAAGTATACCTCCACCAACTAATTTGTGCAGAGTGTATAAAATTGTAAAACAATGGATAAGAATGAATTTAGTATACAAGATAGCATACAATGCC
CAAGTTTAAGGAGACATACTGATTTACTGACTCCATAGGACAATCTTTTGCTTATGAGGACATCAGTTTTCATAAACAATATCAATGTAAAAGGAGGTAA
CAGTTTATGAATTTCCTATGCCAAGCCATTTATAAGTCAAAAAATGTACCTATTTAGCTTTTATGCATTCCAATTAACTTCCAAAGAAGAAGAGTACATA
CTAATGGAAAACAAATATGATATTATCATCTGTTTTGCCCGGTAGTTTCTTGAGTCTCTGATTTGTCCGCACTTCACTCCCAACCAACACAACATTAATA
CACATGGGCAGAGGAATATCAGGTTATCAGGTAAACTGAATACAGTTTGATTGACTGCAGTCAGTTGATGGGACAAGATCTTTCTGGATTGAGCGTCAAG
GAACTCCAAAATCTAGAAAATCAGCTAGAAATGAGCCTACGTTGCATCAGGACAAAAAAGGACCAACTCTTGATTGATGAAATTCATGAACTGAATCGGA
AGGGAAGCCTCATCCAACAAGACAACATGGAACTATACAAAAAGGCCAACCTAATTCGTCAGGAAAACGTTGATTTATACAAGCTCTACGAGAAAGAGGC
AACAAGTGAAATCAACCGTGATTCAACAACTACATACAACTTTGCAGTTGTCGAGAATGCCAACATTCCTGTTCATCTTGAACTTAACAGTCCACCGCAA
GAAAATGACGTTGAGCAAACTGCACCCCCTAAACTAGGGTTACAACTAAATCCATGAAGGCATGCAGCATGGCACCGCTTATGTTCATTATTCTCAACGG
TACGATTCAAAGCAACCAGAAACACTATTGGTTGTAAAGAGCTTAAACACATAGCAAGACTGACGCGGTATGCATGCCATGAGTGATTGATAATGGTAGT
GTAAATCTATTTCAAATAAAATGTATTTTATTGAAGAATACAGCAAATGATTTGTATTATAACTATTTGACACATTCTGCAAGTTTGTTGACCAACGTGA
GTACATGATAGCTACAAATCTTATATTGACAAAAAGATATGGTGTGTGGAAAAAATTCAGGTGCTATTTTTTTCTATTTTGAGAATTGTATGCATAGCAG
AAGAGCACAAATTGCATTCTGGTTTATCCGTGCAGGCATTCCAGAGTATCAGAATGGGGATCAACCTTTCTTCAGCTACATATGTAAATATACGGATGCT
AGTTGCTCACTTAAAATTTTGTAGTGTTCAAAGGAATAACAACAGTAAGACGTTGACGCACCGAAAATAACCAGAAATAACGGATTGTACTATGACAAGA
CACACGACAGATACACTAGCTTAAGTTTGTACAGGAAAATATTTGGCCTATTCTTATGTCACAGTAAATTAAGATTATCACCTTAATCACAGAAATTCAA
GGGGTAAACGATTATAGCACAAAACGATCCTGGACCCCAAGACCTGGGTCTCTGAACGGCTGAACCTCCAATTATACCTCTATACATTTGGAATGTGTGT
AGTCAAACTTTCGCTACTGGTATCTTTACAAATATTTACATTGAACACATCAAAATGATATACATGGATTCCTCTCGATATGTCTTTGAGGTTTGGGTTC
TAAAAATGATATACAAATTATTAGGTTCTAAACTTAAATGATATATACACATCAAAATGATATACAAATTATTGGGTTCTAAACTTAAATGATGTATACA
CATCGAAATGATATACAAATTATTAGGTTCTAAAAATATACTATGGCAGAATTAGGTGGTCATTTGAGTTTTGGAGGCCTTGTCAATGTCCCAAACATCA
CTTATTTGTGACTGGGAGAGCAATGAATTGAAGGAATGAGAGTAACCATACGGTCATTGGTCAATTAAAAGGAACTTTAATATGCTATGACCACATCGAC
CTTTCGTAAAGTAAATAACTCAATTATAACCAAGTAATTGGGTGTCTATTGAATCCATACGGTTCTCAAAACAGTTCTAAACAAAGTGAATTTAAGCATC
CTGAAAAGATATTTGGCAAATTTGCCATACCCTGAATTCACAGCATGTTAACACAAATCATGTGCAAATCATTCAGATCATTGACAAACGCACGCATGCA
CACAAATCAACACCGTTTTCTCATGTATAGAACAGAAAAATTGAGAAACTTACGATTTTCTTGCAAGTTATGCAGTTGTTATCTCTAGCTTGCCGCCTCC
ACTTGCAGTAACTGAGACGCCGGCGCCGAGCTTGAGCTTGAAACCTAGACTACGGGGACAG
>comp76332_c0_seq24
ACTGCTCTGAATTAAGAGGCGTTGAAATATTGTCCGGTCTACATATAGGCACTTGAGGTGTATTGACAGACAGTTCCCCTTAGTTTGCTACAACACATCA
GGCGAATTCCACCAAATTAGGATGATGTTTGTTTCTTAGACACAGGATAGCAATGCCAAAAGTCTTTTATAATTTTCCGCTTCACCGCAGATTTGGCATC
TCAAAGGGAGTTGTGGCAAGATTCTCTTGACCAAGCTTTCTTGCAGATTTGGCAAGAAAGATAAAAAAAATGTTGGTATACTTAAGCATCCTTAATAGAA
ACTAAACAGGTCTTAGATTGAAATTATCATGAATAAACCCTTTTTCACTTTAGCTGTAGTCCGGATGAAATCTTAGGGGCCTTGCTTAGCCTGCCCTGAA
GCTTGCCAGTAAGTCGAAGCTGATGCTGACACAGCACTGACATAGATCCCAATGAACACAAGTTATTTCGAGAAGATAAAATACCAAGCAAAGCCAGGAT
TGCATTAAAAGATAGTGTATTTCTACTATACGATCTGAAAGCTTAATTTATTATATCAATAATCAAATTGGCAGCAGTGTGGAACAGATATAAAGAAGAT
GCTTCAAATCACAAATAACTACAGATAAATTGAAGGGTGAAGGTTCATTCAACCACCAAACACCCAAAATGTCGAGCTGCATATGCACAGAACCTAAGTA
CGGTACAGTTCATAGTTTGGGAAAATAGACTGCAAATGTGCACAGATAATACAGATTGTTACTCGATGAACAGTAAGCACGGTATTTGAAATCCTTCTAA
CCATCCATCCAGGCAGTGCCTCGTATACTCAAACCTCTTTTTGGAGGAAGCCAAACCATGTGATCCTTTTCAACCCAAATACATATATTCAAGATGAAAC
TAACAAAAAGACAGTTGATAAAGAAACACGAAAGAGAGTAGAAAAAGGGTACCTGTTTCAAGTTCTCATCTTCTGAAAATGATCAGCAGTCAATAGATGT
TTTAGTCCATTCTCCAGAAAAATGAGTGCCGCATGAATCCTTTCCGGTTTATAGTGAAACATTTTAGACGATGACCTTTAACATTCTAGAAAACGAAGAG
AAAACCTACAGTCATCTAATTTCAGCTTAAACATGTTCCAGCAAGATCATCACCATCCTGACATGTTCCGTTTTGTCCCTAGACCCGAGCACAAGGTATT
GCGAGGCTAATTTAGCGGGGCAATTGATTTTTTGCCACTATGATACAACTGCAATTACCCGCTGTCACTGACACGTGGGGTCATCTGAGTCACTGAAATG
TGGGCCCAATAGCATTTTGTTTGCCACTCACCCTTCCAGAGTGGCAAAAAGTTAATTGTCCCTAATTTAGCTCAATTCAGTTGTCAGAGCAATTGTGTGT
GAAAAGTGTGAACAACAGCATAGGAGACAATACAGCAAGTGTAGCGTGCCAATATGTGTCCAGCTTGATAGTTCACTATAATAAAGTTCTTTAATTCAAG

TATTTGAATAATATAGAATCTCAACCTTCAAGTTTAAAAGGTTTTGTATTTGCTATTATAATCGTTGCCACCCCCTCACCCCTCTCAAAAAGAGAGTTAC
TTTGTAGCGCACTGTAAATCTCTTGATTGAAATAGTGGCATGTCATCTTATTTAATTCAGTTATTTCATAAGGTACAATTTGTTTAGCTGACAGCTCAGA
CATGCTTCTATGCAACATGTTTATACCAATGCTGTATTTTGGCAATCCTGGATGTGGCATTACTTAACTGTGCTTTCATATTCAATAGTTCTGGCAAAGG
GAGGCTGCAAGCTTGAGACAACAACTGCACAACCTGCAAGAAAGTCATCGGTATGGCTAGAAAACCCTGCAAATTTTACAAATGTACAAGTCAGAAAATT
TGCCTAATGTAAAAGGATCAAACTACAGGATTTGTCAGCATGAGCACAAGTGCCTAACTTCCGAGTTAGATAACTCTGGAGGAAGGAAATCCTTCCTACT
GTTTGTTTCAAGAATATACAAAAATTCAAAACAACATTAGAATAGAACCAAAAGAAGAAACAATATTCACCATGTAGCAATACTCAATTCCTGGTTGTCT
TATTTATAAAGGTATAAAACTAGGTTCTGACGAGACAATTATGAATCAACTACACCATGTTACAGATCATCCATAACTCTTGGTGATGCAAATTAAGGGC
TGTAAAGTGACAAGTATACCTCCACCAACTAATTTGTGCAGAGTGTATAAAATTGTAAAACAATGGATAAGAATGAATTTAGTATACAAGATAGCATACA
ATGCCCAAGTTTAAGGAGACATACTGATTTACTGACTCCATAGGACAATCTTTTGCTTATGAGGACATCAGTTTTCATAAACAATATCAATGTAAAAGGA
GGTAACAGTTTATGAATTTCCTATGCCAAGCCATTTATAAGTCAAAAAATGTACCTATTTAGCTTTTATGCATTCCAATTAACTTCCAAAGAAGAAGAGT
ACATACTAATGGAAAACAAATATGATATTATCATCTGTTTTGCCCGGTAGTTTCTTGAGTCTCTGATTTGTCCGCACTTCACTCCCAACCAACACAACAT
TAATACACATGGGCAGAGGAATATCAGGTTATCAGGTAAACTGAATACAGTTTGATTGACTGCAGTCAGTTGATGGGACAAGATCTTTCTGGATTGAGCG
TCAAGGAACTCCAAAATCTAGAAAATCAGCTAGAAATGAGCCTACGTTGCATCCGGACAAAAAAGGTAAGGATGCTAGCCATATTTCTGTTTTTCGTTAC
CAGATTGCCTCATCCAAGAATACAACTGATGATGACGTTTTTTACTTTCAGGACCAACTCTTGATTGATGAAATTCACGAACTGAATCGAAAGGTTCTGG
AATATTCTGTTATTACTCTGCAATGAGAGGTCTCTGAGTGGATTTCAAGAAGCTGACTCTACTGGCTCATTTTCAGGGAAGTCTCATGCAACAAGACAAG
ATGGAACTATACAAAAAGGTCAACCTAATCCGTCAGGAAAACATTGATTTATACAAGAAGCTCTACGAGAAAGAGGTGACAAGTGAAGTCAACCGAGATT
CATCAACTCTAAACTTTGCAGTTGTCGAGAACGTCAACATTCCTGTTCATCTTGAACTTAACACTCCACCGCAAGAAAATGATGTTGAGCAAACTGCACC
TCCTAAACTGGGGTAACCCTGCTTTTTTCTGCAAAGGAACTATTGATTCTGTTCTTAATTCTTTAGTGGCATATTGTTTACTACTAACTGAAAACTTGCA
GATTACAACTAAATCCATGAAGGCATGCAGCATGGCACCGCTTATGTTCATTATTCTCAACGGTACGATTCAAAGCAACCAGAAACACTATTGGTTGTAA
AGAGCTTAAACACATAGCAAGACTGACGCGGTATGCATGCCATGAGTGATTGATAATGGTAGTGTAAATCTATTTCAAATAAAATGTATTTTATTGAAGA
ATACAGCAAATGATTTGTATTATAACTATTTGACACATTCTGCAAGTTTGTTGACCAACGTGAGTACATGATAGCTACAAATCTTATATTGACAAAAAGA
TATGGTGTGTGGAAAAAATTCAGGTGCTATTTTTTTCTATTTTGAGAATTGTATGCATAGCAGAAGAGCACAAATTGCATTCTGGTTTATCCGTGCAGGC
ATTCCAGAGTATCAGAATGGGGATCAACCTTTCTTCAGCTACATATGTAAATATACGGATGCTAGTTGCTCACTTAAAATTTTGTAGTGTTCAAAGGAAT
AACAACAGTAAGACGTTGACGCACCGAAAATAACCAGAAATAACGGATTGTACTATGACAAGACACACGACAGATACACTAGCTTAAGTTTGTACAGGAA
AATATTTGGCCTATTCTTATGTCACAGTAAATTAAGATTATCACCTTAATCACAGAAATTCAAGGGGTAAACGATTATAGCACAAAACGATCCTGGACCC
CAAGACCTGGGTCTCTGAACGGCTGAACCTCCAATTATACCTCTATACATTTGGAATGTGTGTAGTCAAACTTTCGCTACTGGTATCTTTACAAATATTT
ACATTGAACACATCAAAATGATATACATGGATTCCTCTCGATATGTCTTTGAGGTTTGGGTTCTAAAAATGATATACAAATTATTAGGTTCTAAACTTAA
ATGATATATACACATCAAAATGATATACAAATTATTGGGTTCTAAACTTAAATGATGTATACACATCG
>Locus_18892_Transcript_33/56_Confidence_0.309_Length_1342
TGGTGGCTGCGCCGTCTCCTGCGGCTCTATCTCCCGCCGCCGCCGGGAAGAAGAGCCGGCGGAGGCCGAGGTCCCGGAGCTCGCAGTACAGGGGCGTCAC
CTTCTACAGGAGGACGGGCCGGTGGGAGTCGCACATCTGGGATTGTGGGAAGCAAGTCTACTTAGGTGGTTTCGACACTGCTCACGCGGCTGCGAGGGCT
TATGATCGTGCAGCGATCAAGTTCCGAGGGCTCGACGCGGACATCAACTTCACTTTGAGCGACTATGAGGATGACTTGAAGCAGATGAGCAATTGGACAA
AGGAAGAGTTCGTGCACATACTCCGCCGCCAGAGCACGGGGTTCGCGAGGGGGAGCTCCAAGTACCGCGGCGTGACGCTGCACAAGTGCGGCCGCTGGGA
GGCGAGGATGGGTCAACTTCTTGGCAAGAAGTACATCTATCTTGGACTCTTTGACAGCGAAGTTGAAGCTGCAAGAGCATATGACAGGGCAGCCCTTCGC
TTCAATGGGGGGGAAGCTGTTACTAATTTTGGGCCTAGCTCCTACAATGGAGGAGATGCTCTACCCGACACCGAAAATGAGGCAATTGTTGATGGCGATG
CGGTTGATTTGGATTTGCGGATTTCACAACCTAATGTGCACGACACTAAAAGGGACAATACCTTAGCTGGAGGCCAGCTAACATGCGACTCCCTTGAATC
TTCAAACACGATGGCCTCTCAGCCAATGAGCTCATCGTACCCGTGGCCTGTGTATCACCAAAGCACGACAGCAGTACCACCTCACCATCAACGCTTGTAC
TCATCTGCTTGTCCTGGCTTCTTTTCGAACCTCCAGGAAAGGCCAATGGAGCGAAGGCCTGAGTTGGGTACCCAGTCATTCCCCACCTGGGGATGGCAAA
TGCAGGGCTCCCCTCACATGCCGTTGCATCACTCTGCAGCATCATCAGGATTCTCTACCGCCGTCGGCGCAAACGCCGGCGTCTCGTTGCCTTCTCACCC
GCCGGTGCCGTTCCCGGACCACCAGTTCTACTTCCCACCGACGGCATGAGCTTGTTGGTCTGGTTCAACTCTTGGGACGGCAGCTGATCTTATGTGTGAA
CATTTCGCAGCTTGCCGGTGACCGTTGTTAATTAATCGGGGAGAGAGAGCCAGAAGACCCAGTATGGCTATCTCTCTGTGGCCCGTGCCTTGTTATGATC
TTAGTTATTGCTACTACACCGTGGATTCATCATGGTTTATGAGATTCTTTACTCAAGTTTCAGCTTAAATGTCCAAATACATGAATGCATCATACACAGG
ATCCGTCATTTTCTAAAAAAATACAGGATCTGTGTGAAGTGT
>Locus_17579_Transcript_44/92_Confidence_0.265_Length_2051
GAGGAATCTCTTGTGGATTCCTTTTTCTGGGTGCAACGAAGTGGGAGGTACTTGGTCTTCGCACCTTGATGTACTTGTGGGTGCGTGTGTGCGCGCGCGC
GCGCGCCTTTCTTGATGCTAGAATGATGGTGGTGCTGTTCTTGCCATGATTGACGATTCCCCTAGAAAAAGTTGAACTACTCTGCACTCTTTCTAGCACA
TGGATAGCACCTTGTGTACTTCTCTGATGATGTGTAGTTGGGTGCTCAGATATGTTGTTGCAAATCTGTCCAATTTCTGCTACTCTCATATGTCAAATCT
TTTCTGTTTTTACTCTGTTTCACTCTTCCACTTTGTTGCTTCAAAGGTAGGAGCAGCAGTTGAGATTTTCAATTCTGGCATGGGTTCTTTTGGGATGGAC
TGGAACCAGAAGGGCTCCGTGTTGTGGGATTGGGAGAATTTGCCTCCGATAGGCGGAAATGCATACGAGAACCTCAAGGTTGCACCGCAGGCTGAACCGA
AGGTTGCAGGTGTTGAGGGCACAAGGCATGAATCAGTGCATTCTTCCTGTGGTACTTTCTCTTCCAGCTCGGAGATGGGGTATGGTTCATCCAAGACTTC
CATGTCCGCGTCAACTGATTCTTCGCCCAAGGTGGGAAACAACATGGAGCTCAATTTTGCAGCTGTCAAAGTGCCTGACAAGAACACCGGCAAGAATACT
GATTTGGGTAAAGTTGATGACGCCGGAACTTCTCCGTCATCCGTGATAGCCGGCAGCAGTGGAGAACCGGTGATTGGCTTGAAACTTGGCAAGAGAACCT
ATTTTGAAGATGTATGTGGAGGGCAGAATGCCAAGAGTTCACCGTCAGATTCGACTACAGTGACTCCTGCTGCTTCTGCGAAGAAGGCAAAGATGGCTCA
AAACACACAGAACTCATACTGTCAGGTTGAAGGTTGCAAGGTTGATCTCTCTTCTGCTAAAGATTACCATCAGAAGCACAGAGTCTGTGAAGCTCATTCT
AAGGCTCCCAAGGTTATGGTTGCTGGTCTGGAGCGACGCTTTTGTCAACAGTGTAGCCGGTTTCATGCTTTAACCGAGTTCGATCAGAAAAAACGAAGCT
GCCGGAGGCGCCTCAATGATCACAATACCCGCAGACGGAAGCCACAGCCTGAAGCAATTCCTTTCGGTTCATCAAGGCTCTCTGCAATGTTTTATGATGC
AAGGCAACAGACAAGTCTTCTGTTTGGTCAAGCTCCTTATGGTCAAATGAGAAGCTGTGCAAACTCTTCATGGGATAGCCCAGTAGGAGGCTTCAAATTT
GGAGAAACAAAAGCTCCTTGGTTAAAGCCAACGAGAGCTGCAGGTGTTGATGGGATGCATGTATCAAGCCAGCAGGTGTGGAACAATATTACGCCACACG
GTGCACATCATGATTTTAATGGTATCATGGCTTTCAAGGGAACCAGTGCAAATGTCCTTAATCAAGGTGCCGAAGCTTCTCCGGTCGTCTCCAACTCGAA
TGGAGCCCCAGATCTTCAGCGTGCTCTCTCTCTTCTGTCAAACAATTCAGCTGGTGCTGCCAACGACCAGCCAACTCCTCAGCTGCACCCTGGGCTGACC
ACCTTCGTCGGCTCCTCCAACCCTGTCACTGTGATGGAAGCCTCACCACCAGGACTCTGGCAAGATGGCGCCCCCCTCGATCATCAGGCGCGGTTTCAGC
TTTTCGATCCGGTGGGTGACGGCAGCGCCATTGTCACACCGCATCAGCTCCAGCTCCCAAAACCTCACTCCCTCAACAGCTCCTCTTCCCACTATGACCA
GATGCACTGATGATCTGATGCTTCCTTCAGTTAGCGCAAATGGCGGTTGGGTTGCGCAAAATTAGACATGGCTTCTGATGCCTGAAACTCTGAATTTTGA
AACTGTTATGTTCTTCTGAAAAATAAAAGCCTGTGTGCGCAAACTCAGGCTCCAAAAACTGATCTAGTAGTGTTCCCATTCAAGGGCTTGAATAATCCAT
CCTTGTGGTGGCATGTAACTCAAATGCTTCGATTGCGCCGGCAATTGATCT
>Locus_839_Transcript_102/261_Confidence_0.054_Length_1180
CAGTGCAATTAGACTAAGTATAGTTGGTGAGAAGACAGGAGTAGAGATAAGGGCCAACAGCCCAACAGAAAAACCAAGCAAGACTGCAACTGCAAGAGAG
ATGGGGAGAGAGAGGAAACCTTGCAGAGGAGAGAAGCAAGGTGGGAAAGAGGCAGCAAGCGAGGCGGGCTGCAGGAAGGGGAACATGCTCCCTCCTCATC
TCACAGAAAATGGCACAATAATGATTCAATTTGGTCATCAAATGCCTGATTGCGACTCATCAACTACCCAATCAACCAGCGAGAGTCATCAAGAAGTGTC
TGGAATGAGTGAAGGAAGCCTCAACGAGCATAACGATCGATCAGGTAATCTCGATGGTTACACAAAGAGTGATGAAAATAAGATGATGTCAGCTTTATCT
CTGGGCAATCCAGAAACTACTTATGCGCATCCAAAACCTGACCGTAGTCAGCCCTTTGCCATTTCGTACCCGTATTGTGATTCATTCTATGGTGGTGCAG
TGGCAACGTATGGCTCACATGCTATTATGAATCCCCAGATTGTGGGCATGATGTCGTCCTCCCGAGTGCCATTACCAATTGAACCAGCTGCAGAAGAGCC
CATTTATGTGAATGCAAAACAATACCATGCGATTATCCGAAGGAGACAGCTCCGTGCAAAGCTAGAGGCTGAAAACAAGCTGGTGAAAAGTCGCAAGCCA
TACCTTCACGAGTCTCGGCATCTGCATGCCATGAAGAGAGCTCGGGGAACAGGCGGGCGGTTTCTGAACACGAAGCAGCAGTCAGAGGCTCCAGGTGGTG
GCACCTCAGATGCGCAACACATGGCCGGCAATGGTGGTCTGTTCAGGCAGCACGAGCACATCTTACCACCCAGTGATCTCCATTATCGCGCGAGAGGGGG
CTCTTAAGGAGACCCATGGCAACTCATCCTTGGCTTAGTAACGTGTGGCGCTCAGCGATTCGACCTCGGCTTCGATGTAATGGTGTACGATTGTATCTAT
GGTGCTTGAATTGGCAGCAGCCATTCCTGCAGCAATGCGTCGCAGGTGTTGCCGGTTCTGTGATAGTGCCAGACTTGTGACCGGCACAGAACTGGCATCT
GTTTCCTGTAACTTTAGTTTTTCTAAGACGATGACGATGACTACCCAATAACCATTCTGTCTGTATGGTGCCTTGCGTTT

>comp79373_c0_seq40
CTGTCCTGCCCAGCAGCGCACAAGCAAGCTCGCAGCAGCGCCATTGCACGCTTCCGGTCGGCCGCTGCTGCGCTGCACGTCTGTCCTCCCGCCCCGTACC
GCCTCGTACACATGTAAACTGCTGTGCTGTAGGTTTGGAGCCCCCGTACAAGCGTGTTCGAGTTTGCTGATGTTCATCTAATTATCCCCGGGGAAGGAGT
GATTGTCCGCGGAGAGAAGGAAGAGATCGAATTGAAGGTCAAGATAAGTTATTGAAACGGCAAGATGGTTAATTTTGGAAAGAAGTTGATGACAGATCAA
GTAGAAGAATGGAAAGGGTACTATATCAATTACAAACTGATGAAGAAAATGTTAAAGCAATATGTTCAACAGACCCAATTAGGCGGGAAAGATTGTGAAC
AAATCCTTAAAGAGTTCTCGAGGATTCTTGATGACCAGATTGAAAGGATTGTGCTTTTTCTGCTACAACAACAAGGCCACCTTGCCAGTAGGATTGAGGA
ACTGGGGGAACGACGTGCTCTTCTGAAGCAGTATGATATATCACAAGTTTCTCAGCTACGCGATGCATATAGAGAAGTTGGGCTTGATCTTATAAAGCTT
CTCCGCTTTGTTGACATGAATGCTACCGGTATACACAAGATATTAAAGAAATTTGATAAGCGCTTTGCCTATAAGTTTACAGACTATTATGTCACCACTC
GTGCAAACCATCCTTATTCTCAGCTTCAGCAAGTATTTAAGCAAGTGGGAACCCTAGCTATTACAGGTGCCTTGTCCTGCAACCTTGCATATCTGGAGGA
TCATCAAGGAGGCATTTATCCATCTATGACCACCCATCAATTAACTTCAGGGACCCTATAATTAACCAAGTAAATCATGCAGTGCAGAAACTCACACAT
GCGACAAACTTTATGCAATTCTTAGGACAACATGCAGTTATCATCCCAGAAGATGTGCAAAGCGGGTCAGAGGATCTTGTCAATGATCAGAACTACCATT
TCATGTCTCTTTTGCTTAACCTACTGAACACGTTTCTTTACATGGTGA
>Locus_18892_Transcript_39/56_Confidence_0.235_Length_1428
TGGTGGCTGCGCCGTCTCCTGCGGCTCTATCTCCCGCCGCCGCCGGGAAGAAGAGCCGGCGGAGGCCGAGGTCCCGGAGCTCGCAGTACAGGGGCGTCAC
CTTCTACAGGAGGACGGGCCGGTGGGAGTCGCACATCTGGGATTGTGGGAAGCAAGTCTACTTAGGTGGTTTCGACACTGCTCACGCGGCTGCGAGGGCT
TATGATCGTGCAGCGATCAAGTTCCGAGGGCTCGACGCGGACATCAACTTCACTTTGAGCGACTATGAGGATGACTTGAAGCAGATGAGCAATTGGACAA
AGGAAGAGTTCGTGCACATACTCCGCCGCCAGAGCACGGGGTTCGCGAGGGGGAGCTCCAAGTACCGCGGCGTGACGCTGCACAAGTGCGGCCGCTGGGA
GGCGAGGATGGGTCAACTTCTTGGCAAGAAGTACATCTATCTTGGACTCTTTGACAGCGAAGTTGAAGCTGCAAGAGCATATGACAGGGCAGCCCTTCGC
TTCAATGGGGGGGAAGCTGTTACTAATTTTGGGCCTAGCTCCTACAATGGAGGAGATGCTCTACCCGACACCGAAAATGAGGGTATCACAACCAGCCTCG
CCATTTATTTCTTTTCATATAAGCAAATGTATCTCAGGTGCACCTTATAATCATCGCTGTGTTCTTAGCAACTGTTGATGGTGACGCGGTTGATTTGGAT
TTGCGGATTTCACAACCTAATGTGCACGACACTAAAAGGGACAATACCTTAGCTGGAGGCCAGCTAACATGCGACTCCCTTGAATCTTCAAACACGATGG
CCTCTCAGCCAATGAGCTCATCGTACCCGTGGCCTGTGTATCACCAAAGCACGACAGCAGTACCACCTCACCATCAACGCTTGTACTCATCTGCTTGTCC
TGGCTTCTTTTCGAACCTCCAGGAAAGGCCAATGGAGCGAAGGCCTGAGTTGGGTACCCAGTCATTCCCCACCTGGGGATGGCAAATGCAGGGCTCCCCT
CACATGCCGTTGCATCACTCTGCAGCATCATCAGGATTCTCTACCGCCGTCGGCGCAAACGCCGGCGTCTCGTTGCCTTCTCACCCGCCGGTGCCGTTCC
CGGACCACCAGTTCTACTTCCCACCGACGGCATGAGCTTGTTGGTCTGGTTCAACTCTTGGGACGGCAGCTGATCTTATGTGTGAACATTTCGCAGCTTG
CCGGTGACCGTTGTTAATTAATCGGGGAGAGAGAGCCAGAAGACCCAGTATGGCTATCTCTCTGTGGCCCGTGCCTTGTTATGATCTTAGTTATTGCTAC
TACACCGTGGATTCATCATGGTTTATGAGATTCTTTACTCAAGTTTCAGCTTAAATGTCCAAATACATGAATGCATCATACACAGGATCCGTCATTTTCT
AAAAAAATACAGGATCTGTGTGAAGTGT
>Locus_11912_Transcript_21/63_Confidence_0.322_Length_2924
TGGCGCCTCCCGCCGGGCGCTCGCCCCGGATTTCTCGGCTCCCCATCCGGCGGACGCCTTGACCGGGGTGGGTTCTTGGTTCCGGCGGCTCGGTGATCTG
TGCGTGCGAAAGGCGGGGCTTTTAGCTCGTTCTGTGGGGGGAGGAGGAAGACGGCGGCGAGGCGTTCATGGGTTAGATCTGGCGGTCCGGCGCCGCGAAT
CTGAGCGAGGTTTCGGTGGAGGGATCGGGGCGGAGAAGTCGCAGTCTCTGGCTCGTTCTGAGCAGCTTGGGCCTCTCATGTCCAAATCGGGTATGGATGA
TGGGTGCGGTAACTGTCGGAAGGAGCGTCGCTTGCAGATCTGAAAGCCATCCAGCTCCCTTGTTCGGTTGACCTGGAATGGCCGTGCTTTCTGCTGTTTG
ACTGTCAGTAGTTGCTGCTTAGTGTGTGTGCAAGAAATTTAGCTGGAGGTGATGTGCTTAGGAAACTAGTCTAGTTGCTGATATCTTGGTCTCTGTTGAA
GAACGCTGAAGCTGGAAGGAAGTAGGTCGCATTCCTGTGTTGTTGATATTTTGTTGTTTCTCGAATAACCTGGAGGCTGGAAGGGATCAGATCCAGTTCC
TGTGGCACCGGTAACAAGGTGCTGTTGGGGGAAGGCACCATGACCTACTTCCCTGAGGAGGTGGTGGAGCACATCTTCAGCTTCTTGCCATCGCACAGCG
ACCGGAACACGGTGTCCCTGGTCTGCAAGGTGTGGTATGAGGTCGAGAGGCTGAGCCGGCGAGCTGTCTTCGTGGGGAACTGCTACGCTGTGCGCCCGGA
GCGGGTGGTGCTGCGGTTCCCCAATGTGAAGGCCCTGACGGTGAAGGGGAAGCCGCACTTTGCGGACTTCAACCTGGTGCCGCCCGATTGGGGTGGCTAC
GCGGGGCCGTGGATTGAGGCGGCAGCGAGGGGCTGCGTGGGTCTGGAGGAGCTGCGGATGAAGCGGATGGTGGTGTCGGATGAGAGCCTTGAGCTGCTAG
CTCGGTCCTTCCCGAGATTCAAGGTCCTCGTCCTCATCAGTTGCGAGGGGTTTAGCACCGATGGGCTAGCAGCTATTGCGAGTCACTGCAAGCTCCTGAG
GGAGTTAGATTTGCAGGAAAATGATGTGGAGGACCGTGGGCCTAGGTGGCTCTCCTGCTTCCCTGATTCCTGCACCTCTCTTGTCTCCTTGAATTTTGCC
TGCATCAAAGGGGAGGTGAATCCTGGTTCATTGGAGAGACTTGTTTCTAGGTCCCCGAACCTACGCAGTTTGAGGTTGAATCGATCTGTATCAGTAGATA
CACTCTCAAAGATATTAGTACGCACCCCTAATTTGGAAGACCTGGGGACAGGGAATTTGACGGATGAGTTCCAAACTGAATCCTACTTCAGGCTGACTAA
TGCTTTGGAGAAATGCAAAATGTTGAAGAGTTTATCAGGATTTTGGGATGCTTCTCCTGTTTGCGTTCCATTTATCTATCCCCTCTGTCATCAACTAACT
GGACTAAACTTGAGCTATACTCCCACTCTGGATGCTTCTGATCTCACTAAAATGATCAGCCGCTGTGTGAAACTCCAACGTCTTTGGGTACTGGACTGCA
TCTCAGATAAGGGTTTGCAAGTGGTGGCCTCCAGTTGCAAAGATCTACAAGAACTCAGGGTGTTCCCATCAGATTTTTATGTTGCTGGTTATTCTGCCGT
GACAGAAGAGGGGCTTGTTGCAATATCGTCAGGCTGTCCAAAATTGAGCTCTTTGCTGTATTTCTGTCACCAGATGACCAACGATGCACTTATTACTGTA
GCTAAGAACTGCCCAAATTTCGTAAGATTTAGACTCTGTATTCTTGAGCCAAAGAAGCCAGACGCCATGACAAACCAGCCGTTAGATGAAGGCTTTGGTG
CAATTGTTCGTGAATGCAAGGGGCTAAGGCGACTGTCAATGTCGGGTCTTCTCACTGACAAGGTTTTCATGTATATTGGAAAGTATGCCAAGCAACTTGA
GATGCTTTCTATAGCATTTGCTGGAGATAGTGATAAGGGGATGATGCATGTTATGAATGGATGCAAGAATCTAAGGAAGCTGGAGATTAGAGACAGTCCA
TTTGGTGATGTTGCGCTCTTGGGGAATGTTGCCAAGTATGAAACAATGCGATCCCTTTGGATGTCATCATGCAATGTCACATTAAAGGGGTGCCAAGTCC
TTGCGTCTAAGATGCCGATGCTCAATGTGGAGATCATGAATGAGCTAGATGGAAGCAGTGAAATGGAGGAAAACCATGGAGATCAATCTAAGGTGGAGAA
GCTATATGTTTACCGCACAACTGCTGGAGCGAGGGATGATGCACCAAATTTTTGTTAAAATCCTATAGTTAGCTGAGAGTAGCCTGGTATCTCCTCACTCT
AAAAGATGGTCGGTGGAATCCGGCAGGCTGGGGGGGCCGCGGCAACAGTGGTAAGTTCTTGAGGTGCCTCTCTCTCCCTTTGCAGGAGTCAGGAAAAGCC
ACACCGGTGTGCGCCCTTATCTTGGAGCAACTGGCCGGGATCCGTCATCATCCTCTGTACCACCGCCCCTACCCTTTTAGTGTTTGGTCACTCATATGGC
CACTGAGGCCCTTTATCATCCTGTGCATTCTCACTCGAGAGGACAACGTTCTTTTTCTCCCTTTTATCCCAATTGTAATCGTAGTGTACCAGTACCCATT
TGCTTTTGACATTTGCACAGTAGTGGATTGTGTTACCGCCGCCGACGCTGCTGCTGTAATAGTGTTTGTTTCGGCCTGAATGATCGGGACATGACATTCC
GGGGACGACCATTGGTTTCTTGCTCCTGTGAATGCATTGCATGGAATAATTTGAGGTTCACCTGTGACATTCTTCTATTATTTGGTTTTGAATGATTGGT
GCCCTGGCGAACGTTTTTGACTGT
>comp82796_c1_seq19
CGATTCATTGGTCTTTTATATAGGTTGAGGAACCGGGAGGCGTAGGATAGAGAGATCTCAAGGTCAGGTGAGAGCTCAGTGTAGGTCAATGGCGTCCGTG
CCGTCGCTGGTGCTTCAGTTGCCAGTCAATTGATGCACCGTGGTTACTGGTTCAGCTCGCCGAGCAGGTGGATTTCAAGGTGTGTACGGTGTTGAGGATT
GGGAACGAGATTGTCAGGAAGAGGCAGAGGAATCCGTCGTGTTCTTTCCCCCCAAAAGAATCCGCAGTGTCCTCTTTCTCTTTTTTTTTGTTGGCATGAA
AGGGATTTTAGGTCCTTCATTTTCCCCTTGTTCATTGTTGCAACGAAGACTTTTATTCTGTTCTTGCTCCGAATTCGTCAGTAAATTTTTAACTTTTAAA
TCCGAAAGGAGAAGATGTGATCTATCTATTCTGTTCCAATTTGGCATTTGTATGTGACTTCTGTTCTTTCTTCTTGTTCTTGCAGTGCTAGCAGTTTTTA
CCTGTTCGGGCAGGAGCAATAGTGGAGATCTTTAGTTCTGGCATGGGTTCCTTTGGGATGAACTGGAATCAAAAGAACTCCATGGTGTGGGATTGGGAAA
ATTTAGCACCGTCTGTTCCAAATGCAATCGGAAACCCCAGGAACGGAGCTACGGGGCATGGATCTGTGAATTCATCTGGTGGCACTCTCACTTCTAGCTC
AGAGCTAGGGCATGGTTCATCCAAGAGCTCCATGTCGGCATCCATTGATTCACCGTCCAAAGTAGGGAACAGCTTGGAGTTCAATTTTGCTGCTGTCAAT
ACGTATGGTAAGAACATGGATAAGGATTGTAGAGTTGACGACTCAGGGACTTGTCCATCGTCCATGATAGCATTCAGCCATGGAGAGCCATTAATCAGTC
TTAAGCTTGGAAAAAGGACTTACTTTGAAAATGTCTGCGGAGGACAGGATTCCAAGAGCACTGCACCTTCTACCAGGACTTCTCCATCAACCGTTCTCAA
GAAGACCAAGGTGTCTCAGCAGAATACACAAATGTCATACTGTCAGGTTGAAGGTTGCGGAGTTGATCTATCTTCTGCTAAAGATTACCATCGCAAGCAC
AAAGTCTGCGAAGCTCATTCTAAAGCTCCGAAGGTGGTTGTTGCTGGTGTACAGCGCCGTTTTTGCCAGCAGTGTAGCCGGTTTCATGGCTTAGCCGAGT
TTGACCAGAACAAACGAAGCTGCCGTAGACGTCTTACTCATCATAACGCACGAAGGAGGAAACCACAGGCGGATACAATTTCATTCAGTTCATCACGTCT
CTCAACAATGTTTTATGATACAAGCCAGCGGACAAATCTTTTCTGTAGTCAGCCTCTTTTTGGCCAAGTGAGAAGCAATGCAGTTTCTTCATGGGATAAC
TTGGGAAGCTTCAATTTTATGGAAACAAAACATCTGTTGATGCAGCCAACGAAAATGTTAGGTCTTGATGGGCTGCATTTCTCAACCCCCCAGATATCAA
ATAGTGTTGTGGCTCACTCTGTACATCATCATAATTTTGATGGGTTCACGCCATTCAAGGGAACCAACACAAAGGTCCTCAACCAAGGTGTCGAAGCTTC
TGCGATCGCTTCCAACTCGAGTGGAGACCCGGATCTTGGGTGTGCTCTCTCTCTTCTGTCAAACGGTTCGTGGGGTTCAACTTCAGCCGTCATCCAACAG
CCTAGTTCTCATGCGCAAGCTGGTGCGGTGCCACCCTTCGCCACCGTCGCCGTCTCCAACCCTGCAATGCATCCTCTGGACTCATCCCCGGGAGGATTCT

GGCAAGACGTCCCTCCCCCGCTCGATGAAACTCCACATATTCAGGCATTCACGCACCTCTGATCAAATGCTGCATCCCCTGGGAACAGCAGGTGGTTATA
CCTTCCGTAATCGTCCTAGGTTTGCTTTGCCAGTGACTGGCTCTGCACAATGTCGATCCATCACCGGAGGCATTTGGTAAGGTGAAGACTAAAGACTCAA
GAATAACCGGAGACTAACTCGCTTCTCTTCCAGGCTCAAGACTGTTTGGGTCCAGCGGTGTTGCTACCTGATCCTGTTTTCGGTTCTGAGTACATCCATT
ACTAATCATGGGATTACTTGTTTAGGCCAACCAGACATATTCTGGGGTGAGATTACGACATGTTAATTTACATTGACGGTGTTTCTACATGTGTTAGTAC
TAATGATGTTATCGCTCTTGGCCCCGTTACGTTTCAAGATCTGTTACCATTTGAGT
>comp82180_c0_seq21
AATATAGGGGGCCCCGCTCCCGCAGCTCGCAGTACCGCGGCGTCACCTTCTACCGCCGCACCGGCCGCTGGGAGTCCCATATTTGGGATTGCGGCAAGCA
AGTGTACTTAGGTGGATTTGACACTGCTCATACTGCTGCAAGGGCGTACGATCGAGCTGCCATCAAGTTCCGTGGCTTCGACGCCGACATAAACTTCAAT
CTCAGTGACTATGAGGATGACATGAAGCAGATGAAGAGCCTGTCCAAGGAGGAGTTCGTGCACGTCCTGCGACGGCAGAGCACCGGGTTCTCGCGTGGCA
GCTCCAAGTACAGAGGCGTCACCCTGCACAAGTGCGGCCGGTGGGAGGCTCGCATGGGACAGTTCCTCGGCAAGAAGTACATATATCTTGGGCTATTCGA
CAGCGAAGTAGAGGCTGCAAGGTTGCTGATCTTGGATTCTGATATTGATGCCAAAAAAAAAGTTTTTTTATACCTTTCCTGTTGATTTTCCAAAGGGTTG
AGGACCGACTCGATTCTCTTCTTTCTTGTTTTCCTTCTTTTGGCTTCAAACCTCAGGGCTTATGATAAGGCCGCGATCAAATGCAATGGTCGAGAAGCTG
TGACGAACTTCGAGCCAAGCACGTATGATGGGGAGCTGCTGACTGAAGTTGCTGATGAAGGTGCTGATGTTGATCTGAACTTGAGCATATCTCAACCAGC
TTCTCAGAGCCCGAAAAGGGACAAGAACTCCCTTGGTCTGCAACTCCACCATGGATCGTTTGAAGGCTACGAATTGAAAAGAACAAAGATTGATGCTCCC
TCTGAACTCACGGGCCGACCTCATCGGTTCCCTCTTCTGACCAAGCATCCACCAATCTGGCATGCCCAATCTCATCCCTTCTATTCAAATAATGAGGTTT
GATGATACTAAATTACTAAAATACAGGCAATTACTTTCCCAAACATATTGCTTTGGCTCCTAAACCTCACAATTGCAAGCATGTTCTGAGCATCACATGG
TTCGGATCGAGCTGTCCGGCACCAATTTTGAACGTAATCTTGACCATCTGAGGAACTGATTGTGCAGCCTGAAGGCGTGCGTTCAAATGTTAGATTTGTG
TTACTTTCGTTCATTTTCACTTCTTGTGCCGCTGTCAATTTTGGCTCTAGTCTTACATCACCGCTTCATTTCCTTGGATTCTATAGCCCGCCTGTTTCCT
GTCGATTTCGGAGATATTAATGCGTAGCACAGGTTCATATGTAGGATCTTGAGTTTTCTGAGATGGCCCATTCTGGAAACAGTAATAGTCGCCTTAGGA
ATAGAGATGTGTTTGATTTTTTCCTTCAAAAGGAACATCCTAGAGAGGTGACTATTCGACATTAGGTATGCTAATCCATCGGTCAACTATACCTGCCTG
CGGCTAAGCACCTTTCAATGTTTCTTTTTTCATTCATCGTATCATATAAAAGTAGTAAGTATGACTATTATTCAAACCTTTCTTGAAAAAAATAGTTAGG
ATGTTGCCATCTTGTAATGCAATCATAGAATTGGAAGTGCTGAATTTGCCATTTGTTTCAAACAAGCATTGTATATTGGTTTATCATTCAACTTGAGAGT
TTGGTCAAATTGAACTCTCGAACTTTTCAACTCCTAAACTTTAAGGTCAGGCTCACATATGTCATTTTCAAATTTTATTGGACGGAAAGTTGAGCTGGAC
GTTTCGAGCTCGGCTTTCTATCCAACTGAACTAATGGAAACACTGATTTCATCGCAACAAATTTTCAGCAATAGATATTCAATCTCTTCTTGAATTCATC
CTTCATCAGTATGGTGAATAAATTGTTCTTGGATTATGCAGAATGCATCAAGAGATCTTAACAGGAGGCCAGATCAGGGGGGTGTTCCCAGCTGGGCGTG
GAAGGTGACCGCCCCTCCTCCCACGCAGCCATTGCCGCTGTTCTCGTCGGCGTCATCATCCGCTGCAGCATCATCAGGATTCTCCAATACCGCCACGACA
GCTGCCCACACCGCCCCATCGGCCTCCCTCCGGTTCGACCCGCCGCCGCCGCCGTCCAGCCGCCACCGCTGAACGTTAAGAAGCCACGCTGTAAATTTGC
CAGGAAGCCGACATTTTTTCCTCTCTCGGCGTTGCAACTTTTAGGTTTTGCGCCGGGGTGGTTTCTTGTAGTGGAGTGGATTCATAATTAACTGTATTT
GCATGCTGCCCATGTGAAATGCTTCTCTCTCTTTTTACGCTCTCTGCACCGGATGTTCTGGGAGTTCTTGTTGCCGGGAATTAACTAAATTCACCGTCTG
AGATTGATCTATACATTGTTGTAGAGAATCGAATCATTGTTGAAATTAGTTACAGAATTATAGATATCATAAGGCACCCTCTACTTGTGGACAAAGTTCA
GGAAGATTATTCCGGTTCTTTTCTTGGCTGTCAATTCGGCTCTTGTCCTTGGTGTGGAGCCACTTGCCTACTGTCTTGTTCTGTTGCTGTTTGTTCTTGG
CTTCTTGCACAGTTACAAATTTGTTGGCTCGAATGGATGGTGAGACATTTGGTGCAA
>Locus_5523_Transcript_49/107_Confidence_0.093_Length_633
ACCTCAAGGCTGCAATACCAATCTCAAAAGGGGATAGGATAGGAAAGCTTCATTCCTGCAACAAGGAGGATCAGAAAGAGAGATAGGGAGGCACCAAATC
GGGCTCCTCCAATTAGTCAGAGGAGAGAGAGAGAGAGATCTGTGCTAGCTGTGGGGGAGGAGATGGGGAGAGGGAAGATAGTGATCCAGAGGATCGACAACA
CGACGAGCCGGCAGGTGACGTTCTCCAAGCGGCGGAGCGGGCTGTTCAAGAAGGCCAAGGAGCTCGCCATCCTCTGCGACGCCAACATCGGGCTCATCGT
CTTCTCCAGCACCGGTCGCCTCTATGACTTCTCCAACACCAGCATGAAGTCTGTGATCGAAAGATACAATGAAGCAAAAGAGGATCATCACCTGACCATG
AGCGCAAGTGCGGAGGCTAAGTTTTGGCAAAGGGAGGCAGGAAGCTTGAGGCAGCAACTGCATAACTTGCAAAGAGCACCATCGGTCAGTTGTTGGGTCA
GCAGCTTTCTGGCCTGGATGTAAAAGGTTTGCAAGATTTAGAGAATAAGCTGGAGATGAGCCTAAGAAATATTCGTCTGAAGAAGGACCAACTTATGATT
GATCAAATTCAAGAATTAAACAGGAAGGGAAGC
>comp82180_c0_seq9
AATATAGGGGGCCCCGCTCCCGCAGCTCGCAGTACCGCGGCGTCACCTTCTACCGCCGCACCGGCCGCTGGGAGTCCCATATTTGGGATTGCGGCAAGCA
AGTGTACTTAGGTGGATTTGACACTGCTCATACTGCTGCAAGGGCGTACGATCGAGCTGCCATCAAGTTCCGTGGCTTCGACGCCGACATAAACTTCAAT
CTCAGTGACTATGAGGATGACATGAAGCAGATGAAGAGCCTGTCCAAGGAGGAGTTCGTGCACGTCCTGCGACGGCAGAGCACCGGGTTCTCGCGTGGCA
GCTCCAAGTACAGAGGCGTCACCCTGCACAAGTGCGGCCGGTGGGAGGCTCGCATGGGACAGTTCCTCGGCAAGAAGTACATATATCTTGGGCTATTCGA
CAGCGAAGTAGAGGCTGCAAGGGCTTATGATAAGGCCGCGATCAAATGCAATGGTCGAGAAGCTGTGACGAACTTCGAGCCAAGCACGTATGATGGGGAG
CTGCTGACTGAAGTTGCTGATGAAGGTGCTGATGTTGATCTGAACTTGAGCATATCTCAACCAGCTTCTCAGAGCCCGAAAAGGGACAAGAACTCCCTTG
GTCTGCAACTCCACCATGGATCGTTTGAAGGCTACGAATTGAAAAGAACAAAGATTGATGCTCCCTCTGAACTGGCGGGCCGCCCTCATCGATTCCCTCT
TCTGACCGAGCATCCACCAATCTGGACTGCCCAGTCTCACCCCTTCTATTCAAATGATGAGGTTAAGTGATACTAAATTACTAAAATACATGCAATAACT
TTCTCAAATATATTGCTTTAGCTCCCTAAACCTCACAATTGCAAGCATGTTCTGAGCATCACATGGTTCGGATCGAGCTGTCCGGCACCAATTTTGAACG
TAATCTTGACCATCTGAGGAACTGATTGTGCAGCCTGAAGGCGTGCGTTCAAATGTTAGATTTGTGTTACTTTCGTTCATTTTCACTTCTTGTGCCGCTG
TCAATTTTGGCTCTAGTCTTACATCACCGCTTCATTTCCTTGGATTCTATAGCCCGCCTGTTTCCTGTCGATTTCGGAGATATTAATGCGTAGCACAGGT
TTCATATGTAGGATCTTGAGTTTTCTGAGATGGCCCATTCTGGAAACAGTAATAGTCGCCTTAGGAATAGAGATGTGTTTGATTTTTTTCCTTCAAAAGG
AACATCCTAGAGAGGTGACTATTCGACATTAGGTATGCTAATCCATCGGTCAACTATACCTGCCTGCGGCTAAGAACCTTTGAATATGTTTCCTTTTTTT
ATTTATCGTATCTTATAAAAGTAGCAAATACGACTATTATTCAAACCTTGCTGAAAACAAAAGATAGATAGGATGTTGCCATCTTGTAATGTAATCATAG
AATTGGAATCACTGAATTTGCCATTTGTTTCAACTTCAGAGTTTGGTCAAATTGAACTCTCGAACTTTTCAACTCCTAAACTTTAAGGTCAGGCTCACAT
ATGTCATTTTCAAATTTTATTGGACGGAAAGTTGAGCTGGACGTTTCGAGCTCGGCTTTCTATCCAACTGAACTAATGGAAACACTGATTTCATCGCAAC
AAATTTTCAGCAATAGATATTCAATCTCTTCTTGAATTCATCCTTCATCAGTATGGTGAATAAATTGTTCTTGGATTATGCAGAATGCATCAAGAGATCT
TAACAGGAGGCCAGATCAGGGGGGTGTTCCCAGCTGGGCGTGGAAGGTGACCGCCCCTCCTCCCACGCAGCCATTGCCGCTGTTCTCGTCGGCGTCATCA
TCCGCTGCAGCATCATCAGGATTCTCCAATACCGCCACGACAGCTGCCCACACCGCCCCATCGGCCTCCCTCCGGTTCGACCCGCCGCCGCCGCCGTCCA
GCCGCCACCGCTGAACGTTAAGAAGCCACGCTGTAAATTTGCCAGGAAGCCGACATTTTTTCCTCTCTCGGCGTTGCAACTTTTAGGTTTTGCGCCGGG
GTGGTTTCTTGTAGTGGAGTGGATTCATAATTAACTGTATTTGCATGCTGCCCATGTGAAATGCTTCTCTCTCTTTTTACGCTCTCTGCACCGGATGTTC
TGGGAGTTCTTGTTGCCGGGAATTAACTAAATTCACCGTCTGAGATTGATCTATACATTGTTGTAGAGAATCGAATCATTGTTGAAATTAGTTACAGAAT
TATAGATATCATAAGGCACCCTCTACTTGTGGACAAAGTTCAGGAAGATTATTCCGGTTCTTTTCTTGGCTGTCAATTCGGCTCTTGTCCTTGGTGTGGA
GCCACTTGCCTACTGTCTTGTTCTGTTGCTGTTTGTTCTTGGCTTCTTGCACAGTTACAAATTTGTTGGCTCGAATGGATGGTGAGACATTTGGTGCAA
>comp76332_c0_seq55
ACTGCTCTGAATTAAGAGGCGTTGAAATATTGTCCGGTCTACATATAGGCACTTGAGGTGTATTGACAGACAGTTCCCCTTAGTTTGCTACAACACATCA
GGCGAATTCCACCAAATTAGGATGATGTTTGTTTCTTAGACACAGGATAGCAATGCCAAAAGTCTTTTATAATTTTCCGCTTCACCGCAGATTTGGCATC
TCAAAGGGAGTTGTGGCAAGATTCTCTTGACCAAGCTTTCTTGCAGATTTGGCAAGAAAGATAAAAAAAATGTTGGTATACTTAAGCATCCTTAATAGAA
ACTAAACAGGTCTTAGATTGAAATTATCATGAATAAACCCTTTTTCACTTTAGCTGTAGTCCGGATGAAATCTTAGGGGCCTTGCTTAGCCTGCCCTGAA
GCTTGCCAGTAAGTCGAAGCTGATGCTGACACAGCACTGTTTCAAGTTCTCATCTTCTGAAAATGATCAGCAGTCAATAGATGTTTTAGTCCATTCTCCA
GAAAAATGAGTGCCGCATGAATCCTTTCCGGTTTATAGTGAAACATTTTAGACGATGACCTTTAACATTCTAGAAAACGAAGAGAAAACCTACAGTCATC
TAATTTCAGCTTAAACATGTTCCAGCAAGATCATCACCATCCTGACATGTTCCGTTTTGTCCCTAGACCCGAGCACAAGGTATTGCGAGGCTAATTTAGC
TCAATTCAGTTGTCAGAGCAATTGTGTGTGAAAAGTGTGAACAACAGCATAGGAGACAATACAGCAAGTGTAGCGTGCCAATATGTGTCCAGCTTGATAG
TTCACTATAATAAAGTTCTTTAATTCAAGTATTTGAATAATATAGAATCTCAACCTTCAAGTTTAAAAGGTTTTGTATTTGCTATTATAATCGTTGCCAC
CCCCTCACCCCTCTCAAAAAGAGAGTTACTTTGTAGCGCACTGTAAATCTCTTGATTGAAATAGTGGCATGTCATCTTATTTAATTCAGTTATTTCATAA
GGTACAATTTGTTTAGCTGACAGCTCAGACATGCTTCTATGCAACATGTTTATACCAATGCTGTATTTTGGCAATCCTGGATGTGGCATTACTTAACTGT
GCTTTCATATTCAATAGTTCTGGCAAAGGGAGGCTGCAAGCTTGAGACAACAACTGCACAACCTGCAAGAAAGTCATCGGTATGGCTAGAAAACCCTGCA

AATTTTACAAATGTACAAGTCAGAAAATTTGCCTAATGTAAAAGGATCAAACTACAGGATTTGTCAGCATGAGCACAAGTGCCTAACTTCCGAGTTAGAT
AACTCTGGAGGAAGGAAATCCTTCCTACTGTTTGTTTCAAGAATATACAAAAATTCAAAACAACATTAGAATAGAACCAAAAGAAGAAACAATATTCACC
ATGTAGCAATACTCAATTCCTGGTTGTCTTATTTATAAAGGTATAAAACTAGGTTCTGACGAGACAATTATGAATCAACTACACCATGTTACAGATCATC
CATAACTCTTGGTGATGCAAATTAAGGGCTGTAAAGTGACAAGTATACCTCCACCAACTAATTTGTGCAGAGTGTATAAAATTGTAAAACAATGGATAAG
AATGAATTTAGTATACAAGATAGCATACAATGCCCAAGTTTAAGGAGACATACTGATTTACTGACTCCATAGGACAATCTTTTGCTTATGAGGACATCAG
TTTTCATAAACAATATCAATGTAAAAGGAGGTAACAGTTTATGAATTTCCTATGCCAAGCCATTTATAAGTCAAAAAATGTACCTATTTAGCTTTTATGC
ATTCCAATTAACTTCCAAAGAAGAAGAGTACATACTAATGGAAAACAAATATGATATTATCATCTGTTTTGCCCGGTAGTTTCTTGAGTCTCTGATTTGT
CCGCACTTCACTCCCAACCAACACAACATTAATACACATGGGCAGAGGAATATCAGGTTATCAGGTAAACTGAATACAGTTTGATTGACTGCAGTCAGTT
GATGGGACAAGATCTTTCTGGATTGAGCGTCAAGGAACTCCAAAATCTAGAAAATCAGCTAGAAATGAGCCTACGTTGCATCCGGACAAAAAAGGTAAGG
ATGCTAGCCATATTTCTGTTTTTCGTTACCAGATTGCCTCATCCAAGAATACAACTGATGATGACGTTTTTTACTTTCAGGACCAACTCTTGATTGATGA
AATTCACGAACTGAATCGAAAGGTTCTGGAATATTCTGTTATTACTCTGCAATGAGAGGTCTCTGAGTGGATTTCAAGAAGCTGACTCTACTGGCTCATT
TTCAGGGAAGTCTCATGCAACAAGACAAGATGGAACTATACAAAAAGGTCAACCTAATCCGTCAGGAAAACATTGATTTATACAAGAAGCTCTACGAGAA
AGAGGTGACAAGTGAAGTCAACCGAGATTCATCAACTCTAAACTTTGCAGTTGTCGAGAACGTCAACATTCCTGTTCATCTTGAACTTAACACTCCACCG
CAAGAAAATGATGTTGAGCAAACTGCACCTCCTAAACTGGGGTAACCCTGCTTTTTTCTGCAAAGGAAGATTTTCTTGCAAGTTATGCAGTTGTTGTCTC
TAGCTTGCCGCCTCCACTTGCAGTAACTGAGACGCCGGCGCCGAGCTTGAGCTTGAAACCTAGACTACGGGGACAGGGTAGACAGGAGACGCGAAACCCC
AAACCAGCGCAAAACGACGCGCAGGGAGGGAGTGTCACTGAAGGAGCGACAGGGAAAGCAGCGGCACGCCGGGGGAAATTCCGATCTGAGAGAACGGATC
AGCCATGCATGACAGGAGCTGCGGAGGCAGCAGGGGGGGGGGGCTCCCGCTGCCACGGGACAGTAGGGCACGCCAGATCGGTGAAATCCAGGCGAAGGAC
GAGGCGTGGGCGAGATTTACACGACGGCGCGATAGGATCTAGCCCGGATTTGGATTCGACCGACGAGGGCGGAAGGAACGGCAGGGTGGTGCTCTTGTAG
AGAGAGAAGGAGTCGGAGGGGGCGGAGAGAGAGAAAGGGGGCAGGTGGGGTTTCAGGTAGTAAAATATGGAGCGG
>comp73737_c0_seq3
TAACCAGAGGAAGGGACGGCGGGGAGGGGGCAGGCACAGAGAGCAGCTTTGCTTGGCGGACGCACCGAGGGAGGCGCCTGGGAGCCCTGCTTCTCCCCTC
TTCGTCTTCCTCCTCCGCTTCCAAAGGTTACCGTCTCCCGCCGCCATAAGACTAGTAGCCTTCTTGCTGTGTCAATCCGTCATGCACGCGTCCCTGGATT
GCGCTGGTGACTCCTTCGGGAAAATGGTTAATGATCATACGAGGTCAAATTTGGTTTTTGATAATAAGCAATCTCTATTTGCAAGTCATAACATTGACTA
CGGCCAGCCAATAGCTTGTATATCATACCCATACAATGATTCGGGCTCAGGAGGTGTTTGGGCAGCCTTTGGGTCACGCACTAGCGCTGCAGCTGTGTTC
CATCCCCAAATTGCTTGTGGGGGCACATCTGCGAGAGTTCCCTTACCTCTGGAATTAGCAGATGATGAGCCCATATATGTCAATCCCAAACAATATCATG
GTATACTTCGCAGAAGACAGCTACGTGCTAAGTTAGAGGCTCAGAACAAGCTAGTCAAAAACCGAAAGCCTTACCTTCATGAGTCTCGGCATCTTCATGC
AATGAAGAGGGCAAGAGGTTCTGGCGGACGTTTCCTTAATACTAAACAGCTCCAGGAGCAGCAGCAGCTGAAGTCTCGCAATGCCTCCACCAGGTCCACC
ACAAATGGCGCAAATTCCTCAGGTTCAACACATCTACGGCTTGGTGGTGGTGCAGATGGAGATCAAACCATGTCGGGGACGAAAACAATGGCCTCACAAA
ACAATAGCAAAAAGGCTGTTTCTTCTTCTGCAGCTCTTGCTTTCACCGTGACTCCTATGGTGCGCAAAGATGACACCTTCTTCCAGCACCTCAGCCACAA
TGTCAGCTTCTCCAGCCATTTTGGCCAGGCAAGCGCCCAAACCGGCGTTGGAGGCATCCATAATGAGACCCAGCATAGGGTTTCCGTGATACAATGACGG
TTTGCGAAGCTTATCTGGTGATCCAGGCTTCTAGGTGTCCTCGTGTCCGGTGTAGTCTTGGTCGCTCAGGCAATTCATCCTTGGCTTAGTTTTTGGTGTG
TTAGAACCTACAAATGTTTCATACTCTGTTGTGGTTTGCAGAGCCCATAAATCAGGGCTCTCCTAAAAAAAATCAGGGCTTGATGGCGACACTACTATCC
AAGTATTGTTGTAATGGTGTGTGTTAGAAACTCGCAAAAAACCCTTCTTTGTGCTCGTATTAGTACATTATGCTGCACAATTAGCTGCTATTCTGTTCGT
ACTACAGTCTGGTATGCAGTGGTTAGTGGTCCAGTTTA
>Locus_47312_Transcript_1/1_Confidence_0.000_Length_215
CGATCCTTCGCCTCCAATCACCCCTTCACGTCACAACACAAACTCCAATTCCACGCCAGATCCAACCTCCAAGAAAGCTTGAGGCAGCAACAACAATGGC
GTCTCGCCGCTTCTTCGCGTACGACCCCTACGACTACTACTACACCACCCCGTACGAGTACCCCTACCCGTACTACCACCACCAGAAGGCCTCTAACCGC
AGCGCCGGCGGCTTC
>comp83218_c0_seq1
CGCAGCTCGCAGTACCGCGGCGTCACCTTCTACCGCCGCACCGGCCGCTGGGAGTCCCATATTTGGGATTGCGGCAAGCAAGTGTACTTAGGTGGATTTG
ACACTGCTCATACTGCTGCAAGGGCGTACGATCGAGCTGCCATCAAGTTCCGTGGCTTCGACGCCGACATAAACTTCAATCTCAGTGACTATGAGGACGA
CATGAAGCAGATGAAGAGCCTGTCCAAGGAGGAGTTCGTGCACGTCCTGCGACGGCAGAGCACCGGGTTCTCACGGGGCAGCTCCAAGTACAGAGGCGTG
ACCCTGCACAAGTGCGGCCGATGGGAGGCTCGCATGGGGCAGTTCCTCGGCAAGAAGTACATATATCTTGGGCTATTCGACAGCGAAGTAGAGGCTGCAA
GGGCTTATGATAAGGCCGCGATCAAATGCAATGGTCGAGAAGCTGTGACGAACTTCGAGCCAAGCACGTATGATGGGGAGCTGCTGACTGAAGTTGCTGA
TGAAGGTGCTGATGTTGATCTGAACTTGAGCATATCTCAACCAGCTTCTCAGAGCCCGAAAAGGGACAAGAACTCCCTTGGTCTGCAACTCCACCATGGA
TCGTTTGAAGGCTACGAATTGAAAAGAACAAAGATTGATGCTCCCTCTGAACTCACGGGCCGACCTCATCGGTTCCCTCTTCTGACCAAGCATCCACCAA
TCTGGCATGCCCAATCTCATCCCTTCTATTCAAATAATGAGAATGCATCAAGAGATCTTAACAGGAGGCCAGATCAGGGGGGTGTTCCCAGCTGGGCGTG
GAAGGTGACCGCCCCTCCTCCCACGCAGCCATTGCCGCTGTTCTCGTCGGCGTCATCATCCGCTGCAGCATCATCAGGATTCTCCAATACCGCCACGACA
GCTGCCCACACCGCCCCATCGGCCTCCCTCCGGTTCGACCCGCCGCCGCCGTCGAGCCGCCACCGATGAACATTGAGAAGCCACGCTGT
>Locus_12915_Transcript_8/30_Confidence_0.451_Length_4002
GCTCCTTGAGAAACACCATCCAATTACAAGACTATTTGATGCAACAATCACGCATATGGCTGCGCTAGAACTACTGCTCTGAATTAAGAGGCGTTGAAAT
ATTGTCCGGTCTACATATAGGCACTTGAGGTGTATTGACAGACAGTTCCCCTTAGTTTGCTACAACACATCAGGCGAATTCCACCAAATTAGGATGATGT
TTGTTTCTTAGACACAGGATAGCAATGCCAAAAGTTTTTTATAATTTTCCGCTTCACCGCAGATTTGGCATCTCAAAGGGAGTTGTGGCAAGATTCTCTT
GACCAAGCTTTCTTGCAGATTTGGCAAGAAAGATAAAAAAAATGTTGGTATACTTAAGCATCCTTAATAGAAACTAAACAGGTCTTAGATTGAAATTATC
ATAAATAAACCCTTTTTCACTTTAGCTGTAGTCCGGATGAAATCTTAGGGGCCTTGCTTAGCCTGCCCTGAAGCTTGCCAGTAAGTCGAAGCTGATGCTG
ACACAGCACTGACATAGATCCCAATGAACACAAGTTATTTCGAGAAGATAAAATACCAAGCAAAGCCAGGATTGCATTAAAAGATAGTGTATTTCTACTA
TACGATCTGAAAGCTTAATTTATTATATCAATAATCAAATTGGCAGCAGTGTGGAACAGATATAAAGAAGATGCTTCAAATCACAAATAACTACAGATAA
ATTGAAGGGTGAAGGTTCATTCAACCACCAAACACCCAAAATGTCGAGCTGCATATGCACAGAACCTAAGTACGGTACAGTTCATAGTTTGGGAAAATAG
ACTGCAAATGTGCACAGATAATACAGATTGTTACTCGATGAACAGTAAGCACGGTATTTGAAATCCTTCTAACCATCCATCCAGGCAGTGCCTCGTATAC
TCAAACCTCTTTTTGGAGGAAGCCAAACCATGTGATCCTTTTCAACCCAAATACATATATTCAAGATGAAACTAACAAAAAGACAGTTGATAAAGAAACA
CGAAAGAGAGTAGAAAAAGGGTACCTGTTTCAAGTTCTCATCTTCTGAAAATGATCAGCAGTCAATAGATGTTTTAGTCCATTCTCCAGAAAAAATGAGTG
CCGCATGAATCCTTTCCGGTTTATAGTGAAACATTTTAGACGATGACCTTTAACATTCTAGAAAACGAAGAGAAAACCTACAGTCATCTAATTTCAGCTT
AAACATGTTCCAGCAAGATCATCACCATCCTGACATGTTCCGTTTTGTCCCTAGACCCGAGCACAAGGTATTGCGAGGCTAATTTAGCTCAATTCAGTTG
TCAGAGCAATTGTGTGTGAAAAGTGTGAACAACAGCATAGGAGACAATACAGCAAGTGTAGCGTGCCAATATGTGTCCAGCTTGATAGTTCACTATAATA
AAGTTCTTTAATTCAAGTATTTGAATAATATAGAATCTCAACCTTCAAGTTTAAAAGGTTTTGTATTTGCTATTATAATCGTTGCCACCCCCTCACCCCT
CTCAAAAAGAGAGTTACTTTGTAGCGCACTGTAAATCTCTTGATTGAAATAGTGGCATGTCATCTTATTTAATTCAGTTATTTCATAAGGTACAATTTGT
TTAGCTGACAGCTCAGACATGCTTCTATGCAACATGTTTATACCAATGCTGTATTTTGGCAATCCTGGATGTGGCATTACTTAACTGTGCTTTCATATTC
AATAGTTCTGGCAAAGGGAGGCTGCAAGCTTGAGACAACAACTGCACAACCTGCAAGAAAGTCATCGGTATGGCTAGAAAACCCTGCAAATTTTACAAAT
GTACAAGTCAGAAAATTTGCCTAATGTAAAAGGATCAAACTACAGGATTTGTCAGCATGAGCACAAGTGCCTAACTTCCGAGTTAGATAACTCTGGAGGA
AGGAAATCCTTCCTACTGTTTGTTTCAAGAATATACAAAAATTCAAAACAACATTAGAATAGAACCAAAAGAAGAAACAATATTCACCATGTAGCAATAC
TCAATTCCTGGTTGTCTTATTTATAAAGGTATAAAACTAGGTTCTGACGAGACAATTATGAATCAACTACACCATGTTACAGATCATCCATAACTCTTGG
TGATGCAAATTAAGGGCTGTAAAGTGACAAGTATACCTCCACCAACTAATTTGTGCAGAGTGTATAAAATTGTAAAACAATGGATAAGAATGAATTTAGT
ATACAAGATAGCATACAATGCCCAAGTTTAAGGAGACATACTGATTTACTGACTCCATAGGACAATCTTTTGCTTATGAGGACATCAGTTTTCATAAACA
ATATCAATGTAAAAGGAGGTAACAGTTTATGAATTTCCTATGCCAAGCCATTTATAAGTCAAAAAATGTACCTATTTAGCTTTTATGCATTCCAATTAAC
TTCCAAAGAAGAAGAGTACATACTAATGGAAAACAAATATGATATTATCATCTGTTTTGCCCGGTAGTTTCTTGAGTCTCTGATTTGTCCGCACTTCACT
CCCAACCAACACAACATTAATACACATGGGCAGAGGAATATCAGGTTATCAGGTAAACTGAATACAGTTTGATTGACTGCAGTCAGTTGATGGGACAAGA
TCTTTCTGGATTGAGCGTCAAGGAACTCCAAAATCTAGAAAATCAGCTAGAAATGAGCCTACGTTGCATCCGGACAAAAAAGGTAAGGATGCTAGCCATA
TTTCTGTTTTTCGTTACCAGATTGCCTCATCCAAGAATACAACTGATGATGACGTTTTTTACTTTCAGGACCAACTCTTGATTGATGAAATTCACGAACT

GAATCGAAAGGGAAGTCTCATGCAACAAGACAAGATGGAACTATACAAAAAGGTCAACCTAATCCGTCAGGAAAACATTGATTTATACAAGAAGCTCTAC
GAGAAAGAGGTGACAAGTGAAGTCAACCGAGATTCATCAACTCTAAACTTTGCAGTTGTCGAGAACGTCAACATTCCTGTTCATCTTGAACTTAACACTC
CACCGCAAGAAAATGATGTTGAGCAAACTGCACCTCCTAAACTGGGATTACAACTAAATCCATGAAGGCATGCAGCATGGCACCGCTTATGTTCATTATT
CTCAACGGTACGATTCAAAGCAACCAGAAACACTATTGGTTGTAAAGAGCTTAAACACATAGCAAGACTGACGCGGTATGCATGCCATGAGTGATTGATA
ATGGTAGTGTAAATCTATTTCAAATAAAATGTATTTTATTGAAGAATACAGCAAATGATTTGTATTATAACTATTTGACACATTCTGCAAGTTTGTTGAC
CAACGTGAGTACATGATAGCTACAAATCTTATATTGACAAAAAGATATGGTGTGTGGAAAAAATTCAGGTGCTATTTTTTTCTATTTTGAGAATTGTATG
CATAGCAGAAGAGCACAAATTGCATTCTGGTTTATCCGTGCAGGCATTCCAGAGTATCAGAATGGGGATCAACCTTTCTTCAGCTACATATGTAAATATA
CGGATGCTAGTTGCTCACTTAAAATTTTGTAGTGTTCAAAGGAATAACAACAGTAAGACGTTGACGCACCGAAAATAACCAGAAATAACGGATTGTACTA
TGACAAGACACACGACAGATACACTAGCTTAAGTTTGTACAGGAAAATATTTGGCCTATTCTTATGTCACAGTAAATTAAGATTATCACCTTAATCACAG
AAATTCAAGGGGTAAACGATTATAGCACAAAACGATCCTGGACCCCAAGACCTGGGTCTCTGAACGGCTGAACCTCCAATTATACCTCTATACATTTGGA
ATGTGTGTAGTCAAACTTTCGCTACTGGTATCTTTACAAATATTTACATTGAACACATCAAAATGATATACATGGATTCCTCTCGATATGTCTTTGAGGT
TTGGGTTCTAAAAATGATATACAAATTATTAGGTTCTAAACTTAAATGATATATACACATCAAAATGATATACAAATTATTGGGTTCTAAACTTAAATGA
TG
>Locus_14087_Transcript_46/66_Confidence_0.333_Length_2030
GATTCAAAATTGTAGCAGATTTGTCCTCTACCCACCCCTCTGAATCCTTTTTATACATACACTGACCATGGCACCAATGATCAAACCAAACACCAGCTGC
AAAGATCACCCTGGATTCCTGGAACAAAGAGGGCCTAGAGCAACGCAGCAACCTTCGCCACTTGAAGAGGATAGGTACAATGTGGCTTAGCTAGCACCTT
GCAGTCAGGTAGAATTATTCAACCGGATAAAGATTATTTCAAGTAAAGGAGGGCTACCAGTTTATACCTGTTCAGGTGGGTGCAGCAGTTGAGAACTTTT
TTTCGATACAGAGCAGTTGAGATCTTTAATTCTGGCATGGGTTCCTTTGGGGTGAACTGGAATCAGAAGAACTCCGCGGTGTGCGATTGGGAAAATTTAG
CACCTTCTGTTCCAAATGCAATCGGAAACCCCAGGAACGGAGCTACGAGGCATGGATCTGTGAATTCATCTGGTGGCACTCTCACTTCTAGCTCAGAGCT
AGGGCATGGTTCATCCAAGAGCTCCATGTCGGCATCCATTGATTCACCGTCCAAAGTAGGAAACAGCTTGGAGTTCAACTTTGCTGCTGTCAAAGGCCAT
GTTAAGAACATGGATAAGGATTTTAGATTTGACGACTTGGGGACTTCTCCATCGTCGATGATAGCATTCAGCCATGGAGAGCCATTAATCAGCCTTAAGC
TTGGAAAAAGGACTTACTTTGAAAATGTCTGCGGAGGGCAGGATTCCAAGAGCACTGCACCTTCTACCAGGACTTCTCCATCAACCGTTCTCAAGAAGAC
CAAGGTGTCTCAGCAGAATACACAAATGTCATACTGTCAGGTTGAAGGATGCGGAATTGATCTGTCCTCCGCTAAAGATTACCACCGCAAGCACAAAGTC
TGTGAAGCTCATTCTAAAGCCCCGAAGGTTGTTGTTGCTGGTGTAGAGCGCCGTTTTTGCCAGCAGTGTAGCCGGTTTCATGGCTTAGCCGAGTTTGACC
AGAACAAACGAAGTTGCCGTAAGCGTCTTACTCATCATAACGCACGAAGGAGGAAACCACAGGCAGATACAATTTTATTCAGTTCATCACGTCTCTCAAC
AATGTTTTATGATACAAGCCAGCGGACAAATCTTTTCTGTAGTCAGCCTCTTTTTGGCCAAGTGAGAAGCAATGCAGTTTCTTCATGGGATAACTTGGGA
AGCTTCAATTTTATGGAAACAAAACATCTGTTGATGCAGCCAACGAAAACGGCAGCCCCTGATGAGCTGCATTTCTCAACCCCCCAGATGTCAAATGGTG
TTATGGTTCACGCTGTACATCATCATAATGTCGGTGAGTTCATGCCATTCAAGGATACCACCACAAAGATCCTCAACCAAGGTGTGGAAGCTTCTGCGAT
TGGATCCAACTCAAATGGAGACCCGGATCTTGGGCGTGCTCTCTCTCTTCTGTCAATCGGTTCGTGGGGTTCAACTTCAGCCGTCATCCAACAGCCTAGT
TCTCATGCGCAAGCTGGTGCGGTGCCACCCTTCGCCACCGTCGCCGTCTCCAACCCTGCAATGCATCCTCTGGACTCATCCCCGGGAGGATTCTGGCAAG
ACGTCCCTCCCCCGCTCGATGAAACTCCACATATTCAGGCATTCACGCACCTCTGATCAAATGCTGCATCCCCTGGGAACAGCAGGTGGTTATACCTTCC
GTAATCGTCCTAGGTTTGCTTTGCCAGTGACTGGCTCTGCACAATGTCGATCCATCACCGGAGGCATTTGGTAAGGTGAAGACTAAAGACTCAAGAATAA
CCGGAGACTAACTCGCTTCTCTTCCAGGCTCAAGACTGTTTGGGTCCAGCGGTGTTGCTACCTGATCCTGTTTTCGGTTCTGAGTACATCCATTACTAAT
CATGGGATTACTTGTTTAGGCCAACCAGACATATTCTGGGGTGAGATTACGACATGTTAATTTACATTGACGGTGTTTCTACATGTGTTAGTACTAATGA
TGTTATCGCTCTTGGCCCCGTTACGTTTCA
>Locus_15085_Transcript_26/40_Confidence_0.434_Length_1888
GCTGCTCGAGGAGCTCAGCTTCAAGCGCATGGTTGTCACCGACGAGTGCCTCGAGATGATCGCGGTTTCCTTCAGGAACTTCCAGGTCCTGCGCCTTGTC
TCCTGCGAGGGCTTCAGCACCGCCGGGCTCGCCGCCATTGCCGCCGGCTGCAGAAATCTAAGGGAACTTGACCTGCAGGAGAATGAGATTGAGGATTGCT
CTAGTCATTGGCTCAGTCTCTTTCCAGAATCCCTCACTTCGTTGGTAACACTAAATTTTTCATGCGTAGAGGGGGATGTCAATTTCACTGTACTTGAACG
ACTAGTAACCAGATGTCGCAACCTCAAGACTCTCAAGCTCAACAATGCTATCCCTCTTGACAAGCTTGCTAGCCTCCTTCGTAAGGCTCCTCAAATTGTG
GAGCTTGGAACAGGAAGGTTCTCTGCTGACTACCACCCAGATCTCTTTGCAAAGCTTGAAGCAGCGTTTGCTGGTTGTAAAAGCCTACAAAGGCTTTCTG
GGGCTTGGGATTCTGTTCCAGAGTATCTGCCAGCATTTTATTGTGTATGTGAGGGCCTCACATCACTTAATCTGAGTTATGCTACTGTGCGAGGCCCTGA
GCTGATAAAATTTATTAGCAGATGCAAAAACTTGCAACTATTATGGGTGATGGACTTAATTGAGGACCACGGTCTAGCTGTTGTGGCATCATCTTGCAAT
AAACTACAGGAGTTGCGGGTCTTCCCTTCTGCCCCTTTTGATCCAGCTGAGCAAGTTTCATTGACTGAAAGGGGACTTGTTGATGTTTCTGCCAGTTGCC
CGAATGTTGGAGTCAGTCCTCTACTTCTGCAGACGGATGACTAATGAGGCCCTTATTACCATAGCAAAGAACCGGCCCAACTTCACCTGCTTTCGCTTAT
GCATCATCGAGCCTCACACTCCAGATTACATCACGCATCAGCCTCTTGATGCAGGTTTCAGTGCCATTGTGGAATCATGCAAAGGCCTCAGGCGCCTATC
TGTCTCAGGCCTTCTCACAGATCGTGTATTTAAATCCATTGGGGCACATGCTGATCGTCTTGAGATGCTCTCAATCGCCTTTGCTGGGAATAGCGGATTG
GGCCTTCATTACATCCTTTCGGGCTGCAAGAGCTTGAAGAAGCTGGAGATCAGGGACTGCCCATTTGGTGATAAGCCCTTGCTGGCAAATGCTGCCAAGC
TGGAGACAATGCGATCCCTTTGGATGTCGTCGTGCTCACTGACCCTGGGCGCATGCCGACAGCTTGCACGCAAGATGCCCCGCCTTAGTGTGGAGGTCAT
GAATGATCCTCGACGGGCATGCCCCTTGGATTCACTTACAGATGAAAGCCATGTCGAGACATTGTATGTCTACCGGACAATCGCAGGTCCAAGGTCCGAC
ACACCAGCCTGTGTCCAGATTATTTAGGGGGACCACCTGTGGTATGGGATAGGTACTATGGAAGTGTTCCTTGTCAATCCCGATTTGTACCTTCAACACC
TTCCTAGCATAACATGGATTGTTTCCTTGAAGTATAAAGGCTGGTGGCTTGGCTGATCCTGTCATTACCTCGAAACTGTTTACGACTTTAGGACGAGCCT
CGGGGCCCTTTTCAGGTGTTAATAACTGTAGGTGGAGACTCGATGAGTTACAAAAACTACTAGATAGCGTCTTGAGAAAACTTGGGTTTGTTTTCTTAGC
GTGTTTAGTTCTTTTCATGTATAAAAATATGGGATCATTTCAATCTCGCAATGCACTGTACTATCGGAACAAATTGTACCAGGAACCTCTACTTTTTTTA
CCTCCCCTCCCTAAGTATGGGTTGTTGCTTCAACTTGTTGACTTGTTGTGAATGCGGGAATAATTCAAGCTTGTGGTGCTGGGATGAT
>Locus_16183_Transcript_40/55_Confidence_0.517_Length_2939
AGTAGGCTCATATTTGGCATCAGCAGAGGCCTCGCGGTGCCCCGTATTCTCATTTCCTCGCCGTATTTTCTCTCTCCAGCTCCAGACTCCAGAGTCCGGA
GGAAGCAGAGAGAGAAGCTCGACGGGAAGGTAGAGAGAGGTATAGTGCTCCTGATCCCTCTTTGAAATCCTCGATTGGTCAGTTCTGTTCCTGGATATAC
TGTTGCTGCAGATTGGATGCGTCTGCTTTCCTTTCGGGGAGTGGTGTCTAGCTCTGTTGGTTCTTGCCTTCATAGTTAGTACTATGCCTTAATCTTGCTC
CGCGATTGGTTTGATTCATGTTTTTCCTTTAATCTTGCTACCCCAGGATTTTCCCCTTTGGTTTTCTTGGTTTGCTTCTCGGAAATCCGTGATTTTAACA
GGATTTTTGGCTGGTGTTGCTCGATTTTTGTTCTTGACTGTGCTTCCTTGGGATGAGCTTTTGCCTAATTCGACATTACAAGATGTGTTCTTTCTAGAAG
TTAAATTGATCTTCTATGAGCCGGGCGCTGTATCGTGCATTACATGGGGGTGGCGTACTGGTACTTTGGGCCGTAGAGTTGATGGAAATCTGCAGTCCCT
TTTGTTGGATAAGGGGCTTTTTCCTTGGATAAGGAGGTTTGAAGCTAATTTTGTGCGAATTGTGGCGGTTTCTGGATATCCTGGTTGAAAGTGGTGTGAT
AGATTGTTCTAGAGAATAGGAAGATATATGTTTTATTGTTAGAACATCAAGAGAGTGTTGAATCAGGTATGTCAATGTGTGAACTTATTGATGGGAGCTA
AATGCTTGCTGACGTATCTTCGCTATAATGACCTAATCAAACCTAACAAGCACTAAATACTGATCAATTCTCAGTTATACAAAGATTTTCCAGAGAGTTC
TTCAGAGCTCGAACTTCTGGGATGAAAATAGTTTGGCTCAAGATAGTCCACTGGAAGGAGTTTAGGATATCTGTTTGAGCTCTGCCTGCAATATATGGAA
ATCTGAAGGTGATATGGTAGTAGAGTTGGGGACCCTGATCCCCGAGCCAAAAGAAGTTCGGTAGAATTTCTCAGGAATATTGTGGTAGTAAGAAGCAAGT
TAAAAGGAACCTAATTAGGAAAAAAGACATGCCTTGCTCGCTCATGAGAATATAAAACAGGTATGCCACCTCCAGTCAGTACTTGATTAATTTCATTGAA
AATGGAGGGCTGTAATAGATCCTTATCTATTAATGTGTTGTACCTAAACTTGGAAATCATCAGCTGAGGGCATTGTGCATGGTGCGTGGAGTACTAGCCC
TCGGGAGATGCTTCTTAATTAGGCAGCGAGAAGGATAGAGATCGGTTTGACATATGTTCCATTTGTAGCTGTACTGGATATTTCTGCATGTTCCCCTGGA
GCAGTTAGTTTCAGAATTATGATATTTCTACTTGTGGCAGGATTGTTCTGTTTCTATTATCTGTTCATATTTATGCTAGTCTGTCTTTTATGTTGTAATA
TCTATTGTCTATTGCACTTCAAGTGACTATGCATAACCATATTCCAGTGATTTAGTGAATGGTTATGAATTAAAGTAAGATGACGTTTTCATCAATTATC
CTTGTTCTTTATCACGTCATTATCAATTCAGCTGATTAAATTCTGGCATCTTGTCATGATGGTTACTTTAGCTGATTTAATGTTTCTCAGAGCTAAGGAT
ACCATCTCATAGTGCTCACTCTTTTGTGGGAAATGGAGGATCCCATGCCGAAGTCCAACAATGGCTTCTTGTCAGGAAATGGCTATCAGATGAAGCAATT
TGGTCATAAAATCCACGATAGGGACTCGTCATCAGAGTCCGGTCGGTCTCACCAAGAAGCTTCCACAGCGAGTGAGAACAGTATAAATGAACAGCACACC
TCAACACAATCTGACAATGATGATGGTCATGGGAAGCATAATCAGGACACGATGAAGTCAGCAGTGTCCTTGGGGGAACCAGGATCAGCCTTTTTGGCCC
CAAAACTAGATTACAGCCCATCTTTTGCTTGTGTTCCTTATACTGCTGATGCTTTTTATGGTGGGATCTTGACAGGATATGCTCCACATGCCATTGTTCA
TCCCCAGCAAAATGATTCAGCAAACTCTCGGGTTCCGTTGCCTGTTGAACCTGTAGCAGAAGAGCCAATATATGTTAACGCAAAGCAATACCATGCAATC

CTTAGGAGGAGGCAGATACGTGCTAAACAGGAGGCTGAGAATAAGCTGGTGAAAGGTCGGAAGCCATACCTCCATGAATCTCGACACCGCCACGCAATGA
AGCGAGCCCGTGGATCAGGGGGGCGGTTCCTCAACTCAAAGCAGCTCCAGGAGCAGAACCAGCAGCAGGCATCAGGTGGTTCAAGCTGCACAAAGGC
CATCGGCAATAACACAAGCTCCCAAAGTGGTCCCAGTCACACGCCTCTGACACTTGCTTCCTCTGACACTGCAAGCGCTTCGAGGGCCAACCAGGAGCGC
ACCTGCTTCCCCTCGGCTGGCTTCCGTCCCACAATGAACTTTGGCGCGCAAGGTGGAGGAGACGCAAAGCTAGCTGTGAACGGCATGCAACAGCATATTT
CCTTCATCAGGTGAAGCAAGTGAAGTGGAGCACTGCCTGTGGCAACTCATCCTTGGCTTATGAAACTATGAATATATAATGGACATGTAGCTTCGGACTC
AGAATATAACCAAACATATGGGAGTCCTCAAGTTTCCTGTAGTTCAAAGAACTGATAGGTTATGGCCTTATAGGTTTGCTTCTGGAGACTGAAATCCGTG
CTTGATATACTATCTTGTGCGAGTTCTGAGGTGAAAGACCTTGAAACTTGCTCTTGTGGTTCAGTACCTGTTGCTTTTGTGTCTGCAAGGTTAGCACTGC
AATTTGCAATGTCGGATTTCTGTTATGCCACTAATTTTT
>Locus_10929_Transcript_1/78_Confidence_0.052_Length_415
AGCCAATGAGTTCATCGTCCCCGTGGCCTGTGTATCAACAGAGCACGGCAGTACCACCTCACCATCAGCGTTTGTACTCATCTGCTTGTCCTGGCTTCTT
TCCGAACCTCCAGGAAAGGTCAATGGAGCGGAGGCCTGAATTGGGTCCCCAGCCCTACCCCACCTGGGGATGGCAAATGCAGGGCTCCCTTCACATGCCG
TTGCACCACTCTGCAGCATCATCAGGATTCTCTACCGCCGTCGGCACGAACGCCGGCAACGGGCCGTTGCCGTCTCAACAGCCGGCGCCGTTCTCGGACC
ACCAGTTCTACTTCCCACCAACGGCCTGAGCTTGTTGGTCAGTTCAACTCTTGGGACGGCAGCTGACGTTGTGTGTGAACGTTTCGCAGCTCTCCGGTAA
CAGTTGTTAATTATT
>Locus_13957_Transcript_53/58_Confidence_0.259_Length_1334
GCCGCGGGCCCCGCTCCCGCAGCTCGCAGTACCGCGGCGTCACCTTCTATCGCCGCACCGGCCGCTGGGAGTCCCATATTTGGGATTGCGGCAAGCAAGT
GTACTTAGGTGGATTTGACACTGCTCATGCTGCTGCAAGGGCGTATGATCGAGCGGCTATCAAGTTCCGTGGTGTCGACGCCGACATCAACTTCAATCTC
AGTGACTATGAGGACGACATGAAGCAGATGAAGAGCCTGTCCAAGGAGGAGTTCGTGCACGTCCTGCGACGGCAGAGCACCGGGTTCTCACGGGGCAGCT
CCAAGTACAGAGGCGTGACCCTGCACAAGTGCGGCCGATGGGAGGCTCGCATGGGGCAGTTCCTCGGCAAGAAGTACATATATCTTGGGCTATTCGACAG
CGAAGTAGAGGCTGCAAGGGCTTATGATAAGGCCGCGATCAAATGCAATGGTCGAGAAGCTGTGACGAACTTCGAGCCAAGCACGTATGATGGGGAGCTG
CTGACTGAAGTTGCTGATGAAGGTGCTGATGTTGATCTGAACTTGAGCATATCTCAACCAGCTTCTCAAAGCCCGAAAATGGACAAGAACTCCCTTGGTC
TGCAACTCCACCATGGATCGTTTGAAGGCTACGAATTGAAAAGAACAAAGATTGATGCTCCCTCTGAACTCACGGGCCGACCTCATCGGTTCCCTCTTCT
GACCAAGCATCCACCAATCTGGCATGCCCAATCTCATCCCTTCTATTCAAATAATGAGAATGCATCAAGAGATCTTAACAGGAGGCCTGATCAGGGAGGC
ACTGTTCCTAGATGGGCGTGGAAGGTGACCGCCCCTCCTCCCACCCAACCATTGCCGCTGTTCTCGTCATCGTCGTCGTCGTCCGCTGCAGCATCATCAG
GATTCTCCAATACCGCCACGACAGCTGCCCCCACCGCCCCGTCGTCCTCCTCCCTCCGGTTCGACCCGCCGCCGCCGTCGAGCCGCCACCGATGAACATT
GAGAAGCCACGCTGTAAATTTGCCGGGAAGCCGGCATTTTTTCCCTCTCCAGGCGTTGCAACTTTTTCGGTTTTGCGCCGGGGTGGTTTCTTAATTGTAG
TGGATTGGATTCATAACTATATTTGCATACTACCGCGAATGGGTTTCTAGCTGAATTAGTTAGATGGCTTATTACCACTCCTCAGATTCTAGGTTCGATT
TCTCATGAGAGTGAATTTCAGATTAGGGTTAAAAAAATCTCTTTGTCTGTCCTACGCCAAAGCACAGGTTTAAAGCTCGGTCCGGTCCTCATACGGGCTA
TTGTGCCACTGTGTGGTTCGGAGGTTTTCTGGAT
>comp73418_c0_seq4
TAGGATGACTGTGTGGAGGATATCTAACGTGGTGCTTAAACTCGATGGCAGCCAATGGTGGTTCTTGCCTGCCTTACCATGAACTAGTAGTTTGGATCGG
AGTTGTTCTTGACTTACTCATGCGTATTCTTGGGAGTTGGGATCATATCAGTATGTATACATCTGTTATTGCTTGGTGGTGACTTTTCCTTAGAGGTGCC
TGATATGGTGGCGTTTTTTTCTTTGACACATGTTGTATGGATCATAAGTGATGTTATACTCTTTACTAATTTCATGGCCTTTATTATCTTTAGGTTTCGT
TTTCTCTTTTTCTTTTGTTATTATTGGTAAGAAGTATTCGTGTCCCTAGACTTTTGCCATGTATGTACATGTTCGTAGCGAGGTTAGTGATACTGTGAAC
TGTGGTAGTCTTGTTTCGCTGGGGGGAACTTTGATCTGTTGTTCTTTGCCTCAAAACACTGTAAGCCTATTTCTAATATCTAAAACTTGTAAGTGTGCAT
AATGCAGATCAAGCTTCCGAGGTGTTATTTTTCTCATTATGTTTTAGAAACTTAATGATAGATGCAAAGTTATTTTGTTAAATGGAGTCTCAGTCCCGTA
GCCATAAGGGAGGATCGAATCCAAACTTTAGGCTTGTGTGAGTCTTCTTGAGTGCAGCACATGATAAACATGGATCTTAGGGCATTGAGTTAACAGTTAG
TCATCTTCTTTTCTAAACATAAGGATTAGGGTGCTAGTCACACACCATTAACAATAATGTGAATCCAAATAGCATAAGTAGCACGCTTGAGGCCGGCCAA
CACACACTATTCATCTCTTGCTGATTCACTAATTGTCGGTTTGCATTTGGAAGTGTTCTCTAGTTTCTGTGCAGTAACTATTGAGCTAATGACACACTAT
GTATAAATGACAATCGTAACAGATCATTTTGAACCTTTACTTGATTATTATTAGAAACACCTAGGATTTGAAGGATATCGATGTTACTGACTTACTTCTC
TTTTGCTACATCTGGTTAGGACAGGATTCTACTGTGTTACAATAGGGTTTTCTTTGGAAATTTAACGTTGTACAATAATGCCTTTATTTTTTTTGTATCA
GGTGACTCCTTCGGGAAAATGGTTAATGATCATACGAGGTCAAATTTGGTTTTTGATAATAAGCAATCTCTATTTGCAAGTCATAACATTGACTACGGCC
AGCCAATAGTAAGACTCATGGTGATATAAAACATCAGTTTTTTCAACTCTGTTAAAGTCACCATTCTAGTTTTAAAAAACAATAAAAAGCTCATCCTATT
CTTTACATGGGGATGTGCATCTGATTTTATTTTTCTACCATATTACCCAGGCTTGTATATCATACCCATACAATGATTCGGGCTCAGGAGGTGTTTGGGC
AGCCTTTGGGTCACGCACTAGCGCTGCAGCTGTGTTCCATCCCCAAATTGCTTGTGGGGGCACATCTGCGAGAGTTCCCTTACCTCTGGAATTAGCAGAT
GATGAGCCCATATATGTCAATCCCAAACAATATCATGGTATACTTCGCAGAAGACAGCTACGTGCTAAGTTAGAGGCTCAGAACAAGCTAGTCAAAAACC
GAAAGCCTTACCTTCATGAGTCTCGGCATCTTCATGCAATGAAGAGGGCAAGAGGTTCTGGCGGACGTTTCCTTAATACTAAACAGCTCCAGGAGCAGCA
GCAGCTGAAGTCTCGCAATGCCTCCACCAGGTCCACCACAAATGGCGCAAATTCCTCAGGTTCAACACATCTACGGCTTGGTGGTGGTGCAGATGGAGAT
CAAACCATGTCGGGGACGAAAACAATGGCCTCACAAAACAATAGCAAAAAGGCTGTTTCTTCTTCTGCAGCTCTTGCTTTCACCGTGACTCCTATGGTGC
GCAAAGATGACACCTTCTTCCAGCACCTCAGCCACAATGTCAGCTTCTCCAGCCATTTTGGCCAGGCAAGCGCCCAAACCGGCGTTGGAGGCATCCATAA
TGAGACCCAGCATAGGGTTTCCGTGATACAATGACGGTTTGCGAAGCTTATCTGGTGATCCAGGCTTCTAGGTGTCCTCGTGTCCGGTGTAGTCTTGGTC
GCTCAGGCAATTCATCCTTGGCTTAGTTTTTGGTGTGTTAGAACCTACAAATGTTTCATACTCTGTTGTGGTTTGCAGAGCCCATAAATCAGGGCTCTCC
TAAAAAAAATCAGGGCTTGATGGCGACACTACTATCCAAGTATTGTTGTAATGGTGTGTGTTAGAAACTCGCAAAAAACCCTTCTTTGTGCTCGTATTAG
TACATTATGCTGCACAATTAGCTGCTATTCTGTTCGTACTACAGTCTGGT
>comp82796_c1_seq10
AGAAGCAGTGGCAGAAGTAGTTTGTTGTGGGGAGGGCATGTGCCTGGTTGCTGTGTGGGGGGAGGCAGCCACACACTGGGCCTCACTCGTCCTGCCTCAG
GTGGGGTTTGTGGAGGGAGGTAGGGGAAAGGTTGCCATCTCCATCTTGTGCGTCTCTGTTGGATCTTTGTGAGTCATGGCCTCTTTGCTCTCTTGCTTTT
CTTCGTTTCTTGTTGAACTCATGGTTCTTGTTTTTCTTGTGCTTGCCAGGTAGAATTATTCAACCGGATATAGATTCCTTCAAGTAAAGGAGTGCTAGCG
GTTTTTACCTGTTCGGGTGGGAGCACAAGTTGAGATCTTTAATTCTAGCATGGGTTCCTTTGGGGTGAACTGGAATCAGAAGAACTCCGCGGTGTGGGAT
TGGGAAAAATTAGCTCCATCTGTTCCAAATGCAATCGGAAACCCCAGGAACGGAGCTACGGGGCATGGATCTGTGAATTCATCTGGTGGCACTCTCACTT
CTAGCTCAGAGCTAGGGCATGGTTCATCCAAGAGCTCCATGTCGGCATCCATTGATTCACCGTCCAAAGTAGGGAACAGCTTGGAGTTCAATTTTGCTGC
TGTCAATACGTATGGTAAGAACATGGATAAGGATTGTAGAGTTGACGACTCAGGGACTTGTCCATCGTCCATGATAGCATTCAGCCATGGAGAGCCATTA
ATCAGTCTTAAGCTTGGAAAAAGGACTTACTTTGAAAATGTCTGCGGAGGACAGGATTCCAAGAGCACTGCACCTTCTACCAGGACTTCTCCATCAACCG
TTCTCAAGAAGACCAAGGTGTCTCAGCAGAATACACAAATGTCATACTGTCAGGTTGAAGGTTGCGGAGTTGATCTATCTTCTGCTAAAGATTACCATCG
CAAGCACAAAGTCTGCGAAGCTCATTCTAAAGCTCCGAAGGTGGTTGTTGCTGGTGTACAGCGCCGTTTTTGCCAGCAGTGTAGCCGGTTTCATGGCTTA
GCCGAGTTTGACCAGAACAAACGAAGCTGCCGTAGGCGTCTTACTCATCATAATGCACGGAGGAGAAAACCACAGGCAGATACAATTTCATTCAGTTCAT
CGCGGCTCTCGACAATGTTTTATGATACAAACCAGCAGAAAAATCTTTTCTGTAGTCAACCTCATTTTGGCCAAGCGAGAAGCAATGCAGTTTTTTCTTG
GGAAAACTCGGGAGATTCCAAATTTATGGAAACCAAACATCTGTTGATGCAGCCAACGAAAATGTTAGGTCTTGATGGGCTGCATTTCTCAACCCCCCAG
ATATCAAATAGTGTTGTGGCTCACTCTGTACATCATCATAATTTTGATGGGTTCACGCCATTCAAGGGAACCAACACAAAGGTCCTCAACCAAGGTGTCG
AAGCTTCTGCGATCGCTTCCAACTCGAGTGGAGACCCGGATCTTGGGTGTGCTCTCTCTCTTCTGTCAAACGGTTCGTGGGGTTCAACTTCAGCCGTCAT
CCAACAGCCTAGTTCTCATGCGCAAGCTGGTGCGGTGCCACCCTTCGCCACCGTCGCCGTCTCCAACCCTGCAATGCATCCTCTGGACTCATCCCCAGGA
GGATTCTGGCAAGACGACCCTTCCCCGCTCGATGGAACTCCGGAGATTCAGGCATTCGGACACCTCTAACTATGGTCAAACAAATGTACTCATGCTGCAT
GCCCTCGGAACAGCTGATTTAGTCACCTCATCATAATCTGCAACTCTGCTTTGCCAGTGGCAGATCTCTGCATAATGTCAGTCCTATCATCGGAGGCATT
TGGTGAGGTGAAGACTGAAGAATAATATAGGAGGCAACCCCCTGATCTTCCTGATTTAGGATGCATCTAGGATCCAGTTGTGTGTGTGCTTGTGGTTTTG
CCAGAACCGGTTTTTTGTTTGCCGTCCCGTTAGAGAGAAGGGATCAAGTACATTCATGACTGCTCATGAGATTACTTGTTTCACTAATATTACATGATCT
GGAGCGAGATTTCGCTCGGTTCACGTTGATGGT
>Locus_12915_Transcript_6/30_Confidence_0.431_Length_3994

GCTCCTTGAGAAACACCATCCAATTACAAGACTATTTGATGCAACAATCACGCATATGGCTGCGCTAGAACTACTGCTCTGAATTAAGAGGCGTTGAAAT
ATTGTCCGGTCTACATATAGGCACTTGAGGTGTATTGACAGACAGTTCCCCTTAGTTTGCTACAACACATCAGGCGAATTCCACCAAATTAGGATGATGT
TTGTTTCTTAGACACAGGATAGCAATGCCAAAAGTTTTTTATAATTTTCCGCTTCACCGCAGATTTGGCATCTCAAAGGGAGTTGTGGCAAGATTCTCTT
GACCAAGCTTTCTTGCAGATTTGGCAAGAAAGATAAAAAAAATGTTGGTATACTTAAGCATCCTTAATAGAAACTAAACAGGTCTTAGATTGAAATTATC
ATAAATAAACCCTTTTTCACTTTAGCTGTAGTCCGGATGAAATCTTAGGGGCCTTGCTTAGCCTGCCCTGAAGCTTGCCAGTAAGTCGAAGCTGATGCTG
ACACAGCACTGACATAGATCCCAATGAACACAAGTTATTTCGAGAAGATAAAATACCAAGCAAAGCCAGGATTGCATTAAAAGATAGTGTATTTCTACTA
TACGATCTGAAAGCTTAATTTATTATATCAATAATCAAATTGGCAGCAGTGTGGAACAGATATAAAGAAGATGCTTCAAATCACAAATAACTACAGATAA
ATTGAAGGGTGAAGGTTCATTCAACCACCAAACACCCAAAATGTCGAGCTGCATATGCACAGAACCTAAGTACGGTACAGTTCATAGTTTGGGAAAATAG
ACTGCAAATGTGCACAGATAATACAGATTGTTACTCGATGAACAGTAAGCACGGTATTTGAAATCCTTCTAACCATCCATCCAGGCAGTGCCTCGTATAC
TCAAACCTCTTTTTGGAGGAAGCCAAACCATGTGATCCTGTTTCAAGTTCTCATCTTCTGAAAATGATCAGCAGTCAATAGATGTTTTAGTCCATTCTCC
AGAAAAAATGAGTGCCGCATGAATCCTTTCCGGTTTATAGTGAAACATTTTAGACGATGACCTTTAACATTCTAGAAAACGAAGAGAAAACCTACAGTCAT
CTAATTTCAGCTTAAACATGTTCCAGCAAGATCATCACCATCCTGACATGTTCCGTTTTGTCCCTAGACCCGAGCACAAGGTATTGCGAGGCTAATTTAG
CTCAATTCAGTTGTCAGAGCAATTGTGTGTGAAAAGTGTGAACAACAGCATAGGAGACAATACAGCAAGTGTAGCGTGCCAATATGTGTCCAGCTTGATA
GTTCACTATAATAAAGTTCTTTAATTCAAGTATTTGAATAATATAGAATCTCAACCTTCAAGTTTAAAAGGTTTTGTATTTGCTATTATAATCGTTGCCA
CCCCCTCACCCCTCTCAAAAAGAGAGTTACTTTGTAGCGCACTGTAAATCTCTTGATTGAAATAGTGGCATGTCATCTTATTTAATTCAGTTATTTCATA
AGGTACAATTTGTTTAGCTGACAGCTCAGACATGCTTCTATGCAACATGTTTATACCAATGCTGTATTTTGGCAATCCTGGATGTGGCATTACTTAACTG
TGCTTTCATATTCAATAGTTCTGGCAAAGGGAGGCTGCAAGCTTGAGACAACAACTGCACAACCTGCAAGAAAGTCATCGGTATGGCTAGAAAACCCTGC
AAATTTTACAAATGTACAAGTCAGAAAATTTGCCTAATGTAAAAGGATCAAACTACAGGATTTGTCAGCATGAGCACAAGTGCCTAACTTCCGAGTTAGA
TAACTCTGGAGGAAGGAAATCCTTCCTACTGTTTGTTTCAAGAATATACAAAAATTCAAAACAACATTAGAATGAACCAAAAGAAGAAGAAACAATATTCAC
CATGTAGCAATACTCAATTCCTGGTTGTCTTATTTATAAAGGTATAAAACTAGGTTCTGACGAGACAATTATGAATCAACTACACCATGTTACAGATCAT
CCATAACTCTTGGTGATGCAAATTAAGGGCTGTAAAGTGACAAGTATACCTCCACCAACTAATTTGTGCAGAGTGTATAAAATTGTAAAACAATGGATAA
GAATGAATTTAGTATACAAGATAGCATACAATGCCCAAGTTTAAGGAGACATACTGATTTACTGACTCCATAGGACAATCTTTTGCTTATGAGGACATCA
GTTTTCATAAACAATATCAATGTAAAAGGAGGTAACAGTTTATGAATTTCCTATGCCAAGCCATTTATAAGTCAAAAAATGTACCTATTTAGCTTTTATG
CATTCCAATTAACTTCCAAAGAAGAAGAGTACATACTAATGGAAAACAAATATGATATTATCATCTGTTTTGCCCGGTAGTTTCTTGAGTCTCTGATTTG
TCCGCACTTCACTCCCAACCAACACAACATTAATACACATGGGCAGAGGAATATCAGGTTATCAGGTAAACTGAATACAGTTTGATTGACTGCAGTCAGT
TGATGGGACAAGATCTTTCTGGATTGAGCGTCAAGGAACTCCAAAATCTAGAAAATCAGCTAGAAATGAGCCTACGTTGCATCCGGACAAAAAAGGTAAG
GATGCTAGCCATATTTCTGTTTTTCGTTACCAGATTGCCTCATCCAAGAATACAACTGATGATGACGTTTTTTACTTTCAGGACCAACTCTTGATTGATG
AAATTCACGAACTGAATCGAAAGGGAAGTCTCATGCAACAAGACAAGATGGAACTATACAAAAAGGTCAACCTAATCCGTCAGGAAAACATTGATTTATA
CAAGAAGGTATTTGATAAATCGTGAATGAAAATTCACAGGATATACACCCCATCCATTATTGACTGAGGTTTATTTCTCTACTCAGCTCTACGAGAAAGA
GGTGACAAGTGAAGTCAACCGAGATTCATCAACTCTAAACTTTGCAGTTGTCGAGAACGTCAACATTCCTGTTCATCTTGAACTTAACACTCCACCGCAA
GAAAATGATGTTGAGCAAACTGCACCTCCTAAACTGGGATTACAACTAAATCCATGAAGGCATGCAGCATGGCACCGCTTATGTTCATTATTCTCAACGG
TACGATTCAAAGCAACCAGAAACACTATTGGTTGTAAAGAGCTTAAACACATAGCAAGACTGACGCGGTATGCATGCCATGAGTGATTGATAATGGTAGT
GTAAATCTATTTCAAATAAAATGTATTTTATTGAAGAATACAGCAAATGATTTGTATTATAACTATTTGACACATTCTGCAAGTTTGTTGACCAACGTGA
GTACATGATAGCTACAAATCTTATATTGACAAAAAGATATGGTGTGTGGAAAAAATTCAGGTGCTATTTTTTTCTATTTTGAGAATTGTATGCATAGCAG
AAGAGCACAAATTGCATTCTGGTTTATCCGTGCAGGCATTCCAGAGTATCAGAATGGGGATCAACCTTTCTTCAGCTACATATGTAAATATACGGATGCT
AGTTGCTCACTTAAAATTTTGTAGTGTTCAAAGGAATAACAACAGTAAGACGTTGACGCACCGAAAATAACCAGAAATAACGGATTGTACTATGACAAGA
CACACGACAGATACACTAGCTTAAGTTTGTACAGGAAAATATTTGGCCTATTCTTATGTCACAGTAAATTAAGATTATCACCTTAATCACAGAAATTCAA
GGGGTAAACGATTATAGCACAAAACGATCCTGGACCCCAAGACCTGGGTCTCTGAACGGCTGAACCTCCAATTATACCTCTATACATTTGGAATGTGTGT
AGTCAAACTTTCGCTACTGGTATCTTTACAAATATTTACATTGAACACATCAAAATGATATACATGGATTCCTCTCGATATGTCTTTGAGGTTTGGGTTC
TAAAAATGATATACAAATTATTAGGTTCTAAACTTAAATGATATATACACATCAAAATGATATACAAATTATTGGGTTCTAAACTTAAATGATG
>Locus_14326_Transcript_27/49_Confidence_0.385_Length_2913
GTATTCTCATTTCCTCGCCGTATTTTCTCTCTCCAGCTCCAGACTCCAGAGTCCGGAGGAAGCAGAGAGAGAAGCTCGACGGGAAGGTAGAGAGAGATAT
AGTGCTCCTGATCCCTCTTTGAAATCCTCGATTGGTCAGTTCTGTTCCTGGATATACTGTTGCTGCAGATTGGATGCGTCTGCTTTCCTTTCGGGGAGTG
GTGTCTAGCTCTGTTGGTTCTTGCCTTCATAGTTAGTACTATGCCTTAATCTTGCTCCGCGATTGGTTTGATTCATGTTTTTCCTTTAATCTTGCTACCC
CAGGATTTTCCCCTTTGGTTTTCTTGGTTTGCTTCTCGGAAATCCGTGATTTTAACAGGATTTTTGGCTGGTGTTGCTCGATTTTTGTTCTTGACTGTGC
TTCCTTGGGATGAGCTTTTGCCTAATTCGACATTACAAGATGTGTTCTTTCTAGAAGTTAAATTGATCTTCTATGAGCCGGGCGCTGTATCGTGCATTAC
ATGGGGGTGGCGTACTGGTACTTTGGGCCGTAGAGTTGATGGAAATCTGCAGTCCCTTTTGTTGGATAAGGGGCTTTTTCCTTGGATAAGGAGGTTTGAA
GCTAATTTTGTGCGAATTGTGGCGGTTTCTGGATATCCTGGTTGAATTAGTGGTGTGATAGATTGTTCTAGAGAATAGGAAGATATATGTTTTATTGTTA
GAACATCAAGAGAGTGTTGAATCAGGTATGTCAATGTGTGAACTTATTGATGGGAGCTAAATGCTTGCTGACGTATCTTCGCTATAATGACCTAATCAAA
CCTAACAAGCACTAAATACTGATCAATTCTCAGTTATACAAAGATTTTCCAGAGAGTTCTTCAGAGCTCGAACTTCTGGGATGAAAATAGTTTGGCTCAA
GATAGTCCACTGGAAGGAGTTTAGGATATCTGTTTGAGCTCTGCCTGCAATATATGGAAATCTGAAGGTGATATGGTAGTAGAGTTGGGGACCCTGATCC
CCAAGCCAAAAGAAGTTCGGTAGAATTTCTCAGGAATATTGTGGTAGTAAGAAGCAAGTTAAAAGGAACCTAATTAGAAAAAAAGACATGCCTTGCTCGC
TCATGAGAATATAAAACAGGTATGCCACCTCCAGTCAGTACTTGATTAATTTCATTGAAAATGGAGGGCTGTAATAGATCCTTATCTATTAATGTGTTGT
ACCTAAACTTGGAAATCATCAGCTGAGGGCATTGTGCATGGTGCGTGGAGTACTAGCCCTCGGGAGATGCTTCTTAATTAGGCAGCGAGAAGGATAGAGA
TCGGTTTGACATATGTTCCATTTGTAGCTGTACTGGATATTTCTGCATGTTCCCCTGGAGCAGTTAGTTTCAGAATTATGATATTTCTACTTGTGGCAGG
ATTGTTCTGTTTCTATTATCTGTTCATATTTATGCTAGTCTGTCTTTTATGTTGTAATATCTATTGTCTATTGCACTTCAAGTGACTATGCATAACCATA
TTCCAGTGATTTAGTGAATGGTTATGAATTAAAGTAAGATGACGTTTTCATCAATTATCCTTGTTCTTTATCACGTCATTATCAATTCAGCTGATTAAAT
TCTGGCATCTTGTCATGATGGTTACTTTAGCTGATTTAATGTTTCTCAGAGCTAAGGATACCATCTCATAGTGCTCACTCTTTTGTGGGAAATGGAGGAT
CCCATGCCGAAGTCCAACAATGGCTTCTTGTCAGGAAATGGCTATCAGATGAAGCAATTTGGTCATAAAATCGACGATAGGGACTCGTCATCAGAGTCCG
GTCGGTCTCACCAAGAAGCTTCCACAGCGAGTGAGAACAGTATAAATGAACAGCACACCTCAACACAATCTGACAATGATGATGGTCATGGGAAGCATAA
TGAGGACATGATGAAGTCAGCAGTGTCCTTGGGGGAACCAGGATCAGCCTTTTTGGCCCCAAAACTAGATTACAGCCCATCTTTTGCTTGTGTTCCTTAT
ACTGCTGATGCTTTTTATGGTGGGGTCTTGACAGGATATGCTCCACATGCCATTGTTCATCCCCAGCAAAATGATTCAGCAAACTCTCGGGTTCCGTTGC
CTGTTGAACCTGTAGCAGAAGAGCCAATATATGTTAACGCAAAGCAATACCGTGCAATCCTTAGGAGGAGGCAGATACGTGCTAAACAGGAGGCTGAGAA
TAAGCTGGTGAAAGGTCGGAAGCCATACCTCCATGAATCTCGACACCGCCACGCAATGAAGCGAGCCCGTGGATCAGGGGGGCGGTTCCTCAACTCAAAG
CAGCTCCAGGAGCAGAACCAGCAGCAGCAGGCATCAGGTGGTTCAAGCTGCACAAAGGCCATCGGCAATAACACAAGCTCCCAAAGTGGTCCCAGTCACA
CGCCTCTGACACTTGCTTCCTCTGACACTGCAAGCGCTTCGAGGGCCAACCAGGAGCGCACCTGCTTCCCCTCGGCTGGCTTCCGTCCCACAATGAACTT
CGGCGCGCGAGGTGGAGACGCAAAGCTAGCTGTGAATGGCATACAGCAGCATGTTTCCTTCATCAGGTGAAGCAAGTGAAATGGGGCGCTGCCTGCGGTA
ACTCATCCTTGGCTTATGAAACTGTGAATGTGTAATGGACATGTAACTTCAGTCTCAGAATAACCAAACATATGGGAGTCCTCCTGAAGTTTCCTGTAGT
TCAAAGAACTGATAGGTTATGGCCTTATAGGTTTGCTTCTGGAGACTGAAATCCGTGCTTGATATACTATGTTGTACTGAGTTCTGAGGTGAAAGACATC
GGGAGAGTATCAGGTGCAAATGGCACAAATGTTGAAAACGGTGCAAATTCATCAAAATCTGTACCTAAAAGACTTCAATAAATTTTAAACTTTGCATAGC
TCTAGGTGCATTT
>comp78295_c1_seq20
ACAACTGCACAACTTGCAAGAAAGCCACAAGCAACTAATGGGTGAGAAGCTTTCTGGCCTAGGTGTGAGAGACCTCCAGAGTTTAGAGAATCGTCTTGAA
ATGAGCCTACGTAGTATCAGAACGAGGAAGGACAATCTTTTGAAAAGTGAAATTGAAGAGTTGCACAGGAAGGGTAGCCTAATTCACCAGGAAAACTTGG
AACTCTGTAGAAGAGTAAATACCATGTCACAACAAAATATGGATCTACAAAGAAAGGTTTGATGAACGATCTCATGCAGAATCCTTAACCAAATGAAGGT
GCATTGTCAGCATAAAGTTAAATTACTTAATTTCATCTCCAGGCGAGTGAACCAAGAGGTGTTGCCGATGCAAATAAAAGCTCTAGCACTCCCTACAGAT
CTTGTAGTGCACAAGATGCAGATATCACGGTTAATCTTGAATTGAGCCAATCACAGCAAAAAGAGGGGGAGCAGTGCCAAAGAGGGGCTCCAGAACTGGG
GTAAATATTCTTTTTCCAGAATCCAAAAACTGCATTACTCCAAGAAGTATCAAGAAGCCTAATGAAGAACCCATGCTTTGACATGTAACAGGCTTCAGCT

GCACAAAGAAGATGCACTACAAGCTGGAGAGTATCATGATGTTCCACCAATTCTAACATGCAATGCGGAGCACTTAAACTACAATTAGTATTTTGTGCAA
CATGTTAGATGAGTGAACAAAATGGTTTATGTACAGACTACAGATATGACCATCTATCTTATCTGTGGCTCTACTGTACTGAAATTGCAAGAAATGGTTA
TTGTAATGACTAATATGATACATTGACTGTCATACCGTTGACCTTGAAGATAGTTCTATAACGGAAAGGTCCAATTTGAAACAAGTGTTACGGATGAGCT
AGTAGGATTTTTTTCACAAACAATTAGATAGTCCACGTTAGTGGTAGCTGCTGGGTATGACATTTGACCCAGCCTGCCGACCCTCCCTTTCCCATGGGAG
TTCAATTAACATTAGGAATGAATATCGATATTATGGTTGATTATTTGAATCGTATGTTTTCCACCCTTAGATGTAAGGCCCTATTTTACTGTTTTAATGG
ATTAACAAGGTGAGCTTTCGAGATGGTAGCTCATTGATTCGCATGCATTTTAGTGGCTGTTAAGAGGTACTTAGTGCATCCATGCTTGAAGGTGTTGTTA
GTTCGACAAACCATTTTCTATCCTACTGGATTACTATCTTTTGGTCAGTATGACAAGCAAGAATTTAATCTGCCAATTAAGAATTTAATGTGTTCTTTTG
ACATGAATGTCAACAGCATTTAAGTAACTCAACTTTTAATACAAAGTACTGGAATGTTTTCTCACCGAAAGTAAAGATGTGGTTCCTTGATAGCAAACAA
TGTTTATGCCAATATCGCATTAAGGTGGTTGTATCCTTTAGAATGATCTCCTCATTCGTGCTTAATTTGATGCTATTGTCTTTTTACGGATGCCTAGATG
TGGTTTTGACCATTACTAAGTACTTTAATATATATTTAACAAAAATAATGACATAGCTGTTGTATGGCCAATCTAGATTTTGTTTTCGAAACATATTTGC
AGTCAAACTATAGGAAGTAAGAAACTTCAATGTGGTTCTCGAAAACCATGCTTAAAGTTTAGAAAGTTTGACTACATAGAGTATATACTACATAGTCTTA
AAGCATCAAATGAAGAGGAATGGAGGGAGTAGCAGACAATCATCATTCAATATCTATTTCTTGGTTGCCTTCAAATTTGGGCTTACTAAAATCGATTTAA
AATTCTCGTGTTCCAGTTGAAGCTCACTTCAAGTAAATCAGGAATTACCATATTTTTCATTGCTCATAATTTGGAAACATGGCATGATATTTGATAGAAA
ATGTACAAATCCTCAGCTGCTCAGAAAGTTACAAAGGGAGGAACGCCCATGAAAGAGAATTCATGGCTGCAACAACGAACATAAACACAGCCTATTCTGT
CCAGCAGTCATGGTAATACAAGCTAAAAAGGACCGCTCTGGAACAATAGCTTGAAAAGCAATATTAATACAATTTCAGATGAACTATGTGCGTCGATGTC
TTACTTGTGACTTCCTTGCAAGTAATGCGGTTCCTGCCTCATGCTTGGTGCCGCCGCATGCAATAAACCGATAACTAGTGAAGCAAGAGGATAACAGATG
TCAGAAAACTGAGCAAGCCATGTTAACTGTTTGAGAATTGTATACGGCTATGAGAAGTATATTTTTTTAGGGGGTGTTTCGATCCCTTTAGCTAATTTTT
TAGCTCTATATTTGGATGTTAAATAGGAGGACTGAACATGGGCTAATTATAGACTAATTGCAATTAGTGATTAGAGCCCATAAGCTCTAATTAGCCTATG
ATTAGCCCATGTTTAGCCCATTAGCACATGAGCTAATCATAAGCTAATTAGGGTTAATTAGCTCTAATCATCAATTAGCTAAAGCTAACTATAATTAGTT
CACAATTAGTCCATATTTAGTCCCCCTAATTGGGGTCCAAACATGGGGCTAAAAATTAG

>comp65473_c0_seq7
GCCAAGAAGAGCCGCCGCGGCCCGCGCTCCCGCAGCTCGCAGTACCGCGGCGTCACCTTCTACCGCCGCACCGGCCGCTGGGAGTCCCATATTTGGGATT
GCGGCAAGCAAGTGTACTTAGGTGGATTTGACACTGCTCATACTGCTGCAAGGGCGTACGATCGAGCTGCCATCAAGTTCCGTGGCTTCGACGCCGACAT
AAACTTCAATCTCAGTGACTATGAGGATGACATGAAGCAGATGAAGAGCCTGTCCAAGGAGGAGTTCGTGCACGTCCTGCGACGGCAGAGCACCGGGTTC
TCGCGTGGCAGCTCCAAGTACAGAGGCGTCACCCTGCACAAGTGCGGCCGGTGGGAGGCTCGCATGGGACAGTTCCTCGGCAAGAAGTACATATATCTTG
GGCTATTCGACAGCGAAGTAGAGGCTGCAAGGGCTTATGATAAGGCCGCGATCAAATGCAATGGTAGAGAAGCCATGACGAACTTCGAGCCTAGCACGTA
TGATGGGGAGCTGCTGACTGAAGTTGGTACTGAAGGTGCTGAAGTCGATCTGAACTTGAGCATATCTCAACCAGCTTCTCAAAGCCCGAAAAGGACAAG
AATTCCCTTGGTCTGCAACTCCACTATGGATCGTTTGAAGGCTCGGAATTGAAAAGAACAAAGATTGATGCTTCTTCTGAATTGGCGGGCCGCCCTCATC
GGTTCCCTCTTCTGACCGAGCATCCACCAATCTGGACTGCCCAGTCTCACCCCTTCTATTCAAATAATGAGAATGCATCAAGAGATCTTAACAGGAGGCC
AGATCAGGGGGGTGTTCCCAGCTGGGCGTGGAAGGTGACCGCCCCTCCTCCCACGCAGCCATTGCCGCTGTTCTCGTCGGCGTCATCATCCGCTGCAGCA
TCATCAGGATTCTCCAATACCGCCACGACAGCTGCCCACACCGCCCCATCGGCCTCCCTCCGGTTCGACCCGCCGCCGCCGCCGTCCAGCCGCCACCGCT
GAACGTTAAGAAGCCACGCTGTAAATTTGCCAGGAAGCCGACATTTTTTCCTCTCTCGGCGTTGCAACTTTTTAGGTTTTGCGCCGGGGTGGTTTCTTGT
AGTGGAGTGGATTCATAATTAACTGTATTTGCATGCTGCCCATGTGAAATGCTTCTCTCTCTTTTTACGCTCTCTGCACCGGATGTTCTGGGAGTTCTTG
TTGCCGGGAATTAACTAAATTCACCGTCTGAGATTGATCTATACATTGTTGTAGAGAATCGAATCATTGTTGAAATTAGTTACAGAATTATAGATATCAT
AAGGCACCCTCTACTTGTGGACAAAGTTCAGGAAGATTATTCCGGTTCTTTTCTTGGCTGTCAATTCGGCTCTTGTCCTTGGTGTGGAGCCACTTGCCTA
CTGTCTTGTTCTGTTGCTGTTTGTTTTTGGCTTCTTGCACAGTTACAAATTTGTTGGCTCGAATGGATGGTGAGACATT
>comp65375_c0_seq1
AAAACTAGAATCTGGAAATGTACTGAACTGGCCCCCAGTTGGAACAGGCCCTTCTAGATCATTGTTTGAAATGTTGAATTTGGAAAGGAAGTGAAGGTTC
GCCAGTGCAAAAGGGATTGCACTGTCAGATTGTTGTAGGACAAGTCTAGCACCTGCAGATTCGTGAGGTTGCCAATTGATTGCGGGACTTCGCCGTATAA
GTTGTTGAAGCTCAAGTTGAGTGAAAGGAGAGCTTTCAATTGACCAATCTGTGGGGGGATTACACCGGTGAATTTATTGTTGCCTAAATTCAGCATTTTA
GGGACAGAACTGGATGTAATATATTGATGGAATGGAGACAGATAAACAGGCATCTTAAGTATTATTGGGTCTGAGTGGGCTGACTTAAGCATTGAAATCT
CCATTAGCCCTGCCGGTATATCCCCGGTAAGACTGTTATTGGATATGTCCAGATAGAAAAGAAAGTTCAAACTATTGATCCAGCCCGGTATTGGTCCACT
GAGTTGATTGTCCCTTAAAATCAACGCTTCCAATTTTTTGAGCTTTGACAGCCAGTTAGGTAAACTTCCGTACAACGGACAGTCATTTATGACAAGACAC
ATAAGATTCTCAAAGCCATAAAATATATCATTTTCTGGAAGGGTCTCATGCTTGAAGTTATCCCCCATTATCAAGGAAGAGAGTACCCTTAAGCTACTGA
GGATCTAAAGCATGTTTGTGATATTTGTAAAGTTATTGTGAGCAAAACCCAGGAATCTGAGGGACTTGAGATTTCTGATTCTTGGTGAAAGTTCACCATG
GAATCTGTTGTAAGATAGCTGCAAGGCAACAAGGTTTCTGCAAGAGTAGAGGTTCTCAGGAACTATGCCAGTGAACTTGTTTGACTTAAAATCTAAATTT
TTTAGATTAGATAGGTTGGAGAAGTCGACATTTGTGAGCTCTCCTTGGAAATTGTTGCCCATGAGGGTGATGGTTGTGAGATTTGAGCATTCGCTCAGAG
CTGATGGCAGCTCTCCAGACATGTTGTTGTTGTCTAAGTGAAGCTCCTCCGTTGAGCAGGTTCTGGCCGGCCTTGAGCACTCTCAACACAGAGCAATTAC
CAAGCTCCGGTAGAATTCTACCATAAAACTGGTTGTAACTGAGGTCAAGGACCTCAAAGGATGACTTATCAACACATACCGTGCTCAGAATTTTCCCAGT
GAAGCTATTATTGCTCATATTGAGAGATGCCAATTTTTTCATGCTCTCCACTATGGTGAATGGAAACAATCCTGTGAACAAGTTGCTTGAGATGTTGATT
ACCTTCATAGCTCGGCCAGGGGTAGATGGTAGCTTGTGAAGGTCACCATTGAGCTTGTTAAAGCTGATATCAAGGACGATAATGCTCCTGGACAGCAGCA
GCTCTGGTGGGAGACCACCAGAGAATGAGTTGCCAGACAGGTTGAGGCTCAACAGGCCAGTAAGGTTACCAAGTGAGAGTGATATGTGCCCCTCGAGGCC
CATAGAAGCCAGTGATATATCCTTGACAGATCCATCCACGTTGCAAGTGATGCCGTCCCACAGGCAGCAGTTTGTTTCATTGCGCCATGAAGTGGCAAGG
CCATAGTCTTGCGACAGCCCACCAAGGAACTCGAGGAGGGTGGTCTTCTCATCTCGTTTGCAGGATCTGGCGGGAGATGCCAAGAAGATCAATAGTAGAA
GATCAAGACCAAGTAAAGATATGTGTCGTAATTTTGTTTTGATGTTGTTGCACGAAGAATGGCATGGTCTTTTCCATGAAACAACGTAGGACCTCCCGAA
GCCGTCCTGCTAAATAAAGCACCAACATTGGAATATTGAAA
>Locus_14087_Transcript_62/66_Confidence_0.198_Length_2151
TCTGTTCCAAATGCAATCGGAAACCCCAGGAATGGAGCTACGAGGCATGGATCTGTGAATTCATCTGGTGGCACTCTCACTTCTAGCTCAGAGCTAGGGC
ATGGTTCATCCAAGAGCTCCATATCGGCATCCATTGATTCGCCATCCAAAGTAGGGAACAACTTAGAGTTCGACTTTGCTGCTGTCGAGAGGCATGGTAA
GAACATGGGTAGTGATGGTAGTAGAGTTGAGGACTCAGGAACTTCTCCATCGTCAGCGTTCAACCATGGAGAGCCATTAATCAGCCTGAAGCTTGGAAAA
AGGGCTTACTTTGAAAATGTTTGCGGAGGACAGGATATCAAGAGCTCTGCACCTTCAACAGTGACTTCTCCATCAATGGTTGTCAAGAAGACCAAGATGT
CTCAGCAGAACGCACAAAGCTCAAACTGTCAGGTTGAAGGTTGCAGAGTTGATCTGTCTTCTGCTAAAGAATACCATCGCAAGCACAAAGTCTGTGAAGC
TCATTCTAAAGCTCCCAAGGTTGTTGTTGCTGGTGTAGAGCGCCGTTTTTGCCAGCAGTGTAGCCGGTGATATTTCCTTGACTGAGTGCATTTTCATTAT
ATAGTATGCCTCTTTGTATTCACCGAATCCTTTTTTAATGTTGGCATCTCTAATTGAAAAGAGAGAACATAAAAAATGTGATCAGTTGAAAGCAATACAA
CTATTTAGTCGTAGAACGACCAAGGACCAACTGCTAGATGATGCCCAATTATTTTGCATGACTGAGACTTCCAAAGTATCTGTGATGTTTATTACTCCTT
TGCTGATCAAGGTACACAAGAACACTGGAGTGACATGAACACTGAAGAAAATTGGCATTTCTTTTTGTTTCTGGCACTAGTTTGTTATTGATTTATGAAC
ACTGAAGAAAATTGGCATTTCTTTTTTGTTTCTGGCACTAGTTTGTTATTGATTTGTAAGCGGAATGCTGATAAACATCTTGAAAAGTGAAATTCAGATA
AACTAATTACAATACAGTTTGATCTTTTAGCGACATAATGGACATTTATTTCCTTTTCTTTAATTCTTCATTGGTTTGTAGGTTTCATGGTTTAGCTGAG
TTTGACCAGAACAAACGAAGCTGCCGTAGGCGTCTTACTCATCATAATGCACGGAGGAGAAAACCACAGGCAGATACAATTTCATTCAGTTCATCGCGGC
TCTCGACAATGTTTTATGATACAAACCAGCAGAAAAATCTTTTCTGTAGTCAACCTCATTTTGGCCAAGCGAGAAGCAATGCAGTTTTTTCTTGGGAAAA
CTCGGGAGATTCCAAATTTATGGAAACCAAACATCTGTTGATGCAGCCAACGAAAATGTTAGGTCTTGATGGGCTGCATTTCTCAACCCCCCCAGATATCA
AATAGTGTTGTGGCTCACTCTGTACATCATCGTAATTTCGATGGGCTCATGCCATTCAAGGGAACCAACACAAAGGTCCTCAACCAAGGCGTGGAAGCTT
CTGCGATCGCTTCCAACTCGAGTGGAGACCCGGATCTTGGGTGTGCTCTCTCTCTTCTGTCAAACGGTTCGTGGTGTTCAGGTTCAACTGTCATCCAGCA
GCCTAGTTCTCGTGTACACGCTGGTGCGGTGCCGCCCCTCGCTACTTTTGTGGTCTCTAATCCTGCAATGCATCCTCTGGACCCAGCCCCAGGAGGGTTC
TGGCAAGACGACCCTGCCTCGCTCGATAGAACTCCGGAGATTCAGGCATTCGGACACCTCTAACTATGGTCAAACAAATGTACTCATGCTGCATGCCCTC
GGAACAGCTGATTTAGTCACCTCATCATAATCTGCAACTCTGCTTTGCCAGTGGCAGATCTCTGCATAATGTCAGTCCTATCATCGGAGGCATTTGGTGA
GGTGAAGACTGAAGAATAATATAGGAGGCAACCCCCTGATCTTCCTGATTTAGGATGCATCTAGGATCCAGTTGTGTGTGTGCTTGTGGTTTTGCCAGAA

CCGGTTTTTTGTTTGCCGTCCCGTTAGAGAGAAGGGATCAAGTACATTCATGACTGCTCATGAGATTACTTGTTTCACTAATATTACATGATCTGGAGCG
AGATTTCGCTCGGTTCACGTTGATGGTCCTGCAACTTTTGTGTTTCATAGT
>Locus_12915_Transcript_4/30_Confidence_0.451_Length_4081
GCTCCTTGAGAAACACCATCCAATTACAAGACTATTTGATGCAACAATCACGCATATGGCTGCGCTAGAACTACTGCTCTGAATTAAGAGGCGTTGAAAT
ATTGTCCGGTCTACATATAGGCACTTGAGGTGTATTGACAGACAGTTCCCCTTAGTTTGCTACAACACATCAGGCGAATTCCACCAAATTAGGATGATGT
TTGTTTCTTAGACACAGGATAGCAATGCCAAAAGTTTTTTATAATTTTCCGCTTCACCGCAGATTTGGCATCTCAAAGGGAGTTGTGGCAAGATTCTCTT
GACCAAGCTTTCTTGCAGATTTGGCAAGAAAGATAAAAAAAAATGTTGGTATACTTAAGCATCCTTAATAGAAACTAAACAGGTCTTAGATTGAAATTATC
ATAAATAAACCCTTTTTCACTTTAGCTGTAGTCCGGATGAAATCTTAGGGGCCTTGCTTAGCCTGCCCTGAAGCTTGCCAGTAAGTCGAAGCTGATGCTG
ACACAGCACTGACATAGATCCCAATGAACACAAGTTATTTCGAGAAGATAAAATACCAAGCAAAGCCAGGATTGCATTAAAAGATAGTGTATTTCTACTA
TACGATCTGAAAGCTTAATTTATTATATCAATAATCAAATTGGCAGCAGTGTGGAACAGATATAAAGAAGATGCTTCAAATCACAAATAACTACAGATAA
ATTGAAGGGTGAAGGTTCATTCAACCACCAAACACCCAAAATGTCGAGCTGCATATGCACAGAACCTAAGTACGGTACAGTTCATAGTTTGGGAAAATAG
ACTGCAAATGTGCACAGATAATACAGATTGTTACTCGATGAACAGTAAGCACGGTATTTGAAATCCTTCTAACCATCCATCCAGGCAGTGCCTCGTATAC
TCAAACCTCTTTTTGGAGGAAGCCAAACCATGTGATCCTTTTCAACCCAAATACATATATTCAAGATGAAACTAACAAAAAGACAGTTGATAAAGAAACA
CGAAAGAGAGTAGAAAAAGGGTACCTGTTTCAAGTTCTCATCTTCTGAAAATGATCAGCAGTCAATAGATGTTTTAGTCCATTCTCCAGAAAAATGAGTG
CCGCATGAATCCTTTCCGGTTTATAGTGAAACATTTTAGACGATGACCTTTAACATTCTAGAAAACGAAGAGAAAACCTACAGTCATCTAATTTCAGCTT
AAACATGTTCCAGCAAGATCATCACCATCCTGACATGTTCCGTTTTGTCCCTAGACCCGAGCACAAGGTATTGCGAGGCTAATTTAGCTCAATTCAGTTG
TCAGAGCAATTGTGTGTGAAAAGTGTGAACAACAGCATAGGAGACAATACAGCAAGTGTAGCGTGCCAATATGTGTCCAGCTTGATAGTTCACTATAATA
AAGTTCTTTAATTCAAGTATTTGAATAATATAGAATCTCAACCTTCAAGTTTAAAAGGTTTTGTATTTGCTATTATAATCGTTGCCACCCCCTCACCCCT
CTCAAAAAGAGAGTTACTTTGTAGCGCACTGTAAATCTCTTGATTGAAATAGTGGCATGTCATCTTATTTAATTCAGTTATTTCATAAGGTACAATTTGT
TTAGCTGACAGCTCAGACATGCTTCTATGCAACATGTTTATACCAATGCTGTATTTTGGCAATCCTGGATGTGGCATTACTTAACTGTGCTTTCATATTC
AATAGTTCTGGCAAAGGGAGGCTGCAAGCTTGAGACAACAACTGCACAACCTGCAAGAAAGTCATCGGTATGGCTAGAAAACCCTGCAAATTTTACAAAT
GTACAAGTCAGAAAATTTGCCTAATGTAAAAGGATCAAACTACAGGATTTGTCAGCATGAGCACAAGTGCCTAACTTCCGAGTTAGATAACTCTGGAGGA
AGGAAATCCTTCCTACTGTTTGTTTCAAGAATATACAAAAATTCAAAACAACATTAGAATAGAACCAAAAGAAGAAACAATATTCACCATGTAGCAATAC
TCAATTCCTGGTTGTCTTATTTATAAAGGTATAAAACTAGGTTCTGACGAGACAATTATGAATCAACTACACCATGTTACAGATCATCCATAACTCTTGG
TGATGCAAATTAAGGGCTGTAAAGTGACAAGTATACCTCCACCAACTAATTTGTGCAGAGTGTATAAAATTGTAAAACAATGGATAAGAATGAATTTAGT
ATACAAGATAGCATACAATGCCCAAGTTTAAGGAGACATACTGATTTACTGACTCCATAGGACAATCTTTTGCTTATGAGGACATCAGTTTTCATAAACA
ATATCAATGTAAAAGGAGGTAACAGTTTATGAATTTCCTATGCCAAGCCATTTATAAGTCAAAAAATGTACCTATTTAGCTTTTATGCATTCCAATTAAC
TTCCAAAGAAGAAGAGTACATACTAATGGAAAACAAATATGATATTATCATCTGTTTTGCCCGGTAGTTTCTTGAGTCTCTGATTTGTCCGCACTTCACT
CCCAACCAACACAACATTAATACACATGGGCAGAGGAATATCAGGTTATCAGGTAAACTGAATACAGTTTGATTGACTGCAGTCAGTTGATGGGACAAGA
TCTTTCTGGATTGAGCGTCAAGGAACTCCAAAATCTAGAAAATCAGCTAGAAATGAGCCTACGTTGCATCCGGACAAAAAAGGTAAGGATGCTAGCCATA
TTTCTGTTTTTCGTTACCAGATTGCCTCATCCAAGAATACAACTGATGATGACGTTTTTTACTTTCAGGACCAACTCTTGATTGATGAAATTCACGAACT
GAATCGAAAGGGAAGTCTCATGCAACAAGACAAGATGGAACTATACAAAAAGGTCAACCTAATCCGTCAGGAAAACATTGATTTATACAAGAAGGTATTT
GATAAATCGTGAATGAAAATTCACAGGATATACACCCCATCCATTATTGACTGAGGTTTATTTCTCTACTCAGCTCTACGAGAAAGAGGTGACAAGTGAA
GTCAACCGAGATTCATCAACTCTAAACTTTGCAGTTGTCGAGAACGTCAACATTCCTGTTCATCTTGAACTTAACACTCCACCGCAAGAAAATGATGTTG
AGCAAACTGCACCTCCTAAACTGGGATTACAACTAAATCCATGAAGGCATGCAGCATGGCACCGCTTATGTTCATTATTCTCAACGGTACGATTCAAAGC
AACCAGAAACACTATTGGTTGTAAAGAGCTTAAACACATAGCAAGACTGACGCGGTATGCATGCCATGAGTGATTGATAATGGTAGTGTAAATCTATTTC
AAATAAAATGTATTTTATTGAAGAATACAGCAAATGATTTGTATTATAACTATTTGACACATTCTGCAAGTTTGTTGACCAACGTGAGTACATGATAGCT
ACAAATCTTATATTGACAAAAAGATATGGTGTGTGGAAAAAATTCAGGTGCTATTTTTTTCTATTTTGAGAATTGTATGCATAGCAGAAGAGCACAAATT
GCATTCTGGTTTATCCGTGCAGGCATTCCAGAGTATCAGAATGGGGATCAACCTTTCTTCAGCTACATATGTAAATATACGGATGCTAGTTGCTCACTTA
AAATTTTGTAGTGTTCAAAGGAATAACAACAGTAAGACGTTGACGCACCGAAAATAACCAGAAATAACGGATTGTACTATGACAAGACACACGACAGATA
CACTAGCTTAAGTTTGTACAGGAAAATATTTGGCCTATTCTTATGTCACAGTAAATTAAGATTATCACCTTAATCACAGAAATTCAAGGGGTAAACGATT
ATAGCACAAAACGATCCTGGACCCCAAGACCTGGGTCTCTGAACGGCTGAACCTCCAATTATACCTCTATACATTTGGAATGTGTGTAGTCAAACTTTCG
CTACTGGTATCTTTACAAATATTTACATTGAACACATCAAAATGATATACATGGATTCCTCTCGATATGTCTTTGAGGTTTGGGTTCTAAAAATGATATA
CAAATTATTAGGTTCTAAACTTAAATGATATATACACATCAAAATGATATACAAATTATTGGGTTCTAAACTTAAATGATG
>comp76332_c0_seq49
ACTGCTCTGAATTAAGAGGCGTTGAAATATTGTCCGGTCTACATATAGGCACTTGAGGTGTATTGACAGACAGTTCCCCTTAGTTTGCTACAACACATCA
GGCGAATTCCACCAAATTAGGATGATGTTTGTTTCTTAGACACAGGATAGCAATGCCAAAAGTCTTTTATAATTTTCCGCTTCACCGCAGATTTGGCATC
TCAAAGGGAGTTGTGGCAAGATTCTCTTGACCAAGCTTTCTTGCAGATTTGGCAAGAAAGATAAAAAAAAATGTTGGTATACTTAAGCATCCTTAATAGAA
ACTAAACAGGTCTTAGATTGAAATTATCATGAATAAACCCTTTTTCACTTTAGCTGTAGTCCGGATGAAATCTTAGGGGCCTTGCTTAGCCTGCCCTGAA
GCTTGCCAGTAAGTCGAAGCTGATGCTGACACAGCACTGACATAGATCCCAATGAACACAAGTTATTTCGAGAAGATAAAATACCAAGCAAAGCCAGGAT
TGCATTAAAAGATAGTGTATTTCTACTATACGATCTGAAAGCTTAATTTATTATATCAATAATCAAATTGGCAGCAGTGTGGAACAGATATAAAGAAGAT
GCTTCAAATCACAAATAACTACAGATAAATTGAAGGGTGAAGGTTCATTCAACCACCAAACACCCAAAATGTCGAGCTGCATATGCACAGAACCTAAGTA
CGGTACAGTTCATAGTTTGGGAAAATAGACTGCAAATGTGCACAGATAATACAGATTGTTACTCGATGAACAGTAAGCACGGTATTTGAAATCCTTCTAA
CCATCCATCCAGGCAGTGCCTCGTATACTCAAACCTCTTTTTGGAGGAAGCCAAACCATGTGATCCTTTTCAACCCAAATACATATATTCAAGATGAAAC
TAACAAAAAGACAGTTGATAAAGAAACACGAAAGAGAGTAGAAAAAGGGTACCTGTTTCAAGTTCTCATCTTCTGAAAATGATCAGCAGTCAATAGATGT
TTTAGTCCATTCTCCAGAAAAATGAGTGCCGCATGAATCCTTTCCGGTTTATAGTGAAACATTTTAGACGATGACCTTTAACATTCTAGAAAACGAAGAG
AAAACCTACAGTCATCTAATTTCAGCTTAAACATGTTCCAGCAAGATCATCACCATCCTGACATGTTCCGTTTTGTCCCTAGACCCGAGCACAAGGTATT
GCGAGGCTAATTTAGCGGGGCAATTGATTTTTTGCCACTATGATACAACTGCAATTACCCGCTGTCACTGACACGTGGGGTCATCTGAGTCACTGAAATG
TGGGCCCAATAGCATTTTGTTTGCCACTCACCCTTCCAGAGTGGCAAAAAGTTAATTGTCCCTAATTTAGCTCAATTCAGTTGTCAGAGCAATTGTGTGT
GAAAAGTGTGAACAACAGCATAGGAGACAATACAGCAAGTGTAGCGTGCCAATATGTGTCCAGCTTGATAGTTCACTATAATAAAGTTCTTTAATTCAAG
TATTTGAATAATATAGAATCTCAACCTTCAAGTTTAAAAGGTTTTGTATTTGCTATTATAATCGTTGCCACCCCCTCACCCCTCTCAAAAAGAGAGTTAC
TTTGTAGCGCACTGTAAATCTCTTGATTGAAATAGTGGCATGTCATCTTATTTAATTCAGTTATTTCATAAGGTACAATTTGTTTAGCTGACAGCTCAGA
CATGCTTCTATGCAACATGTTTATACCAATGCTGTATTTTGGCAATCCTGGATGTGGCATTACTTAACTGTGCTTTCATATTCAATAGTTCTGGCAAAGG
GAGGCTGCAAGCTTGAGACAACAACTGCACAACCTGCAAGAAAGTCATCGGTATGGCTAGAAAACCCTGCAAATTTTACAAATGTACAAGTCAGAAAATT
TGCCTAATGTAAAAGGATCAAACTACAGGATTTGTCAGCATGAGCACAAGTGCCTAACTTCCGAGTTAGATAACTCTGGAGGAAGGAAATCCTTCCTACT
GTTTGTTTCAAGAATATACAAAAATTCAAAACAACATTAGAATAGAACCAAAAGAAGAAACAATATTCACCATGTAGCAATACTCAATTCCTGGTTGTCT
TATTTATAAAGGTATAAAACTAGGTTCTGACGAGACAATTATGAATCAACTACACCATGTTACAGATCATCCATAACTCTTGGTGATGCAAATTAAGGGC
TGTAAAGTGACAAGTATACCTCCACCAACTAATTTGTGCAGAGTGTATAAAATTGTAAAACAATGGATAAGAATGAATTTAGTATACAAGATAGCATACA
ATGCCCAAGTTTAAGGAGACATACTGATTTACTGACTCCATAGGACAATCTTTTGCTTATGAGGACATCAGTTTTCATAAACAATATCAATGTAAAAGGA
GGTAACAGTTTATGAATTTCCTATGCCAAGCCATTTATAAGTCAAAAAATGTACCTATTTAGCTTTTATGCATTCCAATTAACTTCCAAAGAAGAAGAGT
ACATACTAATGGAAAACAAATATGATATTATCATCTGTTTTGCCCGGTAGTTTCTTGAGTCTCTGATTTGTCCGCACTTCACTCCCAACCAACACAACAT
TAATACACATGGGCAGAGGAATATCAGGTTATCAGGTAAACTGAATACAGTTTGATTGACTGCAGTCAGTTGATGGGACAAGATCTTTCTGGATTGAGCG
TCAAGGAACTCCAAAATCTAGAAAATCAGCTAGAAATGAGCCTACGTTGCATCCGGACAAAAAAGGTAAGGATGCTAGCCATATTTCTGTTTTTCGTTAC
CAGATTGCCTCATCCAAGAATACAACTGATGATGACGTTTTTTACTTTCAGGACCAACTCTTGATTGATGAAATTCACGAACTGAATCGAAAGGTTCTGG
AATATTCTGTTATTACTCTGCAATGAGAGGTCTCTGAGTGGATTTCAAGAAGCTGACTCTACTGGCTCATTTTCAGGGAAGTCTCATGCAACAAGACAAG
ATGGAACTATACAAAAAGGTCAACCTAATCCGTCAGGAAAACATTGATTTATACAAGAAGGTATTTGATAAATCGTGAATGAAAATTCACAGGATATACA
CCCCATCCATTATTGACTGAGGTTTATTTCTCTACTCAGCTCTACGAGAAAGAGGTGACAAGTGAAGTCAACCGAGATTCATCAACTCTAAACTTTGCAG
TTGTCGAGAACGTCAACATTCCTGTTCATCTTGAACTTAACACTCCACCGCAAGAAAATGATGTTGAGCAAACTGCACCTCCTAAACTGGGGTAACCCTG

CTTTTTTCTGCAAAGGAAGATTTTCTTGCAAGTTATGCAGTTGTTGTCTCTAGCTTGCCGCCTCCACTTGCAGTAACTGAGACGCCGGCGCCGAGCTTGA
GCTTGAAACCTAGACTACGGGGACAGGGTAGACAGGAGACGCGAAACCCCAAACCAGCGCAAAACGACGCGCAGGGAGGGAGTGTCACTGAAGGAGCGAC
AGGGAAAGCAGCGGCACGCCGGGGGAAATTCCGATCTGAGAGAACGGATCAGCCATGCATGACAGGAGCTGCGGAGGCAGCAGGGGGGGGGGGCTCCCGC
TGCCACGGGACAGTAGGGCACGCCAGATCGGTGAAATCCAGGCGAAGGACGAGGCGTGGGCGAGATTTACACGACGGCGCGATAGGATCTAGCCCGGATT
TGGATTCGACCGACGAGGGCGGAAGGAACGGCAGGGTGGTGCTCTTGTAGAGAGAGAAGGAGTCGGAGGGGGCGGAGAGAGAGAAAGGGGGCAGGTGGGG
TTTCAGGTAGTAAAATATGGAGCGG
>Locus_5717_Transcript_10/37_Confidence_0.237_Length_1208
GTTGCAATGGACATGAAAGCTAGGGGCAGGGGAGGTGAGGTGCCAGGTCGAGGGGTGCGGGATAGACCTCGGCGCCGCCAAGGAGTACCACCGGAAGCAC
CGCGTCTGCGAGGCCCACACCAAGTGTCCCCGCGTCATCATCGCCGGCCAGGAGCGCCGCTTCTGCCAGCAGTGCAGCCGGTTCCATGCACTCTCGGAGT
TTGATCAGAAGAAGAGGAGCTGCAGGAGGCGTCTGTCCGATCACAATGCCCGTCGGCGGAAGCCTCAGCCAGATGCATTTGCCTTTGCCTCTGCAAGGCT
ACCGTCATCCTTATTTGATGATAGGCGACAAATAAGTTTTGTCTGGAATAAAGCTCCTCTTAGCCATGTAAGACCTTTCACTTCTTCTACATGGGACAGC
TCATCTGACTTCAAGCTTCCACATGCGAAGGAAATAAGAGAGCTATCAACAGGAGTTGGGACTATTACTGGACAATTTCATCTGGATAAATCTCATCTGT
CCAATGCCATACCAACACTTAGCCATGGCAAAGATGAGCCGTTACCAATGAAAGGTCCGGACACATCTTTAACTGCTTCAAAATTCGATGGAGCACCGGA
TCTTCAGCGTGCTCTCTCTCTTCTGTCAGCTGGCTCTTGTGGATTGCCTGATCCTGTACGGCAAGCATCTCGTCTTATCCAGTTCTCTGGTGCCAGCGAG
AATAGTGGTGGCCTTCATTCATCACATGGAGGGAACTCTGCTCCTGTCTCATGCGCTGAGGAACAGCACATAGCACCTCAGTCTCAGCTAGTTCGTTTTA
CCATGGATGCCAACAGCAATGGCTACGAGTCCACTTACTTTGGTGTAAACCAGATAAATTAAGCGTGAAAGGCACACGCTTCTTGATCATTTGGAGACAG
CCACGAAGATGGCCTGGACTTATGCAAGAACAGCTTCTATCAACTACCAGAGTGCAAAACCCCTCTGTTAATGTTATCTACATATTTATTCGCCACCCCT
GAACTAAGTCGAACTGAGCGTAGGCCAGCTTGTATTGTGTACGCAGTTCCAGCCTGTGATTGACCCATTTTTTCTGTTCTTTTCCCCTTGATGTGCCTAC
TTTGAAACTTCGAAGTGTGATATTGCTACACTTGTACAACCCACTGCCAACTTTTGGATCTCTTCGAAACCTCGATGAATCTGAACATGTATTTTTTGTG
TGTGTATG
>comp78295_c1_seq11
ACAACTGCACAACTTGCAAGAAAGCCACAAGCAACTAATGGGTGAGAAGCTTTCTGGCCTAGGTGTGAGAGACCTCCAGAGTTTAGAGAATCGTCTTGAA
ATGAGCCTACGTAGTATCAGAACGAGGAAGGACAATCTTTTGAAAAGTGAAATTGAAGAGTTGCACAGGAAGGGTAGCCTAATTCACCAGGAAAACTTGG
AACTCTGTAGAAGAGTAAATACCATGTCACAACAAAATATGGATCTACAAAGAAAGGTTTGATGAACGATCTCATGCAGAATCCTTAACCAAATGAAGGT
GCATTGTCAGCATAAAGTTAAATTACTTAATTTCATCTCCAGGCGAGTGAACCAAGAGGTGTTGCCGATGCAAATAAAAGCTCTAGCACTCCCTACAGAT
CTTGTAGTGCACAAGATGCAGATATCACGGTTAATCTTGAATTGAGCCAATCACAGCAAAAAGAGGGGGAGCAGTGCCAAAGAGGGGCTCCAGAACTGGG
GCTTCAGCTGCACAAAGAAGATGCACTACAAGCTGGAGAGTATCATGATGTTCCACCAATTCTAACATGCAATGCGGAGCACTTAAACTACAATTAGTAT
TTTGTGCAACATGTTAGATGAGTGAACAAAATGGTTTATGTACAGACTACAGATATGACCATCTATCTTATCTGTGGCTCTACTGTACTGAAATTGCAAG
AAATGGTTATTGTAATGACTAATATGATACATTGACTGTCATACCGTTGACCTTGAAGATAGTTCTATAACGGAAAGGTCCAATTTGAAACAAGTGTTAC
GGATGAGCTAGTAGGATTTTTTTCACAAACAATTAGATAGTCCACGTTAGTGGTAGCTGCTGGGTATGACATTTGACCCAGCCTGCCGACCCTCCCTTTC
CCATGGGAGTTCAATTAACATTAGGAATGAATATCGATATTATGGTTGATTATTTGAATCGTATGTTTTCCACCCTTAGATGTAAGGCCCTATTTTACTG
TTTTAATGGATTAACAAGGTGAGCTTTCGAGATGGTAGCTCATTGATTCGCATGCATTTTAGTGGCTGTTAAGAGGTACTTAGTGCATCCATGCTTGAAG
GTGTTGTTAGTTCGACAAACCATTTTCTATCCTACTGGATTACTATCTTTTGGTCAGTATGACAAGCAAGAATTTAATCTGCCAATTAAGAATTTAATGT
GTTCTTTTGACATGAATGTCAACAGCATTTAAGTAACTCAACTTTTAATACAAAGTACTGGAATGTTTTCTCACCGAAAGTAAAGATGTGGTTCCTTGAT
AGCAAACAATGTTTATGCCAATATCGCATTAAGGTGGTTGTATCCTTTAGAATGATCTCCTCATTCGTGCTTAATTTGATGCTATTGTCTTTTTACGGAT
GCCTAGATGTGGTTTTGACCATTACTAAGTACTTTAATATATATTTAACAAAAATAATGACATAGCTGTTGTATGGCCAATCTAGATTTTGTTTTCGAAA
CATATTTGCAGTCAAACTATAGGAAGTAAGAAACTTCAATGTGGTTCTCGAAAACCATGCTTAAAGTTTAGAAAGTTTGACTACATAGAGTATATACTAC
ATAGTCTTAAAGCATCAAATGAAGAGGAATGGAGGGAGTAGCAGACAATCATCATTCAATATCTATTTCTTGGTTGCCTTCAAATTTGGGCTTACTAAAA
TCGATTTAAAATTCTCGTGTTCCAGTTGAAGCTCACTTCAAGTAAATCAGGAATTACCATATTTTTCATTGCTCATAATTTGGAAACATGGCATGATATT
TGATAGAAAATGTACAAATCCTCAGCTGCTCAGAAAGTTACAAAGGGAGGAACGCCCATGAAAGAGAATTCATGGCTGCAACAACGAACATAAACACAGC
CTATTCTGTCCAGCAGTCATGGTAATACAAGCTAAAAAGGACCGCTCTGGAACAATAGCTTGAAAAGCAATATTAATACAATTTCAGATGAACTATGTGC
GTCGATGTCTTACTTGTGACTTCCTTGCAAGTAATGCGGTTCCTGCCTCATGCTTGGTGCCGCCGCATGCAATAAACCGATAACTAGTGAAGCAAGAGGA
TAACAGATGTCAGAAAACTGAGCAAGCCATGTTAACTGTTTGAGAATTGTATACGGCTATGAGAAGTATATTTTTTTAGGGGGTGTTTCGATCCCTTTAG
CTAATTTTTTAGCTCTATATTTGGATGTTAAATAGGAGGACTGAACATGGGCTAATTATAGACTAATTGCAATTAGTGATTAGAGCCCATAAGCTCTAAT
TAGCCTATGATTAGCCCATGTTTAGCCCATTAGCACATGAGCTAATCATAAGCTAATTAGGGTTAATTAGCTCTAATCATCAATTAGCTAAAGCTAACTA
TAATTAGTTCACAATTAGTCCATATTTAGTCCCCCTAATTGGGGTCCAAACATGGGGCTAAAAATTAG
>comp76332_c0_seq28
ACTGCTCTGAATTAAGAGGCGTTGAAATATTGTCCGGTCTACATATAGGCACTTGAGGTGTATTGACAGACAGTTCCCCTTAGTTTGCTACAACACATCA
GGCGAATTCCACCAAATTAGGATGATGTTTGTTTCTTAGACACAGGATAGCAATGCCAAAAGTCTTTTATAATTTTCCGCTTCACCGCAGATTTGGCATC
TCAAAGGGAGTTGTGGCAAGATTCTCTTGACCAAGCTTTCTTGCAGATTTGGCAAGAAAGATAAAAAAAATGTTGGTATACTTAAGCATCCTTAATAGAA
ACTAAACAGGTCTTAGATTGAAATTATCATGAATAAACCCTTTTTCACTTTAGCTGTAGTCCGGATGAAATCTTAGGGGCCTTGCTTAGCCTGCCCTGAA
GCTTGCCAGTAAGTCGAAGCTGATGCTGACACAGCACTGTTTCAAGTTCTCATCTTCTGAAAATGATCAGCAGTCAATAGATGTTTTAGTCCATTCTCCA
GAAAAATGAGTGCCGCATGAATCCTTTCCGGTTTATAGTGAAACATTTTAGACGATGACCTTTAACATTCTAGAAAACGAAGAGAAAACCTACAGTCATC
TAATTTCAGCTTAAACATGTTCCAGCAAGATCATCACCATCCTGACATGTTCCGTTTTGTCCCTAGACCCGAGCACAAGGTATTGCGAGGCTAATTTAGC
GGGGCAATTGATTTTTTGCCACTATGATACAACTGCAATTACCCGCTGTCACTGACACGTGGGGTCATCTGAGTCACTGAAATGTGGGCCCAATAGCATT
TTGTTTGCCACTCACCCTTCCAGAGTGGCAAAAAGTTAATTGTCCCTAATTTAGCTCAATTCAGTTGTCAGAGCAATTGTGTGTGAAAAGTGTGAACAAC
AGCATAGGAGACAATACAGCAAGTGTAGCGTGCCAATATGTGTCCAGCTTGATAGTTCACTATAATAAAGTTCTTTAATTCAAGTATTTGAATAATATAG
AATCTCAACCTTCAAGTTTAAAAGGTTTTGTATTTGCTATTATAATCGTTGCCACCCCCTCACCCCTCTCAAAAAGAGAGTTACTTTGTAGCGCACTGTA
AATCTCTTGATTGAAATAGTGGCATGTCATCTTATTTAATTCAGTTATTTCATAAGGTACAATTTGTTTAGCTGACAGCTCAGACATGCTTCTATGCAAC
ATGTTTATACCAATGCTGTATTTTGGCAATCCTGGATGTGGCATTACTTAACTGTGCTTTCATATTCAATAGTTCTGGCAAAGGGAGGCTGCAAGCTTGA
GACAACAACTGCACAACCTGCAAGAAAGTCATCGGTATGGCTAGAAAACCCTGCAAATTTTACAAATGTACAAGTCAGAAAATTTGCCTAATGTAAAAGG
ATCAAACTACAGGATTTGTCAGCATGAGCACAAGTGCCTAACTTCCGAGTTAGATAACTCTGGAGGAAGGAAATCCTTCCTACTGTTTGTTTCAAGAATA
TACAAAAATTCAAAACAACATTAGAATAGAACCAAAAGAAGAAACAATATTCACCATGTAGCAATACTCAATTCCTGGTTGTCTTATTTATAAAGGTATA
AAACTAGGTTCTGACGAGACAATTATGAATCAACTACACCATGTTACAGATCATCCATAACTCTTGGTGATGCAAATTAAGGGCTGTAAAGTGACAAGTA
TACCTCCACCAACTAATTTGTGCAGAGTGTATAAAATTGTAAAACAATGGATAAGAATGAATTTAGTATACAAGATAGCATACAATGCCCAAGTTTAAGG
AGACATACTGATTTACTGACTCCATAGGACAATCTTTTGCTTATGAGGACATCAGTTTTCATAAACAATATCAATGTAAAAGGAGGTAACAGTTTATGAA
TTTCCTATGCCAAGCCATTTATAAGTCAAAAAATGTACCTATTTAGCTTTTATGCATTCCAATTAACTTCCAAAGAAGAAGAGTACATACTAATGGAAAA
CAAATATGATATTATCATCTGTTTTGCCCGGTAGTTTCTTGAGTCTCTGATTTGTCCGCACTTCACTCCCAACCAACACAACATTAATACACATGGGCAG
AGGAATATCAGGTTATCAGGTAAACTGAATACAGTTTGATTGACTGCAGTCAGTTGATGGGACAAGATCTTTCTGGATTGAGCGTCAAGGAACTCCAAAA
TCTAGAAAATCAGCTAGAAATGAGCCTACGTTGCATCCGGACAAAAAAGGTAAGGATGCTAGCCATATTTCTGTTTTTCGTTACCAGATTGCCTCATCCA
AGAATACAACTGATGATGACGTTTTTTACTTTCAGGACCAACTCTTGATTGATGAAATTCACGAACTGAATCGAAAGGTTCTGGAATATTCTGTTATTAC
TCTGCAATGAGAGGTCTCTGAGTGGATTTCAAGAAGCTGACTCTACTGGCTCATTTTCAGGGAAGTCTCATGCAACAAGACAAGATGGAACTATACAAAA
AGGTCAACCTAATCCGTCAGGAAAACATTGATTTATACAAGAAGGTATTTGATAAATCGTGAATGAAAATTCACAGGATATACACCCCATCCATTATTGA
CTGAGGTTTATTTCTCTACTCAGCTCTACGAGAAAGAGGTGACAAGTGAAGTCAACCGAGATTCATCAACTCTAAACTTTGCAGTTGTCGAGAACGTCAA
CATTCCTGTTCATCTTGAACTTAACACTCCACCGCAAGAAAATGATGTTGAGCAAACTGCACCTCCTAAACTGGGGTAACCCTGCTTTTTTCTGCAAAGG
AAGATTTTCTTGCAAGTTATGCAGTTGTTGTCTCTAGCTTGCCGCCTCCACTTGCAGTAACTGAGACGCCGGCGCCGAGCTTGAGCTTGAAACCTAGACT
ACGGGGACAGGGTAGACAGGAGACGCGAAACCCCAAACCAGCGCAAAACGACGCGCAGGGAGGGAGTGTCACTGAAGGAGCGACAGGGAAAGCAGCGGCA
CGCCGGGGGAAATTCCGATCTGAGAGAACGGATCAGCCATGCATGACAGGAGCTGCGGAGGCAGCAGGGGGGGGGGGCTCCCGCTGCCACGGGACAGTAG

GGCACGCCAGATCGGTGAAATCCAGGCGAAGGACGAGGCGTGGGCGAGATTTACACGACGGCGCGATAGGATCTAGCCCGGATTTGGATTCGACCGACGA
GGGCGGAAGGAACGGCAGGGTGGTGCTCTTGTAGAGAGAGAAGGAGTCGGAGGGGGCGGAGAGAGAGAAAGGGGGCAGGTGGGGTTTCAGGTAGTAAAAT
ATGGAGCGG
>comp87577_c0_seq4
AGCTCCAACACAGCAAGCTTTAGGCAGCAATAGGGTAGATCCAAGACTAGTGGTTGGACAGAATGGTCCAAATGCTGAAGCAGCAACAAACGCTCCTCCT
GCGCCAGCACTTGAAGCTGTGCAGCCAGTTGATCCGTGGGGTGATGTTGACCATCTCCTTGACGGATATGATGACCAGCAGAAGGCACTCATACAAAAGG
AAAGGGCAAGACGGATCTCAGAACAGCAAAATCTGTTTTCGGCAAGGAAACTATGCTTAGTCCTTGATTTGGATCATACACTACTCAATTCCGCAAAGTT
TGGAGAAGTGGACCCTGTTCATGAAGAGATTTTGCGAAAGAAAGAGGAACAAGACAGGTCTTTGCTAGAGCGCCATCTGTATCGTTTCCAGCATATGGGA
ATGTGGACCAAGCTGAGACCAGGAATATGGAACTTCCTTAAGAAGGCGAGTAAGCTTTT
>Locus_12915_Transcript_26/30_Confidence_0.706_Length_3421
GCTCCTTGAGAAACACCATCCAATTACAAGACTATTTGATGCAACAATCACGCATATGGCTGCGCTAGAACTACTGCTCTGAATTAAGAGGCGTTGAAAT
ATTGTCCGGTCTACATATAGGCACTTGAGGTGTATTGACAGACAGTTCCCCTTAGTTTGCTACAACACATCAGGCGAATTCCACCAAATTAGGATGATGT
TTGTTTCTTAGACACAGGATAGCAATGCCAAAAGTTTTTTATAATTTTCCGCTTCACCGCAGATTTGGCATCTCAAAGGGAGTTGTGGCAAGATTCTCTT
GACCAAGCTTTCTTGCAGATTTGGCAAGAAAGATAAAAAAAAATGTTGGTATACTTAAGCATCCTTAATAGAAACTAAACAGGTCTTAGATTGAAATTATC
ATAAATAAACCCTTTTTCACTTTAGCTGTAGTCCGGATGAAATCTTAGGGGCCTTGCTTAGCCTGCCCTGAAGCTTGCCAGTAAGTCGAAGCTGATGCTG
ACACAGCACTGACATAGATCCCAATGAACACAAGTTATTTCGAGAAGATAAAATACCAAGCAAAGCCAGGATTGCATTAAAAGATAGTGTATTTCTACTA
TACGATCTGAAAGCTTAATTTATTATATCAATAATCAAATTGGCAGCAGTGTGGAACAGATATAAAGAAGATGCTTCAAATCACAAATAACTACAGATAA
ATTGAAGGGTGAAGGTTCATTCAACCACCAAACACCCAAAATGTCGAGCTGCATATGCACAGAACCTAAGTACGGTACAGTTCATAGTTTGGGAAAATAG
ACTGCAAATGTGCACAGATAATACAGATTGTTACTCGATGAACAGTAAGCACGGTATTTGAAATCCTTCTAACCATCCATCCAGGCAGTGCCTCGTATAC
TCAAACCTCTTTTTGGAGGAAGCCAAACCATGTGATCCTTTTCAACCCAAATACATATATTCAAGATGAAACTAACAAAAAGACAGTTGATAAAGAAACA
CGAAAGAGAGTAGAAAAAGGGTACCTGTTTCAAGTTCTCATCTTCTGAAAATGATCAGCAGTCAATAGATGTTTTAGTCCATTCTCCAGAAAAATGAGTG
CCGCATGAATCCTTTCCGGTTTATAGTGAAACATTTTAGACGATGACCTTTAACATTCTAGAAAACGAAGAGAAAACCTACAGTCATCTAATTTCAGCTT
AAACATGTTCCAGCAAGATCATCACCATCCTGACATGTTCCGTTTTGTCCCTAGACCTGAGCACAAGGTATTGCGAGGCTAATTTAGCGGGGCAATTGAT
TTTTTGCCACTATGATACAACTGCAATTACCCGCTGTCACTGACACGTGGGGTCATCTGAGTCACTGAAATGTGGGCCCAGTGGCATTTTGTTTGCCACT
CACCCTTCCAGAGTGGCAAAAAGTTAATTGTCCCTAATTTAGCTCAATTCAGTTGTCGGAGCAATTGTGTGTGAAAAGTGTGAACAACAGCATAGGAGAC
AATACAGCAAGTGTAGCGTGCCAATATGTGTCCAGCTTGATAGTTCACTATAATAAAGTTCTTTAATTCAAGTATTTGAATAATATAGAATCTCAACCTT
CAAGTTTAAAAGGTTTTGTATTTGCTATTATAATCGTTGCCACCCCCTCACCCCTCTCAAAAAGAGAGTTACTTTGTAGCGCACTGTAAATCTCTTGATT
GAAATAGTGGCATGTCATCTTATTTAATTCAGTTATTTCATAAGGTACAATTTGTTTAGCTGACAGCTCAGACATGCTTCTATGCAACATGTTTATACCA
ATGCTGTATTTTGGCAATCCTGGATGTGGCATTACTTAACTGTGCTTTCATATTCAATAGTTCTGGCAAAGGGAGGCTGCAAGCTTGAGACAACAACTGC
ACAACCTGCAAGAAAGTCATCGTCAGTTGATGGGACAAGATCTTTCTGGATTGAGCGTCAAGGAACTCCAAAATCTAGAAAATCAGCTAGAAATGAGCCT
ACGTTGCATCCGGACAAAAAAGGTAAGGATGCTAGCCATATTTCTGTTTTTCGTTACCAGATTGCCTCATCCAAGAATACAACTGATGATGACGTTTTTT
ACTTTCAGGACCAACTCTTGATTGATGAAATTCACGAACTGAATCGAAAGGGAAGTCTCATGCAACAAGACAAGATGGAACTATACAAAAAGGTCAACCT
AATCCGTCAGGAAAACATTGATTTATACAAGAAGGTATTTGATAAATCGTGAATGAAAATTCACAGGATATACACCCCATCCATTATTGACTGAGGTTTA
TTTCTCTACTCAGCTCTACGAGAAAGAGGTGACAAGTGAAGTCAACCGAGATTCATCAACTCTAAACTTTGCAGTTGTCGAGAACGTCAACATTCCTGTT
CATCTTGAACTTAACACTCCACCGCAAGAAAATGATGTTGAGCAAACTGCACCTCCTAAACTGGGATTACAACTAAATCCATGAAGGCATGCAGCATGGC
ACCGCTTATGTTCATTATTCTCAACGGTACGATTCAAAGCAACCAGAAACACTATTGGTTGTAAAGAGCTTAAACACATAGCAAGACTGACGCGGTATGC
ATGCCATGAGTGATTGATAATGGTAGTGTAAATCTATTTCAAATAAAATGTATTTTATTGAAGAATACAGCAAATGATTTGTATTATAACTATTTGACAC
ATTCTGCAAGTTTGTTGACCAACGTGAGTACATGATAGCTACAAATCTTATATTGACAAAAAGATATGGTGTGTGGAAAAAATTCAGGTGCTATTTTTTT
CTATTTTGAGAATTGTATGCATAGCAGAAGAGCACAAATTGCATTCTGGTTTATCCGTGCAGGCATTCCAGAGTATCAGAATGGGGATCAACCTTTCTTC
AGCTACATATGTAAATATACGGATGCTAGTTGCTCACTTAAAATTTTGTAGTGTTCAAAGGAATAACAACAGTAAGACGTTGACGCACCGAAAATAACCA
GAAATAACGGATTGTACTATGACAAGACACACGACAGATACACTAGCTTAAGTTTGTACAGGAAAATATTTGGCCTATTCTTATGTCACAGTAAATTAAG
ATTATCACCTTAATCACAGAAATTCAAGGGGTAAACGATTATAGCACAAAACGATCCTGGACCCCAAGACCTGGGTCTCTGAACGGCTGAACCTCCAATT
ATACCTCTATACATTTGGAATGTGTGTAGTCAAACTTTCGCTACTGGTATCTTTACAAATATTTACATTGAACACATCAAAATGATATACATGGATTCCT
CTCGATATGTCTTTGAGGTTTGGGTTCTAAAAATGATATACAAATTATTAGGTTCTAAACTTAAATGATATATACACATCAAAATGATATACAAATTATT
GGGTTCTAAACTTAAATGATG
>comp67351_c0_seq25
GGCAAGACGGATTACGACGTGAGAGGCGAGAGTAGCGAAGAGTCACGGAGATCAGGGGAATCAAAAGATGGAAGTGTTGGTCAAGAAAACAACCATGCAA
CATCACATATGCCTGCTTTGGTGTCGGAGTATTTAGCACCATATACACAGCTGGAACTGAACCAATCAGTTGTGTGCAATCTTTTCTCCATGGACTCTGT
TTATTGCCTGTGTTTCCATTTTCTGTTGACTTGTCTCTAATGAGCTTATCGTCTCATGTTCACATCTTTCCTTGTGTAGGCTCCTGCAACATATCAGTAC
CCAGATCCTTACTATGCAGGCATGGTTGGTCCCTATGGAACTCAACCTGTGACCCATTTCCAGCTACCTGGGTTAACTCACCCTCGCATGCCATTACCTC
TTGAAATATCTGAGGAGCCTGTCTATGTAAATGCAAAGCAGTACCATGGAATTTTAAGACGAAGGCAGTCACGTGCCAAGGCTGAGCTTGAGAAAAAGGT
GGTTAAAGTCAGAAAGCCTTATCTTCACGAATCTCGCCATCAACATGCAATGAGGAGGGCAAGAGGAAATGGGGGTCGCTTCCTAAACACAAAGAAAAGC
GACAATGGTACTCCCAACGGTAAAGCTGAACCTAAGAAAGGAGAGCAAAACTCCAAGCATATCCATGTCCCTCCTGATTTGCAACTTCGACAGAAGGAGG
CATGAAGTAGCGGTCTGGCAACCTAAAACAGTGGCTTCTGTCCACAGGCATACACACTTTTGAGTCCGGATTTGGAGCCCCAGGGTTGCGCTGCCATCTT
TGCAGTTCGTCTTCTTTGTGCAAAGAGGTCCAAGGTTCGTCCACCCGAATGAGCTTGGTACCAGTTGTTAGTCCTGGCAGAATGCAATCTGGGTGCAGGA
GACCGTTGAAGTGTCCAATGCCATGGGTAGGTCTGCTGTTTTCAGGCAATTCATTCTTGGCTTTCACATTCCGCCCTTGTTTGTACCTGGTTTGTTGTAA
CTTGTAAGTGGAACTAGCTTGTGCTATCTTCGGCGCCCTGTGAAACATATGTATAATTAAGATAAACTTCGGATCATATTTGTATCCCGGATCATATTTG
TATCCTATGTCTGGCTTTCGTTCAGTTTGAACTGTAACAAGGCCAAAGCTGAACTCGTTAAACAGAGGTAGCAACTCATGTTGGCCTGTTGGGTGTTCAT
CAATGTTGTTGTAATGAATCCTGTACAAAGGCAGAAGCTTATTTCGA
>Locus_839_Transcript_88/261_Confidence_0.073_Length_2104
CAGGAAGGGGAACATGCTCCCTTCTCATCTCACAGGTCTTCCTCGCTGACTCATCTCTCTCCAGCCTCCCTGATCTGCGCTCTATTTCTTCCCTGTGGTG
TTAGTGTGAGCGGAGCTCCGCTCCCAGCCGAGCGGGTTTTCGAACCGTTTCGCGACTCTGGCTGGTTTATGGTGGATCTGGCTGATTTCTTGGTTGCTGG
GTTTGTTTTTGAGTTGATGCGGTGCTGAGATTTGTGGGTGCATTTCTGGGTGGAAGTCAGGTGATGGCTGCAGTGTGTGCCCTGTGCTAAGGCCCTGTTC
GATTTTGTTAAAGTACACCGCATGTTGGATATGCGCTTCTTAATTGTTTGTTTTGTTGGTTTCCTGTGTTCCTGCCTGTGTAATCCGTGTGAATTCACGG
GTGTCAGTTGTGCTTGGCTAGTTATGGTTAAGGGGAAAGGAGGTGATTTGGACAGGGAGCTTGCCACGCTGCAGCTTTGTTGGATTTAAGTAAAATCTAA
GTAGCAGAGATTTTGACATGCAAATTAGTAATGGTTCGCGAATTATTTCAAGAAAACAAAGTCTTACACATAATTGTTGCATGGGATTGTATGAATTGGA
GGTGGAAAGAACCAGTGGCCTTAAGATGCAGCGCCAATACGCATTTGGGCCTAGTGGCAAAACTAACTAGTCATGAAGGCTTGTTTCAGGCTTTCAGCAC
ATAATTCGATTGCACATATAGTTGTTGCGGTTGTGAAGAGACTTGGTTGCATGAACGATCCGAACTTTTAGGGTGTTACCGAGTTGATGCAGGGTACTTA
CTGATGCAAAATAATAGCTATCTTAGATCATATGCATGTCAGTTTGAGTGAATTTTTGTATGGTTTCATGTACCAGCGGCTGTGGATATTAGGCAGACTG
TATTTGTTTCAAATATTTTATCATTATATGTTGTTAGGACTGATCTGTGCTTATATTTGGACAGTATGAACTGAAAAACAGGAATAGAGAATTTCCGTGA
GATGAGACGTAAGGAAAATCTCCATGATACTGTTCAATTTGAATATTGCGAGTACTCTGTCCTTTTTCATGTCTTCGGTAGTTTAGTCTCATGCAGCTAG
AATTTTAACTCCTACGGTGCTCCTCTAGCAAAATGGCACCATAATGATTCAATTTGGTCATCAAATGCCTGATTACGACTCATCAGCTACCCAATCGACC
AGCGAGAGTCATCAAGAAGCGTCTGGAATGAGTGAAGGAAGCTTCAATGAGCATAATGATCGATCAGGTAATCTTGATGGTTACACGAAGAGAGATGAAA
ATAAGATGATATCAGCTTTATCTCTGGGCAATCCGGAAACTGCTTATGCACATCCAAAGCCTGACCGTAGCCAGTCCTTTGCCATATCATACCCGTATGC
TGATTCATTCTATGGTAGTGCAGTGGCGGCTTATGGCTCGCATGCTATTATGAATCCCCAGATTGTGGGCATGATGTCATCTTCTCGAGTGCCATTACCA
ATTGAACCAGCTGCTGAAGAGCCCATTTATGTAAATGCAAAGCAATACCATGCGATTCTCCGAAGGAGACAACTCCGTGCAAAGTTAGAGGCCGAAAACA
AGCTGGTGAAGAGTCGCAAGCCATACCTTCACGAGTCTCGGCATCAGCATGCCATGAAGAGAGCTCGGGGAACAGGTGGGCGGTTTCTGAACACGAAGCA
GCAGTCAGAGGCTGCTGGCGGCGGCACCTCGGACGCGCAACGCATACCAGCAAATGGTGGCCTGTTCAGCTTACCTCCCAGTGATCTCCACTATCGCGCG

AGAGGGGGCGCTTAAGGAGTCCCATGGCAACTCATCCTTGGCTTAAAAAAAATGTGTGGCGCTCAGCAATTCGACCTCTGCTAGTTCAATGTAAAGGTGT
GGGACTGTAACTGTGGGTGCTTGAATTGGCCGCAGCCATTCCTGCGACGCGTCGCAGGTGTTGCCGGTTCTGTGGTCGTGCTAGACTTGTGACCGGCACG
ACGAAACTGGCACCTGTTTCCTGTAACTGTTGTCTAAGATGATGATGACTACTCCAATAACCATTCTGTATGATGCTTTGACGTCGGTGTTGCTCCTTCA
AGTC
>comp73737_c0_seq5
TAACCAGAGGAAGGGACGGCGGGGAGGGGGCAGGCACAGAGAGCAGCTTTGCTTGGCGGACGCACCGAGGGAGGCGCCTGGGAGCCCTGCTTCTCCCCTC
TTCGTCTTCCTCCTCCGCTTCCAAAGGTGACTCCTTCGGGAAAATGGTTAATGATCATACGAGGTCAAATTTGGTTTTTGATAATAAGCAATCTCTATTT
GCAAGTCATAACATTGACTACGGCCAGCCAATAGCTTGTATATCATACCCATACAATGATTCGGGCTCAGGAGGTGTTTGGGCAGCCTTTGGGTCACGCA
CTAGCGCTGCAGCTGTGTTCCATCCCCAAATTGCTTGTGGGGGCACATCTGCGAGAGTTCCCTTACCTCTGGAATTAGCAGATGATGAGCCCATATATGT
CAATCCCAAACAATATCATGGTATACTTCGCAGAAGACAGCTACGTGCTAAGTTAGAGGCTCAGAACAAGCTAGTCAAAAACCGAAAGCCTTACCTTCAT
GAGTCTCGGCATCTTCATGCAATGAAGAGGGCAAGAGGTTCTGGCGGACGTTTCCTTAATACTAAACAGCTCCAGGAGCAGCAGCAGCTGAAGTCTCGCA
ATGCCTCCACCAGGTCCACCACAAATGGCGCAAATTCCTCAGGTTCAACACATCTACGGCTTGGTGGTGGTGCAGATGGAGATCAAACCATGTCGGGGAC
GAAAACAATGGCCTCACAAAACAATAGCAAAAAGGCTGTTTCTTCTTCTGCAGCTCTTGCTTTCACCGTGACTCCTATGGTGCGCAAAGATGACACCTTC
TTCCAGCACCTCAGCCACAATGTCAGCTTCTCCAGCCATTTTGGCCAGGCAAGCGCCCAAACCGGCGTTGGAGGCATCCATAATGAGACCCAGCATAGGG
TTTCCGTGATACAATGACGGTTTGCGAAGCTTATCTGGTGATCCAGGCTTCTAGGTGTCCTCGTGTCCGGTGTAGTCTTGGTCGCTCAGGCAATTCATCC
TTGGCTTAGTTTTTGGTGTGTTAGAACCTACAAATGTTTCATACTCTGTTGTGGTTTGCAGAGCCCATAAATCAGGGCTCTCCTAAAAAAAATCAGGGCT
TGATGGCGACACTACTATCCAAGTATTGTTGTAATGGTGTGTGTTAGAAACTCGCAAAAAACCCTTCTTTGTGCTCGTATTAGTACATTATGCTGCACAA
TTAGCTGCTATTCTGTTCGTACTACAGTCTGGTATGCAGTGGTTAGTGGTCCAGTTTA
>Locus_25981_Transcript_9/14_Confidence_0.095_Length_223
ATGGTTACCTGGCGGAGGTGCACTTGAATCATTACTGTGGTCAAGAGTCAAGACTAAGACTGATGCTTGAACTGTGGCTACTGCAGGAAGTGCCTATGGA
GAAACGACCCGAGTTGTGTCCCCAGTCGTTCCCTCCGTGTGCATGGCAAAAGCAGGGTTCCCCTGCGCCATCACTCCCTACTGCAGCATCATCAGGATTC
TCTACTGCCACCGGCGGCGCGGA
>Locus_5417_Transcript_3/28_Confidence_0.438_Length_5204
ATTACAAGACTATTTGATGCAACAATCACGCATATGGCTGCGCTAGAACTACTGCTCTGAATTAAGAGGCGTTGAAATATTGTCCGGTCTACATATAGGC
ACTTGAGGTGTATTGACAGACAGTTCCCCTTAGTTTGCTACAACACATCAGGCGAATTCCACCAAATTAGGATGATGTTTGTTTCTTAGACACAGGATAG
CAATGCCAAAAGTTTTTTATAATTTTCCGCTTCACCGCAGATTTGGCATCTCAAAGGGAGTTGTGGCAAGATTCTCTTGACCAAGCTTTCTTGCAGATTT
GGCAAGAAAGATAAAAAAAATGTTGGTATACTTAAGCATCCTTAATAGAAACTAAACAGGTCTTAGATTGAAATTATCATAAATAAACCCTTTTTCACTT
TAGCTGTAGTCCGGATGAAATCTTAGGGGCCTTGCTTAGCCTGCCCTGAAGCTTGCCAATAAGTCGAAGCTGATGCTGACACAGCACTGAAATAGATCCC
AATGAACACAAGTTATTTCGAGAAGATAAAATACCAAGCAAAGCCAGGATTGCATTAAAAGATAGTGTATTTCTACTATACGATCTGAAAGCTTAATTTA
TTATATCAATAATCAAATTGGCAGCAGTGTGGAACAGATATAAAGAAGATGCTTCAAATCACAAATAACTACAGATAAATTGAAGGGTGAAGGTTCATTC
AACCACCAAACACCCAAAATGTCGCGCTGCATATACACAGAACCTAAGTACGGTACAGTTCATAGTTTGGGAAAATAGACTGCAAATGTGCACAGATAAT
ACAGATTGTTACTCGATGAACAGTAAACACGGTATTCGAAATCCTTCTAACCATCCATCCAGGCAGTGCCTCGTATACTCAAACCTCTTTTTGGAGGAAG
CCAAACCATGTGATCCTGTTTCAAGTTCTCATCTTCTGAAAATGATCAGCAGTCAATAGATGTTTTAGTCCATTCTCCAGAAAAATGAGTGCCGCATGAA
TCCTTTCCGGTTTATAGTGAAACATTTTAGACGATGACCTTTAACATTCTAGAAAACGAAGAGAAAACCTAAAGTCACCTAATTTCAGCTTAAACATGTT
CCAGCAAGATCATCACCATCCTGACATGTTCCGTTTTGTCCCTAGACCTGAGCACAAGGTATTGCGAGGCTAATTTAGCGGGGCAATTGATTTTTTGCCA
CTATGATACAACTGCAATTACCCGCTGCCACTGACACGTGAGGTCACCTGAGTCACTGAAATGTGGGCCCAGTGGCATTTTGTTTTGCCACTCCCCTTCC
AGAGTGGCAAAAAGTTAATTGTCCCTTAATTTAGCTCAATTCAGTTGTCGGAGCAATTGTGTGTGAAAAGTGTGAACAACAGCATAGGAGACAATACAGC
AAGTGTAGCGTGCCAATATGTGTCCAGCTTGATAGTTCACTATAATAAAGTTCTTTAATTCAAGTATTTGAATAATATAGAATCTCAACCTTCAAGTTTA
AAAGGTTTTGTATTTGCTATTATAATCGTTGCCACCCCCTCACCCCTCTCAAAAAGAGAGTTACTTTGTAGCGCACTGTAAATCTCTTGATTGAAATAGT
GGCATGTCATCTTATTTAATTCAGTTATTTCATAAGGTACAATTTGTTTAGCTGACAGCTCAGACATGCTTCTATGCAACATGTTTATACCAATGCTGTA
TTTTGGCAATCCTGGATGTGGCATTACTTAACTGTGCTTTCATATTCAATAGTTCTGGCAAAGGGAGGCTGCAAGCTTGAGACAACAACTGCACAACCTG
CAAGAAAGTCATCGGTATGGCTAGAAAACCCTGCAAATTTTACAAATGTACAAGTCAGAAAATTTGCCTAATGTAAAAGGATCAAACTACAGGATTTGTC
AGCATGAGCACAAGTGCCTAACTTCCGAGTTAGATAACTCTGGAGGAAGGAAATCCTTCCTACTGTTTGTTTCAAGAATATACAAAAATTCAAAACAACA
TTAGAATAGAACCAAAAGAAGAAACAATATTCACCATGTAGCAATACTCAATTCCTGGTTGTCTTATTTATAAAGGTATAAAACTAGGTTCTGACGAGAC
AATTATGAATCAACTACACCATGTTACAGATCATCCATAACTCTTGGTGATGCAAATTAAGGGCTGTAAAGTGACAAGTATACCTCCACCAACTAATTTG
TGCAGAGTGTATAAAATTGTAAAACAATGGATAAGAATGAATTTAGTATACAAGATAGCATACAATGCCCAAGTTTAAGGAGACATACTGATTTACTGAC
TCCATAGGACAATCTTTTGCTTATGAGGACATCAGTTTTCATAAACAATATCAATGTAAAAGGAGGTAACAGTTTATGAATTTCCTATGCCAAGCCATTT
ATAAGTCAAAAAATGTACCTATTTAGCTTTTATGCATTCCAATTAACTTCCAAAGAAGAAGAGTACATACTAATGGAAAACAAATATGATATTATCATCT
GTTTTGCCCGGTAGTTTCTTGAGTCTCTGATTTGTCCGCACTTCACTCCCAACCAACACAACATTAATACACATGGGCAGAGGAATATCAGGTTATCAGG
TAAACTGAATACAGTTTGATTGACTGCAGTCAGTTGATGGGACAAGATCTTTCTGGATTGAGCGTCAAGGAACTCCAAAATCTAGAAAATCAGCTAGAAA
TGAGCCTACGTTGCATCAGGACAAAAAAGGTAAGGATGCTAGCCATATTTCTGTTTTTCGTTACCAGATTGCCTCATCCAAGAATACAACTGATGATGAC
GTTTTTTACTTTCAGGACCAACTCTTGATTGATGAAATTCACGAACTGAATCGAAAGGTTCTGGAATATTCTGTTATTACTCTGCAATGAGAGGTCTCTG
AGTGGATTTCAAGAAGCTGACTCTACTGGCTCATTTTCAGGGAAGTCTCATGCAACAAGACAAGATGGAACTATACAAAAAGGTCAACCTAATCCGTCAG
GAAAACATTGATTTATACAAGAAGGTATTTGATAAATCGTGAATGAAAATTCACAGGATATACACCCCATCCATTATTGACTGAGGTTTATTTCTCTACT
CAGCTCTACGAGAAAGAGGTGACAAGTGAAGTCAACCGAGATTCATCAACTCTAAACTTTGCAGTTGTCGAGAACGTCAACATTCCTGTTCATCTTGAAC
TTAACACTCCACCGCAAGAAAATGATGTTGAGCAAACTGCACCTCCTAAACTGGGGTAACCCTGCTTTTTTCTGCAAAGGAACTATTGATTCTGTTCTTA
ATTCTTTAGTGGCATATTGTTTACTACTAACTGAAAACTTGCAGATTACAACTAAATCCATGAAGGCATGCAGAATGGCACCGCTTATGTTCATTATTCT
CAACGGTACGATTCAAAGCAACCAGAAACACTATTGGTTGTAAAGAGCTTAAACACATAGCAAGACTGACGCGGTATGCATGCCATGAGTGATTGATAAT
GGTAGTGTAAATCTATTTCAAATAAAATGTATTTTATTGAAGAATACAGCAAATGATTTGTATTATAACTATTTGACACATTCTGCAAGTTTGTTGACCA
ACGTGAGTACATGATAGCTACAAATCTTATATTGACAAAAAGATATGGTGTGTGGAAAAAATTCAGGTGCTATTTTTTTCTATTTTGAGAATTGTATGCA
TAGCAGAAGAGCACAAATTGCATTCTGGTTTATCCGTGCAGGCATTCCAGAGTATCAGAATGGGGATCAACCTTTCTTCAGCTACATATGTAAATATACG
GATGCTAGTTGCTCACTTAAAATTTTGTAGTGTTCAAAGGAATAACAACAGTAAGACGTTGACGCACCGAAAATAACCAGAAATAACGGATTGTACTATG
ACAAGACACACGACAGATACACTAGCTTAAGTTTGTACAGGAAAATATTTGGCCTATTCTTATGTCACAGTAAATTAAGATTATCACCTTAATCACAGAA
ATTCAAGGGGTAAACGATTATAGCACAAAACGATCCTGGACCCCAAGACCTGGGTCTCTGAACGGCTGAACCTCCAATTATACCTCTATACATTTGGAAT
GTGTGTAGTCAAACTTTCGCTACTGGTATCTTTACAAATATTTACATTGAACACATCAAAATGATATACATGGATTCCTCTCGATATGTCTTTGAGGTTT
GGGTTCTAAAAATGATATACAAATTATTAGGTTCTAAACTTAAATGATATATACACATCAAAATGATATACAAATTATTGGGTTCTAAACTTAAATGATG
TATACACATCGAAATGATATACAAATTATTAGGTTCTAAAAATATACTATGGCAGAATTAGGTGGTCATTTGAGTTTTGGAGGCCTTGTCAATGTCCCAA
ACATCACTTATTTGTGACTGGGAGAGCAATGAATTGAAGGAATGAGAGTAACCATACGGTCATTGGTCAATTAAAAGGAACTTTAATATGCTATGACCAC
ATCGACCTTTCGTAAAGTAAATAACTCAATTATAACCAAGTAATTGGGTGTCTATTGAATCCATACGGTTCTCAAAACAGTTCTAAACAAAGTGAATTTA
AGCATCCTGAAAAGATATTTGGCAAATTTGCCATACCCTGAATTCACAGCATGTTAACACAAATCATGTGCAAATCATTCAGATCATTGACAAACGCACG
CATGCACACAAATCAACACCGTTTTCTCATGTATAGAACAGAAAAATTGAGAAACTTACGATTTTCTTGCAAGTTATGCAGTTGTTATCTCTAGCTTGCC
GCCTCCACTTGCAGTAACTGAGACGCCGGCGCCGAGCTTGAGCTTGAAACCTAGACTACGGGGACAGGGTAGACAGGAGACGCGAAACCCCAAACCAGCG
CAAAACGACGCGCAGGGAGGGAGTGTCACTGAAGGAGCGACAGGGAAAGCAGCGGCACGCCGGGGGAAATTCCGATCTGAGAGAACGGATCAGCCATGCA
TGACAGGAGCTGCGGAGGCAGCAGGGGGGGGGGGCTCCCGCTGCCACGGGACAGTAGGGCACGCCAGATCGGTGAAATCCAGGCGAAGGACGAGGCGTGG
GCGAGATTTACACGACGGCGCGATAGGATCTAGCCCGGATTTGGATTCGACCGACGAGGGCGGAAGGAACGGCAGGGTGGTGCTCTTGTAGAGAGAGAAG
GAGT
>Locus_12535_Transcript_38/73_Confidence_0.330_Length_2362

TTCCAATAAGACACAGATTCTTCAGAAAGAGGTTTTCCTCTCCCTGCCCTTTTGATCTCCAACTATGGCAGTGGTGACAAGGAGCAAATTTGCAATCTTA
GGGTGGTTCTCGTCGTGAAACCTGAAAGTTTGGGGAAATAAATTTGGGGGTGTGGATGTCATGATATGTAGGGTCCGGCACTGAAGTGGTTGATGCTGTG
GTTTCCAGGTTAAGGGGAGAAGAGGAATAGGATATAGATCTCAGGAAGCCAGAGATGAGGTCGAGGGGAAGCCAGTGGCCAAGCTTGTGCTCCAGTGAAG
CTGATTCTCTTGTTCCCAGTTGATCCATGCGCCGTGGCCTCAGTTCGCAGGATCAGCAGCTCCATGGTTCTGTGTGGTGCTGCAGTTTGAGAAGAAGGTA
CTTGGTCTTTTGCACCTTGATGTACTTGTGTTTTTTTGCCTTTCTTGATGCTATAATGATGGTGGTGCTGTTCTTGCCATGATTGACGATTCCCCTAGAA
AAGGTTGAACTACTCTGCACTCTCTCTGGCACATGGTAGCACCTCGTGTACTTCTCTGATGATGTATAGTTGAGTGCTCAGATATGTTGTTGCAAATCTG
TCCAATTTCTGCTACCCATATGTCAAATCTTTTCTGTTTTTAGTCTGTTTCACTCTTCCATTTTGTTGCTTCAAAGGTAGCTGCTTTTGCATACAATTAG
GAGCAGCAGTTGAGATATTCAATTCGGGCATGGGTTCTTTTGGGATGGACTGGAACCAGAAAGGCTCGGTGTTGTGGGATTGGGAGAATTTGCCGCCGAT
AGGCAGAAATGCAAACGAGAACCTCAAGATTGCGCCACAGGCTGAACCGAAGTTTGCAGGTGTTGAGGCAACAAGGCATGAATCGGGGCATTCTTCTTGT
GGTACTTTCTCTTCCAGCTCGGAGATGGGGTATGGTTCATCCAAGAGTTCCATATCAGCGTCGATTGATTCTTCACCCAAGGTGGGAAACAACGTGGAGC
TCAATCTTGCAGCTGTCAAAGTGCCTGACAAAAACACCGTCAAGAACACTGATTTGGGTAAAGTTGATGACGCTGGAACTTCTCCATCATCGATGATAGC
CGTCAGCAGTGGAGAACCGGTGATTGGCCTGAAACTTGGCCAAAGAACCTATTTTGAAGATGCATGCGGAGGGCAAAGTGTCAAGAGTTCACCATCCAAT
GTGAGTGCAGTGACCCCTGCTTCTGCGAAGAAAGCAAAGATGATTCAAAACGCACAGAACTCGTACTGTCAGGTTGAAGGTTGCAAGTTTGATCTCTCTT
CTGCTAAAGATTATCATCGGAAGCACCGAGTCTGTGAAACTCATTCTAAGGCTCCCAAGGTTATTGTTGCTGGTCTGGAGCGACGCTTTTGTCAGCAGTG
TAGCCGGTTCCATGCTTTAACTGAGTTCGACCAGAAAAAACGAAGCTGCCGGAGACGCCTCAATGATCACAATGCCCGCAGACGGAAGCCACAGCCTGAA
GCAATTCCTTTCGGTTCATCAAGGCTCTCTGCAATGTTTTATGATGCAAGGCAACAGACAAGTCTTCTGTTTGGTCAAGCTCCTTATGGTCAAATGAGAA
GCTGTGCAAACTCTTCATGGGATAGCCCAGTAGGAGGCTTCAAATTTGGAGAAACAAAAGCTCCTTGGTTAAAGCCAACGAGAGCTGCAGGTGTTGATGG
GATGCATGTATCAAGCCAGCAGGTGTGGAACAATATTACGCCACACGGTGCACATCATGATTTTAATGGTATCATGGCTTTCAAGGGAACCAGTGCAAAT
GTCCTTAATCAAGGTGCCGAAGCTTCTCCGGTCGTCTCCAACTCGAATGGAGCCCCAGATCTTCAGCGTGCTCTCTCTCTTCTGTCAAACAATTCAGCTG
GTGCTGCCAACGACCAGCCAACTCCTCAGCTGCACCCTGGGCTGACCACCTTCGTCGGCTCCTCCAACCCTGTCACTGTGATGGAAGCCTCACCACCAGG
ACTCTGGCAAGACAGCACGCCCCTTGATCATCAGGCGCAGTTCCAGGCTTTCGATCCCATTGCCGCACCGCAACAGCTCCAGCTCCCAAAACCCCCTTCC
TTCAACAGCTCCACTTCCAGCTATGGCCAGATGCAGTGATGGATGCTTCCTTCAGTTCTGCTGTCTGAAACTCTGAAATTTGAAACTGCTGTGTTCTAAA
AAATAAAGAAAAGCCTGTGTGCGAAAACTCAGGCTTGAAAAATTGATCCAGTAGTGTTCCCGTTCAATGGCTTGAATAATCCATCCTTGTGATGGCGTGT
AACTCAAATGCTTGGATTGCGCTGGCAAGTGATCATGGACTCTTGTGTTCTTGATGGCATCT
>Locus_12535_Transcript_53/73_Confidence_0.283_Length_2124
ATCTCCAACTATGGCAGTGGTGACAAGGAGCAAATTTGCAATCTTAGGGTGGTTCTCGTCGTGAAACCTGAAAGTTTGGGGAAATAAATTTGGGGGTGTG
GATGTCATGATATGTAGGGTCCGGCACTGAAGTGGTTGATGCTGTGGTTTCCAGGTTAAGGGGAGAAGAGGAATAGGATATAGATCTCAGGAAGCCAGAG
ATGAGGTCGAGGGGGAGCCAGTGGCCAAGCTTGTGCTCCAGTGAGCCTGATTCTCTTGTTCCCAGCTGATCGATGTGCCACTGCCTTGGTTCGCTGGATC
AGCGGATCTCCATGGTTCTGTGCGGTGCTGCAGATTGAGAAGAAGGTTTTCCAGGGGGAGGAATCTCTTGTGGGTTCCTTTTTCTGGGAGCAACGTAGTG
GGAGGTAGCTGCTTTTGCATACAATTAGGAGCAGCAGTTGAGATATTCAATTCGGGCATGGGTTCTTTTGGGATGGACTGGAACCAGAAAGGCTCGGTGT
TGTGGGATTGGGAGAATTTGCCGCCGATAGGCAGAAATGCAAACGAGAACCTCAAGATTGCGCCACAGGCTGAACCGAAGTTTGCAGGTGTTGAGGCAAC
AAGGCATGAATCGGGGCATTCTTCTTGTGGTACTTTCTCTTCCAGCTCGGAGATGGGGTATGGTTCATCCAAGAGTTCCATATCAGCGTCGATTGATTCT
TCACCCAAGGTGGGAAACAACGTGGAGCTCAATCTTGCAGCTGTCAAAGTGCCTGACAAAAACACCGTCAAGAACACTGATTTGGGTAAAGTTGATGACG
CTGGAACTTCTCCATCATCGATGATAGCCGTCAGCAGTGGAGAACCGGTGATTGGCCTGAAACTTGGCCAAAGAACCTATTTTGAAGATGCATGCGGAGG
GCAAAGTGTCAAGAGTTCACCATCCAATGTGAGTGCAGTGACCCCTGCTTCTGCGAAGAAAGCAAAGATGATTCAAAACGCACAGAACTCGTACTGTCAG
GTTGAAGGTTGCAAGTTTGATCTCTCTTCTGCTAAAGATTATCATCGGAAGCACCGAGTCTGTGAAACTCATTCTAAGGCTCCCAAGGTTATTGTTGCTG
GTCTGGAGCGACGCTTTTGTCAGCAGTGTAGCCGGTTCCATGCTTTAACTGAGTTCGACCAGAAAAAACGAAGCTGCCGGAGACGCCTCAATGATCACAA
TGCCCGCAGACGGAAGCCACAGCCTGAAGCAATTCCTTTCGGTTCATCAAGGCTCTCTGCAATGTTTTATGATGCAAGGCAACAGACAAGTCTTCTGTTT
GGTCAAGCTCCTTATGGTCAAATGAGAAGCTGTGCAAACTCTTCATGGGATAGCCCAGTAGGAGGCTTCAAATTTGGAGAAACAAAAGCTCCTTGGTTAA
AGCCAACGAGAGCTGCAGGTGTTGATGGGATGCATGTATCAAGCCAGCAGGTGTGGAACAATATTACGCCACACGGTGCACATCATGATTTTAATGGTAT
CATGGCTTTCAAGGGAACCAGTGCAAATGTCCTTAATCAAGGTGCCGAAGCTTCTCCGGTCGTCTCCAACTCGAATGGAGCCCCAGATCTTCAGCGTGCT
CTCTCTCTTCTGTCAAACAATTCAGCTGGTGCTGCCAACGACCAGCCAACTCCTCAGCTGCACCCTGGGCTGACCACCCTTGCCAGTACCTCCAACCCTG
TCACTTTGATGCAAGCCTCACCACCAGGACTCTGGCAAGATGGCGCCCCCCTCGATCATCAGGCGCGGTTTCAGCTTTTCGATCCGGTGGGTGACGGCAG
CGCCATTGTCACACCGCATCAGCTCCAGCTCCCAAAACCTCACTCCCTCAACAGCTCCTCTTCCCACTATGACCAGATGCACTGATGATCTGATGCTTCC
TTCAGTTAGCGCAAATGGCGGTTGGGTTGCGCAAAATTAGACATGGCTTCTGATGCCTGAAACTCTGAATTTTGAAACTGTTATGTTCTTCTGAAAAATA
AAAGCCTGTGTGCGCAAACTCAGGCTCCAAAAACTGATCTAGTAGTGTTCCCATTCAAGGGCTTGAATAATCCATCCTTGTGGTGGCATGTAACTCAAAT
GCTTCGATTGCGCCGGCAATTGAT
>comp79373_c0_seq27
CTGTCCTGCCCAGCAGCGCACAAGCAAGCTCGCAGCAGCGCCATTGCACGCTTCCGGTCGGCCGCTGCTGCGCTGCACGTCTGTCCTCCCGCCCCGTACC
GCCTCGTACACATGTAAACTGCTGTGCTGTAGGTTTGGAGCCCCCGTACAAGCGTGTTCGAGTTTGCTGATGTTCATCTAATTATCCCCGGGGAAGGAGT
GATTGTCCGCGGAGAGAAGGAAGAGATCGAATTGAAGGTCAAGATAAGTTATTGAAACGGCAAGATGGTTAATTTTGGAAAGAAGTTGATGACAGATCAA
GTAGAAGAATGGAAAGGGTACTATATCAATTACAAACTGATGAAGAAAATGTTAAAGCAATATGTTCAACAGACCCAATTAGGCGGGAAAGATTGTGAAC
AAATCCTTAAAGAGTTCTCGAGGATTCTTGATGACCAGATTGAAAGGATTGTGCTTTTTCTGCTACAACAACAAGGCCACCTTGCCAGCAGGATTGAGGA
ATTGGGAGAACAACGCACTGCTCTTATGGAACATTATGATATATCACAAGTTTCCCAGCTACGTGATGCGTATAGAGAAGTTGGGAGAGATCTTATTAAG
CTTCTGCGCTTTGTTGACATGAATGCTACTGGTATACGCAAGATACTAAAGAAATTTGATAAGCGTTTTGGCTATAAGTTTACATATTATTATGTCACCA
CTCGTGCAAATCATCCCTATTCTCAGCTTCAACAAGTATTTAAGCAAGTGGGAATTGTAGCTGTTGTAGGTGCATTATCGCGCAATCTTGAATATCTGCA
ACATCATCAAGGAAGCTTTGTATCCATCTATGATCATCCATCAGTTACCTTGAAGGACCCTATAATAGACGAAGTAAACCATGCGGTACAGAAATTTACG
CATGCCACCAATTTTATGAAATTCTTGGGACAACACGCGCTTATTGTTCCAGAGGATGCACGAAGCGGGTCTGAGGATCTTGTTGATGATCAGAGCTACC
ATTTCATGTCTCTGCTGCTTAACCTAGCGAACACTTTTCTTTACATGGTGAACACATATATCATTGTGCCAACTGCAGATGACTATTCAGTAAGCCTTGG
GGCTGCTGCGACTGTCTGTGGTGTAATTATCGGATCAATGGCAGTCACTCAAGTGTTCTCCTCGGTTTATTTCAGTGCATGGTCAAATAAGTCATACTTC
AGACCACTTGTATTCAGTAGCATTATGCTATTTTTTGGGAACCTGCTATACGCATTGGCATATGACCTGAATTCATTAATAGTTCTCCTGATTGGACGAC
TACTATGCGGGTTGGGCTCTGCAAGAGCAGTGAACCGTCGCTATATTAGTGATTGTGTGCCTCTCAAAATCAGGCTACAAGCTTCTGCAGGATTTGTCAG
TGCTAGTGCTCTTGGCATGGCATGTGGCCCTGCTCTTGCTGGTTTCCTCCAGACAAAATTTAAGATATACTCGCTCACTTTTAATCAGAGCACATTGCCC
GGATGGGTCATGTCCATTGCTTGGCTTCTTTACTTGTTGTGGCTGTGGTTTACATTCAAAGAGCCAGAACACTTCGCTAAAACTGTCATCAATGCACAGC
CGTCTGAATCAGGTCACCAAGGAAGTGCTAATTTGGAGGAAGGTCTAGCTCAACCATTGCTTCTGGGTACAGAACAAAGACAGGGCGAGAATGCGGATGA
CAATGATGATAATGAAGTAGACTCTGAAAACTCTCATGAACCAGCAACATCAATTACTTCAGCATACAGATTGCTGACACCATCTGTGAAGGTTTCCTAT
GCTTCCCTTATCGAGTACTCTTTTGTCTTGTGTTCCTCTCAAGCATGCCTAAAACAAAGTTCGCAGTCTAAATAGGGTTCTTTTGTAAAGAAAGTAAAGT
AAATGAACCGTGTAACATTAGGAAATGTATCTTAGTGTTAGTGATAATGATACTAGAAGGTACATCTGCAAGACACAAGCATGCACTGTACTTACACGCT
ATATCAAGTGAATTGAAACCAGATGAAAAACCTTCTTTTCATCTTTGGACCTTTGCATTTTCAATTGCCCTTGACCTCTTTCAGTGTATTTATAGGAGCT
ACCATCTTCTGTATCTTCCATTTCATATGTTAATAAGAGTGGCTATTTCTCAGGTCCAGCTATTGATATACTTTATGCTCAAGTATGCTATGGAAATTTT
ACTATCTGAATCAAGCGTTATCACAACATACTATTTTAGCTGGTCTACGAGTGCTGTGGCTATCTTTTTGGCAATTCTTGGATTAACTGTCCTTCCAGTA
AATGCCATTGTTGGAAGCTACATTACAAATTTGTTTGAGGACAGGCAAATTCTGTTGGCATCTGAAGTCATAGTTCTCATTGGTATCATCATGAGCTTTC
GTTTCACCCCTCACTACTCCATTCCACAATACGTCACTTCAGCTCTCATCACATTTGTGTTTGCTGAGGTACTCGAAGGAGTGAATCTGTCCTTGCTCTC
ACGAGTAATGTCGTCTAGGCTTTCGCGAGGGACCTACAACGGTGGACTCCTCTCGACAGAGGCCGGGACATTGGCCCGTGTAATTGCAGACGCGACTATT
ACTGCAGCAGGTTATCTAGGCCCGGACCTCCTCCTCAACGTCACCCTGCTGCCACCTCTTGTGATCTGCGTAGTCTCCATCGTTGCAACACTCTACACTT
ACAACACTCTGTACTGAAGAAGCAGGCCTCCCTTTGTAGCTGTATAGCACCATTCCCAGTAAAAATGATTCCGGCCCACATTGGGTTGTGTACTTGTACC
AGCATTTATGGTTTGTAAAAAGTCAATGAAATGCATTTGTAGTGTTCAATGAATTGTGTAATTTTTCATCTTGTAATGATTTATGTGCCACGATTGCCAG
TAGTTGTATTAGTGCATTGTGGTCAATTTATTTGTTT

```
>comp86229_c0_seq1
CGGGAGCGCGGCGACGAGCTCGGAGGCGACGCCGCGGACGCCCGGGACAAGCTGGCCTCCTTCGCCAAGACGCTCACGCAGTCCGACGCCAACAACGGCG
GCGGGTTCTCGGTGCCGCGCTACTGCGCGGAGACCATCTTCCCCAAGCTGGACTACCGGGCCGACCCGCCGGTGCAGACGGTGCTCGCCAAGGACGTGCA
CGGGGAGGTGTGGAAGTTCCGGCACATTTACCGGGGCACGCCGCGCCGGCATTTGCTCACCACGGGGTGGAGCACGTTCGTGAACCAGAAGAAGCTCGTC
GCGGGGGATTCCATCGTGTTCTTGCGCACCGAGCATGGCGAACTGTGCGTTGGGATACGGCGTGCGAAGCGGGTGTCTTGTGGTGGCATGGAGTGCATGT
CAGGGTGGAACGCCCCGGGGTATGGGGGATTCTCGGCGTTCTTGAAGGAGGAGAGTAAACTGATGAAGGGTCCAGATGGGTACATGAGGGGCAGGGGGAA
GGTGAAGATTGCGGATGTCGTGGATGCGGCAGGCCTAGCGGCGAGCGGGCAGCCTTTTGAGGTGGTGTACTACCCGAGAGCTAGCACGCCGGAGTTTGTT
GTGAAAGCCGCATCAGTGCAGAATGCGATGAGGATCCAGTGGTGCCCTGGGATGAGGTTCAAGATGGCATTTGAAACGGAGGATTCATCAAGGATTAGCT
GGTTTATGGGGACAATATCTTCTGTTCAGGTTGCTGATCCAATCCGATGGCCGAATTCACCTTGGCGGCTTCTTCAGGTTTTTACTTGATCGAAAGTTTT
TCATAGTTGAATACTTATATTCCATACTGCATTATAGACTTTTGTCTTGAGTGGGTTACTACTTGCTAGTATGATGTGTCTGGTATGTACGGGTGTGTCA
CTATTCTTCAGAAGTTCTTTTCCCTTCATATGTTGGCTCAGCAACTTGCTCAGATTACATCTAGCAAATGAATTGGTACTTGGAAGAGTAGAAAAAAGAA
TATAGAAAAGAAAAACTATTGGGGATTTGTGATGATTGGTAGCAAAATAGTCATGAAGGAATGGTTTTAGTTGTATGGACCGTTATTGGTTCTTTAAACT
TAGTGTTTATCTTTTCACTAAGTTGCTGTCCTCTGCCAAATAATTCGCTTTAAAACTTTTTACTGTTCCTTTTGCAATTACAAATAGCATCCAAAATTCC
TTCCTTGCAGCCTCAGACAGTCCGACTAGCTCGGACATCACATGTTTGTAGCCTTTAGCATAGCCCACATGATGCATCCAAAAGGCATTGGGTTACTTGA
CAAACTTGCAGTTAGAAATGGGATGGCTGAGGTATGCAGACATTCTAAGGTTGGCCGAAAAATTAAAGGTCTTAATTGTGTGGACTAGGATGAGAACAAA
TTGGACATAATTCTAAGAAAAAGGAAAAAGAGTACTACTTTACCTATAGGAAACCATGCTATCTGTTGGGTTGGTTTCTTGTTTGGATCTCGTTCTCTTT
GAATTGTTAGTATGTTTCATCAGTCTCTTGTGGTTACTGAGAACTGGGATGTATCATCATAAGCTTCCGAAAGCCCATTGGTCACCATTATCACCGTTGA
GTAGCCATTGACAGATTCAGGTTTGAGCATCCTTTGGCTGCTTAGCTTTCTGTCTCTACACACTTGAAAAGCTGGTAAGCGTCATTAATGTGACATCTTC
CCTAACTAATACTCACAAGAGGGAGGGAGGGAGATGAACTTGTATTGGTACAGAACTTTCAAAGCTTGCTATTGACTTCGCGAAAAGAGACAGCTGCTGA
ACAAGCAAAAAGGATGACCACCGATTTGGCACAACTACTCGAGCACTCCCATAGCACCAATAAAATTTTCCTACCATCAGACTACCTTTGCTTCTCACTA
TCCTGGTTTAATAATTTCATCACATAAGAGCCCAATGAATCAATTATTGATTTTATTTTGTTAGCCTCAGGAGGCATTATAAACACATTGAATTTAGGGC
TCAATACCAGTTTTGCTTTTTTAAATACATTAATTGATCTCTCTCTTAAAGCCCAAATTTCTCTATGTTTTGTAGCCAAATTAAAGCCTACTGATAGACT
GATATTACATAGTTATAGCTCAAATGTGAGGAGTGTACTCTTAAAACGTTCGTGGACAACAACCAACATAATTTTTAAACTGTGGTTCTGTTGGTCCTGA
ACTCCTGAAGTAGTCGAACCTTTTCCCACTTTGATGGCAGTACCTTACACTTGTCTTTATGATCAACCCCCTGATGTGATTGCTAGCGGATCCTTAAAAG
CAGCCTATAGCTAGCATTGTACATTTGTACCGTGGGAGAGAGGTATTGAACTAGCAGTAATAAGACTGATCTAGGATTAATTTAACCTTGTTTTGCTAGC
TCTATTGTCAAATGTACCCTAGGACATTTTGGCTCTTTATCTCTCTCTTCACCAATAATATATATTGATTGGTATTCTTGGAAGTGCCTCGGTATATACT
TTTGTCTGGCAGTGTAGTAATACGAAGGGTGGATTCTTACCACTTACATGTGTTAGGTGTGATATTGTAAACTCATCAGCTCATAGTAGCTACGATGTCA
TCATTGTAGCCGATATAGAGTCATCAGTTACTTGTGAAATCGATATAATAAATTGTCTGTAATTGCGAGCTTCCTTGCTTCATTGTTTGTCCTTTACCTC
TTTTCTCTTTCATATAGTTGCAAATTTCTTACAGGTGACATGGGATGAACCAGATTTGTTGCGGAATGTGAAATGTATCAGCCCATGGCTTGTGGAACTC
GTATCAATCATTCCCCCAATTCACCTGGGACCATTTTCTCCACCTAGAAAGAAGTTGCGGGTGCCCCAACATCCTGACTTTCCATTTGATGGTCAGCTGT
TGAATCCAATTTTCCACGGCAACCCACTTGATCCTAGCAACAGCCCCCTATGCTGTTTCCCGGACAACGCTCCTGCAGGCATACAGGGAGCCAGGCATGC
GCAATTCGGTCCATCCTTCTCGGATCTCCACATCGGCAACCTGCAGCAAAGCCTGCTGTTCTCCGGCATCCTCTTCCCTGACCACCACGCCCCTCACCCT
CCGAGGATCAGCACCGACCTGACAATTGGCAGCCCACACGCTGGCCAAGACGCGGCGTGCTCCCTATCGTCTGGCGCCAAGAAGCCTGACGACGTCAAGG
CGACGAGACTAGTGCTCTTTGGACGGGCGATACTGACGGAGGAGCAGATCAAGGCCAACGCTTCCGTTGGCCCGTCCTCGCTGGGAGCCACCGGGAACGG
CTCGTCCAAATCCGACTGCGACGCCGAGAAAGCACCTACCAACACGTCCGAAGGCTCCAGCTCAGGTGTCATCCAGGGCAGTCCTACCAAAAACAGCTCG
TCCTCCTGGAGACTGCAGTGGTTTGGCGACAATGGCAGTCAGTCACCATCTGAGTTCGGGCTGGAACCTGGCCAGTGCAAGGTGTTCGTGGAGTCGGACG
CCGTCGGCCGGAACCTTGATCTCTCGGCGCTGGGCTCGTTCGAGGAGCTGTACACCCGCCTGTCTGACATGTTTTGCGTTGACAACGCTGAGCTGAGGAG
CCATATGCTATACCGCACCGCCGCTGGCGAGGTCAAGCACGTCGGCGATGAGCCTTTCAGTGCGTTTGTGAAATCAGTGCGGAGGATCATGATACTGACA
GATGCCGGCAGTGACAACATAGGGAGTTAGGAATGAAGCGATCGGCCGAATAACTTAGGGCACTACTCCTAGGCAGCAATAAGCCGAACTACATGCATGT
GTACAGCCGAACGAGCTGCGGTTTCTTCTTCGATCAGGCCTAGAGAATTCAGTCAGGAAATTAAGCTGACGCAGTGTTCAGTGCTTCAGTTAGGTTGTTT
TCCCACCCTTTTTCATTTAGTTCAGTAAAGCTCTTTTAGTCATGCAAACAAATTGTAATCTTGATCATCAAACGCATTTCTTAAATCTTGTTTCCGTTTT
GGATTTCAAACCTTGAGCCGGGCCACAGAGACAAGAGCAGCAGGTGAAAAGGGTTAAATGCCATTGCTGGCTGTTGGAACATTTACTAGTCACCCACTGA
AACTTCTGAATCTGGGGTTACTTGATCAGACTCCCAGCTCCAGGCATTTCATTTGTTCCCTTGTTTGTCTGGGCAGGCAGATACTGTGCGCTGTGTTGCG
TATCCCTGTTACCTCTGATGCATTGATGCCTCCAGGGCCCTGTCCTTTGCCTGCATAGGGGAAATAATTGTGCTGCTTGCCCATTTCGCCTCAGAATATT
GTTGCATGAAATGGACATCTCAGCTAACACAACCAGAGGAAACAGTGTCCGCAGTAGTGGCAGTGCT
>Locus_1016_Transcript_1001/2070_Confidence_0.002_Length_4336
GTTTCAAGCTCAAGCTCGGCGCCGGCGTCTCAGTGAACCACTCTGCTGGGGGTGTACTTCAATTTGTTGTTGCCAGTGTTATTAGCAGTCACTGCAGCTC
AAGTAGCAGGCTGGAAGCTTTTAATCTGACATTTGTTCTTTCCTTTTCTGATAATTACTGCAAGTGGAGGCGGCAAGCTAGAGATAACAACTGCATAACT
TGCAAGAAAATCGTAAGTTTCTCAATTTTTCTGTTCTATACATGAGAAAACGGTGTTGATTTGTGTGCATGCGTGCGTTTGTCAATGATCTGAATGATTT
GCACATGATTTGTGTTAACATGCTGTGAATTCAGGGTATGGCAAATTTGCCAAATATCTTTTCAGGATGCTTAAATTCACTTTGTTTAGAACTGTTTTGA
GAACCGTATGGATTCAATAGACACCCAATTACTTGGTTATAATTGAGTTATTTACTTTACGAAAGGTCGATGTGGTCATAGCATATTAAAGTTCCTTTTA
ATTGACCAATGACCGTATGGTTACTCTCATTCCTTCAATTCATTGCTCTCCCAGTCACAAATAAGTGATGTTTGGGACATTGACAAGGCCTCCAAAACTC
AAATGACCACCTAATTCTGCCATAGTATATTTTTAGAACCTAATAATTTGTATATCATTTCGATGTGTATACATCATTTAAGTTTAGAACCCAATAATTT
GTATATCATTTTGATGTGTATATATCATTTAAGTTTAGAACCTAATAATTTGTATATCATTTTTAGAACCCAAACCTCAAAGACATATCGAGAGGAATCC
ATGTATATCATTTTGATGTGTTCAATGTAAATATTTGTAAAGATACCAGTAGCGAAAGTTTGACTACACACATTCCAAATGTATAGAGGTATAATTGGAG
GTTCAGCCGTTCAGAGACCCAGGTCTTGGGGTCCAGGATCGTTTTGTGCTATAATCGTTTACCCCTTGAATTTCTGTGATTAAGGTGATAATCTTAATTT
ACTGTGACATAAGAATAGGCCAAATATTTTCCTGTACAAACTTAAGCTAGTGTATCTGTCGTGTGTCTTGTCATAGTACAATCCGTTATTTCTGGTTATT
TTCGGTGCGTCAACGTCTTACTGTTGTTATTCCTTTGAACACTACAAAATTTTAAGTGAGCAACTAGCATCCGTATATTTACATATGTAGCTGAAGAAAG
GTTGATCCCCATTCTGATACTCTGGAATGCCTGCACGGATAAACCAGAATGCAATTTGTGCTCTTCTGCTATGCATACAATTCTCAAAATAGAAAAAAAT
AGCACCTGAATTTTTTCCACACACCATATCTTTTTGTCAATATAAGATTTGTAGCTATCATGTACTCACGTTGGTCAACAAACTTGCAGAATGTGTCAAA
TAGTTATAATACAAATCATTTGCTGTATTCTTCAATAAAATACATTTTATTTGAAATAGATTTACACTACCATTATCAATCACTCATGGCATGCATACCG
CGTCAGTCTTGCTATGTGTTTAAGCTCTTTACAACCAATAGTGTTTCTGGTTGCTTTGAATCGTACCGTTGAGAATAATGAACATAAGCGGTGCCATGCT
GCATGCCTTCATGGATTTAGTTGTAATCTGCAAGTTTTCAGTTAGTAGTAAACAATATGCCACTAAAGAATTAAGAACAGAATCAATAGTTCCTTTGCAG
AAAAAAGCAGGGTTACCCCAGTTTAGGAGGTGCAGTTTGCTCAACATCATTTTCTTGCGGTGGAGTGTTAAGTTCAAGATGAACAGGAATGTTGACGTTC
TCGACAACTGCAAAGTTTAGAGTTGATGAATCTCGGTTGACTTCACTTGTCACCTCTTTCTCGTAGAGCTGAGTAGAGAAATAAACCTCAGTCAATAATG
GATGGGGTGTATATCCTGTGAATTTTCATTCACGATTTATCAAATACCTTCTTGTATAAATCAATGTTTTCCTGACGGATTAGGTTGACCTTTTTGTATA
GTTCCATCTTGTCTTGTTGCATGAGACTTCCCTGAAAATGAGCCAGTAGAGTCAGCTTCTTGAAATCCACTCAGAGACCTCTCATTGCAGAGTAATAACA
GAATATTCCAGAACCTTTCGATTCAGTTCGTGAATTTCATCAATCAAGAGTTGGTCCTGAAAGTAAAAAACGTCATCATCAGTTGTATTCTTGGATGAGG
CAATCTGGTAACGAAAAACAGAAATATGGCTAGCATCCTTACCTTTTTTGTCCGGATGCAACGTAGGCTCATTTCTAGCTGATTTTCTAGATTTTGGAGT
TCCTTGACGCTCAATCCAGAAAGATCTTGTCCCATCAACTGACTGCAGTCAATCAAACTGTATTCAGTTTACCTGATAACCTGATATTCCTCTGCCCATG
TGTATTAATGTTGTGTTGGTTGGGAGTGAAGTGCGGACAAATCAGAGACTCAAGAAACTACCGGGCAAAACAGATGATAATATCATATTTGTTTTCCATT
AGTATGTACTCTTCTTCTTTGGAAGTTAATTGGAATGCATAAAAGCTAAATAGGTACATTTTTTGACTTATAAATGGCTTGGCATAGGAAATTCATAAAC
TGTTACCTCCTTTTACATTGATATTGTTTATGAAAACTGATGTCCTCATAAGCAAAAGATTGTCCTATGGAGTCAGTAAATCAGTATGTCTCCTTAAACT
TGGGCATTGTATGCTATCTTGTATACTAAATTCATTCTTATCCATTGTTTTACAATTTTATACACTCTGCACAAATTAGTTGGTGGAGGTATACTTGTCA
CTTTACAGCCCTTAATTTGCATCACCAAGAGTTATGGATGATCTGTAACATGGTGTAGTTGATTCATAATTGTCTCGTCAGAACCTAGTTTTATACCTTT
ATAAATAAGACAACCAGGAATTGAGTATTGCTACATGGTGAATATTGTTTCTTCTTTTGGTTCTATTCTAATGTTGTTTTGAATTTTTGTATATTCTTGA
AACAAACAGTAGGAAGGATTTCCTTCCTCCAGAGTTATCTAACTCGGAAGTTAGGCACTTGTGCTCATGCTGACAAATCCTATAGTTTGATCCTTTTACA
TTAGGCAAATTTTCTGACTTGTACATTTGTAAAATTTGCAGGGTTTTCTAGCCATACCGATGACTTTCTTGCAGGTTGTGCAGTTGTTGTCTCAAGCTTG
```

CAGCCTCCCTTTGCCAGAACTATTGAATATGAAAGCACAGTTAAGTAATGCCACATCCAGGATTGCCAAAATACAGCATTGGTATAAACATGTTGCATAG
AAGCATGTCTGAGCTGTCAGCTAAACAAATTGTACCTTATGAAATAACTGAATTAAATAAGATGACATGCCACTATTTCAATCAAGAGATTTACAGTGCG
CTACAAAGTAACTCTCTTTTTGAGAGGGGTGAGGGGGTGGCAACGATTATAATAGCAAATACAAAACCTTTTAAACTTGAAGGTTGAGATTCTATATTAT
TCAAATACTTGAATTAAAGAACTTTATTATAGTGAACTATCAAGCTGGACACATATTGGCACGCTACACTTGCTGTATTGTCTCCTATGCTGTTGTTCAC
ACTTTTCACACACAATTGCTCTGACAACTGAATTGAGCTAAATTAGCCTCGCAATACCTTGTGCTCGGGTCTAGGGACAAAACGGAACATGTCAGGATGG
TGATGATCTTGCTGGAACATGTTTAAGCTGAAATTAGATGACTGTAGGTTTTCTCTTCGTTTTCTAGAATGTTAAAGGTCATCGTCTAAAATGTTTCACT
ATAAACCGGAAAGGATTCATGCGGCACTCATTTTTCTGGAGAATGGACTAAAACATCTATTGACTGCTGATCATTTTCAGAAGATGAGAACTTGAAACAG
TGCTGTGTCAGCATCAGCTTCGACTTACTGGCAAGCTTCAGGGCAGGCTAAGCAAGGCCCCTAAGATTTCATCCGGACTACAGCTAAAGTGAAAAAGGGT
TTATTCATGATAATTTCAATCTAAGACCTGTTTAGTTTCTATTAAGGATGCTTAAGTATACCAACATTTTTTTATCTTTCTTGCCAAATCTGCAAGAAA
GCTTGGTCAAGAGAATCTTGCCACAACTCCCTTTGAGATGCCAAATCTGCGGTGAAGCGGAAAATTATAAAAAACTTTTGGCATTGCTATCCTGTGTCTA
AGAAACAAACATCATCCTAATTTGGTGGAATTCGCCTGATGTGTTGTAGCAAACTAAGGGGAACTGTCTGTCAATACACCTCAAGTGCCTATATGTAGAC
CGGACAATATTTCAACGCCTCTTAATTCAGAGCAGT
>comp46215_c0_seq1
AAATTTTTGCTGGTTCTGCCCACAAAATTGAACTATCTGTGTAACTTGCACTTAAGGGTTGCCTCACAGTTAAATGATATAGATTCAACTGATGTGAGAA
TTCCCATCGTATGAGACAAATGTGATGTCCCCAAATCGACATCTTAGTAATACTATACTAGATAAATCCTTATCTTAGGTTGCTCAGCTATAATTCAGTT
GTGGTGGCGCCTTTTATATGTCTGATCTACATTGAGCAAACTACCTCTGTGTCTGGTACTTTAATCAATGCAAATATTATCCTACAGTCATTTGTCCTAT
TTCTTTGGAAAATTTATATTCAGTGGGCCCGCAACCCGCTTTTCTAATGATTGATTACCTTGCCTAGAAGATCAAATTGAGATCACCATACTACCTGGCA
GACTTTATCAAGTGCTCCTCGGTGTCTCAAGAAGTTGGTAGGGTGCCGTATTGCCATATATCTGTTGGAACCATTATACTGTGGTCCAGATGTCCAACAG
GAATTGTACTAGTTTTCTGTGTTTGAGAGGTCCTGAATGTGATAACTTTATTTAAAATGAAGCCCCATGCAACCGATTTGTTTAACTGACACTCTAAAAT
CTGAGTGGTCCAGCTGTGTGCTTTTATTTTTGAAGGCAGGTGATTGCTATTATTGAATATTGAGTGATAAAACATCTGGATCTTTCTTATCTGGCTATGA
GTAGAATTTCAGCCCCATTTGAATTATGCATGCACATTAACTTTTAGTATAGCATTTGTTCAGAAAATTTTGTACAGCAACATCCATGGTGATGGTCCTG
ACTCCTGAGAGACTATTTGTCTCTGTTTTCCTTGAAATTCTCCTGATGTTTATTTCCTAATATAGCAGTTTTGTGGAGTTCCTTTTGCTGCATAACAATG
TGTGCCAATATAAAATGGCAGCAATTAATAATCCTAAAGAATATCCTATTGTTTCGATTTCTTGTCATGAATTTTGCTTCTTTAGGTTTCACTTACTTGC
GGAGTTTGATGAGGCTAAGCGTAGCTGTAGAAAGCGGCTTGATGGGCACAATCGACGTCGCAGGAAGCCACAGCCAGATACCATGAACTCTGGGAGCTTT
ATAACAAGTCAGCAAGGCTTCTTTTCGAGTGCAGGGACAAGGTTTTCGTCGTTCCCTGCTCCAAGACCGGAGCCAAGCTGGTCCGGAGTGATCAAATCCG
AGGACACTGCGTCCTACACGCACCAGGTCGTCAGCAACAGGCCTTCGTACTACACGCACCAGGTCCTCAGCAACAGGCCGCACTTCGCCGGCTCCACGTC
GGCCTACTCCAAGGACGGCCGGCGCTTCCCCTTCCTCCAGGACGGCGATCAAGTGAGCTTCAGCGCAGGCGCGGCGGCGTGCCAGCCCCTCCTCAAGACC
GTGGCCCCGCCGCCCGAGAGCAGCAGCAGCAACAAGATCTTCTCCGACGGGCTCACCCCCGTGCTCGACTCGGATTGTGCTCTCTCTCTTCTGTCATCCC
CAGCCAACTCCTCCAGCGTCGACGTCAGCCGGATGGTCCAGCCGACCGAGCACATCCCCATGGCGCAGCCCCTCGTCCCCAGCCTGCAGCAGCTCGGCAG
CTCCCCCGGCAGCTGGTTCACCTGCTCCCAGACCTCCGGCGGAGGCGGCGCCTCCGGCGCGTTCACCTACCCCCGCATGGGAAG
>Locus_3441_Transcript_416/417_Confidence_0.028_Length_1752
TGTAAATCTCTTGATTGAAATAGTGGCATGTCATCTTATTTAATTCAGTTATTTCATAAGGTACAATTTGTTTAGCTGACAGCTCAGACATGCTTCTATG
CAACATGTTTATACCAATGCTGTATTTTGGCAATCCTGGATGTGGCATTACTTAACTGTGCTTTCATATTCAATAGTTCTGGCAAAGGGAGGCTGCAAGC
TTGAGACAACAACTGCACAACCTGCAAGAAAGTCATCGGTATGGCTAGAAAACCCTGCAAATTTTACAAATGTACAAGTCAGAAAATTTGCCTAATGTAA
AAGGATCAAACTACAGGATTTGTCAGCATGAGCACAAGTGCCTAACTTCCGAGTTAGATAACTCTGGAGGAAGGAAATCCTTCCTACTGTTTGTTTCAAG
AATATACAAAAATTCAAAACACACATTAGAATGGAACCAAAAGAAGAAACAACATTCAGCATGCAGCACTACTCAATTTCCGGTTGGAATGTGCACTTGA
AATCTCAGTCTTATTTATGAAGATAAAACTAGGTTCTGACGAGACAATTATGAATCAACTACACCATGTTACAGATCATCCATAACTCTTGGTGATGCAA
ATTAAGGGCTGTAAAGTGACAAGTATACCTCCACCAACTAATTTGTGCAGAGTGTATAAAATTGTAAAACAATGGATAAGAATGAATTTAGTATACAAGA
TAGCATACAATGCCCAAGTTTAAGGAGACATACTGATTTACTGACTCCATAGGACAATCTTTTGCTTATGAGGACATCAGTTTTCATAAACAATATCAAT
GTAAAAGGAGGTAACAGTTTATGAATTTCCTATGCCAAGCCATTTATAAGTCAAAAAATGTACCTATTTAGCTTTTATGCATTCCAATTAACTTCCAAAG
AAGAAGAGTACATACTAATGGAAAACAAATATGATATTATCATCTGTTTTGCCCGGTAGTTTCTTGAGTCTCTGATTTGTCCGCACTTCACTCCCAACCA
ACACAACATTAATACACATGGGCAGAGGAATATCAGGTTATCAGGTAAACTGAATACAGTTTGATTGACTGCAGGCAGTTGATGGGACAAGATCTTTCTG
GATTGGGCGTCAAGGAACTCCAAAATCTAGAAAATCAGCTAGAAATGAGCCTACGTTGCATCCGGACAAAAAAGGACCAACTCTTGATTGATGAAATTCA
CGAACTGAATCGAAAGGTTCTGGAATATTCTGTTATTACTCTGCAATGAGAGGTCTCTGAGTGGATTTCAAGAAGCTGACTCTACTGGCTCATTTTCAGG
GAAGTCTCATGCAACAAGACAAGATGGAACTATACAAAAAGGTCAACCTAATCCGTCAGGAAAACATTGATTTATACAAGAAGGTATTTGATAAATCGTG
AATGAAAATTCACAGGATATACACCCCATCCATTATTGACTGAGGTTTATTTCTCTACTCAGCTCTACGAGAAAGAGGTGACAAGTGAAGTCAACCGAGA
TTCATCAACTCTAAACTTTGCAGTTGTCGAGAACGTCAACATTCCTGTTCATCTTGAACTTAACACTCCACCGCAAGAAAATGATGTTGAGCAAACTGCA
CCTCCTAAACTGGGGTAACCCTGCTTTTTTCTGCAAAGGAACTATTGATTCTGTTCTTAATTCTTTAGTGGCATATTGTTTACTACTAACTGAAAACTTG
CAGATTACAACTAAATCCATGAAGGCATGCAGCATGGCACCGCTTATGTTCA
>comp67351_c0_seq20
GGCAAGACGGATTACGACGTGAGAGGCGAGAGTAGCGAAGAGTCACGGAGATCAGGGGAATCAAAAGATGGAAGTGTTGGTCAAGAAAACAACCATGCAA
CATCACATATGCCTGCTTTGGTGTCGGAGTATTTAGCACCATATACACAGCTGGAACTGAACCAATCAGCTCCTGCAACATATCAGTACCCAGATCCTTA
CTATGCAGGCATGACCCATTTCCAGCTACCTGGGTTAACTCACCCTCGCATGCCATTACCTCTTGAAATATCTGAGGAGCCTGTCTATGTAAATGCAAAG
CAGTACCATGGAATTTTAAGACGAAGGCAGTCACGTGCCAAGGCTGAGCTTGAGAAAAAGGTGGTTAAAGTCAGAAAGCCTTATCTTCACGAATCTCGCC
ATCAACATGCAATGAGGAGGGCAAGAGGAAATGGGGGTCGCTTCCTAAACACAAAGAAAAGCGACAATGGTACTCCCAACGGTAAAGCTGAACCTAAGAA
AGGAGAGCAAAACTCCAAGCATATCCATGTCCCTCCTGATTTGCAACTTCGACAGAAGGAGGCATGAAGTAGCGGTCTGGCAACCTAAAACAGTGGCTTC
TGTCCACAGGCATACACACTTTTGAGTCCGGATTTGGAGCCCCAGGGTTGCGCTGCCATCTTTGCAGTTCGTCTTCTTTGTGCAAAGAGGTCTAAATTTC
GTTCACCTGAATGAGCTTGGTACCAGTTGTTGGTCCTGGCAGCATGTAATGTGGGTGCAGGAGTCCATCGAAATGTTCAGTGCCGTGGATAGGTCTGCTG
TTTTCAGGCAATTCATTCTTGGCTTTCACATTCCGCCCTTGTTTGTACCTGGCGCGTTGTAAGTGGAACTAGCTTGTGGTGTTGTGTTTTCGGCGTCCTG
TGAAACATATGTATAATTAAAATCAACTTCGGATCATCTTTGTAATCCTATGTC
>comp63680_c0_seq23
AGTATCTCGGCGTGGGACAGAGGCCGGTGGTGGTCGCGAAGAAGACGCGGCGCGGGCCGAGGTCCCGGAGCTCGCAGTACAGGGGCGTCACCTTCTACAG
GAGGACGGGCCGGTGGGAGTCGCACATCTGGGATTGTGGGAAGCAAGTCTACTTAGGTGGTTTCGACACTGCTCACGCGGCTGCGAGGGCTTATGATCGT
GCAGCGATCAAGTTCCGAGGGCTCGACGCGGACATCAACTTCACTTTGAGCGACTATGAGGATGACTTGAAGCAGATGAGCAATTGGACAAAGGAAGAGT
TCGTGCACATACTCCGCCGCCAGAGCACGGGGTTCGCGAGGGGGAGCTCCAAGTACCGCGGCGTGACGCTGCACAAGTGCGGCCGCTGGGAGGCGAGGAT
GGGTCAACTTCTTGGCAAGAAGTACATCTATCTTGGACTCTTTGACAGCGAAGTTGAAGCTGCAAGAGCATATGACAGGGCAGCCCTTCGCTTCAATGGG
GGGGAAGCTGTTACTAATTTTGGGCCTAGCTCCTACAATGGAGGAGATGCTCTACCCGACACCGAAAATGAGGGTATCACAACCAGCCTCGCCATTTATT
TCTTTTCATATAAGCAAATGTATCTCAGGTGCACCTTATAATCATCGCTGTGTTCTTAGCAACTGTTGATGGTGACGCGGTTGATTTGGATTTGCGGATT
TCACAACCTAATGTGCACGACACTAAAAGGGACAATACCTTAGCTGGAGGCCAGCTAACATGCGACTCCCTTGAATCTTCAAACACGATGGCCTCTCAGC
CAATGAGCTCATCGTACCCGTGGCCTGTGTATCACCAAAGCACGACAGCAGTACCACCTCACCATCAACGCTTGTACTCATCTGCTTGTCCTGGCTTCTT
TTCGAACCTCCAGGTATTACCAAGCTAAAGCCTATTTTTTATGTGCAAATTGCTACTCCTACGTGATCAATTAGTTCAAACTGTTATTCGAATCCAGGAT
TGTTTACTGAATATGTCCTGTCTTCAGAAAAAATAACACAAAAAGGAACATAATATGTCCTGTCTAATTGTGGACTGATACTAGCCAATTAAACAGTTTC
CCATATCCATTTCCTGTGTTTATTGTTTTGATTTGCTGGCATGGTTAAACTATAGTAGCTTGATTCGATAAAAACACTAAGCAGGCTTTGATATTTTGAA
TGAAATGTCGTTCTTGGAAATACAGCCTTTTCCTTGGTATATCAAGGACAGTTGTCCATCTGCACTCTGCACCCTCAGCTTGTGTAACTGTTTGCCTTGT
GAACTGATTGATGTGTCAATCAGGCAGTTCTGCCAGTTTGCATCTTTTGAAATAGTGCAGTCTTCCAGTCTAACAGCTCCATGACATAACTTGGGATGCA
TGCTGCAGCTTTCTTACTGAGGATGCTCTAGATTTACAGAAATTCTCGCAGTTAGCAGAGGGCTATCAAATCTGATTTGCTTAGCTTCCTGCAGACAATA
AGAACTCTAGAATCCTGAAGAAATCTTACTTGTACATGTTTACCAAAGTATCCTTACTCCTTAGTTGAGCCAATTTTGGTGAAGAACCGATAGCTCATTA
CTCTGTTTCCTGGGAATGATGTGGGCCTAAACCATTCTGTGGTCAAAGTTTAACAAAGCCCATGCTTGTGCTGTTGCAACTGCAGGAAAGGCCAATGGAG

CGAAGGCCTGAGTTGGGTACCCAGTCATTCCCCACCTGGGGATGGCAAATGCAGGGCTCCCCTCACATGCCGTTGCATCACTCTGCAGCATCATCAGGAT
TCTCTACCGCCGTCGGCGCAAACGCCGGCGTCTCGTTGCCTTCTCACCCGCCGGTGCCGTTCCCGGACCACCAGTTCTACTTCCCACCGACGGCATGAGC
TTGTTGGTCTGGTTCAACTCTTGGGACGGCAGCTGATCTTATGTGTGAACATTTCGCAGCTTGCCGGTGACCGTTGTTAATTAATCGGGGAGAGAGAGCC
AGAAGACCCAGTATGGCTATCTCTCTGTCGCCCGTGCCTTGTTATGATCTTAGTTATTGCTACTACACCGTGGATTCATCATGGTTTATGAGATTCTTTA
CTCAAGTTTCAGCTTAAATGTCCAAATACATGAATGCATCATACACAGGATCCGTCATTTTCTAAAAAAATACAGGATCTGTGTGAAGTGTAAACATAAG
G
>comp82796_c1_seq14
CGATTCATTGGTCTTTTATATAGGTTGAGGAACCGGGAGGCGTAGGATAGAGAGATCTCAAGGTCAGGTGAGAGCTCAGTGTAGGTCAATGGCGTCCGTG
CCGTCGCTGGTGCTTCAGTTGCCAGTCAATTGATGCACCGTGGTTACTGGTTCAGCTCGCCGAGCAGGTGGATTTCAAGGTGTGTACGGTGTTGAGGATT
GGGAACGAGATTGTCAGGAAGAGGCAGAGGAATCCGTCGTGTTCTTTCCCCCCAAAAGAATCCGCAGTGTCCTCTTTCTCTTTTTTTTTGTTGGCATGAA
AGGGATTTTAGGTCCTTCATTTTCCCCTTGTTCATTGTTGCAACGAAGACTTTTATTCTGTTCTTGCTCCGAATTCGTCAGTAAATTTTTAACTTTTAAA
TCCGAAAGGAGAAGATGTGATCTATCTATTCTGTTCCAATTTGGCATTTGTATGTGACTTCTGTTCTTTCTTCTTGTTCTTGCAGTGCTAGCAGTTTTTA
CCTGTTCGGGCAGGAGCAATAGTGGAGATCTTTAGTTCTGGCATGGGTTCCTTTGGGATGAACTGGAATCAAAAGAACTCCATGGTGTGGGATTGGGAAA
ATTTAGCACCGTCTGTTCCAAATGCAATCGGAAACCCCAGGAACGGAGCTACGGGGCATGGATCTGTGAATTCATCTGGTGGCACTCTCACTTCTAGCTC
AGAGCTAGGGCATGGTTCATCCAAGAGCTCCATGTCGGCATCCATTGATTCACCGTCCAAAGTAGGGAACAGCTTGGAGTTCAATTTTGCTGCTGTCAAT
ACGTATGGTAAGAACATGGATAAGGATTGTAGAGTTGACGACTCAGGGACTTGTCCATCGTCCATGATAGCATTCAGCCATGGAGAGCCATTAATCAGTC
TTAAGCTTGGAAAAAGGACTTACTTTGAAAATGTCTGCGGAGGACAGGATTCCAAGAGCACTGCACCTTCTACCAGGACTTCTCCATCAACCGTTCTCAA
GAAGACCAAGGTGTCTCAGCAGAATACACAAATGTCATACTGTCAGGTTGAAGGTTGCGGAGTTGATCTATCTTCTGCTAAAGATTACCATCGCAAGCAC
AAAGTCTGCGAAGCTCATTCTAAAGCTCCGAAGGTGGTTGTTGCTGGTGTACAGCGCCGTTTTTGCCAGCAGTGTAGCCGGTTTCATGGCTTAGCCGAGT
TTGACCAGAACAAACGAAGCTGCCGTAGACGTCTTACTCATCATAACGCACGAAGGAGGAAACCACAGGCGGATACAATTTCATTCAGTTCATCACGTCT
CTCAACAATGTTTTATGATACAAGCCAGCGGACAAATCTTTTCTGTAGTCAGCCTCTTTTTGGCCAAGTGAGAAGCAATGCAGTTTCTTCATGGGATAAC
TTGGGAAGCTTCAATTTTATGGAAACAAAACATCTGTTGATGCAGCCAACGAAAATGTTAGGTCTTGATGGGCTGCATTTCTCAACCCCCCAGATATCAA
ATAGTGTTGTGGCTCACTCTGTACATCATCATAATTTTGATGGGTTCACGCCATTCAAGGGAACCAACACAAAGGTCCTCAACCAAGGTGTCGAAGCTTC
TGCGATCGCTTCCAACTCGAGTGGAGACCCGGATCTTGGGTGTGCTCTCTCTCTTCTGTCAAACGGTTCGTGGGGTTCAACTTCAGCCGTCATCCAACAG
CCTAGTTCTCATGCGCAAGCTGGTGCGGTGCCACCCTTCGCCACCGTCGCCGTCTCCAACCCTGCAATGCATCCTCTGGACTCATCCCCAGGAGGATTCT
GGCAAGACGACCCTTCCCCGCTCGATGGAACTCCGGAGATTCAGGCATTCGGACACCTCTAACTATGGTCAAACAAATGTACTCATGCTGCATGCCCTCG
GAACAGCTGATTTAGTCACCTCATCATAATCTGCAACTCTGCTTTGCCAGTGGCAGATCTCTGCATAATGTCAGTCCTATCATCGGAGGCATTTGGTGAG
GTGAAGACTGAAGAATAATATAGGAGGCAACCCCCTGATCTTCCTGATTTAGGATGCATCTAGGATCCAGTTGTGTGTGTGCTTGTGGTTTTGCCAGAAC
CGGTTTTTTGTTTGCCGTCCCGTTAGAGAGAAGGGATCAAGTACATTCATGACTGCTCATGAGATTACTTGTTTCACTAATATTACATGATCTGGAGCGA
GATTTCGCTCGGTTCACGTTGATGGT
>comp82796_c1_seq6
GGTAGTATGGAGCCCTTTGTTCATGAGTATATATTATTCTCAAAAACACTATCTAAACTGATCACTCATACAGTACCTCTTTTGAGAGGCATTATTTCTG
AACCCTACATGTTGAAAGAGCACTTTCTGTAGGTGTCAAATGGTTAATCTTCTATTATAGCTTGTTCTCTCCATATTGTCAAATTAATGACTGAAGGCTA
ATCCTTTATCCAAATTTATACTTCTGAATATGATGCTGCTGCTGCTGCTTTAAGCATTGTAAATATGCATTGGCTTAGATTGTCGCAGATGCATATTCAC
AGAGTTAGTCGATTATTTGGAAGGGGACTTAACCCAACTTCATGACACTTTTAGTATGCACATACGCTTCTCTTTTGGCGGGACTACTCTTAGAATATTC
TTCTTAAATTATTATAAATGCAATTTTGCTTCTGGGAGAACACTTAAGTTTTAATGCTGGTATTGTTCGCATTTGCAGATACAAACCAGCAGAAAAATCT
TTTCTGTAGTCAACCTCATTTTGGCCAAGCGAGAAGCAATGCAGTTTTTTCTTGGGAAAACTCGGGAGATTCCAAATTTATGGAAACCAAACATCTGTTG
ATGCAGCCAACGAAAATGTTAGGTCTTGATGGGCTGCATTTCTCAACCCCCCAGATATCAAATAGTGTTGTGGCTCACTCTGTACATCATCATAATTTTG
ATGGGTTCACGCCATTCAAGGGAACCAACACAAAGGTCCTCAACCAAGGTGTCGAAGCTTCTGCGATCGCTTCCAACTCGAGTGGAGACCCGGATCTTGG
GTGTGCTCTCTCTCTTCTGTCAAACGGTTCGTGGTGTTCAGGTTCAACTGTCATCCAGCAGCCTAGTTCTCGTGTACACGCTGGTGCGGTGCCGCCCCTC
GCTACTTTTGTGGTCTCTAATCCTGCAATGCATCCTCTGGACCCAGCCCCAGGAGGGTTCTGGCAAGACGACCCTGCCTCGCTCGATAGAACTCCGGAGA
TTCAGGCATTCGGACACCTCTAACTATGGTCAAACAAATGTACTCATGCTGCATGCCCTCGGAACAGCTGATTTAGTCACCTCATCATAATCTGCAACTC
TGCTTTGCCAGTGGCAGATCTCTGCATAATGTCAGTCCTATCATCGGAGGCATTTGGTGAGGTGAAGACTGAAGAATAATATAGGAGGCAACCCCCTGAT
CTTCCTGATTTAGGATGCATCTAGGATCCAGTTGTGTGTGTGCTTGTGGTTTTGCCAGAACCGGTTTTTTGTTTGCCGTCCCGTTAGAGAGAAGGGATCA
AGTACATTCATGACTGCTCATGAGATTACTTGTTTCACTAATATTACATGATCTGGAGCGAGATTTCGCTCGGTTCACGTTGATGGT
>Locus_2827_Transcript_70/166_Confidence_0.091_Length_1163
GCTGTTGATTGGGTCCAGATGCCTGGGATGAAGCCTGGTCCGGATTCGGTTGGTATTGTGTCTGTTTCACATGGTTGCCGAGGTGTTGCTGCCCGTGCCT
GTGGTTTGGTTGATCTAGAACCAAGAAAGCATCTGATTCCAAGACTTAAAGAGCCTTTCGATAAAACTTGGCGCATACTGTTGTATTCAGCAAATTCTCT
GGAGGTTTCTAATGTTAGTGAGCTTTATATATATTGTGGAGATATTGAAAGATCGACCATCTTGGTTCTGCGATTGTCGGAGTCTTGTAGTCTTTACAAT
GTTACCGGCTGGAAATGGTGGAACTGTTAAGCTTGTTTGCATGCAGATGTATGCTGTTACAACTTTAGTTCCTGCACGAGATTTTTGGACGCTGAGATAC
ACAACCACTATACAGGATGGCAGTCTTGTGGTCTGTGAGAGATCTTTAAGTGGTTCAGGAGGTGGTCAAAGTACTGCATCGGCACAACAATTTGTTAGAG
CTGAGATGCTTCCTAGTGGCTATTTAGTCCGTCCATGTGAGGGCGGGGGCTCAATTGTGCATATAGTGGACCATCTGGACCTTGAGGCTTGGAGGGTCCC
TGAAGTTCTTCGTCCGCTTTACGAGTCGTCTAGGGTAGTTGCTCAGAAGATGACAACTGCAGCACTACGGCATATCAGACAAATTGCTCAAGAAACAAGT
GGAGAGGTCGTATATGCCTTGGGGAGACAGCCAGCTGTTCTACGAACATTTAGTCAGAGGCTTAGTAGAGGCTTTAATGATGCTATAAGTGGTTTCAATG
ATGATGGTTGGTCTGTCATGGGTGGAGATGGCGTCGAAGATGTTATCATTGCTTGCAACTCAAAGAAGATAAGGAACAATACCAATCCTGCAAGTGCTTT
TGGAGCCCCTGGTGGTATCATATGTGCTAAGGCATCCATGTTACTGCAGAGTGTTCCACCAGCAGTACTAGTTAGATTCTTGAGGGAACATCGGTCTGAA
TGGGCAGATTATAACTTCGACGCATATTCGGCCTCGGCGTTGAAAACAAAACCTTGTTCCCTTCCTGGGTTGCGGCCTATGAGATTTTCTGGGAGCCAGA
TCATCATGCCACTTGCTCACACAGTGGAAAATGAGGAGATCTTAGAAGTTGTTCGTCTTGAAG
>comp70939_c0_seq5
CCGGGCGCTCGCCCCGGATTTCTCGGCTCCCCATCCGGCGGACGCCTTGACCGGGGTGGGTTCTTGGTTCCGGCGGCTCGGTGATCTGTGCGTGCGAAAG
GCGGGGCTTTTAGCTCGTTCTGTGGGGGGAGGAGGAATACGGCGGCGAGGCGTTCATGGGTTAGATCTGGCGGTCCGGCGCCGCGAATCTGAGCGAGGTT
TCGGTGGAGGGATCGGGGCGGAGAAGTCGCAGTCTCTGGCTCGTTCTGAGCAGCTTGGGCCTCTCATGTCCAAATCGGGTATGGATGATGGGTGCGGTAA
CTGTCGGAAGGAGCGTCGCTCGCAGATCTGAAGGCCGTCCAGCTCCCTTGTTCAGTTGACCTGCTCGCTGGTCAGCTGGATGGAGTGAATGGCAATGCTT
TCTGATGTTTGACTGCCGTTAGTTGCTGCTTAGTGTCTGTACAAGAAATTTAGCAGGAGGTGGTGTGCTTATGAGACTCTTCTAGGTGCTGATATCTTGG
TCTCTGTTGAAGAACGCTGAAGCTGGAAGGAAGTAGGTCGCATTCCTGTGTTGTTGATATTTTGTTGTTTCTCGAATAACCTGGAGGCTGGAAGGGATCA
GATCCAGTTCCTGTGGCACCGGTAACAAGGTGCTGTTGGGGGAAGGCACCATGACCTACTTCCCTGAGGAGGTGGTGGAGCATATATTCAGCTTCTTGCC
GTCGCACAGTGACCGGAACACGGTCTCGTTAGTGTGCAAGGTGTGGTATGAGGTTGAGAGGCTGAGCCGGTGTGCTGTCTTTGTGGGGAACTGCTACGCG
GTGCGCCCAGAACGGGTGGTGCTGCGGTTCCCCAATGTGAAGGCCCTGACGCTGAAGGGGAAGCCGCACTTCGCGGACTTCAACCTCGTGCCGCCGGACT
GGGGTGGCTATGCGGGGCCATGGATTGAGGCTGCGGCGAGGCGATGCGTGGGTCTTGAGGAGCTCCGGATGAAGCGGATGGTGGTGTCAGATGAGAGCCT
TGAATTGCTAGCTCGGTCCTTCCCGAGATTCAAGGTCCTCGTCCTTATCAGCTGCGAGGGGTTCAGCACTGATGGGCTAGCAGCTATTGCGAATCACTGC
AAGCTCCTGAGGGAGTTAGATTTGCAGGAAAATGATGTGGAGGACCGTGGGCCTAGATGGCTCTCCTGCTTCCCTGATTCTTGCACATCACTTGTTTCCT
TGAATTTTGCCTGCATCAAAGGGGAGGTGAATTCTGGTTCATTGGAGAGGCTTGTTGCTAGGTCTCCAAACTTACGCAGCTTGAGGTTAAATCGATCTGT
ATCAGTAGATACACTCTCAAAGATATTAGCATGCACCCCTAATTTGGAAGACTTGGGGACAGGGAATTTGACGGATGAGTTCCAAACTGAATCCTACTTC
AGGCTGGCTAATGCTTTGGAGAAATGCAAAATGTTGAAGAGTTTATCAGGATTTTGGGATGCTTCTCCTGTTTGTGTTCCGTTTATCTATCCCCTATGTC
ATCAACTAACAGGACTGAACTTGAGCTATACTCCCACACTGGATGCCTCTGATCTCACTAAAATGATCAGCCGCTGTGTGAAACTCCAGCGTCTTTGGGT
ACTGGATTGCATCTCGGATAAGGGTTTGCAAGTGGTGGCTTCCAGCTGCAAAGATCTACAAGAACTGAGGGTGTTCCCGTCAGATTTTTATGTTGCTGGT
TATTCTGCAGTGACAGAAGAGGGACTTGTTGCAATATCGTTAGGCTGTCCAAAACTTAGCTCCCTGCTGTATTTCTGTCACCAGATGACCAATGATGCAC
TAGTTACTATAGCTAAGAACTGCCCAAATTTCATACGATTTAGGCTCTGTATTCTTGAGCCAAAGAAGCCAGACGCCATGACAAACCAGCCGTTAGATGA

AGGCTTTGGTGCAATTGTTCGTGAATGCAAGGGGCTAAGGCGACTGTCAATGTCGGGTCTTCTCACTGACAAGGTTTTCATGTATATTGGAAAGTATGCC
AAGCAACTTGAGATGCTTTCTATAGCATTTGCTGGAGATAGTGATAAGGGGATGATGCATGTTATGAATGGATGCAAGAATCTAAGGAAGCTGGAGATTA
GAGACAGTCCATTTGGTGATGTTGCACTGTTGGGGAATGTTGCCAAATATGAAACAATGCGATCCCTTTGGATGTCCTCATGCAATGTCACATTAAAGGG
GTGCCAAGTCCTTGCGTCTAAGATGCCGATGCTCAATGTGGAGATCATGAATGAGCTAGATGGAAGCAGTGAAATGGAGGAAAACCATGGAGATCAATCT
AAGGTGGAGAAGCTATATGTTTACCGCACAACTGCTGGAGCGAGGGATGATGCACCAAATTTTGTTAAAATCCTATAGAGCTAGCTGAGAGTAGTACGGT
ATCTTCTCACTCTAAAAGATGGTCGGTGGAATCCGGCAGGCTGGGGGCCGCGTCAACAGTGGTAAGTTCTTGAGGTGTCTGTCTCTCCCTGTGCAGGGGT
CAGGAAAAGCCACACCGATGTGCACCCTTATCTTGGAGCAACTGGCTGGGATCCATCATCATCCCCTGTACCACCGCCCCCTACCCTTTTGTGTTTGGAA
TTCCTTTGTGTTTGCTCACTCATATCCACTGAGGCCCTTTATCATCCAGAGCATTCTCACTTGAGAGGACCGCCTTTTTTCCGTTTATCCCACTGTAATC
TGTAGTGTACCAGTATCCATTTGCATTTGACATTGGCAGTGGACTGTTTTGCCGCTGTTGTTGTAGTGTTTGTTTCAGCCTGACGATTTGTTTCTTGCTC
CTGTGAATGCAGTGCGTGGAATAATTTGCGGTTGTTATCTGCGACATTCTATTCTCTGGTTTTGAATTCTTGGTGTTAGCCGTACCAGTTTTACGTGCTT
GTTACTGAGCAAGAAGGCACCAGTACTCGTGCATTTTAAGGGCGTGCTCAGTTTCTGATGCCATTTTTCTCGCTTACATCATCTTTCTGTATTGCTGCCC
TCAAATATGAATTCAAATTAACGGT
>Locus_18892_Transcript_43/56_Confidence_0.309_Length_1319
ATCTGGGATTGTGGGAAGCAAGTCTACTTAGGTGAGTAACTGAGTTGAGTGAGTTCTATACGTTCCAGCTCGAGCTTCCATTAACTGCTGATCAGTTTCC
GTCCGTTTGATCTGGTTTACGATGTGCTGATGCTGTTCTATTCAGGTGGTTTCGACACTGCTCACGCGGCTGCGAGGGCTTATGATCGTGCAGCGATCAA
GTTCCGAGGGCTCGACGCGGACATCAACTTCACTTTGAGCGACTATGAGGATGACTTGAAGCAGATGAGCAATTGGACAAAGGAAGAGTTCGTGCACATA
CTCCGCCGCCAGAGCACGGGGTTCGCGAGGGGGAGCTCCAAGTACCGCGGCGTGACGCTGCACAAGTGCGGCCGCTGGGAGGCGAGGATGGGTCAACTTC
TTGGCAAGAAGTACATCTATCTTGGACTCTTTGACAGCGAAGTTGAAGCTGCAAGAGCATATGACAGGGCAGCCCTTCGCTTCAATGGGGGGGAAGCTGT
TACTAATTTTGGGCCTAGCTCCTACAATGGAGGAGATGCTCTACCCGACACCGAAAATGAGGCAACTGTTGATGGTGACGCGGTTGATTTGGATTTGCGG
ATTTCACAACCTAATGTACACGACGCTAAAAGGGACAATACCGTAGCTGGCTTCCAGCTAACATGCGACTCCCCTGAATCTTCAAATATGGTGGCCTCTC
ATCCAATGAGTTCATCGTCCCCGTGGCCTGTGTATCAACAGAGCACGGCAGTACCACCTCACCATCAGCGTTTGTACTCATCTGCTTGTCCTGGCTTCTT
TCCGAACCTCCAGGAAAGGTCAATGGAGCGGAGGCCTGAATTGGGTCCCCAGCCCTACCCCACCTGGGGATGGCAAATGCAGGGCTCCCTTCACATGCCG
TTGCACCACTCTGCAGCATCATCAGGATTCTCTACCGCCGTCGGCACGAACGCCGGCAACGGGCCGTTGCCGTCTCAACAGCCGGCGCCGTTCTCGGACC
ACCAGTTCTACTTCCCACCAACGGCCTGAGCTTGTTGGTCAGTTCAACTCTTGGGACGGCAGCTGACGTTGTGTGTGAACGTTTCGCAGCTCTCCGGTAA
CAGTTGTTAATTATTGGGAGAGAGCCAGAAGACATAGTATGGCTGATCTCTCTGGCCTGTGCCTCGTTATGATCTGAGTTATTGCTACTACTACACCGTG
GATTCATCATGGTTTATGAGATTCTTTACTCAAGTTTCAGCTTAAATGTCCAAATACATGAATGCATCATACACAGGATCCGTCATTTTCTAAAAAAATA
CAGGATCTGTGTGAAGTGT
>Locus_26257_Transcript_1/4_Confidence_0.250_Length_319
GGGAAGAAGCCGCCGGCGCTGCGGTTAGAGGCCTTCTGGTGGTGGTAGTACGGGTAGGGGTACTCGTACGGGGTGGTGTAGTAGTAGTCGTAGGGGTCGT
ACGCGAAGAAGCGGCGAGACCCCTTCACGTCACAACACAAACTCCAATTCCACGCCAGATCCAACCTCCAAGAAAGCTTGAGGCAGCAACAACAATGGCG
TCTCGCCGCTTCTTCGCGTACGACCCCTACGACTACTACTACACCACCCCGTACGAGTACCCCTACCCGTACTACCACCACCAGAAGGCCTCTAACCGCA
GCGCCGGCGGCTTCTTCCC
>Locus_3441_Transcript_296/417_Confidence_0.013_Length_360
AGCTTGAGGATGGGGCGGGGCAAGATAGTGATCCGGCGGATCGACAACTCCACGAGCCGGCAGGGGACGTTCTCGAAGCGGCGGAACGGGATCTTCAAGA
AGGCCAAGGAGCTCGCCATACAAAAAAAAAAAAAAAAAAAAGCCTCATGATCTTCTCCAGCACCGGCCGCCTCTACGAGTACTCGAGCACCAGCATGAAGT
CGGTGATAGATCGATATGGCAAGGCCAAAGAAGAGCATCAGGTCGTCTCAAATCCCAACTCGGAACTTAAGTTTTGGCAAAGGGAGGCAGCAAGCTTGAG
ACAACAACTGCACAACTTGCAAGAAAATCATCGGCAGTTGATGGGTGAAGATCTATCTGG
>comp63492_c0_seq9
GTGGTCAAATCCCTGCCTGTCTGTTAAACGGGAAAGGATTTGGTGGATTGATTATTTACACCTGGGCAATGTACTAATATGTAAGCCTTGCAAGCTTAAC
AATTAGTAGACGGTGTTCTGAAGTAACATTGACCTTAGACTACAACAAAGATTATGTTCGCACATATATACATGAGCGCTCTATATACATGACACAAGAC
GAAGCAATTTTCCTCAACAGTATTAGCAAATTTTCTGTCCACACCGTGCCTAGTAACTCCATACAAGTTAAAGCGTCCTCAAAACAACAAGCTTGATTAT
GGAAAGCTTCTGAATTACCAGGTTAGCGAAATTCAAAACTCCAGAACCTTTCTGCCTGACCATGTGCCATGTAAAGGGGAACACCGTCCAAGCAATCAGG
AAGGAAATACAGTAGTTGGAGCACCCATCTTAAAAGTTGGAGCACCCATCTTAAATTCTCCATGGCCAAAGCCATAACACAGCACTTGAAAACCATTATC
GTTGTAATGAAGCCAGGCCAAAGCCATGCCAAGTCATCCAAAGGTCATCACGGCAGTTTTTCTGCAAGATCAGACTGCACCCTTTTACTCTGAAATAGAT
ACCATATACCACAAATCAAAGGTTGTCATGATGAATGTTACTCAAATCTATTGACAACCTTGGCTTCCGTACTTTAGAGCTCGACATAGTAATTCACACC
TGCTATGGCAACTGGCAAGTTTAAAGGAGATGGCAAAGTGGCCCTTCAAATCATACAGTGATGGATTTGTAACGAATTCAACTGACACTGTTCACTTGCG
CAAGAAAAAAGGGCAGTAAAATATTAAAAGCTGGAAAGAGCCAGAACCAAAAAAATGTAACTGATATTAGCAAGCTAGAAAGAGCCAGAACCATAAAAAT
GTAACTGATATTAGTAAGTATGATACTGATGTTATGTCTAACTGGAGTATGTATGCTATGGATAGACTAGCTCCTTAAAATGTCCTTCCACGTGGATACT
GTTTACCGAAGATTTTTCTTTTTGTAAATATGACGCAAGGCCAAATCATTACCTATAAGGCGTCATGCCGGAGGAACATCATTTTATTGGGCAATATTAT
GCTTTGAAGAAATGAAGATGATGAAACAGGATCCCAATGTAAAGGACTATATCGCAACAGGATCAAAATGTAATATCACACATGCACATTGATAATGCAA
CTGAACAGGCATTATCTGAAAACAGGAAAACCAAACTGAATTTTCCTTGAAATCACATTGGGGACTGTAAATGTTAGAATATGACACTACAGATATTGTG
CCAACTTATTGATTGACCACAACCCCCTGAAGTTAGCTAAGATTGTATGGACATGTATATGTAGTTTCTGTTTTTAAAATAGTTAACGTTATCCCCAAAG
CTGAAGATGTTGCAGTTATTGGGCATAGATTAACTTTAAGCAATGTATTAGTATACTACGCCTTACATTTCTACATTCTTTTTCTAAAATTACTTTTTGT
AGCTCAAAAACTGACAATGTTTCTTGTTTTACTGACAGCTCTGGCAAAGGGAGGCAGCAAGCTTGAGGCAACAACTGCACAACTTGCAAGAAAGCCACAA
GTATGATCTTCCAATTGATTCTGCAATAGAGAGAGATCACTCATCAATACACAGTTTATATGACACATGGAAAGATTTACTAGCATGAGAATTGTCAAAA
ACCAAAATGAAATGTTTCAATCAGCCAGGATGAGGGGAAAATCTATTTACTGAAAGACATTTAAACTATTCAGTATTCAGGAAAGGAAATTGATAGTGGC
TCTCTCCCAACAACATATAAGTGGTTTTGCTTTGACCTGTGTACCCACGAGATTTATATTCGGTTAGTTTCAATGTTGTACACAAGATCTAACGTGATAA
ACTCTTTTGCATTGTTGTAGGCAACTAATGGGTGAGAAGCTTTCTGGCCTAGGTGTGAGAGACCTCCAGAGTTTAGAGAATCGTCTTGAAATGAGCCTAC
GTAGTATCAGAACGAGGAAGGTCATATTCATTTTGTTTCCACGTTTTGTTTTTCTATTCACTCATCTTAATATGCATAAACATCCATCAAACAGGACAAT
CTTTTGAAAAGTGAAATTGAAGAGTTGCACAGGAAGGTAAAGCCTTACAATCCATGTCTTTTTATTATCTCAAACAAATGACCATGCATGGAGGTTGCTG
ACATCTTAACTTAATGCTGATTGAAACAGGGTAGCCTAATTCACCAGGAAAACTTGGAACTTTGTGACTTCCTTGCAAGTAATGCGGTTCCTGCCTCATG
CTTGGTGCCGCCGCATGCAATAAACCGATAACTAGTGAAGCAAGAGGATAACAGATGTCAGAAAACTGAGCAAGCCATGTTAACTGTTTGAGAATTGTAT
ACGGCTATGAGAAGTATATTTTTTTAGGGGGTGTTTCGATCCCTTTAGCTATTTTTTTAGCTCTATATTTGGATGTTAAATAGGAGGACTGAACATGGGC
TAATTATAGACTAATTGCAATTAGTGATTAGAGCCCATAAGCTCTAATTAGCCTATGATTAGCCTATGATTAGCCCATGTTTAGCCCATTAGCACATGAG
CTAATCATAAGCTAATCAG
>Locus_3441_Transcript_399/417_Confidence_0.047_Length_1428
CTGTGCTTTCATATTCAATAGTTCTGGCAAAGGGAGGCTGCAAGCTTGAGACAACAACTGCACAACTTGCAAGAAAGTCATCGGTATGGCTCGAAACCCT
GCAAATTTTACAAATGTACAAGTCAGAAAATTTGCCTAATGTAAAAGGATCAAACTACAGGATTTGTCAGCATGAGCACAAGTGCCTAACTTCCGAGTTA
GATAACTCTGGAGGAAGGAAATCCTTCCTACTGTTTGTTTCAAGAATATACAAAAATTCAAAACAACATTAGAATAGAACCAAAAGAAGAAACAATATTC
ACCATGTAGCAATACTCAATTCCTGGTTGTCTTATTTATAAAGGTATAAAACTAGGTTCTGACGAGACAATTATGAATCAACTACACCATGTTACAGATC
ATCCATAACTCTTGGTAATGCAAATTTTAATGGCTGTGAGGTGCAAGTATACCTCCACCAACTAATCCGTGTAGTGCCCAAGTTTAGTATACACAAGATG
CATAAGCATACAATGCCCAAGTTTAAGAAGGTGATATATACAGACTCCATAGGATAATCCTTTGCTTAAGAGGACACCAGTTTTCATAAAATTTACCTTT
AGTCATATAGCTTTTATGCACTCTTATTAACTTTCAAAGAAGCAGAGTAAATACTAATGGAAAACAAATGTGATATTTTCATCTGTTTTCCCTAGTCTCT
GATTTATCTGCACTTTACTCCCAACCAACAGAACATTAATATACACATTGGCAGAGGTCGAAACTATAAAAATCACGGTAACAATGTCGAGTTATAAATA
TCTGGTAAATTGGATAACTTTGATTGACTGCAGGCAGTTGATGGGACAAGATCTTTCAGGATTGGGCGTCAAGGAACTCCAAAATCTAGAAAATCAGCTA
GAAATGAGCCTACGTTGCATCCGGACAAAAAAGGACCAACTCTTGATTGATGAAATTCACGAACTGAATCGAAAGGGAAGTCTCGTCCAACAAGACAACA
TGGAACTATACAAAAAGGTCAACCTAATCCGTCAGGAAAACATTGATTTATACAAGAAGGTATTTGATAAATCGTGAATGAAAATTCACAGGATATACAC
CCCATCCATTATTGACTGAGGTTTATTTCTCTACTCAGCTCTACGAGAAAGAGGTGACAAGTGAAGTCAACCGAGATTCATCAACTCTAAACTTTGCAGT

TGTCGAGAACGTCAACATTCCTGTTCATCTTGAACTTAACACTCCACCGCAAGAAAATGATGTTGAGCAAACTGCACCTCCTAAACTGGGGTAACCCTGC
TTTTTTCTGCAAAGGAACTATTGATTCTGTTCTTAATTCTTTAGTGGCATATTGTTTACTACTAACTGAAAACTTGCAGATTACAACTAAATCCATGAAG
GCATGCAGCATGGCACCGCTTATGTTCA
>Locus_1016_Transcript_988/2070_Confidence_0.004_Length_4892
GTGGAGGCGGCAAGCTAGAGATAACAACTGCATAACTTGCAAGAAAATCGTAAGTTTCTCAATTTTTCTGTTCTATACATGAGAAAACGGTGTTGATTTG
TGTGCATGCGTGCGTTTGTCAATGATCTGAATGATTTGCACATGATTTGTGTTAACATGCTGTGAATTCAGGGTATGGCAAATTTGCCAAATATCTTTTC
AGGATGCTTAAATTCACTTTGTTTAGAACTGTTTTGAGAACCGTATGGATTCAATAGACACCCAATTACTTGGTTATAATTGAGTTATTTACTTTACGAA
AGGTCGATGTGGTCATAGCATATTAAAGTTCCTTTTAATTGACCAATGACCGTATGGTTACTCTCATTCCTTCAATTCATTGCTCTCCCAGTCACAAATA
AGTGATGTTTGGGACATTGACAAGGCCTCCAAAACTCAAATGACCACCTAATTCTGCCATAGTATATTTTTAGAACCTAATAATTTGTATATCATTTCGA
TGTGTATACATCATTTAAGTTTAGAACCCAATAATTTGTATATCATTTTGATGTGTATATATCATTTAAGTTTAGAACCTAATAATTTGTATATCATTTT
TAGAACCCAAACCTCAAAGACATATCGAGAGGAATCCATGTATATCATTTTGATGTGTTCAATGTAAATATTTGTAAAGATACCAGTAGCGAAAGTTTGA
CTACACACATTCCAAATGTATAGAGGTATAATTGGAGGTTCAGCCGTTCAGAGACCCAGGTCTTGGGGTCCAGGATCGTTTTGTGCTATAATCGTTTACC
CCTTGAATTTCTGTGATTAAGGTGATAATCTTAATTTACTGTGACATAAGAATAGGCCAAATATTTTCCTGTACAAACTTAAGCTAGTGTATCTGTCGTG
TGTCTTGTCATAGTACAATCCGTTATTTCTGGTTATTTTCGGTGCGTCAACGTCTTACTGTTGTTATTCCTTTGAACACTACAAAATTTTAAGTGAGCAA
CTAGCATCCGTATATTTACATATGTAGCTGAAGAAAGGTTGATCCCCATTCTGATACTCTGGAATGCCTGCACGGATAAACCAGAATGCAATTTGTGCTC
TTCTGCTATGCATACAATTCTCAAAATAGAAAAAAATAGCACCTGAATTTTTTCCACACACCATATCTTTTTGTCAATATAAGATTTGTAGCTATCATGT
ACTCACGTTGGTCAACAAACTTGCAGAATGTGTCAAATAGTTATAATACAAATCATTTGCTGTATTCTTCAATAAAATACATTTTATTTGAAATAGATTT
ACACTACCATTATCAATCACTCATGGCATGCATAAATAGATTTACACTACCATTATCAATCACTCATGGCATGCATACCGCGTCAGTCTTGCTATGTGTT
TAAGCTCTTTACAACCAATAGTGTTTCTGGTTGCTTTGAATCGTACCGTTGAGAATAATGAACATAAGCGGTGCCATGCTGCATGCCTTCATGGATTTAG
TTGTAATCTGCAAGTTTTCAGTTAGTAGTAAACAATATGCCACTAAAGAATTAAGAACAGAATCAATAGTTCCTTTGCAGAAAAAAGCAGGGTTACCCCA
GTTTAGGAGGTGCAGTTTGCTCAACATCATTTTCTTGCGGTGGAGTGTTAAGTTCAAGATGAACAGGAATGTTGACGTTCTCGACAACTGCAAAGTTTAG
AGTTGATGAATCTCGGTTGACTTCACTTGTCACCTCTTTCTCGTAGAGCTGAGTAGAGAAATAAACCTCAGTCAATAATGGATGGGGTGTATATCCTGTG
AATTTTCATTCACGATTTATCAAATACCTTCTTGTATAAATCAATGTTTTCCTGACGGATTAGGTTGACCTTTTTGTATAGTTCCATCTTGTCTTGTTGC
ATGAGACTTCCCTGAAAATGAGCCAGTAGAGTCAGCTTCTTGAAATCCACTCAGAGACCTCTCATTGCAGAGTAATAACAGAATATTCCAGAACCTTTCG
ATTCAGTTCGTGAATTTCATCAATCAAGAGTTGGTCCTGAAAGTAAAAAACGTCATCCATCATCAGTTGTATTCTTGGATGAGGCAATCTGGTAACGAAA
AACAGAAATATGGCTAGCATCCTTACCTTTTTTGTCCGGATGCAACGTAGGCTCATTTCTAGCTGATTTTCTAGATTTTGGAGTTCCTTGACGCTCAATC
CAGAAAGATCTTGTCCCATCAACTGACTGCAGTCAATCAAACTGTATTCAGTTTACCTGATAACCTGATATTCCTCTGCCCATGTGTATTAATGTTGTGT
TGGTTGGGAGTGAAGTGCGGACAAATCAGAGACTCAAGAAACTACCGGGCAAAACAGATGATAATATCATATTTGTTTTCCATTAGTATGTACTCTTCTT
CTTTGGAAGTTAATTGGAATGCATAAAAGCTAAATAGGTACATTTTTTGACTTATAAATGGCTTGGCATAGGAAATTCATAAACTGTTACCTCCTTTTAC
ATTGATATTGTTTATGAAAACTGATGTCCTCATAAGCAAAAGATTGTCCTATGGAGTCAGTAAATCAGTATGTCTCCTTAAACTTGGGCATTGTATGCTA
TCTTGTATACTAAATTCATTCTTATCCATTGTTTTACAATTTTATACACTCTGCACAAATTAGTTGGTGGAGGTATACTTGTCACTTTACAGCCCTTAAT
TTGCATCACCAAGAGTTATGGATGATCTGTAACATGGTGTAGTTGATTCATAATTGTCTCGTCAGAACCTAGTTTTATACCTTTATAAATAAGACAACCA
GGAATTGAGTATTGCTACATGGTGAATATTGTTTCTTCTTTTGGTTCTATTCTAATGTTGTTTTGAATTTTTGTATATTCTTGAAACAAACAGTAGGAAG
GATTTCCTTCCTCCAGAGTTATCTAACTCGGAAGTTAGGCACTTGTGCTCATGCTGACAAATCCTATAGTTTGATCCTTTTACATTAGGCAAATTTTCTG
ACTTGTACATTTGTAAAATTTGCAGGGTTTTCTAGCCATACCGATGACTTTCTTGCAGGTTGTGCAGTTGTTGTCTCAAGCTTGCAGCCTCCCTTTGCCA
GAACTATTGAATATGAAAGCACAGTTAAGTAATGCCACATCCAGGATTGCCAAAATACAGCATTGGTATAAACATGTTGCATAGAAGCATGTCTGAGCTG
TCAGCTAAACAAATTGTACCTTATGAAATAACTGAATTAAATAAGATGACATGCCACTATTTCAATCAAGAGATTTACAGTGCGCTACAAAGTAACTCTC
TTTTTGAGAGGGGTGAGGGGGTGGCAACGATTATAATAGCAAATACAAAACCTTTTAAACTTGAAGGTTGAGATTCTATATTATTCAAATACTTGAATTA
AAGAACTTTATTATAGTGAACTATCAAGCTGGACACATATTGGCACGCTACACTTGCTGTATTGTCTCCTATGCTGTTGTTCACACTTTTCACACACAAT
TGCTCCGACAACTGAATTGAGCTAAATTAGGGACAATTAACTTTTTGCCACTCTGGAAGGGTGAGTGGCAAACAAAAATGCCACTGGACTCACATTTCAG
TGACTCAGGTGACCTCACGTGTCAGTGGCAGCGGGTAATTGCAGTTGTATCATAGTGGCAAAAAATCAATTGCCCCGCTAAATTAGCCTCGCAATACCTT
GTGCTCAGGTCTAGGGACAAAACGGAACATGTCAGGATGGTGATGATCTTGCTGGAACATGTTTAAGCTGAAATTAGATGACTGTAGGTTTTCTCTTCGT
TTTCTAGAATGTTAAAGGTCATCGTCTAAAATGTTTCACTATAAACCGGAAAGGATTCATGCGGCACTCATTTTTCTGGAGAATGGACTAAAACATCTAT
TGACTGCTGATCATTTTCAGAAGATGAGAACTTGAAACAGGTACCCTTTTTCTACTCTCTTTCGTGTTTCTTTATCAACTGTCTTTTTGTTAGTTTCATC
TTGAATATATGTATTTGGGTTGAAAAGGATCACATGGTTTGGCTTCCTCCAAAAAGAGGTTTGAGTATACGAGGCACTGCCTGGATGGATGGTTAGAAGG
ATTTCAAATACCGTGCTTACTGTTCATCGAGTAACAATCTGTATTATCTGTGCACATTTGCAGTCTATTTTCCCAAACTATGAACTGTACCGTACTTAGG
TTCTGTGCATATGCAGCTCGACATTTTGGGTGTTTGGTGGTTGAATGAACCTTCACCCTTCAATTTATCTGTAGTTATTTGTGATTTGAAGCATCTTCTT
TATATCTGTTCCACACTGCTGCCAATTTGATTATTGATATAATAAATTAAGCTTTCAGATCGTATAGTAGAAATACACTATCTTTTAATGCAATCCTGGC
TTTGCTTGGTATTTTATCTTCTCGAAATAACTTGTGTTCATTGGGATCTATGTCAGTGCTGTGTCAGCATCAGCTTCGACTTACTGGCAAGCTTCAGGGC
AGGCTAAGCAAGGCCCCTAAGATTTCATCCGGACTACAGCTAAAGTGAAAAAGGGTTTATTCATGATAATTTCAATCTAAGACCTGTTTAGTTTCTATTA
AGGATGCTTAAGTATACCAACATTTTTTTTATCTTTCTTGCCAAATCTGCAAGAAAGCTTGGTCAAGAGAATCTTGCCACAACTCCCTTTGAGATGCCAA
ATCTGCGGTGAAGCGGAAAATTATAAAAAACTTTTGGCATTGCTATCCTGTGTCTAAGAAACAAACATCATCCTAATTTGGTGGAATTCGCCTGATGTGT
TGTAGCAAACTAAGGGGAACTGTCTGTCAATACACCTCAAGTGCCTATATGTAGACCGGACAATATTTCAACGCCTCTTAATTCAGAGCAGT
>comp79373_c0_seq32
GAAACCTGGTAAACCCTGGCCACGGACGCGCATGCCATCCAAGATGACGTAGTCCACACCGAATCTTCCACGTCCGCACACGCGAGCACAACGCCACCGC
AGGCCAAGTGCCGTCTCCACCACAACTCGCTGATCCCTTCCTCCTCGCCGGCTCTCTCTCCATCCCTCGTCCTTCCCCATCTGTCCCTCCCTTGGCTTCT
TCCTCCAAGCTCCAACCAATCCCCACGCGCGCGCGCGCTATAGCCTACAAATAAATACTCGCCACTCTTCCCTTCCTCCCGCCTATCGTCTTGCCAAGCG
CTAGAGCAGCTGCGCCATTGCACGCTTCCGGCCCGTCGTCCTGCACATCTGTCCTCCCCGTACTGCCTTGTACAAACTCCGTATGTAAACTACTGGGTTG
TCGCCAGTGTTCGTGTTTGCTGATGCTCATCTAATTATCGTCGAGGAGATCCAAGGAATTGTCCGCGGAGCGCCGGGGAACAAAGGCTGCGCGCGTTTCA
AAATCAGTTATTGAAACGGCAAGATGGTTAATTTTGGAAAGAAGTTGGCTGCAGATCAAGTAGAAGAATGGAAAGGGTACTATATCAATTACAAACTGAT
GAAGAAAATGTTAAAGCAATATGTTCAACAGACCCAATTAGGCGGGAAAGATTGTGAACAAATCCTTAAAGAGTTCTCGAGGATTCTTGATGACCAGATT
GAAAGGATTGTGCTTTTTCTGCTACAACAACAAGGCCACCTTGCCAGTAGGATTGAGGAACTGGGGGAACGACGTGCTCTTCTGAAGCAGTATGATATAT
CACAAGTTTCTCAGCTACGCGATGCATATAGAGAAGTTGGGCTTGATCTTATAAAGCTTCTCCGCTTTGTTGACATGAATGCTACCGGTATACACAAGAT
ATTAAAGAAATTTGATAAGCGCTTTGCCTATAAGTTTACAGACTATTATGTCACCACTCGTGCAAACCATCCTTATTCTCAGCTTCAGCAAGTATTTAAG
CAAGTGGGAACCCTAGCTATTACAGGTGCCTTGTCCTGCAACCTTGCATATCTGGAGGATCATCAAGGAGGCATTTTATCCATCTATGACCACCCATCAA
TTAACTTCAGGGACCCTATAATTAACCAAGTAAATCATGCAGTGCAGAAACTCACACATGCGACAAACTTTATGCAATTCTTAGGACAACATGCAGTTAT
CATCCCAGAAGATGTGCAAAGCGGGTCAGAGGATCTTGTCAATGATCAGAACTACCATTTCATGTCTCTTTTGCTTAACCTACTGAACACGTTTCTTTAC
ATGGTGA
>comp80692_c0_seq58
GCTCCTTGAGAAACACCATCCAATTACAAGACTATTTGATGCAACAATCACGCATATGGCTGCGCTAGAACTACTGCTCTGAATTAAGAGGCGTTGAAAT
ATTGTCCGGTCTACATATAGGCACTTGAGGTGTATTGACAGACAGTTCCCCTTAGTTTGCTACAACACATCAGGCGAATTCCACCAAATTAGGATGATGT
TTGTTTCTTAGACACAGGATAGCAATGCCAAAAGTTTTTTATAATTTTCCGCTTCACCGCAGATTTGGCATCTCAAAGGGAGTTGTGGCAAGATTCTCTT
GACCAAGCTTTCTTGCAGATTTGGCAAGAAAGATAAAAAAAATGTTGGTATACTTAAGCATCCTTAATAGAAACTAAACAGGTCTTAGATTGAAATTATC
ATAAATAAACCCTTTTTCACTTTAGCTGTAGTCCGGATGAAATCTTAGGGGCCTTGCTTAGCCTGCCCTGAAGCTTGCCAGTAAGTCGAAGCTGATGCTG
ACACAGCACTGTTTCAAGTTCTCATCTTCTGAAAATGATCAGCAGTCAATAGATGTTTTAGTCCATTCTCCAGAAAAATGAGTGCCGCATGAATCCTTTC
CGGTTTATAGTGAAACATTTTAGACGATGACCTTTAACATTCTAGAAAACGAAGAGAAAACCTACAGTCATCTAATTTCAGCTTAAACATGTTCCAGCAA
GATCATCACCATCCTGACATGTTCCGTTTTGTCCCTAGACCCGAGCACAAGGTATTGCGAGGCTAATTTAGCTCAATTCAGTTGTCAGAGCAATTGTGTG
TGAAAAGTGTGAACAACAGCATAGGAGACAATACAGCAAGTGTAGCGTGCCAATATGTGTCCAGCTTGATAGTTCACTATAATAAAGTTCTTTAATTCAA

GTATTTGAATAATATAGAATCTCAACCTTCAAGTTTAAAAGGTTTTGTATTTGCTATTATAATCGTTGCCACCCCCTCACCCCTCTCAAAAAGAGAGTTA
CTTTGTAGCGCACTGTAAATCTCTTGATTGAAATAGTGGCATGTCATCTTATTTAATTCAGTTATTTCATAAGGTACAATTTGTTTAGCTGACAGCTCAG
ACATGCTTCTATGCAACATGTTTATACCAATGCTGTATTTTGGCAATCCTGGATGTGGCATTACTTAACTGTGCTTTCATATTCAATAGTTCTGGCAAAG
GGAGGCTGCAAGCTTGAGACAACAACTGCACAACCTGCAAGAAAGTCATCGGTATGGCTAGAAAACCCTGCAAATTTTACAAATGTACAAGTCAGAAAAT
TTGCCTAATGTAAAAGGATCAAACTACAGGATTTGTCAGCATGAGCACAAGTGCCTAACTTCCGAGTTAGATAACTCTGGAGGAAGGAAATCCTTCCTAC
TGTTTGTTTCAAGAATATACAAAAATTCAAAACAACATTAGAATAGAACCAAAAGAAGAAACAATATTCACCATGTAGCAATACTCAATTCCTGGTTGTC
TTATTTATAAAGGTATAAAACTAGGTTCTGACGAGACAATTATGAATCAACTACACCATGTTACAGATCATCCATAACTCTTGGTGATGCAAATTAAGGG
CTGTAAAGTGACAAGTATACCTCCACCAACTAATTTGTGCAGAGTGTATAAAATTGTAAAACAATGGATAAGAATGAATTTAGTATACAAGATAGCATAC
AATGCCCAAGTTTAAGGAGACATACTGATTTACTGACTCCATAGGACAATCTTTTGCTTATGAGGACATCAGTTTTCATAAACAATATCAATGTAAAAGG
AGGTAACAGTTTATGAATTTCCTATGCCAAGCCATTTATAAGTCAAAAAATGTACCTATTTAGCTTTTATGCATTCCAATTAACTTCCAAAGAAGAAGAG
TACATACTAATGGAAAACAAATATGATATTATCATCTGTTTTGCCCGGTAGTTTCTTGAGTCTCTGATTTGTCCGCACTTCACTCCCAACCAACACAACA
TTAATACACATGGGCAGAGGAATATCAGGTTATCAGGTAAACTGAATACAGTTTGATTGACTGCAGTCAGTTGATGGGACAAGATCTTTCTGGATTGAGC
GTCAAGGAACTCCAAAATCTAGAAAATCAGCTAGAAATGAGCCTACGTTGCATCCGGACAAAAAAGGTAAGGATGCTAGCCATATTTCTGTTTTTCGTTA
CCAGATTGCCTCATCCAAGAATACAACTGATGATGACGTTTTTTACTTTCAGGACCAACTCTTGATTGATGAAATTCACGAACTGAATCGAAAGGGAAGT
CTCATGCAACAAGACAAGATGGAACTATACAAAAAGGTCAACCTAATCCGTCAGGAAAACATTGATTTATACAAGAAGCTCTACGAGAAAGAGGTGACAA
GTGAAGTCAACCGAGATTCATCAACTCTAAACTTTGCAGTTGTCGAGAACGTCAACATTCCTGTTCATCTTGAACTTAACACTCCACCGCAAGAAAATGA
TGTTGAGCAAACTGCACCTCCTAAACTAGGATTACAACTAAATCCATGAAGGCATGCAGCATGGCACTGCTAATGTTCATTATTTTCAACGGCTAGGATT
CAAAGCAGCTAGAATACCATTGGTTGCCTAAACACATAGCAAGACAGACGTGGCATGCTGGGCGACGGTAATGGTAGTGTAAATCTATTTTCAAATAAAA
TGTATTTTATTGAAGACTACAGAAAATTATTTGTCTTATAACTATTTCACGCATTCTGCAAGTTTGTTCACCAACTTGAGTACATGACAGCTACAAATCT
TATATTGACAAAAGATATGGTGTGCGGAAATAATTCAGGTACTGTTTTTCCTAAAAAAA
>comp65473_c0_seq14
GCCAAGAAGAGCCGCCGCGGCCCGCGCTCCCGCAGCTCGCAGTACCGCGGCGTCACCTTCTACCGCCGCACCGGCCGCTGGGAGTCCCATATTTGGGATT
GCGGCAAGCAAGTGTACTTAGGTGGATTTGACACTGCTCATACTGCTGCAAGGGCGTACGATCGAGCTGCCATCAAGTTCCGTGGCTTCGACGCCGACAT
AAACTTCAATCTCAGTGACTATGAGGATGACATGAAGCAGATGAAGAGCCTGTCCAAGGAGGAGTTCGTGCACGTCCTGCGACGGCAGAGCACCGGGTTC
TCGCGTGGCAGCTCCAAGTACAGAGGCGTCACCCTGCACAAGTGCGGCCGGTGGGAGGCTCGCATGGGACAGTTCCTCGGCAAGAAGTACATATATCTTG
GGCTATTCGACAGCGAAGTAGAGGCTGCAAGGTTGCTGATCTTGGATTCTTCTATTGATGCAAAAAAAATTTTGCTTTACCTTCTCTGTTGATTTCCAAA
GGGTTGAGTACCGACTCGATTCTCTTCTTTGTTGTTTTTCTTCTTTCTGTTTCAAAATTCAGGGCTTATGATAAGGCCGCGATCAAATGCAATGGTAGAG
AAGCCATGACGAACTTCGAGCCTAGCACGTATGATGGGGAGCTGCTGACTGAAGTTGGTACTGAAGGTGCTGAAGTCGATCTGAACTTGAGCATATCTCA
ACCAGCTTCTCAAAGCCCGAAAAGGGACAAGAATTCCCTTGGTCTGCAACTCCACTATGGATCGTTTGAAGGCTCGGAATTGAAAAGAACAAAGATTGAT
GCTCCTTCTGAACTGGCGGCCGCCCTCATCGGTTCCCTCTTCTGACCGAGCATCCACCAATCTGGACTGCCCAGTCTCACCCCTTCTATTCAAATAATG
AGGTTAAGTGATACTAAATTACTAAAATACATGCAATAACTTTCTCAAATATATTGCTTTAGCTCCCTAAACCTCACAATTGCAAGCATGTTCTGAGCAT
CACATGGTTCGGATCGAGCTGTCCGGCACCAATTTTGAACGTAATCTTGACCATCTGAGGAACTGATTGTGCAGCCTGAAGGCGTGCGTTCAAATGTTAG
ATTTGTGTTACTTTCGTTCATTTTCACTTCTTGTGCCGCTGTCAATTTTGGCTCTAGTCTTACATCACCGCTTCATTTCCTTGGATTCTATAGCCCGCCT
GTTTCCTGTCGATTTCGGAGATATTAATGCGTAGCACAGGTTTCATATGTAGGATCTTGAGTTTTCTGAGATGGCCCATTCTGGAAACAGTAATAGTCGC
CTTAGGAATAGAGATGTGTTTGATTTTTTTCCTTCAAAAGGAACATCCTAGAGAGGTGACTATTCGACATTAGGTATGCTAATCCATCGGTCAACTATAC
CTGCCTGCGGCTAAGCACCTTTCAATGTTTCTTTTTTCATTCATCGTATCATATAAAAGTAGTAAGTATGACTATTATTCAAACCTTTCTTGAAAAAAAT
AGTTAGGATGTTGCCATCTTGTAATGCAATCATAGAATTGGAAGTGCTGAATTTGCCATTTGTTTCAAACAAGCATTGTATATTGGTTTATCATTCAACT
TGAGAGTTTGGTCAAATTGAACTCTCGAACTTTTCAACTCCTAAACTTTAAGGTCAGGCTCACATATGTCATTTTCAAATTTTATTGGACGGAAAGTTGA
GCTGGACGTTTCGAGCTCGGCTTTCTATCCAACTGAACTAATGGAAACACTGATTTCATCGCAACAAATTTTCAGCAATAGATATTCAATCTCTTCTTGA
ATTCATCCTTCATCAGTATGGTGAATAAATTGTTCTTGGATTATGCAGAATGCATCAAGAGATCTTAACAGGAGGCCAGATCAGGGGGGTGTTCCCAGCT
GGGCGTGGAAGGTGACCGCCCCTCCTCCCACGCAGCCATTGCCGCTGTTCTCGTCGGCGTCATCATCCGCTGCAGCATCATCAGGATTCTCCAATACCGC
CACGACAGCTGCCCACACCGCCCCATCGGCCTCCCTCCGGTTCGACCCGCCGCCGCCGCCGTCCAGCCGCCACCGCTGAACGTTAAGAAGCCACGCTGTA
AATTTGCCAGGAAGCCGACATTTTTTCCTCTCTCGGCGTTGCAACTTTTTAGGTTTTGCGCCGGGGTGGTTTCTTGTAGTGGAGTGGATTCATAATTAAC
TGTATTTGCATGCTGCCCATGTGAAATGCTTCTCTCTCTTTTTACGCTCTCTGCACCGGATGTTCTGGGAGTTCTTGTTGCCGGGAATTAACTAAATTCA
CCGTCTGAGATTGATCTATACATTGTTGTAGAGAATCGAATCATTGTTGAAATTAGTTACAGAATTATAGATATCATAAGGCACCCTCTACTTGTGGACA
AAGTTCAGGAAGATTATTCCGGTTCTTTTCTTGGCTGTCAATTCGGCTCTTGTCCTTGGTGTGGAGCCACTTGCCTACTGTCTTGTTCTGTTGCTGTTTG
TTTTTGGCTTCTTGCACAGTTACAAATTTGTTGGCTCGAATGGATGGTGAGACATT
>Locus_12535_Transcript_50/73_Confidence_0.274_Length_3312
ATCTCCAACTATGGCAGTGGTGACAAGGAGCAAATTTGCAATCTTAGGGTGGTTCTCGTCGTGAAACCTGAAAGTTTGGGGAAATAAATTTGGGGGTGTG
GATGTCATGATATGTAGGGTCCGGCACTGAAGTGGTTGATGCTGTGGTTTCCAGGTTAAGGGGAGAAGAGGAATAGGATATAGATCTCAGGAAGCCAGAG
ATGAGGTCGAGGGGGAGCCAGTGGCCAAGCTTGTGCTCCAGTGAGCCTGATTCTCTTGTTCCCAGCTGATCGATGTGCCACTGCCTTGGTTCGCTGGATC
AGCGGATCTCCATGGTTCTGTGCGGTGCTGCAGATTGAGAAGAAGGTTTTCCAGGGGGAGGAATCTCTTGTGGGTTCCTTTTTCTGGGAGCAACGTAGTG
GGAGGTAGCTGCTTTTGCATACAATTAGGAGCAGCAGTTGAGATATTCAATTCGGGCATGGGTTCTTTTGGGATGGACTGGAACCAGAAAGGCTCGGTGT
TGTGGGATTGGGAGAATTTGCCGCCGATAGGCAGAAATGCAAACGAGAACCTCAAGATTGCGCCACAGGCTGAACCGAAGTTTGCAGGTGTTGAGGCAAC
AAGGCATGAATCGGGGCATTCTTCTTGTGGTACTTTCTCTTCCAGCTCGGAGATGGGGTATGGTTCATCCAAGAGTTCCATATCAGCGTCGATTGATTCT
TCACCCAAGGTGGGAAACAACGTGGAGCTCAATCTTGCAGCTGTCAAAGTGCCTGACAAAAACACCGTCAAGAACACTGATTTGGGTAAAGTTGATGACG
CTGGAACTTCTCCATCATCGATGATAGCCGTCAGCAGTGGAGAACCGGTGATTGGCCTGAAACTTGGCCAAAGAACCTATTTTGAAGATGCATGCGGAGG
GCAAAGTGTCAAGAGTTCACCATCCAATGTGAGTGCAGTGACCCCTGCTTCTGCGAAGAAAGCAAAGATGATTCAAAACGCACAGAACTCGTACTGTCAG
GTTGAAGGTTGCAAGTTTGATCTCTCTTCTGCTAAAGATTATCATCGGAAGCACCGAGTCTGTGAAACTCATTCTAAGGCTCCCAAGGTTATTGTTGCTG
GTCTGGAGCGACGCTTTTGTCAGCAGTGTAGCCGGTTCCATGCTTTAACTGAGTTCGACCAGAAAAAACGAAGCTGCCGGAGACGCCTCAATGATCACAA
TGCCCGCAGACGGAAGCCACAGCCTGAAGCAATTCCTTTCGGTTCATCAAGGCTCTCTGCAATGTTTTATGATGCAAGGCAACAGACAAGTCTTCTGTTT
GGTCAAGCTCCTTATGGTCAAATGAGAAGCTGTGCAAACTCTTCATGGGATAGCCCAGTAGGAGGCTTCAAATTTGGAGAAACAAAAGCTCCTTGGTTAA
AGCCAACGAGAGCTGCAGGTGTTGATGGGATGCATGTATCAAGCCAGCAGGTGTGGAACAATATTACGCCACACGGTGCACATCATGATTTTAATGGTAT
CATGGCTTTCAAGGGAACCAGTGCAAAGGTCCTTAATCAAGGCAGGTTCCTCTCTATTATAACTGACGTGACATTAATAAGTTGTGGTCCTTCACTTGCA
AGAAATCTGTTTTTAATAAAATTCCTAAGTAGAAAAAAATTCATTGATAGATGCACCCATATGATATTATTACTCTAGGAGCCATTCGATAGAATTTTAT
CCTTTGGATGATTCTTCTACATTCTAGCTGTTTGTGTATGACTGCTTGTTTGAAGCTGTTAGGTTGTTAGTACTACCTGACATGAAGTTTTATGAAATTT
GTGACACATACCTACATCATTTCATCATACAAGTGAGTCACCATACAAGTATATGGCATGTTTCTAATGTTGCAATATGAAAGCCTTTCTTTGTGAGGGA
GGATATGAGAACCTAATTGCTAGGCCACATTCTTCTCCTTGTTTTTATCTTGGTCAAGGAATGACCATTACCTGCCATTTTCAGTAACATCCAAGTAATC
TCTGTCTTTGCAAGATGTTTGCACATTTTTCCTCTAGGCTTAAATGCAGTAGTGTACCATATATGTTTTGCAAGTGTTGAGGCAGCAAGGATTTGCAGTT
CATGTAAAAATTATCCAGTTTACCAGAGCTGTCACCATCCTTTAGTGGCTGTCACCTAAACCTTGAGTCAACTCTTCATGATATGGCCACAGGACACAGC
ACGCTAAACCAACTTTTCAGACACCACTCCAGCATGATTGGATCCTAGGACAGTTTGTCTGTAAGGTGCGTGGTGTATGTCATGGTTGGCTTCAAGATTT
GGTACGTTGGGAGAAAGATTGCAACCGCCAAATGCTTTTCGTTGTCTTTAATTGATCAATTGCCTAGAGCATCCATATTCCAGGAAAGGCTAACTGATTG
ATTGCCTTAATGTCACTGTCTAGTGTGAAGCAATGATTGCGCCTCTGCTGCTCAACATGTGTGGGTTGATGGTGTTACAAACGCAGGCAGGCATTGGAGT
CATAATGTTGTTATTCTGTTCAATGATCTTATCATTATTAATTCCAGTAGGACCATGTTCTCCATATGCAAAATCTTGGCTCATCTATTAACTAGAGTAC
TAACTGAATTTCCCATTGCACTCTTCTCATATAGTTGAATTATAGCCTTCATAAGCATGTTCAATAAATTTGAGAATTTTGATTATTGATGAACATAATA
GTAACATTTTTCCTACCTCTTAACATAATAGAAACATTTTTCCTACCTCTTACCCTTTTTCAGGTGCCGAAGCTTCTCCGGTCGTCTCCAACTCGAATGG
AGCCCCAGATCTTCAGCGTGCTCTCTCTCTTCTGTCAAACAATTCAGCTGGTGCTGCCAACGACCAGCCAACTCCTCAGCTGCACCCTGGGCTGACCACC
TTCGTCGGCTCCTCCAACCCTGTCACTGTGATGGAAGCCTCACCACCAGGACTCTGGCAAGACAGCACGCCCCTTGATCATCAGGCGCAGTTCCAGGCTT

TCGATCCCATTGCCGCACCGCAACAGCTCCAGCTCCCAAAACCCCCTTCCTTCAACAGCTCCACTTCCAGCTATGGCCAGATGCAGTGATGGATGCTTCC
TTCAGTTCTGCTGTCTGAAACTCTGAAATTTGAAACTGCTGTGTTCTAAAAAATAAAGAAAAGCCTGTGTGCGAAAACTCAGGCTTGAAAAATTGATCCA
GTAGTGTTCCCGTTCAATGGCTTGAATAATCCATCCTTGTGATGGCGTGTAACTCAAATGCTTGGATTGCGCTGGCAAGTGATCATGGACTCTTGTGTTC
TTGATGGCATCT
>Locus_5523_Transcript_59/107_Confidence_0.112_Length_816
TCTTTGTTAGGGAAGCACCAAGCACCTCAAGGCTGCGAACCAATCTCAAAAGGGGATACAGGATAGGATAGCTTCCTTCCTTCCTGTAAGGAGGATCAGA
AAGAGAGAGATAGGGAGGCCAAATCAGGCTTCTTCAATTAGTTAGAGTAGAGAGAGATCTGTGCTACCTGTGGGGGAGAAGATGGGAAGAGGGAAGATAG
TGATCAAGAGGATTGACAACACGACAAGCCGGCAGGTGACGTTCTCCAAGCGCCGGAGCGGGTTGTTCAAGAAGGCCAAGGAGCTCGCCATCCTTTGCGA
CGCCGAGATCGGACTCATCGTCTTCTCCAGCACCGGTCGCCTCTATGACTTCTCCAACACCAGCATGAAGTCTGTGATCGAAAGATACAATGAAGCAAAA
GAGGATCATCACCTGACCATGAGCGCAAGTGCGGAGGCTAAGTTTTGGCAAAGGGAGGCAGGAAGCTTGAGGCAGCAACTGCATAACTTGCAAAGAGCAC
CATCGGTCAGTTGTTGGGTCAGCAGCTTTCTGGCCTTGATGTAAAAGGCTTGAAAGATTTGGAGAATAAGCTGGAGATGAGCCTAAGAAATATTTGTCTG
AAGAAGGACCAACTTATGATTGATCAAATTCAAGAATTAAACAGGAAGGTTCGCTCTTACAACTTATTTAGCATAACTAGTTTGTTCTCATGTCTTACTT
AACTACACAAACTTCTTATTTGAATTATAGGGAAGCCTCCTGCACCATGAAAACATAGAACTATACAACAAAGTCAACCTTGTTCAACAAGAGAACATCG
AATTACAGAAAAAGGT
>Locus_7001_Transcript_52/84_Confidence_0.209_Length_2572
TCACTTTAGCCACCTCAGATTTGCTGTTTGTAGCTGGTGAATCTGTCCTGTTCTAGTGGTTTTTTTGGTCCCATGCTTGCTGGATAAACGGATTACGGTG
TAGGTGTTCCGTGTTCTTGTTCAAGTGCTTTGGACTTTTGTGTGGTTGCGGTTCTTGCTTCCTCTTTGTTTCTCAGCAGGATCTTCAGAAAATTCCTTCT
GCGAGTTATTGGTTGGAGAACACATTCTTGTCTGTAGGTTCATCCTTGCTTGGCTAACTAGAATACATTAGTCCTTTCATAATTAAAAAAAATACATTAG
TCCTTCAGGCAGTTCAAGCTTGCCTGGAAATCTTGCCTTCTGCCTTCACTTTTGCTGATTTGGTGTTCTATTGTGCTAATTTTTGCGATAATTTTTAAGC
TAAATTTGGGTGGTCAGTACTGCCCTTGAGTGATGCTTCAGATTAGTTACGTTATGTGGCTATCGGAGGAATGGAGAAGGGGTTAAGAAGGAAAGCGCTG
AAAGTACGGAGGACTTCGTCGATATTAGCAGGCATAGGTTTATGTGATTTGTGACAGATATGTTCTGCTTTGAGTAATATCCCAGGTTATGGGGAACACG
GATAATAAAAGTAAGTATTTAACGAAAAAGCTGGATTTCTCTGTTTCGTGCTTGCGACGATTGAAAGGTTTAGAAGGATAATGTAGGATCTTCCGTAGGG
AAGATAGTATAGACTAGGGATCCCTTTGAGTCAATAATTCTTGAGAATCCTGTGGTAATCTGAAAGTAGAGCTGAAGGAAGTATAGTGCGTTGTCAGTTG
CAGGACAGATAAATTATTGCTACTTGGAGTAGCTTAGAATTATCACTATCAGAACTTCTGGATGAAGGTGATATGTTGTGCTTCCTCGTCTTCTATTTGG
ATTAGCTAGCTTGCAGCACTCAGAAGCTCTGCTTATCCTGGAGCAGGTATAGAGCTTAGGGTTAAGTTATCATAAGGATGCCAGAATATTATTTGCTAAT
GAGAATATTGATGCCTCACCATGATCTTTCGATTTATTTTATGATTCTGTCATTTTCCAACTGTTCATCTAGCTGTGTTAGGGTACAACTGTTCATCTAG
CTGTGTTAGGGTACAACTGTTCATCTAGGGTTCAACCTATTTTATATTATATGAGATAATGAATAACCAAATTCAAGTTTCCACATATTTACCTGGTACT
TTGGCATTATTGGTGCATACTGTCAGTTCAGCTGATCAATTCTTGCATCAGCAATTTGTTCTGAATACCTTTTCTAATGTTGAAAACAGGAAGAAGCAAA
ATGTTTCTGGAGCTTGAGTGATGCATCTGATAATGAAAATGCGTCTGCGAGAAATGGATGGTTATCCAGTCCATTCCATGCCCAACTATGATTTCCTGTC
TGGGAATGGCTATTCAATGAAGCAGTTTGTCCTTGGCAATCCTGATGGAGACTCCTCATCAACAAAGTCTGAGCAGTGTTGCCAAGATTTATCTGCAGTG
AGTGACAGCAGCCTCAATGGACAACACACACCAACACAATCTGACAACAATGATAGTTGTGGGAAGGTAGACCAGGGCATGGTAAAGTCTGTACTGTCCT
TGGGAAACCCAGAAGCTACATTTCCCCCTCCAAAGTTTGATTACAGCCAGCCTTTGGCTTGTGTTTCTTATCCGTATGCTGCTGATCCATATTATGGTGG
GGTCTTAACAGGATATACATCAAATGCTGTTGTTCATCCCCAAATTAATGGTACAGCAAATTGTAGGGTCCCATTGCCTATTGAACCTGCAGCCGAAGAG
CCCATATTTGTCAACGCAAAGCAATACCATGCAATCCTCAGGAGAAGGCAAATGCGTGCAAAATTTGAGGCCCAAAATAAACTGGTGAAAGGCCGGAAGC
CGTACCTTCACGAATCTCGACACCGTCATGCAATGAAGCGAGCCCGTGGGTCGGGAGGCCGGTTCCTTACCAAAAAGGAGCTGCAGGAGCAGCAGCAGCA
GAAGACACTGCCTTCACTTCAGACTCCAACATGTGTGGCAAGTAAAATGTCACTCGGCAGGAATCAATGCCCTGAAAACAGCACATCTCGCTCGCCTTCT
ACACCGACAGACTCTGGGATCTCAAGTGTTTCCAACGGAGGTGGCATGCTGGCTCACCAAAAGCACATCACCTTCACATCTACTAATTTCCTTCCCAGCA
TGAACTTCAGCACCCAGAATGGAGGTGAAAAGATGGCTGTCAATGGTGCGCACCACCGTGCCTCCATCGTGAGGTGAACAGCCTCGATTGTGGGGCGGTG
GTATGTACCATCACTGGCGCGCTCCGGTGAGCCCGTGGCAATTCATCCTTGGCTTATGAAGTATCTGTATCTGGTAATAATTTGCTTTCAGTTTAATATG
TAGAGCTTGTCCGAGATGAACTCATTTATATTTGTTGCAACTAAAACGAGGAGCTACTTGTTGATATATGTGGATATTGTGGATACAGAAATATCCTGAT
GTTTCTTGATGTACCAAGTCCACTATTAAAGTATGACTGGAAAAAACGTTTAATAACGTAATCATGGTTTGG
>Locus_11912_Transcript_37/63_Confidence_0.203_Length_2515
CCCTCCCCGCCGGCCACCTCGGGGAGCTCATCCCCGCCGCTTTGCCGGCCGCGCTTTCCCTGGCGCCTCCCGCGCGGGCGCTCGCCCGGGATTTTTCTCG
GCGCCCCCTTCCGGCGGACGCCTTGACCGGGGTGGGTTCTTGGTCGCGGCGGCGCGTGATCTCTGCGTCTTGAAGAAGGTGTTGCTAGCGGGTTCTGCGG
GGGGTGGGGAGGACGGAGGAAGGAAGCCGGGGGGGACTGCGAGGCGTTCATGGGTTAGATCTGGCGGTCCGGCGCCCGCGGATCTGAGCGAGGTTTTAGG
TGGAGGGATTGGGGCCGAGAGGTCGCCGTCGTCGGCTCATTTTGAGTAGCTTGGGCCTCTCATGTCCAAATCGGGGGCGGGTGATGGATGCGGTAACTGT
CGGAAGGAGCGTCGCTCGCAGATCTGAAGGCCGTCCAGCTCCCTTGTTCAGTTGACCTGCTCGCTGGTCAGCTGGATGGAGTGAATGGCAATGCTTTCTG
ATGTTTGACTGCCGTTAGTTGCTGCTTAGTGTCTGTACAAGAAATTTAGCAGGAGGTGGTGTGCTTATGAGACTCTTCTAGGTGCTGATATCTTGGTCTC
TGTTGAATAACGCTGGAGTTGGAAGGAAGAAGATCACATTCCTGTGTTTTTGTTGGTATCTGTGTCTTTCTTGAATAACCTTGAGGCTGGAGGTTATCAG
ATCCAGTTCATGTGGCCCTGCTAATAAGGTGCTGGTGGGGGAAGGTACCATGACCTACTTCCCTGAGGAGGTGGTGGAGCACATATTCAGCTTCTTGCCG
TCGCACAGCGACCGGAACACAGTCTCGCTCGTGTGCAAGGTGTGGTATGAGGTCGAGAGGTTGAGCCGGCGAGCTGTCTTCGCGGGGAACTGCTACGCGG
TGCGCCCGGAGCGGGTTGTGCTGCGGTTCCCCAATGTGAAGGTCCTGACGGTGAAGGGGAAGCCGCACTTCGCGGACTTCAACCTCGTGCCGCCTGATTG
GGGTGGCTACGCGGGGCCATGGATTGAGGCGGCAGCGAGGGGCTGCGTGGGTCTGGAGGAGCTGCGGATGAAGCGGATGGTGGTGTCGGATGAGAGCCTT
GAGCTGCTAGCTCGGTCCTTCCCGAGATTCAAGGTCCTCGTCCTCATCAGTTGCGAGGGGTTTAGCACCGATGGGCTAGCAGCTATTGCGAGTCACTGCA
AGCTCCTGAGGGAGTTAGATTTGCAGGAAAATGATGTGGAGGACCGTGGGCCTAGGTGGCTCTCCTGCTTCCCTGATTCCTGCACCTCTCTTGTCTCCTT
GAATTTTGCCTGCATCAAAGGGGAGGTGAATCCTGGTTCATTGGAGAGACTTGTTGCTAGGTCTCCGAACCTACGCAGTTTGAGGTTGAATCGATCTGTA
TCGGTAGATACACTCTCAAAGATATTAGTACGCACCCCTAATTTGGAGGACTTGGGGACAGGGAATTTGACAGATGAGTTTCAAACTGAATCCTACTTCA
GGCTGGCTAATGCTTTGGAGAAATGCAAAATGTTGAAGAGTTTATCAGGATTTTGGGATGCTTCTCCTGTTTGCGTTCCATTTATCTATCCCCTCTGTCA
TCAACTAACTGGACTAAACTTGAGCTATACTCCCACTCTGGATGCTTCTGATCTCACTAAAATGATCAGCCGCTGTGTGAAACTCCAACGTCTTTGGGTA
CTGGACTGCATCTCAGATAAGGGTTTGCAAGTGGTGGCCTCCAGTTGCAAAGATCTACAAGAACTCAGGGTGTTCCCATCAGATTTTTATGTTGCTGGTT
ATTCTGCCGTGACAGAAGAGGGGCTTGTTGCAATATCGTCAGGCTGTCCAAAATTGAGCTCTTTGCTGTATTTCTGTCACCAGATGACCAACGATGCACT
TATTACTGTAGCTAAGAACTGCCCAAATTTCGTAAGATTTAGACTCTGTATTCTTGAGCCAAAGAAGCCAGACGCCATGACAAACCAGCCGTTAGATGAA
GGCTTTGGTGCAATTGTTCGTGAATGCAAGGGGCTAAGGCGACTGTCAATGTCGGGTCTTCTCACTGACAAGGTTTTCATGTATATTGGAAAGTATGCCA
AGCAACTTGAGATGCTTTCTATAGCATTTGCTGGAGATAGTGATAAGGGGATGATGCATGTTATGAATGGATGCAAGAATCTAAGGAAGCTGGAGATTAG
AGACAGTCCATTTGGTGATGTTGCGCTCTTGGGGAATGTTGCCAAGTATGAAACAATGCGATCCCTTTGGATGTCATCATGCAATGTCACATTAAAGGGG
TGCCAAGTCCTTGCGTCTAAGATGCCGATGCTCAATGTGGAGATCATGAATGAGCTAGATGGAAGCAGTGAAATGGAGGAAAACCATGGAGATCAATCTA
AGGTGGAGAAGCTATATGTTTACCGCACAACTGCTGGAGCGAGGGATGATGCACCAAATTTTGTTAAAATCCTATAGTTAGCTGAGAGTAGCCTGGTATC
TCCTCACTCTAAAAG
>comp82727_c0_seq22
GATTGGATCTTTCGGTTTGGTTCCTTCTTGTCGCCAGGGAAATTCTTGCATCCTCTGGTGCTGTACATGTATGGGCTGGCATGGCCTTCCTGATAAGTTC
TTCCTCCTGAGCTCCGGCTTCGAGGCTGAAATCTTGCTGACCTCCATTTCAGTTCTATGCTTCTCGATGCATATTATCTTTTGATTCAGTTCTTGGAAAA
GTTCGGCCTTCTCGGTTCTTGTTGGTGGGTAGGTGCTTAAATACATCTGACCAAGAGAACATCGGAGCAACCATTTGAGGCGGTTGCTGCGCAGCCAAAC
CAAGCAAACTCAGCTCCTGCCTCTGACTTTCTTGGGAGAAGGCGCAGCACAAGCACAACGATGAGCTTCAGGAGCCATGAGGGGTTTGGGCAGGTCGCCG
CCGCCAACAACAATGGAGCTTCGCTGCCATGGTGGGCAGCGGCGCCTCAGCTGCTGTACGGCGAGCCGTTGGGCCAGGGGAAGGCGTCGGCCATGTCACC
GGAGGAGCCGTGCCGGGAGGGACGGTTTCAGGTTGTGCCGGGGGCTCAGGCGCTGCTGGATCCTCCGGTGCCGTCGCCGCCGAAGGCCGCGCAGCCGCAG
AGAGTGCTCCCTGATGTCCTGAAGTTCTCCATGGCTCAAGGTAAAGTGGGGAAAGGCTCTGAGCATTCTGCCACTGTTGCTTTGCAATCACCATTCACAG
TATACAATGGTCGTTTTGAGCTGGGCCTTGGTCAGTCAATGCCTGCCGGGCGTATGCTTATACCACTGAATATGCCAACTGATGCCCCAATTTATGTGAA
TGCCAAACAATACGAAGGCATCCTTCGTCGCCGCCATGCCCGTGCCAAAGCAGAGAGGGAGAATAGGCTGGTCAAAGTCAGAAAGCCATATCTGCATGAG

TCACGGCATCTTCATGCACTGCGTCGCGCAAGAGGCTCTGGTGGACGCTTCCTGAACACAAAGAAAGAGGTCAATGGGAAGGACACTGGTGTCGGAAGCA
AGGCAATAGTCAGCAATCCACTAATGCGCTTGACTGCATCCCCAAGTTCAGAGATTCAGCAGTCAGACCTGGGCAACCCAAGCAGCGTCTCCAGCCTATC
CGGCTCGGAGGTGACAAGCATGTATGATCATGAAGATGTGGATCACTACCACAGCTTTGATCACCTCCGGACACCCTTTTTCACCCCACTTCCGAGCATC
ATGGACGGTGAGCATGGGGTCGGTAACCCCTTCAAGTGGGCAGCAGCCTCTGAAGGCTGCTGCGACCTCCTCAAAGCATGACTGGAGGGGATACCGGGCA
GCCGAGGGTCAGGGATCGTCCCTGCCATCGTTTGGCTGTCGGTAGGCAATTCATCCTTGGCTCAAGGCATTGCGGTGCTTTCCGCACATCGCCAAATTCT
CCTAGAAACCATAGTTGCTGGGATCTTGTGGTGAGTGGAACACCCCATGCCTGAAAAAAAATCTGATCTAAATTAGTCGTACTAGCTACTGCTATCTGAA
TCGTTTCGCGGTAGCAGGTTTCAGCTTAGTTTTCTGAATAAATCCTGAAACTTAAGTTTAGTTTGGAGTTTCAGAGGTATTGTCATTGTAGTATGTTTGG
ACTTTGGTGTCTGATTAGTATCAAGACTATGGCTTTGTTGGTGATGAATTGTGGTGTGCTTGAAGCTTGTGCGGACTTTGGTTTGTACTTTATGGTGTGT
TCAAAACTTGAATTTGGCAATGTAATAATGTACTGAGATTTGGAGCTGGATTGTGTGTTCTGTT
>Locus_3441_Transcript_409/417_Confidence_0.037_Length_1817
TGTAAATCTCTTGATTGAAATAGTGGCATGTCATCTTATTTAATTCAGTTATTTCATAAGGTACAATTTGTTTAGCTGACAGCTCAGACATGCTTCTATG
CAACATGTTTATACCAATGCTGTATTTTGGCAATCCTGGATGTGGCATTACTTAACTGTGCTTTCATATTCAATAGTTCTGGCAAAGGGAGGCTGCAAGC
TTGAGACAACAACTGCACAACCTGCAAGAAAGTCATCGGTATGGCTAGAAAACCCTGCAAATTTTACAAATGTACAAGTCAGAAAATTTGCCTAATGTAA
AAGGATCAAACTACAGGATTTGTCAGCATGAGCACAAGTGCCTAACTTCCGAGTTAGATAACTCTGGAGGAAGGAAATCCTTCCTACTGTTTGTTTCAAG
AATATACAAAAATTCAAAACAACATTAGAATAGAACCAAAAGAAGAAACAATATTCACCATGTAGCAATACTCAATTCCTGGTTGTCTTATTTATAAAGG
TATAAAACTAGGTTCTGACGAGACAATTATGAATCAACTACACCATGTTACAGATCATCCATAACTCTTGGTGATGCAAATTAAGGGCTGTAAAGTGACA
AGTATACCTCCACCAACTAATTTGTGCAGAGTGTATAAAATTGTAAAACAATGGATAAGAATGAATTTAGTATACAAGATAGCATACAATGCCCAAGTTT
AAGGAGACATACTGATTTACTGACTCCATAGGACAATCTTTTGCTTATGAGGACATCAGTTTTCATAAACAATATCAATGTAAAAGGAGGTAACAGTTTA
TGAATTTCCTATGCCAAGCCATTTATAAGTCAAAAAATGTACCTATTTAGCTTTTATGCATTCCAATTAACTTCCAAAGAAGAAGAGTACATACTAATGG
AAAACAAATATGATATTATCATCTGTTTTGCCCGGTAGTTTCTTGAGTCTCTGATTTGTCCGCACTTCACTCCCAACCAACACAACATTAATACACATGG
GCAGAGGAATATCAGGTTATCAGGTAAACTGAATACAGTTTGATTGACTGCAGGCAGTTGATGGGACAAGATCTTTCTGGATTGGGCGTCAAGGAACTCC
AAAATCTAGAAAATCAGCTAGAAATGAGCCTACGTTGCATCCGGACAAAAAAGGTAAGGATGCTAGCCATATTTCTGTTTTTCGTTACCAGATTGCCTCA
TCCAAGAATACAACTGATGATGACGTTTTTTACTTTCAGGACCAACTCTTGATTGATGAAATTCACGAACTGAATCGAAAGGTTCTGGAATATTCTGTTA
TTACTCTGCAATGAGAGGTCTCTGAGTGGATTTCAAGAAGCTGACTCTACTGGCTCATTTTCAGGGAAGTCTCATGCAACAAGACAAGATGGAACTATAC
AAAAAGGTCAACCTAATCCGTCAGGAAAACATTGATTTATACAAGAAGGTATTTGATAAATCGTGAATGAAAATTCACAGGATATACACCCCATCCATTA
TTGACTGAGGTTTATTTCTCTACTCAGCTCTACGAGAAAGAGGTGACAAGTGAAGTCAACCGAGATTCATCAACTCTAAACTTTGCAGTTGTCGAGAACG
TCAACATTCCTGTTCATCTTGAACTTAACACTCCACCGCAAGAAAATGATGTTGAGCAAACTGCACCTCCTAAACTGGGGTAACCCTGCTTTTTTCTGCA
AAGGAACTATTGATTCTGTTCTTAATTCTTTAGTGGCATATTGTTTACTACTAACTGAAAACTTGCAGATTACAACTAAATCCATGAAGGCATGCAGCAT
GGCACCGCTTATGTTCA
>Locus_17579_Transcript_38/92_Confidence_0.195_Length_1749
CTTTTTCTGGGAGCAACGTAGTGGGAGGTAGCTGCTTTTGCATACAATTAGGAGCAGCAGTTGAGATATTCAATTCGGGCATGGGTTCTTTTGGGATGGA
CTGGAACCAGAAAGGCTCGGTGTTGTGGGATTGGGAGAATTTGCCGCCGATAGGCAGAAATGCAAACGAGAACCTCAAGATTGCGCCACAGGCTGAACCG
AAGTTTGCAGGTGTTGAGGCAACAAGGCATGAATCGGGGCATTCTTCTTGTGGTACTTTCTCTTCCAGCTCGGAGATGGGGTATGGTTCATCCAAGAGTT
CCATATCAGCGTCGATTGATTCTTCACCCAAGGTGGGAAACAACGTGGAGCTCAATCTTGCAGCTGTCAAAGTGCCTGACAAAAACACCGTCAAGAACAC
TGATTTGGGTAAAGTTGATGACGCTGGAACTTCTCCATCATCGATGATAGCCGTCAGCAGTGGAGAACCGGTGATTGGCCTGAAACTTGGCCAAAGAACC
TATTTTGAAGATGCATGCGGAGGGCAAAGTGTCAAGAGTTCACCATCCAATGTGAGTGCAGTGACCCCTGCTTCTGCGAAGAAAGCAAAGATGATTCAAA
ACGCACAGAACTCGTACTGTCAGGTTGAAGGTTGCAAGTTTGATCTCTCTTCTGCTAAAGATTATCATCGGAAGCACCGAGTCTGTGAAACTCATTCTAA
GGCTCCCAAGGTTATTGTTGCTGGTCTGGAGCGACGCTTTTGTCAGCAGTGTAGCCGGTTCCATGCTTTAACTGAGTTCGACCAGAAAAAACGAAGCTGC
CGGAGACGCCTCAATGATCACAATGCCCGCAGACGGAAGCCACAGCCTGAAGCAATTCCTTTCGGTTCATCAAGGCTCTCTGCAATGTTTTATGATGCAA
GGCAACAGACAAGTCTTCTGTTTGGTCAAGCTCCTTATGGTCAAATGAGAAGCTGTGCAAACTCTTCATGGGATAGCCCAGTAGGAGGCTTCAAATTTGG
AGAAACAAAAGCTCCTTGGTTAAAGCCAACGAGAGCTGCAGGTGTTGATGGGATGCATGTATCAAGCCAGCAGGTGTGGAACAATATTACGCCACACGGT
GCACATCATGATTTTAATGGTATCATGGCTTTCAAGGGAACCAGTGCAAATGTCCTTAATCAAGGTGCCGAAGCTTCTCCGGTCGTCTCCAACTCGAATG
GAGCCCCAGATCTTCAGCGTGCTCTCTCTCTTCTGTCAAACAATTCAGCTGGTGCTGCCAACGACCAGCCAACTCCTCAGCTGCACCCTGGGCTGACCAC
CTTCGTCGGCTCCTCCAACCCTGTCACTGTGATGGAAGCCTCACCACCAGGACTCTGGCAAGATGGCGCCCCCCTCGATCATCAGGCGCGGTTTCAGCTT
TTCGATCCGGTGGGTGACGGCAGCGCCATTGTCACACCGCATCAGCTCCAGCTCCCAAAACCTCACTCCCTCAACAGCTCCTCTTCCCACTATGACCAGA
TGCACTGATGATCTGATGCTTCCTTCAGTTAGCGCAAATGGCGGTTGGGTTGCGCAAAATTAGACATGGCTTCTGATGCCTGAAACTCTGAATTTTGAAA
CTGTTATGTTCTTCTGAAAAATAAAAGCCTGTGTGCGCAAACTCAGGCTCCAAAAACTGATCTAGTAGTGTTCCCATTCAAGGGCTTGAATAATCCATCC
TTGTGGTGGCATGTAACTCAAATGCTTCGATTGCGCCGGCAATTGATCT
>Locus_2976_Transcript_14/49_Confidence_0.277_Length_1953
AGTCCTAATATGTATCGCTTCAAAAAAATAAATTTCAGTATATCAAAGTTTCATGATTGGAGCAGCATAGCCAAGCTACACTGATTGGCATATGAAGGGC
ATTGTACTCACTATGGTGCTTAGGGCAGTACAGGTAAATTGCACTTGACAAAATAGTATCGCTCAATTGCTGTGACATGTTTATTAACATGAGATCACAA
CGTTTGAAGGGAAACTCAAGCTAAATAACCTCACTTCAAAAAAGTTGGAATTCATGACTTTGGCCTGGATCCGAAATCTGTAAAAGAGCACTGCTCATCT
TGTACCAGTGAAAAATAAACTTCAGATCGGTATGCTACAAAGCTATTTGACAGATGCTTTTACAGCTTTCTCTGCCGCATCATCAAGATCCTCAGCAGTG
ATCAGTGTCATTCCACTTTCCTTAAGAATCCTCTTTCCTTGGTCCACATTGGTGCCTTCTAACCGAACAACAACAGGAACCTTCAGGTCAACCTGTTTAG
CAGCATTTACAATTCCACTTGCTATCACATCACATTTCATGATGCCCCCAAATATGTTCACTAGAATTGCCTTCACTCTATCATCTGAAGTCAGTATCTT
AAATGCTTCCACAACCTGTCCCTCTGATGCACTTCCACCAACATCAAGGAAATTGGCAGGTGTTCCGCCATGCAACTTGATAATGTCCATGGTAGCCATA
GCCAATCCTGCTCCGTTCACCATGCAACCAATCTCTCCATCAAGACCAATATAGTTCAAATCTGCTTTGGCAGCAGCAACCTGTAAGCATAGAAGTGTCA
GTGCTGCGATTAGATAACCATAAGAGTGAATATGAAAACCTAAAGTATTCGCTCAGAGGCAAGGCTTCCACAAAAATGAGGTCTAAAGGCAGAACAAGAG
TAAAAACACCATCAAAGAAAGCTCAACAAAAAAGGAGAAAAAGTGCAAGATGGGAGAAACAAGCAATTCTGCACCTCCCTGGGGTCTTCCTGTGTTGTAT
CACGCACCCTGGGGTCCTCCTGTGTTGTATCACGCAGTGCAAAGATTTCCTTCTGCCTGAATGCAGCATTGTCATCAAAGTTCAACTTTGCATCAGCAGC
AACAAGCTTGTTGTCAGCTGTCTCAGCAAGAGGATTTATCTCCAGCAGTGTGCAATCGCACTTGCAGAAAAGTTCATACAACTTCTTAATCTGTTCCATA
GAAGACTGCCTGTCTGCTGCCTTTAGGGCCAGACCATCAACAACTTTAGCTGCATCCTCATCTGTGATTCCCTTGAATACATCAATAGGAACCTTAATAA
TCATATCAGGATACTTCTCTGCAAGATCTTCAATACTGGTTCCTCCCTTGCTGCACGCAATTATGAGCGGACCAGCAGTTTTCCTATCAAGGGTGATGGC
AAAGTACATCTCATTAGTAAGTGACAGTTTCTCACACAAGTAGACCTTGCTCACAATCTTCCCCTCTGGACCAGTTTGTTTTGTTACCAGAATCTGGCCT
AGCATTTTACTTGCAATTCCTTCAGCTTCCTCAGCCTTAACAATATGAACACCACCTTTCAGTCCACTTTTGAAAGTTCCCAGTCCTCGTCCGCCAGCAA
GGATTTGACTCTTAACAACTATCTCTTTCTCACTGGGGAATACGTTCTTCAAGGCGTCCTGCACCTCCTGCACGGACCCAGCAGCCGCGCCCCTGGGCAC
GTTGATGCCGTACTTCCCCATCAACTCCGCGCCCTGGTACTCGTGGATGTTGAGGCGGCGGAGCTGCTGGTGCTGCCATCTCCCGGCGACGGAGACGGCG
CGCGACGCGAGCTTCCCCAGAGATCCGCGAACCATGGCTGCGGGGACCTTGGTGGCGCGGGGCTGGCTCCTTCGATCCGGAAACCCTACAAGCGTGGAAG
ACGATTCCTCTTTCCGGTTTGATTTTTTTTTTTTTTTTTTTTTTTTTTTTTT
>Locus_12535_Transcript_42/73_Confidence_0.330_Length_3584
TTCCAATAAGACACAGATTCTTCAGAAAGAGGTTTTCCTCTCCCTGCCCTTTTGATCTCCAACTATGGCAGTGGTGACAAGGAGCAAATTTGCAATCTTA
GGGTGGTTCTCGTCGTGAAACCTGAAAGTTTGGGGAAATAAATTTGGGGGTGTGGATGTCATGATATGTAGGGTCCGGCACTGAAGTGGTTGATGCTGTG
GTTTCCAGGTTAAGGGGAGAAGAGGAATAGGATATAGATCTCAGGAAGCCAGAGATGAGGTCGAGGGGAAGCCAGTGGCCAAGCTTGTGCTCCAGTGAAG
CTGATTCTCTTGTTCCCAGTTGATCCATGCGCCGTGGCCTCAGTTCGCAGGATCAGCAGCTCCATGGTTCTGTGTGGTGCTGCAGTTTGAGAAGAAGGTA
CTTGGTCTTTTGCACCTTGATGTACTTGTGTTTTTTTGCCTTTCTTGATGCTATAATGATGGTGGTGCTGTTCTTGCCATGATTGACGATTCCCCTAGAA
AAGGTTGAACTACTCTGCACTCTCTCTGGCACATGGTAGCACCTCGTGTACTTCTCTGATGATGTATAGTTGAGTGCTCAGATATGTTGTTGCAAATCTG
TCCAATTTCTGCTACCCATATGTCAAATCTTTTCTGTTTTTAGTCTGTTTCACTCTTCCATTTTGTTGCTTCAAAGGTAGCTGCTTTTGCATACAATTAG
GAGCAGCAGTTGAGATATTCAATTCGGGCATGGGTTCTTTTGGGATGGACTGGAACCAGAAAGGCTCGGTGTTGTGGGATTGGGAGAATTTGCCGCCGAT

AGGCAGAAATGCAAACGAGAACCTCAAGATTGCGCCACAGGCTGAACCGAAGTTTGCAGGTGTTGAGGCAACAAGGCATGAATCGGGGCATTCTTCTTGT
GGTACTTTCTCTTCCAGCTCGGAGATGGGGTATGGTTCATCCAAGAGTTCCATATCAGCGTCGATTGATTCTTCACCCAAGGTGGGAAACAACGTGGAGC
TCAATCTTGCAGCTGTCAAAGTGCCTGACAAAAACACCGTCAAGAACACTGATTTGGGTAAAGTTGATGACGCTGGAACTTCTCCATCATCGATGATAGC
CGTCAGCAGTGGAGAACCGGTGATTGGCCTGAAACTTGGCCAAAGAACCTATTTTGAAGATGCATGCGGAGGGCAAAGTGTCAAGAGTTCACCATCCAAT
GTGAGTGCAGTGACCCCTGCTTCTGCGAAGAAAGCAAAGATGATTCAAAACGCACAGAACTCGTACTGTCAGGTTGAAGGTTGCAAGTTTGATCTCTCTT
CTGCTAAAGATTATCATCGGAAGCACCGAGTCTGTGAAACTCATTCTAAGGCTCCCAAGGTTATTGTTGCTGGTCTGGAGCGACGCTTTTGTCAGCAGTG
TAGCCGGTTCCATGCTTTAACTGAGTTCGACCAGAAAAAACGAAGCTGCCGGAGACGCCTCAATGATCACAATGCCCGCAGACGGAAGCCACAGCCTGAA
GCAATTCCTTTCGGTTCATCAAGGCTCTCTGCAATGTTTTATGATGCAAGGCAACAGACAAGTCTTCTGTTTGGTCAAGCTCCTTATGGTCAAATGAGAA
GCTGTGCAAACTCTTCATGGGATAGCCCAGTAGGAGGCTTCAAATTTGGAGAAACAAAAGCTCCTTGGTTAAAGCCAACGAGAGCTGCAGGTGTTGATGG
GATGCATGTATCAAGCCAGCAGGTGTGGAACAATATTACGCCACACGGTGCACATCATGATTTAATGGTATCATGGCTTTCAAGGGAACCAGTGCAAAG
GTCCTTAATCAAGGCAGGTTCCTCTCTATTATAACTGACGTGACATTAATAAGTTGTGGTCCTTCACTTGCAAGAAATCTGTTTTTAATAAAATTCCTAA
GTAGAAAAAAAATTCATTGATAGATGCACCCATATGATATTATTACTCTAGGAGCCATTCGATAGAATTTATCCTTTGGATGATTCTTCTACATTCTAGC
TGTTTGTGTATGACTGCTTGTTTGAAGCTGTTAGGTTGTTAGTACTACCTGACATGAAGTTTTATGAAATTTGTGACACATACCTACATCATTTCATCAT
ACAAGTGAGTCACCATACAAGTATATGGCATGTTTCTAATGTTGCAATATGAAAGCCTTTCTTTGTGAGGGAGGATATGAGAACCTAATTGCTAGGCCAC
ATTCTTCTCCTTGTTTTTATCTTGGTCAAGGAATGACCATTACCTGCCATTTTCAGTAACATCCAAGTAATCTCTGTCTTTGCAAGATGTTTGCACATTT
TTCCTCTAGGCTTAAATGCAGTAGTGTACCATATATGTTTTGCAAGTGTTGAGGCAGCAAGGATTTGCAGTTCATGTAAAAATTATCCAGTTTACCAGAG
CTGTCACCATCCTTTAGTGGCTGTCACCTAAACCTTGAGTCAACTCTTCATGATATGGCCACAGGACACAGCACGCTAAACCAACTTTTCAGACACCACT
CCAGCATGATTGGATCCTAGGACAGTTTGTCTGTAAGGTGCGTGGTGTATGTCATGGTTGGCTTCAAGATTTGGTACGTTGGGAGAAAGATTGCAACCGC
CAAATGCTTTTCGTTGTCTTTAATTGATCAATTGCCTAGAGCATCCATATTCCAGGAAAGGCTAACTGATTGATTGCCTTAATGTCACTGTCTAGTGTGA
AGCAATGATTGCGCCTCTGCTGCTCAACATGTGTGGGTTGATGGTGTTACAAACGCAGGCAGGCATTGGAGTCATAATGTTGTTATTCTGTTCAATGATC
TTATCATTATTAATTCCAGTAGGACCATGTTCTCCATATGCAAAATCTTGGCTCATCTATTAACTAGAGTACTAACTGAATTTCCCATTGCACTCTTCTC
ATATAGTTGAATTATAGCCTTCATAAGCATGTTCAATAAATTTGAGAATTTTGATTATTGATGAACATAATAGTAACATTTTTCCTACCTCTTAACATAA
TAGAAACATTTTTCCTACCTCTTACCCTTTTTCAGGTGCCGAAGCTTCTCCGGTCGTCTCCAACTCGAATGGAGCCCCAGATCTTCAGCGTGCTCTCTCT
CTTCTGTCAAACAATTCAGCTGGTGCTGCCAACGACCAGCCAACTCCTCAGCTGCACCCTGGGCTGACCACCTTCGTCGGCTCCTCCAACCCTGTCACTG
TGATGGAAGCCTCACCACCAGGACTCTGGCAAGACAGCACGCCCCTTGATCATCAGGCGCAGTTCCAGGCTTTCGATCCCATTGCCGCACCGCAACAGCT
CCAGCTCCCAAAACCCCCTTCCTTCAACAGCTCCACTTCCAGCTATGGCCAGATGCAGTGATGGATGCTTCCTTCAGTTCTGCTGTCTGAAACTCTGAAA
TTTGAAACTGCTGTGTTCTAAAAAATAAAGAAAAGCCTGTGTGCGAAAACTCAGGCTTGAAAAATTGATCCAGTAGTGTTCCCGTTCAATGGCTTGAATA
ATCCATCCTTGTGATGGCGTGTAACTCAAATGCTTGGATTGCGCTGGCAAGTGATCATGGACTCTTGTGTTCTTGATGGCATCT
>comp82180_c0_seq8
AATATAGGGGGCCCCGCTCCCGCAGCTCGCAGTACCGCGGCGTCACCTTCTACCGCCGCACCGGCCGCTGGGAGTCCCATATTTGGGATTGCGGCAAGCA
AGTGTACTTAGGTGGATTTGACACTGCTCATACTGCTGCAAGGGCGTACGATCGAGCTGCCATCAAGTTCCGTGGCTTCGACGCCGACATAAACTTCAAT
CTCAGTGACTATGAGGATGACATGAAGCAGATGAAGAGCCTGTCCAAGGAGGAGTTCGTGCACGTCCTGCGACGGCAGAGCACCGGGTTCTCGCGTGGCA
GCTCCAAGTACAGAGGCGTCACCCTGCACAAGTGCGGCCGGTGGGAGGCTCGCATGGGACAGTTCCTCGGCAAGAAGTACATATATCTTGGGCTATTCGA
CAGCGAAGTAGAGGCTGCAAGGGCTTATGATAAGGCCGCGATCAAATGCAATGGTCGAGAAGCTGTGACGAACTTCGAGCCAAGCACGTATGATGGGGAG
CTGCTGACTGAAGTTGCTGATGAAGGTGCTGATGTTGATCTGAACTTGAGCATATCTCAACCAGCTTCTCAGAGCCCGAAAAGGGACAAGAACTCCCTTG
GTCTGCAACTCCACCATGGATCGTTTGAAGGCTACGAATTGAAAAGAACAAAGATTGATGCTCCCTCTGAACTGGCGGGCCGCCCTCATCGATTCCCTCT
TCTGACCGAGCATCCACCAATCTGGACTGCCCAGTCTCACCCCTTCTATTCAAATGATGAGGTTAAGTGATACTAAATTACTAAAATACATGCAATAACT
TTCTCAAATATATTGCTTTAGCTCCCTAAACCTCACAATTGCAAGCATGTTCTGAGCATCACATGGTTCGGATCGAGCTGTCCGGCACCAATTTTGAACG
TAATCTTGACCATCTGAGGAACTGATTGTGCAGCCTGAAGGCGTGCGTTCAAATGTTAGATTTGTGTTACTTTCGTTCATTTTCACTTCTTGTGCCGCTG
TCAATTTTGGCTCTAGTCTTACATCACCGCTTCATTTCCTTGGATTCTATAGCCCGCCTGTTTCCTGTCGATTTCGGAGATATTAATGCGTAGCACAGGT
TTCATATGTAGGATCTTGAGTTTTCTGAGATGGCCCATTCTGGAAACAGTAATAGTCGCCTTAGGAATAGAGATGTGTTTGATTTTTTTCCTTCAAAAGG
AACATCCTAGAGAGGTGACTATTCGACATTAGGTATGCTAATCCATCGGTCAACTATACCTGCCTGCGGCTAAGAACCTTTGAATATGTTTCCTTTTTTT
ATTTATCGTATCTTATAAAAGTAGCAAATACGACTATTATTCAAACCTTGCTGAAAACAAAAGATAGATAGGATGTTGCCATCTTGTAATGTAATCATAG
AATTGGAATCACTGAATTTGCCATTTGTTTCAACTTCAGAGTTTGGTCAAATTGAACTCTCGAACTTTTCAACTCCTAAACTTTAAGGTCAGGCTCACAT
ATGTCATTTTCAAATTTTATTGGACGGAAAGTTGAGCTGGACGTTTCGAGCTCGGCTTTCTATCCAACTGAACTAATGGAAACACTGATTTCATCGCAAC
AAATTTTCAGCAATAGATATTCAATCTCTTCTTGAATTCATCCTTCATCAGTATGGTGAATAAATTGTTCTTGGATTATGCAGAATGCATCAAGAGATCT
TAACAGGAGGCCAGATCAGGGATGCACAGTAGGGGGTGTTCCCAGCTGGGCGTGGAAGGTGACCGCCCCTCCTCCCACGCAGCCATTGCCGCTGTTCTCG
TCGGCGTCATCATCCGCTGCAGCATCATCAGGATTCTCCAATACCGCCACGACAGCTGCCCACACCGCCCCATCGGCCTCCCTCCGGTTCGACCCGCCGC
CGCCGCCGTCCAGCCGCCACCGCTGAACGTTAAGAAGCCACGCTGTAAATTTGCCAGGAAGCCGACATTTTTTCCTCTCTCGGCGTTGCAACTTTTTAGG
TTTTGCGCCGGGGTGGTTTCTTGTAGTGGAGTGGATTCATAATTAACTGTATTTGCATGCTGCCCATGTGAAATGCTTCTCTCTCTTTTTACGCTCTCTG
CACCGGATGTTCTGGGAGTTCTTGTTGCCGGGAATTAACTAAATTCACCGTCTGAGATTGATCTATACATTGTTGTAGAGAATCGAATCATTGTTGAAAT
TAGTTACAGAATTATAGATATCATAAGGCACCCTCTACTTGTGGACAAAGTTCAGGAAGATTATTCCGGTTCTTTTCTTGGCTGTCAATTCGGCTCTTGT
CCTTGGTGTGGAGCCACTTGCCTACTGTCTTGTTCTGTTGCTGTTTGTTCTTGGCTTCTTGCACAGTTACAAATTTGTTGGCTCGAATGGATGGTGAGAC
ATTTGGTGCAA
>Locus_12915_Transcript_29/30_Confidence_0.373_Length_4409
GCTCCTTGAGAAACACCATCCAATTACAAGACTATTTGATGCAACAATCACGCATATGGCTGCGCTAGAACTACTGCTCTGAATTAAGAGGCGTTGAAAT
ATTGTCCGGTCTACATATAGGCACTTGAGGTGTATTGACAGACAGTTCCCCTTAGTTTGCTACAACACATCAGGCGAATTCCACCAAATTAGGATGATGT
TTGTTTCTTAGACACAGGATAGCAATGCCAAAAGTTTTTTATAATTTTCCGCTTCACCGCAGATTTGGCATCTCAAAGGGAGTTGTGGCAAGATTCTCTT
GACCAAGCTTTCTTGCAGATTTGGCAAGAAAGATAAAAAAAATGTTGGTATACTTAAGCATCCTTAATAGAAACTAAACAGGTCTTAGATTGAAATTATC
ATAAATAAACCCTTTTTCACTTTAGCTGTAGTCCGGATGAAATCTTAGGGGCCTTGCTTAGCCTGCCCTGAAGCTTGCCAGTAAGTCGAAGCTGATGCTG
ACACAGCACTGACATAGATCCCAATGAACACAAGTTATTTCGAGAAGATAAAATACCAAGCAAAGCCAGGATTGCATTAAAAGATAGTGTATTTCTACTA
TACGATCTGAAAGCTTAATTTATTATATCAATAATCAAATTGGCAGCAGTGTGGAACAGATATAAAGAAGATGCTTCAAATCACAAATAACTACAGATAA
ATTGAAGGGTGAAGGTTCATTCAACCACCAAACACCCAAAATGTCGAGCTGCATATGCACAGAACCTAAGTACGGTACAGTTCATAGTTTGGGAAAATAG
ACTGCAAATGTGCACAGATAATACAGATTGTTACTCGATGAACAGTAAGCACGGTATTTGAAATCCTTCTAACCATCCATCCAGGCAGTGCCTCGTATAC
TCAAACCTCTTTTTGGAGGAAGCCAAACCATGTGATCCTTTTCAACCCAAATACATATATTCAAGATGAAACTAACAAAAAGACAGTTGATAAAGAAACA
CGAAAGAGAGTAGAAAAAGGGTACCTGTTTCAAGTTCTCATCTTCTGAAAATGATCAGCAGTCAATAGATGTTTAGTCCATTCTCCAGAAAAATGAGTG
CCGCATGAATCCTTTCCGGTTTATAGTGAAACATTTTAGACGATGACCTTTAACATTCTAGAAAACGAAGAGAAACCTACAGTCATCTAATTTCAGCTT
AAACATGTTCCAGCAAGATCATCACCATCCTGACATGTTCCGTTTTGTCCCTAGACCTGAGCACAAGGTATTGCGAGGCTAATTTAGCGGGGCAATTGAT
TTTTTGCCACTATGATACAACTGCAATTACCCGCTGCCACTGACACGTGAGGTCACCTGAGTCACTGAAATGTGAGTCCAGTGGCATTTTTGTTTGCCAC
TCACCCTTCCAGAGTGGCAAAAAGTTAATTGTCCCTAATTTAGCTCAATTCAGTTGTCGGAGCAATTGTGTGTGAAAAGTGTGAACAACAGCATAGGAGA
CAATACAGCAAGTGTAGCGTGCCAATATGTGTCCAGCTTGATAGTTCACTATAATAAAGTTCTTTAATTCAAGTATTTGAATAATATAGAATCTCAACCT
TCAAGTTTAAAAGGTTTTGTATTTGCTATTATAATCGTTGCCACCCCCTCACCCCTCTCAAAAAGAGAGTTACTTTGTAGCGCACTGTAAATCTCTTGAT
TGAAATAGTGGCATGTCATCTTATTTAATTCAGTTATTTCATAAGGTACAATTTGTTTAGCTGACAGCTCAGACATGCTTCTATGCAACATGTTTATACC
AATGCTGTATTTTGGCAATCCTGGATGTGGCATTACTTAACTGTGCTTTCATATTCAATAGTTCTGGCAAAGGGAGGCTGCAAGCTTGAGACAACAACTG
CACAACCTGCAAGAAAGTCATCGGTATGGCTAGAAAACCCTGCAAATTTTACAAATGTACAAGTCAGAAAATTTGCCTAATGTAAAAGGATCAAACTACA
GGATTTGTCAGCATGAGCACAAGTGCCTAACTTCCGAGTTAGATAACTCTGGAGGAAGGAAATCCTTCCTACTGTTTGTTTCAAGAATATACAAAAATTC
AAAACAACATTAGAATAGAACCAAAAGAAGAAACAATATTCACCATGTAGCAATACTCAATTCCTGGTTGTCTTATTTATAAAGGTATAAAACTAGGTTC
TGACGAGACAATTATGAATCAACTACACCATGTTACAGATCATCCATAACTCTTGGTGATGCAAATTAAGGGCTGTAAAGTGACAAGTATACCTCCACCA

ACTAATTTGTGCAGAGTGTATAAAATTGTAAAACAATGGATAAGAATGAATTTAGTATACAAGATAGCATACAATGCCCAAGTTTAAGGAGACATACTGA
TTTACTGACTCCATAGGACAATCTTTTGCTTATGAGGACATCAGTTTTCATAAACAATATCAATGTAAAAGGAGGTAACAGTTTATGAATTTCCTATGCC
AAGCCATTTATAAGTCAAAAAATGTACCTATTTAGCTTTTATGCATTCCAATTAACTTCCAAAGAAGAAGAGTACATACTAATGGAAAACAAATATGATA
TTATCATCTGTTTTGCCCGGTAGTTTCTTGAGTCTCTGATTTGTCCGCACTTCACTCCCAACCAACACAACATTAATACACATGGGCAGAGGAATATCAG
GTTATCAGGTAAACTGAATACAGTTTGATTGACTGCAGTCAGTTGATGGGACAAGATCTTTCTGGATTGAGCGTCAAGGAACTCCAAAATCTAGAAAATC
AGCTAGAAATGAGCCTACGTTGCATCCGGACAAAAAAGGTAAGGATGCTAGCCATATTTCTGTTTTTCGTTACCAGATTGCCTCATCCAAGAATACAACT
GATGATGACGTTTTTTACTTTCAGGACCAACTCTTGATTGATGAAATTCACGAACTGAATCGAAAGGTTCTGGAATATTCTGTTATTACTCTGCAATGAG
AGGTCTCTGAGTGGATTTCAAGAAGCTGACTCTACTGGCTCATTTTCAGGGAAGTCTCATGCAACAAGACAAGATGGAACTATACAAAAAGGTCAACCTA
ATCCGTCAGGAAAACATTGATTTATACAAGAAGGTATTTGATAAATCGTGAATGAAAATTCACAGGATATACACCCCATCCATTATTGACTGAGGTTTAT
TTCTCTACTCAGCTCTACGAGAAAGAGGTGACAAGTGAAGTCAACCGAGATTCATCAACTCTAAACTTTGCAGTTGTCGAGAACGTCAACATTCCTGTTC
ATCTTGAACTTAACACTCCACCGCAAGAAAATGATGTTGAGCAAACTGCACCTCCTAAACTGGGGTAACCCTGCTTTTTTCTGCAAAGGAACTATTGATT
CTGTTCTTAATTCTTTAGTGGCATATTGTTTACTACTAACTGAAAACTTGCAGATTACAACTAAATCCATGAAGGCATGCAGCATGGCACCGCTTATGTT
CATTATTCTCAACGGTACGATTCAAAGCAACCAGAAACACTATTGGTTGTAAAGAGCTTAAACACATAGCAAGACTGACGCGGTATGCATGCCATGAGTG
ATTGATAATGGTAGTGTAAATCTATTTCAAATAAAATGTATTTTATTGAAGAATACAGCAAATGATTTGTATTATAACTATTTGACACATTCTGCAAGTT
TGTTGACCAACGTGAGTACATGATAGCTACAAATCTTATATTGACAAAAAGATATGGTGTGTGGAAAAAATTCAGGTGCTATTTTTTTCTATTTTGAGAA
TTGTATGCATAGCAGAAGAGCACAAATTGCATTCTGGTTTATCCGTGCAGGCATTCCAGAGTATCAGAATGGGGATCAACCTTTCTTCAGCTACATATGT
AAATATACGGATGCTAGTTGCTCACTTAAAATTTTGTAGTGTTCAAAGGAATAACAACAGTAAGACGTTGACGCACCGAAAATAACCAGAAATAACGGAT
TGTACTATGACAAGACACACGACAGATACACTAGCTTAAGTTTGTACAGGAAAATATTTGGCCTATTCTTATGTCACAGTAAATTAAGATTATCACCTTA
ATCACAGAAATTCAAGGGGTAAACGATTATAGCACAAAACGATCCTGGACCCCAAGACCTGGGTCTCTGAACGGCTGAACCTCCAATTATACCTCTATAC
ATTTGGAATGTGTGTAGTCAAACTTTCGCTACTGGTATCTTTACAAATATTTACATTGAACACATCAAAATGATATACATGGATTCCTCTCGATATGTCT
TTGAGGTTTGGGTTCTAAAAATGATATACAAATTATTAGGTTCTAAACTTAAATGATATATACACATCAAAATGATATACAAATTATTGGGTTCTAAACT
TAAATGATG
>comp241103_c0_seq1
GTACCACGACCAGTACTACCCGGCTATTCACCTCAACGGCGCTGCCTCCCTCTACCCCGGCAAGGAAAAGAAGCACTTCCCGTTCTTGACCGACCACGGC
GGGAGCGACGCCGCGGCCTTCGGCTGCCAGCCGTTCGCCATCACCCCTTCCTTAGAGAGCAGCAGTAAGCAGAGCAATGGCAACTGTGCTCTCTCTCTTC
TGTCAGAC
>comp76332_c0_seq52
ACTGCTCTGAATTAAGAGGCGTTGAAATATTGTCCGGTCTACATATAGGCACTTGAGGTGTATTGACAGACAGTTCCCCTTAGTTTGCTACAACACATCA
GGCGAATTCCACCAAATTAGGATGATGTTTGTTTCTTAGACACAGGATAGCAATGCCAAAAGTCTTTTATAATTTTCCGCTTCACCGCAGATTTGGCATC
TCAAAGGGAGTTGTGGCAAGATTCTCTTGACCAAGCTTTCTTGCAGATTTGGCAAGAAAGATAAAAAAAATGTTGGTATACTTAAGCATCCTTAATAGAA
ACTAAACAGGTCTTAGATTGAAATTATCATGAATAAACCCTTTTTCACTTTAGCTGTAGTCCGGATGAAATCTTAGGGGCCTTGCTTAGCCTGCCCTGAA
GCTTGCCAGTAAGTCGAAGCTGATGCTGACACAGCACTGTTTCAAGTTCTCATCTTCTGAAAATGATCAGCAGTCAATAGATGTTTTAGTCCATTCTCCA
GAAAAATGAGTGCCGCATGAATCCTTTCCGGTTTATAGTGAAACATTTTAGACGATGACCTTTAACATTCTAGAAAACGAAGAGAAAACCTACAGTCATC
TAATTTCAGCTTAAACATGTTCCAGCAAGATCATCACCATCCTGACATGTTCCGTTTTGTCCCTAGACCCGAGCACAAGGTATTGCGAGGCTAATTTAGC
TCAATTCAGTTGTCAGAGCAATTGTGTGTGAAAAGTGTGAACAACAGCATAGGAGACAATACAGCAAGTGTAGCGTGCCAATATGTGTCCAGCTTGATAG
TTCACTATAATAAAGTTCTTTAATTCAAGTATTTGAATAATATAGAATCTCAACCTTCAAGTTTAAAAGGTTTTGTATTTGCTATTATAATCGTTGCCAC
CCCCTCACCCCTCTCAAAAAGAGAGTTACTTTGTAGCGCACTGTAAATCTCTTGATTGAAATAGTGGCATGTCATCTTATTTAATTCAGTTATTTCATAA
GGTACAATTTGTTTAGCTGACAGCTCAGACATGCTTCTATGCAACATGTTTATACCAATGCTGTATTTTGGCAATCCTGGATGTGGCATTACTTAACTGT
GCTTTCATATTCAATAGTTCTGGCAAAGGGAGGCTGCAAGCTTGAGACAACAACTGCACAACCTGCAAGAAAGTCATCGGTATGGCTAGAAAACCCTGCA
AATTTTACAAATGTACAAGTCAGAAAATTTGCCTAATGTAAAAGGATCAAACTACAGGATTTGTCAGCATGAGCACAAGTGCCTAACTTCCGAGTTAGAT
AACTCTGGAGGAAGGAAATCCTTCCTACTGTTTGTTTCAAGAATATACAAAAATTCAAAACAACATTAGAATAGAACCAAAAGAAGAAACAATATTCACC
ATGTAGCAATACTCAATTCCTGGTTGTCTTATTTATAAAGGTATAAAACTAGGTTCTGACGAGACAATTATGAATCAACTACACCATGTTACAGATCATC
CATAACTCTTGGTGATGCAAATTAAGGGCTGTAAAGTGACAAGTATACCTCCACCAACTAATTTGTGCAGAGTGTATAAAATTGTAAAACAATGGATAAG
AATGAATTTAGTATACAAGATAGCATACAATGCCCAAGTTTAAGGAGACATACTGATTTACTGACTCCATAGGACAATCTTTTGCTTATGAGGACATCAG
TTTTCATAAACAATATCAATGTAAAAGGAGGTAACAGTTTATGAATTTCCTATGCCAAGCCATTTATAAGTCAAAAAATGTACCTATTTAGCTTTTATGC
ATTCCAATTAACTTCCAAAGAAGAAGAGTACATACTAATGGAAAACAAATATGATATTATCATCTGTTTTGCCCGGTAGTTTCTTGAGTCTCTGATTTGT
CCGCACTTCACTCCCAACCAACACAACATTAATACACATGGGCAGAGGAATATCAGGTTATCAGGTAAACTGAATACAGTTTGATTGACTGCAGTCAGTT
GATGGGACAAGATCTTTCTGGATTGAGCGTCAAGGAACTCCAAAATCTAGAAAATCAGCTAGAAATGAGCCTACGTTGCATCCGGACAAAAAAGGTAAGG
ATGCTAGCCATATTTCTGTTTTTCGTTACCAGATTGCCTCATCCAAGAATACAACTGATGATGACGTTTTTTACTTTCAGGACCAACTCTTGATTGATGA
AATTCACGAACTGAATCGAAAGGTTCTGGAATATTCTGTTATTACTCTGCAATGAGAGGTCTCTGAGTGGATTTCAAGAAGCTGACTCTACTGGCTCATT
TTCAGGGAAGTCTCATGCAACAAGACAAGATGGAACTATACAAAAAGGTCAACCTAATCCGTCAGGAAAACATTGATTTATACAAGAAGGTATTTGATAA
ATCGTGAATGAAAATTCACAGGATATACACCCCATCCATTATTGACTGAGGTTTATTTCTCTACTCAGCTCTACGAGAAAGAGGTGACAAGTGAAGTCAA
CCGAGATTCATCAACTCTAAACTTTGCAGTTGTCGAGAACGTCAACATTCCTGTTCATCTTGAACTTAACACTCCACCGCAAGAAAATGATGTTGAGCAA
ACTGCACCTCCTAAACTGGGGTAACCCTGCTTTTTTCTGCAAAGGAAGATTTTCTTGCAAGTTATGCAGTTGTTGTCTCTAGCTTGCCGCCTCCACTTGC
AGTAACTGAGACGCCGGCGCCGAGCTTGAGCTTGAAACCTAGACTACGGGGACAGGGTAGACAGGAGACGCGAAACCCCAAACCAGCGCAAAACGACGCG
CAGGGAGGGAGTGTCACTGAAGGAGCGACAGGGAAAGCAGCGGCACGCCGGGGGAAATTCCGATCTGAGAGAACGGATCAGCCATGCATGACAGGAGCTG
CGGAGGCAGCAGGGGGGGGGGGCTCCCGCTGCCACGGGACAGTAGGGCACGCCAGATCGGTGAAATCCAGGCGAAGGACGAGGCGTGGGCGAGATTTACA
CGACGGCGCGATAGGATCTAGCCCGGATTTGGATTCGACCGACGAGGGCGGAAGGAACGGCAGGGTGGTGCTCTTGTAGAGAGAGAAGGAGTCGGAGGGG
GCGGAGAGAGAGAAAGGGGGCAGGTGGGGTTTCAGGTAGTAAAATATGGAGCGG
>Locus_4749_Transcript_53/72_Confidence_0.495_Length_4332
GGTACATGTCGCGGAGCTACGCGCCGGCGTTCCACAGCTTCGCGTGGGCGCAGGCCGTGCAGAAGAAGCCGCTCGTGCCAAGGCCGGCCGCTGCTGACGA
GGACGAGGTGGAGCACGCCGTGGACGCGTCGGACGAGGAGAAGGAGGAGGGGCGAGATTGAGGAGGGGGAGACCGTGGAGACCTCCTCCTCGTCGCCCCCG
CGCGCGCAGCCTGAGACTATCGATTTGGACTCTGACGCGCCGGAGAAGTCTGAGTCTGTGGCTGCGGAGGGCAGTGGTAATGCTGCCCCTGCCACCGGCT
TCGCCGAGGAGGAGGAGGAGGAGGTGGATTTTGACCAGCGCGTGGGGAGTATACTCGAGGAGCTCGAGATGGTCTCCATTGAGGAAGCTGAGAAGTCGTT
TGAGGGTGCGTGTGCACGCCTGCGCACTTGCTTTGAGAGCCTGAAGCCGCTGTTCCCGGAGAACGGAAGCCCGATGCCGATGCTTGAACCTCTTGTGCAG
CAGGCGTTTGTCGGAATTGACACCCTCACCACTGTGGCGAATTCGTATAATTTGCCGAGGAAGGAGCAGAACAAGACCATGCTCTTGAAGATGTTGTTCC
ACATAAAGAACCGATATTCAGACATGCTGACACCTGACCAACGAGATGAGCTGGACACCCGTGTGAGGCAGTTAGTTTTTGAGGAAAAAGACGATATCAG
CGACCCAAGTGCCAATTGTGGCATGAAAGCAATGAATGTTTCGGCTCCATCTGGGCAGGTTTCTTCAGGGAGACTACCGTTTGAGTCAGGAGCAGCAAAT
CCATTTAGTGGCTCTAGCTTGCCGAGGTTGGAGATACCTGCAAAAAAGATTAGTCCCTTGTTGGATCTTCATGCAGATTATGATGAGAACAGCTTGCCTT
CGCCCACTCGGGATAGTGCGCCATCTTTTCCTGTGCCGAGGCCCATTGGGTTTGGAGCATTTCCATTGGCACATGAGAAACTATCTTTCCCAGAAAGAGC
TGAGCCTCCAAAGAATTCGTTGTATCCATCCTTAAATGATCCTCTGAAGGCTGTTTCCTCTTACCAGCAGAAATATGGGCAGAAATCTTCCTTTCCAAGT
GATGATCTGCCAAGTCCAACTCCTTCGGGCGATGAGGATAAATCTGCAGACAAAGGTGGTGACCTATTTGGTGAGGTTTCCAGCTTCTCAATTCCAAAGA
AGACTGCATTGCCAAGTACTAGTCAGATGCCTGCTTCTCAACCAAGCACAGTTGGCAGCAGCAATGATAGTTATACAGGTGGTGCGCCTGGTTATGCTAA
ACAAATTGAACAGCCGGCTACAGGACCAAATCATGGACTAAAGGCTACATCTAAAAGCAGGGATCCAAGGCTCAGGTTTTTGAACCGTGATTCTGCTGGT
GCTACAGACGCGAATCGGTGTGCAAATTTTGCAGAACCGAAAGAAGAAAACCTGGGTGATGGAGTATCAGTTAATAACCGTAAGCAAAAGGCAGTTGATG
AACCAAAGGTGGATGAAAACTTGTTAAAAAGGCTTAGGACTGGTAGTGGGAACCAAAGAGACATGCTGGTGCCATCAGGGAATTCTAATTCTAATCTTAG
AACGGGTAATCAGCTGATTAACAGCATAAGTGCTACCACAAATAGTAGCGGAATAAATACCAAAATTTTGCAACCCCCTCAAGCTACTGCCCCACAAATT
AGTGCAGCACCTGCTGTTTCTTTGCCTACTGTGTTAAAGGATATTGCTTTGAACCCAGCAATGATCATGCATTGGATTCAAATGGAACAGCAAAAGATGT

CGGCATCAGAACCTCAGCTGAATGTGGTTGCTCCGGGTGGCATGTCTAGTGGCATGACCAGTACTGTAACTGCAGCAACGGTTTTACCACCTGGCTATAC
TCCAAAGGCCACAGAAGTTCCACAAATTCCATCTGTTAGGCCACATGTTCCAGTGCAGACACCTCCTTTGAACCAACAAAATGATGCTGCTGGAATTATA
CGTATGAAGCCCCGTGATCCCCGGCGGATCCTCCACAGTAACATAGCACAGAAGAATGATACAGTGGGCTCGGAGCAAGTGAAAACTAATGGAGCAACCC
AACCAGACACTCAGGGCGGCAAAGACCATTTGACCAACCGTGAACAACAGCCAGAGCAACATCAGACTAGTTCATTGCCTTCTCAACCAGCCTTGCTGTC
AAACATTACGCGACCATCCACCATTAACTCAAAACCTGTTGATGCTGTCTCAAACTCACAGTTAGCTGCTACAGCACTTATAGCTCCAACACAGCAAGCT
TTAGGCAGCAATAGGGTAGATCCAAGACTAGCGGTTGGACAGAATGGTGCTAATGCTGAAGCAGCAACAAATGCTGCTCCTGCACCAGCACTTGAAGCTC
TGCAGCCAGTTGATCCATGGGGTGATGTTGACCATCTCCTTGATGGTTATGATGACCAGCAGAAGGCTCTCATACAAAAGGAAAGGGCAAGACGGATCTC
GGAACAGCAAAATTTGTTTTCAGCGCGGAAGCTATGCTTAGTCCTTGATTTGGACCACACACTACTCAATTCTGCAAAGTTTGGAGAAGTGAATCCTGTT
CATGAAGAGATTTTGCGGAAGAAAGAGGAACAAGACAGGTCTTTGCTAGAACGGCATCTGTATCGTTTCCAGCATATGGGAATGTGGACCAAGCTGAGAC
CAGGAATATGGAACTTCCTTAAGAAGGCGAGTAAGCTTTTTCAGTTGCATCTGTACACAATGGGGAACAAGCTGTATGCTACTGAGATGGCCAAGGTTCT
CGATCCTACTGGGAAATTGTTTGCAGGGCGAGTCATCTCAAGAGGTGATGATGGTGATCCTTTAGACAGCAGCGATGATCGAGTCAAAAGTAAAGATCTTGAT
GGAGTATTGGGAATGGAATCTGCAGTAGTGATAATAGATGACTCTATAAGAGTCTGGCCTCACAACAAGCACAATTTGATAGTTGTAGAGAGATACACCT
ATTTCCCCTGCAGCAGACGTCAATTTTGCCTTCCTGGACCATCACTTCTCGAAATTGATCGAGATGAGAGGCCGGAGGATGGCACACTTGCTTCTTCACT
GGCAGTTATTGAGCGAATTCACCAAAACTTCTTTGCTCACCCTAACCTCAATGAGGCTGATGTGCGGAGCATACTAGCATCTGAGCAGCGGAGGATCCTC
GCGGGTTGTCGTATTGTCTTTAGCCGGATTTTCCCGGTTGGAGAGGTCAACCCCCACTTGCATCCGCTGTGGGAGATTGCAGAGCAGTTTGGCGCTGTTT
GCACAAACCAGATTGACGATCGGGTTACTCATGTTGTGGCCAACTCCCTTGGGACGGACAAGGTGAACTGGGCACTATCCAATAACAGATTTGTGGTTCA
TCCAGGATGGGTGGAGGCTTCAGCTCTCTTGTACCGGCGTGCCAATGAGCTTGACTTTGCAGTCAAATAACGCATCCCTGAGCCCCAATTCTCCATCTAG
GGCCCCGAGATTCTTCCCGGGATGAGATCCGGTCATGAAAAACTAACATGAAAAGAAACCAAACAACATTCTAAAACCGGACCCCATTAAGAAGCAGACC
ATACCACCTCTAACAGAATCAGGCCTAAACATGAAAGATAACGAAAATACTTAGTGATAGCCTGAAAGAGGAGACGGTGGTTGATGCCATTGCTGAGGTG
ATGAGTACAACTTGATTCAGAAGTGTTTTAGAAAGAACCATCAAAGAAAGATGAGCTAAAAGCGAAGATGACGAAGGCTCATCACCTCCCGGTTGTAACA
TGTAGAATTGATGCATTTCTTATAAGAGTCGTAGGTCCAGATGTTGCCTCTGGCTGCAAGCCTGCTGCTGGCCACAGAAACAGGGTTGGTAGCATGCGCT
GGTAACCCATCAAAGTGGCAACGGCAGCAGCAGTCTTGGGGATGAAGTCATGGACAAGGCAGGCATATAAAGTTTGATGTGTGTATATTTGTGGCTTGGC
TGTAAGTGTGTAGTTGGTTCTCGCTGTGACCTGAAACTGAAAGCCACATGGAAAGGGACACCTGGGGCTGCTCTGCTGCTTGACGAAAGAGTGTTCAGC
TACCATGGAGGATATAACAGGTCCAGAAACGGGGGATGGAGAACTGTGGTCGAAAAACAGGACCAGTGGAGAATTGGTGATGAGCCACCTCGTGTATGAA
ACCAGAGTAAACAGTCAATGGTGGCAGAGAAGAATGCACAAGGTATGATGCGGATGGTTGCGGGCTATGGGGAACCAAGGGTATGACCGGGTTTTTACTG
CAACTGTAAGCGATTATTGGGGGGGGGGGGGG
>Locus_18892_Transcript_21/56_Confidence_0.294_Length_2394
ATCTGGGATTGTGGGAAGCAAGTCTACTTAGGTGAGTAACTGAGTTGAGTGAGTTCTATACGTTCCAGCTCGAGCTTCCATTAACTGCTGATCAGTTTCC
GTCCGTTTGATCTGGTTTACGATGTGCTGATGCTGTTCTATTCAGGTGGTTTCGACACTGCTCACGCGGCTGCGAGGGCTTATGATCGTGCAGCGATCAA
GTTCCGAGGGCTCGACGCGGACATCAACTTCACTTTGAGCGACTATGAGGATGACTTGAAGCAGATGAGCAATTGGACAAAGGAAGAGTTCGTGCACATA
CTCCGCCGCCAGAGCACGGGGTTCGCGAGGGGGAGCTCCAAGTACCGCGGCGTGACGCTGCACAAGTGCGGCCGCTGGGAGGCGAGGATGGGTCAACTTC
TTGGCAAGAAGTACATCTATCTTGGACTCTTTGACAGCGAAGTTGAAGCTGCAAGAGCATATGACAGGGCAGCCCTTCGCTTCAATGGGGGGGAAGCTGT
TACTAATTTTGGGCCTAGCTCCTACAATGGAGGAGATGCTCTACCCGACACCGAAAATGAGGCAACTGTTGATGGTGACGCGGTTGATTTGGATTTGCGG
ATTTCACAACCTAATGTGCACGACACTAAAAGGGACAATACCTTAGCTGGAGGCCAGCTAACATGCGACTCCCTTGAATCTTCAAACACGATGGCCTCTC
AGGTAAACTGACTTCAAGACTACACAGCAGGGAAAAATGCTCGGTTTCTCTATGACTTTCTACTACTTGCAGTAACTAAAAAAGTTTCCCAGAAAAAATAA
AATCTTTAAGATGGAAGCAACTAGGTTGAATTGTCTTGTATCTTTGCGTGGAATCCAACCTTTGATAGTCCCTACGCTATAAAGATTCATAGGAGTATTT
GATTGGTGTGTCGTCGGTTGAGATATTAACTAATTGGTCCAGGTCCTATAACCTTTATTGAACTTACGATATTTGCACCTATCTGTTGCTCCTGTAGCCA
ATGAGCTCATCGTACCCGTGGCCTGTGTATCACCAAAGCACGACAGCAGTACCACCTCACCATCAACGCTTGTACTCATCTGCTTGTCCTGGCTTCTTTT
CGAACCTCCAGGTATTACCAAGCTAAAGCCTATTTTTTATGTGCAAATTGCTACTCCTACGTGATCAATTAGTTCAAACTGTTATTCGAATCCAGGATTG
TTTACTGAATATGTCCTGTCTTCAGAAAAAATAACACAAAAAGGAACATAATATGTCCTGTCTAATTGTGGACTGATACTAGCCAATTAAACAGTTTCCC
ATATCCATTTCCTGTGTTTATTGTTTTGATTTGCTGGCATGGTTAAACTATAGTAGCTTGATTCGATAAAAACACTAAGCAGGCTTTGATATTTTGAATG
AAATGTCGTTCTTGGAAATACAGCCTTTTCCTTGGTATATCAAGGACAGTTGTCCATCTGCACTCTGCACCCTCAGCTTGTGTAACTGTTTGCCTTGTGA
ACTGATTGATGTGTCAATCAGGCAGTTCTGCCAGTTTGCATCTTTTGAAATAGTGCAGTCTTCCAGTCTAACAGCTCCATGACATAACTTGGGATGCATG
CTGCAGCTTTCTTACTGAGGATGCTCTAGATTTACAGAAATTCTCGCAGTTAGCAGAGGGCTATCAAATCTGATTTGCTTAGCTTCCTGCAGACAATAAG
ATAAGAACTCTAGAATCCTGAAGAAATCTTACTTGTACATGTTTACCAAAGTATCCTTACTCCTTAGTTGAGCCAATTTTGGTGAAGAACCGATAGCTCA
TTACTCTGTTTCCTGGGAATGATGTGGGCCTAAACCATTCTGTGGTCAAAGTTTAACAAAGCCCATGCTTGTGCTGTTGCAACTGCAGGAAAGGCCAATG
GAGCGAAGGCCTGAGTTGGGTACCCAGTCATTCCCCCACCTGGGGATGGCAAATGCAGGGCTCCCCTCACATGCCGTTGCATCACTCTGCAGCATCATCAG
GATTCTCTACCGCCGTCGGCGCAAACGCCGGCGTCTCGTTGCCTTCTCACCCGCCGGTGCCGTTCCCGGACCACCAGTTCTACTTCCCACCGACGGCATG
AGCTTGTTGGTCTGGTTCAACTCTTGGGACGGCAGCTGATCTTATGTGTGAACATTTCGCAGCTTGCCGGTGACCGTTGTTAATTAATCGGGGAGAGAGA
GCCAGAAGACCCAGTATGGCTATCTCTCTGTGGCCCGTGCCTTGTTATGATCTTAGTTATTGCTACTACACCGTGGATTCATCATGGTTTATGAGATTCT
TTACTCAAGTTTCAGCTTAAATGTCCAAATACATGAATGCATCATACACAGGATCCGTCATTTTCTAAAAAAATACAGGATCTGTGTGAAGTGT
>Locus_12915_Transcript_12/30_Confidence_0.451_Length_4164
GCTCCTTGAGAAACACCATCCAATTACAAGACTATTTGATGCAACAATCACGCATATGGCTGCGCTAGAACTACTGCTCTGAATTAAGAGGCGTTGAAAT
ATTGTCCGGTCTACATATAGGCACTTGAGGTGTATTGACAGACAGTTCCCCTTAGTTTGCTACAACACATCAGGCGAATTCCACCAAATTAGGATGATGT
TTGTTTCTTAGACACAGGATAGCAATGCCAAAAGTTTTTTATAATTTTCCGCTTCACCGCAGATTTGGCATCTCAAAGGGAGTTGTGGCAAGATTCTCTT
GACCAAGCTTTCTTGCAGATTTGGCAAGAAAGATAAAAAAAATGTTGGTATACTTAAGCATCCTTAATAGAAACTAAACAGGTCTTAGATTGAAATTATC
ATAAATAAACCCTTTTTCACTTTAGCTGTAGTCCGGATGAAATCTTAGGGGCCTTGCTTAGCCTGCCCTGAAGCTTGCCAGTAAGTCGAAGCTGATGCTG
ACACAGCACTGACATAGATCCCAATGAACACAAGTTATTTCGAGAAGATAAAATACCAAGCAAAGCCAGGATTGCATTAAAAGATAGTGTATTTCTACTA
TACGATCTGAAAGCTTAATTTATTATATCAATAATCAAATTGGCAGCAGTGTGGAACAGATATAAAGAAGATGCTTCAAATCACAAATAACTACAGATAA
ATTGAAGGGTGAAGGTTCATTCAACCACCAAACACCCAAAATGTCGAGCTGCATATGCACAGAACCTAAGTACGGTACAGTTCATAGTTTGGGAAAATAG
ACTGCAAATGTGCACAGATAATACAGATTGTTACTCGATGAACAGTAAGCACGGTATTTGAAATCCTTCTAACCATCCATCCAGGCAGTGCCTCGTATAC
TCAAACCTCTTTTTGGAGGAAGCCAAACCATGTGATCCTTTTCAACCCAAATACATATATTCAAGATGAAACTAACAAAAGACAGTTGATAAAGAAACA
CGAAAGAGAGTAGAAAAAGGGTACCTGTTTCAAGTTCTCATCTTCTGAAAATGATCAGCAGTCAATAGATGTTTTAGTCCATTCTCCAGAAAAAATGAGTG
CCGCATGAATCCTTTCCGGTTTATAGTGAAACATTTTAGACGATGACCTTTAACATTCTAGAAAACGAAGAGAAAACCTACAGTCATCTAATTTCAGCTT
AAACATGTTCCAGCAAGATCATCACCATCCTGACATGTTCCGTTTTGTCCCTAGACCCGAGCACAAGGTATTGCGAGGCTAATTTAGCTCAATTCAGTTG
TCAGAGCAATTGTGTGTGAAAAGTGTGAACAACAGCATAGGAGACAATACAGCAAGTGTAGCGTGCCAATATGTGTCCAGCTTGATAGTTCACTATAATA
AAGTTCTTTAATTCAAGTATTTGAATAATATAGAATCTCAACCTTCAAGTTTAAAAGGTTTTGTATTTGCTATTATAATCGTTGCCACCCCCTCACCCCT
CTCAAAAAGAGAGTTACTTTGTAGCGCACTGTAAATCTCTTGATTGAAATAGTGGCATGTCATCTTATTTAATTCAGTTATTTCATAAGGTACAATTTGT
TTAGCTGACAGCTCAGACATGCTTCTATGCAACATGTTTATACCAATGCTGTATTTTGGCAATCCTGGATGTGGCATTACTTAACTGTGCTTTCATATTC
AATAGTTCTGGCAAAGGGAGGCTGCAAGCTTGAGACAACAACTGCACAACCTGCAAGAAAGTCATCGGTATGGCTAGAAAACCCTGCAAATTTTACAAAT
GTACAAGTCAGAAAATTTGCCTAATGTAAAAGGATCAAACTACAGGATTTGTCAGCATGAGCACAAGTGCCTAACTTCCGAGTTAGATAACTCTGGAGGA
AGGAAATCCTTCCTACTGTTTGTTTCAAGAATATACAAAAATTCAAAACAACATTAGAATAGAACCAAAAGAAGAAACAATATTCACCATGTAGCAATAC
TCAATTCCTGGTTGTCTTATTTATAAAGGTATAAAACTAGGTTCTGACGAGACAATTATGAATCAACTACACCATGTTACAGATCATCCATAACTCTTGG
TGATGCAAATTAAGGGCTGTAAAGTGACAAGTATACCTCCACCAACTAATTTGTGCAGAGTGTATAAAATTGTAAAACAATGGATAAGAATGAATTTAGT
ATACAAGATAGCATACAATGCCCAAGTTTAAGGAGACATACTGATTTACTGACTCCATAGGACAATCTTTTGCTTATGAGGACATCAGTTTTCATAAACA
ATATCAATGTAAAAGGAGGTAACAGTTTATGAATTTCCTATGCCAAGCCATTTATAAGTCAAAAAATGTACCTATTTAGCTTTTATGCATTCCAATTAAC
TTCCAAAGAAGAAGAGTACATACTAATGGAAAACAAATATGATATTATCATCTGTTTTGCCCGGTAGTTTCTTGAGTCTCTGATTTGTCCGCACTTCACT
CCCAACCAACACAACATTAATACACATGGGCAGAGGAATATCAGGTTATCAGGTAAACTGAATACAGTTTGATTGACTGCAGTCAGTTGATGGGACAAGA

TCTTTCTGGATTGAGCGTCAAGGAACTCCAAAATCTAGAAAATCAGCTAGAAATGAGCCTACGTTGCATCCGGACAAAAAAGGTAAGGATGCTAGCCATA
TTTCTGTTTTTCGTTACCAGATTGCCTCATCCAAGAATACAACTGATGATGACGTTTTTTACTTTCAGGACCAACTCTTGATTGATGAAATTCACGAACT
GAATCGAAAGGTTCTGGAATATTCTGTTATTACTCTGCAATGAGAGGTCTCTGAGTGGATTTCAAGAAGCTGACTCTACTGGCTCATTTTCAGGGAAGTC
TCATGCAACAAGACAAGATGGAACTATACAAAAAGGTCAACCTAATCCGTCAGGAAAACATTGATTTATACAAGAAGGTATTTGATAAATCGTGAATGAA
AATTCACAGGATATACACCCCATCCATTATTGACTGAGGTTTATTTCTCTACTCAGCTCTACGAGAAAGAGGTGACAAGTGAAGTCAACCGAGATTCATC
AACTCTAAACTTTGCAGTTGTCGAGAACGTCAACATTCCTGTTCATCTTGAACTTAACACTCCACCGCAAGAAAATGATGTTGAGCAAACTGCACCTCCT
AAACTGGGATTACAACTAAATCCATGAAGGCATGCAGCATGGCACCGCTTATGTTCATTATTCTCAACGGTACGATTCAAAGCAACCAGAAACACTATTG
GTTGTAAAGAGCTTAAACACATAGCAAGACTGACGCGGTATGCATGCCATGAGTGATTGATAATGGTAGTGTAAATCTATTTCAAATAAAATGTATTTTA
TTGAAGAATACAGCAAATGATTTGTATTATAACTATTTGACACATTCTGCAAGTTTGTTGACCAACGTGAGTACATGATAGCTACAAATCTTATATTGAC
AAAAAGATATGGTGTGTGGAAAAAATTCAGGTGCTATTTTTTTCTATTTTGAGAATTGTATGCATAGCAGAAGAGCACAAATTGCATTCTGGTTTATCCG
TGCAGGCATTCCAGAGTATCAGAATGGGGATCAACCTTTCTTCAGCTACATATGTAAATATACGGATGCTAGTTGCTCACTTAAAATTTTGTAGTGTTCA
AAGGAATAACAACAGTAAGACGTTGACGCACCGAAAATAACCAGAAATAACGGATTGTACTATGACAAGACACACGACAGATACACTAGCTTAAGTTTGT
ACAGGAAAATATTTGGCCTATTCTTATGTCACAGTAAATTAAGATTATCACCTTAATCACAGAAATTCAAGGGGTAAACGATTATAGCACAAAACGATCC
TGGACCCCAAGACCTGGGTCTCTGAACGGCTGAACCTCCAATTATACCTCTATACATTTGGAATGTGTGTAGTCAAACTTTCGCTACTGGTATCTTTACA
AATATTTACATTGAACACATCAAAATGATATACATGGATTCCTCTCGATATGTCTTTGAGGTTTGGGTTCTAAAAATGATATACAAATTATTAGGTTCTA
AACTTAAATGATATATACACATCAAAATGATATACAAATTATTGGGTTCTAAACTTAAATGATG
>Locus_12915_Transcript_21/30_Confidence_0.706_Length_4325
GCTCCTTGAGAAACACCATCCAATTACAAGACTATTTGATGCAACAATCACGCATATGGCTGCGCTAGAACTACTGCTCTGAATTAAGAGGCGTTGAAAT
ATTGTCCGGTCTACATATAGGCACTTGAGGTGTATTGACAGACAGTTCCCCTTAGTTTGCTACAACACATCAGGCGAATTCCACCAAATTAGGATGATGT
TTGTTTCTTAGACACAGGATAGCAATGCCAAAAGTTTTTTATAATTTTCCGCTTCACCGCAGATTTGGCATCTCAAAGGGAGTTGTGGCAAGATTCTCTT
GACCAAGCTTTCTTGCAGATTTGGCAAGAAAGATAAAAAAAATGTTGGTATACTTAAGCATCCTTAATAGAAACTAAACAGGTCTTAGATTGAAATTATC
ATAAATAAACCCTTTTTCACTTTAGCTGTAGTCCGGATGAAATCTTAGGGGCCTTGCTTAGCCTGCCCTGAAGCTTGCCAGTAAGTCGAAGCTGATGCTG
ACACAGCACTGACATAGATCCCAATGAACACAAGTTATTTCGAGAAGATAAAATACCAAGCAAAGCCAGGATTGCATTAAAAGATAGTGTATTTCTACTA
TACGATCTGAAAGCTTAATTTATTATATCAATAATCAAATTGGCAGCAGTGTGGAACAGATATAAAGAAGATGCTTCAAATCACAAATAACTACAGATAA
ATTGAAGGGTGAAGGTTCATTCAACCACCAAACACCCAAAATGTCGAGCTGCATATGCACAGAACCTAAGTACGGTACAGTTCATAGTTTGGGAAAATAG
ACTGCAAATGTGCACAGATAATACAGATTGTTACTCGATGAACAGTAAGCACGGTATTTGAAATCCTTCTAACCATCCATCCAGGCAGTGCCTCGTATAC
TCAAACCTCTTTTTGGAGGAAGCCAAACCATGTGATCCTTTTCAACCCAAATACATATATTCAAGATGAAACTAACAAAAAGACAGTTGATAAAGAAACA
CGAAAGAGAGTAGAAAAAGGGTACCTGTTTCAAGTTCTCATCTTCTGAAAATGATCAGCAGTCAATAGATGTTTTAGTCCATTCTCCAGAAAAATGAGTG
CCGCATGAATCCTTTCCGGTTTATAGTGAAACATTTTAGACGATGACCTTTAACATTCTAGAAAACGAAGAGAAAACCTACAGTCATCTAATTTCAGCTT
AAACATGTTCCAGCAAGATCATCACCATCCTGACATGTTCCGTTTTGTCCCTAGACCTGAGCACAAGGTATTGCGAGGCTAATTTAGCGGGGCAATTGAT
TTTTTGCCACTATGATACAACTGCAATTACCCGCTGTCACTGACACGTGGGGTCATCTGAGTCACTGAAATGTGGGCCCAGTGGCATTTTGTTTGCCACT
CACCCTTCCAGAGTGGCAAAAAGTTAATTGTCCCTAATTTAGCTCAATTCAGTTGTCGGAGCAATTGTGTGTGAAAAGTGTGAACAACAGCATAGGAGAC
AATACAGCAAGTGTAGCGTGCCAATATGTGTCCAGCTTGATAGTTCACTATAATAAAGTTCTTTAATTCAAGTATTTGAATAATATAGAATCTCAACCTT
CAAGTTTAAAAGGTTTTGTATTTGCTATTATAATCGTTGCCACCCCCTCACCCCTCTCAAAAAGAGAGTTACTTTGTAGCGCACTGTAAATCTCTTGATT
GAAATAGTGGCATGTCATCTTATTTAATTCAGTTATTTCATAAGGTACAATTTGTTTAGCTGACAGCTCAGACATGCTTCTATGCAACATGTTTATACCA
ATGCTGTATTTTGGCAATCCTGGATGTGGCATTACTTAACTGTGCTTTCATATTCAATAGTTCTGGCAAAGGGAGGCTGCAAGCTTGAGACAACAACTGC
ACAACCTGCAAGAAAGTCATCGGTATGGCTAGAAAACCCTGCAAATTTTACAAATGTACAAGTCAGAAAATTTGCCTAATGTAAAAGGATCAAACTACAG
GATTTGTCAGCATGAGCACAAGTGCCTAACTTCCGAGTTAGATAACTCTGGAGGAAGGAAATCCTTCCTACTGTTTGTTTCAAGAATATACAAAAATTCA
AAACAACATTAGAATAGAACCAAAAGAAGAAACAATATTCACCATGTAGCAATACTCAATTCCTGGTTGTCTTATTTATAAAGGTATAAAACTAGGTTCT
GACGAGACAATTATGAATCAACTACACCATGTTACAGATCATCCATAACTCTTGGTGATGCAAATTAAGGGCTGTAAAGTGACAAGTATACCTCCACCAA
CTAATTTGTGCAGAGTGTATAAAATTGTAAAACAATGGATAAGAATGAATTTAGTATACAAGATAGCATACAATGCCCAAGTTTAAGGAGACATACTGAT
TTACTGACTCCATAGGACAATCTTTTGCTTATGAGGACATCAGTTTTCATAAACAATATCAATGTAAAAGGAGGTAACAGTTTATGAATTTCCTATGCCA
AGCCATTTATAAGTCAAAAAATGTACCTATTTAGCTTTTATGCATTCCAATTAACTTCCAAAGAAGAAGAGTACATACTAATGGAAAACAAATATGATAT
TATCATCTGTTTTGCCCGGTAGTTTCTTGAGTCTCTGATTTGTCCGCACTTCACTCCCAACCAACACAACATTAATACACATGGGCAGAGGAATATCAGG
TTATCAGGTAAACTGAATACAGTTTGATTGACTGCAGTCAGTTGATGGGACAAGATCTTTCTGGATTGAGCGTCAAGGAACTCCAAAATCTAGAAAATCA
GCTAGAAATGAGCCTACGTTGCATCCGGACAAAAAAGGTAAGGATGCTAGCCATATTTCTGTTTTTCGTTACCAGATTGCCTCATCCAAGAATACAACTG
ATGATGACGTTTTTTACTTTCAGGACCAACTCTTGATTGATGAAATTCACGAACTGAATCGAAAGGGAAGTCTCATGCAACAAGACAAGATGGAACTATA
CAAAAAGGTCAACCTAATCCGTCAGGAAAACATTGATTTATACAAGAAGGTATTTGATAAATCGTGAATGAAAATTCACAGGATATACACCCCATCCATT
ATTGACTGAGGTTTATTTCTCTACTCAGCTCTACGAGAAAGAGGTGACAAGTGAAGTCAACCGAGATTCATCAACTCTAAACTTTGCAGTTGTCGAGAAC
GTCAACATTCCTGTTCATCTTGAACTTAACACTCCACCGCAAGAAAATGATGTTGAGCAAACTGCACCTCCTAAACTGGGGTAACCCTGCTTTTTTCTGC
AAAGGAACTATTGATTCTGTTCTTAATTCTTTAGTGGCATATTGTTTACTACTAACTGAAAACTTGCAGATTACAACTAAATCCATGAAGGCATGCAGCA
TGGCACCGCTTATGTTCATTATTCTCAACGGTACGATTCAAAGCAACCAGAAACACTATTGGTTGTAAAGAGCTTAAACACATAGCAAGACTGACGCGGT
ATGCATGCCATGAGTGATTGATAATGGTAGTGTAAATCTATTTCAAATAAAATGTATTTTATTGAAGAATACAGCAAATGATTTGTATTATAACTATTTG
ACACATTCTGCAAGTTTGTTGACCAACGTGAGTACATGATAGCTACAAATCTTATATTGACAAAAAGATATGGTGTGTGGAAAAAATTCAGGTGCTATTT
TTTTCTATTTTGAGAATTGTATGCATAGCAGAAGAGCACAAATTGCATTCTGGTTTATCCGTGCAGGCATTCCAGAGTATCAGAATGGGGATCAACCTTT
CTTCAGCTACATATGTAAATATACGGATGCTAGTTGCTCACTTAAAATTTTGTAGTGTTCAAAGGAATAACAACAGTAAGACGTTGACGCACCGAAAATA
ACCAGAAATAACGGATTGTACTATGACAAGACACACGACAGATACACTAGCTTAAGTTTGTACAGGAAAATATTTGGCCTATTCTTATGTCACAGTAAAT
TAAGATTATCACCTTAATCACAGAAATTCAAGGGGTAAACGATTATAGCACAAAACGATCCTGGACCCCAAGACCTGGGTCTCTGAACGGCTGAACCTCC
AATTATACCTCTATACATTTGGAATGTGTGTAGTCAAACTTTCGCTACTGGTATCTTTACAAATATTTACATTGAACACATCAAAATGATATACATGGAT
TCCTCTCGATATGTCTTTGAGGTTTGGGTTCTAAAAATGATATACAAATTATTAGGTTCTAAACTTAAATGATATATACACATCAAAATGATATACAAAT
TATTGGGTTCTAAACTTAAATGATG
>comp74035_c0_seq2
AGGCATACAGGGAGCCAGGCATGCGCAATTCGGTCCATCCTTCTCGGATCTCCACATCGGCAACCTGCAGCAAAGCCTGCTGTTCTCCGGCATCCTCTTC
CCTGACCACCACGCCCCTCACCCTCCGAGGATCAGCACCGACCTGACAATTGGCAGCCCACACGCTGGCCAAGACGCGGCGTGCTCCCTATCGTCTGGCG
CCAAGAAGCCTGACGACGTCAAGGCGACGAGACTAGTGCTCTTTGGACGGGCGATACTGACGGAGGAGCAGATCAAGGCCAACGCTTCCGTTGGCCCGTC
CTCGCTGGGAGCCACCGGGAACGGCTCGTCCAAATCCGACTGCGACGCCGAGAAAGCACCTACCAACACGTCCGAAGGCTCCAGCTCAGGTGTCATCCAG
GGCAGTCCTACCAAAAACAGCTCGTCCTCCTGGAGACTGCAGTGGTTTGGCGACAATGGCAGTCAGTCACCATCTGAGTTCGGGCTGGAACCTGGCCAGT
GCAAGGTGTTCGTGGAGTCGGACGCCATAGGCCGGAACCTTGATCTCTCGGCGCTGGGATCGTGCGAGGAGCTGTACGCCCGCTTGTCTGACATGTTTTG
CGTTGACAACGCTGAGCTGAGGAGCCATATGCTATACCGCACCGCCGCTGGCGAGGTCAAGCACGTCGGCGATGAGCCTTTCAGTGCGTTTGTGAAATCA
GCGCGGAGGATCATGATACTGACAGATGCCGGCAGTGACAACATAGGGAGTTAGGAATGAAGCGATCGGCCGAATAACTTAGGGCACTACTCCTAGGCAG
CAATAAGCCGAACTACATGCATGTGTACAGCCGAGCGAGCTGCGGTTTCTTCTTCGATCAGGCCTAGAGAATTCAGTCAGGAAATTAAGCTGACGCAGTG
TTCAGTGCTTCAGTTAGGTTGTTTTCCCACCCTTTTTCATTTAGTTCAGTAAAGCTCTTTTAGTCATGCAAACAAATTGTAATCTTGATCATCAAACGCA
TTTCTTAAATCTTGTTTCCGTTTTGGATTTCAAACCTTGAGCCGGGCCACAGAGACAAGAGCAGCAGGTGAAAAGGGTTAAATGCCATTGCTGG
>Locus_18892_Transcript_51/56_Confidence_0.074_Length_840
AAAATTTAATACTTACATGAGTATATACATCTCGTTTAGTGCTATAGTTTAACTCTTCACTAAGAGTTTGCTAAGTTTTTTTATTTAATCTCCTTTACA
TAAGGTAAATGATGACATAGACATGTTTAAAGCTAGCTAAGGAGGCTTCTTCGGGGCTCTTAGTTCAGGTCTTATAACCTTTATTGAACTTACGACATTT
GCACCTATCTGTTGCTGCTGTAGCCAATGAGTTCATCGTCCCCGTGGCCTGTGTATCACCTAAGCACAGCAGTACCACCTCACCATCAGCGTTTGTACTC
ATCTGCTTGTCCTGGCTTCTTTCCGAACCTCCAGGAAAGGTCAATGGAGCGGAGGCCTGAATTGGGTCCCCAGCCCTACCCCACCTGGGGATGGCAAATG

CAGGGCTCCCTTCACATGCCGTTGCACCACTCTGCAGCATCATCAGGATTCTCTACCGCCGTCGGCGCAAACGCCGGCGTCTCGTTGCCTTCTCACCCGC
CGGTGCCGTTCCCGGACCACCAGTTCTACTTCCCACCGACGGCATGAGCTTGTTGGTCTGGTTCAACTCTTGGGACGGCAGCTGATCTTATGTGTGAACA
TTTCGCAGCTTGCCGGTGACCGTTGTTAATTAATCGGGGAGAGAGAGCCAGAAGACCCAGTATGGCTATCTCTCTGTGGCCCGTGCCTTGTTATGATCTT
AGTTATTGCTACTACACCGTGGATTCATCATGGTTTATGAGATTCTTTACTCAAGTTTCAGCTTAAATGTCCAAATACATGAATGCATCATACACAGGAT
CCGTCATTTTCTAAAAAAATACAGGATCTGTGTGAAGTGT
>Locus_17579_Transcript_83/92_Confidence_0.239_Length_2695
GAGGAATCTCTTGTGGATTCCTTTTTCTGGGTGCAACGAAGTGGGAGGTACTTGGTCTTCGCACCTTGATGTACTTGTGGGTGCGTGTGTGCGCGCGCGC
GCGCGCCTTTCTTGATGCTAGAATGATGGTGGTGCTGTTCTTGCCATGATTGACGATTCCCCTAGAAAAAGTTGAACTACTCTGCACTCTTTCTAGCACA
TGGATAGCACCTTGTGTACTTCTCTGATGATGTGTAGTTGGGTGCTCAGATATGTTGTTGCAAATCTGTCCAATTTCTGCTACTCTCATATGTCAAATCT
TTTCTGTTTTTACTCTGTTTCACTCTTCCACTTTGTTGCTTCAAAGGTAGGAGCAGCAGTTGAGATTTTCAATTCTGGCATGGGTTCTTTTGGGATGGAC
TGGAACCAGAAGGGCTCCGTGTTGTGGGATTGGGAGAATTTGCCTCCGATAGGCGGAAATGCATACGAGAACCTCAAGGTTGCACCGCAGGCTGAACCGA
AGGTTGCAGGTGTTGAGGGCACAAGGCATGAATCAGTGCATTCTTCCTGTGGTACTTTCTCTTCCAGCTCGGAGATGGGGTATGGTTCATCCAAGACTTC
CATGTCCGCGTCAACTGATTCTTCGCCCAAGGTGGGAAACAACATGGAGCTCAATTTTGCAGCTGTCAAAGTGCCTGACAAGAACACCGGCAAGAATACT
GATTTGGGTAAAGTTGATGACGCCGGAACTTCTCCGTCATCCGTGATAGCCGGCAGCAGTGGAGAACCGGTGATTGGCTTGAAACTTGGCAAGAGAACCT
ATTTTGAAGATGTATGTGGAGGGCAGAATGCCAAGAGTTCACCGTCAGATTCGACTACAGTGACTCCTGCTGCTTCTGCGAAGAAGGCAAAGATGGCTCA
AAACACACAGAACTCATACTGTCAGGTTGAAGGTTGCAAGGTTGATCTCTCTTCTGCTAAAGATTACCATCAGAAGCACAGAGTCTGTGAAGCTCATTCT
AAGGCTCCCAAGGTTATGGTTGCTGGTCTGGAGCGACGCTTTTGTCAACAGTGTAGCCGGTTTCATGCTTTAACCGAGTTCGATCAGAAAAAACGAAGCT
GCCGGAGGCGCCTCAATGATCACAATACCCGCAGACGGAAGCCACAGCCTGAAGCAATTCCTTTCGGTTCATCAAGGCTCTCTGCAATGTTTTATGGTAT
CGCTAAGCCTTTGGTTGCCTAATGGTTTTGGGAAACTGGTGCATTAATTATTGTTCTTTTAGCAACTGTTCTGTTTAAAAATCTTGCATTTTTCATGTAT
GCCCAGAACTAAACGGTGGTAAAATTTATGAGTACAAAGTGCAATGTTGTGTGTCTGAAGTTGTCCCTTATTTGTTTATTCATAACTCAGTGCCATGATT
TTTGAGATGTCATGAATTTACATACTTCTGATATTAGATGACATGATAACTTTGGCAATACCGGTAATTAATGAAACTCCATCTGAGTACTGCCATTTGT
GATGATTACTATAGATGGGACAAAAGAGATATCAATTAAGTTGTATTTATTTTATGAATAGGTGCATAATGCTATATTCTTTTCGATTTAGATATCCATG
ACATGATTCTACTTTATCTGCCATGATGCCATCTCTCCATTATAGGAAGTCGGGAGACAATAGGAAGCTGTTAAGTATCTGAATAAACATCAGTTCAAAT
ATCTACAGATGCATGTTGAATCCACTATATTATCAATAATTTGTACGAAAGTAGCTTTAGCTTTTTGTATTAAGCAAATCGTTTGTCCTCTTTCTGAATC
TGTTTGGCTTTAGTGATGAAAAATTGTCGTGGCGGTCTTAAACAGATGCAAGGCAACAGACAAGTCTTCTGTTTGGTCAAGCTCCTTATGGTCAAATGAG
AAGCTGTGCAAACTCTTCATGGGATAGCCCAGTAGGAGGCTTCAAATTTGGAGAAACAAAAGCTCCTTGGTTAAAGCCAACGAGAGCTGCAGGTGTTGAT
GGGATGCATGTATCAAGCCAGCAGGTGTGGAACAATATTACGCCACACGGTGCACATCATGATTTTAATGGTATCATGGCTTTCAAGGGAACCAGTGCAA
ATGTCCTTAATCAAGGTGCCGAAGCTTCTCCGGTCGTCTCCAACTCGAATGGAGCCCCAGATCTTCAGCGTGCTCTCTCTCTTCTGTCAAACAATTCAGC
TGGTGCTGCCAACGACCAGCCAACTCCTCAGCTGCACCCTGGGCTGACCACCTTCGTCGGCTCCTCCAACCCTGTCACTGTGATGGAAGCCTCACCACCA
GGACTCTGGCAAGACAGCACGCCCCTTGATCATCAGGCGCAGTTCCAGGCTTTCGATCCCATTGCCGCACCGCAACAGCTCCAGCTCCCAAAACCCCCTT
CCTTCAACAGCTCCACTTCCAGCTATGGCCAGATGCAGTGATGGATGCTTCCTTCAGTTCTGCTGTCTGAAACTCTGAAATTTGAAACTGCTGTGTTCTA
AAAAATAAAGAAAAGCCTGTGTGCGAAAACTCAGGCTTGAAAAATTGATCCAGTAGTGTTCCCGTTCAATGGCTTGAATAATCCATCCTTGTGATGGCGT
GTAACTCAAATGCTTGGATTGCGCTGGCAAGTGATCATGGACTCTTGTGTTCTTGATGGCATCTCAATTACACCTTGAATGGAACTCTGGTTCTT
>Locus_20324_Transcript_17/30_Confidence_0.279_Length_1202
CCTCCATTTGTTGATGGGGTTTTTTTACTGCGGGTACCGGAGCTCGCAGTACAGGGGCGTCACCTTCTACAGGAGGACCGGCCGCTGGGAGTCGCACAT
CTGGGACTGCGGGAAGCAGGTCTACCTGGGTGGATTTGATACAGCGCACGCTGCGGCAAGGGCCTATGATCGCGCTGCGATCAAGTTCAGGGGCCTTGAT
GCGGACATCAACTTCCAGTTGAAGGACTATGAGGACGACTGGAAGCAGATGAGGAAATGGTCCAAGGAGGAGTTTGTTCACATACTCCGACGTCAAAGCA
CTGGGTTTACAAGGGGGAGCTCCATGTACCGCGGTGTGACACTGCACAAGTGCGGCCGGTGGGAAGCTCGGATGGGTCAGCTTCTTGGAAAGAAGTACAT
CTACCTTGGGTTGTTTGACAGTGAAGTTGAAGCTGCAAGAGCATATGACAGGGCAACCATTCGCTTCAATGGAAGGGATGCTGTTACTAATTTTGATCCT
AGTTCCTATGATGGAGATGTTCCACCTGAAATTGAGAAAGATGTGGTTGATGTTGATGGGGACATCCTCGATTTAAATTTGAGGATCTCACAACCTAACA
TGCATGATTCCAAAAGTGATGGCGTCCTCACTGGGGGATTAAATTGTGATTCCCCTGAAGCTTCAAGTTCGGTTGTTTCTCAGGAAGTGCCTATGGAGAA
ACGACCCGAGTTGTGTCCCCAGTCGTTCCCTCCGTGTGCATGGCAAAAGCAGGGTTCCCCTGCGCCATCACTCCCTACTGCAGCATCATCAGGATTCTCT
ACTGCCACCGGCGGCGCGGACCCGGCCAGCGTGCCGCCGTCCCGCCCCCGGCCCTTCGCCACCGGCCACCAGCTCCACTTCCCCCCAACCGTCTGACAGC
CTCCCCCTGTTCAACTGGCGACGATAACGCGGTCGTCTGGGCGCCCCATGCTTCGCTGCTGGTCGGCAACAGTCGCAATTGGTTCGGGTAGAGAGAGATG
GAGGCTCCAGCACACGACCTCTCTGTCTCTCTCCCCACCTGGTAATTATCATCAGAACCACCATGCTTATGATTCTTTTATCTTCATTTTGAGGGCGGAT
CCACGGATCATGAGCGTGTACCTCCAAAATTCTAAAATAAACCATTTACGATAACTCTGGCTGAGGCTCTGTTTGTTTGGGCTTCTGGCCAACTTTGAAC
GG
>Locus_9469_Transcript_46/63_Confidence_0.204_Length_1140
GAGAGAGAGAGAGAGAGAGAGAGAGAGAGAGAGAGAGAGACCCTGCAGAGGAGAGGAGCAAGGTGGGAACGAGGCAGCAAGGGAGGAGAGCTGCAGGAAGGG
GAACATGCTCCCTTCTCATCTCACAGTATGAACTGAAAAAACAAGAATCGAGAATTTCCATGAAATGAGACAAAATGGCACAATAATGATTCAGTTTGGT
CATCAAATGCCTGGTTACGACTCATCAGCTACCCAATCAACCAGCGAGAGTCATCAAGAAGTGTCTGGAATGAGTGAAGGAAGCCTCAACGAGCATAACG
ATCGATCAGGTAATCTCGATGGTTACACAAAGAGTGATGAAAATAAGATGATGTCAGCTTTATCTCTGGGCAATCCAGAAACTACTTATGCGCATCCAAA
ACCTGACCGTAGTCAGCCCTTTGCCATTTCGTACCCGTATTGTGATTCATTCTATGGTGGTGCAGTGGCAACGTATGGCTCACATGCTATTATGAATCCC
CAGATTGTGGGCATGATGTCGTCCTCCCGAGTGCCATTACCAATTGAACCAGCTGCAGAAGAGCCCATTTATGTGAATGCAAAACAATACCATGCGATTA
TCCGAAGGAGACAGCTCCGTGCAAAGCTAGAGGCTGAAAACAAGCTGGTGAAAAGTCGCAAGCCATACCTTCACGAGTCTCGGCATCTGCATGCCATGAA
GAGAGCTCGGGGAACAGGCGGGCGGTTTCTCAACACGAAGCAGCAGTCAGAGGCTGCTGGCGGCGGCACCTCGGACGCGCAACGCATACCAGCAAATGGT
GGCCTGTTCAGCTTACCTCCCAGTGATCTCCACTATCGCGCGAGAGGGGGCGCTTAAGGAGTCCCATGGCAACTCATCCTTGGCTTAAAAAAAATGTGTG
GCGCTCAGCAATTCGACCTCTGCTAGTTCAATGTAAAGGTGTGGGACTGTAACTGTGGGTGCTTGAATTGGCCGCAGCCATTCCTGCGACGCGTCGCAGG
TGTTGCCGGTTCTGTGGTCGTGCTAGACTTGTGACCGGCACGACGAAACTGGCACCTGTTTCCTGTAACTGTTGTCTAAGATGATGATGACTACTCCAAT
AACCATTCTGTATGATGCTTTGACGTCGGTGTTGCTCCTT
>Locus_2827_Transcript_137/166_Confidence_0.142_Length_3039
AGGCGGAGAGGCTGTGAGGTCGGTGGGTGTGCTGTGCGTCTGCTCCGGCGGCGCTCCCCTCGGCGAGCGGGAGCTTGTCGGTGGGTCTCGGCGGTGTTCG
CTTCGTTTGCAGATAACTTCGCGAGCTGAAAGTTGAAGTGTAAGCGGGGAGCTAAGAGCAGTAGTTGAACGAAGCGGAAGAGGAGGACGTGATTTGAGTA
GTTGGGCTCTTGCATTGGCGAGGAAATAATGGCTGCGGCCGTGGCGATGCGAGGGGGAAGCAGTGACAGCGGAGGGTTTGATAAGGTTCCGGGGATGGAC
TCGGGGAAATACGTGCGGTACACCCCGGAGCAAGTTGAGGTACTCGAGCGGCTGTACATCGATTGCCCCAAGCCAAGCTCCTCGCGGCGGCAGCAATTGC
TGCGCGAGTGCCCTATACTTGCCAACATTGAGCCCAAGCAGATCAAGGTTTGGTTCCAGAACAGAAGGTGTCGCGATAAGCAGCGGAAGGAGTCTTCGCG
GCTTCAGGCTGTGAACCGGAAGTTAGCGGCAATGAACAAGCTTCTGATGGAAGAGAATGAGCGTCTTCAGAAGCAGGTCTCTCAGTTGGTTCATGAGAAT
GCACACATGCGGCAGCAGCTGCAGAATACTTCTTTGGCCAATGATACAAGCTGTGAATCAAATGTGACTACCCCTCCAAACCCTATAAGGGATGCAAGTA
ACCCTTCTGGACTCCTTTCGATTGCAGAGGAGACCTTCACAGAGTTCCTCTCAAAGGCTACTGGGACAGCTATTGATTGGGTCCAGATGCCTGGGATGAA
GCCTGGTCCGGATTCGGTTGGTATTGTGTCTGTTTCACATGGTTGCCGAGGTGTTGCTGCCCGTGCCTGTGGTTTGGTGAATCTAGAACCAACAAAGGTC
ATAGAGATCTTGAAAGATCGTCCATCTTGGTTCCGTGATTGTCGAAGCCTTGAGGTGTTCACAATGTTTCCAGCTGGAAATGGGGGAACAATTGAACTTG
TCTACATGCAGATGTATGCCCCAACAACTTTAGTCCCTGCACGTGACTTTTGGACGTTACGATACACGACAACAATGGAAGATGGCAGCCTTGTGGTCTG
TGAGAGATCCTTGAGTGGTTCAGGGGGTGGTCCAAGTGCAGCCTCTGCGCAACAATTTGTAAGAGCTGAGATGCTTCCAAGTGGGTATTTAGTTCGCCCA
TGCGAAGGTGGGGGCTCGATTGTGCATATAGTGGACCATCTAGATCTCGAGGCGTGGAGTGTTCCTGAAGTGCTTCGACCACTCTATGAGTCTTCTAGAG
TAGTTGCTCAGAAAATGACTACTGTGGCACTGCGCCACCTTAGACAAATTGCTCAAGAAACAAGTGGGGAAGTAGTGTACGCCTTGGGCAGGCAACCTGC
AGTCCTACGGACATTTAGTCAAAGGCTGAGCAGGGGCTTTAATGATGGCATTAGCGGTTTCAATGATGATGGCTGGTCTATAATGGGGGGAGATGGTATT
GAAGACGTAGTTGTTGCTTGCAACTCAACTAAGAAAATTAGGAACAACAGCAATGCTGGAGTTCCGTTTGGAGCCCTTGGAGGTATTATATGCGCGAAGG
CATCCATGTTACTGCAGAGTGTTCCACCAGCAGTATTGGTTCGATTTCTGAGGGAGCATAGATCTGAATGGGCCGATTACAATATTGATGCATATTTGGC

TTCAGCGCTGAAGGCCAGTGCGGGCTCACTTCCTGGGTTACGGGCCATGAGATTTTCTGGGGGCCAGATGATCCTGCCACTTGCCCACACAGTGGAGAAT
GAGGAGATTCTTGAAGTTGTCCGCCTTGAAGGACAATCTCTTACTCACGATGAGGCTCTTATTTCAAGGGATATCCACCTGCTTCAGCTTTGCACTGGAA
TAGACGAGAAATCTGTGGGGTCCTCCTTCCAGCTCGTGTTTGCACCAATTGATGAACATTTTCCAGATGATGCTCCATTGATTTCTTCTGGCTTTCGTGT
CATACCACTTGATATGAAAACAGACGGCATATCTTCTGGTAGGACGTTAGATTTGGCATCTAGTCTTGATGTTGGTTCTGCCACGCCTCACGCCTCAGGG
GATGCATCTCGAGATGATTGTAATTTGAGATCTGTGCTGACCATCGCCTTTCAATTCCCTTACGAGATGCAGCTCCAAGACAGTGTTGCAACTATGGCCC
GTCAGTATGTTCGCAGTGTTGTTTCTGCTGTGCAAAGAGTGTCGATGGCTATCTCTCCCTCCCAATCTGGTATAAATGCTGGGCAGAGGATGCTTTCTGG
CTTCCCTGAGGCAGCCACACTTGCTCGCTGGGTTTGCCAGAGCTATCATTACCATCTAGGGGTAGAATTACTTAACCAGTCAGATGAAGCTGGCGAAGCA
TTGTTGAAGATGCTCTGGCATCATCCAGATGCTATTTTGTGCTGCTCTTTTAAGGAAAAACCTATGTTTACATTTGCCAACAAGGCAGGGCTTGACATGT
TAGAAACATCCCTTATTGCCTTACAAGACCTCACATTAGACAAGATCTTTGATGAGTCTGGAAGAAAAGCATTATTCTCGGACATCTCTAAATTGATGGA
ACAGGGATACGTGTACCTGCCTTCGGGCGTATGCATGTCAGGAATGGGTCGCCATGTTTCGTTCGATCAAGCTGTGGCGTGGAAGGTCCTTGGTGAGGAC
AGCAGCGTCCACTGCCTGGCCTTCTGCTTCGTGAACTGGTCCTTCGTGTGAGCCTTTGCCATCCTCCGAATGCCGACTTGATCTGTGACGAGGAGATCCA
TTTTTGTATTTATCTGGAGAAGGAAAATCTTCCACTGTGACAAGGCTTATGTTGCACTAGCTAGCTCTAGGTTTGGGAATCCAGTGTAAAATCTGATGTC
TTTCCATTTGGCTACCAAGCTGTGCTCTGGTTGTTTATCTTGTTTGGGACGATGCTAAGTTGCTAACATTGGGTTCCCAACTTACGCGTCGAAGTGTGTT
TCATCGCATCAATCTTCAATCGCAGCTGAATTTTCGTTT
>comp79901_c0_seq35
GACGCGGCGCGGGCCGAGGTCCCGGAGCTCGCAGTACAGGGGCGTCACCTTCTACAGGAGGACGGGCCGGTGGGAGTCGCACATCTGGGATTGTGGGAAG
CAAGTCTACTTAGGTGAGTAACTGAGTTGAGTGAGTTCTATACGTTCCAGCTCGAGCTTCCATTAACTGCTGATCAGTTTCCGTCCGTTTGATCTGGTTT
ACGATGTGCTGATGCTGTTCTATTCAGGTGGTTTCGACACTGCTCACGCGGCTGCGAGGGCTTATGATCGTGCAGCGATCAAGTTCCGAGGGCTCGACGC
GGACATCAACTTCACTTTGAGCGACTATGAGGATGACTTGAAGCAGATGAGCAATTGGACAAAGGAAGAGTTCGTGCACATACTCCGCCGCCAGAGCACG
GGGTTCGCGAGGGGGAGCTCCAAGTACCGCGGCGTGACGCTGCACAAGTGCGGCCGCTGGGAGGCGAGGATGGGTCAACTTCTTGGCAAGAAGTAAGCCA
ACCTCCTGGCTTTGACCTTTCTACTCTGTTAGCCTAGTTAAGTGTTTTTTACCCTGGTTATATGAACAAAATATGTCGAAATGATGTTCAATTAACTTGT
CATTTCACGTTGGGTTCGATCAGGGCTATATCCCTTATGTTCATGTTAAGTAGTTTCAGGTGATTGTAGAGAATACAACAAGGTGATCTTGAAGTATCAC
TGATTATAATCCATGCTACTAGTAGTTTGAGCGTCTCTGTTGCCGGTGAAATCTCCACCCCTCTTGTGACTTTACCCCTCTACCGTTGAATCCGGGAATC
CGGAATGGACCTACAAGATCGAGATATGTGCCTTGATGAACAGTGTTTCGGTGAACAGCAAAATCGGCACCCGTGCACAGTGTCCTGATGGACGCAACCT
CTAGAGTTCACATATATGCATTGGCCAACAATGCAATTCCTGCTTTTTAGTCAGGAACCAGTTTTGTCAAAGTCTACGACACTAGGCATCCCTCTGTTGA
GACATATCTCAGAACTGGAATTTACATGCAGGTACATCTATCTTGGACTCTTTGACAGCGAAGTTGAAGCTGCAAGAGCATATGACAGGGCAGCCCTTCG
CTTCAATGGGGGGGAAGCTGTTACTAATTTTGGGCCTAGCTCCTACAATGGAGGAGATGCTCTACCCGACACCGAAAATGAGGCAACTGTTGATGGTGAC
GCGGTTGATTTGGATTTGCGGATTTCACAACCTAATGTGCACGACACTAAAAGGGACAATACCTTAGCTGGAGGCCAGCTAACATGCGACTCCCTTGAAT
CTTCAAACACGATGGCCTCTCAGCCAATGAGCTCATCGTACCCGTGGCCTGTGTATCACCAAAGCACGACAGCAGTACCACCTCACCATCAACGCTTGTA
CTCATCTGCTTGTCCTGGCTTCTTTTCGAACCTCCAGGTATTACCAAGCTAAAGCCTATTTTTTATGTGCAAATTGCTACTCCTACGTGATCAATTAGTT
CAAACTGTTATTCGAATCCAGGATTGTTTACTGAATATGTCCTGTCTTCAGAAAAAATAACACAAAAAGGAACATAATATGTCCTGTCTAATTGTGGACT
GATACTAGCCAATTAAACAGTTTCCCATATCCATTTCCTGTGTTTATTGTTTTGATTTGCTGGCATGGTTAAACTATAGTAGCTTGATTCGATAAAAACA
CTAAGCAGGCTTTGATATTTTGAATGAAATGTCGTTCTTGGAAATACAGCCTTTTCCTTGGTATATCAAGGACAGTTGTCCATCTGCACTCTGCACCCTC
AGCTTGTGTAACTGTTTGCCTTGTGAACTGATTGATGTGTCAATCAGGCAGTTCTGCCAGTTTGCATCTTTTGAAATAGTGCAGTCTTCCAGTCTAACAG
CTCCATGACATAACTTGGGATGCATGCTGCAGCTTTCTTACTGAGGATGCTCTAGATTTACAGAAATTCTCGCAGTTAGCAGAGGGCTATCAAATCTGAT
TTGCTTAGCTTCCTGCAGACAATAAGATAAGAACTCTAGAATCCTGAAGAAATCTTACTTGTACATGTTTACCAAAGTATCCTTACTCCTTAGTTGAGCC
AATTTTGGTGAAGAACCGATAGCTCATTACTCTGTTTCCTGGGAATGATGTGGGCCTAAACCATTCTGTGGTCAAAGTTTAACAAAGCCCATGCTTGTGC
TGTTGCAACTGCAGGAAAGGCCAATGGAGCGAAGGCCTGAGTTGGGTACCCAGTCATTCCCCACCTGGGGATGGCAAATGCAGGGCTCCCCTCACATGCC
GTTGCATCACTCTGCAGCATCATCAGGATTCTCTACCGCCGTCGGCGCAAACGCCGGCGTCTCGTTGCCTTCTCACCCGCCGGTGCCGTTCCCGGACCAC
CAGTTCTACTTCCCACCGACGGCATGAGCTTGTTGGTCTGGTTCAACTCTTGGGACGGCAGCTGATCTTATGTGTGAACATTTCGCAGCTTGCCGGTGAC
CGTTGTTAATTAATCGGGGAGAGAGAGCCAGAAGACCCAGTATGGCTATCTCTCTGTGGCCCGTGCCTTGTTATGATCTTAGTTATTGCTACTACACCGT
GGATTCATCATGGTTTATGAGATTCTTTACTCAAGTTTCAGCTTAAATGTCCAAATACATGAATGCATCATACACAGGATCCGTCATTTTCTAAAAAAAT
ACAGGATCTGTGTGAAGTGT
>comp79438_c0_seq12
AGTAGGCTCATATTTGGCATCAGCAGAGGCCTCGCGGTGCCCCGTATTCTCATTTCCTCGCCGTATTTTCTCTCTCCGGCTCGAGAACCCGGAGGAAGTA
GAGAGAGAAACTCGAGGGGGAGGTAGAGAGAGGTGTCAATTCATCCGATTTATCAGAAGTACCTCAAGCAAAATGTTGCTCGGAGCTAAGGATACTATCT
CGTAGTGCTCACGCTTTTGTGGGAAATGGAGGATCATCCAGTCCATCCCATGTCGAAGTCCAACAATGGCTTCTTGTCAGGAAATGGCTATCAGATGAAG
CAATTTGGTCATAAAATCCACAATAGGGACTCGTCATCAGAGTCCGGTCGGTCTCACCAAGAAGCTTCCACAGCGAGTGAGAACAGTATAAATGAACAGC
ACACCTCAACACAATCTGACAATGATGATGGTCATGGGAAGCATAATGAGGACATGATGAAGTCAGCAGTGTCCTTGGGGGAACCAGGATCAGCCTTTTT
GGCCCCAAAACTAGATTACAGCCCATCTTTTGCTTGTGTTCCTTATACTGCTGATGCTTTTTATGGTGGGGTCTTGACAGGATATGCTCCACATGCCATT
GTTCATCCCCAGCAAAATGATTCAGCAAACTCTCGGGTTCCGTTGCCTGTTGAACCTGTAGCAGAAGAGCCAATATATGTTAACGCAAAGCAATACCGTG
CTATCCTTAGGAGGAGGCAGATACGTGCTAAACAGGAGGCTGAGAATAAGCTGGTGAAAGGTCGGAAGCCATACCTCCATGAATCTCGACACCGCCACGC
AATGAAGCGAGCCCGTGGATCAGGGGGGCGGTTCCTCAACTCAAAGCAGCTCCAGGAGCAGAACCAGCAGCAGCAGGCATCAGGTGGTTCAAGCTGCACA
AAGGCCATCGGCAATAACACAAGCTCCCAAAGTGGTCCCAGTCACACGCCTCTGACACTTGCTTCCTCTGACACTGCAAGCGCTTCGAGGGCCAACCAGG
AGCGCACCTGCTTCCCCTCGGCTGGCTTCCGTCCCACAATGAACTTCGGCGCGCGAGGTGGAGACGCAAAGCTAGCTGTGAATGGCATACAGCAGCATGT
TTCCTTCATCAGGTGAAGCAAGTGAAATGGGGCGCTGCCTGCGGTAACTCATCCTTGGCTTATGAAACTGTGAATGTGTAATGGACATGTAACTTCAGTC
TCAGAATAACCAAACATATGGGAGTCCTCCTGAAGTTTCCTGTAGTTCAAAGAACTGATAGGTTATGGCCTTATAGGTTTGCTTCTGGAGACTGAAATCC
GTGCTTGATATACTATCTTGTGCGAGTTCTGAGGTGAAAGACCTTGAAACTTGCTCTTGTGGTTCAGTACCTGTTGCTTTTGTGTCTGCAAGGTTAGCAC
TGCAATTTGCAATGTCGGATTTCTGTTATGCCACTAATTTTT
>comp73418_c0_seq6
TAGGATGACTGTGTGGAGGATATCTAACGTGGTGCTTAAACTCGATGGCAGCCAATGGTGGTTCTTGCCTGCCTTACCATGAACTAGTAGTTTGGATCGG
AGTTGTTCTTGACTTACTCATGCGTATTCTTGGGAGTTGGGATCATATCAGTATGTATACATCTGTTATTGCTTGGTGGTGACTTTTCCTTAGAGGTGCC
TGATATGGTGGCGTTTTTTTCTTTGACACATGTTGTATGGATCATAAGTGATGTTATACTCTTTACTAATTTCATGGCCTTTATTATCTTTAGGTTTCGT
TTTCTCTTTTTCTTTTGTTATTATTGGTAAGAAGTATTCGTGTCCCTAGACTTTTGCCATGTATGTACATGTTCGTAGCGAGGTTAGTGATACTGTGAAC
TGTGGTAGTCTTGTTTCGCTGGGGGGAACTTTGATCTGTTGTTCTTTGCCTCAAAACACTGTAAGCCTATTTCTAATATCTAAAACTTGTAAGTGTGCAT
AATGCAGATCAAGCTTCCGAGGTGTTATTTTTCTCATTATGTTTTAGAAACTTAATGATAGATGCAAAGTTATTTTGTTAAATGGAGTCTCAGTCCCGTA
GCCATAAGGGAGGATCGAATCCAAACTTTAGGCTTGTGTGAGTCTTCTTGAGTGCAGCACATGATAAACATGGATCTTAGGGCATTGAGTTAACAGTTAG
TCATCTTCTTTTCTAAACATAAGGATTAGGGTGCTAGTCACACACCATTAACAATAATGTGAATCCAAATAGCATAAGTAGCACGCTTGAGGCCGGCCAA
CACACACTATTCATCTCTTGCTGATTCACTAATTGTCGGTTTGCATTTGGAAGTGTTCTCTAGTTTCTGTGCAGTAACTATTGAGCTAATGACACACTAT
GTATAAATGACAATCGTAACAGATCATTTTGAACCTTTACTTGATTATTATTAGAAACACCTAGGATTTGAAGGATATCGATGTTACTGACTTACTTCTC
TTTTGCTACATCTGGTTAGGACAGGATTCTACTGTGTTACAATAGGGTTTTCTTTGGAAATTTAACGTTGTACAATAATGCCTTTATTTTTTTTGTATCA
GGTGACTCCTTCGGGAAAATGGTTAATGATCATACGAGGTCAAATTTGGTTTTTGATAATAAGCAATCTCTATTTGCAAGTCATAACATTGACTACGGCC
AGCCAATAGCTTGTATATCATACCCATACAATGATTCGGGCTCAGGAGGTGTTTGGGCAGCCTTTGGGTCACGCACTAGCGCTGCAGCTGTGTTCCATCC
CCAAATTGCTTGTGGGGGCACATCTGCGAGAGTTCCCTTACCTCTGGAATTAGCAGATGATGAGCCCATATATGTCAATCCCAAACAATATCATGGTATA
CTTCGCAGAAGACAGCTACGTGCTAAGTTAGAGGCTCAGAACAAGCTAGTCAAAAACCGAAAGCCTTACCTTCATGAGTCTCGGCATCTTCATGCAATGA
AGAGGGCAAGAGGTTCTGGCGGACGTTTCCTTAATACTAAACAGCTCCAGGAGCAGCAGCAGCTGAAGTCTCGCAATGCCTCCACCAGGTCCACCACAAA
TGGCGCAAATTCCTCAGGTTCAACACATCTACGGCTTGGTGGTGGTGCAGATGGAGATCAAACCATGTCGGGGACGAAAACAATGGCCTCACAAAACAAT
AGCAAAAAGGCTGTTTCTTCTTCTGCAGCTCTTGCTTTCACCGTGACTCCTATGGTGCGCAAAGATGACACCTTCTTCCAGCACCTCAGCCACAATGTCA

GCTTCTCCAGCCATTTTGGCCAGGCAAGCGCCCAAACCGGCGTTGGAGGCATCCATAATGAGACCCAGCATAGGGTTTCCGTGATACAATGACGGTTTGC
GAAGCTTATCTGGTGATCCAGGCTTCTAGGTGTCCTCGTGTCCGGTGTAGTCTTGGTCGCTCAGGCAATTCATCCTTGGCTTAGTTTTTGGTGTGTTAGA
ACCTACAAATGTTTCATACTCTGTTGTGGTTTGCAGAGCCCATAAATCAGGGCTCTCCTAAAAAAAATCAGGGCTTGATGGCGACACTACTATCCAAGTA
TTGTTGTAATGGTGTGTGTTAGAAACTCGCAAAAAACCCTTCTTTGTGCTCGTATTAGTACATTATGCTGCACAATTAGCTGCTATTCTGTTCGTACTAC
AGTCTGGT
>Locus_5523_Transcript_15/107_Confidence_0.081_Length_328
AAGCAAAAGAGGATCATCACCTGACCATGAGCGCAAGTGCGGAGGCTAAGGAAGCAGGAAGCTTGAGGCAGCAACTGCATAACTTGCAAGAGCACCATCG
GCAGTTGTTGGGTCAGCAGCTTTCTGGCCTTGATGTAAAAGGCTTGAAAGATTTGGAGAATAAGCTGGAGATGAGCCTAAGAAATATTTGTCTGAAGAAG
GACCAACTTATGATTGATCAAATTCAAGAATTAAACAGGAAGGGAAGCCTCCTGCACCATGAAAACATAGAACTATACAACAAAGTCAACCTTGTTCAAC
AAGAGAACATCGAATTACAGAAAAAGGT
>comp81084_c0_seq6
CGCACAACTTTGTGTGTTTAAATGCTCGTAGTACTTACAACAGGTGCGTTGGTTGCTACTTTTAACCGGAACAAGTAGCAATCATGCACCTACAGTAAAC
AAACTTGTCCAAATTGATTCTTCGTTGTCCAAAATCCGTGAATTGGACGCACGTCTGTAAAGGTACAAACAATAAATAACAGACCATATAAAGCTATTTA
AAAGCCTAAATCCTCAAATTCATGAAAAACCGAAACTCAAAATTCTTCGATGAATTTCATCACGATGCTCTTATCTACCGGTCACCCTTGCTTGCGGCTT
TGAATGCACCAAGGTTCCTTTTTAGAGACTTGACAGCAGACAAAATCTGCAGCTTCTCTTTGGATGCCCCATCAGGTTTCTGATCATCGTTCTTACTGGC
TGCCTCATCAGGTGGCAACCTACCTCCCGCAGACGATCCCTCCACAGGAAGTTCCCTGTCTGCATTGCTGGCATGTGCCCCCTGCTTCCCAGCTTGCTTT
CGCATGAAGGCCGCACTCCTCTTGTACTGGGGATCAGTAGGATCCAATGCGAACAAATGAGATGTGAACATTTCTGAGAACCTCTCATCTTTGCTAAGAT
CAATATCAGGAAGTTTGTCCTCGACAGATTCCTCTTTACCCTTCTTACCCTTGTTACCCTTTTTGTGTTTCAAGTTATAGCCCTTTGCACCATTGGCAGT
ATCCTGGTCAGCAGCAACCAAGAGCTCCAACTCTTCTTTCGTTGCTTCTGGCTCCAAATGTTCCTCTGTTGGTTTGTCCTTTCCTTTCCCCTTTGCTTTA
TCTTTAGTCTTCGCCTTTTGTTTCTTGATTTTAATTGGCTTAGTTTCTTCATCAGACAGTTCCTCAGCGAAGAAATCATCATCGTCATCGGGCTCATCTT
CGCTGCTATAGTCATCGTCATCATCCTTCAACCCCCTCTTCGTAGCCTTCCTTTTCTCTTTCATTTTCTCTTGGTGCCTCTCCCAGACAGTTTTCTCCTC
ATTGGTCTTTCTCTCTAGGATACGTTTACTGAGATCCTCAAGCTCTGTGTTGAATGTAATCTCCATATCCTGGCCACCAGCTTGTTCCTCTTCAGATTTG
TCGCCTTCCAGTAAAAGAGCCAACCTCTCCTCTTTTGTTAGCTTTCGTTTGGATCCCCCATTTGGTAGAGACTCATTATCAGGATTGTCTACACCGTCAT
CATCTGACGCACTGTCATCACTTGCTAAATACATGTCAAGCTCATCTAACTGATCATCAGTGAACTTTCGCCTCAAGACCTTCTTACGTTCTGGTTCATC
ATCATCCCAAGTTAGCTTAACTCTGCTATGTTGCAGAGCACGAGTCTCAAAATCAGGCTCCTTGTAACTCGGAGGTGCCTCTGTAGCAATGTCTCGAGCA
GGATGTTTGAACTCCATAGAATCAGGTATAAACTGCAAATCAAATACATTTGCTGTTTTCAAGAACTCAGTACCATCAAGATTCATGTACAGGTGATTTG
CTGTTGCACTAGAATCACAGACCACAACAGCATAGTAATACCTCAGTCTGTTTAGCTCGTAAGTACGCAGCTTGTTATTCTCTTTGTCAGAGTCAACTTC
TTCTTCGTCGTCATCAGTGTTGAATTCATCATCATCAGTGTCGTCTTCACCATCGTCGTTATCATCATCATCGTTGTCCTCATCATTATCATCAGTATTG
GCATTAACAAGAGCTGCTGGGCCCTGAGTTGATTCAATTTCCATGCACTTCAGTCCAAATTCTGATGGATATATTGAGACTGATAAAACTCGCCCACCTT
TTGGGACGCAAGATGTCATCACCATATACAGGTCAACTGCCTTGATATGATCCCAGTCCATATTAACAACAGCAAGCCTGTGAGTTTCCTTGTCAATCAT
AGGCGTATCATCGTGCCGGCCCATTAAGTAATGAGCAATGTCACTGCCGACAGAGTACTCGTCATCATCAACGTCTTCATCCTCGTCGTCGTCGCTACTA
GAACTCTCTTCTTCCTCCTGCTCATCCTCTTCATCCACGTCCTCGTCCTCCTCTTCTTCAACTAACTTCTCTTTCTCCTTCTTTGCCTTCTCTTTAGTCT
CATCCTCCTCCTCGTCTTGGTTGAGATAGTAACGGAGCATGGGGTTCTCCCTGCCGCTCTTCTTCCGTCGCCTGCCGCGCTTGTCCACCGGCGCCGCCGA
GGAGGAGAACCTCGGGTCCGTCATCATGCTGCTGAACCTCGAGTCCAGCGCCACCTTCGCCTCCTTCCGCCGCATCGACCGGAACCGCGGGTCCATCCGC
GCGGCCGCGAACCGCTCGTCCTGCATCGCCCGCTTCATCCTCTCGTCCCGCCCCTCCACCGCCTCCGCTTCAGCCGCCTTGCCCTCGTGCTTCCCCTCCT
TCTTGGGCTTCTTGCCGTGCTGCCGCTTCTCGTCCTCCTCGCGCCCCTCCTTGTGCTTCCGCTTCTTGCGCTCGGCCTCGGGGCGAGGGGCTTCCTCGGT
GGCCGCCTGACGCTCTTGATTCTTCTTCTTGTGCTTCCTCTCTTCCTTATCGGACCTCGATTTCTTGGCCTTCTTGAGGCGCGCGGGCTCGTCGGAGCCG
GTCGGCGG
>Locus_12915_Transcript_19/30_Confidence_0.725_Length_4236
GCTCCTTGAGAAACACCATCCAATTACAAGACTATTTGATGCAACAATCACGCATATGGCTGCGCTAGAACTACTGCTCTGAATTAAGAGGCGTTGAAAT
ATTGTCCGGTCTACATATAGGCACTTGAGGTGTATTGACAGACAGTTCCCCTTAGTTTGCTACAACACATCAGGCGAATTCCACCAAATTAGGATGATGT
TTGTTTCTTAGACACAGGATAGCAATGCCAAAAGTTTTTTATAATTTTCCGCTTCACCGCAGATTTGGCATCTCAAAGGGAGTTGTGGCAAGATTCTCTT
GACCAAGCTTTCTTGCAGATTTGGCAAGAAAGATAAAAAAAATGTTGGTATACTTAAGCATCCTTAATAGAAACTAAACAGGTCTTAGATTGAAATTATC
ATAAATAAACCCTTTTTCACTTTAGCTGTAGTCCGGATGAAATCTTAGGGGCCTTGCTTAGCCTGCCCTGAAGCTTGCCAGTAAGTCGAAGCTGATGCTG
ACACAGCACTGACATAGATCCCAATGAACACAAGTTATTTCGAGAAGATAAAATACCAAGCAAAGCCAGGATTGCATTAAAAGATAGTGTATTTCTACTA
TACGATCTGAAAGCTTAATTTATTATATCAATAATCAAATTGGCAGCAGTGTGGAACAGATATAAAGAAGATGCTTCAAATCACAAATAACTACAGATAA
ATTGAAGGGTGAAGGTTCATTCAACCACCAAACACCCAAAATGTCGAGCTGCATATGCACAGAACCTAAGTACGGTACAGTTCATAGTTTGGGAAAATAG
ACTGCAAATGTGCACAGATAATACAGATTGTTACTCGATGAACAGTAAGCACGGTATTTGAAATCCTTCTAACCATCCATCCAGGCAGTGCCTCGTATAC
TCAAACCTCTTTTGGAGGAAGCCAAACCATGTGATCCTTTTCAACCCAAATACATATATTCAAGATGAAACTAACAAAAGACAGTTGATAAAGAAACA
CGAAAGAGAGTAGAAAAAGGGTACCTGTTTCAAGTTCTCATCTTCTGAAAATGATCAGCAGTCAATAGATGTTTTAGTCCATTCTCCAGAAAAATGAGTG
CCGCATGAATCCTTTCCGGTTTATAGTGAAACATTTTAGACGATGACCTTTAACATTCTAGAAAACGAAGAGAAAACCTACAGTCATCTAATTTCAGCTT
AAACATGTTCCAGCAAGATCATCACCATCCTGACATGTTCCGTTTTGTCCCTAGACCTGAGCACAAGGTATTGCGAGGCTAATTTAGCGGGGCAATTGAT
TTTTTGCCACTATGATACAACTGCAATTACCCGCTGTCACTGACACGTGGGGTCATCTGAGTCACTGAAATGTGGGCCCAGTGGCATTTTGTTTGCCACT
CACCCTTCCAGAGTGGCAAAAAGTTAATTGTCCCTAATTTAGCTCAATTCAGTTGTCGGAGCAATTGTGTGTGAAAAGTGTGAACAACAGCATAGGAGAC
AATACAGCAAGTGTAGCGTGCCAATATGTGTCCAGCTTGATAGTTCACTATAATAAAGTTCTTTAATTCAAGTATTTGAATAATATAGAATCTCAACCTT
CAAGTTTAAAAGGTTTTGTATTTGCTATTATAATCGTTGCCACCCCCTCACCCCTCTCAAAAAGAGAGTTACTTTGTAGCGCACTGTAAATCTCTTGATT
GAAATAGTGGCATGTCATCTTATTTAATTCAGTTATTTCATAAGGTACAATTTGTTTAGCTGACAGCTCAGACATGCTTCTATGCAACATGTTTATACCA
ATGCTGTATTTTGGCAATCCTGGATGTGGCATTACTTAACTGTGCTTTCATATTCAATAGTTCTGGCAAAGGGAGGCTGCAAGCTTGAGACAACAACTGC
ACAACCTGCAAGAAAGTCATCGGTATGGCTAGAAAACCCTGCAAATTTTACAAATGTACAAGTCAGAAAATTTGCCTAATGTAAAAGGATCAAACTACAG
GATTTGTCAGCATGAGCACAAGTGCCTAACTTCCGAGTTAGATAACTCTGGAGGAAGGAAATCCTTCCTACTGTTTGTTTCAAGAATATACAAAAATTCA
AAACAACATTAGAATAGAACCAAAGAAGAAACAATATTCACCATGTAGCAATACTCAATTCCTGGTTGTCTTATTTATAAAGGTATAAAACTAGGTTCT
GACGAGACAATTATGAATCAACTACACCATGTTACAGATCATCCATAACTCTTGGTGATGCAAATTAAGGGCTGTAAAGTGACAAGTATACCTCCACCAA
CTAATTTGTGCAGAGTGTATAAAATTGTAAAACAATGGATAAGAATGAATTTAGTATACAAGATAGCATACAATGCCCAAGTTTAAGGAGACATACTGAT
TTACTGACTCCATAGGACAATCTTTTGCTTATGAGGACATCAGTTTTCATAAACAATATCAATGTAAAAGGAGGTAACAGTTTATGAATTTCCTATGCCA
AGCCATTTATAAGTCAAAAAATGTACCTATTTAGCTTTTATGCATTCCAATTAACTTCCAAAGAAGAAGAGTACATACTAATGGAAAACAAATATGATAT
TATCATCTGTTTTGCCCGGTAGTTTCTTGAGTCTCTGATTTGTCCGCACTTCACTCCCAACCAACACAACATTAATACACATGGGCAGAGGAATATCAGG
TTATCAGGTAAACTGAATACAGTTTGATTGACTGCAGTCAGTTGATGGGACAAGATCTTTCTGGATTGAGCGTCAAGGAACTCCAAAATCTAGAAAATCA
GCTAGAAATGAGCCTACGTTGCATCCGGACAAAAAAGGTAAGGATGCTAGCCATATTTCTGTTTTTCGTTACCAGATTGCCTCATCCAAGAATACAACTG
ATGATGACGTTTTTTACTTTCAGGACCAACTCTTGATTGATGAAATTCACGAACTGAATCGAAAGGGAAGTCTCATGCAACAAGACAAGATGGAACTATA
CAAAAAGGTCAACCTAATCCGTCAGGAAAACATTGATTTATACAAGAAGGTATTTGATAAATCGTGAATGAAAATTCACAGGATATACACCCCATCCATT
ATTGACTGAGGTTTATTTCTCTACTCAGCTCTACGAGAAAGAGGTGACAAGTGAAGTCAACCGAGATTCATCAACTCTAAACTTTGCAGTTGTCGAGAAC
GTCAACATTCCTGTTCATCTTGAACTTAACACTCCACCGCAAGAAAATGATGTTGAGCAAACTGCACCTCCTAAACTGGGATTACAACTAAATCCATGAA
GGCATGCAGCATGGCACCGCTTATGTTCATTATTCTCAACGGTACGATTCAAAGCAACCAGAAACACTATTGGTTGTAAAGAGCTTAAACACATAGCAAG
ACTGACGCGGTATGCATGCCATGAGTGATTGATAATGGTAGTGTAAATCTATTTCAAATAAAATGTATTTTATTGAAGAATACAGCAAATGATTTGTATT
ATAACTATTTGACACATTCTGCAAGTTTGTTGACCAACGTGAGTACATGATAGCTACAAATCTTATATTGACAAAAAGATATGGTGTGTGGAAAAAATTC
AGGTGCTATTTTTTTCTATTTTGAGAATTGTATGCATAGCAGAAGAGCACAAATTGCATTCTGGTTTATCCGTGCAGGCATTCCAGAGTATCAGAATGGG
GATCAACCTTTCTTCAGCTACATATGTAAATATACGGATGCTAGTTGCTCACTTAAAATTTTGTAGTGTTCAAAGGAATAACAACAGTAAGACGTTGACG
CACCGAAAATAACCAGAAATAACGGATTGTACTATGACAAGACACACGACAGATACACTAGCTTAAGTTTGTACAGGAAAATATTTGGCCTATTCTTATG

TCACAGTAAATTAAGATTATCACCTTAATCACAGAAATTCAAGGGGTAAACGATTATAGCACAAAACGATCCTGGACCCCAAGACCTGGGTCTCTGAACG
GCTGAACCTCCAATTATACCTCTATACATTTGGAATGTGTGTAGTCAAACTTTCGCTACTGGTATCTTTACAAATATTTACATTGAACACATCAAAATGA
TATACATGGATTCCTCTCGATATGTCTTTGAGGTTTGGGTTCTAAAAATGATATACAAATTATTAGGTTCTAAACTTAAATGATATATACACATCAAAAT
GATATACAAATTATTGGGTTCTAAACTTAAATGATG
>comp78487_c0_seq6
ACCCGATTTGTTTAGATTGCAATTAGACTAAGTATAGTTGGTGAGAAGACAGGAGTAGAGATAAGGGCCAACAGCCCAACAGAAAAACCAAGCAAGACTG
CAACTGCAAGAGAGATGGGGAGAGAGAGGAAACCTTGCAGAGGAGAGAAGCAAGGTGGGAAAGAGGCAGCAAGGGAGGAGGGCTACAGGAAGGGGAACAT
GCTCCCTTCTCATCTCACAGTATGAACTGAAAAACAGGAATAGAGAATTTCCGTGAGATGAGACAAAATGGCACCATAATGATTCAATTTGGTCATCAAA
TGCCTGATTACGACTCATCAGCTACCCAATCGACCAGCGAGAGTCATCAAGAAGCGTCTGGAATGAGTGAAGGAAGCTTCAATGAGCATAATGATCGATC
AGGTAATCTTGATGGTTACACGAAGAGAGATGAAAATAAGATGATATCAGCTTTATCTCTGGGCAATCCGGAAACTGCTTATGCACATCCAAAGCCTGAC
CGTAGCCAGTCCTTTGCCATATCATACCCGTATGCTGATTCATTCTATGGTAGTGCAGTGGCGGCTTATGGCTCGCATGCTATTATGAATCCCCAGATTG
TGGGCATGATGTCATCTTCTCGAGTGCCATTACCAATTGAACCAGCTGCTGAAGAGCCCATTTATGTAAATGCAAAGCAATACCATGCGATTCTCCGAAG
GAGACAACTCCGTGCAAAGTTAGAGGCCGAAAACAAGCTGGTGAAGAGTCGCAAGCCATACCTTCACGAGTCTCGGCATCAGCATGCCATGAAGAGAGCT
CGGGGAACAGGCGGGCGGTTTCTGAACACGAAGCAGCAGTCAGAGGCTCCTGGCGGCGGCACCTCGGACGCGCAACGCATACCAGCAAATGGTGGCCTGT
TCAGCTTACCTCCCAGTGATCTCCACTATCGCGCGAGAGGGGGCGCTTAAGGAGTCCCATGGCAACTCATCCTTGGCTTAAAAAAAAATGTGTGGCGCTC
AGCAATTCGACCTCTGCTAGTTCAATGTAAAGGTGTGGGACTGTAACTGTGGGTGCTTGAATTGGCCGCAGCCATTCCTGCGACGCGTCGCAGGTGTTGC
CGGTTCTGTGGTCGTGCTAGACTTGTGACCGGCACGACGAAACTGGCACCTGTTTCCTGTAACTGTTGTCTAAGATGATGATGACTACTCCAATAACCAT
TCTGTATGATGCTTTGACGTCGGTGTTGCTCCTT
>Locus_21011_Transcript_3/9_Confidence_0.273_Length_1077
TGCGAGGCGCACTCCAAGACGCCCGTCGTCACCGTCGCCGGCCAGCAGCAGCGCTTCTGCCAACAGTGCAGCAGGTTCCATTTGCTCGGGGAATTTGACG
AGGTGAAGAGGAGCTGCAGGAAGCGCCTTGATGGGCACAACAGGCGCCGCCGGAAGCCGCAACCGGATCCTCTGAACCCTAGAGGCTTGTTTGCCAATCA
CCATGGAGTGACAAGATTTGCATCGTACCCCCAGCTCTTCTCCGCAGCAACCATGGCAGAGACCAAATGGCCGGCCACCGTCGTCAAGACAGAGACCGAC
TCGTACCACGACCAGTACTACCCGGCCGTTCACCTCAACAACGGCGCCGCCTCTCTCTTCCACGGCAAGGAGAGGAAGCACTTCCCGTTCTTGACTGACC
ACGGCGGGAACGCAGCCTTCTGCCAGCCGTTCACTATCACCCCTTCCTCAGAGAGCAGCAGCAAGCAGAGCAACGGCGATTGTGCTCTCTCTCTTCTGTC
AGACAACCCGACACCAGCACAGATGATGATCCCCACCGCACAGCCCCTCGGCGCGGCGCTGCAGTTCGGCAATGTGGCACGGCTGACTGACGACGGCGAC
GTCTCCCTCACCGGGATGTCTTATGTAAGTGTGGGAGGCAAGCAGACATCCATTCTGGCCACATCTACTAGTCACACTGCAGCAGCTGCTGCTTCTCCTG
CTCCTGCTAGTGTAGCTACAACACAGCTGCAATACCATGGCTACTACCATGTGAGCGGTGGTGATCAGGGCAACCCAGATGGTGCTGCCATTCAGGCCCT
TCCCTTCTCATCGTGGTAGAGGAGTTCCCAATCAGGTGTTCTGATCAATCAGGTGCTGGGCAGTCGCTCTTGTTGTTCATTAAATATGTCGGTCATAATA
GTAGTCTTGATACTATAAACATCGACAAGAGATGTCCATTTTTTAAAACTTATCCTGATAGTTCTAATTTACGGAAATCCCCCCTGTTTAAGATTCAGAA
TCGTGTAGACGAGCACTTTTCATAACTTGCACGGTTGAGTATTTGAAATCTTGGAAGTAATTCTGTTTGTTTTGATT
>Locus_5523_Transcript_78/107_Confidence_0.180_Length_1806
AAGGCAACAAAGAGAATGCAGGGAGATTGTTACATACTGCAGTGTACAACTTGGTTCAAATCGTTCCAAGTTTTACATGCACTTACTGCTAAGAAATGTA
TAGCATGATTGATGAATCAACAACACAGAACTACATTACAAAGCTTAAACAAAAGGAAAAGTTCACCTTGGATCATACTGTAACATCAATCTGCTCCCAC
GTTGCCATAAGGGTCAGCGAGGAGCATGAATGAGATTAGGATGAAACCTGGGGCAAGTCAGTTTCTGCGGAACCGCAGCACAGTCGCTGCGCGTCTATTT
CAAGCATCAGATACAGCAGCCACAAGAACAACTAGGATTTTTGGTCGTTGGCAATCTCTTTTTCTTTCTCTTCCTCTTTCTCTTCACTGGATCCATCCCC
GGTCGTTCCATCCAGGAAGCGGACGATGACAACTGTGATGTTATCAGAACTCCCCCTTTGGTAAGCTTCTTGGAGAAGTTTCTCTGCTGCTTGCTGGGGC
TGGTCCTGTATGGGCTTCACCATGGCAACAGCTTCCTCATTAGTGACAACATCCCAGAGTCCATCACTAGCAAGGATGAGGAATTCAAGGGAGCTATCCA
CCACCTCCTCCTTGATTTCTGGGTCAGCGACAACATACTGCTTCAAGAGTTTATCACCAAATGCTCGAGAAACAGCAAGAACACCACCCACACGCCATGT
CCCAGCCCACATCACAAAGCCCCCAGCATCCTCAATTCGTTGTCTCTCATCAGACTGATCAGGTTTGTGATCTCTTGAAACCGCAATAGCCTCCCCTCCT
TTACAAACAACAGCTCTAGAGTCTCCAACATTTGCAACAAGCAAACGATCACCCACAATGATAGCTGTTGAGGCAGTTGAGCCAGCATCTCGAGTTTGAC
TACTTTCGGCTTTAAGAAATTCTGAATCTGTGTGGTTGTATGTTTCCGCGATAGCAGACTTGGTATCACTGATGAACTTTGGATGTTTTATTAGGTTGCT
GAATAGGTGCTGCTTGACATACTCTGCTGCTCGTGCTCCACCATGACCATCATATACGCCGAACAATCCAACTTTCTCTCCATCAATTTCATCTATCCTT
GCCTCATAAAAATCCTCCATTGATGCTCTTTTCCCAGGAGAACTTGCAAATCCATAACTGAACCGGCCATTTACACTGCGCTGGCCTCCGCTGTCCGCCA
ACCCGGTCCCGACGAGCCCCATGCGCGCCCCTTCCGCGTCCGCCAGGCACCACCAACAGGACGCCCGCCCCGCGAGCAGGAGGAACAGCAGCAAGAAGAG
AAGCAGCGAGGACGGGAGGCGCGCGGCAGATGGTGGCCCCGACGACCTCTGGCGCATGAAAAGAGGAAGGGAGGCAGGAGAGCGGGAGGGAGGAAGAAGG
GGGAGGAATGGATGGACGAGCTGCGATTGCAAGGGGAGAGAGAGAGTAGAGAGAGATCTGTGCTACCTGTGGGGGAGAAGATGGGAAGAGGGAAGATAGT
GATCAAGAGGATTGACAACACGACAAGCCGGCAGGTGACGTTCTCCAAGCGCCGGAGCGGGTTGTTCAAGAAGGCCAAGGAGCTCGCCATCCTTTGCGAC
GCCGAGATCGGACTCATCGTCTTCTCCAGCACCGGTCGCCTCTATGACTTCTCCAACACCAGCATGAAGTCTGTGATCGAAAGATACAATGAAGCAAAAG
AGGATCATCACCTGACCATGAGCGCAAGTGCGGAGGCTAAGTTTTGGCAAAGGGAGGCAGGAAGCTTGAGGCAGCAACTGCATAACTTGCAAAGAGCACC
ATCGGT
>Locus_16183_Transcript_29/55_Confidence_0.367_Length_1386
AGTAGGCTCATATTTGGCATCAGCAGAGGCCTCGCGGTGCCCCGTATTCTCATTTCCTCGCCGTATTTTCTCTCTCCAGCTCCAGACTCCAGAGTCCGGA
GGAAGCAGAGAGAGAAGCTCGACGGGAAGGTAGAGAGAGAGCTAAGGATACCATCTCATAGTGCTCACTCTTTTGTGGGAAATGGAGGATCCCATGCCGA
AGTCCAACAATGGCTTCTTGTCAGGAAATGGCTATCAGATGAAGCAATTTGGTCATAAAATCCACGATAGGGACTCGTCATCAGAGTCCGGTCGGTCTCA
CCAAGAAGCTTCCACAGCGAGTGAGAACAGTATAAATGAACAGCACACCTCAACACAATCTGACAATGATGATGGTCATGGGAAGCATAATCAGGACACG
ATGAAGTCAGCAGTGTCCTTGGGGGAACCAGGATCAGCCTTTTTGGCCCCAAAACTAGATTACAGCCCATCTTTTGCTTGTGTTCCTTATACTGCTGATG
CTTTTTATGGTGGGATCTTGACAGGATATGCTCCACATGCCATTGTTCATCCCCAGCAAAATGATTCAGCAAACTCTCGGGTTCCGTTGCCTGTTGAACC
TGTAGCAGAAGAGCCAATATATGTTAACGCAAAGCAATACCATGCAATCCTTAGGAGGAGGCAGATACGTGCTAAACAGGAGGCTGAGAATAAGCTGGTG
AAAGGTCGGAAGCCATACCTCCATGAATCTCGACACCGCCACGCAATGAAGCGAGCCCGTGGATCAGGGGGGCGGTTCCTCAACTCAAAGCAGCTCCAGG
AGCAGAACCAGCAGCAGCAGGCATCAGGTGGTTCAAGCTGCACAAAGGCCATCGGCAATAACACAAGCTCCCAAAGTGGTCCCAGTCACACGCCTCTGAC
ACTTGCTTCCTCTGACACTGCAAGCGCTTCGAGGGCCAACCAGGAGCGCACCTGCTTCCCCTCGGCTGGCTTCCGTCCCACAATGAACTTCGGCGCGCGA
GGTGGAGACGCAAAGCTAGCTGTGAATGGCATACAGCAGCATGTTTCCTTCATCAGGTGAAGCAAGTGAAATGGGGCGCTGCCTGCGGTAACTCATCCTT
GGCTTATGAAACTGTGAATGTGTAATGGACATGTAACTTCAGTCTCAGAATAACCAAACATATGGGAGTCCTCCTGAAGTTTCCTGTAGTTCAAAGAACT
GATAGGTTATGGCCTTATAGGTTTGCTTCTGGAGACTGAAATCCGTGCTTGATATACTATCTTGTGCGAGTTCTGAGGTGAAAGACCTTGAAACTTGCTC
TTGTGGTTCAGTACCTGTTGCTTTTGTGTCTGCAAGGTTAGCACTGCAATTTGCAATGTCGGATTTCTGTTATGCCACTAATTTTT
>comp76332_c0_seq39
CAATTTCTATCAATAATTCAAGTGTCGCACAGCTACAACGATGCTCCTAATTATGAAGAGAAAAAAACAGATGAATTAAAGGCTGAAGGTTCATTCAACC
ACCAAGCACCCAAAATGTCGATCTGCATATGCACAGAACCTCAGTACAGTTCACTGGTTGAGAAAATAGACTGCAAATGTGCAAATAATACAAATTGTTA
CTGGAAGAACAGTAAACACAATACCAGAAATCCTTCTAACGGTAACCATTGGAGGAAGCCTTGTGATCCTGGTCCAAGTTCCCATCCTCTGAAAAAGATC
AGCAGTCAATAGATGCTTCACTCGTCCTCCAGAAAAATGAGTGCCGCATGAATCCTTTCCGGTTTATAGTGAAACATTTTAGACGATGACCTTTAACATT
CTAGAAAACGAAGAGAAAACCTACAGTCATCTAATTTCAGCTTAAACATGTTCCAGCAAGATCATCACCATCCTGACATGTTCCGTTTTGTCCCTAGACC
CGAGCACAAGGTATTGCGAGGCTAATTTAGCGGGGCAATTGATTTTTTGCCACTATGATACAACTGCAATTACCCGCTGTCACTGACACGTGGGGTCATC
TGAGTCACTGAAATGTGGGCCCAATAGCATTTTGTTTGCCACTCACCCTTCCAGAGTGGCAAAAAGTTAATTGTCCCTAATTTAGCTCAATTCAGTTGTC
AGAGCAATTGTGTGTGAAAAGTGTGAACAACAGCATAGGAGACAATACAGCAAGTGTAGCGTGCCAATATGTGTCCAGCTTGATAGTTCACTATAATAAA
GTTCTTTAATTCAAGTATTTGAATAATATAGAATCTCAACCTTCAAGTTTAAAAGGTTTTGTATTTGCTATTATAATCGTTGCCACCCCCTCACCCCTCT
CAAAAAGAGAGTTACTTTGTAGCGCACTGTAAATCTCTTGATTGAAATAGTGGCATGTCATCTTATTTAATTCAGTTATTTCATAAGGTACAATTTGTTT
AGCTGACAGCTCAGACATGCTTCTATGCAACATGTTTATACCAATGCTGTATTTTGGCAATCCTGGATGTGGCATTACTTAACTGTGCTTTCATATTCAA
TAGTTCTGGCAAAGGGAGGCTGCAAGCTTGAGACAACAACTGCACAACCTGCAAGAAAGTCATCGGTATGGCTAGAAAACCCTGCAAATTTTACAAATGT

ACAAGTCAGAAAATTTGCCTAATGTAAAAGGATCAAACTACAGGATTTGTCAGCATGAGCACAAGTGCCTAACTTCCGAGTTAGATAACTCTGGAGGAAG
GAAATCCTTCCTACTGTTTGTTTCAAGAATATACAAAAATTCAAAACAACATTAGAATAGAACCAAAAGAAGAAACAATATTCACCATGTAGCAATACTC
AATTCCTGGTTGTCTTATTTATAAAGGTATAAAACTAGGTTCTGACGAGACAATTATGAATCAACTACACCATGTTACAGATCATCCATAACTCTTGGTG
ATGCAAATTAAGGGCTGTAAAGTGACAAGTATACCTCCACCAACTAATTTGTGCAGAGTGTATAAAATTGTAAAACAATGGATAAGAATGAATTTAGTAT
ACAAGATAGCATACAATGCCCAAGTTTAAGGAGACATACTGATTTACTGACTCCATAGGACAATCTTTTGCTTATGAGGACATCAGTTTTCATAAACAAT
ATCAATGTAAAAGGAGGTAACAGTTTATGAATTTCCTATGCCAAGCCATTTATAAGTCAAAAAATGTACCTATTTAGCTTTTATGCATTCCAATTAACTT
CCAAAGAAGAAGAGTACATACTAATGGAAAACAAATATGATATTATCATCTGTTTTGCCCGGTAGTTTCTTGAGTCTCTGATTTGTCCGCACTTCACTCC
CAACCAACACAACATTAATACACATGGGCAGAGGAATATCAGGTTATCAGGTAAACTGAATACAGTTTGATTGACTGCAGTCAGTTGATGGGACAAGATC
TTTCTGGATTGAGCGTCAAGGAACTCCAAAATCTAGAAAATCAGCTAGAAATGAGCCTACGTTGCATCCGGACAAAAAAGGTAAGGATGCTAGCCATATT
TCTGTTTTTCGTTACCAGATTGCCTCATCCAAGAATACAACTGATGATGACGTTTTTACTTTCAGGACCAACTCTTGATTGATGAAATTCACGAACTGA
ATCGAAAGGTTCTGGAATATTCTGTTATTACTCTGCAATGAGAGGTCTCTGAGTGGATTTCAAGAAGCTGACTCTACTGGCTCATTTTCAGGGAAGTCTC
ATGCAACAAGACAAGATGGAACTATACAAAAAGGTCAACCTAATCCGTCAGGAAAACATTGATTTATACAAGAAGCTCTACGAGAAAGAGGTGACAAGTG
AAGTCAACCGAGATTCATCAACTCTAAACTTTGCAGTTGTCGAGAACGTCAACATTCCTGTTCATCTTGAACTTAACACTCCACCGCAAGAAAATGATGT
TGAGCAAACTGCACCTCCTAAACTGGGGTAACCCTGCTTTTTTCTGCAAAGGAAGATTTTCTTGCAAGTTATGCAGTTGTTGTCTCTAGCTTGCCGCCTC
CACTTGCAGTAACTGAGACGCCGGCGCCGAGCTTGAGCTTGAAACCTAGACTACGGGGACAGGGTAGACAGGAGACGCGAAACCCCAAACCAGCGCAAAA
CGACGCGCAGGGAG
>comp78295_c1_seq9
ACAACTGCACAACTTGCAAGAAAGCCACAAGCAACTAATGGGTGAGAAGCTTTCTGGCCTAGGTGTGAGAGACCTCCAGAGTTTAGAGAATCGTCTTGAA
ATGAGCCTACGTAGTATCAGAACGAGGAAGGACAATCTTTTGAAAAGTGAAATTGAAGAGTTGCACAGGAAGGGTAGCCTAATTCACCAGGAAAACTTGG
AACTCTGTAGAAGAGTAAATACCATGTCACAACAAAATATGGATCTACAAAGAAAGGCGAGTGAACCAAGAGGTGTTGCCGATGCAAATAAAAGCTCTAG
CACTCCCTACAGATCTTGTAGTGCACAAGATGCAGATATCACGGTTAATCTTGAATTGAGCCAATCACAGCAAAAAGAGGGGGAGCAGTGCCAAAGAGGG
GCTCCAGAACTGGGGCTTCAGCTGCACAAAGAAGATGCACTACAAGCTGGAGAGTATCATGATGTTCCACCAATTCTAACATGCAATGCGGAGCACTTAA
ACTACAATTAGTATTTTGTGCAACATGTTAGATGAGTGAACAAAATGGTTTATGTACAGACTACAGATATGACCATCTATCTTATCTGTGGCTCTACTGT
ACTGAAATTGCAAGAAATGGTTATTGTAATGACTAATATGATACATTGACTGTCATACCGTTGACCTTGAAGATAGTTCTATAACGGAAAGGTCCAATTT
GAAACAAGTGTTACGGATGAGCTAGTAGGATTTTTTTCACAAACAATTAGATAGTCCACGTTAGTGGTAGCTGCTGGGTATGACATTTGACCCAGCCTGC
CGACCCTCCCTTTCCCATGGGAGTTCAATTAACATTAGGAATGAATATCGATATTATGGTTGATTATTTGAATCGTATGTTTTCCACCCTTAGATGTAAG
GCCCTATTTTACTGTTTTAATGGATTAACAAGGTGAGCTTTCGAGATGGTAGCTCATTGATTCGCATGCATTTTAGTGGCTGTTAAGAGGTACTTAGTGC
ATCCATGCTTGAAGGTGTTGTTAGTTCGACAAACCATTTTCTATCCTACTGGATTACTATCTTTTGGTCAGTATGACAAGCAAGAATTTAATCTGCCAAT
TAAGAATTTAATGTGTTCTTTTGACATGAATGTCAACAGCATTTAAGTAACTCAACTTTAATACAAAGTACTGGAATGTTTTCTCACCGAAAGTAAAGA
TGTGGTTCCTTGATAGCAAACAATGTTTATGCCAATATCGCATTAAGGTGGTTGTATCCTTTAGAATGATCTCCTCATTCGTGCTTAATTTGATGCTATT
GTCTTTTTACGGATGCCTAGATGTGGTTTTGACCATTACTAAGTACTTTAATATATATTTAACAAAAATAATGACATAGCTGTTGTATGGCCAATCTAGA
TTTTGTTTTCGAAACATATTTGCAGTCAAACTATAGGAAGTAAGAAACTTCAATGTGGTTCTCGAAAACCATGCTTAAAGTTTAGAAAGTTTGACTACAT
AGAGTATATACTACATAGTCTTAAAGCATCAAATGAAGAGGAATGGAGGGAGTAGCAGACAATCATCATTCAATATCTATTTCTTGGTTGCCTTCAAATT
TGGGCTTACTAAAATCGATTTAAAATTCTCGTGTTCCAGTTGAAGCTCACTTCAAGTAAATCAGGAATTACCATATTTTTCATTGCTCATAATTTGGAAA
CATGGCATGATATTTGATAGAAAATGTACAAATCCTCAGCTGCTCAGAAAGTTACAAAGGGAGGAACGCCCATGAAAGAGAATTCATGGCTGCAACAACG
AACATAAACACAGCCTATTCTGTCCAGCAGTCATGGTAATACAAGCTAAAAAGGACCGCTCTGGAACAATAGCTTGAAAAGCAATATTAATACAATTTCA
GATGAACTATGTGCGTCGATGTCTTACTTGTGACTTCCTTGCAAGTAATGCGGTTCCTGCCTCATGCTTGGTGCCGCCGCATGCAATAAACCGATAACTC
GAATCCTCCGTGACTTCCACCCTCTCCAGCCAATCAGCGGCTTGCTGCGTGCGGGCGCTGGTGTGCGGGGGGCGCCCGGCTTTATATTAGGGGCGGGGGAG
AGTCTTGAAG
>Locus_5983_Transcript_65/82_Confidence_0.096_Length_1037
GATTACCATCGCAAGCACAAAGTCTGTGAAGCTCATTCTAAAGCTCCCAAAGTTGTTGTTGCCGGTCTAGAGCGCCGTTTTTGCCAGCAGTGTAGCCGAT
ACAAGCCAGCGGACAAATCTTTTCTGTAATCAGCCTCTTTTTGGCCAAGTGAGAAGCAGTGCAGTTTCTTCATGGAATAACTTAGGAGGCTTCAGATCTA
TGGAAACGAAACATCTGTTGATGCAGCCATCGAAAACTGTAGGCCTTGATGAGCTGCATTTCTCAACCCCCCAGATATCAAATAGTGCTGTGGCTCATTC
TGTACATCATCGTAATTTCGATGGGCTCATGCCATTCAAGGGAACCAACACAAAGGTCCTCAACCAAGGCGTGGAAGCTTCTGCGATCGCTTCCAACTCG
AGTGGAGACCCGGATCTTGGGTGTGCTCTCTCTTCTGTCAATCGGTTCGTGGGTTTCAAGTTCAACCGTCATCCAGCAGCCTAGTTCTCATGTGCACG
CTGGTGTGGTGCCGCCCCTCGCCACCGTTGCCGTCTCCAACCCTGCAATGCATCCTCTGGACTCATCCCCAGGAGGATTCTGGCAAGACGTCCCTCCCCC
GCTCGATGAAACTCCACATATTCAGGCATTCACGCACCTCTGATCAAATGCTGCATCCCCTGGGAACAGCAGGTGGTTATACCTTCCGTAATCGTCCTAG
GTTTGCTTTGCCAGTGACTGGCTCTGCACAATGTCGATCCATCACCGGAGGCATTTGGTAAGGTGAAGACTAAAGACTCAAGAATAACCGGAGACTAACT
CGCTTCTCTTCCAGGCTCAAGACTGTTTGGGTCCAGCGGTGTTGCTACCTGATCCTGTTTTCGGTTCTGAGTACATCCATTACTAATCATGGGATTACTT
GTTTAGGCCAACCAGACATATTCTGGGGTGAGATTACGACATGTTAATTTACATTGACGGTGTTTCTACATGTGTTAGTACTAATGATGTTATCGCTCTT
GGCCCCGTTACGTTTCAAGATCTGTTACCATTTGAGT
>Locus_15085_Transcript_34/40_Confidence_0.358_Length_1852
GCTGCTCGAGGAGCTCAGCTTCAAGCGCATGGTTGTCACCGACGAGTGCCTCGAGATGATCGCGGTTTCCTTCAGGAACTTCCAGGTCCTGCGCCTTGTC
TCCTGCGAGGGCTTCAGCACCGCCGGGCTCGCCGCCATTGCCGCCGGCTGCAGAAATCTAAGGGAACTTGACCTGCAGGAGAATGAGATTGAGGATTGTT
CTAGTCATTGGCTCAGTCTCTTTCCAGAATCTTTCACTTCTTTGGTAACTCTAAACTTTTCATGCTTAGAGGGGGATGTCAATATCACTGTACTTGAACG
ACTAGTAACCAGATGTCACAACCTCAAGACTCTCAAGCTCAACAATGCTATCCCTCTTGACAAGCTTGCTAGCCTCCTTCGTAAGGCTCCTCAAATTGTG
GAGCTTGGAACAGGAAGGTTCTCTGCTGACTACCACCCAGATCTCTTTGCAAAGCTTGAAGCAGCGTTTGCTGGTTGTAAAAGCCTACAAAGGCTTTCTG
GGGCTTGGGATTCTGTTCCAGAGTATCTGCCAGCATTTTATTGTGTATGTGAGGGCCTCACATCACTTAATCTGAGTTATGCTACTGTGCGAGGCCCTGA
GCTGATAAAATTTATTAGCAGATGCAAAAACTTGCAACTATTATGGGTGATGGACTTAATTGAGGACCACGGTCTAGCTGTTGTGGCATCATCTTGCAAT
AAACTACAGGAGTTGCGGGTCTTCCCTTCTGCCCCTTTTGATCCAGCTGAGCAAGTTTCATTGACTGAAAGGGGACTTGTTGATGTTTCTGCCAGTTGCC
CGAATGTTGGAGTCAGTCCTCTACTTCTGCAGACGGATGACTAATGAGGCCCTTATTACCATAGCAAAGAACCGGCCCAACTTCACCTGCTTTCGCTTAT
GCATCATCGAGCCTCACACTCCAGATTACATCACGCATCAGCCTCTTGATGCAGGTTTCAGTGCCATTGTGGAATCATGCAAAGGCCTCAGGCGCCTATC
TGTCTCAGGCCTTCTCACAGATCGTGTATTTAAATCCATTGGGGCACATGCTGATCGTCTTGAGATGCTCTCAATCGCCTTTGCTGGGAATAGCGGATTG
GGCCTTCATTACATCCTTTCGGGCTGCAAGAGCTTGAAGAAGCTGGAGATCAGGGACTGCCCATTTGGTGATAAGCCCTTGCTGGCAAATGCTGCCAAGC
TGGAGACAATGCGATCCCTTTGGATGTCGTCGTGCTCACTGACCCTGGGCGCATGCCGACAGCTTGCACGCAAGATGCCCCGCCTTAGTGTGGAGGTCAT
GAATGATCCTCGACGGGCATGCCCCTTGGATTCACTTACAGATGAAAGCCATGTCGAGACATTGTATGTCTACCGGACAATCGCAGGTCCAAGGTCCGAC
ACACCAGCCTGTGTCCAGATTATTTAGGGGGACCACCTGTGGTATGGGATAGGTACTATGGAAGTGTTCCTTGTCAATCCCGATTTGTACCTTCAACGCC
TTCCTACCGTAACCTCTGCTCAGCTGTGAAACTGTTTGCGAGTTTAGGACGAGACTCAGGGACCCTTTTCAGGTGTCAATAACTGTAGGTGGAGACTCGA
TGAGTTACAGAAACTACTAGATAGCGTCTTGAGAAAACTTGGGTTTGTTTTCTTAGCATGTTTAGTTCTTTCCATGTATAAAAATATGGGGTCATTTCAA
TCTCGTAATGTACTGTACTATCGGAACAAATTGTACCAGGAGCCTCTACTTTTTTGACCTCCCCTCTCAAATTACGGGTTGTTGCCTCAACTTGTTGTGA
ATGGGGAATAATTCAAGCTTATCTGTGTCTCTTTATTCATGAGGTATTTATC
>Locus_10929_Transcript_37/78_Confidence_0.186_Length_1269
TCGCAGTACAGGGGCGTCACCTTCTACAGGAGGACGGGCCGGTGGGAGTCTCACATCTGGGATTGCGGGAAGCAAGTCTACTTAGGTGGTTTTGACACTG
CGCACGCGGCTGCGAGGGCCTACGATCGTGCTGCGATCAAGTTCCGAGGGCTCGACGCGGACATCAACTTCACTCTGAACGATTACGAGGATGATTTGAA
GCAGATGAGGAACTGGACCAAGGAGGAGTTCGTGCACATCCTCCGCCGCCAGAGCACGGGGTTCGCGAGGGGGAGCTCCAAGTACCGCGGCGTGACGCTG
CACAAGTGCGGCCGCTGGGAGGCGAGGATGGGTCAACTTCTTGGCAAGAAGTACATCTATCTTGGACTCTTTGACAGCGAAGTTGAAGCTGCAAGAGCAT
ATGACAGGGCAGCCCTTCGCTTCAATGGGGGGGAAGCTGTTACTAATTTTGGGCCTAGCTCCTACAATGGAGGAGATGCTCTACCCGACACCGAAAATGA
GGCTGTTGTTGATGGCGATGCGGTTGATTTGGATTTGCTTTCACAACCTAATGTGCACGACACTAAAAGGGACAATACCTTAGCTGGAGGCCAGCTAACA

TGCGACTCCCTTGAATCTTCAAACACGATGGCCTCTCAGCCAATGAGCTCATCGTACCCGTGGCCTGTGTATCACCAAAGCACGACAGCAGTACCACCTC
ACCATCAACGCTTGTACTCATCTGCTTGTCCTGGCTTCTTTTCGAACCTCCAGGAAAGGCCAATGGAGCGAAGGCCTGAGTTGGGTACCCAGTCATTCCC
CACCTGGGGATGGCAAATGCAGGGCTCCCCTCACATGCCGTTGCATCACTCTGCAGCATCATCAGGATTCTCTACCGCCGTCGGCGCAAACGCCGGCGTC
TCGTTGCCTTCTCACCCGCCGGTGCCGTTCCCGGACCACCAGTTCTACTTCCCACCGACGGCATGAGCTTGTTGGTCTGGTTCAACTCTTGGGACGGCAG
CTGATCTTATGTGTGAACATTTCGCAGCTTGCCGGTGACCGTTGTTAATTAATCGGGGAGAGAGAGCCAGAAGACCCAGTATGGCTATCTCTCTGTGGCC
CGTGCCTTGTTATGATCTTAGTTATTGCTACTACACCGTGGATTCATCATGGTTTATGAGATTCTTTACTCAAGTTTCAGCTTAAATGTCCAAATACATG
AATGCATCATACACAGGATCCGTCATTTTCTAAAAAAATACAGGATCTGTGTGAAGTGTAAACATAAGG
>Locus_5417_Transcript_18/28_Confidence_0.312_Length_4712
ATTACAAGACTATTTGATGCAACAATCACGCATATGGCTGCGCTAGAACTACTGCTCTGAATTAAGAGGCGTTGAAATATTGTCCGGTCTACATATAGGC
ACTTGAGGTGTATTGACAGACAGTTCCCCTTAGTTTGCTACAACACATCAGGCGAATTCCACCAAATTAGGATGATGTTTGTTTCTTAGACACAGGATAG
CAATGCCAAAAGTTTTTTATAATTTTCCGCTTCACCGCAGATTTGGCATCTCAAAGGGAGTTGTGGCAAGATTCTCTTGACCAAGCTTTCTTGCAGATTT
GGCAAGAAAGATAAAAAAAATGTTGGTATACTTAAGCATCCTTAATAGAAACTAAACAGGTCTTAGATTGAAATTATCATAAATAAACCCTTTTTCACTT
TAGCTGTAGTCCGGATGAAATCTTAGGGGCCTTGCTTAGCCTGCCCTGAAGCTTGCCAATAAGTCGAAGCTGATGCTGACACAGCACTGAAATAGATCCC
AATGAACACAAGTTATTTCGAGAAGATAAAATACCAAGCAAAGCCAGGATTGCATTAAAAGATAGTGTATTTCTACTATACGATCTGAAAGCTTAATTTA
TTATATCAATAATCAAATTGGCAGCAGTGTGGAACAGATATAAAGAAGATGCTTCAAATCACAAATAACTACAGATAAATTGAAGGGTGAAGGTTCATTC
AACCACCAAACACCCAAAATGTCGCGCTGCATATACACAGAACCTAAGTACGGTACAGTTCATAGTTTGGGAAAATAGACTGCAAATGTGCACAGATAAT
ACAGATTGTTACTCGATGAACAGTAAACACGGTATTCGAAATCCTTCTAACCATCCATCCAGGCAGTGCCTCGTATACTCAAACCTCTTTTTGGAGGAAG
CCAAACCATGTGATCCTTTTCAACCCAAATACATATATTCAAGATGAAACTAACAAAAAGACAGTTGATAAAGAAACACGGAAGAGAGTAGAAAAAGGGT
ACCTGTTTCAAGTTCCCATCTTCTGAAAATGATCAGCAGTCAATAGATGTTTTAGTCCATTCTCCAGAAAAATGAGTGCCGCATGAATCCTTTCCGGTTT
ATAGTGAAACATTTTAGACGATGACCTTTAACATTCTAGAAAACGAAGAGAAAACCTAAAGTCACCTAATTTCAGCTTAAACATGTTCCAGCAAGATCAT
CACCATCCTGACATGTTCCGTTTTGTCCCTAGACCCGAGCACAAGGTATTGCGAGGCTAATTTAGCTCAATTCAGTTGTCAGAGCAATTGTGTGTGAAAA
GTGTGAACAACAGCATAGGAGACAATACAGCAAGTGTAGCGTGCCAATATGTGTCCAGCTTGATAGTTCACTATAATAAAGTTCTTTAATTCAAGTATTT
GAATAATATAGAATCTCAACCTTCAAGTTTAAAAGGTTTTGTATTTGCTATTATAATCGTTGCCACCCCCTCACCCCTCTCAAAAAGAGAGTTACTTTGT
AGCGCACTGTAAATCTCTTGATTGAAATAGTGGCATGTCATCTTATTTAATTCAGTTATTTCATAAGGTACAATTTGTTTAGCTGACAGCTCAGACATGC
TTCTATGCAACATGTTTATACCAATGCTGTATTTTGGCAATCCTGGATGTGGCATTACTTAACTGTGCTTTCATATTCAATAGTTCTGGCAAAGGGAGGC
TGCAAGCTTGAGACAACAACTGCACAACCTGCAAGAAAGTCATCGGTATGGCTAGAAAACCCTGCAAATTTTACAAATGTACAAGTCAGAAAATTTGCCT
AATGTAAAAGGATCAAACTACAGGATTTGTCAGCATGAGCACAAGTGCCTAACTTCCGAGTTAGATAACTCTGGAGGAAGGAAATCCTTCCTACTGTTTG
TTTCAAGAATATACAAAAATTCAAAACAACATTAGAATAGAACCAAAAGAAGAAACAATATTCACCATGTAGCAATACTCAATTCCTGGTTGTCTTATTT
ATAAAGGTATAAAACTAGGTTCTGACGAGACAATTATGAATCAACTACACCATGTTACAGATCATCCATAACTCTTGGTGATGCAAATTAAGGGCTGTAA
AGTGACAAGTATACCTCCACCAACTAATTTGTGCAGAGTGTATAAAATTGTAAAACAATGGATAAGAATGAATTTAGTATACAAGATAGCATACAATGCC
CAAGTTTAAGGAGACATACTGATTTACTGACTCCATAGGACAATCTTTTGCTTATGAGGACATCAGTTTTCATAAACAATATCAATGTAAAAGGAGGTAA
CAGTTTATGAATTTCCTATGCCAAGCCATTTATAAGTCAAAAAATGTACCTATTTAGCTTTTATGCATTCCAATTAACTTCCAAAGAAGAAGAGTACATA
CTAATGGAAAACAAATATGATATTATCATCTGTTTTGCCCGGTAGTTTCTTGAGTCTCTGATTTGTCCGCACTTCACTCCCAACCAACACAACATTAATA
CACATGGGCAGAGGAATATCAGGTTATCAGGTAAACTGAATACAGTTTGATTGACTGCAGTCAGTTGATGGGACAAGATCTTTCTGGATTGAGCGTCAAG
GAACTCCAAAATCTAGAAAATCAGCTAGAAATGAGCCTACGTTGCATCAGGACAAAAAAGGACCAACTCTTGATTGATGAAATTCATGAACTGAATCGAA
AGGTTCTGGAATATTCTGTTATTACTCTGCAATGAGAGGTCTCTGAGTGGATTTCAAGAAGCTGACTCTACTGGCTCATTTTCAGGGAAGTCTCATGCAA
CAAGACAAGATGGAACTATACAAAAAGGTCAACCTAATCCGTCAGGAAAACATTGATTTATACAAGAAGGTATTTGATAAATCGTGAATGAAAATTCACA
GGATATACACCCCATCCATTATTGACTGAGGTTTATTTCTCTACTCAGCTCTACGAGAAAGAGGTGACAAGTGAAGTCAACCGAGATTCATCAACTCTAA
ACTTTGCAGTTGTCGAGAACGTCAACATTCCTGTTCATCTTGAACTTAACACTCCACCGCAAGAAAATGATGTTGAGCAAACTGCACCTCCTAAACTGGG
GTAACCCTGCTTTTTTCTGCAAAGGAACTATTGATTCTGTTCTTAATTCTTTAGTGGCATATTGTTTACTACTAACTGAAAACTTGCAGATTACAACTAA
ATCCATGAAGGCATGCAGAATGGCACCGCTTATGTTCATTATTCTCAACGGTACGATTCAAAGCAACCAGAAACACTATTGGTTGTAAAGAGCTTAAACA
CATAGCAAGACTGACGCGGTATGCATGCCATGAGTGATTGATAATGGTAGTGTAAATCTATTTCAAATAAAATGTATTTTATTGAAGAATACAGCAAATG
ATTTGTATTATAACTATTTGACACATTCTGCAAGTTTGTTGACCAACGTGAGTACATGATAGCTACAAATCTTATATTGACAAAAAGATATGGTGTGTGG
AAAAAATTCAGGTGCTATTTTTTTCTATTTTGAGAATTGTATGCATAGCAGAAGAGCACAAATTGCATTCTGGTTTATCCGTGCAGGCATTCCAGAGTAT
CAGAATGGGGATCAACCTTTCTTCAGCTACATATGTAAATATACGGATGCTAGTTGCTCACTTAAAATTTTGTAGTGTTCAAAGGAATAACAACAGTAAG
ACGTTGACGCACCGAAAATAACCAGAAATAACGGATTGTACTATGACAAGACACACGACAGATACACTAGCTTAAGTTTGTACAGGAAAATATTTGGCCT
ATTCTTATGTCACAGTAAATTAAGATTATCACCTTAATCACAGAAATTCAAGGGGTAAACGATTATAGCACAAAACGATCCTGGACCCCAAGACCTGGGT
CTCTGAACGGCTGAACCTCCAATTATACCTCTATACATTTGGAATGTGTGTAGTCAAACTTTCGCTACTGGTATCTTTACAAATATTTACATTGAACACA
TCAAAATGATATACATGGATTCCTCTCGATATGTCTTTGAGGTTTGGGTTCTAAAAATGATATACAAATTATTAGGTTCTAAACTTAAATGATATATACA
CATCAAAATGATATACAAATTATTGGGTTCTAAACTTAAATGATGTATACACATCGAAATGATATACAAATTATTAGGTTCTAAAAATATACTATGGCAG
AATTAGGTGGTCATTTGAGTTTTGGAGGCCTTGTCAATGTCCCAAACATCACTTATTTGTGACTGGGAGAGCAATGAATTGAAGGAATGAGAGTAACCAT
ACGGTCATTGGTCAATTAAAAGGAACTTTAATATGCTATGACCACATCGACCTTTCGTAAAGTAAATAACTCAATTATAACCAAGTAATTGGGTGTCTAT
TGAATCCATACGGTTCTCAAAACAGTTCTAAACAAAGTGAATTTAAGCATCCTGAAAAGATATTTGGCAAATTTGCCATACCCTGAATTCACAGCATGTT
AACACAAATCATGTGCAAATCATTCAGATCATTGACAAACGCACGCATGCACACAAATCAACACCGTTTTCTCATGTATAGAACAGAAAAATTGAGAAAC
TTACGATTTTCTTGCAAGTTATGCAGTTGTTATCTCTAGCTTGCCGCCTCCACTTGCAGTAACTGAGACGCCGGCGCCGAGCTTGAGCTTGAAACCTAGA
CTACGGGGACAG
>Locus_14326_Transcript_28/49_Confidence_0.423_Length_2986
GTATTCTCATTTCCTCGCCGTATTTTCTCTCTCCAGCTCCAGACTCCAGAGTCCGGAGGAAGCAGAGAGAGAAGCTCGACGGGAAGGTAGAGAGAGATAT
AGTGCTCCTGATCCCTCTTTGAAATCCTCGATTGGTCAGTTCTGTTCCTGGATATACTGTTGCTGCAGATTGGATGCGTCTGCTTTCCTTTCGGGGAGTG
GTGTCTAGCTCTGTTGGTTCTTGCCTTCATAGTTAGTACTATGCCTTAATCTTGCTCCGCGATTGGTTTGATTCATGTTTTTCCTTTAATCTTGCTACCC
CAGGATTTTCCCCTTTGGTTTTCTTGGTTTGCTTCTCGGAAATCCGTGATTTTAACAGGATTTTTGGCTGGTGTTGCTCGATTTTTGTTCTTGACTGTGC
TTCCTTGGGATGAGCTTTTGCCTAATTCGACATTACAAGATGTGTTCTTTCTAGAAGTTAAATTGATCTTCTATGAGCCGGGCGCTGTATCGTGCATTAC
ATGGGGGTGGCGTACTGGTACTTTGGGCCGTAGAGTTGATGGAAATCTGCAGTCCCTTTTGTTGGATAAGGGGCTTTTTCCTTGGATAAGGAGGTTTGAA
GCTAATTTTGTGCGAATTGTGGCGGTTTCTGGATATCCTGGTTGAATTAGTGGTGTGATAGATTGTTCTAGAGAATAGCTAGATATATGTTATATTGTTA
GGACATCAAGAGTGTTGAATCAGGTATGTTAATGTGTGAACTTGTTGATGGGAGCTAAATGGTTGCTGACGTTCCCTATAATGACCTAATCAAACCTAAC
AAGCACTAAATTAATGATAAACAGCACTGATCAGTAAGCCTACTGAAGTAAAAACCAATCACAATTCATACAGTATGCAACAGTATAAAGTAGTGAAAGA
GTTCTTCAGAGCTCGAACTTTTGGGATGAAAATAATTTGGCTCAGGATAGTCCACTGAAAGGAGTTTAGGATATCTGTTTGCGCTCTGCCTGCAATATAT
GAAAATCTGAAGCTGATATGGTAGTAGAGTTGGGTACCCTGATTTCCGAGCCAAAAGAAGTTTAAGGTAGAATTTCTCAGGAATTATGTGGTAGTAAGAA
GCAATTTAAAAGGAACCTAATTAGGAAAAAAAGACATGCCTTGCTTGGTCATGAGAAAATATTATTGCTGTATAAAATAGGTATGCCACCTCCAGTCAGT
ACTTGATTAATTTCATTGAAAATGGAGGGCTGTAATAGATCCTTATCTATTAATGTGTTGTACCTAAACTTGGAAATCATCAGCTGAGGGCATTGTGCAT
GGTGCGTGGAGTACTAGCCCTCGGGAGATGCTTCTTAATTAGGCAGCGAGAAGGATAGAGATCGGTTTGACATATGTTCCATTTGTAGCTGTACTGGATA
TTTCTGCATGTTCCCCTGGAGCAGTTAGTTTCAGAATTATGATATTTCTACTTGTGGCAGGATTGTTCTGTTTCTATTATCTGTTCATATTTATGCTAGT
CTGTCTTTTATGTTGTAATATCTATTGTCTATTGCACTTCAAGTGACTATGTATAACCATATTCCAGTGATTCAGTGAATGATTATGAATTAAAGTAAGA
GGACATTTACATCAATTATCCTTGTTCTTTCTCACGTCATTATCAATTCGGTTGATTAAATTCTGGCAACTTGCCATGATGGTTACTTTAGCTGATTAAA
TGCTACTCGGAGCTAAGGATACCTTCTCGTAGTGCTCCCGCTTTTGTGAGAAATGGAGGATCATCCAGTCCATCCCATGTCGAAGTCCAACAATGGCTTC
TTGTCAGGAAATGGCTATCAGATGAAGCAATTTGGTCATAAAATCGACGATAGGGACTCGTCATCAGAGTCCGGTCGGTCTCACCAAGAAGCTTCCACAG
CGAGTGAGAACAGTATAAATGAACAGCACACCTCAACACAATCTGACAATGATGATGGTCATGGGAAGCATAATGAGGACATGATGAAGTCAGCAGTGTC
CTTGGGGGAACCAGGATCAGCCTTTTTGGCCCCAAAACTAGATTACAGCCCATCTTTTGCTTGTGTTCCTTATACTGCTGATGCTTTTTATGGTGGGGTC

TTGACAGGATATGCTCCACATGCCATTGTTCATCCCCAGCAAAATGATTCAGCAAACTCTCGGGTTCCGTTGCCTGTTGAACCTGTAGCAGAAGAGCCAA
TATATGTTAACGCAAAGCAATACCGTGCAATCCTTAGGAGGAGGCAGATACGTGCTAAACAGGAGGCTGAGAATAAGCTGGTGAAAGGTCGGAAGCCATA
CCTCCATGAATCTCGACACCGCCACGCAATGAAGCGAGCCCGTGGATCAGGGGGGCGGTTCCTCAACTCAAAGCAGCTCCAGGAGCAGAACCAGCAGCAG
CAGGCATCAGGTGGTTCAAGCTGCACAAAGGCCATCGGCAATAACACAAGCTCCCAAAGTGGTCCCAGTCACACGCCTCTGACACTTGCTTCCTCTGACA
CTGCAAGCGCTTCGAGGGCCAACCAGGAGCGCACCTGCTTCCCCTCGGCTGGCTTCCGTCCCACAATGAACTTCGGCGCGCGAGGTGGAGACGCAAAGCT
AGCTGTGAATGGCATACAGCAGCATGTTTCCTTCATCAGGTGAAGCAAGTGAAATGGGGCGCTGCCTGCGGTAACTCATCCTTGGCTTATGAAACTGTGA
ATGTGTAATGGACATGTAACTTCAGTCTCAGAATAACCAAACATATGGGAGTCCTCCTGAAGTTTCCTGTAGTTCAAAGAACTGATAGGTTATGGCCTTA
TAGGTTTGCTTCTGGAGACTGAAATCCGTGCTTGATATACTATGTTGTACTGAGTTCTGAGGTGAAAGACATCGGGAGAGTATCAGGTGCAAATGGCACA
AATGTTGAAAACGGTGCAAATTCATCAAAATCTGTACCTAAAAGACTTCAATAAATTTTAAACTTTGCATAGCTCTAGGTGCATTT
>Locus_16183_Transcript_46/55_Confidence_0.450_Length_2770
CAGAGTCCGGAGGAAGCAGAGAGAGAAGCTCGACGGGAAGGTAGAGAGAGGTATAGTGCTCCTGATCCCTCTTTGAAATCCTCGATTGGTCAGTTCTGTT
CCTGGATATACTGTTGCTGCAGATTGGATGCGTCTGCTTTCCTTTCGGGGAGTGGTGTCTAGCTCTGTTGGTTCTTGCCTTCATAGTTAGTACTATGCCT
TAATCTTGCTCCGCGATTGGTTTGATTCATGTTTTTCCTTTAATCTTGCTACCCCAGGATTTTCCCCTTTGGTTTTCTTGGTTTGCTTCTCGGAAATCCG
TGATTTTAACAGGATTTTTGGCTGGTGTTGCTCGATTTTTGTTCTTGACTGTGCTTCCTTGGGATGAGCTTTTGCCTAATTCGACATTACAAGATGTGTT
CTTTCTAGAAGTTAAATTGATCTTCTATGAGCCGGGCGCTGTATCGTGCATTACATGGGGGTGGCGTACTGGTACTTTGGGCCGTAGAGTTGATGGAAAT
CTGCAGTCCCTTTTGTTGGATAAGGGGCTTTTTCCTTGGATAAGGAGGTTTGAAGCTAATTTTGTGCGAATTGTGGCGGTTTCTGGATATCCTGGTTGAA
AGTGGTGTGATAGATTGTTCTAGAGAATAGGAAGATATATGTTTTATTGTTAGAACATCAAGAGAGTGTTGAATCAGGTATGTCAATGTGTGAACTTATT
GATGGGAGCTAAATGCTTGCTGACGTATCTTCGCTATAATGACCTAATCAAACCTAACAAGCACTAAATACTGATCAATTCTCAGTTATACAAAGATTTT
CCAGAGAGTTCTTCAGAGCTCGAACTTCTGGGATGAAAATAGTTTGGCTCAAGATAGTCCACTGGAAGGAGTTTAGGATATCTGTTTGAGCTCTGCCTGC
AATATATGGAAATCTGAAGGTGATATGGAATATATGGAAATCTGAAGGGGATATGGTAGTAGAGTTGGGGACCCTGATCCCCGAGCCAAAAGAAGTTCGG
TAGAATTTCTCAGGAATATTGTGGTAGTAAGAAGCAAGTTAAAAGGAACCTAATTAGGAAAAAAGACATGCCTTGCTCGCTCATGAGAATATAAAACAGG
TATGCCACCTCCAGTCAGTACTTGATTAATTTCATTGAAAATGGAGGGCTGTAATAGATCCTTATCTATTAATGTGTTGTACCTAAACTTGGAAATCATC
AGCTGAGGGCATTGTGCATGGTGCGTGGAGTACTAGCCCTCGGGAGATGCTTCTTAATTAGGCAGCGAGAAGGATAGAGATCGGTTTGACATATGTTCCA
TTTGTAGCTGTACTGGATATTTCTGCATGTTCCCCTGGAGCAGTTAGTTTCAGAATTATGATATTTCTACTTGTGGCAGGATTGTTCTGTTTCTATTATC
TGTTCATATTTATGCTAGTCTGTCTTTTATGTTGTAATATCTATTGTCTATTGCACTTCAAGTGACTATGCATAACCATATTCCAGTGATTTAGTGAATG
GTTATGAATTAAAGTAAGATGACGTTTTCATCAATTATCCTTGTTCTTTATCACGTCATTATCAATTCAGCTGATTAAATTCTGGCATCTTGTCATGATG
GTTACTTTAGCTGATTTAATGTTTCTCAGAGCTAAGGATACCATCTCATAGTGCTCACTCTTTTGTGTGGGAAATGGAGGATCCCATGCCGAAGTCCAACAA
TGGCTTCTTGTCAGGAAATGGCTATCAGATGAAGCAATTTGGTCATAAAATCCACGATAGGGACTCGTCATCAGAGTCCGGTCGGTCTCACCAAGAAGCT
TCCACAGCGAGTGAGAACAGTATAAATGAACAGCACACCTCAACACAATCTGACAATGATGATGGTCATGGGAAGCATAATCAGGACACGATGAAGTCAG
CAGTGTCCTTGGGGGAACCAGGATCAGCCTTTTTGGCCCCAAAACTAGATTACAGCCCATCTTTTGCTTGTGTTCCTTATACTGCTGATGCTTTTTATGG
TGGGATCTTGACAGGATATGCTCCACATGCCATTGTTCATCCCCAGCAAAATGATTCAGCAAACTCTCGGGTTCCGTTGCCTGTTGAACCTGTAGCAGAA
GAGCCAATATATGTTAACGCAAAGCAATACCATGCAATCCTTAGGAGGAGGCAGATACGTGCTAAACAGGAGGCTGAGAATAAGCTGGTGAAAGGTCGGA
AGCCATACCTCCATGAATCTCGACACCGCCACGCAATGAAGCGAGCCCGTGGATCAGGGGGGCGGTTCCTCAACTCAAAGCAGCTCCAGGAGCAAAACCA
GCAGCATCAGGCATCAGGTGGTTCAAGCTGCACAAATGCCATCGGCAATAACACAAGCTCCCAAAGCGGCCCCACTCACACGCCTTCGACACCTGCTTCC
TCTGACACCGCAAGCGCTTCGAGGGCCAACCAGGACCACATCTGCTTCCCCTCCGCTGGCTTCCACCCCGCAATGAACATCAGCGATCATGGTGGAGGAG
ACACAAAGCTGTCTGCGAACGGCATGCAGCAACGTGTTTCCTTCATCAGGTGAAGAAAAGTGGCACTGCCTGTGGCAACTCATCCTTGGCTTATGAAACT
ATGGATATGTAATGGGCATGTAGGTTCGGTCTCAGAATAACCAAACATATGTGAGACCTTTTTAAGTTTCCTTTAGTTCAAAGAATGATAGGTTATGACC
TTATGGCTTTGTTTCTGGAGACTGAAATCCGTGCTTGATATACTATGTTGTACTGAGTTCTGAGGTGAAA
>comp64193_c0_seq1
GCTTTGCCAGCGAGCTCTTTCACCTGGCTGGTGGCGTGGTTCTCGCGCTCGTGTTCAAGACGGGGTTATGGGGCACTGACGTGTCAGTGGGCTGCGCGCT
GATTGACATGTTTGCGAAGAATGGGGACCTGGTCGCGGCGCGCCGGGTGTTCGATGGATTGGTTGAGAGGACGGTGGTCGTCTGGACACTGCTAATTACA
CGCTATGTGCAGGGCGGCTACGCGGATATGGCTGTAGAGCTTTTTCTTGACATGCCTGAGAATGGTTTTTGGCCGGATCAGTACACCATGAGCAGCATGT
TATCAGCTTGTATAGAGTTGGGATCATTCAGGTTGGGGCAGCAACTGCACTCTCTAGCACTCAGGCTTGGGCTGGTATCAGATAGTTGTGTGAGCTGTGG
GCTTGTGGACATGTATGCAAAGTCTAATATTGGGCAGCCGATGCAAAATGCGAGGAAAGTCTTTGAACGGATGCCCAAGCATAATGTGATGTCATGGACG
GCACTCTTCTCAGGGTATGTGCAATGTGGATCACAGGACAGCAAAGTGATGGCGCTATTTTGTGTGATGTTGAATGAAGGCATCAGACCAAACCATATCA
CGTATTCTAGTATACTTAAGGCATGTGCAAACATTTGCGATCAAGATTCAGGCAGACAAATTCATGCCCATTGCATGAAATCCGGCCTAGCTCCTGTAAA
TGTTGTTGGGAATGCTCTTGTTAGCATGTATGCTGAATCTGGTTGCATGGAGGAGGCTAGAAGGGCGTTTGACCAGCTATATGAAAAGAACATGGTTTCC
TTCAGTTGCGATTTTGATGGAACAGTGAGGAGCGGTGCTTCTCAAGATTATCAGATTGAGAGAATGGATGTAGGAATCAGCACTTTCACATTTGCTAGTC
TTATTAGTGCAGCAGCCAGTATAGGGTTGCTAACCAAAGGTCAGCAGTTGCATGCCCTCTCGCTTAAGGCTGGATTTGGGTCCGATAGAGGCATATGCAA
CTCCCTTGTGTCTATGTATTCTAGGTGTGGATACCTAGAAGATGCTTGTCGAGCCTTTGATGAAATGAATGACCATAATGTGATCTCATGGACTTCAATG
ATTAGTGGCTTGGCCAAACATGGATATGCAGAACGGGCGCTGGAATTGTTTCACTATATGATCTCAGCTTGCTTGAAACCAAATGATGTCACATACATTG
CTGTTTTATCTGCATGCAGTCATGCTGGTCTAGTTAAAGAAGGGAAGGAGCACTTCAGACTGATGCAGAAGGATCACGGGCTCGTACCAAGGATGGAGCA
TTACACTTGCATGGTTGATCTGCTTGGGCGATCAGGCCTTGTGGAAGAAGCCCTAGATTTCATTAACAAAATGCCTTGTAAAGCAGATGCATTGGTCTGG
AAGACACTTCTTGGTGCTTGTAAGACTCATGACAATATGGAGATTGGTGAAATTGCCGCTAACCATGTCATAGAGCTCGAGCCACAA
>Locus_9727_Transcript_19/27_Confidence_0.377_Length_1523
GCCATGCATGTGCAGTGGTGCCCGGGCATGCGGTTCAAGATGGCGTTCGAGACCGAGGACTCGTCGCGGATCAGCTGGTTCATGGGCACCATCGCCGGCG
TCCAGACGGCCGACCCCGTCCGGTGGCCGCATTCGCCCTGGCGACTCCTTCAGGTGACCTGGGACGAGCCGGAGCTCCTGCAGAACGTGAAGCGCGTGTG
CCCGTGGCTGGTCGAGATGGTGTCGACCATGCCCAACCTCCACCTGCCGTCCTTCTCGCCGCCCCGTAAGAAGCCGCGCATCCCGCCCTACACGGACTTC
CCCTTCGAGGGCCAGCTCTTCCCGCCCAACCCGCTGCAACTGGTGCCCCACCCGCACCCGCACCACGACCAAAACCACGGCTTCGCCCCCTCCTTCCCCT
TCCCGTACAGCAGCGCTACTGCAGGCATACAGGGAGCCAGGCATGCGCAATTCGCTCCATCCTTCTCGGATCTCCACATCGGCAACCTGCAGCAGAGCCT
TCTGTTCTCCGGCATCCGCAACCCTGACCACCACCACGCCCCTCGCTCGCCGAGGATCAGCACCGACCTGACAATCGGCAGCCCGTCCGCCGGCCAAGAC
GCGCCGCCGTGCTCCCCATCGACTGCCACCAAGAAGCCCGACGAGGTCAAGCCGCCGGGGCTGGTGCTCTTCGGACGGGCGATACTGACGGAGGAGCAGA
TCAAAGCGAACAGTTCCGGTGCTCTGACCTCGCCGGGGGCTACTGGCAGCGGCCCTTCCAAGTCCGACTGCGACGCCGAGAAAGCACCCAACACATCTGA
AGGTTCCGGCTCCGGTGTCATCCAGGGCAGTCCTACCAAATACAACTCGTCCTCCTGGAGATTACAGTGGTTGGGAGACAATGGCAGCCAGGCATCATCT
GAGTTTGGGCTGGAGCCCGGCCAGTGCAAGGTGTTCGTGGAGTCGGACGCCATAGGCCGGAACCTTGATCTCTCGGCGCTGGGATCGTGCGAGGAGCTGT
ACGCCCGCTTGTCTGACATGTTTTGCGTTGACAACGCTGAGCTGAGGAGCCATATGCTATACCGCACCGCCGCTGGCGAGGTCAAGCACGTCGGCGATGA
GCCTTTCAGTGCGTTTGTGAAATCAGTGCGGAGGATCATGATACTGACAGATGCCGGCAGTGACAACATAGGGAGTTAGGAATGAAGCGATCGGCCGAAT
AACTTAGGGCACTACTCCTAGGCAGCAATAAGCCGAACTACATGCATGTGTACAGCCGAACGAGCTGCGGTTTCTTCTTCGATCAGGCCTAGAGAATTCA
GTCAGGAAATTAAGCTGACGCAGTGTTCAGTGCTTCAGTTAGGTTGTTTTCCCACCCTTTTTCATTTAGTTCAGTAAAGCTCTTTTAGTCATGCAAACAA
ATTGTAATCTTGATCATCAAACGCATTTCTTAAATCTTGTTTCCGTTTTGGATTTCAAACCTTGCGCTGGTTCAGAAAACCGGCTGTTCCTGGCCTGACA
GCCTGGCCAGAGAGACGAGCACC
>Locus_2827_Transcript_160/166_Confidence_0.116_Length_2750
GAGGAGACCTTGACAGAGTTCCTCTCAAAGGCTACAGGGACTGCTGTTGATTGGGTCCAGATGCCTGGGATGAAGCCTGGTCCGGATTTGTTTGGTATTG
TGTCTGTTTCACATGGTTGCCGAGGTGTTGCTGCCCGTGCCTGTGGTTTGGTGAATCTAGAACCAACAAAGGTCATAGAGATCTTGAAAGATCGTCCATC
TTGGTTCCGTGATTGTCGAAGCCTTGAGGTGTTCACAATGTTTCCAGCTGGAAATGGGGGAACAATTGAACTTGTCTACATGCAGATGTATGCCCCAACA
ACTTTAGTCCCTGCACGTGACTTTTGGACGTTACGATACACGACAACAATGGAAGATGGCAGCCTTGTGGTCTGTGAGAGATCCTTGAGTGGTTCAGGGG
GTGGTCCAAGTGCAGCCTCTGCGCAACAATTTGTAAGAGCTGAGATGCTTCCAAGTGGGTATTTAGTTCGCCCATGCGAAGGCGGGGGCTCGATTGTGCA
TATAGTGGACCATCTGGATCTCGAGGCGTGGAGTGTACCTGAAGTGCTTCGACCACTGTATGAGTCTTCTAGAGTAGTTGCTCAGAAAATGACTACTGTG

GCACTGCGCCACCTTAGACAAATTGCTCAAGAAACAAGTGGGGAAGTAGTGTACGCCTTGGGCAGGCAACCTGCAGTCCTACGGACATTTAGTCAAAGGC
TGAGCAGGGGCTTTAATGATGGCATTAGCGGTTTCAATGATGATGGCTGGTCTATAATGGGGGGAGATGGTATTGAAGACGTAGTTGTTGCTTGCAACTC
AACTAAGAAAATTAGGAACAACAGCAATGCTGGAGTTCCGTTTGGAGCCCTTGGAGGTATTATATGCGCGAAGGCATCCATGTTACTGCAGAGTGTTCCA
CCAGCAGTATTGGTTCGATTTCTGAGGGAGCATAGATCTGAATGGGCCGATTACAATATTGATGCATATTTGGCTTCAGCACTGAAGGCCAGTGCATGCT
CGCTTCCTGGAATGCGGCCCATGAGATTTTCTGGGGGGCAGATGATCCTGCCACTTGCTCACACAGTGGAGAATGAGGAGATTCTTGAAGTTGTCCGCCT
TGAAGGACAATCTCTTACTCACGATGAGGCTCTTATTTCAAGGGATATCCACCTGCTTCAGCTTTGCACTGGAATAGACGAGAAATCTGTGGGGTCCTCC
TTCCAGCTCGTGTTTGCACCAATTGATGAACATTTTCCAGATGATGCTCCATTGATTTCTTCTGGCTTTCGTGTCATACCACTTGATATGAAAATAGTTA
AGCATCGCCAACAATAGTACATTTACCTTTCATTTTTACCCTGTCTTAAGACACTGTTTGCTTCCTCATATTGCAATGATTGGTAGAGGATTGTGGGCGT
CTATTAGATTTTGCAGTCAGCTTTAATAGTTCTCATGTATTGTAACTTAATTCAGATCAAGTGTAGCGTTGCTCACATGACTCTTCGTATCCCTATAAAA
TGATAGTTCATTTGCCACAAATTGTTTGTTGTTTCAGGTCTTTTGTTTGATTATGTGTCTTGTATGTCTTCTAAAAAAATACTTTCTGTTCTAGCTAACC
ATAATACGCTACTTAGTAGTTTGGACATCTGTTGCTAATCATTAGGTTCTCTGTAATTTATTTCCGTCTCTACTTCAGTTTTTTTACTATTATATCTCAT
GCAGGATGGCGTATCCTCTGGTAGGACGTTGGATATGGCGTATCCTCTGGTAGGACGTTGGATTTGGCTTCTAGTCTTGATGTTGGTTCTGCCATGCCCC
ACGCCTCAGGGGATGCATCTCGAGATGATTGTAATTTGAGATCTGTGCTGACCATCGCCTTTCAATTCCCTTACGAGATGCAGCTCCAAGACAGTGTTGC
AACTATGGCCCGTCAGTATGTTCGCAGTGTTGTTTCTGCTGTGCAAAGAGTGTCGATGGCTATCTCTCCCTCCCAATCTGGTATAAATGCTGGGCAGAGG
ATGCTTTCTGGCTTCCCTGAGGCAGCCACACTTGCTCGCTGGGTTTGCCAGAGCTATCATTACCATCTAGGGGTAGAATTACTTAACCAGTCAGATGAAG
CTGGCGAAGCATTGTTGAAGATGCTCTGGCATCATCCAGATGCTATTTTGTGCTGCTCTTTTAAGGAAAAACCTATGTTTACATTTGCCAACAAGGCAGG
GCTTGACATGTTAGAAACATCCCTTATTGCCTTACAAGACCTCACATTAGACAAGATCTTTGATGAGTCTGGAAGAAAAGCGTTATTCTCAGATATCTCT
AAATTGATGGAGCAGGGCTACGTGTACCTGCCATCGGGCGTGTGCATGTCAGGAATGGGTCGCCATGTTTCGTTCGACCAAGCTGTGGCGTGGAAGGTCC
TTGGTGAAGACAGCAACGTCCACTGCCTGGCCTTCTGCTTCGTGAACTGGTCCTTCGTGTGAGCCTTTGCCATCCTCCGAATGCCGACTTGATCTGTGAC
GAGGAGATCCATTTTTGTATTTATCTGGAGAAGGAAAATCTTCCACTGTGACAAGGCTTATGTTGCACTAGCTAGCTCTAGGTTTGGGAATCCAGTGTAA
AATCTGATGTCTTTCCATTTGGCTACCAAGCTGTGCTCTGGTTGTTTATCTTGTTTGGGACGATGCTAAGTTGCTAACATTGGGTTCCCAACTTACGCGT
CGAAGTGTGTTTCATCGCATCAATCTTCAATCGCAGCTGAATTTTCGTTT
>comp81084_c0_seq1
CGCACAACTTTGTGTGTTTAAATGCTCGTAGTACTTACAACAGGTGCGTTGGTTGCTACTTTTAACCGGAACAAGTAGCAATCATGCACCTACAGTAAAC
AAACTTGTCCAAATTGATTCTTCGTTGTCCAAAATCCGTGAATTGGACGCACGTCTGTAAAGGTACAAACAATAAATAACAGACCATATAAAGCTATTTA
AAAGCCTAAATCCTCAAATTCATGAAAAACCGAAACTCAAAATTCTTCGATGAATTTCATCACGATGCTCTTATCTACCGGTCACCCTTGCTTGCGGCTT
TGAATGCACCAAGGTTCCTTTTTAGAGACTTGACAGCAGACAAAATCTGCAGCTTCTCTTTGGATGCCCCATCAGGTTTCTGATCATCGTTCTTACTGGC
TGCCTCATCAGGTGGCAACCTACCTCCCGCAGACGATCCCTCCACAGGAAGTTCCCTGTCTGCATTGCTGGCATGTGCCCCCTGCTTCCCAGCTTGCTTT
CGCATGAAGGCCGCACTCCTCTTGTACTGGGGATCAGTAGGATCCAATGCGAACAAATGAGATGTGAACATTTCTGAGAACCTCTCATCTTTGCTAAGAT
CAATATCAGGAAGTTTGTCCTCGACAGATTCCTCTTTACCCTTCTTACCCTTGTTACCCTTTTTGTGTTTCAAGTTATAGCCCTTTGCACCATTGGCAGT
ATCCTGGTCAGCAGCAACCAAGAGCTCCAACTCTTCTTTCGTTGCTTCTGGCTCCAAATGTTCCTCTGTTGGTTTGTCCTTTCCTTTCCCCTTTGCTTTA
TCTTTAGTCTTCGCCTTTTGTTTCTTGATTTTAATTGGCTTAGTTTCTTCATCAGACAGTTCCTCAGCGAAGAAATCATCATCGTCATCGGGCTCATCTT
CGCTGCTATAGTCATCGTCATCATCCTTCAACCCCCTCTTCGTAGCCTTCCTTTTCTCTTTCATTTTCTCTTGGTGCCTCTCCCAGACAGTTTTCTCCTC
ATTGGTCTTTCTCTCTAGGATACGTTTACTGAGATCCTCAAGCTCTGTGTTGAATGTAATCTCCATATCCTGGCCACCAGCTTGTTCCTCTTCAGATTTG
TCGCCTTCCAGTAAAAGAGCCAACCTCTCCTCTTTTGTTAGCTTTCGTTTGGATCCCCCATTTGGTAGAGACTCATTATCAGGATTGTCTACACCGTCAT
CATCTGACGCACTGTCATCACTTGCTAAATACATGTCAAGCTCATCTAACTGATCATCAGTGAACTTTCGCCTCAAGACCTTCTTACGTTCTGGTTCATC
ATCATCCCAAGTTAGCTTAACTCTGCTATGTTGCAGAGCACGAGTCTCAAAATCAGGCTCCTTGTAACTCGGAGGTGCCTCTGTAGCAATGTCTCGAGCA
GGATGTTTGAACTCCATAGAATCAGGTATAAACTGCAAATCAAATACATTTGCTGTTTTCAAGAACTCAGTACCATCAAGATTCATGTACAGGTGATTTG
CTGTTGCACTAGAATCACAGACCACAACAGCATAATAATACCTCAGTCTGTTTAGCTCATAAGTACGCAACTTGTTATTCTCTTTGTCGGAGTCAACTTC
TTCGTCGTCGTCATCATCAGTGTTGTCTTCATCATCATCGTTGCTATCATCGTCGTTGTCATCATCATTATCATCAATATTGGCATTAACAAGAGCAGAT
GGGCCCTGAGTTGACTCAATTTCCATGCACTTCAGTCCAAATTCTGATGGATATATTGAGACTGATAAAACTCGCCCACCTTTTGGGACGCAAGATGTCA
TCACCATATACAGGTCAACTGCCTTGATATGATCCCAGTCCATATTAACAACAGCAAGCCTGTGAGTTTCCTTGTCAATCATAGGCGTATCATCGTGCCG
GCCCATTAAGTAATGAGCAATGTCACTGCCGACAGAGTACTCGTCATCATCAACGTCTTCATCCTCGTCGTCGTCGCTACTAGAACTCTCTTCTTCCTCC
TGCTCATCCTCTTCATCCACGTCCTCGTCCTCCTCTTCTTCAACTAACTTCTCTTTCTCCTTCTTTGCCTTCTCTTTAGTCTCATCCTCCTCCTCGTCTT
GGTTGAGATAGTAACGGAGCATGGGGTTCTCCCTGCCGCTCTTCTTCCGTCGCCTGCCGCGCTTGTCCACCGGCGCCGCCGAGGAGGAGAACCTCGGGTC
CGTCATCATGCTGCTGAACCTCGAGTCCAGCGCCACCTTCGCCTCCTTCCGCCGCATCGACCGGAACCGCGGGTCCATCCGCGCGGCCGCGAACCGCTCG
TCCTGCATCGCCCGCTTCATCCTCTCGTCCCGCCCCTCCACCGCCTCCGCTTCAGCCGCCTTGCCCTCGTGCTTCCCCTCCTTCTTGGGCTTCTTGCCGT
GCTGCCGCTTCTCGTCCTCCTCGCGCCCCTCCTTGTGCTTCCGCTTCTTGCGCTCGGCCTCGGGGCGAGGGGCTTCCTCGGTGGCCGCCTGACGCTCTTG
ATTCTTCTTCTTGTGCTTCCTCTCTTCCTTATCGGACCTCGATTTCTTGGCCTTCTTGAGGCGCGCGGGCTCGTCGGAGCCGGTCGGCGG
>Locus_3441_Transcript_36/417_Confidence_0.042_Length_1648
TGTAAATCTCTTGATTGAAATAGTGGCATGTCATCTTATTTAATTCAGTTATTTCATAAGGTACAATTTGTTTAGCTGACAGCTCAGACATGCTTCTATG
CAACATGTTTATACCAATGCTGTATTTTGGCAATCCTGGATGTGGCATTACTTAACTGTGCTTTCATATTCAATAGTTCTGGCAAAGGGAGGCTGCAAGC
TTGAGACAACAACTGCACAACCTGCAAGAAAGTCATCGGTATGGCTAGAAAACCCTGCAAATTTTACAAATGTACAAGTCAGAAAATTTGCCTAATGTAA
AAGGATCAAACTACAGGATTTGTCAGCATGAGCACAAGTGCCTAACTTCCGAGTTAGATAACTCTGGAGGAAGGAAATCCTTCCTACTGTTTGTTTCAAG
AATATACAAAAATTCAAAACAACATTAGAATAGAACCAAAAGAAGAAACAATATTCACCATGTAGCAATACTCAATTCCTGGTTGTCTTATTTATAAAGG
TATAAAACTAGGTTCTGACGAGACAATTATGAATCAACTACACCATGTTACAGATCATCCATAACTCTTGGTGATGCAAATTAAGGGCTGTAAAGTGACA
AGTATACCTCCACCAACTAATTTGTGCAGAGTGTATAAAATTGTAAAACAATGGATAAGAATGAATTTAGTATACAAGATAGCATACAATGCCCAAGTTT
AAGGAGACATACTGATTTACTGACTCCATAGGACAATCTTTTGCTTATGAGGACATCAGTTTTCATAAACAATATCAATGTAAAAGGAGGTAACAGTTTA
TGAATTTCCTATGCCAAGCCATTTATAAGTCAAAAAATGTACCTATTTAGCTTTTATGCATTCCAATTAACTTCCAAAGAAGAAGAGTACATACTAATGG
AAAACAAATATGATATTATCATCTGTTTTGCCCGGTAGTTTCTTGAGTCTCTGATTTGTCCGCACTTCACTCCCAACCAACACAACATTAATACACATGG
GCAGAGGAATATCAGGTTATCAGGTAAACTGAATACAGTTTGATTGACTGCAGGCAGTTGATGGGACAAGATCTTTCTGGATTGGGCGTCAAGGAACTCC
AAAATCTAGAAAATCAGCTAGAAATGAGCCTACGTTGCATCCGGACAAAAAAGGACCAACTCTTGATTGATGAAATTCACGAACTGAATCGAAAGGGAAG
TCTCGTCCAACAAGACAACATGGAACTATACAAAAAGGTCAACCTAATCCGTCAGGAAAACATTGATTTATACAAGAAGGTATTTGATAAATCGTGAATG
AAAATTCACAGGATATACACCCCATCCATTATTGACTGAGGTTTATTTCTCTACTCAGCTCTACGAGAAAGAGGTGACAAGTGAAGTCAACCGAGATTCA
TCAACTCTAAACTTTGCAGTTGTCGAGAACGTCAACATTCCTGTTCATCTTGAACTTAACACTCCACCGCAAGAAAATGATGTTGAGCAAACTGCACCTC
CTAAACTGGGGTAACCCTGCTTTTTTCTGCAAAGGAACTATTGATTCTGTTCTTAATTCTTTAGTGGCATATTGTTTACTACTAACTGAAAACTTGCAGA
TTACAACTAAATCCATGAAGGCATGCAGCATGGCACCGCTTATGTTCA
>Locus_13957_Transcript_52/58_Confidence_0.153_Length_1038
AAAAAAAATTTTGCTTTACCTTCTCTGTTGATTTCCAAAGGGTTGAGTACCGACTCGATTCTCTTCTTTGTTGTTTTTCTTCTTTCTGTTTCAAAATTCA
GGGCTTATGATAAGGCCGCGATCAAATCAATGGTCGAGAAGCTGTGACGAACTTCGAGCCAAGCACGTATGATGGGGAGCTGCTGACTGAAGTTGCTGAT
GAAGGTGCTGATGTTGATCTGAACTTGAGCATATCTCAACCAGCTTCTCAAAGCCCGAAAATGGACAAGAACTCCCTTGGTCTGCAACTCCACCATGGAT
CGTTTGAAGGCTACGAATTGAAAAGAACAAAGATTGATGCTCCCTCTGAACTCACGGGCCGACCTCATCGGTTCCCTCTTCTGACCAAGCATCCACCAAT
CTGGCATGCCCAATCTCATCCCTTCTATTCAAATAATGAGAATGCATCAAGAGATCTTAACAGGAGGCCTGATCAGGGAGGCACTGTTCCTAGATGGGCG
TGGAAGGTGACCGCCCCTCCTCCCACCCAACCATTGCCGCTGTTCTCGTCATCGTCGTCGTCGTCCGCTGCAGCATCATCAGGATTCTCCAATACCGCCA
CGACAGCTGCCCCCACCGCCCCGTCGTCCTCCTCCCTCCGGTTCGACCCGCCGCCGCCGTCGAGCCGCCACCGATGAACATTGAGAAGCCACGCTGTAAA
TTTGCCGGGAAGCCGGCATTTTTTCCCTCTCCAGGCGTTGCAACTTTTTCGGTTTTGCGCCGGGGTGGTTTCTTAATTGTAGTGGATTGGATTCATAACT
GTATTTGCATACTGCCCCATGTGAAATGCTTCGTTCTCACTCTCTCTTTACACACTCTGCAACGGATAGATGTTCTTGTTGATGGAAATTAGCTAACTTC

ACCGTCTGAGATGATCTATACATTGTTGTAGAGAATCGAATCATTGTTGAAATTAGTTACAGAATTATAGATATCATAAGGCACCCTCTACTTGTGGACA
AAGTTCAGGAAGATTATTCCGGTTCTTTTCTTGGCTGT
>Locus_19994_Transcript_19/149_Confidence_0.053_Length_550
CTTTATAGTCGCCATAGACTAGCCATCCCATGTTGAATATAACTCCAAACATATATAATTGTACATTTTGCCAGTACAAACTATCATTGTTCTTCTTCAT
CAAGTACTCTGTGTAGACACCAGCTAGAGCTGAAAGACAAGCCGAAAGTATCCCGAGCATGTAACCCTGAAATGGTGCTGAGAAAAGAGAATCACACGGT
GCATCTCCACATCCTTTCACCTGGCTAGTAGTTGTACCAACAGCCAGTAAGATGATTGCCATCCATTGCAGATTTGATAGCTTCCTTTTTAGTACAAGCC
TAAACAATATTCCTGTTGTGACAATTTTCAGGTTTCCCATTATCTGGTAGGTAGATGGATCAACATAGGTCAAGGTTGCGAACTGGACATTGTTGTGGAT
GAGGTATATTACTGAAGGAACAAGATATAGCCGCACACTCCTCCATTCTTTTGTCATCCTTGGTGGCGATGAAGACTGGAATTCCTTCCAAAGGAAGAAG
CTTGAGACAGACAACTGCACTGAGAACTAGGTAATGTTACAGAAAATTGA
>Locus_5523_Transcript_57/107_Confidence_0.124_Length_734
TCTTTGTTAGGGAAGCACCAAGCACCTCAAGGCTGCGAACCAATCTCAAAAGGGGATACAGGATAGGATAGCTTCCTTCCTTCCTGTAAGGAGGATCAGA
AAGAGAGAGAGATAGGGAGGCCAAATCAGGCTTCTTCAATTAGTTAGAGTAGAGAGAGATCTGTGCTACCTGTGGGGGAGAAGATGGGAAGAGGGAAGATAG
TGATCAAGAGGATTGACAACACGACAAGCCGGCAGGTGACGTTCTCCAAGCGCCGGAGCGGGTTGTTCAAGAAGGCCAAGGAGCTCGCCATCCTTTGCGA
CGCCGAGATCGGACTCATCGTCTTCTCCAGCACCGGTCGCCTCTATGACTTCTCCAACACCAGCATGAAGTCTGTGATCGAAAGATACAATGAAGCAAAA
GAGGATCATCACCTGACCATGAGCGCAAGTGCGGAGGCTAAGTTTTGGCAAAGGGAGGCAGGAAGCTTGAGGCAGCAACTGCATAACTTGCAAAGAGCAC
CATCGGTCAGTTGTTGGGTCAGCAGCTTTCTGGCCTTGATGTAAAAGGCTTGAAAGATTTGGAGAATAAGCTGGAGATGAGCCTAAGAAATATTTGTCTG
AAGAAGGACCAACTTATGATTGATCAAATTCAAGAATTAAACAGGAAGGGAAGCCTCCTGCACCATGAAAACATAGAACTATACAACAAAGTCAACCTTG
TTCAACAAGAGAACATCGAATTACAGAAAAAGGT
>Locus_1016_Transcript_990/2070_Confidence_0.002_Length_4649
GTGGAGGCGGCAAGCTAGAGATAACAACTGCATAACTTGCAAGAAAATCGTAAGTTTCTCAATTTTTCTGTTCTATACATGAGAAAACGGTGTTGATTTG
TGTGCATGCGTGCGTTTGTCAATGATCTGAATGATTTGCACATGATTTGTGTTAACATGCTGTGAATTCAGGGTATGGCAAATTTGCCAAATATCTTTTC
AGGATGCTTAAATTCACTTTGTTTAGAACTGTTTTGAGAACCGTATGGATTCAATAGACACCCAATTACTTGGTTATAATTGAGTTATTTACTTTACGAA
AGGTCGATGTGGTCATAGCATATTAAAGTTCCTTTTAATTGACCAATGACCGTATGGTTACTCTCATTCCTTCAATTCATTGCTCTCCCAGTCACAAATA
AGTGATGTTTGGGACATTGACAAGGCCTCCAAAACTCAAATGACCACCTAATTCTGCCATAGTATATTTTTAGAACCTAATAATTTGTATATCATTTCGA
TGTGTATACATCATTTAAGTTTAGAACCCAATAATTTGTATATCATTTTGATGTGTATATATCATTTAAGTTTAGAACCTAATAATTTGTATATCATTTT
TAGAACCCAAACCTCAAAGACATATCGAGAGGAATCCATGTATATCATTTTGATGTGTTCAATGTAAATATTTGTAAAGATACCAGTAGCGAAAGTTTGA
CTACACACATTCCAAATGTATAGAGGTATAATTGGAGGTTCAGCCGTTCAGAGACCCAGGTCTTGGGGTCCAGGATCGTTTTGTGCTATAATCGTTTACC
CCTTGAATTTCTGTGATTAAGGTGATAATCTTAATTTACTGTGACATAAGAATAGGCCAAATATTTTCCTGTACAAACTTAAGCTAGTGTATCTGTCGTG
TGTCTTGTCATAGTACAATCCGTTATTTCTGGTTATTTTCGGTGCGTCAACGTCTTACTGTTGTTATTCCTTTGAACACTACAAAATTTTAAGTGAGCAA
CTAGCATCCGTATATTTACATATGTAGCTGAAGAAAGGTTGATCCCCATTCTGATACTCTGGAATGCCTGCACGGATAAACCAGAATGCAATTTGTGCTC
TTCTGCTATGCATACAATTCTCAAAATAGAAAAAAATAGCACCTGAATTTTTTCCACACACCATATCTTTTTGTCAATATAAGATTTGTAGCTATCATGT
ACTCACGTTGGTCAACAAACTTGCAGAATGTGTCAAATAGTTATAATACAAATCATTTGCTGTATTCTTCAATAAAATACATTTTATTTGAAATAGATTT
ACACTACCATTATCAATCACTCATGGCATGCATAAATAGATTTACACTACCATTATCAATCACTCATGGCATGCATACCGCGTCAGTCTTGCTATGTGTT
TAAGCTCTTTACAACCAATAGTGTTTCTGGTTGCTTTGAATCGTACCGTTGAGAATAATGAACATAAGCGGTGCCATGCTGCATGCCTTCATGGATTTAG
TTGTAATCTGCAAGTTTTCAGTTAGTAGTAAACAATATGCCACTAAAGAATTAAGAACAGAATCAATAGTTCCTTTGCAGAAAAAAGCAGGGTTACCCCA
GTTTAGGAGGTGCAGTTTGCTCAACATCATTTTCTTGCGGTGGAGTGTTAAGTTCAAGATGAACAGGAATGTTGACGTTCTCGACAACTGCAAAGTTTAG
AGTTGATGAATCTCGGTTGACTTCACTTGTCACCTCTTTCTCGTAGAGCTGAGTAGAGAAATAAACCTCAGTCAATAATGGATGGGGTGTATATCCTGTG
AATTTTCATTCACGATTTATCAAATACCTTCTTGTATAAATCAATGTTTTCCTGACGGATTAGGTTGACCTTTTTGTATAGTTCCATCTTGTCTTGTTGC
ATGAGACTTCCCTGAAAATGAGCCAGTAGAGTCAGCTTCTTGAAATCCACTCAGAGACCTCTCATTGCAGAGTAATAACAGAATATTCCAGAACCTTTCG
ATTCAGTTCGTGAATTTCATCAATCAAGAGTTGGTCCTGAAAGTAAAAAACGTCATCCATCATCAGTTGTATTCTTGGATGAGGCAATCTGGTAACGAAA
AACAGAAATATGGCTAGCATCCTTACCTTTTTTGTCCGGATGCAACGTAGGCTCATTTCTAGCTGATTTTCTAGATTTTGGAGTTCCTTGACGCTCAATC
CAGAAAGATCTTGTCCCATCAACTGACTGCAGTCAATCAAACTGTATTCAGTTTACCTGATAACCTGATATTCCTCTGCCCATGTGTATTAATGTTGTGT
TGGTTGGGAGTGAAGTGCGGACAAATCAGAGACTCAAGAAACTACCGGGCAAAACAGATGATAATATCATATTTGTTTTCCATTAGTATGTACTCTTCTT
CTTTGGAAGTTAATTGGAATGCATAAAAGCTAAATAGGTACATTTTTTGACTTATAAATGGCTTGGCATAGGAAATTCATAAACTGTTACCTCCTTTTAC
ATTGATATTGTTTATGAAAACTGATGTCCTCATAAGCAAAAGATTGTCCTATGGAGTCAGTAAATCAGTATGTCTCCTTAAACTTGGGCATTGTATGCTA
TCTTGTATACTAAATTCATTCTTATCCATTGTTTTACAATTTATACACTCTGCACAAATTAGTTGGTGGAGGTATACTTGTCACTTTACAGCCCTTAAT
TTGCATCACCAAGAGTTATGGATGATCTGTAACATGGTGTAGTTGATTCATAATTGTCTCGTCAGAACCTAGTTTTATACCTTTATAAATAAGACAACCA
GGAATTGAGTATTGCTACATGGTGAATATTGTTTCTTCTTTTGGTTCTATTCTAATGTTGTTTTGAATTTTTGTATATTCTTGAAACAAACAGTAGGAAG
GATTTCCTTCCTCCAGAGTTATCTAACTCGGAAGTTAGGCACTTGTGCTCATGCTGACAAATCCTATAGTTTGATCCTTTTACATTAGGCAAATTTTCTG
ACTTGTACATTTGTAAAATTTGCAGGGTTTTCTAGCCATACCGATGACTTTCTTGCAGGTTGTGCAGTTGTTGTCTCAAGCTTGCAGCCTCCCTTTGCCA
GAACTATTGAATATGAAAGCACAGTTAAGTAATGCCACATCCAGGATTGCCAAAATACAGCATTGGTATAAACATGTTGCATAGAAGCATGTCTGAGCTG
TCAGCTAAACAAATTGTACCTTATGAAATAACTGAATTAAATAAGATGACATGCCACTATTTCAATCAAGAGATTTACAGTGCGCTACAAAGTAACTCTC
TTTTTGAGAGGGGTGAGGGGGTGGCAACGATTATAATAGCAAATACAAAACCTTTTAAACTTGAAGGTTGAGATTCTATATTATTCAAATACTTGAATTA
AAGAACTTTATTATAGTGAACTATCAAGCTGGACACATATTGGCACGCTACACTTGCTGTATTGTCTCCTATGCTGTTGTTCACACTTTTCACACACAAT
TGCTCTGACAACTGAATTGAGCTAAATTAGCCTCGCAATACCTTGTGCTCGGGTCTAGGGACAAAACGGAACATGTCAGGATGGTGATGATCTTGCTGGA
ACATGTTTAAGCTGAAATTAGATGACTGTAGGTTTTCTCTTCGTTTTCTAGAATGTTAAAGGTCATCGTCTAAAATGTTTCACTATAAACCGGAAAGGAT
TCATGCGGCACTCATTTTTCTGGAGAATGGACTAAAACATCTATTGACTGCTGATCATTTTCAGAAGATGAGAACTTGAAACAGGATCACATGGTTTGGC
TTCCTCCAAAAAGAGGTTTGAGTATACGAGGCACTGCCTGGATGGATGGTTAGAAGGATTTCAAATACCGTGCTTACTGTTCATCGAGTAACAATCTGTA
TTATCTGTGCACATTTGCAGTCTATTTTCCCAAACTATGAACTGTACCGTACTTAGGTTCTGTGCATATGCAGCTCGACATTTTGGGTGTTTGGTGGTTG
AATGAACCTTCACCCTTCAATTTATCTGTAGTTATTTGTGATTTGAAGCATCTTCTTTATATCTGTTCCACACTGCTGCCAATTTGATTATTGATATAAT
AAATTAAGCTTTCAGATCGTATAGTAGAAATACACTATCTTTTAATGCAATCCTGGCTTTGCTTGGTATTTTATCTTCTCGAAATAACTTGTGTTCATTG
GGATCTATGTCAGTGCTGTGTCAGCATCAGCTTCGACTTACTGGCAAGCTTCAGGGCAGGCTAAGCAAGGCCCCTAAGATTTCATCCGGACTACAGCTAA
AGTGAAAAAGGGTTTATTCATGATAATTTCAATCTAAGACCTGTTTAGTTTCTATTAAGGATGCTTAAGTATACCAACATTTTTTTTATCTTTCTTGCCA
AATCTGCAAGAAAGCTTGGTCAAGAGAATCTTGCCACAACTCCCTTTGAGATGCCAAATCTGCGGTGAAGCGGAAAATTATAAAAAACTTTTGGCATTGC
TATCCTGTGTCTAAGAAACAAACATCATCCTAATTTGGTGGAATTCGCCTGATGTGTTGTAGCAAACTAAGGGGAACTGTCTGTCAATACACCTCAAGTG
CCTATATGTAGACCGGACAATATTTCAACGCCTCTTAATTCAGAGCAGT
>Locus_3441_Transcript_396/417_Confidence_0.038_Length_1793
TGTAAATCTCTTGATTGAAATAGTGGCATGTCATCTTATTTAATTCAGTTATTTCATAAGGTACAATTTGTTTAGCTGACAGCTCAGACATGCTTCTATG
CAACATGTTTATACCAATGCTGTATTTTGGCAATCCTGGATGTGGCATTACTTAACTGTGCTTTCATATTCAATAGTTCTGGCAAAGGGAGGCTGCAAGC
TTGAGACAACAACTGCACAACCTGCAAGAAAGTCATCGGTATGGCTAGAAAACCCTGCAAATTTTACAAATGTACAAGTCAGAAAATTTGCCTAATGTAA
AAGGATCAAACTACAGGATTTGTCAGCATGAGCACAAGTGCCTAACTTCCGAGTTAGATAACTCTGGAGGAAGGAAATCCTTCCTACTGTTTGTTTCAAG
AATATACAAAAATTCAAAACAACATTAGAATAGAACCAAAAGAAGAAACAATATTCACCATGTAGCAATACTCAATTCCTGGTTGTCTTATTTATAAAGG
TATAAAACTAGGTTCTGACGAGACAATTATGAATCAACTACACCATGTTACAGATCATCCATAACTCTTGGTGATGCAAATTAAGGGCTGTAAAGTGACA
AGTATACCTCCACCAACTAATTTGTGCAGAGTGTATAAAATTGTAAAACAATGGATAAGAATGAATTTAGTATACAAGATAGCATACAATGCCCAAGTTT
AAGGAGACATACTGATTTACTGACTCCATAGGACAATCTTTTGCTTATGAGGACATCAGTTTTCATAAACAATATCAATGTAAAAGGAGGTAACAGTTTA
TGAATTTCCTATGCCAAGCCATTTATAAGTCAAAAAATGTACCTATTTAGCTTTTATGCATTCCAATTAACTTCCAAAGAAGAAGAGTACATACTAATGG
AAAACAAATATGATATTATCATCTGTTTTGCCCGGTAGTTTCTTGAGTCTCTGATTTGTCCGCACTTCACTCCCAACCAACACAACATTAATACACATGG
GCAGAGGAATATCAGGTTATCAGGTAAACTGAATACAGTTTGATTGACTGCAGGCAGTTGATGGGACAAGATCTTTCTGGATTGGGCGTCAAGGAACTCC

AAAATCTAGAAAATCAGCTAGAAATGAGCCTACGTTGCATCCGGACAAAAAAGGACCAACTCTTGATTGATGAAATTCACGAACTGAATCGAAAGGGAAG
TCTCGTCCAACAAGACAACATGGAACTATACAAAAAGGTCAACCTAATCCGTCAGGAAAACATTGATTTATACAAGAAGCTCTACGAGAAAGAGGCAACA
AGTGAAATCAACCGTGATTCAACAACTACATACAACTTTGCAGTTGTCGAGAATGCCAACATTCCTGTTCATCTTGAACTTAACAGTCCACCGCAAGAAA
ATGACGTTGAGCAAACTGCACCCCCTAAACTAGGGTTACAACTAAATCCATGAAGGCATGCAGCATGGCACTTGCTTATGTTCTTTATTCTCAACAGCTA
CGATTCAAAGCAGCCTGAAAAACTATTGGTTGTAAAGAGCCTAAATACAGCAAGGCAGACGCAGTATGCATGCCATGGGCGACCGATAATGGTCGTGTAA
ATCTATTTCCAATAAATTGTATTTTATTGAAGGGATGCACCAAACGATTGTCTTACAACTATTTGACACATTCTGCAAGTTTGTTCACCAACGTGACTAC
GTGAGTACATGACAGCTAAAAATCTTATATTGACAGAAAGATATGGTGTGCGGAAAAATTCAAGTACTGTCTTTTTCTATTTTGAAAGGTGTA
>Locus_3441_Transcript_415/417_Confidence_0.048_Length_1513
CTGTGCTTTCATATTCAATAGTTCTGGCAAAGGGAGGCTGCAAGCTTGAGACAACAACTGCACAACTTGCAAGAAAGTCATCGGTATGGCTCGAAACCCT
GCAAATTTTACAAATGTACAAGTCAGAAAATTTGCCTAATGTAAAAGGATCAAACTACAGGATTTGTCAGCATGAGCACAAGTGCCTAACTTCCGAGTTA
GATAACTCTGGAGGAAGGAAATCCTTCCTACTGTTTGTTTCAAGAATATACAAAAATTCAAAACACACATTAGAATGGAACCAAAAGAAGAAACAACATT
CAGCATGCAGCACTACTCAATTTCCGGTTGGAATGTGCACTTGAAATCTCAGTCTTATTTATGAAGATAAAACTAGGTTCTGACGAGACAATTATGAATC
AACTACACCATGTTACAGATCATCCATAACTCTTGGTGATGCAAATTAAGGGCTGTAAAGTGACAAGTATACCTCCACCAACTAATTTGTGCAGAGTGTA
TAAAATTGTAAAACAATGGATAAGAATGAATTTAGTATACAAGATAGCATACAATGCCCAAGTTTAAGGAGACATACTGATTTACTGACTCCATAGGACA
ATCTTTTGCTTATGAGGACATCAGTTTTCATAAACAATATCAATGTAAAAGGAGGTAACAGTTTATGAATTTCCTATGCCAAGCCATTTATAAGTCAAAA
AATGTACCTATTTAGCTTTTATGCATTCCAATTAACTTCCAAAGAAGAAGAGTACATACTAATGGAAAACAAATATGATATTATCATCTGTTTTGCCCGG
TAGTTTCTTGAGTCTCTGATTTGTCCGCACTTCACTCCCAACCAACACAACATTAATACACATGGGCAGAGGAATATCAGGTTATCAGGTAAACTGAATA
CAGTTTGATTGACTGCAGGCAGTTGATGGGACAAGATCTTTCTGGATTGGGCGTCAAGGAACTCCAAAATCTAGAAAATCAGCTAGAAATGAGCCTACGT
TGCATCCGGACAAAAAAGGACCAACTCTTGATTGATGAAATTCACGAACTGAATCGAAAGGGAAGTCTCGTCCAACAAGACAACATGGAACTATACAAAA
AGGTCAACCTAATCCGTCAGGAAAACATTGATTTATACAAGAAGGTATTTGATAAATCGTGAATGAAAATTCACAGGATATACACCCCATCCATTATTGA
CTGAGGTTTATTTCTCTACTCAGCTCTACGAGAAAGAGGTGACAAGTGAAGTCAACCGAGATTCATCAACTCTAAACTTTGCAGTTGTCGAGAACGTCAA
CATTCCTGTTCATCTTGAACTTAACACTCCACCGCAAGAAAATGATGTTGAGCAAACTGCACCTCCTAAACTGGGGTAACCCTGCTTTTTTCTGCAAAGG
AACTATTGATTCTGTTCTTAATTCTTTAGTGGCATATTGTTTACTACTAACTGAAAACTTGCAGATTACAACTAAATCCATGAAGGCATGCAGCATGGCA
CCGCTTATGTTCA
>comp65473_c0_seq13
GCCAAGAAGAGCCGCCGCGGCCCGCGCTCCCGCAGCTCGCAGTACCGCGGCGTCACCTTCTACCGCCGCACCGGCCGCTGGGAGTCCCATATTTGGGATT
GCGGCAAGCAAGTGTACTTAGGTGGATTTGACACTGCTCATACTGCTGCAAGGGCGTACGATCGAGCTGCCATCAAGTTCCGTGGCTTCGACGCCGACAT
AAACTTCAATCTCAGTGACTATGAGGATGACATGAAGCAGATGAAGAGCCTGTCCAAGGAGGAGTTCGTGCACGTCCTGCGACGGCAGAGCACCGGGTTC
TCGCGTGGCAGCTCCAAGTACAGAGGCGTCACCCTGCACAAGTGCGGCCGGTGGGAGGCTCGCATGGGACAGTTCCTCGGCAAGAAGTACATATATCTTG
GGCTATTCGACAGCGAAGTAGAGGCTGCAAGGTTGCTGATCTTGGATTCTTCTATTGATGCAAAAAAAATTTTGCTTTACCTTCTCTGTTGATTTCCAAA
GGGTTGAGTACCGACTCGATTCTCTTCTTTGTTGTTTTTCTTCTTTCTGTTTCAAAATTCAGGGCTTATGATAAGGCCGCGATCAAATGCAATGGTAGAG
AAGCCATGACGAACTTCGAGCCTAGCACGTATGATGGGGAGCTGCTGACTGAAGTTGGTACTGAAGGTGCTGAAGTCGATCTGAACTTGAGCATATCTCA
ACCAGCTTCTCAAAGCCCGAAAAGGGACAAGAATTCCCTTGGTCTGCAACTCCACTATGGATCGTTTGAAGGCTCGGAATTGAAAAGAACAAAGATTGAT
GCTCCTTCTGAACTGGCGGGCCGCCCTCATCGGTTCCCTCTTCTGACCGAGCATCCACCAATCTGGACTGCCCAGTCTCACCCCTTCTATTCAAATAATG
AGAATGCATCAAGAGATCTTAACAGGAGGCCAGATCAGGGATGCACAGTAGGGGGTGTTCCCAGCTGGGCGTGGAAGGTGACCGCCCCTCCTCCCACACA
GCCATTGGCGCTGTTCTCGTCGTCATCGTCGTCCGCTGCAGCATCATCAGGATTCTCCAATACCGCCACGACAGCTGCCCACACCGCCCCATCGGCCTCC
CTCCGGTTCGACCCGCCGCCGCCGCCGTCCAGCCGCCACCGCTGAACGTTAAGAAGCCACGCTGTAAATTTGCCAGGAAGCCGACATTTTTTCCTCTCTC
GGCGTTGCAACTTTTTAGGTTTTGCGCCGGGGTGGTTTCTTGTAGTGGAGTGGATTCATAATTAACTGTATTTGCATGCTGCCCATGTGAAATGCTTCTC
TCTCTTTTTACGCTCTCTGCACCGGATGTTCTGGGAGTTCTTGTTGCCGGGAATTAACTAAATTCACCGTCTGAGATTGATCTATACATTGTTGTAGAGA
ATCGAATCATTGTTGAAATTAGTTACAGAATTATAGATATCATAAGGCACCCTCTACTTGTGGACAAAGTTCAGGAAGATTATTCCGGTTCTTTTCTTGG
CTGTCAATTCGGCTCTTGTCCTTGGTGTGGAGCCACTTGCCTACTGTCTTGTTCTGTTGCTGTTTGTTTTTGGCTTCTTGCACAGTTACAAATTTGTTGG
CTCGAATGGATGGTGAGACATT
>Locus_12915_Transcript_11/30_Confidence_0.412_Length_4166
GCTCCTTGAGAAACACCATCCAATTACAAGACTATTTGATGCAACAATCACGCATATGGCTGCGCTAGAACTACTGCTCTGAATTAAGAGGCGTTGAAAT
ATTGTCCGGTCTACATATAGGCACTTGAGGTGTATTGACAGACAGTTCCCCTTAGTTTGCTACAACACATCAGGCGAATTCCACCAAATTAGGATGATGT
TTGTTTCTTAGACACAGGATAGCAATGCCAAAAGTTTTTTATAATTTTCCGCTTCACCGCAGATTTGGCATCTCAAAGGGAGTTGTGGCAAGATTCTCTT
GACCAAGCTTTCTTGCAGATTTGGCAAGAAAGATAAAAAAAATGTTGGTATACTTAAGCATCCTTAATAGAAACTAAACAGGTCTTAGATTGAAATTATC
ATAAATAAACCCTTTTTCACTTTAGCTGTAGTCCGGATGAAATCTTAGGGGCCTTGCTTAGCCTGCCCTGAAGCTTGCCAGTAAGTCGAAGCTGATGCTG
ACACAGCACTGACATAGATCCCAATGAACACAAGTTATTTCGAGAAGATAAAATACCAAGCAAAGCCAGGATTGCATTAAAAGATAGTGTATTTCTACTA
TACGATCTGAAAGCTTAATTTATTATATCAATAATCAAATTGGCAGCAGTGTGGAACAGATATAAAGAAGATGCTTCAAATCACAAATAACTACAGATAA
ATTGAAGGGTGAAGGTTCATTCAACCACCAAACACCCAAAATGTCGAGCTGCATATGCACAGAACCTAAGTACGGTACAGTTCATAGTTTGGGAAAATAG
ACTGCAAATGTGCACAGATAATACAGATTGTTACTCGATGAACAGTAAGCACGGTATTTGAAATCCTTCTAACCATCCATCCAGGCAGTGCCTCGTATAC
TCAAACCTCTTTTTGGAGGAAGCCAAACCATGTGATCCTGTTTCAAGTTCTCATCTTCTGAAAATGATCAGCAGTCAATAGATGTTTTAGTCCATTCTCC
AGAAAAATGAGTGCCGCATGAATCCTTTCCGGTTTATAGTGAAACATTTTAGACGATGACCTTTAACATTCTAGAAAACGAAGAGAAAACCTACAGTCAT
CTAATTTCAGCTTAAACATGTTCCAGCAAGATCATCACCATCCTGACATGTTCCGTTTTGTCCCTAGACCCGAGCACAAGGTATTGCGAGGCTAATTTAG
CTCAATTCAGTTGTCAGAGCAATTGTGTGTGAAAAGTGTGAACAACAGCATAGGAGACAATACAGCAAGTGTAGCGTGCCAATATGTGTCCAGCTTGATA
GTTCACTATAATAAAGTTCTTTAATTCAAGTATTTGAATAATATAGAATCTCAACCTTCAAGTTTAAAAGGTTTTGTATTTGCTATTATAATCGTTGCCA
CCCCCTCACCCCTCTCAAAAAGAGAGTTACTTTGTAGCGCACTGTAAATCTCTTGATTGAAATAGTGGCATGTCATCTTATTTAATTCAGTTATTTCATA
AGGTACAATTTGTTTAGCTGACAGCTCAGACATGCTTCTATGCAACATGTTTATACCAATGCTGTATTTTGGCAATCCTGGATGTGGCATTACTTAACTG
TGCTTTCATATTCAATAGTTCTGGCAAAGGGAGGCTGCAAGCTTGAGACAACAACTGCACAACCTGCAAGAAAGTCATCGGTATGGCTAGAAAACCCTGC
AAATTTTACAAATGTACAAGTCAGAAAATTTGCCTAATGTAAAAGGATCAAACTACAGGATTTGTCAGCATGAGCACAAGTGCCTAACTTCCGAGTTAGA
TAACTCTGGAGGAAGGAAATCCTTCCTACTGTTTGTTTCAAGAATATACAAAAATTCAAAACAACATTAGAATAGAACCAAAAGAAGAAACAATATTCAC
CATGTAGCAATACTCAATTCCTGGTTGTCTTATTTATAAAGGTATAAAACTAGGTTCTGACGAGACAATTATGAATCAACTACACCATGTTACAGATCAT
CCATAACTCTTGGTGATGCAAATTAAGGGCTGTAAAGTGACAAGTATACCTCCACCAACTAATTTGTGCAGAGTGTATAAAATTGTAAAACAATGGATAA
GAATGAATTTAGTATACAAGATAGCATACAATGCCCAAGTTTAAGGAGACATACTGATTTACTGACTCCATAGGACAATCTTTTGCTTATGAGGACATCA
GTTTTCATAAACAATATCAATGTAAAAGGAGGTAACAGTTTATGAATTTCCTATGCCAAGCCATTTATAAGTCAAAAAATGTACCTATTTAGCTTTTATG
CATTCCAATTAACTTCCAAAGAAGAAGAGTACATACTAATGGAAAACAAATATGATATTATCATCTGTTTTGCCCGGTAGTTTCTTGAGTCTCTGATTTG
TCCGCACTTCACTCCCAACCAACACAACATTAATACACATGGGCAGAGGAATATCAGGTTATCAGGTAAACTGAATACAGTTTGATTGACTGCAGTCAGT
TGATGGGACAAGATCTTTCTGGATTGAGCGTCAAGGAACTCCAAAATCTAGAAAATCAGCTAGAAATGAGCCTACGTTGCATCCGGACAAAAAAGGTAAG
GATGCTAGCCATATTTCTGTTTTTCGTTACCAGATTGCCTCATCCAAGAATACAACTGATGATGACGTTTTTTACTTTCAGGACCAACTCTTGATTGATG
AAATTCACGAACTGAATCGAAAGGTTCTGGAATATTCTGTTATTACTCTGCAATGAGAGGTCTCTGAGTGGATTTCAAGAAGCTGACTCTACTGGCTCAT
TTTCAGGGAAGTCTCATGCAACAAGACAAGATGGAACTATACAAAAAGGTCAACCTAATCCGTCAGGAAAACATTGATTTATACAAGAAGGTATTTGATA
AATCGTGAATGAAAATTCACAGGATATACACCCCATCCATTATTGACTGAGGTTTATTTCTCTACTCAGCTCTACGAGAAAGAGGTGACAAGTGAAGTCA
ACCGAGATTCATCAACTCTAAACTTTGCAGTTGTCGAGAACGTCAACATTCCTGTTCATCTTGAACTTAACACTCCACCGCAAGAAAATGATGTTGAGCA
AACTGCACCTCCTAAACTGGGGTAACCCTGCTTTTTTCTGCAAAGGAACTATTGATTCTGTTCTTAATTCTTTAGTGGCATATTGTTTACTACTAACTGA
AAACTTGCAGATTACAACTAAATCCATGAAGGCATGCAGCATGGCACCGCTTATGTTCATTATTCTCAACGGTACGATTCAAAGCAACCAGAAACACTAT
TGGTTGTAAAGAGCTTAAACACATAGCAAGACTGACGCGGTATGCATGCCATGAGTGATTGATAATGGTAGTGTAAATCTATTTCAAATAAAATGTATTT
TATTGAAGAATACAGCAAATGATTTGTATTATAACTATTTGACACATTCTGCAAGTTTGTTGACCAACGTGAGTACATGATAGCTACAAATCTTATATTG

ACAAAAAGATATGGTGTGTGGAAAAAATTCAGGTGCTATTTTTTTCTATTTTGAGAATTGTATGCATAGCAGAAGAGCACAAATTGCATTCTGGTTTATC
CGTGCAGGCATTCCAGAGTATCAGAATGGGGATCAACCTTTCTTCAGCTACATATGTAAATATACGGATGCTAGTTGCTCACTTAAAATTTTGTAGTGTT
CAAAGGAATAACAACAGTAAGACGTTGACGCACCGAAAATAACCAGAAATAACGGATTGTACTATGACAAGACACACGACAGATACACTAGCTTAAGTTT
GTACAGGAAAATATTTGGCCTATTCTTATGTCACAGTAAATTAAGATTATCACCTTAATCACAGAAATTCAAGGGGTAAACGATTATAGCACAAAACGAT
CCTGGACCCCAAGACCTGGGTCTCTGAACGGCTGAACCTCCAATTATACCTCTATACATTTGGAATGTGTGTAGTCAAACTTTCGCTACTGGTATCTTTA
CAAATATTTACATTGAACACATCAAAATGATATACATGGATTCCTCTCGATATGTCTTTGAGGTTTGGGTTCTAAAAATGATATACAAATTATTAGGTTC
TAAACTTAAATGATATATACACATCAAAATGATATACAAATTATTGGGTTCTAAACTTAAATGATG
>Locus_4567_Transcript_9/65_Confidence_0.063_Length_344
ATAAACTGCAAATCAAATACATTTGCTGTTTTCAAGAACTCAGTACCATCAAGATTCATGTACAGGTGATTTGCAGTTGCACTAGAATCACACACCACAA
CTGCATAGTAATACCTCAGTCTGTTTAGCTCATAAGTACGCAACTTGTTATTCTCTTTGTCGGAGTCAACTTCTTCGTCGTCGTCATCATCAGTGTTGTC
TTCATCATCATCGTTGCTATCATCGTCGTTGTCATCATCATTATCATCAATATTGGCATTAACAAGAGCAGATGGGCCCTGAGTTGACTCAATTTCCATG
CACTTCAGTCCAAATTCTGATGGATATATTGAGACTGATAAAAC
>Locus_18892_Transcript_36/56_Confidence_0.294_Length_1881
TGGTGGCTGCGCCGTCTCCTGCGGCTCTATCTCCCGCCGCCGCCGGGAAGAAGAGCCGGCGGAGGCCGAGGTCCCGGAGCTCGCAGTACAGGGGCGTCAC
CTTCTACAGGAGGACGGGCCGGTGGGAGTCGCACATCTGGGATTGTGGGAAGCAAGTCTACTTAGGTGGTTTCGACACTGCTCACGCGGCTGCGAGGGCT
TATGATCGTGCAGCGATCAAGTTCCGAGGGCTCGACGCGGACATCAACTTCACTTTGAGCGACTATGAGGATGACTTGAAGCAGATGAGCAATTGGACAA
AGGAAGAGTTCGTGCACATACTCCGCCGCCAGAGCACGGGGTTCGCGAGGGGGAGCTCCAAGTACCGCGGCGTGACGCTGCACAAGTGCGGCCGCTGGGA
GGCGAGGATGGGTCAACTTCTTGGCAAGAAGTAAGCCAACCTCCTGGCTTTGACCTTTCTACTCTGTTAGCCTAGTTAAGTGTTTTTTACCCTGGTTATA
TGAACAAAATATGTCGAAATGATGTTCAATTAACTTGTCATTTCACGTTGGGTTCGATCAGGGCTATATCCCTTATGTTCATGTTAAGTAGTTTCAGGTG
ATTGTAGAGAATACAACAAGGTGATCTTGAAGTATCACTGATTATAATCCATGCTACTAGTAGTTTGAGCGTCTCTGTTGCCGGTGAAATCTCCACCCCT
CTTGTGACTTTACCCCTCTACCGTTGAATCCGGGAATCCGGAATGGACCTACAAGATCGAGATATGTGCCTTGATGAACAGTGTTTCGGTGAACAGCAAA
ATCGGCACCCGTGCACAGTGTCCTGATGGACGCAACCTCTAGAGTTCACATATATGCATTGGCCAACAATGCAATTCCTGCTTTTTAGTCAGGAACCAGT
TTTGTCAAAGTCTACGACACTAGGCATCCCTCTGTTGAGACATATCTCAGAACTGGAATTTACATGCAGGTACATCTATCTTGGACTCTTTGACAGCGAA
GTTGAAGCTGCAAGAGCATATGACAGGGCAGCCCTTCGCTTCAATGGGGGGGAAGCTGTTACTAATTTTGGGCCTAGCTCCTACAATGGAGGAGATGCTC
TACCCGACACCGAAAATGAGGCAATTGTTGATGGCGATGCGGTTGATTTGGATTTGCGGATTTCACAACCTAATGTGCACGACACTAAAAGGGACAATAC
CTTAGCTGGAGGCCAGCTAACATGCGACTCCCTTGAATCTTCAAACACGATGGCCTCTCAGCCAATGAGCTCATCGTACCCGTGGCCTGTGTATCACCAA
AGCACGACAGCAGTACCACCTCACCATCAACGCTTGTACTCATCTGCTTGTCCTGGCTTCTTTTCGAACCTCCAGGAAAGGCCAATGGAGCGAAGGCCTG
AGTTGGGTACCCAGTCATTCCCCACCTGGGGATGGCAAATGCAGGGCTCCCCTCACATGCCGTTGCATCACTCTGCAGCATCATCAGGATTCTCTACCGC
CGTCGGCGCAAACGCCGGCGTCTCGTTGCCTTCTCACCCGCCGGTGCCGTTCCCGGACCACCAGTTCTACTTCCCACCGACGGCATGAGCTTGTTGGTCT
GGTTCAACTCTTGGGACGGCAGCTGATCTTATGTGTGAACATTTCGCAGCTTGCCGGTGACCGTTGTTAATTAATCGGGGAGAGAGAGCCAGAAGACCCA
GTATGGCTATCTCTCTGTGGCCCGTGCCTTGTTATGATCTTAGTTATTGCTACTACACCGTGGATTCATCATGGTTTATGAGATTCTTTACTCAAGTTTC
AGCTTAAATGTCCAAATACATGAATGCATCATACACAGGATCCGTCATTTTCTAAAAAAATACAGGATCTGTGTGAAGTGT
>Locus_5983_Transcript_44/82_Confidence_0.316_Length_2385
TGCCATCTCCATCTTGTGCGTCTCTGTTGGATCTTTGTGAGTCATGGCCTCTTTGCTCTCTTGCTTTTCTTCGTTTCTTGTTGAACTCATGGTTCTTGTT
TTTCTTGTGCTTGCCAGGTAGAATTATTCAACCGGATATAGATTCCTTCAAGTAAAGGAGTGCTAGCGGTTTTTACCTGTTCGGGTGGGAGCACAAGTTG
AGATCTTTAATTCTAGCATGGGTTCCTTTGGGGTGAACTGGAATCAGAAGAACTCCGCGGTGTGGGATTGGGAAAAATTAGCTCCATCTGTTCCAAATGC
AATCGGAAACCCCAGGAACGGAGCTACGGGGCATGGATCTGTGAATTCATCTGGTGGCACTCTCACTTCTAGCTCAGAGCTAGGGCATGGTTCATCCAAA
AGCTCCATGTCGGCATCCATTGATTCACCGTCCAAAGTAGGGAACAGCTTGGAGTTCAATTTTGCTGCTGTCAATACGTATGGTAAGAACATGGATAAGG
ATTGTAGAGTTGACGACTCAGGGACTTGTCCATCGTCCATGATAGCATTCAGCCATGGAGAGCCATTAATCAGTCTTAAGCTTGGAAAAAGGACTTACTT
TGAAAATGTCTGCGGAGGACAGGATTCCAAGAGCACTGCACCTTCTACCAGGACTTCTCCATCAACCGTTCTCAAGAAGACCAAGGTGTCTCAGCAGAAT
ACACAAATGTCATACTGTCAGGTTGAAGGTTGCGGAGTTGATCTATCTTCTGCTAAAGATTACCATCGCAAGCACAAAGTCTGTGAAGCTCATTCTAAAG
CTCCCAAGGTTGTTGTTGCTGGTCTAGAGCGCCGTTTTTGCCAGCAGTGTAGCCGGTGATATTTCCTTGACTGAGTGCATTTTCATTATATAGTATGCCT
CTTTGTATTCACCGAATCCTTTTTTAATGTTGGCATCTCTAATTGAAAAGAGAGAACATAAAAAATGTGATCAGTTGAAAGCAATACAACTATTTAGTGG
TAGAACGACCAAGGACCAACTGCTAGATGATGCCCAATTATTTTGCATGACTGAGACTTCCAAAGTATCTGTGATGTTTATTACTCCTTTGCTGATCAAG
GTACACAAGAACACTGGAGTGACATGAACACTGAAGAAAATTGGCATTTCTTTTTGTTTCTGGCACTAGTTTGTTATTGATTTGTAAGCGGAATGCTGAT
AAACATCTTGAAAAGTGAAATTCAGATAAACTAATTACAATACAGTTTGATCTTTTAGCGACATAATGGACATTTATTTCCTTTTCTTTAATTCTTCATT
GGTTTGTAGGTTTCATGGTTTAGCTGAGTTTGACCAGAACAAACGAAGCTGCCGTAGGCGTCTTACTCATCATAATGCACGGAGGAGAAAACCACAGGCA
GATACAATTTCATTCAGTTCATCGCGGCTCTCGACAATGTTTTATGATACAAACCAGCAGAAAAATCTTTTCTGTAGTCAACCTCATTTTGGCCAAGCGA
GAAGCAATGCAGTTTTTTCTTGGGAAAACTCGGGAGATTCCAAATTTATGGAAACCAAACATCTGTTGATGCAGCCAACGAAAATGTTAGGTCTTGATGG
GCTGCATTTCTCAACCCCCCAGATATCAAATAGTGTTGTGGCTCACTCTGTACATCATCGTAATTTCGATGGGCTCATGCCATTCAAGGGAACCAACACA
AAGGTCCTCAACCAAGGCGTGGAAGCTTCTGCGATCGCTTCCAACTCGAGTGGAGACCCGGATCTTGGGTGTGCTCTCTCTCTTCTGTCAATCGGTTCGT
GGGTTTCAAGTTCAACCGTCATCCAGCAGCCTAGTTCTCATGTGCACGCTGGTGTGGTGCCGCCCCTCGCCACCGTTGCCGTCTCCAACCCTGCAATGCA
TCCTCTGGACTCATCCCCAGGAGGATTCTGGCAAGACGTCCCTCCCCCGCTCGATGAAACTCCACATATTCAGGCATTCACGCACCTCTGATCAAATGCT
GCATCCCCTGGGAACAGCAGGTGGTTATACCTTCCGTAATCGTCCTAGGTTTGCTTTGCCAGTGACTGGCTCTGCACAATGTCGATCCATCACCGGAGGC
ATTTGGTAAGGTGAAGACTAAAGACTCAAGAATAACCGGAGACTAACTCGCTTCTCTTCCAGGCTCAAGACTGTTTGGGTCCAGCGGTGTTGCTACCTGA
TCCTGTTTTCGGTTCTGAGTACATCCATTACTAATCATGGGATTACTTGTTTAGGCCAACCAGACATATTCTGGGGTGAGATTACGACATGTTAATTTAC
ATTGACGGTGTTTCTACATGTGTTAGTACTAATGATGTTATCGCTCTTGGCCCCGTTACGTTTCAAGATCTGTTACCATTTGAGT
>comp79344_c0_seq4
CTCTCTCTCTCTCTCTCTCTCCAGCTAGCCGCCGTTTGACCCTGCAGATTGCTTCCTTCTGCACGGCGCGATGGTACGGGGAAAGACCATATTGAAGAAG
ATCGAGAACGAATCAAGCCGGCTTGTGACCTTCTCCAAGAGGAGGGGCGGGTTGTTCAAGAAGGCAAAGGAACTGGCAATCCTATGCGACGCTCAGGTCG
CTGTTCTCATCTTCTCCAGCACCGGCCAACTCTACGACTTCTCCAGCTGCAGCATGAAATCAATAATCGAAAGATACCGCCAAGTAAAAGAAGGTCAGCA
AAATGTAACGGCAAGTAGGGAGGCCAAGTACTGGCAAGGGGAGGCGGAAAGCTTGAGGCAGCAACTACACAACTTGGAAGAGAATCATAGGTAAGAAAAT
CCACCAACAGTATTGACAGAGGTGAATACTTTCTTACTGAGCAACCTTGGATGAAACTTAAAATGCAGTGTAATGGAGTCCATCTAATAGCAACGAATGT
GAAAACTTTAATAACAAGTCATATATTTTTATGTCCTTAGGGAGCCATTGGGGTCCATGGATTCTGTAACATCCTAGCTGGCATGTTTATAGTGGTCTTG
GCCTATGTGGTCTAGATGTTGAAGTGTGATGGGATTAGTTCTATCTTGGGTCTTTAGGTAGGTCAGAGCCTACATATAAACCTTGTCTTTTAGAACATAG
CACATCTGGATTAACCCTTTTACCAGAAGTGAACATGAAAGCATAAGAAACATGCAATGCCCATGCGTGAGCTTGCTCGATGTGGGACTTGATAATAAAC
AGGTTTTAGATATAGGGTGTTACAAATTCTTATTTGTGGTTGACAGAACAGGGAGCATGCATTGTTGTAGGGGAAATCATGCAAACGTTTTCAGGTTGTC
AATTTGGCACCACAGTACAAGCCTGAAACACAAGATAACAAGTGCAAACTGCCACAAGATGCACTTGCTGGGACAACATCTTTCTGGCTTGTCAATGAGA
GACCTGCAGACTTTAGAGAACCAGCTGGAAACGAGCCTAACTAATATTCGGCTAGCAAAGGAACGAATTATGATTGATCTGATTCAAGAATTGAACAAGA
AGTCAAGCCTCATAGACATCGAAAACAAGGAGCTACACCACAAAATCTGTGCCATTAGTCAAGAGAATGCCAATTTGAAGAAAGAGGTACATGGACAGCC
GGATGCCATTGAAGAAAATACTGGTTCTGTTACTCAGTATAGTACTAACACTCCTGAAGACCTAAATGCGACGTGTCTCGAGCTACGCCAACCACATCAT
GTAGAAATAGAGCAACCAGATGTGCCTACATTGGAACTGCGTCTCCACTGATTGCAAGAATCAGCAGCAGAATCATCGGAAATGAGGAGGCCAGCCATTC
AACGCAAATTCATAAGACTTTGTCTCCACTGATTAAATACAAAGAGGAGTTCCTGTGCAAATTACTATTATATGAATGTAACAGATGTAATTTATTTTTT
ATGTATCCGCAGTACACATATGGCCACCATAAACTTATGTTCAAAAGGCATATATACCTATTGCTATCAAGCTACG
>Locus_17579_Transcript_71/92_Confidence_0.204_Length_1744
CTTTTTCTGGGAGCAACGTAGTGGGAGGTAGCTGCTTTTGCATACAATTAGGAGCAGCAGTTGAGATATTCAATTCGGGCATGGGTTCTTTTGGGATGGA
CTGGAACCAGAAAGGCTCGGTGTTGTGGGATTGGGAGAATTTGCCGCCGATAGGCAGAAATGCAAACGAGAACCTCAAGATTGCGCCACAGGCTGAACCG
AAGTTTGCAGGTGTTGAGGCAACAAGGCATGAATCGGGGCATTCTTCTTGTGGTACTTTCTCTTCCAGCTCGGAGATGGGGTATGGTTCATCCAAGAGTT

CCATATCAGCGTCGATTGATTCTTCACCCAAGGTGGGAAACAACGTGGAGCTCAATCTTGCAGCTGTCAAAGTGCCTGACAAAAACACCGTCAAGAACAC
TGATTTGGGTAAAGTTGATGACGCTGGAACTTCTCCATCATCGATGATAGCCGTCAGCAGTGGAGAACCGGTGATTGGCCTGAAACTTGGCCAAAGAACC
TATTTTGAAGATGCATGCGGAGGGCAAAGTGTCAAGAGTTCACCATCCAATGTGAGTGCAGTGACCCCTGCTTCTGCGAAGAAAGCAAAGATGATTCAAA
ACGCACAGAACTCGTACTGTCAGGTTGAAGGTTGCAAGTTTGATCTCTCTTCTGCTAAAGATTATCATCGGAAGCACCGAGTCTGTGAAACTCATTCTAA
GGCTCCCAAGGTTATTGTTGCTGGTCTGGAGCGACGCTTTTGTCAGCAGTGTAGCCGGTTCCATGCTTTAACTGAGTTCGACCAGAAAAAACGAAGCTGC
CGGAGACGCCTCAATGATCACAATGCCCGCAGACGGAAGCCACAGCCTGAAGCAATTCCTTTCGGTTCATCAAGGCTCTCTGCAATGTTTTATGATGCAA
GGCAACAGACAAGTCTTCTGTTTGGTCAAGCTCCTTATGGTCAAATGAGAAGCTGTGCAAACTCTTCATGGGATAGCCCAGTAGGAGGCTTCAAATTTGG
AGAAACAAAAGCTCCTTGGTTAAAGCCAACGAGAGCTGCAGGTGTTGATGGGATGCATGTATCAAGCCAGCAGGTGTGGAACAATATTACGCCACACGGT
GCACATCATGATTTTAATGGTATCATGGCTTTCAAGGGAACCAGTGCAAATGTCCTTAATCAAGGTGCCGAAGCTTCTCCGGTCGTCTCCAACTCGAATG
GAGCCCCAGATCTTCAGCGTGCTCTCTCTCTTCTGTCAAACAATTCAGCTGGTGCTGCCAACGACCAGCCAACTCCTCAGCTGCACCCTGGGCTGACCAC
CTTCGTCGGCTCCTCCAACCCTGTCACTGTGATGGAAGCCTCACCACCAGGACTCTGGCAAGACAGCACGCCCCTTGATCATCAGGCGCAGTTCCAGGCT
TTCGATCCCATTGCCGCACCGCAACAGCTCCAGCTCCCAAAACCCCCTTCCTTCAACAGCTCCACTTCCAGCTATGGCCAGATGCAGTGATGGATGCTTC
CTTCAGTTCTGCTGTCTGAAACTCTGAAATTTGAAACTGCTGTGTTCTAAAAAATAAAGAAAAGCCTGTGTGCGAAAACTCAGGCTTGAAAAATTGATCC
AGTAGTGTTCCCGTTCAATGGCTTGAATAATCCATCCTTGTGATGGCGTGTAACTCAAATGCTTGGATTGCGCTGGCAAGTGATCATGGACTCTTGTGTT
CTTGATGGCATCTCAATTACACCTTGAATGGAACTCTGGTTCTT
>comp79373_c0_seq36
CTGTCCTGCCCAGCAGCGCACAAGCAAGCTCGCAGCAGCGCCATTGCACGCTTCCGGTCGGCCGCTGCTGCGCTGCACGTCTGTCCTCCCGCCCCGTACC
GCCTCGTACACATGTAAACTGCTGTGCTGTAGGTTTGGAGCCCCCGTACAAGCGTGTTCGAGTTTGCTGATGTTCATCTAATTATCCCCGGGGAAGGAGT
GATTGTCCGCGGAGAGAAGGAAGAGATCGAATTGAAGGTCAAGATAAGTTATTGAAACGGCAAGATGGTTAATTTTGGAAAGAAGTTGATGACAGATCAA
GTAGAAGAATGGAAAGGGTACTATATCAATTACAAACTGATGAAGAAAATGTTAAAGCAATATGTTCAACAGACCCAATTAGGCGGGAAAGATTGTGAAC
AAATCCTTAAAGAGTTCTCGAGGATTCTTGATGACCAGATTGAAAGGATTGTGCTTTTTCTGCTACAACAACAAGGCCACCTTGCCAGCAGGATTGAGGA
ATTGGGAGAACAACGCACTGCTCTTATGGAACATTATGATATATCACAAGTTTCCCAGCTACGTGATGCGTATAGAGAAGTTGGGAGAGATCTTATTAAG
CTTCTGCGCTTTGTTGACATGAATGCTACTGGTATACGCAAGATACTAAAGAAATTTGATAAGCGTTTTGGCTATAAGTTTACATATTATTATGTCACCA
CTCGTGCAAATCATCCCTATTCTCAGCTTCAACAAGTATTTAAGCAAGTGGGAATTGTAGCTGTTGTAGGTGCATTATCGCGCAATCTTGAATATCTGCA
ACATCATCAAGGAAGCTTTGTATCCATCTATGATCATCCATCAGTTACCTTGAAGGACCCTATAATAGACGAAGTAAACCATGCGGTACAGAAATTTACG
CATGCCACCAATTTTATGAAATTCTTGGGACAACACGCGCTTATTGTTCCAGAGGATGCACGAAGCGGGTCTGAGGATCTTGTTGATGATCAGAGCTACC
ATTTCATGTCTCTGCTGCTTAACCTAGCGAACACTTTTCTTTACATGGTGAACACATATATCATTGTGCCAACTGCAGATGACTATTCAGTAAGCCTTGG
GGCTGCTGCGACTGTCTGTGGTGTAATTATCGGATCAATGGCAGTCACTCAAGTGTTCTCCTCGGTTTATTTCAGTGCATGGTCAAATAAGTCATACTTC
AGACCACTTGTATTCAGTAGCATTATGCTATTTTTTGGGAACCTGCTATACGCATTGGCATATGACCTGAATTCATTAATAGTTCTCCTGATTGGACGAC
TACTATGCGGGTTGGGCTCTGCAAGAGCAGTGAACCGTCGCTATATTAGTGATTGTGTGCCTCTCAAAATCAGGCTACAAGCTTCTGCAGGATTTGTCAG
TGCTAGTGCTCTTGGCATGGCATGTGGCCCTGCTCTTGCTGGTTTCCTCCAGACAAAATTTAAGATATACTCGCTCACTTTTAATCAGAGCACATTGCCC
GGATGGGTCATGTCCATTGCTTGGCTTCTTTACTTGTTGTGGCTGTGGTTTACATTCAAAGAGCCAGAACACTTCGCTAAAACTGTCATCAATGCACAGC
CGTCTGAATCAGGTCACCAAGGAAGTGCTAATTTGGAGGAAGGTCTAGCTCAACCATTGCTTCTGGGTACAGAACAAAGACAGGGCGAGAATGCGGATGA
CAATGATGATAATGAAGTAGACTCTGAAAACTCTCATGAACCAGCAACATCAATTACTTCAGCATACAGATTGCTGACACCATCTGTGAAGGTCCAGCTA
TTGATATACTTTATGCTCAAGTATGCTATGGAAATTTTACTATCTGAATCAAGCGTTATCACAACATACTATTTTAGCTGGTCTACGAGTGCTGTGGCTA
TCTTTTTGGCAATTCTTGGATTAACTGTCCTTCCAGTAAATGCCATTGTTGGAAGCTACATTACAAATTTGTTTGAGGACAGGCAAATTCTGTTGGCATC
TGAAGTCATAGTTCTCATTGGTATCATCATGAGCTTTCGTTTCACCCCTCACTACTCCATTCCACAATACGTCACTTCAGCTCTCATCACATTTGTGTTT
GCTGAGGTACTCGAAGGTATGTATTTTGTACATGTGTGCAACCATCTCAAAGCATCATGCACAGTAAGACATGAAAACAATCATACGCTATTGCTATAAC
CATCATACAATTTCTGATGACGAGCAATGCAGGAGTGAATCTGTCCTTGCTCTCACGAGTAATGTCGTCTAGGCTTTCGCGAGGGACCTACAACGGTGGA
CTCCTCTCGACAGAGGCCGGGACATTGGCCCGTGTAATTGCAGACGCGACTATTACTGCAGCAGGTTATCTAGGCCCGGACCTCCTCCTCAACGTCACCC
TGCTGCCACCTCTTGTGATCTGCGTAGTCTCCATCGTTGCAACACTCTACACTTACAACACTCTGTACTGAAGAAGCAGGCCTCCCTTTGTAGCTGTATA
GCACCATTCCCAGTAAAAATGATTCCGGCCCACATTGGGTTGTGTACTTGTACCAGCATTTATGGTTTGTAAAAAGTCAATGAAATGCATTTGTAGTGTT
CAATGAATTGTGTAATTTTTCATCTTGTAATGATTTATGTGCCACGATTGCCAGTAGTTGTATTAGTGCATTGTGGTCAATTTATTTGTTT
>Locus_1016_Transcript_1000/2070_Confidence_0.003_Length_4765
GTTTCAAGCTCAAGCTCGGCGCCGGCGTCTCAGTGAACCACTCTGCTGGGGGTGTACTTCAATTTGTTGTTGCCAGTGTTATTAGCAGTCACTGCAGCTC
AAGTAGCAGGCTGGAAGCTTTTAATCTGACATTTGTTCTTTCCTTTTCTGATAATTACTGCAAGTGGAGGCGGCAAGCTAGAGATAACAACTGCATAACT
TGCAAGAAAATCGTAAGTTTCTCAATTTTTCTGTTCTATACATGAGAAAACGGTGTTGATTTGTGTGCATGCGTGCGTTTGTCAATGATCTGAATGATTT
GCACATGATTTGTGTTAACATGCTGTGAATTCAGGGTATGGCAAATTTGCCAAATATCTTTTCAGGATGCTTAAATTCACTTTGTTTAGAACTGTTTTGA
GAACCGTATGGATTCAATAGACACCCAATTACTTGGTTATAATTGAGTTATTTACTTTACGAAAGGTCGATGTGGTCATAGCATATTAAAGTTCCTTTTA
ATTGACCAATGACCGTATGGTTACTCTCATTCCTTCAATTCATTGCTCTCCCAGTCACAAATAAGTGATGTTTGGGACATTGACAAGGCCTCCAAAACTC
AAATGACCACCTAATTCTGCCATAGTATATTTTTAGAACCTAATAATTTGTATATCATTTCGATGTGTATACATCATTTAAGTTTAGAACCCAATAATTT
GTATATCATTTTGATGTGTATATATCATTTAAGTTTAGAACCTAATAATTTGTATATCATTTTTAGAACCCAAACCTCAAAGACATATCGAGAGGAATCC
ATGTATATCATTTTGATGTGTTCAATGTAAATATTTGTAAAGATACCAGTAGCGAAAGTTTGACTACACACATTCCAAATGTATAGAGGTATAATTGGAG
GTTCAGCCGTTCAGAGACCCAGGTCTTGGGGTCCAGGATCGTTTTGTGCTATAATCGTTTACCCCTTGAATTTCTGTGATTAAGGTGATAATCTTAATTT
ACTGTGACATAAGAATAGGCCAAATATTTTCCTGTACAAACTTAAGCTAGTGTATCTGTCGTGTGTCTTGTCATAGTACAATCCGTTATTTCTGGTTATT
TTCGGTGCGTCAACGTCTTACTGTTGTTATTCCTTTGAACACTACAAAATTTTAAGTGAGCAACTAGCATCCGTATATTTACATATGTAGCTGAAGAAAG
GTTGATCCCCATTCTGATACTCTGGAATGCCTGCACGGATAAACCAGAATGCAATTTGTGCTCTTCTGCTATGCATACAATTCTCAAAATAGAAAAAAAT
AGCACCTGAATTTTTTCCACACACCATATCTTTTTGTCAATATAAGATTTGTAGCTATCATGTACTCACGTTGGTCAACAAACTTGCAGAATGTGTCAAA
TAGTTATAATACAAATCATTTGCTGTATTCTTCAATAAAATACATTTTATTTGAAATAGATTTACACTACCATTATCAATCACTCATGGCATGCATACCG
CGTCAGTCTTGCTATGTGTTTAAGCTCTTTACAACCAATAGTGTTTCTGGTTGCTTTGAATCGTACCGTTGAGAATAATGAACATAAGCGGTGCCATGCT
GCATGCCTTCATGGATTTAGTTGTAATCTGCAAGTTTTCAGTTAGTAGTAAACAATATGCCACTAAAGAATTAAGAACAGAATCAATAGTTCCTTTGCAG
AAAAAAGCAGGGTTACCCCAGTTTAGGAGGTGCAGTTTGCTCAACATCATTTTCTTGCGGTGGAGTGTTAAGTTCAAGATGAACAGGAATGTTGACGTTC
TCGACAACTGCAAAGTTTAGAGTTGATGAATCTCGGTTGACTTCACTTGTCACCTCTTTCTCGTAGAGCTGAGTAGAGAAATAAACCTCAGTCAATAATG
GATGGGGTGTATATCCTGTGAATTTTCATTCACGATTTATCAAATACCTTCTTGTATAAATCAATGTTTTCCTGACGGATTAGGTTGACCTTTTTGTATA
GTTCCATCTTGTCTTGTTGCATGAGACTTCCCTGAAAATGAGCCAGTAGAGTCAGCTTCTTGAAATCCACTCAGAGACCTCTCATTGCAGAGTAATAACA
GAATATTCCAGAACCTTTCGATTCAGTTCGTGAATTTCATCAATCAAGAGTTGGTCCTGAAAGTAAAAAACGTCATCATCAGTTGTATTCTTGGATGAGG
CAATCTGGTAACGAAAAACAGAAATATGGCTAGCATCCTTACCTTTTTTGTCCGGATGCAACGTAGGCTCATTTCTAGCTGATTTTCTAGATTTTGGAGT
TCCTTGACGCTCAATCCAGAAAGATCTTGTCCCATCAACTGACTGCAGTCAATCAAACTGTATTCAGTTTACCTGATAACCTGATATTCCTCTGCCCATG
TGTATTAATGTTGTGTTGGTTGGGAGTGAAGTGCGGACAAATCAGAGACTCAAGAAACTACCGGGCAAAACAGATGATAATATCATATTTGTTTTCCATT
AGTATGTACTCTTCTTCTTTGGAAGTTAATTGGAATGCATAAAAGCTAAATAGGTACATTTTTTGACTTATAAATGGCTTGGCATAGGAAATTCATAAAC
TGTTACCTCCTTTTACATTGATATTGTTTATGAAAACTGATGTCCTCATAAGCAAAAGATTGTCCTATGGAGTCAGTAAATCAGTATGTCTCCTTAAACT
TGGGCATTGTATGCTATCTTGTATACTAAATTCATTCTTATCCATTGTTTTACAATTTTATACACTCTGCACAAATTAGTTGGTGGAGGTATACTTGTCA
CTTTACAGCCCTTAATTTGCATCACCAAGAGTTATGGATGATCTGTAACATGGTGTAGTTGATTCATAATTGTCTCGTCAGAACCTAGTTTTATACCTTT
ATAAATAAGACAACCAGGAATTGAGTATTGCTACATGGTGAATATTGTTTCTTCTTTTGGTTCTATTCTAATGTTGTTTTGAATTTTTGTATATTCTTGA
AACAAACAGTAGGAAGGATTTCCTTCCTCCAGAGTTATCTAACTCGGAAGTTAGGCACTTGTGCTCATGCTGACAAATCCTATAGTTTGATCCTTTTACA
TTAGGCAAATTTTCTGACTTGTACATTTGTAAAATTTGCAGGGTTTTCTAGCCATACCGATGACTTTCTTGCAGGTTGTGCAGTTGTTGTCTCAAGCTTG
CAGCCTCCCTTTGCCAGAACTATTGAATATGAAAGCACAGTTAAGTAATGCCACATCCAGGATTGCCAAAATACAGCATTGGTATAAACATGTTGCATAG
AAGCATGTCTGAGCTGTCAGCTAAACAAATTGTACCTTATGAAATAACTGAATTAAATAAGATGACATGCCACTATTTCAATCAAGAGATTTACAGTGCG

CTACAAAGTAACTCTCTTTTTGAGAGGGGTGAGGGGGTGGCAACGATTATAATAGCAAATACAAAACCTTTTAAACTTGAAGGTTGAGATTCTATATTAT
TCAAATACTTGAATTAAAGAACTTTATTATAGTGAACTATCAAGCTGGACACATATTGGCACGCTACACTTGCTGTATTGTCTCCTATGCTGTTGTTCAC
ACTTTTCACACACAATTGCTCTGACAACTGAATTGAGCTAAATTAGCCTCGCAATACCTTGTGCTCGGGTCTAGGGACAAAACGGAACATGTCAGGATGG
TGATGATCTTGCTGGAACATGTTTAAGCTGAAATTAGATGACTGTAGGTTTTCTCTTCGTTTTCTAGAATGTTAAAGGTCATCGTCTAAAATGTTTCACT
ATAAACCGGAAAGGATTCATGCGGCACTCATTTTTCTGGAGAATGGACTAAAACATCTATTGACTGCTGATCATTTTCAGAAGATGAGAACTTGAAACAG
GATCACATGGTTTGGCTTCCTCCAAAAAGAGGTTTGAGTATACGAGGCACTGCCTGGATGGATGGTTAGAAGGATTTCAAATACCGTGCTTACTGTTCAT
CGAGTAACAATCTGTATTATCTGTGCACATTTGCAGTCTATTTTCCCAAACTATGAACTGTACCGTACTTAGGTTCTGTGCATATGCAGCTCGACATTTT
GGGTGTTTGGTGGTTGAATGAACCTTCACCCTTCAATTTATCTGTAGTTATTTGTGATTTGAAGCATCTTCTTTATATCTGTTCCACACTGCTGCCAATT
TGATTATTGATATAATAAATTAAGCTTTCAGATCGTATAGTAGAAATACACTATCTTTTAATGCAATCCTGGCTTTGCTTGGTATTTTATCTTCTCGAAA
TAACTTGTGTTCATTGGGATCTATGTCAGTGCTGTGTCAGCATCAGCTTCGACTTACTGGCAAGCTTCAGGGCAGGCTAAGCAAGGCCCCTAAGATTTCA
TCCGGACTACAGCTAAAGTGAAAAAGGGTTTATTCATGATAATTTCAATCTAAGACCTGTTTAGTTTCTATTAAGGATGCTTAAGTATACCAACATTTTT
TTTATCTTTCTTGCCAAATCTGCAAGAAAGCTTGGTCAAGAGAATCTTGCCACAACTCCCTTTGAGATGCCAAATCTGCGGTGAAGCGGAAAATTATAAA
AAACTTTTGGCATTGCTATCCTGTGTCTAAGAAACAAACATCATCCTAATTTGGTGGAATTCGCCTGATGTGTTGTAGCAAACTAAGGGGAACTGTCTGT
CAATACACCTCAAGTGCCTATATGTAGACCGGACAATATTTCAACGCCTCTTAATTCAGAGCAGT
>Locus_21273_Transcript_23/23_Confidence_0.083_Length_1325
TCCAATTATATCATTCGTTCTCGAGCTTAGCAGCATCTAAAATCTCGATACAAATTAGTCCTCTCTATCCCAAACAACCCTTCTAAATAAACCACAAACA
ACTCATGTAGCCTTGCTACGGCAAGCCAGTAACTTTGCTTCGGGGGTCCGTACCATTTACACTTTCTTCTTCCTAATTTTTGCTCTGCCCCCACCAGAGG
AAGGGACGGCGGGGAGGGGGCAGGCACAGAGAGCAGCTTTGCTTGGCGGACGCACCGAGGGAGGCGCCTGGGAGCCCTGCTTCTCCCCTCTTCGTCTTCC
TCCTCCGCTTCAAAGGCTTGTATATCATACCCATACAATGATTCGGGCTCAGGAGGTGTTTGGGCAGCCTTTGGGTCACGCACTAGCGCTGCAGCTGTGT
TCCATCCCCAAATTGCTTGTGGGGGCACATCTGCGAGAGTTCCCTTACCTCTGGAATTAGCAGATGATGAGCCCATATATGTCAATCCCAAACAATATCA
TGGTATACTTCGCAGAAGACAGCTACGTGCTAAGTTAGAGGCTCAGAACAAGCTAGTCAAAAACCGAAAGCCTTACCTTCATGAGTCTCGGCATCTTCAT
GCAATGAAGAGAGCAAGAGGTTCTGGCGGACGTTTCCTTAATACTAAACAGCTCCAGGAGCAGCAGCAGCTGAAGTCTCGCAATGCCTCCACCAGGTCCA
CCACAAATGGCGCAAATTCCTCAGGTTCAACACATCTACGGCTTGGTGGTGGTGCAGATGGAGATCAAACCATGTCGGGGACGAAAACAATGGCCTCACA
AAACAATAGCAAAAAGGCTGTTTCTTCTTCTGCAGCTCTTGCTTTCACCGTGACTCCTATGGTGCGCAAAGATGACACCTTCTTCCAGCACCTCAGCCAC
AATGTCAGCTTCTCCAGCCATTTTGGCCAGGCAAGCGCCCAAACCGGCGTTGGAGGCATCCATAATGAGACCCAGCATAGGGTTTCCGTGATACAATGAC
GGTTTGCGAAGCTTATCTGGTGATCCAGGCTTCTAGGTGTCCTCGTGTCCGGTGTAGTCTTGGTCGCTCAGGCAATTCATCCTTGGCTTAGTTTTTGGTG
TGTTAGAACCTACAAATGTTTCATACTCTGTTGTGGTTTGCAGAGCCCATAAATCAGGGCTCTCCTAAAAAAAATCAGGGCTTGATGGCGACACTACTAT
CCAAGTATTGTTGTAATGGTGTGTGTTAGAAACTCGCAAAAAACCCTTCTTTGTGCTCGTATTAGTACATTATGCTGCACAATTAGCTGCTATTCTGTTC
GTACTACAGTCTGGTATGCAGTGGT
>Locus_2366_Transcript_123/132_Confidence_0.128_Length_2917
AAATGAAAGTATCAAGCAATGCTATTAAAAGTTCAAAACCGTGTAACAAGGAGAAGATATATCTTGTTCAAACTATAGAATTGAAGCTACACTTCCGTAG
ACAAAGATACATCTGAACAATTCTTTTTCTCCATGAACAATGAAACAGAGATACTAGAACCGGAAAAAATAAAAAGGAGAAAGGCCTCTCTGCTAAATTG
AAACGTACATATATGAAATGAAGGGCTTAATCATAATCAGAATTCAACTCATACATCCACAGCAGTGAGTGAGAGTCACTGCAAAAACACCAGGAAAGCA
GAGAACACACAGGCCAGAGGAAGCCATGACACTGCCCTGCTAACATGGCATGCCCCTGACGGGAAGAACCCTGGGTCCTGAGGATACACCGTCATCGGCG
GCGGTGGCGACCAGGGGAAGGGCATCGTCGAGACCCCGGCCGGCCGCGTCGGGCAGAACGGGAACGGCGTGGAGGTTGCCGGCGGCAAGAATGCGTCGTC
GTCTGCGATGCATGTGGCTGCCCACAACGACACGGCCGCGACGAGCAGGATTGGCCGTAGAGATGGCATCTTTGCCTGCTCGCCGCCGCCTCGCTTTCAC
TCGTCGGAGCTTGTGGTTACTCGATTCTAGATGACGTCCGTCCTTAATCCGGCGTGTCAATGGCTAAATAGTGTGGAGATGGAAATGTCGGCTGGGACAC
GCTGTTGGCCGTCCGATGCCCATCAGAGGGCTGTCGGTGGCGTTGATGCGGTACCGGAGGCTTTTCTCTCATCCGCTTTCCCTTCCTTTCCCTCTGATCC
CGGAATGCTTCATAGCTGCTGTTCATGAGTTCTGTCAGAATATGAGCACAACTGTAGGAACGAGAATGGCGACTAGAGGGGGGGTGAATAGACGCCTTAA
CAAATTTCTTTCGAAATAGATAATCTTTTCCTATTTCACACCCAACGCACCTCAAAAAGCTCAAACGGAAGCGAAAGAATTAAAGAGACAAAGCAAGAAG
ACTACTTCCATGACAACTTGACTCCACAGATGAAATATGAATCATAGGTTCCATTCAGGACCATTCCATGTGTCTTTGTGCACAAAAACTTGCAACTTCC
AAAGAAACTAGATAGGATAGACACCGGACAAGGTTATCCTCCAAGATAATAGTCTAGTGGCAAGGGGACTCGAGACCTGCTCAAATACATGAGTATGTGA
GTATTTATGCCACTAGCATCAAGAAAAACAACCCTGCAAAGGTGAAATATTGCAACTTAAAGAAAAAGATCACCGGGCAAGAAATCTCTCCAAGTTGATG
ATCTAGAGGCAGGGGTACTCAAAACCCATGAGCATACACTCGTATGTGAGTTTTTAGGCTTCTAGCAACAAGAAGAATGATCTCACAAGGTGCTGAAATT
TCAGTCCGAAAATCAAAACGAAAATCAAAACCAAAAATCGAAAAGCTTGCAAACTTGCAAGGGAACCAATAGAATAAAACTAGAAGGAGGGGAATTCAAG
CATATGCAAAAATATAAACTAAATTACTTAAAGAGGTCAGCCCTATTTTAGGCGGATTACAAAGATTTATCTCTCAAAACTCTCACCTATTACATAGGCT
AAGCACTCATCCTTCTCTCCTCTAAAAACCTATCTCTAAGGCTCTCAAATGAGTAGCACAAGAGGGCTCAAGTGGCTGGTTCAAAGCTCTTTCTAGCCCT
ACCCCTCTATTTATAGGACTAGGAAACTTGGCCCTTAAGTTTCCTAGCTTTTCCCCAAAATACCCCTCTTATAGTGCACTTCTACCTACCACCAAGAGCA
TTTTGGTCCATTTTCTTCTCCATTCATCGGACAGTCGCGGCGCCTTCACGACTTAGCTTCGCCTTGACGCAAGCTTCGCGATGGTGCCACATACTCATAC
AGTCCTCACACGGTTTTGAGGCCAAACCGCGAAACCACCTGTACGCTTCTCAAAGCGTGACTCACCGCTTTGCTTACACCTTAAGCAAGCGCTTCGATGT
CGACGCGTGTACTCCGTCATGCGATCCTGACCGCCGGCAAGTCTCTCCCGCTCCCGATCCCTCGGGCCATCTTGTCATTGCACCGGCATCCCTTTCGCTT
GACTTTGTCAACACGCCATCTTCATCTACCTTCAATGCTTTACTTGACCTCCACGTGTTCAGTTAGGATCACCCTTGACTCCGCCCGGCCTCCTTGATCG
TCTGGCACCAAGCACTCCTACTTGGCCCCGATCATCCCGCCGTCGATCACCAAATTGCATCCATCACCTCAAGAAACCATTAATTGAGGCCTCGAACCCT
ACCGGTCGATCGGTTAGCGATGTGTAGCTCTCCAAATTCACGATCCATGCAAGCCCATCGACGACGTTGCGCGGGCGCCGGCGAATCGATAGACGACGTT
GGCTCGCGTGGAGACCGATGGAAGCGTAGGTACGGAGGCGCAAGTGGAAAATTCCAAAGGGATTGCATTGATCTTGTCCTCACGCGCAGCCATGCACGCA
TCCATCTGTGACATGCATATATACGTGTGCTTCCATGCCGGCGGCAGCGAGCTGATTAAGCATGCAGCGCAGGTGGGATCAATGCGATCCCTTTGGAGTC
CTCCACTAGCTTCCTGAATGAAATTGAAGAAGGAGAAGAAGAAGAAAGCGCCTAGCTCATCACTGACAAAGAATAATTGCTGCTGCTTACTTTCTGATAT
ATGCATGCCTGCCATGGTAGTAGCTAGCATAGCTAGGGGAGAGGGGTTTATAATTATAACGCCATGGATTGGATAGGAAGAGAAGAGCTCGCATAGAGAC
ACACACAAGAGGTAGAGAGAGACGAGGGAAGGGGAGGGGAGGCAGGTGAAAGCTACATGGCAGGCAGGTGTTGGGGATGGCTGTTGCTTCGCTCGCTATA
GCTGCCTCCCTTCCCCT
>comp78295_c1_seq32
ACAACTGCACAACTTGCAAGAAAGCCACAAGCAACTAATGGGAGAGGAGCTTTCAGGCCTAAGTGTGAGAGACCTCCAAGGTTTAGAGAATCGTCTTGAA
ATGAGCCTACTTAGTGTCAGAATGAGGAAGGGCCACCTTTTGACAAGTGAAATTGAAGAGTTACATAGGAAGGGCAGCCTAATTCATCAGGAAAACACGG
AACTCTGTAGAAGAGTGAATATCATGTCACAACAAAAAATGGAACTTCATAGAAAGCTCCTGGCCAGTGAACCAGGAGGTGTTGCTGATGCAAATAAAAG
CTCTAGCACTCCCTACGGCTTTGGTACAGCACAAGGCGCAGATGTCCCAGCTAATCTTGAACTGAGCCAGTCATCACAAAAAGAGGGAGAGCAATGCAAA
ACAGGGGCCCCGGAACTGGGACTTCAGCTGCACTAAGGAGAAGCAGTACAAGCTGGACAATGCCATAATGGTCCACAAATTTCACCATGCGATGCAAGAG
CATTTTGGTTGCAATGACTATTTTGTGCAATATACTAGCTGGGTAAACAAATGGGTTGTGCAAAGACTACAGATAGGACCAGTTATGTTATCTGAGGCTC
TGCTGTGTCTAAAATGCAACATATTCGTTATTGTAATGACACATGATAAACTGACTGTCCTACCGTTGACCTTGAAGATAGCTCTGCAGTGAAAAGGTGT
ATTATGGGCTTGCCAATATTAAAGAAGTGTTAGGGATCAACTAGTAGGATCTCACAAAGAAGTAATTAGTCCACATTAGTGCCAGCTGCTGGGGATGATA
TCTGACTCAGCCTGCCAACCCTCTCTTAGCCATGGTGTTTCAACTAGTATTAGAAAAGAATGTTCATCCAATTGAGTTCCCAAACAACGAAATTTATATT
ATGTGTTGCAAAGTGTCGACACCAGTGGAACAGAGTCCATATATGTCAAGCAAATGCCAAATGTCAATTTTGCTCCAGTGCACGTGGATTCTGTTCTATT
CTCAAG
>Locus_839_Transcript_98/261_Confidence_0.054_Length_1125
AGTATAGAGAATTTCCATGAGATGAGACGTAAGGAAAATCTCCACGATATTCTTCAATTTGAATATTGCGAGTACTTGGTCCTTTTTCATGTCTTTAGTA
GTTTAGTTTCATGCACCTAGAAATTTAACACCTACTGTGTCCATGTAGCAGAAAATGGCACAATAATGATTCAATTTGGTCATCAAATGCCTGATTGCGA
CTCATCAACTACCCAATCAACCAGCGAGAGTCATCAAGAAGTGTCTGGAATGAGTGAAGGAAGCCTCAACGAGCATAACGATCGATCAGGTAATCTCGAT
GGTTACACAAAGAGTGATGAAAATAAGATGATGTCAGCTTTATCTCTGGGCAATCCAGAAACTACTTATGCGCATCCAAAACCTGACCGTAGTCAGCCCT
TTGCCATTTCGTACCCGTATTGTGATTCATTCTATGGTGGTGCAGTGGCAACGTATGGCTCACATGCTATTATGAATCCCCAGATTGTGGGCATGATGTC

GTCCTCCCGAGTGCCATTACCAATTGAACCAGCTGCAGAAGAGCCCATTTATGTGAATGCAAAACAATACCATGCGATTATCCGAAGGAGACAGCTCCGT
GCAAAGCTAGAGGCTGAAAACAAGCTGGTGAAAAGTCGCAAGCCATACCTTCACGAGTCTCGGCATCTGCATGCCATGAAGAGAGCTCGGGGAACAGGTG
GGCGGTTTCTGAACACGAAGCAGCAGTCAGAGGCTCCAGGTGGTGGCACCTCAGATGCGCAACACATGGCCGGCAATGGTGGTCTGTTCAGGCTGCACGA
GCACAACTTACCACCCAGTGATCTCCATTATGGCGCGAGAGGTGGCTCTTAAGGAGTCCCCTGGCAACTCATCCTTGGCTTAGTAACGTGTGGCGCTCAG
CGATTCGACCTCGGCTTCGATGTAATGGTGTACGATTGTATCTATGGTGCTTGAATTGGCAGCAGCCATTCCTGCAGCAATGCGTCGCAGGTGTTGCCGG
TTCTGTGATAGTGCCAGACTTGTGACCGGCACAGAACTGGCATCTGTTTCCTGTAACTTTAGTTTTTCTAAGACGATGACGATGACTACCCAATAACCAT
TCTGTCTGTATGGTGCCTTGCGTTT
>Locus_17813_Transcript_4/20_Confidence_0.067_Length_226
ATGACTACCACAGCTTCGGTCACCTTCGGACATCCTTCTTCACCCCGCTCCCGAGCATCATGGACGGTGAGCATGGGGTCGGTAACCCCTTCAAGTGGGC
AGCAGCCTCTGAAGGCTGCTGCGACCTCCTCAAAGCATGACTAGAGGGGATACCGGGCAGCCGAGGGTCAGGGATCGTCCCTGCCATCGTTTGGCTGTCG
GTAGGCAAATCATTCTTGGCTCAAGG
>Locus_10795_Transcript_58/70_Confidence_0.408_Length_2732
ACATTAATCTGAGATCTGAACAATTTTCAAGTACCACATCAAAAGCTTCTGTTGAATGTTTATTTGAATGGAACACTTGAATGCATATCATGAATGTGGA
GGAATTGGGCGGCCAAGAACTAGGCAGACATCACATCCCATCTTCCTTCACGCAACTTATGTCTAGCAGTCTAGCTAGAAAAAGAAAAGAAAAGAAGGGG
GAAAACAAACTATGTACAGAGCAAGGCAAAAGAAAAGAAAAGAAGGAAAAAAAAACTATGTACAGAGCAAGGCCATCATCTCGGCATGGACCTCCGGTAG
GTGCTCAATTACTCAAATCCTATTGCAATCCTGTAAAGGATGGCTTTATTCTAGATCTGCATAGGTTACCACCTGCAAGAAAGCCACAACAAACAGCAGC
AGCCGAACAGATGCTCACCTCCCTACTGCGCTAACTCAAAACTGGCAAGAAGTGCCCAGCCTCTGCTTCGCCCTTTGCTCTGTAGCGCAGCACTTGCGCG
AGCGAAATGCCTCAGGCAGCAGCTCCTGGAACTTCTCAAGGAACAACCCCCATGTCTCTGGTCCCAAGTCAGCCAGCTTGACAAAACTCACACTTCCTGG
GAGCATATCGTTTGCACTCCCCCAGTTACCAACTAGAGAGCCAGTTTGGTTGCTGTTGCTCTCAGAAGTTTGCACAGAATTCACACCCTTTGGAACAGCA
GACAACCGACGCTGGCTTTTGTCAGTTAGAGTGACACCCTTCAGTGAGGTTGACAGCTTCGAAATGTTAGCAAGCCACTTATTGACAGGTTCAGGGCAAG
CTAACTTTCCTGCATTGCTTTCTACCTCCTCCTCAATGTCGTTGTCGTCGTCGTCATCATCATCATCATCATCATCTTCTTCTTCTTTCTTCTTTGGCCT
CATCCAGTTTAGACAATGAGGTTCTGGAGGTTTGCCCCTCCCCTTACATTCAAAATGGTAGGCTGATGGTTCTTTTGGCATTTCATGATCTTCACTATCG
AGTTCAAATTGAGTTAAATTCTCATTGCCAGTGTCATGTTCTATGGAACAGTCTTCTTGTTCTGTTGCTAGCCTCCTTGCCTCCATGCAGACAAACTCTA
GCTCACTTGGCTGATCTTCCATCCCAGTAAACTCTCTGCTCATCATCTCCCCAATCTCTGCAAGGCAGAGACCAAATGCTGTGGCTTCTTTCAGAAATAT
GGTTGAGAGTATTAGTACTCTGAGGCATGCTTCATGGATCATAGGCAACTCCATACGAAGCATGTCAGAATCTTTGATTGGATCAAGATTTGTGATGTAC
TCAAGCTCATCCTCGGAGAATGGGATTGATGCCTGTGGCCAGTGGATCCATTCAAAATAGGGATCCTCCAAACACTCTGGGAGGCAGAGACCATGATCAA
TAGGAATAAGCTCAGTCTGATTCCCAAATTTTCCTGCTCCAGTCTGCTTCCTTACCAGAAGATTTCCAGCATGTCTATCAGTATTGAAGATCCTGATGTC
AAGAATACCAATCCTATGAACAGCAGAGACAGGAAAACTTGAAGTGCCGTAGTCACTGGCATCAAAATCATGAGGAATAAACTGCTGAAATGATGCAATC
TTGCTAACAGCTTGTGTACCACCATCAGCAGCATTCTTGTTAGCACAATTGACGCTTTCATTCATATGAAATACAGGATGAGAAATTTTAACAAGAACCG
TCGGAGGGACATTAGCAGAGTTATCATAATCCAGAAGGTAGGCAGCAACCTCTCTGAAACCAGTCTCCCCGACTCGAACTGATCTCTTGAGACCTGGCTG
CCCAAGGGCTTTCCCTGTAAAACCTTTCGGATTGTTGGGTGCAAATGGCTCCTCATCATTTGGCTTCACAATGGCAGCATTCTCACCTTTGCTATTCCTG
AAATAGTAGGCACCACCAAGCCCACTACTGACAGGTATGGGATCCACACCGTTTCTTATAGCCCTAGTAACATCCTTCACAAGCTGCCTTGTTCTAGAGC
AGCGATTCGGGCATACTAACAGTTCAATTGGACTACCCCTATCTCTTTGCTGCTGGAGATCCTTGCCTGTAGGAGATGACATGCATGGTGTGGAGGAACT
CCGATGCATGAAACTCTTGGTGAGAAGTAACGGGGAGTCATTTCTGATGGTGCTGAGGTCATTTTTTAGGACACGATCACCTAATGTCAGAGAACTCTCC
TCGGTGGGAAAATTGAAGGCCACCTGCAACTTTCTTTTGACTGTGTGTGCATTGTCGCCACGATCTAGTTGAACAGCCAGGACACAACCAGTGTCAGTTT
GGACAAATAGGCGTTTCCATCCGCCAGCCCTGGTCTCGCTTCTACTTTTGGTAGGGTACTCAGCACTAAGAGCTCGGCCCAAAGCTGAAACTGCCATCTG
GGTCTGCACTGGGCTCTCCAAGTTGCGAGACATGGCGGACAACGACAAAGGAATTGAGGCCAATGCACTAGAGGCAGCTGCCACAATGAGGCCTTTCTTG
CTCCTCTCTTGTTTTCTTCTGCGCTCGTCAAATTCAGCAGACGAGTGCGAGAGGGATGTCACGGCAGTGATCATCAGGCAATGAAGGACATCTTGCACAC
TAGCTAGATGGCACCAGAAAGCGACAATAAATACGGAAGGTGAAGACAGCAGCAACCCCCTACAACTGGGAGCCGTTATGAAACCCGACAAGCCTGTTTG
CCGATCACTAGGTCCCGTATCTGATCCAATGC
>Locus_17579_Transcript_8/92_Confidence_0.044_Length_745
TTGGCTCATCTATTAACTAGAGTACTAACTGAATTTCCCATTGCACTCTTATCTCTCATAGTTGAATTATAGCCATCATAAGCATGTTCAATACATTTGC
GAATTTTGATTAGTGATGAACATAATAGTAACATTTTCCCTATCTCTGATCCTTTTTCAGGCGTCGAAGCTTCTGCGGTCGTCTCCAACTCGAATGGAGC
CCCAGATCTTCAGCGTGCTCTCTCTCTTCTGTCAAACAATTCAGCTGGTGCTGCCAACGACCAGCCAACTCCTCAGCTGCACCCTGGGCTGACCACCTTC
GTCGGCTCCTCCAACCCTGTCACTGTGATGGAAGCCTCACCACCAGGACTCTGGCAAGATGGCGCCCCCCTCGATCATCAGGCGCGGTTTCAGCTTTTCG
ATCCGGTGGGTGACGGCAGCGCCATTGTCACACCGCATCAGCTCCAGCTCCCAAAACCTCACTCCCTCAACAGCTCCTCTTCCCACTATGACCAGATGCA
CTGATGATCTGATGCTTCCTTCAGTTAGCGCAAATGGCGGTTGGGTTGCGCAAAATTAGACATGGCTTCTGATGCCTGAAACTCTGAATTTTGAAACTGT
TATGTTCTTCTGAAAAATAAAAGCCTGTGTGCGCAAACTCAGGCTCCAAAAACTGATCTAGTAGTGTTCCCATTCAAGGGCTTGAATAATCCATCCTTGT
GGTGGCATGTAACTCAAATGCTTCGATTGCGCCGGCAATTGATCT
>Locus_839_Transcript_91/261_Confidence_0.050_Length_848
AGGTAATCTCGATGGTTACACAAAGAGTGATGAAAATAAGATGATGTCAGCTTTATCTCTGGGCAATCCAGAAACTACTTATGCGCATCCAAAACCTGAC
CGTAGTCAGCCCTTTGCCATTTCGTACCCGTATTGTGATTCATTCTATGGTGGTGCAGTGGCAACGTATGGCTCACATGCTATTATGAATCCCCAGATTG
TGGGCATGATGTCATCTTCTCGAGTGCCATTACCAATTGAACCAGCTGCTGAAGAGCCCATTTATGTAAATGCAAAGCAATACCATGCGATTCTCCGAAG
GAGACAACTCCGTGCAAAGTTAGAGGCCGAAAACAAGCTGGTGAAGAGTCGCAAGCCATACCTTCACGAGTCTCGGCATCAGCATGCCATGAAGAGAGCT
CGGGGAACAGGTGGGCGGTTTCTGAACACGAAGCAGCAGTCAGAGGCTCCAGGTGGTGGCACCTCAGATGCGCAACACATGGCCGGCAATGGTGGTCTGT
TCAGGCTGCACGAGCACAACTTACCACCCAGTGATCTCCATTATGGCGCGAGAGGTGGCTCTTAAGGAGTCCCCTGGCAACTCATCCTTGGCTTAGTAAC
GTGTGGCGCTCAGCGATTCGACCTCGGCTTCGATGTAATGGTGTACGATTGTATCTATGGTGCTTGAATTGGCAGCAGCCATTCCTGCAACGCGTCGCAG
CTGTTCCCGGTTCTGTGATCATGCTAGACTTGTGACCGACCGGCACGAAACTGGCACCTGTTTCCTGTAACTGTTGTCTAAGATGATGATGACTACTCCA
ATAACCATTCTGTATGATGCTTTGACGTCGGTGTTGCTCCTTCAAGTC
>Locus_839_Transcript_110/261_Confidence_0.052_Length_1352
CAGTGCAATTAGACTAAGTATAGTTGGTGAGAAGACAGGAGTAGAGATAAGGGCCAACAGCCCAACAGAAAAACCAAGCAAGACTGCAACTGCAAGAGAG
ATGGGGAGAGAGAGGAAACCTTGCAGAGGAGAGAAGCAAGGTGGGAAAGAGGCAGCAAGCGAGGCGGGCTGCAGGAAGGGGAACATGCTCCCTCCTCATC
TCACAGAAAATGGCACAATAATGATTCAATTTGGTCATCAAATGCCTGATTGCGACTCATCAACTACCCAATCAACCAGCGAGAGTCATCAAGAAGTGTC
TGGAATGAGTGAAGGAAGCCTCAACGAGCATAACGATCGATCAGGTTTACTGTAGTCCAATACCCATCCTGTTGTATTCATACATAATGTTGTCACATCA
TATCTCTTGCAAACAAAAAAAAGGGTAAAAAAGAAAAAGGGATTTTGATAGTTGGAAACCTTTTGTGCACATGTACAATCGAAGTTGTGACACTCTTGTC
CTTGTATTAATGCTAGGTAATCTCGATGGTTACACAAAGAGTGATGAAAATAAGATGATGTCAGCTTTATCTCTGGGCAATCCAGAAACTACTTATGCGC
ATCCAAAACCTGACCGTAGTCAGCCCTTTGCCATTTCGTACCCGTATTGTGATTCATTCTATGGTGGTGCAGTGGCAACGTATGGCTCACATGCTATTAT
GAATCCCCAGATTGTGGGCATGATGTCGTCCTCCCGAGTGCCATTACCAATTGAACCAGCTGCAGAAGAGCCCATTTATGTGAATGCAAAACAATACCAT
GCGATTATCCGAAGGAGACAGCTCCGTGCAAAGCTAGAGGCTGAAAACAAGCTGGTGAAAAGTCGCAAGCCATACCTTCACGAGTCTCGGCATCTGCATG
CCATGAAGAGAGCTCGGGGAACAGGTGGGCGGTTTCTGAACACGAAGCAGCAGTCAGAGGCTCCAGGTGGTGGCACCTCAGATGCGCAACACATGGCCGG
CAATGGTGGTCTGTTCAGGCTGCACGAGCACAACTTACCACCCAGTGATCTCCATTATGGCGCGAGAGGTGGCTCTTAAGGAGTCCCCTGGCAACTCATC
CTTGGCTTAGTAACGTGTGGCGCTCAGCGATTCGACCTCGGCTTCGATGTAATGGTGTACGATTGTATCTATGGTGCTTGAATTGGCAGCAGCCATTCCT
GCAGCAATGCGTCGCAGGTGTTGCCGGTTCTGTGATAGTGCCAGACTTGTGACCGGCACAGAACTGGCATCTGTTTCCTGTAACTTTAGTTTTTCTAAGA
CGATGACGATGACTACCCAATAACCATTCTGTCTGTATGGTGCCTTGCGTTT
>comp79373_c0_seq31
GAAACCTGGTAAACCCTGGCCACGGACGCGCATGCCATCCAAGATGACGTAGTCCACACCGAATCTTCCACGTCCGCACACGCGAGCACAACGCCACCGC
AGGCCAAGTGCCGTCTCCACCACAACTCGCTGATCCCTTCCTCCTCGCCGGCTCTCTCTCCATCCCTCGTCCTTCCCCATCTGTCCCTCCCTTGGCTTCT
TCCTCCAAGCTCCAACCAATCCCCACGCGCGCGCGCGCTATAGCCTACAAATAAATACTCGCCACTCTTCCCTTCCTCCCGCCTATCGTCTTGCCAAGCG

CTAGAGCAGCTGCGCCATTGCACGCTTCCGGCCCGTCGTCCTGCACATCTGTCCTCCCCGTACTGCCTTGTACAAACTCCGTATGTAAACTACTGGGTTG
TCGCCAGTGTTCGTGTTTGCTGATGCTCATCTAATTATCGTCGAGGAGATCCAAGGAATTGTCCGCGGAGCGCCGGGGAACAAAGGCTGCGCGCGGTACT
ATATCAATTACAAACTGATGAAGAAAATGTTAAAGCAATATGTTCAACAGACCCAACTTGGTGGGAAAGATCATGAACAAGTTCTTAAAGAGTTCTCAAG
GATTCTTGATGACCAGATTGAAAGGATTGTGCTTTTTCTGCTACAACAACAAGGCCACCTTGCCAGTAGGATTGAGGAACTGGGGGAACGACGTGCTCTT
CTGAAGCAGTATGATATATCACAAGTTTCTCAGCTACGCGATGCATATAGAGAAGTTGGGCTTGATCTTATAAAGCTTCTCCGCTTTGTTGACATGAATG
CTACCGGTATACACAAGATATTAAAGAAATTTGATAAGCGCTTTGCCTATAAGTTTACAGACTATTATGTCACCACTCGTGCAAACCATCCTTATTCTCA
GCTTCAGCAAGTATTTAAGCAAGTGGGAACCCTAGCTATTACAGGTGCCTTGTCCTGCAACCTTGCATATCTGGAGGATCATCAAGGAGGCATTTTATCC
ATCTATGACCACCCATCAATTAACTTCAGGGACCCTATAATTAACCAAGTAAATCATGCAGTGCAGAAACTCACACATGCGACAAACTTTATGCAATTCT
TAGGACAACATGCAGTTATCATCCCAGAAGATGTGCAAAGCGGGTCAGAGGATCTTGTCAATGATCAGAACTACCATTTCATGTCTCTTTTGCTTAACCT
ACTGAACACGTTTCTTTACATGGTGA
>Locus_5523_Transcript_58/107_Confidence_0.118_Length_825
TCTTTGTTAGGGAAGCACCAAGCACCTCAAGGCTGCGAACCAATCTCAAAAGGGGATACAGGATAGGATAGCTTCCTTCCTTCCTGTAAGGAGGATCAGA
AAGAGAGAGATAGGGAGGCCAAATCAGGCTTCTTCAATTAGTTAGAGTAGAGAGAGATCTGTGCTACCTGTGGGGGAGAAGATGGGAAGAGGGAAGATAG
TGATCAAGAGGATTGACAACACGACAAGCCGGCAGGTGACGTTCTCCAAGCGCCGGAGCGGGTTGTTCAAGAAGGCCAAGGAGCTCGCCATCCTTTGCGA
CGCCGAGATCGGACTCATCGTCTTCTCCAGCACCGGTCGCCTCTATGACTTCTCCAACACCAGCATGAAGTCTGTGATCGAAAGATACAATGAAGCAAAA
GAGGATCATCACCTGACCATGAGCGCAAGTGCGGAGGCTAAGTTTTGGCAAAGGGAGGCAGGAAGCTTGAGGCAGCAACTGCATAACTTGCAAAGAGCAC
CATCGGTCAGTTGTTGGGTCAGCAGCTTTCTGGCCTTGATGTAAAAGGCTTGAAAGATTTGGAGAATAAGCTGGAGATGAGCCTAAGAAATATTTGTCTG
AAGAAGGTTACATTGCAATTTGTTGAGACCATGTCATCATATAGTTCTTTCAGAACATGGATATCATTACTACTTTTGGCTATTTTTCATTTTACAGGAC
CAACTTATGATTGATCAAATTCAAGAATTAAACAGGAAGGGAAGCCTCCTGCACCATGAAAACATAGAACTATACAACAAAGTCAACCTTGTTCAACAAG
AGAACATCGAATTACAGAAAAAGGT
>Locus_14326_Transcript_48/49_Confidence_0.295_Length_2957
GTATTCTCATTTCCTCGCCGTATTTTCTCTCTCCAGCTCCAGACTCCAGAGTCCGGAGGAAGCAGAGAGAGAAGCTCGACGGGAAGGTAGAGAGAGATAT
AGTGCTCCTGATCCCTCTTTGAAATCCTCGATTGGTCAGTTCTGTTCCTGGATATACTGTTGCTGCAGATTGGATGCGTCTGCTTTCCTTTCGGGGAGTG
GTGTCTAGCTCTGTTGGTTCTTGCCTTCATAGTTAGTACTATGCCTTAATCTTGCTCCGCGATTGGTTTGATTCATGTTTTTCCTTTAATCTTGCTACCC
CAGGATTTTCCCCTTTGGTTTTCTTGGTTTGCTTCTCGGAAATCCGTGATTTTAACAGGATTTTTGGCTGGTGTTGCTCGATTTTTGTTCTTGACTGTGC
TTCCTTGGGATGAGCTTTTGCCTAATTCGACATTACAAGATGTGTTCTTTCTAGAAGTTAAATTGATCTTCTATGAGCCGGGCGCTGTATCGTGCATTAC
ATGGGGGTGGCGTACTGGTACTTTGGGCCGTAGAGTTGATGGAAATCTGCAGTCCCTTTTGTTGGATAAGGGGCTTTTTCCTTGGATAAGGAGGTTTGAA
GCTAATTTTGTGCGAATTGTGGCGGTTTCTGGATATCCTGGTTGAATTAGTGGTGTGATAGATTGTTCTAGAGAATAGGAAGATATATGTTTTATTGTTA
GAACATCAAGAGAGTGTTGAATCAGGTATGTCAATGTGTGAACTTATTGATGGGAGCTAAATGCTTGCTGACGTATCTTCGCTATAATGACCTAATCAAA
CCTAACAAGCACTAAATACTGATCAATTCTCAGTTATACAAAGATTTTCCAGAGAGTTCTTCAGAGCTCGAACTTCTGGGATGAAAATAGTTTGGCTCAA
GATAGTCCACTGGAAGGAGTTTAGGATATCTGTTTGAGCTCTGCCTGCAATATATGGAAATCTGAAGGTGATATGGTAGTAGAGTTGGGGACCCTGATCC
CCAAGCCAAAAGAAGTTCGGTAGAATTTCTCAGGAATATTGTGGTAGTAAGAAGCAAGTTAAAAGGAACCTAATTAGAAAAAAAGACATGCCTTGCTCGC
TCATGAGAATATAAAACAGGTATGCCACCTCCAGTCAGTACTTGATTAATTTCATTGAAAATGGAGGGCTGTAATAGATCCTTATCTATTAATGTGTTGT
ACCTAAACTTGGAAATCATCAGCTGAGGGCATTGTGCATGGTGCGTGGAGTACTAGCCCTCGGGAGATGCTTCTTAATTAGGCAGCGAGAAGGATAGAGA
TCGGTTTGACATATGTTCCATTTGTAGCTGTACTGGATATTTCTGCATGTTCCCCTGGAGCAGTTAGTTTCAGAATTATGATATTTCTACTTGTGGCAGG
ATTGTTCTGTTTCTATTATCTGTTCATATTTATGCTAGTCTGTCTTTTATGTTGTAATATCTATTGTCTATTGCACTTCAAGTGACTATGTATAACCATA
TTCCAGTGATTCAGTGAATGATTATGAATTAAAGTAAGAGGACGTTTTCATCAATTACCCTTGTTCTTTATCATGTCATTATCAATTCAGCAGATTAAAT
TCTGGCAACTTGTCATGATGGTTACTTTAGGTGTCAATTCATCCGATTTATCAGAAGTACCTCAAGCAAAATGTTGCTCGGAGCTAAGGATACTATCTCG
TAGTGCTCACGCTTTTGTGGGAAATGGAGGATCATCCAGTCCATCCCATGTCGAAGTCCAACAATGGCTTCTTGTCAGGAAATGGCTATCAGATGAAGCA
ATTTGGTCATAAAATCGACGATAGGGACTCGTCATCAGAGTCCGGTCGGTCTCACCAAGAAGCTTCCACAGCGAGTGAGAACAGTATAAATGAACAGCAC
ACCTCAACACAATCTGACAATGATGATGGTCATGGGAAGCATAATGAGGACATGATGAAGTCAGCAGTGTCCTTGGGGGAACCAGGATCAGCCTTTTTGG
CCCCAAAACTAGATTACAGCCCATCTTTTGCTTGTGTTCCTTATACTGCTGATGCTTTTTATGGTGGGGTCTTGACAGGATATGCTCCACATGCCATTGT
TCATCCCCAGCAAAATGATTCAGCAAACTCTCGGGTTCCGTTGCCTGTTGAACCTGTAGCAGAAGAGCCAATATATGTTAACGCAAAGCAATACCGTGCA
ATCCTTAGGAGGAGGCAGATACGTGCTAAACAGGAGGCTGAGAATAAGCTGGTGAAAGGTCGGAAGCCATACCTCCATGAATCTCGACACCGCCACGCAA
TGAAGCGAGCCCGTGGATCAGGGGGGCGGTTCCTCAACTCAAAGCAGCTCCAGGAGCAGAACCAGCAGCAGCAGGCATCAGGTGGTTCAAGCTGCACAAA
GGCCATCGGCAATAACACAAGCTCCCAAAGTGGTCCCAGTCACACGCCTCTGACACTTGCTTCCTCTGACACTGCAAGCGCTTCGAGGGCCAACCAGGAG
CGCACCTGCTTCCCCTCGGCTGGCTTCCGTCCCACAATGAACTTCGGCGCGCGAGGTGGAGACGCAAAGCTAGCTGTGAATGGCATACAGCAGCATGTTT
CCTTCATCAGGTGAAGCAAGTGAAATGGGGCGCTGCCTGCGGTAACTCATCCTTGGCTTATGAAACTGTGAATGTGTAATGGACATGTAACTTCAGTCTC
AGAATAACCAAACATATGGGAGTCCTCCTGAAGTTTCCTGTAGTTCAAAGAACTGATAGGTTATGGCCTTATAGGTTTGCTTCTGGAGACTGAAATCCGT
GCTTGATATACTATGTTGTACTGAGTTCTGAGGTGAAAGACATCGGGAGAGTATCAGGTGCAAATGGCACAAATGTTGAAAACGGTGCAAATTCATCAAA
ATCTGTACCTAAAAGACTTCAATAAATTTTAAACTTTGCATAGCTCTAGGTGCATTT
>comp82180_c0_seq3
AATATAGGGGGCCCCGCTCCCGCAGCTCGCAGTACCGCGGCGTCACCTTCTACCGCCGCACCGGCCGCTGGGAGTCCCATATTTGGGATTGCGGCAAGCA
AGTGTACTTAGGTGGATTTGACACTGCTCATACTGCTGCAAGGGCGTACGATCGAGCTGCCATCAAGTTCCGTGGCTTCGACGCCGACATAAACTTCAAT
CTCAGTGACTATGAGGATGACATGAAGCAGATGAAGAGCCTGTCCAAGGAGGAGTTCGTGCACGTCCTGCGACGGCAGAGCACCGGGTTCTCACGGGGCA
GCTCCAAGTACAGAGGCGTGACCCTGCACAAGTGCGGCCGATGGGAGGCTCGCATGGGGCAGTTCCTCGGCAAGAAGGCTTATGATAAGGCCGCGATCAA
ATGCAATGGTCGAGAAGCTGTGACGAACTTCGAGCCAAGCACGTATGATGGGGAGCTGCTGACTGAAGTTGCTGATGAAGGTGCTGATGTTGATCTGAAC
TTGAGCATATCTCAACCAGCTTCTCAGAGCCCGAAAAGGGACAAGAACTCCCTTGGTCTGCAACTCCACCATGGATCGTTTGAAGGCTACGAATTGAAAA
GAACAAAGATTGATGCTCCCTCTGAACTCACGGGCCGACCTCATCGGTTCCCTCTTCTGACCAAGCATCCACCAATCTGGCATGCCCAATCTCATCCCTT
CTATTCAAATAATGAGGTTTGATGATACTAAATTACTAAAATACAGGCAATTACTTTCCCAAACATATTGCTTTGGCTCCTAAACCTCACAATTGCAAGC
ATGTTCTGAGCATCACATGGTTCGGATCGAGCTGTCCGGCACCAATTTTGAACGTAATCTTGACCATCTGAGGAACTGATTGTGCAGCCTGAAGGCGTGC
GTTCAAATGTTAGATTTGTGTTACTTTCGTTCATTTTCACTTCTTGTGCCGCTGTCAATTTTGGCTCTAGTCTTACATCACCGCTTCATTTCCTTGGATT
CTATAGCCCGCCTGTTTCCTGTCGATTTCGGAGATATTAATGCGTAGCACAGGTTTCATATGTAGGATCTTGAGTTTTCTGAGATGGCCCATTCTGGAAA
CAGTAATAGTCGCCTTAGGAATAGAGATGTGTTTGATTTTTTTCCTTCAAAAGGAACATCCTAGAGAGGTGACTATTCGACATTAGGTATGCTAATCCAT
CGGTCAACTATACCTGCCTGCGGCTAAGAACCTTTGAATATGTTTCCTTTTTTTATTTATCGTATCTTATAAAAGTAGCAAATACGACTATTATTCAAAC
CTTGCTGAAAACAAAAGATAGATAGGATGTTGCCATCTTGTAATGTAATCATAGAATTGGAATCACTGAATTTGCCATTTGTTTCAACTTCAGAGTTTGG
TCAAATTGAACTCTCGAACTTTTCAACTCCTAAACTTTAAGGTCAGGCTCACATATGTCATTTTCAAATTTTATTGGACGGAAAGTTGAGCTGGACGTTT
CGAGCTCGGCTTTCTATCCAACTGAACTAATGGAAACACTGATTTCATCGCAACAAATTTTCAGCAATAGATATTCAATCTCTTCTTGAATTCATCCTTC
ATCAGTATGGTGAATAAATTGTTCTTGGATTATGCAGAATGCATCAAGAGATCTTAACAGGAGGCCAGATCAGGGGGGTGTTCCCAGCTGGGCGTGGAAG
GTGACCGCCCCTCCTCCCACGCAGCCATTGCCGCTGTTCTCGTCGGCGTCATCATCCGCTGCAGCATCATCAGGATTCTCCAATACCGCCACGACAGCTG
CCCACACCGCCCCATCGGCCTCCCTCCGGTTCGACCCGCCGCCGCCGCCGTCCAGCCGCCACCGCTGAACGTTAAGAAGCCACGCTGTAAATTTGCCAGG
AAGCCGACATTTTTTCCTCTCTCGGCGTTGCAACTTTTTAGGTTTTGCGCCGGGGTGGTTTCTTGTAGTGGAGTGGATTCATAATTAACTGTATTTGCAT
GCTGCCCATGTGAAATGCTTCTCTCTCTTTTTACGCTCTCTGCACCGGATGTTCTGGGAGTTCTTGTTGCCGGGAATTAACTAAATTCACCGTCTGAGAT
TGATCTATACATTGTTGTAGAGAATCGAATCATTGTTGAAATTAGTTACAGAATTATAGATATCATAAGGCACCCTCTACTTGTGGACAAAGTTCAGGAA
GATTATTCCGGTTCTTTTCTTGGCTGTCAATTCGGCTCTTGTCCTTGGTGTGGAGCCACTTGCCTACTGTCTTGTTCTGTTGCTGTTTGTTCTTGGCTTC
TTGCACAGTTACAAATTTGTTGGCTCGAATGGATGGTGAGACATTTGGTGCAA
>Locus_3441_Transcript_295/417_Confidence_0.015_Length_303

CGGCGGAATGGGATCTTCAAGAAGGCCAAGGAGCTGGCCATCCTCTGCGACGCCGAGGTCGGACTGGTCATCTTCTCAAGCACCGGCCGCCTCTACGAGT
ACTCGAGCACCAGCATGAAGTCGGTGATAGATCGATATGGCAAGGCCAAAGAAGAGCATCAGGTCGTCTCAAATCCCAACTCGGAACTTAAGTTTTGGCA
AAGGGAGGCAGCAAGCTTGAGACAACAACTGCACAACTTGCAAGAAAATCATCGGCAGTTGATGGGTGAAGATCTATCTGGGCTGAATGCTAAGGAACTG
CAG
>comp82796_c1_seq3
CGATTCATTGGTCTTTTATATAGGTTGAGGAACCGGGAGGCGTAGGATAGAGAGATCTCAAGGTCAGGTGAGAGCTCAGTGTAGGTCAATGGCGTCCGTG
CCGTCGCTGGTGCTTCAGTTGCCAGTCAATTGATGCACCGTGGTTACTGGTTCAGCTCGCCGAGCAGGTGGATTTCAAGTGCTAGCAGTTTTTACCTGTT
CGGGCAGGAGCAATAGTGGAGATCTTTAGTTCTGGCATGGGTTCCTTTGGGATGAACTGGAATCAAAAGAACTCCATGGTGTGGGATTGGAAAATTTAG
CACCGTCTGTTCCAAATGCAATCGGAAACCCCAGGAACGGAGCTACGGGGCATGGATCTGTGAATTCATCTGGTGGCACTCTCACTTCTAGCTCAGAGCT
AGGGCATGGTTCATCCAAGAGCTCCATGTCGGCATCCATTGATTCACCGTCCAAAGTAGGGAACAGCTTGGAGTTCAATTTTGCTGCTGTCAATACGTAT
GGTAAGAACATGGATAAGGATTGTAGAGTTGACGACTCAGGGACTTGTCCATCGTCCATGATAGCATTCAGCCATGGAGAGCCATTAATCAGTCTTAAGC
TTGGAAAAAGGACTTACTTTGAAAATGTCTGCGGAGGACAGGATTCCAAGAGCACTGCACCTTCTACCAGGACTTCTCCATCAACCGTTCTCAAGAAGAC
CAAGGTGTCTCAGCAGAATACACAAATGTCATACTGTCAGGTTGAAGGTTGCGGAGTTGATCTATCTTCTGCTAAAGATTACCATCGCAAGCACAAAGTC
TGCGAAGCTCATTCTAAAGCTCCGAAGGTGGTTGTTGCTGGTGTACAGCGCCGTTTTTGCCAGCAGTGTAGCCGGTTTCATGGCTTAGCCGAGTTTGACC
AGAACAAACGAAGCTGCCGTAGACGTCTTACTCATCATAACGCACGAAGGAGGAAACCACAGGCGGATACAATTTCATTCAGTTCATCACGTCTCTCAAC
AATGTTTTATGATACAAGCCAGCGGACAAATCTTTTCTGTAGTCAGCCTCTTTTTGGCCAAGTGAGAAGCAATGCAGTTTCTTCATGGGATAACTTGGGA
AGCTTCAATTTTATGGAAACAAAACATCTGTTGATGCAGCCAACGAAAATGTTAGGTCTTGATGGGCTGCATTTCTCAACCCCCCAGATATCAAATAGTG
TTGTGGCTCACTCTGTACATCATCATAATTTTGATGGGTTCACGCCATTCAAGGGAACCAACACAAAGGTCCTCAACCAAGGTGTCGAAGCTTCTGCGAT
CGCTTCCAACTCGAGTGGAGACCCGGATCTTGGGTGTGCTCTCTCTCTTCTGTCAAACGGTTCGTGGGGTTCAACTTCAGCCGTCATCCAACAGCCTAGT
TCTCATGCGCAAGCTGGTGCGGTGCCACCCTTCGCCACCGTCGCCGTCTCCAACCCTGCAATGCATCCTCTGGACTCATCCCCAGGAGGATTCTGGCAAG
ACGACCCTTCCCCGCTCGATGGAACTCCGGAGATTCAGGCATTCGGACACCTCTAACTATGGTCAAACAAATGTACTCATGCTGCATGCCCTCGGAACAG
CTGATTTAGTCACCTCATCATAATCTGCAACTCTGCTTTGCCAGTGGCAGATCTCTGCATAATGTCAGTCCTATCATCGGAGGCATTTGGTGAGGTGAAG
ACTGAAGAATAATATAGGAGGCAACCCCCTGATCTTCCTGATTTAGGATGCATCTAGGATCCAGTTGTGTGTGTGCTTGTGGTTTTGCCAGAACCGGTTT
TTTGTTTGCCGTCCCGTTAGAGAGAAGGGATCAAGTACATTCATGACTGCTCATGAGATTACTTGTTTCACTAATATTACATGATCTGGAGCGAGATTTC
GCTCGGTTCACGTTGATGGT
>comp81084_c0_seq8
GTTGCTTCTGGCTCCAAATGTTCCTCTGTTGGTTTGTCCTTTCCTTTCCCCTTTGCTTTATCTTTATTTTTCGCCTTTTGTTTCTTGATTTTGCTTGGTT
TAGTTTCTTCATCAGACAGCTCCTCGGCGAAGAAATCATCGTCGTCGTCAGGCTCATCTTTGCTGCTATAGTCATCATCATCATCATCCTTCAACCCCCT
CTTCGTAGCCTTCCTTTTCTCTTGGTGCCTCTCCCAGACAGTCTTCTCGTTGGTCTTTCTCTCTAGGATACGTTTACTGAGATCCTCAAGCTCTGTGTTG
AATGTAATCTCCATATCCTGGCCACCAGCTTGTTCCTCTTCAGATTTGTCGCCTTCCAGTAAAAGAGCCAACCTCTCCTCTTTTGTTAGCTTTCGTTTGG
ATCCCCCATTTGGTAGAGACTCATTATCAGGATTGTCTACACCGTCATCATCTGACGCACTGTCATCACTTGCTAAATACATGTCAAGCTCATCTAACTG
ATCATCAGTGAACTTTCGCCTCAAGACCTTCTTACGTTCTGGTTCATCATCATCCCAAGTTAGCTTAACTCTGCTATGTTGCAGAGCACGAGTCTCAAAA
TCAGGCTCCTTGTAACTCGGAGGTGCCTCTGTAGCAATGTCTCGAGCAGGATGTTTGAACTCCATAGAATCAGGTATAAACTGCAAATCAAATACATTTG
CTGTTTTCAAGAACTCAGTACCATCAAGATTCATGTACAGGTGATTTGCTGTTGCACTAGAATCACAGACCACAACAGCATAGTAATACCTCAGTCTGTT
TAGCTCGTAAGTACGCAGCTTGTTATTCTCTTTGTCAGAGTCAACTTCTTCTTCGTCGTCATCAGTGTTGAATTCATCATCATCAGTGTCGTCTTCACCA
TCGTCGTTATCATCATCATCGTTGTCCTCATCATTATCATCAGTATTGGCATTAACAAGAGCTGCTGGGCCCTGAGTTGATTCAATTTCCATGCACTTCA
GTCCAAATTCTGATGGATATATTGAGACTGATAAAACTCGCCCACCTTTTGGGACGCAAGATGTCATCACCATATACAGGTCAACTGCCTTGATATGATC
CCAGTCCATATTAACAACAGCAAGCCTGTGAGTTTCCTTGTCAATCATAGGCGTATCATCGTGCCGGCCCATTAAGTAATGAGCAATGTCACTGCCGACA
GAGTACTCGTCATCATCAACGTCTTCATCCTCGTCGTCGTCGCTACTAGAACTCTCTTCTTCCTCCTGCTCATCCTCTTCATCCACGTCCTCGTCCTCCT
CTTCTTCAACTAACTTCTCTTTCTCCTTCTTTGCCTTCTCTTTAGTCTCATCCTCCTCCTCGTCTTGGTTGAGATAGTAACGGAGCATGGGGTTCTCCCT
GCCGCTCTTCTTCCGTCGCCTGCCGCGCTTGTCCACCGGCGCCGCCGAGGAGGAGAACCTCGGGTCCGTCATCATGCTGCTGAACCTCGAGTCCAGCGCC
ACCTTCGCCTCCTTCCGCCGCATCGACCGGAACCGCGGGTCCATCCGCGCGGCCGCGAACCGCTCGTCCTGCATCGCCCGCTTCATCCTCTCGTCCCGCC
CCTCCACCGCCTCCGCTTCAGCCGCCTTGCCCTCGTGCTTCCCCTCCTTCTTGGGCTTCTTGCCGTGCTGCCGCTTCTCGTCCTCCTCGCGCCCCTCCTT
GTGCTTCCGCTTCTTGCGCTCGGCCTCGGGGCGAGGGGCTTCCTCGGTGGCCGCCTGACGCTCTTGATTCTTCTTCTTGTGCTTCCTCTCTTCCTTATCG
GACCTCGATTTCTTGGCCTTCTTGAGGCGCGCGGGCTCGTCGGAGCCGGTCGGCGG
>Locus_10929_Transcript_2/78_Confidence_0.041_Length_445
GTTTGTACTCATCTGCTTGTCCTGGCTTCTTTCCGAACCTCCAGGAAAGGTCAATGGAGCGGAGGCCTGAATTGGGTCCCCAGCCCTACCCCACCTGGGG
ATGGCAAATGCAGGGCTCCCTTCACATGCCGTTGCACCACTCTGCAGCATCATCAGGATTCTCTACCGCCGTCGGCACGAACGCCGGCAACGGGCCGTTG
CCGTCTCAACAGCCGGCGCCGTTCTCGGACCACCAGTTCTACTTCCCACCAACGGCCTGAGCTTGTTGGTCAGTTCAACTCTTGGGACGGCAGCTGACGT
TGTGTGTGAACGTTTCGCAGCTCTCCGGTAACAGTTGTTAATTATTGGGAGAGAGCCAGAAGACATAGTATGGCTGATCTCTCTGGCCTGTGCCTCGTTA
TGATCTGAGTTATTGCTACTACTACACCGTGGATTCATCATGGTT
>Locus_17579_Transcript_70/92_Confidence_0.115_Length_2150
TGTGGTACTTTCTCTTCCAGCTCGGAGATGGGGTATGGTTCATCCAAGAGTTCCATATCAGCGTCGATTGATTCTTCACCCAAGGTGGGAAACAACGTGG
AGCTCAATCTTGCAGCTGTCAAAGTGCCTGACAAAAACACCGTCAAGAACACTGATTTGGGTAAAGTTGATGACGCTGGAACTTCTCCATCATCGATGAT
AGCCGTCAGCAGTGGAGAACCGGTGATTGGCCTGAAACTTGGCCAAAGAACCTATTTTGAAGATGCATGCGGAGGGCAAAGTGTCAAGAGTTCACCATCC
AATGTGAGTGCAGTGACCCCTGCTTCTGCGAAGAAAGCAAAGATGATTCAAAACGCACAGAACTCGTACTGTCAGGTTGAAGGTTGCAAGTTTGATCTCT
CTTCTGCTAAAGATTATCATCGGAAGCACCGAGTCTGTGAAACTCATTCTAAGGCTCCCAAGGTTATTGTTGCTGGTCTGGAGCGACGCTTTTGTCAGCA
GTGTAGCCGGTTCCATGCTTTAACTGAGTTCGACCAGAAAAAACGAAGCTGCCGGAGACGCCTCAATGATCACAATGCCCGCAGACGGAAGCCACAGCCT
GAAGCAATTCCTTTCGGTTCATCAAGGCTCTCTGCAATGTTTTATGGTATCGCTAAGCCTTTGGTTGCCTAATGGTTTTGGGAAACTGGTGCATTAATTA
TTGTTCTTTTAGCAACTGTTCTGTTTAAAAATCTTGCATTTTTCATGTATGCCCAGAACTAAACGGTGGTAAAATTTATGAGTACAAAGTGCAATGTTGT
GTGTCTGAAGTTGTCCCTTATTTGTTTATTCATAACTCAGTGCCATGATTTTTGAGATGTCATGAATTTACATACTTCTGATATTAGATGACATGATAAC
TTTGGCAATACCGGTAATTAATGAAACTCCATCTGAGTACTGCCATTTGTGATGATTACTATAGATGGGACAAAAGAGATATCAATTAAGTTGTATTTAT
TTTATGAATAGGTGCATAATGCTATATTCTTTTCGATTTAGATATCCATGACATGATTCTACTTTATCTGCCATGATGCCATCTCTCCATTATAGGAAGT
CGGGAGACAATAGGAAGCTGTTAAGTATCTGAATAAACATCAGTTCAAATATCTACAGATGCATGTTGAATCCACTATATTATCAATAATTTGTACGAAA
GTAGCTTTAGCTTTTTGTATTAAGCAAATCGTTTGTCCTCTTTCTGAATCTGTTTGGCTTTAGTGATGAAAAATTGTCGTGGCGGTCTTAAACAGATGCA
AGGCAACAGACAAGTCTTCTGTTTGGTCAAGCTCCTTATGGTCAAATGAGAAGCTGTGCAAACTCTTCATGGGATAGCCCAGTAGGAGGCTTCAAATTTG
GAGAAACAAAAGCTCCTTGGTTAAAGCCAACGAGAGCTGCAGGTGTTGATGGGATGCATGTATCAAGCCAGCAGGTGTGGAACAATATTACGCCACACGG
TGCACATCATGATTTTAATGGTATCATGGCTTTCAAGGGAACCAGTGCAAATGTCCTTAATCAAGGTGCCGAAGCTTCTCCGGTCGTCTCCAACTCGAAT
GGAGCCCCAGATCTTCAGCGTGCTCTCTCTCTTCTGTCAAACAATTCAGCTGGTGCTGCCAACGACCAGCCAACTCCTCAGCTGCACCCTGGGCTGACCA
CCTTCGTCGGCTCCTCCAACCCTGTCACTGTGATGGAAGCCTCACCACCAGGACTCTGGCAAGATGGCGCCCCCCTCGATCATCAGGCGCGGTTTCAGCT
TTTCGATCCGGTGGGTGACGGCAGCGCCATTGTCACACCGCATCAGCTCCAGCTCCCAAAACCTCACTCCCTCAACAGCTCCTCTTCCCACTATGACCAG
ATGCACTGATGATCTGATGCTTCCTTCAGTTAGCGCAAATGGCGGTTGGGTTGCGCAAAATTAGACATGGCTTCTGATGCCTGAAACTCTGAATTTTGAA
ACTGTTATGTTCTTCTGAAAAATAAAAGCCTGTGTGCGCAAACTCAGGCTCCAAAAACTGATCTAGTAGTGTTCCCATTCAAGGGCTTGAATAATCCATC
CTTGTGGTGGCATGTAACTCAAATGCTTCGATTGCGCCGGCAATTGATCT
>comp67036_c0_seq6
GCTTCCTCTGACACTGCAAGCGCTTCGAGGGCCAACCAGGACCACATCTGCTTCCCCTCCGCTGGCTTCCACCCCGCAATGAACATCAGCGATCATGGTG
GAGGAGACACAAAGCTGTCTGCGAACGGCATGCAGCAACGTGTTTCCTTCATCAGGTGAAGAAAAGTGGCACTGCCTGTGGCAACTCATCCTTGGCTTAT
GAAACTATGGATATGTAATGGGCATGTAGGTTCGGTCTCAGAATAACCAAACATATGTGAGACCTTTTTAAGTTTCCTTTAGT

>Locus_4425_Transcript_39/75_Confidence_0.071_Length_849
CCCGCCGGAGACTACAACCTCAACTTCTCCATGTCCTCGGGTCTCGTGGGTGTCAACAGTGGGGGGGCCCTTCAGTCCAATTCGCAGCCGCACATCTCCG
GCCACCACCACCACCACCACCAGCTCCAGAGGCTGTCTCCTCCACTGGACGCTCCAAACATACCCTTCCTGTTCAGCCCGGCGGCAGCCGCGGTGGCCAT
GCCCACCCAGGCGGAGAGCCAGTTTGCCGCGTTCCAGCTCTGGAACGGGTTCCAGCACGCGGACATGAAGGAGAAGGGCAAGAACTAATCAACAACTGCG
CAATGCAGTGCTTTTCTGGATGGATGTGAAGACATCAAATAATCGCAACCTGTTTCTGCGAGCACTATCCATTGCTTGAAAAACGCTAAAAGTGCATTGG
GGATGATTCGCTGCATTCTTGGAAGTGGATCATTATTCGGACATCTACTCTGGTCATTGAAGAACCTCATCACAACCTTCTGCTCTTTCTTTTTTCTGCC
TGCTGTTGTTGTATTCATTTTGTGGAGGTGAGATGTAGCAAGATTTGGTTGAGTGCCCTTTCAGAAGCCATTGTAATCCTTTCCATTCTAAACTTTGTCA
ATGGATGGATGCGGCCCTTGATCGGGTTTTGAATCCAAGGGATCAGTAATCCCTGCCATTTTTATGGTTCTCGGTTGTGTGTTGTGAGAGAAGAGAACTC
ATGATTCAATCCATCAGATGTAGTCATCTGTGGTCAAGTCTGTTCTCCTGTTGTGTGCTCTGAGATGTAAAATTAATTTTTCTGCAGTGATTATTTTGTA
CTGCTCCCTGATTTGTGAAAAATCTAGTAATGTGTGGTCGACAGATGCT
>comp82180_c0_seq42
AATATAGGGGGCCCCGCTCCCGCAGCTCGCAGTACCGCGGCGTCACCTTCTACCGCCGCACCGGCCGCTGGGAGTCCCATATTTGGGATTGCGGCAAGCA
AGTGTACTTAGGTGGATTTGACACTGCTCATACTGCTGCAAGGGCGTACGATCGAGCTGCCATCAAGTTCCGTGGCTTCGACGCCGACATAAACTTCAAT
CTCAGTGACTATGAGGATGACATGAAGCAGATGAAGAGCCTGTCCAAGGAGGAGTTCGTGCACGTCCTGCGACGGCAGAGCACCGGGTTCTCGCGTGGCA
GCTCCAAGTACAGAGGCGTCACCCTGCACAAGTGCGGCCGGTGGGAGGCTCGCATGGGACAGTTCCTCGGCAAGAAGTACATATATCTTGGGCTATTCGA
CAGCGAAGTAGAGGCTGCAAGGGCTTATGATAAGGCCGCGATCAAATGCAATGGTCGAGAAGCTGTGACGAACTTCGAGCCAAGCACGTATGATGGGGAG
CTGCTGACTGAAGTTGCTGATGAAGGTGCTGATGTTGATCTGAACTTGAGCATATCTCAACCAGCTTCTCAGAGCCCGAAAAGGGACAAGAACTCCCTTG
GTCTGCAACTCCACCATGGATCGTTTGAAGGCTACGAATTGAAAAGAACAAAGATTGATGCTCCCTCTGAACTGGCGGGCCGCCCTCATCGATTCCCTCT
TCTGACCGAGCATCCACCAATCTGGACTGCCCAGTCTCACCCCTTCTATTCAAATGATGAGGTTAAGTGATACTAAATTACTAAAATACATGCAATAACT
TTCTCAAATATATTGCTTTAGCTCCCTAAACCTCACAATTGCAAGCATGTTCTGAGCATCACATGGTTCGGATCGAGCTGTCCGGCACCAATTTTGAACG
TAATCTTGACCATCTGAGGAACTGATTGTGCAGCCTGAAGGCGTGCGTTCAAATGTTAGATTTGTGTTACTTTCGTTCATTTTCACTTCTTGTGCCGCTG
TCAATTTTGGCTCTAGTCTTACATCACCGCTTCATTTCCTTGGATTCTATAGCCCGCCTGTTTCCTGTCGATTTCGGAGATATTAATGCGTAGCACAGGT
TTCATATGTAGGATCTTGAGTTTTCTGAGATGGCCCATTCTGGAAACAGTAATAGTCGCCTTAGGAATAGAGATGTGTTTGATTTTTTTCCTTCAAAAGG
AACATCCTAGAGAGGTGACTATTCGACATTAGGTATGCTAATCCATCGGTCAACTATACCTGCCTGCGGCTAAGCACCTTTCAATGTTTCTTTTTTCATT
CATCGTATCATATAAAAGTAGTAAGTATGACTATTATTCAAACCTTTCTTGAAAAAAATAGTTAGGATGTTGCCATCTTGTAATGCAATCATAGAATTGG
AAGTGCTGAATTTGCCATTTGTTTCAAACAAGCATTGTATATTGGTTTATCATTCAACTTGAGAGTTTGGTCAAATTGAACTCTCGAACTTTTCAACTCC
TAAACTTTAAGGTCAGGCTCACATATGTCATTTTCAAATTTTATTGGACGGAAAGTTGAGCTGGACGTTTCGAGCTCGGCTTTCTATCCAACTGAACTAA
TGGAAACACTGATTTCATCGCAACAAATTTTCAGCAATAGATATTCAATCTCTTCTTGAATTCATCCTTCATCAGTATGGTGAATAAATTGTTCTTGGAT
TATGCAGAATGCATCAAGAGATCTTAACAGGAGGCCAGATCAGGGGGGTGTTCCCAGCTGGGCGTGGAAGGTGACCGCCCCTCCTCCCACGCAGCCATTG
CCGCTGTTCTCGTCGGCGTCATCATCCGCTGCAGCATCATCAGGATTCTCCAATACCGCCACGACAGCTGCCCACACCGCCCCATCGGCCTCCCTCCGGT
TCGACCCGCCGCCGCCGCCGTCCAGCCGCCACCGCTGAACGTTAAGAAGCCACGCTGTAAATTTGCCAGGAAGCCGACATTTTTTCCTCTCTCGGCGTTG
CAACTTTTTAGGTTTTGCGCCGGGGTGGTTTCTTGTAGTGGAGTGGATTCATAATTAACTGTATTTGCATGCTGCCCATGTGAAATGCTTCTCTCTCTTT
TTACGCTCTCTGCACCGGATGTTCTGGGAGTTCTTGTTGCCGGGAATTAACTAAATTCACCGTCTGAGATTGATCTATACATTGTTGTAGAGAATCGAAT
CATTGTTGAAATTAGTTACAGAATTATAGATATCATAAGGCACCCTCTACTTGTGGACAAAGTTCAGGAAGATTATTCCGGTTCTTTTCTTGGCTGTCAA
TTCGGCTCTTGTCCTTGGTGTGGAGCCACTTGCCTACTGTCTTGTTCTGTTGCTGTTTGTTCTTGGCTTCTTGCACAGTTACAAATTTGTTGGCTCGAAT
GGATGGTGAGACATTTGGTGCAA
>Locus_7001_Transcript_54/84_Confidence_0.209_Length_2544
TCACTTTAGCCACCTCAGATTTGCTGTTTGTAGCTGGTGAATCTGTCCTGTTCTAGTGGTTTTTTTGGTCCCATGCTTGCTGGATAAACGGATTACGGTG
TAGGTGTTCCGTGTTCTTGTTCAAGTGCTTTGGACTTTTGTGTGGTTGCGGTTCTTGCTTCCTCTTTGTTTCTCAGCAGGATCTTCAGAAAATTCCTTCT
GCGAGTTATTGGTTGGAGAACACATTCTTGTCTGTAGGTTCATCCTTGCTTGGCTAACTAGAATACATTAGTCCTTTCATAATTAAAAAAAATACATTAG
TCCTTCAGGCAGTTCAAGCTTGCCTGGAAATCTTGCCTTCTGCCTTCACTTTTGCTGATTTGGTGTTCTATTGTGCTAATTTTTGCGATAATTTTTAAGC
TAAATTTGGGTGGTCAGTACTGCCCTTGAGTGATGCTTCAGATTAGTTACGTTATGTGGCTATCGGAGGAATGGAGAAGGGGTTAAGAAGGAAAGCGCTG
AAAGTACGGAGGACTTCGTCGATATTAGCAGGCATAGGTTTATGTGATTTGTGACAGATATGTTCTGCTTTGAGTAATATCCCAGGTTATGGGGAACACG
GATAATAAAAGTAAGTATTTAACGAAAAAGCTGGATTTCTCTGTTTCGTGCTTGCGACGATTGAAAGGTTTAGAAGGATAATGTAGGATCTTCCGTAGGG
AAGATAGTATAGACTAGGGATCCCTTTGAGTCAATAATTCTTGAGAATCCTGTGGTAATCTGAAAGTAGAGCTGAAGGAAGTATAGTGCGTTGTCAGTTG
CAGGACAGATAAATTATTGCTACTTGGAGTAGCTTAGAATTATCACTATCAGAACTTCTGGATGAAGGTGATATGTTGTGCTTCCTCGTCTTCTATTTGG
ATTAGCTAGCTTGCAGCACTCAGAAGCTCTGCTTATCCTGGAGCAGGTATAGAGCTTAGGGTTAAGTTATCATAAGGATGCCAGAATATTATTTGCTAAT
GAGAATATTGATGCCTCACCATGATCTTTCGATTTATTTTATGATTCTGTCATTTTCCAACTGTTCATCTAGCTGTGTTAGGGTACAACTGTTCATCTAG
GGTTCAACCTATTTTATATTATATGAGATAATGAATAACCAAATTCAAGTTTCCACATATTTACCTGGTACTTTGGCATTATTGGTGCATACTGTCAGTT
CAGCTGATCAATTCTTGCATCAGCAATTTGTTCTGAATACCTTTTCTAATGTTGAAAACAGGAAGAAGCAAAATGTTTCTGGAGCTTGAGTGATGCATCT
GATAATGAAAATGCGTCTGCGAGAAATGGATGGTTATCCAGTCCATTCCATGCCCAACTATGATTTCCTGTCTGGGAATGGCTATTCAATGAAGCAGTTT
GTCCTTGGCAATCCTGATGGAGACTCCTCATCAACAAAGTCTGAGCAGTGTTGCCAAGATTTATCTGCAGTGAGTGACAGCAGCCTCAATGGACAACACA
CACCAACACAATCTGACAACAATGATAGTTGTGGGAAGGTAGACCAGGGCATGGTAAAGTCTGTACTGTCCTTGGGAAACCCAGAAGCTACATTTCCCCC
TCCAAAGTTTGATTACAGCCAGCCTTTGGCTTGTGTTTCTTATCCGTATGCTGCTGATCCATATTATGGTGGGGTCTTAACAGGATATACATCAAATGCT
GTTGTTCATCCCCAAATTAATGGTACAGCAAATTGTAGGGTCCCATTGCCTATTGAACCTGCAGCCGAAGAGCCCATATTTGTCAACGCAAAGCAATACC
ATGCAATCCTCAGGAGAAGGCAAATGCGTGCAAAATTTGAGGCCCAAAATAAACTGGTGAAAGGCCGGAAGCCGTACCTTCACGAATCTCGACACCGTCA
TGCAATGAAGCGAGCCCGTGGGTCGGGAGGCCGGTTCCTTACCAAAAAGGAGCTGCAGGAGCAGCAGCAGCAGAAGACACTGCCTTCACTTCAGACTCCA
ACATGTGTGGCAAGTAAAATGTCACTCGGCAGGAATCAATGCCCTGAAAACAGCACATCTCGCTCGCCTTCTACACCGACAGACTCTGGGATCTCAAGTG
TTTCCAACGGAGGTGGCATGCTGGCTCACCAAAAGCACATCACCTTCACATCTACTAATTTCCTTCCCAGCATGAACTTCAGCACCCAGAATGGAGGTGA
AAAGATGGCTGTCAATGGTGCGCACCACCGTGCCTCCATCGTGAGGTGAACAGCCTCGATTGTGGGGCGGTGGTATGTACCATCACTGGCGCGCTCCGGT
GAGCCCGTGGCAATTCATCCTTGGCTTATGAAGTATCTGTATCTGGTAATAATTTGCTTTCAGTTTAATATGTAGAGCTTGTCCGAGATGAACTCATTTA
TATTTGTTGCAACTAAAACGAGGAGCTACTTGTTGATATATGTGGATATTGTGGATACAGAAATATCCTGATGTTTCTTGATGTACCAAGTCCACTATTA
AAGTATGACTGGAAAAAACGTTTAATAACGTAATCATGGTTTGG
>Locus_11912_Transcript_27/63_Confidence_0.339_Length_3472
TGGCGCCTCCCGCCGGGCGCTCGCCCCGGATTTCTCGGCTCCCCATCCGGCGGACGCCTTGACCGGGGTGGGTTCTTGGTTCCGGCGGCTCGGTGATCTG
TGCGTGCGAAAGGCGGGGCTTTTAGCTCGTTCTGTGGGGGGAGGAGGAAGACGGCGGCGAGGCGTTCATGGGTTAGATCTGGCGGTCCGGCGCCGCGAAT
CTGAGCGAGGTTTCGGTGGAGGGATCGGGGCGGAGAAGTCGCAGTCTCTGGCTCGTTCTGAGCAGCTTGGGCCTCTCATGTCCAAATCGGGTATGGATGA
TGGGTGCGGTAACTGTCGGAAGGAGCGTCGCTTGCAGATCTGAAAGCCATCCAGCTCCCTTGTTCGGTTGACCTGGAATGGCCGTGCTTTCTGCTGTTTG
ACTGTCAGTAGTTGCTGCTTAGTGTGTGTGCAAGAAATTTAGCTGGAGGTGATGTGCTTAGGAAACTAGTCTAGTTGCTGATATCTTGGTCTCTGTTGAA
GAACGCTGAAGCTGGAAGGAAGTAGGTCGCATTCCTGTGTTGTTGATATTTTGTTGTTTCTCGAATAACCTGGAGGCTGGAAGGGATCAGATCCAGTTCC
TGTGGCACCGGTAACAAGGTGCTGTTGGGGGAAGGCACCATGACCTACTTCCCTGAGGAGGTGGTGGAGCATATATTCAGCTTCTTGCCGTCGCACAGTG
ACCGGAACACGGTCTCGTTAGTGTGCAAGGTGTGGTATGAGGTTGAGAGGCTGAGCCGGTGTGCTGTCTTTGTGGGGAACTGCTACGCGGTGCGCCCAGA
ACGGGTGGTGCTGCGGTTCCCCAATGTGAAGGCCCTGACGGTGAAGGGGAAGCCGCACTTTGCGGACTTCAACCTGGTGCCGCCGGACTGGGGTGGCTAT
GCGGGGCCATGGATTGAGGCTGCGGCGAGGCGATGCGTGGGTCTTGAGGAGCTCCGGATGAAGCGGATGGTGGTGTCAGATGAGAGCCTTGAATTGCTAG
CTCGGTCCTTCCCGAGATTCAAGGTCCTCGTCCTCATCAGTTGCGAGGGGTTTAGCACCGATGGGCTAGCAGCTATTGCGAGTCACTGCAAGCTCCTGAG
GGAGTTAGATTTGCAGGAAAATGATGTGGAGGACCGTGGGCCTAGGTGGCTCTCCTGCTTCCCTGATTCCTGCACCTCTCTTGTCTCCTTGAATTTTGCC
TGCATCAAAGGGGAGGTGAATTCTGGTTCATTGGAGAGGCTTGTTGCTAGGTCTCCAAACTTACGCAGCTTGAGGTTAAATCGATCTGTATCAGTAGATA
CACTCTCAAAGATATTAGCATGCACCCCTAATTTGGAAGACTTGGGGACAGGGAATTTGACGGATGAGTTCCAAACTGAATCCTACTTCAGGCTGACTAA

TGCTTTGGAGAAATGCAAAATGTTGAAGAGTTTATCAGGATTTTGGGATGCTTCTCCTGTTTGCGTTCCATTTATCTATCCCCTCTGTCATCAACTAACT
GGACTAAACTTGAGCTATACTCCCACTCTGGATGCTTCTGATCTCACTAAAATGATCAGCCGCTGTGTGAAACTCCAACGTCTTTGGGTACTGGACTGCA
TCTCAGATAAGGGTTTGCAAGTGGTGGCCTCCAGTTGCAAAGATCTACAAGAACTCAGGGTGTTCCCATCAGATTTTTATGTTGCTGGTTATTCTGCCGT
GACAGAAGAGGGGCTTGTTGCAATATCGTCAGGCTGTCCAAAATTGAGCTCTTTGCTGTATTTCTGTCACCAGATGACCAACGATGCACTTATTACTGTA
GCTAAGAACTGCCCAAATTTCGTAAGATTTAGACTCTGTATTCTTGAGCCAAAGAAGCCAGACGCCATGACAAACCAGCCGTTAGATGAAGGCTTTGGTG
CAATTGTTCGTGAATGCAAGGGGCTAAGGCGACTGTCAATGTCGGGTCTTCTCACTGACAAGGTTTTCATGTATATTGGAAAGTATGCCAAGCAACTTGA
GATGCTTTCTATAGCATTTGCTGGAGATAGTGATAAGGGGATGATGCATGTTATGAATGGATGCAAGAATCTAAGGAAGCTGGAGATTAGAGACAGTCCA
TTTGGTGATGTTGCGCTCTTGGGGAATGTTGCCAAGTATGAAACAATGCGATCCCTTTGGATGTCATCATGCAATGTCACATTAAAGGGGTGCCAAGTCC
TTGCGTCTAAGATGCCGATGCTCAATGTGGAGATCATGAATGAGCTAGATGGAAGCAGTGAAATGGAGGAAAACCATGGAGATCAATCTAAGGTGGAGAA
GCTATATGTTTACCGCACAACTGCTGGAGCGAGGGATGATGCACCAAATTTTGTTAAAATCCTATAGTTAGCTGAGAGTAGCCTGGTATCTCCTCACTCT
AAAAGGTGATCTTTCTTCCAGATCTATCTTCTTTCATTTGGAAATGTCTTGTCTGTTGGACAATTTGTCCGCTACTCTGCTAGCCCAGTGCTAGTTTGTG
TCTACCGTTCAAATTTAGATATTTCATGCTTAGTAGCTCTATTGAATACTGCATCTTATACCGAATGCTCCGCAACATGCAAACTAGCGTCTTTAGGTTC
ATATAGCTCATTATGTTGGCTTAAATGTTTCATTCAGTAGCGTCTGAATTAACTCGTTTCCTATTTGTACGTCATTATATCAGAACTGTATTATTAAGGT
TTGAAATGCTGCAGGAAGAAGCTTGTGCTTTCATCATTAACTTAAACCAAGACTGATTTCTGAAAAGATCCGAATGGGTAGTAATTGAACTGATGTTTGA
ACCGCCTTTATTCTGCAAAATCTTAGCTGCTCTTTTTGTTTCTTCCTTAGTACATCGATCGCTTAGCTACTCATAGAGCTAATTTCCGTCAACCTGTGTA
TCATTCATATTCCATTGCGAGTATGGAGCAGCAGAGCATCTGATATGTCTAACTCTGTCTTCCCACTCCCTACTCGATTGCTTTTACAGATGGTCGGTGG
AATCCGGCAGGCTGGGGGGCCGCGGCAACAGTGGTAAGTTCTTGAGGTGCCTCTCTCTCCCTTTGCAGGAGTCAGGAAAAGCCACACCGGTGTGCGCCCT
TATCTTGGAGCAACTGGCCGGGATCCGTCATCATCCTCTGTACCACCGCCCCTACCCTTTTAGTGTTTGGTCACTCATATGGCCACTGAGGCCCTTTATC
ATCCTGTGCATTCTCACTCGAGAGGACAACGTTCTTTTTCTCCCTTTTATCCCAATTGTAATCGTAGTGTACCAGTACCCATTTGCTTTTGACATTTGCA
CAGTAGTGGATTGTGTTACCGCCGCCGACGCTGCTGCTGTAATAGTGTTTGTTTCGGCCTGAATGATCGGGACATGACATTCCGGGGACGACCATTGTTT
TCTTGCTCCTGTGAATGAAGTGCGTGGAATAATTTGAGGTTCACCTGTGACATTCTATTACTGAGTTTTGAA
>Locus_12915_Transcript_23/30_Confidence_0.706_Length_4149
GCTCCTTGAGAAACACCATCCAATTACAAGACTATTTGATGCAACAATCACGCATATGGCTGCGCTAGAACTACTGCTCTGAATTAAGAGGCGTTGAAAT
ATTGTCCGGTCTACATATAGGCACTTGAGGTGTATTGACAGACAGTTCCCCTTAGTTTGCTACAACACATCAGGCGAATTCCACCAAATTAGGATGATGT
TTGTTTCTTAGACACAGGATAGCAATGCCAAAAGTTTTTATAATTTTCCGCTTCACCGCAGATTTGGCATCTCAAAGGGAGTTGTGGCAAGATTCTCTT
GACCAAGCTTTCTTGCAGATTTGGCAAGAAAGATAAAAAAAATGTTGGTATACTTAAGCATCCTTAATAGAAACTAAACAGGTCTTAGATTGAAATTATC
ATAAATAAACCCTTTTTCACTTTAGCTGTAGTCCGGATGAAATCTTAGGGGCCTTGCTTAGCCTGCCCTGAAGCTTGCCAGTAAGTCGAAGCTGATGCTG
ACACAGCACTGACATAGATCCCAATGAACACAAGTTATTTCGAGAAGATAAAATACCAAGCAAAGCCAGGATTGCATTAAAAGATAGTGTATTTCTACTA
TACGATCTGAAAGCTTAATTTATTATATCAATAATCAAATTGGCAGCAGTGTGGAACAGATATAAAGAAGATGCTTCAAATCACAAATAACTACAGATAA
ATTGAAGGGTGAAGGTTCATTCAACCACCAAACACCCAAAATGTCGAGCTGCATATGCACAGAACCTAAGTACGGTACAGTTCATAGTTTGGGAAAATAG
ACTGCAAATGTGCACAGATAATACAGATTGTTACTCGATGAACAGTAAGCACGGTATTTGAAATCCTTCTAACCATCCATCCAGGCAGTGCCTCGTATAC
TCAAACCTCTTTTTGGAGGAAGCCAAACCATGTGATCCTGTTTCAAGTTCTCATCTTCTGAAAATGATCAGCAGTCAATAGATGTTTTAGTCCATTCTCC
AGAAAAATGAGTGCCGCATGAATCCTTTCCGGTTTATAGTGAAACATTTTAGACGATGACCTTTAACATTCTAGAAAACGAAGAGAAAACCTACAGTCAT
CTAATTTCAGCTTAAACATGTTCCAGCAAGATCATCACCATCCTGACATGTTCCGTTTTGTCCCTAGACCTGAGCACAAGGTATTGCGAGGCTAATTTAG
CGGGGCAATTGATTTTTTGCCACTATGATACAACTGCAATTACCCGCTGTCACTGACACGTGGGGTCATCTGAGTCACTGAAATGTGGGCCCAGTGGCAT
TTTGTTTGCCACTCACCCTTCCAGAGTGGCAAAAAGTTAATTGTCCCTAATTTAGCTCAATTCAGTTGTCGGAGCAATTGTGTGTGAAAAGTGTGAACAA
CAGCATAGGAGACAATACAGCAAGTGTAGCGTGCCAATATGTGTCCAGCTTGATAGTTCACTATAATAAAGTTCTTTAATTCAAGTATTTGAATAATATA
GAATCTCAACCTTCAAGTTTAAAAGGTTTTGTATTTGCTATTATAATCGTTGCCACCCCCTCACCCCTCTCAAAAAGAGAGTTACTTTGTAGCGCACTGT
AAATCTCTTGATTGAAATAGTGGCATGTCATCTTATTTAATTCAGTTATTTCATAAGGTACAATTTGTTTAGCTGACAGCTCAGACATGCTTCTATGCAA
CATGTTTATACCAATGCTGTATTTTGGCAATCCTGGATGTGGCATTACTTAACTGTGCTTTCATATTCAATAGTTCTGGCAAAGGGAGGCTGCAAGCTTG
AGACAACAACTGCACAACCTGCAAGAAAGTCATCGGTATGGCTAGAAAACCCTGCAAATTTTACAAATGTACAAGTCAGAAAATTTGCCTAATGTAAAAG
GATCAAACTACAGGATTTGTCAGCATGAGCACAAGTGCCTAACTTCCGAGTTAGATAACTCTGGAGGAAGGAAATCCTTCCTACTGTTTGTTTCAAGAAT
ATACAAAAATTCAAAACAACATTAGAATAGAACCAAAAGAAGAAACAATATTCACCATGTAGCAATACTCAATTCCTGGTTGTCTTATTTATAAAGGTAT
AAAACTAGGTTCTGACGAGACAATTATGAATCAACTACACCATGTTACAGATCATCCATAACTCTTGGTGATGCAAATTAAGGGCTGTAAAGTGACAAGT
ATACCTCCACCAACTAATTTGTGCAGAGTGTATAAAATTGTAAAACAATGGATAAGAATGAATTTAGTATACAAGATAGCATACAATGCCCAAGTTTAAG
GAGACATACTGATTTACTGACTCCATAGGACAATCTTTTGCTTATGAGGACATCAGTTTTCATAAACAATATCAATGTAAAAGGAGGTAACAGTTTATGA
ATTTCCTATGCCAAGCCATTTATAAGTCAAAAAATGTACCTATTTAGCTTTTATGCATTCCAATTAACTTCCAAAGAAGAAGAGTACATACTAATGGAAA
ACAAATATGATATTATCATCTGTTTTGCCCGGTAGTTTCTTGAGTCTCTGATTTGTCCGCACTTCACTCCCAACCAACACAACATTAATACACATGGGCA
GAGGAATATCAGGTTATCAGGTAAACTGAATACAGTTTGATTGACTGCAGTCAGTTGATGGGACAAGATCTTTCTGGATTGAGCGTCAAGGAACTCCAAA
ATCTAGAAAATCAGCTAGAAATGAGCCTACGTTGCATCCGGACAAAAAAGGTAAGGATGCTAGCCATATTTCTGTTTTTCGTTACCAGATTGCCTCATCC
AAGAATACAACTGATGATGACGTTTTTTACTTTCAGGACCAACTCTTGATTGATGAAATTCACGAACTGAATCGAAAGGGAAGTCTCATGCAACAAGACA
AGATGGAACTATACAAAAAGGTCAACCTAATCCGTCAGGAAAACATTGATTTATACAAGAAGGTATTTGATAAATCGTGAATGAAAATTCACAGGATATA
CACCCCATCCATTATTGACTGAGGTTTATTTCTCTACTCAGCTCTACGAGAAAGAGGTGACAAGTGAAGTCAACCGAGATTCATCAACTCTAAACTTTGC
AGTTGTCGAGAACGTCAACATTCCTGTTCATCTTGAACTTAACACTCCACCGCAAGAAAATGATGTTGAGCAAACTGCACCTCCTAAACTGGGATTACAA
CTAAATCCATGAAGGCATGCAGCATGGCACCGCTTATGTTCATTATTCTCAACGGTACGATTCAAAGCAACCAGAAACACTATTGGTTGTAAAGAGCTTA
AACACATAGCAAGACTGACGCGGTATGCATGCCATGAGTGATTGATAATGGTAGTGTAAATCTATTTCAAATAAAATGTATTTTATTGAAGAATACAGCA
AATGATTTGTATTATAACTATTTGACACATTCTGCAAGTTTGTTGACCAACGTGAGTACATGATAGCTACAAATCTTATATTGACAAAAAGATATGGTGT
GTGGAAAAAATTCAGGTGCTATTTTTTTCTATTTTGAGAATTGTATGCATAGCAGAAGAGCACAAATTGCATTCTGGTTTATCCGTGCAGGCATTCCAGA
GTATCAGAATGGGGATCAACCTTTCTTCAGCTACATATGTAAATATACGGATGCTAGTTGCTCACTTAAAATTTTGTAGTGTTCAAAGGAATAACAACAG
TAAGACGTTGACGCACCGAAAATAACCAGAAATAACGGATTGTACTATGACAAGACACACGACAGATACACTAGCTTAAGTTTGTACAGGAAAATATTTG
GCCTATTCTTATGTCACAGTAAATTAAGATTATCACCTTAATCACAGAAATTCAAGGGGTAAACGATTATAGCACAAAACGATCCTGGACCCCAAGACCT
GGGTCTCTGAACGGCTGAACCTCCAATTATACCTCTATACATTTGGAATGTGTGTAGTCAAACTTTCGCTACTGGTATCTTTACAAATATTTACATTGAA
CACATCAAAATGATATACATGGATTCCTCTCGATATGTCTTTGAGGTTTGGGTTCTAAAAATGATATACAAATTATTAGGTTCTAAACTTAAATGATATA
TACACATCAAAATGATATACAAATTATTGGGTTCTAAACTTAAATGATG
>comp78295_c1_seq31
ACAACTGCACAACTTGCAAGAAAGCCACAAGCAACTAATGGGTGAGAAGCTTTCTGGCCTAGGTGTGAGAGACCTCCAGAGTTTAGAGAATCGTCTTGAA
ATGAGCCTACGTAGTATCAGAACGAGGAAGGACAATCTTTTGAAAAGTGAAATTGAAGAGTTGCACAGGAAGGGTAGCCTAATTCACCAGGAAAACTTGG
AACTCTGTAGAAGAGTAAATACCATGTCACAACAAAATATGGATCTACAAAGAAAGGCGAGTGAACCAAGAGGTGTTGCCGATGCAAATAAAAGCTCTAG
CACTCCCTACAGATCTTGTAGTGCACAAGATGCAGATATCACGGTTAATCTTGAATTGAGCCAATCACAGCAAAAAGAGGGGGAGCAGTGCCAAAGAGGG
GCTCCAGAACTGGGGTAAATATTCTTTTTCCAGAATCCAAAAACTGCATTACTCCAAGAAGTATCAAGAAGCCTAATGAAGAACCCATGCTTTGACATGT
AACAGGCTTCAGCTGCACAAAGAAGATGCACTACAAGCTGGAGAGTATCATGATGTTCCACCAATTCTAACATGCAATGCGGAGCACTTAAACTACAATT
AGTATTTTGTGCAACATGTTAGATGAGTGAACAAAATGGTTTATGTACAGACTACAGATATGACCATCTATCTTATCTGTGGCTCTACTGTACTGAAATT
GCAAGAAATGGTTATTGTAATGACTAATATGATACATTGACTGTCATACCGTTGACCTTGAAGATAGTTCTATAACGGAAAGGTCCAATTTGAAACAAGT
GTTACGGATGAGCTAGTAGGATTTTTTTCACAAACAATTAGATAGTCCACGTTAGTGGTAGCTGCTGGGTATGACATTTGACCCAGCCTGCCGACCCTCC
CTTTCCCATGGGAGTTCAATTAACATTAGGAATGAATATCGATATTATGGTTGATTATTTGAATCGTATGTTTTCCACCCTTAGATGTAAGGCCCTATTT
TACTGTTTTAATGGATTAACAAGGTGAGCTTTCGAGATGGTAGCTCATTGATTCGCATGCATTTTAGTGGCTGTTAAGAGGTACTTAGTGCATCCATGCT
TGAAGGTGTTGTTAGTTCGACAAACCATTTTCTATCCTACTGGATTACTATCTTTTGGTCAGTATGACAAGCAAGAATTTAATCTGCCAATTAAGAATTT
AATGTGTTCTTTTGACATGAATGTCAACAGCATTTAAGTAACTCAACTTTTAATACAAAGTACTGGAATGTTTTCTCACCGAAAGTAAAGATGTGGTTCC

TTGATAGCAAACAATGTTTATGCCAATATCGCATTAAGGTGGTTGTATCCTTTAGAATGATCTCCTCATTCGTGCTTAATTTGATGCTATTGTCTTTTTA
CGGATGCCTAGATGTGGTTTTGACCATTACTAAGTACTTTAATATATATTTAACAAAAATAATGACATAGCTGTTGTATGGCCAATCTAGATTTTGTTTT
CGAAACATATTTGCAGTCAAACTATAGGAAGTAAGAAACTTCAATGTGGTTCTCGAAAACCATGCTTAAAGTTTAGAAAGTTTGACTACATAGAGTATAT
ACTACATAGTCTTAAAGCATCAAATGAAGAGGAATGGAGGGAGTAGCAGACAATCATCATTCAATATCTATTTCTTGGTTGCCTTCAAATTTGGGCTTAC
TAAAATCGATTTAAAATTCTCGTGTTCCAGTTGAAGCTCACTTCAAGTAAATCAGGAATTACCATATTTTTCATTGCTCATAATTTGGAAACATGGCATG
ATATTTGATAGAAAATGTACAAATCCTCAGCTGCTCAGAAAGTTACAAAGGGAGGAACGCCCATGAAAGAGAATTCATGGCTGCAACAACGAACATAAAC
ACAGCCTATTCTGTCCAGCAGTCATGGTAATACAAGCTAAAAAGGACCGCTCTGGAACAATAGCTTGAAAAGCAATATTAATACAATTTCAGATGAACTA
TGTGCGTCGATGTCTTACTTGTGACTTCCTTGCAAGTAATGCGGTTCCTGCCTCATGCTTGGTGCCGCCGCATGCAATAAACCGATAACTCGAATCCTCC
GTGACTTCCACCCTCTCCAGCCAATCAGCGGCTTGCTGCGTGCGGGCGCTGGTGTGCGGGGGGCGCCGGCTTTATATTAGGGGCGGGGGAGAGTCTTGAA
G
>Locus_8503_Transcript_56/83_Confidence_0.566_Length_5268
CCTCGGCGCAGCTCGTCGCGTCACTCCTCGACGCCGCCTGCAACGCGCTCCACCTCGACGACGAGCACATTGAGCTGCTTCTCCTCAAGACGCTCCTCTC
GGCGGTCACCTCCACGTCAGTGCGCCTCCACGGTGACTGCCTGCTCCGCGCCGTGCGCGCGTGCTATGACATGTACCTGGGGAGCCGCAGCGCCGTCAAC
CAGGCCACTGCCAAGGCGTCGCTCGTGCAGATGCTAGTGATCGTGTTCCGCCGCATGGAGGCTGACTCGTCCACGGTGCCCGTACAGCCCATAGTCGTGG
CTGACGTGATTGAGCTGCCTGAAGCTGGTTCTGGTTCCTCGCCAACTGCTGACCCCAACGTTGTACAGGGATTCATTTCGAAGATCATTGGGGACTTTGA
CGGTGCACTCACACCTCTGGCACGGACAACTTCATCAGCAGGGGCCACCGTGGCTCATGATGGTGCGTTTGAGACAACAGCAGCAGCAGAGGAAGGGGCG
AACCCTGCGGATTTGCTTGATTCGACGGATAAGGACATGCTGGATGCCAAGTACTGGGAGATCAGCATGTACAGAACAGCTATTGAGGGTCGCAAGGACG
AGCTTGGTGTGGAGGGGGCAGTGGTGGGCACTTTGGAGGATGATGCTGATGTGAGGATCGGGAATAAGTTGCGAAGGGATGCTTTCCTAGTTTTCCGGGC
ATTGTGTAAGCTTTCCATGAAGACACCACCCAAGGATGCACCAGCAGATCCTATAGTGATGCGGGGGAAGATCCTTGCTCTCGAGCTTCTCAAGATCTTG
CTTGAAAATGCTGGAGCTGTGTTCCGGACCAGCGAGAGGTTTCTCGGTGCTATCAAGCAGTATTTATGCCTGTCACTATTGAAGAACTGTGCCTCATCAC
ATGTGATTGTTTTTCAGTTATCTTGTTCTATTTTTATTAGCTTGGTGTCAAGATTTAGACCAGGATTGAAGGCAGAGATTGGAGTGTTCTTTCCCATGAT
CATTTTGAGAGTTTTGGAAAATATCGCTCAGCCAAATTTTCAGGCAAAGATGATCGTTCTTCGTTTTTTGGAGAAGCTCTGTGACGACTCTCAAATCTTG
GTCGATGTTTTTCTTAATTATGACTGTGATGTCCACTCATCAAACATATTTGAGAGGATGGTGAATGGTCTACTAAAGACAGCACAAGGACCTCCTGCTG
GTGTCCCTACCACATTGGTACCGCCTCAGGATACTGCAATGAAAAGCGAAGCAATGAAATGCTTAGTTGCTATTCTCAGATCCATGGGAGATTGGATGAA
TAGGCAGCTGCGCATTCCAGGTCCTGATTCTCCTAAAGTAGAGTCAGAGAAAAACGATAATGATGGTGGAATCAAGCCTCCCCAGACGGATAACAACGGA
GATGAGTCTAGTGAAGCATCTGATTCACATTCTGAACTATCGAATGGGATTTCAGAGGCTGCATCTTTGGAGCAGCGTCGAGCTTACAAGATGGAGCTTC
AGGAGGGCATCTCTTTGTTTAATCAGAAGCCTAAGAAGGGAATTGAGTTCCTAGTAAATGCCAGCAAGGTGGGGGAGTCACCCGAGGAAATAACTTCTTT
CCTAAAAAGTGCTTCTGGCTTGAACAAGACTATGATTGGTGATTATTTAGGGGAAAGGGAAGATTTATCTCTCAAAGTCATGCATGCATACGTGGACTCA
TTTGATTTTCAAAGCATGGAGTTTGATGAAGCAATTAGAGCTTTCCTTCAAGGCTTCAGGTTGCCTGGAGAAGCTCAAAAGATTGATCGGATCATGGAAA
AGTTTGCAGAACGTTACTGCAAATGTAACCCAAAGGCCTTTTCCAGTGCAGATACAGCTTACGTCCTTGCTTATTCTGTCATAATGCTTAACACTGATGC
TCATAACCCAATGGTCAAAAACAAGATGTCACCAGAAGATTTCATAAGAAACAATCGTGGTATTGATGATGGGAAAGACCTGCCTGAAGAATTTATGAGA
TCCTTGTATGATAGGATTTGGAAAAAGGAGATTAAGATGAAAGAAGATGAATTTGTTCCCCATCAGCAACAATCAACAAGTTCTAACAAAATTCTTGGGT
TGGATAACATTCTTAACATTGTCATACGCAAGCGAGGTTCATCAATGGAGACGAGTGATGACCTCATTAAACATATGCAGGAGCAATTCAAAGAAAAGGC
CCGCATGTCTGAGTCAGTATTTTATCCTGCCACAGACGTAGTTGTTTTGAAGTTCATGGTCGAGGTTTGCTGGGCTCCTATGCTTGCTGCCTTCAGTGTT
CCACTTGACCAGAGTGATGATGAGATTGTCATATCCCAGTGCCTCGAAGGATTTCGCAGTGCAATCCATGTTACTGCAGCCATGTCAATGAAGACTCAAA
GGGATGCATTTATCACTTCGCTTGCCAAGTTTACATCACTTCACTCTGCTGCCGATATCAAGCAGAAAAATATTGAAGCCATTAAGGCAATTCTGTTAAT
TGCAGATGAAGATGGAAATTACTTGCAAGAGGCATGGGAGCATATATTGACTTGCGTTTCTCGTTTTGAAAATCTACATCTTGTTGGAGAAGGAGCGCCT
CCAGATGCCACTTTCTTTGCACTGCAACAGCCAGACCTTGATAAATCAAAACAGACTAAGTCATCGATTCTTCCTGTGTTGAAAAAGAAAGCTCCTAATG
CTGGTTCAGCTTCTAAAAGAGGTTCCTATGACAGTGCTGGTGTGGGTGGTAAAGCTTCTGGTGTTGATCAAATGAATAACGAGGGGACAAACCTCTTGAC
AAACCTCTTGGAGCAAGTTGGGATGGCTGAAATGAACCGTGTATTTGTAAGAAGTCAAAAGCTCAACAGCGAGGGCATAATTGATTTTGTGAAGGCTCTC
TGCAAGGTTTCAATGGAGGAATTACGTTCTGCATCTGATCCACGAGTTTTTAGTCTGACAAAGATTGTTGAGATAGCGCATTATAATATGAACCGCATCA
GGCTTGTCTGGTCAAGCATATGGCATGTCTTGTCTGAATTTTTTGTCACCATTGGCTGCTCAGAAAATCTTTCAATTGCAATATTTGCTATGGACTCATT
GCGGCAGTTGGCAATGAAGTTCCTGGAGCGAGAAGAGCTGGCTAACTACAATTTCCAAAATGAATTTATGAAGCCTTTTGTTGTTGTCATGAGAAAGAGC
CGAGCTGTTGAGATCCGAGAGCTAATCATTAGGTGTGTCTCTCAAATGGTCTTGGCCCGTGTTAGTCATGTGAAGTCAGGGTGGAAGAGCATGTTTATGG
TCTTTGCGACGGCATCGTACGATGATCACAAAAATATTGTTCTATTGGCCTTTGAAATTATAGAGAAGATTTTGCGGGAGTACTTTCCCTACATTACTGA
GACTGAATCAACTACTTTCAATGATTGTGTAAACTGTCTTATTGCATTCACTAACAGTAGGTTTAACAAGGATATAAGCCTTAATGCAATTGGCTTTCTT
CGATTCTGTGCGGCAAAGTTGGCCGAAGGGGACATTGGATCCTCAAGGTTGAAGGAGAATCCCTCTTCAAACAGTAATCCACCCTCACCTCATTTGACCA
ATGATGGAAAGCAGGAAGGTGCAGTTCTTGCCGATAAGGATGACCATATACACTTTTGGTTTCCTTTACTAGCAGGGTTGTCTGAACTTACCTTCGACCT
AAGACCTGAAATCAGAAAAAGTGCTTTGCAGGTGTTATTTGACACATTAAGAAATCATGGTCATCTTTTCTCTCTGCCTTTATGGGAGAAGGTGTTTGAT
TCAGTGCTTTTCCCAATATTTGATTATGTACGTCATGCTATTGATCCATCTGGTAGTTCACAAGGGCAAAGTGTGGAAAATGATCCTGCAGAATTTGATC
AAGATGCCTGGCTATACGAGACATGCACTTTGGCCCTTCAGCTAGTTGTAGATCTGTTTGTTAAGTTCTATGACACGGTGAATCCACTTCTAAAGAAGGT
TCTCTCGCTCTTGACGAGTTTCATAAAGCGTCCTCATCAGAGTCTTGCTGGTATAGGTATTGCTGCATTTGTTCGTTTGATGAGCAGTGCAGGATCTGTG
TTTGTGGACGAAAAATGGTTAGAGGTTGTATTGTCCTTGAAAGAAGCTGCCACCGAGACACTTCCCGATTTCTCTTATGTTGCATCTGGAGCTTACCTGG
AAAATGTTCCAACGGAAAATGAAGGCTCTTTGGATAGGAGAGAAGATGAACCTCGACCATCAGAAGATGACAATGATGAAACCTCTAGATCAAGGAACCT
ATATTTCGCAATTGGCGATGCCAAGTGTCGAGCTGCTGTGCAACTCCTGCTGATTCAGGCTGTGATGGAGGTATATAACATGTACAGAGCTCAGTTGTCA
GCACAGAACACAGTAATCCTCTTTGAGGCCTTGCACACTGTAGCCACTCATGCGCACAAAATAAACAGTGATAATGACCTGCGATCCAAGTTGCAGGAGT
TGGGCTCGATGACCCAAATGCAGGACCCACCATTGTTACGCCTTGAGAACGAATCATACCAGCTGTGCCTCACTATCCTCCAGAACATATTCTTGGACAG
ATCTTCTGACGAAGGGAGCATAGAGGTGGTGGAGAGCCACCTTATTGGCTTGTGCAAGGAGGTCCTTGAGGTATACTTGAGCACAGCAAGACCTGCTCAG
CTTTCCAGTGGCATGCAGCCGCTTGGCCATTGGCTTATTCCCGTCGGTTCATCGAAACGGAGAGAGCTTGCAGCTCGCGCACCCCTTGTTGTCTCGACTC
TGCAAGCTATCAGTGGCCTAGGTGATTCGCCTTTCGAGAAGAACCTTGGCCAGTTCTTCCCGCTCCTAGCTGGCCTCATAAGCTGCGAACATGGTTCCAG
TGAAGTGCAGGTGGCCTTGAGTGATATGTTTAGCACTTGGGTTGGCCCGCTCGTGCTCCAGTCCTGCTGATGAGCTGAGCGCCATGTGTTATATAGTATA
CTTTCACATGTAAGTTATTTTTTGTTTAAGTGAAATCCAGTTCTGTTGCGCCGTTCCCCCATTTTGTTCTGTTCATCATTTAGTTGCTGGGGGAAATTGT
ATTGTGCCGAACTAATCGTGCAGATTTTTTTCTTTTTTTACATTCTTTTCCTGTTCTCATTGGAAGGAGTGTATGTTACTATAGAGGACTGAATTCTGTA
CCGTAGTTTAGTTAAAATTATGCCCATTTTGTTCGCCACGTACGGAATCAGATCTATTCAATAGTTTC
>comp79373_c0_seq24
GAAACCTGGTAAACCCTGGCCACGGACGCGCATGCCATCCAAGATGACGTAGTCCACACCGAATCTTCCACGTCCGCACACGCGAGCACAACGCCACCGC
AGGCCAAGTGCCGTCTCCACCACAACTCGCTGATCCCTTCCTCCTCGCCGGCTCTCTCTCCATCCCTCGTCCTTCCCCATCTGTCCCTCCCTTGGCTTCT
TCCTCCAAGCTCCAACCAATCCCCACGCGCGCGCGCGCTATAGCCTACAAATAAATACTCGCCACTCTTCCCTTCCTCCCGCCTATCGTCTTGCCAAGCG
CTAGAGCAGCTGCGCCATTGCACGCTTCCGGCCCGTCGTCCTGCACATCTGTCCTCCCCGTACTGCCTTGTACAAACTCCGTATGTAAACTACTGGGTTG
TCGCCAGTGTTCGTGTTTGCTGATGCTCATCTAATTATCGTCGAGGAGATCCAAGGAATTGTCCGCGGAGCGCCGGGGAACAAAGGCTGCGCGCGTTTCA
AAATCAGTTATTGAAACGGCAAGATGGTTAATTTTGGAAAGAAGTTGGCTGCAGATCAAGTAGAAGAATGGAAAGGGTACTATATCAATTACAAACTGAT
GAAGAAAATGTTAAAGCAATATGTTCAACAGACCCAATTAGGCGGGAAAGATTGTGAACAAATCCTTAAAGAGTTCTCGAGGATTCTTGATGACCAGATT
GAAAGGATTGTGCTTTTTCTGCTACAACAACAAGGCCACCTTGCCAGCAGGATTGAGGAATTGGGAGAACAACGCACTGCTCTTATGGAACATTATGATA
TATCACAAGTTTCCCAGCTACGTGATGCGTATAGAGAAGTTGGGAGAGATCTTATTAAGCTTCTGCGCTTTGTTGACATGAATGCTACTGGTATACGCAA
GATACTAAAGAAATTTGATAAGCGTTTTGGCTATAAGTTTACATATTATTATGTCACCACTCGTGCAAATCATCCCTATTCTCAGCTTCAACAAGTATTT
AAGCAAGTGGGAATTGTAGCTGTTGTAGGTGCATTATCGCGCAATCTTGAATATCTGCAACATCATCAAGGAAGCTTTGTATCCATCTATGATCATCCAT
CAGTTACCTTGAAGGACCCTATAATAGACGAAGTAAACCATGCGGTACAGAAATTTACGCATGCCACCAATTTTATGAAATTCTTGGGACAACACGCGCT
TATTGTTCCAGAGGATGCACGAAGCGGGTCTGAGGATCTTGTTGATGATCAGAGCTACCATTTCATGTCTCTGCTGCTTAACCTAGCGAACACTTTTCTT

TACATGGTGAACACATATATCATTGTGCCAACTGCAGATGACTATTCAGTAAGCCTTGGGGCTGCTGCGACTGTCTGTGGTGTAATTATCGGATCAATGG
CAGTCACTCAAGTGTTCTCCTCGGTTTATTTCAGTGCATGGTCAAATAAGTCATACTTCAGACCACTTGTATTCAGTAGCATTATGCTATTTTTTGGGAA
CCTGCTATACGCATTGGCATATGACCTGAATTCATTAATAGTTCTCCTGATTGGACGACTACTATGCGGGTTGGGCTCTGCAAGAGCAGTGAACCGTCGC
TATATTAGTGATTGTGTGCCTCTCAAAATCAGGCTACAAGCTTCTGCAGGATTTGTCAGTGCTAGTGCTCTTGGCATGGCATGTGGCCCTGCTCTTGCTG
GTTTCCTCCAGACAAAATTTAAGATATACTCGCTCACTTTTAATCAGAGCACATTGCCCGGATGGGTCATGTCCATTGCTTGGCTTCTTTACTTGTTGTG
GCTGTGGTTTACATTCAAAGAGCCAGAACACTTCGCTAAAACTGTCATCAATGCACAGCCGTCTGAATCAGGTCACCAAGGAAGTGCTAATTTGGAGGAA
GGTCTAGCTCAACCATTGCTTCTGGGTACAGAACAAAGACAGGGCGAGAATGCGGATGACAATGATGATAATGAAGTAGACTCTGAAAACTCTCATGAAC
CAGCAACATCAATTACTTCAGCATACAGATTGCTGACACCATCTGTGAAGGTTTCCTATGCTTCCCTTATCGAGTACTCTTTTGTCTTGTGTTCCTCTCA
AGCATGCCTAAAACAAAGTTCGCAGTCTAAATAGGGTTCTTTTGTAAAGAAAGTAAAGTAAATGAACCGTGTAACATTAGGAAATGTATCTTAGTGTTAG
TGATAATGATACTAGAAGGTACATCTGCAAGACACAAGCATGCACTGTACTTACACGCTATATCAAGTGAATTGAAACCAGATGAAAAACCTTCTTTTCA
TCTTTGGACCTTTGCATTTTCAATTGCCCTTGACCTCTTTCAGTGTATTTATAGGAGCTACCATCTTCTGTATCTTCCATTTCATATGTTAATAAGAGTG
GCTATTTCTCAGGTCCAGCTATTGATATACTTTATGCTCAAGTATGCTATGGAAATTTTACTATCTGAATCAAGCGTTATCACAACATACTATTTTAGCT
GGTCTACGAGTGCTGTGGCTATCTTTTTGGCAATTCTTGGATTAACTGTCCTTCCAGTAAATGCCATTGTTGGAAGCTACATTACAAATTTGTTTGAGGA
CAGGCAAATTCTGTTGGCATCTGAAGTCATAGTTCTCATTGGTATCATCATGAGCTTTCGTTTCACCCCTCACTACTCCATTCCACAATACGTCACTTCA
GCTCTCATCACATTTGTGTTTGCTGAGGTACTCGAAGGTATGTATTTTGTACATGTGTGCAACCATCTCAAAGCATCATGCACAGTAAGACATGAAAACA
ATCATACGCTATTGCTATAACCATCATACAATTTCTGATGACGAGCAATGCAGGAGTGAATCTGTCCTTGCTCTCACGAGTAATGTCGTCTAGGCTTTCG
CGAGGGACCTACAACGGTGGACTCCTCTCGACAGAGGCCGGGACATTGGCCCGTGTAATTGCAGACGCGACTATTACTGCAGCAGGTTATCTAGGCCCGG
ACCTCCTCCTCAACGTCACCCTGCTGCCACCTCTAGTGATCTGCATAGTGTCCATCGTCGCAACGTTCTGCACTTACAACAACCTTTGCTGATAAAGCAG
CCCCTCTGTGTCCAGCCAGTTAAAATGACTCCTGCCATATTGGGCCGTACCGTACTACTTGAAGTAGCATCAGCGTTGTAAATCTGGTAAATCTACAAAT
ACCATGGACGGTGCCGGTGTGGCGGTGTGTAGTGTATTGTTATGCATTCAACAACTTTCTTGTGTCTAGTAGAGTTAAATATTCGTCAAATTGAAAATTC
AAATGCATTAACCAAGGAACATCACTGTGTTGGAAAGACAAGATGATTTATTGCAA
>Locus_5983_Transcript_45/82_Confidence_0.325_Length_2408
TGCCATCTCCATCTTGTGCGTCTCTGTTGGATCTTTGTGAGTCATGGCCTCTTTGCTCTCTTGCTTTTCTTCGTTTCTTGTTGAACTCATGGTTCTTGTT
TTTCTTGTGCTTGCCAGGTAGAATTATTCAACCGGATATAGATTCCTTCAAGTAAAGGAGTGCTAGCGGTTTTTACCTGTTCGGGTGGGAGCACAAGTTG
AGATCTTTAATTCTAGCATGGGTTCCTTTGGGGTGAACTGGAATCAGAAGAACTCCGCGGTGTGGGATTGGGAAAAATTAGCTCCATCTGTTCCAAATGC
AATCGGAAACCCCAGGAACGGAGCTACGGGGCATGGATCTGTGAATTCATCTGGTGGCACTCTCACTTCTAGCTCAGAGCTAGGGCATGGTTCATCCAAA
AGCTCCATGTCGGCATCCATTGATTCACCGTCCAAAGTAGGGAACAGCTTGGAGTTCAATTTTGCTGCTGTCAATACGTATGGTAAGAACATGGATAAGG
ATTGTAGAGTTGACGACTCAGGGACTTGTCCATCGTCCATGATAGCATTCAGCCATGGAGAGCCATTAATCAGTCTTAAGCTTGGAAAAAGGACTTACTT
TGAAAATGTCTGCGGAGGACAGGATTCCAAGAGCACTGCACCTTCTACCAGGACTTCTCCATCAACCGTTCTCAAGAAGACCAAGGTGTCTCAGCAGAAT
ACACAAATGTCATACTGTCAGGTTGAAGGTTGCGGAGTTGATCTATCTTCTGCTAAAGATTACCATCGCAAGCACAAAGTCTGTGAAGCTCATTCTAAAG
CTCCCAAGGTTGTTGTTGCTGGTCTAGAGCGCCGTTTTTGCCAGCAGTGTAGCCGGTTTCATGGTTTAGCTGAGTTTGACCAGAACAAACGAAGCTGCCG
TAGGCGTCTTACTCATCATAATGCACGGAGGAGAAAACCACAGGCAGATACAATTTCATTCAGTTCATCGCGGCTCTCGACAATGTTTTATGGTAGTATG
GAGCCCTTTGTTCATGAGTATATATTATTCTCAAAAACACTATCTAAACTGATCACTCATACAGTACCTCTTTTGAGAGGCATTATTTCTGAACCCTACA
TGTTGAAAGAGCACTTTCTGTAGGTGTCAAATGGTTAATCTTCTATTATAGCTTGTTCTCTCCATATTGTCAAATTAATGACTGAAGGCTAATCCTTTAT
CCAAATTTATACTTCTGAATATGATGCTGCTGCTGCTGCTTTAAGCATTGTAAATATGCATTGGCTTAGATTGTCGCAGATGCATATTCACAGAGTTAGT
CGATTATTTGGAAGGGGACTTAACCCAACTTCATGACACTTTTAGTATGCACATACGCTTCTCTTTTGGCGGGACTACTCTTAGAATATTCTTCTTAAAT
TATTATAAATGCAATTTTGCTTCTGGGAGAACACTTAAGTTTTAATGCTGGTATTGTTCGCATTTGCAGATACAAACCAGCAGAAAAATCTTTTCTGTAG
TCAACCTCATTTTGGCCAAGCGAGAAGCAATGCAGTTTTTTCTTGGGAAAACTCGGGAGATTCCAAATTTATGGAAACCAAACATCTGTTGATGCAGCCA
ACGAAAATGTTAGGTCTTGATGGGCTGCATTTCTCAACCCCCCAGATATCAAATAGTGTTGTGGCTCACTCTGTACATCATCGTAATTTCGATGGGCTCA
TGCCATTCAAGGGAACCAACACAAAGGTCCTCAACCAAGGCGTGGAAGCTTCTGCGATCGCTTCCAACTCGAGTGGAGACCCGGATCTTGGGTGTGCTCT
CTCTCTTCTGTCAATCGGTTCGTGGGTTTCAAGTTCAACCGTCATCCAGCAGCCTAGTTCTCATGTGCACGCTGGTGTGGTGCCGCCCCTCGCCACCGTT
GCCGTCTCCAACCCTGCAATGCATCCTCTGGACTCATCCCCAGGAGGATTCTGGCAAGACGTCCCTCCCCCGCTCGATGAAACTCCACATATTCAGGCAT
TCACGCACCTCTGATCAAATGCTGCATCCCCTGGGAACAGCAGGTGGTTATACCTTCCGTAATCGTCCTAGGTTTGCTTTGCCAGTGACTGGCTCTGCAC
AATGTCGATCCATCACCGGAGGCATTTGGTAAGGTGAAGACTAAAGACTCAAGAATAACCGGAGACTAACTCGCTTCTCTTCCAGGCTCAAGACTGTTTG
GGTCCAGCGGTGTTGCTACCTGATCCTGTTTTCGGTTCTGAGTACATCCATTACTAATCATGGGATTACTTGTTTAGGCCAACCAGACATATTCTGGGGT
GAGATTACGACATGTTAATTTACATTGACGGTGTTTCTACATGTGTTAGTACTAATGATGTTATCGCTCTTGGCCCCGTTACGTTTCAAGATCTGTTACC
ATTTGAGT
>comp76332_c0_seq42
CAATTTCTATCAATAATTCAAGTGTCGCACAGCTACAACGATGCTCCTAATTATGAAGAGAAAAAAACAGATGAATTAAAGGCTGAAGGTTCATTCAACC
ACCAAGCACCCAAAATGTCGATCTGCATATGCACAGAACCTCAGTACAGTTCACTGGTTGAGAAAATAGACTGCAAATGTGCAAATAATACAAATTGTTA
CTGGAAGAACAGTAAACACAATACCAGAAATCCTTCTAACGGTAACCATTGGAGGAAGCCTTGTGATCCTTTTCAACCCAAGTACATATATTGAAGATGA
AATTAACAAAAGATAGAATTGATAAAGCATAAGAAACAGAGGAACAAAAAAAAAGTACCTGGTCCAAGTTCCCATCCTCTGAAAAAGATCAGCAGTCAAT
AGATGCTTCACTCGTCCTCCAGAAAAATGAGTGCCGCATGAATCCTTTCCGGTTTATAGTGAAACATTTTAGACGATGACCTTTAACATTCTAGAAAACG
AAGAGAAAACCTACAGTCATCTAATTTCAGCTTAAACATGTTCCAGCAAGATCATCACCATCCTGACATGTTCCGTTTTGTCCCTAGACCCGAGCACAAG
GTATTGCGAGGCTAATTTAGCTCAATTCAGTTGTCAGAGCAATTGTGTGTGAAAAGTGTGAACAACAGCATAGGAGACAATACAGCAAGTGTAGCGTGCC
AATATGTGTCCAGCTTGATAGTTCACTATAATAAAGTTCTTTAATTCAAGTATTTGAATAATATAGAATCTCAACCTTCAAGTTTAAAAGGTTTTGTATT
TGCTATTATAATCGTTGCCACCCCCTCACCCCTCTCAAAAAGAGAGTTACTTTGTAGCGCACTGTAAATCTCTTGATTGAAATAGTGGCATGTCATCTTA
TTTAATTCAGTTATTTCATAAGGTACAATTTGTTTAGCTGACAGCTCAGACATGCTTCTATGCAACATGTTTATACCAATGCTGTATTTTGGCAATCCTG
GATGTGGCATTACTTAACTGTGCTTTCATATTCAATAGTTCTGGCAAAGGGAGGCTGCAAGCTTGAGACAACAACTGCACAACCTGCAAGAAAGTCATCG
GTATGGCTAGAAAACCCTGCAAATTTTACAAATGTACAAGTCAGAAAATTTGCCTAATGTAAAAGGATCAAACTACAGGATTTGTCAGCATGAGCACAAG
TGCCTAACTTCCGAGTTAGATAACTCTGGAGGAAGGAAATCCTTCCTACTGTTTGTTTCAAGAATATACAAAAATTCAAAACAACATTAGAATAGAACCA
AAAGAAGAAACAATATTCACCATGTAGCAATACTCAATTCCTGGTTGTCTTATTTATAAAGGTATAAAACTAGGTTCTGACGAGACAATTATGAATCAAC
TACACCATGTTACAGATCATCCATAACTCTTGGTGATGCAAATTAAGGGCTGTAAAGTGACAAGTATACCTCCACCAACTAATTTGTGCAGAGTGTATAA
AATTGTAAAACAATGGATAAGAATGAATTTAGTATACAAGATAGCATACAATGCCCAAGTTTAAGGAGACATACTGATTTACTGACTCCATAGGACAATC
TTTTGCTTATGAGGACATCAGTTTTCATAAACAATATCAATGTAAAAGGAGGTAACAGTTTATGAATTTCCTATGCCAAGCCATTTATAAGTCAAAAAAT
GTACCTATTTAGCTTTTATGCATTCCAATTAACTTCCAAAGAAGAAGAGTACATACTAATGGAAAACAAATATGATATTATCATCTGTTTTGCCCGGTAG
TTTCTTGAGTCTCTGATTTGTCCGCACTTCACTCCCAACCAACACAACATTAATACACATGGGCAGAGGAATATCAGGTTATCAGGTAAACTGAATACAG
TTTGATTGACTGCAGTCAGTTGATGGGACAAGATCTTTCTGGATTGAGCGTCAAGGAACTCCAAAATCTAGAAAATCAGCTAGAAATGAGCCTACGTTGC
ATCCGGACAAAAAAGGTAAGGATGCTAGCCATATTTCTGTTTTTCGTTACCAGATTGCCTCATCCAAGAATACAACTGATGATGACGTTTTTTACTTTCA
GGACCAACTCTTGATTGATGAAATTCACGAACTGAATCGAAAGGTTCTGGAATATTCTGTTATTACTCTGCAATGAGAGGTCTCTGAGTGGATTTCAAGA
AGCTGACTCTACTGGCTCATTTTCAGGGAAGTCTCATGCAACAAGACAAGATGGAACTATACAAAAAGGTCAACCTAATCCGTCAGGAAAACATTGATTT
ATACAAGAAGGTATTTGATAAATCGTGAATGAAAATTCACAGGATATACACCCCATCCATTATTGACTGAGGTTTATTTCTCTACTCAGCTCTACGAGAA
AGAGGTGACAAGTGAAGTCAACCGAGATTCATCAACTCTAAACTTTGCAGTTGTCGAGAACGTCAACATTCCTGTTCATCTTGAACTTAACACTCCACCG
CAAGAAAATGATGTTGAGCAAACTGCACCTCCTAAACTGGGGTAACCCTGCTTTTTTCTGCAAAGGAA
>Locus_11912_Transcript_57/63_Confidence_0.288_Length_2961
TTTTCCTGGCGCCTCCCGCCGGGCGCTCGCCCCGGATTTCTCGGCTCCCCTTTCGGTGGATGCCTTGACCGGGGTGGGTTCTTGGCTGCGGCGGCGCGTG
ATCTCTGCGTACTTAAGGAGGGGTTGCTAGCGGGGTTTGTGGGAGAAGGCGAAGGAAGCCGGGGGCTGCGAGGCGTTCATGGGTTAGATCTGGCCGTCCG
GCGCCGCAGATCTGAGCGAGGTTTCGGTGGAGGGATCGGGGCCGAGAGGTCGCCGTCTTCGGCTCATTTGGAGTAGCTTGGGCCTCTCATGTCCAAATCG

GGTATGGATGGTGGATGCGGTAACTGTCGGAAGGAGGGCTTGTTGCAGATCTGAAAGCCATCCAGCTCCCTTGTTCAGTTTACCTGCTTGCTGGTCAGCT
GGATGGAGTGAATGGCAATGCTTTCTGATGTTTGACTGCCGTTAGTTGCTGCTTAGTGTCTGTACAAGAAATTTAGCAGGAGGTGGTGTGCTTATGAGAC
TCTTCTAGGTGCTGATATCTTGGTCTCTGTTGAATAACGCTGGAGTTGGAAGGAAGAAGATCACATTCCTGTGTTTTTGTTGGTATCTGTGTCTTTCTTG
AATAACCTTGAGGCTGGAGGTTATCAGATCCAGTTCATGTGGCCCTGCTAATAAGGTGCTGGTGGGGGAAGGTACCATGACCTACTTCCCTGAGGAGGTG
GTGGAGCACATATTCAGCTTCTTGCCGTCGCACAGCGACCGGAACACAGTCTCGCTCGTGTGCAAGGTGTGGTATGAGGTCGAGAGGTTGAGCCGGCGAG
CTGTCTTCGCGGGGAACTGCTACGCGGTGCGCCCGGAGCGGGTTGTGCTGCGGTTCCCCAATGTGAAGGTCCTGACGGTGAAGGGGAAGCCGCACTTCGC
GGACTTCAACCTCGTGCCGCCTGATTGGGGTGGCTACGCGGGGCCATGGATTGAGGCGGCAGCGAGGGGCTGCGTGGGTCTGGAGGAGCTGCGGATGAAG
CGGATGGTGGTGTCGGATGAGAGCCTTGAGCTGCTAGCTCGGTCCTTCCCGAGATTCAAGGTCCTCGTCCTCATCAGTTGCGAGGGGTTTAGCACCGATG
GGCTAGCAGCTATTGCGAGTCACTGCAAGCTCCTGAGGGAGTTAGATTTGCAGGAAAATGATGTGGAGGACCGTGGGCCTAGGTGGCTCTCCTGCTTCCC
TGATTCCTGCACCTCTCTTGTCTCCTTGAATTTTGCCTGCATCAAAGGGGAGGTGAATCCTGGTTCATTGGAGAGACTTGTTTCTAGGTCCCCGAACCTA
CGCAGTTTGAGGTTGAATCGATCTGTATCAGTAGATACACTCTCAAAGATATTAGTACGCACCCCTAATTTGGAAGACCTGGGGACAGGGAATTTGACGG
ATGAGTTCCAAACTGAATCCTACTTCAGGCTGACTAATGCTTTGGAGAAATGCAAAATGTTGAAGAGTTTATCAGGATTTTGGGATGCTTCTCCTGTTTG
CGTTCCATTTATCTATCCCCTCTGTCATCAACTAACTGGACTAAACTTGAGCTATACTCCCACTCTGGATGCTTCTGATCTCACTAAAATGATCAGCCGC
TGTGTGAAACTCCAACGTCTTTGGGTACTGGACTGCATCTCAGATAAGGGTTTGCAAGTGGTGGCCTCCAGTTGCAAAGATCTACAAGAACTCAGGGTGT
TCCCATCAGATTTTTATGTTGCTGGTTATTCTGCCGTGACAGAAGAGGGGCTTGTTGCAATATCGTCAGGCTGTCCAAAATTGAGCTCTTTGCTGTATTT
CTGTCACCAGATGACCAACGATGCACTTATTACTGTAGCTAAGAACTGCCCAAATTTCGTAAGATTTAGACTCTGTATTCTTGAGCCAAAGAAGCCAGAC
GCCATGACAAACCAGCCGTTAGATGAAGGCTTTGGTGCAATTGTTCGTGAATGCAAGGGGCTAAGGCGACTGTCAATGTCGGGTCTTCTCACTGACAAGG
TTTTCATGTATATTGGAAAGTATGCCAAGCAACTTGAGATGCTTTCTATAGCATTTGCTGGAGATAGTGATAAGGGGATGATGCATGTTATGAATGGATG
CAAGAATCTAAGGAAGCTGGAGATTAGAGACAGTCCATTTGGTGATGTTGCGCTCTTGGGGAATGTTGCCAAGTATGAAACAATGCGATCCCTTTGGATG
TCATCATGCAATGTCACATTAAAGGGGTGCCAAGTCCTTGCGTCTAAGATGCCGATGCTCAATGTGGAGATCATGAATGAGCTAGATGGAAGCAGTGAAA
TGGAGGAAAACCATGGAGATCAATCTAAGGTGGAGAAGCTATATGTTTACCGCACAACTGCTGGAGCGAGGGATGATGCACCAAATTTTGTTAAAATCCT
ATAGTTAGCTGAGAGTAGCCTGGTATCTCCTCACTCTAAAAGATGGTCGGTGGAATCCGGCAGGCTGGGGGGGCCGCGGCAACAGTGGTAAGTTCTTGAG
GTGCCTCTCTCTCCCTTTGCAGGAGTCAGGAAAAGCCACACCGGTGTGCGCCCTTATCTTGGAGCAACTGGCCGGGATCCGTCATCATCCTCTGTACCAC
CGCCCCTACCCTTTTAGTGTTTGGTCACTCATATGGCCACTGAGGCCCTTTATCATCCTGTGCATTCTCACTCGAGAGGACAACGTTCTTTTTCTCCCTT
TTATCCCAATTGTAATCGTAGTGTACCAGTACCCATTTGCTTTTGACATTTGCACAGTAGTGGATTGTGTTACCGCCGCCGACGCTGCTGCTGTAATAGT
GTTTGTTTCGGCCTGAATGATCGGGACATGACATTCCGGGGACGACCATTGGTTTCTTGCTCCTGTGAATGCATTGCATGGAATAATTTGAGGTTCACCT
GTGACATTCTTCTATTATTTGGTTTTGAATGATTGGTGCCCTGGCGAACGTTTTTGACTGT
>Locus_5983_Transcript_76/82_Confidence_0.132_Length_2066
TCTGTTCCAAATGCAATCGGAAACCCCAGGAATGGAGCTACGAGGCATGGATCTGTGAATTCATCTGGTGGCACTCTCACTTCTAGCTCAGAGCTAGGGC
ATGGTTCATCCAAAAGCTCCATATCGGCATCCATTGATTCGCCATCCAAAGTAGGGAACAACTTAGAGTTCGACTTTGCTGCTGTCGAGAGGCATGGTAA
GAACATGGGTAGTGATGGTAGTAGAGTTGAGGACTCAGGAACTTCTCCATCGTCAGCGTTCAACCATGGAGAGCCATTAATCAGCCTGAAGCTTGGAAAA
AGGGCTTACTTTGAAAATGTTTGCGGAGGACAGGATATCAAGAGCTCTGCACCTTCAACAGTGACTTCTCCATCAATGGTTGTCAAGAAGACCAAGATGT
CTCAGCAGAACGCACAAAGCTCAAACTGTCAGGTTGAAGGTTGCAGAGTTGATCTGTCTTCTGCTAAAGAATACCATCGCAAGCACAAAGTCTGTGAAGC
TCATTCTAAAGCTCCCAAGGTTGTTGTTGCTGGTCTAGAGCGCCGTTTTTGCCAGCAGTGTAGCCGGTGATATTTCCTTGACTGAGTGCATTTTCATTAT
ATAGTATGCCTCTTTGTATTCACCGAATCCTTTTTTAATGTTGGCATCTCTAATTGAAAAGAGAGAACATAAAAAATGTGATCAGTTGAAAGCAATACAA
CTATTTAGTGGTAGAACGACCAAGGACCAACTGCTAGATGATGCCCAATTATTTTGCATGACTGAGACTTCCAAAGTATCTGTGATGTTTATTACTCCTT
TGCTGATCAAGGTACACAAGAACACTGGAGTGACATGAACACTGAAGAAAATTGGCATTTCTTTTTGTTTCTGGCACTAGTTTGTTATTGATTTGTAAGC
GGAATGCTGATAAACATCTTGAAAAGTGAAATTCAGATAAACTAATTACAATACAGTTTGATCTTTTAGCGACATAATGGACATTTATTTCCTTTTCTTT
AATTCTTCATTGGTTTGTAGGTTTCATGGTTTAGCTGAGTTTGACCAGAACAAACGAAGCTGCCGTAGGCGTCTTACTCATCATAATGCACGGAGGAGAA
AACCACAGGCAGATACAATTTCATTCAGTTCATCGCGGCTCTCGACAATGTTTTATGATACAAACCAGCAGAAAAATCTTTTCTGTAGTCAACCTCATTT
TGGCCAAGCGAGAAGCAATGCAGTTTTTTCTTGGGAAAACTCGGGAGATTCCAAATTTATGGAAACCAAACATCTGTTGATGCAGCCAACGAAAATGTTA
GGTCTTGATGGGCTGCATTTCTCAACCCCCCAGATATCAAATAGTGTTGTGGCTCACTCTGTACATCATCGTAATTTCGATGGGCTCATGCCATTCAAGG
GAACCAACACAAAGGTCCTCAACCAAGGCGTGGAAGCTTCTGCGATCGCTTCCAACTCGAGTGGAGACCCGGATCTTGGGTGTGCTCTCTCTCTTCTGTC
AAACGGTTCGTGGTGTTCAGGTTCAACTGTCATCCAGCAGCCTAGTTCTCGTGTACACGCTGGTGCGGTGCCGCCCCTCGCTACTTTTGTGGTCTCTAAT
CCTGCAATGCATCCTCTGGACCCAGCCCCAGGAGGGTTCTGGCAAGACGACCCTGCCTCGCTCGATAGAACTCCGGAGATTCAGGCATTCGGACACCTCT
AACTATGGTCAAACAAATGTACTCATGCTGCATGCCCTCGGAACAGCTGATTTAGTCACCTCATCATAATCTGCAACTCTGCTTTGCCAGTGGCAGATCT
CTGCATAATGTCAGTCCTATCATCGGAGGCATTTGGTGAGGTGAAGACTGAAGAATAATATAGGAGGCAACCCCCTGATCTTCCTGATTTAGGATGCATC
TAGGATCCAGTTGTGTGTGTGCTTGTGGTTTTGCCAGAACCGGTTTTTTGTTTGCCGTCCCGTTAGAGAGAAGGGATCAAGTACATTCATGACTGCTCAT
GAGATTACTTGTTTCACTAATATTACATGATCTGGAGCGAGATTTCGCTCGGTTCACGTTGATGGT
>Locus_18892_Transcript_23/56_Confidence_0.279_Length_1152
ATCTGGGATTGTGGGAAGCAAGTCTACTTAGGTGAGTAACTGAGTTGAGTGAGTTCTATACGTTCCAGCTCGAGCTTCCATTAACTGCTGATCAGTTTCC
GTCCGTTTGATCTGGTTTACGATGTGCTGATGCTGTTCTATTCAGGTGGTTTCGACACTGCTCACGCGGCTGCGAGGGCTTATGATCGTGCAGCGATCAA
GTTCCGAGGGCTCGACGCGGACATCAACTTCACTTTGAGCGACTATGAGGATGACTTGAAGCAGATGAGCAATTGGACAAAGGAAGAGTTCGTGCACATA
CTCCGCCGCCAGAGCACGGGGTTCGCGAGGGGGAGCTCCAAGTACCGCGGCGTGACGCTGCACAAGTGCGGCCGCTGGGAGGCGAGGATGGGCCAACTTC
TTGGCAAGAAGTACATCTATCTTGGGCTCTTTGACAGCGAAGTTGAAGCTGCAAGAGCGTATGACAGGGCAGCCCTTCGCTTCAATGGGAGGGAAGCTGT
TACTAACTTTGAGCCTTCCTCCTACAATCGAGGAGATGCTCTACCCGACACCGAAAAAGAGGCAATTGTTGATGGCGATGCGGTTGATTTGGATTTGCGG
ATTTCACAACCTAATGTACACGACGCTAAAAGGGACAATACCGTAGCTGGCTTCCAGCTAACATGCGACTCCCCTGAATCTTCAAATATGGTGGCCTCTC
ATCCAATGAGTTCATCGTCCCCGTGGCCTGTGTATCAACAGAGCACGGCAGTACCACCTCACCATCAGCGTTTGTACTCATCTGCTTGTCCTGGCTTCTT
TCCGAACCTCCAGGAAAGGCCAGTGGAGCGAAGGCCTGATTTGGGCCCCCAGTCGTTCCCCACCTGGGGACGGCAAACGCAGGGCTCCCCTCACATGCCG
TTGCACCACTCCGCAGCATCATCAGGATTCTCTACCGCCGCGAACGCCGGCGTCCCATTGCCGTCTCACCCGCCGGCGCCGTTCTCGGACCACCAGTTCT
ACCTCCCACCGACGGCCTGAGCTTGTTGGTCCGGTTCAACTCTTGGGACAGCAGCTGACGCTCTGTGTGTGAACGTCTCGCAGCTTGCCGGTAACAGCTG
TTAATTAATGGGAGAGAGAGAGAGAGAGAGAGAGAGAGAGAGAGAGAGAGAAAA
>Locus_2827_Transcript_105/166_Confidence_0.155_Length_3065
GGGGAGGCTGTGAGGTCGGTGGGTGTGCTGCTCGTCTGTTCCGGCGGCGCTCCCTTCGTCTTGCGGGAGCCTGTCGGTGTGTCTCGGCGGCGTTCGCTTC
GTTTGCAGAAATCTTCACGAGCTGAAAGTTGAGGCGAAACCGAGGAGCTAAGAGCAGCAGTTGAGGTGTTGAAGTGCATAGGACAACTAAGAGGAAGAGG
AGGACGAGATTTGTGTGGTTGGGCTTTTGCATTGGCGAGGGAACAATGGCTGCGGCGGTGGCGATGCGAGGGGGAAGCAGTGACAGCGGAGGGTTTGATA
AGGTTCCGGGGATGGACTCGGGGAAATACGTGCGGTACACCCCGGAGCAAGTTGAGGTACTCGAGCGGCTGTACATCGATTGCCCCAAGCCAAGCTCCTC
GCGGCGGCAGCAATTGCTGCGCGAGTGCCCTATACTTGCCAACATTGAGCCCAAGCAGATCAAGGTTTGGTTCCAGAACAGAAGGTGTCGCGATAAGCAG
CGGAAGGAGTCTTCGCGGCTTCAGGCTGTGAACCGGAAGTTAGCGGCAATGAACAAGCTTCTGATGGAAGAGAATGAGCGTCTTCAGAAGCAGGTCTCTC
AGTTGGTTCATGAGAATGCACACATGCGGCAGCAGCTGCAGAATACTTCTTTGGCCAATGATACAAGCTGTGAATCAAATGTGACTACCCCTCCAAACCC
TATAAGGGATGCAAGTAACCCTTCTGGACTCCTTTCGATTGCAGAGGAGACCTTCACAGAGTTCCTCTCAAAGGCTACTGGGACAGCTATTGATTGGGTC
CAGATGCCTGGGATGAAGCCTGGTCCGGATTCGGTTGGTATTGTGTCTGTTTCACATGGTTGCCGAGGTGTTGCTGCCCGTGCCTGTGGTTTGGTGAATC
TAGAACCAACAAAGGTCATAGAGATCTTGAAAGATCGTCCATCTTGGTTCCGTGATTGTCGAAGCCTTGAGGTGTTCACAATGTTTCCAGCTGGAAATGG
GGGAACAATTGAACTTGTCTACATGCAGATGTATGCCCCAACAACTTTAGTCCCTGCACGTGACTTTTGGACGTTACGATACACGACAACAATGGAAGAT
GGCAGCCTTGTGGTCTGTGAGAGATCCTTGAGTGGTTCAGGGGGTGGTCCAAGTGCAGCCTCTGCGCAACAATTTGTAAGAGCTGAGATGCTTCCAAGTG
GGTATTTAGTTCGCCCATGCGAAGGTGGGGGCTCGATTGTGCATATAGTGGACCATCTAGATCTCGAGGCGTGGAGTGTTCCTGAAGTGCTTCGACCACT
CTATGAGTCTTCTAGAGTAGTTGCTCAGAAAATGACTACTGTGGCACTGCGCCACCTTAGACAAATTGCTCAAGAAACAAGTGGGGAAGTAGTGTACGCC
TTGGGCAGGCAACCTGCAGTCCTACGGACATTTAGTCAAAGGCTGAGCAGGGGCTTTAATGATGGCATTAGCGGTTTCAATGATGATGGCTGGTCTATAA

TGGGGGGAGATGGTATTGAAGACGTAGTTGTTGCTTGCAACTCAACTAAGAAAATTAGGAACAACAGCAATGCTGGAGTTCCGTTTGGAGCCCTTGGAGG
TATTATATGCGCGAAGGCATCCATGTTACTGCAGAGTGTTCCACCAGCAGTATTGGTTCGATTTCTGAGGGAGCATAGATCTGAATGGGCCGATTACAAT
ATTGATGCATATTTGGCTTCAGCGCTGAAGGCCAGTGCGGGCTCACTTCCTGGGTTACGGGCCATGAGATTTTCTGGGGGCCAGATGATCCTGCCACTTG
CCCACACAGTGGAGAATGAGGAGATTCTTGAAGTTGTCCGCCTTGAAGGACAATCTCTTACTCACGATGAGGCTCTTATTTCAAGGGATATCCACCTGCT
TCAGCTTTGCACTGGAATAGACGAGAAATCTGTGGGGTCCTCCTTCCAGCTCGTGTTTGCACCAATTGATGAACATTTTCCAGATGATGCTCCATTGATT
TCTTCTGGCTTTCGTGTCATACCACTTGATATGAAAACAGACGGCATATCTTCTGGTAGGACGTTAGATTTGGCATCTAGTCTTGATGTTGGTTCTGCCA
CGCCTCACGCCTCAGGGGATGCATCTCGAGATGATTGTAATTTGAGATCTGTGCTGACCATCGCCTTTCAATTCCCTTACGAGATGCAGCTCCAAGACAG
TGTTGCAACTATGGCCCGTCAGTATGTTCGCAGTGTTGTTTCTGCTGTGCAAAGAGTGTCGATGGCTATCTCTCCCTCCCAATCTGGTATAAATGCTGGG
CAGAGGATGCTTTCTGGCTTCCCTGAGGCAGCCACACTTGCTCGCTGGGTTTGCCAGAGCTATCATTACCATCTAGGGGTAGAATTACTTAACCAGTCAG
ATGAAGCTGGCGAAGCATTGTTGAAGATGCTCTGGCATCATCCAGATGCTATTTTGTGCTGCTCTTTTAAGGAAAAACCTATGTTTACATTTGCCAACAA
GGCAGGGCTTGACATGTTAGAAACATCCCTTATTGCCTTACAAGACCTCACATTAGACAAGATCTTTGATGAGTCTGGAAGAAAAGCATTATTCTCGGAC
ATCTCTAAATTGATGGAACAGGGATACGTGTACCTGCCTTCGGGCGTATGCATGTCAGGAATGGGTCGCCATGTTTCGTTCGATCAAGCTGTGGCGTGGA
AGGTCCTTGGTGAGGACAGCAGCGTCCACTGCCTGGCCTTCTGCTTCGTGAACTGGTCCTTCGTGTGAGCCTTTGCCATCCTCCGAATGCCGACTTGATC
TGTGACGAGGAGATCCATTTTTGTATGATCCAGAGAAGGAAAATCATCAACTGTGGCAAGTCTTAGTTTGCACTAGCTCTAAGTATGGGAATCATCAACT
GGAGAGTATGCAAATCTGATCTCGTTTCATTTGGCTGCCAAGCCGCGCCCTGGCTGCTTGTTTGGGATATTGTGTTTCCCATTTTATGCGTCGAACTGTG
GTACATTGCTTCAGTCTTCAAATGGCAGCTGAATTTTCAACTGGTTCTCTGTTGAGATGAAATTT
>Locus_15005_Transcript_8/11_Confidence_0.429_Length_659
AGGAGCGCCAGGCCCTCTTCAGCCGCATTGCCCCCGTGTACGACCAGCTGAACGACATGCTCAGCCTGGGGCAGCACCGGACGTGGAAGAGCATCTGCGT
CTCTTGGTCAAGGGCGAAGAGAGGGGATCGTGTTCTTGATCTCTGCTGTGGGAGCGGGGATTTATCGTTCCTGCTGTCTCAGAAGGTCGGCCTAGATGGA
GAGGTGATGGCTGTCGATTTCTCGAGGCAGCAACTGCAGACTGCTGCTGACCGTCAGGAGCAACGCTGGAAGCCGTGCTACAAGAACATCAAATGGATGG
AGGGTGATGCACTTGATTTACCTTTTGCAGACTGCTACTTCGATGCTATCATCGTCGGCTACGGATTGCGCAATGTTGTTGACAAATCCAAAGCAATGCG
AGAAATATTTAGAGTCCTGAAACCAGGATCAAGAGTGTCTGTTCTTGATTTTAACAAGAGCTCGTCTCTTTTCATGGCATCGTTACAGGGTTGGATGATT
GACAATGTCGTGGTTCCTTTAGCTAGTGGCTATGGACTCACTGAAGAATACAAGTACTTGAAAAGCTCCATATCGCAGTATCTAACAGGAGAAGAGTTGG
AGATGTTAGCCAAAGAATCTGGGTTTTCTCCTGTTAGATACTGCGATATGGAGCTTTTC
>comp79344_c0_seq14
CTCTCTCTCTCTCTCTCTCCAGCTAGCCGCCGTTTGACCCTGCAGATTGCTTCCTTCTGCACGGCGCGATGGTACGGGGAAAGACCATATTGAAGAAG
ATCGAGAACGAATCAAGCCGGCTTGTGACCTTCTCCAAGAGGAGGGGCGGGTTGTTCAAGAAGGCAAAGGAACTGGCAATCCTATGCGACGCTCAGGTCG
CTGTTCTCATCTTCTCCAGCACCGGCCAACTCTACGACTTCTCCAGCTGCAGCATGAAATCAATAATCGAAAGATACCGCCAAGTAAAAGAAGGTCAGCA
AAATGTAACGGCAAGTAGGGAGGCCAAGTACTGGCAAGGGGAGGCGGAAAGCTTGAGGCAGCAACTACACAACTTGGAAGAGAATCATAGGTAAGAAAAT
CCACCAACAGTATTGACAGAGGTGAATACTTTCTTACTGAGCAACCTTGGATGAAACTTAAAATGCAGTGTAATGGAGTCCATCTAATAGCAACGAATGT
GAAAACTTTAATAACAAGTCATATATTTTTATGTCCTTAGGGAGCCATTGGGGTCCATGGATTCTGTAACATCCTAGCTGGCATGTTTATAGTGGTCTTG
GCCTATGTGGTCTAGATGTTGAAGTGTGATGGGATTAGTTCTATCTTGGGTCTTTAGGTAGGTCAGAGCCTACATATAAACCTTGTCTTTTAGAACATAG
CACATCTGGATTAACCCTTTTACCAGAAGTGAACATGAAAGCATAAGAAACATGCAATGCCCATGCGTGAGCTTGCTCGATGTGGGACTTGATAATAAAC
AGGTTTTAGATATAGGGTGTTACAAATTCTTATTTGTGGTTGACAGAACAGGGAGCATGCATTGTTGTAGGGGAAATCATGCAAACGTTTTCAGGTTGTC
AATTTGGCACCACAGTACAAGCCTGAAACACAAGATAACAAGTGCAAACTGCCACAAGATGCACTTGCTGGGACAACATCTTTCTGGCTTGTCAATGAGA
GACCTGCAGACTTTAGAGAACCAGCTGGAAACGAGCCTAACTAATATTCGGCTAGCAAAGGAACGAATTATGATTGATCTGATTCAAGAATTGAACAAGA
AGTCAAGCCTCATAGACATCGAAAACAAGGAGCTACACCACAAAATCTGTGCCATTAGTCAAGAGAATGCCAATTTGAAGAAAGAGGTAACACAAAGAGT
ATCCACAATAATGTCTTAATTGTGAGCTATCTTCCGTCAGTGTTTATGTCTTTCCATTCATCAGGTACATGGACAGCCGGATGCCATTGAAGAAAATACT
GGTTCTGTTACTCAGTATAGTACTAACACTCCTGAAGACCTAAATGCGACGTGTCTCGAGCTACGCCAACCACATCATGTAGAAATAGAGCAACCAGATG
TGCCTACATTGGAACTGCGTCTCCACTGATTGCAAGAATCAGCAGCAGAATCATCGGAAATGAGGAGGCCAGCCATTCAACGCAAATTCATAAGACTTTG
TCTCCACTGATTAAATACAAAGAGGAGTTCCTGTGCAAATTACTATTATATGAATGTAACAGATGTAATTTATTTTTTATGTATCCGCAGTACACATATG
GCCACCATAAACTTATGTTCAAAAGGCATATATACCTATTGCTATCAAGCTACG
>comp79373_c0_seq33
GAAACCTGGTAAACCCTGGCCACGGACGCGCATGCCATCCAAGATGACGTAGTCCACACCGAATCTTCCACGTCCGCACACGCGAGCACAACGCCACCGC
AGGCCAAGTGCCGTCTCCACCACAACTCGCTGATCCCTTCCTCCTCGCCGGCTCTCTCTCCATCCCTCGTCCTTCCCCATCTGTCCCTCCCTTGGCTTCT
TCCTCCAAGCTCCAACCAATCCCCACGCGCGCGCGCGCTATAGCCTACAAATAAATACTCGCCACTCTTCCCTTCCTCCCGCCTATCGTCTTGCCAAGCG
CTAGAGCAGCTGCGCCATTGCACGCTTCCGGCCCGTCGTCCTGCACATCTGTCCTCCCCGTACTGCCTTGTACAAACTCCGTATGTAAACTACTGGGTTG
TCGCCAGTGTTCGTGTTTGCTGATGCTCATCTAATTATCGTCGAGGAGATCCAAGGAATTGTCCGCGGAGCGCCGGGGAACAAAGGCTGCGCGCGTTTCA
AAATCAGTTATTGAAACGGCAAGATGGTTAATTTTGGAAAGAAGTTGGCTGCAGATCAAGTAGAAGAATGGAAAGGGTACTATATCAATTACAAACTGAT
GAAGAAAATGTTAAAGCAATATGTTCAACAGACCCAATTAGGCGGGAAAGATTGTGAACAAATCCTTAAAGAGTTCTCGAGGATTCTTGATGACCAGATT
GAAAGGATTGTGCTTTTTCTGCTACAACAACAAGGCCACCTTGCCAGCAGGATTGAGGAATTGGGAGAACAACGCACTGCTCTTATGGAACATTATGATA
TATCACAAGTTTCCCAGCTACGTGATGCGTATAGAGAAGTTGGGAGAGATCTTATTAAGCTTCTGCGCTTTGTTGACATGAATGCTACTGGTATACGCAA
GATACTAAAGAAATTTGATAAGCGTTTTGGCTATAAGTTTACATATTATTATGTCACCACTCGTGCAAATCATCCCTATTCTCAGCTTCAACAAGTATTT
AAGCAAGTGGGAATTGTAGCTGTTGTAGGTGCATTATCGCGCAATCTTGAATATCTGCAACATCATCAAGGAAGCTTTGTATCCATCTATGATCATCCAT
CAGTTACCTTGAAGGACCCTATAATAGACGAAGTAAACCATGCGGTACAGAAATTTACGCATGCCACCAATTTTATGAAATTCTTGGGACAACACGCGCT
TATTGTTCCAGAGGATGCACGAAGCGGGTCTGAGGATCTTGTTGATGATCAGAGCTACCATTTCATGTCTCTGCTGCTTAACCTAGCGAACACTTTTCTT
TACATGGTGAACACATATATCATTGTGCCAACTGCAGATGACTATTCAGTAAGCCTTGGGGCTGCTGCGACTGTCTGTGGTGTAATTATCGGATCAATGG
CAGTCACTCAAGTGTTCTCCTCGGTTTATTTCAGTGCATGGTCAAATAAGTCATACTTCAGACCACTTGTATTCAGTAGCATTATGCTATTTTTTGGGAA
CCTGCTATACGCATTGGCATATGACCTGAATTCATTAATAGTTCTCCTGATTGGACGACTACTATGCGGGTTGGGCTCTGCAAGAGCAGTGAACCGTCGC
TATATTAGTGATTGTGTGCCTCTCAAAATCAGGCTACAAGCTTCTGCAGGATTTGTCAGTGCTAGTGCTCTTGGCATGGCATGTGGCCCTGCTCTTGCTG
GTTTCCTCCAGACAAAATTTAAGATATACTCGCTCACTTTTAATCAGAGCACATTGCCCGGATGGGTCATGTCCATTGCTTGGCTTCTTTACTTGTTGTG
GCTGTGGTTTACATTCAAAGAGCCAGAACACTTCGCTAAAACTGTCATCAATGCACAGCCGTCTGAATCAGGTCACCAAGGAAGTGCTAATTTGGAGGAA
GGTCTAGCTCAACCATTGCTTCTGGGTACAGAACAAAGACAGGGCGAGAATGCGGATGACAATGATGATAATGAAGTAGACTCTGAAAACTCTCATGAAC
CAGCAACATCAATTACTTCAGCATACAGATTGCTGACACCATCTGTGAAGGTTTCCTATGCTTCCCTTATCGAGTACTCTTTTGTCTTGTGTTCCTCTCA
AGCATGCCTAAAACAAAGTTCGCAGTCTAAATAGGGTTCTTTTGTAAAGAAAGTAAAGTAAATGAACCGTGTAACATTAGGAAATGTATCTTAGTGTTAG
TGATAATGATACTAGAAGGTACATCTGCAAGACACAAGCATGCACTGTACTTACACGCTATATCAAGTGAATTGAAACCAGATGAAAAACCTTCTTTTCA
TCTTTGGACCTTTGCATTTTCAATTGCCCTTGACCTCTTTCAGTGTATTTATAGGAGCTACCATCTTCTGTATCTTCCATTTCATATGTTAATAAGAGTG
GCTATTTCTCAGGTCCAGCTATTGATATACTTTATGCTCAAGTATGCTATGGAAATTTTACTATCTGAATCAAGCGTTATCACAACATACTATTTTAGCT
GGTCTACGAGTGCTGTGGCTATCTTTTTGGCAATTCTTGGATTAACTGTCCTTCCAGTAAATGCCATTGTTGGAAGCTACATTACAAATTTGTTTGAGGA
CAGGCAAATTCTGTTGGCATCTGAAGTCATAGTTCTCATTGGTATCATCATGAGCTTTCGTTTCACCCCTCACTACTCCATTCCACAATACGTCACTTCA
GCTCTCATCACATTTGTGTTTGCTGAGGTACTCGAAGGAGTGAATCTGTCCTTGCTCTCACGAGTAATGTCGTCTAGGCTTTCGCGAGGGACCTACAACG
GTGGACTCCTCTCGACAGAGGCCGGGACATTGGCCCGTGTAATTGCAGACGCGACTATTACTGCAGCAGGTTATCTAGGCCCGGACCTCCTCCTCAACGT
CACCCTGCTGCCACCTCTAGTGATCTGCATAGTGTCCATCGTCGCAACGTTCTGCACTTACAACAACCTTTGCTGATAAAGCAGCCCCTCTGTGTCCAGC
CAGTTAAAATGACTCCTGCCATATTGGGCCGTACCGTACTACTTGAAGTAGCATCAGCGTTGTAAATCTGGTAAATCTACAAATACCATGGACGGTGCCG
GTGTGGCGGTGTGTAGTGTATTGTTATGCATTCAACAACTTTCTTGTGTCTAGTAGAGTTAAATATTCGTCAAATTGAAAATTCAAATGCATTAACCAAG
GAACATCACTGTGTTGGAAAGACAAGATGATTTATTGCAA
>Locus_10795_Transcript_55/70_Confidence_0.316_Length_1886

ACATTAATCTGAGATCTGAACAATTTTCAAGTACCACATCAAAAGCTTCTGTTGAATGTTTATTTGAATGGAACACTTGAATGCATATCATGAATGTGGA
GGAATTGGGCGGCCAAGAACTAGGCAGACATCACATCCCATCTTCCTTCACGCAACTTATGTCTAGCAGTCTAGCTAGAAAAAGAAAAGAAAAGAAGGGG
GAAAACAAACTATGTACAGAGCAAGGCAAAAGAAAAGAAAAGAAGGAAAAAAAAACTATGTACAGAGCAAGGCCATCATCTCGGCATGGACCTCCGGTAG
GTGCTCAATTACTCAAATCCTATTGCAATCCTGTAAAGGATGGCTTTATTCTAGATCTGCATAGGTTACCACCTGCAAGAAAGCCACAACAAACAGCAGC
AGCCGAACAGATGCTCACCTCCCTACTGCGCTAACTCAAAACTGGCAAGAAGTGCCCAGCCTCTGCTTCGCCCTTTGCTCTGTAGCGCAGCACTTGCGCG
AGCGAAATGCCTCAGGCAGCAGCTCCTGGAACTTCTCAAGGAACAACCCCCATGTCTCTGGTCCCATGTCAGCCAGCTTGACGAAACTCACACTTGTTGG
GAGCATATCGTTTGCACTCCCCCAGTTACCAGTTTGGTTGCTGTTGCTCTCTGAAGTTTTCACAGAATTCACACCCTTTGGAACAGCAGACAGCCGATGC
TGGCTTTTGTCAGTTAGAGTGACACCCTTCAGTGAGGTTGACAGCTTCGAAATGTTAGCAAGCCACTTATTGACAGGTTCAGGGCAAGCTAACTTTCCTG
CATTGCTTTCTACCTCCTCCTCAATGTCGTCATCTTCTTCTTCTTCTTCTTCTTCTTCATTGGTCTCATCCAATTTAGACAATGGGTTTCTGGAGTTTTG
CCCCTTACATTCAAAATGGTAGGCTGATGGTTCTTTTGGCATTTCATGATCTTCACTATCGAGTTCAAATTGAGTTAAATTCTCATTGCCAGTGTCATGT
TCTATGGAACAGTCTTCTTGTTCTGTTGCTAGCCTCCTTGCCTCCATGCAGACAAACTCTAGCTCACTTGGCTGATCTTCCATCCCAGTAAACTCTCTGC
TCATCATCTCCCCAATCTCTGCAAGGCAGAGACCAAATGCTGTGGCTTCTTTCAGAAATATGGTTGAGAGTATTAGTACTCTGAGGCATGCTTCATGGAT
CATAGGCAACTCCATACGAAGCATGTCAGAATCTTTGATTGGATCAAGACTTGTTATGTACTGGAGCTCATCCTCAGAGAATGGTATTGATGCCTGTGGC
CAGTGGATCCATTCAAAATAAGGATCCTCCAAGCACTCCGGGAGGCAGAGACCATGATCAATAGGAATAAGCTCAGTCTGATTCCCAAATTTTCCTGCTC
CAGTCTGCTTCCTTACCAGAAGATTTCCAGCATGTCTATCAGTATTGAAGATCCTGATGTCAAGAATACCAATCCTATGAACAGCAGAGACAGGAAAACT
TGAAGTGCCGTAGTCACTGGCATCAAAATCATGAGGAATAAACTGCTGAAATGAAGCAATCTTCCTAACAGCTTGTGTACCACCATCAGCAGCATTTTTG
TTAGCACAGTTGACACTTTCATTCATATGAAATACGGGATGAGAAATCTTAACAAGAACTGTCGGAGGGACATTAGCAGAGTTATCATAATCCAGAAGGT
AGGCAGCAACCTCTCTGAAACCAGTCTCGCCGACACGAACTGATCTCTTGAGGCCTGGCTGCCCGAGGGCTTTCCCTATAAAACCTTTTGGATTGTTGGG
TGCAAATGGCTCCTCATCATTTGGCTTCACAATGGCAGCATTCTCACCTTTGCTATTCCTGAAATAGTAGGCACCACCAAGCCCAC
>Locus_12874_Transcript_15/17_Confidence_0.375_Length_4044
TAATCAACTACATGCAAAAAAATCATATATGGCCAATTATACACTCAAGAAAGAAGCCGTATATTAGGTTTAGGATCCGTTGCATTACAACAAAATGAGA
CGACGAGAAGAAGCTGGTACCACTAACTTGACAATCCACAGGCGATGCTACTGATTGACAATACCATGAATAAAGCCCCAAACCAATAGCTCGCACATAC
TTGGCATTCAAGTGCTGCAGCTCACGTCATCATCCTTCTTGTGCTCCTCGCACTCATCTAATTTTAATATGTTACAAAACGCCCACCTTTTTTTCAGAAG
GACCGCGTGCCATTCCATTATAGAAGTAAAACTCAGTTTTTTCCTACAACTCACACAACAGATTGAAAATAAAGCAAGGAAAGGAAAACGAACACCACAC
AATTAGCAGAAGTCAGGCCCTAGCACCTGCATTGAACTCGGATTCCTCACGTCATCAGAATGGTGATCCAGGGATAGACCCTGTCATTGCGCTCTGGCCA
GCAGCTGGGCTGGGCCATTCCACGAGCACCGGCCATGCACCATAAGTCGGCTTCCTCCTTGACTGAACGAAGCACCGTAGCTGTGCTCGGTCTGGCCGCT
TGGAAGACACACTAGTTGAGATGCTTCCAAATGATCCATGCTACTAGCATGACCAGGGATTTGACGCCTTTCCTGATGTCACCTCCAACGCTATCCTCCA
CGTGACGTCACCAATCGACGAAGATCACATCATGCGGTTGCGGGGTGTGCTACTGAAGACCGAGCTCCGCCAGCACCATGTGTCAAACCTCCCTCGTGAA
GATACAGGTAGTCAGGAGCTGCTGCATCGTCTCATCTTTCTGATCACAGAGAGGGCATTTGTCCGGTTGTTGTAACGCGCTATGCTTGAGTAGTACTCCA
TTCCCTCACACAAGATGTTGGCTGTAGGACGACCTGGATGCATGTTAACGGCATCCTTGAACATGGACTCTACAGCCCTTCTGGTGGGTTCGTCGGTACA
CTCGACCTCAATGTGTGCATCTATTCCCTTGAGGGCCGGTAGGTGCTTGATGCCAGCAGGCGCAGCGCCGTACCTCTCCCATTCTTTCTGCACATTAGCA
TAAAGCACCAACCTTTCGAGCCTGGCCATTGCCCCTGCCTTGAAGGTCCAGTACGACATCCTGCTACAGCGAACTTGAAAATGCTTCAGAACAGGGAACC
AGCTTCCACGGATGATGATCTTGTCTTCAGGAGCTCTTAGGATGTTCATGCTGAGTTGGGTAAGGGAGGGCAACTGTGCTAGCTTTCCGACATCCTCCTC
CAACACCTCGTTAATAACAAGCTTCAGGTCACAAAGATCATGGAGTTGGCCGATCCACTCAGGAACCCTTGAGAACCAAGAATTATATACATGGAATCTC
TGCAGATGGCAGAATGAAGCGGGTACTGAACTAAATACATCCAAGCAGCTGCCAGCCATAGGCTTCAAGCATAGATATCTGAGGTTGCACAATTTCTCAA
GGCAAGTGCGCAGAACTTCCCTACCTCTTTCTGCCAACTTATCACTAGGTATAGAATTGCCATAGTGAGGGTGCAAAACAATTTCAAGATCGGTCAAATT
GGTCAGTTCTCCGAGACCCTTGAGATTGTCTGGTTCCATGGCCAAATTAAAGCCGCGTAAGGTATGCAATGATTTCATGTATCCTATCCCATCAGGAAAT
GTTCTCCAACTTGGAACAATCAGACACGATAACCGGCTCATATGAACAATGTCCATTGGAAGTTGCTGATACTGCTGTCCTTCAGCTTGAAGTGATGAAG
ATCTAGTTTCTACTTCATAAAATGTTTCCAACTGTTGCAGACCCCCAATTTTACTAGGCAACAACACTACATGGTAGTAACTCGCTGCAATCCTTAAGTA
TCTCAGTTGAAATAAATGGCATATTGCACTGAAGTCCAGAAACACCAACAACGACTGTGACCCTTCTGGAATTTCAATTGTTAAAACTCGGAGATGCTTG
AACAGTGCAAAAGGAGGTAAATATGAAGAAGTTCCAAATCTTGCAAGCATTCGTATTTGGGATAGCGGGACAGATCCCACTACTCTATCGTCATCTATTG
CACCATCCAATTGGAGGCTGAGTCGACGGATCTTGCCATTCTGTCCTGTCAAATCTTGTACATCATCTATTACAGTGATAAAATTCTCTTCTCTGGACTT
ATGTACGATAAGATCAAGCATCATATCATGTACCTTGCAAGACACCACCTCACCGTTGTAGTCTGTATCTGCGGGTTGAATCATACTCCTATTGATGAGC
TCATTGAAATAGCTCTTTGCAACATGCTCCGGGTCTGTCCCGTGAGCTTTACTTATAAAACCTTCAGCTGTCCATTGCCTAGCCAAATCATTCTTGGCTA
TTGTGTAATCCTCTGGATAAATACCCAAATATAGCATGCAACTCTTTAAATAATGAGGAAGATGTATGTAGCTGAGGTTCAATATTTGTCTCATGCCTTC
CAAGCTCGGGCTCACTTGAAAGTTGGAGCCCAAAGAGTTCCGTATATACTCCCATCGCGCCTTCAGTTTGTTCGGCTGGCTGGCAAGAAGGCTAGATATT
GTGATAATAGCAAGTGGCAAACCACCACACCTTTTCAAAATTTCATCTGAAACTTCTTTCAAATATGGAGGGCAAGCATCCTCTGAATCAAATACTCTTC
TGAAGAATAACCTCCTTGCATCTTGGTTACTAAGTGGCTTCATCTTATAAACATATTCAGGATGATCAGCGCAGCACGCCCTTGCCACTGTCTCAACTCG
TGTAGTTGTAATTACCCTGCTACCATTCGTGTTTTGTGGAAGAGCACATCTGATAATATGCCATGCAGAATTATCCCATATATCATCCACTACAATAAAA
TACAGGCAAACAAGGTTGGAAGACGGAACATCAGAACCTCCCAAATGCTAGCAAGTTGAAGAAGAATAGCTCTTGTCAAAAAGGAATTCTCGCAGTGCAG
TAATCATTTCATACTCATCCCATATTTCCATGTTTGTGTTCCCAGGGGCAACAAAGCCAACTTGAGATAGTATCTTTCTCAGGATCATCCTTGTGTTTGG
TTTCTGGGATACAGGAACTAGTGCTCGGCACTGGAACTTCCCCTCTAGCTTGCGATATATCTCATTTGCAAGCGTAGTTTTTCCAAGGCCCCCGAATCCT
ACAATAGAGAGCACCTTTCTTTCATGTGCAGACACACCCTCTTCATCCATCAACTGAATTAATTCGTCCCTCGGGCCCTCAATACCCACAAGGCCTGATG
TCTCTGCATAAATTGCCAACAAGCGGAGATCAATGGTTGTGTCCTTTGGCTTTGAGACAGCGTCGTCAACCTTGTACCTTGTGCGCCGCTCAGTCACCTC
CTTGACACGGCTCTTGAGGCCTTCAAACTCTTTGGCAATCTCATGCCGAGTATTCATGGTTGTCAGCAAGTTCATGCTCCTGTCGATGAACCCCTTGAAT
CCAGGAGACTTGCTGGGCTGGCATTCCACGCGGAGCATGAACTCATTGATACTGTCCTCAATGTCATAGGACAGCTCACGTACCTCCTTAGCCCAGCACT
TGTCTTTCTCGTCGGGCTCCTCCGTATCTGACATCCTCTTGAGGAATGCATGCATGCTCTCGAGCTCAGCTTTGAGAAACCTGATCTGTCCCTTAGCTTC
TTTGATTAGCTTGTACTTGTCTGTGAGCAAATCACCAAGCTTCCCGAGCAGGGAGGCCATGGCGCCATGTGATGCGGTCACCACTGCAGCCTCCATCAGT
TCTTCTTTCCTTTCTTGTGCTTGCTCTCAATATGACAGTGTACAGTTTTCTTTGGAGTGCATTCTAAGGTCTGTCAAGCAAGTGCTTGGAATAAATGAGG
CCCCGAGCAAAGATTCCTCTCTTCAGTTACTGATATATGAAGTCCTTTTTCTTTCTTTCTTTCCTTTTCCTATTCTTTTCACATGTGTGCTCTCTTATCC
TGAAGTCCTAATAATGGAGAATAGGATGCTCTTTGCTCGATACC
>Locus_3441_Transcript_133/417_Confidence_0.070_Length_2789
CGCATATGGCTGCGCTAGAACTACTGCTCTGAATTAAGAGGCGTTGAAATATTGTCCGGTCTACATATAGGCACTTGAGGTGTATTGACAGACAGTTCCC
CTTAGTTTGCTACAACACATCAGGCGAATTCCACCAAATTAGGATGATGTTTGTTTCTTAGACACAGGATAGCAATGCCAAAAGTTTTTTATAATTTTCC
GCTTCACCGCAGATTTGGCATCTCAAAGGGAGTTGTGGCAAGATTCTCTTGACCAAGCTTTCTTGCAGATTTGGCAAGAAAGATAAAAAAAATGTTGGTA
TACTTAAGCATCCTTAATAGAAACTAAACAGGTCTTAGATTGAAATTATCATGAATAAACCCTTTTTCACTTTAGCTGTAGTCCGGATGAAATCTTAGGG
GCCTTGCTTAGCCTGCCCTGAAGCTTGCCAGTAAGTCGAAGCTGATGCTGACACAGCACTGACATAGATCCCAATGAACACAAGTTATTTCGAGAAGATA
AAATACCAAGCAAAGCCAGGATTGCATTAAAAGATAGTGTATTTCTACTATACGATCTGAAAGCTTAATTTATTATATCAATAATCAAATTGGCAGCAGT
GTGGAACAGATATAAAGAAGATGCTTCAAATCACAAATAACTACAGATAAATTGAAGGGTGAAGGTTCATTCAACCACCAAACACCCAAAATGTCGAGCT
GCATATGCACAGAACCTAAGTACGGTACAGTTCATAGTTTGGGAAAATAGACTGCAAATGTGCACAGATAATACAGATTGTTACTCGATGAACAGTAAGC
ACGGTATTTGAAATCCTTCTAACCATCCATCCAGGCAGTGCCTCGTATACTCAAACCTCTTTTTGGAGGAAGCCAAACCATGTGATCCTTTTCAACCCAA
ATACATATATTCAAGATGAAACTAACAAAAAGACAGTTGATAAAGAAACACGAAAGAGAGTAGAAAAAGGGTACCTGTTTCAAGTTCTCATCTTCTGAAA
ATGATCAGCAGTCAATAGATGTTTTAGTCCATTCTCCAGAAAAATGAGTGCCGCATGAATCCTTTCCGGTTTATAGTGAAACATTTTAGACGATGACCTT
TAACATTCTAGAAAACGAAGAGAAAACCTACAGTCATCTAATTTCAGCTTAAACATGTTCCAGCAAGATCATCACCATCCTGACATGTTCCGTTTTGTCC
CTAGACCTGAGCACAAGGTATTGCGAGGCTAATTTAGCGGGGCAATTGATTTTTTGCCACTATGATACAACTGCAATTACCCGCTGTCACTGACACGTGG
GGTCGTCTGAGTCACTGAAATGTGAGTCCAGTGGCATTTTTGTTTGCCACTCACCCTTCCAGAGTGGCAAAAAGTTAATTGTCCCTAATTTAGCTCAATT
CAGTTGTCGGAGCAATTGTGTGTGAAAAGTGTGAACAACAGCATAGGAGACAATACAGCAAGTGTAGCGTGCCAATATGTGTCCAGCTTGATAGTTCACT
ATAATAAAGTTCTTTAATTCAAGTATTTGAATAATATAGAATCTCAACCTTCAAGTTTAAAAGGTTTTGTATTTGCTATTATAATCGTTGCCACCCCCTC

ACCCCTCTCAAAAAGAGAGTTACTTTGTAGCGCACTGTAAATCTCTTGATTGAAATAGTGGCATGTCATCTTATTTAATTCAGTTATTTCATAAGGTACA
ATTTGTTTAGCTGACAGCTCAGACATGCTTCTATGCAACATGTTTATACCAATGCTGTATTTTGGCAATCCTGGATGTGGCATTACTTAACTGTGCTTTC
ATATTCAATAGTTCTGGCAAAGGGAGGCTGCAAGCTTGAGACAACAACTGCACAACCTGCAAGAAAGTCATCGGTATGGCTAGAAAACCCTGCAAATTTT
ACAAATGTACAAGTCAGAAAATTTGCCTAATGTAAAAGGATCAAACTACAGGATTTGTCAGCATGAGCACAAGTGCCTAACTTCCGAGTTAGATAACTCT
GGAGGAAGGAAATCCTTCCTACTGTTTGTTTCAAGAATATACAAAAATTCAAAACAACATTAGAATAGAACCAAAAGAAGAAACAATATTCACCATGTAG
CAATACTCAATTCCTGGTTGTCTTATTTATAAAGGTATAAAACTAGGTTCTGACGAGACAATTATGAATCAACTACACCATGTTACAGATCATCCATAAC
TCTTGGTGATGCAAATTAAGGGCTGTAAAGTGACAAGTATACCTCCACCAACTAATTTGTGCAGAGTGTATAAAATTGTAAAACAATGGATAAGAATGAA
TTTAGTATACAAGATAGCATACAATGCCCAAGTTTAAGGAGACATACTGATTTACTGACTCCATAGGACAATCTTTTGCTTATGAGGACATCAGTTTTCA
TAAACAATATCAATGTAAAAGGAGGTAACAGTTTATGAATTTCCTATGCCAAGCCATTTATAAGTCAAAAAATGTACCTATTTAGCTTTTATGCATTCCA
ATTAACTTCCAAAGAAGAAGAGTACATACTAATGGAAAACAAATATGATATTATCATCTGTTTTGCCCGGTAGTTTCTTGAGTCTCTGATTTGTCCGCAC
TTCACTCCCAACCAACACAACATTAATACACATGGGCAGAGGAATATCAGGTTATCAGGTAAACTGAATACAGTTTGATTGACTGCAGGCAGTTGATGGG
ACAAGATCTTTCTGGATTGGGCGTCAAGGAACTCCAAAATCTAGAAAATCAGCTAGAAATGAGCCTACATTGCATCCGGACAAAAAAGG
>Locus_5983_Transcript_70/82_Confidence_0.070_Length_914
GTTTCATGGTTTAGCTGAGTTTGACCAGAACAAAGGAAGCTGCCGTAGGTGTCTTACTCATCATAATGCACGAAGGAGGAAACCACAGGCAGATACAATT
TCATTCAGTTTATCACGGCTCTCAACAATGTTTTATGATACAAGCCAGCGGACAAATCTTTTCTGTAATCAGCCTCTTTTTGGCCAAGTGAGAAGCAGTG
CAGTTTCTTCATGGAATAACTTAGGAGGCTTCAGATCTATGGAAACGAAACATCTGTTGATGCAGCCATCGAAAACTGTAGGCCTTGATGAGCTGCATTT
CTCAACCCCCCAGATATCAAATAGTGCTGTGGCTCATTCTGTACATCATCGTAATTTCGATGGGCTCATGCCATTCAAGGGAACCAACACAAAGGTCCTC
AACCAAGGCGTGGAAGCTTCTGCGATCGCTTCCAACTCGAGTGGAGACCCGGATCTTGGGTGTGCTCTCTCTCTTCTGTCAATCGGTTCGTGGGTTTCAA
GTTCAACCGTCATCCAGCAGCCTAGTTCTCATGTGCACGCTGGTGTGGTGCCGCCCCTCGCCACCGTTGCCGTCTCCAACCCTGCAATGCATCCTCTGGA
CCCATCCCTGGGAGAATTCTGGCAAGACGACTCTCCCTCGCTCGATGAAACTCCACAGATTCAGGCATTCACGCACCTCTGATCAAATGCTGCGTACCCT
GGGAACAGTTGGTTAGACCTCTCGTAATCGTCCTAGGTTTGCTTTGCCAGTGACTGGTTCTGCACAATGTTGACCCATCGCCGGAGGGATTTGGTAAGGT
GAAGACTAAATATTGAAGAATAATAGGCCCCGTTCTCTTCCAGGTTCAGGCTGTTTGGGTGCAGCGGTGTTGCTACCTGATCCTGTTTTCGGTTCTGCCA
TTAGAGAGCTGATT
>Locus_8503_Transcript_68/83_Confidence_0.531_Length_5297
CCTCGGCGCAGCTCGTCGCGTCACTCCTCGACGCCGCCTGCAACGCGCTCCACCTCGACGACGAGCACATTGAGCTGCTTCTCCTCAAGACGCTCCTCTC
GGCGGTCACCTCCACGTCAGTGCGCCTCCACGGCGACTGCCTGCTCCGCGCCGTCCGCGCGTGCTATGACATGTACCTGAGCAGCCGCAGTGCCGTCAAC
CAGGCCACTGCCAAGGCGTCGCTCGTGCAGATGCTAGTGATCGTGTTCCGCCGCATGGAGGCTGACTCGTCCACGGTGCCCGTACAGCCCATAGTCGTGG
CTGACGTGATTGAGCTGCCTGAAGCTGGTTCTGGTTCCTCGCCAACTGCTGACCCCAACGTTGTACAGGGATTCATTTCGAAGATCATTGGGGACTTTGA
CGGTGCACTCACACCTCTGGCACGAACAACTTCAGCAGCAGGGGCCACCGTGGCTCATGACGGTGCGTTTGAGACTACAGCAGCAGCAGAGGAAGGGGCG
AACCCAGCAGATTTGCTTGATTCGACGGATAAGGACATGCTAGATGCCAAGTACTGGGAGATCAGCATGTACAGAACAGCTATTGAGGGTCGCAAGGACG
AGCTTGGTGTGGAGGGGGCAGTGGTGGGCACTTTGGAGGATGATGCTGATGTGAGGATCGGGAATAAGTTGCGAAGGGATGCTTTCCTAGTTTTCCGGGC
ATTGTGTAAGCTTTCCATGAAGACACCACCCAAGGATGCACCAGCAGATCCTATAGTGATGCGGGGGAAGATCCTTGCTCTCGAGCTTCTCAAGATCTTG
CTTGAAAATGCTGGAGCTGTGTTCCGGACCAGCGAGAGGTTTCTCGGTGCTATCAAGCAGTATTTATGCCTGTCACTATTGAAGAACTGTGCCTCATCAC
ATGTGATTGTTTTTCAGTTATCTTGTTCTATTTTTATTAGCTTGGTGTCAAGATTTAGACCAGGATTGAAGGCAGAGATTGGAGTGTTCTTTCCCATGAT
CATTTTGAGAGTTTTGGAAAATATCGCTCAGCCAAATTTTCAGGCAAAGATGATCGTTCTTCGTTTTTTGGAGAAGCTCTGTGACGACTCTCAAATCTTG
GTCGATGTTTTTCTTAATTATGACTGTGATGTCCACTCATCAAACATATTTGAGAGGATGGTGAATGGTCTACTAAAGACAGCACAAGGACCTCCTGCTG
GTGTCCCTACCACATTGGTACCGCCTCAGGATACTGCAATGAAAAGCGAAGCAATGAAATGCTTAGTTGCTATTCTCAGATCCATGGGAGATTGGATGAA
TAGGCAGCTGCGCATTCCAGGTCCTGATTCTCCTAAAGTAGAGTCAGAGAAAAACGATAATGATGGTGGAATCAAGCCTCCCCAGACGGATAACAACGGA
GATGAGTCTAGTGAAGCATCTGATTCACATTCTGAACTATCGAATGGGATTTCAGAGGCTGCATCTTTGGAGCAGCGTCGAGCTTACAAGATGGAGCTTC
AGGAGGGCATCTCTTTGTTTAATCAGAAGCCTAAGAAGGGAATTGAGTTCCTAGTAAATGCCAGCAAGGTGGGGGAGTCACCCGAGGAAATAACTTCTTT
CCTAAAAAGTGCTTCTGGCTTGAACAAGACTATGATTGGTGATTATTTAGGGGAAAGGGAAGATTTATCTCTCAAAGTCATGCATGCATACGTGGACTCA
TTTGATTTTCAAAGCATGGAGTTTGATGAAGCAATTAGAGCTTTCCTTCAAGGCTTCAGGTTGCCTGGAGAAGCTCAAAAGATTGATCGGATCATGGAAA
AGTTTGCAGAACGTTACTGCAAATGTAACCCAAAGGCCTTTTCCAGTGCAGATACAGCTTACGTCCTTGCTTATTCTGTCATAATGCTTAACACTGATGC
TCATAACCCAATGGTCAAAAACAAGATGTCACCAGAAGATTTCATAAGAAACAATCGTGGTATTGATGATGGGAAAGACCTGCCTGAAGAATTTATGAGA
TCCTTGTATGATAGGATTTGGAAAAAGGAGATTAAGATGAAAGAAGATGAATTTGTTCCCCATCAGCAACAATCAACAAGTTCTAACAAAATTCTTGGGT
TGGATAACATTCTTAACATTGTCATACGCAAGCGAGGTTCATCAATGGAGACGAGTGATGACCTCATTAAACATATGCAGGAGCAATTCAAAGAAAAGGC
CCGCATGTCTGAGTCAGTATTTTATCCTGCCACAGACGTAGTTGTTTTGAAGTTCATGGTCGAGGTTTGCTGGGCTCCTATGCTTGCTGCCTTCAGTGTT
CCACTTGACCAGAGTGATGATGAGATTGTCATATCCCAGTGCCTCGAAGGATTTCGCAGTGCAATCCATGTTACTGCAGCCATGTCAATGAAGACTCAAA
GGGATGCATTTATCACTTCGCTTGCCAAGTTTACATCACTTCACTCTGCTGCCGATATCAAGCAGAAAAATATTGAAGCCATTAAGGCAATTCTGTTAAT
TGCAGATGAAGATGGAAATTACTTGCAAGAGGCATGGGAGCATATATTGACTTGCGTTTCTCGTTTTGAAAATCTACATCTTGTTGGAGAAGGAGCGCCT
CCAGATGCCACTTTCTTTGCACTGCAACAGCCAGACCTTGATAAATCAAAACAGACTAAGTCATCGATTCTTCCTGTGTTGAAAAAGAAAGCTCCTAATG
CTGGTTCAGCTTCTAAAAGAGGTTCCTATGACAGTGCTGGTGTGGGTGGTAAAGCTTCTGGTGTTGATCAAATGAATAACGAGGTGACAAACCTCTTGGA
GCAAGTTGGGATGGCTGAAATGAACCGTGTATTTGTAAGAAGTCAAAAGCTCAACAGCGAGGGCATAATTGATTTTGTGAAGGCTCTCTGCAAGGTTTCA
ATGGAGGAATTACGTTCTGCATCTGATCCACGAGTTTTTAGTCTGACAAAGATTGTTGAGATAGCGCATTATAATATGAACCGCATCAGGCTTGTCTGGT
CAAGCATATGGCATGTCTTGTCTGAATTTTTTGTCACCATTGGCTGCTCAGAAAATCTTTCAATTGCAATATTTGCTATGGACTCATTGCGGCAGTTGGC
AATGAAGTTCCTGGAGCGAGAAGAGCTGGCTAACTACAATTTCCAAAATGAATTTATGAAGCCTTTTGTTGTTGTCATGAGAAAGAGCCGAGCTGTTGAG
ATCCGAGAGCTAATCATTAGGTGTGTCTCTCAAATGGTCTTGGCCCGTGTTAGTCATGTGAAGTCAGGGTGGAAGAGCATGTTTATGGTCTTTGCGACGG
CATCGTACGATGATCACAAAAATATTGTTCTATTGGCCTTTGAAATTATAGAGAAGATTTTGCGGGAGTACTTTCCCTACATTACTGAGACTGAATCAAC
TACTTTCAATGATTGTGTAAACTGTCTTATTGCATTCACTAACAGTAGGTTTAACAAGGATATAAGCCTTAATGCAATTGGCTTTCTTCGATTCTGTGCG
GCAAAGTTGGCCGAAGGGGACATTGGATCCTCAAGGTTGAAGGAGAATCCCTCTTCAAACAGTAATCCACCCTCACCTCATTTGACCAATGATGGAAAGC
AGGAAGGTGCAGTTCTTGCCGATAAGGATGACCATATACACTTTTGGTTTCCTTTACTAGCAGGGTTGTCTGAACTTACCTTCGACCTAAGACCTGAAAT
CAGAAAAAGTGCTTTGCAGGTGTTATTTGACACATTAAGAAATCATGGTCATCTTTTCTCTCTGCCTTTATGGGAGAAGGTGTTTGATTCAGTGCTTTTC
CCAATATTTGATTATGTACGTCATGCTATTGATCCATCTGGTAGTTCACAAGGGCAAAGTGTGGAAAATGATCCTGCAGAATTTGATCAAGATGCCTGGC
TATACGAGACATGCACTTTGGCCCTTCAGCTAGTTGTAGATCTGTTTGTTAAGTTCTATGACACGGTGAATCCACTTCTAAAGAAGGTTCTCTCGCTCTT
GACGAGTTTCATAAAGCGTCCTCATCAGAGTCTTGCTGGTATAGGTATTGCTGCATTTGTTCGTTTGATGAGCAGTGCAGGATCTGTGTTTGTGGACGAA
AAATGGTTAGAGGTTGTATTGTCCTTGAAAGAAGCTGCCACCGAGACACTTCCCGATTTCTCTTATGTTGCATCTGGAGCTTACCTGGAAAATGTTCCAA
CGGAAAATGAAGGCTCTTTGGATAGGAGAGAAGATGAACCTCGACCATCAGAAGATGACAATGATGAAACCTCTAGATCAAGGAACCTATATTTCGCAAT
TGGCGATGCCAAGTGTCGAGCTGCTGTGCAACTCCTGCTGATTCAGGCTGTGATGGAGGTATATAACATGTACAGAGCTCAGTTGTCAGCACAGAACACA
GTAATCCTCTTTGAGGCCTTGCACACTGTAGCCACTCATGCGCACAAAATAAACAGTGATAATGACCTGCGATCCAAGTTGCAGGAGTTGGGCTCGATGA
CCCAAATGCAGGACCCACCATTGTTACGCCTTGAGAACGAATCATACCAGCTGTGCCTCACTATCCTCCAGAACATATTCTTGGACAGATCTTCTGACGA
AGGGAGCATAGAGGTGGTGGAGAGCCACCTTATTGGCTTGTGCAAGGAGGTCCTTGAGGTATACTTGAGCACAGCAAGACCTGCTCAGCTTTCCAGTGGC
ATGCAGCCGCTTGGCCATTGGCTTATTCCCGTCGGTTCATCGAAACGGAGAGAGCTTGCAGCTCGCGCACCCCTTGTTGTCTCGACTCTGCAAGCTATCA
GTGGCCTAGGTGATTCGCCTTTCGAGAAGAACCTTGGCCAGTTCTTCCCGCTCCTAGCTGGCCTCATAAGCTGCGAACATGGTTCCAGTGAAGTGCAGGT
GGCCTTGAGTGATATGTTTAGCACTTGGGTTGGCCCGCTCGTGCTTCAGTCCTGCTGATGCACTGAGCACCATGTGATATATAGTATACCTCCAGATGTA
AGTTATTCTTTGTTTCAGTGAAATCCCATTATGTTCCTCCGTTCCCCCATTTTGTTATGTTCATCATTAGTTGCTGGGGGAGTTTATATTGTGCTGGACT
GATCTTTTTTCCCCGTTTTTACATACTTTCTCGTTCTCATTGGGAGGAGTGTATGTTACTATAGAGAATTATGTACCGTAGTTTAGTTAGAATTGTGCCC
TTTTTGTTGGCCACGTACTGAATCAGATCCATCCCATTGTGACAATTCCATTCTTCTATTGAGGTGCTGCTTCCTGGCTTCTGGTAGCGCCGAAACT
>Locus_839_Transcript_89/261_Confidence_0.061_Length_1057

GAGAGGCTTTGCAGAAGAGAGAAGCAAGGTGGGAAAGAGGCAGCAAGGGATTCAGGAGGGCTACAGGAAGGGGAACATGCTCCCTTCTCATCTCACAGTA
TGAACTGAAAAACAGGAATAGAGAATTTCCGTGAGATGAGACAAAATGGCACCATAATGATTCAATTTGGTCATCAAATGCCTGATTACGACTCATCAGC
TACCCAATCGACCAGCGAGAGTCATCAAGAAGCGTCTGGAATGAGTGAAGGAAGCTTCAATGAGCATAATGATCGATCAGGTAATCTTGATGGTTACACG
AAGAGAGATGAAAATAAGATGATATCAGCTTTATCTCTGGGCAATCCGGAAACTGCTTATGCACATCCAAAGCCTGACCGTAGCCAGTCCTTTGCCATAT
CATACCCGTATGCTGATTCATTCTATGGTAGTGCAGTGGCGGCTTATGGCTCGCATGCTATTATGAATCCCCAGATTGTGGGCATGATGTCATCTTCTCG
AGTGCCATTACCAATTGAACCAGCTGCTGAAGAGCCCATTTATGTAAATGCAAAGCAATACCATGCGATTCTCCGAAGGAGACAACTCCGTGCAAAGTTA
GAGGCCGAAAACAAGCTGGTGAAGAGTCGCAAGCCATACCTTCACGAGTCTCGGCATCAGCATGCCATGAAGAGAGCTCGGGGAACAGGTGGGCGGTTTC
TGAACACGAAGCAGCAGTCAGAGGCTGCTGGCGGCGGCACCTCGGACGCGCAACGCATACCAGCAAATGGTGGCCTGTTCAGCTTACCTCCCAGTGATCT
CCACTATCGCGCGAGAGGGGGCGCTTAAGGAGTCCCATGGCAACTCATCCTTGGCTTAAAAAATGTGTGGCGCTCAGCAACTCGACCTCTGCTAGTTCCA
TGTAAAGGTGTGTGACTGTAAATGCTGGTGCTTGAATTGGCAGCAGCCATTCCTGCAACGCGTCGCAGCTGTTCCCGGTTCTGTGATCATGCTAGACTTG
TGACCGACCGGCACGAAACTGGCACCTGTTTCCTGTAACTGTTTTCCAAGTTGATGA
>comp73370_c0_seq7
GGGCGTCACCTTCTACAGGAGGACCGGCCGCTGGGAGTCCCACATCTGGGATTGCGGGAAGCAGGTCTACCTGGGTGGATTTGATACGGCGCACGCCGCG
GCAAGGGCCTACGATCGCGCTGCTATCAAGTTCAGGGGCCTCGACGCGGACATCAACTTCCATTTGAAGGACTATGAGGACGACCTGAATCAGACGAGGA
ATTGGACCAAAGAGGAGTTTGTTCACATACTCCGACGCCAAAGCACTGGGTTTACAAGGGGGAGCTCCATGTACCGCGGTGTGACACTGCACAAGTGCGG
CCGGTGGGAAGCTCGGATGGGTCAGCTTCTTGGAAAGAAGTACATCTACCTTGGGTTGTTTGACAGTGAAGTCGAAGCAGCAAGAGCATATGACAGGGCA
ACCATTCGCTTCAATGGAAGGGATGCTGTTACTAATTTTGATCCTAGTTCCTATGATGGAGATGTTCCACCTGAAATTGAGAAAGATGTGGTTGATGTTG
ATGGGGACATCCTCGATTTGAATTTGAGGATCTCACAACCTAACATGCATGATTCCAAAAGTGATGGCATCCTCACTGGGTTCGGATTAAACTGTGATTC
CCCTGAAGCTTCAAGTTCGGTTGTTTCTCAGCCAATAAGCCCTCAGTGGCCTGTACATCACCAGAGCACATCGACTGCACCCCAGCATCCACATTTGTAT
GCATCTCCTTGTCCGGGCTTCTCTGTGAACCTCAGGGAAGTGCCTATGGAGAAACGACCCGAGTTGTGTCCCCAGTCGTTCCCTCCGTGTGCATGGCAAA
AGCAGGGTTCCCCTGCGCCATCACTCCCTACTGCAGCATCATCAGGATTCTCTA
>comp79438_c0_seq8
GAGGATCATCCAGTCCATTCCATGTCGAAGTCCAACAATGTAAGTTGTTACTATTTATGTTGGAAACCAGGCATGTTGCCACTTTGCTACATCACAAATA
ATAAACTTATTTGCAAGGCTACATATATAGGGCTTCTTGTCAGGAAATGGCTATCAGATGAAGCAATTTGGTCATAAAATCCATGATAGGGACTCGTCAT
CAGAGTCTGGTCGGTCTCACCAAGAAGCATCCGCAGTGAGTGAGAACAGTATAAATGAACAGCACACCTCAACACAATCTGACAATGATGATGGTCATGG
GAAGCATAATCAGGACACAATGAAGTCACTAGTGTCCTTGGGGGAACCAGGATCAGCCTTTTTGGCCCCAAAACTAGATTACAACCCATCTTTTGCTTGT
ATTCCTTATACTGCTGATGCTTATTATGGTGGGGTCTTGACAGGATATGCTCCACATGCCATTGCTCATCCCCAGCAAAATGATACTGCAAACTCTCGGG
TTCCATTGCCTGTTCAACCTGTAGCAGAAGAGCCAATATATGTTAACGCAAAGCAATACCGTGCTATCCTTAGGAGGAGGCAGATACGTGCTAAACAGGA
GGCTGAGAATAAGCTGGTGAAAGGTCGGAAGCCATACCTCCATGAATCTCGACACCGCCACGCAATGAAGCGAGCCCGTGGATCAGGGGGGCGGTTCCTC
AACTCAAAGCAGCTCCAGGAGCAGAACCAGCAGCAGCAGGCATCAGGTGGTTCAAGCTGCACAAAGGCCATCGGCAATAACACAAGCTCCCAAAGTGGTC
CCAGTCACACGCCTCTGACACTTGCTTCCTCTGACACTGCAAGCGCTTCGAGGGCCAACCAGGAGCGCACCTGCTTCCCCTCGGCTGGCTTCCGTCCCAC
AATGAACTTCGGCGCGCGAGGTGGAGACGCAAAGCTAGCTGTGAATGGCATACAGCAGCATGTTTCCTTCATCAGGTGAAGCAAGTGAAATGGGGCGCTG
CCTGCGGTAACTCATCCTTGGCTTATGAAACTGTGAATGTGTAATGGACATGTAACTTCAGTCTCAGAATAACCAAACATATGGGAGTCCTCCTGAAGTT
TCCTGTAGTTCAAAGAACTGATAGGTTATGGCCTTATAGGTTTGCTTCTGGAGACTGAAATCCGTGCTTGATATACTATCTTGTGCGAGTTCTGAGGTGA
AAGACCTTGAAACTTGCTCTTGTGGTTCAGTACCTGTTGCTTTTGTGTCTGCAAGGTTAGCACTGCAATTTGCAATGTCGGATTTCTGTTATGCCACTAA
TTTTT
>Locus_20324_Transcript_8/30_Confidence_0.328_Length_2043
CCTCCATTTGTTGATGGGGTTTTTTTACTGCGGGTACCGGAGCTCGCAGTACAGGGGCGTCACCTTCTACAGGAGGACCGGCCGCTGGGAGTCGCACAT
CTGGGACTGCGGGAAGCAGGTCTACCTGGGTGGATTTGATACAGCGCACGCTGCGGCAAGGGCCTATGATCGCGCTGCGATCAAGTTCAGGGGCCTTGAT
GCGGACATCAACTTCCAGTTGAAGGACTATGAGGACGACTGGAAGCAGATGAGGAAATGGTCCAAGGAGGAGTTTGTTCACATACTCCGACGTCAAAGCA
CTGGGTTTACAAGGGGGAGCTCCATGTACCGCGGTGTGACACTGCACAAGTGCGGCCGGTGGGAAGCTCGGATGGGTCAGCTTCTTGGAAAGAAGTACAT
CTACCTTGGGTTGTTTGACAGTGAAGTTGAAGCTGCAAGAGCATATGACAGGGCAACCATTCGCTTCAATGGAAGGGATGCTGTTACTAATTTTGATCCT
AGTTCCTATGATGGAGATGTTCCACCTGAAATTGAGAAAGAAGGGGTTGATGGGGACATCCTTGATTTAAATTTGAGGATCTCGCAACCTAACATGCATG
ATTCGAAAAGTGATGGCATCCTGACTGGGTTCGGATTAAGTTGTGATTTTCCTGAAGCTTCAAGTTCTGTTGTTTCTCAGCCAATAAGCCCTCAGTGGTC
TGTACATCCCCAGAGCACAACTACTCCACCCCAGCATCAACATTTGTATGCATTTCCTTGTCCGGGCATCTTTGTGAACCTCAGGGTATCTCCAACATCG
CTATTTTTTATTCTGTCACAAGTGTGCTTATAATTACGATTAAATTCAGAATAGATGATGTTTCGAACACACATTTTGAATGTTGTGAAAATTATGTAGA
GCTTTGCTTAACCAATACGTATATTTCACCAGTAACTATGATTTTGCACCATGGCCTGATATGTTTATGTTCTTTCCTGTGATACTTAGATTTTGTCCTA
GTGTGCACATATTTCTTCTTGAGATAAAGATGCCATGTATTTTTTTCCTGCAAATAGTATATATAAGTAGTTGCCGATACTGTTTGCCTTGCGAACTGTG
GTAGTTCAATCAAATGAATCTGTCATCCTGCAACCCTCTATGAGCTATAGCTGTCAAACTTTGCAGATGATCACACAGCGTTTGACATAGTAGTACTTTC
TTGTTACATTCTGCAGCCTTAGTTCTGAAGATGCTGTCGATCTACATGAATGCTCCACCTATATCTGCTCAAACCTGCACTGCTATTACCTGCTCAGTCG
TGCATTACCATCTTTCTTCTTTATGTTATGGTATTGCACAAGCTCTGTTTATCTAAGGGACTTGCATTTTTATATGTAATTTGAACTTAGGCCAATGATT
AGAGATAATAAAAATCTTCAATAGTAAATAGTGTAGAATATATGGTTACCTGGCGGAGGTGCACTTGAATCATTACTGTGGTCAAAAGTCAAGACTAAGA
CTGCTTGAACTGTGGCTACTGCAGGAAGTGCCTATGGAGAAACGACCCGAGTTGTGTCCCCAGTCGTTCCCTCCGTGTGCATGGCAAAAGCAGGGTTCCC
CTGCGCCATCACTCCCTACTGCAGCATCATCAGGATTCTCTACTGCCACCGGCGGCGCGGACCCGGCCAGCGTGCCGCCGTCCCGCCCCCGGCCCTTCGC
CACCGGCCACCAGCTCCACTTCCCCCCAACCGTCTGACAGCCTCCCCCTGTTCAACTGGCGACGATAACGCGGTCGTCTGGGCGCCCCATGCTTCGCTGC
TGGTCGGCAACAGTCGCAATTGGTTCGGGTAGAGAGAGATGGAGGCTCCAGCACACGACCTCTCTGTCTCTCTCCCCACCTGGTAATTATCATCAGAACC
ACCATGCTTATGATTCTTTTATCTTCATTTTGAGGGCGGATCCACGGATCATGAGCGTGTACCTCCAAAATTCTAAAATAAACCATTTACGATAACTCTG
GCTGAGGCTCTGTTTGTTTGGGCTTCTGGCCAACTTTGAACGG
>Locus_17579_Transcript_39/92_Confidence_0.212_Length_1794
CTTTTTCTGGGAGCAACGTAGTGGGAGGTAGCTGCTTTTGCATACAATTAGGAGCAGCAGTTGAGATATTCAATTCGGGCATGGGTTCTTTTGGGATGGA
CTGGAACCAGAAAGGCTCGGTGTTGTGGGATTGGGAGAATTTGCCGCCGATAGGCAGAAATGCAAACGAGAACCTCAAGATTGCGCCACAGGCTGAACCG
AAGTTTGCAGGTGTTGAGGCAACAAGGCATGAATCGGGGCATTCTTCTTGTGGTACTTTCTCTTCCAGCTCGGAGATGGGGTATGGTTCATCCAAGAGTT
CCATATCAGCGTCGATTGATTCTTCACCCAAGGTGGGAAACAACGTGGAGCTCAATCTTGCAGCTGTCAAAGTGCCTGACAAAAACACCGTCAAGAACAC
TGATTTGGGTAAAGTTGATGACGCTGGAACTTCTCCATCATCGATGATAGCCGTCAGCAGTGGAGAACCGGTGATTGGCCTGAAACTTGGCCAAAGAACC
TATTTTGAAGATGCATGCGGAGGGCAAAGTGTCAAGAGTTCACCATCCAATGTGAGTGCAGTGACCCCTGCTTCTGCGAAGAAAGCAAAGATGATTCAAA
ACGCACAGAACTCGTACTGTCAGGTTGAAGGTTGCAAGTTTGATCTCTCTTCTGCTAAAGATTATCATCGGAAGCACCGAGTCTGTGAAACTCATTCTAA
GGCTCCCAAGGTTATTGTTGCTGGTCTGGAGCGACGCTTTTGTCAGCAGTGTAGCCGGTTCCATGCTTTAACTGAGTTCGACCAGAAAAAACGAAGCTGC
CGGAGACGCCTCAATGATCACAATGCCCGCAGACGGAAGCCACAGCCTGAAGCAATTCCTTTCGGTTCATCAAGGCTCTCTGCAATGTTTTATGATGCAA
GGCAACAGACAAGTCTTCTGTTTGGTCAAGCTCCTTATGGTCAAATGAGAAGCTGTGCAAACTCTTCATGGGATAGCCCAGTAGGAGGCTTCAAATTTGG
AGAAACAAAAGCTCCTTGGTTAAAGCCAACGAGAGCTGCAGGTGTTGATGGGATGCATGTATCAAGCCAGCAGGTGTGGAACAATATTACGCCACACGGT
GCACATCATGATTTTAATGGTATCATGGCTTTCAAGGGAACCAGTGCAAATGTCCTTAATCAAGGTGCCGAAGCTTCTCCGGTCGTCTCCAACTCGAATG
GAGCCCCAGATCTTCAGCGTGCTCTCTCTCTTCTGTCAAACAATTCAGCTGGTGCTGCCAACGACCAGCCAACTCCTCAGCTGCACCCTGGGCTGACCAC
CTTCGTCGGCTCCTCCAACCCTGTCACTGTGATGGAAGCCTCACCACCAGGACTCTGGCAAGACGGCACGCCCCTCGATCATCAGGCGCGGTTTCAGTTT
TTTGATCCCCTGGGCGACGGCAGTGCCATTGCCACACCGCATCAGCTCCAGCTCCTAAAACCCTTCGCCTACGACAGCTCCTCGTCCCACTATGACCAGA
TGCACTGATGCTTCCTTCAGTTAGCGCAAACCGCGGTTCAGTTGCGCGTAATCAGACATGGTTTCCACTGTCTGAAACTCTGAATTTTGAAACTGCGATG
TTCTGAAAAATAAAAGCCTGCGTGCCGCAAACTCAGGTTCGAGTTGATCTAGTAGTGTTCCCGTTCAGGGGCTTGAATAATCCATCCCTGTGATGGCGTG
TAACACAAATGCTTCGATTGCGCCGGCAATTGATCATTGACTCTTTTCGTGTTCTTGATAACATCTCAATTACACCTTGAATGGAACACTGGTT
>Locus_4023_Transcript_12/33_Confidence_0.362_Length_2182

AAACCCAGCGGTTCTCCTCGGTTCCTCTCTGCCCTCTCTGGCCGCGGCCTCGCAATCCAATCCATTCCGGCCGCTTCAAATTCTCCAGAAATCCCCAAAT
CCAAATTTCATCCCTTCCCAATCCCTCCTTCCATATGCCTCGATCTCCGAATCTGTTCCCCATGCTTCTCCAGCGAGTTCACACCGCAAGAACTGCTCAT
GCTGCTTGAGAAAAACCCCAACATCCTCCAAGATCCCGGCCGATCGCGTCCGGCGCTGCGGAGACCCGTGAAATCTCTTTAGGTTCGTGCTAAAGATTCG
ATTTTGTTCGGGCCCTTTCGACCCTACGCAGATCCGTACCGTCTTCCGGCTGATTCCGCCATTCCATGGGTAGCAGGTTCGTTCTTGCTGTTCGTGGATT
GGTGTCTCCTTCCATGGGGTGGTTTTGCAGGCGTATAAGTGTGTATTCCATCTGATTGATCGGTGGAGAGCAAGCAACTTCTGCGATTGCTCGAGCATGG
CGGGCGATGAGGCTGCGGAGGGCAGCCGAAGCAGGAGGCGGACGGATCTAAACCTGTACCTCGGTCTCCCTCCGTTGCCACGGCCTCCTGGCCGGCTCGA
CGCCGCGCTGGATTGCCCGGTGTTGATACCGAACTCGTCCAGTCCTGCGCCGGGAGCCCCAAGAGCGGATGAGCCTGAGGAATTGCTCACCTCGGCGGTG
GCCTACTCGCCGTCGAATGCGCTCTCCACCCCGGAGGAGCAATCGATGCTGGATCCCATAGTTTATGCGTGGCTCGATGGCCACAGCACCGATGGTGAAG
ATGGCACTGATGCTGTTGACCCGGCTGTGGTGCTTGGTGCACCGGTGGACGGTGCCAATGTCTCGCCGCCGCTGGCTGCTGGATTGGGAGGGGATGATCT
TCCACCGTGGGTGGAAAGGTTTGTACACCCTCGTCGAGTGGGTGGTGGAGTGGAGATGGTCAGCACAACCAGCATACTTAGTCGGTCCATTCAAGGGGCT
GTTGGAATTGAAGCAAGGACTCCGGAGCTCCGCTTTCAGAGGTTGATCCAGATTAGCCAGCATCATAACATTCTGAGGTCAGGGTCGGAGAACCGCAGCC
AACGGGCTAGTAGTCCCGAAGCGGATAGGCTGGTTTGGGCTATTCAACGTTCCCATAACTCTCTAGACTCAGCAAGGCGGCAAAAGCTGGATGGCGATGA
CAAGGTGGGAGGGAAGGGTGCTGGTAAGAAGGATGGGTGCTGTGGATGCAACTCCAATTTTGAGTGCAATATCTGCCTTGATGCAGCTAAAGAGCCTGTG
GTTACACCGTGCGGTCACCTCTTCTGTTGGCCATGCTTGTACCAGTGGCTTCATGCGCATTCAGCGCTCTCTGAGTGTCCTGTCTGCAAGGGCGGGGTGC
TCAAAATGAATGTCACTCCGATTTATGGAAGAGGTGACGATGAACGGGATTCCTCCATCCCTGATATGCCTCCCAGGCCACGCGCAAACAGAATGGAGAG
CCTGAGACAGCAACTGCAAATGGCAGGCACTAGAGAAATTGCAACAGTAGTGAGACAGTTGATAGAAAACCAGGGTATAGTGAGAGGCCAAGCAAACCCA
GCTGTAGGGGTGGAGGTGCCTCTGGTTCCTGCAAGCCGGTCAAGGGCTAGGGCTCGAAGACAACAGAGGCAAGATTCCTTATCTGCAATAATGCTGAATA
TGGGCAATGTTGCCCCTGAGAGCAGCAATCAGACCCCATCGCCGCCTTCGAATGCAGACAATACTGCGCCAGCGGTTCCTGCAGCTCCTCAGCAGTCATC
ATCTGTGGAGCAACCGTCGACTTCTAGCACTGCAGCTGTTATAGTGGGGGAGCCAGGGTCAAGTAGGAGGTCAAGGCCTTCGGAATCCCCAACGACAAGA
AGAACCAGGAGGAGGCAGCAGGGGTTATCATAAGTGCTGCCATTCTCTCCAATAAAAACCTATCGACCATGCCATGTTCCCAGTTGTTTACCTTGAATAG
GTCCAAAAACCTTCAAAGTTTAGAAGGTAGGAATGTATATGCCATCTTCCTCTCTCTTACATACCATGTATGCACCATCTTCATGGTGAAGGAATGGATA
TGTCAGTTTATCCATAAATCAGAGCGAAGGAATGTTGTTCCGTGTATGCTTGAATGAAATGTTCAGTTTATCTGTTTCTGGT
>Locus_17579_Transcript_81/92_Confidence_0.274_Length_2046
GAGGAATCTCTTGTGGATTCCTTTTTCTGGGTGCAACGAAGTGGGAGGTACTTGGTCTTCGCACCTTGATGTACTTGTGGGTGCGTGTGTGCGCGCGCGC
GCGCGCCTTTCTTGATGCTAGAATGATGGTGGTGCTGTTCTTGCCATGATTGACGATTCCCCTAGAAAAAGTTGAACTACTCTGCACTCTTTCTAGCACA
TGGATAGCACCTTGTGTACTTCTCTGATGATGTGTAGTTGGGTGCTCAGATATGTTGTTGCAAATCTGTCCAATTTCTGCTACTCTCATATGTCAAATCT
TTTCTGTTTTTACTCTGTTTCACTCTTCCACTTTGTTGCTTCAAAGGTAGGAGCAGCAGTTGAGATTTTCAATTCTGGCATGGGTTCTTTTGGGATGGAC
TGGAACCAGAAGGGCTCCGTGTTGTGGGATTGGGAGAATTTGCCTCCGATAGGCGGAAATGCATACGAGAACCTCAAGGTTGCACCGCAGGCTGAACCGA
AGGTTGCAGGTGTTGAGGGCACAAGGCATGAATCAGTGCATTCTTCCTGTGGTACTTTCTCTTCCAGCTCGGAGATGGGGTATGGTTCATCCAAGACTTC
CATGTCCGCGTCAACTGATTCTTCGCCCAAGGTGGGAAACAACATGGAGCTCAATTTTGCAGCTGTCAAAGTGCCTGACAAGAACACCGGCAAGAATACT
GATTTGGGTAAAGTTGATGACGCCGGAACTTCTCCGTCATCCGTGATAGCCGGCAGCAGTGGAGAACCGGTGATTGGCTTGAAACTTGGCAAGAGAACCT
ATTTTGAAGATGTATGTGGAGGGCAGAATGCCAAGAGTTCACCGTCAGATTCGACTACAGTGACTCCTGCTGCTTCTGCGAAGAAGGCAAAGATGGCTCA
AAACACACAGAACTCATACTGTCAGGTTGAAGGTTGCAAGGTTGATCTCTCTTCTGCTAAAGATTACCATCAGAAGCACAGAGTCTGTGAAGCTCATTCT
AAGGCTCCCAAGGTTATGGTTGCTGGTCTGGAGCGACGCTTTTGTCAACAGTGTAGCCGGTTTCATGCTTTAACCGAGTTCGATCAGAAAAAACGAAGCT
GCCGGAGGCGCCTCAATGATCACAATACCCGCAGACGGAAGCCACAGCCTGAAGCAATTCCTTTCGGTTCATCAAGGCTCTCTGCAATGTTTTATGATGC
AAGGCAACAGACAAGTCTTCTGTTTGGTCAAGCTCCTTATGGTCAAATGAGAAGCTGTGCAAACTCTTCATGGGATAGCCCAGTAGGAGGCTTCAAATTT
GGAGAAACAAAAGCTCCTTGGTTAAAGCCAACGAGAGCTGCAGGTGTTGATGGGATGCATGTATCAAGCCAGCAGGTGTGGAACAATATTACGCCACACG
GTGCACATCATGATTTTAATGGTATCATGGCTTTCAAGGGAACCAGTGCAAATGTCCTTAATCAAGGTGCCGAAGCTTCTCCGGTCGTCTCCAACTCGAA
TGGAGCCCCAGATCTTCAGCGTGCTCTCTCTCTTCTGTCAAACAATTCAGCTGGTGCTGCCAACGACCAGCCAACTCCTCAGCTGCACCCTGGGCTGACC
ACCTTCGTCGGCTCCTCCAACCCTGTCACTGTGATGGAAGCCTCACCACCAGGACTCTGGCAAGACAGCACGCCCCTTGATCATCAGGCGCAGTTCCAGG
CTTTCGATCCCATTGCCGCACCGCAACAGCTCCAGCTCCCAAAACCCCCTTCCTTCAACAGCTCCACTTCCAGCTATGGCCAGATGCAGTGATGGATGCT
TCCTTCAGTTCTGCTGTCTGAAACTCTGAAATTTGAAACTGCTGTGTTCTAAAAAATAAAGAAAAGCCTGTGTGCGAAAACTCAGGCTTGAAAAATTGAT
CCAGTAGTGTTCCCGTTCAATGGCTTGAATAATCCATCCTTGTGATGGCGTGTAACTCAAATGCTTGGATTGCGCTGGCAAGTGATCATGGACTCTTGTG
TTCTTGATGGCATCTCAATTACACCTTGAATGGAACTCTGGTTCTT
>comp65473_c0_seq9
GCCAAGAAGAGCCGCCGCGGCCCGCGCTCCCGCAGCTCGCAGTACCGCGGCGTCACCTTCTACCGCCGCACCGGCCGCTGGGAGTCCCATATTTGGGATT
GCGGCAAGCAAGTGTACTTAGGTGGATTTGACACTGCTCATACTGCTGCAAGGGCGTACGATCGAGCTGCCATCAAGTTCCGTGGCTTCGACGCCGACAT
AAACTTCAATCTCAGTGACTATGAGGATGACATGAAGCAGATGAAGAGCCTGTCCAAGGAGGAGTTCGTGCACGTCCTGCGACGGCAGAGCACCGGGTTC
TCGCGTGGCAGCTCCAAGTACAGAGGCGTCACCCTGCACAAGTGCGGCCGGTGGGAGGCTCGCATGGGACAGTTCCTCGGCAAGAAGGCTTATGATAAGG
CCGCGATCAAATGCAATGGTAGAGAAGCCATGACGAACTTCGAGCCTAGCACGTATGATGGGGAGCTATTGACTGAAGTTGGTACTGAAGGTGCTGAAGT
CGATCTGAACTTGAGCATATCTCAACCAGCTTCTCAAAGCCCGAAAAGGGACAAGAATTCCCTTGGTCTGCAACTCCACTATGGATCGTTTGAAGGCTCG
GAATTGAAAAGAACAAAGATTGATGCTTCTTCTGAATTGGCGGGCCGCCCTCATCGGTTCCCTCTTCTGACCGAGCATCCACCAATCTGGACTGCCCAGT
CTCACCCCTTCTATTCAAATAATGAGAATGCATCAAGAGATCTTAACAGGAGGCCAGATCAGGGATGCACAGTAGGGGGTGTTCCCAGCTGGGCGTGGAA
GGTGACCGCCCCTCCTCCCACACAGCCATTGGCGCTGTTCTCGTCGTCATCGTCGTCCGCTGCAGCATCATCAGGATTCTCCAATACCGCCACGACAGCT
GCCCACACCGCCCCATCGGCCTCCCTCCGGTTCGACCCGCCGCCGCCGCCGTCCAGCCGCCACCGCTGAACGTTAAGAAGCCACGCTGTAAATTTGCCAG
GAAGCCGACATTTTTTCCTCTCTCGGCGTTGCAACTTTTTAGGTTTTGCGCCGGGGTGGTTTCTTGTAGTGGAGTGGATTCATAATTAACTGTATTTGCA
TGCTGCCCATGTGAAATGCTTCTCTCTCTTTTTACGCTCTCTGCACCGGATGTTCTGGGAGTTCTTGTTGCCGGGAATTAACTAAATTCACCGTCTGAGA
TTGATCTATACATTGTTGTAGAGAATCGAATCATTGTTGAAATTAGTTACAGAATTATAGATATCATAAGGCACCCTCTACTTGTGGACAAAGTTCAGGA
AGATTATTCCGGTTCTTTTCTTGGCTGTCAATTCGGCTCTTGTCCTTGGTGTGGAGCCACTTGCCTACTGTCTTGTTCTGTTGCTGTTTGTTTTTGGCTT
CTTGCACAGTTACAAATTTGTTGGCTCGAATGGATGGTGAGACATT
>Locus_11957_Transcript_43/43_Confidence_0.108_Length_2177
AAACCCAGCGGTTCTCCTCTGTTCCTGTCTGCCTCCCCTGGGTCGCGGCCTCGCAATCCAATCCATTTTGGCCGCTCTAAATTCTCCTAAAAAATCCCCA
AATCCAAATTTCATCCCTTCCCAATCCTCCTTCCGTATGCTTCAATCTCCGGATCAGTCTCCCATGCTTCACCACCTATTTCACACCGCAAGAATCGCTC
GTGCTGCGTGAGAAAAACCCCGACGTCCTCCAAGATCCCGGCCGATCGCGTCCGGTGCTGCGGAGACCCGAGAAATCTCTGTAGGTCCGTGCTAAAGATT
CGATTTTGTTCGGGTCCTTTCGATCCTATGCAGATCCGTACGGTCTTCCGGCTGATTCTGCCGTTCCAGGGGTAGCAAGCTCGTTCTTGCTGTTCGTGGA
TTGGTGTCTCCTTCCATGGGGAGTTTTCGCAGGTGCGGAAGTTTGTATTCTGTCTGATTGATCGGTGGAGAGCTAGCAACTGCTGCGGTTGCTCGGTCAT
GGCCGGAGATGAGGCTGCGGAGGGTAGCCGGAGCAGGAGGCGAACGGATCTAAACCTGTACGTCGGCCTCCCTCCCTTGCCGCGGCCTCCTGGCCGGCTC
GACGCCGCGCTGGATTGTCCAGTGTTGATTCCGAACTCGGCCGGTCCTGCGCTGGAAGCCCCAAGAACGGAGGAGCCGGAAGAATTGCTCACCCCGGCGG
TGGCGTACTCGCCGTCGAATGCGCTCTCCACCCCGGAAGATCAGTCGATGCTGGATCCCGTAGTTTATGCGTGGCTCGATGGCCACAGCACCGATGGTGA
AGATGGCACTGATGCTGTTGACCCGGCTGTGGTGCTTGGTGCACCGGTGGACGGTGCCAATGTCTCGCCGCCGCTGGCTGCTGGATTGGGAGGGGATGAT
CTTCCACCGTGGGTGGAAAGGTTTGTACACCCTCGTCGAGTGGGTGGTGGAGTGGAGATGGTCAGCACAACCAGCATACTTAGTCGGTCCATTCAAGGGG
CTGTTGGAATTGAAGCAAGGACTCCGGAGCTCCGCTTTCAGAGGTTGATCCAGATTAGCCAGCATCATAACATTCTGAGGTCAGGGTCGGAGAACCGCAG
CCAACGGGCTAGTAGTCCCGAAGCGGATAGGCTGGTTTGGGCTATTCAACGTTCCCATAACTCTCTAGACTCAGCAAGGCGGCAAAAGCTGGATGGCGAT
GACAAGGTGGGAGGGAAGGGTGCTGGTAAGAAGGATGGGTGCTGTGGATGCAACTCCAATTTTGAGTGCAATATCTGCCTTGATGCAGCTAAAGAGCCTG
TGGTTACACCGTGCGGTCACCTCTTCTGTTGGCCATGCTTGTACCAGTGGCTTCATGCGCATTCAGCGCTCTCTGAGTGTCCTGTCTGCAAGGGCGGGGT
GCTCAAAATGAATGTCACTCCGATTTATGGAAGAGGTGACGATGAACGGGATTCCTCCATCCCTGATATGCCTCCCAGGCCACGCGCAAACAGAATGGAG
AGCCTGAGACAGCAACTGCAAATGGCAGGCACTAGAGAAATTGCAACAGTAGTGAGACAGTTGATAGAAAACCAGGGTATAGTGAGAGGCCAAGCAAACC
CAGCTGTAGGGGTGGAGGTGCCTCTGGTTCCTGCAAGCCGGTCAAGGGCTAGGGCTCGAAGACAACAGAGGCAAGATTCCTTATCTGCAATAATGCTGAA

TATGGGCAATGTTGCCCCTGAGAGCAGCAATCAGACCCCATCGCCGCCTTCGAATGCAGACAATACTGCGCCAGCGGTTCCTGCAGCTCCTCAGCAGTCA
TCATCTGTGGAGCAACCGTCGACTTCTAGCACTGCAGCTGTTATAGTGGGGGAGCCAGGGTCAAGTAGGAGGTCAAGGCCTTCGGAATCCCCAACGACAA
GAAGAACCAGGAGGAGGCAGCAGGGGTTATCATAAGTGCTGCCATTCTCTCCAATAAAAACCTATCGACCATGCCATGTTCCCAGTTGTTTACCTTGAAT
AGGTCCAAAAACCTTCAAAGTTTAGAAGGTAGGAATGTATATGCCATCTTCCTCTCTCTTACATACCATGTATGCACCATCTTCATGGTGAAGGAATGGA
TATGTCAGTTTATCCATAAATCAGAGCGAAGGAATGTTGTTCCGTGTATGCTTGAATGAAATGTTCAGTTTATCTGT
>comp79344_c0_seq5
CTCTCTCTCTCTCTCTCTCTCCAGCTAGCCGCCGTTTGACCCTGCAGATTGCTTCCTTCTGCACGGCGCGATGGTACGGGGAAAGACCATATTGAAGAAG
ATCGAGAACGAATCAAGCCGGCTTGTGACCTTCTCCAAGAGGAGGGGCGGGTTGTTCAAGAAGGCAAAGGAACTGGCAATCCTATGCGACGCTCAGGTCG
CTGTTCTCATCTTCTCCAGCACCGGCCAACTCTACGACTTCTCCAGCTGCAGCATGAAATCAATAATCGAAAGATACCGCCAAGTAAAAGAAGGTCAGCA
AAATGTAACGGCAAGTAGGGAGGCCAAGTACTGGCAAGGGGAGGCGGAAAGCTTGAGGCAGCAACTACACAACTTGGAAGAGAATCATAGGTAAGAAAAT
CCACCAACAGTATTGACAGAGGTGAATACTTTCTTACTGAGCAACCTTGGATGAAACTTAAAATGCAGTGTAATGGAGTCCATCTAATAGCAACGAATGT
GAAAACTTTAATAACAAGTCATATATTTTTATGTCCTTAGGGAGCCATTGGGGTCCATGGATTCTGTAACATCCTAGCTGGCATGTTTATAGTGGTCTTG
GCCTATGTGGTCTAGATGTTGAAGTGTGATGGGATTAGTTCTATCTTGGGTCTTTAGGTAGGTCAGAGCCTACATATAAACCTTGTCTTTTAGAACATAG
CACATCTGGATTAACCCTTTTACCAGAAGTGAACATGAAAGCATAAGAAACATGCAATGCCCATGCGTGAGCTTGCTCGATGTGGGACTTGATAATAAAC
AGGTTTTAGATATAGGGTGTTACAAATTCTTATTTGTGGTTGACAGAACAGGGAGCATGCATTGTTGTAGGGGAAATCATGCAAACGTTTTCAGGTTGTC
AATTTGGCACCACAGTACAAGCCTGAAACACAAGATAACAAGTGCAAACTGCCACAAGATGTACGAAAATTGTGAGAAGATCAATATTACTGCAAGCAAT
GTATCACAGCCAGGTGGTTATATAATATAGAGAAGTAGAACGCATGACATCTACCTTCAACAACTCCTGCAGGCACTTGCTGGGACAACATCTTTCTGGC
TTGTCAATGAGAGACCTGCAGACTTTAGAGAACCAGCTGGAAACGAGCCTAACTAATATTCGGCTAGCAAAGGAACGAATTATGATTGATCTGATTCAAG
AATTGAACAAGAAGTCAAGCCTCATAGACATCGAAAACAAGGAGCTACACCACAAAATCTGTGCCATTAGTCAAGAGAATGCCAATTTGAAGAAAGAGGT
ACATGGACAGCCGGATGCCATTGAAGAAAATACTGGTTCTGTTACTCAGTATAGTACTAACACTCCTGAAGACCTAAATGCGACGTGTCTCGAGCTACGC
CAACCACATCATGTAGAAATAGAGCAACCAGATGTGCCTACATTGGAACTGCGTCTCCACTGATTGCAAGAATCAGCAGCAGAATCATCGGAAATGAGGA
GGCCAGCCATTCAACGCAAATTCATAAGACTTTGTCTCCACTGATTAAATACAAAGAGGAGTTCCTGTGCAAATTACTATTATATGAATGTAACAGATGT
AATTTATTTTTTATGTATCCGCAGTACACATATGGCCACCATAAACTTATGTTCAAAAGGCATATATACCTATTGCTATCAAGCTACG
>comp79901_c0_seq22
GACGCGGCGCGGGCCGAGGTCCCGGAGCTCGCAGTACAGGGGCGTCACCTTCTACAGGAGGACGGGCCGGTGGGAGTCGCACATCTGGGATTGTGGGAAG
CAAGTCTACTTAGGTGGTTTCGACACTGCTCACGCGGCTGCGAGGGCTTATGATCGTGCAGCGATCAAGTTCCGAGGGCTCGACGCGGACATCAACTTCA
CTTTGAGCGACTATGAGGATGACTTGAAGCAGATGAGCAATTGGACAAAGGAAGAGTTCGTGCACATACTCCGCCGCCAGAGCACGGGGTTCGCGAGGGG
GAGCTCCAAGTACCGCGGCGTGACGCTGCACAAGTGCGGCCGCTGGGAGGCGAGGATGGGTCAACTTCTTGGCAAGAAGTAAGCCAACCTCCTGGCTTTG
ACCTTTCTACTCTGTTAGCCTAGTTAAGTGTTTTTTACCCTGGTTATATGAACAAAATATGTCGAAATGATGTTCAATTAACTTGTCATTTCACGTTGGG
TTCGATCAGGGCTATATCCCTTATGTTCATGTTAAGTAGTTTCAGGTGATTGTAGAGAATACAACAAGGTGATCTTGAAGTATCACTGATTATAATCCAT
GCTACTAGTAGTTTGAGCGTCTCTGTTGCCGGTGAAATCTCCACCCCTCTTGTGACTTTACCCCTCTACCGTTGAATCCGGGAATCCGGAATGGACCTAC
AAGATCGAGATATGTGCCTTGATGAACAGTGTTTCGGTGAACAGCAAAATCGGCACCCGTGCACAGTGTCCTGATGGACGCAACCTCTAGAGTTCACATA
TATGCATTGGCCAACAATGCAATTCCTGCTTTTTAGTCAGGAACCAGTTTTGTCAAAGTCTACGACACTAGGCATCCCTCTGTTGAGACATATCTCAGAA
CTGGAATTTACATGCAGGTACATCTATCTTGGACTCTTTGACAGCGAAGTTGAAGCTGCAAGAGCATATGACAGGGCAGCCCTTCGCTTCAATGGGGGGG
AAGCTGTTACTAATTTTGGGCCTAGCTCCTACAATGGAGGAGATGCTCTACCCGACACCGAAAATGAGGGTATCACAACCAGCCTCGCCATTTATTTCTT
TTCATATAAGCAAATGTATCTCAGGTGCACCTTATAATCATCGCTGTGTTCTTAGCAACTGTTGATGGTGACGCGGTTGATTTGGATTTGCGGATTTCAC
AACCTAATGTGCACGACACTAAAAGGGACAATACCTTAGCTGGAGGCCAGCTAACATGCGACTCCCTTGAATCTTCAAACACGATGGCCTCTCAGGTAAA
CTGACTTCAAGACTACACAGCAGGGAAAAATGCTCGGTTTCTCTATGACTTTCTACTACTTGCAGTAACTAAAAAAGTTTCCCAGAAAAATAAAATCTTT
AAGATGGAAGCAACTAGGTTGAATTGTCTTGTATCTTTGCGTGGAATCCAACCTTTGATAGTCCCTACGCTATAAAGATTCATAGGAGTATTTGATTGGT
GTGTCGTCGGTTGAGATATTAACTAATTGGTCCAGGTCCTATAACCTTTATTGAACTTACGATATTTGCACCTATCTGTTGCTCCTGTAGCCAATGAGCT
CATCGTACCCGTGGCCTGTGTATCACCAAAGCACGACAGCAGTACCACCTCACCATCAACGCTTGTACTCATCTGCTTGTCCTGGCTTCTTTTCGAACCT
CCAGGTATTACCAAGCTAAAGCCTATTTTTTATGTGCAAATTGCTACTCCTACGTGATCAATTAGTTCAAACTGTTATTCGAATCCAGGATTGTTTACTG
AATATGTCCTGTCTTCAGAAAAAAATAACACAAAAAGGAACATAATATGTCCTGTCTAATTGTGGACTGATACTAGCCAATTAAACAGTTTCCCATATCCA
TTTCCTGTGTTTATTGTTTTGATTTGCTGGCATGGTTAAACTATAGTAGCTTGATTCGATAAAAACACTAAGCAGGCTTTGATATTTTGAATGAAATGTC
GTTCTTGGAAATACAGCCTTTTCCTTGGTATATCAAGGACAGTTGTCCATCTGCACTCTGCACCCTCAGCTTGTGTAACTGTTTGCCTTGTGAACTGATT
GATGTGTCAATCAGGCAGTTCTGCCAGTTTGCATCTTTTGAAATAGTGCAGTCTTCCAGTCTAACAGCTCCATGACATAACTTGGGATGCATGCTGCAGC
TTTCTTACTGAGGATGCTCTAGATTTACAGAAATTCTCGCAGTTAGCAGAGGGCTATCAAATCTGATTTGCTTAGCTTCCTGCAGACAATAAGATAAGAA
CTCTAGAATCCTGAAGAAATCTTACTTGTACATGTTTACCAAAGTATCCTTACTCCTTAGTTGAGCCAATTTTGGTGAAGAACCGATAGCTCATTACTCT
GTTTCCTGGGAATGATGTGGGCCTAAACCATTCTGTGGTCAAAGTTTAACAAAGCCCATGCTTGTGCTGTTGCAACTGCAGGAAAGGCCAATGGAGCGAA
GGCCTGAGTTGGGTACCCAGTCATTCCCCACCTGGGGATGGCAAATGCAGGGCTCCCCTCACATGCCGTTGCATCACTCTGCAGCATCATCAGGATTCTC
TACCGCCGTCGGCGCAAACGCCGGCGTCTCGTTGCCTTCTCACCCGCCGGTGCCGTTCCCGGACCACCAGTTCTACTTCCCACCGACGGCATGAGCTTGT
TGGTCTGGTTCAACTCTTGGGACGGCAGCTGATCTTATGTGTGAACATTTCGCAGCTTGCCGGTGACCGTTGTTAATTAATCGGGGAGAGAGAGAGCCAGAA
GACCCAGTATGGCTATCTCTCTGTGGCCCGTGCCTTGTTATGATCTTAGTTATTGCTACTACACCGTGGATTCATCATGGTTTATGAGATTCTTTACTCA
AGTTTCAGCTTAAATGTCCAAATACATGAATGCATCATACACAGGATCCGTCATTTTCTAAAAAAATACAGGATCTGTGTGAAGTGT
>Locus_11912_Transcript_51/63_Confidence_0.314_Length_3693
CACTGTCCAGATTCGCCTCCCTCCCCTCCCCCTAGCTCCTACTTCCCGCTCCTCCAGCTACGGCCGCGCGCTCCCCCTCCCCGCCGGCCACCTTGGGGGG
CTCCTCCCCGCCGCGCGTCCGGCCGCTCTTTTCCTGGCGCCTCCCGCCGGGCGCTCGCCCCGGATTTCTCGGCTCCCCATCCGGCGGACGCCTTGACCGG
GGTGGGTTCTTGGTTCCGGCGGCTCGGTGATCTGTGCGTGCGAAAGGCGGGGCTTTTAGCTCGTTCTGTGGGGGGAGGAGGAAGACGGCGGCGAGGCGTT
CATGGGTTAGATCTGGCGGTCCGGCGCCGCGAATCTGAGCGAGGTTTCGGTGGAGGGATCGGGGCGGAGAAGTCGCAGTCTCTGGCTCGTTCTGAGCAGC
TTGGGCCTCTCATGTCCAAATCGGGTATGGATGATGGGTGCGGTAACTGTCGGAAGGAGCGTCGCTTGCAGATCTGAAAGCCATCCAGCTCCCTTGTTCG
GTTGACCTGGAATGGCCGTGCTTTCTGCTGTTTGACTGTCAGTAGTTGCTGCTTAGTGTGTGTGCAAGAAATTTAGCTGGAGGTGATGTGCTTAGGAAAC
TAGTCTAGTTGCTGATATCTTGGTCTCTGTTGAAGAACGCTGAAGCTGGAAGGAAGTAGGTCGCATTCCTGTGTTGTTGATATTTTGTTGTTTCTCGAAT
AACCTGGAGGCTGGAAGGGATCAGATCCAGTTCCTGTGGCACCGGTAACAAGGTGCTGTTGGGGGAAGGCACCATGACCTACTTCCCTGAGGAGGTGGTG
GAGCATATATTCAGCTTCTTGCCGTCGCACAGTGACCGGAACACGGTCTCGTTAGTGTGCAAGGTGTGGTATGAGGTTGAGAGGCTGAGCCGGTGTGCTG
TCTTTGTGGGGAACTGCTACGCGGTGCGCCCAGAACGGGTGGTGCTGCGGTTCCCCAATGTGAAGGCCCTGACGCTGAAGGGGAAGCCGCACTTCGCGGA
CTTCAACCTCGTGCCGCCTGATTGGGGTGGCTACGCGGGGCCATGGATTGAGGCGGCAGCGAGGGGCTGCGTGGGTCTGGAGGAGCTGCGGATGAAGCGG
ATGGTGGTGTCGGATGAGAGCCTTGAGCTGCTAGCTCGGTCCTTCCCGAGATTCAAGGTCCTCGTCCTCATCAGTTGCGAGGGGTTTAGCACCGATGGGC
TAGCAGCTATTGCGAGTCACTGCAAGCTCCTGAGGGAGTTAGATTTGCAGGAAAATGATGTGGAGGACCGTGGGCCTAGGTGGCTCTCCTGCTTCCCTGA
TTCCTGCACCTCTCTTGTCTCCTTGAATTTTGCCTGCATCAAAGGGGAGGTGAATTCTGGTTCATTGGAGAGGCTTGTTGCTAGGTCTCCAAACTTACGC
AGCTTGAGGTTAAATCGATCTGTATCAGTAGATACACTCTCAAAGATATTAGCATGCACCCCTAATTTGGAGGACTTGGGGACAGGGAATTTGACAGATG
AGTTTCAAACTGAATCCTACTTCAGGCTGGCTAATGCTTTGGAGAAATGCAAAATGTTGAAGAGTTTATCAGGATTTTGGGATGCTTCTCCTGTTTGCGT
TCCATTTATCTATCCCCTCTGTCATCAACTAACTGGACTAAACTTGAGCTATACTCCCACTCTGGATGCTTCTGATCTCACTAAAATGATCAGCCGCTGT
GTGAAACTCCAGCGTCTTTGGGTACTGGATTGCATCTCAGATAAGGGTTTGCAAGTGGTGGCCTCCAGCTGCAAAGATCTACAAGAACTGAGGGTGTTCC
CGTCAGATTTTTATGTTGCTGGTTATTCTGCCGTGACAGAAGAGGGGCTTGTTGCAATATCGTTAGGCTGTCCAAAACTTAGCTCCCTGCTGTATTTCTG
TCACCAGATGACCAATGATGCACTAGTTACTATAGCTAAGAACTGCCCAAATTTCATAAGATTTAGACTCTGTATTCTTGAGCCAAAGAAGCCAGACGCC
ATGACAAACCAGCCGTTAGATGAAGGCTTTGGTGCAATTGTTCGTGAATGCAAGGGGCTAAGGCGACTGTCAATGTCGGGTCTTCTCACTGACAAGGTTT
TCATGTATATTGGAAAGTATGCCAAGCAACTTGAGATGCTTTCTATAGCATTTGCTGGAGATAGTGATAAGGGGATGATGCATGTTATGAATGGATGCAA
GAATCTAAGGAAGCTGGAGATTAGAGACAGTCCATTTGGTGATGTTGCGCTCTTGGGGAATGTTGCCAAGTATGAAACAATGCGATCCCTTTGGATGTCA

TCATGCAATGTCACATTAAAGGGGTGCCAAGTCCTTGCGTCTAAGATGCCGATGCTCAATGTGGAGATCATGAATGAGCTAGATGGAAGCAGTGAAATGG
AGGAAAACCATGGAGATCAATCTAAGGTGGAGAAGCTATATGTTTACCGCACAACTGCTGGAGCGAGGGATGATGCACCAAATTTTGTTAAAATCCTATA
GTTAGCTGAGAGTAGCCTGGTATCTCCTCACTCTAAAAGGTGATCCTTTTTCCAGATATATTTTCTTTCATTTGGGTTTTTTGTGTCTGTTGGGCAATTT
GTCTGCTACTCTGTGGTAGCCCAAAAGTAGTTTGCATATACTGTTCAAATTTTGATTTTTTTTCATGCTTAATAGCTCTGTTGGATACTGCATCTTACTT
TATGGTCCACAACACGCAAATGTTGGCTTAAATGTTACATTTAGAAGCGTCTGAGTTAACTTGTTTCTTAGTTTGTAAGTGCATTATATCAGAAGGGTCT
ATAATTATTGCTTGAAATGCTGCATGAAGAAGCTTGTGCCATCATCATTAACTAAAACCAAGACTGATTTCTAAAAGATCCGAATGGATAATACTTGAGA
ACTGATGTTTGAACACCTTTATTCTGGCAAATTTTTAGCCGCTCTTTTTGTTTCTTCCCCAGCATATCATTCATATTCCATTGCGAGTATGGAGCAGGAG
AGCATCTGATATGTCTAACTCTGTCTTCCCACTCCCTACTCGATTGCTTTTACAGATGGTCGGTGGAATCCGGCAGGCTGGGGGCCGCGTCAACAGTGGT
AAGTTCTTGAGGTGTCTGTCTCTCCCTGTGCAGGGGTCAGGAAAAGCCACACCGATGTGCACCCTTATCTTGGAGCAACTGGCTGGGATCCATCATCATC
CCCTGTACCACCGCCCCCTACCCTTTTGTGTTTGGAATTCCTTTGTGTTTGCTCACTCATATCCACTGAGGCCCTTTATCATCCAGAGCATTCTCACTTG
AGAGGACCGCCTTTTTTCCGTTTATCCCACTGTAATCTGTAGTGTACCAGTATCCATTTGCATTTGACATTGGCAGTGGACTGTTTTGCCGCTGTTGTTG
TAGTGTTTGTTTCAGCCTGACGATTTGTTTCTTGCTCCTGTGAATGCAGTGCGTGGAATAATTTGCGGTTGTTATCTGCGACATTCTATTCTCTGGTTTT
GAATTCTTGGTGTTAGCCGTACCAGTTTTACGTGCTTGTTACTGAGCAAGAAGGCACCAGTACTCGTGCATTTTAAGGGCGTGCTCAGTTTCTGATGCCA
TTTTTCTCGCTTACATCATCTTTCTGTATTGCTGCCCTCAAATATGAATTCAAATTAACGGTGAAATCCAAAGCAACTTCTGAATCAATCCCG
>comp73418_c0_seq8
TAGGATGACTGTGTGGAGGATATCTAACGTGGTGCTTAAACTCGATGGCAGCCAATGGTGGTTCTTGCCTGCCTTACCATGAACTAGTAGTTTGGATCGG
AGTTGTTCTTGACTTACTCATGCGTATTCTTGGGAGTTGGGATCATATCAGTATGTATACATCTGTTATTGCTTGGTGGTGACTTTTCCTTAGAGGTGCC
TGATATGGTGGCGTTTTTTTCTTTGACACATGTTGTATGGATCATAAGTGATGTTATACTCTTTACTAATTTCATGGCCTTTATTATCTTTAGGTTTCGT
TTTCTCTTTTTCTTTTGTTATTATTGGTAAGAAGTATTCGTGTCCCTAGACTTTTGCCATGTATGTACATGTTCGTAGCGAGGTTAGTGATACTGTGAAC
TGTGGTAGTCTTGTTTCGCTGGGGGGAACTTTGATCTGTTGTTCTTTGCCTCAAAACACTGTAAGCCTATTTCTAATATCTAAAACTTGTAAGTGTGCAT
AATGCAGATCAAGCTTCCGAGGTGTTATTTTTCTCATTATGTTTTAGAAACTTAATGATAGATGCAAAGTTATTTTGTTAAATGGAGTCTCAGTCCCGTA
GCCATAAGGGAGGATCGAATCCAAACTTTAGGCTTGTGTGAGTCTTCTTGAGTGCAGCACATGATAAACATGGATCTTAGGGCATTGAGTTAACAGTTAG
TCATCTTCTTTTCTAAACATAAGGATTAGGGTGCTAGTCACACACCATTAACAATAATGTGAATCCAAATAGCATAAGTAGCACGCTTGAGGCCGGCCAA
CACACACTATTCATCTCTTGCTGATTCACTAATTGTCGGTTTGCATTTGGAAGTGTTCTCTAGTTTCTGTGCAGTAACTATTGAGCTAATGACACACTAT
GTATAAATGACAATCGTAACAGATCATTTTGAACCTTTACTTGATTATTATTAGAAACACCTAGGATTTGAAGGATATCGATGTTACTGACTTACTTCTC
TTTTGCTACATCTGGTTAGGACAGGATTCTACTGTGTTACAATAGGGTTTTCTTTGGAAATTTAACGTTGTACAATAATGCCTTTATTTTTTTTGTATCA
GGTGACTCCTTCGGGAAAATGGTTAATGATCATACGAGGTCAAATTTGGTTTTTGATAATAAGCAATCTCTATTTGCAAGTCATAACATTGACTACGGCC
AGCCAATAGTAAGACTCATGGTGATATAAAACATCAGTTTTTTCAACTCTGTTAAAGTCACCATTCTAGTTTTAAAAAACAATAAAAAGCTCATCCTATT
CTTTACATGGGGATGTGCATCTGATTTTATTTTTCTACCATATTACCCAGGCTTGTATATCATACCCATACAATGATTCGGGCTCAGGAGGTGTTTGGGC
AGCCTTTGGGTCACGCACTAGCGCTGCAGCTGTGGTATATCCTTCTAATAAACTGTTGTACTAAAATCTCTCAAGACCTTTGAACTTCAGTGCTGATGTC
GCATTTCAGCATTTAAAATTTTACATGAGAATGTTAAACATATATGATCCTAGTTTGGGGAATCTCCTAGTTTTCAGTTCATTCTGATTGGGCTACAGTA
TGCTGTCTGAACGGTCCATCTTACCATGGTCTAATCAAATGTAGAAACTTAAGTAGGCTAGTCGCCTAATACCTTAACTATGTTTATTTCAAGCAGACAC
TGCTTAATATGATCCTTGGTTCTTTGCATAAGCATGCATGGGCCATGACAAAAGGTCTTTTTTATCACTGCTATAACCGATATACTAATGGCAACAAAAT
GCTCAAAAACTACCACATCTTTTTGCAGCATATTTAGCCATAACCATAATGGGAAACCGTGTGCTAAGCTAGTTGCAACTGCTTCTATTCTCCAGCGACA
AATGATAATATCTAGAAAGCTAAGATCGCAGCACTTAATTGTGGCCTATTTGCACAGGTTTGAACCAGATTTTGCATTTCTTTCTCATGCACATACACTG
TTACGTAAATCACTCTATTATTTAGAAGCAACAAAAGGACCGTACATTAATTAATTCTCTAGCTGGTTATAACAGAAGCTAAACTAGCTGGATTTTGGCA
GAGTTGATCTTAGTATTAGGGTTAAAAGTGAAAACAATACTTGACTGGATGCAGCAACAGTGCTTTGTTAATTCTCTGCTCCAGTAATGTTTTCAGAACT
GCATCGTTGTTTGTGTGTCCAAATTGTGCTTTCTCAGTTCATGCACGACTCCATATATATCTATCTCAGTCTGCAGTTACTAGCGTGCTGGCAGCTCATA
GACACATAGTGTCCGTTTAGATGATTTCGTTTAGATGATTGCTGCTGTTTGTAGTTGTATAAATTGACTCATGATATCAACCAAGGTTACACATAATTGT
TGCCAGGATTCCATTATATGCTAACGTTAGCTCACATATAAGGCAGGGCCATCCATCCACCGTACAGCTTGTTTATCTGTAAGTTCATCACTTTTGAGGG
TTTTGTCTAATCAATTGTACTTAAAGCAGTTCCATCCCCAAATTGCTTGTGGGGGCACATCTGCGAGAGTTCCCTTACCTCTGGAATTAGCAGATGATGA
GCCCATATATGTCAATCCCAAACAATATCATGGTATACTTCGCAGAAGACAGCTACGTGCTAAGTTAGAGGCTCAGAACAAGCTAGTCAAAAACCGAAAG
CCTTACCTTCATGAGTCTCGGCATCTTCATGCAATGAAGAGGGCAAGAGGTTCTGGCGGACGTTTCCTTAATACTAAACAGCTCCAGGAGCAGCAGCAGC
TGAAGTCTCGCAATGCCTCCACCAGGTCCACCACAAATGGCGCAAATTCCTCAGGTTCAACACATCTACGGCTTGGTGGTGGTGCAGATGGAGATCAAAC
CATGTCGGGGACGAAAACAATGGCCTCACAAAACAATAGCAAAAAGGCTGTTTCTTCTTCTGCAGCTCTTGCTTTCACCGTGACTCCTATGGTGCGCAAA
GATGACACCTTCTTCCAGCACCTCAGCCACAATGTCAGCTTCTCCAGCCATTTTGGCCAGGCAAGCGCCCAAACCGGCGTTGGAGGCATCCATAATGAGA
CCCAGCATAGGGTTTCCGTGATACAATGACGGTTTGCGAAGCTTATCTGGTGATCCAGGCTTCTAGGTGTCCTCGTGTCCGGTGTAGTCTTGGTCGCTCA
GGCAATTCATCCTTGGCTTAGTTTTTGGTGTGTTAGAACCTACAAATGTTTCATACTCTGTTGTGGTTTGCAGAGCCCATAAATCAGGGCTCTCCTAAAA
AAAATCAGGGCTTGATGGCGACACTACTATCCAAGTATTGTTGTAATGGTGTGTGTTAGAAACTCGCAAAAAACCCTTCTTTGTGCTCGTATTAGTACAT
TATGCTGCACAATTAGCTGCTATTCTGTTCGTACTACAGTCTGGT
>Locus_3441_Transcript_39/417_Confidence_0.038_Length_1731
TGTAAATCTCTTGATTGAAATAGTGGCATGTCATCTTATTTAATTCAGTTATTTCATAAGGTACAATTTGTTTAGCTGACAGCTCAGACATGCTTCTATG
CAACATGTTTATACCAATGCTGTATTTTGGCAATCCTGGATGTGGCATTACTTAACTGTGCTTTCATATTCAATAGTTCTGGCAAAGGGAGGCTGCAAGC
TTGAGACAACAACTGCACAACCTGCAAGAAAGTCATCGGTATGGCTAGAAAACCCTGCAAATTTTACAAATGTACAAGTCAGAAAATTTGCCTAATGTAA
AAGGATCAAACTACAGGATTTGTCAGCATGAGCACAAGTGCCTAACTTCCGAGTTAGATAACTCTGGAGGAAGGAAATCCTTCCTACTGTTTGTTTCAAG
AATATACAAAAATTCAAAACAACATTAGAATAGAACCAAAAGAAGAAACAATATTCACCATGTAGCAATACTCAATTCCTGGTTGTCTTATTTATAAAGG
TATAAAACTAGGTTCTGACGAGACAATTATGAATCAACTACACCATGTTACAGATCATCCATAACTCTTGGTGATGCAAATTAAGGGCTGTAAAGTGACA
AGTATACCTCCACCAACTAATTTGTGCAGAGTGTATAAAATTGTAAAACAATGGATAAGAATGAATTTAGTATACAAGATAGCATACAATGCCCAAGTTT
AAGGAGACATACTGATTTACTGACTCCATAGGACAATCTTTTGCTTATGAGGACATCAGTTTTCATAAACAATATCAATGTAAAAGGAGGTAACAGTTTA
TGAATTTCCTATGCCAAGCCATTTATAAGTCAAAAAATGTACCTATTTAGCTTTTATGCATTCCAATTAACTTCCAAAGAAGAAGAGTACATACTAATGG
AAAACAAATATGATATTATCATCTGTTTTGCCCGGTAGTTTCTTGAGTCTCTGATTTGTCCGCACTTCACTCCCAACCAACACAACATTAATACACATGG
GCAGAGGAATATCAGGTTATCAGGTAAACTGAATACAGTTTGATTGACTGCAGGCAGTTGATGGGACAAGATCTTTCTGGATTGGGCGTCAAGGAACTCC
AAAATCTAGAAAATCAGCTAGAAATGAGCCTACGTTGCATCCGGACAAAAAAGGACCAACTCTTGATTGATGAAATTCACGAACTGAATCGAAAGGTTCT
GGAATATTCTGTTATTACTCTGCAATGAGAGGTCTCTGAGTGGATTTCAAGAAGCTGACTCTACTGGCTCATTTTCAGGGAAGTCTCATGCAACAAGACA
AGATGGAACTATACAAAAAGGTCAACCTAATCCGTCAGGAAAACATTGATTTATACAAGAAGGTATTTGATAAATCGTGAATGAAAATTCACAGGATATA
CACCCCATCCATTATTGACTGAGGTTTATTTCTCTACTCAGCTCTACGAGAAAGAGGTGACAAGTGAAGTCAACCGAGATTCATCAACTCTAAACTTTGC
AGTTGTCGAGAACGTCAACATTCCTGTTCATCTTGAACTTAACACTCCACCGCAAGAAAATGATGTTGAGCAAACTGCACCTCCTAAACTGGGGTAACCC
TGCTTTTTTCTGCAAAGGAACTATTGATTCTGTTCTTAATTCTTTAGTGGCATATTGTTTACTACTAACTGAAAACTTGCAGATTACAACTAAATCCATG
AAGGCATGCAGCATGGCACCGCTTATGTTCA
>Locus_7001_Transcript_68/84_Confidence_0.092_Length_1287
TCAAATGCTGTTGTGAGTTTTCTTTGTGGTTTACGGTTCTACAATATTTTTGAAGTATGTACTGATTAACATGGTAAACTTACATACAAATATTATCCAT
CTGTTTTGGTGTAGCTTCTGACGTTTCAGTTACGGCTAGTTCAAAATAACTTTATCAAGAAAACATTTTGTCCCCACCTATCATTTGTGCTTTGCTATTT
TTATTACCCAAAATGCATTCAAGATTCAATGAAAATGGAAAACGGAAAAACTAATTGGACTTTTATGTGTTATATGAGAAAATAATTTCTTATTGGTTAA
ACTAATAACGACATTTAACAGGAGTGCACCCAATTCAACAACAAAATCCAGGAGAATGTGAAGCTCTTATGCCCATAATTTATGAGCAAAGATGTAAATA
TGTGAAATTGATATGTGTGTGCTCTAACTCATGTTTTATCACACAGGTTCATCCCCAAATTAATGGTACAGCAAATTGTAGGGTCCCATTGCCTGTTGAA
CCTGCAGCCGAAGAGCCCATATTTGTCAACGCAAAGCAATACCATGCAATCCTCAGGAGAAGGCAAATGCGTGCAAAATTTGAGGCCCAAAATAAACTGG
TGAAAGGCCGGAAGCCGTACCTTCACGAATCTCGACACCGTCATGCAATGAAGCGAGCCCGTGGGTCGGGAGGCCGGTTCCTTACCAAAAAGGAGCTGCA
GGAGCAGCAGCAGCAGAAGACACTGCCTTCACTTCAGACTCCAACATGTGTGGCAAGTAAAATGTCACTCGGCAGGAATCAATGCCCTGAAAACAGCACA

TCTCGCTCGCCTTCTACACCGACAGACTCTGGGATCTCAAGTGTTTCCAACGGAGGTGGCATGCTGGCTCACCAAAAGCACATCACCTTCACATCTACTA
ATTTCCTTCCCAGCATGAACTTCAGCACCCAGAATGGAGGTGAAAAGATGGCTGTCAATGGTGCGCACCACCGTGCCTCCATCGTGAGGTGAACAGCCTC
GATTGTGGGGCGGTGGTATGTACCATCACTGGCGCGCTCCGGTGAGCCCGTGGCAATTCATCCTTGGCTTATGAAGTATCTGTATCTGGTAATAATTTGC
TTTCAGTTTAATATGTAGAGCTTGTCCGAGATGAACTCATTTATATTTGTTGCAACTAAAACGAGGAGCTACTTGTTGATATATGTGGATATTGTGGATA
CAGAAATATCCTGATGTTTCTTGATGTACCAAGTCCACTATTAAAGTATGACTGGAAAAAACGTTTAATAACGTAATCATGGTTTGG
>comp65473_c0_seq12
GCCAAGAAGAGCCGCCGCGGCCCGCGCTCCCGCAGCTCGCAGTACCGCGGCGTCACCTTCTACCGCCGCACCGGCCGCTGGGAGTCCCATATTTGGGATT
GCGGCAAGCAAGTGTACTTAGGTGGATTTGACACTGCTCATACTGCTGCAAGGGCGTACGATCGAGCTGCCATCAAGTTCCGTGGCTTCGACGCCGACAT
AAACTTCAATCTCAGTGACTATGAGGATGACATGAAGCAGATGAAGAGCCTGTCCAAGGAGGAGTTCGTGCACGTCCTGCGACGGCAGAGCACCGGGTTC
TCGCGTGGCAGCTCCAAGTACAGAGGCGTCACCCTGCACAAGTGTGGCCGGTGGGAGGCTCGCATGGGTCAGTTCCTTGGCAAGAAGTAAGAACACTTCA
CAACCTGAATTACCTAATACTAGACGTGTTATTTCTATACTGCAGATCCTTGTGATTTTTCCTATCTCAGTCTCATTCTAGAGTTATGAACCTAATACAT
GAACTCCCATGATTTAATAGTCTAGAAATAGTAGGTTAGATTGATGTGGCATAATGGTTGTTTGAACAAAAGAAATTAAAGAGAAAAAAGGCAGTGGATA
GGGAATTCCTCACCGGTGGGAACGCCATGCTTGGGTGCAGGTACATATATCTTGGGCTATTCGACAGCGAAGTAGAGGCTGCAAGGGCTTATGATAAGGC
CGCGATCAAATGCAATGGTAGAGAAGCCATGACGAACTTCGAGCCTAGCACGTATGATGGGGAGCTGCTGACTGAAGTTGGTACTGAAGGTGCTGAAGTC
GATCTGAACTTGAGCATATCTCAACCAGCTTCTCAAAGCCCGAAAAGGGACAAGAATTCCCTTGGTCTGCAACTCCACTATGGATCGTTTGAAGGCTCGG
AATTGAAAAGAACAAAGATTGATGCTTCTTCTGAATTGGCGGGCCGCCCTCATCGGTTCCCTCTTCTGACCGAGCATCCACCAATCTGGACTGCCCAGTC
TCACCCCTTCTATTCAAATAATGAGGTTAAGTGATACTAAATTACTAAAATACATGCAATAACTTTCTCAAATATATTGCTTTAGCTCCCTAAACCTCAC
AATTGCAAGCATGTTCTGAGCATCACATGGTTCGGATCGAGCTGTCCGGCACCAATTTTGAACGTAATCTTGACCATCTGAGGAACTGATTGTGCAGCCT
GAAGGCGTGCGTTCAAATGTTAGATTTGTGTTACTTTCGTTCATTTTCACTTCTTGTGCCGCTGTCAATTTTGGCTCTAGTCTTACATCACCGCTTCATT
TCCTTGGATTCTATAGCCCGCCTGTTTCCTGTCGATTTCGGAGATATTAATGCGTAGCACAGGTTTCATATGTAGGATCTTGAGTTTTCTGAGATGGCCC
ATTCTGGAAACAGTAATAGTCGCCTTAGGAATAGAGATGTGTTTGATTTTTTTCCTTCAAAAGGAACATCCTAGAGAGGTGACTATTCGACATTAGGTAT
GCTAATCCATCGGTCAACTATACCTGCCTGCGGCTAAGCACCTTTCAATGTTTCTTTTTTCATTCATCGTATCATATAAAAGTAGTAAGTATGACTATTA
TTCAAACCTTTCTTGAAAAAAATAGTTAGGATGTTGCCATCTTGTAATGCAATCATAGAATTGGAAGTGCTGAATTTGCCATTTGTTTCAAACAAGCATT
GTATATTGGTTTATCATTCAACTTGAGAGTTTGGTCAAATTGAACTCTCGAACTTTTCAACTCCTAAACTTTAAGGTCAGGCTCACATATGTCATTTTCA
AATTTTATTGGACGGAAAGTTGAGCTGGACGTTTCGAGCTCGGCTTTCTATCCAACTGAACTAATGGAAACACTGATTTCATCGCAACAAATTTTCAGCA
ATAGATATTCAATCTCTTCTTGAATTCATCCTTCATCAGTATGGTGAATAAATTGTTCTTGGATTATGCAGAATGCATCAAGAGATCTTAACAGGAGGCC
AGATCAGGGGGGTGTTCCCAGCTGGGCGTGGAAGGTGACCGCCCCTCCTCCCACGCAGCCATTGCCGCTGTTCTCGTCGGCGTCATCATCCGCTGCAGCA
TCATCAGGATTCTCCAATACCGCCACGACAGCTGCCCACACCGCCCCATCGGCCTCCCTCCGGTTCGACCCGCCGCCGCCGCCGTCCAGCCGCCACCGCT
GAACGTTAAGAAGCCACGCTGTAAATTTGCCAGGAAGCCGACATTTTTTCCTCTCTCGGCGTTGCAACTTTTTAGGTTTTGCGCCGGGGTGGTTTCTTGT
AGTGGAGTGGATTCATAATTAACTGTATTTGCATGCTGCCCATGTGAAATGCTTCTCTCTCTTTTTACGCTCTCTGCACCGGATGTTCTGGGAGTTCTTG
TTGCCGGGAATTAACTAAATTCACCGTCTGAGATTGATCTATACATTGTTGTAGAGAATCGAATCATTGTTGAAATTAGTTACAGAATTATAGATATCAT
AAGGCACCCTCTACTTGTGGACAAAGTTCAGGAAGATTATTCCGGTTCTTTTCTTGGCTGTCAATTCGGCTCTTGTCCTTGGTGTGGAGCCACTTGCCTA
CTGTCTTGTTCTGTTGCTGTTTGTTTTTGGCTTCTTGCACAGTTACAAATTTGTTGGCTCGAATGGATGGTGAGACATT
>comp76332_c0_seq2
CAATTTCTATCAATAATTCAAGTGTCGCACAGCTACAACGATGCTCCTAATTATGAAGAGAAAAAAACAGATGAATTAAAGGCTGAAGGTTCATTCAACC
ACCAAGCACCCAAAATGTCGATCTGCATATGCACAGAACCTCAGTACAGTTCACTGGTTGAGAAAATAGACTGCAAATGTGCAAATAATACAAATTGTTA
CTGGAAGAACAGTAAACACAATACCAGAAATCCTTCTAACGGTAACCATTGGAGGAAGCCTTGTGATCCTGGTCCAAGTTCCCATCCTCTGAAAAAGATC
AGCAGTCAATAGATGCTTCACTCGTCCTCCAGAAAAATGAGTGCCGCATGAATCCTTTCCGGTTTATAGTGAAACATTTTAGACGATGACCTTTAACATT
CTAGAAAACGAAGAGAAAACCTACAGTCATCTAATTTCAGCTTAAACATGTTCCAGCAAGATCATCACCATCCTGACATGTTCCGTTTTGTCCCTAGACC
CGAGCACAAGGTATTGCGAGGCTAATTTAGCTCAATTCAGTTGTCAGAGCAATTGTGTGTGAAAAGTGTGAACAACAGCATAGGAGACAATACAGCAAGT
GTAGCGTGCCAATATGTGTCCAGCTTGATAGTTCACTATAATAAAGTTCTTTAATTCAAGTATTTGAATAATATAGAATCTCAACCTTCAAGTTTAAAAG
GTTTTGTATTTGCTATTATAATCGTTGCCACCCCCTCACCCCTCTCAAAAAGAGAGTTACTTTGTAGCGCACTGTAAATCTCTTGATTGAAATAGTGGCA
TGTCATCTTATTTAATTCAGTTATTTCATAAGGTACAATTTGTTTAGCTGACAGCTCAGACATGCTTCTATGCAACATGTTTATACCAATGCTGTATTTT
GGCAATCCTGGATGTGGCATTACTTAACTGTGCTTTCATATTCAATAGTTCTGGCAAAGGGAGGCTGCAAGCTTGAGACAACAACTGCACAACCTGCAAG
AAAGTCATCGGTATGGCTAGAAAACCCTGCAAATTTTACAAATGTACAAGTCAGAAAATTTGCCTAATGTAAAAGGATCAAACTACAGGAATTTGTCAGCA
TGAGCACAAGTGCCTAACTTCCGAGTTAGATAACTCTGGAGGAAGGAAATCCTTCCTACTGTTTGTTTCAAGAATATACAAAAATTCAAAACAACATTAG
AATAGAACCAAAAGAAGAAACAATATTCACCATGTAGCAATACTCAATTCCTGGTTGTCTTATTTATAAAGGTATAAAACTAGGTTCTGACGAGACAATT
ATGAATCAACTACACCATGTTACAGATCATCCATAACTCTTGGTGATGCAAATTAAGGGCTGTAAAGTGACAAGTATACCTCCACCAACTAATTTGTGCA
GAGTGTATAAAATTGTAAAACAATGGATAAGAATGAATTTAGTATACAAGATAGCATACAATGCCCAAGTTTAAGGAGACATACTGATTTACTGACTCCA
TAGGACAATCTTTTGCTTATGAGGACATCAGTTTTCATAAACAATATCAATGTAAAAGGAGGTAACAGTTTATGAATTTCCTATGCCAAGCCATTTATAA
GTCAAAAAATGTACCTATTTAGCTTTTATGCATTCCAATTAACTTCCAAAGAAGAAGAGTACATACTAATGGAAAACAAATATGATATTATCATCTGTTT
TGCCCGGTAGTTTCTTGAGTCTCTGATTTGTCCGCACTTCACTCCCAACCAACACAACATTAATACACATGGGCAGAGGAATATCAGGTTATCAGGTAAA
CTGAATACAGTTTGATTGACTGCAGTCAGTTGATGGGACAAGATCTTTCTGGATTGAGCGTCAAGGAACTCCAAAATCTAGAAAATCAGCTAGAAATGAG
CCTACGTTGCATCCGGACAAAAAAGGTAAGGATGCTAGCCATATTTCTGTTTTTCGTTACCAGATTGCCTCATCCAAGAATACAACTGATGATGACGTTT
TTTACTTTCAGGACCAACTCTTGATTGATGAAATTCACGAACTGAATCGAAAGGTTCTGGAATATTCTGTTATTACTCTGCAATGAGAGGTCTCTGAGTG
GATTTCAAGAAGCTGACTCTACTGGCTCATTTTCAGGGAAGTCTCATGCAACAAGACAAGATGGAACTATACAAAAAGGTCAACCTAATCCGTCAGGAAA
ACATTGATTTATACAAGAAGGTATTTGATAAATCGTGAATGAAAATTCACAGGATATACACCCCATCCATTATTGACTGAGGTTTATTTCTCTACTCAGC
TCTACGAGAAAGAGGTGACAAGTGAAGTCAACCGAGATTCATCAACTCTAAACTTTGCAGTTGTCGAGAACGTCAACATTCCTGTTCATCTTGAACTTAA
CACTCCACCGCAAGAAAATGATGTTGAGCAAACTGCACCTCCTAAACTGGGGTAACCCTGCTTTTTTCTGCAAAGGAA
>comp86229_c0_seq14
CGGGAGCGCGGCGACGAGCTCGGAGGCGACGCCGCGGACGCCCGGGACAAGCTGGCCTCCTTCGCCAAGACGCTCACGCAGTCCGACGCCAACAACGGCG
GCGGGTTCTCGGTGCCGCGCTACTGCGCGGAGACCATCTTCCCCAAGCTGGACTACCGGGCCGACCCGCCGGTGCAGACGGTGCTCGCCAAGGACGTGCA
CGGGGAGGTGTGGAAGTTCCGGCACATTTACCGGGGCACGCCGCGCCGGCATTTGCTCACCACGGGGTGGAGCACGTTCGTGAACCAGAAGAAGCTCGTC
GCGGGGGATTCCATCGTGTTCTTGCGCACCGAGCATGGCGAACTGTGCGTTGGGATACGGCGTGCGAAGCGGGTGTCTTGTGGTGGCATGGAGTGCATGT
CAGGGTGGAACGCCCCGGGGTATGGGGGATTCTCGGCGTTCTTGAAGGAGGAGAGTAAACTGATGAAGGGTCCAGATGGGTACATGAGGGGCAGGGGGAA
GGTGAAGATTGCGGATGTCGTGGATGCGGCAGGCCTAGCGGCGAGCGGGCAGCCTTTTGAGGTGGTGTACTACCCGAGAGCTAGCACGCCGGAGTTTGTT
GTGAAAGCCGCATCAGTGCAGAATGCGATGAGGATCCAGTGGTGCCCTGGGATGAGGTTCAAGATGGCATTTGAAACGGAGGATTCATCAAGGATTAGCT
GGTTTATGGGGACAATATCTTCTGTTCAGGTTGCTGATCCAATCCGATGGCCGAATTCACCTTGGCGGCTTCTTCAGGTTTTTACTTGATCGAAAGTTTT
TCATAGTTGAATACTTATATTCCATACTGCATTATAGACTTTTGTCTTGAGTGGGTTACTACTTGCTAGTATGATGTGTCTGGTATGTACGGGTGTGTCA
CTATTCTTCAGAAGTTCTTTTCCCTTCATATGTTGGCTCAGCAACTTGCTCAGATTACATCTAGCAAATGAATTGGTACTTGGAAGAGTAGAAAAAAGAA
TATAGAAAAGAAAAACTATTGGGGATTTGTGATGATTGGTAGCAAAATAGTCATGAAGGAATGGTTTTAGTTGTATGGACCGTTATTGGTTCTTTAAACT
TAGTGTTTATCTTTTCACTAAGTTGCTGTCCTCTGCCAAATAATTCGCTTTAAAACTTTTTACTGTTCCTTTTGCAATTACAAATAGCATCCAAAATTCC
TTCCTTGCAGCCTCAGACAGTCCGACTAGCTCGGACATCACATGTTTGTAGCCTTTAGCATAGCCCACATGATGCATCCAAAAGGCATTGGGTTACTTGA
CAAACTTGCAGTTAGAAATGGGATGGCTGAGGTATGCAGACATTCTAAGGTTGGCCGAAAAATTAAAGGTCTTAATTGTGTGGACTAGGATGAGAACAAA
TTGGACATAATTCTAAGAAAAAGGAAAAAGAGTACTACTTTACCTATAGGAAACCATGCTATCTGTTGGGTTGGTTTCTTGTTTGGATCTCGTTCTCTTT
GAATTGTTAGTATGTTTCATCAGTCTCTTGTGGTTACTGAGAACTGGGATGTATCATCATAAGCTTCCGAAAGCCCATTGGTCACCATTATCACCGTTGA
GTAGCCATTGACAGATTCAGGTTTGAGCATCCTTTGGCTGCTTAGCTTTCTGTCTCTACACACTTGAAAAGCTGGTAAGCGTCATTAATGTGACATCTTC
CCTAACTAATACTCACAAGAGGGAGGGAGGGAGATGAACTTGTATTGGTACAGAACTTTCAAAGCTTGCTATTGACTTCGCGAAAAGAGACAGCTGCTGA

ACAAGCAAAAAGGATGACCACCGATTTGGCACAACTACTCGAGCACTCCCATAGCACCAATAAAATTTTCCTACCATCAGACTACCTTTGCTTCTCACTA
TCCTGGTTTAATAATTTCATCACATAAGAGCCCAATGAATCAATTATTGATTTTATTTTGTTAGCCTCAGGAGGCATTATAAACACATTGAATTTAGGGC
TCAATACCAGTTTTGCTTTTTTAAATACATTAATTGATCTCTCTCTTAAAGCCCAAATTTCTCTATGTTTTGTAGCCAAATTAAAGCCTACTGATAGACT
GATATTACATAGTTATAGCTCAAATGTGAGGAGTGTACTCTTAAAACGTTCGTGGACAACAACCAACATAATTTTTAAACTGTGGTTCTGTTGGTCCTGA
ACTCCTGAAGTAGTCGAACCTTTTCCCACTTTGATGGCAGTACCTTACACTTGTCTTTATGATCAACCCCCTGATGTGATTGCTAGCGGATCCTTAAAAG
CAGCCTATAGCTAGCATTGTACATTTGTACCGTGGGAGAGAGGTATTGAACTAGCAGTAATAAGACTGATCTAGGATTAATTTAACCTTGTTTTGCTAGC
TCTATTGTCAAATGTACCCTAGGACATTTTGGCTCTTTATCTCTCTCTTCACCAATAATATATATTGATTGGTATTCTTGGAAGTGCCTCGGTATATACT
TTTGTCTGGCAGTGTAGTAATACGAAGGGTGGATTCTTACCACTTACATGTGTTAGGTGTGATATTGTAAACTCATCAGCTCATAGTAGCTACGATGTCA
TCATTGTAGCCGATATAGAGTCATCAGTTACTTGTGAAATCGATATAATAAATTGTCTGTAATTGCGAGCTTCCTTGCTTCATTGTTTGTCCTTTACCTC
TTTTCTCTTTCATATAGTTGCAAATTTCTTACAGGTGACATGGGATGAACCAGATTTGTTGCGGAATGTGAAATGTATCAGCCCATGGCTTGTGGAACTC
GTATCAATCATTCCCCCAATTCACCTGGGACCATTTTCTCCACCTAGAAAGAAGTTGCGGGTGCCCCAACATCCTGACTTTCCATTTGATGGTCAGCTGT
TGAATCCAATTTTCCACGGCAACCCACTTGATCCTAGCAACAGCCCCCTATGCTGTTTCCCGGACAACGCTCCTGCAGGCATACAGGGAGCCAGGCATGC
GCAATTCGGTCCATCCTTCTCGGATCTCCACATCGGCAACCTGCAGCAAAGCCTGCTGTTCTCCGGCATCCTCTTCCCTGACCACCACGCCCCTCACCCT
CCGAGGATCAGCACCGACCTGACAATTGGCAGCCCACACGCTGGCCAAGACGCGGCGTGCTCCCTATCGTCTGGCGCCAAGAAGCCTGACGACGTCAAGG
CGACGAGACTAGTGCTCTTTGGACGGGCGATACTGACGGAGGAGCAGATCAAGGCCAACGCTTCCGTTGGCCCGTCCTCGCTGGGAGCCACCGGGAACGG
CTCGTCCAAATCCGACTGCGACGCCGAGAAAGCACCTACCAACACGTCCGAAGGCTCCAGCTCAGGTGTCATCCAGGGCAGTCCTACCAAAAACAGCTCG
TCCTCCTGGAGACTGCAGTGGTTTGGCGACAATGGCAGTCAGTCACCATCTGAGTTCGGGCTGGAACCTGGCCAGTGCAAGGTGTTCGTGGAGTCGGACG
CCGTCGGCCGGAACCTTGATCTCTCGGCGCTGGGCTCGTTCGAGGAGCTGTACACCCGCCTGTCTGACATGTTTTGCGTTGACAACGCTGAGCTGAGGAG
CCATATGCTATACCGCACCGCCGCTGGCGAGGTCAAGCACGTCGGCGATGAGCCTTTCAGTGTGTTTGTGAAATCGGCGCGGAGGATCATAATACTGACA
GATGCGGGCAGCGACAACATAGGGAGTTAGGAACGAAGCGATCAGCCAAATAACTTAGGCACTACTCCTAGGCAGTAATAAGCCGCCTACATGCATGTAC
AGCCGAACGAGATGCGGTTTCTTCTTCAATCAGGCCTAGAGAGTTTAGTCGGGAAATTAAGCTGACGATGTGTTCATTGCTTCAGTTGAGTTCTTTTAGT
CATGCGAACAAAATGTAATCTTGATAATCAAACGCACTTGTTAATGTTGTTTCCGTTTTGGATTTCAAACCTTGAGCCGGGCCACAGAGACAAGAGCAGC
AGGTGAAAAGGGTTAAATGCCATTGCTGGCTGTTGGAACATTTACTAGTCACCCACTGAAACTTCTGAATCTGGGGTTACTTGATCAGACTCCCAGCTCC
AGGCATTTCATTTGTTCCCTTGTTTGTCTGGGCAGGCAGATACTGTGCGCTGTGTTGCGTATCCCTGTTACCTCTGATGCATTGATGCCTCCAGGGCCCT
GTCCTTTGCCTGCATAGGGGAAATAATTGTGCTGCTTGCCCATTTCGCCTCAGAATATTGTTGCATGAAATGGACATCTCAGCTAACACAACCAGAGGAA
ACAGTGTCCGCAGTAGTGGCAGTGCT
>Locus_10929_Transcript_65/78_Confidence_0.320_Length_1838
GAGCAGGGATTGCGGGAAGCAAGTCTACTTAGGTAAGTGATTGAGTCCTATACGTTCCAGCTCGAGCTTCTATTAACTGCTGATCAGTTTCCGCCTGTTT
GATCTGGTTTACGATGTGCTGATGCTGCTTCTATTCAGGTGGTTTTGACACTGCGCACGCGGCTGCGAGGGCCTACGATCGTGCTGCGATCAAGTTCCGA
GGGCTCGACGCGGACATCAACTTCACTCTGAACGATTACGAGGATGATTTGAAGCAGATGAGGAACTGGACCAAGGAGGAGTTCGTGCACATCCTCCGCC
GCCAGAGCACGGGGTTCGCGAGGGGGAGCTCCAAGTACCGCGGCGTGACGCTGCACAAGTGCGGCCGCTGGGAGGCGAGGATGGGTCAACTTCTTGGCAA
GAAGTACATCTATCTTGGACTCTTTGACAGCGAAGTTGAAGCTGCAAGAGCATATGACAGGGCAGCCCTTCGCTTCAATGGGGGGGAAGCTGTTACTAAT
TTTGGGCCTAGCTCCTACAATGGAGGAGATGCTCTACCCGACACCGAAAATGAGGCTGTTGTTGATGGCGATGCGGTTGATTTGGATTTGCTTTCACAAC
CTAATGTACACGACGCTAAAAGGGATAATACCTTAGCTGGCTTCCAGCTAACATGCGACTCCCCTGAATCTTCAAATATGGTGGCCTCTCATGTAAACTT
TCTTCACGATTACACCCCATTTAAATGCTCGGTTCCATTTTTTATGACTTACTACTGTTAGTAGTAGCTAAAAAGGTACCCGGAAAAATAAAATCTTAAG
ATGGAAGCAATCTAGGTTGAATTGTCTTGTATCTCTGTGCGGAATCCTTGACCTTTGTTAGTTCCTAAGCTATAAAGATTCATAGGAGCATTTGATTGGC
GTGTCGTTGGTTGAGATATTAACTATTAGAGTGTCCCCAATGAGAGTTAGCTTATACTAGCTCTAAACATATTCATGTTATTTCATATATTGCACTAAAG
TTTAGATTTTAGTAACTATCTTTTAGCTTTTTTTTAAATTTAATACTTACGTGAGTATATACATCTCGTTTAGTGCTATAGTTTAACTCTTCACTAAGAG
TTGCTAAGTTTTTTATATTTAATCTCCTTTACATAAAGTAAATGATGACATAGACATGTTTAAAGCTAGCTAAGGAGGCTTCTTCGGGGCTCTTAGTTCA
GGTCTTATAACCTTTATTGAACTTACGACATTTGCACCTATCTGTTGCTGCTGTAGCCAATGAGTTCATCGTCCCCGTGGCCTGTGTATCAACAGAGCAC
GGCAGTACCACCTCACCATCAGCGTTTGTACTCATCTGCTTGTCCTGGCTTCTTTCCGAACCTCCAGGAAAGGCCAGTGGAGCGAAGGCCTGATTTGGGC
CCCCAGTCGTTCCCCACCTGGGGACGGCAAACGCAGGGCTCCCCTCACATGCCGTTGCACCACTCCGCAGCATCATCAGGATTCTCTACCGCCGCGAACG
CCGGCGTCCCATTGCCGTCTCACCCGCCGGCGCCGTTCTCGGACCACCAGTTCTACCTCCCACCGACGGCCTGAGCTTGTTGGTCCGGTTCAACTCTTGG
GACAGCAGCTGACGCTCTGTGTGTGAACGTCTCGCAGCTTGCCGGTAACAGCTGTTAATTAATGGGAGAGAGAGAGAGAGAGAGAGAGAGAGAGAGAAGA
GAGAGAGCCAGAAGACCCAGTATGGCTGACTCTGTGGCCTGTGCCTTGTTATGATCTGAGTTATTGCTACTACTACTACACCGTGGATTCATCATGGTTT
GAGATTCTTTACCCAAGTTTCAACTTGAAAGTGCAAAT
>Locus_3441_Transcript_37/417_Confidence_0.040_Length_1652
TGTAAATCTCTTGATTGAAATAGTGGCATGTCATCTTATTTAATTCAGTTATTTCATAAGGTACAATTTGTTTAGCTGACAGCTCAGACATGCTTCTATG
CAACATGTTTATACCAATGCTGTATTTTGGCAATCCTGGATGTGGCATTACTTAACTGTGCTTTCATATTCAATAGTTCTGGCAAAGGGAGGCTGCAAGC
TTGAGACAACAACTGCACAACCTGCAAGAAAGTCATCGGTATGGCTAGAAAACCCTGCAAATTTTACAAATGTACAAGTCAGAAAATTTGCCTAATGTAA
AAGGATCAAACTACAGGATTTGTCAGCATGAGCACAAGTGCCTAACTTCCGAGTTAGATAACTCTGGAGGAAGGAAATCCTTCCTACTGTTTGTTTCAAG
AATATACAAAAATTCAAAACAACATTAGAATAGAACCAAAAGAAGAAACAATATTCACCATGTAGCAATACTCAATTCCTGGTTGTCTTATTTATAAAGG
TATAAAACTAGGTTCTGACGAGACAATTATGAATCAACTACACCATGTTACAGATCATCCATAACTCTTGGTGATGCAAATTAAGGGCTGTAAAGTGACA
AGTATACCTCCACCAACTAATTTGTGCAGAGTGTATAAAATTGTAAAACAATGGATAAGAATGAATTTAGTATACAAGATAGCATACAATGCCCAAGTTT
AAGGAGACATACTGATTTACTGACTCCATAGGACAATCTTTTGCTTATGAGGACATCAGTTTTCATAAACAATATCAATGTAAAAGGAGGTAACAGTTTA
TGAATTTCCTATGCCAAGCCATTTATAAGTCAAAAAATGTACCTATTTAGCTTTTATGCATTCCAATTAACTTCCAAAGAAGAAGAGTACATACTAATGG
AAAACAAATATGATATTATCATCTGTTTTGCCCGGTAGTTTCTTGAGTCTCTGATTTGTCCGCACTTCACTCCCAACCAACACAACATTAATACACATGG
GCAGAGGAATATCAGGTTATCAGGTAAACTGAATACAGTTTGATTGACTGCAGGCAGTTGATGGGACAAGATCTTTCTGGATTGGGCGTCAAGGAACTCC
AAAATCTAGAAAATCAGCTAGAAATGAGCCTACGTTGCATCCGGACAAAAAAGGACCAACTCTTGATTGATGAAATTCACGAACTGAATCGAAAGGTTCT
GGAATATTCTGTTATTACTCTGCAATGAGAGGTCTCTGAGTGGATTTCAAGAAGCTGACTCTACTGGCTCATTTTCAGGGAAGTCTCATGCAACAAGACA
AGATGGAACTATACAAAAAGGTCAACCTAATCCGTCAGGAAAACATTGATTTATACAAGAAGCTCTACGAGAAAGAGGTGACAAGTGAAGTCAACCGAGA
TTCATCAACTCTAAACTTTGCAGTTGTCGAGAACGTCAACATTCCTGTTCATCTTGAACTTAACACTCCACCGCAAGAAAATGATGTTGAGCAAACTGCA
CCTCCTAAACTGGGGTAACCCTGCTTTTTTCTGCAAAGGAACTATTGATTCTGTTCTTAATTCTTTAGTGGCATATTGTTTACTACTAACTGAAAACTTG
CAGATTACAACTAAATCCATGAAGGCATGCAGCATGGCACCGCTTATGTTCA
>Locus_5983_Transcript_75/82_Confidence_0.149_Length_1612
TCTGTTCCAAATGCAATCGGAAACCCCAGGAATGGAGCTACGAGGCATGGATCTGTGAATTCATCTGGTGGCACTCTCACTTCTAGCTCAGAGCTAGGGC
ATGGTTCATCCAAAAGCTCCATATCGGCATCCATTGATTCGCCATCCAAAGTAGGGAACAACTTAGAGTTCGACTTTGCTGCTGTCGAGAGGCATGGTAA
GAACATGGGTAGTGATGGTAGTAGAGTTGAGGACTCAGGAACTTCTCCATCGTCAGCGTTCAACCATGGAGAGCCATTAATCAGCCTGAAGCTTGGAAAA
AGGGCTTACTTTGAAAATGTTTGCGGAGGACAGGATATCAAGAGCTCTGCACCTTCAACAGTGACTTCTCCATCAATGGTTGTCAAGAAGACCAAGATGT
CTCAGCAGAACGCACAAAGCTCAAACTGTCAGGTTGAAGGTTGCAGAGTTGATCTGTCTTCTGCTAAAGAATACCATCGCAAGCACAAAGTCTGTGAAGC
TCATTCTAAAGCTCCCAAGGTTGTTGTTGCTGGTCTAGAGCGCCGTTTTTGCCAGCAGTGTAGCCGGTTTCATGGTTTAGCTGAGTTTGACCAGAACAAA
CGAAGCTGCCGTAGGCGTCTTACTCATCATAATGCACGGAGGAGAAAACCACAGGCAGATACAATTTCATTCAGTTCATCGCGGCTCTCGACAATGTTTT
ATGATACAAACCAGCAGAAAAATCTTTTCTGTAGTCAACCTCATTTTGGCCAAGCGAGAAGCAATGCAGTTTTTTCTTGGGAAAACTCGGGAGATTCCAA
ATTTATGGAAACCAAACATCTGTTGATGCAGCCAACGAAAATGTTAGGTCTTGATGGGCTGCATTTCTCAACCCCCCAGATATCAAATAGTGTTGTGGCT
CACTCTGTACATCATCGTAATTTCGATGGGCTCATGCCATTCAAGGGAACCAACACAAAGGTCCTCAACCAAGGCGTGGAAGCTTCTGCGATCGCTTCCA
ACTCGAGTGGAGACCCGGATCTTGGGTGTGCTCTCTCTCTTCTGTCAAACGGTTCGTGGTGTTCAGGTTCAACTGTCATCCAGCAGCCTAGTTCTCGTGT
ACACGCTGGTGCGGTGCCGCCCCTCGCTACTTTTGTGGTCTCTAATCCTGCAATGCATCCTCTGGACCCAGCCCCAGGAGGGTTCTGGCAAGACGACCCT
GCCTCGCTCGATAGAACTCCGGAGATTCAGGCATTCGGACACCTCTAACTATGGTCAAACAAATGTACTCATGCTGCATGCCCTCGGAACAGCTGATTTA

GTCACCTCATCATAATCTGCAACTCTGCTTTGCCAGTGGCAGATCTCTGCATAATGTCAGTCCTATCATCGGAGGCATTTGGTGAGGTGAAGACTGAAGA
ATAATATAGGAGGCAACCCCCTGATCTTCCTGATTTAGGATGCATCTAGGATCCAGTTGTGTGTGTGCTTGTGGTTTTGCCAGAACCGGTTTTTTGTTTG
CCGTCCCGTTAGAGAGAAGGGATCAAGTACATTCATGACTGCTCATGAGATTACTTGTTTCACTAATATTACATGATCTGGAGCGAGATTTCGCTCGGTT
CACGTTGATGGT
>Locus_9469_Transcript_54/63_Confidence_0.151_Length_1135
CAGTGCAATTAGACTAAGTATAGTTGGTGAGAAGACAGGAGTAGAGATAAGGGCCAACAGCCCAACAGAAAAACCAAGCAAGACTGCAACTGCAAGAGAG
ATGGGGAGAGAGAGGAAACCTTGCAGAGGAGAGAAGCAAGGTGGGAAAGAGGCAGCAAGCGAGGCGGGCTGCAGGAACGGGAACATGCTCCCTCCTCATC
TCACAGAAAATGGCACAATAATGATTCAATTTGGTCATCAAATGCCTGATTGCGACTCATCAACTACCCAATCAACCAGCGAGAGTCATCAAGAAGTGTC
TGGAATGAGTGAAGGAAGCCTCAACGAGCATAACGATCGATCAGGTAATCTCGATGGTTACACAAAGAGTGATGAAAATAAGATGATGTCAGCTTTATCT
CTGGGCAACCCGGAAACTGCTTATGCACATCCAAAGCCTGACCGTAGTCAGTCATTTGCAATATCATACACGTATGCTGATTCATTCTATGGTGGTGCGG
TAGCAGCTTATGGCTCGCATTCTATTATGCACCCCCAGATCGTGGGCATGATGTCGTCCTCTCGAGTGCCATTACCAATTGAACCAGCTGTCGTGAAGAGCC
CATATATGTAAATGCAAAGCAGTACCATGCCATTCTTCGAAGGAGACAGCTCCGTGCAAAGTTAGAGGCTGAAAACAAGCTGGTGAAGAGTCGCAAGCCA
TACCTTCACGAGTCTCGGCATCAGCATGCCATGAAGAGAGCTCGGGGAACAGGCGGGCGGTTTCTCAACACGAAGCAGCAGTCAGAGGCTGCTGGCGGCG
GCACCTCGGACGCGCAACGCATACCAGCAAATGGTGGCCTGTTCAGCTTACCTCCCAGTGATCTCCACTATCGCGCGAGAGGGGGCGCTTAAGGAGTCCC
ATGGCAACTCATCCTTGGCTTAAAAAATGTGTGGCGCTCAGCAACTCGACCTCTGCTAGTTCCATGTAAAGGTGTGTGACTGTAAATGCTGGTGCTTGAA
TTGGCAGCAGCCATTCCTGCAACGCGTCGCAGCTGTTCCCGGTTCTGTGATCATGCTAGACTTGTGACCGACCGGCACGAAACTGGCACCTGTTTCCTGT
AACTGTTTTCCAAGTTGATGATGATGACTACTCCA
>Locus_4567_Transcript_60/65_Confidence_0.400_Length_2601
TGCTCTACTTCTTTCTCAGCGCACAACTTTGTGTGTTTAAATGCTCGTAGTACTTACAACAGGTGCGTTGGTTGCTACTTTTAACCGGAACAAGTAGCAA
TCATGCACCTACAGTAAACAAACTTGTCCAAATTGATTCTTCGTTGTCCAAAATCCGTGAATTGGACGCACGTCTGTAAAGGTACAAACAATAAATAACA
GACCATATAAAGCTATTTAAAAGCCTAAATCCTCAAATTCATGAAAAACCGAAACTCAAAATTCTTCGATGAATTTCATCACGATGCTCTTATCTACCGG
TCACCCTTGCTTGCGGCTTTGAATGCACCAAGGTTCCTTTTGAGAGACTTCACAGCAGACAAAATCTGCAGCTTCTCTTTGGATGCCCCATCAGGTTTCT
GATCATCGTTCTTACTGGCTGCCTCATCAGGTGGCAACCTACCTCCCGCAGACGATCCCTCCACAGGAAGTTCCCTGTCTGCATTGCTGGCATGTGCCCC
CTGCTTCCCAGCTTGCTTTCGCATGAAGGGCGCACTCCTCTTGTACTGGGGATCAGTAGGATCCAATGCGAACAAATGTGACGTGAACATTGCTGAGAAC
CTCTCGTCCTTGCTAAGATCGATATCAGGAAGTTTGTCCTCAACCGAGTCCTCTTTATCCTTCTTGCCCTTTTTACCCTTTTTGCGCTTCAAATTATAAC
CCTTTGCAACATTGGCAGTATCCTGGTCAGCAGCAACCAAGAGCTCCAACTCTTCTTTCGTTGCTTCTGGCTCCAAATGTTCCTCTGGTGATTTGTCCTT
TCCTTTCCCCTTTGCTTTATCTTTATTTTTCGCCTTTTGTTTCTTGATTTTGCTTGGTTTAGTTTCTTCATCAGACAGCTCCTCGGCGAAGAAATCATCG
TCGTCGTCAGGCTCATCTTTGCTGCTATAGTCATCATCATCATCATCCTTCAACCCCCTCTTCCTAGCCTTCCTTTTCTCTTTCATTTTCTTCTTGGTGCC
TCTCCCAGACAGTTTTCTCCTCATTGGTCTTTCTCTCTAGGATACGTTTACTGAGATCCTCAAGCTCTGTGTTGAATGTAATCTCCATATCCTGGCCACC
AGCTTGTTCCTCTTCAGATTTGTCGCCTTCCAGTAAAAGAGCCAACCTCTCCTCTTTTGTTAGCTTTCGTTTGGATCCCCCATTTGGTAGAGACTCGTCA
TCAGAATTGTCGACACCGTCATCATCTGACGCACTGTCATCACTTGCTAAATACATGTCAAGCTCATCTAACTGATCGTCAGTGAACTTTCGCCTCAAGA
CCTTCTTACGTTCTGGTTCATCATCATCCCAAGTTAGCTTAACTCTGCTATGTTGCAGAGCACGAGTCTCAAAATCAGGCTCCTTGTAACTCAGAGGTGC
CTCTGTAGCAACGTCTCGAGCAGGATGTTTGAACTCCATAGAGTCAGGAATAAACTGCAAATCAAATACATTTGCTGTTTTCAAGAACTCAGTACCATCA
AGATTCATGTACAGGTGATTTGCAGTTGCACTAGAATCACACACCACAACTGCATAGTAATACCTCAGTCTGTTTAGCTCGTAAGTACGCAGCTTGTTAT
TCTCTTTGTCAGAGTCAACTTCTTCTTCGTCGTCATCAGTGTTGAATTCATCATCATCAGTGTCGTCTTCACCATCGTCGTTATCATCATCATCGTTGTC
CTCATCATTATCATCAGTATTGGCATTAACAAGAGCTGCTGGGCCCTGAGTTGATTCAATTTCCATGCACTTCAGTCCAAATTCTGATGGATATATTGAG
ACTGATAAAACCCGCCCACCTTTTGGGACGCAAGATGTCATCACCATATACAGGTCAACTGCCTTGATATGATCCCAGTCCATATTAACAGCAGCAAGCC
TGTGGGTTTCCTTGTCAATCATAGGTGTATCATCGTGCCGGCCCATTAAGTAATGAGCAATGTCACTGCCGACAGAGTACTCGTCATCATCAACGTCTTC
ATCCTCGTCGTCGTCGCTACTAGAACTCTCTTCTTCCTCCTGCTCATCCTCTTCATCCACGTCCTCGTCCTCCTCTTCTTCAACTAACTTCTCTTTCTCC
TTCTTTGCCTTCTCTTTTCCCTCATCTTCCTCCTCGTCTTGGTTGAGATAGTAATGGAGCATGGGGTTCTCCTTGCCGCTCTTCTTTCGTCGCCTGCCGC
GCTTGTCCACCGGCGCCGCCGTCGAGGCGAAACTCGGGTCCGTCATCATGCTGCTGAACCTCGCGTCCAGCGCCACCTTGGCCTCCTTCCGCCGCATCGG
CCGGAACCTCGGGTCCGTCCGCGCCGCCGCGAACCGCTCGTCCTCCATTGCCCGCCTCATCTTCTCGTCCCTCCCCTCCACCTCCTCCGCTCTCACCGTC
TTGCCCTCAGGCTTCCCCTCCTTCTTGGACTTCTTGCCGTCCCGCTTCTCCTCGCGCCCCTCCTTGTGCTTCCGCTTCTTGCGCTCGGCCTCGGGGCGAG
GGGCTTCCTCGGTGGCCGCCTGACGCTCTTGATTCTTCTTCTTGTGCTTCCTCTCTTCCTTATCGGACCTCGATTTCTTGGCCTTCTTGAGGCGCGCGGG
C
>Locus_21273_Transcript_19/23_Confidence_0.458_Length_2528
TCCAATTATATCATTCGTTCTCGAGCTTAGCAGCATCTAAAATCTCGATACAAATTAGTCCTCTCTATCCCAAACAACCCTTCTAAATAAACCACAAACA
ACTCATGTAGCCTTGCTACGGCAAGCCAGTAACTTTGCTTCGGGGGTCCGTACCATTTACACTTTCTTCTTCCTAATTTTTGCTCTGCCCCCACCAGAGG
AAGGGACGGCGGGGAGGGGGCAGGCACAGAGAGCAGCTTTGCTTGGCGGACGCACCGAGGGAGGCGCCTGGGAGCCCTGCTTCTCCCCTCTTCGTCTTCC
TCCTCCGCTTCCAAAGGTGACTCCTTCGGGAAAATGGTTAATGATCATACGAGGTCAAATTTGGTTTTTGATAATAAGCAATCTCTATTTGCAAGTCATA
ACATTGACTACGGCCAGCCAATAGCTTGTATATCATACCCATACAATGATTCGGGCTCAGGAGGTGTTTGGGCAGCCCTTTGGGTCACGCACTAGCGCTGC
AGCTGTGGTATATCCTTCTAATAAACTGTTGTACTAAAATCTCTCAAGACCTTTGAACTTCAGTGCTGATGTCGCATTTCAGCATTTAAAATTTTACATG
AGAATGTTAAACATATATGATCCTAGTTTGGGGAATCTCCTAGTTTTCAGTTCATTCTGATTGGGCTACAGTATGCTGTCTGAACGGTCCATCTTACCAT
GGTCTAATCAAATGTAGAAACTTAAGTAGGCTAGTCGCCTAATACCTTAACTATGTTTATTTCAAGCAGACACTGCTTAATATGATCCTTGGTTCTTTGC
ATAAGCATGCATGGGCCATGACAAAAGGTCTTTTTTATCACTGCTATAACCGATATACTAATGGCAACAAAATGCTCAAAAACTACCACATCTTTTTGCA
GCATATTTAGCCATAACCATAATGGGAAACCGTGTGCTAAGCTAGTTGCAACTGCTTCTATTCTCCAGCGACAAATGATAATATCTAGAAAGCTAAGATC
GCAGCACTTAATTGTGGCCTATTTGCACAGGTTTGAACCAGATTTTGCATTTCTTTCTCATGCACATACACTGTTACGTAAATCACTCTATTATTTAGAA
GCAACAAAAGGACCGTACATTAATTAATTCTCTAGCTGGTTATAACAGAAGCTAAACTAGCTGGATTTTGGCAGAGTTGATCTTAGTATTAGGGTTAAAA
GTGAAAACAATACTTGACTGGATGCAGCAACAGTGCTTTGTTAATTCTCTGCTCCAGTAATGTTTTCAGAACTGCATCGTTGTTTGTGTGTCCAAATTGT
GCTTTCTCAGTTCATGCACGACTCCATATATATCTATCTCAGTCTGCAGTTACTAGCGTGCTGGCAGCTCATAGACACATAGTGTCCGTTTAGATGATTT
CGTTTAGATGATTGCTGCTGTTTGTAGTTGTATAAATTGACTCATGATATCAACCAAGGTTACACATAATTGTTGCCAGGATTCCATTATATGCTAACGT
TAGCTCACATATAAGGCAGGGCCATCCATCCACCGTACAGCTTGTTTATCTGTAAGTTCATCACTTTTGAGGGTTTTGTCTAATCAATTGTACTTAAAGC
AGTTCCATCCCCAAATTGCTTGTGGGGGCACATCTGCGAGAGTTCCCTTACCTCTGGAATTAGCAGATGATGAGCCCATATATGTCAATCCCAAACAATA
TCATGGTATACTTCGCAGAAGACAGCTACGTGCTAAGTTAGAGGCTCAGAACAAGCTAGTCAAAAACCGAAAGCCTTACCTTCATGAGTCTCGGCATCTT
CATGCAATGAAGAGAGCAAGAGGTTCTGGCGGACGTTTCCTTAATACTAAACAGCTCCAGGAGCAGCAGCAGCTGAAGTCTCGCAATGCCTCCACCAGGT
CCACCACAAATGGCGCAAATTCCTCAGGTTCAACACATCTACGGCTTGGTGGTGGTGCAGATGGAGATCAAACCATGTCGGGGACGAAAACAATGGCCTC
ACAAAACAATAGCAAAAAGGCTGTTTCTTCTTCTGCAGCTCTTGCTTTCACCGTGACTCCTATGGTGCGCAAAGATGACACCTTCTTCCAGCACCTCAGC
CACAATGTCAGCTTCTCCAGCCATTTTGGCCAGGCAAGCGCCCAAACCGGCGTTGGAGGCATCCATAATGAGACCCAGCATAGGGTTTCCGTGATACAAT
GACGGTTTGCGAAGCTTATCTGGTGATCCAGGCTTCTAGGTGTCCTCGTGTCCGGTGTAGTCTTGGTCGCTCAGGCAATTCATCCTTGGCTTAGTTTTTG
GTGTGTTAGAACCTACAAATGTTTCATACTCTGTTGTGGTTTGCAGAGCCCATAAATCAGGGCTCTCCTAAAAAAAAATCAGGGCTTGATGGCGACACTAC
TATCCAAGTATTGTTGTAATGGTGTGTGTGTTAGAAACTCGCAAAAAAACCCTTCTTTGTGCTCGTATTAGTACATTATGCTGCACAATTAGCTGCTATTCTG
TTCGTACTACAGTCTGGTATGCAGTGGT
>Locus_39694_Transcript_1/7_Confidence_0.750_Length_2302
TTCTTGTTTTACTGATAGCTGTGGCAAAGGGAGGCAGCAAGCTTGAGGCAACAACTGCACAACTTGCAAGAAAGCCACAAGTATGATCTTCCAATTGATT
CTGCAATGAAAATACATCTCATCAATACAAAGTTTAGATGCCACGTGGAAAATTTTGTAATAACTGTAAAACCAAAATGAAACGTATCATCCAGTGAAGA
TGAGGGAGAAAAAAATCTAGTTACTGAAAGACATTTATAGTGTTCACTATTCAGAAAGGAAATTGAAAGTGGCTTTGTCACAGCAACATATATGTGGTTT
TGCTTCGAACCGTGTACCCACGTAAATTGATATTGGTTTAGTTTCAATCTACACAAGATAATCTGACGAACTCTTTTGCATTGTTGTAGGCAACTAATGG
GAGAGGAGCTTTCAGGCCTAAGTGTGAGAGACCTCCAAGGTTTAGAGAATCGTCTTGAAATGAGCCTACTTAGTGTCAGAATGAGGAAGGTCATATTCAT

TTTATTTCCACATTCTGTTTTTCTGTTCACTTCAATCGAATATGCAGTAAACATCTATCAAACAGGGCCACCTTTTGACAAGTGAAATTGAAGAGTTACA
TAGGAAGGTTAAGCCCTACAATGAATGCCCTTTTATTATTTCAAACAAATGATCATGCATGGAAGTTGCTGACATCTATACTGATGATGAAACAGGGCAG
CCTAATTCATCAGGAAAACACGGAACTCTGTAGAAGAGTGAATATCATGTCACAACAAAAAATGGAACTTCATAGAAAGGTTTGATGAACTATCTTTTGC
AGAAACTTCAACCAAACGAAGATGCATTGTCAGCAGAAAGCTAAAATGACTTAATTTCAGCTCCTGGCCAGTGAACCAGGAGGTGTTGCTGATGCAAATA
AAAGCTCTAGCACTCCCTACGGCTTTGGTACAGCACAAGGCGCAGATGTCCCAGCTAATCTTGAACTGAGCCAGTCATCACAAAAAGAGGGAGAGCAATG
CAAAACAGGGGCCCCGGAACTGGGGTAAATATTCTTCTTTAGGATCCAAAATTCCCATTACTCCAAGAAGTATGAGGAAGCTTAATGAGAACTCTATGCT
TTAATATGTAGCAGACTTCAGCTGCACTAAGGAGAAGCAGTACAAGCTGGACAATGCCATAATGGTCCACAAATTTCACCATGCGATGCAAGAGCATTTT
GGTTGCAATGACTATTTTGTGCAATATACTAGCTGGGTAAACAAATGGGTTGTGCAAAGACTACAGATAGGACCAGTTATGTTATCTGAGGCTCTGCTGT
GTCTAAAATGCAACATATTCGTTATTGTAATGACACATGATAAACTGACTGTCCTACCGTTGACCTTGAAGATAGCTCTGCAGTGAAAAGGTGTATTATG
GGCTTGCCAATATTAAAGAAGTGTTAGGGATCAACTAGTAGGATCTCACAAAGAAGTAATTAGTCCACATTAGTGCCAGCTGCTGGGGATGATATCTGAC
TCAGCCTGCCAACCCTCTCTTAGCCATGGTGTTTCAACTAGTATTAGAAAAGAATGTTCATCCAATTGAGTTCCCAAACAACGAAATTTATATTATGTGT
TGCAAAGTGTCGACACCAGTGGAACAGAGTCCATATATGTCAAGCAAATGCCAAATGTCAATTTTGCTCCAGTGCACGTGGATTCTGTTCTATTCTCAAG
AATTTCAACCCTTGATAAGGCCCTTTTTTCCTGGTGAAAGGATTAACAACGATGAAGCTTCGAGATGGTTTAGTGATTGTTAATAGAGGTACTTAGTGCA
TCCATGCTTGAAAGTGTTCTTCCTTTCAGCGAAAGGTTTTCTATTCTACTTGCTTGTTATCTTTTGGCCAGTATGTCAAGCAAGAATTTAATCTGCTAAT
CAAGAATGTAATGCGTTCTTTTAACATGAATATCAACAGTTTTCAAGTAACTGAAAACAGTAATGTAGTATTCTCTCGCCGAAAATAAAGCTGTGGTTCC
TTGATAGCAACCGGCAGTTATGCCAATACCACATTAAAGTGGTTGTGTCCCTTATAATACTCTCTCCATTCTACTTAATTCAATGCTATTTGTTTTTTAA
CGCATGTCAAGATCTGTCTTTGACCATTCCTTTAATCCTTTTGAATATATATTTTTTTAAAAAGTGGTGATATAGCTTTCGTAGGGCCAATATAAGATTT
TTTAAGAACATATTAGAGTTGGTCGTGTTTAGGTAGAGCTGTGTGAGGGTATGTGGTGATGCTAATCTGTAACAAGACGGTCTAAAGATACCGCCTTGCG
CC
>Locus_5417_Transcript_24/28_Confidence_0.375_Length_4726
ATTACAAGACTATTTGATGCAACAATCACGCATATGGCTGCGCTAGAACTACTGCTCTGAATTAAGAGGCGTTGAAATATTGTCCGGTCTACATATAGGC
ACTTGAGGTGTATTGACAGACAGTTCCCCTTAGTTTGCTACAACACATCAGGCGAATTCCACCAAATTAGGATGATGTTTGTTTCTTAGACACAGGATAG
CAATGCCAAAAGTTTTTTATAATTTTCCGCTTCACCGCAGATTTGGCATCTCAAAGGGAGTTGTGGCAAGATTCTCTTGACCAAGCTTTCTTGCAGATTT
GGCAAGAAAGATAAAAAAAATGTTGGTATACTTAAGCATCCTTAATAGAAACTAAACAGGTCTTAGATTGAAATTATCATAAATAAACCCTTTTTCACTT
TAGCTGTAGTCCGGATGAAATCTTAGGGGCCTTGCTTAGCCTGCCCTGAAGCTTGCCAATAAGTCGAAGCTGATGCTGACACAGCACTGAAATAGATCCC
AATGAACACAAGTTATTTCGAGAAGATAAAATACCAAGCAAAGCCAGGATTGCATTAAAAGATAGTGTATTTCTACTATACGATCTGAAAGCTTAATTTA
TTATATCAATAATCAAATTGGCAGCAGTGTGGAACAGATATAAAGAAGATGCTTCAAATCACAAATAACTACAGATAAATTGAAGGGTGAAGGTTCATTC
AACCACCAAACACCCAAAATGTCGCGCTGCATATACACAGAACCTAAGTACGGTACAGTTCATAGTTTGGGAAAATAGACTGCAAATGTGCACAGATAAT
ACAGATTGTTACTCGATGAACAGTAAACACGGTATTCGAAATCCTTCTAACCATCCATCCAGGCAGTGCCTCGTATACTCAAACCTCTTTTTGGAGGAAG
CCAAACCATGTGATCCTTTTCAACCCAAATACATATATTCAAGATGAAACTAACAAAAAGACAGTTGATAAAGAAACACGGAAGAGAGTAGAAAAAGGGT
ACCTGTTTCAAGTTCCCATCTTCTGAAAATGATCAGCAGTCAATAGATGTTTTAGTCCATTCTCCAGAAAAAATGAGTGCCGCATGAATCCTTTCCGGTTT
ATAGTGAAACATTTTAGACGATGACCTTTAACATTCTAGAAAACGAAGAGAAAACCTAAAGTCACCTAATTTCAGCTTAAACATGTTCCAGCAAGATCAT
CACCATCCTGACATGTTCCGTTTTGTCCCTAGACCTGAGCACAAGGTATTGCGAGGCTAATTTAGCGGGGCAATTGATTTTTTGCCACTATGATACAACT
GCAATTACCCGCTGCCACTGACACGTGAGGTCACCTGAGTCACTGAAATGTGGGCCCAGTGGCATTTTGTTTTGCCACTCCCCTTCCAGAGTGGCAAAAA
GTTAATTGTCCCTTAATTTAGCTCAATTCAGTTGTCGGAGCAATTGTGTGTGAAAAGTGTGAACAACAGCATAGGAGACAATACAGCAAGTGTAGCGTGC
CAATATGTGTCCAGCTTGATAGTTCACTATAATAAAGTTCTTTAATTCAAGTATTTGAATAATATAGAATCTCAACCTTCAAGTTTAAAAGGTTTTGTAT
TTGCTATTATAATCGTTGCCACCCCCTCACCCCTCTCAAAAAGAGAGTTACTTTGTAGCGCACTGTAAATCTCTTGATTGAAATAGTGGCATGTCATCTT
ATTTAATTCAGTTATTTCATAAGGTACAATTTGTTTAGCTGACAGCTCAGACATGCTTCTATGCAACATGTTTATACCAATGCTGTATTTTGGCAATCCT
GGATGTGGCATTACTTAACTGTGCTTTCATATTCAATAGTTCTGGCAAAGGGAGGCTGCAAGCTTGAGACAACAACTGCACAACCTGCAAGAAAGTCATC
GGTATGGCTAGAAAACCCTGCAAATTTTACAAATGTACAAGTCAGAAAATTTGCCTAATGTAAAAGGATCAAACTACAGGATTTGTCAGCATGAGCACAA
GTGCCTAACTTCCGAGTTAGATAACTCTGGAGGAAGGAAATCCTTCCTACTGTTTGTTTCAAGAATATACAAAAATTCAAAACAACATTAGAATAGAACC
AAAAGAAGAAACAATATTCACCATGTAGCAATACTCAATTCCTGGTTGTCTTATTTATAAAGGTATAAAACTAGGTTCTGACGAGACAATTATGAATCAA
CTACACCATGTTACAGATCATCCATAACTCTTGGTGATGCAAATTAAGGGCTGTAAAGTGACAAGTATACCTCCACCAACTAATTTGTGCAGAGTGTATA
AAATTGTAAAACAATGGATAAGAATGAATTTAGTATACAAGATAGCATACAATGCCCAAGTTTAAGGAGACATACTGATTTACTGACTCCATAGGACAAT
CTTTTGCTTATGAGGACATCAGTTTTCATAAACAATATCAATGTAAAAGGAGGTAACAGTTTATGAATTTCCTATGCCAAGCCATTTATAAGTCAAAAAA
TGTACCTATTTAGCTTTTATGCATTCCAATTAACTTCCAAAGAAGAAGAGTACATACTAATGGAAAACAAATATGATATTATCATCTGTTTTGCCCGGTA
GTTTCTTGAGTCTCTGATTTGTCCGCACTTCACTCCCAACCAACACAACATTAATACACATGGGCAGAGGAATATCAGGTTATCAGGTAAACTGAATACA
GTTTGATTGACTGCAGTCAGTTGATGGGACAAGATCTTTCTGGATTGAGCGTCAAGGAACTCCAAAATCTAGAAAATCAGCTAGAAATGAGCCTACGTTG
CATCAGGACAAAAAAGGACCAACTCTTGATTGATGAAATTCATGAACTGAATCGAAAGGGAAGTCTCATGCAACAAGACAAGATGGAACTATACAAAAAG
GTCAACCTAATCCGTCAGGAAAACATTGATTTATACAAGAAGGTATTTGATAAATCGTGAATGAAAATTCACAGGATATACACCCCATCCATTATTGACT
GAGGTTTATTTCTCTACTCAGCTCTACGAGAAAGAGGTGACAAGTGAAGTCAACCGAGATTCATCAACTCTAAACTTTGCAGTTGTCGAGAACGTCAACA
TTCCTGTTCATCTTGAACTTAACACTCCACCGCAAGAAAATGATGTTGAGCAAACTGCACCTCCTAAACTGGGGTAACCCTGCTTTTTTCTGCAAAGGAA
CTATTGATTCTGTTCTTAATTCTTTAGTGGCATATTGTTTACTACTAACTGAAAACTTGCAGATTACAACTAAATCCATGAAGGCATGCAGAATGGCACC
GCTTATGTTCATTATTCTCAACGGTACGATTCAAAGCAACCAGAAACACTATTGGTTGTAAAGAGCTTAAACACATAGCAAGACTGACGCGGTATGCATG
CCATGAGTGATTGATAATGGTAGTGTAAATCTATTTCAAATAAAATGTATTTTATTGAAGAATACAGCAAATGATTTGTATTATAACTATTTGACACATT
CTGCAAGTTTGTTGACCAACGTGAGTACATGATAGCTACAAATCTTATATTGACAAAAAGATATGGTGTGTGGAAAAAAATTCAGGTGCTATTTTTTTCTA
TTTTGAGAATTGTATGCATAGCAGAAGAGCACAAATTGCATTCTGGTTTATCCGTGCAGGCATTCCAGAGTATCAGAATGGGGATCAACCTTTCTTCAGC
TACATATGTAAATATACGGATGCTAGTTGCTCACTTAAAATTTTGTAGTGTTCAAAGGAATAACAACAGTAAGACGTTGACGCACCGAAAATAACCAGAA
ATAACGGATTGTACTATGACAAGACACACGACAGATACACTAGCTTAAGTTTGTACAGGAAAATATTTGGCCTATTCTTATGTCACAGTAAATTAAGATT
ATCACCTTAATCACAGAAATTCAAGGGGTAAACGATTATAGCACAAAACGATCCTGGACCCCAAGACCTGGGTCTCTGAACGGCTGAACCTCCAATTATA
CCTCTATACATTTGGAATGTGTGTAGTCAAACTTTCGCTACTGGTATCTTTACAAATATTTACATTGAACACATCAAAATGATATACATGGATTCCTCTC
GATATGTCTTTGAGGTTTGGGTTCTAAAAATGATATACAAATTATTAGGTTCTAAACTTAAATGATATATACACATCAAAATGATATACAAATTATTGGG
TTCTAAACTTAAATGATGTATACACATCGAAATGATATACAAATTATTAGGTTCTAAAAATATACTATGGCAGAATTAGGTGGTCATTTGAGTTTTGGAG
GCCTTGTCAATGTCCCAAACATCACTTATTTGTGACTGGGAGAGCAATGAATTGAAGGAATGAGAGTAACCATACGGTCATTGGTCAATTAAAAGGAACT
TTAATATGCTATGACCACATCGACCTTTCGTAAAGTAAATAACTCAATTATAACCAAGTAATTGGGTGTCTATTGAATCCATACGGTTCTCAAAACAGTT
CTAAACAAAGTGAATTTAAGCATCCTGAAAAGATATTTGGCAAATTTGCCATACCCTGAATTCACAGCATGTTAACACAAATCATGTGCAAATCATTCAG
ATCATTGACAAACGCACGCATGCACACAAATCAACACCGTTTTCTCATGTATAGAACAGAAAAATTGAGAAACTTACGATTTTCTTGCAAGTTATGCAGT
TGTTATCTCTAGCTTGCCGCCTCCAC
>Locus_10929_Transcript_62/78_Confidence_0.134_Length_667
CTCCTACAATCGAGGAGATGCTCTACCCGACGAAAATGAGGCAATTGTTGTTGATGGCGATGCGGTTGATTTGGATTTGCTTTCACAACCTAATGTACAC
GACGCTAAAAGGGATAATACCTTAGCTGGCTTCCAGCTAACATGCGACTCCCCTGAATCTTCAAATATGATGGCCTCTCAGCCAATGAGCTTATCGTCCC
CGTCGCCTGTCTATCACCTAAGCACAGCAGTACCACCTCACCATCAGCGTTTGTACTCATCTGCTTGTCCTGGCTTCTTTCCGAACCTCCAGGAAAGGCC
AGTGGAGCGAAGGCCTGATTTGGGCCCCCAGTCGTTCCCCACCTGGGGACGGCAAACGCAGGGCTCCCCTCACATGCCGTTGCACCACTCCGCAGCATCA
TCAGGATTCTCTACCGCCGCGAACGCCGGCGTCCCATTGCCGTCTCACCCGCCGGCGCCGTTCTCGGACCACCAGTTCTACCTCCCACCGACGGCCTGAG
CTTGTTGGTCCGGTTCAACTCTTGGGACAGCAGCTGACGCTCTGTGTGTGAACGTCTCGCAGCTTGCCGGTAACAGCTGTTAATTAATGAGAGAGAGAGA
GAGAGAGAGAGAGAGAGAGAGAGAGATAGCGCGGGAAGGAGAGAGAGAGAGAGAGAGAGAGAGTCGT
>Locus_10929_Transcript_52/78_Confidence_0.206_Length_2094

GAGCAGGGATTGCGGGAAGCAAGTCTACTTAGGTAAGTGATTGAGTCCTATACGTTCCAGCTCGAGCTTCTATTAACTGCTGATCAGTTTCCGCCTGTTT
GATCTGGTTTACGATGTGCTGATGCTGCTTCTATTCAGGTGGTTTTGACACTGCGCACGCGGCTGCGAGGGCCTACGATCGTGCTGCGATCAAGTTCCGA
GGGCTCGACGCGGACATCAACTTCACTCTGAACGATTACGAGGATGATTTGAAGCAGATGAGGAACTGGACCAAGGAGGAGTTCGTGCACATCCTCCGCC
GCCAGAGCACGGGGTTCGCGAGGGGGGAGCTCCAAGTACCGCGGCGTGACGCTGCACAAGTGCGGCCGCTGGGAGGCGAGGATGGGTCAACTTCTTGGCAA
GAAGTACATCTATCTTGGACTCTTTGACAGCGAAGTTGAAGCTGCAAGAGCATATGACAGGGCAGCCCTTCGCTTCAATGGGGGGGAAGCTGTTACTAAT
TTTGGGCCTAGCTCCTACAATGGAGGAGATGCTCTACCCGACACCGAAAATGAGGCTGTTGTTGATGGCGATGCGGTTGATTTGGATTTGCTTTCACAAC
CTAATGTGCACGACACTAAAAGGGACAATACCTTAGCTGGAGGCCAGCTAACATGCGACTCCCTTGAATCTTCAAACACGATGGCCTCTCAGCCAATGAG
CTCATCGTACCCGTGGCCTGTGTATCACCAAAGCACGACAGCAGTACCACCTCACCATCAACGCTTGTACTCATCTGCTTGTCCTGGCTTCTTTTCGAAC
CTCCAGGTATTACCAAGCTAAAGCCTATTTTTTATGTGCAAATTGCTACTCCTACGTGATCAATTAGTTCAAACTGTTATTCGAATCCAGGATTGTTTAC
TGAATATGTCCTGTCTTCAGAAAAAAATAACACAAAAAGGAACATAATATGTCCTGTCTAATTGTGGACTGATACTAGCCAATTAAACAGTTTCCCATATC
CATTTCCTGTGTTTATTGTTTTGATTTGCTGGCATGGTTAAACTATATAGTAGCTTGATTCGATAAAAACACTAAGCAGGCTTTGATATTTTGAATGAAATG
TCGTTCTTGGAAATACAGCCTTTTCCTTGGTATATCAAGGACAGTTGTCCATCTGCACTCTGCACCCTCAGCTTGTGTAACTGTTTGCCTTGTGAACTGA
TTGATGTGTCAATCAGGCAGTTCTGCCAGTTTGCATCTTTTGAAATAGTGCAGTCTTCCAGTCTAACAGCTCCATGACATAACTTGGGATGCATGCTGCA
GCTTTCTTACTGAGGATGCTCTAGATTTACAGAAATTCTCGCAGTTAGCAGAGGGCTATCAAATCTGATTTGCTTAGCTTCCTGCAGACAATAAGAACTC
TAGAATCCTGAAGAAATCTTACTTGTACATGTTTACCAAAGTATCCTTACTCCTTAGTTGAGCCAATTTTGGTGAAGAACCGATAGCTCATTACTCTGTT
TCCTGGGAATGATGTGGGCCTAAACCATTCTGTGGTCAAAGTTTAACAAAGCCCATGCTTGTGCTGTTGCAACTGCAGGAAAGGCCAATGGAGCGAAGGC
CTGAGTTGGGTACCCAGTCATTCCCCACCTGGGGATGGCAAATGCAGGGCTCCCCTCACATGCCGTTGCATCACTCTGCAGCATCATCAGGATTCTCTAC
CGCCGTCGGCGCAAACGCCGGCGTCTCGTTGCCTTCTCACCCGCCGGTGCCGTTCCCGGACCACCAGTTCTACTTCCCACCGACGGCATGAGCTTGTTGG
TCTGGTTCAACTCTTGGGACGGCAGCTGATCTTATGTGTGAACATTTCGCAGCTTGCCGGTGACCGTTGTTAATTAATCGGGGAGAGAGAGAGCCAGAAGAC
CCAGTATGGCTATCTCTCTGTGGCCCGTGCCTTGTTATGATCTTAGTTATTGCTACTACACCGTGGATTCATCATGGTTTATGAGATTCTTTACTCAAGT
TTCAGCTTAAATGTCCAAATACATGAATGCATCATACACAGGATCCGTCATTTTCTAAAAAAATACAGGATCTGTGTGAAGTGTAAACATAAGG
>comp78487_c0_seq4
ACCCGATTTGTTTAGATTGCAATTAGACTAAGTATAGTTGGTGAGAAGACAGGAGTAGAGATAAGGGCCAACAGCCCAACAGAAAAACCAAGCAAGACTG
CAACTGCAAGAGAGATGGGGAGAGAGAGGAAACCTTGCAGAGGAGAGAAGCAAGGTGGGAAAGAGGCAGCAAGCGAGGCGGGCTGCAGGAACGGAACAT
GCTCCCTCCTCATCTCACAGAAAATGGCACAATAATGATTCAATTTGGTCATCAAATGCCTGATTGCGACTCATCAACTACCCAATCAACCAGCGAGAGT
CATCAAGAAGTGTCTGGAATGAGTGAAGGAAGCCTCAACGAGCATAACGATCGATCAGGTAATCTCGATGGTTACACAAAGAGTGATGAAAATAAGATGA
TGTCAGCTTTATCTCTGGGCAATCCAGAAACTACTTATGCGCATCCAAAACCTGACCGTAGTCAGCCCTTTGCCATTTCGTACCCGTATTGTGATTCATT
CTATGGTGGTGCAGTGGCAACGTATGGCTCACATGCTATTATGAATCCCCAGATTGTGGGCATGATGTCGTCCTCCCGAGTGCCATTACCATTGAACCA
GCTGCAGAAGAGCCCATTTATGTGAATGCAAAACAATACCATGCGATTATCCGAAGGAGACAGCTCCGTGCAAAGCTAGAGGCTGAAAACAAGCTGGTGA
AAAGTCGCAAGCCATACCTTCACGAGTCTCGGCATCAGCATGCCATGAAGAGAGCTCGGGGAACAGGCGGGCGGTTTCTGAACACGAAGCAGCAGTCAGA
GGCTCCTGGCGGCGGCACCTCGGACGCGCAACGCATACCAGCAAATGGTGGCCTGTTCAGCTTACCTCCCAGTGATCTCCACTATCGCGCGAGAGGGGGC
GCTTAAGGAGTCCCATGGCAACTCATCCTTGGCTTAAAAAAAAATGTGTGGCGCTCAGCAATTCGACCTCTGCTAGTTCAATGTAAAGGTGTGGGACTGT
AACTGTGGGTGCTTGAATTGGCCGCAGCCATTCCTGCGACGCGTCGCAGGTGTTGCCGGTTCTGTGGTCGTGCTAGACTTGTGACCGGCACGACGAAACT
GGCACCTGTTTCCTGTAACTGTTGTCTAAGATGATGATGACTACTCCAATAACCATTCTGTATGATGCTTTGACGTCGGTGTTGCTCCTT
>comp83121_c0_seq29
GGAGGCAGCAAGCTTGAGACAACAGCTGCACAGCTTGCAAGAAAGTCACCGACAGTTGATGGGACAAGATCTTTCAGGATTGGGTGTCAAGGAGCTCCAA
AATCTGGAAAATCAGCTAGAAATGAGCATACGTTGCATCCGGACAAAAAAGGACCAAGTCTTGATTGATGAAATTCACGAACTGAATCGAAAGGGAAGTC
TCGTCCAACAAGACAACATGGAACTATACAAAAAGGCCAACCTAATTCGTCAGGAAAACGTTGATTTATACAAGAAGCTCTACGAGAAAGAGGCAACAAG
TGAAATCAACCGTGATTCAACAACTACATACAACTTTGCAGTTGTCGAGAATGCCAACATTCCTGTTCATCTTGAACTTAACAGTCCACCGCAAGAAAAT
GACGTTGAGCAAACTGCACCCCCTAAACTAGGGTAACTGCTTTTTCTGCATAGGACCTATTGATTCTGTTCTTAGTTCTTTAGACACATATTATTTACTA
CTAACTGAAATCTT
>comp67351_c0_seq14
GAGCGGCGCACGGATTAGCCTGTCCAGCAACCCGGTGGGTGGCGCGACCAAAGGCGCCGCGTTCCAGACGGGTGACGATGCGAGAGGCGAGAGCAGCGAG
GAGTCACGGAGATCAGGGGAATCAAAAGATGGAAGTGTTGGTCAAGACAGCAACCATGCCACATCACATATGCCTGCTTTGGTGTCGGAGTATTTAGCAC
CATATACACAGCTGGAACTGAACCAATCAGCTCCTGCAACATATCAGTACCCAGATCCTTACTATGCAGGCATGGTTGGTCCCTATGGAACTCAACCTGT
GACCCATTTCCAGCTACCTGGGTTAACTCACCCTCGCATGCCATTACCTCTTGAAATATCTGAGGAGCCTGTCTATGTAAATGCAAAGCAGTACCATGGA
ATTTTAAGACGAAGGCAGTCACGTGCCAAGGCTGAGCTTGAGAAAAAGGTGGTTAAAGTCAGAAAGCCTTATCTTCACGAATCTCGCCATCAACATGCAA
TGAGGAGGGCAAGAGGAAATGGGGGTCGCTTCCTAAACACAAAGAAAAGCGACAATGGTACTCCCAACGGTAAAGCTGAACCTAAGAAAGGAGAGCAAAA
CTCCAAGCATATCCATGTCCCTCCTGATTTGCAACTTCGACAGAAGGAGGCATGAAGTAGCGGTCTGGCAACCTAAAACAGTGGCTTCTGTCCACAGGCA
TACACACTTTTGAGTCCGGATTTGGAGCCCCAGGGTTGCGCTGCCATCTTTGCAGTTCGTCTTCTTTGTGCAAAGAGGTCCAAGGTTCGTCCACCCGAAT
GAGCTTGGTACCAGTTGTTAGTCCTGGCAGAATGCAATCTGGGTGCAGGAGACCGTTGAAGTGTCCAATGCCATGGGTAGGTCTGCTGTTTTCAGGCAAT
TCATTCTTGGCTTTCACATTCCGCTCTTGTTTGTACCTGGTGTGTTGTAAGTGGAACTAGCTTGTGTTGTGTCTTCGGCGTCCTGTGAGACATGTATAAT
TAAGATCAACTTGGGATCATCGTTGTAATCCTATGTCTGGCTTTCTTTTAGTTTTGAACTGTAACAAGGCCAAAGCTGAACTCTTTATACAGAGATAGCA
ACTCTTGCTGGCCTGTTCGTTGTTCAGCAACGTCAGTGCTGTACTTAATCCTGTGCAAATGAATAAGCTT
>comp82180_c0_seq22
AATATAGGGGGCCCCGCTCCCGCAGCTCGCAGTACCGCGGCGTCACCTTCTACCGCCGCACCGGCCGCTGGGAGTCCCATATTTGGGATTGCGGCAAGCA
AGTGTACTTAGGTGGATTTGACACTGCTCATACTGCTGCAAGGGCGTACGATCGAGCTGCCATCAAGTTCCGTGGCTTCGACGCCGACATAAACTTCAAT
CTCAGTGACTATGAGGATGACATGAAGCAGATGAAGAGCCTGTCCAAGGAGGAGTTCGTGCACGTCCTGCGACGGCAGAGCACCGGGTTCTCGCGTGGCA
GCTCCAAGTACAGAGGCGTCACCCTGCACAAGTGCGGCCGGTGGGAGGCTCGCATGGGACAGTTCCTCGGCAAGAAGTACATATATCTTGGGCTATTCGA
CAGCGAAGTAGAGGCTGCAAGGGCTTATGATAAGGCCGCGATCAAATGCAATGGTCGAGAAGCTGTGACGAACTTCGAGCCAAGCACGTATGATGGGGAG
CTGCTGACTGAAGTTGCTGATGAAGGTGCTGATGTTGATCTGAACTTGAGCATATCTCAACCAGCTTCTCAGAGCCCGAAAAGGGACAAGAACTCCCTTG
GTCTGCAACTCCACCATGGATCGTTTGAAGGCTACGAATTGAAAAGAACAAAGATTGATGCTCCCTCTGAACTGGCGGGCCGCCCTCATCGATTCCCTCT
TCTGACCGAGCATCCACCAATCTGGACTGCCCAGTCTCACCCCTTCTATTCAAATGATGAGGTTAAGTGATACTAAATTACTAAAATACATGCAATAACT
TTCTCAAATATATTGCTTTAGCTCCCTAAACCTCACAATTGCAAGCATGTTCTGAGCATCACATGGTTCGGATCGAGCTGTCCGGCACCAATTTTGAACG
TAATCTTGACCATCTGAGGAACTGATTGTGCAGCCTGAAGGCGTGCGTTCAAATGTTAGATTTGTGTTACTTTCGTTCATTTTCACTTCTTGTGCCGCTG
TCAATTTTGGCTCTAGTCTTACATCACCGCTTCATTTCCTTGGATTCTATAGCCCGCCTGTTTCCTGTCGATTTCGGAGATATTAATGCGTAGCACAGGT
TTCATATGTAGGATCTTGAGTTTTCTGAGATGGCCCATTCTGGAAACAGTAATAGTCGCCTTAGGAATAGAGATGTGTTTGATTTTTTTCCTTCAAAAGG
AACATCCTAGAGAGGTGACTATTCGACATTAGGTATGCTAATCCATCGGTCAACTATACCTGCCTGCGGCTAAGCACCTTTCAATGTTTCTTTTTTCATT
CATCGTATCATATAAAAGTAGTAAGTATGACTATTATTCAAACCTTTCTTGAAAAAAATAGTTAGGATGTTGCCATCTTGTAATGCAATCATAGAATTGG
AAGTGCTGAATTTGCCATTTGTTTCAAACAAGCATTGTATATTGGTTTATCATTCAACTTGAGAGTTTGGTCAAATTGAACTCTCGAACTTTTCAACTCC
TAAACTTTAAGGTCAGGCTCACATATGTCATTTTCAAATTTTATTGGACGGAAAGTTGAGCTGGACGTTTCGAGCTCGGCTTTCTATCCAACTGAACTAA
TGGAAACACTGATTTCATCGCAACAAATTTTCAGCAATAGATATTCAATCTCTTCTTGAATTCATCCTTCATCAGTATGGTGAATAAATTGTTCTTGGAT
TATGCAGAATGCATCAAGAGATCTTAACAGGAGGCCAGATCAGGGATGCACAGTAGGGGGGTGTTCCCAGCTGGGCGTGGAAGGTGACCGCCCCTCCTCCC
ACGCAGCCATTGCCGCTGTTCTCGTCGGCGTCATCATCCGCTGCAGCATCATCAGGATTCTCCAATACCGCCACGACAGCTGCCCACACCGCCCCATCGG
CCTCCCTCCGGTTCGACCCGCCGCCGCCGCCGTCCAGCCGCCACCGCTGAACGTTAAGAAGCCACGCTGTAAATTTGCCAGGAAGCCGACATTTTTTCCT
CTCTCGGCGTTGCAACTTTTTAGGTTTTGCGCCGGGGTGGTTTCTTGTAGTGGAGTGGATTCATAATTAACTGTATTTGCATGCTGCCCATGTGAAATGC
TTCTCTCTCTTTTTACGCTCTCTGCACCGGATGTTCTGGGAGTTCTTGTTGCCGGGAATTAACTAAATTCACCGTCTGAGATTGATCTATACATTGTTGT
AGAGAATCGAATCATTGTTGAAATTAGTTACAGAATTATAGATATCATAAGGCACCCTCTACTTGTGGACAAAGTTCAGGAAGATTATTCCGGTTCTTTT

CTTGGCTGTCAATTCGGCTCTTGTCCTTGGTGTGGAGCCACTTGCCTACTGTCTTGTTCTGTTGCTGTTTGTTCTTGGCTTCTTGCACAGTTACAAATTT
GTTGGCTCGAATGGATGGTGAGACATTTGGTGCAA
>Locus_4522_Transcript_3/58_Confidence_0.100_Length_1519
TGCTCAACAGATGATGAAATCTCATGCATGCATAAATCAAGAATTGAATCAAGAAAGCCGAGTTCCCAAACAAAAAAAGCATGATCCTTCAATCTGCCAG
CTAAAGGCCACACTAAATCAGATAATGAAGAACCTAAAGACAAAACATCTTTCAGATTGGAAATGGGTCTATCTGTGTGAGAAGGAAATGCTCCACACGA
TGATAGCATACGATCAAGTGATTCCAGGTCAGCGAAAGGTTTTGTCAAGATTGAAAGTAGAGCCTTGCTTCGTCTCAAATGAAATTCGCTTTCCTGGTTA
GGATCCACCATAGATAAGAAGTACTGTTTAGAAGAAAGTAACGAATTCATGACACTGACTTCTACTTTCATAAGTGATGAAATCCGCATATCTTTCAAAT
CCGCATATCTTTCTGATTCCATGTTTCATGGAGTTGTTCCAATTGTGCCCTACAGACTTCCTCCTGTGAAGCTAGCTCCTCTGCTTCTTCCCAAGATAGA
TGTTCTCTACCTCTCACAGCAGCTTCTTCAAGAGCCATTTGCTGCTCAGTAATTCGACCAACTTTCACAACATAGCTCTGTTCCAGTTCAGTAAGAGACA
ATCTCTCAAGTTCTACCTCAACCAGCTTCTCTAGACCTTTATCGACAGAACCACGTATCTGAGAAATTCAACCAAAGGCTTCCATTGCCTCTGAATTGTA
TGAAACAAATGATCTAATAAATTCTGGAAGAAAAGCTTCAGTCATCTGTTCAATCATATTGATACTTGAGCGCAAAACAGCCTGAAAAGTAGAAGCCCAG
TGCCTAGGTCGGGGCTTTGCATAAGCAGCACTCGTTTCAGCTAATTTTTTCTCAATCATTTGATGCACTTCATCAACAACTCCCGAAACTAGTGCACATT
TCTCTATCTGCTGTTCTAACAATTGCAAGCCCATTCCAGCGTCAACTTGAAGACCAGAATCATCTGTCCTAAATATAGCTTTTCCAATAGCTTTACTTTG
AGAGGTCAGACACTTTGGCTCTAACATCACTATATAGCTGCCCCACAGCAATCCCAAGAATAAGACCTTCCGTGTTGTCTCCTGTATGTCATCCTTGGCT
GGAGTTTTGATCTCACCCTGTATAGGACTTCCTGAATGCGATAAATGAACATTATCCCCATCCTTTTGTGATGACAACTGAACAGAATTCATGAATGCAG
AAAGTATCCGCTCTTTGGATTGCACTTCATTATCCAACCCAATGGAATTCATCAGTTCAGAACATTCCCTCGCAAGTCTTTCCACACATGCAACATAATT
TTCCATTTGATTTAAGAGATCCCGGTATCTTTGCTGCACAAGATTAAGGCCTTCACCTTGAACCTTAGCAATGACTTCTGCAGCCTTTCTTTTGGCAAGT
GGAAGCATATCTTGTGATGCATTGCTTACAGATAGCTGCAAAACTTGCGCCCAACTAGTAATTGGACTGGTTGTGATGTAGTTAACTGGAAGAACTTGCT
GCAAGACTAAAGCATATTC
>comp82180_c0_seq13
AATATAGGGGGCCCCGCTCCCGCAGCTCGCAGTACCGCGGCGTCACCTTCTACCGCCGCACCGGCCGCTGGGAGTCCCATATTTGGGATTGCGGCAAGCA
AGTGTACTTAGGTGGATTTGACACTGCTCATACTGCTGCAAGGGCGTACGATCGAGCTGCCATCAAGTTCCGTGGCTTCGACGCCGACATAAACTTCAAT
CTCAGTGACTATGAGGATGACATGAAGCAGATGAAGAGCCTGTCCAAGGAGGAGTTCGTGCACGTCCTGCGACGGCAGAGCACCGGGTTCTCACGGGGCA
GCTCCAAGTACAGAGGCGTGACCCTGCACAAGTGCGGCCGATGGGAGGCTCGCATGGGGCAGTTCCTCGGCAAGAAGGCTTATGATAAGGCCGCGATCAA
ATGCAATGGTCGAGAAGCTGTGACGAACTTCGAGCCAAGCACGTATGATGGGGAGCTGCTGACTGAAGTTGCTGATGAAGGTGCTGATGTTGATCTGAAC
TTGAGCATATCTCAACCAGCTTCTCAGAGCCCGAAAAGGGACAAGAACTCCCTTGGTCTGCAACTCCACCATGGATCGTTTGAAGGCTACGAATTGAAAA
GAACAAAGATTGATGCTCCCTCTGAACTCACGGGCCGACCTCATCGGTTCCCTCTTCTGACCAAGCATCCACCAATCTGGCATGCCCAATCTCATCCCTT
CTATTCAAATAATGAGGTTTGATGATACTAAATTACTAAAATACAGGCAATTACTTTCCCAAACATATTGCTTTGGCTCCTAAACCTCACAATTGCAAGC
ATGTTCTGAGCATCACATGGTTCGGATCGAGCTGTCCGGCACCAATTTTGAACGTAATCTTGACCATCTGAGGAACTGATTGTGCAGCCTGAAGGCGTGC
GTTCAAATGTTAGATTTGTGTTACTTTCGTTCATTTTCACTTCTTGTGCCGCTGTCAATTTTGGCTCTAGTCTTACATCACCGCTTCATTTCCTTGGATT
CTATAGCCCGCCTGTTTCCTGTCGATTTCGGAGATATTAATGCGTAGCACAGGTTTCATATGTAGGATCTTGAGTTTTCTGAGATGGCCCATTCTGGAAA
CAGTAATAGTCGCCTTAGGAATAGAGATGTGTTTGATTTTTTTCCTTCAAAAGGAACATCCTAGAGAGGTGACTATTCGACATTAGGTATGCTAATCCAT
CGGTCAACTATACCTGCCTGCGGCTAAGCACCTTTCAATGTTTCTTTTTTCATTCATCGTATCATATAAAAGTAGTAAGTATGACTATTATTCAAACCTT
TCTTGAAAAAAATAGTTAGGATGTTGCCATCTTGTAATGCAATCATAGAATTGGAAGTGCTGAATTTGCCATTTGTTTCAAACAAGCATTGTATATTGGT
TTATCATTCAACTTGAGAGTTTGGTCAAATTGAACTCTCGAACTTTTCAACTCCTAAACTTTAAGGTCAGGCTCACATATGTCATTTTCAAATTTTATTG
GACGGAAAGTTGAGCTGGACGTTTCGAGCTCGGCTTTCTATCCAACTGAACTAATGGAAACACTGATTTCATCGCAACAAATTTTCAGCAATAGATATTC
AATCTCTTCTTGAATTCATCCTTCATCAGTATGGTGAATAAATTGTTCTTGGATTATGCAGAATGCATCAAGAGATCTTAACAGGAGGCCAGATCAGGGA
TGCACAGTAGGGGGTGTTCCCAGCTGGGCGTGGAAGGTGACCGCCCCTCCTCCCACGCAGCCATTGCCGCTGTTCTCGTCGGCGTCATCATCCGCTGCAG
CATCATCAGGATTCTCCAATACCGCCACGACAGCTGCCCACACCGCCCCATCGGCCTCCCTCCGGTTCGACCCGCCGCCGCCGCCGTCCAGCCGCCACCG
CTGAACGTTAAGAAGCCACGCTGTAAATTTGCCAGGAAGCCGACATTTTTTCCTCTCTCGGCGTTGCAACTTTTTAGGTTTTGCGCCGGGGTGGTTTCTT
GTAGTGGAGTGGATTCATAATTAACTGTATTTGCATGCTGCCCATGTGAAATGCTTCTCTCTCTTTTTACGCTCTCTGCACCGGATGTTCTGGGAGTTCT
TGTTGCCGGGAATTAACTAAATTCACCGTCTGAGATTGATCTATACATTGTTGTAGAGAATCGAATCATTGTTGAAATTAGTTACAGAATTATAGATATC
ATAAGGCACCCTCTACTTGTGGACAAAGTTCAGGAAGATTATTCCGGTTCTTTTCTTGGCTGTCAATTCGGCTCTTGTCCTTGGTGTGGAGCCACTTGCC
TACTGTCTTGTTCTGTTGCTGTTTGTTCTTGGCTTCTTGCACAGTTACAAATTTGTTGGCTCGAATGGATGGTGAGACATTTGGTGCAA
>Locus_18892_Transcript_37/56_Confidence_0.309_Length_1330
TGGTGGCTGCGCCGTCTCCTGCGGCTCTATCTCCCGCCGCCGCCGGGAAGAAGAGCCGGCGGAGGCCGAGGTCCCGGAGCTCGCAGTACAGGGGCGTCAC
CTTCTACAGGAGGACGGGCCGGTGGGAGTCGCACATCTGGGATTGTGGGAAGCAAGTCTACTTAGGTGGTTTCGACACTGCTCACGCGGCTGCGAGGGCT
TATGATCGTGCAGCGATCAAGTTCCGAGGGCTCGACGCGGACATCAACTTCACTTTGAGCGACTATGAGGATGACTTGAAGCAGATGAGCAATTGGACAA
AGGAAGAGTTCGTGCACATACTCCGCCGCCAGAGCACGGGGTTCGCGAGGGGGAGCTCCAAGTACCGCGGCGTGACGCTGCACAAGTGCGGCCGCTGGGA
GGCGAGGATGGGTCAACTTCTTGGCAAGAAGTACATCTATCTTGGACTCTTTGACAGCGAAGTTGAAGCTGCAAGAGCATATGACAGGGCAGCCCTTCGC
TTCAATGGGGGGGAAGCTGTTACTAATTTTGGGCCTAGCTCCTACAATGGAGGAGATGCTCTACCCGACACCGAAAATGAGGCAATTGTTGATGGCGATG
CGGTTGATTTGGATTTGCGGATTTCACAACCTAATGTACACGACGCTAAAAGGGACAATACCGTAGCTGGCTTCCAGCTAACATGCGACTCCCCTGAATC
TTCAAATATGATGGCCTCTCAGCCAATGAGCTTATCGTCCCCGTCGCCTGTCTATCACCTAAGCACAGCAGTACCACCTCACCATCAGCGTTTGTACTCA
TCTGCTTGTCCTGGCTTCTTTCCGAACCTCCAGGAAAGGCCAGTGGAGCGAAGGCCTGATTTGGGCCCCCAGTCGTTCCCCACCTGGGGACGGCAAACGC
AGGGCTCCCCTCACATGCCGTTGCACCACTCCGCAGCATCATCAGGATTCTCTACCGCCGCGAACGCCGGCGTCCCATTGCCGTCTCACCCGCCGGCGCC
GTTCTCGGACCACCAGTTCTACTTCCCACCAACGGCCTGAGCTTGTTGGTCAGTTCAACTCTTGGGACGGCAGCTGACGTTGTGTGTGAACGTTTCGCAG
CTCTCCGGTAACAGTTGTTAATTATTGGGAGAGAGCCAGAAGACATAGTATGGCTGATCTCTCTGGCCTGTGCCTCGTTATGATCTGAGTTATTGCTACT
ACTACACCGTGGATTCATCATGGTTTATGAGATTCTTTACTCAAGTTTCAGCTTAAATGTCCAAATACATGAATGCATCATACACAGGATCCGTCATTTT
CTAAAAAAATACAGGATCTGTGTGAAGTGT
>Locus_12915_Transcript_15/30_Confidence_0.373_Length_3737
GCTCCTTGAGAAACACCATCCAATTACAAGACTATTTGATGCAACAATCACGCATATGGCTGCGCTAGAACTACTGCTCTGAATTAAGAGGCGTTGAAAT
ATTGTCCGGTCTACATATAGGCACTTGAGGTGTATTGACAGACAGTTCCCCTTAGTTTGCTACAACACATCAGGCGAATTCCACCAAATTAGGATGATGT
TTGTTTCTTAGACACAGGATAGCAATGCCAAAAGTTTTTTATAATTTTCCGCTTCACCGCAGATTTGGCATCTCAAAGGGAGTTGTGGCAAGATTCTCTT
GACCAAGCTTTCTTGCAGATTTGGCAAGAAAGATAAAAAAAAATGTTGGTATACTTAAGCATCCTTAATAGAAACTAAACAGGTCTTAGATTGAAATTATC
ATAAATAAACCCTTTTTCACTTTAGCTGTAGTCCGGATGAAATCTTAGGGGCCTTGCTTAGCCTGCCCTGAAGCTTGCCAGTAAGTCGAAGCTGATGCTG
ACACAGCACTGTTTCAAGTTCTCATCTTCTGAAAATGATCAGCAGTCAATAGATGTTTTAGTCCATTCTCCAGAAAAATGAGTGCCGCATGAATCCTTTC
CGGTTTATAGTGAAACATTTTAGACGATGACCTTTAACATTCTAGAAAACGAAGAGAAAACCTACAGTCATCTAATTTCAGCTTAAACATGTTCCAGCAA
GATCATCACCATCCTGACATGTTCCGTTTTGTCCCTAGACCCGAGCACAAGGTATTGCGAGGCTAATTTAGCTCAATTCAGTTGTCAGAGCAATTGTGTG
TGAAAAGTGTGAACAACAGCATAGGAGACAATACAGCAAGTGTAGCGTGCCAATATGTGTCCAGCTTGATAGTTCACTATAATAAAGTTCTTTAATTCAA
GTATTTGAATAATATAGAATCTCAACCTTCAAGTTTAAAAGGTTTTGTATTTGCTATTATAATCGTTGCCACCCCCTCACCCCTCTCAAAAAGAGAGTTA
CTTTGTAGCGCACTGTAAATCTCTTGATTGAAATAGTGGCATGTCATCTTATTTAATTCAGTTATTTCATAAGGTACAATTTGTTTAGCTGACAGCTCAG
ACATGCTTCTATGCAACATGTTTATACCAATGCTGTATTTTGGCAATCCTGGATGTGGCATTACTTAACTGTGCTTTCATATTCAATAGTTCTGGCAAAG
GGAGGCTGCAAGCTTGAGACAACAACTGCACAACCTGCAAGAAAGTCATCGGTATGGCTAGAAAACCCTGCAAATTTTACAAATGTACAAGTCAGAAAAT
TTGCCTAATGTAAAAGGATCAAACTACAGGATTTGTCAGCATGAGCACAAGTGCCTAACTTCCGAGTTAGATAACTCTGGAGGAAGGAAATCCTTCCTAC
TGTTTGTTTCAAGAATATACAAAAATTCAAAACAACATTAGAATAGAACCAAAAGAAGAAACAATATTCACCATGTAGCAATACTCAATTCCTGGTTGTC
TTATTTATAAAGGTATAAAACTAGGTTCTGACGAGACAATTATGAATCAACTACACCATGTTACAGATCATCCATAACTCTTGGTGATGCAAATTAAGGG
CTGTAAAGTGACAAGTATACCTCCACCAACTAATTTGTGCAGAGTGTATAAAATTGTAAAACAATGGATAAGAATGAATTTAGTATACAAGATAGCATAC
AATGCCCAAGTTTAAGGAGACATACTGATTTACTGACTCCATAGGACAATCTTTTGCTTATGAGGACATCAGTTTTCATAAACAATATCAATGTAAAAGG

AGGTAACAGTTTATGAATTTCCTATGCCAAGCCATTTATAAGTCAAAAAATGTACCTATTTAGCTTTTATGCATTCCAATTAACTTCCAAAGAAGAAGAG
TACATACTAATGGAAAACAAATATGATATTATCATCTGTTTTGCCCGGTAGTTTCTTGAGTCTCTGATTTGTCCGCACTTCACTCCCAACCAACACAACA
TTAATACACATGGGCAGAGGAATATCAGGTTATCAGGTAAACTGAATACAGTTTGATTGACTGCAGTCAGTTGATGGGACAAGATCTTTCTGGATTGAGC
GTCAAGGAACTCCAAAATCTAGAAAATCAGCTAGAAATGAGCCTACGTTGCATCCGGACAAAAAAGGTAAGGATGCTAGCCATATTTCTGTTTTTCGTTA
CCAGATTGCCTCATCCAAGAATACAACTGATGATGACGTTTTTTACTTTCAGGACCAACTCTTGATTGATGAAATTCACGAACTGAATCGAAAGGTTCTG
GAATATTCTGTTATTACTCTGCAATGAGAGGTCTCTGAGTGGATTTCAAGAAGCTGACTCTACTGGCTCATTTTCAGGGAAGTCTCATGCAACAAGACAA
GATGGAACTATACAAAAAGGTCAACCTAATCCGTCAGGAAAACATTGATTTATACAAGAAGGTATTTGATAAATCGTGAATGAAAATTCACAGGATATAC
ACCCCATCCATTATTGACTGAGGTTTATTTCTCTACTCAGCTCTACGAGAAAGAGGTGACAAGTGAAGTCAACCGAGATTCATCAACTCTAAACTTTGCA
GTTGTCGAGAACGTCAACATTCCTGTTCATCTTGAACTTAACACTCCACCGCAAGAAAATGATGTTGAGCAAACTGCACCTCCTAAACTGGGGTAACCCT
GCTTTTTTCTGCAAAGGAACTATTGATTCTGTTCTTAATTCTTTAGTGGCATATTGTTTACTACTAACTGAAAACTTGCAGATTACAACTAAATCCATGA
AGGCATGCAGCATGGCACCGCTTATGTTCATTATTCTCAACGGTACGATTCAAAGCAACCAGAAACACTATTGGTTGTAAAGAGCTTAAACACATAGCAA
GACTGACGCGGTATGCATGCCATGAGTGATTGATAATGGTAGTGTAAATCTATTTCAAATAAAATGTATTTTATTGAAGAATACAGCAAATGATTTGTAT
TATAACTATTTGACACATTCTGCAAGTTTGTTGACCAACGTGAGTACATGATAGCTACAAATCTTATATTGACAAAAAGATATGGTGTGTGGAAAAAATT
CAGGTGCTATTTTTTTCTATTTTGAGAATTGTATGCATAGCAGAAGAGCACAAATTGCATTCTGGTTTATCCGTGCAGGCATTCCAGAGTATCAGAATGG
GGATCAACCTTTCTTCAGCTACATATGTAAATATACGGATGCTAGTTGCTCACTTAAAATTTTGTAGTGTTCAAAGGAATAACAACAGTAAGACGTTGAC
GCACCGAAAATAACCAGAAATAACGGATTGTACTATGACAAGACACACGACAGATACACTAGCTTAAGTTTGTACAGGAAAATATTTGGCCTATTCTTAT
GTCACAGTAAATTAAGATTATCACCTTAATCACAGAAATTCAAGGGGTAAACGATTATAGCACAAAACGATCCTGGACCCCAAGACCTGGGTCTCTGAAC
GGCTGAACCTCCAATTATACCTCTATACATTTGGAATGTGTGTAGTCAAACTTTCGCTACTGGTATCTTTACAAATATTTACATTGAACACATCAAAATG
ATATACATGGATTCCTCTCGATATGTCTTTGAGGTTTGGGTTCTAAAAATGATATACAAATTATTAGGTTCTAAACTTAAATGATATATACACATCAAAA
TGATATACAAATTATTGGGTTCTAAACTTAAATGATG
>Locus_2976_Transcript_15/49_Confidence_0.323_Length_1918
AGTCCTAATATGTATCGCTTCAAAAAAATAAATTTCAGTATATCAAAGTTTCATGATTGGAGCAGCATAGCCAAGCTACACTGATTGGCATATGAAGGGC
ATTGTACTCACTATGGTGCTTAGGGCAGTACAGGTAAATTGCACTTGACAAAATAGTATCGCTCAATTGCTGTGACATGTTTATTAACATGAGATCACAA
CGTTTGAAGGGAAACTCAAGCTAAATAACCTCACTTCAAAAAAGTTGGAATTCATGACTTTGGCCTGGATCCGAAATCTGTAAAAGAGCACTGCTCATCT
TGTACCAGTGAAAAATAAACTTCAGATCGGTATGCTACAAAGCTATTTGACAGATGCTTTTACAGCTTTCTCTGCCGCATCATCAAGATCCTCAGCAGTG
ATCAGTGTCATTCCACTTTCCTTAAGAATCCTCTTTCCTTGGTCCACATTGGTGCCTTCTAACCGAACAACAACAGGAACCTTCAGGTCAACCTGTTTAG
CAGCATTTACAATTCCACTTGCTATCACATCACATTTCATGATGCCCCCAAATATGTTCACTAGAATTGCCTTCACTCTATCATCTGAAGTCAGTATCTT
AAATGCTTCCACAACCTGTCCCTCTGATGCACTTCCACCAACATCAAGGAAATTGGCAGGTGTTCCGCCATGCAACTTGATAATGTCCATGGTAGCCATA
GCCAATCCTGCTCCGTTCACCATGCAACCAATCTCTCCATCAAGACCAATATAGTTCAAATCTGCTTTGGCAGCAGCAACCTGTAAGCATAGAAGTGTCA
GTGCTGCGATTAGATAACCATAAGAGTGAATATGAAAACCTAAAGTATTCGCTCAGAGGCAAGGCTTCCACAAAAATGAGGTCTAAAGGCAGAACAAGAG
TAAAAACACCATCAAAGAAAGCTCAACAAAAAAGGAGAAAAAGTGCAAGATGGGAGAAACAAGCAATTCTGCACCTCCCTGGGGTCTTCCTGTGTTGTAT
CACGCAGTGCAAAGATTTCCTTCTGCCTGAATGCAGCATTGTCATCAAAGTTCAACTTTGCATCAGCAGCAACAAGCTTGTTGTCAGCTGTCTCAGCAAG
AGGATTTATCTCCAGCAGTGTGCAATCGCACTTGCAGAAAAGTTCATACAACTTCTTAATCTGTTCCATAGAAGACTGCCTGTCTGCTGCCTTTAGGGCC
AGACCATCAACAACTTTAGCTGCATCCTCATCTGTGATTCCCTTGAATACATCAATAGGAACCTTAATAATCATATCAGGATACTTCTCTGCAAGATCTT
CAATACTGGTTCCTCCCTTGCTGCACGCAATTATGAGCGGACCAGCAGTTTTCCTATCAAGGGTGATGGCAAAGTACATCTCATTAGTAAGTGACAGTTT
CTCACACAAGTAGACCTTGCTCACAATCTTCCCCTCTGGACCAGTTTGTTTTGTTACCAGAATCTGGCCTAGCATTTTACTTGCAATTCCTTCAGCTTCC
TCAGCCTTAACAATATGAACACCACCTTTCAGTCCACTTTTGAAAGTTCCCAGTCCTCGTCCGCCAGCAAGGATTTGACTCTTAACAACTATCTCTTTCT
CACTGGGGAATACGTTCTTCAAGGCGTCCTGCACCTCCTGCACGGACCCAGCAGCCGCGCCCCTGGGCACGTTGATGCCGTACTTCCCCATCAACTCCGC
GCCCTGGTACTCGTGGATGTTGAGGCGGCGGAGCTGCTGGTGCTGCCATCTCCCGGCGACGGAGACGGCGCGCGACGCGAGCTTCCCGAGAGATCCGCGA
ACCATGGCTGCGGCGACCTTGGTGGGCGGGGCTGGCTCCTTCGATCCGGAAACCCACAAGCGTGGAAGACGATTCCTCTTTCCGGTTTGATTTTTTTTT
TTTTTTTTTTTTTTTAAA
>Locus_3441_Transcript_11/417_Confidence_0.038_Length_678
CTGTGCTTTCATATTCAATAGTTCTGGCAAAGGGAGGCTGCAAGCTTGAGACAACAACTGCACAACTTGCAAGAAAGTCATCGGCAGTTGATGGGACAAG
ATCTTTCAGGATTGGGCGTCAAGGAGCTCCAAAATCTAGAAAATCAGCTAGAAATGAGCCTACGTTGCATCCGGACAAAAAAGGACCAACTCTTGATTGA
TGAAATTCACGAACTGAATCGAAAGGGAAGTCTCGTCCAACAAGACAACATGGAACTATACAAAAAGGTCAACCTAATCCGTCAGGAAAACATTGATTTA
TACAAGAAGGTATTTGATAAATCGTGAATGAAAATTCACAGGATATACACCCCATCCATTATTGACTGAGGTTTATTTCTCTACTCAGCTCTACGAGAAA
GAGGTGACAAGTGAAGTCAACCGAGATTCATCAACTCTAAACTTTGCAGTTGTCGAGAACGTCAACATTCCTGTTCATCTTGAACTTAACACTCCACCGC
AAGAAAATGATGTTGAGCAAACTGCACCTCCTAAACTGGGGTAACCCTGCTTTTTTCTGCAAAGGAACTATTGATTCTGTTCTTAATTCTTTAGTGGCAT
ATTGTTTACTACTAACTGAAAACTTGCAGATTACAACTAAATCCATGAAGGCATGCAGCATGGCACCGCTTATGTTCA
>Locus_2827_Transcript_125/166_Confidence_0.185_Length_3349
CTCTCTCTTTCTCTCACTCTCTCTCTACCTGTGTTGCTCGCGACTGCACGCTGCTGCTGCGGCTGCCGTTCCACGTCTCTGTCTCCGCTTTAAGGCTGTC
ACCTTTCCCCCCTCGCTCTCCTTCTCTCTCCTCAATTACGACGCCACAACCAGACCCAAACCCTAGTACCACGCGCCGGAGTAGGTAGGTGGTGCTGCCC
CTTGATCCGATCCACCCGGCGCTGTAGAGCTTTCCGGCTTGTGCGTGCTCACCGGCGATCGATGGGAGGCGCCGCGGGAGGCGGCTCAGGCGCGGGGAGG
CTGTGAGGTCGGTGGGTGTGCTGCGCGTCTGTTCCGGCGGCGCTCCCTTCGTCTTGCGGGAGCCTGTCGGTGTGTCTCGGCGGCGTTCGCTTCGTTTGCA
GAAATCTTCACGAGCTGAAAGTTGAGGCGAAACCGAGGAGCTAAGAGCAGCAGTTGAGGTGTTGAAGTGCATAGGACAACTAAGAGGAAGAGGAGGACGA
GATTTGTGTGGTTGGGCTTTTGCATTGGCGAGGGAACAATGGCTGCGGCGGTGGCGATGCGAGGGGGAAGCAGTGACAGCGGAGGGTTTGATAAGGTTCC
GGGGATGGACTCGGGGAAATACGTGCGGTACACCCCGGAGCAAGTTGAGGTACTCGAGCGGCTGTACATCGATTGCCCCAAGCCAAGCTCCTCGCGGCGG
CAGCAATTGCTGCGCGAGTGCCCTATACTTGCCAACATTGAGCCCAAGCAGATCAAGGTTTGGTTCCAGAACAGAAGGTGTCGCGATAAGCAGCGGAAGG
AGTCTTCGCGGCTTCAGGCTGTGAACCGGAAGTTAGCGGCAATGAACAAGCTTCTGATGGAAGAGAATGAGCGTCTTCAGAAGCAGGTCTCTCAGTTGGT
TCATGAGAATGCACACATGCGGCAGCAGCTGCAGAATACTTCTTTGGCCAATGATACAAGCTGTGAATCAAATGTGACTACCCCTCCAAACCCTATAAGG
GATGCAAGTAACCCTTCTGGACTCCTTTCGATTGCAGAGGAGACCTTCACAGAGTTCCTCTCAAAGGCTACTGGGACAGCTATTGATTGGGTCCAGATGC
CTGGGATGAAGCCTGGTCCGGATTCGGTTGGTATTGTGTCTGTTTCACATGGTTGCCGAGGTGTTGCTGCCCGTGCCTGTGGTTTGGTGAATCTAGAACC
AACAAAGGTCATAGAGATCTTGAAAGATCGTCCATCTTGGTTCCGTGATTGTCGAAGCCTTGAGGTGTTCACAATGTTTCCAGCTGGAAATGGGGGAACA
ATTGAACTTGTCTACATGCAGATGTATGCCCCAACAACTTTAGTCCCTGCACGTGACTTTTGGACGTTACGATACACGACAACAATGGAAGATGGCAGCC
TTGTGGTCTGTGAGAGATCCTTGAGTGGTTCAGGGGGTGGTCCAAGTGCAGCCTCTGCGCAACAATTTGTAAGAGCTGAGATGCTTCCAAGTGGGTATTT
AGTTCGCCCATGCGAAGGTGGGGGCTCGATTGTGCATATAGTGGACCATCTAGATCTCGAGGCGTGGAGTGTTCCTGAAGTGCTTCGACCACTCTATGAG
TCTTCTAGAGTAGTTGCTCAGAAAATGACTACTGTGGCACTGCGCCACCTTAGACAAATTGCTCAAGAAACAAGTGGGGAAGTAGTGTACGCCTTGGGCA
GGCAACCTGCAGTCCTACGGACATTTAGTCAAAGGCTGAGCAGGGGCTTTAATGATGGCATTAGCGGTTTCAATGATGATGGCTGGTCTATAATGGGGGG
AGATGGTATTGAAGACGTAGTTGTTGCTTGCAACTCAACTAAGAAAATTAGGAACAACAGCAATGCTGGAGTTCCGTTTGGAGCCCTTGGAGGTATTATA
TGCGCGAAGGCATCCATGTTACTGCAGAGTGTTCCACCAGCAGTACTAGTTAGATTCTTGAGGGAACATCGGTCTGAATGGGCAGATTATAACTTCGACG
CATATTCGGCCTCGGCGTTGAAAACAAAACCTTGTTCCCTTCCTGGGTTGCGGCCTATGAGATTTTCTGGGAGCCAGATCATCATGCCACTTGCTCACAC
AGTGGAAAATGAGGAGATCTTAGAAGTTGTTCGTCTTGAAGGACAGGCTCTTACACAAGATGAAGGTCTTTTATCAAGAGATATTCACTTGCTTCAGCTT
TGCACTGGAATAGATGAGAAATCTGTGGGGTCCTCCTTCCAGCTCGTGTTTGCACCAATTGATGAACATTTTCCAGATGATGCTCCATTGATTTCTTCTG
GCTTTCGTGTCATACCACTTGATATGAAAACAGACGGCATATCTTCTGGTAGGACGTTAGATTTGGCATCTAGTCTTGATGTTGGTTCTACCACGCCCCA
CGCCTCAGGAGATGCATCACAGGATGATTGTAATTTGAGATCTGTGCTGACAATTGCCTTTCAATTCCCTTACGAGATGCATCTCCAAGACAGTGTTGCA
ACTATGGCCCGTCAGTATGTTCGCAGTGTTGTTTCTGCTGTGCAAAGAGTGTCGATGGCTATCTCTCCCTCCCAATCTGGTATAAATGCTGGGCAGAGGA
TGCTTTCTGGCTTCCCTGAGGCAGCCACACTTGCTCGCTGGGTTTGCCAGAGCTATCATTACCATCTAGGGGTAGAATTACTTAACCAGTCAGATGAAGC
TGGCGAAGCATTGTTGAAGATGCTCTGGCATCATCCAGATGCTATTTTGTGCTGCTCTTTTAAGGAAAAACCTATGTTTACATTCGCCAACAAGGCAGGG

CTTGACATGTTAGAAACGTCCCTTATTGCCTTACAAGACCTCAAATTAGACAAGATCTTTGATGAGTCTGGAAGAAAAGCGTTATTCTCAGATATCTCTA
AATTGATGGAGCAGGGCTACGTGTACCTGCCATCGGGCGTGTGCATGTCAGGAATGGGTCGCCATGTTTCGTTCGACCAAGCTGTGGCGTGGAAGGTCCT
TGGTGAAGACAGCAACGTCCACTGCCTGGCCTTCTGCTTCGTGAACTGGTCCTTCGTGTGAGCCTTTGCCATCCTCCGAATGCCGACTTGATCTGTGACG
AGGAGATCCATTTTTGTATTTATCTGGAGAAGGAAAATCTTCCACTGTGACAAGGCTTATGTTGCACTAGCTAGCTCTAGGTTTGGGAATCCAGTGTAAA
ATCTGATGTCTTTCCATTTGGCTACCAAGCTGTGCTCTGGTTGTTTATCTTGTTTGGGACGATGCTAAGTTGCTAACATTGGGTTCCCAACTTACGCGTC
GAAGTGTGTTTCATCGCATCAATCTTCAATCGCAGCTGAATTTTCGTTT
>comp70939_c0_seq6
CCGGGCGCTCGCCCCGGATTTCTCGGCTCCCCATCCGGCGGACGCCTTGACCGGGGTGGGTTCTTGGTTCCGGCGGCTCGGTGATCTGTGCGTGCGAAAG
GCGGGGCTTTTAGCTCGTTCTGTGGGGGGAGGAGGAATACGGCGGCGAGGCGTTCATGGGTTAGATCTGGCGGTCCGGCGCCGCGAATCTGAGCGAGGTT
TCGGTGGAGGGATCGGGGCGGAGAAGTCGCAGTCTCTGGCTCGTTCTGAGCAGCTTGGGCCTCTCATGTCCAAATCGGGTATGGATGATGGGTGCGGTAA
CTGTCGGAAGGAGCGTCGCTTGCAGATCTGAAAGCCATCCAGCTCCCTTGTTCGGTTGACCTGGAATGGCCGTGCTTTCTGCTGTTTGACTGTCAGTAGT
TGCTGCTTAGTGTGTGTGCAAGAAATTTAGCTGGAGGTGATGTGCTTAGGAAACTAGTCTAGTTGCTGATATCTTGGTCTCTGTTGAAGAACGCTGAAGC
TGGAAGGAAGTAGGTCGCATTCCTGTGTTGTTGATATTTTGTTGTTTCTCGAATAACCTGGAGGCTGGAAGGGATCAGATCCAGTTCCTGTGGCACCGGT
AACAAGGTGCTGTTGGGGGAAGGCACCATGACCTACTTCCCTGAGGAGGTGGTGGAGCATATATTCAGCTTCTTGCCGTCGCACAGTGACCGGAACACGG
TCTCGTTAGTGTGCAAGGTGTGGTATGAGGTTGAGAGGCTGAGCCGGTGTGCTGTCTTTGTGGGGAACTGCTACGCGGTGCGCCCAGAACGGGTGGTGCT
GCGGTTCCCCAATGTGAAGGCCCTGACGCTGAAGGGGAAGCCGCACTTCGCGGACTTCAACCTCGTGCCGCCGGACTGGGGTGGCTATGCGGGGCCATGG
ATTGAGGCTGCGGCGAGGCGATGCGTGGGTCTTGAGGAGCTCCGGATGAAGCGGATGGTGGTGTCAGATGAGAGCCTTGAATTGCTAGCTCGGTCCTTCC
CGAGATTCAAGGTCCTCGTCCTTATCAGCTGCGAGGGGTTCAGCACTGATGGGCTAGCAGCTATTGCGAATCACTGCAAGCTCCTGAGGGAGTTAGATTT
GCAGGAAAATGATGTGGAGGACCGTGGGCCTAGATGGCTCTCCTGCTTCCCTGATTCTTGCACATCACTTGTTTCCTTGAATTTTGCCTGCATCAAAGGG
GAGGTGAATTCTGGTTCATTGGAGAGGCTTGTTGCTAGGTCTCCAAACTTACGCAGCTTGAGGTTAAATCGATCTGTATCAGTAGATACACTCTCAAAGA
TATTAGCATGCACCCCTAATTTGGAAGACTTGGGGACAGGGAATTTGACGGATGAGTTCCAAACTGAATCCTACTTCAGGCTGGCTAATGCTTTGGAGAA
ATGCAAAATGTTGAAGAGTTTATCAGGATTTTGGGATGCTTCTCCTGTTTGTGTTCCGTTTATCTATCCCCTATGTCATCAACTAACAGGACTGAACTTG
AGCTATACTCCCACACTGGATGCCTCTGATCTCACTAAAATGATCAGCCGCTGTGTGAAACTCCAGCGTCTTTGGGTACTGGATTGCATCTCGGATAAGG
GTTTGCAAGTGGTGGCTTCCAGCTGCAAAGATCTACAAGAACTGAGGGTGTTCCCGTCAGATTTTTATGTTGCTGGTTATTCTGCAGTGACAGAAGAGGG
ACTTGTTGCAATATCGTTAGGCTGTCCAAAACTTAGCTCCCTGCTGTATTTCTGTCACCAGATGACCAATGATGCACTAGTTACTATAGCTAAGAACTGC
CCAAATTTCATACGATTTAGGCTCTGTATTCTTGAGCCAAAGAAGCCAGACGCCATGACAAACCAGCCGTTAGATGAAGGCTTTGGTGCAATTGTTCGTG
AATGCAAGGGGCTAAGGCGACTGTCAATGTCGGGTCTTCTCACTGACAAGGTTTTCATGTATATTGGAAAGTATGCCAAGCAACTTGAGATGCTTTCTAT
AGCATTTGCTGGAGATAGTGATAAGGGGATGATGCATGTTATGAATGGATGCAAGAATCTAAGGAAGCTGGAGATTAGAGACAGTCCATTTGGTGATGTT
GCACTGTTGGGGAATGTTGCCAAATATGAAACAATGCGATCCCTTTGGATGTCCTCATGCAATGTCACATTAAAGGGGTGCCAAGTCCTTGCGTCTAAGA
TGCCGATGCTCAATGTGGAGATCATGAATGAGCTAGATGGAAGCAGTGAAATGGAGGAAAACCATGGAGATCAATCTAAGGTGGAGAAGCTATATGTTTA
CCGCACAACTGCTGGAGCGAGGGATGATGCACCAAATTTTGTTAAAATCCTATAGAGCTAGCTGAGAGTAGTACGGTATCTTCTCACTCTAAAAGATGGT
CGGTGGAATCCGGCAGGCTGGGGCCGCGTCAACAGTGGTAAGTTCTTGAGGTGTCTGTCTCTCCCTGTGCAGGGGTCAGGAAAAGCCACACCGATGTGC
ACCCTTATCTTGGAGCAACTGGCTGGGATCCATCATCATCCCCTGTACCACCGCCCCCTACCCTTTTGTGTTTGGAATTCCTTTGTGTTTGCTCACTCAT
ATCCACTGAGGCCCTTTATCATCCAGAGCATTCTCACTTGAGAGGACCGCCTTTTTTCCGTTTATCCCACTGTAATCTGTAGTGTACCAGTATCCATTTG
CATTTGACATTGGCAGTGGACTGTTTTGCCGCTGTTGTTGTAGTGTTTGTTTCAGCCTGACGATTTGTTTCTTGCTCCTGTGAATGCAGTGCGTGGAATA
ATTTGCGGTTGTTATCTGCGACATTCTATTCTCTGGTTTTGAATTCTTGGTGTTAGCCGTACCAGTTTTACGTGCTTGTTACTGAGCAAGAAGGCACCAG
TACTCGTGCATTTTAAGGGCGTGCTCAGTTTCTGATGCCATTTTTCTCGCTTACATCATCTTTCTGTATTGCTGCCCTCAAATATGAATTCAAATTAACG
GT
>Locus_5417_Transcript_10/28_Confidence_0.172_Length_854
GCCAGCACCAGCATGAAGTCAGTGATTGATCGATATGGCAGAGCGAAGGAGGAGCAGCAGCTTGTGGCAAATCCCAATTCAGAACTTAAGTTCTGGCAAA
GGGAGGCAGCAAGCTTGAGACAACAACTGCACAACTTGCAAGAAAGTCATCGGCAGTTGATGGGACAAGATCTTTCAGGATTGGGCGTCAAGGAGCTCCA
AAATCTAGAAAATCAGCTAGAAATGAGCCTACGTTGCATCAGGACAAAAAAGGACCAACTCTTGATTGATGAAATTCATGAACTGAATCGGAAGGGAAGC
CTCATCCAACAAGACAACATGGAACTATACAAAAAGGCCAACCTAATTCGTCAGGAAAACGTTGATTTATACAAGCTCTACGAGAAAGAGGCAACAAGTG
AAATCAACCGCAAGCTCTACGAGAAAGAGGCAACAAGTGAAATCAACCGTGATTCAACAACTACATACAACTTTGCAGTTGTCGAGAATGCCAACATTCC
TGTTCATCTTGAACTTAACAGTCCACCGCAAGAAAATGACGTTGAGCAAACTGCACCCCCTAAACTAGGGTTACAACTAAATCCATGAAGGCATGCAGCA
TGGCACTTGCTTATGTTCTTTATTCTCAACAGCTACGATTCAAAGCAGCCTGAAAAACTATTGGTTGTAAAGAGCCTAAATACAGCAAGGCAGACGCAGT
ATGCATGCCATGGGCGACCGATAATGGTCGTGTAAATCTATTTCCAATAAATTGTATTTTATTGAAGGGATGCACCAAACGATTGTCTTACAACTATTTG
ACACATTCTGCAAGTTTGTTCACCAACGTGACTACGTGAGTACATGACAGCTAA
>comp67351_c0_seq22
GAGCGGCGCACGGATTAGCCTGTCCAGCAACCCGGTGGGTGGCGCGACCAAAGGCGCCCGCGTTCCAGACGGGTGACGATGCGAGAGGCGAGAGCAGCGAG
GAGTCACGGAGATCAGGGGAATCAAAAGATGGAAGTGTTGGTCAAGACAGCAACCATGCCACATCACATATGCCTGCTTTGGTGTCGGAGTATTTAGCAC
CATATACACAGCTGGAACTGAACCAATCAGCTCCTGCAACATATCAGTACCCAGATCCTTACTATGCAGGCATGACCCATTTCCAGCTACCTGGGTTAAC
TCACCCTCGCATGCCATTACCTCTTGAAATATCTGAGGAGCCTGTCTATGTAAATGCAAAGCAGTACCATGGAATTTTAAGACGAAGGCAGTCACGTGCC
AAGGCTGAGCTTGAGAAAAAGGTGGTTAAAGTCAGAAAGCCTTATCTTCACGAATCTCGCCATCAACATGCAATGAGGAGGGCAAGAGGAAATGGGGGTC
GCTTCCTAAACACAAAGAAAAGCGACAATGGTACTCCCAACGGTAAAGCTGAACCTAAGAAAGGAGAGCAAAACTCCAAGCATATCCATGTCCCTCCTGA
TTTGCAACTTCGACAGAAGGAGGCATGAAGTAGCGGTCTGGCAACCTAAAACAGTGGCTTCTGTCCACAGGCATACACACTTTTGAGTCCGGATTTGGAG
CCCCAGGGTTGCGCTGCCATCTTTGCAGTTCGTCTTCTTTGTGCAAAGAGGTCCAAGGTTCGTCCACCCGAATGAGCTTGGTACCAGTTGTTAGTCCTGG
CAGAATGCAATCTGGGTGCAGGAGACCGTTGAAGTGTCCAATGCCATGGGTAGGTCTGCTGTTTTCAGGCAATTCATTCTTGGCTTTCACATTCCGCCCT
TGTTTGTACCTGGTTTGTTGTAACTTGTAAGTGGAACTAGCTTGTGCTATCTTCGGCGCCCTGTGAAACATATGTATAATTAAGATAAACTTCGGATCAT
ATTTGTATCCCGGATCATATTTGTATCCTATGTCTGGCTTTCGTTCAGTTTGAACTGTAACAAGGCCAAAGCTGAACTCGTTAAACAGAGGTAGCAACTC
ATGTTGGCCTGTTGGGTGTTCATCAATGTTGTTGTAATGAATCCTGTACAAAGGCAGAAGCTTATTTCGA
>Locus_9469_Transcript_41/63_Confidence_0.204_Length_1132
GAGAGAGAGAGAGAGAGAGAGAGAGAGAGAGAGAGAGAGACCCTGCAGAGGAGAGGAGCAAGGTGGGAACGAGGCAGCAAGGGAGGAGAGCTGCAGGAAGGG
GAACATGCTCCCTTCTCATCTCACAGTATGAACTGAAAAAACAAGAATCGAGAATTTCCATGAAATGAGACAAAATGGCACAATAATGATTCAGTTTGGT
CATCAAATGCCTGGTTACGACTCATCAGCTACCCAATCAACCAGCGAGAGTCATCAAGAAGTGTCTGGAATGAGTGAAGGAAGCCTCAACGAGCATAACG
ATCGATCAGGTAATCTCGATGGTTACACAAAGAGTGATGAAAATAAGATGATGTCAGCTTTATCTCTGGGCAATCCAGAAACTACTTATGCGCATCCAAA
ACCTGACCGTAGTCAGCCCTTTGCCATTTCGTACCCGTATTGTGATTCATTCTATGGTGGTGCAGTGGCAACGTATGGCTCACATGCTATTATGAATCCC
CAGATTGTGGGCATGATGTCGTCCTCCCGAGTGCCATTACCAATTGAACCAGCTGCAGAAGAGCCCATTTATGTGAATGCAAAACAATACCATGCGATTA
TCCGAAGGAGACAGCTCCGTGCAAAGCTAGAGGCTGAAAACAAGCTGGTGAAAAGTCGCAAGCCATACCTTCACGAGTCTCGGCATCTGCATGCCATGAA
GAGAGCTCGGGGAACAGGCGGGCGGTTTCTGAACACGAAGCAGCAGTCAGAGGCTCCAGGTGGTGGCACCTCAGATGCGCAACACATGGCCGGCAATGGT
GGTCTGTTCAGGCTGCACGAGCACAACTTACCACCCAGTGATCTCCATTATGGCGCGAGAGGTGGCTCTTAAGGAGTCCCCTGGCAACTCATCCTTGGCT
TAGTAACGTGTGGCGCTCAGCGATTCGACCTCGGCTTCGATGTAATGGTGTACGATTGTATCTATGGTGCTTGAATTGGCAGCAGCCATTCCTGCAGCAA
TGCGTCGCAGGTGTTGCCGGTTCTGTGATAGTGCCAGACTTGTGACCGGCACAGAACTGGCATCTGTTTCCTGTAACTTTAGTTTTTCTAAGACGATGAC
GATGACTACCCAATAACCATTCTGTCTGTATG
>Locus_14326_Transcript_40/49_Confidence_0.333_Length_3120
TGACGTGCGAGCGCCGAGCATAGGAGGGAAGGCGAACCATTTCTTGCTTTGTTTATTGCACCGGAACAATAAGAAACTGGAAGAAGACAGAAAAGAGGTT
ATACAGTAGGCTCATATTTGGCATCAGCAGAGGCCTCGCGGTGCCCCGTATTCTCATTTCCTCGCCGTATTTTCTCTCTCCAGCTCCAGAGTCCGGAGGA
AGCAGAGAGAGAAACTCAACGGGAAGGTAGAGAGAGGTACAATGCTCCTGATCCCTATTTGAAATCCTCGATTGGTCAGTTCTGTTCTTGGATACACTGT

TGCTGCAGAATGGATGACTGTGCTTTCCTTTCGGGGAGTGGCGTCGTGCTGTGTTGGTTCTTGCCTTCCTAATTAGTAATATGCCTTAGTCTTGCTCTAC
GGTTGGTTTGATTCACGTTTTTCCTGTAATCTTTCTACCCCCAGGATTTTCCCCTTCGGTTTTCTTGGTTTGCTTCTCGGAAATTTGTGATTTTTAAGAG
GATTTTTGGCTTGTGTTGCTCGATTTTGGTTCTTGACCGTGCTTCCTTGGGATGAGCTTCTTGACCGTGCTTCCTTGGGATGAGCTTTTGCCTGGTTCGA
CATTACAAGATGTGTTATCCTAGAAGTTAAATTGATCTTCTATGAGCTGGGCGCTGCATTGTGGATTGCATGGGGTGGCATACTGTGGGCCGTGGAGTTG
ATGGAATCATGGAAATCCGCAGTCCCTTTTGTTGGATAAGGGTGGTTGAAGCTAATTTTTTGCGAATTGTGGCGGTTTCTGGATATCCTGCTTGAATTAG
TGGTGTGATAGATTGTTCTAGAGAATAGCTAGATATATGTTATATTGTTAGGACATCAAGAGTGTTGAATCAGGTATGTTAATGTGTGAACTTGTTGATG
GGAGCTAAATGGTTGCTGACGTTCCCTATAATGACCTAATCAAACCTAACAAGCACTAAATTAATGATAAACAGCACTGATCAGTAAGCCTACTGAAGTA
AAAACCAATCACAATTCATACAGTATGCAACAGTATAAAGTAGTGAAAGAGTTCTTCAGAGCTCGAACTTTTGGGATGAAAATAATTTGGCTCAGGATAG
TCCACTGAAAGGAGTTTAGGATATCTGTTTGCGCTCTGCCTGCAATATATGAAAATCTGAAGCTGATATGGTAGTAGAGTTGGGTACCCTGATTTCCGAG
CCAAAAGAAGTTTAAGGTAGAATTTCTCAGGAATTATGTGGTAGTAAGAAGCAATTTAAAAGGAACCTAATTAGGAAAAAAAGACATGCCTTGCTTGGTC
ATGAGAAAATATTATTGCTGTATAAAATAGGTATGCCACCTCCAGTCAGTACTTGATTAATTTCATTGAAAATGGAGGGCTGTAATAGATCCTTATCTAT
TAATGTGTTGTACCTAAACTTGGAAATCATCAGCTGAGGGCATTGTGCATGGTGCGTGGAGTACTAGCCCTCGGGAGATGCTTCTTAATTAGGCAGCGAG
AAGGATAGAGATCGGTTTGACATATGTTCCATTTGTAGCTGTACTGGATATTTCTGCATGTTCCCCTGGAGCAGTTAGTTTCAGAATTATGATATTTCTA
CTTGTGGCAGGATTGTTCTGTTTCTATTATCTGTTCATATTTATGCTAGTCTGTCTTTTATGTTGTAATATCTATTGTCTATTGCACTTCAAGTGACTAT
GCATAACCATATTCCAGTGATTTAGTGAATGGTTATGAATTAAAGTAAGATGACGTTTTCATCAATTATCCTTGTTCTTTATCACGTCATTATCAATTCA
GCTGATTAAATTCTGGCATCTTGTCATGATGGTTACTTTAGCTGATTTAATGTTTCTCAGAGCTAAGGATACCATCTCATAGTGCTCACTCTTTTGTGGG
AAATGGAGGATCCCATGCCGAAGTCCAACAATGGCTTCTTGTCAGGAAATGGCTATCAGATGAAGCAATTTGGTCATAAAATCGACGATAGGGACTCGTC
ATCAGAGTCCGGTCGGTCTCACCAAGAAGCTTCCACAGCGAGTGAGAACAGTATAAATGAACAGCACACCTCAACACAATCTGACAATGATGATGGTCAT
GGGAAGCATAATGAGGACATGATGAAGTCAGCAGTGTCCTTGGGGGAACCAGGATCAGCCTTTTTGGCCCCAAAACTAGATTACAGCCCATCTTTTGCTT
GTGTTCCTTATACTGCTGATGCTTTTTATGGTGGGGTCTTGACAGGATATGCTCCACATGCCATTGTTCATCCCCAGCAAAATGATTCAGCAAACTCTCG
GGTTCCGTTGCCTGTTGAACCTGTAGCAGAAGAGCCAATATATGTTAACGCAAAGCAATACCGTGCAATCCTTAGGAGGAGGCAGATACGTGCTAAACAG
GAGGCTGAGAATAAGCTGGTGAAAGGTCGGAAGCCATACCTCCATGAATCTCGACACCGCCACGCAATGAAGCGAGCCCGTGGATCAGGGGGGCGGTTCC
TCAACTCAAAGCAGCTCCAGGAGCAAAACCAGCAGCATCAGGCATCAGGTGGTTCAAGCTGCACAAATGCCATCGGCAATAACACAAGCTCCCAAAGCGG
CCCCACTCACACGCCTTCGACACCTGCTTCCTCTGACACCGCAAGCGCTTCGAGGGCCAACCAGGACCACATCTGCTTCCCCTCCGCTGGCTTCCGCCCC
GCAATGAACATCAGCGATCATGGTGGAGGAGACACAAAGCTGTCTGCGAACGGCATGCAGCAACGTGTTTCCTTCATCAGGTGAAGAAAAGTGGCACTGC
CTGTGGCAACTCATCCTTGGCTTATGAAACTATGGATATGTAATGGGCATGTAGGTTCGGTCTCAGAATAACCAAACATATGTGAGACCTTTTTAAGTTT
CCTTTAGTTCAAAGAATGATAGGTTATGACCTTATGGCTTTGTTTCTGGAGACTGAAATCCGTGCTTGATATACTATGTTGTACTGAGTTCTGAGGTGAA
AGACATCGGGAGAGTATCAGGTGCAAATGGCACAAATGTTGAAAACGGTGCAAATTCATCAAAATCTGTACCTAAAAGACTTCAATAAATTTTAAACTTT
GCATAGCTCTAGGTGCATTT
>Locus_4749_Transcript_50/72_Confidence_0.505_Length_4493
GGTACATGTCGCGGAGCTACGCGCCGGCGTTCCACAGCTTCGCGTGGGCGCAGGCCGTGCAGAAGAAGCCGCTCGTGCCAAGGCCGGCCGCTGCTGACGA
GGACGAGGTGGAGCACGCCGTGGACGCGTCGGACGAGGAGAAGGAGGAGGGCGAGATTGAGGAGGGGGAGACCGTGGAGACCTCCTCCTCGTCGCCCCCG
CGCGCGCAGCCTGAGACTATCGATTTGGACTCTGACGCGCCGGAGAAGTCTGAGTCTGTGGCTGCGGAGGGCAGTGGTAATGCTGCCCCTGCCACCGGCT
TCGCCGAGGAGGAGGAGGAGGAGGTGGATTTTGACCAGCGCGTGGGGAGTATACTCGAGGAGCTCGAGATGGTCTCCATTGAGGAAGCTGAGAAGTCGTT
TGAGGGTGCGTGTGCACGCCTGCGCACTTGCTTTGAGAGCCTGAAGCCGCTGTTCCCGGAGAACGGAAGCCCGATGCCGATGCTTGAACCTCTTGTGCAG
CAGGCGTTTGTCGGAATTGACACCCTCACCACTGTGGCGAATTCGTATAATTTGCCGAGGAAGGAGCAGAACAAGACCATGCTCTTGAAGATGTTGTTCC
ACATAAAGAACCGATATTCAGACATGCTGACACCTGACCAACGAGATGAGCTGGACACCCGTGTGAGGCAGTTAGTTTTTGAGGAAAAAGACGATATCAG
CGACCCAAGTGCCAATTGTGGCATGAAAGCAATGAATGTTTCGGCTCCATCTGGGCAGGTTTCTTCAGGGAGACTACCGTTTGAGTCAGGAGCAGCAAAT
CCATTTAGTGGCTCTAGCTTGCCGAGGTTGGAGATACCTGCAAAAAAGATTAGTCCCTTGTTGGATCTTCATGCAGATTATGATGAGAACAGCTTGCCTT
CGCCCACTCGGGATAGTGCGCCATCTTTTCCTGTGCCGAGGCCCATTGGGTTTGGAGCATTTCCATTGGCACATGAGAAACTATCTTTCCCAGAAAGAGC
TGAGCCTCCAAAGAATTCGTTGTATCCATCCTTAAATGATCCTCTGAAGGCTGTTTCCTCTTACCAGCAGAAATATGGGCAGAAATCTTCCTTTCCAAGT
GATGATCTGCCAAGTCCAACTCCTTCGGGCGATGAGGATAAATCTGCAGACAAAGGTGGTGACCTATTTGGTGAGGTTTCCAGCTTCTCAATTCCAAAGA
AGACTGCATTGCCAAGTACTAGTCAGATGCCTGCTTCTCAACCAAGCACAGTTGGCAGCAGCAATGATAGTTATACAGGTGGTGCGCCTGGTTATGCTAA
ACAAATTGAACAGCCGGCTACAGGACCAAATCATGGACTAAAGGCTACATCTAAAAGCAGGGATCCAAGGCTCAGGTTTTTGAACCGTGATTCTGCTGGT
GCTACAGACGCGAATCGGTGTGCAAATTTTGCAGAACCGAAAGAAGAAAACCTGGGTGATGGAGTATCAGTTAATAACCGTAAGCAAAAGGCAGTTGATG
AACCAAAGGTGGATGAAAACTTGTTAAAAAGGCTTAGGACTGGTAGTGGGAACCAAAGAGACATGCTGGTGCCATCAGGGAATTCTAATTCTAATCTTAG
AACGGGTAATCAGCTGATTAACAGCATAAGTGCTACCACAAATAGTAGCGGAATAAATACCAAAATTTTGCAACCCCCTCAAGCTACTGCCCCACAAATT
AGTGCAGCACCTGCTGTTTCTTTGCCTACTGTGTTAAAGGATATTGCTTTGAACCCAGCAATGATCATGCATTGGATTCAAATGGAACAGCAGAAGATGT
CAACATCAGAACCTCAGCTGAAGGTGGCTGCTCCGGGTGGCATGACCAGTATTGTAACTGCAACAATGGTTTTACCACCTGGTTATACTCCAAAGACCAC
GGAAATTCCTCAAATTCCACCTGTTAGGCCACAAGTTCCAGTGCAGACACCTTTGAACCCACAAAACGATGTTGCTGGAATAATACGTATGAAGCCCCGT
GATCCCCGGCGGATCCTCCACAGTAACATAGCACAGAAGAATGATACAGTGGGCTCGGAGCAAGTGAAAACTAATGGAGCAACCCAACCAGACACTCAGG
GCGGCAAAGACCATTTGACCAACCGTGAACAACAGCCAGAGCAACATCAGACTAGTTCATTGCCTTCTCAACCAGCCTTGCTGTCAAACATTACGCGACC
ATCCACCATTAACTCAAAACCTGTTGATGCTGTCTCAAACTCACAGTTAGCTGCTACAGCACTTATAGCTCCAACACAGCAAGCTTTAGGCAGCAATAGG
GTAGATCCAAGACTAGCGGTTGGACAGAATGGTGCTAATGCTGAAGCAGCAACAAATGCTGCTCCTGCACCAGCACTTGAAGCTCTGCAGCCAGTTGATC
CATGGGGTGATGTTGACCATCTCCTTGATGGTTATGATGACCAGCAGAAGGCTCTCATACAAAAGGAAAGGGCAAGACGGATCTCGGAACAGCAAAATTT
GTTTTCAGCGCGGAAGCTATGCTTAGTCCTTGATTTGGACCACACACTACTCAATTCTGCAAAGTTTGGAGAAGTGAATCCTGTTCATGAAGAGATTTTG
CGGAAGAAAGAGGAACAAGACAGGTCTTTGCTAGAACGGCATCTGTATCGTTTCCAGCATATGGGAATGTGGACCAAGCTGAGACCAGGAATATGGAACT
TCCTTAAGAAGGCGAGTAAGCTTTTTCAGTTGCATCTGTACACAATGGGGAACAAGCTGTATGCTACTGAGATGGCCAAGGTTCTCGATCCTACTGGGAA
ATTGTTTGCAGGGCGAGTCATCTCAAGAGGTGATGATGGTGATCCTTTAGACAGCGATGATCGAGTCAAAAGTAAAGATCTTGATGGAGTATTGGGAATG
GAATCTGCAGTAGTGATAATAGATGACTCTATAAGAGTCTGGCCTCACAACAAGCACAATTTGATAGTTGTAGAGAGATACACCTATTTCCCCTGCAGCA
GACGTCAATTTTGCCTTCCTGGACCATCACTTCTCGAAATTGATCGAGATGAGAGGCCGGAGGATGGCACACTTGCTTCTTCACTGGCAGTTATTGAGCG
AATTCACCAAAACTTCTTTGCTCACCCTAACCTCAATGAGGCTGATGTGCGGAGCATACTAGCATCTGAGCAGCGGAGGATCCTCGCGGGTTGTCGTATT
GTCTTTAGCCGGATTTTCCCGGTTGGAGAGGTCAACCCCCACTTGCATCCGCTGTGGGAGATTGCAGAGCAGTTTGGCGCTGTTTGCACAAACCAGATTG
ACGATCGGGTTACTCATGTTGTGGCCAACTCCCTTGGGACGGACAAGGTGAACTGGGCACTATCCAATAACAGATTTGTGGTTCATCCAGGATGGGTGGA
GGCTTCAGCTCTCTTGTACCGGCGTGCCAATGAGCTTGACTTTGCAGTCAAATAACGCATCCCTGAGCCCCAATTCTCCATCTAGGGCCCCGAGATTCTT
CCCGGGATGAGATCCGGTCATGAAAAACTAACATGAAAAGAAACCAAACAACATTCTAAAACCGGACCCCATTAAGAAGCAGACCATACCACCTCTAACA
GAATCAGGCCTAAACATGAAAGATAACGAAAATACTTAGTGATAGCCTGAAAGAGGAGACGGTGGTTGATGCCATTGCTGAGGTGATGAGTACAACTTGA
TTCAGAAGTGTTTTAGAAAGAACCATCAAAGAAAGATGAGCTAAAAGCGAAGATGACGAAGGCTCATCACCTCCCGGTTGTAACATGTAGAATTGATGCA
TTTCTTATAAGAGTCGTAGGTCCAGATGTTGCCTCTGGCTGCAAGCCTGCTGCTGGCCACAGAAACAGGGTTGGTAGCATGCGCTGGTAACCCATCAAAG
TGGCAACGGCAGCAGCAGTCTTGGGGATGAAGTCATGGACAAGGCAGGCATATAAAGTTTGATGTGTGTATATTTGTGGCTTGGCTGTAAGTGTGTAGTT
GGTTCTCGCTGTGACCTGAAACTGAAAGCCACATGGAAAAGGACACCTGGGGCTGCGCCTCTGCTGCTCAACGAAAAGAGTGTACAGAAAAGGGTCCAGA
AAAGGGGAGTGGAGAACTGTTGTAGAATACCAGAACCAGTAGACACCTGGGCGCTTTCTTGTATGAAACCAAGTAACCGGAGTAAATGGTGGCACAGAAG
AATGCGTTAAGTGTGATCAGTTGGATACTGGTTATGGAGAACGAAGCGTATGACCGGGTTTTTACTGAAACTGTAAGCGATTATTGGGGTTGCTCGTGGC
ATGGCTATATTCTGTAAGGTCCGTTCGAAGGGTTTTTTCTTTTTCTTTTTGGTGTGGTTGGGGTGGGGGTTGTTTCATGACAGCGTTTTTACCGTTTCTT
TTTGTGATTCTTTTTAAAATTTATTTGTAAAATATGTGTTCCAAACTGGTCCAAATGTGCGGGGCTTCATTCTAATTTCAGTAAGTTAAACGG
>Locus_25981_Transcript_6/14_Confidence_0.381_Length_856
TCTACAGGAGGACCGGCCGCTGGGAGTCGCACATCTGGGACTGCGGGAAGCAGGTCTACCTGGGTGGATTTGATACAGCGCACGCTGCGGCAAGGGCCTA
TGATCGCGCTGCGATCAAGTTCAGGGGCCTTGATGCGGACATCAACTTCCAGTTGAAGGACTATGAGGACGACTGGAAGCAGATGAGGAAATGGTCCAAG

```
GAGGAGTTTGTTCACATACTCCGACGTCAAAGCACTGGGTTTACAAGGGGGAGCTCAAAGTACCGCGGTGTGACACTGCACAAGTGCGGCCGGTGGGAAG
CTCGGATGGGTCAGCTTCTTGGAAAGAAGTACATCTACCTTGGGTTGTTTGACAGTGAAGTTGAAGCTGCAAGAGCATATGACAGGGCAACCATTCGCTT
CAATGGAAGGGATGCTGTTACTAATTTTGATCCTAGTTCCTATGATGGAGATGTTCCACCTGAAATTGAGAAAGAAGGGGTTGATGGGGACATCCTTGAT
TTAAATTTGAGGATCTCGCAACCTAACATGCATGATTCGAAAAGTGATGGCATCCTGACTGGGTTCGGATTAAGTTGTGATTTTCCTGAAGCTTCAAGTT
CTGTTGTTTCTCAGCCAATAAGCCCTCAGTGGTCTGTACATCCCCAGAGCACAACTACTCCACCCCAGCATCAACATTTGTATGCATTTCCTTGTCCGGG
CATCTTTGTGAACCTCAGGGAAGTGCCTATGGAGAAACGACCCGAGTTGTGTCCCCAGTCGTTCCCTCCGTGTGCATGGCAAAAGCAGGGTTCCCCTGCG
CCATCACTCCCTACTGCAGCATCATCAGGATTCTCTACTGCCACCGGCGGCGCGGA
>Locus_19346_Transcript_10/16_Confidence_0.321_Length_2394
CGTCGTCTCGGCGTTCCTCAACCTCGCCGTAGCTCCCGGCTCCCCCTCGCCTCCTACCCTCGCTCCTCCGTCCTGTGAGCCCTCCGCCGCCACTTGCTCC
ACCGTCGTCCTCGGTCTGCCGCACGTCCAAGGCACACCGACAATCTCCCGCCGAGCAATCCCACACGCTCCGATGACCCTCCCTCGAGATCCCCCATGCA
CGATGATGACCTCCAGCCGATCTACCCCAATCCGGACGACCAACCGCAGCCACAAGGTCGTGGAGATGATGAGCGGGAACGAATCCGGGGGTGCAGGACA
TGAGCGCAGAGGTGGTGGGCAGCAACCCCTAGTTCCCTACAGAAGTCCCACGGTGCTTGGTGCTGCAGCGGATGGGCGTCGTCGACAACTACGAGCGCAT
CGAGGACTACTCCGTCGCCATCATCATAAGTGCCTTTTTCTTAGCCCTGCTCTTCAATTGGTACTGCTTTGCTTTAGTTTGGTTACATCGGCTTCTGAAG
GTCAAAATCGGCATAGACTGATAGACCTGGTCTGTACAAGTTGCTTCGGAGTAGAGGAGGTATGGATCTTTCGAAACTCGAAATGTGTTGGCTCCGACCA
CCCATCTCGTCATGTCTAATAAGGAAAATGCAGTGATAATACATGTTTTGGACAGCCATTTGTTGTTATCATTTTAGCATTAATTTGAGCAGAGGATTCA
AAGCTTCATCACAAAAAAAATCATAGCTCAAAAGATTCAGTTGATGTTAAATTGTTAATGAGTGTGTAAGAATTCCAAATCAGAAAAAAAGTTTTTAACT
CATTGCCATATGCTCCACACGGTATAGGTGGTATTGGTACTGCTGTTAGGATTTCTGTGTGGGCCATCTTTACTTCTAACCAAATATCTATCATCAGCAG
ATAACATACTCTATGTTGTATTGCCGCCCTGCATATGCAGATACCAAATTAGGTGTGAATTCTCATTCTGCTAATTTGCAGGTTTAACTATTACATTGCA
ACGAAAACTTTGCATCTTGGGTCTATTTTATCCTATTAATTTCCTTACCTACTGAACATCATTTGAGCTGAAAATTGCATCTTGCAGGTTACTGAAAATT
GCTGTTTTAGTTCAACTGCAGTATGCAACACGCATACATGTTTTGTTGGAATTGTTTTGCAGCCCTAAATTCCTGAAATCTTTTGGCTTGGTGCAGCATC
TCTGTAGAGTTGGGGAGCTGAAAATTGCATCTTGCAGAACACTACGTGCATCCATTATTGAGAGGGGAGCCCGTTCATGGTTGCCTACTACCCAAGGCAG
GGCACAGGGGAGTTCGTTGTCCCGCGCAAGGAGGTGAAGGATGCGCTGACAACCCACTGGGAGCCTGAGATGGAGGTGCACATGCAGGTCATAGAGGCCG
AAGACGCACGCCGCACCGATTGGGCCAACAGCACCGTCAAGGCCCTCCACCCATCCATCTGGCGCGCGCTTGAGATTGAGTGGGATGACTCCACGCCATC
TTCATCCACAAAGAGCAGACATGTAAATGTTTGGCAAGTCCAATTTGTTGGATACCCTCCTCTTGTGAAGAGACTCAGGTTTTCTGACACAGTTGGTGAT
GACCCCATGACTGCTCCACTGATTATTGGACCCGAAGGCATACTACTGGGAGCACCAATTCCTGCAGCTGGCATGCAGGGAGCCAGGCACAACGTCCCTA
CTGATGTACCCTCTTCTTCCTCTGCAATGATCACAACTCAGCAGCTGTTCCCCATGGATCTTCCGATGCCACCGAGTCTAAGTGGTGGCTCCTCTGAGAT
TTTGGATCCTGAAATTGGCTCTCCTCCCAACAACTCAGTAAACATGCATCCATCCAACATTCCTGTGGAAGCGAGGAGCATACAGCTTTTTGGCACCACA
ATCACACCCCATGTAGAGCAAAGTGCTACCGATGGTGCTTCTGAAGAAGTGAATGAAGTTCCTGATGGTGTGGTTGATGAAAATGCTGAAGGAGTTGTTT
AAACCTTGCAGCTGTACTTGAAGATGGTTCGATCAGACTGGGACTAATACAAGGGCGTGCGGCTGCCTTTTGTACTGCAGGATCCTACCAAGTGTCTCGT
TTATTCCTCGTCCATCTGACGGGATTGGGTGTTTCTGATGCATGCAGTTTATCTGTAGTTTTAGTAGGGTAGGCTGGGCCGTTCACCCCCCAGCGCCCTG
TTGAACTTGCTACAGGTTGTTACCTGGAAAAGAGGAGAACTTGGGTGTAGTTGAACTATTGCTACTTGTTACAGAGGAGCTGGCGCTGCTCTGTTCCATT
TAGTTTGCTAGACTAATGTTGATTCTGGGAGACTCGTATTGGTCTAGTCTAATCTCTTCCTTGTGGAACTACTATTTTTGTTATCTGTCTGTCA
>Locus_2698_Transcript_8/112_Confidence_0.109_Length_1002
ACAACGCAGGCATACAGGGAGCCAGGCATGCTCAATTTGGTATATCTTTATCAGATCTCCACCTTAACAAACTGCAGTCGAGTCTGTCACCACACGGGCT
TCACCAGCTCGATCACGGCATGCAGCAGAGGATCGCTGCTGGGCTTATCATCGGTCATCCGGCGGCTCGAGATGATATTCCCTGCTTGCTGACTATTGGT
ACTCCACAGAACAAGAAATCTGATGTCAAGAAGGCGGCGGCACCGCAATTGCTTTTCGGAAAACCAATACTCACTGAGCAGCAGATATCTTTAGGTAACG
CTGGGGGCTTCCCTCTGGGAGCTGCTAAGAAGAGCCCTTCTGATAGCAATGCCGAGAAGACGGTGAGTAACTCGGATATTTCAAGCCCTGGAAGCAATCA
AGATGGCACTACAGAGAACTTGTCCTGTGGAGGAGCCCCATTGTGCCAAGATAACAAGGTACTTGATCTCGGGTTGGAAACCGGACACTGCAAGGTGTTC
ATGCAGTCAGAGGATGTTGGGCGCACACTTGATCTCTCTGTTGTTGGGTCATATGAGGAGCTATATCAGAGGCTTGCTGACATGTTTGGCATCGAGAAAG
CTGAGTTGATGAGCCATGTTTTCTACCGCGACGCATCCGGGACACTGAAACACACTGGTGATAAACCTTTCAGCGAGTTCACCAAAACCGCGCGTAGGCT
GACCATACTAACGGACACAAGCGGTGATAGCTTAGCGAGGTAGGAAGGCCAAAAAGCTCTCGTCACTTGTACAGCTGGAGGAGCGTGGTGGCGATTTTGA
AGGCAAATTGCCACTTCCCAGTGCTGTTGCAATTTCAAAGAGAGGCAATTCAGTTCGGTTTTCTTCGATTGGAGCTCCCGGAACGGGATCGAGTGTAATC
CAGGACCCATAATTTATTGGCAGATTTTATCAGACCCTATTGTCTTTTGAACTGATCATAGCATTTGCATTCTACCACTGTGTAATGTACTCTGCCACGA
AA
>comp78295_c1_seq16
ACAACTGCACAACTTGCAAGAAAGCCACAAGCAACTAATGGGAGAGGAGCTTTCAGGCCTAAGTGTGAGAGACCTCCAAGGTTTAGAGAATCGTCTTGAA
ATGAGCCTACTTAGTGTCAGAATGAGGAAGGTCATATTCATTTTATTTCCACATTCTGTTTTTCTGTTCACTTCAATCGAATATGCAGTAAACATCTATC
AAACAGGGCCACCTTTTGACAAGTGAAATTGAAGAGTTACATAGGAAGGGCAGCCTAATTCATCAGGAAAACACGGAACTCTGTAGAAGAGTGAATATCA
TGTCACAACAAAAAATGGAACTTCATAGAAAGCTCCTGGCCAGTGAACCAGGAGGTGTTGCTGATGCAAATAAAAGCTCTAGCACTCCCTACGGCTTTGG
TACAGCACAAGGCGCAGATGTCCCAGCTAATCTTGAACTGAGCCAGTCATCACAAAAAGAGGGAGAGCAATGCAAAACAGGGGCCCCGGAACTGGGACTT
CAGCTGCACTAAGGAGAAGCAGTACAAGCTGGACAATGCCATAATGGTCCACAAATTTCACCATGCGATGCAAGAGCATTTTGGTTGCAATGACTATTTT
GTGCAATATACTAGCTGGGTAAACAAATGGGTTGTGCAAAGACTACAGATAGGACCAGTTATGTTATCTGAGGCTCTGCTGTGTCTAAAATGCAACATAT
TCGTTATTGTAATGACACATGATAAACTGACTGTCCTACCGTTGACCTTGAAGATAGCTCTGCAGTGAAAAGGTGTATTATGGGCTTGCCAATATTAAAG
AAGTGTTAGGGATCAACTAGTAGGATCTCACAAAGAAGTAATTAGTCCACATTAGTGCCAGCTGCTGGGGATGATATCTGACTCAGCCTGCCAACCCTCT
CTTAGCCATGGTGTTTCAACTAGTATTAGAAAAGAATGTTCATCCAATTGAGTTCCCAAACAACGAAATTTATATTATGTGTTGCAAAGTGTCGACACCA
GTGGAACAGAGTCCATATATGTCAAGCAAATGCCAAATGTCAATTTTGCTCCAGTGCACGTGGATTCTGTTCTATTCTCAAG
>comp79901_c0_seq9
GACGCGGCGCGGGCCGAGGTCCCGGAGCTCGCAGTACAGGGGCGTCACCTTCTACAGGAGGACGGGCCGGTGGGAGTCGCACATCTGGGATTGTGGGAAG
CAAGTCTACTTAGGTGGTTTCGACACTGCTCACGCGGCTGCGAGGGCTTATGATCGTGCAGCGATCAAGTTCCGAGGGCTCGACGCGGACATCAACTTCA
CTTTGAGCGACTATGAGGATGACTTGAAGCAGATGAGCAATTGGACAAAGGAAGAGTTCGTGCACATACTCCGCCGCCAGAGCACGGGGTTCGCGAGGGG
GAGCTCCAAGTACCGCGGCGTGACGCTGCACAAGTGCGGCCGCTGGGAGGCGAGGATGGGTCAACTTCTTGGCAAGAAGTAAGCCAACCTCCTGGCTTTG
ACCTTTCTACTCTGTTAGCCTAGTTAAGTGTTTTTTACCCTGGTTATATGAACAAAATATGTCGAAATGATGTTCAATTAACTTGTCATTTCACGTTGGG
TTCGATCAGGGCTATATCCCTTATGTTCATGTTAAGTAGTTTCAGGTGATTGTAGAGAATACAACAAGGTGATCTTGAAGTATCACTGATTATAATCCAT
GCTACTAGTAGTTTGAGCGTCTCTGTTGCCGGTGAAATCTCCACCCCTCTTGTGACTTTACCCCTCTACCGTTGAATCCGGGAATCCGGAATGGACCTAC
AAGATCGAGATATGTGCCTTGATGAACAGTGTTTCGGTGAACAGCAAAATCGGCACCCGTGCACAGTGTCCTGATGGACGCAACCTCTAGAGTTCACATA
TATGCATTGGCCAACAATGCAATTCCTGCTTTTTAGTCAGGAACCAGTTTTGTCAAAGTCTACGACACTAGGCATCCCTCTGTTGAGACATATCTCAGAA
CTGGAATTTACATGCAGGTACATCTATCTTGGACTCTTTGACAGCGAAGTTGAAGCTGCAAGAGCATATGACAGGGCAGCCCTTCGCTTCAATGGGGGGG
AAGCTGTTACTAATTTTGGGCCTAGCTCCTACAATGGAGGAGATGCTCTACCCGACACCGAAAATGAGGCAACTGTTGATGGTGACGCGGTTGATTTGGA
TTTGCGGATTTCACAACCTAATGTGCACGACACTAAAAGGGACAATACCTTAGCTGGAGGCCAGCTAACATGCGACTCCCTTGAATCTTCAAACACGATG
GCCTCTCAGGTAAACTGACTTCAAGACTACACAGCAGGGAAAAATGCTCGGTTTCTCTATGACTTTCTACTACTTGCAGTAACTAAAAAAGTTTCCCAGA
AAAATAAAATCTTTAAGATGGAAGCAACTAGGTTGAATTGTCTTGTATCTTTGCGTGGAATCCAACCTTTGATAGTCCCTACGCTATAAAGATTCATAGG
AGTATTTGATTGGTGTGTCGTCGGTTGAGATATTAACTAATTGGTCCAGGTCCTATAACCTTTATTGAACTTACGATATTTGCACCTATCTGTTGCTCCT
GTAGCCAATGAGCTCATCGTACCCGTGGCCTGTGTATCACCAAAGCACGACAGCAGTACCACCTCACCATCAACGCTTGTACTCATCTGCTTGTCCTGGC
TTCTTTTCGAACCTCCAGGTATTACCAAGCTAAAGCCTATTTTTTATGTGCAAATTGCTACTCCTACGTGATCAATTAGTTCAAACTGTTATTCGAATCC
AGGATTGTTTACTGAATATGTCCTGTCTTCAGAAAAAATAACACAAAAAGGAACATAATATGTCCTGTCTAATTGTGGACTGATACTAGCCAATTAAACA
GTTTCCCATATCCATTTCCTGTGTTTATTGTTTTGATTTGCTGGCATGGTTAAACTATAGTAGCTTGATTCGATAAAAACACTAAGCAGGCTTTGATATT
TTGAATGAAATGTCGTTCTTGGAAATACAGCCTTTTCCTTGGTATATCAAGGACAGTTGTCCATCTGCACTCTGCACCCTCAGCTTGTGTAACTGTTTGC
CTTGTGAACTGATTGATGTGTCAATCAGGCAGTTCTGCCAGTTTGCATCTTTTGAAATAGTGCAGTCTTCCAGTCTAACAGCTCCATGACATAACTTGGG
```

ATGCATGCTGCAGCTTTCTTACTGAGGATGCTCTAGATTTACAGAAATTCTCGCAGTTAGCAGAGGGCTATCAAATCTGATTTGCTTAGCTTCCTGCAGA
CAATAAGATAAGAACTCTAGAATCCTGAAGAAATCTTACTTGTACATGTTTACCAAAGTATCCTTACTCCTTAGTTGAGCCAATTTTGGTGAAGAACCGA
TAGCTCATTACTCTGTTTCCTGGGAATGATGTGGGCCTAAACCATTCTGTGGTCAAAGTTTAACAAAGCCCATGCTTGTGCTGTTGCAACTGCAGGAAAG
GCCAATGGAGCGAAGGCCTGAGTTGGGTACCCAGTCATTCCCCACCTGGGGATGGCAAATGCAGGGCTCCCCTCACATGCCGTTGCATCACTCTGCAGCA
TCATCAGGATTCTCTACCGCCGTCGGCGCAAACGCCGGCGTCTCGTTGCCTTCTCACCCGCCGGTGCCGTTCCCGGACCACCAGTTCTACTTCCCACCGA
CGGCATGAGCTTGTTGGTCTGGTTCAACTCTTGGGACGGCAGCTGATCTTATGTGTGAACATTTCGCAGCTTGCCGGTGACCGTTGTTAATTAATCGGGG
AGAGAGAGCCAGAAGACCCAGTATGGCTATCTCTCTGTGGCCCGTGCCTTGTTATGATCTTAGTTATTGCTACTACACCGTGGATTCATCATGGTTTATG
AGATTCTTTACTCAAGTTTCAGCTTAAATGTCCAAATACATGAATGCATCATACACAGGATCCGTCATTTTCTAAAAAAATACAGGATCTGTGTGAAGTG
T
>comp75655_c1_seq1
AAGCGGGTGAGCCCGTGGCTGATCGAGCTGGTGTCGAGCATGCCGGCCATCCACCTCGCCTCCTTCTCGCCGCCGCGCAAGAAGCCCCGGATCCCGACGT
ACCCGGAGTTCCCCTTCGAGGGGCAGCTCCTCAACCCGGCGTTCCCCCCAAATTCACTGGCCCGCGGCCACCACCACCACCACCATTTCCTTCACACCCA
CCCGTCGTTCTTCCCGTTCCCGGACGGCAGTGCTCCTGCAGCCATACAGGGAGCCAGGCATGCGCA
>Locus_12535_Transcript_43/73_Confidence_0.349_Length_2396
TTCCAATAAGACACAGATTCTTCAGAAAGAGGTTTTCCTCTCCCTGCCCTTTTGATCTCCAACTATGGCAGTGGTGACAAGGAGCAAATTTGCAATCTTA
GGGTGGTTCTCGTCGTGAAACCTGAAAGTTTGGGGAAATAAATTTGGGGGTGTGGATGTCATGATATGTAGGGTCCGGCACTGAAGTGGTTGATGCTGTG
GTTTCCAGGTTAAGGGGAGAAGAGGAATAGGATATAGATCTCAGGAAGCCAGAGATGAGGTCGAGGGGAAGCCAGTGGCCAAGCTTGTGCTCCAGTGAAG
CTGATTCTCTTGTTCCCAGTTGATCCATGCGCCGTGGCCTCAGTTCGCAGGATCAGCAGCTCCATGGTTCTGTGTGGTGCTGCAGTTTGAGAAGAAGGTA
CTTGGTCTTTTGCACCTTGATGTACTTGTGTTTTTTTGCCTTTCTTGATGCTATAATGATGGTGGTGCTGTTCTTGCCATGATTGACGATTCCCCTAGAA
AAGGTTGAACTACTCTGCACTCTCTCTGGCACATGGTAGCACCTCGTGTACTTCTCTGATGATGTATAGTTGAGTGCTCAGATATGTTGTTGCAAATCTG
TCCAATTTCTGCTACCCATATGTCAAATCTTTTCTGTTTTTAGTCTGTTTCACTCTTCCATTTTGTTGCTTCAAAGGTAGCTGCTTTTGCATACAATTAG
GAGCAGCAGTTGAGATATTCAATTCGGGCATGGGTTCTTTTGGGATGGACTGGAACCAGAAAGGCTCGGTGTTGTGGGATTGGGAGAATTTGCCGCCGAT
AGGCAGAAATGCAAACGAGAACCTCAAGATTGCGCCACAGGCTGAACCGAAGTTTGCAGGTGTTGAGGCAACAAGGCATGAATCGGGGCATTCTTCTTGT
GGTACTTTCTCTTCCAGCTCGGAGATGGGGTATGGTTCATCCAAGAGTTCCATATCAGCGTCGATTGATTCTTCACCCAAGGTGGGAAACAACGTGGAGC
TCAATCTTGCAGCTGTCAAAGTGCCTGACAAAAACACCGTCAAGAACACTGATTTGGGTAAAGTTGATGACGCTGGAACTTCTCCATCATCGATGATAGC
CGTCAGCAGTGGAGAACCGGTGATTGGCCTGAAACTTGGCCAAAGAACCTATTTTGAAGATGCATGCGGAGGGCAAAGTGTCAAGAGTTCACCATCCAAT
GTGAGTGCAGTGACCCCTGCTTCTGCGAAGAAAGCAAAGATGATTCAAAACGCACAGAACTCGTACTGTCAGGTTGAAGGTTGCAAGTTTGATCTCTCTT
CTGCTAAAGATTATCATCGGAAGCACCGAGTCTGTGAAACTCATTCTAAGGCTCCCAAGGTTATTGTTGCTGGTCTGGAGCGACGCTTTTGTCAGCAGTG
TAGCCGGTTCCATGCTTTAACTGAGTTCGACCAGAAAAAACGAAGCTGCCGGAGACGCCTCAATGATCACAATGCCCGCAGACGGAAGCCACAGCCTGAA
GCAATTCCTTTCGGTTCATCAAGGCTCTCTGCAATGTTTTATGATGCAAGGCAACAGACAAGTCTTCTGTTTGGTCAAGCTCCTTATGGTCAAATGAGAA
GCTGTGCAAACTCTTCATGGGATAGCCCAGTAGGAGGCTTCAAATTTGGAGAAACAAAAGCTCCTTGGTTAAAGCCAACGAGAGCTGCAGGTGTTGATGG
GATGCATGTATCAAGCCAGCAGGTGTGGAACAATATTACGCCACACGGTGCACATCATGATTTTAATGGTATCATGGCTTTCAAGGGAACCAGTGCAAAT
GTCCTTAATCAAGGTGCCGAAGCTTCTCCGGTCGTCTCCAACTCGAATGGAGCCCCAGATCTTCAGCGTGCTCTCTCTCTTCTGTCAAACAATTCAGCTG
GTGCTGCCAACGACCAGCCAACTCCTCAGCTGCACCCTGGGCTGACCACCCTTGCCAGTACCTCCAACCCTGTCACTTTGATGCAAGCCTCACCACCAGG
ACTCTGGCAAGATGGCGCCCCCCTCGATCATCAGGCGCGGTTTCAGCTTTTCGATCCGGTGGGTGACGGCAGCGCCATTGTCACACCGCATCAGCTCCAG
CTCCCAAAACCTCACTCCCTCAACAGCTCCTCTTCCCACTATGACCAGATGCACTGATGATCTGATGCTTCCTTCAGTTAGCGCAAATGGCGGTTGGGTT
GCGCAAAATTAGACATGGCTTCTGATGCCTGAAACTCTGAATTTTGAAACTGTTATGTTCTTCTGAAAAATAAAAGCCTGTGTGCGCAAACTCAGGCTCC
AAAAACTGATCTAGTAGTGTTCCCATTCAAGGGCTTGAATAATCCATCCTTGTGGTGGCATGTAACTCAAATGCTTCGATTGCGCCGGCAATTGAT
>Locus_2827_Transcript_99/166_Confidence_0.017_Length_392
TGCTGCGCAAGTGCCCCATCCTCGCCAATATCGAGCCCATGCAGATCAAGGTGCCGTGATAAGCAGAGGAAGAAGTCGTTGAGGCTCCACGCTGTGAACA
GCAAATTAACTGCGATGAACAGGCTTGTCATGGAAGAGCATAAGCAGCTCCAGAAGCTGGTCTCCCAGCTGGTTCATGAGAATGAGTACATGAAGCAGCA
GCTGCAGAATCCGACATTGCCAAATGATACAAGCTGTGAATCAAATGTCACCACTCCTGTGAATCTAAGGGATGCAAGTAACCCATCGGGACTCCTTTCA
CTTGCAGAGGAGACGTTGACAGAGTTCCTCTCAAAGGCTACAGGGACTGCTATTGATTGGGTCCAGATGCCTGGGATGAAGCCTGGTCCGGA
>comp79373_c0_seq15
GAAACCTGGTAAACCCTGGCCACGGACGCGCATGCCATCCAAGATGACGTAGTCCACACCGAATCTTCCACGTCCGCACACGCGAGCACAACGCCACCGC
AGGCCAAGTGCCGTCTCCACCACAACTCGCTGATCCCTTCCTCCTCGCCGGCTCTCTCTCCATCCCTCGTCCTTCCCCATCTGTCCCTCCCTTGGCTTCT
TCCTCCAAGCTCCAACCAATCCCCACGCGCGCGCGCGCTATAGCCTACAAATAAATACTCGCCACTCTTCCCTTCCTCCCGCCTATCGTCTTGCCAAGCG
CTAGAGCAGCTGCGCCATTGCACGCTTCCGGCCCGTCGTCCTGCACATCTGTCCTCCCCGTACTGCCTTGTACAAACTCCGTATGTAAACTACTGGGTTG
TCGCCAGTGTTCGTGTTTGCTGATGCTCATCTAATTATCGTCGAGGAGATCCAAGGAATTGTCCGCGGAGCGCCGGGGAACAAAGGCTGCGCGCGGTACT
ATATCAATTACAAACTGATGAAGAAAATGTTAAAGCAATATGTTCAACAGACCCAACTTGGTGGGAAAGATCATGAACAAGTTCTTAAAGAGTTCTCAAG
GATTCTTGATGACCAGATTGAAAGGATTGTGCTTTTTCTGCTACAACAACAAGGCCACCTTGCCAGCAGGATTGAGGAATTGGGAGAACAACGCACTGCT
CTTATGGAACATTATGATATATCACAAGTTTCCCAGCTACGTGATGCGTATAGAGAAGTTGGGAGAGATCTTATTAAGCTTCTGCGCTTTGTTGACATGA
ATGCTACTGGTATACGCAAGATACTAAAGAAATTTGATAAGCGTTTTGGCTATAAGTTTACATATTATTATGTCACCACTCGTGCAAATCATCCCTATTC
TCAGCTTCAACAAGTATTTAAGCAAGTGGGAATTGTAGCTGTTGTAGGTGCATTATCGCGCAATCTTGAATATCTGCAACATCATCAAGGAAGCTTTGTA
TCCATCTATGATCATCCATCAGTTACCTTGAAGGACCCTATAATAGACGAAGTAAACCATGCGGTACAGAAATTTACGCATGCCACCAATTTTATGAAAT
TCTTGGGACAACACGCGCTTATTGTTCCAGAGGATGCACGAAGCGGGTCTGAGGATCTTGTTGATGATCAGAGCTACCATTTCATGTCTCTGCTGCTTAA
CCTAGCGAACACTTTTCTTTACATGGTGAACACATATATCATTGTGCCAACTGCAGATGACTATTCAGTAAGCCTTGGGGCTGCTGCGACTGTCTGTGGT
GTAATTATCGGATCAATGGCAGTCACTCAAGTGTTCTCCTCGGTTTATTTCAGTGCATGGTCAAATAAGTCATACTTCAGACCACTTGTATTCAGTAGCA
TTATGCTATTTTTTGGGAACCTGCTATACGCATTGGCATATGACCTGAATTCATTAATAGTTCTCCTGATTGGACGACTACTATGCGGGTTGGGCTCTGC
AAGAGCAGTGAACCGTCGCTATATTAGTGATTGTGTGCCTCTCAAAATCAGGCTACAAGCTTCTGCAGGATTTGTCAGTGCTAGTGCTCTTGGCATGGCA
TGTGGCCCTGCTCTTGCTGGTTTCCTCCAGACAAAATTTAAGATATACTCGCTCACTTTTAATCAGAGCACATTGCCCGGATGGGTCATGTCCATTGCTT
GGCTTCTTTACTTGTTGTGGCTGTGGTTTACATTCAAAGAGCCAGAACACTTCGCTAAAACTGTCATCAATGCACAGCCGTCTGAATCAGGTCACCAAGG
AAGTGCTAATTTGGAGGAAGGTCTAGCTCAACCATTGCTTCTGGGTACAGAACAAAGACAGGGCGAGAATGCGGATGACAATGATGATAATGAAGTAGAC
TCTGAAAACTCTCATGAACCAGCAACATCAATTACTTCAGCATACAGATTGCTGACACCATCTGTGAAGGTCCAGCTATTGATATACTTTATGCTCAAGT
ATGCTATGGAAATTTTACTATCTGAATCAAGCGTTATCACAACATACTATTTTAGCTGGTCTACGAGTGCTGTGGCTATCTTTTTGGCAATTCTTGGATT
AACTGTCCTTCCAGTAAATGCCATTGTTGGAAGCTACATTACAAATTTGTTTGAGGACAGGCAAATTCTGTTGGCATCTGAAGTCATAGTTCTCATTGGT
ATCATCATGAGCTTTCGTTTCACCCCTCACTACTCCATTCCACAATACGTCACTTCAGCTCTCATCACATTTGTGTTTGCTGAGGTACTCGAAGGAGTGA
ATCTGTCCTTGCTCTCACGAGTAATGTCGTCTAGGCTTTCGCGAGGGACCTACAACGGTGGACTCCTCTCGACAGAGGCCGGGACATTGGCCCGTGTAAT
TGCAGACGCGACTATTACTGCAGCAGGTTATCTAGGCCCGGACCTCCTCCTCAACGTCACCCTGCTGCCACCTCTAGTGATCTGCATAGTGTCCATCGTC
GCAACGTTCTGCACTTACAACAACCTTTGCTGATAAAGCAGCCCCTCTGTGTCCAGCCAGTTAAAATGACTCCTGCCATATTGGGCCGTACCGTACTACT
TGAAGTAGCATCAGCGTTGTAAATCTGGTAAATCTACAAATACCATGGACGGTGCCGGTGTGGCGGTGTGTAGTGTATTGTTATGCATTCAACAACTTTC
TTGTGTCTAGTAGAGTTAAATATTCGTCAAATTGAAAATTCAAATGCATTAACCAAGGAACATCACTGTGTTGGAAAGACAAGATGATTTATTGCAA
>comp63680_c0_seq4
AGTATCTCGGCGTGGGACAGAGGCCGGTGGTGGTCGCGAAGAAGACGCGGCGCGGGCCGAGGTCCCGGAGCTCGCAGTACAGGGGCGTCACCTTCTACAG
GAGGACGGGCCGGTGGGAGTCGCACATCTGGGATTGTGGGAAGCAAGTCTACTTAGGTGAGTAACTGAGTTGAGTGAGTTCTATACGTTCCAGCTCGAGC
TTCCATTAACTGCTGATCAGTTTCCGTCCGTTTGATCTGGTTTACGATGTGCTGATGCTGTTCTATTCAGGTGGTTTCGACACTGCTCACGCGGCTGCGA
GGGCTTATGATCGTGCAGCGATCAAGTTCCGAGGGCTCGACGCGGACATCAACTTCACTTTGAGCGACTATGAGGATGACTTGAAGCAGATGAGCAATTG
GACAAAGGAAGAGTTCGTGCACATACTCCGCCGCCAGAGCACGGGGTTCGCGAGGGGGAGCTCCAAGTACCGCGGCGTGACGCTGCACAAGTGCGGCCGC

TGGGAGGCGAGGATGGGTCAACTTCTTGGCAAGAAGTAAGCCAACCTCCTGGCTTTGACCTTTCTACTCTGTTAGCCTAGTTAAGTGTTTTTACCCTGG
TTATATGAACAAAATATGTCGAAATGATGTTCAATTAACTTGTCATTTCACGTTGGGTTCGATCAGGGCTATATCCCTTATGTTCATGTTAAGTAGTTTC
AGGTGATTGTAGAGAATACAACAAGGTGATCTTGAAGTATCACTGATTATAATCCATGCTACTAGTAGTTTGAGCGTCTCTGTTGCCGGTGAAATCTCCA
CCCCTCTTGTGACTTTACCCCTCTACCGTTTAATCCGGGAATCCGGAATGGACCTACAGATCGAGATATGTGCCTTGATGAACAGTGTTTCGGTGAACAG
CAAAATCGGCACCCGTGCACAGTGTCCTGATGGACGGTACATCTATCTTGGACTCTTTGACAGCGAAGTTGAAGCTGCAAGAGCATATGACAGGGCAGCC
CTTCGCTTCAATGGGGGGGAAGCTGTTACTAATTTTGGGCCTAGCTCCTACAATGGAGGAGATGCTCTACCCGACACCGAAAATGAGGCAACTGTTGATG
GTGACGCGGTTGATTTGGATTTGCGGATTTCACAACCTAATGTGCACGACACTAAAAGGGACAATACCTTAGCTGGAGGCCAGCTAACATGCGACTCCCT
TGAATCTTCAAACACGATGGCCTCTCAGCCAATGAGCTCATCGTACCCGTGGCCTGTGTATCACCAAAGCACGACAGCAGTACCACCTCACCATCAACGC
TTGTACTCATCTGCTTGTCCTGGCTTCTTTTCGAACCTCCAGGTATTACCAAGCTAAAGCCTATTTTTTATGTGCAAATTGCTACTCCTACGTGATCAAT
TAGTTCAAACTGTTATTCGAATCCAGGATTGTTTACTGAATATGTCCTGTCTTCAGAAAAAATAACACAAAAAGGAACATAATATGTCCTGTCTAATTGT
GGACTGATACTAGCCAATTAAACAGTTTCCCATATCCATTTCCTGTGTTTATTGTTTTGATTTGCTGGCATGGTTAAACTATAGTAGCTTGATTCGATAA
AAACACTAAGCAGGCTTTGATATTTTGAATGAAATGTCGTTCTTGGAAATACAGCCTTTTCCTTGGTATATCAAGGACAGTTGTCCATCTGCACTCTGCA
CCCTCAGCTTGTGTAACTGTTTGCCTTGTGAACTGATTGATGTGTCAATCAGGCAGTTCTGCCAGTTTGCATCTTTTGAAATAGTGCAGTCTTCCAGTCT
AACAGCTCCATGACATAACTTGGGATGCATGCTGCAGCTTTCTTACTGAGGATGCTCTAGATTTACAGAAATTCTCGCAGTTAGCAGAGGGCTATCAAAT
CTGATTTGCTTAGCTTCCTGCAGACAATAAGAACTCTAGAATCCTGAAGAAATCTTACTTGTACATGTTTACCAAAGTATCCTTACTCCTTAGTTGAGCC
AATTTTGGTGAAGAACCGATAGCTCATTACTCTGTTTCCTGGGAATGATGTGGGCCTAAACCATTCTGTGGTCAAAGTTTAACAAAGCCCATGCTTGTGC
TGTTGCAACTGCAGGAAAGGCCAATGGAGCGAAGGCCTGAGTTGGGTACCCAGTCATTCCCCACCTGGGGATGGCAAATGCAGGGCTCCCCTCACATGCC
GTTGCATCACTCTGCAGCATCATCAGGATTCTCTACCGCCGTCGGCGCAAACGCCGGCGTCTCGTTGCCTTCTCACCCGCCGGTGCCGTTCCCGGACCAC
CAGTTCTACTTCCCACCGACGGCATGAGCTTGTTGGTCTGGTTCAACTCTTGGGACGGCAGCTGATCTTATGTGTGAACATTTCGCAGCTTGCCGGTGAC
CGTTGTTAATTAATCGGGGAGAGAGAGCCAGAAGACCCAGTATGGCTATCTCTCTGTCGCCCGTGCCTTGTTATGATCTTAGTTATTGCTACTACACCGT
GGATTCATCATGGTTTATGAGATTCTTTACTCAAGTTTCAGCTTAAATGTCCAAATACATGAATGCATCATACACAGGATCCGTCATTTTCTAAAAAAAT
ACAGGATCTGTGTGAAGTGTAAACATAAGG
>Locus_3441_Transcript_379/417_Confidence_0.018_Length_562
TTGGGAAACCTTTTGAAGCTTTCTTCTTCCAGCTCCCGTCCCAGATCTCCCCCTTACCTTCTTTGGCACATTCGGTGCCGATCGCTCCCGGCCGGAGATC
CTGCCGTGTTCATCACCGTCGTCCTTGCTCTTTGAGGCCATCTCCATTTTCAGTGTGTGAGAGGAAGAAAAGTCGAGAGAGGAGAGAGAGAGAGAGAGAG
AGAGAGAGAGAGATGGGGAGGGGGAAGATTGTGATCCGCAGGATTGACAACTCCACGAGCCGGCAGGTGACGTTCTCGAAGCGGCGCAACGGGATCTTCA
AGAAGGCCAAGGAGCTCGCCATACTCTGTGACGCCGAGGTCGGCCTCATGATCTTCTCCAGCACCGGCCGCCTCTACGAGTACTCGAGCACCAGCATGAA
GTCGGTGATAGATCGATATGGCAAGGCCAAAGAAGAGCATCAGGTCGTCTCAAATCCCAACTCGGAACTTAAGTTTTGGCAAAGGGAGGCAGCAAGCTTG
AGACAACAACTGCACAACTTGCAAGAAAATCATCGGCAGTTGATGGGTGAAGATCTATCTGG
>Locus_2827_Transcript_85/166_Confidence_0.263_Length_3063
GTGCGGTACACGCCGGAGCAGGTGGAGGCGCTGGAGCGGGTGTACGCGGAGTGCCCCAAGCCCAGCTCCGCGCGCAGGCAGCAGCTGCTGCGCGAGTGCC
CCATCCTGGCTAACATCGAGCCCAAGCAGATAAAGGTCTGGTTCCAGAACCGGAGGTGCCGTGATAAGCAGCGGAAGGAGTCGTCGAGGCTTCAGGCTGT
GAACAGAAAATTGACCGCGATGAACAGGCTTCTCATGGAAGAGAATGAGCGGCTCCAGAAGCAGGTCTCCCAGCTGGTTCACGAGAATGAGTACATGAAG
CAGCAGATACAGAATCCGACATTGCCAAATGATACAAGCTGTGAATCAAATGTCACCACTCCTGTGAATCTAAGGGATGCAAGTAACCCATCGGGACTCC
TTTCACTTGCAGAGGAGACGTTGACAGAGTTCCTCTCAAAGGCTACAGGGACTGCTGTTGATTGGGTCCAGATGCCTGGGATGAAGCCTGGTCCGGATTT
GTTTGGTATTGTGTCTGTTTCACATGGTTGCCGAGGTGTTGCTGCCCGTGCCTGTGGTTTGGTGAATCTAGAACCAACAAAGATTGTGGAGATCTTGAAA
GACCGCCCATCTTGGTTGCGTGATTGTCGGAGCCTTGAAGTCTTTACAATGTTACCAGCTGGAAATGGTGGAACTGTTGAGCTTGTTTGCATGCAGATGT
ATGCGCCTACTACTTTAGTTCCTGCACGAGATTTTTGGACGCTGAGATACACAACCACTATACAGGATGGCAGTCTTGTGGTCTGTGAGAGATCTTTAAG
TGGTTCAGGAGGTGGTCAAAGTACTGCATCGGCACAACAATTTGTTAGAGCTGAGATGCTTCCTAGTGGCTATTTAGTCCGTCCATGTGAGGGCGGGGGC
TCAATTGTGCATATAGTGGACCATCTGGACCTTGAGGCTTGGAGTGTTCCTGAAGTTCTTCGGCCGCTCTACGAGTCGTCTAGGGTAGTTGCTCGGAAGA
TGACAACTGCAGCACTACGGCACATCAGACAAATTGCTCAAGAAACAAGTGGAGATGTCATATATGCCTTGGGGAGACAGCCAGCTGTTCTACGAACATT
TAGTCAGAGGCTTAGTAGAGGCTTTAATGATGCTATAAGTGGTTTCAATGATGATGGTTGGTCTGTCATGGGTGGAGATGGCGTCGAAGATGTTATCATT
GCTTGCAACTCAAAGAAGATAAGGAACAATACCAATCCTGCAAGTGCTTTTGGAGCCCCTGGTGGTATCATATGTGCTAAGGCATCCATGTTACTGCAGA
GTGTTCCACCAGCAGTACTAGTTAGATTCTTGAGGGAACATCGGTCTGAATGGGCAGATTATAACTTCGACGCATATTCGGCCTCGGCGTTGAAAACAAA
ACCTTGTTCCCTTCCTGGGTTGCGGCCTATGAGATTTTCTGGGAGCCAGATCATCATGCCACTTGCTCACACAGTGGAAAATGAGGAGATCTTAGAAGTT
GTTCGTCTTGAAGGACAGGCTCTTACACAAGATGAAGGTCTTTTATCAAGAGATATTCACTTGCTTCAGCTTTGCACTGGAATAGATGAGAAATCAATGG
GATCCTGCGTCCAGCTTGTCTTTGCACCTATCGATGAGCTTTTCCCCGATGACGCTCCATTGATATCTTCAGGTTTTCGTGTTATACCATTGGACATAAA
AACATATGGTGTACCCTCTGGAAGAACATTAGATTTGGCCTCCAGCCTTGAAGTTGGTTCAACTATGCAACAAGCCTCGGGAAATGCATCTCAGGATGAT
TGCAGCCTACGATCTGTGCTAACAATTGCCTTTCAGTTCCCCTACGAAGTGCATCTCCAAGATAGTGTTGCAACTATGGCCCGCCAATATGTTCGGAGCA
TTGTCTCTGCTGTGCAGAGAGTATCCATGGCTATTTCTCCCTCGCGGTCTGGCTTGAATACTGGACAGAAGATAATTTCTGGCTTCCCTGAAGCTGCGAC
ACTTGTTCGCTGGATTTGCCGAAGCTACCAGTATCACTTGGGGGTGGAATTAGTCCCTCACTCAGATGAAGCTGGTGAATCATTATTGAGAATGTTCTGG
GATCATCAGGATGCTATCTTGTGCTGCTCCTTCAAGGAGAAGCCTGTGTTTACTTTTGGCAACCAGATGGGAATTGATATGTTAGAAACAACTCTAATTG
CTCTACAAGATCTCTCCCTGGACAAGATCTTTGATGAACCCGGTAGAAAAGCATTATACGCAGAGATCCCGAAATTGATGGAACAGGGTTATGTGTACCT
GCCGGCCGGTGTCTGTTTGTCCGGAATGGGCCGCCATGTCTCCTTCGAGCAAGTCGTTGCGTGGAAAGTTCTTGGAGAGGACAGCAACGTGCACTGCCTC
GCCTTCTGCTTCATGAACTGGTCGTTCGTCTGATCGAATCTGGCCAGCCTGCGCGAACCTTACGACCAGCCTCCATTTTTGCGTTCTCTGTAGAAGAGAA
CTGTATGCCACGGACTTCGCGCTGTTAGGTGATCCTGTGATTCAGTTAGTTATAGACCTGGAAGAAAGTCAGCTATGTAAAGATGCTGTGCCCTTTGTAA
ATCTGTTCTAGTTCTCGCTAATTTAACCCGGCGAAATCTGGCTGTTGGTCAATGGGATGAAGGTGAAACACAAGTGGCAGTTTTCAGATGAATCGTATAT
CAGATCGCCGCCTTGCAGGTCTATTTGTTGTGCCTCTCAACTTGTTGCCCTGGTCATGGTGTGTAGGTCATGCTGAAGTCGCAGATGGCCGCCGGGCCAG
GGGGATAAAGGCCATCATCGCCGTCCTTCCCGCTGCAGTTTCTCTGCCTTGCTGCCACTGGATTCAAATCCGTTGAGCAGTAGCTATTTTTACTGCTGGA
TCCAAAATCAAGGGTTTTATTTAGTGCAAACACAATATCAAATTAGCTCATGTAACTATTATCCTTCTCATGATCAAATCAGCAAACTAGCTGTTTAAAG
ATCGGTCCCAAATCTATATGTGCAATATTCTTCAAAACATATTGTGCGATACTGGAAAACATT
>comp79584_c0_seq13
GGATTTGACACTGCTCATGCTGCTGCAAGGGCGTATGATCGAGCGGCTATCAAGTTCCGTGGTGTCGACGCCGACATCAACTTCAATCTCAGTGACTATG
AGGACGACATGAAGCAGATGAAGAGCCTGTCCAAGGAGGAGTTCGTGCACGTCCTGCGACGGCAGAGCACCGGGTTCTCGCGTGGCAGCTCCAAGTACAG
AGGCGTCACCCTGCACAAGTGCGGCCGGTGGGAGGCTCGCATGGGACAGTTCCTCGGCAAGAAGTACATATATCTTGGGCTATTCGACAGCGAAGTAGAG
GCTGCAAGGTTGCTGATCTTGGATTCTGATATTGATGCCAAAAAAAAAGTTTTTTTATACCTTTCCTGTTGATTTTCCAAAGGGTTGAGGACCGACTCGA
TTCTCTTCTTTCTTGTTTTCCTTCTTTTGGCTTCAAACCTCAGGGCTTATGATAAGGCCGCGATCAAATGCAATGGTCGAGAAGCTGTGACGAACTTCGA
GCCAAGCACGTATGATGGGGAGCTGCTGACTGAAGTTGCTGATGAAGGTGCTGATGTTGATCTGAACTTGAGCATATCTCAACCAGCTTCTCAGAGCCCG
AAAAGGGACAAGAACTCCCTTGGTCTGCAACTCCACCATGGATCGTTTGAAGGCTACGAATTGAAAAGAACAAAGATTGATGCTCCCTCTGAACTCACGG
GCCGACCTCATCGGTTCCCTCTTCTGACCAAGCATCCACCAATCTGGCATGCCCAATCTCATCCCTTCTATTCAAATAATGAGAATGCATCAAGAGATCT
TAACAGGAGGCCTGATCAGGGAGGCACTGTTCCTAGATGGGCGTGGAAGGTGACCGCCCCTCCTCCCACCCAACCATTGCCGCTGTTCTCGTCATCGTCG
TCGTCGTCCGCTGCAGCATCATCAGGATTCTCCAATACCGCCACGACAGCTGCCCCCACCGCCCCGTCGTCCTCCTCCCTCCGGTTCGACCCGCCGCCGC
CGCCGTCCAGCCGCCACCGCTGAACGTTAAGAAGCCACGCTGTAAATTTGCCAGGAAGCCGACATTTTTTCCTCTCTCGGCGTTGCAACTTTTTAGGTTT
TGCGCCGGGGTGGTTTCTTGTAGTGGAGTGGATTCATAATTAACTGTATTTGCATGCTGCCCATGTGAAATGCTTCTCTCTCTTTTTACGCTCTCTGCAC
CGGATGTTCTGGGAGTTCTTGTTGCCGGGAATTAACTAAATTCACCGTCTGAGATTGATCTATACATTGTTGTAGAGAATCGAATCATTGTTGAAATTAG
TTACAGAATTATAGATATCATAAGGCACCCTCTACTTGTGGACAAAGTTCAGGAAGATTATTCCGGTTCTTTTCTTGGCTGT
>comp88323_c0_seq6

TTCTCTCTCTCTCTCTCTCTGTTGCTCGCGACTGCACGCTGCTGCTGCTGTTCCACATCTCTGTCGCCGCTTTAAGGCTGTCACCTTTCCCCCCTCGCTC
TCCTTCTCTCTCCTCAATTACGACGCCACAACCAGACCCAAACCCTAGTACTACCACGAGGCGGAGTAGGTGTGCAGCCGGCGGCGCTGCCCCTTGATCC
GATCCGTCCGGCGCTGCGGGGGATTACGACTTGTGAGTGCTCACCGGCGATCGATGGGAGGCGCCGCGGGAGGCGGCTCAGGCGCGGGGAGGCTGTGAGG
TCGGTGGGTGTGCTGTGCGTCTGCTCCGGCGGCGCTCCCCTCGGCGAGCGGGAGCTTGTCGGTGGGTCTCGGCGGTGTTCGCTTCGTTTGCAGATAACTT
CGCGAGCTGAAAGTTGAAGTGTAAGCGGGGAGCTAAGAGCAGTAGTTGAACGAAGCGGAAGAGGAGGACGTGATTTGAGTAGTTGGGCTCTTGCATTGGC
GAGGAAATAATGGCTGCGGCCGTGGCGATGCGAGGGGGAAGCAGTGACAGCGGAGGGTTTGATAAGGTTCCGGGGATGGACTCGGGGAAATACGTGCGGT
ACACCCCGGAGCAAGTTGAGGTACTCGAGCGGCTGTACATCGATTGCCCCAAGCCAAGCTCCTCGCGGCGGCAGCAATTGCTGCGCGAGTGCCCTATACT
TGCCAACATTGAGCCCAAGCAGATCAAGGTTTGGTTCCAGAACAGAAGGTGTCGCGATAAGCAGCGGAAGGAGTCTTCGCGGCTTCAGGCTGTGAACCGG
AAGTTAGCGGCAATGAACAAGCTTCTGATGGAAGAGAATGAGCGTCTTCAGAAGCAGGTCTCTCAGTTGGTTCATGAGAATGCACACATGCGGCAGCAGC
TGCAGAATACTTCATTGGCCAATGATACAAGCTGTGAATCAAATGTGACTACCCCTCCAAACCCTATAAGGGATGCAAGTAACCCTTCTGGACTCCTTTC
GATTGCAGAGGAGACCTTCACAGAGTTCCTCTCAAAGGCTACTGGGACAGCTATTGATTGGGTCCAGATGCCTGGGATGAAGCCTGGTCCGGATTCGGTT
GGTATTGTGGCCATTTCGCATGGTTGCCGTGGTGTTGCTGCCCGCGCCTGTGGTTTGGTGAATCTAGAACCAACAAAGATTGTGGAGATCTTGAAAGACC
GCCCATCTTGGTTCCGTGATTGTCGGAGTCTTGAAGTCTTTACAATGTTACCAGCTGGAAATGGTGGAACAGTTGAGCTTGTTTGCATGCAGATGTATGC
GCCTACTACTTTAGTTCCTGCACGAGATTTTTGGACGCTGAGATACACAACCACTATACAGGATGGCAGTCTTGTGGTCTGTGAGAGATCTTTAAGTGGT
TCAGGAGGTGGTCAAAGTACTGCATCGGCACAACAATTTGTTAGAGCTGAGATGCTTCCTAGTGGCTATTTAGTCCGCCCATGTGAGGGCGGGGGCTCAA
TTGTGCATATAGTGGACCATCTGGACCTTGAGGCTTGGAGTGTCCCTGAAGTTCTTCGTCCGCTTTACGAGTCGTCTAGGGTAGTTGCTCAGAAGATGAC
AACTGCAGCACTACGGCACATCAGACAAATTGCTCAAGAAACAAGTGGGGAAGTAGTGTACGCCTTGGGCAGGCAACCTGCAGTCCTACGGACATTTAGT
CAAAGGCTGAGCAGGGGCTTCAATGATGCCATTAGCGGTTTCAATGATGATGGCTGGTCTATAATGGGGGGAGATGGTATTGAAGACGTAGTTGTTGCTT
GCAACTCAACTAAGAAAATTAGGAACAACAGCAATGCTGGAGTTGCGTTTGGAGCCCCTGGAGGTATTATATGTGCGAAGGCATCCATGTTACTGCAGAG
TGTTCCACCAGCAGTATTGGTTCGATTTCTGAGGGAGCATAGATCTGAATGGGCCGATTACAATATTGATGCATATTTGGCTTCAGCGCTGAAGGCCAGT
GCGGGCTCACTTCCTGGGTTACGGGCCATGAGATTTTCTGGGGGCCAGATGATCCTGCCACTTGCCCACACAGTGGAGAATGAGGAGATTCTTGAAGTTG
TCCGCCTTGAAGGACAATCTCTTACTCACGATGAGGCTCTTATTTCAAGGGATATCCACCTGCTTCAGCTTTGCACTGGAATAGATGAGAAATCAATGGG
ATCCTGCTTCCAGCTTGTCTTTGCACCTATCGATGAGCTTTTCCCTGATGACGCTCCATTGATATCTTCAGGCTTCCGTGTTATACCGTTGGACATAAAA
ACATATGGTGTACCCTCTGGAAGAACATTAGATTTGGCCTCCAGCCTTGAAGTTGGTTCAACTATGCAACAAGCCTCGGGAAATGCATCTCAGGATGATT
GCAGCCTACGATCTGTGCTAACAATTGCCTTTCAGTTCCCCTTCGAAGTGGATCTCCAAGATAGTGTTGCAACTATGGCCCGCCAATATGTTCGGAGCAT
TGTCTCTGCTGTGCAGAGAGTATCCATGGCTATTTCTCCCTCGCGGTCTGGCTTGAATACTGGACAGAAGATAATTTCTGGCTTCCCTGATGCTGGGACA
TTTGTGCGCTGGATTTGCCGAAGCTACCAGTATCACTTGGGGGTGGAATTAGTCCCTCACTCAGATGAAGCTGGTGAATCATTATTGAGAATGTTCTGGG
ATCATCAGGACGCTATCTTGTGCTGCTCCTTCAAGGAAAAGCCTGTGTTTACTTTTGGCAACCAGATGGGAATTGACATGTTAGAAACGACTCTAATTGC
TCTACAAGATCTCTCCCTGGACAAGATCTTCGATGAACCCGGTAGAAAGGCATTATACGCAGAGATCCGGAAATTGATGGAACAGGGCTATGCATACCTG
CCGGCTGGTGTCTGCTTGTCCGGAATGGGCCGCCACATCTCCTTCGAGCAAGCCGTAGCTTGGAAAGTTCTTGGCGAGGACAGCAACGTGCACTGCCTCG
CCTTCTGCTTCATGAACTGGTCGTTCGTCTGATCCAATCTGGCCGCCCTGCGCGAACCTTACAAGCAGCCTCCGTTTTTGCGTTCTCTGTAGAAGAGAAC
GGTACGTCTCGCTGTTTGGTGATCCTATGTTTCAGCTAGTTCTAGACCTGGAAGAAAGTCAGCTATGTAAAGATGCTGTGCCCTTTGTAAATCTGTTCTA
GTTCCCGCTGCTGGTCTATGGGATGGTGAAACACAAGCCGCAGTTTTCAGATGTATCATATCAGATCGCCGCATTGCATTAGGTCTATCTGTTGTGCCTC
TGAACTTGTTGCCCCTACTGATCGACCAAGTAACTGAAACTACTGTTAGCACAAGCAACAGTCAACATAATGTGCAGTGCTATGTTCCCTGTTCAAAAAA
AAAAAAAAAAAAAA
>Locus_1016_Transcript_993/2070_Confidence_0.004_Length_4845
GTGGAGGCGGCAAGCTAGAGATAACAACTGCATAACTTGCAAGAAAATCGTAAGTTTCTCAATTTTTCTGTTCTATACATGAGAAAACGGTGTTGATTTG
TGTGCATGCGTGCGTTTGTCAATGATCTGAATGATTTGCACATGATTTGTGTTAACATGCTGTGAATTCAGGGTATGGCAAATTTGCCAAATATCTTTTC
AGGATGCTTAAATTCACTTTGTTTAGAACTGTTTTGAGAACCGTATGGATTCAATAGACACCCAATTACTTGGTTATAATTGAGTTATTTACTTTACGAA
AGGTCGATGTGGTCATAGCATATTAAAGTTCCTTTTAATTGACCAATGACCGTATGGTTACTCTCATTCCTTCAATTCATTGCTCTCCCAGTCACAAATA
AGTGATGTTTGGGACATTGACAAGGCCTCCAAAACTCAAATGACCACCTAATTCTGCCATAGTATATTTTTAGAACCTAATAATTTGTATATCATTTCGA
TGTGTATACATCATTTAAGTTTAGAACCCAATAATTTGTATATCATTTTGATGTGTATATATCATTTAAGTTTAGAACCTAATAATTTGTATATCATTTT
TAGAACCCAAACCTCAAAGACATATCGAGAGGAATCCATGTATATCATTTTGATGTGTTCAATGTAAATATTTGTAAAGATACCAGTAGCGAAAGTTTGA
CTACACACATTCCAAATGTATAGAGGTATAATTGGAGGTTCAGCCGTTCAGAGACCCAGGTCTTGGGGTCCAGGATCGTTTTGTGCTATAATCGTTTACC
CCTTGAATTTCTGTGATTAAGGTGATAATCTTAATTTACTGTGACATAAGAATAGGCCAAATATTTTCCTGTACAAACTTAAGCTAGTGTATCTGTCGTG
TGTCTTGTCATAGTACAATCCGTTATTTCTGGTTATTTTCGGTGCGTCAACGTCTTACTGTTGTTATTCCTTTGAACACTACAAAATTTTAAGTGAGCAA
CTAGCATCCGTATATTTACATATGTAGCTGAAGAAAGGTTGATCCCCATTCTGATACTCTGGAATGCCTGCACGGATAAACCAGAATGCAATTTGTGCTC
TTCTGCTATGCATACAATTCTCAAAATAGAAAAAAATAGCACCTGAATTTTTTCCACACACCATATCTTTTTGTCAATATAAGATTTGTAGCTATCATGT
ACTCACGTTGGTCAACAAACTTGCAGAATGTGTCAAATAGTTATAATACAAATCATTTGCTGTATTCTTCAATAAAATACATTTTATTTGAAATAGATTT
ACACTACCATTATCAATCACTCATGGCATGCATACCGCGTCAGTCTTGCTATGTGTTTAAGCTCTTTACAACCAATAGTGTTTCTGGTTGCTTTGAATCG
TACCGTTGAGAATAATGAACATAAGCGGTGCCATGCTGCATGCCTTCATGGATTTAGTTGTAATCTGCAAGTTTTCAGTTAGTAGTAAACAATATGCCAC
TAAAGAATTAAGAACAGAATCAATAGTTCCTTTGCAGAAAAAAGCAGGGTTACCCCAGTTTAGGAGGTGCAGTTTGCTCAACATCATTTTCTTGCGGTGG
AGTGTTAAGTTCAAGATGAACAGGAATGTTGACGTTCTCGACAACTGCAAAGTTTAGAGTTGATGAATCTCGGTTGACTTCACTTGTCACCTCTTTCTCG
TAGAGCTGAGTAGAGAAATAAACCTCAGTCAATAATGGATGGGGTGTATATCCTGTGAATTTTCATTCACGATTTATCAAATACCTTCTTGTATAAATCA
ATGTTTTCCTGACGGATTAGGTTGACCTTTTTGTATAGTTCCATCTTGTCTTGTTGCATGAGACTTCCCTGAAAATGAGCCAGTAGAGTCAGCTTCTTGA
AATCCACTCAGAGACCTCTCATTGCAGAGTAATAACAGAATATTCCAGAACCTTTCGATTCAGTTCGTGAATTTCATCAATCAAGAGTTGGTCCTGAAAG
TAAAAAACGTCATCATCAGTTGTATTCTTGGATGAGGCAATCTGGTAACGAAAAACAGAAATATGGCTAGCATCCTTACCTTTTTTGTCCGGATGCAACG
TAGGCTCATTTCTAGCTGATTTTCTAGATTTTGGAGTTCCTTGACGCTCAATCCAGAAAGATCTTGTCCCATCAACTGACTGCAGTCAATCAAACTGTAT
TCAGTTTACCTGATAACCTGATATTCCTCTGCCCATGTGTATTAATGTTGTGTTGGTTGGGAGTGAAGTGCGGACAAATCAGAGACTCAAGAAACTACCG
GGCAAAACAGATGATAATATCATATTTGTTTTCCATTAGTATGTACTCTTCTTCTTTGGAAGTTAATTGGAATGCATAAAAGCTAAATAGGTACATTTTT
TGACTTATAAATGGCTTGGCATAGGAAATTCATAAACTGTTACCTCCTTTTACATTGATATTGTTTATGAAAACTGATGTCCTCATAAGCAAAAGATTGT
CCTATGGAGTCAGTAAATCAGTATGTCTCCTTAAACTTGGGCATTGTATGCTATCTTGTATACTAAATTCATTCTTATCCATTGTTTTACAATTTTATAC
ACTCTGCACAAATTAGTTGGTGGAGGTATACTTGTCACTTTACAGCCCTTAATTTGCATCACCAAGAGTTATGGATGATCTGTAACATGGTGTAGTTGAT
TCATAATTGTCTCGTCAGAACCTAGTTTTATACCTTTATAAATAAGACAACCAGGAATTGAGTATTGCTACATGGTGAATATTGTTTCTTCTTTTGGTTC
TATTCTAATGTTGTTTTGAATTTTTGTATATTCTTGAAACAAACAGTAGGAAGGATTTCCTTCCTCCAGAGTTATCTAACTCGGAAGTTAGGCACTTGTG
CTCATGCTGACAAATCCTATAGTTTGATCCTTTTACATTAGGCAAATTTTCTGACTTGTACATTTGTAAAATTTGCAGGGTTTTCTAGCCATACCGATGA
CTTTCTTGCAGGTTGTGCAGTTGTTGTCTCAAGCTTGCAGCCTCCCTTTGCCAGAACTATTGAATATGAAAGCACAGTTAAGTAATGCCACATCCAGGAT
TGCCAAAATACAGCATTGGTATAAACATGTTGCATAGAAGCATGTCTGAGCTGTCAGCTAAACAAATTGTACCTTATGAAATAACTGAATTAAATAAGAT
GACATGCCACTATTTCAATCAAGAGATTTACAGTGCGCTACAAAGTAACTCTCTTTTTGAGAGGGGTGAGGGGGTGGCAACGATTATAATAGCAAATACA
AAACCTTTTAAACTTGAAGGTTGAGATTCTATATTATTCAAATACTTGAATTAAAGAACTTTATTATAGTGAACTATCAAGCTGGACACATATTGGCACG
CTACACTTGCTGTATTGTCTCCTATGCTGTTGTTCACACTTTTCACACACAATTGCTCCGACAACTGAATTGAGCTAAATTAGGGACAATTAACTTTTTG
CCACTCTGGAAGGGTGAGTGGCAAACAAAAATGCCACTGGACTCACATTTCAGTGACTCAGGTGACCTCACGTGTCAGTGGCAGCGGGTAATTGCAGTTG
TATCATAGTGGCAAAAAATCAATTGCCCCGCTAAATTAGCCTCGCAATACCTTGTGCTCAGGTCTAGGGACAAAACGGAACATGTCAGGATGGTGATGAT
CTTGCTGGAACATGTTTAAGCTGAAATTAGATGACTGTAGGTTTTCTCTTCGTTTTCTAGAATGTTAAAGGTCATCGTCTAAAATGTTTCACTATAAACC
GGAAAGGATTCATGCGGCACTCATTTTTCTGGAGAATGGACTAAAACATCTATTGACTGCTGATCATTTTCAGAAGATGAGAACTTGAAACAGGTACCCT
TTTTCTACTCTCTTTCGTGTTTCTTTATCAACTGTCTTTTTGTTAGTTTCATCTTGAATATATGTATTTGGGTTGAAAAGGATCACATGGTTTGGCTTCC
TCCAAAAAGAGGTTTGAGTATACGAGGCACTGCCTGGATGGATGGTTAGAAGGATTTCAAATACCGTGCTTACTGTTCATCGAGTAACAATCTGTATTAT
CTGTGCACATTTGCAGTCTATTTTCCCAAACTATGAACTGTACCGTACTTAGGTTCTGTGCATATGCAGCTCGACATTTTGGGTGTTTGGTGGTTGAATG

AACCTTCACCCTTCAATTTATCTGTAGTTATTTGTGATTTGAAGCATCTTCTTTATATCTGTTCCACACTGCTGCCAATTTGATTATTGATATAATAAAT
TAAGCTTTCAGATCGTATAGTAGAAATACACTATCTTTTAATGCAATCCTGGCTTTGCTTGGTATTTTATCTTCTCGAAATAACTTGTGTTCATTGGGAT
CTATGTCAGTGCTGTGTCAGCATCAGCTTCGACTTACTGGCAAGCTTCAGGGCAGGCTAAGCAAGGCCCCTAAGATTTCATCCGGACTACAGCTAAAGTG
AAAAAGGGTTTATTCATGATAATTTCAATCTAAGACCTGTTTAGTTTCTATTAAGGATGCTTAAGTATACCAACATTTTTTTTATCTTTCTTGCCAAATC
TGCAAGAAAGCTTGGTCAAGAGAATCTTGCCACAACTCCCTTTGAGATGCCAAATCTGCGGTGAAGCGGAAAATTATAAAAAACTTTTGGCATTGCTATC
CTGTGTCTAAGAAACAAACATCATCCTAATTTGGTGGAATTCGCCTGATGTGTTGTAGCAAACTAAGGGGAACTGTCTGTCAATACACCTCAAGTGCCTA
TATGTAGACCGGACAATATTTCAACGCCTCTTAATTCAGAGCAGT
>Locus_9469_Transcript_48/63_Confidence_0.140_Length_1434
GGTTAGGGGGGAAAGAAGGTGATTTGGACAGGGAGCTTGCAAAGCTGCAGCTTTGTTGGATTCAAGTAAACTTCACGTTGCAGAGATTTACATGCAAGTT
AGTAGTGGTTCACGAATTATTTTAAGAACACAATATGTCGTCCACATAATTGTTGTATGGAACTGGAGGTGGGAAGAGCCAATGGCCTTAAGATGCAGCG
CTAATTCACCGGCGGGCCTAGTTTCAAAACTAGGTAGTCATGAGGAACCTGTCTCAGGATCCAATCACATAGTTTGACTGCTTATAAAGCTGTTGCGTTT
GTGAAGAGATATGGTTGCATGAACGATCAGAACTTCAGAGGGAGCTACCGAGTTGATGCATAGTACTTACTGTTGAAAATAACAGCTACCTTACAGCATA
TGCATGGATAAGTTACACATCTAACTAAGATGATCAGTTCCAGTGAATTTTAGGGGCTAATAGCTATGTGTAATGAGATAGCTAAGTTCCACTAAGTTTT
CTTTATCACGAATATGGTAGGTTCTAGTATGGTTTCATGTATCAGCGGTTCCATATTAGGCAGACTGTATTTGTTTCAAATATCTCATTATTATGTTCTT
GGGACTGATCTGTGCTTATATTTGGACAGTATGAACTGAAAAAACAAGAATCGAGAATTTCCATGAAATGAGACAAAATGGCACAATAATGATTCAGTTT
GGTCATCAAATGCCTGGTTACGACTCATCAGCTACCCAATCAACCAGCGAGAGTCATCAAGAAGTGTCTGGAATGAGTGAAGGAAGCCTCAACGAGCATA
ACGATCGATCAGGTAATCTCGATGGTTACACAAAGAGTGATGAAAATAAGATGATGTCAGCTTTATCTCTGGGCAATCCAGAAACTACTTATGCGCATCC
AAAACCTGACCGTAGTCAGCCCTTTGCCATTTCGTACCCGTATTGTGATTCATTCTATGGTGGTGCAGTGGCAACGTATGGCTCACATGCTATTATGAAT
CCCCAGATTGTGGGCATGATGTCGTCCTCCCGAGTGCCATTACCAATTGAACCAGCTGCAGAAGAGCCCATTTATGTGAATGCAAAACAATACCATGCGA
TTATCCGAAGGAGACAGCTCCGTGCAAAGCTAGAGGCTGAAAACAAGCTGGTGAAAAGTCGCAAGCCATACCTTCACGAGTCTCGGCATCTGCATGCCAT
GAAGAGAGCTCGGGGAACAGGCGGGCGGTTTCTGAACACGAAGCAGCAGTCAGAGGCTCCAGGTGGTGGCACCTCAGATGCGCAACACATGGCCGGCAAT
GGTGGTCTGTTCAGGCTGCACGAGCACAACTTACCACCCAGTGATCTCCATTATGGCGCGAGAGGTGGCTCTTAAGGAGTCCCCTGGCAACTCATCCTTG
GCTTAGTAACGTGTGGCGCTCAGCGATTCGACCT
>comp73737_c0_seq7
GCTTGTATATCATACCCATACAATGATTCGGGCTCAGGAGGTGTTTGGGCAGCCTTTGGGTCACGCACTAGCGCTGCAGCTGTGGTATATCCTTCTAATA
AACTGTTGTACTAAAATCTCTCAAGACCTTTGAACTTCAGTGCTGATGTCGCATTTCAGCATTTAAAATTTTACATGAGAATGTTAAACATATATGATCC
TAGTTTGGGGAATCTCCTAGTTTTCAGTTCATTCTGATTGGGCTACAGTATGCTGTCTGAACGGTCCATCTTACCATGGTCTAATCAAATGTAGAAACTT
AAGTAGGCTAGTCGCCTAATACCTTAACTATGTTTATTTCAAGCAGACACTGCTTAATATGATCCTTGGTTCTTTGCATAAGCATGCATGGGCCATGACA
AAAGGTCTTTTTTATCACTGCTATAACCGATATACTAATGGCAACAAAATGCTCAAAAACTACCACATCTTTTTGCAGCATATTTAGCCATAACCATAAT
GGGAAACCGTGTGCTAAGCTAGTTGCAACTGCTTCTATTCTCCAGCGACAAATGATAATATCTAGAAAGCTAAGATCGCAGCACTTAATTGTGGCCTATT
TGCACAGGTTTGAACCAGATTTTGCATTTCTTTCTCATGCACATACACTGTTACGTAAATCACTCTATTATTTAGAAGCAACAAAAGGACCGTACATTAA
TTAATTCTCTAGCTGGTTATAACAGAAGCTAAACTAGCTGGATTTTGGCAGAGTTGATCTTAGTATTAGGGTTAAAAGTGAAAACAATACTTGACTGGAT
GCAGCAACAGTGCTTTGTTAATTCTCTGCTCCAGTAATGTTTTCAGAACTGCATCGTTGTTTGTGTGTCCAAATTGTGCTTTCTCAGTTCATGCACGACT
CCATATATATCTATCTCAGTCTGCAGTTACTAGCGTGCTGGCAGCTCATAGACACATAGTGTCCGTTTAGATGATTTCGTTTAGATGATTGCTGCTGTTT
GTAGTTGTATAAATTGACTCATGATATCAACCAAGGTTACACATAATTGTTGCCAGGATTCCATTATATGCTAACGTTAGCTCACATATAAGGCAGGGCC
ATCCATCCACCGTACAGCTTGTTTATCTGTAAGTTCATCACTTTTGAGGGTTTTGTCTAATCAATTGTACTTAAAGCAGTTCCATCCCCAAATTGCTTGT
GGGGGCACATCTGCGAGAGTTCCCTTACCTCTGGAATTAGCAGATGATGAGCCCATATATGTCAATCCCAAACAATATCATGGTATACTTCGCAGAAGAC
AGCTACGTGCTAAGTTAGAGGCTCAGAACAAGCTAGTCAAAAACCGAAAGCCTTACCTTCATGAGTCTCGGCATCTTCATGCAATGAAGAGGGCAAGAGG
TTCTGGCGGACGTTTCCTTAATACTAAACAGCTCCAGGAGCAGCAGCAGCTGAAGTCTCGCAATGCCTCCACCAGGTCCACCACAAATGGCGCAAATTCC
TCAGGTTCAACACATCTACGGCTTGGTGGTGGTGCAGATGGAGATCAAACCATGTCGGGGACGAAAACAATGGCCTCACAAAACAATAGCAAAAAGGCTG
TTTCTTCTTCTGCAGCTCTTGCTTTCACCGTGACTCCTATGGTGCGCAAAGATGACACCTTCTTCCAGCACCTCAGCCACAATGTCAGCTTCTCCAGCCA
TTTTGGCCAGGCAAGCGCCCAAACCGGCGTTGGAGGCATCCATAATGAGACCCAGCATAGGGTTTCCGTGATACAATGACGGTTTGCGAAGCTTATCTGG
TGATCCAGGCTTCTAGGTGTCCTCGTGTCCGGTGTAGTCTTGGTCGCTCAGGCAATTCATCCTTGGCTTAGTTTTTGGTGTGTTAGAACCTACAAATGTT
TCATACTCTGTTGTGGTTTGCAGAGCCCATAAATCAGGGCTCTCCTAAAAAAAAATCAGGGCTTGATGGCGACACTACTATCCAAGTATTGTTGTAATGGT
GTGTGTTAGAAACTCGCAAAAAACCCTTCTTTGTGCTCGTATTAGTACATTATGCTGCACAATTAGCTGCTATTCTGTTCGTACTACAGTCTGGTATGCA
GTGGTTAGTGGTCCAGTTTA
>Locus_5417_Transcript_25/28_Confidence_0.312_Length_5135
ATTACAAGACTATTTGATGCAACAATCACGCATATGGCTGCGCTAGAACTACTGCTCTGAATTAAGAGGCGTTGAAATATTGTCCGGTCTACATATAGGC
ACTTGAGGTGTATTGACAGACAGTTCCCCTTAGTTTGCTACAACACATCAGGCGAATTCCACCAAATTAGGATGATGTTTGTTTCTTAGACACAGGATAG
CAATGCCAAAAGTTTTTTATAATTTTCCGCTTCACCGCAGATTTGGCATCTCAAAGGGAGTTGTGGCAAGATTCTCTTGACCAAGCTTTCTTGCAGATTT
GGCAAGAAAGATAAAAAAAATGTTGGTATACTTAAGCATCCTTAATAGAAACTAAACAGGTCTTAGATTGAAATTATCATAAATAAACCCTTTTTCACTT
TAGCTGTAGTCCGGATGAAATCTTAGGGGCCTTGCTTAGCCTGCCCTGAAGCTTGCCAATAAGTCGAAGCTGATGCTGACACAGCACTGAAATAGATCCC
AATGAACACAAGTTATTTCGAGAAGATAAAATACCAAGCAAAGCCAGGATTGCATTAAAAGATAGTGTATTTCTACTATACGATCTGAAAGCTTAATTTA
TTATATCAATAATCAAATTGGCAGCAGTGTGGAACAGATATAAAGAAGATGCTTCAAATCACAAATAACTACAGATAAATTGAAGGGTGAAGGTTCATTC
AACCACCAAACACCCAAAATGTCGCGCTGCATATACACAGAACCTAAGTACGGTACAGTTCATAGTTTGGGAAAATAGACTGCAAATGTGCACAGATAAT
ACAGATTGTTACTCGATGAACAGTAAACACGGTATTCGAAATCCTTCTAACCATCCATCCAGGCAGTGCCTCGTATACTCAAACCTCTTTTTGGAGGAAG
CCAAACCATGTGATCCTTTTCAACCCAAATACATATATTCAAGATGAAACTAACAAAAAGACAGTTGATAAAGAAACACGGAAGAGAGTAGAAAAAGGGT
ACCTGTTTCAAGTTCCCATCTTCTGAAAATGATCAGCAGTCAATAGATGTTTTAGTCCATTCTCCAGAAAAATGAGTGCCGCATGAATCCTTTCCGGTTT
ATAGTGAAACATTTTAGACGATGACCTTTAACATTCTAGAAAACGAAGAGAAAACCTAAAGTCACCTAATTTCAGCTTAAACATGTTCCAGCAAGATCAT
CACCATCCTGACATGTTCCGTTTTGTCCCTAGACCCGAGCACAAGGTATTGCGAGGCTAATTTAGCTCAATTCAGTTGTCAGAGCAATTGTGTGTGAAAA
GTGTGAACAACAGCATAGGAGACAATACAGCAAGTGTAGCGTGCCAATATGTGTCCAGCTTGATAGTTCACTATAATAAAGTTCTTTAATTCAAGTATTT
GAATAATATAGAATCTCAACCTTCAAGTTTAAAAGGTTTTGTATTTGCTATTATAATCGTTGCCACCCCCTCACCCCTCTCAAAAAGAGAGTTACTTTGT
AGCGCACTGTAAATCTCTTGATTGAAATAGTGGCATGTCATCTTATTTAATTCAGTTATTTCATAAGGTACAATTTGTTTAGCTGACAGCTCAGACATGC
TTCTATGCAACATGTTTATACCAATGCTGTATTTTGGCAATCCTGGATGTGGCATTACTTAACTGTGCTTTCATATTCAATAGTTCTGGCAAAGGGAGGC
TGCAAGCTTGAGACAACAACTGCACAACCTGCAAGAAAGTCATCGGTATGGCTAGAAAACCCTGCAAATTTTACAAATGTACAAGTCAGAAAATTTGCCT
AATGTAAAAGGATCAAACTACAGGATTTGTCAGCATGAGCACAAGTGCCTAACTTCCGAGTTAGATAACTCTGGAGGAAGGAAATCCTTCCTACTGTTTG
TTTCAAGAATATACAAAAATTCAAAACAACATTAGAATAGAACCAAAAGAAGAAACAATATTCACCATGTAGCAATACTCAATTCCTGGTTGTCTTATTT
ATAAAGGTATAAAACTAGGTTCTGACGAGACAATTATGAATCAACTACACCATGTTACAGATCATCCATAACTCTTGGTGATGCAAATTAAGGGCTGTAA
AGTGACAAGTATACCTCCACCAACTAATTTGTGCAGAGTGTATAAAATTGTAAAACAATGGATAAGAATGAATTTAGTATACAAGATAGCATACAATGCC
CAAGTTTAAGGAGACATACTGATTTACTGACTCCATAGGACAATCTTTTGCTTATGAGGACATCAGTTTTCATAAACAATATCAATGTAAAAGGAGGTAA
CAGTTTATGAATTTCCTATGCCAAGCCATTTATAAGTCAAAAAATGTACCTATTTAGCTTTTATGCATTCCAATTAACTTCCAAAGAAGAAGAGTACATA
CTAATGGAAAACAAATATGATATTATCATCTGTTTTGCCCGGTAGTTTCTTGAGTCTCTGATTTGTCCGCACTTCACTCCCAACCAACACAACATTAATA
CACATGGGCAGAGGAATATCAGGTTATCAGGTAAACTGAATACAGTTTGATTGACTGCAGTCAGTTGATGGGACAAGATCTTTCTGGATTGAGCGTCAAG
GAACTCCAAAATCTAGAAAATCAGCTAGAAATGAGCCTACGTTGCATCAGGACAAAAAAGGTAAGGATGCTAGCCATATTTCTGTTTTTCGTTACCAGAT
TGCCTCATCCAAGAATACAACTGATGATGACGTTTTTTACTTTCAGGACCAACTCTTGATTGATGAAATTCACGAACTGAATCGAAAGGTTCTGGAATAT
TCTGTTATTACTCTGCAATGAGAGGTCTCTGAGTGGATTTCAAGAAGCTGACTCTACTGGCTCATTTTCAGGGAAGTCTCATGCAACAAGACAAGATGGA
ACTATACAAAAAGGTCAACCTAATCCGTCAGGAAAACATTGATTTATACAAGAAGGTATTTGATAAATCGTGAATGAAAATTCACAGGATATACACCCCA
TCCATTATTGACTGAGGTTTATTTCTCTACTCAGCTCTACGAGAAAGAGGTGACAAGTGAAGTCAACCGAGATTCATCAACTCTAAACTTTGCAGTTGTC

GAGAACGTCAACATTCCTGTTCATCTTGAACTTAACACTCCACCGCAAGAAAATGATGTTGAGCAAACTGCACCTCCTAAACTGGGGTAACCCTGCTTTT
TTCTGCAAAGGAACTATTGATTCTGTTCTTAATTCTTTAGTGGCATATTGTTTACTACTAACTGAAAACTTGCAGATTACAACTAAATCCATGAAGGCAT
GCAGAATGGCACCGCTTATGTTCATTATTCTCAACGGTACGATTCAAAGCAACCAGAAACACTATTGGTTGTAAAGAGCTTAAACACATAGCAAGACTGA
CGCGGTATGCATGCCATGAGTGATTGATAATGGTAGTGTAAATCTATTTCAAATAAAATGTATTTTATTGAAGAATACAGCAAATGATTTGTATTATAAC
TATTTGACACATTCTGCAAGTTTGTTGACCAACGTGAGTACATGATAGCTACAAATCTTATATTGACAAAAAGATATGGTGTGTGGAAAAAAATTCAGGTG
CTATTTTTTTCTATTTTGAGAATTGTATGCATAGCAGAAGAGCACAAATTGCATTCTGGTTTATCCGTGCAGGCATTCCAGAGTATCAGAATGGGGATCA
ACCTTTCTTCAGCTACATATGTAAATATACGGATGCTAGTTGCTCACTTAAAATTTTGTAGTGTTCAAAGGAATAACAACAGTAAGACGTTGACGCACCG
AAAATAACCAGAAATAACGGATTGTACTATGACAAGACACACGACAGATACACTAGCTTAAGTTTGTACAGGAAAATATTTGGCCTATTCTTATGTCACA
GTAAATTAAGATTATCACCTTAATCACAGAAATTCAAGGGGTAAACGATTATAGCACAAAACGATCCTGGACCCCAAGACCTGGGTCTCTGAACGGCTGA
ACCTCCAATTATACCTCTATACATTTGGAATGTGTGTAGTCAAACTTTCGCTACTGGTATCTTTACAAATATTTACATTGAACACATCAAAATGATATAC
ATGGATTCCTCTCGATATGTCTTTGAGGTTTGGGTTCTAAAAATGATATACAAATTATTAGGTTCTAAACTTAAATGATATATACACATCAAAATGATAT
ACAAATTATTGGGTTCTAAACTTAAATGATGTATACACATCGAAATGATATACAAATTATTAGGTTCTAAAAATATACTATGGCAGAATTAGGTGGTCAT
TTGAGTTTTGGAGGCCTTGTCAATGTCCCAAACATCACTTATTTGTGACTGGGAGAGCAATGAATTGAAGGAATGAGAGTAACCATACGGTCATTGGTCA
ATTAAAAGGAACTTTAATATGCTATGACCACATCGACCTTTCGTAAAGTAAATAACTCAATTATAACCAAGTAATTGGGTGTCTATTGAATCCATACGGT
TCTCAAAACAGTTCTAAACAAAGTGAATTTAAGCATCCTGAAAAGATATTTGGCAAATTTGCCATACCCTGAATTCACAGCATGTTAACACAAATCATGT
GCAAATCATTCAGATCATTGACAAACGCACGCATGCACACAAATCAACACCGTTTTCTCATGTATAGAACAGAAAAATTGAGAAACTTACGATTTTCTTG
CAAGTTATGCAGTTGTTATCTCTAGCTTGCCGCCTCCACTTGCAGTAACTGAGACGCCGGCGCCGAGCTTGAGCTTGAAACCTAGACTACGGGGACAGGG
TAGACAGGAGACGCGAAACCCCAAACCAGCGCAAAACGACGCGCAGGGAGGGAGTGTCACTGAAGGAGCGACAGGGAAAGCAGCGGCACGCCGGGGGAAA
TTCCGATCTGAGAGAACGGATCAGCCATGCATGACAGGAGCTGCGGAGGCAGCAGGGGGGGGGGGCTCCCGCTGCCACGGGACAGTAGGGCACGCCAGAT
CGGTGAAATCCAGGCGAAGGACGAGGCGTGGGCGAGATTTACACGACGGCGCGATAGGATCTAGCCCGGATTTGGATTCGACCGACGAGGGCGGAAGGAA
CGGCAGGGTGGTGCTCTTGTAGAGAGAGAAGGAGT
>Locus_20324_Transcript_24/30_Confidence_0.377_Length_1421
CCTCCATTTGTTGATGGGGTTTTTTTACTGCGGGTACCGGAGCTCGCAGTACAGGGGCGTCACCTTCTACAGGAGGACCGGCCGCTGGGAGTCGCACAT
CTGGGATTGCGGGAAGCAGGTCTACCTGGGTGAGCTCGAATTCTAGTTTCCTAGCTCAGGCTGATGTGTTGATGTTAGTGTCTAGCTTTCGGCGTGCTTA
TCTTGATTGTGTTTCTCTGATGCCTTCTTTCCATTTCCCCTTTCAGGTGGATTTGATACGGCGCACGCCGCCGCAAGAGCCTATGATCGTGCTGCGATCA
AGTTCAGGGGCTTCGATGCTGACATCAACTTCCATTTGAAGGACTATGAGGACGACCTGAAGCAGATGAGGAATTGGACCAAAGAGGAGTTTGTTCACAT
ACTCCGACGCCAAAGCACTGGGTTTACAAGGGGGAGCTCCATGTACCGCGGTGTGACACTGCACAAGTGCGGCCGGTGGGAAGCTCGGATGGGTCAGCTT
CTTGGAAAGAAGTACATCTACCTTGGGTTGTTTGACAGTGAAGTTGAAGCTGCAAGAGCATATGACAGGGCAACCATTCGCTTCAATGGAAGGGATGCTG
TTACTAATTTTGATCCTAGTTCCTATGATGGAGATGTTCCACCTGAAATTGAGAAAGAAGGGGTTGATGGGGACATCCTTGATTTAAATTTGAGGATCTC
GCAACCTAACATGCATGATTCGAAAAGTGATGGCATCCTGACTGGGTTCGGATTAAGTTGTGATTTTCCTGAAGCTTCAAGTTCTGTTGTTTCTCAGCCA
ATAAGCCCTCAGTGGTCTGTACATCCCCAGAGCACAACTACTCCACCCCAGCATCAACATTTGTATGCATTTCCTTGTCCGGGCATCTTTGTGAACCTCA
GGGAAGTGCCTATGGAGAAACGACCCGAGTTGTGTCCCCAGTCGTTCCCTCCGTGTGCATGGCAAAAGCAGGGTTCCCCTGCGCCATCACTCCCTACTGC
AGCATCATCAGGATTCTCTACTGCCACCGGCGGCGCGGACCCGGCCAGCGTGCCGCCGTCCCGCCCCCGGCCCTTCGCCACCGGCCACCAGCTCCACTTC
CCCCCAACCGTCTGACAGCCTCCCCCTGTTCAACTGGCGACGATAACGCGGTCGTCTGGGCGCCCCATGCTTCGCTGCTGGTCGGCAACAGTCGCAATTG
GTTCGGGTAGAGAGAGATGGAGGCTCCAGCACACGACCTCTCTGTCTCTCTCCCCACCTGGTAATTATCATCAGAACCACCATGCTTATGATTCTTTTAT
CTTCATTTTGAGGGCGGATCCACGGATCATGAGCGTGTACCTCCAAAATTCTAAAATAAACCATTTACGATAACTCTGGCTGAGGCTCTGTTTGTTTGGG
CTTCTGGCCAACTTTGAACGG
>comp79373_c0_seq43
GAAACCTGGTAAACCCTGGCCACGGACGCGCATGCCATCCAAGATGACGTAGTCCACACCGAATCTTCCACGTCCGCACACGCGAGCACAACGCCACCGC
AGGCCAAGTGCCGTCTCCACCACAACTCGCTGATCCCTTCCTCCTCGCCGGCTCTCTCTCCATCCCTCGTCCTTCCCCATCTGTCCCTCCCTTGGCTTCT
TCCTCCAAGCTCCAACCAATCCCCACGCGCGCGCGCGCTATAGCCTACAAATAAATACTCGCCACTCTTCCCTTCCTCCCGCCTATCGTCTTGCCAAGCG
CTAGAGCAGCTGCGCCATTGCACGCTTCCGGCCCGTCGTCCTGCACATCTGTCCTCCCCGTACTGCCTTGTACAAACTCCGTATGTAAACTACTGGGTTG
TCGCCAGTGTTCGTGTTTGCTGATGCTCATCTAATTATCGTCGAGGAGATCCAAGGAATTGTCCGCGGAGCGCCGGGGAACAAAGGCTGCGCGCGGTACT
ATATCAATTACAAACTGATGAAGAAAATGTTAAAGCAATATGTTCAACAGACCCAACTTGGTGGGAAAGATCATGAACAAGTTCTTAAAGAGTTCTCAAG
GATTCTTGATGACCAGATTGAAAGGATTGTGCTTTTTCTGCTACAACAACAAGGCCACCTTGCCAGCAGGATTGAGGAATTGGGAGAACAACGCACTGCT
CTTATGGAACATTATGATATATCACAAGTTTCCCAGCTACGTGATGCGTATAGAGAAGTTGGGAGAGATCTTATTAAGCTTCTGCGCTTTGTTGACATGA
ATGCTACTGGTATACGCAAGATACTAAAGAAATTTGATAAGCGTTTTGGCTATAAGTTTACATATTATTATGTCACCACTCGTGCAAATCATCCCTATTC
TCAGCTTCAACAAGTATTTAAGCAAGTGGGAATTGTAGCTGTTGTAGGTGCATTATCGCGCAATCTTGAATATCTGCAACATCATCAAGGAAGCTTTGTA
TCCATCTATGATCATCCATCAGTTACCTTGAAGGACCCTATAATAGACGAAGTAAACCATGCGGTACAGAAATTTACGCATGCCACCAATTTTATGAAAT
TCTTGGGACAACACGCGCTTATTGTTCCAGAGGATGCACGAAGCGGGTCTGAGGATCTTGTTGATGATCAGAGCTACCATTTCATGTCTCTGCTGCTTAA
CCTAGCGAACACTTTTCTTTACATGGTGAACACATATATCATTGTGCCAACTGCAGATGACTATTCAGTAAGCCTTGGGGCTGCTGCGACTGTCTGTGGT
GTAATTATCGGATCAATGGCAGTCACTCAAGTGTTCTCCTCGGTTTATTTCAGTGCATGGTCAAATAAGTCATACTTCAGACCACTTGTATTCAGTAGCA
TTATGCTATTTTTTGGGAACCTGCTATACGCATTGGCATATGACCTGAATTCATTAATAGTTCTCCTGATTGGACGACTACTATGCGGGTTGGGCTCTGC
AAGAGCAGTGAACCGTCGCTATATTAGTGATTGTGTGCCTCTCAAAATCAGGCTACAAGCTTCTGCAGGATTTGTCAGTGCTAGTGCTCTTGGCATGGCA
TGTGGCCCTGCTCTTGCTGGTTTCCTCCAGACAAAATTTAAGATATACTCGCTCACTTTTAATCAGAGCACATTGCCCGGATGGGTCATGTCCATTGCTT
GGCTTCTTTACTTGTTGTGGCTGTGGTTTACATTCAAAGAGCCAGAACACTTCGCTAAAACTGTCATCAATGCACAGCCGTCTGAATCAGGTCACCAAGG
AAGTGCTAATTTGGAGGAAGGTCTAGCTCAACCATTGCTTCTGGGTACAGAACAAAGACAGGGCGAGAATGCGGATGACAATGATGATAATGAAGTAGAC
TCTGAAAACTCTCATGAACCAGCAACATCAATTACTTCAGCATACAGATTGCTGACACCATCTGTGAAGGTCCAGCTATTGATATACTTTATGCTCAAGT
ATGCTATGGAAATTTTACTATCTGAATCAAGCGTTATCACAACATACTATTTTAGCTGGTCTACGAGTGCTGTGGCTATCTTTTTGGCAATTCTTGGATT
AACTGTCCTTCCAGTAAATGCCATTGTTGGAAGCTACATTACAAATTTGTTTGAGGACAGGCAAATTCTGTTGGCATCTGAAGTCATAGTTCTCATTGGT
ATCATCATGAGCTTTCGTTTCACCCCTCACTACTCCATTCCACAATACGTCACTTCAGCTCTCATCACATTTGTGTTTGCTGAGGTACTCGAAGGTATGT
ATTTTGTACATGTGTGCAACCATCTCAAAGCATCATGCACAGTAAGACATGAAAACAATCATACGCTATTGCTATAACCATCATACAATTTCTGATGACG
AGCAATGCAGGAGTGAATCTGTCCTTGCTCTCACGAGTAATGTCGTCTAGGCTTTCGCGAGGGACCTACAACGGTGGACTCCTCTCGACAGAGGCCGGGA
CATTGGCCCGTGTAATTGCAGACGCGACTATTACTGCAGCAGGTTATCTAGGCCCGGACCTCCTCCTCAACGTCACCCTGCTGCCACCTCTAGTGATCTG
CATAGTGTCCATCGTCGCAACGTTCTGCACTTACAACAACCTTTGCTGATAAAGCAGCCCCTCTGTGTCCAGCCAGTTAAAATGACTCCTGCCATATTGG
GCCGTACCGTACTACTTGAAGTAGCATCAGCGTTGTAAATCTGGTAAATCTACAAATACCATGGACGGTGCCGGTGTGGCGGTGTGTAGTGTATTGTTAT
GCATTCAACAACTTTCTTGTGTCTAGTAGAGTTAAATATTCGTCAAATTGAAAATTCAAATGCATTAACCAAGGAACATCACTGTGTTGGAAAGACAAGA
TGATTTATTGCAA
>comp81587_c0_seq8
GGCCATCTTCTGGCTGAAGAGAGCAGCCACGCTAGAAGCAACGCATGCAACGGTTTCTCCGGCACGCTGGGTCCCGAGTGTGCTCTCTCTCTTCTGTCAT
CGCCGCTGCCTCCCTCCCCTGTCGGCATCCCCACCGCAGGCCAAGCGCAGGTTGCCTCTTCCCTCGCGCGGTTTGCTCCTGCGTCGCAGGCTGCAACCGC
TGCGTTTGCATCTGGTGGTGGCCACCATGTGTTCATTCCTGATGCTGTGTTTGAGGATCCTTTCCCTTGGCAGTAGTAGAGTAGGGTGGAAAATTTCTTG
TGATTTTGTGTTGCCGCTGAATGCTGATTTGCTGGTAATGATCATGAGGGCTGGGACTTGGGGTGCTTGTGCACCTGCAGGATCAGAGGTGGATCAAGTG
GAACGGTCAAGTCCTGTCATCTGGATGATAGAGCCATGAAGGTGGTGGCTGCTGTGCAGATTGTTTAGTCGATTAATTCTGCTCTGTTCTGTTCGTAAGT
TTGTGCAATTTCTGTGCATATTTTGTAGCAAAAATTATATGCATGATGTACAGTTTTGTGCATATTTTGTAGCAAAAAATGTATGCGTTCATGCAACACT
GGAGGGCTTGAACCAGGCCTGAAACGCGTAATGCACCTGACGGCCAGTCTGGGGGATGCCTTCAGAGAACTCAAGGCCTGGTTTTCCCTGGCTGATGCTT
TTTTTTTTTGGAAACGTGTGTGTGCGTGTGGTGCCAGTGTGGAGTGTGTGGGTATAGTATG
>Locus_32123_Transcript_15/16_Confidence_0.346_Length_1080

CTCTCTCTCTCTCTCTCTCTCTCTCTCTCTCTCCAGATAGCCGCCGTTTGACCCTGCAGATTGCTTCCTTCTGCACGGCGCGATGGTACGGGGAAAGACC
ATATTGAAGAAGATCGAGAACGAATCAAGCCGGCTTGTGACCTTCTCCAAGAGGAGGGGCGGGTTGTTCAAGAAGGCAAAGGAACTGGCAATCCTATGCG
ACGCTCAGGTCGCTGTTCTCATCTTCTCCAGCACCGGCCAACTCTACGACTTCTCCAGCTGCAGCATGAAATCAATAATCGAAAGATACCGCCAAGTAAA
AGAAGGTCAGCAAAATGTAACGGCAAGTAGGGAGGCCAAGTACTGGCAAGGGGAGGCGGAAAGCTTGAGGCAGCAACTACACAACTTGGAAGAGAATCAT
AGGGGAAATCATGCAAACGTTTTCAGGTTGTCAATTTGGCACCACAGTACAAGCCTGAAACACAAGATAACAAGTGCAAACTGCCACAAGATGCACTTGC
TGGGACAACATCTTTCTGGCTTGTCAATGAGAGACCTGCAGACTTTAGAGAACCAGCTGGAAACGAGCCTAACTAATATTCGGCTAGCAAAGGAACGAAT
TATGATTGATCTGATTCAAGAATTGAACAAGAAGTCAAGCCTCATAGACATCGAAAACAAGGAGCTACACCACAAAATCTGTGCCATTAGTCAAGAGAAT
GCCAATTTGAAGAAAGAGGTACATGGACAGCCGGATGCCATTGAAGAAAATACTGGTTCTGTTACTCAGTATAGTACTAACACTCCTGAAGACCTAAATG
CGACGTGTCTCGAGCTACGCCAACCACATCATGTAGAAATAGAGCAACCAGATGTGCCTACATTGGAACTGCGTCTCCACTGATTGCAAGAATCAGCAGC
AGAATCATCGGAAATGAGGAGGCCAGCCATTCAACGCAAATTCATAAGACTTTGTCTCCACTGATTAAATACAAAGAGGAGTTCCTGTGCAAATTACTAT
TATATGAATGTAACAGATGTAATTTATTTTTTATGTATCCGCAGTACACATATGGCCACCATAAACTTATGTTCAAAAGG
>Locus_5523_Transcript_72/107_Confidence_0.155_Length_1838
GCATCAAAAGACAACGAAGCAAATGTAGGGAGACTGTTACATGCTGCAGTGTACAACTTGGTTCAAATCGTTCCACAGTTTTACATGCACTTACTGCTAA
GAAATCTCTAGCATGATTCATGAATCAACAGCACAGAAGCTACATTACAAAGCTTAAAAATGGGGAAAAGTTCACCTTGGATTGGATCATACTGTAACAA
CACCAGCCCCGTCATCAACCTGCTCCAACGTTGCCATATGGGTCAGCAAGGAGCATGAACGATATTAGGATGGAACTTGGGGCAAGTCAGTTTCCGAACA
GCAGCGCAGTTGCTGCGCATCTATTTCAAGCATTAGATACAGCAGTCACAAGAACGACTAGGAGTTTTGGTCGTTGGCGATCCCTTTGTCTTTTTCTTTT
TCGTTCCCTTTCTCTTCACCGGATCCATCTCCAGTCGTTCCATCCAGGAAGCGAACTATGACAACTGTGATGTTATCAGAACTCCCCCTTTGGTAAGCTT
CTTGGAGAAGTTTCTCTGCTGCTTGCTGGGGCTGGTCCTGTATGGGCTTCACCATGGCAACAGCTTCCTCATTAGTGACAACATCCCAGAGTCCATCACT
AGCAAGGATGAGGAATTCAAGGGAGCTATCCACCACCTCCTCCTTGATTTCTGGGTCAGCGACAACATACTGCTTCAAGAGTTTATCACCAAATGCTCGA
GAAACAGCAAGAACACCACCCACACGCCATGTCCCAGCCCACATCACAAAGCCCCCAGCATCCTCAATTCGTTGTCTCTCATCAGACTGATCAGGTTTGT
GATCTCTTGAAACCGCAATAGCCTCCCCTCCTTTACAAACAACAGCTCTAGAGTCTCCAACATTTGCAACAAGCAAACGATCACCCACAATGATAGCTGT
TGAGGCAGTTGAGCCAGCATCTCGAGTTTGACTACTTTCGGCTTTAAGAAATTCTGAATCTGTGTGGTTGTATGTTTCCGCGATAGCAGACTTGGTATCA
CTGATGAACTTTGGATGTTTTATTAGGTTGCTGAATAGGTGCTGCTTGACATACTCTGCTGCTCGTGCTCCACCATGACCATCATATACGCCGAACAATC
CAACTTTCTCTCCATCAATTTCATCTATCCTTGCCTCATAAAAATCCTCCATTGATGCTCTTTTCCCAGGAGAACTTGCAAATCCATAACTGAACCGGCC
ATTTACACTGCGCTGGCCTCCGCTGTCCGCCAACCCGGTCCCGACGAGCCCCATGCGCGCCCCTTCCGCGTCCGCCAGGCACCACCAACAGGACGCCCGC
CCCGCGAGCAGGAGGAACAGCAGCAAGAAGAGAAGCAGCGAGGACGGGAGGCGCGCGGCAGATGGTGGCCCCGACGACCTCTGGCGCATGAAAAGAGGAA
GGGAGGCAGGAGAGCGGGAGGGAGGAAGAAGGGGGAGGAATGGATGGACGAGCTGCGATTGCAAGGGGAGAGAGAGAGTAGAGAGAGATCTGTGCTACCT
GTGGGGGAGAAGATGGGAAGAGGGAAGATAGTGATCAAGAGGATTGACAACACGACAAGCCGGCAGGTGACGTTCTCCAAGCGCCGGAGCGGGTTGTTCA
AGAAGGCCAAGGAGCTCGCCATCCTTTGCGACGCCGAGATCGGACTCATCGTCTTCTCCAGCACCGGTCGCCTCTATGACTTCTCCAACACCAGCATGAA
GTCTGTGATCGAAAGATACAATGAAGCAAAAGAGGATCATCACCTGACCATGAGCGCAAGTGCGGAGGCTAAGTTTTGGCAAAGGGAGGCAGGAAGCTTG
AGGCAGCAACTGCATAACTTGCAAAGAGCACCATCGGT
>Locus_5717_Transcript_28/37_Confidence_0.305_Length_1065
AACGACAAGGGGCGGCTGAGCAAGGAGGAGGGGAGGTGAGGTGCCAGGTCGAGGGGTGCGGGATAGACCTCGGCGCCGCCAAGGAGTACCACCGGAAGCA
CCGCGTCTGCGAGGCCCACACCAAGTGTCCCCGCGTCATCATCGCCGGCCAGGAGCGCCGCTTCTGCCAGCAGTGCAGCCGGTTCCATGCACTCTCGGAG
TTTGATCAGAAGAAGAGGAGCTGCAGGAGGCGTCTGTCCGATCACAATGCCCGTCGGCGGAAGCCTCAGCCAGATGCATTTGCCTTTGCCTCTGCAAGGC
TACCGTCATCCTTATTTGATGATAGGCGACAAATAAGTTTTGTCTGGAATAAAGCTCCTCTTAGCCATGTAAGACCTTTCACTTCTTCTCCATGGACAG
CTCGACTGACTTCAAGCTCCCACATGCAAAGGAAATAAGAGAGATGTCAACAAAAGTTGGGACAATCACTGGACAAGTTCATTTGCATAAATCTCATCTG
TCCAATGCCATTCCAACTCTTGGTCATGGAAAAGATGAGCTGTTACCAATGAAAGGTCCGGGCACATCTATTACTGCTTCAAAATTCGATGGAGCACCGG
ATCTTCAGCGTGCTCTCTCTCTTCTGTCAGCTGGCTCTTGTGGATTGCCTGATCCTGTACAGCAAGCATCTTGTCTTATCCAGTTCTCTGGTGCCAGCGA
GAACAGCAGTGACCTTCATTCATCACATGGTGGGAACTCTGCTCCAGTCTCATGCGCTGATGAACAGCACATAGCACCTCAGTCTCAGCTAGTCCGTTTT
ACCATGGATGCCAACAGCAGTGGCTACGAGTCCACTTTCTTCGGTGTAAACCAGATAAATTAAGGTGTGAAAGGCACACGCCTCTTGATCATTTGGATAA
AGAGCCATGACGATGGCTTGGACTTGTGCAAGAATAGCTTCTGTGAACTACCAGAGTTCAAAACCTCTCTGTTAATGTTATCTACATATTTATTCGCCAC
CCCTGAACTAAGGGTCTGTTTGTTTGGGCTGGAGCTTTTGGGCTTTTGGCTGAAAAGCTGCTTTT
>comp86229_c0_seq13
CGGGAGCGCGGCGACGAGCTCGGAGGCGACGCCGCGGACGCCCGGGACAAGCTGGCCTCCTTCGCCAAGACGCTCACGCAGTCCGACGCCAACAACGGCG
GCGGGTTCTCGGTGCCGCGCTACTGCGCGGAGACCATCTTCCCCAAGCTGGACTACCGGGCCGACCCGCCGGTGCAGACGGTGCTCGCCAAGGACGTGCA
CGGGGAGGTGTGGAAGTTCCGGCACATTTACCGGGGCACGCCGCGCCGGCATTTGCTCACCACGGGGTGGAGCACGTTCGTGAACCAGAAGAAGCTCGTC
GCGGGGGATTCCATCGTGTTCTTGCGCACCGAGCATGGCGAACTGTGCGTTGGGATACGGCGTGCGAAGCGGGTGTCTTGTGGTGGCATGGAGTGCATGT
CAGGGTGGAACGCCCCGGGGTATGGGGGATTCTCGGCGTTCTTGAAGGAGGAGAGTAAACTGATGAAGGGTCCAGATGGGTACATGAGGGGCAGGGGGAA
GGTGAAGATTGCGGATGTCGTGGATGCGGCAGGCCTAGCGGCGAGCGGGCAGCCTTTTGAGGTGGTGTACTACCCGAGAGCTAGCACGCCGGAGTTTGTT
GTGAAAGCCGCATCAGTGCAGAATGCGATGAGGATCCAGTGGTGCCCTGGGATGAGGTTCAAGATGGCATTTGAAACGGAGGATTCATCAAGGATTAGCT
GGTTTATGGGGACAATATCTTCTGTTCAGGTTGCTGATCCAATCCGATGGCCGAATTCACCTTGGCGGCTTCTTCAGGTTTTTACTTGATCGAAAGTTTT
TCATAGTTGAATACTTATATTCCATACTGCATTATAGACTTTTGTCTTGAGTGGGTTACTACTTGCTAGTATGATGTGTCTGGTATGTACGGGTGTGTCA
CTATTCTTCAGAAGTTCTTTTCCCTTCATATGTTGGCTCAGCAACTTGCTCAGATTACATCTAGCAAATGAATTGGTACTTGGAAGAGTAGAAAAAAGAA
TATAGAAAAGAAAAACTATTGGGGATTTGTGATGATTGGTAGCAAAATAGTCATGAAGGAATGGTTTTAGTTGTATGGACCGTTATTGGTTCTTTAAACT
TAGTGTTTATCTTTTCACTAAGTTGCTGTCCTCTGCCAAATAATTCGCTTTAAAACTTTTTACTGTTCCTTTTGCAATTACAAATAGCATCCAAAATTCC
TTCCTTGCAGCCTCAGACAGTCCGACTAGCTCGGACATCACATGTTTGTAGCCTTTAGCATAGCCCACATGATGCATCCAAAAGGCATTGGGTTACTTGA
CAAACTTGCAGTTAGAAATGGGATGGCTGAGGTATGCAGACATTCTAAGGTTGGCCGAAAAATTAAAGGTCTTAATTGTGTGGACTAGGATGAGAACAAA
TTGGACATAATTCTAAGAAAAAGGAAAAAGAGTACTACTTTACCTATAGGAAACCATGCTATCTGTTGGGTTGGTTTCTTGTTTGGATCTCGTTCTCTTT
GAATTGTTAGTATGTTTCATCAGTCTCTTGTGGTTACTGAGAACTGGGATGTATCATCATAAGCTTCCGAAAGCCCATTGGTCACCATTATCACCGTTGA
GTAGCCATTGACAGATTCAGGTTTGAGCATCCTTTGGCTGCTTAGCTTTCTGTCTCTACACACTTGAAAAGCTGGTAAGCGTCATTAATGTGACATCTTC
CCTAACTAATACTCACAAGAGGGAGGGAGGGAGATGAACTTGTATTGGTACAGAACTTTCAAAGCTTGCTATTGACTTCGCGAAAAGAGACAGCTGCTGA
ACAAGCAAAAAGGATGACCACCGATTTGGCACAACTACTCGAGCACTCCCATAGCACCAATAAAATTTTCCTACCATCAGACTACCTTTGCTTCTCACTA
TCCTGGTTTAATAATTTCATCACATAAGAGCCCAATGAATCAATTATTGATTTTATTTTGTTAGCCTCAGGAGGCATTATAAACACATTGAATTTAGGGC
TCAATACCAGTTTTGCTTTTTTAAATACATTAATTGATCTCTCTCTTAAAGCCCAAATTTCTCTATGTTTTGTAGCCAAATTAAAGCCTACTGATAGACT
GATATTACATAGTTATAGCTCAAATGTGAGGAGTGTACTCTTAAAACGTTCGTGGACAACAACCAACATAATTTTTAAACTGTGGTTCTGTTGGTCCTGA
ACTCCTGAAATAGTGGCAGTACCTTACATTTGTCTTTATGATCAGCCCCCTCATAAGATCGCTAGCGGATCCTTAAAAGCAGCCTGTAGCTAGCATTGTA
CTACATTTTGTACCGTGGGAAAGAGGTGTTGAACTAGTAGTAATAAGACTGATCTAGCATTAATTTAACCTAAACTTGCTAGCTCTATTTTCAGATGTAC
CTTAGGACCTTTTGGCTCTGTATCTCTCTCTTCGCCAATAGTATGTATTGATTGGTATTTTTGGAAGTGCTCGCTATATACTTTATCTGGCAATGTAGTA
CTACAAAGGGTGGAGTCTCACTAGTTACTACTAGTGTTAGGTGTGATATTGGAAACTCATCAGCTTATAGTAGCTATGATGTCATCAACTATAGCCGATC
TAGCAAGTCATCAATTACTTGTGAAATTGGTATAATAAATTGTCTGTAATTGCCTGCGTACTTGCTTTCATTGTTTGTACTGTATCTCTGCTCTTTCACA
TAGCTTCAAATTTCGTACAGGTGACATGGGATGAACCAGATTTGTTGCGGAATGTGAAATGTATCAGCCCATGGCTTGTGGAACTCGTATCAATCATTCC
CCCAATTCACCTGGGACCATTTTCTCCACCTAGAAAGAAGTTGCGGGTGCCCCAACATCCTGACTTTCCATTTGATGGTCAGCTGTTGAATCCAATTTTC
CACGGCAACCCACTTGATCCTAGCAACAGCCCCCTATGCTGTTTCCCGGACAACGCTCCTGCAGGCATACAGGGAGCCAGGCATGCGCAATTCGGTCCAT
CCTTCTCGGATCTCCACATCGGCAACCTGCAGCAAAGCCTGCTGTTCTCCGGCATCCTCTTCCCTGACCACCACGCCCCTCACCCTCCGAGGATCAGCAC
CGACCTGACAATTGGCAGCCCACACGCTGGCCAAGACGCGGCGTGCTCCCTATCGTCTGGCGCCAAGAAGCCTGACGACGTCAAGGCGACGAGACTAGTG
CTCTTTGGACGGGCGATACTGACGGAGGAGCAGATCAAGGCCAACGCTTCCGTTGGCCCGTCCTCGCTGGGAGCCACCGGGAACGGCTCGTCCAAATCCG
ACTGCGACGCCGAGAAAGCACCTACCAACACGTCCGAAGGCTCCAGCTCAGGTGTCATCCAGGGCAGTCCTACCAAAAACAGCTCGTCCTCCTGGAGACT

GCAGTGGTTTGGCGACAATGGCAGTCAGTCACCATCTGAGTTCGGGCTGGAACCTGGCCAGTGCAAGGTGTTCGTGGAGTCGGACGCCGTCGGCCGGAAC
CTTGATCTCTCGGCGCTGGGCTCGTTCGAGGAGCTGTACACCCGCCTGTCTGACATGTTTTGCGTTGACAACGCTGAGCTGAGGAGCCATATGCTATACC
GCACCGCCGCTGGCGAGGTCAAGCACGTCGGCGATGAGCCTTTCAGTGCGTTTGTGAAATCAGTGCGGAGGATCATGATACTGACAGATGCCGGCAGTGA
CAACATAGGGAGTTAGGAATGAAGCGATCGGCCGAATAACTTAGGGCACTACTCCTAGGCAGCAATAAGCCGAACTACATGCATGTGTACAGCCGAACGA
GCTGCGGTTTCTTCTTCGATCAGGCCTAGAGAATTCAGTCAGGAAATTAAGCTGACGCAGTGTTCAGTGCTTCAGTTAGGTTGTTTTCCCACCCTTTTTC
ATTTAGTTCAGTAAAGCTCTTTTAGTCATGCAAACAAATTGTAATCTTGATCATCAAACGCATTTCTTAAATCTTGTTTCCGTTTTGGATTTCAAACCTT
GAGCCGGGCCACAGAGACAAGAGCAGCAGGTGAAAAGGGTTAAATGCCATTGCTGGCTGTTGGAACATTTACTAGTCACCCACTGAAACTTCTGAATCTG
GGGTTACTTGATCAGACTCCCAGCTCCAGGCATTTCATTTGTTCCCTTGTTTGTCTGGGCAGGCAGATACTGTGCGCTGTGTTGCGTATCCCTGTTACCT
CTGATGCATTGATGCCTCCAGGGCCCTGTCCTTTGCCTGCATAGGGGAAATAATTGTGCTGCTTGCCCATTTCGCCTCAGAATATTGTTGCATGAAATGG
ACATCTCAGCTAACACAACCAGAGGAAACAGTGTCCGCAGTAGTGGCAGTGCT
>Locus_12535_Transcript_57/73_Confidence_0.321_Length_2433
TTCCAATAAGACACAGATTCTTCAGAAAGAGGTTTTCCTCTCCCTGCCCTTTTGATCTCCAACTATGGCAGTGGTGACAAGGAGCAAATTTGCAATCTTA
GGGTGGTTCTCGTCGTGAAACCTGAAAGTTTGGGGAAATAAATTTGGGGGTGTGGATGTCATGATATGTAGGGTCCGGCACTGAAGTGGTTGATGCTGTG
GTTTCCAGGTTAAGGGGAGAAGAGGAATAGGATATAGATCTCAGGAAGCCAGAGATGAGGTCGAGGGGGAGCCAGTGGCCAAGCTTGTGCTCCAGTGAGC
CTGATTCTCTTGTTCCCAGCTGATCGATGTGCCACTGCCTTGGTTCGCTGGATCAGCGGATCTCCATGGTTCTGTGCGGTGCTGCAGATTGAGAAGAAGG
TTTTCCAGGGGGAGGAATCTCTTGTGGGTTCCTTTTTCTGGGAGCAACGTAGTGGGAGGTACTCGGTCTTTTGCACCTTCATGTACTTGTGTGTTTTTGC
CTTTCTTGATGCTAGAATGATGGTGGCGCTGTTCTTGCCATGATTGATGATTCCTCTAGTAAAAGTTGAACTACTCTGCACCTCTCTCTAGCACATGGAT
AGCACCTTATGTACTTCTCTGAAAATTCAATGATGTATAGTTGAGTGCTCAGATATGTTGTTGCAAATCTGTCCAATTTCTGCTACCCATATGTCAAATC
TTTTCTGTTTTTAGTCTGTTTCACTCTTCCATTTTGTTGCTTCAAAGGTAGCTGCTTTTGCATACAATTAGGAGCAGCAGTTGAGATATTCAATTCGGGC
ATGGGTTCTTTTGGGATGGACTGGAACCAGAAAGGCTCGGTGTTGTGGGATTGGGAGAATTTGCCGCCGATAGGCAGAAATGCAAACGAGAACCTCAAGA
TTGCGCCACAGGCTGAACCGAAGTTTGCAGGTGTTGAGGCAACAAGGCATGAATCGGGGCATTCTTCTTGTGGTACTTTCTCTTCCAGCTCGGAGATGGG
GTATGGTTCATCCAAGAGTTCCATATCAGCGTCGATTGATTCTTCACCCAAGGTGGGAAACAACGTGGAGCTCAATCTTGCAGCTGTCAAAGTGCCTGAC
AAAAACACCGTCAAGAACACTGATTTGGGTAAAGTTGATGACGCTGGAACTTCTCCATCATCGATGATAGCCGTCAGCAGTGGAGAACCGGTGATTGGCC
TGAAACTTGGCCAAAGAACCTATTTTGAAGATGCATGCGGAGGGCAAAGTGTCAAGAGTTCACCATCCAATGTGAGTGCAGTGACCCCTGCTTCTGCGAA
GAAAGCAAAGATGATTCAAAACGCACAGAACTCGTACTGTCAGGTTGAAGGTTGCAAGTTTGATCTCTCTTCTGCTAAAGATTATCATCGGAAGCACCGA
GTCTGTGAAACTCATTCTAAGGCTCCCAAGGTTATTGTTGCTGGTCTGGAGCGACGCTTTTGTCAGCAGTGTAGCCGGTTCCATGCTTTAACTGAGTTCG
ACCAGAAAAAACGAAGCTGCCGGAGACGCCTCAATGATCACAATGCCCGCAGACGGAAGCCACAGCCTGAAGCAATTCCTTTCGGTTCATCAAGGCTCTC
TGCAATGTTTTATGATGCAAGGCAACAGACAAGTCTTCTGTTTGGTCAAGCTCCTTATGGTCAAATGAGAAGCTGTGCAAACTCTTCATGGGATAGCCCA
GTAGGAGGCTTCAAATTTGGAGAAACAAAAGCTCCTTGGTTAAAGCCAACGAGAGCTGCAGGTGTTGATGGGATGCATGTATCAAGCCAGCAGGTGTGGA
ACAATATTACGCCACACGGTGCACATCATGATTTTAATGGTATCATGGCTTTCAAGGGAACCAGTGCAAATGTCCTTAATCAAGGTGCCGAAGCTTCTCC
GGTCGTCTCCAACTCGAATGGAGCCCCAGATCTTCAGCGTGCTCTCTCTCTTCTGTCAAACAATTCAGCTGGTGCTGCCAACGACCAGCCAACTCCTCAG
CTGCACCCTGGGCTGACCACCTTCGTCGGCTCCTCCAACCCTGTCACTGTGATGGAAGCCTCACCACCAGGACTCTGGCAAGACAGCACGCCCCTTGATC
ATCAGGCGCAGTTCCAGGCTTTCGATCCCATTGCCGCACCGCAACAGCTCCAGCTCCCAAAACCCCCTTCCTTCAACAGCTCCACTTCCAGCTATGGCCA
GATGCAGTGATGGATGCTTCCTTCAGTTCTGCTGTCTGAAACTCTGAAATTTGAAACTGCTGTGTTCTAAAAAATAAAGAAAAGCCTGTGTGCGAAAACT
CAGGCTTGAAAAATTGATCCAGTAGTGTTCCCGTTCAATGGCTTGAATAATCCATCCTTGTGATGGCGTGTAACTCAAATGCTTGGATTGCGCTGGCAAG
TGATCATGGACTCTTGTGTTCTTGATGGCATCT
>Locus_7685_Transcript_12/54_Confidence_0.366_Length_2212
AGCGGCGAGAGCTGGGATGCTGTGCTCGGCAACGCCGCCGCGGCGGGACAGGACCAGTCTTTCTTGAACTGGATCATTGGGGCCGCCGGCGACCTGGATC
AGCAAGGGCCGCCTCTTCCGGTGCACCAGCAGCCGATTCTTGACAATGCGGGGTTCGGTTTCCCGGCCACGGATCCCCTGGGCTTCTCGCTGGACCCCCA
CCTCGGCGGCGTCGCTTCCGACCTGTCGTCCCCTGGTGCGGTGTCGCACACTACCAACAGCGGCGGTGGCACCAAAGGGTCCTTAGCGTTCGGCCTCTTC
TCGTCAGAGTCTGCCTCCCTCCAGCCGCCGCCGCCTCCGATGTTGTACCACGAAGGTATCGACACAAAGCCTTCTCTTCTTGCTGCGCAGCCGCCCGGCC
TCCATCACCAGTACCAGCACCAGCCGCCTCCTCCCGCCTCAACTTTCTTCATGCCCCTCCCGTCCTTCCCTGATCACAACCAGCAGTCCCCACAGCTCCA
GCCACGGCCCAAACACCATCAATCCATGGCAGACGACCTCTACCTCGCCCGAAACCGGCTCCTTCCGGCAGCGGCGGCGCAAGGACTCGCCTTTCCACCA
TTACATGGCCCTGCTTCATTCCAGCTCCAGCCTTCGCCGCCACCACATCGTGGGGCGATGAAGACGACGGCTGTGGAGGCGGCGCAGCAGCAATTGCTGG
ACGAGCTGGCGGCAGCGGCAAAGGCCGCCGAAGCCGGCAATTCCACTGGCGCGCGAGAGATATTGGCGCGGCTCAATCAGCAGCTTCCCCCAATTGGGAA
GCCTCTCCTCCGCTCCGCCTCCTACCTGAAGGAGGCCCTCCTCCTCGCGCTCACCGACGGCCACCGTGGCTCCACCCGCCTCACCTCCCCACTCGACGTT
GCCCTTAAGCTCGCCGCGTACAAGTCATTCTCCGACCTCTCGCCCGTGCTACAGTTTGCCAATTTCACGGCCACCCAGGCGCTTCTTGACGAGATCGCCT
GCACAACTGCTTCTTGTATCCGCATCATTGATTTCGACCTCGGCGTCGGCGGCCAGTGGGCTTCCTTCTTGCAGGAGCTTGCACACCGCCGTGGAACTGG
GGGTGTTTCCCTGTCAATGTTCAAGCTCACAGCCTTCGTCTCCAGTACCTCCCACCACCCGCTTGAGCTGCATCTTACTCGGGACAATCTCTCGCAGTTT
GCTGCTGACCTTGGCATTTCATTCGAGTTCAGTGCCATCAATCTCGATGCATTTGACCCTGCGGAACTCATTGCTCCCACAGCTGATGAGGTTGTTGCGG
TTAGCCTCCCGGTCGGTTGCTCTGCACGCACTCCACCACTCCCGATGCTGCTTCAGTTGGTGAAACAGCTTGCTCCTAAGATTGTGGTGGCTATTGATCA
TGGCAGTGACCGGGGTGACCTCCCATTTTCGCAGCACTTCATGAATTGCTTCCAGTCCTGTATGTTCCTTCTCGACTCACTTGATGCCGCTGGCACTGAT
GCGGATGCTGCCAGTAAGATTGAGAGGTTTCTGGTTCAACCAAGAGTGGAGGATGCAGTACTTGGGCGGCGTAGAGCTGAGAAGGCAATGGCTTGGCGGA
CAGCGTTCACATCAGCTGGGTTTGCCCCAGTGCCCCTCAGCAATCTTGCGGAAGCACAAGCTGACTGCCTCTTGAAGCGGGTGCAGGTCCGGGGCTTCCA
TGTGGAGAAGTGTGGAGTGGGTCTAGCACTCTACTGGCAGCGCGGCGAGCTTGTGTCCGTCTCGGCATGGAGATGCTGATCGTACCTACCTCCATCATCA
TTCCTACACCATTTGCAGGCATGTTTAACTCCAGTAATATGCATTTTGTCTTGCAATTAAAACATTGCAAAGAAACTATTGTCTTTGTCGTTGCACACCT
CTGATTGCTTTACTACTGTTGTTTATTAGCTAGGCCTAATGAATTTAGCAATCTTGGTGTGGGTGTCTCCATAATGTAACATGTTGGTCTAGGGTCTCAC
CATGTTCGTGCTTTCCTCTCAGTTAGCCAAAGGATAATTTGGCATATTTGATGACCTCATGGTGTACTATTAGTGTTTCTGGGATGCTTGTAGTTCTTTT
GGCAATTCTTCACCGTTTTCCTTTTTTTGTGGATGTGCGATAAACCAGATCGCTACATAGTGGCTATAATGGTCGTCATTTTACAAGGTAAATTTGTGCA
AGACTGGCATAT
>Locus_2090_Transcript_230/268_Confidence_0.037_Length_1641
AATTTTCCTAGAACACGCAGATAACACTCGGGATCCGGACACGGGTGCGGAATCGGACACGGACGCGGGGTGTCCGACTCGGCAAATTTTTTTGGACACG
GGCTACTGGTAAAATTCTAGTTGCAGCTGCTTGTATTCTCCAGCGACGAACACTATCTAGAAGGCTAGGATCGCAGAACTTAACTGTGGCCTATTTGCAC
TAGTTTGAGCCAGATTTTGCATTCCATTTTCATGCACATACATTGTTCCATAAATTACTACTATTTACAACTGAGAAAAGGAGCATATAATTAATTCTCT
AGCTCGTTAGAGCAGAAGCTAAACTAGCTGGATTTTGGCAGAATTGATTTTTGTATCAGGGTTGATGAAAAGCGAAAACAATACTTGAATGGATGCCGCA
CCAGTGTTTTGTTAATTCCCTGGTCCAGTACTGTTTTCAGAACAATGGCTGTACCATTTCTTTCTATGTCCAAATTGTGCTTTCTCAGGTCATGCACGAC
TCCATACCCGATTCTACAGTTACTAGTGTGCTGGCAGCTCACAGACACATAATGTCCATTTAGATGATTGCTGCTGTTTGTAGTTGTGTAATTAATTCAT
GCTATTAACACATCACAATTGTTGCTAGGATTTCATTATATGCTGATGTTTATCCACATATTGAGGCATCCATCCATCATACAGCTGTTTATCTGTATGT
TCATCACTTTTATGAGTTATTTGTCTGATCAGTTGTACTTAAATCAGTTCCATCCCCAAATTGCTTGTGCGGGCACATCGGCTAGAGTTCCCTTGCCTCT
GGAATTAGCAGAGGATGAGCCAATATATGTCAACCCCAAACAATATCACGGCATACTTCGCAGAAGACAGCTACGTGCTAAGTTAGAGGCTCAGAACAAG
CTAGTCAAAAACCGAAAGCCTTACCTTCATGAGTCTCGGCATCTTCATGCAATGAAGAGGGCAAGAGGTTCTGGCGGACGTTTCCTTAATACTAAACAGC
TCCAGGAGCAGCAGCAGCTGAAGTCTCGCAATGCCTCCACCAGGTCCACCACAAATGGCGCAAATTCCTCAGGTTCAACACATCTACGGCTTGGTGGTGG
TGCAGATGGAGATCAAACCATGTCGGGGACGAAAACAATGGCCTCACAAAACAATAGCAAAAAGGCTGTTTCTTCTTCTGCAGCTCTTGCTTTCACCGTG
ACTCCTATGGTGCGCAAAGATGACACCTTCTTCCAGCACCTCAGCCACAATGTCAGCTTCTCCAGCCATTTTGGCCAGGCAAGCGCCCAAACCGGCGTTG
GAGGCATCCATAATGAGACCCAGCATAGGGTTTCCGTGATACAATGACGGTTTGCGAAGCTTATCTGGTGATCCAGGCTTCTAGGTGTCCTCGTGTCCGG
TGTAGTCTTGGTCGCTCAGGCAATTCATCCTTGGCTTAGTTTTTGGTGTGTTAGAACCTACAAATGTTTCATACTCTGTTGTGGTTTGCAGAGCCCATAA
ATCAGGGCTTGATGGTGACAGTGACACTACTATCCAAGTATTGTTGTAATGGTGTGTGTTAGAGTAAACCCTTCTTTGTGTTCATGTTCGTACATTATGC
TGCACAATTTGCTCTTTTTTCTGTCACTACTACAGTCTGGT

>comp65473_c0_seq11
GCCAAGAAGAGCCGCCGCGGCCCGCGCTCCCGCAGCTCGCAGTACCGCGGCGTCACCTTCTACCGCCGCACCGGCCGCTGGGAGTCCCATATTTGGGATT
GCGGCAAGCAAGTGTACTTAGGTGGATTTGACACTGCTCATACTGCTGCAAGGGCGTACGATCGAGCTGCCATCAAGTTCCGTGGCTTCGACGCCGACAT
AAACTTCAATCTCAGTGACTATGAGGATGACATGAAGCAGATGAAGAGCCTGTCCAAGGAGGAGTTCGTGCACGTCCTGCGACGGCAGAGCACCGGGTTC
TCGCGTGGCAGCTCCAAGTACAGAGGCGTCACCCTGCACAAGTGCGGCCGGTGGGAGGCTCGCATGGGACAGTTCCTCGGCAAGAAGGCTTATGATAAGG
CCGCGATCAAATGCAATGGTAGAGAAGCCATGACGAACTTCGAGCCTAGCACGTATGATGGGGAGCTATTGACTGAAGTTGGTACTGAAGGTGCTGAAGT
CGATCTGAACTTGAGCATATCTCAACCAGCTTCTCAAAGCCCGAAAAGGGACAAGAATTCCCTTGGTCTGCAACTCCACTATGGATCGTTTGAAGGCTCG
GAATTGAAAAGAACAAAGATTGATGCTTCTTCTGAATTGGCGGGCCGCCCTCATCGGTTCCCTCTTCTGACCGAGCATCCACCAATCTGGACTGCCCAGT
CTCACCCCTTCTATTCAAATAATGAGGTTAAGTGATACTAAATTACTAAAATACATGCAATAACTTTCTCAAATATATTGCTTTAGCTCCCTAAACCTCA
CAATTGCAAGCATGTTCTGAGCATCACATGGTTCGGATCGAGCTGTCCGGCACCAATTTTGAACGTAATCTTGACCATCTGAGGAACTGATTGTGCAGCC
TGAAGGCGTGCGTTCAAATGTTAGATTTGTGTTACTTTCGTTCATTTTCACTTCTTGTGCCGCTGTCAATTTTGGCTCTAGTCTTACATCACCGCTTCAT
TTCCTTGGATTCTATAGCCCGCCTGTTTCCTGTCGATTTCGGAGATATTAATGCGTAGCACAGGTTTCATATGTAGGATCTTGAGTTTTCTGAGATGGCC
CATTCTGGAAACAGTAATAGTCGCCTTAGGAATAGAGATGTGTTTGATTTTTTTCCTTCAAAAGGAACATCCTAGAGAGGTGACTATTCGACATTAGGTA
TGCTAATCCATCGGTCAACTATACCTGCCTGCGGCTAAGCACCTTTCAATGTTTCTTTTTTCATTCATCGTATCATATAAAAGTAGTAAGTATGACTATT
ATTCAAACCTTTCTTGAAAAAAATAGTTAGGATGTTGCCATCTTGTAATGCAATCATAGAATTGGAAGTGCTGAATTTGCCATTTGTTTCAAACAAGCAT
TGTATATTGGTTTATCATTCAACTTGAGAGTTTGGTCAAATTGAACTCTCGAACTTTTCAACTCCTAAACTTTAAGGTCAGGCTCACATATGTCATTTTC
AAATTTTATTGGACGGAAAGTTGAGCTGGACGTTTCGAGCTCGGCTTTCTATCCAACTGAACTAATGGAAACACTGATTTCATCGCAACAAATTTTCAGC
AATAGATATTCAATCTCTTCTTGAATTCATCCTTCATCAGTATGGTGAATAAATTGTTCTTGGATTATGCAGAATGCATCAAGAGATCTTAACAGGAGGC
CAGATCAGGGGGGTGTTCCCAGCTGGGCGTGGAAGGTGACCGCCCCTCCTCCCACGCAGCCATTGCCGCTGTTCTCGTCGGCGTCATCATCCGCTGCAGC
ATCATCAGGATTCTCCAATACCGCCACGACAGCTGCCCACACCGCCCCATCGGCCTCCCTCCGGTTCGACCCGCCGCCGCCGCCGTCCAGCCGCCACCGC
TGAACGTTAAGAAGCCACGCTGTAAATTTGCCAGGAAGCCGACATTTTTTCCTCTCTCGGCGTTGCAACTTTTTAGGTTTTGCGCCGGGGTGGTTTCTTG
TAGTGGAGTGGATTCATAATTAACTGTATTTGCATGCTGCCCATGTGAAATGCTTCTCTCTCTTTTTACGCTCTCTGCACCGGATGTTCTGGGAGTTCTT
GTTGCCGGGAATTAACTAAATTCACCGTCTGAGATTGATCTATACATTGTTGTAGAGAATCGAATCATTGTTGAAATTAGTTACAGAATTATAGATATCA
TAAGGCACCCTCTACTTGTGGACAAAGTTCAGGAAGATTATTCCGGTTCTTTTCTTGGCTGTCAATTCGGCTCTTGTCCTTGGTGTGGAGCCACTTGCCT
ACTGTCTTGTTCTGTTGCTGTTTGTTTTTGGCTTCTTGCACAGTTACAAATTTGTTGGCTCGAATGGATGGTGAGACATT
>Locus_14326_Transcript_33/49_Confidence_0.256_Length_1527
TGACGTGCGAGCGCCGAGCATAGGAGGGAAGGCGAACCATTTCTTGCTTTGTTTATTGCACCGGAACAATAAGAAACTGGAAGAAGACAGAAAAGAGGTT
ATACAGTAGGCTCATATTTGGCATCAGCAGAGGCCTCGCGGTGCCCCGTATTCTCATTTCCTCGCCGTATTTTCTCTCTCCAGCTCCAGAGTCCGGAGGA
AGCAGAGAGAGAAACTCAACGGGAAGGTAGAGAGAGCTGATTAAATGCTACTCGGAGCTAAGGATACCTTCTCGTAGTGCTCCCGCTTTTGTGAGAAATG
GAGGATCATCCAGTCCATCCCATGTCGAAGTCCAACAATGGCTTCTTGTCAGGAAATGGCTATCAGATGAAGCAATTTGGTCATAAAATCGACAATAGGG
ACTCGTCATCAGAGTCCGGTCGGTCTCACCAAGAAGCATCTGCAGTGAGTGAGAGCAGTATAAATGAACAGCACACCTCAACACAATCTGACAATGATGA
TGGTCATGGGAAGCATAATGAGGACATGATGAAGTCAGCAGTGTCCTTGGGGGAACCAGGATCAGCCTTTTTGGCCCCAAAACTAGATTACAGCCCATCT
TTTGCTTGTGTTCCTTATACTGCTGATGCTTTTTATGGTGGGGTCTTGACAGGATATGCTCCACATGCCATTGTTCATCCCCAGCAAAATGATTCAGCAA
ACTCTCGGGTTCCGTTGCCTGTTGAACCTGTAGCAGAAGAGCCAATATATGTTAACGCAAAGCAATACCGTGCAATCCTTAGGAGGAGGCAGATACGTGC
TAAACAGGAGGCTGAGAATAAGCTGGTGAAAGGTCGGAAGCCATACCTCCATGAATCTCGACACCGCCACGCAATGAAGCGAGCCCGTGGATCAGGGGGG
CGGTTCCTCAACTCAAAGCAGCTCCAGGAGCAAAACCAGCAGCATCAGGCATCAGGTGGTTCAAGCTGCACAAATGCCATCGGCAATAACACAAGCTCCC
AAAGCGGCCCCACTCACACGCCTTCGACACCTGCTTCCTCTGACACCGCAAGCGCTTCGAGGGCCAACCAGGACCACATCTGCTTCCCCTCCGCTGGCTT
CCGCCCCGCAATGAACATCAGCGATCATGGTGGAGGAGACACAAAGCTGTCTGCGAACGGCATGCAGCAACGTGTTTCCTTCATCAGGTGAAGAAAAGTG
GCACTGCCTGTGGCAACTCATCCTTGGCTTATGAAACTATGGATATGTAATGGGCATGTAGGTTCGGTCTCAGAATAACCAAACATATGTGAGACCTTTT
TAAGTTTCCTTTAGTTCAAAGAATGATAGGTTATGACCTTATGGCTTTGTTTCTGGAGACTGAAATCCGTGCTTGATATACTATGTTGTACTGAGTTCTG
AGGTGAAAGACATCGGGAGAGTATCAGGTGCAAATGGCACAAATGTTGAAAACGGTGCAAATTCATCAAAATCTGTACCTAAAAGACTTCAATAAATTTT
AAACTTTGCATAGCTCTAGGTGCATTT
>Locus_3441_Transcript_135/417_Confidence_0.055_Length_2546
CGCATATGGCTGCGCTAGAACTACTGCTCTGAATTAAGAGGCGTTGAAATATTGTCCGGTCTACATATAGGCACTTGAGGTGTATTGACAGACAGTTCCC
CTTAGTTTGCTACAACACATCAGGCGAATTCCACCAAATTAGGATGATGTTTGTTTCTTAGACACAGGATAGCAATGCCAAAAGTTTTTTATAATTTTCC
GCTTCACCGCAGATTTGGCATCTCAAAGGGAGTTGTGGCAAGATTCTCTTGACCAAGCTTTCTTGCAGATTTGGCAAGAAAGATAAAAAAAATGTTGGTA
TACTTAAGCATCCTTAATAGAAACTAAACAGGTCTTAGATTGAAATTATCATGAATAAACCCTTTTTCACTTTAGCTGTAGTCCGGATGAAATCTTAGGG
GCCTTGCTTAGCCTGCCCTGAAGCTTGCCAGTAAGTCGAAGCTGATGCTGACACAGCACTGACATAGATCCCAATGAACACAAGTTATTTCGAGAAGATA
AAATACCAAGCAAAGCCAGGATTGCATTAAAAGATAGTGTATTTCTACTATACGATCTGAAAGCTTAATTTATTATATCAATAATCAAATTGGCAGCAGT
GTGGAACAGATATAAAGAAGATGCTTCAAATCACAAATAACTACAGATAAATTGAAGGGTGAAGGTTCATTCAACCACCAAACACCCAAAATGTCGAGCT
GCATATGCACAGAACCTAAGTACGGTACAGTTCATAGTTTGGGAAAATAGACTGCAAATGTGCACAGATAATACAGATTGTTACTCGATGAACAGTAAGC
ACGGTATTTGAAATCCTTCTAACCATCCATCCAGGCAGTGCCTCGTATACTCAAACCTCTTTTTGGAGGAAGCCAAACCATGTGATCCTGTTTCAAGTTC
TCATCTTCTGAAAATGATCAGCAGTCAATAGATGTTTTAGTCCATTCTCCAGAAAAATGAGTGCCGCATGAATCCTTTCCGGTTTATAGTGAAACATTTT
AGACGATGACCTTTAACATTCTAGAAAACGAAGAGAAAACCTACAGTCATCTAATTTCAGCTTAAACATGTTCCAGCAAGATCATCACCATCCTGACATG
TTCCGTTTTGTCCCTAGACCCGAGCACAAGGTATTGCGAGGCTAATTTAGCTCAATTCAGTTGTCAGAGCAATTGTGTGTGAAAAGTGTGAACAACAGCA
TAGGAGACAATACAGCAAGTGTAGCGTGCCAATATGTGTCCAGCTTGATAGTTCACTATAATAAAGTTCTTTAATTCAAGTATTTGAATAATATAGAATC
TCAACCTTCAAGTTTAAAAGGTTTTGTATTTGCTATTATAATCGTTGCCACCCCCTCACCCCTCTCAAAAAGAGAGTTACTTTGTAGCGCACTGTAAATC
TCTTGATTGAAATAGTGGCATGTCATCTTATTTAATTCAGTTATTTCATAAGGTACAATTTGTTTAGCTGACAGCTCAGACATGCTTCTATGCAACATGT
TTATACCAATGCTGTATTTTGGCAATCCTGGATGTGGCATTACTTAACTGTGCTTTCATATTCAATAGTTCTGGCAAAGGGAGGCTGCAAGCTTGAGACA
ACAACTGCACAACCTGCAAGAAAGTCATCGGTATGGCTAGAAAACCCTGCAAATTTTACAAATGTACAAGTCAGAAAATTTGCCTAATGTAAAAGGATCA
AACTACAGGATTTGTCAGCATGAGCACAAGTGCCTAACTTCCGAGTTAGATAACTCTGGAGGAAGGAAATCCTTCCTACTGTTTGTTTCAAGAATATACA
AAAATTCAAAACAACATTAGAATAGAACCAAAAGAAGAAACAATATTCACCATGTAGCAATACTCAATTCCTGGTTGTCTTATTTATAAAGGTATAAAAC
TAGGTTCTGACGAGACAATTATGAATCAACTACACCATGTTACAGATCATCCATAACTCTTGGTGATGCAAATTAAGGGCTGTAAAGTGACAAGTATACC
TCCACCAACTAATTTGTGCAGAGTGTATAAAATTGTAAAACAATGGATAAGAATGAATTTAGTATACAAGATAGCATACAATGCCCAAGTTTAAGGAGAC
ATACTGATTTACTGACTCCATAGGACAATCTTTTGCTTATGAGGACATCAGTTTTCATAAACAATATCAATGTAAAAGGAGGTAACAGTTTATGAATTTC
CTATGCCAAGCCATTTATAAGTCAAAAAATGTACCTATTTAGCTTTTATGCATTCCAATTAACTTCCAAAGAAGAAGAGTACATACTAATGGAAAACAAA
TATGATATTATCATCTGTTTTGCCCGGTAGTTTCTTGAGTCTCTGATTTGTCCGCACTTCACTCCCAACCAACACAACATTAATACACATGGGCAGAGGA
ATATCAGGTTATCAGGTAAACTGAATACAGTTTGATTGACTGCAGGCAGTTGATGGGACAAGATCTTTCTGGATTGGGCGTCAAGGAACTCCAAAATCTA
GAAAATCAGCTAGAAATGAGCCTACATTGCATCCGGACAAAAAAGG
